# The exosomal miRNA/FOXN3 axis empowers fibroblasts with tumor-promoting functions via enhanced migration and altered secretome

**DOI:** 10.1186/s12951-026-04633-6

**Published:** 2026-06-05

**Authors:** Jian Zhang, Meng Li, Qian Li, Bin Fu, Yangyang Li, Shuangli Mi

**Affiliations:** 1 Beijing Institute of Genomics, Chinese Academy of Sciences/China National Center for Bioinformation, Beijing, 100101 China; 2 University of Chinese Academy of Sciences, Beijing, 100049 China; 3 Beijing Key Laboratory of Intelligent Governance and Application of Biological Big Data, China National Center for Bioinformation, Beijing, 100101 China; 4 Department of Molecular Pharmacology, Key Laboratory of Cancer Prevention and Therapy, Tianjin Medical University Cancer Institute and Hospital, National Clinical Research Center for Cancer, Tianjin’s Clinical Research Center for Cancer, Tianjin, 300060 China; 5 State Key Laboratory of Medical Proteomics, Beijing Proteome Research Center, National Center for Protein Sciences (Beijing), Beijing Institute of Lifeomics, Beijing, 102206 China

**Keywords:** Cancer-associated fibroblasts, Exosome, FOXN3, Lung adenocarcinoma, Malignant proliferation, Microenvironment, Stroma

## Abstract

Malignant tumor tissues with high proliferative activity are frequently characterized by a rich abundance of cancer-associated fibroblasts (CAFs). Nevertheless, the precise molecular mechanisms by which CAF–tumor cell crosstalk drives malignant proliferation remain incompletely elucidated. In this study, we demonstrate that exosomal microRNAs derived from lung adenocarcinoma cells reprogram adjacent normal fibroblasts into CAFs through downregulation of their common target, FOXN3. Suppression of this transcriptional repressor enhances the expression of genes associated with cell migration and extracellular matrix remodeling, thereby potentiating CAF infiltration into the tumor core. The consequent spatial proximity between CAFs and tumor cells enables the aberrantly altered secretome of CAFs to exert robust paracrine effects on malignancy. By focusing on receptors that interact with these dysregulated secreted proteins, we evaluated the drug sensitivity of patients bearing highly proliferative tumors to receptor-targeting agents, revealing potential therapeutic opportunities. Collectively, our work offers a detailed molecular dissection of tumor cell proliferation and proposes translationally relevant strategies for cancer therapy.

**Graphic abstract:**

**Figure:**
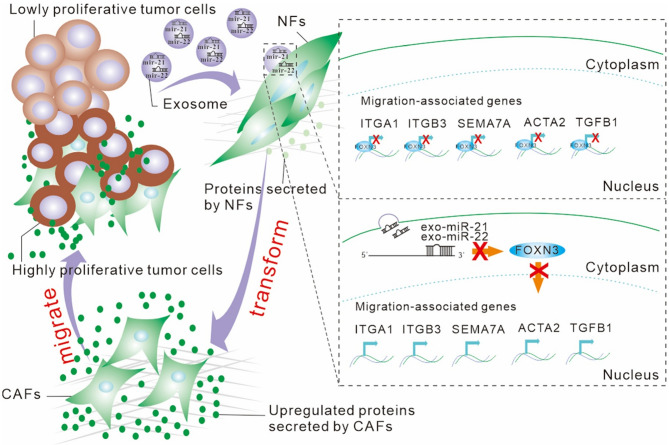

**Supplementary Information:**

The online version contains supplementary material available at 10.1186/s12951-026-04633-6.

## Introduction

Malignant proliferation is a feature of most tumor cells and is correlated with tumor progression, treatment resistance, and poor clinical outcomes [1, 2]. Biological macromolecules that support the rapid proliferation of tumor cells are derived mainly from the tumor microenvironment (TME), which is composed of stromal cells and the extracellular matrix (ECM) [3]. The main component of stromal cells is activated fibroblasts, also termed cancer-associated fibroblasts (CAFs), which play crucial roles in accelerating tumor proliferation and are present in tumor tissues at all stages of cancer progression [3]. Some studies have shown that a greater proportion of CAFs is associated with a more advanced AJCC stage [4]. Previous studies have shown that tumor cells can facilitate their own growth with the help of CAFs, which indicates that an increase in the number of CAFs in tumor tissue could promote the growth of tumor cells [3]. The presence of CAFs in tumor tissue is not merely due to the passive incorporation of fibroblasts during tumor mass formation; rather, the high proportion of CAFs in tumor tissue is primarily formed through active migration into the tumor mass. The proximity of CAFs to tumor cells enables them to promote rapid proliferation of tumor cells more efficiently through the secretion of soluble factors [5, 6].

On the basis of current research, CAFs can be divided into two categories according to the distance between CAFs and tumor masses. The first category comprises CAFs located at a distance from the tumor mass. For example, bone marrow-derived circulating mesenchymal stem cells (MSCs) are recruited into the tumor mass and transformed into CAFs in breast, gastric, and prostate cancer [6–8]. The second type encompasses CAFs located near the tumor mass. Instances include the transformation of local sessile precursors into CAFs in primary intestinal tumors and the conversion of intestinal pericryptal (*Lepr*^*+*^) cells into ACTA2^+^ CAFs [9, 10]. Enhanced migratory capacity is the fundamental prerequisite that enables CAFs, regardless of their origin, to infiltrate tumor tissues. Thus, understanding the molecular mechanisms of CAF migration into the tumor mass is a key step for understanding how the microenvironment promotes tumor progression. However, these mechanisms are still poorly understood.

After entering the tumor mass, CAFs contribute to tumor progression via the secretion of growth factors, cytokines, and the ECM. CAF-secreted stromal cell-derived factor-1 (SDF-1) stimulates tumor growth through the cognate receptor CXCR4, which is expressed by breast cancer cells [5]. Growth differentiation factor 15 (GDF15) derived from CAFs exerts prominent paracrine effects on prostate cancer cell growth [11]. Leukemia inhibitory factor (LIF) is a key paracrine factor produced by activated pancreatic stellate cells that acts on cancer cells to promote tumor progression [12]. Considering the lack of studies on the systematic identification of proteins secreted by CAFs, our previous study revealed alterations in the overall protein secretome of CAFs activated by tumor-derived exosomes [13]. Although the systematic identification of secreted proteins has advanced our understanding of the role of tumor cell–CAF crosstalk in malignant proliferation, the discovery of effective drug targets for clinical use demands a deeper investigation into the underlying molecular mechanisms involved. This information is important for understanding the entire continuum of CAF activity, from activation and migration to the execution of procancerous effects.

In this study, we identified a key transcriptional repressor, FOXN3, whose expression is regulated by a set of highly expressed exosomal microRNAs. Downregulation of FOXN3 enhances the capacity of CAFs to migrate into tumors and alters the profile of secreted matrisome-associated proteins. In addition, we developed a method to identify drug targets for rapidly proliferating tumors on the basis of crosstalk between CAFs and tumor cells. These results enhance our understanding that the proliferation rate of tumor cells depends on the support of CAFs and provide potential targets for the treatment of lung adenocarcinoma (LUAD) from the perspective of the TME.

## Results

### Highly proliferative LUAD tissues contain more CAFs

**Fig. 1:**
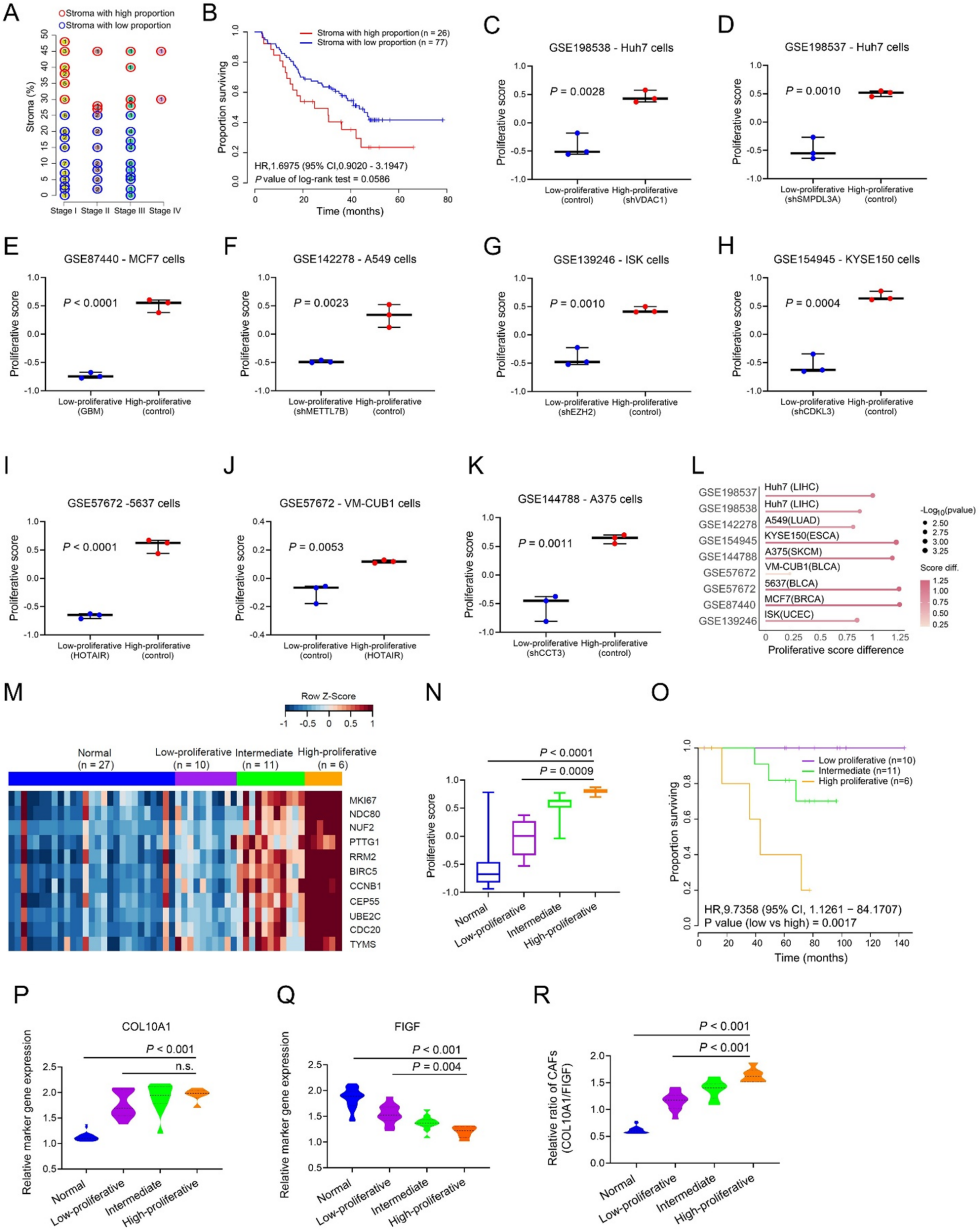
Highly proliferative tumor tissues contain more CAFs than lowly proliferative tumor tissues. (**A**) Distribution of the proportion of stromal cells in tumor tissues from 103 LUAD patients with stage I to IV. (**B**) Kaplan‒Meier curves showing disease-free survival times between patients with tumor stroma ratio greater than 25% (high proportion) and those with tumor stroma ratio less than or equal to 25% (low proportion). (**C-K**) Proliferation scores of cancer cell lines (Huh7, MCF7, A549, ISK, KYSE150, 5637, VM-CUB1, and A375) with high and low proliferation rates in 8 datasets. Two-sided Student’s *t* tests were used to assess differences. The blue points denote cells with low proliferation. The red points denote highly proliferative cells. (**L**) Summarized differences in the proliferative scores of cancer cell lines under different conditions from (C-K). (**M**) Heatmap showing unsupervised hierarchical clustering to classify samples (including tumor and paracancerous tissue, x-axis) in the GSE87340 dataset on the basis of the expression levels of the 11-gene proliferation signature (y-axis). The four subclusters were assigned to the normal group (blue bar), proliferation score-high (orange bar), proliferation score-intermediate (green bar), and proliferation score-low (purple bar) groups. (**N**) The 11-gene signature successfully stratified the samples, with adjacent normal tissues demonstrating the lowest baseline proliferation scores (two-sided Student’s *t*-test, *P* < 0.05). The boxes show the median ± 1 quartile, with whiskers extending from the hinge to the smallest or largest value. (**O**) Kaplan‒Meier curves showing overall survival times among patients with high-, intermediate-, and low-proliferative tumors. A two-sided log-rank test with *P* < 0.05 was considered statistically significant. The mRNA expression levels of the CAF marker (*COL10A1*) (**P**) and NF marker (*FIGF*) (**Q**), and the ratio of the expression of *COL10A1* to *FIGF* (**R**) in the normal groups and the proliferation score-high, -intermediate, and -low groups

The tumor stroma, which is primarily composed of CAFs and their secreted ECM, influences the malignant proliferation of tumor cells [4]. To explore the association between stromal abundance and patient prognosis, we utilized data generated by Xu et al., who quantified the proportions of tumor cells, stroma, immune cells, normal tissue, and necrosis on the basis of hematoxylin and eosin (H&E) staining of 103 LUAD tissue samples spanning stages I to IV (Fig. 1A) [14]. Patients with stroma-rich tumors (defined as a stroma percentage greater than 25%) exhibited a relatively poorer prognosis (hazard ratio [HR] = 1.6975, *P* value of log-rank test = 0.0586) (Fig. 1B). One reason for this poor prognosis may be that the presence of more CAFs in stroma-rich tumors accelerates tumor cell proliferation.

Next, we sought to confirm whether an increased presence of CAFs corresponds with highly proliferative lung cancer tissues. To classify the proliferative status of the tumor samples, we employed an 11-gene expression signature previously established to assess proliferation in breast cancer [15]. To test the robustness of this signature across multiple cancer types, we analyzed 9 independent gene expression datasets of cancer cell lines with defined proliferative statuses and calculated proliferative scores for each sample on the basis of this 11-gene signature. In all cases, cells with a highly proliferative status presented significantly higher proliferative scores than those with a low proliferative status did (Fig. 1C − L), demonstrating that the 11-gene signature is robust enough to define the proliferative status in different cancer types, including LUAD.

We next analyzed 3 independent LUAD transcriptomic datasets, GSE87340 (*n* = 54), TCGA-LUAD (*n* = 518), and GSE40419 (*n* = 164), which included a large number of tumor samples and adjacent normal tissues as controls. We classified samples from these datasets into proliferative score-high, proliferative score-intermediate, and proliferative score-low groups on the basis of the unsupervised clustering pattern of the 11 genes (Fig. 1M, Figures S1A and S2A). The different groups presented distinct proliferative score distributions, and the lowest proliferative scores in adjacent normal samples further validated the reliability of this 11-gene signature (Fig. 1N, Figures S1B and S2B). Survival analysis revealed that patients with highly proliferative tumors had a worse prognosis than those with lowly proliferative tumors in GSE87340 (HR = 9.7358, *P* value of log-rank test [proliferative score-low vs. -high] = 0.0017) (Fig. 1O) and TCGA-LUAD cohort (HR = 1.8864, *P* value of log-rank test [proliferative score-low vs. -high] = 0.03) (Figure S1C), indicating that the tumor proliferation rate is a critical risk factor for LUAD patient prognosis.

We further determined whether more CAFs are present in highly malignant proliferative lung cancer tissues. On the basis of the study by Lambrechts et al. [16], which identified *COL10A1* and *FIGF* as key markers for activated CAFs and quiescent normal fibroblasts (NFs), we analyzed the GSE87340 dataset and found that the mRNA expression levels of *COL10A1* was significantly higher in tumor tissues with high, intermediate, and low proliferative scores than those in adjacent normal tissues (*P* value [proliferative score-high vs. normal tissues] < 0.001; Fig. 1P), whereas the mRNA expression level of the *FIGF* was significantly lower in tumor tissues with high proliferative scores than in those with low proliferative scores (*P* value [proliferative score-high vs. -low] = 0.004; *P* value [proliferative score-high vs. normal tissues] < 0.001; Fig. 1Q). The ratio of *COL10A1* to *FIGF* expression was significantly higher in tumor tissues with high proliferative scores than in those with low proliferative scores (*P* value [proliferative score-high vs. -low] < 0.001; *P* value [proliferative score-high vs. normal tissues] < 0.001; Fig. 1R). These findings were independently validated in two additional LUAD cohorts, including the TCGA-LUAD cohort (Figure S1D − F) and the GSE40419 cohort (Figure S2C − E), both of which showed the similar pattern. These findings indicate that the presence of more CAFs in tumor tissues is associated with the malignant proliferation of tumor cells. Further exploration is needed to understand the specific mechanisms by which a large number of CAFs appear in highly proliferative tumor tissues and the roles these CAFs play in tumor cell proliferation.

### The presence of CAFs in highly proliferative LUAD tissues is associated with tumor exosomes

**Fig. 2:**
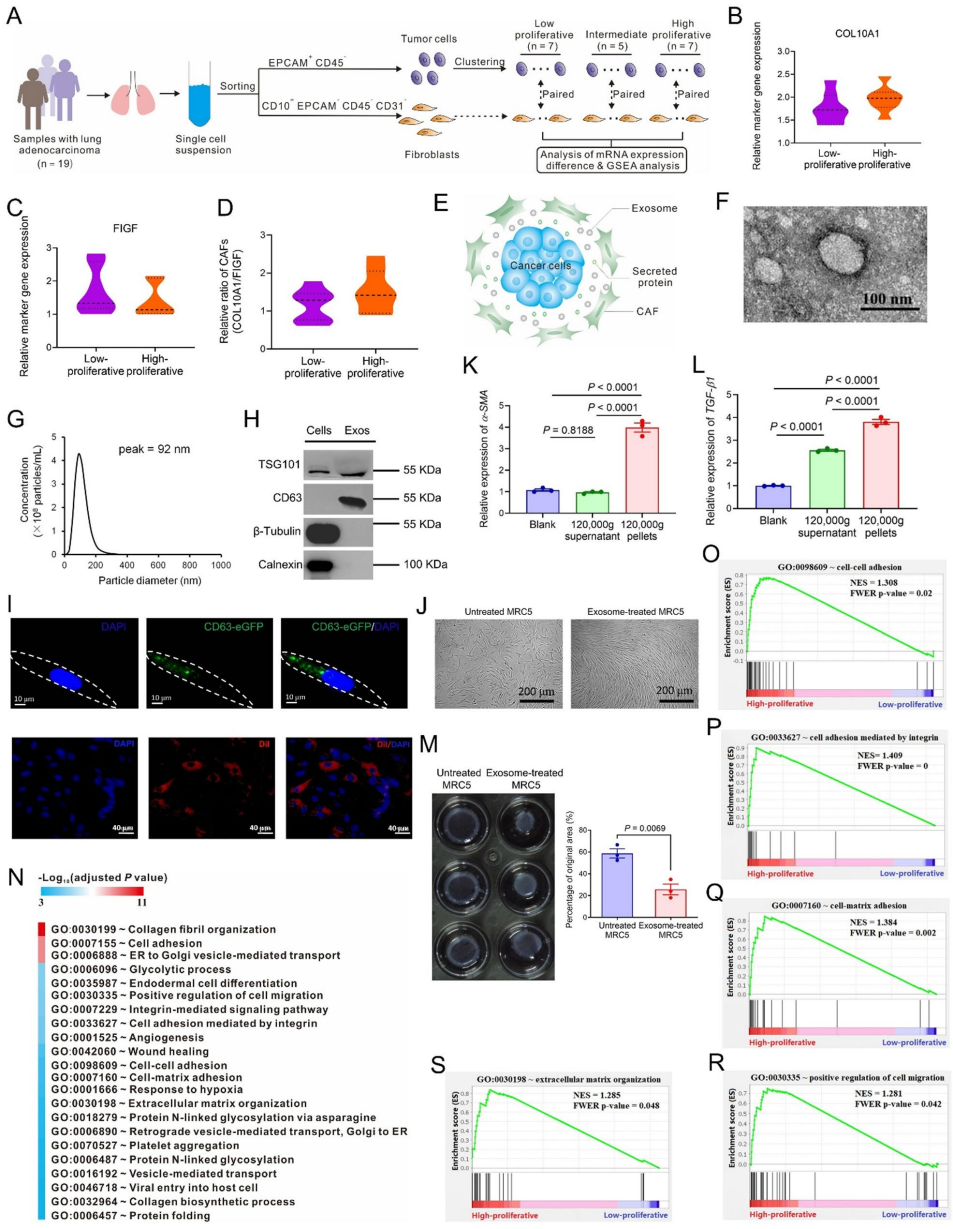
The presence of CAFs in highly proliferative LUAD tissues is associated with tumor-derived exosomes. (**A**) Workflow of the dataset (GSE111907) reanalysis. Paired tumor cells and fibroblasts were isolated from 19 LUAD tissues. Unsupervised hierarchical clustering was applied to classify tumor cells into proliferation score-low, proliferation score-intermediate, and proliferation score-high groups on the basis of the expression levels of the 11-gene proliferation signature. The differentially expressed genes were compared between fibroblasts paired with highly proliferative tumor cells and fibroblasts paired with lowly proliferative tumor cells. (**B** and **C**) mRNA expression levels of a CAF marker (*COL10A1*) and an NF marker (*FIGF*) in fibroblasts paired with highly proliferative and lowly proliferative tumor cells. (**D**) The ratio of the expression of *COL10A1* to *FIGF* in the proliferation score-high and proliferation score-low groups. (**E**) Schematic of the model of crosstalk between tumor cells and fibroblasts. (**F**) Transmission electron micrograph of exosomes derived from A549 cells. Scale bar: 100 nm. (**G**) The nanoparticle concentration and size distribution of exosomes derived from A549 cells. (**H**) Immunoblots of TSG101, CD63, β-tubulin, and calnexin in cell lysates and purified exosomes. (**I**) Fluorescence images showing the delivery of eGFP-CD63-labeled exosomes (green) and DiI-labeled exosomes (red) to MRC5 cells. The white dotted outline indicates MRC5 cells. The blue color indicates nuclei stained with DAPI. (**J**) The morphologies of exosome-treated MRC5 cells and untreated MRC5 cells were imaged via light microscopy. The mRNA levels of *α-SMA* (**K**) and *TGF-β1* (**L**) in MRC5 cells incubated with A549-derived exosomes (120,000 g pellets), supernatant (culture medium depleted of 120,000 g pellets), or control medium (blank) were assessed via qPCR. The data are representative of three experiments (mean ± SEM). (**M**) Exosome-treated MRC5 cells and untreated MRC5 cells were assessed for their ability to contract collagen. The areas of collagen contraction were quantified via Image-Pro Plus software. (**N**) GO analyses of 355 genes whose expression was upregulated in MRC5 cells after treatment with A549-derived exosomes. The genes enriched in ECM remodeling and migration-associated GO terms in (N) were enriched among genes showing higher expression in fibroblasts paired with highly proliferative tumor cells than in those paired with lowly proliferative tumor cells from the GSE111907 dataset (**O-S**)

To further identify the proportion of CAFs in fibroblasts surrounding tumor cells with different proliferative statuses, we utilized public data from *Gentles et al.* [17], in which epithelial markers (EPCAM^+^ and CD45^−^) and fibroblast markers (CD10^+^, EPCAM^−^, CD45^−^, and CD31^−^) were used to sort epithelial cells and paired fibroblasts from matched tumor tissue. Based on the 11-gene signature, we stratified the sorted malignant epithelial cells into high-, intermediate-, and low- proliferation groups and analyzed their corresponding paired fibroblasts (Fig. 2A). Despite the limited sample size, clear differences were observed: the expression levels of the CAF marker were higher in fibroblasts paired with highly proliferative tumor cells than in those paired with lowly proliferative tumor cells (fold change [proliferative score-high vs. -low] = 1.12; Fig. 2B); the expression levels of the NF marker were lower in fibroblasts paired with highly proliferative tumor cells (fold change [proliferative score-high vs. -low] = 0.83; Fig. 2C); and the proportion of CAFs was greater in fibroblasts paired with highly proliferative tumor cells. (fold change [proliferative score-high vs. -low] = 1.32; Fig. 2D). These results are consistent with those shown in Fig. 1P − R, Figure S1D − F, and Figure S2C − E, suggesting that highly proliferating tumor cells are often accompanied by large numbers of CAFs in tumor tissues.

As shown in our previous study, A549 cells (malignant type II alveolar epithelial cells) transform normal MRC5 cells (human embryonic lung fibroblasts) into CAFs via their released exosomes, and CAFs further promote the proliferation of A549 cells via abnormally secreted proteins [13]. Based on this evidence, we hypothesized that the abundance of CAFs in highly proliferative tumors results from tumor cells releasing exosomes that alter the phenotype of surrounding fibroblasts (Fig. 2E). To test this hypothesis and elucidate the underlying molecular mechanism, we employed a crosstalk model of A549 and MRC5 cells.

In accordance with standard exosome isolation methods [13, 18–20], we isolated exosome-enriched pellets from conditioned medium of A549 cells. The pellets exhibited a vesicular-like structure, with a size distribution ranging from 30 nm to 210 nm and a predominant diameter of 92 nm (Fig. 2F and G). The presence of exosomal markers (CD63 and TSG101) and the absence of cellular markers (calnexin and β-tubulin) further verified that the pellets were exosomes (Fig. 2H). To determine whether A549 cell-derived exosomes can be internalized by fibroblasts, we employed two complementary labeling strategies. First, we engineered A549 cells to stably express CD63-eGFP fusion proteins and collected their released exosomes. After MRC5 cells were incubated with eGFP-labeled exosomes for 4 h, CD63-eGFP signals were detected within the MRC5 cells, confirming successful exosome delivery (Fig. 2I). Because CD63-eGFP labeling preferentially marks the CD63⁺ exosome subpopulation rather than the total exosome population, we performed a complementary tracking experiment using DiI membrane dye, which broadly labels all exosomal lipid bilayers. Following 4-hour incubation, a strong cytoplasmic red signal appeared within the MRC5 cells, supporting a broader exosome uptake beyond the CD63⁺ population alone (Fig. 2I).

To assess the capacity of A549 cell-derived exosomes to educate fibroblasts, we treated MRC5 cells with these isolated exosomes. Following treatment, the cells underwent a dramatic morphological shift, adopting a spindle-shaped structure with planar polarity typical of activated fibroblasts (Fig. 2J). For the in vitro functional validation of fibroblast activation, we assessed the canonical CAF markers *α-SMA* alongside the critical profibrotic cytokine *TGF-β1*. As expected, the expression levels of both characteristic genes were significantly upregulated in exosome-treated MRC5 cells compared to untreated controls. Notably, while the exosome-depleted medium (120,000 × *g* supernatant) induced a moderate elevation in *TGF-β1* expression (~ 2.5-fold, *P* < 0.0001), it entirely failed to alter *α-SMA* levels (*P* = 0.8188). More importantly, the modest effect of the depleted medium on *TGF-β1* was substantially weaker than the robust upregulation (~ 3.8-fold) induced by intact exosomes. Collectively, these comparative data confirm that profound CAF transformation is primarily driven by tumor-derived exosomes rather than soluble factors alone (Fig. 2K and L).

Given that activated fibroblasts typically exhibit enhanced contractility [3], we assessed the effect of A549-derived exosomes on MRC5 cell-mediated collagen gel contraction. The contraction ability of MRC5 cells was markedly enhanced after treatment with A549-derived exosomes (Fig. 2M), suggesting a pronounced transformation effect. To further explore the downstream signaling pathway in fibroblasts activated by A549 cell-derived exosomes, we performed mRNA transcriptomic sequencing of fibroblasts before and after exosome treatment (Table S1). Gene ontology (GO) analysis of the differential transcriptome (fold change [MRC5-treated with exosomes vs. control] > 1.5, *Q* value < 1E − 3) revealed significant enrichment in terms associated with cell migration (i.e., GO:0007155 ~ cell adhesion; GO:0030335 ~ positive regulation of cell migration; GO:0033627 ~ cell adhesion mediated by integrin; GO:0098609 ~ cell‒cell adhesion; GO:0007160 ~ cell‒matrix adhesion) and ECM remodeling (i.e., GO:0030199 ~ collagen fibril organization; GO:0030198 ~ ECM organization) (Fig. 2N), suggesting that tumor-derived exosomes may regulate fibroblast migration and alter the fibroblast secretome.

We next leveraged Gene Set Enrichment Analysis (GSEA) to determine whether these exact exosome-activated pathways are upregulated in vivo within fibroblasts paired with high-proliferation tumors. Indeed, the specific terms identified in our in vitro model (Fig. 2N) were significantly enriched in these patient-derived fibroblasts, including cell‒cell adhesion (normalized enrichment score [NES] = 1.308, family-wise error rate [FWER] *P* value = 0.02; Fig. 2O, Figure S3A), cell adhesion mediated by integrin (NES = 1.409, FWER *P* value = 0; Fig. 2P, Figure S3B), cell‒matrix adhesion (NES = 1.384, FWER *P* value = 0.002; Fig. 2Q, Figure S3C), positive regulation of cell migration (NES = 1.281, FWER *P* value = 0.042; Fig. 2R, Figure S3D), and ECM organization (NES = 1.285, FWER *P* value = 0.048; Fig. 2S, Figure S3E).

**Fig. 3:**
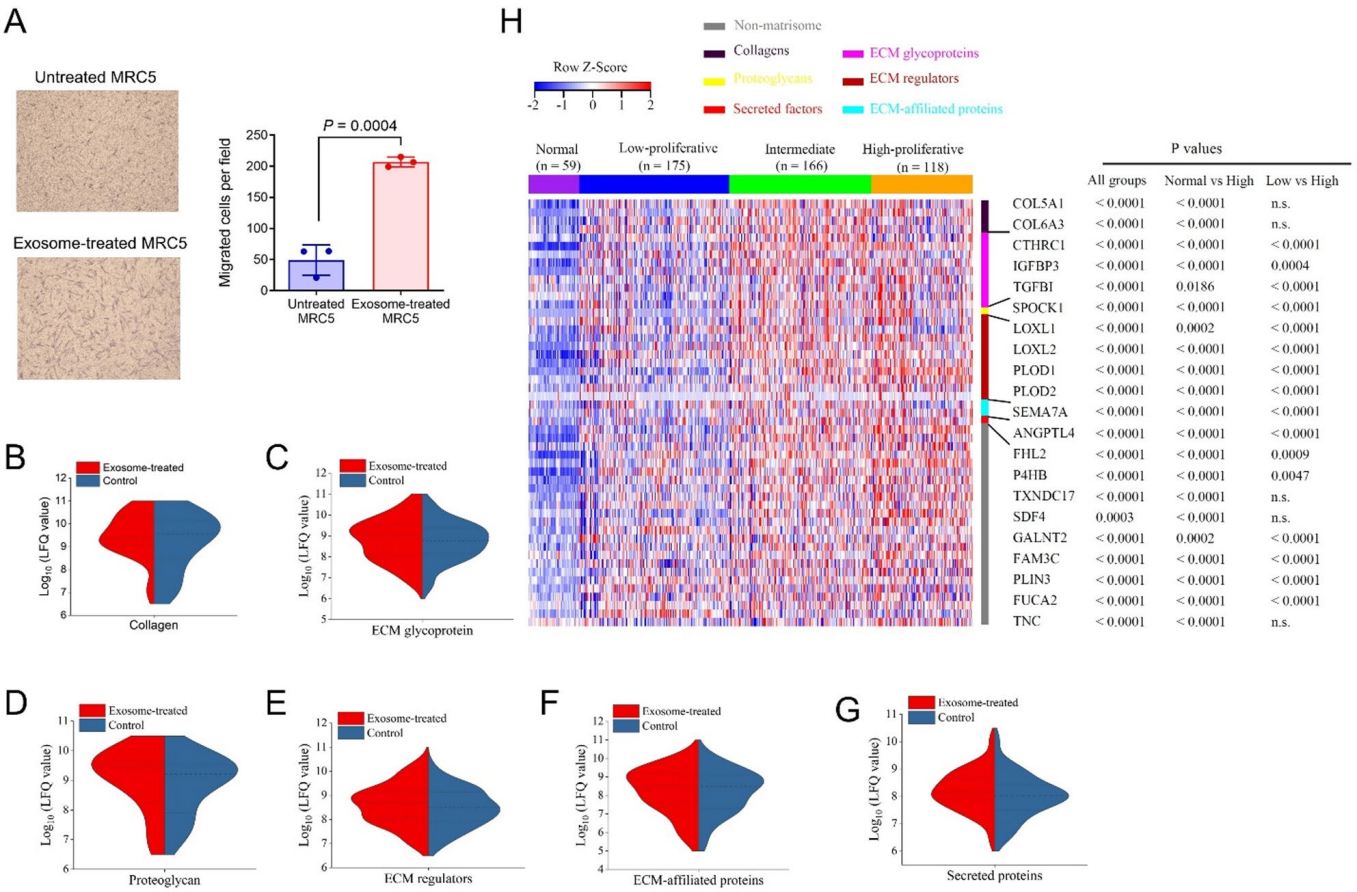
Tumor-derived exosomes promote CAF migration and ECM remodeling. (**A**) Detection of the migration capability of A549-derived exosome-treated MRC5 cells and untreated MRC5 cells. The migrated cells were counted, and representative images are shown. The matrisome-associated extracellular proteins (PXD016888) from both untreated fibroblasts and exosome-treated fibroblasts were categorized into 6 subclasses, and the distributions of protein abundance of each subclass are shown in (**B-G**). (**H**) The abundance of 51 proteins secreted by A549-derived exosome-induced MRC5 cells was significantly higher than that of 51 proteins secreted by untreated MRC5 cells. The expression levels of mRNAs corresponding to these 51 secreted proteins are shown in four subclusters of LUAD tissues in the TCGA-LUAD cohort, i.e., the normal group (blue bar), proliferation score-low (purple bar), proliferation score-intermediate (green bar), and proliferation score-high (orange bar) groups. The gene symbols on the right of the heatmap are representative genes from the 51 mRNAs. Two-sided Student’s *t* test *P* < 0.05 was considered statistically significant; otherwise, it was considered nonsignificant (n.s.)

Subsequent functional assays firmly validated these transcriptomic predictions. Exosome-educated MRC5 cells exhibited a significantly greater migratory capacity than untreated control cells (Fig. 3A). Furthermore, proteomic profiling confirmed that A549 exosomes fundamentally remodeled the fibroblast secretome, particularly increasing the secretion of specific matrisome proteins (Fig. 3B − G). In our previous study, the exosome-induced increase in the abundance of matrisome proteins secreted by CAFs promoted tumor cell proliferation via paracrine signaling [13]. Re-examining the TCGA-LUAD cohort, we found that genes encoding these exosome-induced upregulated proteins (e.g., SPOCK1, *P* value [tumor with proliferative score-high vs. -low] < 0.0001) were also expressed at higher levels in tumor tissues with high proliferative scores than in those with low proliferative scores (Fig. 3H). This in vivo correlation was reliably reproduced in the GSE87340 and GSE40419 datasets (Figure S4). Taken together, these findings strongly suggest that CAFs enriched within highly proliferative tumors are actively converted by tumor cell-derived exosomes, a transformation that endows them with augmented migratory abilities and an oncogenic matrisome secretome.

### A group of specific exosomal microRNAs downregulate *FOXN3* expression in recipient fibroblasts

**Fig. 4:**
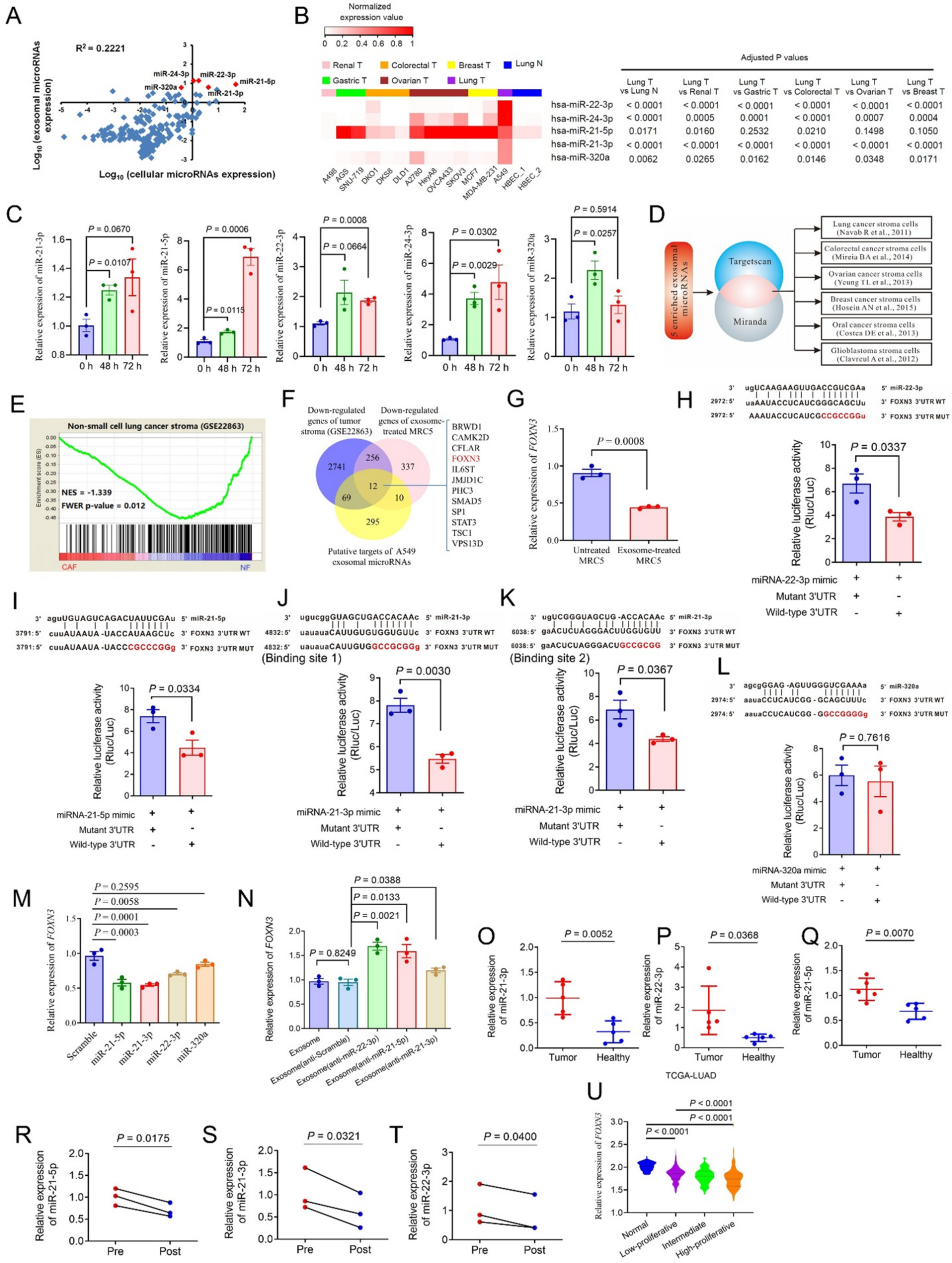
The target of the specific exosomal microRNAs is FOXN3. (**A**) The expression levels of microRNAs in A549 cells and A549-derived exosomes were detected via microRNA sequencing. The red points denote the top five microRNAs highly expressed in the exosomes. (**B**) The expression levels of the five microRNAs in exosomes derived from 6 cancer cell lines and a normal lung cell line. (**C**) The expression levels of the five microRNAs in MRC5 cells after incubation with A549-derived exosomes for 0 h, 48 h, and 72 h. (**D**) The targets of the top five highly expressed exosomal microRNAs were predicted via miRanda and TargetScan. The overlapping targets were used to perform enrichment analyses with the downregulated genes of the tumor stroma from breast cancer, ovarian cancer, colorectal cancer, LUAD, glioblastoma, and oral squamous carcinoma. (**E**) The overlapping targets enriched with the downregulated genes of the tumor stroma from LUAD. (**F**) Overlapping genes among the three gene lists. The three gene lists included (1) the overlap of the top five A549-derived exosomal microRNA targets predicted by miRanda and TargetScan; (2) the genes whose expression was downregulated in MRC5 cells after treatment with A549-derived exosomes; and (3) the genes whose expression was downregulated in the tumor stroma compared with that in the normal stroma from LUAD patients (GSE22863). (**G**) *FOXN3* expression levels in exosome-treated MRC5 cells and untreated MRC5 cells. (**H-L**) Relative luciferase activities of MRC5 cells in the presence of the indicated treatments. (**M**) *FOXN3* expression levels in MRC5 cells transfected with mimic microRNAs, including miR-21-5p, miR-21-3p, miR-22-3p, miR-320a, and the negative control. (**N**) *FOXN3* expression levels in MRC5 cells after treatment with A549-derived exosomes or antagomiR-containing exosomes. The data are representative of three experiments (mean ± SEM). (**O-Q**) The expression levels of exosomal miR-21-5p, miR-21-3p, and miR-22-3p in the plasma of LUAD patients and healthy controls. (**R-T**) The expression levels of exosomal miR-21-5p, miR-21-3p, and miR-22-3p in the plasma of pre- and post tumor resection LUAD patients. (**U**) *FOXN3* expression levels in high-, intermediate-, and low-proliferative tumors and normal tissues from LUAD patients in the TCGA-LUAD cohort. A two-sided log-rank test with *P* < 0.05 was considered statistically significant

We next determined whether NFs were activated through the regulation of specific gene expression by exosomal microRNAs. Considering that more than one hundred types of microRNAs are encapsulated in exosomes [21–23], we performed microRNA transcriptomic sequencing to analyze A549-derived exosomal microRNAs. The abundance of microRNA profiles in exosomes is not exclusively determined by the abundance of microRNA profiles within cells (R^2^ = 0.2221), implying that microRNAs are selectively secreted from cells into exosomes (Fig. 4A; Tables S2 and S3). To verify whether the nonrandomly secreted exosomal microRNAs form a unique expression pattern, we first selected the top five most abundant exosomal microRNAs, i.e., miR-21-5p, miR-21-3p, miR-320a, miR-24-3p, and miR-22-3p, which accounted for 49% of the total exosomal microRNAs, as representative A549-derived exosomal microRNAs. Quantitative PCR (qPCR) confirmed that the expression levels of these five exosomal microRNAs were higher than those of randomly selected exosomal microRNAs (Figure S5A). Next, we analyzed other exosomal microRNA sequencing data from normal lung epithelial cells (HBECs; GSE158624) [24], renal cancer cells (A498; GSE155810), gastric cancer cells (AGS, SNU-719; GSE198354), breast cancer cells (MCF7 and MDA-MB-231; GSE83669) [21], ovarian cancer cells (A2780, HeyA8, OVCA433, and SKOV3; GSE77318) [22], and colorectal cancer cells (DKO1, DKS8, and DLD1; GSE67004) [23]. The expression profile of these five microRNAs in A549 exosomes was distinct from that in HBECs, indicating a tumor-specific signature. Furthermore, their expression pattern in LUAD also differed from those observed in other cancer types, suggesting that LUAD cell-derived exosomal microRNAs possess a unique expression pattern (Fig. 4B). qPCR results revealed that the expression levels of miR-21-5p, miR-21-3p, miR-320a, miR-24-3p, and miR-22-3p in MRC5 cells were increased after incubation with A549-derived exosomes for 48 h or 72 h (Fig. 4C).

To further determine whether the regulatory effects of LUAD cell-derived exosomal microRNAs on stromal cells are cancer type-specific, we first used two bioinformatics tools (Miranda and TargetScan) [25, 26] to predict the common targets of the five exosomal microRNAs. On the basis of the rule that genes mutually regulated by four or five of the exosomal microRNAs are defined as common targets, a group of common targets was identified and subjected to enrichment analysis with downregulated genes from tumor stromal cells in breast cancer (GSE29270) [27], ovarian cancer (GSE40595) [28], colorectal cancer (GSE46824) [29], oral cancer (GSE38517) [30], glioblastoma (GSE24100) [31], and LUAD (GSE22863) [32] (Fig. 4D). The common targets were significantly enriched in the downregulated genes of LUAD stromal cells (Fig. 4E) but not in those of stromal cells from other cancer types (Figure S5B − F). These analyses suggest that LUAD cell-derived exosomal microRNAs specifically target genes in lung stromal cells.

We hypothesized that key genes regulated by exosomal microRNAs are among the common targets. To identify the critical target(s), we narrowed the panel of common targets by overlapping predicted common targets with two gene lists: (1) downregulated genes in MRC5 cells treated with A549-derived exosomes (Table S1) and (2) downregulated genes in the tumor stroma compared with adjacent normal stroma in NSCLC (GSE22863) [32]. Among the 12 overlapping genes, forkhead box N3 (*FOXN3*), a transcriptional repressor known to negatively regulate genes related to tumor cell progression [33, 34], was identified as a key core target (Fig. 4F). qPCR confirmed that *FOXN3* expression in MRC5 cells decreased after treatment with A549-derived exosomes (Fig. 4G). *FOXN3* is predicted to be regulated by miR-21-5p, miR-21-3p, miR-320a, or miR-22-3p. Alignments between the 3’UTR of *FOXN3* and miR-21-5p, miR-21-3p, miR-320a, or miR-22-3p suggest that these microRNAs may directly regulate *FOXN3* expression. Wild-type and mutated binding sites for these four microRNAs were cloned and inserted into luciferase vectors. Luciferase activity was decreased significantly in HEK293T cells cotransfected with wild-type binding site vectors and miR-21-5p, miR-21-3p (for both of its two distinct binding sites), or miR-22-3p but not in cells cotransfected with mutated binding site vectors (Fig. 4H–L), indicating that miR-21-5p, miR-21-3p, and miR-22-3p directly regulate *FOXN3* expression. Verification assays revealed that *FOXN3* expression in MRC5 cells was decreased after transfection with miR-22-3p, miR-21-3p, or miR-21-5p (Fig. 4M).

To determine the direct regulatory effects of exosomes containing miR-21-5p, miR-21-3p, or miR-22-3p on *FOXN3* expression in MRC5 cells, A549 cells were transfected with antagomirs against miR-21-5p, miR-21-3p, or miR-22-3p to disrupt the silencing function of these microRNAs. Exosomes derived from antagomir-transfected A549 cells were collected and incubated with MRC5 cells. *FOXN3* expression was not significantly suppressed in MRC5 cells treated with these exosomes (Fig. 4N). To assess the relative expression levels of exosomal miR-21-5p, miR-21-3p, and miR-22-3p in LUAD patients, we collected blood samples from LUAD patients, isolated exosomes, and quantified the expression levels of these microRNAs. Exosomal miR-21-5p, miR-21-3p, and miR-22-3p levels were significantly higher in the blood of LUAD patients than in that of healthy controls (Fig. 4O–Q) and significantly decreased after surgery (Fig. 4R–T), confirming that these exosomal microRNAs are specifically derived from LUAD. The clustered tumor tissue samples shown in Fig. 1M, Figure S1A, and Figure S2A revealed that *FOXN3* expression was significantly lower in highly proliferative tumor tissues than in normal tissues in the TCGA-LUAD, GSE87340, and GSE40419 cohorts (Fig. 4U, Figure S6A and B). Also, *FOXN3* expression was significantly lower in highly proliferative tumor tissues than in lowly proliferative tumor tissues in the TCGA-LUAD cohort (Fig. 4U). Additionally, compared with that in paired peritumoral stroma, *FOXN3* expression was significantly lower in tumor stroma containing more CAFs (Figure S6C and D). To validate these findings at single-cell resolution, we reanalyzed a publicly available scRNA-seq dataset (GSE131907). Consistent with our in vitro data, fibroblast populations from tumor tissues exhibited significantly lower *FOXN3* expression (*P* < 0.001) and concurrently higher *α-SMA* expression (*P* = 0.006) compared to fibroblasts from non-malignant lung tissues (Figure S6E and F). Collectively, these in vivo findings independently confirm that the inverse relationship between *FOXN3* suppression and CAF activation is a conserved feature of the human LUAD microenvironment, and indicate that the transcriptional repressor *FOXN3* is a direct target mutually regulated by LUAD-derived exosomal microRNAs.

**Fig. 5:**
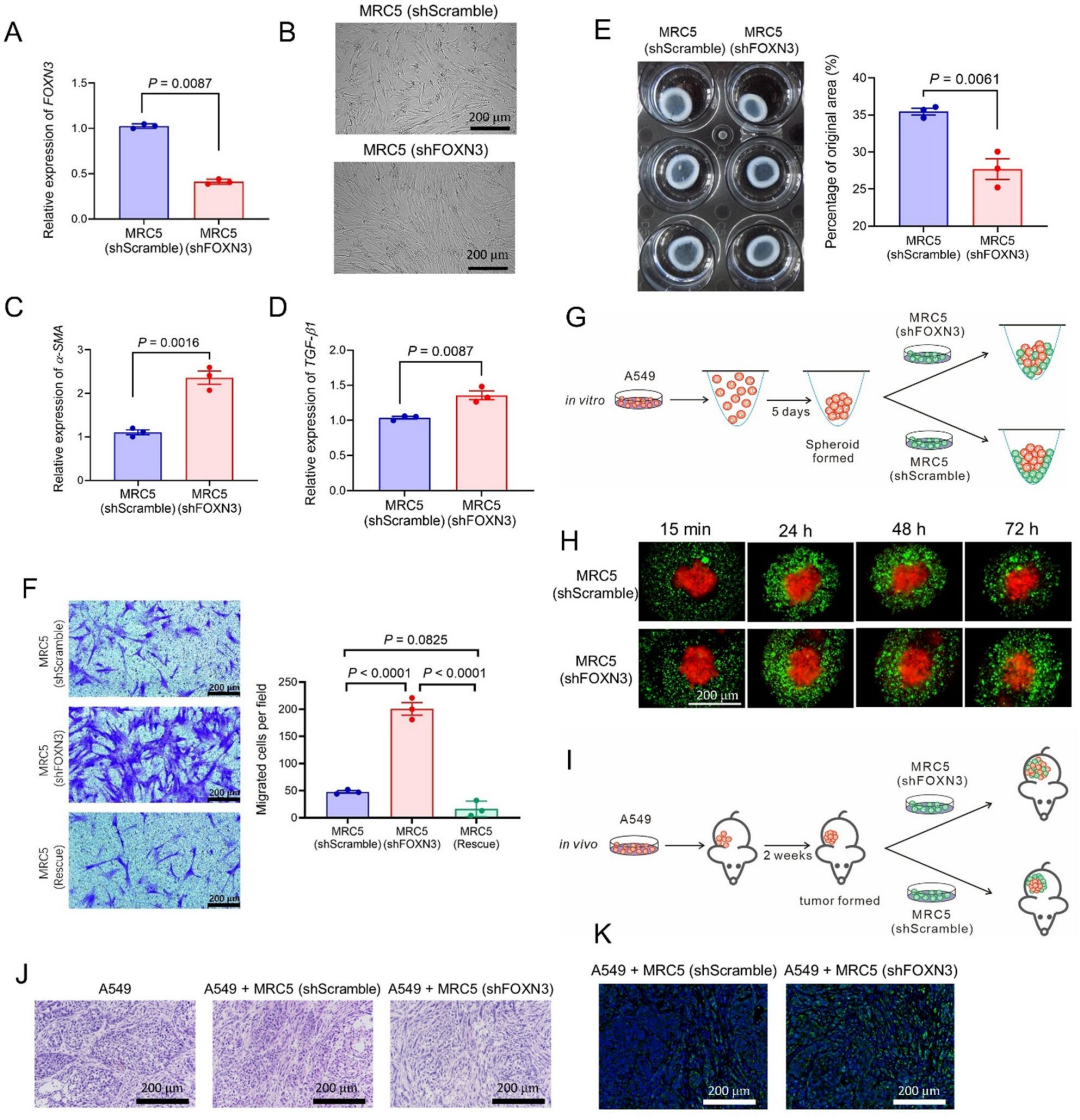
Downregulation of FOXN3 activated the expression of migration-associated genes. (**A**) Overlapping genes whose expression was upregulated in both MRC5 cells with *FOXN3* shRNAs and MRC5 cells treated with A549-derived exosomes. (**B**) KEGG and GO analyses of the overlapping genes in (A). (**C**) Migration-associated genes were enriched among genes whose expression was higher in fibroblasts paired with highly proliferative tumor cells than in those paired with lowly proliferative tumor cells from the GSE111907 dataset. (**D**) Heatmap of the *FOXN3* binding signal in the transcription start site (TSS) regions. Each row represents a 6 kb genomic window centered on TSS regions. The red color intensity indicates the number of CUT&RUN-seq reads. (**E-G**) Tracks of binding peaks of *FOXN3* in MRC5 cells with *FOXN3* shRNA and MRC5 cells with scrambled shRNA across the gene bodies of *ACTA2*, *ITGB3*, and *TGFB1* are shown in red and blue, respectively. The red dashed box denotes the transcription start region. (**H**) Schematic representation of a network consisting of migration-associated proteins (B) and their directly interacting proteins (gray). Red denotes proteins encoded by migration-associated genes directly regulated by FOXN3, and orange denotes those indirectly regulated by FOXN3. Gene expression profiles of MRC5 cells treated with A549-derived exosomes (**I**) and MRC5 cells transfected with *FOXN3* shRNAs (**J**). Gene expression levels are normalized as reads per kilobase million (RPKM) values. After MRC5 cells were transfected with *FOXN3* shRNAs for 24 h, qPCR was used to determine *FN1* expression in MRC5 cells (**K**). Subsequently, MRC5 cells were transfected with vectors expressing *FOXN3* for 48 h. *FN1* expression in MRC5 cells was detected again (**L**)

### Downregulation of *FOXN3* promotes the migration of CAFs into the tumor mass

To determine whether the effects of exosomes on fibroblast activation and migration are mediated by *FOXN3* regulation, we knocked down *FOXN3* expression in MRC5 cells (Fig. 5A). The cells formed elongated spindle-shaped structures and were aligned in the same direction (Fig. 5B). Compared with cells transfected with scrambled shRNAs, MRC5 cells transfected with short hairpin RNAs (shRNAs) targeting *FOXN3* presented increased *α-SMA* and *TGF-β1* expression (Fig. 5C and D) and enhanced collagen contraction (Fig. 5E), indicating that *FOXN3* downregulation promotes fibroblast activation.

**Fig. 6:**
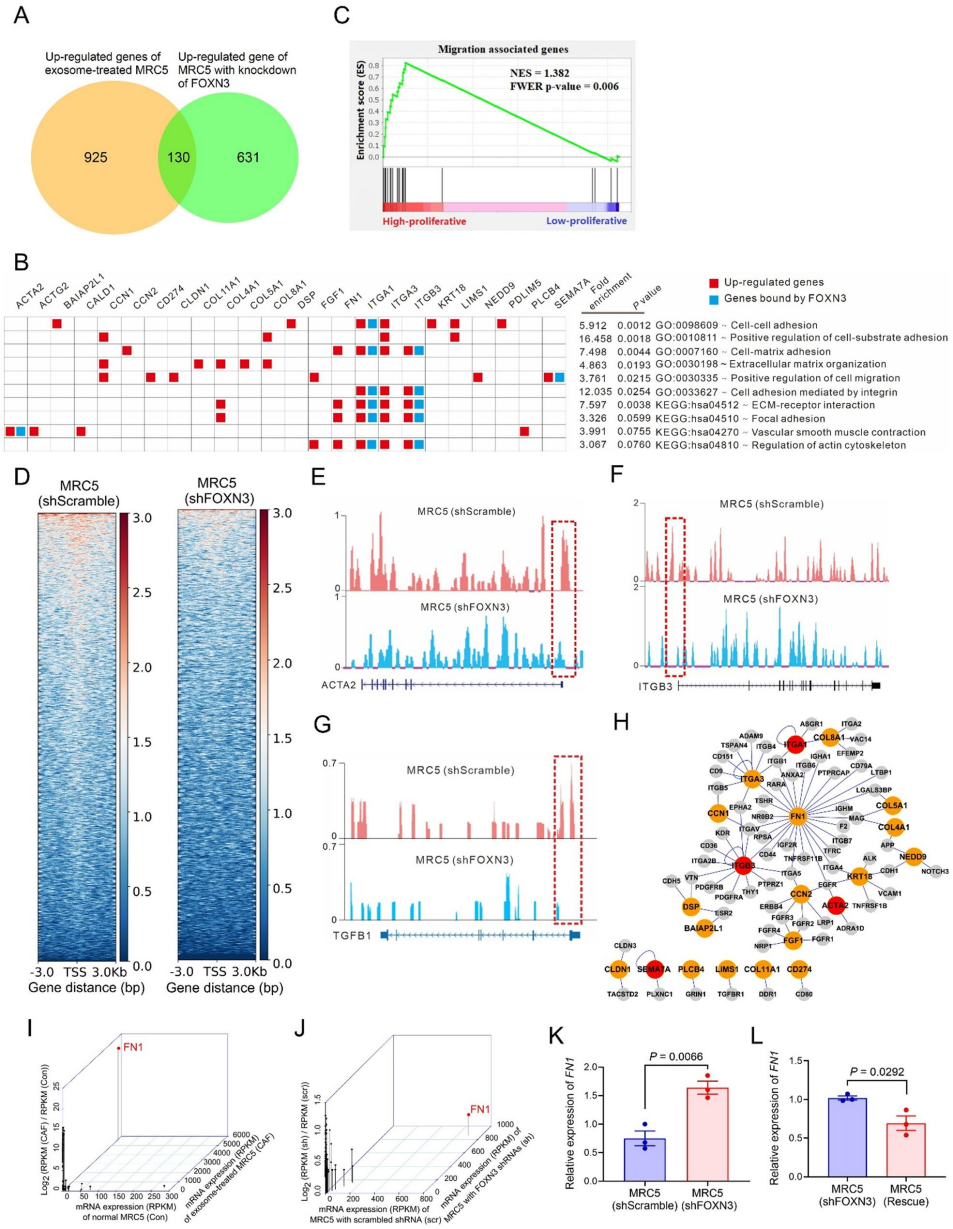
Downregulation of *FOXN3* contributes to the migration of CAFs into the tumor mass. After transfection with *FOXN3* shRNA for 24 h, the expression of *FOXN3* was downregulated (**A**). The morphology (**B**), expression levels of *α-SMA* (**C**) and *TGF-β1* (**D**), and ability to contract collagen (**E**) of MRC5 cells were detected. Collagen contraction was quantified by Image-Pro Plus software. After transfection with *FOXN3* shRNA for 24 h, the migration ability of MRC5 cells was detected. The cells were subsequently transfected with *FOXN3*-expressing vectors for 48 h, and the migration ability of the MRC5 cells was detected again (**F**). The migrating cells were counted and representative images are shown. (**G**) Schematic plot of the in vitro migration assays. (**H**) Fluorescence images of the migration of eGFP-labeled MRC5 cells (green) into RFP-labeled A549 spheroids (red) at 15 min, 24 h, 48 h, and 72 h. Representative images are presented. (**I**) Schematic plot of the in vivo migration assays. H&E staining (**J**) and immunofluorescence (**K**) of sections from tumor tissues of mice bearing A549 cells alone, A549 cells and eGFP-labeled MRC5 cells transfected with *FOXN3* shRNAs, or A549 cells and eGFP-labeled MRC5 cells transfected with scrambled shRNAs. Blue indicates nuclei labeled with DAPI, and green indicates MRC5 cells labeled with eGFP

During tumor formation, the migration of exosome-activated fibroblasts into the tumor mass is a key prerequisite for the proliferative effects of fibroblast-secreted proteins on tumor cells via paracrine mechanisms [3, 5]; however, the molecular mechanism underlying this migration remains unclear. Given that enhanced migration facilitates fibroblast infiltration into the tumor mass, we first evaluated the functional role of *FOXN3* in MRC5 cell migration. Transwell assays revealed that knockdown of *FOXN3* significantly augmented the migratory capacity of MRC5 cells. To establish a causal link and exclude potential off-target effects, we performed a functional rescue experiment by transfecting sh*FOXN3*-expressing cells with a pLEX-MCS-*FOXN3* vector encoding only the coding sequence (CDS). Since this construct lacked the 3’ UTR targeted by the shRNA, it successfully restored exogenous *FOXN3* expression. Notably, this restoration significantly abrogated the enhanced migratory phenotype induced by *FOXN3* silencing (Fig. 5F). These findings collectively demonstrate that *FOXN3* is a key regulator of the migratory ability in recipient fibroblasts and that its suppression is essential for the observed pro-migratory effects.

To validate whether *FOXN3* knockdown promotes fibroblast migration into the tumor mass, we performed in vitro and in vivo assays. For in vitro assays, we used the hanging drop method to form RFP-labeled A549 spheroids and added eGFP-labeled MRC5 cells with *FOXN3* shRNAs (in vitro shFOXN3 group) or eGFP-labeled MRC5 cells with scrambled shRNAs to the hanging drops to form an exterior coating around the A549 spheroids. After 72 h, the red spheroids in the in vitro shFOXN3 group became loose, and green spots appeared within the red spheroids, indicating that *FOXN3* downregulation promoted MRC5 cell migration into A549 spheroids (Fig. 5G and H). For in vivo assays, we subcutaneously injected A549 cells into nude mice. After two weeks, tumor masses formed. eGFP- and *FOXN3* shRNA-expressing MRC5 cells (in vivo shFOXN3 group) or eGFP- and scrambled shRNA-expressing MRC5 cells were injected into peritumoral sites (Fig. 5I). After another two weeks, the tumor masses were excised and subjected to H&E staining. The highest proportion of fibroblasts in tumor tissues was observed in the shFOXN3 group (Fig. 5J). Immunofluorescence assays for MRC5 indicators (eGFP) confirmed that these fibroblasts were MRC5 cells (Fig. 5K). These results suggest that *FOXN3* downregulation promotes fibroblast migration into the tumor mass.

### FOXN3 directly or indirectly regulates genes that promote CAF migration

FOXN3 is a critical transcriptional repressor, and its downregulation leads to the upregulation of downstream gene expression [33, 34]. To further understand the downstream signaling pathways mediated by FOXN3, we performed mRNA sequencing of MRC5 cells treated with A549-derived exosomes versus control cells to generate one list of upregulated genes (fold change [MRC5-treated with exosomes vs. control] > 1.5, *Q* value < 0.001 ; Table S1) and performed sequencing of MRC5 cells transfected with *FOXN3* shRNAs versus their controls to obtain another set of upregulated genes (fold change [MRC5-transfected with *FOXN3* shRNAs vs. control] > 1.5, *Q* value < 0.001; Table S4). The 130 upregulated genes that overlapped between these two groups were considered to result from *FOXN3* downregulation mediated by LUAD-derived exosomal microRNAs (Fig. 6A). Some of these 130 genes were enriched in the Kyoto encyclopedia of genes and genomes (KEGG) and GO terms associated with cell adhesion, including “cell‒cell adhesion”, “positive regulation of cell-substrate adhesion”, “cell-matrix adhesion”, “positive regulation of cell migration”, “cell adhesion mediated by integrin”, “ECM-receptor interaction”, and “focal adhesion”, as well as terms related to cytoskeleton and ECM regulation, such as “ECM organization”, “vascular smooth muscle contraction”, and “regulation of the actin cytoskeleton” (Fig. 6B). The regulation of cell adhesion, the cytoskeleton, and the ECM contributes to cell migration [35, 36]. Notably, a significant enrichment was observed for the migration-associated genes from Fig. 6B within the gene set from Fig. 2A that is upregulated in fibroblasts paired with highly proliferative tumors compared to those paired with lowly proliferative tumors (NES = 1.382, FWER *P* value = 0.006; Fig. 6C), demonstrating that in fibroblasts paired with highly proliferative tumor cells, the activation of migration-associated signaling pathways is linked to the exosomal microRNA-mediated downregulation of *FOXN3*.

CUT&RUN was used to identify genes directly regulated by FOXN3 (Fig. 6D; Table S5). Among the migration-associated genes, ACTA2 (Fig. 6E), a fibroblast activation marker involved in cell contraction, ITGB3 (Fig. 6F), ITGA1 (Figure S7A), and SEMA7A (Figure S7B), were directly regulated by FOXN3. Additionally, another fibroblast activation marker, TGFB1 [37], was also directly regulated by FOXN3 (Fig. 6G). Protein-protein interaction (PPI) analysis of the proteins encoded by direct and indirect FOXN3 targets revealed ITGB3 and FN1 as core regulatory nodes (Fig. 6H). ITGB3, an integrin family member implicated in tumor migration, proliferation, and drug resistance [38, 39], serves as a receptor for FN1 [40] and facilitates fibronectin assembly [41]. FN1, a cell surface/ECM glycoprotein known to promote migration in various cancers [42, 43], was found to be indirectly regulated by FOXN3. mRNA sequencing showed that *FN1* expression was significantly upregulated in MRC5 cells upon either exosome treatment or FOXN3 knockdown (Fig. 6I and J). qPCR confirmed that *FN1* expression was significantly increased in MRC5 cells after *FOXN3* shRNA transfection and was partially downregulated following transfection with pLEX-MCS-FOXN3 (Fig. 6K and L). Furthermore, mass spectrometry (MS) data revealed that the relative label-free quantification (LFQ) intensity of FN1 secreted by *FOXN3*-knockdown MRC5 cells was 1.4E8 higher than that of control MRC5 cells (Table S6). These results establish FN1 as a key downstream effector of FOXN3. In addition, this regulation is likely mediated through TGFB1, a direct FOXN3 target that itself induces *FN1* expression [44]. Collectively, the cooperation between FN1 and its receptor ITGB3 promotes invadopodia formation [45], elucidating a mechanistic pathway through which exosomal miRNAs and FOXN3 knockdown enhance CAF migration.

### Fibroblasts with downregulated FOXN3 expression promote LUAD progression via aberrantly secreted proteins

**Fig. 7:**
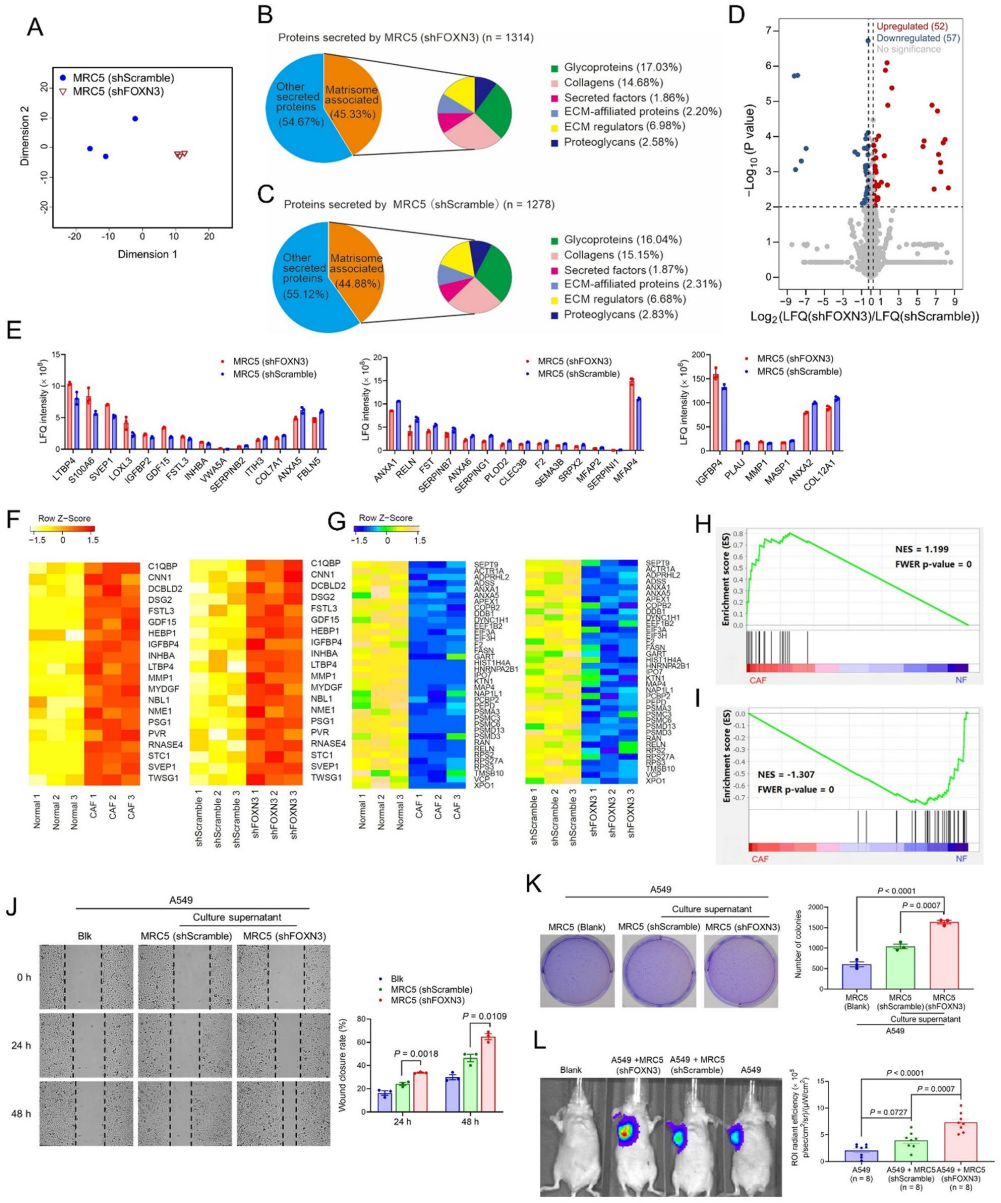
Downregulation of *FOXN3* remodels the ECM composition and promotes tumor cell proliferation. (**A**) MDS of all normalized proteomic data. Each point represents sample replication (fibroblasts with *FOXN3* shRNA, red; fibroblasts with scrambled shRNA, blue). The x-axis indicates the first dimension, and the y-axis indicates the second dimension. Functional classification of proteins secreted by MRC5 cells with *FOXN3* shRNA (**B**) and MRC5 cells with scrambled shRNA (**C**) via the Matrisome project database. (**D**) Volcano plot of mass spectrometry (MS)-based quantitative proteomics data showing the relative abundance of proteins secreted by MRC5 cells with *FOXN3* shRNA compared with that secreted by MRC5 cells with scrambled shRNA. Compared with the proteins secreted by MRC5 cells with scrambled shRNA, proteins differentially secreted by MRC5 cells with *FOXN3* shRNA were identified using a threshold of a 1.2-fold change cutoff and a *P* value less than 0.05. (**E**) Extracellular abundance of 34 matrisome proteins from both MRC5 cells transfected with *FOXN3* shRNA and MRC5 cells transfected with scrambled shRNA. The data are representative of three experiments (mean ± SEM). Heatmaps showing the 20 consistently upregulated (**F**) and 33 consistently downregulated (**G**) proteins secreted by both MRC5 cells transfected with *FOXN3* shRNA and those treated with A549-derived exosomes. The LFQ values shown are mean-centered. The 20 upregulated (**H**) and 33 downregulated (**I**) proteins were separately enriched in the upregulated and downregulated proteins of MRC5 cells after treatment with A549-derived exosomes. (**J**) Wound-healing assay of A549 cells treated with control medium (Blk), conditioned medium from MRC5 cells transfected with scrambled shRNA, and conditioned medium from MRC5 cells transfected with *FOXN3* shRNA. (**K**) Colony-forming assay of A549 cells treated with control medium (Blk), conditioned medium from MRC5 cells transfected with scrambled shRNAs, and conditioned medium from MRC5 cells transfected with *FOXN3* shRNAs. The data are presented as the means ± SEMs. Wound-healing and colony-forming assays were performed separately in triplicate and representative images are shown. (**L**) Representative bioluminescence images of 3 groups of mice (*n* = 8 each group) bearing luciferase-labeled A549 cells alone, luciferase-labeled A549 cells and MRC5 cells transfected with *FOXN3* shRNA, and luciferase-labeled A549 cells and MRC5 cells transfected with scrambled shRNA. The bar graph shows the quantification of the normalized total photon counts from the subcutaneous xenografts in the mice

Previous studies have shown that fibroblasts in tumor tissue promote tumor progression through paracrine signaling [3, 5]. We used MS to analyze changes in the secretome of activated fibroblasts systematically. We employed LFQ values to represent the relative abundance of secreted proteins and calculated the Euclidean distance between samples on the basis of these values. The results revealed that the protein secretome of MRC5 cells transfected with *FOXN3* shRNAs differed from that of MRC5 cells transfected with scrambled shRNAs, indicating that *FOXN3* downregulation alters the fibroblast secretome (Fig. 7A; Table S6). To determine the proportions of ECM core proteins and ECM-associated proteins affected by *FOXN3* among the total secretory proteins, the identified proteins were mapped to the Matrisome Project database [46, 47], which includes ECM core proteins and ECM-associated proteins. Among the proteins identified by MS, 202 proteins secreted by FOXN3-knockdown MRC5 cells and 200 proteins secreted by control MRC5 cells were annotated in the Matrisome database, accounting for 45.33% and 44.88% of the total protein abundance, respectively. These matrisome proteins were classified according to Naba et al.’s criteria: (1) core matrisome proteins: ECM glycoproteins, collagens, and proteoglycans; (2) ECM-associated proteins: ECM-affiliated proteins, ECM regulators, and secreted factors [46, 47]. The 202 matrisome proteins secreted by FOXN3-knockdown MRC5 cells included 64 glycoproteins, 16 collagens, 8 proteoglycans, 20 ECM-affiliated proteins, 63 ECM regulators, and 31 secreted factors, accounting for 17.03%, 14.68%, 2.58%, 2.20%, 6.98%, and 1.86% of the total protein abundance, respectively (Fig. 7B). The 200 matrisome proteins secreted by control MRC5 cells included 62 glycoproteins, 17 collagens, 8 proteoglycans, 18 ECM-affiliated proteins, 63 ECM regulators, and 32 secreted factors, accounting for 16.04%, 15.15%, 2.83%, 2.31%, 6.68%, and 1.87% of the total protein abundance, respectively (Fig. 7C).

We next compared the abundance of each secreted protein between FOXN3-knockdown and control MRC5 cells. With thresholds of a *P* value ≤ 0.05 and a fold change (MRC5 cells with FOXN3 shRNAs vs. control) in LFQ > 1.2, the abundance of 109 secreted proteins changed significantly (Fig. 7D). To further understand the functions of these differentially secreted proteins, we mapped the 109 proteins against the Matrisome database to identify ECM core/ECM-associated proteins according to Naba et al.’s classification [46, 47]. Among the 109 differentially secreted proteins, 34 proteins were ECM core/ECM-associated proteins, with 13 proteins showing increased abundance and 21 proteins showing decreased abundance. These 34 proteins include collagens involved in ECM structural support, such as COL12A1 and COL7A1 [48, 49]; ECM glycoproteins involved in intercellular adhesion and signal recognition, such as SVEP1, LTBP4, and RELN [50, 51]; and ECM regulators involved in ECM synthesis and degradation, such as MMP1, LOXL3, and SERPINB2 [52, 53] (Fig. 7E).

Similarly, our previous studies revealed that A549-derived exosomes alter the secretome of MRC5 cells and that the altered secretome promotes the proliferation and migration of A549 cells [13]. By comparing the secreted proteins affected by A549-derived exosomes with those affected by *FOXN3* downregulation, we detected consistent changes in the abundance of 57 secreted proteins (20 upregulated proteins and 37 downregulated proteins) (Fig. 7F and G). Gene set enrichment analysis (GSEA) revealed that the 20 upregulated proteins secreted from FOXN3-knockdown MRC5 cells were significantly enriched in the upregulated proteins secreted by exosome-treated MRC5 cells (NES = 1.199, FWER *P* value = 0; Fig. 7H), and the 37 downregulated secreted proteins were significantly enriched in the downregulated proteins secreted by exosome-treated MRC5 cells (NES = − 1.307, FWER *P* value = 0; Fig. 7I).

To further validate the effects of the fibroblast-derived secretome from FOXN3-downregulated fibroblasts on tumor cell proliferation and migration, we performed wound-healing assays and soft agar colony-forming assays using fibroblast-derived conditioned medium, which demonstrated that the fibroblast-derived secretome of FOXN3-knockdown MRC5 cells promote the proliferation and migration of A549 cells (Fig. 7J and K). To assess the contribution of FOXN3-downregulated fibroblasts to tumor progression in vivo, we labeled A549 cells with luciferase-expressing vectors (A549-Luc), enabling tracing of A549 cells in mice via an in vivo imaging system (IVIS) after luciferin administration (the substrate of luciferase). A549-Luc cells were subcutaneously injected into nude mice. After two weeks, the tumor mass formed, and MRC5 cells expressing *FOXN3* shRNAs (in vivo shFOXN3 group, *n* = 8), MRC5 cells expressing scrambled shRNAs (in vivo shScramble group, *n* = 8), or PBS (blank group, *n* = 8) were injected into the peritumoral sites. After another two weeks, the IVIS results revealed that MRC5 cells stably expressing *FOXN3* shRNAs significantly promoted tumor progression, as indicated by the highest fluorescence intensity in the shFOXN3 group (Fig. 7L). These results indicate that fibroblasts activated by tumor exosomes promote cancer progression via their secretion, which is primarily mediated by exosomal microRNAs target *FOXN3*.

### A strategy for identifying drugs sensitive to highly proliferative tumor cells on the basis of the identified secreted proteins

**Fig. 8:**
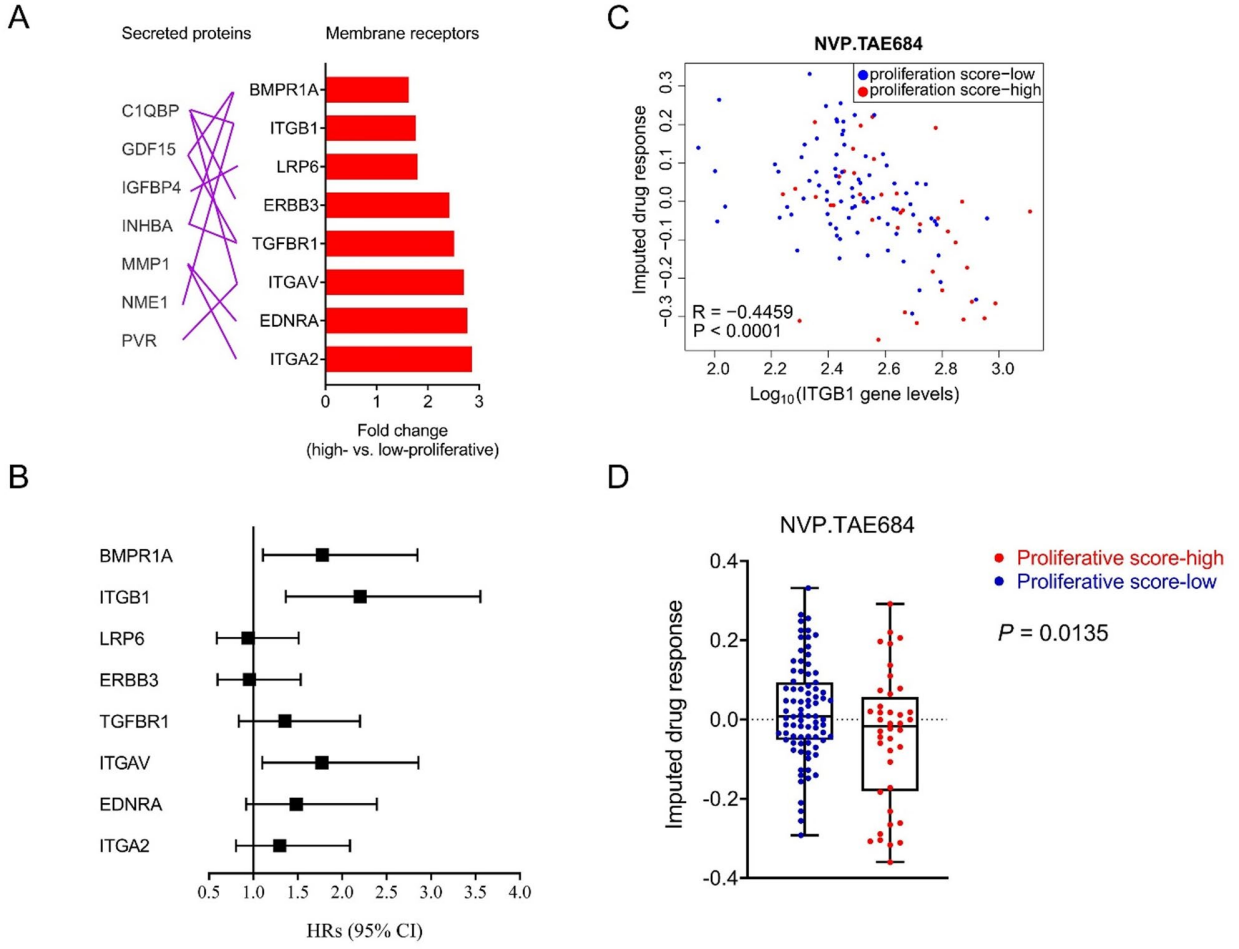
Identification of drugs sensitive to malignant proliferating tumor cells. (**A**) Purple lines connect secreted proteins to their cognate cell receptors. The right panel displays receptors with upregulated mRNAs (fold change [high- vs. low-proliferative] > 1.5, *P* value < 0.2; GSE111907), indicated by red bars. (**B**) Hazard ratios for overall survival based on the mRNA expression of membrane receptors in the TCGA-LUAD cohort. (**C**) Spearman’s correlation analysis between *ITGB1* mRNA levels and the imputed drug response to NVP.TAE684 in the TCGA-LUAD cohort. Red points denote high-proliferative tumors, and blue points denote low-proliferative tumors. (**D**) Value of the imputed drug response of NVP.TAE684 expression was significantly higher in the high-proliferative score group than in the low-proliferative score group within the TCGA-LUAD cohort (*P* value = 0.0135). The box shows the median ± 1 quartile, with whiskers extending from the hinge to the smallest or largest value within the 1.5 interquartile range from the box boundaries

Blocking the crosstalk between tumor cells and fibroblasts by inhibiting tumor cell surface receptors that bind to proteins secreted by fibroblasts represents a potential therapeutic strategy for tumors. Therefore, we assessed the associations between the expression of receptors bound by the 20 upregulated secreted proteins shown in Fig. 7F and their response to 138 Food and Drug Administration (FDA)-approved anticancer drugs. We used a cell‒cell interaction database, i.e., cellphoneDB (https://www.cellphonedb.org/), interaction files from the Bader laboratory (version 1.0; https://baderlab.org/CellCellInteractions#ref6), and a list of membrane receptors curated by Worzfeld et al. [54] to identify receptors that bind to the 20 highly expressed secreted proteins. We retained the receptors whose gene expression levels were upregulated (fold change [tumor with proliferative score-high vs. -low] > 1.5; *P* value < 0.2) in highly proliferative tumor cells compared with those in lowly proliferative tumor cells, on the basis of expression profiles of tumor cells sorted by EPCAM^+^ and CD45^−^ from GSE111907 (Fig. 8A).

To identify risk receptors and predict drugs sensitive to these receptors, we next calculated the HRs of the genes encoding the receptors via prognostic information from LUAD patients in the TCGA-LUAD cohort. The HRs of 3 genes were greater than 1, with *ITGB1* having the highest HR (Fig. 8B). Using the “imputed drug-wide association study (IDWAS)” approach [55], we found that *ITGB1* expression was negatively correlated (drug sensitive) with the response to NVP.TAE684, an inhibitor known to induce cell cycle arrest and apoptosis [56] (*R*_*s*_ = − 0.4459, *P* value < 0.0001; Fig. 8C; Table S7). Furthermore, tumors with high proliferative scores were indeed more sensitive to NVP.TAE684 (*P* value = 0.0135) (Fig. 8D). This analysis provides a method to identify receptors involved in the malignant proliferation of tumor cells and predict sensitive targeted drugs on the basis of the identified secreted proteins.

**Fig. 9:**
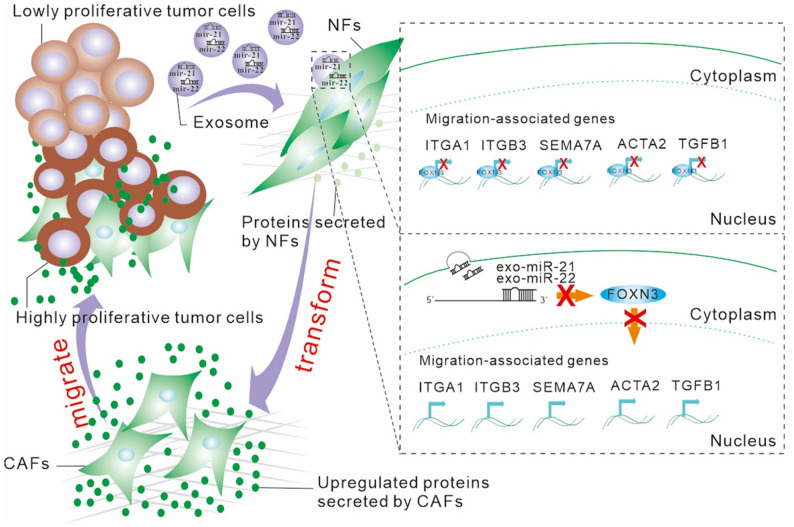
Schematic diagram of exosome-mediated tumor cell–CAF crosstalk that promotes tumor proliferation. The delivery of NSCLC cell-derived exosomal microRNAs into fibroblasts results in fibroblast activation and the enhancement of fibroblast motility by downregulating *FOXN3* expression levels, which contributes to the migration of fibroblasts into the tumor mass. Tumor cells in tumor tissue with a relatively high proportion of CAFs have increased proliferation ability in response to ECM-associated proteins aberrantly released by migrated CAFs

In summary, our study revealed that a set of highly expressed NSCLC cell-derived exosomal microRNAs, including miR-21-5p, miR-21-3p, and miR-22-3p, transform lung fibroblasts into CAFs by downregulating FOXN3 expression. A group of FOXN3-directly/indirectly regulated genes associated with migration and ECM remodeling subsequently contribute to fibroblast migration into tumor tissue. CAFs, in turn, further promote malignant tumor proliferation via abnormally secreted proteins that act in a paracrine manner (Fig. 9).

## Discussion

Owing to the influence of the external environment and the genetic defects of the cell itself, normal lung epithelial cells are prone to transform into tumor cells [57, 58]. Malignant proliferation is a key driving force for tumor progression [1, 2, 59]. During in vitro culture of tumor cells, a continuous supply of serum and culture medium enables tumor cells to proliferate rapidly, whereas in vivo, tumor cells require continuous “nutrition” from the surrounding stromal cells for their proliferation. As the main component of stromal cells, fibroblasts are responsible for the secretion of growth factors and ECM proteins into the microenvironment to provide necessary molecular crosstalk and structural support for tumor cell growth [3]. During the development of tumors, continuous communication between tumor cells and fibroblasts is a necessary process that promotes the malignant proliferation of tumor cells [3, 5, 60, 61]. Therefore, revealing the molecular mechanism and key regulatory genes involved in this process provides a mechanistic framework for developing stromal-targeted therapeutic modalities with translational potential.

In the TME, the origin of CAFs has always been controversial. Some studies in breast, gastric, and prostate cancers indicate that progenitors from bone marrow are the source of CAFs [6–8, 62, 63]. However, Arina et al. reported that most CAFs originate from local sessile precursors in colon cancer‌. Pathological sections showed that fibroblasts implanted subcutaneously, 2.5–6 mm away from the tumor tissue, could appear inside the tumor tissue [9]. Although CAFs may originate from multiple sources, a shared characteristic after local or distant cells transform into CAFs is the enhancement of their migratory ability, enabling more CAFs to infiltrate into the tumor.

Here, we revealed the mechanism by which local fibroblasts enter the tumor mass at the molecular level. We believe that through the paracrine diffusion of tumor exosomes, local NFs are converted into CAFs and that increasing the migration ability of CAFs is a prerequisite for their entry into the tumor mass. The higher the migration ability of CAFs is, the easier it is for them to enter tumor tissues. A large amount of clinical sequencing data has confirmed that, compared with tumor tissues with low proliferative capacity, tumor tissues with high proliferative capacity have a larger proportion of CAFs, and the migration ability of CAFs is significantly higher. In addition, the process by which local fibroblasts are transformed by tumor exosomes and migrate into the tumor mass is similar to the biological process by which fibroblasts participate in epithelial tissue damage repair. Under physiological conditions, once an injury signal appears, fibroblasts are activated and migrate to the wound site. Tumors are referred to as wounds that never heal. Fibroblasts are activated by tumor cell-derived exosomes and migrate into the tumor mass to repair so-called wounds via abnormally secreted proteins. Tumors hijack this physiological mechanism to establish a microenvironment for rapid proliferation.

The FOX family in mammals can be categorized into 19 subfamilies, from FOXA to FOXS, according to the sequence homology of their forkhead domains [64, 65]. In addition to the forkhead domain, other domains, such as the activation and repression domains, are poorly conserved. Under normal physiological conditions, the FOX family is responsible for regulating tissue development and cell metabolism [33, 66]. Conversely, abnormal expression and dysfunction of the FOX family can cause tumorigenesis [34]. For example, FOXP3^+^ regulatory T cells have an immunosuppressive function, which may promote the immune escape of cancer cells and the occurrence and development of tumors [67]. FOXC1 promotes hepatocellular carcinoma cell proliferation and metastasis through the formation of a ROS-FOXC1-cysteine metabolism-ROS positive feedback loop [68]. Compared with those of FOXA, FOXC, FOXO, FOXP and other transcriptional activators, the role of the transcriptional repressor FOXN protein in the progression of different types of tumors and its related molecular mechanisms have been less studied. In this study, FOXN3 was confirmed to be the target whose downregulation by exosomal microRNAs mediates the conversion of NFs to CAFs. Only a few existing studies have shown that FOXN3 is aberrantly expressed in several types of tumor cells. For example, the long noncoding RNA NEAT1 interacts with the FOXN3-SIN3A complex to form the FOXN3-NEAT1-SIN3A repressor complex to promote the progression of ERα^+^ breast cancer by inhibiting the expression of the GATA3 and TJP1 genes, which are responsible for maintaining luminal epithelial cell polarity and differentiation [34]. The downregulation of FOXN3 can also upregulate PIM2 expression to promote the proliferation of U2OS cells and H1299 cells [69] and is correlated with the oncogenesis of leukemia, tongue squamous carcinoma, osteosarcoma, and breast cancer [70–73]. In our study, we revealed in detail the mechanism by which FOXN3 is involved in LUAD progression through regulating the gene expression of fibroblasts. We found that the downregulation of FOXN3 promoted the malignant transformation of NFs and increased their migration into the tumor mass, which demonstrated that CAFs in tumor tissues are derived from local fibroblasts at the molecular level. This study expands our understanding of the distinct functions of different subtypes of FOX family proteins. The roles of other unreported FOX family proteins in tumor progression warrant further investigation.

Given the importance of CAF-secreted protein binding to tumor cell receptors in promoting malignant proliferation, we reasoned that targeting these receptors is a viable strategy for drug development. Thus, we propose a strategy for identifying potential receptor targets that are bound to significantly upregulated proteins secreted by CAFs in tumors with high proliferative capacity. We subsequently screen drugs on the basis of the sensitivity of the targets to the drugs. In addition, as one ligand can bind to several receptors on tumor cells, targeting a single receptor may lead to compensatory activation of alternative receptors, resulting in disappointing therapeutic outcomes [74–77]. Hence, rational combination therapies targeting multiple receptors are necessary. This strategy can provide more candidate targets for combined treatments.

In our study, we meticulously selected fibroblast markers to account for the inherent heterogeneity of the lung tumor microenvironment. For transcriptomic analyses of bulk tumor tissues and sorted patient samples, we utilized *COL10A1* and *FIGF* as high-specificity markers between activated CAFs and quiescent NFs, respectively. This selection was based on landmark single-cell RNA sequencing (scRNA-seq) data in lung cancer, which identified *COL10A1* as a robust marker for matrix-producing CAF subpopulations, while *FIGF* specifically marks the quiescent resident fibroblast state. While α-SMA remains the gold-standard marker for validating fibroblast activation in controlled in vitro functional assays, its utility in characterizing complex in vivo tissues is often confounded by its broad expression across multiple CAF lineages, including those derived from epithelial or endothelial transitions (EMT/EndMT) [61]. By prioritizing *COL10A1*, we focused our in vivo analysis on matrix-producing CAFs whose developmental trajectories stem directly from resident tissue fibroblasts, strictly adhering to definitions established by recent scRNA-seq mapping of the lung tumor microenvironment. Furthermore, to bridge our in vitro and in vivo findings, we employed the *ACTA2*/*FIGF* ratio as a quantitative indicator of CAF enrichment. The significant elevation of this ratio in tumor tissues with high proliferative scores across the TCGA-LUAD, GSE87340, and GSE40419 cohorts independently corroborates our *COL10A1*-based analyses (Figure S8). Collectively, this multi-marker approach ensures that the observed association between *FOXN3* suppression and CAF activation is both robust and specific to the lung adenocarcinoma stroma.

Notably, with advancements in single-cell sequencing technology, CAFs have been classified into several subtypes on the basis of specific molecular markers. Lambrechts et al. presented a comprehensive catalog of stromal cells in human lung tumors at single-cell resolution and classified fibroblasts into 7 subgroups, revealing that the lung TME is more complex and heterogeneous than previously appreciated [16]. However, only the annotation of CAF subtypes identified by single-cell sequencing has been performed. There is no relevant experimental evidence for the origin of each subtype of CAFs. Here, the CAFs that can be transformed by exosomes and migrate into the tumor mass are likely just one type among the diverse types of fibroblasts present in the tumor tissue. However, the specific subtype they represent and the origin of the NFs that can transform into this CAF subtype remain unknown and require further study.

While our initial mechanistic investigations primarily utilized the A549-MRC5 co-culture system, we recognized that reliance on a single cell line pair might limit the generalizability of our findings. To definitively establish the universality and broader relevance of the exosomal miRNA-*FOXN3* axis, we extended our validation across multiple independent models. We demonstrated that the selective enrichment of miR-21-3p, miR-21-5p, and miR-22-3p in exosomes is a conserved feature across different LUAD molecular subtypes, as evidenced by similar microRNA profiles in exosomes derived from the EGFR-mutant H1975 cell line (Figure S9A). Crucially, the functional impact of these exosomes was not limited to embryonic fibroblasts (MRC5); primary adult human lung fibroblasts (HPF-a) similarly underwent CAF-like activation, characterized by *FOXN3* downregulation and *α-SMA* induction, upon exposure to either A549- or H1975-derived exosomes (Figure S9B). Direct functional validation in HPF-a cells further confirmed that *FOXN3* knockdown is sufficient to drive a comprehensive CAF-like phenotype initially characterized in our A549-MRC5 model, including enhanced migratory capacity and the upregulation of *TGF-β1* and extracellular matrix (ECM) remodeling genes such as *ITGB3*, *ITGA1*, and *FN1* (Figure S9C-E). Collectively, these findings across independent LUAD subtypes and primary adult fibroblast models underscore that the *FOXN3*-CAF axis is a robust and conserved driver of the tumor microenvironment in lung adenocarcinoma, irrespective of the specific genetic background of the cancer or the developmental stage of the resident fibroblasts.

In summary, our study elucidates how nearby fibroblasts are transformed by tumor cells, enabling their migration and proproliferative functions within the tumor. More importantly, targeting the critical steps in fibroblast activation, migration, and tumor cell receptors that bind CAF-secreted proteins could yield promising therapies for aggressive LUAD.

## Materials and methods

### Patient samples

Our study enrolled patients from the Peking Union Medical College Hospital (Beijing, China). Preoperative blood was collected the day before surgical resection of the primary tumor, and postoperative blood was collected the third day after surgical resection. Peripheral blood in EDTA vacuum blood collection tubes was centrifuged at 1500 × g for 15 min at 4 °C. The supernatant was retained and stored at −80 °C. The healthy donors included in our study were volunteers who underwent routine physical examinations without lung disease or viral infection. Blood samples from healthy donors were collected as controls. Written informed consent was obtained from all the participants, and study protocol was approved by the clinical research ethics committee of the Beijing Institute of Genomics, Chinese Academy of Sciences (2018S001).

### Cell culture

MRC5 cells (RRID: CVCL_0440; Cat No. 1101HUM-PUMC000044) and H1975 cells (Cat. No. 1101HUM-PUMC000252) were purchased from the National Infrastructure of Cell Line Resources (Beijing, China) and cultured in minimum essential medium (MEM; Cat No. 41500-034; Gibco, USA) supplemented with GlutaMAX-I (Cat No. 35050-061; Gibco, USA). A549 cells (RRID: CVCL_0023; Cat No. CCL-185) and HEK293T cells (RRID: CVCL_0063; Cat No. CRL-3216) were both purchased from American Type Culture Collection (ATCC, USA) and cultured in RPMI-1640 medium (Cat No. 31800-022; Gibco, USA) and Dulbecco’s modified Eagle’s medium (DMEM; Cat No. 12800-058; Gibco, USA), respectively. HPF-a cells (Cat. No. ScienCell #3310) were purchased from the ScienCell Research Laboratories (Cat No. #3310; ScienCell, USA) and cultured in DMEM medium. All media were supplemented with 10% fetal bovine serum (FBS) (Cat No. 10270-106; Gibco, USA), 100 U/mL penicillin, and 100 U/mL streptomycin (Cat No. 15140-122; Gibco, USA). All the cell lines were cultured in an incubator containing 5% CO_2_ at 37 °C. All cell lines used were confirmed to be free of mycoplasma contamination and were authenticated.

### Isolation and identification of exosomes

Exosomes were isolated from media cultured with A549 cells. Briefly, A549 cells were first seeded into 10 cm dishes and cultured until 80% confluency. The cells were then washed with phosphate-buffered saline (PBS) twice and incubated with serum-free medium for another 48 h. The medium was collected, centrifuged at 3000 × g for 15 min and 16,000 × g for 1 h to remove cellular debris, and concentrated to 20% of its original volume via an Ultra15 centrifugal filter concentrator (Cat No. UFC900396; Millipore, Germany). After being filtered through 0.22 μm filters (Millipore), the concentrate was subjected to ultracentrifugation at 120,000 × g for 2 h (Beckman Coulter, USA). For isolation from the plasma of patients, 4 mL of plasma was centrifuged at 3000 × g for 10 min to remove cell debris. After being filtered through 0.22 μm filters, the samples were centrifuged at 120,000 × g for 2 h. The resulting exosome-enriched pellets were washed twice and resuspended in PBS or MEM. Exosomes were observed with a JEM 1010 transmission electron microscope (JEOL, Japan) at an acceleration voltage of 80 kV. The size distribution of the exosome-enriched pellets was directly tracked via a NanoSight NS 300 (Malvern, UK).

### Animal studies

Four-week-old female BALB/c nude mice received a subcutaneous injection of 1 × 10^6^ luciferase-labeled A549 cells into the right flank. After two weeks, the mice were randomly divided into three groups, and each group received peritumoral injections of 1 × 10^6^ eGFP-labeled MRC5 cells with *FOXN3* shRNA, 1 × 10^6^ eGFP-labeled MRC5 cells with scrambled shRNA, or an equal volume of PBS. Two weeks post-injection, the tumor sizes were measured, and the tumors were quantified via ex vivo bioluminescent imaging via an IVIS (Caliper LifeScience, USA). The tumors were subsequently excised for H&E staining and immunofluorescence assays. All experiments involving laboratory animals were conducted in accordance with protocols approved by the Laboratory Animal Science Department, Peking University Health Science Center. Animal care and use followed the ethical guidelines of the Chinese Council on Animal Care and were reviewed and approved by Beijing Institute of Genomics, Chinese Academy of Sciences (2018S001).

### RNA extraction and RT‒qPCR

Total RNA was extracted from cells or exosomes via TRIzol reagent (Cat No. 15596-018; Invitrogen, USA) according to the manufacturer’s protocol. mRNA was reverse-transcribed via the Reverse Transcription System (Cat No. A3500; Promega, USA) and quantified via SYBR Green PCR Master Mix (Cat No. K0251; Thermo, USA). The microRNA was reverse-transcribed via a miScript Reverse Transcription Kit (Cat No. 218061; Qiagen, USA) and quantified via a miScript SYBR Green PCR Kit (Cat No. 218073; Qiagen, USA). Quantification assays were performed on a CFX96 Real-Time PCR detection system (Bio-Rad, USA). The sequences of all the primers are listed in Table S8.

### Western blotting

The protein concentration of the exosome-enriched pellets was determined via a MicroBCA kit (Thermo, USA). After immunoblotting on a nitrocellulose filter, the transferred proteins were incubated with specific antibodies. The immunocomplex was incubated with a horseradish peroxidase (HRP)-conjugated secondary antibody and detected with enhanced chemiluminescence (ECL) reagents (Cat No. RPN2232, GE Healthcare, Sweden). The primary antibodies used were as follows: CD63 (1:1400; Cat No. ab118307; Abcam, USA), TSG101 (1:1000; Cat No. 612697; BD Biosciences, USA), β-Tubulin (1:500; Cat No. 2148S; Cell Signaling Technology, USA), and calnexin (1:2000; Cat No. 10427-2-AP; Proteintech, USA).

### Luciferase reporter assay

The luciferase construct containing *FOXN3* with the wild-type or a mutated version of the binding site was cotransfected with a microRNA mimic (miR-21-5p, miR-21-3p, miR-22-3p, or miR-320a) into HEK293T cells. After transfection for 24 h, luciferase activity was detected via the Dual-Luciferase Reporter Assay System (Cat. No. E1910; Promega, USA) according to the manufacturer’s instructions. The primers used to construct the luciferase reporter vectors are listed in Table S8.

### RNA interference assay

Mimics and antagomiRs targeting specific microRNAs, along with a scrambled negative control, were designed and synthesized by RiboBio (Guangzhou, China). To investigate the direct effects of miRNAs, MRC5 cells were transfected with mimics of miR-21-5p, miR-21-3p, miR-22-3p, miR-320a, or scrambled control. After 24 h, FOXN3 mRNA expression was quantified via qPCR. Similarly, A549 cells were transfected with antagomiRs against miR-21-5p, miR-21-3p, miR-22-3p, or the scrambled control. After 24 h, A549-derived exosomes were collected as described in the ‘isolation and identification of exosomes’ section. After incubation with the collected exosomes for 24 h, MRC5 cells were harvested for analysis of *FOXN3* expression.

### Collagen contraction assay

A total of 2 × 10^5^ MRC5 cells were suspended in 100 µL of MEM. The cell suspension was then mixed with 100 µL of a collagen mixture containing 69 µL of MEM, 1 µL of 1 mol/L NaOH, and 31 µL of Type I Rat Tail Collagen (Cat No. 08-115, Millipore, USA) and added to a 24-well plate to solidify for 45 min at 37 °C. After being incubated with medium containing A549-derived exosomes or a blank control for 72 h, the gels were photographed, and the contracted area was measured via Image-Pro Plus software (version 6.0).

### Soft agar colony formation assay

First, a bottom layer was prepared by adding 1 mL of RPMI-1640 medium containing 1% agar (Cat No. A1296, Sigma, USA) to each well of a 6-well plate and allowing it to solidify. A549 cells (1 × 10^5^ cells/well) were subsequently suspended in 1 mL of RPMI-1640 medium containing 0.35% low-melt agarose (Cat No. A9414; Sigma, USA). This cell suspension was layered on top of the solidified base layer to form the top layer. Next, 1 mL of culture medium from MRC5 cells with *FOXN3* shRNAs, 1 mL of culture medium from MRC5 cells with scrambled shRNAs, or 1 mL of control medium (MEM) was added to 6-well plates. After incubation for 4 weeks, A549 cells were stained with 0.005% crystal violet (Cat No. C0775, Sigma, USA). The number of spheroids larger than 0.5 mm in diameter was counted, and representative images were captured.

### Transwell assay

MRC5 cells (5 × 10^4^) were suspended in serum-free medium and seeded into the upper chambers (Cat No. 3422, Corning, USA). The lower chambers were filled with medium containing 1% FBS. Then, A549-derived exosomes were added to the upper chambers at the time of seeding. After 16 h, the cells were fixed with 4% paraformaldehyde for 5 min at room temperature and stained with 0.005% crystal violet (Cat No. C0775, Sigma, USA). Representative images were captured by a phase-contrast microscope, and the number of migrating cells was quantified by counting five randomly selected visual fields. Similarly, to detect the role of FOXN3 in cell migration, 5 × 10^4^ MRC5 cells transfected with *FOXN3* shRNAs and MRC5 cells transfected with scrambled shRNAs were separately plated into the upper chambers in serum-free medium, with the lower chambers containing 1% FBS medium. After 16 h, the cells were fixed and stained. The number of migrating cells was quantified.

### Wound-healing assay

When the A549 cells covered the 6-well plates, the monolayers were wounded with a pipette tip to create a gap in the plates. After washing with PBS to remove detached cells, the wells were replenished with 2 mL of culture medium from MRC5 cells with *FOXN3* shRNAs, 2 mL of culture medium from MRC5 cells with scrambled shRNAs, or 2 mL of a blank control (MEM). Next, the migration of A549 cells into the cleared section was observed and captured at 0 h, 24 h, and 48 h. The migratory ability of the cells was quantified by measuring the percentage of wound closure at each time point via Image-Pro Plus software (version 6.0).

### Migration of fibroblasts into the tumor mass

A 20 µL suspension of RFP-labeled A549 cells (2.5 × 10^4^ cells/mL) was pipetted onto the lid of a 96-well plate, and the lid was then inverted to form hanging drops. The tumor spheroids that formed were cultured for 5 days. On the 5th day of culture, a 10 µL of MRC5 cell suspension (5 × 10^4^ cells/mL) was added to the hanging drop containing the A549 spheroid to form an exterior coating around the inner core. These MRC5 cells consisted of two groups: one group was transfected with vectors expressing eGFP and scrambled shRNAs, and the other group was transfected with vectors expressing eGFP and *FOXN3* shRNAs. The cocultures were incubated at 37 °C. After incubation for 15 min, 24 h, 48 h, or 72 h, the spheroids in the hanging drops were imaged via fluorescence microscopy (Leica, Germany).

### CUT&RUN

The CUT&RUN procedure was conducted in accordance with the manufacturer’s protocol for the CUT&RUN Assay Kit (Cat No. 86652; Cell Signaling Technology, USA). Briefly, the cells were grown to 80% confluence, harvested from fresh cultures, and counted. Approximately 200,000 cells were used per CUT&RUN sample. The cells were washed and incubated for 5 min at room temperature with 10 µL of concanavalin A-coated beads. The cells bound to the beads were permeabilized with digitonin buffer and incubated with an anti-FOXN3 antibody (Cat No. AP19255B; Abgent, USA) at 4 °C overnight. The cells were incubated with 50 µL of pAG-MNase at 4 °C for 1 h. Next, pAG-MNase was activated with 3 µL of precooled CaCl_2,_ and digestion was performed for 30 min at 4 °C. The reaction was stopped with 150 µL of stop buffer for 10 min at 37 °C. The release of the DNA fragments was achieved by incubating samples with 3 µL of 10% SDS and 2 µL of proteinase K at 65 °C for 2 h. DNA was extracted via DNA purification buffers and spin columns (Cat No. 14209; Cell Signaling Technology). Extracted DNA was used for Illumina high-throughput sequencing. For sequencing, barcoded CUT&RUN-seq libraries were constructed via the *NEBNext Ultra II DNA* Library Prep Kit (Cat. No. E7645, NEB, USA) following the manufacturer’s instructions. High-throughput sequencing of libraries was performed on an Illumina NovaSeq 6000, with 2 × 150 bp paired-end reads. After demultiplexing according to their index barcode, the total number of reads ranged from 4.7 to 5.7 million per library. The reads were aligned to the GRCh37 (hg19) human genome assembly via Bowtie 2 (version 2.3.4.2) [78], with a paired-end option fragment length for valid paired-end alignments set from 10 to 700, and otherwise default options. Peak predictions for FOXN3 occupancy were performed with model-based analysis for ChIP-seq MACS2 (version v2.1.1.20160309.0) [79], with the following parameters: -f BAMPE, --bw 300, -m and -p set to default values and --bdg. The samples were analyzed against the control, rabbit IgG sample. The read density was visualized with the UCSC genome browser, and heatmap profiles of FOXN3 occupancy were generated via deepTools (version 2.0.1) [80]. The heatmaps show samples for each condition without normalization.

### Protein identification and quantification

MS sequencing was performed at the National Center for Protein Sciences (Beijing, China). Raw data from triplicate samples were searched against a UniProt human database (January 2018) via MaxQuant software (version 1.6.1.0). LFQ values represent relative protein abundance. Only proteins quantified in at least two out of three replicates were retained. The detailed methods for characterizing the abundance of proteins secreted by fibroblasts are described in our previous study [13].

### H&E staining and immunofluorescence

H&E staining and immunofluorescence assays were performed at the Experimental Animal Pathological Laboratory, Institute of Biophysics (Beijing, China). In brief, tumor tissues were fixed in 10% neutral buffered formalin, embedded in paraffin, and sectioned at a thickness of 4 µm. For H&E staining, the sections were processed via standard protocols. For the immunofluorescence assay, the sections were incubated with a primary antibody against eGFP (1:1000; Cat No. 2956; Cell Signaling Technology, USA) overnight at 4°C and then incubated with an HRP-conjugated secondary antibody (1:500; Cat No. PV-6001; Zhongshanjinqiao, China) for 1 h at 37°C. The eGFP signal was subsequently amplified and visualized via an OPAL 7-color fIHC kit (Cat No. NEL797001KT; Perkin Elmer, USA) according to the manufacturer’s protocol. Finally, the sections were counterstained with 4’-6-diamidino-2-phenylindole (DAPI), mounted, and imaged under a fluorescence microscope (Leica, Germany).

### Classification of proliferation status

We adopted an 11-gene proliferation signature (MKI67, NDC80, NUF2, PTTG1, RRM2, BIRC5, CCNB1, CEP55, UBE2C, CDC20, TYMS) that has been shown to perform the best when classifying proliferation status. A proliferation score for each tumor sample or cancer cell line was calculated via gene set variation analysis (GSVA) on the basis of the expression of these 11 genes. Significant differences between highly proliferative and lowly proliferative cancer cell lines were assessed via Student’s *t* test. For the LUAD samples, the proliferative status was classified via unsupervised hierarchical clustering on the basis of the 11-gene signature. The three subclusters were designated the proliferative score-high, proliferative score-intermediate, and proliferative score-low groups. To avoid confounding factors from the potential mixture, samples from the proliferative score-intermediate group were excluded from subsequent analyses.

### Functional annotation

GSEA (version 2.0) [81] was used to calculate the enrichment score of exosomal microRNA targets within the set of downregulated genes from the tumor stroma. The FWER *P* value threshold for statistical significance was set at 0.05. GO and KEGG enrichment analyses were performed via DAVID (version 6.7) online tools [82]. The enrichment significance of a gene set with a specific term was based on Fisher’s exact test, and the *P* value was adjusted by the Benjamini and Hochberg method and was set at 0.05. PPI data were obtained by querying the Interologous Interaction Database (I2D, version 2.9). The PPI network was visualized via Cytoscape software (version 2.8.2).

### Sample similarity analysis by multidimensional scaling (MDS)

MDS analysis, a dimensionality reduction method used to calculate the distance between samples to assess their similarity, is used to visualize the similarity/dissimilarity of the samples. The Euclidean distance is measured on the basis of the gene expression level or the abundance of proteins. The coordinates of the samples in the first two dimensions of the MDS are displayed.

### Sequencing and data analyses

mRNA sequencing (mRNA-Seq) and microRNA sequencing (miRNA-Seq) were both conducted on the HiSeq 2000 platform (Illumina, USA). For mRNA-Seq analysis, raw reads were aligned to the human reference genome (hg19) via TopHat (version 2.0.9) [83] and Bowtie (version 2.1.0) [84] with default parameters. The reads aligned to the specific gene were counted and annotated via HTseq (version 0.5.4) [85] with the parameters –s no –a 10. DEGseq (version 1.14.0) [86] was used to search for differentially expressed genes (fold change ≥ 1.2 and FDR ≤ 0.05; see Tables S1 and S4). The data analysis for miRNA-Seq was performed via our established pipeline. Briefly, raw reads were first trimmed to remove 3’ end adaptors and converted to FASTA file format. The clean reads were then mapped to the human microRNA reference (miRBase, version 23) [87] via a basic local alignment search tool (BLAST) [88] with default parameters. Reads with no mismatches or at most 1 base deletion at the 3’ end were retained (see Tables S2 and S3).

### Survival analysis

Survival analysis was performed to assess the associations between proliferative scores and patient prognosis. Overall survival was compared across different score groups via Kaplan‒Meier curves, and the statistical significance of the differences was evaluated with the log-rank test via the ‘survival’ R package (version 2.39.2). A *P* value ≤ 0.05 was considered significant.

### Analysis of drug response

For the effects of *ITGB1* expression levels in LUAD patients from the TCGA-LUAD cohort on drug response, we downloaded the imputed tumor response to 138 FDA-approved anticancer drugs in LUAD patients from a previous study [57]. We calculated Spearman’s correlation coefficients between the imputed drug responses and *ITGB1* expression levels, considering |*R*_*s*_| > 0.4 and *P* value < 0.01 as statistically significant. To determine the effects of the proliferative status of LUAD patients on drug response, the proliferative score of each LUAD patient in the groups with high and low proliferative scores was calculated via GSVA. Spearman’s correlations were calculated between the imputed drug responses and proliferative scores.

### Statistical analyses

The data were analyzed via GraphPad Prism 8, Microsoft Excel, and R software (version 3.1.0). Except for special instructions, all experiments were carried out at least in triplicate, and all the statistical results are presented as the mean ± standard error of the mean (s.e.m.). Differences were assessed via Student’s *t* test (unpaired, two-tailed) for comparisons between two groups. For comparisons among multiple groups, differences were assessed via one-way analysis of variance (ANOVA) followed by Bonferroni correction. A *P* value ≤ 0.05 was considered significant.

## Supplementary Information

Below is the link to the electronic supplementary material.

Figures S1-S9

Table S1 mRNA profiles of exosome-treated MRC5 cells and untreated MRC5 cells

Table S2 miRNA profiles of A549-derived exosomes

Table S3 miRNA profiles of A549 cells

Table S4 mRNA profiles of MRC5 cells with FOXN3 shRNAs and MRC5 cells with scrambled shRNAs

Table S5 CUT&RUN data

Table S6 List of differentially secreted proteins

Table S7 ITGB1 vs drug response

Table S8 Sequences of primers and shRNAs

## Data Availability

The mRNA-Seq data and miRNA-Seq data that support the findings of this study have been deposited in the Gene Expression Omnibus (GEO; http://www.ncbi.nlm.nih.gov/geo) database under accession code GSE144178. CUT&RUN data have been deposited in the Genome Sequence Archive at the National Genomics Data Center, China National Center for Bioinformation/Beijing Institute of Genomics, Chinese Academy of Sciences, which are publicly accessible at https://ngdc.cncb.ac.cn/gsa-human/browse/HRA004494. The MS data have been deposited in iProX (https://www.iprox.cn/) with the primary accession codes IPX0001932000/PXD016888. Information on all primers is available in Table S8. The processed miRNA data are available in Tables S2 and S3. The processed mRNA data are available in Tables S1 and S4. The processed MS data are available in Table S6. All remaining data are included in the article and supplementary files or are available from the authors upon reasonable request.

## Abbreviations

CAFs: Cancer-associated fibroblasts
TME: Tumor microenvironment
ECM: Extracellular matrix
MSCs: Mesenchymal stem cells
SDF-1: Stromal cell-derived factor-1
GDF15: Growth differentiation factor 15
LIF: Leukemia inhibitory factor
LUAD: Lung adenocarcinoma
H&E: Hematoxylin and eosin
HR: Hazard ratio
NF: Normal fibroblast
GO: Gene ontology
NES: Normalized enrichment score
FWER: Family-wise error rate
qPCR: Quantitative PCR
FOXN3: Forkhead box N3
shRNAs: Short hairpin RNAs
KEGG: Kyoto encyclopedia of genes and genomes
PPI: Protein-protein interaction
MS: Mass spectrometry
LFQ: Label-free quantification
GSEA: Gene set enrichment analysis
IVIS: *In vivo* imaging system
FDA: Food and drug administration
IDWAS: Imputed drug-wide association study
