# Supplementary material for: The exosomal miRNA/FOXN3 axis empowers fibroblasts with tumor-promoting functions via enhanced migration and altered secretome
Source: J Nanobiotechnology. 2026 Jun 5;24:742. doi: 10.1186/s12951-026-04633-6 (PMC13459620; doi:10.1186/s12951-026-04633-6)
Supplement: Supplementary file 1 [file 12951_2026_4633_MOESM2_ESM.pdf]

Table S1 mRNA profiles of exosome-treated MRC5 cells and untreated MRC5 cells

Gene symbol	Count (untreated MRC5 [con])	Count (exosome-treated MRC5 [exo])	Log2(exo/con) normalized	*P* value	*Q* value (Benjamini et al. 1995)
FN1	0	2303611	22.02674131	0	0
COL6A3	0	395242	19.48365236	0	0
IGFBP5	0	282959	19.00150912	0	0
EEF2	0	119684	17.7601464	0	0
PXDN	0	107348	17.60321141	0	0
COL3A1	0	85422	17.27359568	0	0
CALR	0	67862	16.94159195	0	0
TPM4	0	62828	16.83039566	0	0
P4HB	0	57848	16.71125509	0	0
FTL	0	53998	16.61189398	0	0
COL5A2	0	51027	16.53024883	0	0
LTBP1	0	45807	16.37455608	0	0
TMSB10	0	41489	16.23171689	0	0
SERPINE2	0	39068	16.14497541	0	0
RPL37A	0	38107	16.10904404	0	0
CALM2	0	26998	15.61184054	0	0
RPS11	0	24381	15.4647453	0	0
RPL18A	0	23621	15.41905805	0	0
LBH	0	23183	15.39205527	0	0
CRIM1	0	22076	15.32146679	0	0
LMAN1	0	21686	15.29575197	0	0
RPL28	0	21235	15.26543211	0	0
ACTG2	0	20844	15.23862016	0	0
RTN4	0	19616	15.15101889	0	0
CNN2	0	18880	15.09584677	0	0
ACTN4	0	18715	15.08318305	0	0
RPS7	0	18240	15.04609373	0	0
EFEMP1	0	17659	14.99939165	0	0
GALNT5	0	17081	14.95138044	0	0

CDC42EP3	0	16770	14.92487069	0	0
RPS19	0	15842	14.84274249	0	0
EMILIN1	0	15821	14.84082879	0	0
BSG	0	15723	14.83186452	0	0
ITGAV	0	15659	14.82598009	0	0
ACTR2	0	15389	14.80088749	0	0
PLD3	0	14618	14.72673394	0	0
FAP	0	14140	14.67877012	0	0
MGAT5	0	14070	14.67161033	0	0
RPL31	0	13814	14.64511913	0	0
RPL36	0	13751	14.63852454	0	0
ANTXR1	0	13565	14.61887705	0	0
OAZ1	0	13420	14.60337268	0	0
TWSG1	0	12805	14.53569526	0	0
RPS5	0	12794	14.53445539	0	0
LAPTM4A	0	12790	14.53400427	0	0
EPAS1	0	11973	14.43877269	0	0
EHD2	0	11971	14.43853168	0	0
AXL	0	11910	14.43116142	0	0
UBA52	0	11664	14.40105063	0	0
RND3	0	11636	14.39758321	0	0
MYL12A	0	11569	14.38925217	0	0
SLC1A5	0	11251	14.34904124	0	0
ATP5A1	0	11152	14.33629047	0	0
RPS9	0	11043	14.32212016	0	0
RPS16	0	10973	14.31294601	0	0
ZAK	0	10557	14.25718792	0	0
MYO1B	0	10536	14.25431525	0	0
PRKCSH	0	10472	14.24552501	0	0
RPS28	0	10201	14.20769859	0	0
ACTR3	0	10147	14.20004126	0	0
NCL	0	10130	14.19762218	0	0

ARPC2	0	10061	14.18776171	0	0
SF3B1	0	9886	14.16244682	0	0
FHL2	0	9855	14.15791578	0	0
MCFD2	0	9210	14.06026107	0	0
KDELR1	0	9206	14.05963435	0	0
PCYOX1	0	9154	14.0514622	0	0
RPS15	0	9130	14.04767477	0	0
SH3BP4	0	9115	14.04530257	0	0
CAPNS1	0	8907	14.0119995	0	0
ITM2C	0	8771	13.98980125	0	0
PDIA6	0	8451	13.93618197	0	0
NCKAP1	0	8387	13.92521476	0	0
TGOLN2	0	8360	13.92056285	0	0
CHPF	0	8356	13.9198724	0	0
EEF1B2	0	8176	13.88845511	0	0
NUCB1	0	8155	13.88474479	0	0
AES	0	8025	13.8615613	0	0
C19orf10	0	8002	13.85742054	0	0
UGGT1	0	7867	13.83287349	0	0
SPTBN1	0	7785	13.81775695	0	0
MAP4K4	0	7754	13.81200064	0	0
RPL18	0	7746	13.81051141	0	0
GLS	0	7634	13.7894991	0	0
PTMA	0	7609	13.78476677	0	0
RPS27A	0	7476	13.75932648	0	0
SPATS2L	0	7371	13.73892027	0	0
BMPR2	0	7267	13.71841982	0	0
CYBRD1	0	7172	13.6994354	0	0
DCTN1	0	7122	13.68934234	0	0
XRCC5	0	7029	13.67037936	0	0
MYL12B	0	6984	13.66111347	0	0
HNRNPA3	0	6949	13.65386529	0	0

LIMS1	0	6890	13.64156389	0	0
TNS1	0	6871	13.63757999	0	0
TCF4	0	6756	13.61322924	0	0
GFPT1	0	6743	13.61045051	0	0
FKBP8	0	6721	13.60573581	0	0
TRIP12	0	6649	13.59019729	0	0
SSFA2	0	6562	13.5711955	0	0
BZW1	0	6559	13.57053578	0	0
TUBB6	0	6498	13.55705565	0	0
ARHGDIA	0	6463	13.5492639	0	0
STAT1	0	6457	13.54792394	0	0
SLC39A6	0	6237	13.49791217	0	0
ALPK2	0	6172	13.48279797	0	0
RAB1A	0	6159	13.47975604	0	0
USP34	0	6157	13.47928748	0	0
DNAJC10	0	6145	13.47647292	0	0
BIRC6	0	6111	13.46846839	0	0
CHN1	0	6054	13.45494858	0	0
YWHAQ	0	5923	13.42338799	0	0
ERLEC1	0	5815	13.3968391	0	0
SLC44A2	0	5799	13.39286405	0	0
FAM98A	0	5779	13.38787978	0	0
SLC16A3	0	5726	13.37458758	0	0
RCN3	0	5706	13.36953966	0	0
MTHFD2	0	5695	13.36675575	0	0
GPX4	0	5674	13.36142606	0	0
MEGF8	0	5638	13.35224339	0	0
FASN	0	5533	13.32512183	0	0
LTBP4	0	5526	13.32329547	0	0
PYCR1	0	5526	13.32329547	0	0
SNRNP200	0	5485	13.31255153	0	0
FAM168B	0	5437	13.29987074	0	0

HNRNPUL1	0	5391	13.28761282	0	0
KANK2	0	5315	13.2671296	0	0
HSPD1	0	5221	13.24138607	0	0
FZD7	0	5206	13.23723522	0	0
PPP1CB	0	5194	13.23390592	0	0
AP3D1	0	5167	13.22638679	0	0
XPO1	0	5143	13.21967006	0	0
TGFB1	0	5091	13.20500897	0	0
EPG5	0	5085	13.20330768	0	0
UBXN4	0	5029	13.18733146	0	0
TRIM28	0	5022	13.18532194	0	0
ILF3	0	5005	13.18042998	0	0
IRS1	0	4986	13.17494279	0	0
CCT4	0	4980	13.17320565	0	0
LDLR	0	4939	13.16127888	0	0
CLIP3	0	4904	13.15101889	0	0
GPI	0	4892	13.14748431	0	0
TMEM214	0	4859	13.13771934	0	0
TRAK2	0	4740	13.10194697	0	0
NOTCH3	0	4709	13.09248063	0	0
MYO9B	0	4701	13.09002759	0	0
NFE2L2	0	4694	13.08787775	0	0
DYNC1I2	0	4662	13.07800891	0	0
CIRBP	0	4617	13.06401564	0	0
MYADM	0	4598	13.05806637	0	0
NBAS	0	4593	13.05649669	0	0
NARS	0	4564	13.0473587	0	0
CTIF	0	4541	13.04006995	0	0
FKBP7	0	4529	13.03625245	0	0
PVRL2	0	4520	13.03338268	0	0
HADHA	0	4507	13.02922736	0	0
ACSL3	0	4506	13.02890722	0	0

GLT25D1	0	4467	13.01636616	0	0
CALM3	0	4326	12.97009357	0	0
STK17B	0	4300	12.96139657	0	0
AP2A1	0	4251	12.94486217	0	0
CCT7	0	4232	12.93839954	0	0
NFIC	0	4128	12.90250288	0	0
HS6ST1	0	4115	12.89795234	0	0
PLIN3	0	4109	12.89584724	0	0
RANBP2	0	4100	12.89268382	0	0
MAT2A	0	4084	12.88704277	0	0
GORASP2	0	4039	12.87105805	0	0
SCN9A	0	4034	12.86927099	0	0
PPP2R1A	0	4028	12.86712359	0	0
PSME4	0	3975	12.84801477	0	0
SMCHD1	0	3940	12.83525554	0	0
SMARCA4	0	3878	12.81237271	0	0
NFIX	0	3813	12.78798644	0	0
PSMD1	0	3765	12.76970977	0	0
MIB1	0	3753	12.7651042	0	0
PTBP1	0	3753	12.7651042	0	0
PVR	0	3718	12.75158668	0	0
FXYD5	0	3703	12.74575446	0	0
EHD3	0	3697	12.74341495	0	0
TMX3	0	3667	12.73166017	0	0
NPLOC4	0	3615	12.71105556	0	0
PTPRS	0	3612	12.7098578	0	0
ZFP36L2	0	3592	12.70184726	0	0
ATP8B1	0	3587	12.69983765	0	0
HNRNPL	0	3551	12.68528527	0	0
LSM14A	0	3536	12.67917818	0	0
MAP2K2	0	3530	12.67672809	0	0
FSTL3	0	3448	12.64281968	0	0

PCBP1	0	3429	12.63484781	0	0
RALBP1	0	3429	12.63484781	0	0
PRDX2	0	3426	12.63358506	0	0
MAP4K3	0	3405	12.62471471	0	0
RABAC1	0	3405	12.62471471	0	0
SLC35E1	0	3370	12.6098085	0	0
PLEKHB2	0	3353	12.60251239	0	0
SOGA2	0	3351	12.6016516	0	0
CDC37	0	3331	12.59301526	0	0
PSMD8	0	3324	12.58998029	0	0
KIDINS220	0	3313	12.58519811	0	0
DDX1	0	3294	12.57690046	0	0
AAK1	0	3292	12.57602424	0	0
EPN1	0	3283	12.57207466	0	0
NABP1	0	3280	12.57075572	0	0
CSNK1D	0	3259	12.56148926	0	0
COPE	0	3251	12.55794346	0	0
WDFY1	0	3238	12.55216289	0	0
ELP2	0	3230	12.54859407	0	0
UGP2	0	3228	12.54770049	0	0
NRP2	0	3225	12.54635907	0	0
SUMO1	0	3195	12.53287584	0	0
GLTSCR2	0	3186	12.52880618	0	0
RAB3GAP1	0	3178	12.52517903	0	0
ROCK1	0	3147	12.51103709	0	0
RAB10	0	3116	12.49675514	0	0
ZNF146	0	3116	12.49675514	0	0
SS18	0	3114	12.49582885	0	0
CDH2	0	3110	12.49397449	0	0
IDH1	0	3097	12.48793129	0	0
RHOQ	0	3083	12.4813948	0	0
TFPI	0	3082	12.48092677	0	0

DSEL	0	3074	12.47717707	0	0
DPY19L3	0	3062	12.47153419	0	0
CLASP1	0	3059	12.47012002	0	0
EML4	0	3052	12.46681487	0	0
TMEM131	0	3023	12.45304089	0	0
LENG8	0	3017	12.45017461	0	0
TIA1	0	3011	12.44730262	0	0
PRPF40A	0	2995	12.43961591	0	0
EIF5B	0	2970	12.42752284	0	0
SUPT5H	0	2938	12.4118943	0	0
COX7A2L	0	2929	12.4074681	0	0
SUGP2	0	2928	12.40697546	0	0
CLPTM1	0	2923	12.40450974	0	0
EMP3	0	2922	12.40401609	0	0
MBOAT7	0	2916	12.40105063	0	0
PIK3R2	0	2907	12.39659098	0	0
TMEM259	0	2885	12.38563123	1.53E-306	2.03E-305
PARD3B	0	2862	12.37408358	9.24E-305	1.22E-303
ARHGAP35	0	2847	12.3665024	1.35E-303	1.78E-302
SLC35F5	0	2815	12.35019483	4.15E-301	5.45E-300
PUM2	0	2809	12.34711653	1.22E-300	1.59E-299
RBMS1	0	2785	12.33473724	9.10E-299	1.18E-297
CLIP4	0	2780	12.33214479	2.24E-298	2.91E-297
STARD7	0	2778	12.33110651	3.21E-298	4.16E-297
PTOV1	0	2757	12.32015918	1.42E-296	1.83E-295
ZNF532	0	2728	12.30490355	2.68E-294	3.44E-293
MARCH4	0	2724	12.30278661	5.53E-294	7.07E-293
LPPR2	0	2722	12.30172698	7.95E-294	1.01E-292
CLEC11A	0	2719	12.30013606	1.37E-293	1.74E-292
MARCH7	0	2714	12.29748063	3.39E-293	4.30E-292
VASP	0	2711	12.29588502	5.84E-293	7.40E-292
YPEL5	0	2688	12.28359305	3.81E-291	4.80E-290

NRBP1	0	2670	12.27389965	1.01E-289	1.26E-288
SNX17	0	2668	12.27281858	1.46E-289	1.82E-288
ROCK2	0	2659	12.26794369	7.52E-289	9.35E-288
AFF3	0	2652	12.26414068	2.70E-288	3.35E-287
GGCX	0	2649	12.26250775	4.67E-288	5.77E-287
FCGRT	0	2638	12.25650447	3.49E-287	4.29E-286
SNRPD2	0	2636	12.25541028	5.03E-287	6.18E-286
EIF3G	0	2632	12.2532194	1.05E-286	1.28E-285
MMADHC	0	2624	12.24882763	4.53E-286	5.49E-285
IER3IP1	0	2622	12.24772759	6.53E-286	7.91E-285
WDR45B	0	2613	12.24276703	3.40E-285	4.10E-284
PRMT1	0	2594	12.23223839	1.12E-283	1.34E-282
TMBIM1	0	2593	12.23168211	1.34E-283	1.60E-282
ZNF24	0	2584	12.22666598	7.03E-283	8.39E-282
AP2S1	0	2554	12.20981843	1.78E-280	2.11E-279
MAN2B1	0	2539	12.2013203	2.85E-279	3.37E-278
KHSRP	0	2532	12.19733731	1.04E-278	1.23E-277
SNRNP70	0	2529	12.19562695	1.81E-278	2.14E-277
USP14	0	2527	12.19448557	2.63E-278	3.09E-277
SH3GL1	0	2523	12.19220012	5.52E-278	6.47E-277
NDUFA11	0	2496	12.17667784	8.31E-276	9.69E-275
KIAA0355	0	2491	12.17378493	2.11E-275	2.45E-274
DAPK3	0	2490	12.17320565	2.54E-275	2.95E-274
VAPA	0	2483	12.16914417	9.35E-275	1.08E-273
HSPB6	0	2479	12.16681818	1.97E-274	2.27E-273
TNPO2	0	2473	12.16332215	6.03E-274	6.93E-273
U2AF2	0	2468	12.1604023	1.53E-273	1.76E-272
PPP3R1	0	2466	12.15923271	2.23E-273	2.55E-272
ZNF638	0	2461	12.15630457	5.67E-273	6.47E-272
GALNT1	0	2447	12.14807401	7.77E-272	8.85E-271
NACC1	0	2439	12.14334967	3.48E-271	3.94E-270
SEPT10	0	2433	12.13979623	1.07E-270	1.21E-269

LRPPRC	0	2420	12.13206696	1.23E-269	1.38E-268
ATRAID	0	2415	12.1290831	3.14E-269	3.52E-268
PIKFYVE	0	2412	12.12728982	5.51E-269	6.17E-268
HADHB	0	2399	12.11949307	6.36E-268	7.07E-267
SLC39A10	0	2397	12.11828982	9.27E-268	1.03E-266
AKT2	0	2394	12.11648306	1.63E-267	1.81E-266
LANCL1	0	2388	12.11286275	5.05E-267	5.59E-266
CUL3	0	2387	12.11225848	6.10E-267	6.74E-266
UPF1	0	2386	12.11165395	7.37E-267	8.13E-266
TMEM237	0	2382	12.10923332	1.57E-266	1.72E-265
DNM2	0	2379	12.10741518	2.76E-266	3.03E-265
SAE1	0	2372	12.10316392	1.04E-265	1.13E-264
NDUFS1	0	2370	12.10194697	1.51E-265	1.65E-264
SPEG	0	2369	12.10133811	1.82E-265	1.99E-264
PKN1	0	2364	12.09828994	4.70E-265	5.12E-264
TMEM87B	0	2363	12.09767954	5.67E-265	6.18E-264
LONP1	0	2359	12.09523533	1.21E-264	1.31E-263
SSB	0	2356	12.09339945	2.13E-264	2.31E-263
SLC20A1	0	2355	12.09278697	2.58E-264	2.78E-263
SOS1	0	2354	12.09217423	3.12E-264	3.36E-263
PREPL	0	2350	12.08972067	6.65E-264	7.16E-263
MLLT1	0	2348	12.08849232	9.71E-264	1.04E-262
IMMT	0	2329	12.07677055	3.57E-262	3.81E-261
PTPRM	0	2316	12.06869516	4.23E-261	4.49E-260
CNN1	0	2312	12.06620131	9.05E-261	9.59E-260
SMAD4	0	2299	12.05806637	1.08E-259	1.14E-258
UBXN6	0	2296	12.05618255	1.91E-259	2.01E-258
AGPS	0	2291	12.05303737	4.96E-259	5.21E-258
SYDE1	0	2290	12.05240751	6.00E-259	6.29E-258
EIF3K	0	2271	12.04038761	2.27E-257	2.36E-256
COX5B	0	2250	12.02698491	1.27E-255	1.31E-254
PNPLA6	0	2246	12.02441784	2.74E-255	2.82E-254

ACP1	0	2239	12.01991444	1.05E-254	1.08E-253
TCF3	0	2218	12.00631927	5.98E-253	6.10E-252
MOGS	0	2213	12.00306336	1.57E-252	1.60E-251
TPGS2	0	2198	11.9932513	2.84E-251	2.87E-250
PLEKHG2	0	2186	11.98535331	2.90E-250	2.92E-249
UBL5	0	2172	11.97608401	4.36E-249	4.37E-248
BTBD2	0	2158	11.96675477	6.61E-248	6.58E-247
CFLAR	0	2146	11.95870998	6.83E-247	6.78E-246
HK2	0	2145	11.95803756	8.29E-247	8.22E-246
UBA2	0	2134	11.95062008	7.07E-246	6.98E-245
LPIN2	0	2133	11.94994387	8.59E-246	8.47E-245
RIOK3	0	2127	11.94587994	2.77E-245	2.73E-244
SP3	0	2119	11.9404435	1.32E-244	1.30E-243
AGFG1	0	2115	11.93771757	2.89E-244	2.83E-243
STEAP3	0	2096	11.92469863	1.19E-242	1.16E-241
COX6B1	0	2093	11.92263222	2.15E-242	2.09E-241
CAB39	0	2090	11.92056285	3.87E-242	3.75E-241
PIP5K1C	0	2087	11.91849051	6.97E-242	6.74E-241
EMC10	0	2080	11.91364344	2.75E-241	2.66E-240
SCAF1	0	2076	11.91086635	6.05E-241	5.82E-240
SMEK2	0	2075	11.91017125	7.36E-241	7.08E-240
BRD4	0	2069	11.90599355	2.40E-240	2.30E-239
PTPRN	0	2058	11.89830289	2.09E-239	2.00E-238
PPP6R1	0	2053	11.89479354	5.61E-239	5.36E-238
NDUFA13	0	2045	11.88916075	2.72E-238	2.59E-237
LMNB2	0	2038	11.88421396	1.08E-237	1.03E-236
ADI1	0	2034	11.88137959	2.39E-237	2.27E-236
RAB11FIP5	0	2028	11.87711756	7.84E-237	7.42E-236
LOXL3	0	2027	11.876406	9.56E-237	9.03E-236
TMEM127	0	2026	11.87569408	1.16E-236	1.10E-235
FBXO11	0	2016	11.86855555	8.45E-236	7.97E-235
POLR2E	0	2010	11.86425541	2.78E-235	2.61E-234

RAPH1	0	2007	11.86210053	5.04E-235	4.73E-234
SF3B14	0	2004	11.85994242	9.15E-235	8.57E-234
ASAP2	0	2003	11.85922233	1.12E-234	1.04E-233
ZNF516	0	2003	11.85922233	1.12E-234	1.04E-233
MEIS1	0	1978	11.84110233	1.62E-232	1.50E-231
FEZ2	0	1976	11.83964286	2.41E-232	2.23E-231
EIF2AK2	0	1974	11.8381819	3.60E-232	3.32E-231
TXNL1	0	1966	11.83232323	1.78E-231	1.63E-230
C19orf53	0	1964	11.83085484	2.65E-231	2.43E-230
SDC1	0	1961	11.82864944	4.83E-231	4.43E-230
SLC25A23	0	1946	11.81757162	9.73E-230	8.87E-229
ACVR1	0	1938	11.81162848	4.84E-229	4.40E-228
SBNO2	0	1933	11.80790155	1.32E-228	1.20E-227
ELAVL1	0	1930	11.80566076	2.41E-228	2.19E-227
ATP5G3	0	1928	11.80416496	3.61E-228	3.26E-227
GTF2F1	0	1921	11.79891743	1.47E-227	1.33E-226
MSH6	0	1921	11.79891743	1.47E-227	1.33E-226
NCLN	0	1920	11.79816622	1.80E-227	1.62E-226
CARM1	0	1909	11.78987701	1.65E-226	1.48E-225
FAM32A	0	1908	11.78912108	2.02E-226	1.81E-225
SMAD2	0	1908	11.78912108	2.02E-226	1.81E-225
UBE2E3	0	1907	11.78836475	2.47E-226	2.21E-225
TSN	0	1904	11.78609339	4.53E-226	4.04E-225
GIGYF2	0	1902	11.78457715	6.77E-226	6.05E-225
GPS1	0	1898	11.7815399	1.52E-225	1.35E-224
SAMD4B	0	1897	11.78077959	1.86E-225	1.65E-224
KXD1	0	1894	11.77849624	3.41E-225	3.02E-224
RNMT	0	1893	11.77773432	4.17E-225	3.70E-224
DNAJB1	0	1883	11.77009291	3.15E-224	2.79E-223
SYMPK	0	1879	11.76702498	7.08E-224	6.26E-223
MIDN	0	1875	11.7639505	1.59E-223	1.40E-222
ZC3H15	0	1875	11.7639505	1.59E-223	1.40E-222

DDX18	0	1864	11.75546177	1.48E-222	1.30E-221
MDH1	0	1851	11.7453648	2.08E-221	1.81E-220
MRPL19	0	1848	11.74302467	3.83E-221	3.33E-220
SLC8A1	0	1842	11.73833297	1.30E-220	1.13E-219
ZEB2	0	1838	11.73519668	2.94E-220	2.54E-219
ABI2	0	1836	11.73362597	4.42E-220	3.82E-219
KDSR	0	1829	11.72811498	1.84E-219	1.58E-218
RPL13A	0	1826	11.72574667	3.40E-219	2.91E-218
OST4	0	1813	11.71543883	4.85E-218	4.15E-217
MEX3C	0	1810	11.71304961	8.96E-218	7.66E-217
CIAO1	0	1805	11.70905875	2.49E-217	2.13E-216
YES1	0	1805	11.70905875	2.49E-217	2.13E-216
RRAS	0	1802	11.70665892	4.61E-217	3.92E-216
ATG9A	0	1801	11.70585809	5.66E-217	4.80E-216
STAM2	0	1799	11.7042551	8.54E-217	7.23E-216
PRKD3	0	1798	11.70345293	1.05E-216	8.87E-216
KDM4B	0	1795	11.70104375	1.94E-216	1.64E-215
TXNL4A	0	1789	11.6962133	6.65E-216	5.61E-215
KCMF1	0	1787	11.69459954	1.00E-215	8.45E-215
AP1M1	0	1786	11.69379199	1.23E-215	1.04E-214
CHMP2A	0	1785	11.69298398	1.51E-215	1.27E-214
BAX	0	1783	11.69136661	2.28E-215	1.91E-214
DBI	0	1778	11.68731523	6.39E-215	5.33E-214
PSMC4	0	1777	11.68650359	7.85E-215	6.54E-214
TEX261	0	1776	11.68569149	9.64E-215	8.02E-214
VPS4B	0	1776	11.68569149	9.64E-215	8.02E-214
DMPK	0	1773	11.68325245	1.79E-214	1.48E-213
HGS	0	1771	11.68162412	2.70E-214	2.24E-213
UQCR11	0	1768	11.67917818	5.01E-214	4.14E-213
CAD	0	1767	11.67836195	6.16E-214	5.09E-213
STRN4	0	1762	11.67427383	1.73E-213	1.43E-212
LPIN1	0	1751	11.66523899	1.68E-212	1.38E-211

IWS1	0	1744	11.65945995	7.14E-212	5.86E-211
SSC5D	0	1744	11.65945995	7.14E-212	5.86E-211
AKT1S1	0	1743	11.65863248	8.78E-212	7.19E-211
YIPF4	0	1730	11.64783195	1.30E-210	1.06E-209
PLAUR	0	1716	11.63610946	2.38E-209	1.93E-208
ARL5A	0	1715	11.63526849	2.93E-209	2.38E-208
TSHZ3	0	1705	11.62683165	2.35E-208	1.90E-207
JUND	0	1701	11.62344305	5.42E-208	4.37E-207
TLK1	0	1694	11.61749378	2.33E-207	1.88E-206
PPM1G	0	1685	11.6098085	1.53E-206	1.23E-205
MFF	0	1678	11.60380262	6.62E-206	5.29E-205
GRAMD1A	0	1677	11.6029426	8.17E-206	6.52E-205
UXS1	0	1675	11.601221	1.24E-205	9.89E-205
ATP5D	0	1673	11.59949735	1.89E-205	1.50E-204
YIF1B	0	1673	11.59949735	1.89E-205	1.50E-204
GIPC1	0	1665	11.59258209	1.01E-204	8.03E-204
GYPC	0	1661	11.58911198	2.34E-204	1.86E-203
ECH1	0	1660	11.58824315	2.89E-204	2.29E-203
URI1	0	1655	11.58389113	8.26E-204	6.53E-203
FOSL2	0	1652	11.5812736	1.55E-203	1.22E-202
PPP4R1	0	1652	11.5812736	1.55E-203	1.22E-202
STK39	0	1648	11.57777615	3.60E-203	2.83E-202
FHOD3	0	1645	11.57514749	6.77E-203	5.31E-202
SP100	0	1645	11.57514749	6.77E-203	5.31E-202
AFG3L2	0	1641	11.57163515	1.57E-202	1.23E-201
NDUFV2	0	1636	11.56723266	4.51E-202	3.53E-201
UBR3	0	1631	11.56281669	1.30E-201	1.01E-200
MOB1A	0	1629	11.56104651	1.98E-201	1.54E-200
FOXK2	0	1620	11.55305372	1.33E-200	1.03E-199
AAMP	0	1619	11.55216289	1.64E-200	1.27E-199
SLC1A4	0	1616	11.54948711	3.09E-200	2.39E-199
NCKAP5	0	1614	11.54770049	4.72E-200	3.65E-199

DAZAP1	0	1608	11.54232732	1.68E-199	1.30E-198
FAM134A	0	1602	11.53693406	6.01E-199	4.62E-198
NUMBL	0	1599	11.53422985	1.14E-198	8.71E-198
CCDC88A	0	1594	11.52971154	3.29E-198	2.52E-197
MAP3K2	0	1583	11.51972116	3.41E-197	2.61E-196
HDGFRP2	0	1579	11.51607108	8.00E-197	6.11E-196
CSNK1G2	0	1577	11.51424257	1.23E-196	9.35E-196
HNRNPM	0	1576	11.51332744	1.52E-196	1.15E-195
ALS2	0	1560	11.49860594	4.63E-195	3.49E-194
CEBPG	0	1548	11.48746538	6.04E-194	4.54E-193
ODC1	0	1541	11.48092677	2.71E-193	2.03E-192
EHBP1	0	1538	11.47811541	5.16E-193	3.86E-192
CCBE1	0	1530	11.47059156	2.88E-192	2.15E-191
RPRD1A	0	1530	11.47059156	2.88E-192	2.15E-191
SLC35F6	0	1528	11.46870445	4.43E-192	3.29E-191
KLF7	0	1523	11.46397585	1.30E-191	9.63E-191
WDR43	0	1521	11.46208006	2.00E-191	1.48E-190
OLA1	0	1515	11.4563777	7.30E-191	5.37E-190
RAD23A	0	1515	11.4563777	7.30E-191	5.37E-190
SIN3B	0	1511	11.45256357	1.73E-190	1.27E-189
PRKACA	0	1508	11.44969634	3.31E-190	2.43E-189
OSBPL1A	0	1507	11.44873933	4.10E-190	3.01E-189
DPP9	0	1500	11.44202241	1.86E-189	1.36E-188
RALB	0	1496	11.43817008	4.43E-189	3.24E-188
RETSAT	0	1489	11.43140366	2.02E-188	1.47E-187
GCC2	0	1477	11.41972973	2.73E-187	1.98E-186
PSMD14	0	1470	11.41287606	1.25E-186	9.06E-186
GADD45B	0	1467	11.40992878	2.41E-186	1.74E-185
OBSL1	0	1467	11.40992878	2.41E-186	1.74E-185
SLC30A6	0	1466	11.40894501	2.99E-186	2.16E-185
PKP4	0	1465	11.40796057	3.72E-186	2.68E-185
S1PR2	0	1460	11.40302828	1.11E-185	7.95E-185

CHMP1B	0	1458	11.40105063	1.71E-185	1.23E-184
PPP1R12C	0	1457	11.40006079	2.13E-185	1.53E-184
IMPACT	0	1456	11.39907026	2.65E-185	1.90E-184
SIPA1L3	0	1456	11.39907026	2.65E-185	1.90E-184
KIAA1715	0	1453	11.39609461	5.09E-185	3.64E-184
MEIS3	0	1451	11.39410743	7.89E-185	5.63E-184
ARHGEF18	0	1444	11.38713065	3.64E-184	2.60E-183
GATAD2A	0	1443	11.38613121	4.53E-184	3.23E-183
CLK1	0	1440	11.38312872	8.74E-184	6.22E-183
SOCS6	0	1439	11.3821265	1.09E-183	7.73E-183
DNASE2	0	1437	11.38011997	1.69E-183	1.20E-182
SOCS5	0	1437	11.38011997	1.69E-183	1.20E-182
TYK2	0	1437	11.38011997	1.69E-183	1.20E-182
GYS1	0	1433	11.37609852	4.05E-183	2.86E-182
EIF4E2	0	1425	11.36802183	2.34E-182	1.65E-181
SERPINB7	0	1425	11.36802183	2.34E-182	1.65E-181
C19orf43	0	1423	11.36599557	3.64E-182	2.55E-181
TANC1	0	1422	11.36498137	4.53E-182	3.18E-181
BCAM	0	1421	11.36396646	5.64E-182	3.95E-181
RIF1	0	1421	11.36396646	5.64E-182	3.95E-181
ERCC2	0	1419	11.3619345	8.76E-182	6.13E-181
FAM108A1	0	1417	11.35989967	1.36E-181	9.50E-181
COPS8	0	1413	11.35582137	3.28E-181	2.28E-180
KDM3A	0	1405	11.34763004	1.91E-180	1.32E-179
RAVER1	0	1394	11.33629047	2.17E-179	1.49E-178
CTDSP1	0	1389	11.33110651	6.55E-179	4.49E-178
KANSL3	0	1384	11.32590385	1.98E-178	1.36E-177
ATP13A1	0	1381	11.32277323	3.85E-178	2.63E-177
LOC541471	0	1377	11.31858847	9.34E-178	6.38E-177
MBD3	0	1375	11.31649153	1.46E-177	9.94E-177
PPP1R15A	0	1374	11.31544191	1.82E-177	1.24E-176
TRIP10	0	1374	11.31544191	1.82E-177	1.24E-176

MARK4	0	1373	11.31439153	2.27E-177	1.54E-176
R3HDM1	0	1372	11.31334039	2.83E-177	1.92E-176
CNDP2	0	1365	11.30596086	1.34E-176	9.07E-176
UBE2M	0	1363	11.30384547	2.09E-176	1.41E-175
ZADH2	0	1359	11.29960536	5.10E-176	3.43E-175
ARHGEF1	0	1358	11.29854339	6.37E-176	4.28E-175
NR1H2	0	1350	11.29001932	3.79E-175	2.54E-174
ACAA2	0	1346	11.28573832	9.25E-175	6.18E-174
RAB8A	0	1345	11.28466608	1.16E-174	7.70E-174
STK11	0	1345	11.28466608	1.16E-174	7.70E-174
DNAJB2	0	1344	11.28359305	1.45E-174	9.61E-174
UQCRFS1	0	1343	11.28251921	1.81E-174	1.20E-173
BABAM1	0	1342	11.28144458	2.26E-174	1.50E-173
MBD1	0	1342	11.28144458	2.26E-174	1.50E-173
RNF181	0	1341	11.28036915	2.83E-174	1.87E-173
ATF2	0	1340	11.27929291	3.54E-174	2.34E-173
EPB41L5	0	1339	11.27821587	4.42E-174	2.92E-173
WIZ	0	1339	11.27821587	4.42E-174	2.92E-173
ZBTB7A	0	1339	11.27821587	4.42E-174	2.92E-173
PMAIP1	0	1335	11.27389965	1.08E-173	7.13E-173
CREB1	0	1334	11.27281858	1.35E-173	8.90E-173
KEAP1	0	1333	11.27173669	1.69E-173	1.11E-172
IRGQ	0	1331	11.26957048	2.65E-173	1.74E-172
SDPR	0	1325	11.26305227	1.02E-172	6.66E-172
GMPPA	0	1317	11.25431525	6.12E-172	4.00E-171
CIR1	0	1315	11.25212271	9.60E-172	6.27E-171
SH3RF3	0	1310	11.24662672	2.95E-171	1.92E-170
MAU2	0	1307	11.24331905	5.80E-171	3.77E-170
RANBP3	0	1307	11.24331905	5.80E-171	3.77E-170
NAPA	0	1300	11.23557153	2.81E-170	1.82E-169
CIC	0	1299	11.23446134	3.52E-170	2.27E-169
GDF15	0	1294	11.22889753	1.09E-169	7.01E-169

DOCK6	0	1289	11.22331217	3.37E-169	2.17E-168
ANXA4	0	1288	11.2221925	4.22E-169	2.71E-168
PALM	0	1288	11.2221925	4.22E-169	2.71E-168
CDC34	0	1286	11.21995055	6.64E-169	4.25E-168
POLR1A	0	1285	11.21882827	8.32E-169	5.33E-168
JUNB	0	1281	11.21433038	2.06E-168	1.31E-167
FBL	0	1280	11.21320372	2.58E-168	1.65E-167
TBCB	0	1280	11.21320372	2.58E-168	1.65E-167
RAB11B	0	1278	11.21094775	4.06E-168	2.59E-167
YIPF2	0	1271	11.20302394	1.99E-167	1.26E-166
ANAPC11	0	1269	11.20075198	3.13E-167	1.98E-166
CNOT11	0	1269	11.20075198	3.13E-167	1.98E-166
MKNK2	0	1266	11.19733731	6.18E-167	3.90E-166
R3HDM4	0	1266	11.19733731	6.18E-167	3.90E-166
PGLS	0	1263	11.19391455	1.22E-166	7.69E-166
ZNF561	0	1263	11.19391455	1.22E-166	7.69E-166
AHSA2	0	1261	11.19162818	1.93E-166	1.21E-165
AUP1	0	1259	11.18933819	3.04E-166	1.90E-165
WIPF1	0	1256	11.18589637	6.01E-166	3.76E-165
NDUFB7	0	1253	11.18244632	1.19E-165	7.44E-165
DARS	0	1251	11.1801417	1.88E-165	1.17E-164
AKAP8L	0	1245	11.17320565	7.39E-165	4.60E-164
DYM	0	1241	11.16856302	1.84E-164	1.15E-163
EPHA4	0	1239	11.1662361	2.91E-164	1.81E-163
SRSF7	0	1239	11.1662361	2.91E-164	1.81E-163
IL11	0	1237	11.16390541	4.60E-164	2.85E-163
SAFB	0	1237	11.16390541	4.60E-164	2.85E-163
CPSF3	0	1236	11.16273865	5.78E-164	3.58E-163
SMPD4	0	1232	11.15806216	1.44E-163	8.93E-163
WDR33	0	1226	11.15101889	5.71E-163	3.52E-162
ASNSD1	0	1223	11.14748431	1.14E-162	6.99E-162
DMWD	0	1222	11.14630419	1.43E-162	8.79E-162

ITGA6	0	1220	11.14394106	2.26E-162	1.39E-161
SPIRE1	0	1219	11.14275803	2.85E-162	1.74E-161
USF2	0	1218	11.14157404	3.58E-162	2.19E-161
FARSA	0	1217	11.14038908	4.51E-162	2.75E-161
PSG5	0	1217	11.14038908	4.51E-162	2.75E-161
MED29	0	1214	11.13682833	8.98E-162	5.47E-161
NBEAL1	0	1212	11.13444961	1.42E-161	8.61E-161
USP39	0	1212	11.13444961	1.42E-161	8.61E-161
DNMT1	0	1211	11.13325877	1.79E-161	1.08E-160
NDUFA3	0	1210	11.13206696	2.25E-161	1.36E-160
MBD2	0	1209	11.13087415	2.84E-161	1.71E-160
LINC00152	0	1204	11.1248953	8.98E-161	5.41E-160
RHOB	0	1204	11.1248953	8.98E-161	5.41E-160
ACTR1B	0	1202	11.1224968	1.42E-160	8.57E-160
COQ10B	0	1202	11.1224968	1.42E-160	8.57E-160
CCNT2	0	1199	11.11889157	2.85E-160	1.71E-159
SGTA	0	1196	11.1152773	5.69E-160	3.40E-159
GSK3A	0	1194	11.11286275	9.03E-160	5.39E-159
CYTH2	0	1192	11.11044414	1.43E-159	8.56E-159
UBE2S	0	1188	11.10559474	3.62E-159	2.15E-158
KIF3C	0	1186	11.10316392	5.74E-159	3.41E-158
TRIB2	0	1186	11.10316392	5.74E-159	3.41E-158
SHKBP1	0	1183	11.09950998	1.15E-158	6.81E-158
ITGB1BP1	0	1182	11.09828994	1.45E-158	8.58E-158
ASXL2	0	1179	11.09462363	2.91E-158	1.72E-157
C17orf62	0	1177	11.09217423	4.62E-158	2.72E-157
TANK	0	1177	11.09217423	4.62E-158	2.72E-157
FAM195B	0	1176	11.09094797	5.83E-158	3.43E-157
HNRPLL	0	1166	11.0786277	5.96E-157	3.48E-156
SPRED2	0	1166	11.0786277	5.96E-157	3.48E-156
ZNF521	0	1166	11.0786277	5.96E-157	3.48E-156
NOP58	0	1163	11.07491101	1.20E-156	6.98E-156

TMEM185B	0	1162	11.07366998	1.51E-156	8.81E-156
ZNF587	0	1161	11.07242788	1.91E-156	1.11E-155
ILVBL	0	1159	11.06994048	3.04E-156	1.76E-155
PPIG	0	1159	11.06994048	3.04E-156	1.76E-155
SNRPG	0	1159	11.06994048	3.04E-156	1.76E-155
ZNF83	0	1156	11.06620131	6.12E-156	3.54E-155
GADD45GIP1	0	1155	11.06495276	7.72E-156	4.46E-155
NR2F6	0	1152	11.06120063	1.56E-155	8.98E-155
PGPEP1	0	1151	11.05994774	1.96E-155	1.13E-154
MRPL30	0	1150	11.05869377	2.48E-155	1.43E-154
STRN	0	1150	11.05869377	2.48E-155	1.43E-154
LSM7	0	1143	11.04988531	1.27E-154	7.30E-154
PAF1	0	1139	11.04482766	3.25E-154	1.86E-153
C17orf70	0	1129	11.03210539	3.39E-153	1.93E-152
KCTD15	0	1127	11.02954742	5.42E-153	3.08E-152
MAFG	0	1126	11.02826674	6.86E-153	3.89E-152
MBOAT2	0	1124	11.02570194	1.10E-152	6.21E-152
MLL4	0	1124	11.02570194	1.10E-152	6.21E-152
PRKRA	0	1123	11.02441784	1.39E-152	7.85E-152
ATIC	0	1122	11.02313258	1.76E-152	9.93E-152
EPT1	0	1121	11.02184619	2.22E-152	1.26E-151
ANAPC1	0	1120	11.02055864	2.81E-152	1.59E-151
ERCC1	0	1115	11.01410362	9.13E-152	5.13E-151
GLI2	0	1114	11.01280914	1.16E-151	6.49E-151
SUCLG1	0	1106	11.00241129	7.63E-151	4.26E-150
TRAPPC8	0	1106	11.00241129	7.63E-151	4.26E-150
BIN1	0	1100	10.99456343	3.15E-150	1.75E-149
TGIF1	0	1100	10.99456343	3.15E-150	1.75E-149
RUVBL2	0	1098	10.99193796	5.06E-150	2.80E-149
DOK1	0	1090	10.98138804	3.36E-149	1.86E-148
TSHZ1	0	1090	10.98138804	3.36E-149	1.86E-148
IAH1	0	1085	10.97475495	1.10E-148	6.08E-148

AGAP1	0	1066	10.94926735	1.01E-146	5.56E-146
CCDC93	0	1063	10.94520151	2.08E-146	1.13E-145
CCDC124	0	1062	10.94384367	2.64E-146	1.44E-145
DDA1	0	1061	10.94248457	3.35E-146	1.82E-145
TRAPPC12	0	1061	10.94248457	3.35E-146	1.82E-145
MAPRE2	0	1058	10.93839954	6.86E-146	3.72E-145
ERF	0	1053	10.93156535	2.27E-145	1.23E-144
AMMECR1L	0	1046	10.92194276	1.22E-144	6.53E-144
PGAP1	0	1044	10.91918162	1.96E-144	1.05E-143
TXNDC9	0	1042	10.91641519	3.18E-144	1.70E-143
ZNF358	0	1041	10.91502998	4.04E-144	2.16E-143
STAMBP	0	1040	10.91364344	5.13E-144	2.74E-143
ORMDL1	0	1038	10.91086635	8.30E-144	4.42E-143
RAB12	0	1037	10.9094758	1.06E-143	5.61E-143
EVI5L	0	1034	10.90529609	2.17E-143	1.15E-142
NAPG	0	1033	10.90390016	2.77E-143	1.47E-142
INPP4A	0	1030	10.89970425	5.70E-143	3.01E-142
PLEKHH2	0	1028	10.89690017	9.22E-143	4.87E-142
USP40	0	1026	10.89409064	1.49E-142	7.87E-142
PRR12	0	1018	10.88279747	1.03E-141	5.41E-141
ALYREF	0	1017	10.88137959	1.31E-141	6.89E-141
TEAD2	0	1011	10.87284291	5.61E-141	2.94E-140
PIGN	0	1010	10.8714152	7.15E-141	3.74E-140
ERCC3	0	1009	10.86998608	9.11E-141	4.75E-140
PTCD3	0	1009	10.86998608	9.11E-141	4.75E-140
MUM1	0	1007	10.86712359	1.48E-140	7.70E-140
RNF103	0	1004	10.86281918	3.06E-140	1.59E-139
PIK3C3	0	1002	10.85994242	4.98E-140	2.58E-139
SNRNP27	0	1002	10.85994242	4.98E-140	2.58E-139
TTL	0	1002	10.85994242	4.98E-140	2.58E-139
SUPT7L	0	999	10.85561649	1.03E-139	5.33E-139
MPV17	0	998	10.85417163	1.32E-139	6.78E-139

NARF	0	998	10.85417163	1.32E-139	6.78E-139
MALT1	0	996	10.85127756	2.14E-139	1.10E-138
VPS54	0	995	10.84982834	2.73E-139	1.40E-138
APLP1	0	992	10.84547193	5.67E-139	2.91E-138
PPP5C	0	991	10.84401687	7.23E-139	3.71E-138
ADAM23	0	990	10.84256034	9.23E-139	4.73E-138
MED16	0	984	10.83379013	3.99E-138	2.04E-137
ZNF160	0	984	10.83379013	3.99E-138	2.04E-137
TBCD	0	983	10.83232323	5.09E-138	2.60E-137
RHBDD1	0	981	10.82938495	8.29E-138	4.22E-137
FMNL2	0	980	10.82791356	1.06E-137	5.38E-137
SCAMP4	0	979	10.82644067	1.35E-137	6.86E-137
KLF16	0	978	10.82496628	1.73E-137	8.74E-137
RQCD1	0	978	10.82496628	1.73E-137	8.74E-137
SERTAD2	0	978	10.82496628	1.73E-137	8.74E-137
TRPM4	0	973	10.81757162	5.87E-137	2.96E-136
CDC42EP5	0	971	10.81460311	9.58E-137	4.82E-136
SIRT2	0	971	10.81460311	9.58E-137	4.82E-136
ADAM17	0	970	10.81311656	1.22E-136	6.15E-136
KIAA1468	0	966	10.807155	3.26E-136	1.63E-135
STRADB	0	966	10.807155	3.26E-136	1.63E-135
ZDBF2	0	965	10.80566076	4.17E-136	2.09E-135
ARMC9	0	962	10.80116871	8.71E-136	4.35E-135
GNA11	0	962	10.80116871	8.71E-136	4.35E-135
C19orf70	0	961	10.79966824	1.11E-135	5.55E-135
HMG20B	0	960	10.79816622	1.42E-135	7.08E-135
PEPD	0	955	10.79063255	4.87E-135	2.42E-134
RAB31	0	954	10.78912108	6.23E-135	3.09E-134
NAB1	0	952	10.78609339	1.02E-134	5.04E-134
ZNF260	0	944	10.77391867	7.34E-134	3.61E-133
PRPF31	0	943	10.77238958	9.39E-134	4.62E-133
C19orf12	0	941	10.76932654	1.54E-133	7.56E-133

CHERP	0	941	10.76932654	1.54E-133	7.56E-133
ZNF512	0	934	10.75855436	8.70E-133	4.26E-132
DUS1L	0	922	10.73989856	1.71E-131	8.32E-131
MRPL33	0	922	10.73989856	1.71E-131	8.32E-131
ANKRD12	0	919	10.73519668	3.60E-131	1.75E-130
GTF3C3	0	919	10.73519668	3.60E-131	1.75E-130
TMEM147	0	919	10.73519668	3.60E-131	1.75E-130
SMIM7	0	917	10.73205355	5.93E-131	2.87E-130
BAZ2B	0	915	10.72890356	9.76E-131	4.72E-130
ETFB	0	914	10.72732598	1.25E-130	6.06E-130
PPM1B	0	908	10.71782411	5.59E-130	2.70E-129
MPHOSPH10	0	907	10.71623436	7.18E-130	3.46E-129
SLC4A1AP	0	906	10.71464286	9.21E-130	4.44E-129
ISOC2	0	905	10.71304961	1.18E-129	5.69E-129
NUP62	0	903	10.7098578	1.95E-129	9.36E-129
SPOPL	0	899	10.70345293	5.30E-129	2.54E-128
PNKD	0	895	10.6970195	1.44E-128	6.92E-128
SF3A2	0	894	10.69540665	1.86E-128	8.88E-128
TBC1D17	0	890	10.68893715	5.06E-128	2.41E-127
WDR83OS	0	890	10.68893715	5.06E-128	2.41E-127
GPR155	0	887	10.68406592	1.08E-127	5.10E-127
XAB2	0	881	10.67427383	4.87E-127	2.30E-126
CNOT3	0	873	10.66111347	3.66E-126	1.73E-125
FECH	0	870	10.65614721	7.80E-126	3.67E-125
MIER2	0	867	10.65116381	1.66E-125	7.81E-125
NAGK	0	865	10.64783195	2.76E-125	1.29E-124
PDCD5	0	864	10.64616313	3.56E-125	1.66E-124
PREB	0	862	10.64281968	5.90E-125	2.75E-124
ASNA1	0	860	10.63946847	9.80E-125	4.55E-124
FASTKD2	0	855	10.63105623	3.48E-124	1.61E-123
STX10	0	855	10.63105623	3.48E-124	1.61E-123
BCL3	0	852	10.62598524	7.46E-124	3.45E-123

DSG2	0	852	10.62598524	7.46E-124	3.45E-123
SEH1L	0	849	10.62089637	1.60E-123	7.38E-123
IER2	0	843	10.61066445	7.37E-123	3.39E-122
TCF7L1	0	843	10.61066445	7.37E-123	3.39E-122
CEBPZ	0	839	10.60380262	2.04E-122	9.39E-122
COL5A3	0	838	10.60208206	2.64E-122	1.21E-121
PLEKHA3	0	837	10.60035944	3.41E-122	1.56E-121
ICAM5	0	836	10.59863476	4.40E-122	2.01E-121
PCYT2	0	836	10.59863476	4.40E-122	2.01E-121
ARL4C	0	835	10.59690801	5.68E-122	2.60E-121
CLPP	0	834	10.5951792	7.33E-122	3.35E-121
COPS7B	0	832	10.59171534	1.22E-121	5.58E-121
MBD5	0	831	10.58998029	1.58E-121	7.20E-121
SMARCAL1	0	831	10.58998029	1.58E-121	7.20E-121
FAM171B	0	826	10.5812736	5.68E-121	2.58E-120
TSEN34	0	824	10.57777615	9.49E-121	4.31E-120
MTA3	0	823	10.57602424	1.23E-120	5.56E-120
DGUOK	0	822	10.57427021	1.58E-120	7.18E-120
ZNF841	0	820	10.57075572	2.65E-120	1.20E-119
HOMER3	0	817	10.56546789	5.73E-120	2.59E-119
PIAS2	0	817	10.56546789	5.73E-120	2.59E-119
ZC3H4	0	815	10.56193187	9.58E-120	4.32E-119
CTDP1	0	813	10.55838717	1.60E-119	7.21E-119
RFNG	0	813	10.55838717	1.60E-119	7.21E-119
WDR75	0	812	10.55661154	2.07E-119	9.31E-119
RPE	0	807	10.54770049	7.52E-119	3.37E-118
PEX13	0	806	10.54591165	9.73E-119	4.36E-118
TECR	0	805	10.5441206	1.26E-118	5.63E-118
TMEM59L	0	803	10.5405318	2.11E-118	9.41E-118
PDE4A	0	802	10.53873405	2.73E-118	1.22E-117
EIF2AK3	0	800	10.53513181	4.59E-118	2.04E-117
MRPL12	0	798	10.53152056	7.69E-118	3.41E-117

KRCC1	0	796	10.52790024	1.29E-117	5.72E-117
SNRPD1	0	795	10.52608667	1.67E-117	7.39E-117
DUSP11	0	794	10.52427082	2.17E-117	9.57E-117
MTX2	0	793	10.52245268	2.81E-117	1.24E-116
PDK1	0	791	10.51880951	4.72E-117	2.08E-116
PET100	0	791	10.51880951	4.72E-117	2.08E-116
MAP1S	0	790	10.51698447	6.11E-117	2.69E-116
RNF149	0	790	10.51698447	6.11E-117	2.69E-116
POLR2D	0	789	10.51515711	7.93E-117	3.48E-116
FZR1	0	788	10.51332744	1.03E-116	4.51E-116
NOL10	0	787	10.51149545	1.33E-116	5.84E-116
ETHE1	0	785	10.50782447	2.24E-116	9.81E-116
LOC10050587	0	782	10.50230042	4.89E-116	2.13E-115
C18orf25	0	781	10.50045436	6.34E-116	2.77E-115
CC2D1A	0	775	10.48932812	3.03E-115	1.32E-114
CYB5A	0	775	10.48932812	3.03E-115	1.32E-114
RNASEH1	0	775	10.48932812	3.03E-115	1.32E-114
SMC6	0	773	10.48560023	5.10E-115	2.22E-114
AGBL5	0	771	10.48186267	8.60E-115	3.73E-114
RBM42	0	769	10.47811541	1.45E-114	6.28E-114
TMED1	0	768	10.47623812	1.88E-114	8.14E-114
TRAPPC5	0	768	10.47623812	1.88E-114	8.14E-114
TTC7A	0	767	10.47435839	2.45E-114	1.06E-113
ELOF1	0	766	10.47247621	3.18E-114	1.37E-113
PSTPIP2	0	765	10.47059156	4.13E-114	1.78E-113
DPP4	0	763	10.46681487	6.98E-114	3.00E-113
SPAST	0	763	10.46681487	6.98E-114	3.00E-113
MRPL35	0	762	10.46492281	9.07E-114	3.89E-113
ARID3A	0	759	10.4592317	1.99E-113	8.53E-113
INSR	0	754	10.44969634	7.40E-113	3.15E-112
UNC50	0	754	10.44969634	7.40E-113	3.15E-112
XRCC1	0	754	10.44969634	7.40E-113	3.15E-112

CD320	0	752	10.44586448	1.25E-112	5.33E-112
NCOA1	0	752	10.44586448	1.25E-112	5.33E-112
ALKBH7	0	751	10.44394472	1.63E-112	6.92E-112
SNRPA	0	747	10.43624006	4.67E-112	1.98E-111
MOB3A	0	746	10.43430744	6.08E-112	2.57E-111
ZNF264	0	744	10.43043444	1.03E-111	4.35E-111
CEP192	0	742	10.426551	1.75E-111	7.35E-111
ZNF507	0	742	10.426551	1.75E-111	7.35E-111
GTF3C2	0	741	10.42460536	2.27E-111	9.55E-111
POLI	0	741	10.42460536	2.27E-111	9.55E-111
FAM136A	0	739	10.42070618	3.86E-111	1.62E-110
TOMM40	0	737	10.41679643	6.54E-111	2.74E-110
ALMS1	0	736	10.41483758	8.53E-111	3.57E-110
REV1	0	736	10.41483758	8.53E-111	3.57E-110
PIN1	0	732	10.40697546	2.46E-110	1.03E-109
MEX3D	0	731	10.40500322	3.20E-110	1.34E-109
ZNF317	0	727	10.39708718	9.26E-110	3.85E-109
CD97	0	725	10.39311281	1.58E-109	6.54E-109
DHX57	0	725	10.39311281	1.58E-109	6.54E-109
MRPS5	0	720	10.38312872	5.95E-109	2.47E-108
CCNYL1	0	718	10.37911566	1.01E-108	4.20E-108
PLEKHM3	0	717	10.37710493	1.32E-108	5.47E-108
LMAN2L	0	714	10.37105589	2.95E-108	1.21E-107
NDUFB3	0	714	10.37105589	2.95E-108	1.21E-107
ITSN2	0	713	10.36903389	3.85E-108	1.58E-107
CCDC9	0	712	10.36700906	5.02E-108	2.07E-107
ICAM1	0	712	10.36700906	5.02E-108	2.07E-107
ZNF271	0	711	10.36498137	6.56E-108	2.69E-107
GAMT	0	709	10.36091744	1.12E-107	4.59E-107
LIMS2	0	708	10.35888117	1.46E-107	5.99E-107
ATP5SL	0	707	10.35684203	1.91E-107	7.82E-107
DCAF17	0	704	10.35070724	4.27E-107	1.74E-106

MAP2K7	0	702	10.34660284	7.29E-107	2.97E-106
HEATR5B	0	698	10.33835885	2.13E-106	8.68E-106
ARHGAP28	0	696	10.33421912	3.65E-106	1.48E-105
EPS15L1	0	696	10.33421912	3.65E-106	1.48E-105
LSM4	0	695	10.33214479	4.77E-106	1.94E-105
NOSIP	0	695	10.33214479	4.77E-106	1.94E-105
IL1R1	0	694	10.33006748	6.24E-106	2.53E-105
METRNL	0	692	10.32590385	1.07E-105	4.32E-105
CNPPD1	0	691	10.32381752	1.40E-105	5.64E-105
ANKRD27	0	690	10.32172818	1.83E-105	7.37E-105
TIMM44	0	689	10.3196358	2.40E-105	9.64E-105
ADNP2	0	685	10.3112358	7.05E-105	2.83E-104
CYP20A1	0	684	10.30912814	9.23E-105	3.70E-104
PARD6G	0	680	10.30066656	2.72E-104	1.09E-103
SMG9	0	677	10.29428765	6.13E-104	2.45E-103
SAP130	0	675	10.29001932	1.05E-103	4.20E-103
SPPL2B	0	675	10.29001932	1.05E-103	4.20E-103
FAM126B	0	674	10.28788041	1.38E-103	5.49E-103
FBXL12	0	673	10.28573832	1.81E-103	7.19E-103
MFSD12	0	673	10.28573832	1.81E-103	7.19E-103
TIMM13	0	673	10.28573832	1.81E-103	7.19E-103
ZSWIM4	0	672	10.28359305	2.37E-103	9.42E-103
EIF2B4	0	670	10.27929291	4.08E-103	1.62E-102
STK36	0	670	10.27929291	4.08E-103	1.62E-102
PSMG2	0	664	10.26631506	2.09E-102	8.21E-102
WDR7	0	660	10.25759784	6.20E-102	2.43E-101
NAT14	0	659	10.25541028	8.14E-102	3.19E-101
FARSB	0	658	10.2532194	1.07E-101	4.19E-101
PAK4	0	656	10.24882763	1.85E-101	7.21E-101
TIMM50	0	656	10.24882763	1.85E-101	7.21E-101
RMND5A	0	655	10.24662672	2.43E-101	9.47E-101
CCDC8	0	652	10.24000378	5.51E-101	2.14E-100

SH3YL1	0	652	10.24000378	5.51E-101	2.14E-100
ATL2	0	651	10.23778936	7.24E-101	2.81E-100
OGFOD3	0	649	10.23335029	1.25E-100	4.85E-100
REXO1	0	646	10.22666598	2.85E-100	1.10E-99
SAFB2	0	646	10.22666598	2.85E-100	1.10E-99
POLRMT	0	643	10.21995055	6.48E-100	2.50E-99
SESTD1	0	642	10.21770511	8.53E-100	3.29E-99
DOCK10	0	640	10.21320372	1.48E-99	5.68E-99
SEPW1	0	636	10.20415858	4.44E-99	1.70E-98
GPN1	0	635	10.20188841	5.85E-99	2.24E-98
ZNF302	0	635	10.20188841	5.85E-99	2.24E-98
CPS1	0	634	10.19961465	7.70E-99	2.95E-98
MGC2752	0	633	10.19733731	1.01E-98	3.88E-98
ZNF580	0	632	10.19505637	1.34E-98	5.11E-98
IMP4	0	630	10.19048364	2.32E-98	8.85E-98
NCK2	0	626	10.18129447	7.01E-98	2.66E-97
CPT1C	0	624	10.17667784	1.22E-97	4.62E-97
IFT172	0	624	10.17667784	1.22E-97	4.62E-97
ZNF266	0	622	10.17204639	2.12E-97	8.01E-97
ZNF579	0	621	10.16972508	2.80E-97	1.06E-96
RNF126	0	619	10.16507122	4.87E-97	1.84E-96
SERPINB8	0	619	10.16507122	4.87E-97	1.84E-96
EXOC6B	0	616	10.15806216	1.12E-96	4.21E-96
DOT1L	0	615	10.15571822	1.48E-96	5.55E-96
ME2	0	614	10.15337047	1.95E-96	7.32E-96
MEMO1	0	614	10.15337047	1.95E-96	7.32E-96
THADA	0	614	10.15337047	1.95E-96	7.32E-96
ORC4	0	612	10.14866347	3.40E-96	1.27E-95
IMPA2	0	611	10.14630419	4.49E-96	1.68E-95
IRF3	0	610	10.14394106	5.94E-96	2.22E-95
BCAT2	0	608	10.13920314	1.04E-95	3.86E-95
DNPEP	0	608	10.13920314	1.04E-95	3.86E-95

TP53I3	0	606	10.13444961	1.81E-95	6.73E-95
MZT2B	0	604	10.12968036	3.16E-95	1.18E-94
ZNF562	0	604	10.12968036	3.16E-95	1.18E-94
EPC2	0	602	10.1248953	5.53E-95	2.05E-94
FKRP	0	599	10.11768782	1.28E-94	4.73E-94
GPR108	0	599	10.11768782	1.28E-94	4.73E-94
THOP1	0	599	10.11768782	1.28E-94	4.73E-94
GMIP	0	598	10.1152773	1.69E-94	6.24E-94
TMEM205	0	598	10.1152773	1.69E-94	6.24E-94
MRPL4	0	596	10.11044414	2.96E-94	1.09E-93
MTIF2	0	594	10.10559474	5.19E-94	1.91E-93
SLC14A1	0	594	10.10559474	5.19E-94	1.91E-93
AFTPH	0	593	10.10316392	6.87E-94	2.52E-93
ORC2	0	590	10.09584677	1.59E-93	5.83E-93
MRPL44	0	588	10.09094797	2.80E-93	1.02E-92
PRKD2	0	588	10.09094797	2.80E-93	1.02E-92
ZNF865	0	587	10.08849232	3.71E-93	1.35E-92
PSENEN	0	586	10.08603248	4.91E-93	1.79E-92
SEMA4C	0	582	10.07615097	1.52E-92	5.50E-92
ZNF805	0	581	10.07366998	2.01E-92	7.29E-92
CNRIP1	0	580	10.07118471	2.66E-92	9.64E-92
NPC1	0	578	10.06620131	4.69E-92	1.69E-91
TET3	0	578	10.06620131	4.69E-92	1.69E-91
NDUFA7	0	577	10.06370313	6.22E-92	2.23E-91
MZT2A	0	576	10.06120063	8.25E-92	2.96E-91
HAT1	0	575	10.05869377	1.09E-91	3.92E-91
PPP1R37	0	575	10.05869377	1.09E-91	3.92E-91
ITPKC	0	573	10.05366695	1.93E-91	6.88E-91
GULP1	0	572	10.05114696	2.56E-91	9.12E-91
PKDCC	0	571	10.04862256	3.40E-91	1.21E-90
PRKCE	0	571	10.04862256	3.40E-91	1.21E-90
COMMD1	0	569	10.04356047	5.99E-91	2.13E-90

INO80D	0	569	10.04356047	5.99E-91	2.13E-90
DHPS	0	567	10.03848055	1.06E-90	3.74E-90
STON1	0	566	10.03593387	1.40E-90	4.96E-90
CRIPT	0	565	10.03338268	1.86E-90	6.58E-90
EML2	0	565	10.03338268	1.86E-90	6.58E-90
NPAS2	0	565	10.03338268	1.86E-90	6.58E-90
TTC21B	0	565	10.03338268	1.86E-90	6.58E-90
ZSCAN30	0	563	10.02826674	3.29E-90	1.16E-89
HIBCH	0	562	10.02570194	4.37E-90	1.54E-89
CHRNA1	0	560	10.02055864	7.73E-90	2.71E-89
CCDC104	0	558	10.01539694	1.37E-89	4.79E-89
TGFBRAP1	0	558	10.01539694	1.37E-89	4.79E-89
A1BG	0	557	10.01280914	1.82E-89	6.36E-89
ZNF142	0	554	10.00501779	4.28E-89	1.50E-88
AKAP8	0	552	9.999800081	7.58E-89	2.64E-88
MED25	0	552	9.999800081	7.58E-89	2.64E-88
EID2	0	550	9.994563433	1.34E-88	4.68E-88
PQLC1	0	550	9.994563433	1.34E-88	4.68E-88
ADAMTS10	0	549	9.991937963	1.79E-88	6.21E-88
OPA3	0	545	9.981388044	5.63E-88	1.95E-87
PAPOLG	0	543	9.976084012	1.00E-87	3.45E-87
WDR18	0	543	9.976084012	1.00E-87	3.45E-87
IRF2BP1	0	540	9.968091221	2.37E-87	8.15E-87
VAMP5	0	539	9.965417087	3.16E-87	1.09E-86
DNMT3A	0	536	9.957364815	7.49E-87	2.57E-86
SLC27A1	0	536	9.957364815	7.49E-87	2.57E-86
INSIG2	0	535	9.954670705	1.00E-86	3.43E-86
NEDD4L	0	535	9.954670705	1.00E-86	3.43E-86
BLVRB	0	534	9.951971556	1.33E-86	4.57E-86
TMEM161A	0	534	9.951971556	1.33E-86	4.57E-86
ELL	0	530	9.941124174	4.23E-86	1.44E-85
LINC00667	0	530	9.941124174	4.23E-86	1.44E-85

FEM1A	0	529	9.938399536	5.65E-86	1.92E-85
SCRN3	0	528	9.935669743	7.55E-86	2.57E-85
ANKZF1	0	527	9.932934776	1.01E-85	3.43E-85
NDUFS7	0	525	9.927449237	1.80E-85	6.10E-85
LCLAT1	0	522	9.919181621	4.30E-85	1.45E-84
ZNF526	0	522	9.919181621	4.30E-85	1.45E-84
ZNF787	0	522	9.919181621	4.30E-85	1.45E-84
ZNF444	0	521	9.916415186	5.74E-85	1.94E-84
INPP1	0	518	9.908083912	1.37E-84	4.62E-84
BRE	0	517	9.905296094	1.84E-84	6.16E-84
DDX49	0	517	9.905296094	1.84E-84	6.16E-84
SIX5	0	517	9.905296094	1.84E-84	6.16E-84
SPRED3	0	517	9.905296094	1.84E-84	6.16E-84
MARCH2	0	515	9.899704246	3.29E-84	1.10E-83
SUGP1	0	515	9.899704246	3.29E-84	1.10E-83
ZNF226	0	515	9.899704246	3.29E-84	1.10E-83
HPCAL1	0	514	9.896900173	4.40E-84	1.47E-83
GPD2	0	511	9.888455105	1.06E-83	3.51E-83
RFTN2	0	511	9.888455105	1.06E-83	3.51E-83
PLEKHJ1	0	510	9.885629061	1.41E-83	4.69E-83
SERTAD3	0	510	9.885629061	1.41E-83	4.69E-83
CCDC97	0	509	9.88279747	1.89E-83	6.28E-83
KCTD18	0	509	9.88279747	1.89E-83	6.28E-83
ZNF407	0	507	9.877117561	3.40E-83	1.12E-82
SATB2	0	506	9.874269199	4.55E-83	1.50E-82
QPCTL	0	505	9.871415202	6.10E-83	2.01E-82
WTIP	0	505	9.871415202	6.10E-83	2.01E-82
DPY30	0	503	9.865690214	1.10E-82	3.61E-82
ADAMTSL5	0	502	9.862819178	1.47E-82	4.83E-82
CRTC1	0	501	9.859942417	1.97E-82	6.47E-82
DIRAS1	0	500	9.857059909	2.64E-82	8.66E-82
PPP1R21	0	500	9.857059909	2.64E-82	8.66E-82

EVA1A	0	499	9.854171629	3.54E-82	1.16E-81
MRPL53	0	499	9.854171629	3.54E-82	1.16E-81
POU2F2	0	499	9.854171629	3.54E-82	1.16E-81
PPIL3	0	498	9.851277556	4.75E-82	1.55E-81
POP4	0	497	9.848377666	6.38E-82	2.08E-81
C2orf68	0	496	9.845471934	8.56E-82	2.79E-81
GTDC1	0	496	9.845471934	8.56E-82	2.79E-81
PMS1	0	496	9.845471934	8.56E-82	2.79E-81
ZNF542	0	492	9.833790129	2.78E-81	9.04E-81
ETAA1	0	491	9.830854838	3.73E-81	1.21E-80
SSBP4	0	491	9.830854838	3.73E-81	1.21E-80
TTN	0	491	9.830854838	3.73E-81	1.21E-80
CEP68	0	490	9.827913563	5.01E-81	1.62E-80
SMYD5	0	490	9.827913563	5.01E-81	1.62E-80
ZSCAN18	0	490	9.827913563	5.01E-81	1.62E-80
PTPN4	0	488	9.822012962	9.05E-81	2.92E-80
ATP9B	0	485	9.813116561	2.20E-80	7.08E-80
ID2	0	485	9.813116561	2.20E-80	7.08E-80
ZNF254	0	485	9.813116561	2.20E-80	7.08E-80
ZNF776	0	483	9.807155003	3.97E-80	1.28E-79
RBM43	0	482	9.80416496	5.34E-80	1.72E-79
MAST3	0	481	9.801168708	7.18E-80	2.30E-79
MSH2	0	481	9.801168708	7.18E-80	2.30E-79
SLC25A12	0	481	9.801168708	7.18E-80	2.30E-79
ZNF627	0	481	9.801168708	7.18E-80	2.30E-79
CCDC115	0	480	9.79816622	9.67E-80	3.09E-79
GRWD1	0	479	9.79515747	1.30E-79	4.15E-79
PLEKHA4	0	479	9.79515747	1.30E-79	4.15E-79
SERTAD1	0	479	9.79515747	1.30E-79	4.15E-79
ZNF791	0	479	9.79515747	1.30E-79	4.15E-79
ATG16L1	0	478	9.792142432	1.75E-79	5.58E-79
PTPN2	0	478	9.792142432	1.75E-79	5.58E-79

C1D	0	476	9.786093387	3.17E-79	1.01E-78
CAPG	0	476	9.786093387	3.17E-79	1.01E-78
FN3KRP	0	476	9.786093387	3.17E-79	1.01E-78
LOC728730	0	476	9.786093387	3.17E-79	1.01E-78
LRP3	0	475	9.783059327	4.27E-79	1.36E-78
SNAPC2	0	474	9.780018873	5.75E-79	1.83E-78
CACNA1A	0	473	9.776971997	7.74E-79	2.45E-78
GMCL1	0	473	9.776971997	7.74E-79	2.45E-78
ARL16	0	471	9.770858874	1.41E-78	4.45E-78
PNO1	0	471	9.770858874	1.41E-78	4.45E-78
DGKD	0	469	9.764719737	2.55E-78	8.06E-78
VRK3	0	469	9.764719737	2.55E-78	8.06E-78
ATAD2B	0	467	9.758554364	4.64E-78	1.46E-77
PNPT1	0	467	9.758554364	4.64E-78	1.46E-77
HKR1	0	465	9.75236253	8.44E-78	2.66E-77
RTN2	0	465	9.75236253	8.44E-78	2.66E-77
ZC3H6	0	465	9.75236253	8.44E-78	2.66E-77
HDHD2	0	458	9.730479412	6.88E-77	2.16E-76
CNNM3	0	457	9.727325979	9.29E-77	2.91E-76
SLC39A3	0	457	9.727325979	9.29E-77	2.91E-76
UHRF1	0	457	9.727325979	9.29E-77	2.91E-76
ZNF91	0	457	9.727325979	9.29E-77	2.91E-76
CWC22	0	456	9.724165638	1.25E-76	3.92E-76
CXXC1	0	455	9.720998359	1.70E-76	5.30E-76
ZNF581	0	454	9.717824111	2.29E-76	7.15E-76
ESCO1	0	453	9.714642864	3.10E-76	9.63E-76
PIH1D1	0	451	9.708259247	5.66E-76	1.76E-75
BCKDHA	0	450	9.705056815	7.65E-76	2.37E-75
ZFP36	0	450	9.705056815	7.65E-76	2.37E-75
MCM6	0	449	9.701847259	1.03E-75	3.20E-75
RPL23AP7	0	448	9.698630546	1.40E-75	4.33E-75
ZNF134	0	446	9.692175524	2.56E-75	7.91E-75

ZNF761	0	446	9.692175524	2.56E-75	7.91E-75
TTC31	0	443	9.682438513	6.35E-75	1.96E-74
METTL8	0	441	9.67591047	1.16E-74	3.58E-74
RASGRP3	0	441	9.67591047	1.16E-74	3.58E-74
CCDC137	0	440	9.672635338	1.58E-74	4.84E-74
FAHD2A	0	439	9.669352754	2.14E-74	6.55E-74
ZNF766	0	438	9.666062684	2.90E-74	8.86E-74
CARD8	0	433	9.649498839	1.33E-73	4.04E-73
IKZF2	0	432	9.646163126	1.80E-73	5.47E-73
ZNF333	0	431	9.642819683	2.44E-73	7.41E-73
C2orf43	0	430	9.639468474	3.31E-73	1.01E-72
CLASRP	0	430	9.639468474	3.31E-73	1.01E-72
MXD1	0	430	9.639468474	3.31E-73	1.01E-72
ZNF780A	0	430	9.639468474	3.31E-73	1.01E-72
METTL21A	0	429	9.636109462	4.50E-73	1.36E-72
CCDC130	0	428	9.632742611	6.10E-73	1.84E-72
FAM210A	0	426	9.625985244	1.13E-72	3.39E-72
MKI67IP	0	425	9.622594655	1.53E-72	4.60E-72
ZNF616	0	425	9.622594655	1.53E-72	4.60E-72
PIAS4	0	423	9.615789477	2.82E-72	8.46E-72
TRMT1	0	423	9.615789477	2.82E-72	8.46E-72
TTC27	0	423	9.615789477	2.82E-72	8.46E-72
C19orf48	0	421	9.608952047	5.21E-72	1.56E-71
SOWAHC	0	421	9.608952047	5.21E-72	1.56E-71
ZNF236	0	420	9.605521142	7.08E-72	2.12E-71
ARMC6	0	416	9.591715342	2.42E-71	7.20E-71
C2orf69	0	416	9.591715342	2.42E-71	7.20E-71
SP140L	0	412	9.577776151	8.32E-71	2.47E-70
MRPS9	0	410	9.570755724	1.54E-70	4.57E-70
FAM49A	0	407	9.560160608	3.91E-70	1.15E-69
TIMM21	0	407	9.560160608	3.91E-70	1.15E-69
ANGPTL4	0	406	9.556611541	5.33E-70	1.57E-69

CDK15	0	406	9.556611541	5.33E-70	1.57E-69
FASTKD1	0	404	9.549487107	9.90E-70	2.91E-69
RTKN	0	403	9.545911653	1.35E-69	3.96E-69
UBXN2A	0	401	9.538734051	2.51E-69	7.34E-69
GATA6	0	400	9.535131814	3.43E-69	1.00E-68
HNMT	0	399	9.53152056	4.68E-69	1.37E-68
RRM2	0	399	9.53152056	4.68E-69	1.37E-68
CADM4	0	398	9.527900245	6.40E-69	1.86E-68
MRI1	0	398	9.527900245	6.40E-69	1.86E-68
RNF25	0	397	9.524270821	8.73E-69	2.54E-68
DYRK1B	0	396	9.520632244	1.19E-68	3.47E-68
ZNF558	0	396	9.520632244	1.19E-68	3.47E-68
MRPL34	0	395	9.516984467	1.63E-68	4.73E-68
ZNF274	0	395	9.516984467	1.63E-68	4.73E-68
ZNF528	0	394	9.513327444	2.23E-68	6.46E-68
PQLC3	0	393	9.509661126	3.04E-68	8.82E-68
STK16	0	393	9.509661126	3.04E-68	8.82E-68
C19orf60	0	391	9.502300421	5.69E-68	1.64E-67
CEACAM19	0	391	9.502300421	5.69E-68	1.64E-67
KRI1	0	391	9.502300421	5.69E-68	1.64E-67
SPAG16	0	391	9.502300421	5.69E-68	1.64E-67
SRBD1	0	390	9.498605938	7.78E-68	2.24E-67
DACT3	0	389	9.494901969	1.06E-67	3.06E-67
HSPBP1	0	389	9.494901969	1.06E-67	3.06E-67
MVB12A	0	389	9.494901969	1.06E-67	3.06E-67
ZNF229	0	389	9.494901969	1.06E-67	3.06E-67
FNDC4	0	387	9.48746538	1.99E-67	5.72E-67
NFU1	0	386	9.483732661	2.73E-67	7.81E-67
WDR12	0	386	9.483732661	2.73E-67	7.81E-67
SEMA4F	0	385	9.47999026	3.73E-67	1.07E-66
ZNF574	0	385	9.47999026	3.73E-67	1.07E-66
CABLES1	0	383	9.472476206	7.00E-67	2.00E-66

OLFM2	0	383	9.472476206	7.00E-67	2.00E-66
GLTSCR1	0	382	9.468704452	9.59E-67	2.74E-66
MRPS12	0	381	9.464922812	1.31E-66	3.74E-66
DYNC2LI1	0	379	9.457329662	2.47E-66	7.02E-66
MFSD6	0	379	9.457329662	2.47E-66	7.02E-66
E2F6	0	378	9.453518048	3.38E-66	9.62E-66
POLR2I	0	378	9.453518048	3.38E-66	9.62E-66
HCN2	0	377	9.449696337	4.64E-66	1.32E-65
PODNL1	0	377	9.449696337	4.64E-66	1.32E-65
WDR92	0	377	9.449696337	4.64E-66	1.32E-65
ZNF529	0	376	9.445864476	6.36E-66	1.80E-65
ZNF780B	0	373	9.434307444	1.64E-65	4.64E-65
GCFC2	0	372	9.430434435	2.26E-65	6.36E-65
TMEM150A	0	372	9.430434435	2.26E-65	6.36E-65
MAP3K10	0	370	9.422657085	4.26E-65	1.19E-64
ZNF320	0	370	9.422657085	4.26E-65	1.19E-64
MAPRE3	0	369	9.41875263	5.85E-65	1.64E-64
POLR1B	0	369	9.41875263	5.85E-65	1.64E-64
ZNF304	0	367	9.410911877	1.10E-64	3.09E-64
ZFP30	0	366	9.406975462	1.52E-64	4.24E-64
C19orf66	0	365	9.403028278	2.09E-64	5.82E-64
ABCB6	0	364	9.399070264	2.87E-64	8.00E-64
ECSIT	0	364	9.399070264	2.87E-64	8.00E-64
TTLL4	0	364	9.399070264	2.87E-64	8.00E-64
B3GNT2	0	363	9.395101362	3.95E-64	1.10E-63
C19orf24	0	363	9.395101362	3.95E-64	1.10E-63
ZNF468	0	363	9.395101362	3.95E-64	1.10E-63
ZNF514	0	362	9.391121511	5.43E-64	1.51E-63
HS1BP3	0	361	9.387130651	7.47E-64	2.07E-63
THOC1	0	358	9.375091401	1.95E-63	5.38E-63
ZNF397	0	358	9.375091401	1.95E-63	5.38E-63
TMEM18	0	357	9.371055888	2.68E-63	7.40E-63

ALS2CR8	0	356	9.367009055	3.70E-63	1.02E-62
KANSL1L	0	356	9.367009055	3.70E-63	1.02E-62
C2orf44	0	355	9.362950839	5.10E-63	1.40E-62
TICAM1	0	355	9.362950839	5.10E-63	1.40E-62
ZNF45	0	354	9.358881174	7.03E-63	1.93E-62
MRPL54	0	352	9.350707243	1.34E-62	3.66E-62
NFKBIB	0	350	9.342486736	2.54E-62	6.93E-62
ZNF814	0	350	9.342486736	2.54E-62	6.93E-62
ADCY3	0	347	9.330067477	6.68E-62	1.82E-61
SHANK1	0	344	9.317540379	1.76E-61	4.78E-61
USP37	0	344	9.317540379	1.76E-61	4.78E-61
ZNF419	0	343	9.31334039	2.43E-61	6.59E-61
DEDD2	0	342	9.309128139	3.36E-61	9.09E-61
SLC40A1	0	342	9.309128139	3.36E-61	9.09E-61
CACTIN	0	340	9.30066656	6.43E-61	1.74E-60
DIS3L2	0	340	9.30066656	6.43E-61	1.74E-60
SOX11	0	339	9.296417087	8.89E-61	2.40E-60
LOC150776	0	338	9.29215506	1.23E-60	3.31E-60
CASP8	0	337	9.287880405	1.70E-60	4.58E-60
ZNF331	0	337	9.287880405	1.70E-60	4.58E-60
BLOC1S3	0	336	9.283593047	2.36E-60	6.33E-60
CNNM4	0	336	9.283593047	2.36E-60	6.33E-60
FDX1L	0	334	9.274979917	4.52E-60	1.21E-59
ZNF234	0	334	9.274979917	4.52E-60	1.21E-59
ZNF417	0	333	9.270653991	6.26E-60	1.68E-59
LAMA1	0	332	9.266315055	8.67E-60	2.32E-59
NIF3L1	0	332	9.266315055	8.67E-60	2.32E-59
ZNF471	0	332	9.266315055	8.67E-60	2.32E-59
CEP89	0	331	9.261963031	1.20E-59	3.20E-59
COA5	0	331	9.261963031	1.20E-59	3.20E-59
KCTD1	0	328	9.248827629	3.20E-59	8.52E-59
ZNF480	0	328	9.248827629	3.20E-59	8.52E-59

SLC25A42	0	327	9.24442245	4.44E-59	1.18E-58
ASPSCR1	0	325	9.235571532	8.56E-59	2.26E-58
C18orf8	0	325	9.235571532	8.56E-59	2.26E-58
KLHL29	0	325	9.235571532	8.56E-59	2.26E-58
ZNF227	0	325	9.235571532	8.56E-59	2.26E-58
SLC5A6	0	324	9.231125627	1.19E-58	3.14E-58
ZNF329	0	324	9.231125627	1.19E-58	3.14E-58
METTL5	0	323	9.226665979	1.65E-58	4.35E-58
ZNF584	0	323	9.226665979	1.65E-58	4.35E-58
CCDC106	0	321	9.217705111	3.18E-58	8.38E-58
ELMOD3	0	321	9.217705111	3.18E-58	8.38E-58
ICAM3	0	321	9.217705111	3.18E-58	8.38E-58
MCEE	0	321	9.217705111	3.18E-58	8.38E-58
BRSK1	0	320	9.213203719	4.42E-58	1.16E-57
TTC39C	0	320	9.213203719	4.42E-58	1.16E-57
ARL6IP6	0	317	9.199614654	1.19E-57	3.11E-57
DHX34	0	315	9.190483642	2.30E-57	6.00E-57
HAUS1	0	315	9.190483642	2.30E-57	6.00E-57
RFX1	0	315	9.190483642	2.30E-57	6.00E-57
ZNF544	0	315	9.190483642	2.30E-57	6.00E-57
ANKRD44	0	312	9.176677843	6.21E-57	1.61E-56
PNKP	0	311	9.172046394	8.65E-57	2.24E-56
ISYNA1	0	310	9.167400029	1.21E-56	3.12E-56
THUMPD2	0	310	9.167400029	1.21E-56	3.12E-56
TYW5	0	310	9.167400029	1.21E-56	3.12E-56
ZNF667	0	310	9.167400029	1.21E-56	3.12E-56
ATF5	0	309	9.162738652	1.68E-56	4.34E-56
FBXO17	0	309	9.162738652	1.68E-56	4.34E-56
ZNF548	0	308	9.158062165	2.34E-56	6.03E-56
DDX39A	0	307	9.153370469	3.27E-56	8.40E-56
VRK2	0	306	9.148663467	4.56E-56	1.17E-55
C19orf52	0	305	9.143941057	6.36E-56	1.63E-55

CTXN1	0	305	9.143941057	6.36E-56	1.63E-55
ZNF555	0	305	9.143941057	6.36E-56	1.63E-55
CRLF1	0	304	9.139203138	8.87E-56	2.28E-55
FOXN2	0	303	9.134449608	1.24E-55	3.17E-55
MPV17L2	0	303	9.134449608	1.24E-55	3.17E-55
ADCK4	0	301	9.124895301	2.42E-55	6.17E-55
NDUFAF7	0	301	9.124895301	2.42E-55	6.17E-55
MPPE1	0	299	9.115277298	4.72E-55	1.20E-54
TAF1B	0	299	9.115277298	4.72E-55	1.20E-54
ZNF614	0	299	9.115277298	4.72E-55	1.20E-54
C19orf25	0	298	9.110444145	6.59E-55	1.68E-54
EPB41L3	0	297	9.105594745	9.22E-55	2.34E-54
PRR24	0	296	9.10072899	1.29E-54	3.27E-54
RTTN	0	296	9.10072899	1.29E-54	3.27E-54
TMEM160	0	296	9.10072899	1.29E-54	3.27E-54
BBC3	0	294	9.090947969	2.52E-54	6.38E-54
BCS1L	0	293	9.086032479	3.53E-54	8.93E-54
RAC3	0	292	9.081100183	4.95E-54	1.25E-53
ZNF585B	0	290	9.071184714	9.71E-54	2.44E-53
SLC4A5	0	289	9.066201307	1.36E-53	3.42E-53
ZBTB45	0	289	9.066201307	1.36E-53	3.42E-53
ZNF772	0	289	9.066201307	1.36E-53	3.42E-53
HSBP1L1	0	288	9.061200626	1.91E-53	4.77E-53
RBBP8	0	288	9.061200626	1.91E-53	4.77E-53
LOC10012825	0	287	9.056182551	2.68E-53	6.68E-53
WDR35	0	286	9.051146961	3.75E-53	9.36E-53
ZNF470	0	285	9.046093733	5.27E-53	1.31E-52
METTL4	0	284	9.041022744	7.39E-53	1.84E-52
STK11IP	0	284	9.041022744	7.39E-53	1.84E-52
ZNF211	0	282	9.030826977	1.46E-52	3.62E-52
POLE4	0	280	9.020558641	2.88E-52	7.12E-52
RNF138	0	279	9.015396936	4.04E-52	1.00E-51

ZNF428	0	279	9.015396936	4.04E-52	1.00E-51
ZNF432	0	279	9.015396936	4.04E-52	1.00E-51
ZNF557	0	278	9.010216697	5.68E-52	1.40E-51
DCXR	0	277	9.00501779	7.99E-52	1.97E-51
ZFAND2B	0	277	9.00501779	7.99E-52	1.97E-51
ZFP28	0	276	8.999800081	1.12E-51	2.77E-51
ZNF404	0	275	8.994563433	1.58E-51	3.89E-51
ZNF175	0	274	8.989307707	2.23E-51	5.47E-51
ABCA7	0	273	8.984032765	3.14E-51	7.69E-51
SPHK2	0	271	8.973424665	6.23E-51	1.52E-50
CHCHD5	0	270	8.968091221	8.78E-51	2.14E-50
LOC284454	0	270	8.968091221	8.78E-51	2.14E-50
RBFA	0	270	8.968091221	8.78E-51	2.14E-50
BOLA3	0	268	8.957364815	1.74E-50	4.25E-50
ZNF426	0	267	8.951971556	2.46E-50	5.99E-50
COX7A1	0	266	8.94655806	3.47E-50	8.44E-50
GEMIN7	0	266	8.94655806	3.47E-50	8.44E-50
OSR1	0	266	8.94655806	3.47E-50	8.44E-50
C19orf47	0	265	8.941124174	4.90E-50	1.19E-49
GCDH	0	265	8.941124174	4.90E-50	1.19E-49
PDE6D	0	264	8.935669743	6.93E-50	1.68E-49
ZNF585A	0	264	8.935669743	6.93E-50	1.68E-49
ABHD8	0	263	8.930194613	9.78E-50	2.36E-49
C18orf21	0	263	8.930194613	9.78E-50	2.36E-49
FBXO46	0	262	8.924698626	1.38E-49	3.33E-49
STRA13	0	261	8.919181621	1.95E-49	4.70E-49
USE1	0	260	8.913643437	2.76E-49	6.62E-49
CLHC1	0	259	8.908083912	3.91E-49	9.36E-49
SMAD7	0	258	8.90250288	5.53E-49	1.32E-48
ZNF440	0	256	8.891275624	1.11E-48	2.64E-48
PHLPP1	0	255	8.885629061	1.57E-48	3.74E-48
DCAF15	0	253	8.874269199	3.15E-48	7.47E-48

LYPD1	0	253	8.874269199	3.15E-48	7.47E-48
ZNF181	0	253	8.874269199	3.15E-48	7.47E-48
ZNF700	0	252	8.868555548	4.47E-48	1.06E-47
ZNF564	0	251	8.862819178	6.33E-48	1.50E-47
ALKBH6	0	250	8.857059909	8.98E-48	2.12E-47
JOSD2	0	250	8.857059909	8.98E-48	2.12E-47
MED26	0	250	8.857059909	8.98E-48	2.12E-47
MYPOP	0	250	8.857059909	8.98E-48	2.12E-47
PRX	0	250	8.857059909	8.98E-48	2.12E-47
ANKRD36	0	249	8.851277556	1.27E-47	3.00E-47
PDCL3	0	249	8.851277556	1.27E-47	3.00E-47
ZNF606	0	249	8.851277556	1.27E-47	3.00E-47
FAM117B	0	248	8.845471934	1.81E-47	4.25E-47
RBM45	0	246	8.833790129	3.65E-47	8.55E-47
ARID5A	0	245	8.827913563	5.19E-47	1.21E-46
SIRT6	0	244	8.822012962	7.38E-47	1.72E-46
APBA3	0	243	8.816088128	1.05E-46	2.44E-46
C2orf49	0	243	8.816088128	1.05E-46	2.44E-46
RAB40B	0	243	8.816088128	1.05E-46	2.44E-46
BBS5	0	242	8.810138861	1.49E-46	3.47E-46
RAB3D	0	241	8.80416496	2.12E-46	4.93E-46
ZNF513	0	241	8.80416496	2.12E-46	4.93E-46
RHPN2	0	240	8.79816622	3.02E-46	7.00E-46
PPP1R13L	0	239	8.792142432	4.30E-46	9.95E-46
ZFP14	0	239	8.792142432	4.30E-46	9.95E-46
ZNF611	0	238	8.786093387	6.13E-46	1.41E-45
QTRT1	0	237	8.780018873	8.74E-46	2.01E-45
SPR	0	237	8.780018873	8.74E-46	2.01E-45
ZNF347	0	237	8.780018873	8.74E-46	2.01E-45
PTPN18	0	236	8.773918673	1.25E-45	2.86E-45
LRFN3	0	235	8.767792571	1.78E-45	4.07E-45
PELI1	0	235	8.767792571	1.78E-45	4.07E-45

TNFAIP8L1	0	235	8.767792571	1.78E-45	4.07E-45
WASH5P	0	234	8.761640344	2.53E-45	5.80E-45
RELB	0	233	8.755461769	3.62E-45	8.27E-45
SFT2D3	0	232	8.749256619	5.16E-45	1.18E-44
ACVR2A	0	231	8.743024666	7.37E-45	1.68E-44
TSSC1	0	231	8.743024666	7.37E-45	1.68E-44
GPATCH1	0	230	8.736765675	1.05E-44	2.39E-44
KLF11	0	230	8.736765675	1.05E-44	2.39E-44
ZNF132	0	230	8.736765675	1.05E-44	2.39E-44
HOOK2	0	229	8.730479412	1.51E-44	3.41E-44
MOCOS	0	228	8.724165638	2.15E-44	4.87E-44
PIEZO2	0	228	8.724165638	2.15E-44	4.87E-44
ZNF649	0	228	8.724165638	2.15E-44	4.87E-44
C19orf54	0	227	8.717824111	3.08E-44	6.95E-44
LOC339803	0	227	8.717824111	3.08E-44	6.95E-44
LPHN1	0	226	8.711454587	4.41E-44	9.93E-44
ZNF441	0	226	8.711454587	4.41E-44	9.93E-44
FIZ1	0	225	8.705056815	6.31E-44	1.42E-43
ZNF473	0	225	8.705056815	6.31E-44	1.42E-43
ZNF549	0	225	8.705056815	6.31E-44	1.42E-43
HSD11B1L	0	224	8.698630546	9.03E-44	2.03E-43
SAMD1	0	224	8.698630546	9.03E-44	2.03E-43
ZNF324	0	224	8.698630546	9.03E-44	2.03E-43
ZNF559	0	224	8.698630546	9.03E-44	2.03E-43
LIG1	0	223	8.692175524	1.29E-43	2.89E-43
NMI	0	223	8.692175524	1.29E-43	2.89E-43
SP110	0	223	8.692175524	1.29E-43	2.89E-43
SFXN5	0	222	8.68569149	1.85E-43	4.14E-43
OSBPL6	0	221	8.679178184	2.66E-43	5.93E-43
MCOLN1	0	220	8.672635338	3.81E-43	8.48E-43
NUDT19	0	220	8.672635338	3.81E-43	8.48E-43
ZNF43	0	220	8.672635338	3.81E-43	8.48E-43

ANO8	0	219	8.666062684	5.47E-43	1.22E-42
C19orf26	0	219	8.666062684	5.47E-43	1.22E-42
C2orf47	0	219	8.666062684	5.47E-43	1.22E-42
ZNF568	0	218	8.659459949	7.85E-43	1.74E-42
KCNK6	0	217	8.652826857	1.13E-42	2.49E-42
PRADC1	0	217	8.652826857	1.13E-42	2.49E-42
ZNF28	0	216	8.646163126	1.62E-42	3.58E-42
FBXO41	0	215	8.639468474	2.33E-42	5.13E-42
FSD1	0	214	8.632742611	3.34E-42	7.34E-42
LPPR3	0	214	8.632742611	3.34E-42	7.34E-42
RFXANK	0	214	8.632742611	3.34E-42	7.34E-42
ZFP112	0	214	8.632742611	3.34E-42	7.34E-42
GTPBP3	0	213	8.625985244	4.81E-42	1.05E-41
C18orf54	0	212	8.619196079	6.92E-42	1.51E-41
CHST10	0	212	8.619196079	6.92E-42	1.51E-41
PTRHD1	0	212	8.619196079	6.92E-42	1.51E-41
SIRT7	0	212	8.619196079	6.92E-42	1.51E-41
ZNF439	0	212	8.619196079	6.92E-42	1.51E-41
ZNF71	0	212	8.619196079	6.92E-42	1.51E-41
ZNF737	0	212	8.619196079	6.92E-42	1.51E-41
CEP76	0	211	8.612374813	9.96E-42	2.17E-41
CHAF1A	0	207	8.584762582	4.29E-41	9.30E-41
ZNF566	0	207	8.584762582	4.29E-41	9.30E-41
CCDC142	0	206	8.577776151	6.19E-41	1.34E-40
MATN3	0	206	8.577776151	6.19E-41	1.34E-40
ZNF225	0	206	8.577776151	6.19E-41	1.34E-40
ZNF430	0	206	8.577776151	6.19E-41	1.34E-40
LINC00116	0	205	8.570755724	8.93E-41	1.93E-40
PIGF	0	205	8.570755724	8.93E-41	1.93E-40
ST3GAL5	0	204	8.563700966	1.29E-40	2.77E-40
TPRKB	0	204	8.563700966	1.29E-40	2.77E-40
ZNF136	0	204	8.563700966	1.29E-40	2.77E-40

ZNF155	0	204	8.563700966	1.29E-40	2.77E-40
LOC285074	0	203	8.556611541	1.86E-40	3.99E-40
HSPE1	0	202	8.549487107	2.69E-40	5.76E-40
SH3RF3-AS1	0	202	8.549487107	2.69E-40	5.76E-40
OXLD1	0	200	8.535131814	5.62E-40	1.20E-39
LRRC8E	0	199	8.527900245	8.13E-40	1.73E-39
PPAP2C	0	199	8.527900245	8.13E-40	1.73E-39
TRABD2A	0	199	8.527900245	8.13E-40	1.73E-39
ZBTB14	0	198	8.520632244	1.18E-39	2.50E-39
OCEL1	0	197	8.513327444	1.70E-39	3.62E-39
TMEM241	0	197	8.513327444	1.70E-39	3.62E-39
LOC400657	0	196	8.505985468	2.47E-39	5.23E-39
LOC654433	0	196	8.505985468	2.47E-39	5.23E-39
ZNF576	0	195	8.498605938	3.58E-39	7.57E-39
PHLDB3	0	194	8.491188466	5.18E-39	1.10E-38
ZNF773	0	194	8.491188466	5.18E-39	1.10E-38
SDHAF1	0	192	8.476238125	1.09E-38	2.30E-38
TRMT61B	0	192	8.476238125	1.09E-38	2.30E-38
ATG4D	0	191	8.468704452	1.58E-38	3.32E-38
WDR54	0	191	8.468704452	1.58E-38	3.32E-38
ZNF708	0	191	8.468704452	1.58E-38	3.32E-38
CCDC57	0	190	8.461131232	2.30E-38	4.81E-38
LOC654342	0	190	8.461131232	2.30E-38	4.81E-38
ZNF2	0	190	8.461131232	2.30E-38	4.81E-38
ZNF569	0	190	8.461131232	2.30E-38	4.81E-38
ZSCAN22	0	190	8.461131232	2.30E-38	4.81E-38
SLC4A3	0	189	8.453518048	3.34E-38	6.97E-38
GEN1	0	188	8.445864476	4.86E-38	1.01E-37
ZNF415	0	188	8.445864476	4.86E-38	1.01E-37
ZNF506	0	188	8.445864476	4.86E-38	1.01E-37
ALDH16A1	0	187	8.438170084	7.07E-38	1.47E-37
ZNF416	0	187	8.438170084	7.07E-38	1.47E-37

CENPO	0	186	8.430434435	1.03E-37	2.13E-37
HTRA2	0	186	8.430434435	1.03E-37	2.13E-37
RPIA	0	186	8.430434435	1.03E-37	2.13E-37
TFPT	0	186	8.430434435	1.03E-37	2.13E-37
ZCCHC2	0	186	8.430434435	1.03E-37	2.13E-37
ZNF845	0	186	8.430434435	1.03E-37	2.13E-37
ANKRD36B	0	185	8.422657085	1.50E-37	3.10E-37
ZNF14	0	185	8.422657085	1.50E-37	3.10E-37
KIAA1841	0	184	8.41483758	2.18E-37	4.51E-37
FAM161A	0	183	8.406975462	3.18E-37	6.55E-37
KLHL26	0	183	8.406975462	3.18E-37	6.55E-37
ZNF350	0	183	8.406975462	3.18E-37	6.55E-37
ZNF567	0	183	8.406975462	3.18E-37	6.55E-37
CCDC94	0	181	8.391121511	6.76E-37	1.39E-36
LOC90784	0	181	8.391121511	6.76E-37	1.39E-36
MZF1	0	181	8.391121511	6.76E-37	1.39E-36
ZNF414	0	181	8.391121511	6.76E-37	1.39E-36
ZNF578	0	180	8.38312872	9.86E-37	2.02E-36
ZNF446	0	179	8.375091401	1.44E-36	2.95E-36
ZNF765	0	179	8.375091401	1.44E-36	2.95E-36
MITD1	0	178	8.367009055	2.10E-36	4.29E-36
ZNF461	0	178	8.367009055	2.10E-36	4.29E-36
ZNF577	0	178	8.367009055	2.10E-36	4.29E-36
DOHH	0	177	8.358881174	3.07E-36	6.25E-36
PTOV1-AS1	0	177	8.358881174	3.07E-36	6.25E-36
GALM	0	176	8.350707243	4.49E-36	9.12E-36
MFSD9	0	175	8.342486736	6.57E-36	1.33E-35
NFATC1	0	175	8.342486736	6.57E-36	1.33E-35
SARS2	0	175	8.342486736	6.57E-36	1.33E-35
ZNF490	0	174	8.33421912	9.62E-36	1.94E-35
NPAS1	0	173	8.325903852	1.41E-35	2.84E-35
POLD1	0	173	8.325903852	1.41E-35	2.84E-35

PUS10	0	173	8.325903852	1.41E-35	2.84E-35
WDSUB1	0	173	8.325903852	1.41E-35	2.84E-35
ZNF701	0	173	8.325903852	1.41E-35	2.84E-35
SEC11C	0	172	8.317540379	2.06E-35	4.15E-35
ZNF615	0	172	8.317540379	2.06E-35	4.15E-35
ZNF420	0	171	8.309128139	3.02E-35	6.06E-35
CERS4	0	170	8.30066656	4.43E-35	8.88E-35
KLF2	0	169	8.29215506	6.51E-35	1.30E-34
LOC10028891	0	169	8.29215506	6.51E-35	1.30E-34
DTNA	0	167	8.274979917	1.40E-34	2.79E-34
LIN37	0	167	8.274979917	1.40E-34	2.79E-34
ZNF524	0	166	8.266315055	2.06E-34	4.10E-34
LINC00662	0	165	8.257597838	3.03E-34	6.01E-34
PAFAH1B3	0	165	8.257597838	3.03E-34	6.01E-34
MORN2	0	164	8.248827629	4.46E-34	8.83E-34
RNF152	0	164	8.248827629	4.46E-34	8.83E-34
ZNF8	0	164	8.248827629	4.46E-34	8.83E-34
COBLL1	0	163	8.240003778	6.57E-34	1.29E-33
ZNF607	0	162	8.231125627	9.67E-34	1.90E-33
SGOL2	0	161	8.222192502	1.42E-33	2.80E-33
FAHD2B	0	160	8.213203719	2.10E-33	4.11E-33
NHEJ1	0	160	8.213203719	2.10E-33	4.11E-33
MARS2	0	159	8.204158579	3.10E-33	6.05E-33
EPOR	0	158	8.195056372	4.57E-33	8.90E-33
KPTN	0	158	8.195056372	4.57E-33	8.90E-33
PID1	0	158	8.195056372	4.57E-33	8.90E-33
ZNF17	0	158	8.195056372	4.57E-33	8.90E-33
C2orf42	0	157	8.185896373	6.75E-33	1.31E-32
APOE	0	156	8.176677843	9.98E-33	1.94E-32
CTU1	0	156	8.176677843	9.98E-33	1.94E-32
ZNF180	0	156	8.176677843	9.98E-33	1.94E-32
ZNF846	0	156	8.176677843	9.98E-33	1.94E-32

TNFAIP6	0	155	8.167400029	1.48E-32	2.86E-32
ZNF671	0	155	8.167400029	1.48E-32	2.86E-32
ZNF804A	0	155	8.167400029	1.48E-32	2.86E-32
OSGEPL1	0	154	8.158062165	2.18E-32	4.22E-32
ZNF493	0	153	8.148663467	3.23E-32	6.24E-32
DNAJC27	0	152	8.139203138	4.79E-32	9.21E-32
GPATCH11	0	152	8.139203138	4.79E-32	9.21E-32
LRRC45	0	152	8.139203138	4.79E-32	9.21E-32
ZNF816	0	152	8.139203138	4.79E-32	9.21E-32
ZNF527	0	151	8.129680363	7.09E-32	1.36E-31
ZNF738	0	151	8.129680363	7.09E-32	1.36E-31
BOLA3-AS1	0	150	8.120094315	1.05E-31	2.02E-31
PCGF1	0	150	8.120094315	1.05E-31	2.02E-31
THAP8	0	150	8.120094315	1.05E-31	2.02E-31
ZNF714	0	150	8.120094315	1.05E-31	2.02E-31
LPAR2	0	149	8.110444145	1.56E-31	2.98E-31
DUS3L	0	148	8.10072899	2.32E-31	4.42E-31
ELAC1	0	148	8.10072899	2.32E-31	4.42E-31
KIAA1328	0	148	8.10072899	2.32E-31	4.42E-31
LOC284385	0	147	8.090947969	3.44E-31	6.55E-31
WASH2P	0	147	8.090947969	3.44E-31	6.55E-31
FBXW9	0	146	8.081100183	5.11E-31	9.70E-31
HAUS5	0	146	8.081100183	5.11E-31	9.70E-31
MPND	0	145	8.071184714	7.60E-31	1.44E-30
VMAC	0	144	8.061200626	1.13E-30	2.14E-30
ZIK1	0	144	8.061200626	1.13E-30	2.14E-30
ZNF583	0	144	8.061200626	1.13E-30	2.14E-30
APC2	0	143	8.051146961	1.68E-30	3.18E-30
GLB1L	0	143	8.051146961	1.68E-30	3.18E-30
ZNF570	0	142	8.041022744	2.51E-30	4.72E-30
CCDC61	0	141	8.030826977	3.74E-30	7.03E-30
ZNF699	0	141	8.030826977	3.74E-30	7.03E-30

ARRDC2	0	140	8.020558641	5.58E-30	1.04E-29
ZNF587B	0	140	8.020558641	5.58E-30	1.04E-29
ZNF154	0	139	8.010216697	8.33E-30	1.55E-29
ZNF554	0	139	8.010216697	8.33E-30	1.55E-29
AOX1	0	138	7.999800081	1.24E-29	2.32E-29
MPP4	0	138	7.999800081	1.24E-29	2.32E-29
SCN1B	0	138	7.999800081	1.24E-29	2.32E-29
TMEM143	0	138	7.999800081	1.24E-29	2.32E-29
CDKN2D	0	137	7.989307707	1.86E-29	3.45E-29
FAM98C	0	137	7.989307707	1.86E-29	3.45E-29
DOK6	0	136	7.978738465	2.78E-29	5.15E-29
ZNF431	0	136	7.978738465	2.78E-29	5.15E-29
ZNF784	0	136	7.978738465	2.78E-29	5.15E-29
C2orf74	0	135	7.968091221	4.17E-29	7.68E-29
FIGN	0	135	7.968091221	4.17E-29	7.68E-29
RMDN2	0	135	7.968091221	4.17E-29	7.68E-29
ZNF626	0	135	7.968091221	4.17E-29	7.68E-29
ITGA4	0	133	7.94655806	9.35E-29	1.71E-28
ZNF586	0	133	7.94655806	9.35E-29	1.71E-28
KCNE4	0	132	7.935669743	1.40E-28	2.56E-28
ZNF836	0	132	7.935669743	1.40E-28	2.56E-28
RPSAP58	0	131	7.924698626	2.10E-28	3.84E-28
ATOH8	0	129	7.90250288	4.75E-28	8.63E-28
FN3K	0	129	7.90250288	4.75E-28	8.63E-28
HSD17B14	0	129	7.90250288	4.75E-28	8.63E-28
TRAPPC6A	0	128	7.891275624	7.14E-28	1.29E-27
ZNF844	0	128	7.891275624	7.14E-28	1.29E-27
FAM228B	0	127	7.879960311	1.07E-27	1.94E-27
ZNF44	0	127	7.879960311	1.07E-27	1.94E-27
CYP27A1	0	125	7.857059909	2.44E-27	4.38E-27
TTC30A	0	125	7.857059909	2.44E-27	4.38E-27
ZNF284	0	125	7.857059909	2.44E-27	4.38E-27

ZNF93	0	125	7.857059909	2.44E-27	4.38E-27
BCL2L12	0	124	7.845471934	3.68E-27	6.60E-27
CCNE1	0	124	7.845471934	3.68E-27	6.60E-27
FKBP1B	0	124	7.845471934	3.68E-27	6.60E-27
ZNF222	0	124	7.845471934	3.68E-27	6.60E-27
PLA2R1	0	123	7.833790129	5.55E-27	9.92E-27
RNASEH2A	0	123	7.833790129	5.55E-27	9.92E-27
ZFP82	0	123	7.833790129	5.55E-27	9.92E-27
ZNF880	0	123	7.833790129	5.55E-27	9.92E-27
FBXO36	0	122	7.822012962	8.38E-27	1.49E-26
GEMIN6	0	122	7.822012962	8.38E-27	1.49E-26
LGALSL	0	122	7.822012962	8.38E-27	1.49E-26
TMEM178A	0	122	7.822012962	8.38E-27	1.49E-26
ZNF546	0	122	7.822012962	8.38E-27	1.49E-26
ZNF628	0	122	7.822012962	8.38E-27	1.49E-26
ZNF808	0	122	7.822012962	8.38E-27	1.49E-26
ZNF256	0	121	7.810138861	1.27E-26	2.25E-26
ZNF283	0	121	7.810138861	1.27E-26	2.25E-26
C19orf55	0	120	7.79816622	1.91E-26	3.39E-26
CBWD2	0	120	7.79816622	1.91E-26	3.39E-26
GALNT3	0	120	7.79816622	1.91E-26	3.39E-26
ZNF121	0	120	7.79816622	1.91E-26	3.39E-26
CCDC68	0	119	7.786093387	2.90E-26	5.12E-26
ZNF547	0	119	7.786093387	2.90E-26	5.12E-26
ATP8B3	0	118	7.773918673	4.39E-26	7.73E-26
CERS6	0	117	7.761640344	6.65E-26	1.17E-25
TLE2	0	117	7.761640344	6.65E-26	1.17E-25
ZNF565	0	117	7.761640344	6.65E-26	1.17E-25
ZNF613	0	117	7.761640344	6.65E-26	1.17E-25
ZNF223	0	116	7.749256619	1.01E-25	1.77E-25
ILF3-AS1	0	115	7.736765675	1.53E-25	2.68E-25
PNMAL1	0	115	7.736765675	1.53E-25	2.68E-25

RABL2A	0	115	7.736765675	1.53E-25	2.68E-25
FUZ	0	114	7.724165638	2.33E-25	4.06E-25
GNG7	0	114	7.724165638	2.33E-25	4.06E-25
ZNF551	0	112	7.698630546	5.38E-25	9.34E-25
ENOSF1	0	111	7.68569149	8.20E-25	1.42E-24
HEXDC	0	111	7.68569149	8.20E-25	1.42E-24
JAK3	0	111	7.68569149	8.20E-25	1.42E-24
TMEM177	0	111	7.68569149	8.20E-25	1.42E-24
ZNF790	0	111	7.68569149	8.20E-25	1.42E-24
CYP2S1	0	110	7.672635338	1.25E-24	2.15E-24
ICAM4	0	110	7.672635338	1.25E-24	2.15E-24
INO80C	0	110	7.672635338	1.25E-24	2.15E-24
WDPCP	0	110	7.672635338	1.25E-24	2.15E-24
APLF	0	109	7.659459949	1.91E-24	3.28E-24
ZNF600	0	109	7.659459949	1.91E-24	3.28E-24
DLGAP1-AS1	0	108	7.646163126	2.91E-24	4.98E-24
PKI55	0	108	7.646163126	2.91E-24	4.98E-24
SUV420H2	0	108	7.646163126	2.91E-24	4.98E-24
ZNF230	0	108	7.646163126	2.91E-24	4.98E-24
GCSHP3	0	107	7.632742611	4.45E-24	7.59E-24
LENG1	0	107	7.632742611	4.45E-24	7.59E-24
ZNF788	0	106	7.619196079	6.80E-24	1.16E-23
PECR	0	105	7.605521142	1.04E-23	1.77E-23
WDR83	0	105	7.605521142	1.04E-23	1.77E-23
ZNF460	0	105	7.605521142	1.04E-23	1.77E-23
ZNF599	0	105	7.605521142	1.04E-23	1.77E-23
NUP35	0	104	7.591715342	1.59E-23	2.69E-23
PPP1R14A	0	104	7.591715342	1.59E-23	2.69E-23
ZNF382	0	104	7.591715342	1.59E-23	2.69E-23
ZRANB3	0	104	7.591715342	1.59E-23	2.69E-23
CACNG7	0	103	7.577776151	2.44E-23	4.11E-23
CCDC159	0	103	7.577776151	2.44E-23	4.11E-23

LOC10013069	0	103	7.577776151	2.44E-23	4.11E-23
RGL3	0	103	7.577776151	2.44E-23	4.11E-23
ZNF253	0	103	7.577776151	2.44E-23	4.11E-23
GAREML	0	102	7.563700966	3.75E-23	6.28E-23
LIPE	0	102	7.563700966	3.75E-23	6.28E-23
TPGS1	0	102	7.563700966	3.75E-23	6.28E-23
ZNF69	0	102	7.563700966	3.75E-23	6.28E-23
CABYR	0	101	7.549487107	5.76E-23	9.61E-23
IL27RA	0	101	7.549487107	5.76E-23	9.61E-23
ZNF550	0	100	7.535131814	8.86E-23	1.47E-22
LOC10050612	0	99	7.520632244	1.36E-22	2.26E-22
PDCD2L	0	99	7.520632244	1.36E-22	2.26E-22
SCN7A	0	99	7.520632244	1.36E-22	2.26E-22
ZNF675	0	99	7.520632244	1.36E-22	2.26E-22
ZNF813	0	98	7.505985468	2.10E-22	3.47E-22
C19orf44	0	97	7.491188466	3.23E-22	5.34E-22
HMHA1	0	97	7.491188466	3.23E-22	5.34E-22
RDH13	0	96	7.476238125	4.99E-22	8.21E-22
TMEM182	0	96	7.476238125	4.99E-22	8.21E-22
TPM3P9	0	95	7.461131232	7.71E-22	1.26E-21
ZNF429	0	95	7.461131232	7.71E-22	1.26E-21
ZNF677	0	95	7.461131232	7.71E-22	1.26E-21
HECW2	0	94	7.445864476	1.19E-21	1.95E-21
MEF2BNB	0	94	7.445864476	1.19E-21	1.95E-21
ZNF829	0	94	7.445864476	1.19E-21	1.95E-21
C2orf76	0	93	7.430434435	1.84E-21	3.00E-21
TIGD1	0	93	7.430434435	1.84E-21	3.00E-21
ZNF383	0	93	7.430434435	1.84E-21	3.00E-21
ZNF486	0	93	7.430434435	1.84E-21	3.00E-21
ZNF497	0	93	7.430434435	1.84E-21	3.00E-21
ZNF543	0	93	7.430434435	1.84E-21	3.00E-21
ACYP2	0	92	7.41483758	2.85E-21	4.64E-21

GRIN3B	0	92	7.41483758	2.85E-21	4.64E-21
PTGIR	0	92	7.41483758	2.85E-21	4.64E-21
PLEKHF1	0	91	7.399070264	4.42E-21	7.17E-21
QPCT	0	91	7.399070264	4.42E-21	7.17E-21
B3GNTL1	0	90	7.38312872	6.87E-21	1.11E-20
ZNF792	0	90	7.38312872	6.87E-21	1.11E-20
SHC2	0	89	7.367009055	1.07E-20	1.72E-20
TMEM200C	0	89	7.367009055	1.07E-20	1.72E-20
ZNF433	0	89	7.367009055	1.07E-20	1.72E-20
GCA	0	88	7.350707243	1.66E-20	2.66E-20
TMEM91	0	88	7.350707243	1.66E-20	2.66E-20
ZNF552	0	88	7.350707243	1.66E-20	2.66E-20
LOC285033	0	87	7.33421912	2.58E-20	4.12E-20
PLCD4	0	87	7.33421912	2.58E-20	4.12E-20
ZNF235	0	87	7.33421912	2.58E-20	4.12E-20
ZNF610	0	87	7.33421912	2.58E-20	4.12E-20
LOC10050737	0	86	7.317540379	4.02E-20	6.40E-20
SLC6A16	0	86	7.317540379	4.02E-20	6.40E-20
ZNF749	0	86	7.317540379	4.02E-20	6.40E-20
METAP1D	0	85	7.30066656	6.27E-20	9.95E-20
MSL3P1	0	85	7.30066656	6.27E-20	9.95E-20
TBC1D8	0	85	7.30066656	6.27E-20	9.95E-20
ICA1L	0	84	7.283593047	9.79E-20	1.55E-19
ZNF345	0	84	7.283593047	9.79E-20	1.55E-19
LYPD6	0	82	7.248827629	2.39E-19	3.77E-19
MAFG-AS1	0	82	7.248827629	2.39E-19	3.77E-19
SULT1C4	0	82	7.248827629	2.39E-19	3.77E-19
ZNF491	0	82	7.248827629	2.39E-19	3.77E-19
CAPS	0	81	7.231125627	3.75E-19	5.88E-19
ANKRD39	0	80	7.213203719	5.88E-19	9.20E-19
LOC10050605	0	80	7.213203719	5.88E-19	9.20E-19
TYMS	0	80	7.213203719	5.88E-19	9.20E-19

BUB1	0	79	7.195056372	9.24E-19	1.44E-18
SETBP1	0	79	7.195056372	9.24E-19	1.44E-18
RAB3A	0	78	7.176677843	1.45E-18	2.26E-18
CYS1	0	77	7.158062165	2.28E-18	3.54E-18
SLMO1	0	77	7.158062165	2.28E-18	3.54E-18
ZNF135	0	77	7.158062165	2.28E-18	3.54E-18
ZNF77	0	77	7.158062165	2.28E-18	3.54E-18
DUSP19	0	76	7.139203138	3.60E-18	5.56E-18
NRTN	0	76	7.139203138	3.60E-18	5.56E-18
ZNF793	0	76	7.139203138	3.60E-18	5.56E-18
ZNF818P	0	76	7.139203138	3.60E-18	5.56E-18
BCL2L11	0	75	7.120094315	5.68E-18	8.75E-18
LIPT1	0	75	7.120094315	5.68E-18	8.75E-18
ZNF30	0	75	7.120094315	5.68E-18	8.75E-18
ZNF418	0	75	7.120094315	5.68E-18	8.75E-18
CALCRL	0	74	7.10072899	8.96E-18	1.38E-17
LINC00085	0	74	7.10072899	8.96E-18	1.38E-17
AP1S3	0	73	7.081100183	1.42E-17	2.17E-17
ARHGAP33	0	73	7.081100183	1.42E-17	2.17E-17
LOC10050571	0	73	7.081100183	1.42E-17	2.17E-17
RFX2	0	73	7.081100183	1.42E-17	2.17E-17
ZNF57	0	73	7.081100183	1.42E-17	2.17E-17
DFNB59	0	72	7.061200626	2.24E-17	3.43E-17
LENG9	0	72	7.061200626	2.24E-17	3.43E-17
SERPINB2	0	72	7.061200626	2.24E-17	3.43E-17
TUBA4A	0	72	7.061200626	2.24E-17	3.43E-17
GAREM	0	71	7.041022744	3.56E-17	5.40E-17
ZC3H8	0	71	7.041022744	3.56E-17	5.40E-17
ZNF709	0	71	7.041022744	3.56E-17	5.40E-17
HJURP	0	70	7.020558641	5.64E-17	8.53E-17
IFIH1	0	70	7.020558641	5.64E-17	8.53E-17
LINC00665	0	70	7.020558641	5.64E-17	8.53E-17

NPB	0	70	7.020558641	5.64E-17	8.53E-17
NPHP1	0	70	7.020558641	5.64E-17	8.53E-17
SECTM1	0	70	7.020558641	5.64E-17	8.53E-17
ZNF221	0	70	7.020558641	5.64E-17	8.53E-17
C2orf16	0	69	6.999800081	8.97E-17	1.35E-16
LIN7B	0	69	6.999800081	8.97E-17	1.35E-16
REEP6	0	69	6.999800081	8.97E-17	1.35E-16
ZNF324B	0	69	6.999800081	8.97E-17	1.35E-16
CAMKMT	0	68	6.978738465	1.43E-16	2.14E-16
FLT3LG	0	68	6.978738465	1.43E-16	2.14E-16
ZNF530	0	68	6.978738465	1.43E-16	2.14E-16
ZNF850	0	68	6.978738465	1.43E-16	2.14E-16
COLEC12	0	67	6.957364815	2.27E-16	3.40E-16
COMP	0	67	6.957364815	2.27E-16	3.40E-16
PCSK4	0	67	6.957364815	2.27E-16	3.40E-16
TRAPPC2P1	0	67	6.957364815	2.27E-16	3.40E-16
ZNF573	0	67	6.957364815	2.27E-16	3.40E-16
CILP2	0	66	6.935669743	3.63E-16	5.39E-16
EID2B	0	66	6.935669743	3.63E-16	5.39E-16
EXOSC5	0	66	6.935669743	3.63E-16	5.39E-16
REL	0	66	6.935669743	3.63E-16	5.39E-16
ZNF823	0	66	6.935669743	3.63E-16	5.39E-16
CDCA7	0	65	6.913643437	5.79E-16	8.58E-16
RAB11B-AS1	0	64	6.891275624	9.27E-16	1.37E-15
ZNF100	0	64	6.891275624	9.27E-16	1.37E-15
LOC10012839	0	63	6.868555548	1.48E-15	2.18E-15
ANKAR	0	62	6.845471934	2.38E-15	3.49E-15
C2orf27A	0	62	6.845471934	2.38E-15	3.49E-15
LOC729950	0	62	6.845471934	2.38E-15	3.49E-15
ABHD3	0	61	6.822012962	3.82E-15	5.57E-15
ATCAY	0	61	6.822012962	3.82E-15	5.57E-15
IFI30	0	61	6.822012962	3.82E-15	5.57E-15

LOC254128	0	61	6.822012962	3.82E-15	5.57E-15
MREG	0	61	6.822012962	3.82E-15	5.57E-15
ZNF571	0	61	6.822012962	3.82E-15	5.57E-15
SLC25A10	0	60	6.79816622	6.15E-15	8.92E-15
LMTK3	0	59	6.773918673	9.91E-15	1.43E-14
LOC10050646	0	59	6.773918673	9.91E-15	1.43E-14
PPFIA3	0	59	6.773918673	9.91E-15	1.43E-14
SCN3A	0	59	6.773918673	9.91E-15	1.43E-14
ZNF137P	0	59	6.773918673	9.91E-15	1.43E-14
CD3EAP	0	58	6.749256619	1.60E-14	2.30E-14
CGREF1	0	58	6.749256619	1.60E-14	2.30E-14
SEMA6B	0	58	6.749256619	1.60E-14	2.30E-14
NDC80	0	57	6.724165638	2.59E-14	3.70E-14
ZNF653	0	57	6.724165638	2.59E-14	3.70E-14
ARHGEF4	0	56	6.698630546	4.19E-14	5.96E-14
BARD1	0	56	6.698630546	4.19E-14	5.96E-14
DTNB	0	56	6.698630546	4.19E-14	5.96E-14
GPR75	0	56	6.698630546	4.19E-14	5.96E-14
LRFN1	0	56	6.698630546	4.19E-14	5.96E-14
SLC16A14	0	56	6.698630546	4.19E-14	5.96E-14
THNSL2	0	56	6.698630546	4.19E-14	5.96E-14
MYOM1	0	55	6.672635338	6.79E-14	9.62E-14
NR2C2AP	0	55	6.672635338	6.79E-14	9.62E-14
ZNF85	0	55	6.672635338	6.79E-14	9.62E-14
GRHL1	0	54	6.646163126	1.10E-13	1.56E-13
TMEM86B	0	54	6.646163126	1.10E-13	1.56E-13
ZNF575	0	54	6.646163126	1.10E-13	1.56E-13
ZNF582	0	53	6.619196079	1.79E-13	2.52E-13
GGN	0	52	6.591715342	2.92E-13	4.09E-13
KCNS3	0	52	6.591715342	2.92E-13	4.09E-13
PDE11A	0	52	6.591715342	2.92E-13	4.09E-13
ZNF285	0	52	6.591715342	2.92E-13	4.09E-13

ZNF519	0	52	6.591715342	2.92E-13	4.09E-13
ZNF625	0	52	6.591715342	2.92E-13	4.09E-13
PTGER1	0	51	6.563700966	4.77E-13	6.63E-13
TBXA2R	0	51	6.563700966	4.77E-13	6.63E-13
TMEM194B	0	51	6.563700966	4.77E-13	6.63E-13
ZNF563	0	51	6.563700966	4.77E-13	6.63E-13
SCN2A	0	50	6.535131814	7.79E-13	1.08E-12
VSIG10L	0	50	6.535131814	7.79E-13	1.08E-12
GIPR	0	49	6.505985468	1.28E-12	1.76E-12
U2AF1L4	0	49	6.505985468	1.28E-12	1.76E-12
DIRC3	0	48	6.476238125	2.09E-12	2.88E-12
DPF1	0	48	6.476238125	2.09E-12	2.88E-12
ZNF224	0	48	6.476238125	2.09E-12	2.88E-12
ZNF101	0	47	6.445864476	3.44E-12	4.71E-12
CCDC74A	0	46	6.41483758	5.67E-12	7.73E-12
CFD	0	46	6.41483758	5.67E-12	7.73E-12
LOC10050554	0	45	6.38312872	9.35E-12	1.27E-11
PLIN4	0	45	6.38312872	9.35E-12	1.27E-11
TMEM17	0	45	6.38312872	9.35E-12	1.27E-11
XDH	0	45	6.38312872	9.35E-12	1.27E-11
BCL2	0	44	6.350707243	1.54E-11	2.09E-11
C2orf88	0	44	6.350707243	1.54E-11	2.09E-11
CIRBP-AS1	0	44	6.350707243	1.54E-11	2.09E-11
NR4A2	0	44	6.350707243	1.54E-11	2.09E-11
CCDC138	0	43	6.317540379	2.56E-11	3.44E-11
FANCL	0	43	6.317540379	2.56E-11	3.44E-11
FBXO27	0	43	6.317540379	2.56E-11	3.44E-11
IL18R1	0	43	6.317540379	2.56E-11	3.44E-11
TMEM198	0	43	6.317540379	2.56E-11	3.44E-11
ZNF665	0	43	6.317540379	2.56E-11	3.44E-11
ZNF837	0	43	6.317540379	2.56E-11	3.44E-11
LOC400680	0	42	6.283593047	4.25E-11	5.68E-11

SLC27A5	0	42	6.283593047	4.25E-11	5.68E-11
ZNF682	0	42	6.283593047	4.25E-11	5.68E-11
MYO1F	0	41	6.248827629	7.06E-11	9.40E-11
TTC30B	0	41	6.248827629	7.06E-11	9.40E-11
CKAP2L	0	40	6.213203719	1.18E-10	1.56E-10
RDH14	0	40	6.213203719	1.18E-10	1.56E-10
ZNF702P	0	40	6.213203719	1.18E-10	1.56E-10
C19orf71	0	39	6.176677843	1.97E-10	2.60E-10
DNER	0	39	6.176677843	1.97E-10	2.60E-10
PEX11G	0	39	6.176677843	1.97E-10	2.60E-10
APCDD1	0	38	6.139203138	3.29E-10	4.32E-10
CD37	0	38	6.139203138	3.29E-10	4.32E-10
CHAC2	0	38	6.139203138	3.29E-10	4.32E-10
FZD5	0	38	6.139203138	3.29E-10	4.32E-10
LINC00663	0	38	6.139203138	3.29E-10	4.32E-10
TTC32	0	38	6.139203138	3.29E-10	4.32E-10
ZNF233	0	38	6.139203138	3.29E-10	4.32E-10
ANKRD23	0	37	6.10072899	5.52E-10	7.22E-10
FSCN2	0	37	6.10072899	5.52E-10	7.22E-10
MYO5B	0	37	6.10072899	5.52E-10	7.22E-10
STXBP2	0	37	6.10072899	5.52E-10	7.22E-10
ZNF560	0	37	6.10072899	5.52E-10	7.22E-10
HAUS8	0	36	6.061200626	9.28E-10	1.21E-09
KCNA7	0	36	6.061200626	9.28E-10	1.21E-09
KCNG2	0	36	6.061200626	9.28E-10	1.21E-09
RAPGEF4	0	36	6.061200626	9.28E-10	1.21E-09
ZSCAN5A	0	36	6.061200626	9.28E-10	1.21E-09
DLX1	0	35	6.020558641	1.56E-09	2.02E-09
FRZB	0	35	6.020558641	1.56E-09	2.02E-09
KIAA1683	0	35	6.020558641	1.56E-09	2.02E-09
PCBP1-AS1	0	35	6.020558641	1.56E-09	2.02E-09
VAX2	0	35	6.020558641	1.56E-09	2.02E-09

WBP1	0	35	6.020558641	1.56E-09	2.02E-09
GALNT13	0	34	5.978738465	2.64E-09	3.40E-09
NCAPH	0	34	5.978738465	2.64E-09	3.40E-09
TMEFF2	0	34	5.978738465	2.64E-09	3.40E-09
TSPAN10	0	34	5.978738465	2.64E-09	3.40E-09
ZNF763	0	34	5.978738465	2.64E-09	3.40E-09
ANKRD24	0	33	5.935669743	4.48E-09	5.73E-09
DDX11L2	0	33	5.935669743	4.48E-09	5.73E-09
EPS8L1	0	33	5.935669743	4.48E-09	5.73E-09
FOSB	0	33	5.935669743	4.48E-09	5.73E-09
LRP1B	0	33	5.935669743	4.48E-09	5.73E-09
KATNAL2	0	32	5.891275624	7.60E-09	9.66E-09
KCNJ14	0	32	5.891275624	7.60E-09	9.66E-09
LINC00526	0	32	5.891275624	7.60E-09	9.66E-09
TNNT1	0	32	5.891275624	7.60E-09	9.66E-09
ZNF799	0	32	5.891275624	7.60E-09	9.66E-09
FBLN7	0	31	5.845471934	1.29E-08	1.64E-08
PAX8	0	31	5.845471934	1.29E-08	1.64E-08
PHOSPHO2	0	31	5.845471934	1.29E-08	1.64E-08
TTN-AS1	0	31	5.845471934	1.29E-08	1.64E-08
XIRP2	0	31	5.845471934	1.29E-08	1.64E-08
ATP6V1B1	0	30	5.79816622	2.21E-08	2.77E-08
FBXO48	0	30	5.79816622	2.21E-08	2.77E-08
FXYD1	0	30	5.79816622	2.21E-08	2.77E-08
IGFBP2	0	30	5.79816622	2.21E-08	2.77E-08
NLRC4	0	30	5.79816622	2.21E-08	2.77E-08
UNC13A	0	30	5.79816622	2.21E-08	2.77E-08
ZNF681	0	30	5.79816622	2.21E-08	2.77E-08
ZNF781	0	30	5.79816622	2.21E-08	2.77E-08
ZNF443	0	29	5.749256619	3.78E-08	4.72E-08
ZNF582-AS1	0	29	5.749256619	3.78E-08	4.72E-08
ASF1B	0	28	5.698630546	6.49E-08	8.05E-08

CCDC121	0	28	5.698630546	6.49E-08	8.05E-08
LOC10028812	0	28	5.698630546	6.49E-08	8.05E-08
NFKBID	0	28	5.698630546	6.49E-08	8.05E-08
PNMAL2	0	28	5.698630546	6.49E-08	8.05E-08
RPL17	0	28	5.698630546	6.49E-08	8.05E-08
TGFBR3L	0	28	5.698630546	6.49E-08	8.05E-08
DBP	0	27	5.646163126	1.12E-07	1.38E-07
GIPC3	0	27	5.646163126	1.12E-07	1.38E-07
LOC728752	0	27	5.646163126	1.12E-07	1.38E-07
ADAT3	0	26	5.591715342	1.93E-07	2.37E-07
CENPA	0	26	5.591715342	1.93E-07	2.37E-07
CERS1	0	26	5.591715342	1.93E-07	2.37E-07
DYSF	0	26	5.591715342	1.93E-07	2.37E-07
LOC10013169	0	26	5.591715342	1.93E-07	2.37E-07
PALM3	0	26	5.591715342	1.93E-07	2.37E-07
RASIP1	0	26	5.591715342	1.93E-07	2.37E-07
SLC23A3	0	26	5.591715342	1.93E-07	2.37E-07
TMC4	0	26	5.591715342	1.93E-07	2.37E-07
TNFSF9	0	26	5.591715342	1.93E-07	2.37E-07
B3GNT8	0	25	5.535131814	3.34E-07	4.07E-07
BST2	0	25	5.535131814	3.34E-07	4.07E-07
GRIN2D	0	25	5.535131814	3.34E-07	4.07E-07
LYPD3	0	25	5.535131814	3.34E-07	4.07E-07
PBX4	0	25	5.535131814	3.34E-07	4.07E-07
SPINT2	0	25	5.535131814	3.34E-07	4.07E-07
TAF4B	0	25	5.535131814	3.34E-07	4.07E-07
TMPRSS9	0	25	5.535131814	3.34E-07	4.07E-07
TSSK6	0	25	5.535131814	3.34E-07	4.07E-07
ZNF396	0	25	5.535131814	3.34E-07	4.07E-07
ZNF540	0	25	5.535131814	3.34E-07	4.07E-07
B9D2	0	24	5.476238125	5.81E-07	7.04E-07
CYP1B1	0	24	5.476238125	5.81E-07	7.04E-07

INHA	0	24	5.476238125	5.81E-07	7.04E-07
NANOS3	0	24	5.476238125	5.81E-07	7.04E-07
S1PR5	0	24	5.476238125	5.81E-07	7.04E-07
VN1R1	0	24	5.476238125	5.81E-07	7.04E-07
WDR62	0	24	5.476238125	5.81E-07	7.04E-07
ATP6V1E2	0	23	5.41483758	1.01E-06	1.22E-06
BCL11A	0	23	5.41483758	1.01E-06	1.22E-06
C19orf40	0	23	5.41483758	1.01E-06	1.22E-06
CBX3P2	0	23	5.41483758	1.01E-06	1.22E-06
ETV2	0	23	5.41483758	1.01E-06	1.22E-06
LOC113230	0	23	5.41483758	1.01E-06	1.22E-06
TM6SF2	0	23	5.41483758	1.01E-06	1.22E-06
CACNG8	0	22	5.350707243	1.77E-06	2.12E-06
EMILIN2	0	22	5.350707243	1.77E-06	2.12E-06
GPR39	0	22	5.350707243	1.77E-06	2.12E-06
SPC25	0	22	5.350707243	1.77E-06	2.12E-06
STAT4	0	22	5.350707243	1.77E-06	2.12E-06
YJEFN3	0	22	5.350707243	1.77E-06	2.12E-06
ZGLP1	0	22	5.350707243	1.77E-06	2.12E-06
ZNF525	0	22	5.350707243	1.77E-06	2.12E-06
B4GALT6	0	21	5.283593047	3.11E-06	3.69E-06
CA11	0	21	5.283593047	3.11E-06	3.69E-06
CAPN12	0	21	5.283593047	3.11E-06	3.69E-06
FAM84A	0	21	5.283593047	3.11E-06	3.69E-06
INPP5D	0	21	5.283593047	3.11E-06	3.69E-06
NRXN1	0	21	5.283593047	3.11E-06	3.69E-06
PAIP2B	0	21	5.283593047	3.11E-06	3.69E-06
SWSAP1	0	21	5.283593047	3.11E-06	3.69E-06
TMEM169	0	21	5.283593047	3.11E-06	3.69E-06
ZNF790-AS1	0	21	5.283593047	3.11E-06	3.69E-06
ASB3	0	20	5.213203719	5.48E-06	6.46E-06
FLJ43681	0	20	5.213203719	5.48E-06	6.46E-06

FUT2	0	20	5.213203719	5.48E-06	6.46E-06
HMSD	0	20	5.213203719	5.48E-06	6.46E-06
KCNIP3	0	20	5.213203719	5.48E-06	6.46E-06
MBP	0	20	5.213203719	5.48E-06	6.46E-06
PARD6G-AS1	0	20	5.213203719	5.48E-06	6.46E-06
SPTBN4	0	20	5.213203719	5.48E-06	6.46E-06
STAP2	0	20	5.213203719	5.48E-06	6.46E-06
C11orf91	4	23	2.41483758	0.000245505	0.000276126
KISS1	5	144	4.739272531	2.28E-33	4.45E-33
HTR1D	5	43	2.995612284	1.86E-08	2.34E-08
IL21R	5	21	1.961664952	0.002078095	0.002279512
CALB2	6	20	1.628241218	0.008235004	0.008846767
ELMOD1	7	33	2.128314821	4.74E-05	5.43E-05
SNCG	7	28	1.891275624	0.000521169	0.000581235
PRG4	8	39	2.176677843	7.28E-06	8.54E-06
GAL	10	112	3.376702451	9.92E-22	1.63E-21
NLRP10	10	57	2.402237543	8.66E-09	1.10E-08
GPR157	11	24	1.016806506	0.044476259	0.046738677
PHOSPHO1	13	38	1.438763419	0.000881898	0.00097823
CHST6	13	32	1.190835906	0.008367285	0.008985166
PTGES3L	13	22	0.650267525	0.18981119	0.195339428
CSMD2	14	48	1.668883203	3.10E-05	3.57E-05
SLC22A31	14	29	0.941901697	0.03817112	0.040172274
SPINT1	14	23	0.607482658	0.207049909	0.21263187
PPM1J	14	20	0.405848797	0.415651232	0.421644644
MMP10	15	95	2.554240637	1.42E-14	2.04E-14
PLXDC1	16	95	2.461131232	4.90E-14	6.96E-14
KRT7	16	62	1.845471934	3.81E-07	4.64E-07
S100A2	17	31	0.758009093	0.07496069	0.078195034
PAPPA2	18	89	2.197084054	9.41E-12	1.28E-11
KRTAP2-3	18	40	1.043278718	0.008056084	0.008661705
RHBDL2	18	25	0.365206812	0.409530241	0.415597355

PKP2	18	21	0.113668045	0.805883372	0.809199338
ST6GALNAC5	19	65	1.665715924	1.29E-06	1.55E-06
CCDC154	19	2	-3.356651889	4.39E-05	5.03E-05
HSD11B2	19	3	-2.771689389	0.000188439	0.000212586
HOXC6	19	5	-2.034723794	0.001841999	0.00202366
LRP4-AS1	19	6	-1.771689389	0.004533576	0.004917041
RUNDC3A	19	6	-1.771689389	0.004533576	0.004917041
SUMO1P3	19	6	-1.771689389	0.004533576	0.004917041
CYP27B1	19	7	-1.549296967	0.009889791	0.010585893
GLTPD2	19	7	-1.549296967	0.009889791	0.010585893
CNKSR1	19	8	-1.356651889	0.019513337	0.02074039
RET	19	9	-1.186726888	0.035370496	0.037270032
HTR7	19	10	-1.034723794	0.059625617	0.06243181
KCNJ5	19	13	-0.656212171	0.202129385	0.20779731
PRH1-PRR4	19	14	-0.549296967	0.276746081	0.282830801
PALD1	19	15	-0.449761294	0.364902858	0.371418981
CSNK2A3	19	24	0.228310611	0.604955343	0.610190684
UNC5B-AS1	19	23	0.166910067	0.708278637	0.713118602
ABCG4	19	19	-0.108724376	0.816255436	0.819298033
RTN4RL1	20	1	-4.430652471	4.35E-06	5.14E-06
SERTAD4-AS	20	60	1.476238125	2.04E-05	2.37E-05
ZSWIM5	20	2	-3.430652471	2.34E-05	2.70E-05
C1orf229	20	3	-2.84568997	0.00010309	0.00011718
CHI3L2	20	4	-2.430652471	0.000364991	0.000408369
PLBD1	20	4	-2.430652471	0.000364991	0.000408369
RXFP4	20	5	-2.108724376	0.001077286	0.001191407
ACOT4	20	7	-1.623297549	0.00618598	0.006675832
GNAO1	20	7	-1.623297549	0.00618598	0.006675832
KCNRG	20	7	-1.623297549	0.00618598	0.006675832
C1orf204	20	8	-1.430652471	0.012604213	0.013462768
ACSBG1	20	9	-1.260727469	0.023565246	0.024967235
HIST2H2BA	20	9	-1.260727469	0.023565246	0.024967235

TIAF1	20	9	-1.260727469	0.023565246	0.024967235
WFIKKN2	20	9	-1.260727469	0.023565246	0.024967235
RDH5	20	10	-1.108724376	0.040923352	0.043036894
TMEM56-RW	20	10	-1.108724376	0.040923352	0.043036894
CCDC153	20	11	-0.971220852	0.066660828	0.069667345
DNASE1L2	20	11	-0.971220852	0.066660828	0.069667345
GLS2	20	11	-0.971220852	0.066660828	0.069667345
MPP7	20	35	0.698630546	0.07808227	0.081429547
CATSPER2	20	12	-0.84568997	0.102672674	0.106533472
HIST2H3D	20	12	-0.84568997	0.102672674	0.106533472
LOC254100	20	12	-0.84568997	0.102672674	0.106533472
TMEM30B	20	13	-0.730212753	0.15052929	0.155446076
LDLRAD2	20	14	-0.623297549	0.211257809	0.216867653
FAM167B	20	15	-0.523761875	0.285179163	0.291296806
RASD1	20	28	0.376702451	0.368927602	0.375466655
LOC10013098	20	16	-0.430652471	0.371826586	0.378145967
LOC653653	20	16	-0.430652471	0.371826586	0.378145967
MEDAG	21	0	-5.501041799	1.20E-06	1.44E-06
PLEKHH1	21	3	-2.916079298	5.62E-05	6.43E-05
LCTL	21	58	1.356939196	8.48E-05	9.67E-05
TMEM255B	21	57	1.331848215	0.000122539	0.000139024
CYP46A1	21	4	-2.501041799	0.000205298	0.000231338
COX20	21	6	-1.916079298	0.001646523	0.001812092
H19	21	6	-1.916079298	0.001646523	0.001812092
MKNK1-AS1	21	6	-1.916079298	0.001646523	0.001812092
PPP1R36	21	6	-1.916079298	0.001646523	0.001812092
CASZ1	21	7	-1.693686877	0.003833752	0.004169025
FAM106A	21	7	-1.693686877	0.003833752	0.004169025
PPYR1	21	7	-1.693686877	0.003833752	0.004169025
LOC388152	21	8	-1.501041799	0.008058053	0.008662628
ADPGK-AS1	21	9	-1.331116797	0.015525027	0.016554234
GNN	21	9	-1.331116797	0.015525027	0.016554234

OAZ3	21	14	-0.693686877	0.159023136	0.164022146
BHLHE41	21	26	0.199397919	0.636506403	0.641766107
TMEM72-AS1	22	0	-5.568155995	6.63E-07	8.00E-07
DDI1	22	1	-4.568155995	1.23E-06	1.48E-06
CHD5	22	2	-3.568155995	6.61E-06	7.76E-06
MTRNR2L4	22	2	-3.568155995	6.61E-06	7.76E-06
SDR42E1	22	2	-3.568155995	6.61E-06	7.76E-06
SAMD15	22	3	-2.983193494	3.06E-05	3.53E-05
ZRANB2-AS2	22	3	-2.983193494	3.06E-05	3.53E-05
NOXRED1	22	4	-2.568155995	0.000114966	0.000130547
ADAM20P1	22	6	-1.983193494	0.000981089	0.001086096
RIC3	22	6	-1.983193494	0.000981089	0.001086096
TAS2R19	22	6	-1.983193494	0.000981089	0.001086096
PTPRCAP	22	7	-1.760801072	0.002355812	0.002578532
ST8SIA1	22	7	-1.760801072	0.002355812	0.002578532
LOC595101	22	8	-1.568155995	0.005102698	0.005521265
MAB21L3	22	9	-1.398230993	0.010121842	0.010829082
CNNM1	22	10	-1.2462279	0.018610162	0.019799286
LOC10012953	22	11	-1.108724376	0.032025994	0.033791513
KIF21A	22	12	-0.983193494	0.051998886	0.054533884
C17orf82	22	13	-0.867716276	0.080190651	0.083578084
MFSD4	22	13	-0.867716276	0.080190651	0.083578084
C12orf61	22	14	-0.760801072	0.118124887	0.1222905
PCSK6	22	14	-0.760801072	0.118124887	0.1222905
TMED6	22	14	-0.760801072	0.118124887	0.1222905
FOXN3-AS1	22	16	-0.568155995	0.227581146	0.233348367
IFI27	22	20	-0.2462279	0.580315607	0.585715985
PLCH2	22	25	0.075700195	0.857354155	0.859665973
PCED1B	23	126	2.344993592	3.29E-17	5.00E-17
FBXL22	23	90	1.859566764	7.75E-10	1.01E-09
KCNE3	23	2	-3.632286332	3.52E-06	4.16E-06
OMG	23	4	-2.632286332	6.41E-05	7.33E-05

ACBD7	23	5	-2.310358237	0.000207647	0.000233934
ASIC2	23	5	-2.310358237	0.000207647	0.000233934
LOC10050608	23	6	-2.047323831	0.000580649	0.000646924
NOVA1	23	7	-1.82493141	0.001436267	0.001583482
FAM13C	23	8	-1.632286332	0.003202723	0.003492781
FOXD2	23	8	-1.632286332	0.003202723	0.003492781
FCRLB	23	9	-1.462361331	0.006535226	0.007044945
LRFN5	23	9	-1.462361331	0.006535226	0.007044945
SCARNA12	23	9	-1.462361331	0.006535226	0.007044945
GNG12-AS1	23	12	-1.047323831	0.036347515	0.038281441
SYT2	23	12	-1.047323831	0.036347515	0.038281441
TMEM45B	23	14	-0.82493141	0.086647793	0.09018161
MTL5	23	15	-0.725395736	0.125317236	0.129633368
PDZD7	23	19	-0.384358819	0.388889812	0.395164677
ADAMTS4	23	20	-0.310358237	0.480983515	0.487285972
ZNF774	23	26	0.068153386	0.868751362	0.870870268
CLYBL	24	4	-2.693686877	3.56E-05	4.10E-05
RAMP2-AS1	24	4	-2.693686877	3.56E-05	4.10E-05
ESAM	24	5	-2.371758782	0.000118676	0.0001347
CRNDE	24	6	-2.108724376	0.000341517	0.000382791
ABCC2	24	7	-1.886331955	0.000869275	0.000964776
CHRNA10	24	8	-1.693686877	0.001993681	0.002188914
RNLS	24	8	-1.693686877	0.001993681	0.002188914
LRRC46	24	9	-1.523761875	0.004181339	0.004540371
TTC36	24	9	-1.523761875	0.004181339	0.004540371
AUNIP	24	10	-1.371758782	0.008114879	0.008720117
RLTPR	24	12	-1.108724376	0.025128282	0.026581787
MSTO2P	24	15	-0.786796281	0.092886993	0.096559442
C11orf88	24	16	-0.693686877	0.132168556	0.136675396
RPS10P7	24	16	-0.693686877	0.132168556	0.136675396
IGF2-AS	24	36	0.476238125	0.205529964	0.211182038
ZYG11A	24	27	0.061200626	0.879663317	0.881695654

SERP2	25	0	-5.752580566	1.14E-07	1.40E-07
C1QTNF9	25	1	-4.752580566	1.88E-07	2.31E-07
SLC16A9	25	1	-4.752580566	1.88E-07	2.31E-07
PIPOX	25	3	-3.167618065	4.87E-06	5.74E-06
SLC5A9	25	3	-3.167618065	4.87E-06	5.74E-06
KCTD4	25	72	1.417344436	6.00E-06	7.06E-06
CDH15	25	67	1.313508625	3.79E-05	4.35E-05
APITD1	25	6	-2.167618065	0.000199714	0.000225159
EFCAB5	25	6	-2.167618065	0.000199714	0.000225159
CALML6	25	7	-1.945225644	0.000522556	0.000582657
RNASEK	25	7	-1.945225644	0.000522556	0.000582657
FAM72D	25	8	-1.752580566	0.001231549	0.001359509
TMEM88	25	10	-1.430652471	0.005283395	0.005713618
NEURL	25	11	-1.293148947	0.009824605	0.010521166
PMEL	25	14	-0.945225644	0.045010969	0.047294222
RHOV	25	16	-0.752580566	0.09890496	0.102733285
ASAH2	25	17	-0.665117724	0.138692435	0.143317351
SNORA40	25	36	0.417344436	0.262381712	0.268466823
KDM4A-AS1	25	21	-0.360263143	0.397819884	0.404081097
AKAP3	26	2	-3.809164094	5.30E-07	6.42E-07
CRYM-AS1	26	6	-2.224201593	0.000116169	0.000131883
TMEM254-AS	26	6	-2.224201593	0.000116169	0.000131883
HOGA1	26	7	-2.001809172	0.000312153	0.000350255
OTOGL	26	7	-2.001809172	0.000312153	0.000350255
LINC00327	26	8	-1.809164094	0.000755316	0.000839969
BTBD18	26	10	-1.487235999	0.003410248	0.003714692
TAS1R3	26	11	-1.349732475	0.006499174	0.007008984
FRMD5	26	48	0.775798407	0.023769906	0.025175532
LINC00304	26	16	-0.809164094	0.073174773	0.076383078
CTSS	26	42	0.583153329	0.100253172	0.104092144
PLEKHN1	26	33	0.235230025	0.532490824	0.538350704
ANAPC15	26	26	-0.108724376	0.78576183	0.789604186

ZMYND15	27	2	-3.863611878	2.82E-07	3.44E-07
LOC10013099	27	3	-3.278649377	1.42E-06	1.70E-06
PRKCQ-AS1	27	4	-2.863611878	6.01E-06	7.06E-06
CGN	27	5	-2.541683783	2.16E-05	2.50E-05
SEZ6	27	6	-2.278649377	6.72E-05	7.68E-05
ANKRD65	27	7	-2.056256956	0.000185374	0.000209173
GOLGA2P5	27	7	-2.056256956	0.000185374	0.000209173
ZMIZ1-AS1	27	9	-1.693686877	0.001042926	0.001153734
C16orf3	27	11	-1.404180259	0.004261918	0.004625296
SYCE1L	27	11	-1.404180259	0.004261918	0.004625296
TAS2R10	27	12	-1.278649377	0.007818203	0.008410573
SHBG	27	13	-1.16317216	0.013567536	0.014485766
CCR7	27	14	-1.056256956	0.022398974	0.023752524
ARHGDIB	27	46	0.659950078	0.05474335	0.057381319
FLJ27352	27	17	-0.776149037	0.078167198	0.08150723
LOC554206	27	20	-0.541683783	0.200442353	0.206090103
USP2	27	22	-0.404180259	0.327909785	0.334200929
HSPB2	27	24	-0.278649377	0.490626072	0.49692594
WNT9A	27	30	0.043278718	0.909869075	0.911269412
CCDC30	28	3	-3.331116797	7.63E-07	9.21E-07
IL34	28	4	-2.916079298	3.30E-06	3.91E-06
C1orf115	28	7	-2.108724376	0.000109483	0.000124393
PPFIA2	28	8	-1.916079298	0.00027856	0.000312854
LOC10050567	28	10	-1.594151203	0.001387191	0.001530349
SGCG	28	10	-1.594151203	0.001387191	0.001530349
TNFSF13	28	12	-1.331116797	0.00520197	0.005627121
HSD3BP4	28	15	-1.009188702	0.025090416	0.026552522
ITGB4	28	15	-1.009188702	0.025090416	0.026552522
MRVI1-AS1	28	15	-1.009188702	0.025090416	0.026552522
TC2N	28	16	-0.916079298	0.038789372	0.040811938
KCNC1	29	2	-3.966705371	7.99E-08	9.89E-08
LINC00597	29	2	-3.966705371	7.99E-08	9.89E-08

LOC10012678	29	3	-3.38174287	4.11E-07	4.99E-07
CASC2	29	5	-2.644777276	6.80E-06	7.98E-06
FLJ31813	29	5	-2.644777276	6.80E-06	7.98E-06
AGT	29	75	1.262113319	2.38E-05	2.75E-05
LOC284581	29	8	-1.966705371	0.000167644	0.000189537
P2RX5-TAX1I	29	9	-1.79678037	0.000398688	0.000445975
AKR7L	29	12	-1.38174287	0.003432578	0.003737451
SLC9A7P1	29	14	-1.159350449	0.010723231	0.011466207
FAM90A1	29	22	-0.507273752	0.211296989	0.216879362
LLGL2	29	24	-0.38174287	0.336368858	0.342598723
TMEM132B	30	318	3.297267984	1.84E-57	4.79E-57
KRT4	30	0	-6.015614971	6.45E-09	8.23E-09
DDX12P	30	5	-2.693686877	3.80E-06	4.50E-06
AKR7A3	30	6	-2.430652471	1.27E-05	1.48E-05
HAP1	30	6	-2.430652471	1.27E-05	1.48E-05
BATF2	30	8	-2.015614971	0.000100335	0.000114098
SMG7-AS1	30	9	-1.84568997	0.000244172	0.000274667
PRKCH	30	11	-1.556183353	0.001145854	0.001265984
EGR2	30	57	0.817275043	0.009971684	0.010671356
CCDC7	30	16	-1.015614971	0.019764123	0.020998373
PKD1L2	30	16	-1.015614971	0.019764123	0.020998373
TMC7	30	16	-1.015614971	0.019764123	0.020998373
DICER1-AS1	30	17	-0.92815213	0.030571818	0.032272434
SH3GL1P2	30	17	-0.92815213	0.030571818	0.032272434
HNF1A	30	19	-0.767687458	0.06589258	0.068882886
FGF11	30	42	0.376702451	0.271151801	0.277258663
KCNJ12	30	24	-0.430652471	0.274057009	0.280119265
SIRT4	30	26	-0.315175253	0.414123408	0.420203962
RAB11FIP4	30	27	-0.260727469	0.495250278	0.501349419
OLAH	31	3	-3.477958186	1.19E-07	1.46E-07
IQCH	31	8	-2.062920686	5.97E-05	6.83E-05
C12orf60	31	9	-1.892995685	0.000148667	0.000168277

MRPL42P5	31	13	-1.362480968	0.002761574	0.003016102
CNTD1	31	14	-1.255565764	0.004957098	0.005368929
DISP2	31	14	-1.255565764	0.004957098	0.005368929
GSTO2	31	14	-1.255565764	0.004957098	0.005368929
ALG1L9P	31	15	-1.156030091	0.00848667	0.009108983
KIF26A	31	15	-1.156030091	0.00848667	0.009108983
UBAP1L	31	18	-0.892995685	0.033336615	0.035145893
DLG2	31	20	-0.740992591	0.06984543	0.072961402
LOC10013095	31	20	-0.740992591	0.06984543	0.072961402
RPH3AL	31	26	-0.362480968	0.343656653	0.349930213
FLVCR1-AS1	31	31	-0.108724376	0.766614599	0.771008231
LINC00854	31	31	-0.108724376	0.766614599	0.771008231
PROX1	32	3	-3.523761875	6.37E-08	7.91E-08
FAM19A2	32	6	-2.523761875	4.10E-06	4.85E-06
C1orf101	32	8	-2.108724376	3.54E-05	4.07E-05
LIPT2	32	8	-2.108724376	3.54E-05	4.07E-05
BCO2	32	9	-1.938799374	9.00E-05	0.000102518
CLEC4A	32	9	-1.938799374	9.00E-05	0.000102518
LINC00622	32	9	-1.938799374	9.00E-05	0.000102518
C10orf68	32	12	-1.523761875	0.000942227	0.001043962
EVI2A	32	12	-1.523761875	0.000942227	0.001043962
GOLGA6L9	32	12	-1.523761875	0.000942227	0.001043962
RPSAP52	32	62	0.845471934	0.00566405	0.006120183
SCN3B	32	15	-1.20183378	0.005812762	0.006277831
ZNF488	32	15	-1.20183378	0.005812762	0.006277831
FAM86C2P	32	16	-1.108724376	0.009713481	0.010408587
SH3GL1P1	32	16	-1.108724376	0.009713481	0.010408587
TBX19	32	18	-0.938799374	0.024134392	0.025547712
CCDC65	32	20	-0.786796281	0.052341454	0.054885781
FAM132A	32	21	-0.716406953	0.073709941	0.076926285
LURAP1	32	21	-0.716406953	0.073709941	0.076926285
SLFN13	32	22	-0.649292757	0.101040678	0.10489586

TMEM180	32	28	-0.301369454	0.418844423	0.424828692
MIR137HG	32	30	-0.20183378	0.581742364	0.58700428
TPTE2P1	33	3	-3.568155995	3.42E-08	4.27E-08
FSIP1	33	8	-2.153118495	2.09E-05	2.42E-05
ESRRG	33	9	-1.983193494	5.42E-05	6.21E-05
HIST2H2BF	33	9	-1.983193494	5.42E-05	6.21E-05
KTN1-AS1	33	14	-1.345763573	0.002220008	0.002432787
LOH12CR2	33	15	-1.2462279	0.003949963	0.004293605
PKP3	33	20	-0.8311904	0.038863941	0.040884887
LOC10013331	33	21	-0.760801072	0.055632889	0.058286339
RASGRP2	33	21	-0.760801072	0.055632889	0.058286339
ALPK3	33	23	-0.629556539	0.105196157	0.109101107
MAP3K14-AS	33	23	-0.629556539	0.105196157	0.109101107
ST6GALNAC2	33	25	-0.509262305	0.180832334	0.186197217
RUNX3	33	36	0.016806506	0.961415113	0.962277701
LMO3	34	5	-2.874259122	3.61E-07	4.39E-07
MYCBPAP	34	6	-2.611224716	1.31E-06	1.57E-06
PTGDR2	34	86	1.230077538	9.21E-06	1.08E-05
C16orf46	34	11	-1.736755599	0.000180582	0.000204047
SOX15	34	11	-1.736755599	0.000180582	0.000204047
TEX9	34	11	-1.736755599	0.000180582	0.000204047
FAM24B	34	15	-1.289296622	0.002663928	0.002911289
GOLGA6L10	34	15	-1.289296622	0.002663928	0.002911289
LOC10050576	34	17	-1.108724376	0.007688552	0.008274518
CCR10	34	20	-0.874259122	0.028601773	0.030215266
KIF5A	34	53	0.531733237	0.089389802	0.092998263
COL4A2-AS1	34	24	-0.611224716	0.109216093	0.113172568
KLRD1	35	6	-2.653044892	7.35E-07	8.87E-07
LOC643733	35	9	-2.068082391	1.94E-05	2.25E-05
CDK18	35	10	-1.916079298	4.84E-05	5.54E-05
PPM1E	35	13	-1.537567675	0.000498084	0.000555966
BMI1	35	14	-1.430652471	0.000965957	0.001069648

PDE3B	35	15	-1.331116797	0.001783676	0.001960136
RRAD	35	65	0.78436042	0.007907634	0.008503265
TPTE2P5	35	18	-1.068082391	0.008699486	0.009334197
AGAP9	35	24	-0.653044892	0.084750469	0.088242188
ADIRF	35	26	-0.537567675	0.147719752	0.152585124
PCSK9	35	51	0.434417949	0.166215706	0.171327673
NAGS	35	28	-0.430652471	0.237442289	0.243267755
SLC22A15	36	2	-4.278649377	9.91E-10	1.29E-09
C11orf65	36	7	-2.471294455	1.38E-06	1.65E-06
GAS2	36	9	-2.108724376	1.15E-05	1.34E-05
GAS5-AS1	36	10	-1.956721282	2.93E-05	3.38E-05
LOC10013041	36	10	-1.956721282	2.93E-05	3.38E-05
TEX22	36	15	-1.371758782	0.001186069	0.001309673
LOC10012888	36	16	-1.278649377	0.002131303	0.002336236
LOC283335	36	18	-1.108724376	0.006093173	0.006577493
FNDC5	36	19	-1.030721864	0.009753062	0.010448849
OR7E12P	36	22	-0.819217759	0.033104456	0.03491056
SNAI3	36	23	-0.755087421	0.047038146	0.049377723
ACAP1	36	29	-0.420668382	0.241031998	0.246864564
RYR3	36	29	-0.420668382	0.241031998	0.246864564
SLC25A19	36	34	-0.191186536	0.57926631	0.584808095
GATA3	36	41	0.078902627	0.810494332	0.813724638
ZNF32-AS2	37	6	-2.733215241	2.31E-07	2.82E-07
CES4A	37	9	-2.14825274	6.81E-06	7.99E-06
DLEU2L	37	11	-1.858746123	4.25E-05	4.88E-05
SEPT1	37	14	-1.510822819	0.000409393	0.000457688
TRIM69	37	15	-1.411287146	0.000783489	0.000870679
TTC40	37	16	-1.318177742	0.00143257	0.00157963
CTAGE7P	37	17	-1.2307149	0.002511651	0.002746794
SPESP1	37	17	-1.2307149	0.002511651	0.002746794
UNC79	37	19	-1.070250228	0.006891897	0.007423286
LOC399715	37	20	-0.996249647	0.010844893	0.011594711

SERTAD4	37	46	0.205384215	0.51775945	0.523728439
LPAR3	38	124	1.597544421	8.60E-11	1.14E-10
JAKMIP3	38	4	-3.356651889	7.53E-09	9.57E-09
ATPBD4-AS1	38	5	-3.034723794	3.31E-08	4.13E-08
SORBS1	38	6	-2.771689389	1.29E-07	1.59E-07
ART5	38	7	-2.549296967	4.47E-07	5.43E-07
CDHR5	38	8	-2.356651889	1.40E-06	1.68E-06
HPX	38	12	-1.771689389	5.97E-05	6.82E-05
ABCA4	38	13	-1.656212171	0.000129001	0.000146239
ABI3	38	13	-1.656212171	0.000129001	0.000146239
BRICD5	38	17	-1.269189048	0.001705398	0.00187583
CCDC103	38	17	-1.269189048	0.001705398	0.00187583
FAM222A	38	18	-1.186726888	0.002923823	0.003191074
SLFNL1-AS1	38	25	-0.7127957	0.052347561	0.054884815
NMB	38	57	0.476238125	0.111169955	0.115136098
SNORA18	38	46	0.166910067	0.596697875	0.601939505
SNORA8	38	40	-0.034723794	0.915340475	0.916631671
GNG4	39	8	-2.394126595	8.08E-07	9.74E-07
CLCA2	39	11	-1.934694976	1.58E-05	1.84E-05
SYT17	39	11	-1.934694976	1.58E-05	1.84E-05
FLJ39639	39	14	-1.586771673	0.000169387	0.00019148
NHLRC4	39	31	-0.439930284	0.203406086	0.209082286
LOC10050750	39	34	-0.306663754	0.36423444	0.370786956
FLG	40	3	-3.84568997	4.36E-10	5.71E-10
ABCC6P1	40	7	-2.623297549	1.43E-07	1.76E-07
SOX8	40	9	-2.260727469	1.38E-06	1.65E-06
DGAT2	40	12	-1.84568997	2.28E-05	2.64E-05
PROCA1	40	12	-1.84568997	2.28E-05	2.64E-05
DLG5-AS1	40	14	-1.623297549	0.000108046	0.000122778
ADPRHL1	40	16	-1.430652471	0.000418514	0.000467684
SBK1	40	17	-1.34318963	0.000770948	0.000856865
CYP2E1	40	19	-1.182724957	0.002328714	0.002550124

NUDT4	40	22	-0.971220852	0.009497591	0.010184934
LOC143666	40	32	-0.430652471	0.206598486	0.212224096
AGPHD1	40	55	0.350707243	0.238936913	0.244766936
PLEK2	40	37	-0.221199105	0.501145649	0.507251636
LINC00673	40	49	0.184057373	0.548201308	0.553947206
RGS18	40	41	-0.073100466	0.819580386	0.822423957
CELF6	41	8	-2.466276381	2.66E-07	3.25E-07
TMPO-AS1	41	10	-2.144348286	2.23E-06	2.66E-06
DCDC2B	41	15	-1.559385785	0.000140392	0.000159025
ERBB3	41	16	-1.466276381	0.000274371	0.000308238
OIP5	41	19	-1.218348867	0.001600128	0.001762149
LOC644656	41	24	-0.88131388	0.015513617	0.016545457
STAC3	41	25	-0.822420191	0.022512908	0.023866862
BZRAP1-AS1	41	27	-0.711388878	0.044229226	0.046485333
SLC2A1-AS1	41	31	-0.51208007	0.133839933	0.138357961
DACH1	42	5	-3.179113704	2.96E-09	3.80E-09
RAMP2	42	14	-1.693686877	4.33E-05	4.97E-05
OVCA2	42	15	-1.594151203	9.01E-05	0.00010255
LINC00324	42	17	-1.413578957	0.000340071	0.000381225
IMMP1L	42	18	-1.331116797	0.000621056	0.000691449
SEC14L1P1	42	19	-1.253114285	0.001092518	0.00120791
FAM21B	42	22	-1.04161018	0.004866935	0.005274933
IFI44L	42	27	-0.746154297	0.033755122	0.035577507
SLC6A17	42	28	-0.693686877	0.046400772	0.048721747
GABRA5	42	30	-0.594151203	0.082631063	0.086058411
SNX22	42	35	-0.371758782	0.25904954	0.265126849
FERMT3	42	37	-0.291588433	0.369382024	0.375831169
SLC29A2	42	41	-0.143489794	0.650401369	0.655691233
RAB42	42	49	0.113668045	0.707381907	0.712307584
ATP2A3	42	48	0.083920702	0.782768698	0.786798914
E2F8	43	6	-2.95002663	6.76E-09	8.61E-09
OCLM	43	12	-1.95002663	5.19E-06	6.12E-06

PLEKHG6	43	13	-1.834549412	1.22E-05	1.42E-05
ZNF423	43	13	-1.834549412	1.22E-05	1.42E-05
MYLK3	43	14	-1.727634209	2.72E-05	3.14E-05
LOC10065351	43	15	-1.628098535	5.75E-05	6.58E-05
ACOT1	43	17	-1.447526289	0.000223922	0.000252033
LAMB3	43	23	-1.011427175	0.005419424	0.005859911
HOMER2	43	26	-0.834549412	0.017978071	0.019137235
DNAJC12	43	72	0.634935871	0.020223399	0.021471723
GPR3	43	59	0.347653919	0.22637826	0.232145481
F11R	43	36	-0.365064129	0.261550397	0.267651297
C11orf35	43	38	-0.287061617	0.370864014	0.377289875
HBG2	44	0	-6.568155995	3.07E-12	4.21E-12
SMIM5	44	9	-2.398230993	1.56E-07	1.92E-07
F7	44	11	-2.108724376	1.24E-06	1.49E-06
LOC145474	44	12	-1.983193494	3.15E-06	3.73E-06
LOC10050687	44	17	-1.480693153	0.00014664	0.00016603
USP6	44	19	-1.320228481	0.00050012	0.000558159
GLYATL2	44	20	-1.2462279	0.000874724	0.000970549
HSD11B1	44	20	-1.2462279	0.000874724	0.000970549
LOC10012979	44	20	-1.2462279	0.000874724	0.000970549
C16orf86	44	22	-1.108724376	0.002427808	0.002656032
ATRNL1	44	23	-1.044594038	0.003867955	0.004205048
C14orf80	44	27	-0.813268492	0.019231712	0.020446622
CORO7	44	35	-0.438872978	0.177664192	0.183007476
LOC650368	44	37	-0.358702629	0.263928288	0.269978517
LOC10065276	44	58	0.289825001	0.312495486	0.318865445
ABCA6	45	0	-6.600577472	1.81E-12	2.49E-12
E2F2	45	4	-3.600577472	1.00E-10	1.33E-10
CLEC1A	45	5	-3.278649377	4.79E-10	6.26E-10
UBQLNL	45	10	-2.278649377	2.67E-07	3.25E-07
LOC149134	45	11	-2.141145854	7.35E-07	8.87E-07
AGAP11	45	12	-2.015614971	1.90E-06	2.27E-06

CDNF	45	12	-2.015614971	1.90E-06	2.27E-06
PDZK1	45	17	-1.513114631	9.55E-05	0.000108677
LRRC48	45	19	-1.352649959	0.000335453	0.000376157
MAP3K9	45	20	-1.278649377	0.000595148	0.000662888
EME1	45	23	-1.077015516	0.002742886	0.00299632
KDM4D	45	23	-1.077015516	0.002742886	0.00299632
HIST2H2BC	45	27	-0.84568997	0.01436318	0.015331065
MMRN2	45	33	-0.556183353	0.090486869	0.094114539
PIK3AP1	45	59	0.282065577	0.320841249	0.327210306
HBBP1	46	2	-4.632286332	2.01E-12	2.76E-12
WDR65	46	6	-3.047323831	1.13E-09	1.47E-09
MYO15A	46	11	-2.172854713	4.35E-07	5.29E-07
DNAJC6	46	13	-1.931846614	2.81E-06	3.34E-06
CCDC88B	46	14	-1.82493141	6.56E-06	7.71E-06
TNK1	46	16	-1.632286332	3.07E-05	3.54E-05
TECTA	46	17	-1.544823491	6.19E-05	7.08E-05
JAG2	46	25	-0.988430142	0.00476977	0.005171059
PDF	46	28	-0.82493141	0.01539094	0.016416863
CKLF	46	32	-0.632286332	0.054827438	0.057461745
GPR68	46	34	-0.544823491	0.092979306	0.096642539
C16orf89	47	4	-3.663313228	2.91E-11	3.91E-11
RBM26-AS1	47	15	-1.756422632	9.10E-06	1.06E-05
NRGN	47	22	-1.203881609	0.000816386	0.000906593
BCDIN3D-AS	47	25	-1.019457038	0.003428377	0.003733658
SLC25A35	47	25	-1.019457038	0.003428377	0.003733658
CAPS2	47	28	-0.855958306	0.011480086	0.012268782
UCP3	47	62	0.290883083	0.294699984	0.300903788
TCTEX1D4	47	51	0.009112114	0.97506148	0.975311272
UNC13D	48	10	-2.371758782	5.24E-08	6.51E-08
LOC10029436	48	11	-2.234255258	1.51E-07	1.86E-07
IL23A	48	19	-1.445759363	9.80E-05	0.000111486
SDR39U1	48	26	-0.993247158	0.003797433	0.004130681

ISLR	48	29	-0.835705881	0.012321084	0.013163954
PPL	48	30	-0.786796281	0.017482911	0.018617762
TDRKH	48	70	0.43559614	0.103938844	0.107818601
NDUFA4L2	48	41	-0.336134872	0.272334084	0.278431115
RPS6KA1	48	58	0.164294119	0.558458342	0.564092768
ITGA10	49	456	3.109455794	1.53E-77	4.81E-77
GDF10	49	2	-4.72343422	3.18E-13	4.45E-13
BEND5	49	7	-2.916079298	7.60E-10	9.92E-10
NOXO1	49	7	-2.916079298	7.60E-10	9.92E-10
NDRG2	49	10	-2.401506125	3.03E-08	3.78E-08
AGAP8	49	18	-1.553509219	3.31E-05	3.81E-05
ISG20	49	25	-1.07957803	0.00173913	0.00191226
LOC10050671	49	27	-0.968546718	0.004183059	0.004541291
PAX6	49	28	-0.916079298	0.006264411	0.006758606
PTPRE	49	31	-0.76923791	0.018543186	0.019730718
ASGR1	49	34	-0.635971379	0.046354927	0.048680154
PTCH2	49	35	-0.594151203	0.0608362	0.063665221
BSCL2	49	37	-0.513980854	0.100090466	0.103937028
SENP3	49	39	-0.438032001	0.155609802	0.160586355
AOC3	50	3	-4.167618065	8.61E-13	1.19E-12
DIRAS3	50	11	-2.293148947	5.18E-08	6.45E-08
DNAJC9-AS1	50	15	-1.84568997	2.18E-06	2.60E-06
TAS2R14	50	15	-1.84568997	2.18E-06	2.60E-06
LRRC37A	50	16	-1.752580566	4.88E-06	5.75E-06
REP15	50	18	-1.582655564	2.14E-05	2.47E-05
DLEU2	50	19	-1.504653052	4.21E-05	4.83E-05
TFAP2E	50	23	-1.22901861	0.000449596	0.000502202
RRN3P3	50	30	-0.84568997	0.009866555	0.010564641
ABCA3	50	36	-0.582655564	0.0627752	0.0656504
FBXO2	50	42	-0.360263143	0.231794781	0.237606385
TMEM51-AS1	51	6	-3.196187217	5.59E-11	7.46E-11
AGBL2	51	7	-2.973794796	2.33E-10	3.07E-10

RASL12	51	9	-2.611224716	3.10E-09	3.98E-09
RTN1	51	17	-1.693686877	6.60E-06	7.76E-06
FLRT1	51	18	-1.611224716	1.37E-05	1.60E-05
LOC642846	51	18	-1.611224716	1.37E-05	1.60E-05
CCDC144C	51	19	-1.533222204	2.74E-05	3.16E-05
ANK3	51	34	-0.693686877	0.028179442	0.029781196
LOC338817	51	41	-0.423597713	0.160183966	0.165197648
RPL13P5	51	42	-0.388832295	0.194639043	0.20022873
ITPKA	51	68	0.306313123	0.249105069	0.255015926
PRR26	51	45	-0.289296622	0.326219374	0.332608316
RAB26	51	46	-0.257587762	0.379419369	0.385742202
C11orf96	52	3	-4.224201593	2.49E-13	3.49E-13
ADORA1	52	9	-2.639239093	1.76E-09	2.27E-09
KCNIP2	52	12	-2.224201593	5.02E-08	6.25E-08
ADAM11	52	13	-2.108724376	1.35E-07	1.66E-07
APBB1IP	52	15	-1.902273498	8.26E-07	9.96E-07
NAALAD2	52	17	-1.721701253	4.17E-06	4.92E-06
RPL23AP64	52	18	-1.639239093	8.77E-06	1.03E-05
CCDC78	52	19	-1.561236581	1.77E-05	2.06E-05
SNX32	52	19	-1.561236581	1.77E-05	2.06E-05
AURKB	52	25	-1.165307904	0.000601872	0.000670186
CASKIN1	52	27	-1.054276592	0.001555198	0.001713153
KLHL35	52	28	-1.001809172	0.002411958	0.002639431
ASH1L-AS1	52	29	-0.951183099	0.003658162	0.003980296
ATP1A1OS	52	33	-0.764769975	0.015805448	0.016847494
FAM5C	53	3	-4.25168233	1.34E-13	1.89E-13
GREM2	53	11	-2.377213212	1.02E-08	1.29E-08
TSNAXIP1	53	14	-2.029289908	2.07E-07	2.54E-07
LOC646214	53	20	-1.514716736	2.26E-05	2.62E-05
FOXD2-AS1	53	25	-1.192788641	0.00041802	0.000467199
PPM1H	53	27	-1.081757328	0.001105681	0.001222118
BATF3	53	46	-0.313082874	0.280741167	0.286838646

B4GALNT1	53	61	0.094092507	0.727934959	0.732531455
FOS	53	60	0.070245765	0.79591463	0.799600842
MST1L	54	12	-2.278649377	1.73E-08	2.18E-08
MAPT	54	31	-0.909415568	0.004343003	0.004711657
RASAL2-AS1	54	36	-0.693686877	0.023920154	0.025329511
SLC25A21	54	36	-0.693686877	0.023920154	0.025329511
INSC	54	55	-0.082252165	0.76592019	0.770458684
LOC10028897	55	6	-3.305121589	4.95E-12	6.77E-12
PALMD	55	6	-3.305121589	4.95E-12	6.77E-12
C15orf59	55	9	-2.720159088	3.16E-10	4.15E-10
CCDC89	55	19	-1.642156576	4.70E-06	5.55E-06
SPTBN2	55	26	-1.189644371	0.000334665	0.000375327
CCDC24	55	29	-1.032103094	0.001383848	0.001526984
C11orf70	55	34	-0.802621248	0.009658195	0.010352895
FIGNL2	55	38	-0.642156576	0.033267658	0.035077928
ASPA	56	0	-6.916079298	6.17E-15	8.95E-15
ANGPTL1	56	6	-3.331116797	2.70E-12	3.70E-12
ANKRD34A	56	153	1.341308545	2.50E-10	3.29E-10
SPTBN5	56	11	-2.456647679	1.96E-09	2.53E-09
PGAM1P5	56	17	-1.828616457	6.35E-07	7.68E-07
PRSS27	56	17	-1.828616457	6.35E-07	7.68E-07
CCDC73	56	19	-1.668151785	2.99E-06	3.55E-06
FAM186B	56	20	-1.594151203	6.14E-06	7.22E-06
HOXB8	56	36	-0.746154297	0.014229678	0.015190643
CNIH3	56	87	0.526864198	0.030912581	0.032625537
BTBD11	56	42	-0.523761875	0.073715671	0.076911707
LINC00115	56	42	-0.523761875	0.073715671	0.076911707
PLEKHA8P1	56	47	-0.361490446	0.204261973	0.209906797
KCNK1	56	63	0.061200626	0.817111672	0.820052056
SYT14	57	12	-2.356651889	3.44E-09	4.41E-09
TBC1D10C	57	13	-2.241174672	9.86E-09	1.25E-08
TROAP	57	24	-1.356651889	5.22E-05	5.98E-05

TNFSF18	57	76	0.306313123	0.223057948	0.228770622
LINC00263	57	57	-0.108724376	0.687340822	0.692394799
ATP1B2	58	8	-2.966705371	1.47E-11	1.99E-11
RPP25	58	8	-2.966705371	1.47E-11	1.99E-11
CEMP1	58	13	-2.266265653	5.79E-09	7.39E-09
LOC10013058	58	13	-2.266265653	5.79E-09	7.39E-09
NR2E3	58	19	-1.718777858	1.20E-06	1.44E-06
GDPGP1	58	20	-1.644777276	2.52E-06	3.00E-06
MUC6	58	20	-1.644777276	2.52E-06	3.00E-06
RPS15AP10	58	20	-1.644777276	2.52E-06	3.00E-06
C17orf104	58	32	-0.966705371	0.001864382	0.002047961
HIST3H2A	58	39	-0.681303152	0.021238663	0.022534336
RAG1	58	44	-0.507273752	0.077098011	0.080413833
SMOC1	58	52	-0.266265653	0.333308338	0.339614407
CGNL1	59	3	-4.406404925	3.27E-15	4.77E-15
NT5M	59	12	-2.406404925	1.16E-09	1.50E-09
CARD14	59	16	-1.991367425	6.23E-08	7.74E-08
GABRB3	59	21	-1.599050002	3.29E-06	3.90E-06
LOC440354	59	21	-1.599050002	3.29E-06	3.90E-06
XRCC6BP1	59	32	-0.991367425	0.001355056	0.001495426
FGFBP3	59	50	-0.347511235	0.209220889	0.214804881
NUDT8	59	54	-0.236479923	0.383696312	0.390039639
CTSH	59	62	-0.037171115	0.887288114	0.888995751
NOS1AP	60	18	-1.84568997	2.13E-07	2.61E-07
RHD	60	22	-1.556183353	4.24E-06	5.02E-06
SEMA4A	60	22	-1.556183353	4.24E-06	5.02E-06
LOC440288	60	24	-1.430652471	1.55E-05	1.81E-05
DUOX1	60	35	-0.886331955	0.003262876	0.003557638
MT1L	61	241	1.873427623	4.77E-24	8.13E-24
LOC10050563	61	8	-3.039461713	2.48E-12	3.41E-12
KANSL1-AS1	61	20	-1.717533619	6.46E-07	7.81E-07
LOC729737	61	21	-1.647144291	1.35E-06	1.62E-06

LOC10028863	61	26	-1.339021995	3.43E-05	3.94E-05
ATP2A1	61	28	-1.232106791	0.000102918	0.000117002
BIRC3	61	38	-0.7915342	0.007084277	0.007629447
SLC16A13	61	39	-0.754059495	0.009820733	0.010518462
SYT16	61	96	0.545500787	0.019298966	0.020515331
MED31	61	57	-0.206571699	0.436719302	0.442671473
GPR65	62	13	-2.362480968	6.75E-10	8.81E-10
GLI1	62	134	1.003168504	2.66E-06	3.16E-06
LGALS9	62	35	-0.933637669	0.001782038	0.001958611
DHRS13	62	36	-0.892995685	0.002617156	0.002861175
IQSEC3	62	104	0.637519032	0.005094264	0.005512903
SPHK1	63	218	1.682180025	4.31E-19	6.75E-19
TTC18	63	14	-2.278649377	1.14E-09	1.48E-09
SMPD3	63	17	-1.998541458	2.07E-08	2.61E-08
ZNF485	63	28	-1.278649377	4.85E-05	5.55E-05
AOC2	63	32	-1.086004299	0.000359779	0.000402682
CD160	63	34	-0.998541458	0.000866265	0.000961573
PPP1R32	63	39	-0.800602081	0.005715866	0.006175318
TMEM81	63	52	-0.385564581	0.152658117	0.157602755
C16orf71	63	53	-0.358083845	0.182004295	0.18735454
OVCH1	64	5	-3.786796281	4.07E-15	5.92E-15
AKR1C2	64	8	-3.108724376	4.15E-13	5.79E-13
KCND3	64	14	-2.301369454	6.68E-10	8.72E-10
CARNS1	64	18	-1.938799374	3.06E-08	3.82E-08
NEK2	64	25	-1.464868186	5.56E-06	6.54E-06
LOC283663	64	29	-1.250743381	5.69E-05	6.51E-05
FAM21A	64	32	-1.108724376	0.000255216	0.000286966
AQP11	64	35	-0.979441359	0.00095405	0.001056612
CCT6B	64	42	-0.716406953	0.01143245	0.012219546
C15orf61	64	44	-0.649292757	0.020391218	0.021644016
BLOC1S1	64	49	-0.494014532	0.069895462	0.072994138
PDSS1	64	49	-0.494014532	0.069895462	0.072994138

VWF	65	7	-3.323737267	5.21E-14	7.40E-14
GDPD3	65	22	-1.67166057	4.65E-07	5.64E-07
CHAD	65	24	-1.546129688	1.90E-06	2.26E-06
MT1X	65	109	0.637092136	0.00415897	0.004517652
FGF14	65	91	0.376702451	0.105282269	0.109168668
C1orf228	66	3	-4.568155995	4.40E-17	6.67E-17
CHRDL2	66	3	-4.568155995	4.40E-17	6.67E-17
PGBD5	66	14	-2.345763573	2.28E-10	3.01E-10
NPFF	66	20	-1.8311904	6.24E-08	7.74E-08
PRR5L	66	149	1.066050025	1.94E-07	2.38E-07
MGC12916	66	25	-1.509262305	2.40E-06	2.86E-06
MGC16275	66	27	-1.398230993	8.42E-06	9.86E-06
DNM1P41	66	33	-1.108724376	0.000204276	0.000230253
HOXC8	66	41	-0.795566491	0.004902838	0.005313109
BAIAP3	66	45	-0.661265399	0.016689306	0.017777491
SLC16A12	67	12	-2.589851066	1.36E-11	1.85E-11
CRABP2	67	165	1.191508648	2.09E-09	2.70E-09
ATF7IP2	67	17	-2.087350725	2.76E-09	3.54E-09
MMP3	67	159	1.138069389	1.44E-08	1.81E-08
SLC26A10	67	23	-1.65125161	3.92E-07	4.77E-07
HVCN1	67	30	-1.267922971	3.15E-05	3.63E-05
CD58	67	54	-0.419926064	0.110306404	0.114272049
ATP8B4	68	13	-2.495747499	2.52E-11	3.39E-11
CILP	68	17	-2.108724376	1.65E-09	2.14E-09
MYH3	68	19	-1.948259704	1.02E-08	1.30E-08
TAS2R20	68	21	-1.803869794	5.42E-08	6.74E-08
FAM227B	68	34	-1.108724376	0.000163564	0.000185004
ADAM8	68	36	-1.026262216	0.000401078	0.000448521
SMIM1	68	54	-0.441299715	0.092119831	0.095774699
SFXN2	68	69	-0.08766276	0.722043053	0.726695992
SLC44A5	69	12	-2.632286332	4.42E-12	6.04E-12
DNM1P46	69	13	-2.516809115	1.45E-11	1.96E-11

KCNMB4	69	19	-1.969321319	6.24E-09	7.96E-09
BACE1-AS	69	21	-1.82493141	3.38E-08	4.22E-08
FAM229A	69	21	-1.82493141	3.38E-08	4.22E-08
C17orf53	69	26	-1.516809115	1.30E-06	1.56E-06
ALOX12	69	29	-1.359267838	8.30E-06	9.72E-06
GP1BA	69	41	-0.859696828	0.002111347	0.002315011
CDON	69	42	-0.82493141	0.003001193	0.003274143
PPFIA4	69	113	0.60293013	0.005471372	0.005914444
ELMO3	69	44	-0.757817214	0.00581439	0.006278287
AKAP5	69	73	-0.027424274	0.909810576	0.911327705
ANKRD1	69	74	-0.007795467	0.974225379	0.974724662
TNFSF13B	70	14	-2.430652471	2.59E-11	3.48E-11
FOXO1	70	18	-2.068082391	1.53E-09	1.98E-09
MIPEPP3	70	24	-1.653044892	2.12E-07	2.59E-07
BRSK2	70	25	-1.594151203	4.30E-07	5.22E-07
C11orf82	70	26	-1.537567675	8.48E-07	1.02E-06
DNAH17	70	30	-1.331116797	9.96E-06	1.16E-05
C1orf51	70	58	-0.380026398	0.136924712	0.141528137
FOXA1	71	12	-2.673508995	1.42E-12	1.96E-12
OLFML1	71	14	-2.451116573	1.50E-11	2.03E-11
SAMD13	71	18	-2.088546494	9.20E-10	1.20E-09
FAM106CP	71	26	-1.558031777	5.52E-07	6.69E-07
SKA3	71	28	-1.451116573	2.03E-06	2.42E-06
DBIL5P	71	29	-1.4004905	3.74E-06	4.42E-06
NTN3	71	30	-1.3515809	6.73E-06	7.90E-06
TMEM92	71	40	-0.936543401	0.000801882	0.000890612
MFSD2A	71	98	0.356238349	0.110657493	0.114620555
CITED4	71	67	-0.192382305	0.433413172	0.439377315
UCP2	72	482	2.634239959	1.49E-69	4.36E-69
RRN3P2	72	16	-2.278649377	7.57E-11	1.01E-10
NUDT17	72	31	-1.324453067	8.06E-06	9.45E-06
ARHGAP27	72	37	-1.069196012	0.000165735	0.000187406

SHMT1	72	44	-0.819217759	0.002583138	0.002824381
LOC10013055	72	51	-0.606224035	0.02067919	0.021946697
LOC10028731	72	52	-0.578209659	0.02658163	0.028111585
EFNA3	72	72	-0.108724376	0.651028852	0.656239099
TRPC4	73	7	-3.491194013	4.00E-16	5.94E-16
PTGS2	73	23	-1.774986979	2.53E-08	3.17E-08
DNM3	73	34	-1.211086094	2.77E-05	3.21E-05
TGM1	73	34	-1.211086094	2.77E-05	3.21E-05
SEPT4	73	38	-1.050621421	0.00018518	0.000209001
RTN4RL2	73	40	-0.97662084	0.000431622	0.000482195
CPEB3	73	42	-0.906231512	0.000945235	0.001046999
C16orf95	73	53	-0.57062848	0.027343852	0.028909848
GCH1	74	23	-1.794615785	1.59E-08	2.00E-08
BLM	74	31	-1.363981431	3.69E-06	4.36E-06
TMEM53	74	40	-0.996249647	0.000314645	0.000352976
C15orf37	74	49	-0.703467897	0.007439654	0.008008859
PAN3-AS1	74	54	-0.563290239	0.028102225	0.02970361
NRXN3	74	103	0.368322786	0.091531392	0.095175584
PLA2G4A	75	6	-3.752580566	2.37E-17	3.61E-17
NLRC3	75	19	-2.089615553	3.08E-10	4.05E-10
PAPLN	75	19	-2.089615553	3.08E-10	4.05E-10
HHIPL1	75	28	-1.530188144	3.80E-07	4.62E-07
FLJ37453	75	33	-1.293148947	7.75E-06	9.08E-06
FAM211A	75	35	-1.208260049	2.23E-05	2.58E-05
AGAP7	75	36	-1.167618065	3.68E-05	4.23E-05
HID1	75	46	-0.81398111	0.002223127	0.002435522
INCA1	75	49	-0.722833222	0.005790413	0.006254992
LRRC29	75	50	-0.693686877	0.007777899	0.008368367
TAC3	75	71	-0.187795947	0.431539787	0.437534952
FLVCR2	76	15	-2.449761294	2.97E-12	4.07E-12
PKMYT1	76	18	-2.186726888	7.01E-11	9.33E-11
SCARF1	76	21	-1.964334467	1.15E-09	1.49E-09

SLC25A34	77	20	-2.053582822	2.84E-10	3.73E-10
LINC00857	77	34	-1.288048075	6.26E-06	7.36E-06
IFITM10	77	62	-0.421314606	0.085963115	0.089480934
HENMT1	78	17	-2.306663754	8.81E-12	1.20E-11
MMP25	78	17	-2.306663754	8.81E-12	1.20E-11
AMY2B	78	26	-1.693686877	2.51E-08	3.14E-08
NUF2	78	27	-1.639239093	5.19E-08	6.45E-08
GNRHR2	78	32	-1.394126595	1.36E-06	1.62E-06
RELT	78	59	-0.511483545	0.038871889	0.04088774
P2RX7	79	4	-4.412505124	7.40E-20	1.17E-19
LOC10013333	79	24	-1.827542623	3.36E-09	4.31E-09
CCDC15	79	39	-1.127102905	3.84E-05	4.41E-05
MKX	79	50	-0.768648934	0.002810496	0.003069104
PITPNA-AS1	79	65	-0.390137311	0.105390173	0.109266046
STXBP6	79	69	-0.303980667	0.200316611	0.20598794
TBC1D30	80	14	-2.623297549	1.00E-13	1.42E-13
GSG2	80	27	-1.675764969	2.13E-08	2.67E-08
ST20	80	39	-1.145250252	2.71E-05	3.13E-05
ACE	80	49	-0.815942627	0.001548006	0.001705711
FABP3	80	53	-0.702732016	0.005430345	0.005870908
PAQR6	81	16	-2.448574379	5.88E-13	8.17E-13
EME2	81	24	-1.863611878	1.30E-09	1.69E-09
C16orf59	81	35	-1.319291362	2.36E-06	2.81E-06
CAMK1G	81	37	-1.239121013	6.95E-06	8.15E-06
CDK5R1	81	38	-1.200646865	1.16E-05	1.35E-05
ZNF843	81	39	-1.16317216	1.91E-05	2.21E-05
TSC22D1-AS1	81	41	-1.091022374	4.89E-05	5.60E-05
C1orf145	81	52	-0.748134661	0.003126019	0.003409845
SRGAP2D	81	53	-0.720653924	0.004234976	0.004597015
ANG	81	92	0.074987577	0.73264881	0.737180074
ZNF503-AS2	82	29	-1.608295385	3.66E-08	4.56E-08
RGS4	82	32	-1.466276381	2.67E-07	3.26E-07

C11orf45	82	34	-1.378813539	9.00E-07	1.08E-06
LINC00643	82	38	-1.218348867	8.08E-06	9.46E-06
FLJ39051	82	56	-0.658921458	0.007825742	0.008417523
C17orf96	82	67	-0.40018719	0.091166401	0.094808687
LOC10050634	83	43	-1.057499053	6.13E-05	7.01E-05
MAP10	83	45	-0.991910711	0.000143541	0.000162568
NUDT14	83	57	-0.650873793	0.008128771	0.008733844
FMO4	84	12	-2.916079298	7.87E-16	1.16E-15
PSMC3IP	85	30	-1.611224716	1.98E-08	2.49E-08
SLC7A5P2	85	31	-1.563919002	3.93E-08	4.90E-08
GINS3	85	33	-1.473721193	1.45E-07	1.79E-07
MORN1	85	40	-1.196187217	7.52E-06	8.81E-06
GALNT18	85	119	0.376702451	0.063986202	0.066898949
C1orf226	86	47	-0.980400279	0.000126685	0.000143708
EXTL1	86	122	0.395748207	0.049552203	0.051995855
NOG	86	79	-0.231208382	0.30338487	0.309609573
HYDIN	87	15	-2.644777276	6.50E-15	9.42E-15
SLC16A6	87	26	-1.851228154	3.86E-10	5.06E-10
CDCA3	87	44	-1.092236253	2.53E-05	2.92E-05
ZNF487P	87	45	-1.059814775	3.95E-05	4.54E-05
NXPH3	88	15	-2.661265399	3.69E-15	5.38E-15
C1orf220	88	20	-2.2462279	9.79E-13	1.36E-12
RAD54L	88	24	-1.983193494	4.35E-11	5.81E-11
CDKN3	88	31	-1.613959684	1.07E-08	1.36E-08
JMJD4	88	75	-0.339337304	0.13371115	0.138243131
CHST11	88	77	-0.301369454	0.180041405	0.18540727
FDXACB1	89	36	-1.414532805	1.81E-07	2.22E-07
MRPL45P2	89	39	-1.299055588	1.01E-06	1.22E-06
CYB5R2	89	59	-0.701814757	0.0033984	0.003702819
LTK	89	71	-0.434710687	0.057390452	0.060115632
MST1P2	90	9	-3.430652471	2.87E-19	4.51E-19
SCN2B	90	14	-2.79322255	3.36E-16	5.01E-16

FMO3	90	24	-2.015614971	1.61E-11	2.18E-11
FANK1	90	37	-1.391124107	2.20E-07	2.69E-07
PITRM1-AS1	90	39	-1.315175253	6.93E-07	8.36E-07
METTL12	90	52	-0.900137754	0.000261872	0.000294323
LOC10050606	90	55	-0.819217759	0.000754094	0.00083873
TEX29	90	113	0.21960149	0.279580184	0.285689826
SNX29P2	91	13	-2.916079298	5.07E-17	7.67E-17
ORC1	91	20	-2.294590921	2.01E-13	2.82E-13
MYH11	91	29	-1.758538021	6.42E-10	8.38E-10
ALX3	91	34	-1.529056175	2.25E-08	2.82E-08
FNDC8	91	39	-1.331116797	4.72E-07	5.73E-07
MARCH10	92	20	-2.310358237	1.18E-13	1.67E-13
ANP32A-IT1	92	22	-2.172854713	8.95E-13	1.24E-12
GOLGA6L4	92	31	-1.678090022	1.82E-09	2.36E-09
RNF207	92	33	-1.587892213	7.51E-09	9.55E-09
FRMD6-AS1	92	74	-0.422832966	0.059677472	0.062477728
SNAI3-AS1	92	76	-0.384358819	0.084840101	0.088323737
SLC35G1	93	44	-1.188451568	3.15E-06	3.74E-06
SERINC4	93	46	-1.124321231	8.26E-06	9.68E-06
ISL2	93	48	-1.062920686	2.05E-05	2.37E-05
C1orf192	93	57	-0.814993173	0.00065048	0.000724106
RSC1A1	93	74	-0.438429821	0.050239728	0.052710202
MBL1P	94	8	-3.663313228	5.17E-21	8.35E-21
WNT3	94	35	-1.534030211	1.22E-08	1.55E-08
MIF4GD	94	67	-0.597224037	0.009091986	0.009751314
C1orf74	94	76	-0.415385714	0.061148976	0.063983968
C10orf107	95	11	-3.219148366	2.86E-19	4.49E-19
LOC730183	95	25	-2.034723794	3.30E-12	4.52E-12
BMP8A	95	26	-1.978140266	8.06E-12	1.10E-11
LOC10050623	95	48	-1.093617483	1.05E-05	1.23E-05
DHODH	95	56	-0.871225062	0.000264127	0.000296814
DHRS4	95	64	-0.678579984	0.003304044	0.003601016

GDPD1	95	80	-0.356651889	0.102526448	0.106410025
ST6GALNAC3	96	12	-3.108724376	6.69E-19	1.04E-18
GJA3	96	25	-2.049830687	2.00E-12	2.75E-12
NRG4	96	26	-1.993247158	4.92E-12	6.73E-12
UBXN11	96	33	-1.649292757	1.30E-09	1.69E-09
ASB16	96	36	-1.523761875	1.01E-08	1.29E-08
GJC2	96	43	-1.267422122	6.32E-07	7.64E-07
CRHR1-IT1	96	74	-0.484233511	0.029279915	0.030927479
PYY2	97	4	-4.708637218	1.23E-24	2.12E-24
SMG1P1	97	34	-1.621174377	1.71E-09	2.20E-09
RAB20	97	38	-1.460709705	2.40E-08	3.01E-08
LOC644961	97	40	-1.386709123	8.05E-08	9.95E-08
ESPL1	97	41	-1.351085213	1.44E-07	1.77E-07
HELB	97	51	-1.036211876	2.04E-05	2.36E-05
RNF112	97	64	-0.708637218	0.002041741	0.002240893
CCDC163P	97	67	-0.642548028	0.004674141	0.005068087
FAM117A	98	114	0.109455794	0.581124013	0.586456115
SKIDA1	99	25	-2.094224806	4.41E-13	6.15E-13
FAM64A	99	40	-1.416152901	3.63E-08	4.53E-08
ARHGAP5-AS	99	51	-1.065655654	1.06E-05	1.24E-05
PGBD4	99	62	-0.783884686	0.000669136	0.000744767
TSPAN15	99	84	-0.345763573	0.105465971	0.109330117
C11orf75	99	120	0.1688096	0.387529545	0.393833708
MAF	100	24	-2.167618065	1.02E-13	1.44E-13
LOC282997	100	30	-1.84568997	2.12E-11	2.86E-11
LOC728407	100	43	-1.326315811	1.39E-07	1.72E-07
RIMS3	100	48	-1.167618065	1.89E-06	2.25E-06
GRAMD1B	100	57	-0.919690551	8.92E-05	0.000101684
TMED8	101	23	-2.243373903	2.24E-14	3.20E-14
NBPF7	101	34	-1.679473017	2.91E-10	3.83E-10
MTRNR2L8	101	52	-1.066496141	8.53E-06	9.98E-06
ZNF214	101	64	-0.766935859	0.000747037	0.000831117

IGF1	101	70	-0.637652842	0.004145815	0.004504615
TLCD1	101	119	0.127881905	0.511495335	0.517593245
LOC387723	102	21	-2.388832295	1.61E-15	2.37E-15
MNS1	102	32	-1.781149718	4.09E-11	5.47E-11
CMTM1	102	33	-1.736755599	8.81E-11	1.17E-10
KIF18B	102	39	-1.495747499	5.74E-09	7.32E-09
LINC00260	102	45	-1.289296622	1.96E-07	2.40E-07
CEP128	102	50	-1.137293528	2.45E-06	2.91E-06
ENO3	102	50	-1.137293528	2.45E-06	2.91E-06
JMJD7	102	50	-1.137293528	2.45E-06	2.91E-06
KCNC4	102	58	-0.923168723	7.20E-05	8.22E-05
GAS6-AS1	102	98	-0.166439874	0.414511362	0.420542968
RRN3P1	103	23	-2.271662947	7.82E-15	1.13E-14
LOC338758	103	34	-1.707762062	1.18E-10	1.57E-10
CLUHP3	103	47	-1.240636051	3.90E-07	4.74E-07
ECHDC3	103	61	-0.864487566	0.000160852	0.000181989
PLD6	103	76	-0.54729739	0.011600421	0.012395688
ZNF215	103	141	0.344326449	0.063836462	0.06675133
KDM5B-AS1	103	128	0.204775097	0.281990842	0.288077771
CNIH2	103	104	-0.094785185	0.636358632	0.641699969
XAF1	104	20	-2.487235999	1.79E-16	2.67E-16
MCM10	105	22	-2.363538275	9.44E-16	1.39E-15
PTPRQ	105	29	-1.964988898	8.25E-13	1.14E-12
KLLN	105	32	-1.822969894	1.01E-11	1.37E-11
PROX2	105	34	-1.735507052	4.79E-11	6.39E-11
SPA17	105	47	-1.268381042	1.86E-07	2.29E-07
KIF14	105	51	-1.150544552	1.40E-06	1.67E-06
EID3	105	61	-0.892232556	9.09E-05	0.000103521
CEND1	105	70	-0.693686877	0.001637154	0.00180267
DSTNP2	105	93	-0.283811082	0.166576028	0.171676416
S100A4	105	128	0.177030106	0.350008755	0.356351808
OVGP1	106	58	-0.978663835	2.15E-05	2.49E-05

HYLS1	106	70	-0.707361814	0.00128681	0.001420311
REC8	107	46	-1.326629406	5.09E-08	6.33E-08
METTL20	107	50	-1.206335173	4.23E-07	5.13E-07
MLKL	107	86	-0.423926608	0.041774282	0.043916985
ZFHX2	107	90	-0.358338266	0.081777249	0.085191907
HAS3	107	105	-0.135945845	0.492562753	0.49882279
POLE2	108	47	-1.309023026	6.05E-08	7.51E-08
ZRANB2-AS1	108	58	-1.005630883	1.15E-05	1.34E-05
TSPAN2	108	120	0.043278718	0.820884187	0.823626444
IQCH-AS1	109	39	-1.591506482	2.96E-10	3.90E-10
NRIP2	109	40	-1.554980606	5.82E-10	7.60E-10
HSD17B7P2	109	45	-1.385055604	1.32E-08	1.67E-08
N4BP2L2-IT2	110	37	-1.680630724	4.65E-11	6.22E-11
ANXA9	110	43	-1.463819335	2.65E-09	3.40E-09
RAD9A	110	50	-1.2462279	1.42E-07	1.76E-07
LINC00338	110	54	-1.135196587	1.03E-06	1.23E-06
BCDIN3D	110	67	-0.823994899	0.000181604	0.000205113
KIAA1324	110	70	-0.760801072	0.000475318	0.000530706
FAM196A	111	5	-4.581212147	1.12E-27	2.03E-27
TSSK4	111	35	-1.773857225	6.58E-12	8.97E-12
LINC00467	111	53	-1.175219788	4.50E-07	5.47E-07
PRKCZ	111	78	-0.617738023	0.003482109	0.003790853
ADAP2	112	48	-1.331116797	2.29E-08	2.87E-08
CDC7	112	55	-1.134719584	8.27E-07	9.97E-07
MIR17HG	112	55	-1.134719584	8.27E-07	9.97E-07
RHBDL1	112	81	-0.576229295	0.00582777	0.006291864
LAT	113	29	-2.070922343	1.56E-14	2.24E-14
C15orf62	113	39	-1.643501119	5.18E-11	6.92E-11
FAM86C1	113	89	-0.453169907	0.026076445	0.02758106
DLEU1	113	133	0.126379097	0.492663607	0.498860232
RARRES3	114	4	-4.94161439	4.25E-29	7.83E-29
RGMA	114	11	-3.482182771	2.99E-24	5.12E-24

NGF	114	31	-1.98741808	5.77E-14	8.19E-14
RASSF7	114	255	1.052739047	1.59E-11	2.14E-11
GAS2L3	114	45	-1.449761294	1.79E-09	2.31E-09
RAD51	114	49	-1.326904546	1.86E-08	2.34E-08
RND2	114	54	-1.186726888	2.55E-07	3.12E-07
DUSP23	114	60	-1.034723794	3.95E-06	4.68E-06
LOC441666	115	31	-2.000018116	3.53E-14	5.03E-14
C11orf63	115	41	-1.596662422	8.75E-11	1.16E-10
C10orf25	115	43	-1.527949672	3.34E-10	4.38E-10
ACYP1	115	85	-0.544823491	0.007904106	0.008500642
LOC145783	116	38	-1.718777858	6.57E-12	8.96E-12
MYPN	116	65	-0.944337558	1.60E-05	1.86E-05
LOC284023	117	157	0.315531653	0.071691904	0.074855205
ZNF19	118	45	-1.499514329	3.47E-10	4.55E-10
EFCAB11	118	50	-1.347511235	7.02E-09	8.94E-09
ROM1	118	79	-0.687586677	0.000924294	0.001024821
PTGFRN	119	29	-2.145561144	7.45E-16	1.10E-15
LINC00638	119	42	-1.611224716	3.09E-11	4.14E-11
DENND2C	119	68	-0.916079298	2.04E-05	2.37E-05
LOC10050564	119	70	-0.874259122	4.22E-05	4.84E-05
SNHG10	119	72	-0.833617138	8.41E-05	9.59E-05
C17orf67	119	75	-0.774723449	0.000222477	0.000250443
OTUB2	119	95	-0.433686531	0.028336206	0.029938768
CUBN	120	28	-2.208260049	1.68E-16	2.51E-16
RFFL	120	45	-1.523761875	1.51E-10	2.00E-10
MAGEL2	120	70	-0.886331955	3.18E-05	3.66E-05
SUZ12P1	120	73	-0.825790413	8.96E-05	0.000102105
SIGIRR	120	92	-0.492053015	0.013399293	0.014308093
KIAA1804	121	29	-2.169606618	2.67E-16	3.98E-16
AKR1E2	121	37	-1.818134248	3.06E-13	4.28E-13
PLXNA2	121	41	-1.670035609	6.33E-12	8.64E-12
CREB3L4	121	62	-1.073391303	9.68E-07	1.17E-06

TESK2	121	71	-0.877840494	3.42E-05	3.93E-05
NOMO3	121	117	-0.157222894	0.400425396	0.406621836
SMAGP	121	142	0.122159506	0.492874732	0.499009307
KRT80	122	270	1.037353884	7.28E-12	9.91E-12
DENND2D	122	44	-1.580030095	3.33E-11	4.47E-11
KIAA1024	122	202	0.618749769	0.000144055	0.000163127
SENP8	122	80	-0.717533619	0.000470879	0.000525825
THEMIS2	122	99	-0.410105093	0.035035523	0.036922051
TRIM17	123	48	-1.466276381	2.94E-10	3.87E-10
RHBDF2	123	132	-0.006844762	0.969780979	0.970526677
RGS9	124	393	1.555464816	9.18E-30	1.71E-29
ZNF286B	124	53	-1.335000232	3.78E-09	4.84E-09
PRSS53	124	79	-0.759139938	0.000213093	0.000239983
LOC728431	124	85	-0.65352975	0.00116754	0.001289578
RAPGEF3	124	162	0.276929317	0.105991345	0.109845576
EML5	125	34	-1.987045819	3.65E-15	5.33E-15
FAM86A	125	84	-0.682191238	0.00070955	0.000789636
IDI2	125	85	-0.665117724	0.000929952	0.001030801
LOC729970	125	169	0.326370776	0.053706314	0.056301866
FMO2	126	4	-5.086004299	3.25E-32	6.28E-32
FMO5	126	8	-4.086004299	1.50E-29	2.79E-29
GBAP1	126	47	-1.531415448	4.49E-11	6.01E-11
ARHGAP44	126	74	-0.876550934	2.41E-05	2.79E-05
RAB3IP	126	91	-0.578209659	0.003350317	0.003650939
LRRC37A2	127	52	-1.396969345	6.64E-10	8.67E-10
SCARNA9	127	66	-1.053014943	7.81E-07	9.42E-07
FAM86FP	127	81	-0.75755906	0.000183498	0.000207162
NS3BP	127	87	-0.654465567	0.001002212	0.001108851
LOC10050602	127	111	-0.302993196	0.10550045	0.109351343
FAM46B	127	154	0.169377478	0.326053586	0.332482691
KCNAB3	127	144	0.072515939	0.679264241	0.684435414
IFI44	128	47	-1.554135524	1.93E-11	2.61E-11

ARMC4	128	51	-1.436299034	2.45E-10	3.23E-10
GGACT	128	59	-1.226081327	2.09E-08	2.63E-08
C12orf66	128	65	-1.086356563	3.64E-07	4.42E-07
PSTK	128	68	-1.021261535	1.32E-06	1.58E-06
ZNF597	128	74	-0.89927101	1.36E-05	1.59E-05
BORA	128	108	-0.353836874	0.059949117	0.062753707
PIWIL4	129	37	-1.910498266	6.92E-15	1.00E-14
HEXIM2	129	55	-1.338591918	1.71E-09	2.20E-09
LOC729013	130	50	-1.487235999	5.85E-11	7.80E-11
RGS11	130	55	-1.349732475	1.16E-09	1.51E-09
RSG1	130	78	-0.84568997	3.17E-05	3.65E-05
SCG5	130	202	0.527119294	0.001001787	0.001108539
SCN4B	130	107	-0.389625203	0.038018998	0.040022966
CAMKK1	130	119	-0.236274426	0.196364519	0.20197715
RAB38	131	32	-2.142147377	2.88E-17	4.39E-17
SLITRK5	131	40	-1.820219283	3.12E-14	4.46E-14
ANKRD20A12	131	43	-1.715882623	3.13E-13	4.39E-13
INHBE	132	498	1.806883437	1.12E-45	2.58E-45
BTBD8	132	46	-1.629556539	1.75E-12	2.42E-12
C1orf53	132	50	-1.509262305	2.56E-11	3.44E-11
ARSG	132	61	-1.222381158	1.37E-08	1.73E-08
RHOD	132	76	-0.905190982	8.92E-06	1.04E-05
GSG1	133	20	-2.842078717	1.41E-23	2.39E-23
C1orf56	133	62	-1.209810501	1.58E-08	1.99E-08
LDB3	134	548	1.923218517	2.44E-54	6.16E-54
N4BP2L1	134	23	-2.65125161	3.47E-22	5.73E-22
BRI3BP	134	53	-1.446893112	7.39E-11	9.83E-11
LOC619207	134	58	-1.316832571	1.34E-09	1.74E-09
PGBD3	134	63	-1.197533643	1.82E-08	2.29E-08
CPEB1	135	44	-1.726108354	1.09E-13	1.54E-13
COMTD1	135	46	-1.661978017	4.70E-13	6.55E-13
PARPBP	135	64	-1.185539973	2.09E-08	2.63E-08

PPCDC	135	66	-1.141145854	5.45E-08	6.77E-08
CYP2R1	135	83	-0.810500542	4.34E-05	4.98E-05
MIR210HG	136	313	1.09383163	1.32E-14	1.91E-14
GCHFR	136	47	-1.641598365	6.16E-13	8.56E-13
C11orf71	136	53	-1.468266763	3.28E-11	4.40E-11
STPG1	136	66	-1.151793098	3.87E-08	4.82E-08
IQCC	136	70	-1.0669042	2.38E-07	2.91E-07
USP32P2	136	78	-0.910784998	5.84E-06	6.87E-06
C14orf142	136	83	-0.821147786	3.33E-05	3.83E-05
BOLA1	136	84	-0.803869794	4.61E-05	5.28E-05
ADAM12	137	319	1.110656155	3.45E-15	5.03E-15
PAQR4	137	50	-1.562900269	3.14E-12	4.30E-12
CDCA8	137	73	-1.0169319	6.16E-07	7.45E-07
GEMIN2	137	116	-0.348775464	0.054854875	0.057482785
FUOM	137	135	-0.129940862	0.457495443	0.463550268
ATP1A2	138	15	-3.310358237	6.80E-28	1.23E-27
FRRS1	138	58	-1.359267838	2.91E-10	3.83E-10
OSCP1	138	120	-0.310358237	0.084309036	0.087794275
HCN3	139	37	-2.018212083	5.30E-17	8.02E-17
LINC00346	139	63	-1.250385525	3.02E-09	3.87E-09
KIF2C	139	69	-1.119140992	5.63E-08	6.99E-08
TSPAN18	139	88	-0.76823383	7.46E-05	8.51E-05
WRAP53	139	109	-0.459481124	0.012438707	0.013287807
KCNJ2-AS1	140	6	-4.653044892	1.13E-34	2.25E-34
MRM1	140	78	-0.952605174	1.80E-06	2.15E-06
FAM124A	141	10	-3.926347633	4.06E-32	7.83E-32
RTL1	141	44	-1.78884411	7.02E-15	1.02E-14
LOC283143	141	67	-1.182186538	1.10E-08	1.39E-08
RBPMS2	141	76	-1.000348215	6.03E-07	7.29E-07
FBXL8	141	79	-0.94449498	1.96E-06	2.34E-06
SLC41A2	141	106	-0.520355274	0.004787987	0.005190087
LOC10012828	142	44	-1.799039877	4.42E-15	6.44E-15

SCAMP5	143	37	-2.059142347	7.26E-18	1.12E-17
MEG8	143	49	-1.653885869	1.18E-13	1.67E-13
LOC81691	143	55	-1.487235999	6.55E-12	8.94E-12
SHC4	143	281	0.865830608	1.85E-09	2.39E-09
CTNND1	143	76	-1.020668199	3.23E-07	3.94E-07
C10orf114	143	83	-0.893556281	4.81E-06	5.68E-06
SH2D5	143	207	0.424891245	0.006284267	0.006779091
ABCC6	144	14	-3.471294455	3.97E-30	7.44E-30
RAD51AP1	144	44	-1.819217759	1.75E-15	2.57E-15
SLC6A15	144	92	-0.755087421	7.13E-05	8.14E-05
SPRYD4	144	109	-0.510465053	0.005087713	0.005507339
HSPB7	144	113	-0.458470415	0.011121637	0.011888961
LRRC56	145	47	-1.734044614	1.12E-14	1.61E-14
RAPGEFL1	146	76	-1.050621421	1.24E-07	1.53E-07
CCDC81	146	168	0.093768488	0.565048544	0.570601847
PRELP	147	13	-3.607957003	1.33E-31	2.55E-31
LIN9	148	56	-1.510822819	1.57E-12	2.17E-12
SPON1	148	71	-1.168430622	6.62E-09	8.44E-09
UBE2T	148	89	-0.842444311	9.64E-06	1.13E-05
TOM1L1	148	92	-0.794615785	2.57E-05	2.97E-05
TMEM121	148	121	-0.399314504	0.023520738	0.024928534
GPR135	149	61	-1.397155559	2.25E-11	3.03E-11
C16orf55	149	66	-1.283498777	3.69E-10	4.84E-10
SNHG9	149	100	-0.684036707	0.000211008	0.00023767
MED11	149	102	-0.655467554	0.000359277	0.000402236
LOC439994	150	49	-1.722833222	5.23E-15	7.60E-15
CCDC23	150	70	-1.208260049	2.01E-09	2.59E-09
DNAH14	151	100	-0.703272925	0.000132168	0.000149797
LPPR5	152	3	-5.771689389	4.46E-39	9.42E-39
LOC643529	152	40	-2.034723794	1.26E-18	1.96E-18
IGDCC3	152	48	-1.771689389	9.88E-16	1.46E-15
SLC25A45	152	59	-1.47400884	2.02E-12	2.78E-12

SLC44A3	152	91	-0.848857249	6.37E-06	7.49E-06
ZBTB3	152	91	-0.848857249	6.37E-06	7.49E-06
EXO1	153	29	-2.508131223	1.05E-23	1.78E-23
RBP5	153	51	-1.693686877	5.96E-15	8.64E-15
AMDHD2	153	64	-1.366112219	2.68E-11	3.60E-11
C17orf97	153	92	-0.842550263	6.81E-06	7.99E-06
C17orf100	153	106	-0.638191764	0.000413751	0.000462494
CACNB2	154	25	-2.731654727	5.07E-26	8.93E-26
ABTB2	154	57	-1.542620902	2.46E-13	3.45E-13
KDM8	154	83	-1.000471485	1.83E-07	2.25E-07
TUBD1	154	122	-0.444773579	0.010675531	0.011418329
CHRNB1	154	137	-0.277478834	0.101073768	0.104916264
STK33	155	46	-1.861286825	5.03E-17	7.63E-17
CHTF18	155	50	-1.740992591	1.15E-15	1.70E-15
LOC440028	155	61	-1.454111444	2.06E-12	2.83E-12
SFR1	155	74	-1.175395416	2.46E-09	3.17E-09
C1orf213	155	122	-0.454111444	0.009028635	0.009684699
ARID3B	155	126	-0.407568858	0.018159874	0.019328125
DHRS11	155	128	-0.384848781	0.025126511	0.026583515
DAPK2	156	13	-3.693686877	5.51E-34	1.09E-33
MSS51	156	58	-1.5361456	2.05E-13	2.88E-13
ATPBD4	156	71	-1.244379475	3.87E-10	5.07E-10
DDX28	156	102	-0.721701253	7.06E-05	8.06E-05
ERCC5	156	103	-0.707626068	9.37E-05	0.00010669
MGAT5B	157	55	-1.621985411	1.77E-14	2.54E-14
FBXL14	157	82	-1.04579312	4.71E-08	5.86E-08
ZNHIT2	157	116	-0.54536413	0.0018945	0.002080753
SLC45A3	157	122	-0.472607787	0.006409954	0.006913719
SLC46A1	157	153	-0.145957282	0.372970709	0.379235451
RAD51B	158	65	-1.390137311	6.82E-12	9.29E-12
OAS3	159	19	-3.173679818	8.66E-31	1.64E-30
C12orf45	159	80	-1.099679236	1.03E-08	1.30E-08

ASPHD1	159	283	0.723050912	2.43E-07	2.96E-07
C14orf79	159	90	-0.929754235	6.17E-07	7.46E-07
EPHX4	159	96	-0.83664483	5.13E-06	6.04E-06
SFMBT2	160	49	-1.815942627	5.38E-17	8.14E-17
CCDC40	160	65	-1.408284658	3.10E-12	4.26E-12
EPSTI1	160	80	-1.108724376	7.37E-09	9.38E-09
EMC9	160	112	-0.623297549	0.000406437	0.000454448
SLC7A6OS	160	120	-0.523761875	0.002503555	0.002738516
NEIL1	161	38	-2.191713741	2.02E-21	3.29E-21
ABCB9	161	93	-0.900482443	1.04E-06	1.25E-06
CYB561	161	125	-0.473856969	0.005653236	0.006109344
NUDT13	162	46	-1.925012423	1.80E-18	2.79E-18
NEK8	162	51	-1.776149037	1.02E-16	1.53E-16
CCNB2	162	79	-1.144793631	2.37E-09	3.05E-09
TRIM46	162	154	-0.181787838	0.262634423	0.268690188
ETV4	163	445	1.340208996	4.10E-27	7.35E-27
LOC729683	163	45	-1.965599434	4.71E-19	7.37E-19
TMEM86A	163	64	-1.45745253	5.13E-13	7.13E-13
C16orf91	163	114	-0.624562516	0.000349796	0.000391959
ELL3	163	150	-0.22863384	0.16098403	0.165978914
ZNF670	164	80	-1.144348286	1.91E-09	2.47E-09
CPM	164	89	-0.99054295	9.39E-08	1.16E-07
HSD17B7	164	102	-0.793851039	9.57E-06	1.12E-05
DNAH10	164	116	-0.608295385	0.000460522	0.000514333
TMEM240	165	62	-1.52085028	6.42E-14	9.10E-14
TOE1	165	70	-1.345763573	7.90E-12	1.07E-11
MAP2K6	165	90	-0.983193494	1.03E-07	1.27E-07
STK32C	165	92	-0.951484634	2.24E-07	2.74E-07
AK4	165	237	0.413696659	0.004339352	0.004708351
RILP	166	55	-1.702404094	3.37E-16	5.02E-16
QRICH2	166	62	-1.529567497	4.23E-14	6.02E-14
DLGAP5	166	68	-1.396300966	1.71E-12	2.35E-12

PNP	166	227	0.34278468	0.019198766	0.020414374
FUCA1	167	127	-0.503743982	0.002876679	0.00314006
LPXN	167	140	-0.363145651	0.027686423	0.029268076
ZFP3	168	65	-1.478673986	1.25E-13	1.77E-13
FRAT2	168	101	-0.842830316	2.40E-06	2.86E-06
FOXC2	168	259	0.515766489	0.000259851	0.000292136
PRIM1	169	30	-2.602713217	4.86E-27	8.71E-27
TNFRSF14	170	39	-2.232713093	5.18E-23	8.64E-23
CDCA5	170	73	-1.328290753	6.25E-12	8.53E-12
FBXL19-AS1	170	80	-1.196187217	2.40E-10	3.16E-10
TMEM170A	170	93	-0.978956501	7.36E-08	9.11E-08
CNTN5	171	15	-3.619686295	1.44E-36	2.95E-36
LOC10050757	171	86	-1.100312136	2.83E-09	3.64E-09
CDYL2	171	92	-1.003014935	3.64E-08	4.54E-08
AKAP6	171	285	0.628241218	4.72E-06	5.58E-06
ZNF684	172	87	-1.092045635	3.18E-09	4.08E-09
SPIRE2	173	46	-2.019790647	8.48E-21	1.37E-20
THAP10	173	65	-1.52098479	1.60E-14	2.30E-14
CYB5RL	173	77	-1.276566063	1.77E-11	2.38E-11
LOC399744	174	63	-1.574387948	2.82E-15	4.13E-15
NR4A1	174	82	-1.194115867	1.57E-10	2.07E-10
ZBTB42	174	119	-0.656850108	0.000111873	0.00012709
ZNF232	174	144	-0.38174287	0.01852962	0.01971897
MOK	174	213	0.183041748	0.213047572	0.218618724
WNK4	175	183	-0.044235649	0.771707196	0.775880198
SDSL	176	54	-1.813268492	1.66E-18	2.58E-18
COL9A2	176	61	-1.637418657	3.03E-16	4.51E-16
LOC10050684	176	83	-1.193116563	1.27E-10	1.68E-10
FBXL15	176	145	-0.388246905	0.016128503	0.017187156
SGK494	177	86	-1.150065171	3.77E-10	4.94E-10
ZNF511	177	87	-1.13338643	6.00E-10	7.84E-10
HARBI1	177	104	-0.875890208	5.67E-07	6.86E-07

SNRPN	177	168	-0.184012503	0.2361078	0.241932267
YPEL4	178	60	-1.677567211	6.19E-17	9.34E-17
FRAT1	178	65	-1.562089994	1.97E-15	2.89E-15
TIPIN	178	76	-1.336530293	1.58E-12	2.18E-12
TMEM251	178	110	-0.803098093	3.19E-06	3.79E-06
CLEC2B	178	219	0.190329253	0.189770256	0.195323044
DNA2	179	65	-1.57017234	1.29E-15	1.90E-15
CEP55	179	70	-1.463257136	3.19E-14	4.54E-14
AGAP5	179	73	-1.402715594	1.93E-13	2.72E-13
PLK1	179	107	-0.851073167	9.15E-07	1.10E-06
PIGL	179	115	-0.747050102	1.15E-05	1.34E-05
MGC57346	179	139	-0.47359908	0.003541177	0.003854084
GPR56	180	49	-1.985867628	3.88E-21	6.28E-21
ZBTB37	180	61	-1.669840135	5.25E-17	7.95E-17
LOC10050666	180	104	-0.900137754	2.44E-07	2.98E-07
RPS6KA5	181	10	-4.286642168	8.98E-43	1.99E-42
CIB2	181	61	-1.677832925	3.38E-17	5.13E-17
LRRC8B	181	67	-1.542481073	2.08E-15	3.05E-15
CORO6	181	84	-1.21625284	3.55E-11	4.75E-11
ORC6	181	91	-1.100775623	9.70E-10	1.26E-09
C12orf76	181	107	-0.867103277	5.29E-07	6.42E-07
GINS2	182	49	-2.001809172	1.46E-21	2.38E-21
LOC10050631	182	94	-1.061930164	2.61E-09	3.35E-09
PSD	182	98	-1.001809172	1.38E-08	1.74E-08
SNAPC5	182	113	-0.796340054	2.93E-06	3.48E-06
POLR3K	182	114	-0.783629002	4.00E-06	4.73E-06
PIF1	183	79	-1.320643466	1.25E-12	1.72E-12
SLC18A2	183	131	-0.591001213	0.000311354	0.000349435
ZNF365	183	178	-0.148690783	0.327372965	0.333697361
KBTBD4	184	98	-1.017576488	7.40E-09	9.42E-09
KBTBD3	184	102	-0.95986099	3.62E-08	4.52E-08
IGF2	185	281	0.494320484	0.000252511	0.000283966

MMP17	185	141	-0.500554484	0.001814651	0.001993895
ACSL5	186	6	-5.062920686	1.10E-46	2.57E-46
MANEAL	186	47	-2.093294335	3.26E-23	5.47E-23
FAM22D	186	48	-2.062920686	8.21E-23	1.36E-22
CCDC113	186	81	-1.308033184	1.20E-12	1.66E-12
LOC10050673	186	96	-1.062920686	1.70E-09	2.19E-09
CD163L1	186	328	0.709668818	4.54E-08	5.65E-08
C17orf59	186	120	-0.740992591	8.97E-06	1.05E-05
TMEM256	186	195	-0.040552873	0.783781487	0.787715526
IFI27L1	186	196	-0.033173343	0.822174312	0.824814902
LHX4	187	35	-2.526335819	7.90E-29	1.45E-28
GPR89A	187	74	-1.44616547	1.47E-14	2.11E-14
CRADD	187	111	-0.861202969	4.01E-07	4.88E-07
ZC3H12A	187	196	-0.040908992	0.781373116	0.785497254
SYPL2	188	13	-3.962873509	1.72E-42	3.80E-42
CXCL16	188	32	-2.663313228	1.31E-30	2.48E-30
PAOX	188	114	-0.830423213	8.37E-07	1.01E-06
CACNA1G	189	36	-2.501041799	8.26E-29	1.52E-28
ZNF124	189	77	-1.404180259	3.97E-14	5.66E-14
LOC283922	189	79	-1.367186052	1.26E-13	1.78E-13
DDI2	189	104	-0.970527082	1.76E-08	2.21E-08
ENKD1	189	113	-0.850787838	4.59E-07	5.57E-07
PDIA3P	189	195	-0.063636486	0.665514761	0.670754359
MAP3K8	190	60	-1.771689389	2.81E-19	4.42E-19
TMEM133	190	69	-1.570055527	1.76E-16	2.64E-16
ANKRD35	190	77	-1.411793444	2.68E-14	3.83E-14
EFCAB2	190	84	-1.286262561	1.36E-12	1.87E-12
FAM66C	190	112	-0.871225062	2.48E-07	3.03E-07
TMEM107	190	128	-0.678579984	3.25E-05	3.75E-05
C11orf80	190	150	-0.449761294	0.004167031	0.004525778
MUTYH	191	72	-1.516228202	8.25E-16	1.22E-15
SOCS2	191	149	-0.466984683	0.002940555	0.003208887

ZFP69B	191	150	-0.457334513	0.003530712	0.003843229
TMEM223	192	144	-0.523761875	0.000928185	0.001028988
LOC10013108	193	64	-1.701181413	1.45E-18	2.26E-18
SHISA9	193	68	-1.613718572	2.48E-17	3.79E-17
LOC653160	193	78	-1.415779194	1.48E-14	2.13E-14
COQ10A	193	137	-0.60314933	0.00016257	0.000183907
FANCA	194	50	-2.064781028	9.48E-24	1.61E-23
THNSL1	194	77	-1.441850677	5.47E-15	7.94E-15
ANKRD9	194	140	-0.579354201	0.00026569	0.000298528
PIGW	194	149	-0.489468698	0.001752606	0.001926805
KRTAP1-5	194	178	-0.232903787	0.119546966	0.123697132
DPF3	194	192	-0.123674717	0.399558466	0.40579426
BUB1B	195	73	-1.526230131	2.99E-16	4.45E-16
MMACHC	196	86	-1.297169465	4.26E-13	5.95E-13
ME3	196	94	-1.168845368	2.44E-11	3.29E-11
TFAP4	197	40	-2.4088481	9.19E-29	1.69E-28
N6AMT2	197	74	-1.52132283	2.48E-16	3.70E-16
CAMK1D	197	93	-1.191617384	1.07E-11	1.44E-11
SHCBP1	197	102	-1.058350853	6.48E-10	8.47E-10
KIF17	198	130	-0.715713183	8.80E-06	1.03E-05
SLC45A1	198	130	-0.715713183	8.80E-06	1.03E-05
ADORA2B	198	235	0.138435951	0.318815615	0.32518693
CENPV	198	202	-0.079869513	0.57971644	0.585186877
TMEM183A	199	97	-1.145436154	3.60E-11	4.82E-11
DHRS12	199	111	-0.95093313	1.32E-08	1.66E-08
PARS2	200	112	-0.945225644	1.43E-08	1.80E-08
TEX30	200	165	-0.386258351	0.010693963	0.011436478
WWOX	202	75	-1.538117168	5.92E-17	8.94E-17
ALG10	202	87	-1.323992363	7.76E-14	1.10E-13
CCDC34	202	104	-1.066496141	3.07E-10	4.03E-10
CEP85	202	120	-0.860045263	1.43E-07	1.76E-07
METTL18	202	127	-0.778251172	1.37E-06	1.64E-06

C11orf83	202	189	-0.204693434	0.16064917	0.165655535
WDR78	203	43	-2.347795538	9.30E-29	1.71E-28
SASS6	203	108	-1.019172791	1.20E-09	1.55E-09
ACP6	203	155	-0.497935888	0.001147576	0.001267707
PROSER2	203	173	-0.339432065	0.02271049	0.024073057
WNT2B	204	64	-1.781149718	1.01E-20	1.62E-20
LINC00842	204	134	-0.715060527	6.54E-06	7.69E-06
RCE1	205	146	-0.598379917	0.00011387	0.000129321
CCNF	205	155	-0.51208007	0.000801809	0.000890658
ZFP69	206	83	-1.420185472	1.69E-15	2.48E-15
PGBD2	206	96	-1.210262402	1.97E-12	2.71E-12
DEPDC7	206	392	0.819484941	1.26E-11	1.71E-11
FBXL16	206	200	-0.151368713	0.290342276	0.296493113
CAPN3	207	23	-3.278649377	1.13E-40	2.44E-40
SLC1A2	207	60	-1.895320738	1.02E-22	1.69E-22
LHPP	207	128	-0.802211333	5.22E-07	6.32E-07
CTH	208	362	0.690681793	2.03E-08	2.55E-08
NR1D1	208	168	-0.416846671	0.005199848	0.005625604
C16orf74	209	51	-2.143658166	1.27E-26	2.26E-26
FAM22A	209	56	-2.008728586	1.26E-24	2.17E-24
TAF1A	209	98	-1.201373664	1.83E-12	2.52E-12
TIMM10	209	114	-0.983193494	2.11E-09	2.72E-09
SPSB2	209	118	-0.933440459	9.73E-09	1.23E-08
ARG2	209	146	-0.626258949	4.94E-05	5.66E-05
LOC10050645	210	68	-1.735507052	1.38E-20	2.21E-20
ARL17A	210	87	-1.380026398	3.64E-15	5.32E-15
TICRR	210	87	-1.380026398	3.64E-15	5.32E-15
PPP1R3E	210	89	-1.347236463	1.13E-14	1.63E-14
CCDC77	211	107	-1.088356578	6.10E-11	8.14E-11
VWCE	212	45	-2.344791734	6.46E-30	1.21E-29
ASB16-AS1	212	77	-1.56985829	3.31E-18	5.12E-18
C1orf112	212	85	-1.427253894	5.15E-16	7.64E-16

ZNF239	212	100	-1.192788641	1.70E-12	2.35E-12
PPP1R13B	212	111	-1.042228964	2.47E-10	3.25E-10
DEPDC1	213	101	-1.185222513	1.95E-12	2.69E-12
LINC00339	213	112	-1.036079074	2.73E-10	3.60E-10
RFXAP	213	137	-0.745401913	1.78E-06	2.13E-06
HAGHL	213	151	-0.605029257	7.11E-05	8.12E-05
LYSMD2	214	131	-0.816768361	2.20E-07	2.69E-07
PMF1	214	131	-0.816768361	2.20E-07	2.69E-07
TMEM186	214	156	-0.564789143	0.000181725	0.000205219
CACNA1H	214	284	0.299555757	0.021105023	0.022395587
NME1	214	266	0.205091073	0.120366269	0.124528378
CDC20	215	79	-1.553136477	3.52E-18	5.44E-18
DHX58	215	85	-1.447526289	1.56E-16	2.34E-16
LINS	215	110	-1.075557512	6.16E-11	8.21E-11
LOC283070	216	72	-1.693686877	1.80E-20	2.88E-20
NUDT7	216	88	-1.404180259	6.27E-16	9.28E-16
LOC10086267	216	116	-1.005630883	5.52E-10	7.22E-10
MUC1	216	132	-0.819217759	1.79E-07	2.21E-07
ATG16L2	217	63	-1.892995685	1.06E-23	1.79E-23
KRBA2	217	97	-1.270362766	6.26E-14	8.89E-14
XRRA1	217	156	-0.584873389	0.000100336	0.000114084
ZNF593	217	169	-0.469396172	0.001448143	0.00159635
HDDC3	217	185	-0.338894148	0.018672345	0.019862736
ATAD5	218	84	-1.484591278	2.51E-17	3.82E-17
EP400NL	218	104	-1.176468983	1.46E-12	2.02E-12
L2HGDH	218	124	-0.92271239	6.59E-09	8.40E-09
DAB1	220	91	-1.382289449	7.52E-16	1.11E-15
DNAJC17	220	106	-1.162163635	1.90E-12	2.62E-12
B9D1	220	188	-0.335495238	0.018984226	0.020191749
ATAD3B	221	129	-0.88539968	1.69E-08	2.13E-08
CDKN2C	221	136	-0.809164094	1.75E-07	2.16E-07
LOC10028806	222	49	-2.288430398	2.12E-30	4.00E-30

GAS8	222	116	-1.045159247	8.49E-11	1.13E-10
COX10-AS1	222	117	-1.032775523	1.29E-10	1.71E-10
CEP152	222	120	-0.996249647	4.36E-10	5.71E-10
SLC16A5	223	73	-1.719799717	1.66E-21	2.70E-21
TNNT2	223	415	0.78734325	1.66E-11	2.24E-11
FAM195A	223	270	0.167191321	0.199107446	0.204771506
C1QL1	224	45	-2.424226202	1.05E-32	2.04E-32
CCDC101	225	98	-1.307795723	5.55E-15	8.06E-15
CCDC18	226	40	-2.606975243	1.18E-35	2.38E-35
SLC43A3	226	52	-2.22846362	5.49E-30	1.03E-29
FGGY	226	97	-1.328990496	2.21E-15	3.24E-15
ARRB2	226	128	-0.928903338	2.85E-09	3.67E-09
AMN1	226	129	-0.917676083	4.12E-09	5.27E-09
MECR	226	182	-0.421108698	0.003277906	0.003573526
FAM83G	226	231	-0.077154297	0.567445551	0.57294832
AKR1C1	227	46	-2.411710907	6.24E-33	1.21E-32
TGFB3	227	57	-2.102382849	4.09E-28	7.44E-28
PPP4R4	227	111	-1.140856997	1.82E-12	2.51E-12
KCNJ8	227	115	-1.089782812	1.11E-11	1.50E-11
MAP6	227	117	-1.064908144	2.64E-11	3.54E-11
PWWP2B	227	154	-0.668486322	7.40E-06	8.68E-06
FICD	227	180	-0.443419767	0.001994472	0.002189321
HERC2P3	228	101	-1.283402907	9.07E-15	1.31E-14
NBR2	228	108	-1.186726888	3.13E-13	4.38E-13
LRRC23	228	152	-0.693686877	3.47E-06	4.11E-06
MPP3	228	162	-0.601764387	4.31E-05	4.94E-05
TMCC2	228	164	-0.584062385	6.83E-05	7.80E-05
TTC12	229	84	-1.555610741	2.65E-19	4.16E-19
MASP2	229	89	-1.472194733	6.43E-18	9.90E-18
KLRAP1	230	71	-1.804467307	1.50E-23	2.54E-23
CDT1	230	81	-1.614364424	2.30E-20	3.68E-20
TSEN54	230	110	-1.172854713	4.09E-13	5.71E-13

LRRC37B	230	117	-1.083849707	1.00E-11	1.36E-11
TMEM234	231	144	-0.790548416	1.65E-07	2.03E-07
CCDC176	231	216	-0.205585915	0.131931846	0.13645772
BIRC5	232	130	-0.944337558	1.05E-09	1.36E-09
ACCS	233	64	-1.972910521	1.14E-26	2.03E-26
TTYH2	233	79	-1.669129772	1.54E-21	2.51E-21
TSSK3	233	106	-1.244990066	1.97E-14	2.83E-14
SH3D21	233	200	-0.329054331	0.017766332	0.018914422
AAMDC	234	131	-0.945666094	8.51E-10	1.11E-09
POLG2	234	134	-0.912999905	2.57E-09	3.31E-09
METTL1	234	190	-0.409233487	0.003572821	0.003887982
PSRC1	235	118	-1.102598273	3.03E-12	4.16E-12
NEU3	235	134	-0.919152132	1.93E-09	2.49E-09
DIO2	235	167	-0.60153703	3.31E-05	3.81E-05
LOC400027	235	178	-0.509507891	0.000353393	0.000395932
LOC730102	236	80	-1.66943933	8.41E-22	1.38E-21
DNAJC22	236	103	-1.304866898	1.36E-15	2.01E-15
TAF5	236	136	-0.903904584	2.99E-09	3.84E-09
EFNA4	236	139	-0.872426353	8.57E-09	1.09E-08
RCOR2	236	178	-0.515633994	0.00029524	0.000331492
ACADS	237	101	-1.339256142	3.14E-16	4.68E-16
LINC00565	237	380	0.572387984	1.16E-06	1.40E-06
TRPT1	237	284	0.152279495	0.229126255	0.234901789
CCDC28B	237	259	0.019340663	0.881346451	0.883269306
UBE2Q2P1	238	72	-1.833617138	8.14E-25	1.41E-24
LOC613037	238	79	-1.699761391	1.69E-22	2.80E-22
CUTC	238	183	-0.487842301	0.000549393	0.000612274
POP5	238	196	-0.388832295	0.005081775	0.005501675
LOC731275	239	59	-2.126948135	6.31E-30	1.18E-29
LTB4R	239	106	-1.281670729	2.23E-15	3.26E-15
LRTOMT	239	127	-1.020906497	3.92E-11	5.25E-11
SPATA2L	239	159	-0.696708229	1.85E-06	2.21E-06

TRPV1	240	76	-1.767687458	7.33E-24	1.25E-23
GLUD1P3	240	111	-1.221199105	2.02E-14	2.89E-14
IRF7	240	153	-0.758227129	2.66E-07	3.24E-07
PER1	240	285	0.139203138	0.269690547	0.275800614
BEST1	242	91	-1.519792973	1.11E-19	1.76E-19
THOC6	242	129	-1.016360358	3.50E-11	4.69E-11
RPUSD2	242	200	-0.383731423	0.005260177	0.005689296
PIGH	243	102	-1.361111538	5.59E-17	8.46E-17
PEX11A	243	120	-1.126646284	5.25E-13	7.31E-13
SAC3D1	243	160	-0.711608785	9.61E-07	1.16E-06
DUS2L	243	224	-0.226181957	0.090283459	0.093915483
HERC2P7	244	101	-1.381250231	2.15E-17	3.29E-17
ADCK1	244	142	-0.889714594	2.65E-09	3.41E-09
ASB13	244	172	-0.613196959	1.68E-05	1.95E-05
MPP2	244	192	-0.454499213	0.001043663	0.001154386
MVK	244	236	-0.156818664	0.233622495	0.239417068
CCDC144A	245	98	-1.430652471	2.51E-18	3.89E-18
C17orf58	245	126	-1.068082391	3.88E-12	5.30E-12
ZBTB25	245	144	-0.875437313	4.04E-09	5.17E-09
ZNF778	245	168	-0.653044892	5.10E-06	6.01E-06
TMEM9B-AS1	246	77	-1.784452341	1.02E-24	1.76E-24
ZEB1-AS1	246	114	-1.218348867	1.07E-14	1.55E-14
PARP16	246	181	-0.551392994	8.59E-05	9.79E-05
RHOJ	246	264	-0.006844762	0.957273919	0.958255613
ZFAND4	247	89	-1.581358177	3.69E-21	5.99E-21
NALCN	247	506	0.925901967	1.39E-17	2.13E-17
SNHG12	247	161	-0.726174729	4.96E-07	6.01E-07
CISD3	248	143	-0.903049349	1.23E-09	1.60E-09
PAR-SN	250	94	-1.519919809	2.79E-20	4.45E-20
TRIM58	250	108	-1.319621158	1.10E-16	1.65E-16
BMS1P4	250	114	-1.241618646	2.58E-15	3.77E-15
EXO5	250	147	-0.874836316	2.87E-09	3.68E-09

CDADC1	251	117	-1.20990321	8.12E-15	1.17E-14
IL18BP	251	159	-0.767384975	1.05E-07	1.30E-07
C12orf73	252	190	-0.516148691	0.000181458	0.000204977
COMMD3	253	141	-0.952166598	1.40E-10	1.85E-10
ZNF691	253	158	-0.787937202	4.69E-08	5.84E-08
NOX4	253	402	0.559333741	9.71E-07	1.17E-06
CMC2	253	215	-0.343525101	0.010103575	0.010811019
PTHLH	254	43	-2.671144308	7.04E-41	1.52E-40
TRIM16L	254	96	-1.512446562	1.92E-20	3.08E-20
GOLGA6L5	255	88	-1.643646194	5.99E-23	9.99E-23
KIF18A	256	84	-1.716406953	2.18E-24	3.73E-24
STAT5A	256	105	-1.394478858	2.05E-18	3.18E-18
SLC1A7	256	159	-0.795841421	2.98E-08	3.73E-08
C14orf28	256	258	-0.09749712	0.443471034	0.44939856
PDIK1L	257	154	-0.847562384	4.59E-09	5.86E-09
NFASC	258	0	-9.119951631	2.56E-51	6.29E-51
ATXN7L2	258	80	-1.798023536	4.25E-26	7.49E-26
TMEM220	258	86	-1.693686877	3.86E-24	6.59E-24
SAMD11	258	90	-1.628098535	6.59E-23	1.10E-22
FBXO6	258	137	-1.021919548	6.41E-12	8.75E-12
TOMM40L	258	179	-0.636135854	4.82E-06	5.69E-06
TMEM106A	258	283	0.024706612	0.842162554	0.844650319
TMEM116	259	93	-1.586373852	3.33E-22	5.50E-22
TRDMT1	259	97	-1.525619821	4.62E-21	7.48E-21
PTGR2	259	123	-1.183018158	9.24E-15	1.34E-14
LOC728190	259	160	-0.803604569	1.89E-08	2.38E-08
APOLD1	260	97	-1.531179347	3.06E-21	4.96E-21
MC1R	260	135	-1.054276592	1.49E-12	2.06E-12
SMCR7	260	190	-0.561236581	4.09E-05	4.69E-05
AMIGO1	261	77	-1.869843832	9.69E-28	1.75E-27
ZNF205	261	157	-0.842009624	4.26E-09	5.45E-09
RFC3	262	73	-1.952322819	2.14E-29	3.96E-29

WASH7P	262	104	-1.441707659	1.07E-19	1.69E-19
MMP15	262	208	-0.441707659	0.000936383	0.001037783
RNASEH2C	262	216	-0.387259875	0.003405025	0.00370952
MACROD1	262	222	-0.347731511	0.008103222	0.008709388
ZNF771	262	222	-0.347731511	0.008103222	0.008709388
BRCA2	263	49	-2.532933521	5.73E-40	1.22E-39
BEND7	263	131	-1.114220364	9.85E-14	1.39E-13
CASC5	264	67	-2.087029305	3.86E-32	7.45E-32
SETD6	264	113	-1.332939533	8.77E-18	1.35E-17
TK1	264	121	-1.234255258	5.95E-16	8.81E-16
ZNF669	264	137	-1.055086412	9.75E-13	1.35E-12
ALDH1A3	266	83	-1.78896738	1.11E-26	1.97E-26
IQGAP3	266	102	-1.491581469	6.23E-21	1.01E-20
LOC642361	266	114	-1.331116797	7.15E-18	1.10E-17
TNFRSF25	266	120	-1.257116216	1.74E-16	2.61E-16
TMEM79	266	154	-0.897220271	3.89E-10	5.10E-10
SOX13	267	95	-1.599564699	4.27E-23	7.13E-23
RMI2	267	99	-1.540063687	6.08E-22	1.00E-21
GABPB2	267	114	-1.336530293	4.90E-18	7.56E-18
PTER	267	116	-1.311439312	1.46E-17	2.24E-17
WDR63	267	116	-1.311439312	1.46E-17	2.24E-17
GPR162	267	129	-1.158193052	1.02E-14	1.48E-14
ADAL	267	154	-0.902633767	2.94E-10	3.87E-10
CACNB1	267	241	-0.256530971	0.045148321	0.047432159
BZRAP1	268	77	-1.908027026	3.54E-29	6.53E-29
METTL25	268	111	-1.3803977	6.19E-19	9.67E-19
SLC4A8	269	106	-1.452266284	2.19E-20	3.49E-20
MARC1	270	42	-2.79322255	2.39E-45	5.47E-45
CENPL	270	167	-0.80183568	1.01E-08	1.28E-08
PELI3	270	212	-0.457619518	0.000519416	0.000579363
SSTR1	270	272	-0.098077132	0.428580191	0.434590665
PFKFB2	271	107	-1.449406431	1.80E-20	2.88E-20

FAM76A	271	111	-1.396457551	1.93E-19	3.04E-19
GALK2	271	282	-0.051322065	0.675665313	0.680896945
GPR133	272	50	-2.552331027	1.29E-41	2.81E-41
HELLS	272	68	-2.108724376	1.77E-33	3.47E-33
PYCARD	272	132	-1.151793098	7.62E-15	1.10E-14
SRR	272	139	-1.077246144	1.77E-13	2.49E-13
SFT2D2	272	145	-1.016278127	2.19E-12	3.01E-12
FAM173A	272	216	-0.441299715	0.000755354	0.000839892
ROGDI	272	220	-0.414827504	0.001466962	0.001616867
TMEM99	273	143	-1.04161018	7.04E-13	9.77E-13
PRPF40B	273	162	-0.861631514	8.99E-10	1.17E-09
NME7	273	217	-0.439930284	0.000764967	0.000850459
WDR89	273	264	-0.157087397	0.206957774	0.212565205
ZWINT	274	66	-2.162362339	9.67E-35	1.93E-34
CBLN3	274	68	-2.119293618	6.47E-34	1.28E-33
HHLA3	274	123	-1.264241954	4.52E-17	6.84E-17
SPAG5	274	130	-1.184388646	1.49E-15	2.19E-15
DFFB	274	148	-0.997303093	3.95E-12	5.40E-12
WHAMMP2	275	112	-1.404657262	7.25E-20	1.15E-19
PTGFR	275	122	-1.281274847	1.86E-17	2.84E-17
EFNB3	275	194	-0.612099342	5.09E-06	6.00E-06
ADAM1A	276	91	-1.709454192	5.00E-26	8.80E-26
NUBP1	276	135	-1.140433236	7.93E-15	1.15E-14
ALKBH2	276	155	-0.941124427	3.22E-11	4.31E-11
KCNK2	276	157	-0.922628084	6.72E-11	8.95E-11
PANK1	277	169	-0.821587106	3.12E-09	4.00E-09
KCNAB2	278	143	-1.067794112	1.44E-13	2.03E-13
WHAMMP3	278	145	-1.047756359	3.36E-13	4.70E-13
MTRF1	278	146	-1.03784089	5.10E-13	7.09E-13
GNB3	278	154	-0.960878908	1.23E-11	1.66E-11
PLAC9	278	224	-0.420310527	0.001132129	0.001251174
FAM111B	279	47	-2.678256836	7.47E-45	1.70E-44

AP4B1	279	197	-0.610793868	4.53E-06	5.35E-06
CCDC84	280	127	-1.249322706	4.04E-17	6.13E-17
SLC37A4	280	251	-0.266463839	0.033401181	0.035209209
C11orf1	281	160	-0.921222601	4.80E-11	6.40E-11
CDC14A	282	91	-1.740481088	3.41E-27	6.13E-27
CDC6	282	106	-1.520355274	1.11E-22	1.84E-22
NANOS1	282	131	-1.214852727	1.48E-16	2.22E-16
KLHL11	282	163	-0.899547574	1.06E-10	1.41E-10
SYNGR3	282	447	0.555855293	2.82E-07	3.44E-07
CCDC3	283	54	-2.498495117	3.03E-42	6.67E-42
VRK1	283	150	-1.024563928	5.57E-13	7.74E-13
CBX2	284	140	-1.129188478	5.37E-15	7.80E-15
ATPAF2	284	209	-0.551112363	2.47E-05	2.86E-05
RAP1GAP2	284	355	0.213203719	0.062500327	0.065371692
MYO1D	286	55	-2.487235999	1.83E-42	4.03E-42
CIT	286	104	-1.568155995	5.68E-24	9.67E-24
CDK11A	286	129	-1.257368457	1.29E-17	1.99E-17
RAB3IL1	286	144	-1.098670711	1.67E-14	2.40E-14
ZNF438	286	173	-0.833967485	1.07E-09	1.39E-09
FAM216A	286	200	-0.624739523	2.17E-06	2.58E-06
HIVEP3	286	413	0.421402259	0.000127933	0.000145081
FANCM	287	97	-1.67371846	2.97E-26	5.25E-26
ACD	289	197	-0.661598239	5.42E-07	6.57E-07
SLC25A33	289	259	-0.266841771	0.030459076	0.032159941
ATP5S	290	106	-1.560713011	3.96E-24	6.77E-24
ZNF821	290	119	-1.393815703	1.23E-20	1.97E-20
ELP4	290	227	-0.462084979	0.000284443	0.000319416
STX3	290	249	-0.328631534	0.008257578	0.008868578
C14orf93	291	113	-1.473420756	2.28E-22	3.78E-22
GSTZ1	292	127	-1.309864248	5.00E-19	7.82E-19
LOC10012805	293	37	-3.094027865	4.77E-54	1.20E-53
PLEKHG4	294	85	-1.899005785	1.18E-31	2.25E-31

GAN	294	180	-0.816543624	1.26E-09	1.64E-09
GPSM2	295	185	-0.78191406	4.64E-09	5.92E-09
ARMC7	295	188	-0.758706668	1.14E-08	1.44E-08
C1orf159	296	117	-1.447813022	3.53E-22	5.82E-22
BTBD19	296	182	-0.810383101	1.42E-09	1.84E-09
SLC29A3	297	148	-1.113590131	2.66E-15	3.90E-15
C10orf2	297	206	-0.63654297	9.18E-07	1.11E-06
NTN1	298	32	-3.327892896	1.73E-58	4.55E-58
FOXM1	298	100	-1.684036707	2.03E-27	3.65E-27
SLC15A3	298	105	-1.613647379	6.79E-26	1.19E-25
E2F7	299	224	-0.525371128	3.41E-05	3.93E-05
DAGLA	301	126	-1.365064129	9.62E-21	1.55E-20
TMEM62	301	152	-1.094416539	4.23E-15	6.16E-15
GOLGA8T	302	121	-1.428265878	3.59E-22	5.92E-22
ORMDL2	303	397	0.281096838	0.010265769	0.01098156
GLRX2	303	275	-0.248610551	0.038358217	0.040363741
LOC10049948	304	103	-1.670151362	1.24E-27	2.25E-27
TEFM	304	178	-0.880918458	4.47E-11	5.98E-11
SPATA5L1	304	186	-0.817493078	6.38E-10	8.33E-10
LOC645212	304	198	-0.727295269	2.33E-08	2.92E-08
NXPH4	304	418	0.350707243	0.001168009	0.001289914
MYO15B	305	47	-2.806800957	3.04E-51	7.46E-51
ERN1	305	151	-1.122985069	7.26E-16	1.07E-15
ALG14	305	164	-1.003837804	1.83E-13	2.58E-13
TRIM47	305	166	-0.986350377	4.03E-13	5.63E-13
LIN52	305	188	-0.806800957	9.28E-10	1.21E-09
CLSPN	306	91	-1.858317578	5.57E-32	1.07E-31
GJD3	306	95	-1.79625661	1.32E-30	2.49E-30
BDNF-AS	306	105	-1.651866701	2.14E-27	3.86E-27
HES4	306	134	-1.300023028	1.19E-19	1.88E-19
SAAL1	306	167	-0.982407926	4.42E-13	6.16E-13
DHRS4L2	306	208	-0.6656725	2.17E-07	2.66E-07

OSGIN1	307	91	-1.863024581	3.49E-32	6.73E-32
KLHDC1	307	150	-1.142000531	2.36E-16	3.52E-16
ZNF276	307	159	-1.057936266	1.26E-14	1.81E-14
ZNF26	309	159	-1.067304449	6.65E-15	9.63E-15
ZNF747	309	176	-0.920755785	5.42E-12	7.40E-12
DECR2	309	203	-0.714851487	2.91E-08	3.64E-08
MAPKAPK5-A	309	244	-0.449450066	0.000260585	0.000292918
PKNOX2	310	139	-1.265907708	3.80E-19	5.96E-19
ITGB3BP	310	162	-1.044998778	1.72E-14	2.47E-14
ZNF174	310	179	-0.901033004	1.20E-11	1.62E-11
INADL	310	192	-0.79988628	9.02E-10	1.18E-09
C1orf131	311	163	-1.040766992	1.90E-14	2.73E-14
AVIL	312	58	-2.5361456	3.74E-47	8.76E-47
TRIM45	313	150	-1.169924532	3.07E-17	4.68E-17
YEATS4	315	136	-1.320469553	1.23E-20	1.97E-20
SLC9A3R1	315	603	0.828081798	2.33E-17	3.55E-17
TM7SF2	315	302	-0.169527655	0.14434984	0.149143675
CLEC2D	316	193	-0.820048087	2.64E-10	3.48E-10
ZNF688	317	122	-1.486326068	1.62E-24	2.79E-24
TAPBPL	317	159	-1.104180451	4.98E-16	7.39E-16
CASP9	317	175	-0.965852294	3.65E-13	5.11E-13
PEX10	317	205	-0.737583307	7.74E-09	9.83E-09
STIL	318	129	-1.410380076	7.47E-23	1.24E-22
PUS3	318	177	-0.954001781	5.82E-13	8.09E-13
RNMTL1	319	175	-0.974925878	2.02E-13	2.84E-13
TCEA3	319	261	-0.398230993	0.000910757	0.001009955
RFC5	320	173	-0.996024243	6.84E-14	9.69E-14
ADPRM	320	179	-0.946836694	6.87E-13	9.54E-13
PEX16	320	215	-0.682459621	5.98E-08	7.43E-08
ARHGEF19	320	268	-0.36456328	0.002221675	0.002434272
YRDC	320	308	-0.16386593	0.154567828	0.15953214
LOC10027183	321	77	-2.168367322	1.54E-40	3.30E-40

SLC7A7	321	102	-1.762728521	3.04E-31	5.80E-31
LRRC49	321	132	-1.390759744	1.33E-22	2.20E-22
AP4S1	321	159	-1.122270908	1.33E-16	2.00E-16
SERGEF	321	211	-0.714054674	1.62E-08	2.05E-08
SLC19A2	321	257	-0.429529314	0.000358079	0.00040101
PLEKHM1P	322	110	-1.65828154	6.83E-29	1.26E-28
TMEM216	322	163	-1.0909131	5.68E-16	8.42E-16
PQLC2	322	242	-0.520778017	1.99E-05	2.31E-05
ZSWIM7	323	188	-0.889525879	7.57E-12	1.03E-11
KLHL25	324	174	-1.005630883	3.02E-14	4.31E-14
ITGA8	325	37	-3.243566918	2.85E-62	7.76E-62
KCTD13	325	181	-0.953174397	3.38E-13	4.73E-13
DCPS	325	194	-0.853107442	3.40E-11	4.55E-11
ADCK3	326	83	-2.082413099	3.51E-39	7.44E-39
MARC2	326	113	-1.637273568	9.86E-29	1.81E-28
TMEM200B	326	120	-1.550561935	1.11E-26	1.97E-26
KIAA0101	326	139	-1.338511457	1.02E-21	1.67E-21
CCDC41	326	141	-1.317901178	3.04E-21	4.93E-21
FAM161B	326	152	-1.209525017	8.91E-19	1.39E-18
PGAP2	326	162	-1.117602527	9.83E-17	1.48E-16
FLVCR1	326	186	-0.918293719	1.60E-12	2.21E-12
DBNDD1	326	378	0.104789894	0.335726437	0.34198901
SCUBE2	327	66	-2.417477082	8.39E-47	1.96E-46
FES	327	127	-1.473186515	6.27E-25	1.09E-24
TPT1-AS1	327	210	-0.747625684	2.96E-09	3.79E-09
ADSSL1	327	216	-0.706983699	1.59E-08	2.01E-08
HEATR3	327	237	-0.573127952	2.75E-06	3.27E-06
PLEKHG3	328	95	-1.896420772	4.65E-35	9.30E-35
OGFOD2	328	192	-0.88131388	7.63E-12	1.04E-11
PPIH	328	238	-0.571458617	2.82E-06	3.35E-06
JMJD8	328	276	-0.357751924	0.002345384	0.002567658
SEC16B	329	287	-0.305761223	0.008589657	0.009217622

CDK1	330	148	-1.265593225	2.84E-20	4.53E-20
MRPS31P5	330	170	-1.065655654	8.89E-16	1.31E-15
BANP	330	191	-0.897617762	3.09E-12	4.24E-12
TRIT1	330	212	-0.747126136	2.56E-09	3.29E-09
RNF157	331	171	-1.061559268	9.89E-16	1.46E-15
STX1B	331	225	-0.665630591	6.99E-08	8.66E-08
GDPD5	331	254	-0.490727096	4.18E-05	4.80E-05
GALNT16	332	94	-1.929174956	3.01E-36	6.12E-36
CD27-AS1	332	188	-0.929174956	5.98E-13	8.31E-13
RPP38	332	210	-0.76951829	8.74E-10	1.14E-09
LRRN4CL	332	316	-0.179983059	0.112240164	0.116229073
PDPN	332	358	5.197E-05	0.9996225	0.9996225
ITGA7	334	417	0.211474905	0.045168609	0.04744709
METTL21D	335	218	-0.728557336	4.30E-09	5.50E-09
FMNL1	336	141	-1.361490446	6.96E-23	1.16E-22
SELPLG	336	188	-0.946452947	1.88E-13	2.65E-13
LCMT2	337	166	-1.130289726	1.55E-17	2.38E-17
PEX12	337	173	-1.070700929	3.42E-16	5.09E-16
PLA2G16	337	199	-0.868704537	7.17E-12	9.76E-12
NTHL1	338	198	-0.880247192	3.87E-12	5.30E-12
TRIM6	339	98	-1.899155995	3.03E-36	6.17E-36
LYSMD4	339	142	-1.36411872	3.88E-23	6.50E-23
IFI35	339	148	-1.304412473	1.05E-21	1.72E-21
XKR8	339	288	-0.343940838	0.002873854	0.003137414
DTL	340	76	-2.270187799	3.18E-45	7.29E-45
DISP1	340	143	-1.358243975	4.65E-23	7.77E-23
RWDD3	340	171	-1.100262797	5.43E-17	8.22E-17
NPHP4	340	173	-1.083487084	1.30E-16	1.96E-16
LIN7A	340	181	-1.018269425	3.73E-15	5.44E-15
ANO3	341	14	-4.714997383	2.71E-82	8.88E-82
TVP23C	341	173	-1.087724077	9.44E-17	1.42E-16
MKS1	341	188	-0.967763453	4.34E-14	6.18E-14

TYSND1	341	198	-0.892995685	1.69E-12	2.33E-12
ACBD4	341	209	-0.814993173	6.62E-11	8.82E-11
FAM103A1	341	234	-0.651987585	7.75E-08	9.59E-08
MMP1	342	6288	4.091808611	0	0
GALNT6	342	809	1.133419002	3.31E-37	6.81E-37
ZC3H10	342	168	-1.134259468	7.29E-18	1.12E-17
C14orf169	342	182	-1.018782251	3.02E-15	4.42E-15
ZFPM1	342	201	-0.8755252	3.63E-12	4.97E-12
CPPED1	342	258	-0.515349635	1.33E-05	1.55E-05
ADC	342	432	0.228310611	0.028186016	0.029784113
TIRAP	343	172	-1.104524387	3.17E-17	4.82E-17
INA	343	483	0.385090237	0.000139375	0.00015792
GALE	343	313	-0.240770295	0.032607409	0.034395685
ABCA5	344	58	-2.677008135	7.33E-55	1.86E-54
DBF4B	344	91	-2.02719449	6.64E-40	1.42E-39
GOLGA8S	344	144	-1.365064129	1.77E-23	2.99E-23
PPOX	344	184	-1.011427175	3.67E-15	5.35E-15
EXPH5	345	159	-1.226293972	3.52E-20	5.62E-20
LYSMD1	345	195	-0.931846614	1.86E-13	2.62E-13
CDCA4	345	197	-0.917125108	3.86E-13	5.38E-13
GPATCH3	345	210	-0.82493141	3.23E-11	4.33E-11
ZNF385A	345	218	-0.770992603	3.85E-10	5.04E-10
LPAR6	346	126	-1.56607268	1.32E-28	2.42E-28
C2CD2L	346	199	-0.906727983	5.95E-13	8.27E-13
IL15RA	346	274	-0.445320521	0.000127461	0.000144567
EFCAB13	347	138	-1.438991772	1.68E-25	2.93E-25
SHPK	347	210	-0.833270711	1.91E-11	2.58E-11
CBX8	347	214	-0.806049242	6.84E-11	9.11E-11
CDK11B	347	231	-0.695767187	9.46E-09	1.20E-08
GLIS1	349	90	-2.063954506	2.28E-41	4.97E-41
VAMP1	349	252	-0.578527679	1.04E-06	1.24E-06
ORAI1	350	197	-0.937883668	9.22E-14	1.31E-13

ZNF720	350	201	-0.908883797	3.94E-13	5.50E-13
CGRRF1	350	207	-0.86644853	3.17E-12	4.35E-12
CTU2	350	297	-0.345616367	0.00234413	0.002566645
FLI1	351	243	-0.639239093	8.50E-08	1.05E-07
ZNF200	352	198	-0.938799374	7.49E-14	1.06E-13
POLA2	353	237	-0.6835055	1.20E-08	1.52E-08
SLC46A3	353	237	-0.6835055	1.20E-08	1.52E-08
SERPINA3	353	330	-0.205926535	0.062136706	0.065008781
FZD8	353	341	-0.15862082	0.147437499	0.152313723
DPCD	354	277	-0.46258776	5.98E-05	6.84E-05
DNHD1	355	189	-1.018157166	9.40E-16	1.39E-15
USP54	356	153	-1.327069964	2.78E-23	4.67E-23
THSD1	356	196	-0.969747963	1.09E-14	1.58E-14
MRPL46	356	208	-0.884018089	8.68E-13	1.20E-12
ZNF408	356	292	-0.394633248	0.000512143	0.000571332
HAPLN3	357	632	0.715276108	2.11E-14	3.03E-14
CYBA	357	205	-0.90902454	2.29E-13	3.21E-13
DKK1	357	457	0.247545715	0.014714447	0.01570171
OR51E2	358	97	-1.992627311	1.41E-40	3.03E-40
RAD51D	358	162	-1.25269015	1.55E-21	2.53E-21
HOXB-AS3	358	219	-0.817753094	1.96E-11	2.64E-11
HSD17B1	358	288	-0.422615152	0.000204766	0.000230772
P4HA3	358	416	0.107899565	0.298674112	0.304881849
LOC338799	359	152	-1.348636896	5.17E-24	8.81E-24
LOC10028877	359	186	-1.057405599	7.81E-17	1.18E-16
HSF4	360	133	-1.545295037	3.74E-29	6.90E-29
GPHN	360	148	-1.391124107	3.66E-25	6.38E-25
NUSAP1	360	168	-1.208260049	1.54E-20	2.47E-20
IL17D	360	210	-0.886331955	5.76E-13	8.01E-13
PXMP2	361	185	-1.073197942	2.70E-17	4.11E-17
TBCE	361	300	-0.375760712	0.000831067	0.000922764
RRNAD1	362	192	-1.023607762	3.67E-16	5.45E-16

PUS1	362	203	-0.943234346	2.65E-14	3.79E-14
TRPV4	362	219	-0.833783203	6.90E-12	9.40E-12
PPIEL	363	99	-1.983193494	7.10E-41	1.53E-40
ZNF267	363	192	-1.027587613	2.69E-16	4.02E-16
KAZALD1	363	283	-0.467891871	4.01E-05	4.60E-05
ALDH6A1	363	296	-0.403096748	0.00034583	0.00038757
DNASE1	364	133	-1.561236581	7.03E-30	1.31E-29
PHLDA2	364	532	0.438763419	6.40E-06	7.52E-06
ZNF764	365	198	-0.99112041	1.62E-15	2.37E-15
COA6	366	299	-0.40042254	0.000355786	0.000398555
CROCCP2	367	193	-1.035903591	1.17E-16	1.76E-16
PDE3A	368	7	-5.82493141	4.97E-92	1.79E-91
TACC2	368	240	-0.725395736	8.71E-10	1.14E-09
GRIK4	369	94	-2.08161253	4.25E-44	9.58E-44
STARD5	369	99	-2.006844762	3.82E-42	8.39E-42
PVRL1	369	115	-1.790711331	2.07E-36	4.22E-36
LRR1	369	191	-1.058772554	2.71E-17	4.13E-17
FUK	369	192	-1.051238881	4.13E-17	6.26E-17
HERC2P9	370	143	-1.4802345	3.52E-28	6.42E-28
NOC4L	370	231	-0.788356795	3.87E-11	5.18E-11
TAF6L	370	244	-0.709368499	1.65E-09	2.13E-09
PHKG2	370	285	-0.485287727	1.80E-05	2.09E-05
EFCAB7	371	136	-1.556536911	2.70E-30	5.07E-30
HOMEZ	371	229	-0.804795964	1.62E-11	2.19E-11
SMYD3	371	401	0.003458674	0.973431433	0.974055107
LRRC37A3	372	124	-1.693686877	4.49E-34	8.88E-34
C12orf52	372	271	-0.565734146	7.71E-07	9.30E-07
KIF26B	373	378	-0.089513772	0.395063769	0.401333807
FAIM2	374	90	-2.163765739	7.99E-47	1.86E-46
FOXRED1	374	246	-0.71310433	1.13E-09	1.47E-09
PCTP	374	253	-0.672625261	7.29E-09	9.28E-09
C1orf50	374	256	-0.655618836	1.56E-08	1.97E-08

GPR137C	375	63	-2.682191238	9.22E-60	2.46E-59
BRCA1	375	110	-1.878111448	2.42E-39	5.13E-39
SLC27A3	375	175	-1.208260049	2.50E-21	4.06E-21
SELRC1	375	239	-0.758604353	1.23E-10	1.63E-10
PRTG	376	81	-2.323463225	3.48E-51	8.53E-51
MTMR9LP	376	258	-0.652085972	1.68E-08	2.11E-08
C1orf35	377	235	-0.790628143	2.26E-11	3.05E-11
MTERFD3	377	238	-0.772327326	5.61E-11	7.48E-11
KLHDC4	377	267	-0.606449157	1.18E-07	1.46E-07
CLPB	377	291	-0.482269746	1.68E-05	1.95E-05
GMEB1	378	178	-1.195233369	3.79E-21	6.14E-21
DDX59	378	221	-0.883064241	1.83E-13	2.57E-13
WRAP73	378	250	-0.705182515	1.34E-09	1.74E-09
STARD10	378	434	0.090584432	0.371398081	0.37778398
KIF22	379	167	-1.291074122	1.01E-23	1.72E-23
PYGO1	381	161	-1.351454685	1.84E-25	3.22E-25
ZDHHC1	382	201	-1.035101513	2.96E-17	4.50E-17
RAD52	383	139	-1.570983885	1.27E-31	2.43E-31
TUBG2	383	191	-1.11249613	2.95E-19	4.63E-19
C10orf12	383	207	-0.996438	2.44E-16	3.65E-16
DPY19L2	384	64	-2.693686877	2.01E-61	5.44E-61
HHEX	384	172	-1.267422122	2.23E-23	3.75E-23
C15orf57	384	237	-0.804943628	7.13E-12	9.72E-12
LINC00672	384	316	-0.389906128	0.000359621	0.000402563
ARHGAP19	385	226	-0.877260049	1.50E-13	2.11E-13
IFIT2	386	89	-2.225447982	6.44E-50	1.56E-49
ANKRD16	386	278	-0.58224034	2.38E-07	2.91E-07
CDKL1	387	26	-4.004474414	4.35E-86	1.48E-85
OLFML2B	387	58	-2.846933137	1.90E-65	5.36E-65
LOC646471	388	136	-1.621174377	2.00E-33	3.92E-33
TADA1	388	263	-0.669718229	4.39E-09	5.61E-09
SEMA7A	389	967	1.205021359	1.75E-48	4.17E-48

LOC374443	389	275	-0.609062912	6.64E-08	8.23E-08
GPN3	389	289	-0.537425038	1.40E-06	1.68E-06
LRRC28	389	339	-0.307209258	0.004099691	0.00445512
DGKH	390	142	-1.56630757	4.89E-32	9.41E-32
DET1	390	170	-1.306663754	8.64E-25	1.49E-24
LOC388692	390	187	-1.16916023	4.49E-21	7.28E-21
P2RX4	391	259	-0.702940886	7.83E-10	1.02E-09
ANKRD42	391	264	-0.675355054	2.93E-09	3.76E-09
MAP3K14	391	292	-0.529924614	1.79E-06	2.13E-06
IQCK	392	226	-0.903255258	2.16E-14	3.10E-14
HINFP	392	240	-0.816543624	2.35E-12	3.24E-12
SLC25A15	392	255	-0.729080783	2.07E-10	2.73E-10
HS3ST3A1	392	300	-0.49461553	7.09E-06	8.32E-06
SHF	393	179	-1.243294101	3.15E-23	5.28E-23
ZSCAN20	393	228	-0.894219864	3.31E-14	4.72E-14
SLC35F2	393	492	0.215404627	0.026780894	0.028318484
FAM89A	394	188	-1.176187344	1.84E-21	3.00E-21
ZBTB48	394	219	-0.955989136	9.47E-16	1.40E-15
SFXN4	394	253	-0.747782621	7.28E-11	9.69E-11
TMEM206	394	305	-0.478110763	1.27E-05	1.48E-05
SLC9A5	395	205	-1.054953119	2.65E-18	4.11E-18
ALKBH1	395	271	-0.652284178	7.25E-09	9.23E-09
AFG3L1P	396	161	-1.407164118	5.73E-28	1.04E-27
MMAB	396	373	-0.195049176	0.060814703	0.063651255
SLC22A18	396	453	0.085286244	0.389463183	0.39569581
SEMA6C	397	113	-1.921540611	8.67E-43	1.92E-42
RNF166	397	257	-0.736095024	1.12E-10	1.48E-10
C17orf89	397	280	-0.612436556	4.18E-08	5.21E-08
GRASP	397	298	-0.522551053	2.02E-06	2.41E-06
RBM15	398	203	-1.080013079	4.35E-19	6.81E-19
DLL4	398	697	0.699665849	3.46E-15	5.06E-15
KCNT2	399	84	-2.356651889	4.26E-55	1.09E-54

FBF1	399	140	-1.619686295	2.79E-34	5.54E-34
COQ6	399	187	-1.202074852	2.02E-22	3.34E-22
PTRH2	399	341	-0.335341383	0.001589514	0.001750708
LOC148413	400	250	-0.786796281	6.88E-12	9.38E-12
DHRS7B	400	258	-0.74135331	7.27E-11	9.67E-11
ZNF18	400	279	-0.628459254	1.78E-08	2.24E-08
HIST2H2BE	401	94	-2.201593951	3.96E-51	9.71E-51
LOH12CR1	401	197	-1.134130983	1.14E-20	1.83E-20
LNX2	401	324	-0.416332799	0.00010639	0.000120914
PLEKHH3	402	189	-1.197533643	1.88E-22	3.11E-22
ZNF48	402	308	-0.492989526	5.78E-06	6.80E-06
HIRIP3	403	126	-1.786080481	1.80E-39	3.82E-39
CLP1	404	305	-0.514270426	2.31E-06	2.75E-06
ECHDC2	407	182	-1.26981472	9.49E-25	1.64E-24
BUD13	407	224	-0.970254438	1.38E-16	2.07E-16
THAP2	407	232	-0.919628365	2.70E-15	3.95E-15
HSPA14	407	238	-0.882791597	2.24E-14	3.21E-14
PCGF2	407	249	-0.817607428	8.52E-13	1.18E-12
CLN6	408	192	-1.196187217	1.00E-22	1.66E-22
AZI1	408	203	-1.115813801	1.68E-20	2.69E-20
TRMT61A	408	210	-1.0669042	3.56E-19	5.58E-19
CCDC122	409	124	-1.830485098	2.40E-41	5.21E-41
AGAP6	410	214	-1.046737489	1.02E-18	1.59E-18
FEN1	410	229	-0.949000687	3.79E-16	5.63E-16
IL32	410	392	-0.173494631	0.088525353	0.09211119
ANO1	412	293	-0.600468049	4.07E-08	5.07E-08
TMTC2	413	59	-2.916079298	2.27E-71	6.75E-71
SNRNP35	413	230	-0.953232296	2.30E-16	3.44E-16
ARV1	413	370	-0.267340887	0.009552318	0.010242215
ANKRD36BP1	414	175	-1.351000222	1.65E-27	2.97E-27
RILPL2	414	205	-1.122731234	5.68E-21	9.18E-21
SVIP	414	248	-0.848015023	9.86E-14	1.40E-13

ARL2	414	273	-0.709454192	1.79E-10	2.37E-10
MARCH9	414	275	-0.698923525	3.08E-10	4.05E-10
BMF	414	294	-0.602538989	3.43E-08	4.28E-08
COX14	414	348	-0.359267838	0.000599409	0.000667539
KIAA1737	415	279	-0.68157059	7.07E-10	9.23E-10
LOC645638	416	982	1.13041512	1.31E-44	2.97E-44
CALML4	416	177	-1.341558544	2.34E-27	4.21E-27
HAUS4	416	184	-1.285602138	1.02E-25	1.78E-25
NOL3	416	256	-0.809164094	7.66E-13	1.06E-12
TEAD4	417	272	-0.725165108	6.80E-11	9.05E-11
ETV3	417	276	-0.704103493	2.04E-10	2.69E-10
TXNL4B	418	196	-1.201373664	2.16E-23	3.63E-23
ZFYVE19	418	254	-0.827398821	2.43E-13	3.40E-13
TSFM	418	268	-0.749994318	1.73E-11	2.33E-11
ALG11	418	284	-0.666336388	1.32E-09	1.72E-09
GSDMB	418	342	-0.398230993	0.000146643	0.00016601
SIX1	419	266	-0.764248374	7.56E-12	1.03E-11
SLC50A1	419	414	-0.126043852	0.207225476	0.212784187
CLCF1	420	135	-1.746154297	7.55E-40	1.61E-39
FLJ10038	420	172	-1.396705139	3.00E-29	5.55E-29
WASH3P	420	193	-1.230512856	2.43E-24	4.17E-24
AIFM2	420	196	-1.208260049	1.07E-23	1.82E-23
ATL1	421	169	-1.425521363	3.51E-30	6.60E-30
DCLRE1B	421	213	-1.091691179	1.99E-20	3.19E-20
PIGB	421	244	-0.895663461	3.78E-15	5.51E-15
NBPF16	422	160	-1.50789547	9.56E-33	1.86E-32
PUSL1	423	279	-0.709116917	1.16E-10	1.54E-10
BBIP1	423	290	-0.653329139	2.04E-09	2.62E-09
CDK2AP2	423	646	0.502152126	1.81E-08	2.28E-08
SSSCA1	423	358	-0.349422452	0.000725697	0.00080749
C15orf40	424	186	-1.297486019	1.55E-26	2.75E-26
DCTPP1	424	209	-1.129285698	1.26E-21	2.06E-21

MIIP	424	236	-0.954001781	8.85E-17	1.33E-16
ACACB	424	274	-0.738612747	2.29E-11	3.09E-11
ZNF624	425	126	-1.862763483	6.93E-44	1.56E-43
ST3GAL4	425	249	-0.880041475	7.11E-15	1.03E-14
TNFSF12	425	267	-0.779347475	2.33E-12	3.20E-12
ZNF689	425	267	-0.779347475	2.33E-12	3.20E-12
RABIF	425	286	-0.68017207	4.76E-10	6.22E-10
EPB41	425	346	-0.405415179	9.92E-05	0.000112815
MDM1	426	152	-1.595506483	9.72E-36	1.96E-35
LYPLAL1	426	173	-1.408805768	5.20E-30	9.73E-30
PRC1	426	184	-1.31987204	2.53E-27	4.55E-27
SNRPF	426	242	-0.924570759	4.55E-16	6.75E-16
GHDC	426	313	-0.553415149	2.16E-07	2.64E-07
SLC35E3	427	217	-1.085265403	1.64E-20	2.62E-20
DNAJC24	427	370	-0.315435175	0.002046159	0.002245427
RNF19B	428	233	-0.986005218	8.62E-18	1.32E-17
UCHL3	428	238	-0.955373599	5.84E-17	8.82E-17
ALKBH3	428	342	-0.432338847	3.38E-05	3.89E-05
CENPBD1	429	211	-1.132459025	5.91E-22	9.72E-22
SCNN1D	429	265	-0.803709664	4.62E-13	6.44E-13
DCAF4	429	272	-0.766095372	3.86E-12	5.27E-12
SLC25A22	429	336	-0.461240791	1.05E-05	1.23E-05
TADA2A	430	201	-1.205865534	3.76E-24	6.43E-24
THAP3	430	240	-0.95002663	6.90E-17	1.04E-16
SPRN	430	311	-0.576146455	6.59E-08	8.17E-08
NDUFAF1	430	362	-0.357071338	0.000506092	0.000564743
ZDHHC24	430	376	-0.302328374	0.002954479	0.003223631
VPS33B	431	301	-0.626648758	5.50E-09	7.03E-09
PABPN1	432	153	-1.606224035	1.49E-36	3.05E-36
MORN4	432	211	-1.142512689	2.17E-22	3.59E-22
PEMT	432	246	-0.921097373	3.52E-16	5.23E-16
NGDN	432	277	-0.749869712	8.02E-12	1.09E-11

NARS2	432	281	-0.729185558	2.50E-11	3.36E-11
DCLRE1A	433	209	-1.159588459	6.13E-23	1.02E-22
WDR66	433	237	-0.978204342	9.14E-18	1.40E-17
POU6F1	433	249	-0.906945659	7.76E-16	1.15E-15
TMEM56	434	144	-1.700350607	1.12E-39	2.38E-39
ARNTL	435	258	-0.862368711	1.00E-14	1.44E-14
INCENP	435	259	-0.856787679	1.40E-14	2.01E-14
BCL7A	435	274	-0.775563884	1.60E-12	2.21E-12
SUV39H2	436	211	-1.155809512	5.64E-23	9.42E-23
EBF3	438	48	-3.298548935	1.80E-84	6.06E-84
NCR3LG1	438	252	-0.906231512	5.55E-16	8.22E-16
C15orf39	438	381	-0.309864248	0.00213541	0.002340409
ZBTB8OS	438	391	-0.272486638	0.006570521	0.00708006
ATP7B	438	429	-0.138677598	0.156875142	0.16187077
AMIGO2	439	2738	2.532105226	0	0
LYRM9	439	126	-1.909521582	9.46E-47	2.21E-46
WDR76	440	161	-1.559167211	1.06E-35	2.15E-35
MTFMT	440	278	-0.771143017	1.54E-12	2.13E-12
AFMID	440	495	0.061200626	0.516825267	0.522918957
PER3	442	225	-1.082845744	4.17E-21	6.75E-21
SWT1	443	165	-1.53356505	4.12E-35	8.25E-35
ZNF287	443	214	-1.158420278	2.14E-23	3.60E-23
C12orf43	443	264	-0.855493145	8.70E-15	1.26E-14
MT1E	444	964	1.009749094	9.43E-37	1.93E-36
RHOU	445	240	-0.999495306	8.06E-19	1.26E-18
ACSF3	445	250	-0.940601617	3.68E-17	5.59E-17
ESRRA	446	244	-0.978886938	2.85E-18	4.41E-18
NT5C	447	235	-1.036338451	5.81E-20	9.23E-20
NUDT15	447	269	-0.841393035	1.56E-14	2.25E-14
DDX19B	447	279	-0.788734085	3.67E-13	5.12E-13
TRAPPC2L	447	333	-0.53347703	2.78E-07	3.39E-07
LINC00839	448	198	-1.286722678	1.32E-27	2.38E-27

TCTN2	448	226	-1.095900336	9.26E-22	1.52E-21
SNRNP25	448	425	-0.184760267	0.058448067	0.061215257
PSEN2	450	187	-1.375611107	1.52E-30	2.88E-30
ZNF594	450	187	-1.375611107	1.52E-30	2.88E-30
OSGEP	450	210	-1.208260049	2.84E-25	4.95E-25
RCAN3	450	237	-1.033762318	5.21E-20	8.27E-20
CENPN	450	306	-0.665117724	3.34E-10	4.39E-10
ACSS3	451	94	-2.371119147	1.77E-62	4.85E-62
MAGOH	451	329	-0.563764225	5.79E-08	7.19E-08
EMG1	451	330	-0.559385785	7.16E-08	8.87E-08
DDX11	453	175	-1.480880504	3.81E-34	7.54E-34
LRRC27	453	205	-1.252611516	8.09E-27	1.44E-26
MRPL55	453	440	-0.150731902	0.118388197	0.122530607
TET1	454	194	-1.335360021	1.66E-29	3.09E-29
STOML1	455	233	-1.074260966	2.01E-21	3.28E-21
RACGAP1	456	249	-0.981612458	1.00E-18	1.56E-18
DHRS1	456	442	-0.153711831	0.110291061	0.114271315
RNPC3	457	192	-1.35981223	1.76E-30	3.32E-30
RASSF5	457	304	-0.696847218	4.21E-11	5.64E-11
MLYCD	458	213	-1.213218544	7.54E-26	1.32E-25
BAIAP2	458	262	-0.914505162	7.10E-17	1.07E-16
TMEM134	458	328	-0.590376159	1.21E-08	1.53E-08
HSD3B7	458	401	-0.300469738	0.002289857	0.002508275
RPUSD1	459	323	-0.615684365	3.15E-09	4.04E-09
ROBO3	459	423	-0.226560866	0.019710741	0.020947357
AP1G2	460	187	-1.407319967	3.19E-32	6.16E-32
PIK3C2B	460	238	-1.059396664	3.44E-21	5.58E-21
AKAP1	461	168	-1.565029894	1.62E-37	3.35E-37
DCLRE1C	461	194	-1.357434474	1.18E-30	2.23E-30
PPIP5K1	461	262	-0.923924315	3.04E-17	4.62E-17
TMEM54	461	452	-0.137168354	0.150739617	0.155642691
NBEA	462	125	-1.994689133	6.40E-52	1.58E-51

LOC652276	462	322	-0.629556539	1.34E-09	1.74E-09
SNX11	462	390	-0.353143104	0.000360595	0.000403538
OTUD1	463	142	-1.81384564	5.11E-46	1.18E-45
LRP8	463	403	-0.308956731	0.00164171	0.001807432
NDUFC2	464	165	-1.600383157	6.15E-39	1.30E-38
RRP8	464	285	-0.811887262	3.17E-14	4.53E-14
THTPA	465	207	-1.276324324	2.97E-28	5.42E-28
LRFN4	465	340	-0.560420346	4.27E-08	5.32E-08
ALDH4A1	465	394	-0.347759462	0.000419261	0.000468451
MASTL	466	175	-1.521699409	1.90E-36	3.88E-36
SUOX	466	295	-0.768339376	4.05E-13	5.65E-13
PMVK	466	313	-0.682891674	6.01E-11	8.01E-11
TNFRSF1B	467	139	-1.857062043	7.41E-48	1.75E-47
CTC1	467	220	-1.194643402	9.94E-26	1.74E-25
NAGPA	467	269	-0.904540753	6.85E-17	1.03E-16
TMEM39B	467	273	-0.883245975	2.77E-16	4.12E-16
EED	467	289	-0.801077433	5.14E-14	7.30E-14
MRPL48	467	291	-0.791127773	9.50E-14	1.35E-13
ICT1	467	348	-0.53305962	1.54E-07	1.90E-07
ELTD1	468	317	-0.670750065	1.09E-10	1.44E-10
TMEM231	468	337	-0.582484314	1.26E-08	1.59E-08
PCDH9	469	142	-1.832421369	3.21E-47	7.51E-47
PBLD	469	226	-1.161989526	8.68E-25	1.50E-24
CCDC86	469	357	-0.502388224	6.27E-07	7.58E-07
C1orf54	469	396	-0.352811868	0.00032926	0.000369318
CA12	470	81	-2.64539132	2.31E-73	7.02E-73
CRYL1	470	350	-0.534030211	1.34E-07	1.65E-07
FANCF	471	180	-1.496454529	5.80E-36	1.18E-35
CALHM2	472	140	-1.862084408	1.61E-48	3.82E-48
TRADD	472	403	-0.336731397	0.000564789	0.000629344
LOXL4	472	611	0.263661145	0.002737254	0.002990796
BRIP1	473	352	-0.534989131	1.17E-07	1.44E-07

EIF2B2	473	421	-0.276744326	0.004151899	0.004510598
ANKDD1A	474	192	-1.412505124	2.50E-33	4.89E-33
SRGN	474	919	0.846453427	1.53E-26	2.71E-26
HNRNPA1L2	474	234	-1.127102905	6.43E-24	1.09E-23
CRYAB	474	293	-0.80271077	3.01E-14	4.30E-14
MOB2	475	303	-0.757334096	4.74E-13	6.60E-13
SEMA4G	475	385	-0.411793444	2.98E-05	3.43E-05
MYEF2	476	234	-1.13317742	3.31E-24	5.66E-24
AXIN2	477	71	-2.856822712	1.65E-80	5.32E-80
KBTBD7	477	203	-1.341233915	4.10E-31	7.79E-31
ZNF839	477	246	-1.064055327	4.64E-22	7.63E-22
TRNAU1AP	477	302	-0.768165093	2.17E-13	3.05E-13
DPM3	477	441	-0.221934986	0.019783725	0.021010623
MED18	478	288	-0.839666182	2.19E-15	3.21E-15
RAD51C	480	306	-0.758227129	3.39E-13	4.74E-13
TM6SF1	480	670	0.372402314	1.37E-05	1.60E-05
XYLT1	481	2084	2.006522103	9.42E-213	7.76E-212
ZNF219	481	184	-1.495055504	1.22E-36	2.49E-36
HOXB6	482	274	-0.923581629	6.00E-18	9.23E-18
TTC5	484	256	-1.027587613	3.30E-21	5.35E-21
CCDC102A	484	257	-1.021963064	4.95E-21	8.01E-21
DUSP5	484	567	0.119617312	0.17947635	0.184849751
ANKRD13B	485	147	-1.830892968	1.02E-48	2.44E-48
ABHD15	485	271	-0.948416272	8.43E-19	1.32E-18
ASIC1	485	364	-0.522770673	1.49E-07	1.83E-07
PIGC	486	234	-1.16317216	1.15E-25	2.01E-25
TDP1	487	290	-0.856593248	3.89E-16	5.78E-16
LOC645166	487	301	-0.802882661	1.33E-14	1.92E-14
HSD17B6	487	343	-0.614437572	1.13E-09	1.46E-09
C12orf65	487	358	-0.552686561	3.07E-08	3.84E-08
TRIM21	488	234	-1.169096994	5.82E-26	1.02E-25
RAB30	488	243	-1.11464921	3.61E-24	6.16E-24

GSTM4	489	206	-1.355914504	2.31E-32	4.47E-32
SMUG1	490	250	-1.07957803	4.05E-23	6.77E-23
MRPL21	490	384	-0.460399814	2.58E-06	3.07E-06
KIAA1614	491	235	-1.171786644	3.35E-26	5.92E-26
KAT5	491	265	-0.998455041	1.43E-20	2.30E-20
FAM213A	491	273	-0.955546449	3.11E-19	4.89E-19
TIGD7	492	236	-1.168595832	3.82E-26	6.74E-26
KLHL17	492	245	-1.114600942	2.36E-24	4.06E-24
PPFIBP2	492	338	-0.650359445	1.20E-10	1.59E-10
RNASEL	493	145	-1.874259122	4.94E-51	1.21E-50
XRCC3	493	206	-1.367667685	5.05E-33	9.82E-33
WDHD1	493	208	-1.353728494	1.55E-32	3.00E-32
PITPNM3	494	208	-1.356651889	1.06E-32	2.05E-32
ZNF213	494	274	-0.959059525	1.89E-19	2.98E-19
C15orf38	495	148	-1.850555725	2.18E-50	5.32E-50
KIAA1549L	495	332	-0.68496966	1.36E-11	1.84E-11
LYRM5	495	398	-0.42338447	1.22E-05	1.43E-05
LOC440434	496	213	-1.328211066	7.87E-32	1.51E-31
SLC38A6	498	256	-1.068726308	4.10E-23	6.86E-23
GPATCH2	498	273	-0.975969167	3.98E-20	6.33E-20
MAP4K2	498	335	-0.680709023	1.53E-11	2.06E-11
PRMT3	499	334	-0.687916089	9.43E-12	1.28E-11
HIP1R	500	320	-0.752580566	1.58E-13	2.23E-13
DNALI1	501	254	-1.088706482	6.63E-24	1.13E-23
PEX14	501	367	-0.557754916	1.49E-08	1.88E-08
PCCA	501	476	-0.182573406	0.047906729	0.050275991
FMOD	502	112	-2.272913008	5.24E-66	1.49E-65
TRPV2	502	599	0.146144263	0.09335197	0.097016973
WDR90	503	197	-1.461087146	5.19E-37	1.07E-36
MRPS31	504	288	-0.916079298	1.83E-18	2.85E-18
ARMC5	504	296	-0.876550934	3.00E-17	4.57E-17
IGHMBP2	504	301	-0.852384623	1.61E-16	2.41E-16

NUDT22	504	431	-0.33446024	0.000399654	0.000446992
NSMCE4A	505	218	-1.320679629	4.14E-32	7.97E-32
ZNF32	506	237	-1.202974702	4.98E-28	9.05E-28
COX10	506	298	-0.87254943	3.44E-17	5.23E-17
NR1H3	507	117	-2.224201593	5.91E-65	1.66E-64
ACER3	507	230	-1.249076262	1.08E-29	2.01E-29
KIF20B	508	116	-2.239428068	1.31E-65	3.69E-65
SIAH1	509	192	-1.515283721	2.04E-39	4.34E-39
IKBKE	509	296	-0.890792856	7.64E-18	1.17E-17
RASGRP1	511	250	-1.140119572	4.18E-26	7.36E-26
TMEM199	511	275	-1.002616048	1.77E-21	2.88E-21
TRMT13	511	280	-0.97662084	1.24E-20	2.00E-20
ZNF830	511	304	-0.857976343	6.74E-17	1.02E-16
RNF219	512	216	-1.353836874	9.93E-34	1.95E-33
ZNF641	512	246	-1.166209871	4.67E-27	8.36E-27
PALB2	512	264	-1.064330257	1.43E-23	2.42E-23
ISCA2	512	306	-0.851336533	1.00E-16	1.51E-16
RBMXL1	512	331	-0.738036969	2.10E-13	2.95E-13
MRP63	512	400	-0.464868186	1.24E-06	1.49E-06
NUAK2	512	465	-0.24763747	0.007317172	0.007878091
ZDHHC13	513	229	-1.272335603	7.44E-31	1.41E-30
WHAMMP1	514	177	-1.646743375	1.13E-44	2.57E-44
TARS2	514	423	-0.389835072	3.67E-05	4.22E-05
IRAK3	515	95	-2.54729739	5.12E-77	1.61E-76
TCEANC2	515	219	-1.342365938	1.69E-33	3.31E-33
ALKBH8	515	252	-1.139873074	2.74E-26	4.84E-26
HMBS	516	454	-0.293403144	0.001551305	0.001709105
ERVK13-1	517	251	-1.151201292	8.84E-27	1.58E-26
SCAND2P	517	255	-1.128391409	5.51E-26	9.69E-26
CIDEB	517	337	-0.726140065	3.49E-13	4.88E-13
PRTFDC1	518	137	-2.027500581	3.34E-59	8.88E-59
SLC36A4	518	383	-0.544332082	1.77E-08	2.23E-08

MLST8	518	420	-0.411287146	1.33E-05	1.55E-05
ERI2	519	168	-1.735997681	1.91E-48	4.54E-48
TCHP	519	282	-0.988763752	2.49E-21	4.04E-21
CDK8	519	301	-0.894695427	2.75E-18	4.26E-18
DNAAF2	519	304	-0.880387591	7.76E-18	1.19E-17
NET1	520	376	-0.576503337	2.78E-09	3.58E-09
UBALD1	520	386	-0.538635152	2.27E-08	2.85E-08
RAB15	521	485	-0.212023001	0.019765781	0.020994421
LRRC20	522	126	-2.159350449	1.76E-64	4.93E-64
MEG9	522	218	-1.368446048	6.78E-35	1.35E-34
TMEM69	522	331	-0.765942966	1.91E-14	2.74E-14
MED9	523	275	-1.036103704	4.46E-23	7.46E-23
MOB3C	523	342	-0.721538997	3.45E-13	4.83E-13
KNDC1	524	190	-1.572291769	1.11E-42	2.45E-42
ZNF785	525	247	-1.196530757	8.68E-29	1.59E-28
URB2	525	361	-0.649042962	3.14E-11	4.21E-11
COL8A2	525	749	0.40392392	7.07E-07	8.53E-07
SLC24A1	526	216	-1.392755863	4.67E-36	9.47E-36
TMEM52B	527	152	-1.902456014	1.70E-55	4.35E-55
TATDN3	527	234	-1.280018808	6.20E-32	1.19E-31
ABCA8	528	33	-4.108724376	1.63E-118	7.25E-118
C1orf233	528	164	-1.795566491	1.57E-51	3.86E-51
LIMD2	528	186	-1.613959684	1.38E-44	3.13E-44
PCGF6	528	240	-1.2462279	9.46E-31	1.79E-30
PLK3	528	613	0.106624768	0.212411623	0.217994791
OMA1	530	217	-1.397021693	1.77E-36	3.61E-36
CYB5D1	530	252	-1.181293002	1.71E-28	3.12E-28
SSX2IP	530	400	-0.514716736	6.12E-08	7.60E-08
PTPLA	530	669	0.227289475	0.006539628	0.007047744
EXOSC8	531	230	-1.315802376	1.71E-33	3.35E-33
HS3ST3B1	531	277	-1.047550261	8.42E-24	1.43E-23
SYT1	531	278	-1.042351354	1.27E-23	2.16E-23

CLN3	531	353	-0.697768053	1.09E-12	1.51E-12
MED19	533	322	-0.835799221	7.37E-17	1.11E-16
TACO1	533	418	-0.459356967	9.92E-07	1.19E-06
TGDS	533	462	-0.314967057	0.000581383	0.000647649
ZNF143	534	285	-1.014602199	8.73E-23	1.45E-22
NMRAL1	534	399	-0.529175371	2.48E-08	3.11E-08
DUSP8	535	189	-1.609877033	5.37E-45	1.22E-44
PGP	535	326	-0.823391303	1.58E-16	2.37E-16
RCCD1	536	316	-0.871032818	4.56E-18	7.03E-18
METTL2A	536	382	-0.597384738	4.65E-10	6.08E-10
TFB2M	536	442	-0.386911007	2.85E-05	3.29E-05
C11orf74	537	359	-0.68966262	1.38E-12	1.91E-12
ITPK1	538	233	-1.316000594	6.39E-34	1.26E-33
TMEM101	538	456	-0.347296724	0.000150813	0.000170681
GLT25D2	540	245	-1.248902034	1.64E-31	3.13E-31
OSBPL7	540	282	-1.045988621	4.02E-24	6.86E-24
RAB40C	540	422	-0.464440784	6.51E-07	7.87E-07
WDFY2	540	591	0.021474347	0.802388458	0.805897294
FJX1	541	497	-0.231107118	0.009871066	0.010568022
C10orf54	542	99	-2.561516797	1.90E-81	6.17E-81
SOX5	542	279	-1.066752105	6.08E-25	1.05E-24
ALDH3A2	544	134	-2.130098027	5.20E-66	1.47E-65
ANKRD49	544	299	-0.972185543	1.04E-21	1.71E-21
SCRN2	544	453	-0.372819977	4.64E-05	5.32E-05
IFT81	545	270	-1.122021199	4.53E-27	8.11E-27
FLYWCH2	545	340	-0.789445859	9.68E-16	1.43E-15
METTL23	545	483	-0.282957417	0.001675019	0.001842804
PRDM11	546	106	-2.473561062	6.12E-79	1.94E-78
CISD1	546	259	-1.184673229	1.98E-29	3.67E-29
TMEM126A	546	418	-0.494122385	1.19E-07	1.47E-07
TMEM208	546	560	-0.0721985	0.405169773	0.411332651
CHKA	547	320	-0.882193304	8.95E-19	1.40E-18

METTL21B	547	391	-0.593096601	4.01E-10	5.25E-10
TTLL7	547	424	-0.476200944	2.99E-07	3.65E-07
CKS1B	548	301	-0.973136782	6.87E-22	1.13E-21
PAAF1	548	353	-0.743232086	2.13E-14	3.05E-14
C16orf70	548	363	-0.702930721	3.37E-13	4.71E-13
WDR25	549	349	-0.762303489	5.29E-15	7.68E-15
KNSTRN	550	391	-0.600987387	2.22E-10	2.93E-10
SNRPA1	550	490	-0.275374245	0.002095616	0.002298085
TPCN2	551	194	-1.614720042	1.75E-46	4.07E-46
PRDM10	552	303	-0.974074849	4.53E-22	7.46E-22
BLZF1	552	432	-0.462361331	5.45E-07	6.61E-07
TMEM11	552	450	-0.403467641	1.00E-05	1.17E-05
TIMM8B	553	311	-0.939089276	6.81E-21	1.10E-20
AKTIP	553	434	-0.458308814	6.57E-07	7.93E-07
SPOCD1	553	769	0.366979742	4.33E-06	5.11E-06
ACOT2	554	261	-1.194560545	3.27E-30	6.15E-30
WDR73	554	273	-1.129709401	8.92E-28	1.62E-27
TRIM3	554	319	-0.905053928	9.16E-20	1.45E-19
LOXL1-AS1	554	365	-0.710713888	1.47E-13	2.08E-13
FANCI	555	170	-1.815677401	7.01E-55	1.78E-54
TTF2	555	238	-1.330250574	1.70E-35	3.43E-35
ERO1LB	555	279	-1.100947025	9.33E-27	1.66E-26
SPATA7	557	239	-1.329391085	1.39E-35	2.80E-35
METRN	557	384	-0.645295393	1.02E-11	1.38E-11
MEIS3P1	559	219	-1.460641789	6.38E-41	1.38E-40
ULK3	559	224	-1.428073927	1.28E-39	2.72E-39
ENTHD2	559	264	-1.191034729	2.47E-30	4.64E-30
RPUSD4	560	416	-0.537567675	7.01E-09	8.93E-09
PIP4K2C	560	484	-0.319144156	0.000356446	0.000399238
ADK	561	548	-0.142549254	0.099808915	0.103658441
ALDOC	562	971	0.680176789	1.25E-19	1.98E-19
MSI2	562	356	-0.767417265	1.75E-15	2.57E-15

TARBP2	562	372	-0.703991885	1.57E-13	2.22E-13
EIF2B3	562	563	-0.106159584	0.217016062	0.22266173
NUP85	564	297	-1.033956607	1.11E-24	1.92E-24
GALK1	564	475	-0.356492025	6.99E-05	7.99E-05
MTHFSD	566	262	-1.219959617	8.14E-32	1.56E-31
PYROXD1	566	302	-1.014977879	4.56E-24	7.77E-24
ASPM	567	116	-2.397948306	4.40E-79	1.40E-78
KIF11	567	191	-1.678500473	2.12E-50	5.16E-50
TMEM254	567	289	-1.081003618	1.50E-26	2.66E-26
WDR74	567	309	-0.984466273	5.32E-23	8.88E-23
TMEM138	568	332	-0.883432064	1.77E-19	2.80E-19
PRRT2	569	165	-1.894687004	1.86E-59	4.95E-59
RORA	570	262	-1.230119483	1.99E-32	3.85E-32
LOC10013224	571	233	-1.401885167	2.29E-39	4.86E-39
NEK3	572	248	-1.314399402	6.82E-36	1.38E-35
MIPOL1	572	319	-0.951183099	5.47E-22	9.01E-22
APIP	572	409	-0.59263868	1.65E-10	2.19E-10
PC	572	502	-0.297052159	0.000747486	0.000831498
METTL22	572	593	-0.056707418	0.502219022	0.508272206
FAM213B	573	304	-1.023188191	1.20E-24	2.06E-24
NIT1	573	405	-0.609337607	5.48E-11	7.31E-11
RCBTB2	574	199	-1.637006682	2.83E-49	6.78E-49
GLMN	574	233	-1.409445158	7.14E-40	1.52E-39
ZNHIT3	574	427	-0.535539043	5.14E-09	6.57E-09
MPI	574	450	-0.459850111	3.72E-07	4.52E-07
FUT4	575	339	-0.871001059	2.88E-19	4.52E-19
ST3GAL3	575	502	-0.304598968	0.00053673	0.000598248
NPIPL3	576	243	-1.353836874	9.87E-38	2.05E-37
SOCS1	577	133	-2.225869449	9.85E-74	3.00E-73
KAT8	577	263	-1.242232895	2.74E-33	5.35E-33
CRLF3	577	312	-0.995749666	8.59E-24	1.46E-23
CTNS	578	305	-1.030984626	3.85E-25	6.69E-25

FOSL1	578	623	-0.000561705	0.994617042	0.994744426
ZNF202	579	295	-1.08157277	4.24E-27	7.61E-27
ENOX1	580	61	-3.357896128	6.82E-113	2.91E-112
ZFP1	580	266	-1.23335103	4.27E-33	8.31E-33
CPNE7	581	156	-2.00571651	2.78E-65	7.83E-65
ZNF10	581	218	-1.522934405	4.59E-45	1.05E-44
ZNF75A	581	243	-1.366306226	1.48E-38	3.10E-38
WIBG	581	424	-0.563198275	7.65E-10	9.98E-10
IFIT3	582	160	-1.971671624	5.57E-64	1.55E-63
TANGO6	582	443	-0.50243683	2.84E-08	3.55E-08
ATP5G1	582	600	-0.064781028	0.440039997	0.445979537
CROCCP3	583	221	-1.50817389	1.36E-44	3.07E-44
SNHG3	584	571	-0.141201999	0.096172774	0.099921926
ENTPD5	585	254	-1.312332504	1.39E-36	2.84E-36
GPATCH4	585	315	-1.001809172	2.51E-24	4.30E-24
NSUN6	586	163	-1.954753076	1.06E-63	2.94E-63
NRDE2	586	247	-1.355113999	2.07E-38	4.34E-38
ZNF500	587	295	-1.101369925	3.21E-28	5.84E-28
COG8	587	447	-0.501810048	2.57E-08	3.22E-08
SPRYD7	587	508	-0.317256382	0.000277958	0.000312223
BRMS1L	588	281	-1.173970401	3.88E-31	7.39E-31
ATF3	588	299	-1.084395047	1.33E-27	2.39E-27
RIN1	588	308	-1.04161018	5.92E-26	1.04E-25
YPEL2	588	331	-0.937709314	4.59E-22	7.57E-22
INO80E	588	370	-0.77701526	1.90E-16	2.85E-16
PLXDC2	589	955	0.588508723	2.74E-15	4.00E-15
IFT88	590	206	-1.626794993	3.66E-50	8.89E-50
LOC643837	590	256	-1.31329552	6.37E-37	1.31E-36
MSANTD2	591	307	-1.053643851	1.53E-26	2.71E-26
GFOD2	592	362	-0.818331854	5.98E-18	9.21E-18
CENPJ	592	373	-0.775145921	1.74E-16	2.60E-16
FAR2	593	165	-1.954290456	2.03E-64	5.66E-64

FOXO3B	593	229	-1.481408882	3.45E-44	7.79E-44
ATP1B1	593	526	-0.281693681	0.00109827	0.001214099
MRPS7	594	499	-0.360147491	3.78E-05	4.35E-05
GRIA4	595	70	-3.196187217	5.23E-111	2.19E-110
BEND3P3	595	438	-0.550683174	1.06E-09	1.37E-09
ARHGAP20	596	114	-2.495002882	7.95E-87	2.73E-86
KIAA1467	596	216	-1.573005394	2.46E-48	5.84E-48
UBALD2	596	380	-0.758037288	5.14E-16	7.62E-16
IBA57	597	224	-1.522956575	2.94E-46	6.81E-46
ZNF692	597	253	-1.347317922	9.05E-39	1.91E-38
CWF19L2	597	302	-1.091906758	2.68E-28	4.89E-28
LCAT	598	272	-1.245263209	1.46E-34	2.90E-34
TMEM218	598	342	-0.914873535	1.44E-21	2.35E-21
GPN2	598	391	-0.721701253	7.18E-15	1.04E-14
FAM118B	598	404	-0.674514567	2.26E-13	3.18E-13
LOC10052796	599	110	-2.553776855	1.61E-89	5.65E-89
PYROXD2	599	278	-1.216195496	2.04E-33	3.99E-33
SPRTN	600	449	-0.526971432	3.94E-09	5.05E-09
HLX	601	219	-1.565158497	2.19E-48	5.21E-48
SLC48A1	601	310	-1.063821151	2.31E-27	4.15E-27
SP2	602	226	-1.52216509	1.35E-46	3.14E-46
ZNF286A	602	329	-0.980400279	3.64E-24	6.23E-24
ZNF319	602	360	-0.850490956	2.31E-19	3.64E-19
ANKS3	602	486	-0.417531549	1.92E-06	2.30E-06
ARHGAP22	603	1051	0.692808386	9.30E-22	1.52E-21
MPHOSPH9	603	358	-0.860922791	9.13E-20	1.45E-19
YARS2	603	422	-0.623639379	6.52E-12	8.90E-12
MAD2L2	603	551	-0.238830059	0.004932146	0.005344128
NRIP3	603	674	0.051866213	0.520809863	0.526745787
TMEM204	604	364	-0.839334475	5.04E-19	7.89E-19
DPH2	607	452	-0.53409812	2.05E-09	2.65E-09
SLC15A4	607	456	-0.521387068	4.53E-09	5.79E-09

C10orf88	607	496	-0.400080772	4.33E-06	5.11E-06
PITPNC1	608	242	-1.437788652	2.32E-43	5.17E-43
ZNF668	608	593	-0.144763595	0.081984724	0.085396654
NLE1	609	327	-1.005875968	2.04E-25	3.56E-25
SLC25A16	609	418	-0.651663662	7.13E-13	9.89E-13
CHEK1	609	506	-0.376029219	1.40E-05	1.63E-05
MAPK8IP1	610	255	-1.367036371	1.99E-40	4.27E-40
NUBPL	610	339	-0.956248345	1.55E-23	2.62E-23
PARP11	611	283	-1.219094703	3.56E-34	7.06E-34
ZSCAN2	611	300	-1.134934255	1.12E-30	2.12E-30
NARFL	611	302	-1.125348206	2.77E-30	5.20E-30
PGAM5	611	442	-0.575850386	1.22E-10	1.62E-10
ZNF678	612	179	-1.882296441	2.88E-63	7.92E-63
SMYD4	612	306	-1.108724376	1.19E-29	2.21E-29
GTF2B	612	401	-0.718653792	4.41E-15	6.42E-15
B3GALTL	613	255	-1.374114203	6.34E-41	1.37E-40
NLRX1	613	433	-0.610244425	1.11E-11	1.50E-11
ALG1	613	516	-0.357240384	3.27E-05	3.77E-05
USP35	614	359	-0.882979187	6.55E-21	1.06E-20
POC1B	614	468	-0.500454502	1.34E-08	1.69E-08
NUFIP1	615	344	-0.946902221	2.35E-23	3.95E-23
NSUN4	615	375	-0.822420191	9.86E-19	1.54E-18
AGTRAP	615	484	-0.454303739	1.96E-07	2.40E-07
SBF2-AS1	616	909	0.452625568	1.27E-09	1.65E-09
TMEM106C	616	479	-0.471629071	7.06E-08	8.75E-08
NUDT16L1	617	484	-0.458987818	1.43E-07	1.77E-07
SYNM	618	742	0.155087973	0.047840102	0.050212817
MYRF	619	232	-1.52453898	5.73E-48	1.36E-47
COMMD4	619	305	-1.129854543	7.60E-31	1.44E-30
TUBG1	620	383	-0.803648199	3.37E-18	5.21E-18
RABGGTA	620	550	-0.281560973	0.000849778	0.000943406
ZNF672	621	490	-0.450535895	2.12E-07	2.60E-07

NPM3	621	517	-0.373153364	1.33E-05	1.55E-05
KIF23	623	293	-1.197055874	7.17E-34	1.41E-33
HNRNPU-AS1	624	264	-1.349732475	1.50E-40	3.21E-40
LDLRAD3	625	205	-1.716956656	4.11E-57	1.07E-56
MTMR11	625	298	-1.177268235	3.94E-33	7.69E-33
TMEM55B	625	511	-0.399257275	3.26E-06	3.86E-06
COQ7	627	214	-1.659579022	1.10E-54	2.79E-54
ABCC3	627	225	-1.587264817	2.10E-51	5.17E-51
PRR11	627	327	-1.047899183	8.01E-28	1.45E-27
DHRS4-AS1	628	352	-0.943913506	1.07E-23	1.81E-23
ZBTB18	628	395	-0.777636282	1.75E-17	2.68E-17
HOXB5	628	421	-0.685668702	2.47E-14	3.54E-14
EXOC6	628	456	-0.570455111	9.78E-11	1.30E-10
CD68	629	1291	0.928632703	1.57E-42	3.47E-42
TRAPPC4	630	492	-0.465417889	7.27E-08	9.00E-08
PIGV	631	366	-0.894520733	6.93E-22	1.14E-21
SNUPN	631	419	-0.699414137	7.48E-15	1.08E-14
C12orf29	631	560	-0.280937554	0.000783877	0.000870987
PARP2	632	373	-0.869473304	5.80E-21	9.36E-21
APOA1BP	632	532	-0.357222689	2.47E-05	2.86E-05
NT5DC3	632	565	-0.270398067	0.001191498	0.001315483
CHCHD1	632	584	-0.222680565	0.007120791	0.007667713
TUT1	633	384	-0.829823565	1.65E-19	2.61E-19
PNPO	635	358	-0.93552138	1.30E-23	2.20E-23
ZWILCH	635	419	-0.708530724	3.02E-15	4.42E-15
TIMM9	635	493	-0.473893321	3.88E-08	4.84E-08
MED6	636	469	-0.548163219	3.36E-10	4.41E-10
ZBTB7B	636	609	-0.171308913	0.03613391	0.038064171
UBTD1	636	673	-0.027144637	0.733534736	0.737976392
MCTP2	636	687	0.002558958	0.974266211	0.974640641
VDR	637	1476	1.103603068	1.05E-63	2.91E-63
C16orf88	637	413	-0.733875967	3.66E-16	5.44E-16

KIAA1522	637	484	-0.505010701	5.39E-09	6.88E-09
FPGT	638	316	-1.122356241	2.02E-31	3.86E-31
KANSL2	638	397	-0.793141793	2.68E-18	4.15E-18
CHMP6	638	413	-0.736139018	2.90E-16	4.32E-16
NMNAT1	639	360	-0.936543401	8.60E-24	1.46E-23
VPS9D1	639	361	-0.93254147	1.24E-23	2.10E-23
KLF5	639	427	-0.690304237	1.03E-14	1.48E-14
MRPL24	639	463	-0.573528114	5.44E-11	7.26E-11
MSRB1	639	543	-0.343588109	4.34E-05	4.98E-05
LPCAT4	640	401	-0.783194044	5.47E-18	8.44E-18
TLE3	640	483	-0.514773092	2.66E-09	3.42E-09
CXCL12	641	46	-3.909342966	3.21E-139	1.65E-138
RABEP2	641	362	-0.933059035	1.01E-23	1.71E-23
DDX51	642	339	-1.0300124	1.06E-27	1.91E-27
FLAD1	642	418	-0.727794731	4.59E-16	6.81E-16
RTKN2	643	101	-2.77918782	6.47E-105	2.60E-104
C17orf75	643	387	-0.841209547	3.20E-20	5.10E-20
MZT1	643	568	-0.287652184	0.000525905	0.000586266
KATNB1	644	309	-1.168178226	1.09E-33	2.14E-33
HSPB11	646	282	-1.304563378	7.22E-40	1.54E-39
TXNDC16	647	132	-2.401952159	3.82E-90	1.34E-89
DDX55	647	324	-1.106496275	3.72E-31	7.09E-31
YAF2	647	391	-0.835321481	4.10E-20	6.53E-20
SLC43A1	647	429	-0.70151244	2.91E-15	4.25E-15
ALG6	648	235	-1.572057432	2.36E-52	5.85E-52
ORAOV1	648	312	-1.16317216	1.15E-33	2.25E-33
SLC30A4	648	424	-0.720653924	5.98E-16	8.86E-16
ANKRD13D	649	391	-0.839774247	2.43E-20	3.89E-20
DTX3	650	288	-1.283095282	3.96E-39	8.38E-39
TMEM201	651	262	-1.421815108	1.46E-45	3.36E-45
GCSH	651	407	-0.786353125	2.18E-18	3.39E-18
PIANP	651	423	-0.730724256	2.26E-16	3.38E-16

CINP	651	548	-0.357206026	1.87E-05	2.16E-05
CUEDC2	652	450	-0.643671339	2.06E-13	2.89E-13
ATAD3A	652	543	-0.372644142	8.32E-06	9.74E-06
CLEC14A	652	565	-0.315345473	0.000139424	0.000157952
LINC00294	653	555	-0.343319596	3.62E-05	4.17E-05
TMEM26	654	53	-3.733950747	9.99E-138	5.08E-137
NETO2	654	411	-0.778876618	3.45E-18	5.34E-18
ITPKB	655	167	-2.08037118	8.12E-77	2.55E-76
FAM63A	655	275	-1.360787664	5.44E-43	1.21E-42
HOXB7	655	365	-0.952322819	5.43E-25	9.42E-25
ZSCAN32	655	424	-0.736155018	1.17E-16	1.76E-16
AGO3	656	375	-0.915529595	1.57E-23	2.65E-23
B3GAT3	657	557	-0.346940419	2.87E-05	3.31E-05
DYRK4	657	669	-0.082611536	0.296974197	0.303186234
USP31	658	275	-1.367380341	1.74E-43	3.88E-43
POLL	658	434	-0.709116917	9.17E-16	1.35E-15
LMF1	658	523	-0.440001013	1.74E-07	2.14E-07
CTF1	659	278	-1.353917958	6.36E-43	1.41E-42
TRIM16	659	435	-0.70798744	9.57E-16	1.41E-15
STXBP4	660	301	-1.241426913	8.55E-38	1.78E-37
HTATIP2	660	334	-1.091342298	4.39E-31	8.34E-31
C1orf109	660	372	-0.935887779	1.71E-24	2.94E-24
MR1	661	181	-1.97738495	1.10E-72	3.33E-72
KNTC1	662	289	-1.3044861	8.28E-41	1.79E-40
DNAJC9	662	320	-1.157483688	4.20E-34	8.31E-34
TDRD3	665	238	-1.591117143	1.35E-54	3.43E-54
PPTC7	665	384	-0.900972406	3.07E-23	5.15E-23
ENDOV	665	446	-0.685035006	4.57E-15	6.64E-15
FAH	665	447	-0.681803885	5.92E-15	8.58E-15
ABAT	665	505	-0.505795329	2.37E-09	3.06E-09
DPYSL4	666	363	-0.984277005	1.00E-26	1.79E-26
STUB1	666	486	-0.563290239	4.47E-11	5.97E-11

PCBD1	666	487	-0.560324781	5.51E-11	7.36E-11
CPNE2	667	424	-0.762346873	6.71E-18	1.03E-17
DARS2	667	526	-0.451348338	7.21E-08	8.93E-08
CHAC1	669	1360	0.91480416	1.22E-43	2.73E-43
ATG4C	669	314	-1.199966028	2.23E-36	4.53E-36
ID3	669	731	0.019140819	0.803994033	0.807406045
SLX4	670	419	-0.785935228	7.31E-19	1.14E-18
EXOSC6	671	342	-1.081040817	4.07E-31	7.74E-31
METTL10	671	453	-0.675526092	7.42E-15	1.07E-14
COQ9	671	461	-0.650270392	5.51E-14	7.82E-14
EIF1AD	672	519	-0.48145107	9.66E-09	1.22E-08
MNAT1	672	678	-0.095900336	0.221844626	0.227556112
LRRC8D	672	716	-0.017226021	0.823965202	0.826505364
POLD3	673	226	-1.683008108	1.05E-59	2.80E-59
PRUNE	673	353	-1.039662697	2.21E-29	4.09E-29
DUSP12	673	377	-0.944766357	2.57E-25	4.49E-25
DAK	673	406	-0.837851153	5.87E-21	9.48E-21
B4GALT3	673	570	-0.348368961	2.13E-05	2.47E-05
PHYH	674	339	-1.100187694	4.20E-32	8.10E-32
CCNJ	674	398	-0.868704537	3.21E-22	5.31E-22
RNF135	675	370	-0.976086607	1.03E-26	1.83E-26
DEDD	677	414	-0.818249442	2.68E-20	4.28E-20
GLTPD1	677	542	-0.429587358	2.17E-07	2.66E-07
TIMELESS	680	165	-2.151793098	4.48E-83	1.48E-82
IFT46	680	412	-0.831614785	6.54E-21	1.06E-20
LSM10	680	502	-0.546571758	9.31E-11	1.24E-10
WARS2	681	320	-1.198307269	6.35E-37	1.31E-36
USP21	681	409	-0.844278331	1.91E-21	3.11E-21
USP30	682	362	-1.022506418	5.39E-29	9.91E-29
NUP37	682	374	-0.975457845	6.03E-27	1.08E-26
LRRK1	683	160	-2.202538049	7.79E-86	2.65E-85
HSD17B2	683	492	-0.581951639	6.42E-12	8.76E-12

PANK4	683	536	-0.458376954	3.23E-08	4.04E-08
CYGB	685	190	-1.958828945	2.39E-74	7.31E-74
AMZ2P1	685	267	-1.467988622	4.14E-50	1.00E-49
RPA2	686	387	-0.934599386	2.44E-25	4.25E-25
IFT43	686	432	-0.77590164	6.92E-19	1.08E-18
YOD1	687	357	-1.053110401	1.47E-30	2.77E-30
RNASEH2B	688	218	-1.766804806	3.63E-65	1.02E-64
DYRK3	688	390	-0.927658817	4.10E-25	7.13E-25
INTS7	690	398	-0.902552307	4.05E-24	6.91E-24
PRMT6	691	302	-1.302861537	1.93E-42	4.25E-42
C14orf37	691	571	-0.383919341	2.38E-06	2.84E-06
CWC25	692	408	-0.870927262	7.32E-23	1.22E-22
TTC13	692	513	-0.540537588	9.85E-11	1.31E-10
SEC31B	693	225	-1.731654727	7.26E-64	2.02E-63
CAMTA1	693	556	-0.426494845	1.89E-07	2.32E-07
TSPAN31	694	559	-0.420811756	2.61E-07	3.19E-07
CORO2B	695	214	-1.808126557	6.93E-68	2.00E-67
THEM4	695	285	-1.394775434	3.49E-47	8.18E-47
FKBP11	695	1251	0.739272531	1.78E-28	3.26E-28
LRRK2	697	97	-2.95382638	1.27E-120	5.74E-120
MTG1	697	312	-1.268337003	4.08E-41	8.84E-41
GANC	697	350	-1.10252811	3.00E-33	5.86E-33
C11orf48	697	636	-0.240856266	0.002309734	0.002529694
MED4	698	459	-0.713457259	8.61E-17	1.30E-16
VPS37C	698	502	-0.584264048	3.17E-12	4.35E-12
COMMD9	698	561	-0.423950641	1.99E-07	2.44E-07
SCYL3	699	279	-1.433751709	2.19E-49	5.27E-49
EFTUD1	699	348	-1.114929525	6.45E-34	1.27E-33
NVL	699	370	-1.026491561	7.42E-30	1.38E-29
MBIP	699	382	-0.980444193	8.50E-28	1.54E-27
GORAB	700	390	-0.952605174	1.31E-26	2.33E-26
OBFC1	700	490	-0.623297549	1.40E-13	1.98E-13

CEP112	701	280	-1.432711993	1.81E-49	4.35E-49
DMAP1	701	399	-0.921750074	2.66E-25	4.63E-25
FBXO33	701	426	-0.82728539	2.49E-21	4.05E-21
BICD1	701	429	-0.817161172	6.44E-21	1.04E-20
MAPKAPK5	701	454	-0.735446523	1.08E-17	1.66E-17
INTS5	701	526	-0.523076021	2.62E-10	3.45E-10
WHAMM	703	307	-1.30401041	3.33E-43	7.41E-43
TRIM11	703	468	-0.695740535	3.09E-16	4.59E-16
AVEN	703	946	0.319591118	7.65E-06	8.96E-06
BAD	704	503	-0.593741405	1.23E-12	1.71E-12
MRPL11	705	486	-0.64539132	1.92E-14	2.75E-14
NTF3	705	555	-0.453859862	2.61E-08	3.27E-08
GPAM	706	475	-0.680465046	9.84E-16	1.45E-15
ARHGAP42	707	258	-1.563063525	1.70E-56	4.37E-56
NBPF15	707	313	-1.284271934	1.82E-42	4.03E-42
TELO2	708	393	-0.957944424	3.95E-27	7.08E-27
CDH24	709	317	-1.270027163	7.06E-42	1.54E-41
C16orf80	709	421	-0.86068977	5.97E-23	9.95E-23
TTLL5	711	400	-0.938573936	2.23E-26	3.94E-26
RBM4B	711	441	-0.79779528	2.12E-20	3.38E-20
TMCO4	712	174	-2.141514311	2.19E-86	7.49E-86
ATE1	712	292	-1.394633248	2.75E-48	6.53E-48
GABPB1	712	409	-0.908500774	4.28E-25	7.44E-25
ING1	712	469	-0.711013694	5.28E-17	7.99E-17
ITIH5	713	107	-2.845015656	6.75E-119	3.02E-118
SPATA6	713	232	-1.728501647	1.71E-65	4.83E-65
SRGAP2C	713	416	-0.886042924	3.73E-24	6.37E-24
DNAL1	714	477	-0.690659184	2.82E-16	4.21E-16
CNTROB	715	425	-0.859204777	4.55E-23	7.61E-23
TMEM117	715	429	-0.84568997	1.70E-22	2.82E-22
E4F1	716	408	-0.920114811	9.77E-26	1.71E-25
MUS81	716	565	-0.450433096	2.55E-08	3.19E-08

POLR3B	717	441	-0.809918839	4.65E-21	7.53E-21
PTPMT1	717	486	-0.669741181	1.48E-15	2.18E-15
ENO2	718	863	0.156652339	0.031162864	0.032885244
CYTH1	719	382	-1.021143508	2.05E-30	3.86E-30
CDK10	721	365	-1.090827171	8.92E-34	1.75E-33
NUBP2	721	510	-0.608226388	2.08E-13	2.92E-13
ZNF140	722	380	-1.034723794	3.62E-31	6.88E-31
COL11A1	723	2063	1.403951893	7.06E-128	3.36E-127
CREM	724	326	-1.259842109	3.25E-42	7.15E-42
BCL7C	724	474	-0.719827014	1.31E-17	2.02E-17
SPRYD3	725	689	-0.182201388	0.01754886	0.018685444
LRIG2	727	411	-0.931541346	1.28E-26	2.27E-26
SIRT3	728	311	-1.335748246	2.67E-46	6.18E-46
DGKE	728	389	-1.012892671	2.18E-30	4.11E-30
ARHGEF10L	728	440	-0.835159303	2.00E-22	3.31E-22
TMEM25	728	504	-0.639239093	1.22E-14	1.76E-14
MTIF3	729	560	-0.489216363	1.33E-09	1.73E-09
WDR24	730	474	-0.731733781	3.27E-18	5.06E-18
C1orf174	730	629	-0.323560823	3.63E-05	4.18E-05
PFAS	731	459	-0.780101628	3.40E-20	5.41E-20
CCPG1	731	706	-0.158927599	0.036744751	0.038691985
ISG15	731	747	-0.077487539	0.301699186	0.307929545
PAFAH2	732	427	-0.886331955	9.09E-25	1.57E-24
AVPI1	732	493	-0.678980378	3.36E-16	5.00E-16
TMTC4	734	261	-1.600454632	1.44E-60	3.88E-60
LINC00341	734	288	-1.458435627	4.60E-53	1.15E-52
MLLT11	734	1086	0.456447759	2.27E-11	3.06E-11
CWF19L1	735	460	-0.784834765	1.71E-20	2.74E-20
VWA1	735	493	-0.684880979	1.74E-16	2.60E-16
ZFPL1	735	602	-0.396705139	5.26E-07	6.38E-07
TMEM5	736	402	-0.981234641	3.23E-29	5.96E-29
PRR14	736	405	-0.970508234	1.02E-28	1.86E-28

JMJD6	736	852	0.102423288	0.157598437	0.162595613
IFFO2	736	835	0.073346055	0.313976835	0.320293286
OTUD3	738	530	-0.586352832	6.49E-13	9.01E-13
SNAP47	738	579	-0.458781844	8.86E-09	1.12E-08
KIAA1107	739	225	-1.824373739	6.23E-73	1.88E-72
PTBP2	739	277	-1.524412764	6.50E-57	1.69E-56
RUNDC1	739	518	-0.621346642	3.51E-14	5.01E-14
SEMA4B	740	254	-1.65142115	8.94E-64	2.48E-63
NME3	740	541	-0.560621053	4.70E-12	6.42E-12
PSMB10	741	324	-1.302204105	2.37E-45	5.43E-45
PIBF1	741	439	-0.863976979	4.65E-24	7.93E-24
ADPRHL2	741	477	-0.744208652	5.73E-19	8.96E-19
GPX7	741	565	-0.499947051	4.51E-10	5.90E-10
HPS6	741	574	-0.477147182	2.31E-09	2.97E-09
AMPD3	742	529	-0.59687584	2.38E-13	3.35E-13
RNFT1	743	254	-1.65725809	2.43E-64	6.79E-64
FAM109A	743	319	-1.328530163	7.68E-47	1.79E-46
COIL	744	490	-0.711245248	1.04E-17	1.60E-17
GABPB1-AS1	745	237	-1.761077742	4.19E-70	1.23E-69
C1RL-AS1	747	173	-2.219060581	1.51E-94	5.56E-94
IFT20	747	477	-0.755843353	1.37E-19	2.17E-19
EPS8L2	747	591	-0.446674489	1.65E-08	2.08E-08
LEPR	748	782	-0.044594038	0.545389836	0.551177589
TAF3	749	360	-1.165693188	8.91E-39	1.88E-38
NDE1	749	441	-0.872911439	1.06E-24	1.83E-24
C15orf41	750	318	-1.346588206	3.22E-48	7.62E-48
IFIT1	751	66	-3.616999354	2.70E-154	1.55E-153
CCDC59	751	472	-0.778750424	1.20E-20	1.93E-20
C16orf13	752	700	-0.212102116	0.005090985	0.005510118
NOP9	753	434	-0.903679198	3.18E-26	5.61E-26
LIX1L	753	583	-0.477878357	1.63E-09	2.10E-09
MRPL9	754	473	-0.781448716	7.73E-21	1.25E-20

CDKN1C	755	357	-1.189276946	2.78E-40	5.94E-40
PAQR5	756	401	-1.023508374	5.22E-32	1.00E-31
KIAA1704	756	516	-0.659739545	6.26E-16	9.26E-16
HRAS	756	526	-0.632047811	7.13E-15	1.03E-14
FADD	756	552	-0.562442343	2.40E-12	3.29E-12
HSPA12A	756	592	-0.461513434	4.83E-09	6.17E-09
PRKAA2	757	263	-1.633954877	3.05E-64	8.50E-64
PHF11	757	278	-1.553932793	7.26E-60	1.94E-59
EXOSC1	757	710	-0.201198651	0.007560761	0.008138109
FAM35A	758	629	-0.377862207	1.13E-06	1.35E-06
MSRB2	759	418	-0.969321319	1.66E-29	3.07E-29
KIN	759	483	-0.760801072	4.36E-20	6.93E-20
PRPSAP2	759	603	-0.440666259	1.91E-08	2.40E-08
MTX1	759	637	-0.361530889	2.95E-06	3.50E-06
VTN	760	38	-4.430652471	2.00E-176	1.35E-175
SARM1	760	135	-2.601764387	2.26E-115	9.85E-115
TARBP1	760	374	-1.131685524	1.40E-37	2.91E-37
UTP18	761	463	-0.825608636	6.05E-23	1.01E-22
C11orf49	762	658	-0.32042779	2.91E-05	3.36E-05
ZNF768	763	469	-0.81081951	2.40E-22	3.96E-22
MAGOHB	765	408	-1.015614971	5.56E-32	1.07E-31
HMOX2	765	669	-0.302177913	7.41E-05	8.45E-05
GSKIP	767	421	-0.97413072	4.93E-30	9.24E-30
SYDE2	767	520	-0.66943933	1.60E-16	2.40E-16
PRMT7	768	377	-1.135266163	3.85E-38	8.03E-38
CRIP2	768	940	0.18283007	0.008974316	0.009627756
TXNDC17	768	853	0.042715055	0.551279309	0.556985396
ACVRL1	770	384	-1.112476511	4.61E-37	9.50E-37
PLA2G15	770	682	-0.283811082	0.000179655	0.000203057
ADAMTS14	770	744	-0.1582802	0.032753704	0.034545337
SPSB1	772	1465	0.815503536	7.28E-39	1.54E-38
MRPS11	774	517	-0.690893662	1.60E-17	2.44E-17

SDCCAG8	775	420	-0.992531358	3.11E-31	5.93E-31
GFER	775	443	-0.915613987	1.66E-27	2.99E-27
DNAJC4	775	558	-0.582655564	2.37E-13	3.33E-13
MGMT	776	442	-0.920734659	8.74E-28	1.58E-27
CRYZ	776	624	-0.423234999	4.40E-08	5.47E-08
GPRC5B	776	720	-0.216784122	0.003660512	0.003982298
COL24A1	777	159	-2.397612209	1.20E-107	4.93E-107
MYO19	777	443	-0.919332276	9.44E-28	1.71E-27
GEMIN4	777	465	-0.849408258	1.78E-24	3.05E-24
MRPS14	777	472	-0.827852115	1.71E-23	2.88E-23
MRPL20	778	505	-0.732211143	2.51E-19	3.95E-19
NFIA	780	74	-3.506601324	1.82E-156	1.06E-155
SMAD6	780	183	-2.200354851	1.23E-97	4.67E-97
SNIP1	780	470	-0.839537743	4.11E-24	7.02E-24
CHMP4A	780	520	-0.693686877	9.21E-18	1.41E-17
STX8	781	576	-0.547978113	3.44E-12	4.72E-12
VSIG10	782	194	-2.119836331	1.66E-93	6.05E-93
PELI2	783	241	-1.808703537	2.81E-76	8.75E-76
FAM53B	783	286	-1.561721536	2.68E-62	7.30E-62
ZNF195	783	315	-1.422384855	1.80E-54	4.56E-54
ACAD8	783	510	-0.727239436	3.14E-19	4.93E-19
C10orf32	785	303	-1.482099236	5.89E-58	1.54E-57
RPGRIP1L	785	580	-0.54536413	3.76E-12	5.14E-12
DEXI	785	621	-0.446823761	7.08E-09	9.02E-09
TSPAN11	786	195	-2.119779564	5.67E-94	2.08E-93
MAPK8	786	405	-1.06533178	2.39E-35	4.79E-35
SLC25A28	786	496	-0.772913568	2.81E-21	4.57E-21
VANGL2	787	365	-1.217191548	2.03E-43	4.53E-43
MAGI3	787	442	-0.941041642	3.83E-29	7.07E-29
TTC9C	790	609	-0.484134801	4.06E-10	5.32E-10
IMP3	791	586	-0.541501406	4.28E-12	5.85E-12
UVRAG	793	444	-0.945485565	1.43E-29	2.66E-29

COPRS	793	617	-0.470774752	1.03E-09	1.34E-09
NFYC	794	425	-1.010400542	7.29E-33	1.42E-32
CPT2	795	557	-0.622001909	3.63E-15	5.29E-15
KIAA1598	796	414	-1.051862039	4.47E-35	8.96E-35
DHX37	796	444	-0.95093313	6.01E-30	1.12E-29
SAMD14	796	450	-0.931567805	5.45E-29	1.00E-28
MRPL52	797	784	-0.132450446	0.067869128	0.070911162
RNASE4	798	658	-0.387025538	3.25E-07	3.97E-07
NCAPD3	799	391	-1.139751272	7.50E-40	1.60E-39
LIG3	799	614	-0.488681223	2.28E-10	3.01E-10
KIAA1217	800	454	-0.926032078	7.46E-29	1.37E-28
ISG20L2	800	585	-0.560287751	6.71E-13	9.31E-13
RIC8B	801	433	-0.996159594	2.07E-32	4.01E-32
HAGH	801	645	-0.421227458	3.07E-08	3.84E-08
HTR7P1	802	309	-1.484719774	2.53E-59	6.74E-59
H2AFY2	802	334	-1.37247851	6.62E-53	1.65E-52
MAP3K6	803	595	-0.541234695	3.02E-12	4.14E-12
C1orf86	804	561	-0.627919106	1.48E-15	2.17E-15
COG2	807	568	-0.61540212	4.12E-15	6.00E-15
BRMS1	807	579	-0.587729701	4.91E-14	6.98E-14
EDNRB	808	222	-1.972519992	3.79E-88	1.31E-87
LOC10013156	808	292	-1.5771113	3.98E-65	1.12E-64
ACTR6	808	491	-0.827356644	2.41E-24	4.14E-24
BCAS2	808	558	-0.642814547	3.16E-16	4.70E-16
TBKBP1	808	559	-0.640231386	4.02E-16	5.96E-16
DCUN1D2	809	449	-0.958148634	8.96E-31	1.70E-30
DDX10	809	537	-0.69994199	1.24E-18	1.93E-18
LYPLA2	809	693	-0.332008726	8.41E-06	9.85E-06
CEP95	812	358	-1.290244516	7.54E-49	1.80E-48
PRPF18	812	467	-0.906781553	2.65E-28	4.83E-28
ZBTB39	813	419	-1.065029484	1.73E-36	3.54E-36
NABP2	813	621	-0.49738646	7.99E-11	1.06E-10

FBXO44	814	461	-0.92898642	1.77E-29	3.29E-29
USPL1	814	528	-0.733215241	3.42E-20	5.45E-20
MANSC1	816	208	-2.08071	2.99E-95	1.11E-94
MESDC1	816	432	-1.026262216	1.47E-34	2.92E-34
CCDC91	816	435	-1.016278127	4.91E-34	9.71E-34
PDP2	816	737	-0.255628909	0.000482328	0.000538455
PAPD5	817	468	-0.912551924	9.35E-29	1.71E-28
MOAP1	817	481	-0.87302356	8.43E-27	1.50E-26
NDUFA12	817	781	-0.173737906	0.016057134	0.017113437
RASSF4	819	243	-1.861631514	8.05E-83	2.65E-82
LEO1	820	435	-1.023332885	1.45E-34	2.88E-34
DIEXF	821	367	-1.270326535	3.12E-48	7.41E-48
BCAS3	821	578	-0.615037105	2.48E-15	3.63E-15
CDK2	821	597	-0.568375666	1.66E-13	2.34E-13
SCAPER	822	413	-1.101720988	7.33E-39	1.55E-38
ERCC6	823	423	-1.068959143	3.97E-37	8.17E-37
CCS	825	555	-0.680630724	3.92E-18	6.06E-18
MTPAP	826	401	-1.151263921	8.86E-42	1.93E-41
DOK4	826	544	-0.711259506	1.73E-19	2.73E-19
LRRC32	827	1039	0.220512044	0.000995357	0.001101579
COA3	828	510	-0.807857897	5.68E-24	9.66E-24
CCSAP	830	423	-1.081178049	4.27E-38	8.91E-38
LUZP2	831	248	-1.853232732	1.67E-83	5.53E-83
TMEM198B	831	263	-1.768510053	1.99E-78	6.29E-78
ITFG2	831	372	-1.268270232	1.11E-48	2.66E-48
INTS2	832	498	-0.849162162	4.41E-26	7.76E-26
CTNNBIP1	832	713	-0.331405828	6.50E-06	7.63E-06
MIEN1	834	669	-0.426765549	1.07E-08	1.36E-08
ZW10	835	489	-0.880666108	9.75E-28	1.76E-27
CLK3	835	551	-0.708448255	1.47E-19	2.33E-19
LRRC37A4P	836	337	-1.419478727	7.32E-58	1.92E-57
CCNB1IP1	836	800	-0.172227318	0.015739231	0.016779202

KIAA1377	837	155	-2.541683783	1.15E-123	5.29E-123
HHAT	837	778	-0.214181843	0.00285247	0.003114505
ITGAE	839	632	-0.517470628	7.56E-12	1.03E-11
KIRREL3	841	374	-1.277791906	8.44E-50	2.04E-49
SEC61A2	841	385	-1.235971731	2.30E-47	5.40E-47
METTL17	841	444	-1.0302705	8.76E-36	1.77E-35
SUPT4H1	841	601	-0.593465185	8.58E-15	1.24E-14
SUPV3L1	842	449	-1.015829164	4.85E-35	9.70E-35
FAM76B	843	345	-1.397660645	5.05E-57	1.31E-56
TUBGCP4	844	423	-1.105309711	4.68E-40	1.00E-39
CASKIN2	844	442	-1.041921005	1.53E-36	3.13E-36
CCDC53	844	465	-0.968736659	1.36E-32	2.63E-32
SYNC	844	475	-0.938039861	5.64E-31	1.07E-30
MRPS23	844	534	-0.769127633	1.50E-22	2.48E-22
MRTO4	846	522	-0.805332232	2.46E-24	4.22E-24
UCHL5	846	551	-0.72732972	1.18E-20	1.90E-20
MKNK1	847	495	-0.88365782	2.90E-28	5.28E-28
LCMT1	847	646	-0.499552181	2.71E-11	3.63E-11
FBXO34	847	677	-0.431930512	5.62E-09	7.17E-09
ANGEL1	850	485	-0.91820247	3.82E-30	7.18E-30
RSAD1	851	520	-0.819371885	3.70E-25	6.43E-25
ELP5	851	658	-0.479795924	1.23E-10	1.63E-10
LOC146880	852	287	-1.678527069	8.10E-75	2.49E-74
CYB561D1	852	361	-1.347578969	1.36E-54	3.44E-54
FDX1	853	410	-1.165646208	6.46E-44	1.45E-43
ERAL1	853	594	-0.630807186	1.56E-16	2.34E-16
MFSD5	853	625	-0.557413928	1.52E-13	2.15E-13
POLR2M	855	441	-1.06387014	3.21E-38	6.71E-38
RECQL5	856	403	-1.195555334	8.35E-46	1.92E-45
PIGM	857	408	-1.179450428	6.46E-45	1.47E-44
AP5B1	857	482	-0.938986434	1.78E-31	3.39E-31
SHISA4	857	667	-0.470332819	2.28E-10	3.01E-10

UBIAD1	858	396	-1.224201593	1.37E-47	3.22E-47
INTS4	859	518	-0.838430409	2.47E-26	4.37E-26
PARG	860	487	-0.929139263	4.67E-31	8.86E-31
MTF2	860	553	-0.745781555	7.79E-22	1.28E-21
TRIM62	860	935	0.011905329	0.861235926	0.863447342
SLC26A11	861	363	-1.354768065	1.38E-55	3.53E-55
FBXW4	861	471	-0.979010554	8.97E-34	1.77E-33
GID4	863	502	-0.890397571	4.06E-29	7.48E-29
LRRC40	864	547	-0.768214855	5.29E-23	8.83E-23
TMEM104	864	884	-0.075709319	0.272465646	0.278529157
SLC24A6	865	352	-1.40584908	5.75E-59	1.52E-58
TMEM80	865	392	-1.250570854	1.55E-49	3.73E-49
LOC10012936	866	686	-0.444882824	1.40E-09	1.81E-09
PIK3CD	866	694	-0.428155738	5.06E-09	6.46E-09
JRKL	867	662	-0.497925152	1.82E-11	2.45E-11
FAM222B	868	592	-0.660822243	4.14E-18	6.39E-18
FBXL19	868	618	-0.59881258	1.93E-15	2.83E-15
MVD	869	850	-0.140617712	0.043251748	0.045464113
C1orf216	870	415	-1.17662844	2.10E-45	4.81E-45
TRIAP1	870	660	-0.507273752	7.56E-12	1.03E-11
CIAPIN1	870	665	-0.496385436	1.91E-11	2.58E-11
SIPA1	870	846	-0.149082114	0.032271635	0.034046096
H2AFX	871	496	-0.921056974	5.33E-31	1.01E-30
PIGQ	871	499	-0.912357279	1.55E-30	2.93E-30
TMEM126B	871	549	-0.774590945	1.73E-23	2.92E-23
IPO4	871	586	-0.68049643	4.74E-19	7.42E-19
CLCN6	871	613	-0.615510021	3.41E-16	5.07E-16
WDR20	872	634	-0.568569671	2.92E-14	4.18E-14
SYNGR2	873	1775	0.91504109	1.94E-56	5.00E-56
TOP3A	873	507	-0.892720283	1.47E-29	2.73E-29
PDXDC2P	874	468	-1.009849126	5.67E-36	1.15E-35
ASB7	874	546	-0.787456704	3.39E-24	5.79E-24

IKZF4	875	316	-1.578082834	1.96E-70	5.79E-70
ZBTB17	875	492	-0.939349077	4.02E-32	7.75E-32
SNAPIN	875	512	-0.881863583	4.75E-29	8.74E-29
FAM168A	876	627	-0.591189803	2.99E-15	4.38E-15
FAHD1	877	643	-0.556482481	7.60E-14	1.08E-13
MAPK7	878	528	-0.842407386	4.39E-27	7.86E-27
YPEL3	879	476	-0.993625968	2.94E-35	5.89E-35
ZMYM1	879	531	-0.83587568	8.91E-27	1.59E-26
F10	879	917	-0.047665807	0.483752436	0.4900276
DHX32	880	499	-0.927188084	1.23E-31	2.36E-31
ZFYVE27	881	545	-0.801610165	4.36E-25	7.56E-25
TRIM66	882	263	-1.854440232	1.37E-88	4.77E-88
THYN1	882	545	-0.803246802	3.39E-25	5.90E-25
TRIP4	882	576	-0.72343422	2.80E-21	4.55E-21
METTL7A	883	726	-0.391168266	5.73E-08	7.12E-08
USMG5	883	795	-0.260182953	0.00022195	0.000249886
LOC440300	884	283	-1.751968692	2.98E-82	9.76E-82
LRIF1	884	585	-0.704334121	2.03E-20	3.24E-20
KLHL28	884	635	-0.586014154	3.70E-15	5.39E-15
ZMYM6NB	885	400	-1.254401831	7.09E-51	1.73E-50
ZKSCAN2	885	429	-1.153424183	9.10E-45	2.07E-44
LSMD1	885	767	-0.315175253	9.18E-06	1.07E-05
PAK1	885	936	-0.027893301	0.679899103	0.684986723
VIPAS39	886	546	-0.807130124	1.68E-25	2.94E-25
IDH3A	886	596	-0.680718744	2.31E-19	3.64E-19
FIBIN	887	1590	0.73329638	2.94E-35	5.90E-35
C16orf52	887	603	-0.665500478	1.10E-18	1.71E-18
RPF1	887	773	-0.307190066	1.46E-05	1.70E-05
BPNT1	889	621	-0.626314526	5.73E-17	8.65E-17
PAR5	890	352	-1.446954283	3.17E-63	8.72E-63
RPP30	890	454	-1.079837414	1.20E-40	2.59E-40
COA4	890	644	-0.575469024	8.25E-15	1.19E-14

RRP12	890	683	-0.490644133	1.85E-11	2.50E-11
CELSR2	891	494	-0.959638766	8.34E-34	1.64E-33
TIMM22	891	565	-0.76589894	1.48E-23	2.50E-23
GATC	891	626	-0.61798715	1.22E-16	1.84E-16
DACT1	893	317	-1.602901711	1.91E-73	5.80E-73
LYRM1	893	440	-1.129881027	9.61E-44	2.15E-43
METTL3	893	536	-0.845151551	1.16E-27	2.09E-27
PGAP3	895	389	-1.310841903	6.01E-55	1.53E-54
NUP88	895	585	-0.722175434	1.65E-21	2.70E-21
KRT8	895	991	0.038272999	0.564686943	0.570310442
RNF34	896	485	-0.994238361	6.08E-36	1.23E-35
PEAR1	896	606	-0.672905314	3.36E-19	5.28E-19
TTC23	898	508	-0.930611324	1.95E-32	3.77E-32
UNC93B1	899	404	-1.262690199	3.61E-52	8.94E-52
SDHAF2	899	689	-0.492541509	1.24E-11	1.68E-11
C14orf1	899	759	-0.352945606	6.57E-07	7.93E-07
DHRS3	900	551	-0.816597059	2.28E-26	4.04E-26
LRRC8C	901	795	-0.289296622	3.68E-05	4.23E-05
RILPL1	903	436	-1.159122229	5.46E-46	1.26E-45
CROCC	904	375	-1.378156553	9.10E-60	2.43E-59
LRRC57	904	416	-1.22846362	2.60E-50	6.32E-50
DCP1B	906	418	-1.224732484	3.46E-50	8.42E-50
NIP7	906	580	-0.752182526	3.09E-23	5.19E-23
SYNE3	907	382	-1.356254288	1.48E-58	3.91E-58
MRPL27	907	639	-0.614010995	9.84E-17	1.48E-16
TCP11L2	908	356	-1.459539432	2.77E-65	7.79E-65
KRR1	908	389	-1.331646518	4.81E-57	1.25E-56
CCDC85C	908	712	-0.459539432	1.67E-10	2.21E-10
USP6NL	910	476	-1.043629348	2.29E-39	4.86E-39
ACSF2	910	519	-0.918856383	3.44E-32	6.64E-32
TCP11L1	911	792	-0.310675	9.01E-06	1.05E-05
CACHD1	914	269	-1.873312368	5.66E-93	2.06E-92

INPP5K	914	631	-0.643278536	3.56E-18	5.51E-18
POLR3GL	914	867	-0.184886548	0.006836266	0.007365398
AXIN1	915	483	-1.03047293	8.57E-39	1.81E-38
RAB7L1	916	473	-1.062231791	1.00E-40	2.16E-40
MLX	916	657	-0.588178604	9.55E-16	1.41E-15
DTD2	917	454	-1.122953812	1.90E-44	4.30E-44
PGS1	917	498	-0.989500367	1.81E-36	3.70E-36
DFFA	917	563	-0.812511187	1.28E-26	2.27E-26
KDELC1	917	913	-0.11503125	0.087991784	0.091568209
DRG2	919	581	-0.770251074	1.81E-24	3.11E-24
LINC00641	919	805	-0.299800454	1.62E-05	1.89E-05
CENPF	921	167	-2.57207743	1.16E-137	5.87E-137
BAMBI	921	176	-2.496350103	3.43E-133	1.68E-132
POLE	923	280	-1.829628197	5.71E-91	2.03E-90
PPCS	923	608	-0.7109837	1.41E-21	2.31E-21
RAB27A	924	422	-1.239374229	4.43E-52	1.10E-51
MIS12	924	485	-1.03863248	1.20E-39	2.56E-39
TNFAIP2	924	598	-0.736471743	7.19E-23	1.20E-22
SLC25A44	926	621	-0.685143301	2.23E-20	3.56E-20
DERA	926	678	-0.558451296	1.28E-14	1.85E-14
ARL4D	927	477	-1.067304449	1.68E-41	3.66E-41
RPS6KB2	927	755	-0.40481707	9.41E-09	1.19E-08
IGSF8	928	953	-0.070372967	0.28999307	0.29617524
C16orf87	928	1042	0.058434191	0.368942049	0.375432429
GPS2	929	492	-1.025744657	4.46E-39	9.43E-39
IKZF5	930	508	-0.981126595	1.81E-36	3.70E-36
C2CD3	930	1556	0.633815063	4.34E-27	7.78E-27
TTC8	931	501	-1.00269494	8.82E-38	1.83E-37
SLC16A4	932	400	-1.329054331	2.46E-58	6.49E-58
C12orf10	932	564	-0.833359168	3.73E-28	6.79E-28
TUBGCP3	933	486	-1.049645143	1.11E-40	2.38E-40
NIPA1	933	745	-0.433361031	8.50E-10	1.11E-09

R3HCC1L	934	466	-1.111816971	1.52E-44	3.44E-44
TRHDE-AS1	935	609	-0.727248513	1.18E-22	1.96E-22
CRY1	935	720	-0.485693834	9.02E-12	1.23E-11
ABCD4	936	393	-1.360703593	1.16E-60	3.12E-60
PLCE1	937	111	-3.186213747	3.48E-173	2.28E-172
UNC119	937	560	-0.851346597	2.75E-29	5.10E-29
MRPL10	938	623	-0.699080135	2.62E-21	4.25E-21
C14orf159	939	368	-1.460143767	1.72E-67	4.94E-67
CC2D1B	939	554	-0.869963557	2.20E-30	4.15E-30
C11orf84	939	569	-0.831420881	3.01E-28	5.49E-28
TSR3	939	643	-0.655030796	3.47E-19	5.44E-19
KRT10	939	1035	0.031709329	0.625434422	0.630765517
TMEM51	941	298	-1.767606768	1.70E-88	5.89E-88
RPAP2	941	349	-1.539692062	5.28E-73	1.60E-72
ZNF33B	942	186	-2.449148814	2.76E-133	1.35E-132
ZNF436	942	537	-0.919529348	2.66E-33	5.20E-33
CASP7	942	551	-0.882399117	3.55E-31	6.75E-31
KIAA1731	944	338	-1.590487989	1.09E-76	3.42E-76
ENGASE	946	388	-1.394507907	1.48E-63	4.10E-63
ASUN	946	812	-0.329084832	1.78E-06	2.13E-06
SLC43A2	947	140	-2.866661974	1.87E-158	1.11E-157
AHNAK2	948	1838	0.846453427	2.23E-51	5.47E-51
LZIC	948	480	-1.090577029	7.48E-44	1.68E-43
ZBTB8A	948	481	-1.087574541	1.15E-43	2.58E-43
CTAGE5	948	795	-0.362656575	1.61E-07	1.98E-07
CYB5R1	949	714	-0.519208389	2.79E-13	3.92E-13
KIAA0586	950	427	-1.262415819	5.23E-55	1.33E-54
TBL3	950	799	-0.358456386	2.13E-07	2.61E-07
STAMBPL1	951	340	-1.592634971	2.17E-77	6.83E-77
IPP	951	469	-1.128581794	2.18E-46	5.06E-46
NOP2	951	604	-0.763621167	6.51E-25	1.13E-24
SPSB3	954	545	-0.916457412	1.59E-33	3.12E-33

SLCO3A1	955	250	-2.042297014	1.46E-108	6.04E-108
CCP110	955	415	-1.311113773	1.58E-58	4.17E-58
RBM14	955	483	-1.09220192	2.89E-44	6.52E-44
NFKB2	955	890	-0.210419773	0.001732421	0.001905151
TVP23B	955	993	-0.052431391	0.422412682	0.428392291
PRPF38A	956	558	-0.885469872	8.60E-32	1.65E-31
TAF5L	956	603	-0.773576992	1.45E-25	2.53E-25
SMIM12	956	676	-0.608711748	2.62E-17	4.00E-17
NLRC5	960	475	-1.123831268	1.66E-46	3.87E-46
MFSD11	960	498	-1.055613039	3.50E-42	7.68E-42
FBRSL1	960	533	-0.957623249	3.60E-36	7.31E-36
POLR3E	960	689	-0.587254799	2.18E-16	3.27E-16
RGCC	960	827	-0.323871452	2.14E-06	2.55E-06
ZCCHC8	961	611	-0.762088427	4.48E-25	7.77E-25
ADAT1	962	462	-1.166868418	2.21E-49	5.31E-49
SLC38A7	962	683	-0.602875691	3.90E-17	5.92E-17
HSPB8	963	576	-0.850191362	5.49E-30	1.03E-29
ALG8	964	808	-0.363402229	1.20E-07	1.48E-07
PTS	964	945	-0.137443193	0.037344382	0.039318091
SCARB1	966	875	-0.251464548	0.000185114	0.000208956
ATP6V0A1	966	915	-0.186975822	0.004938788	0.005350582
WDR61	967	789	-0.402214965	5.59E-09	7.14E-09
NOC3L	968	536	-0.961498423	1.08E-36	2.22E-36
GDAP2	969	635	-0.71846445	5.83E-23	9.72E-23
PHLPP2	970	486	-1.105752809	8.37E-46	1.93E-45
LRRC37BP1	970	507	-1.044723376	6.46E-42	1.41E-41
SIVA1	970	567	-0.883360388	4.14E-32	7.98E-32
CCDC43	970	709	-0.560923496	2.37E-15	3.48E-15
SMAP2	971	708	-0.564446311	1.60E-15	2.35E-15
UNG	972	447	-1.229405858	5.03E-54	1.27E-53
HAUS2	972	559	-0.906832407	1.49E-33	2.92E-33
WDR77	973	634	-0.726681341	1.76E-23	2.97E-23

KRT18	974	1618	0.623493554	2.44E-27	4.39E-27
AMBRA1	974	612	-0.779114495	2.55E-26	4.51E-26
KIAA0895L	975	282	-1.898431432	8.29E-101	3.22E-100
BBS4	975	506	-1.05498921	8.95E-43	1.98E-42
NHLRC3	976	507	-1.053619776	9.93E-43	2.20E-42
COQ5	976	614	-0.777366868	2.83E-26	5.00E-26
TMEM120B	976	935	-0.170639159	0.009711126	0.010408205
ALPL	978	289	-1.867489348	6.11E-99	2.34E-98
H2AFJ	978	809	-0.382419138	2.24E-08	2.81E-08
TMEM19	979	440	-1.262529712	1.22E-56	3.15E-56
GPR137	979	595	-0.827143567	3.89E-29	7.18E-29
AACS	979	1047	-0.011843699	0.85340449	0.855815547
VPS33A	980	632	-0.741581567	1.97E-24	3.39E-24
THAP11	981	523	-1.016166566	1.46E-40	3.13E-40
PPP2R2D	982	716	-0.564487813	1.10E-15	1.63E-15
TRIM68	983	357	-1.569991718	2.52E-78	7.97E-78
MRPL16	983	715	-0.567972551	7.42E-16	1.10E-15
EXD2	984	552	-0.942714424	4.06E-36	8.24E-36
TMEM135	984	611	-0.796210311	1.59E-27	2.87E-27
CCNDBP1	984	864	-0.296351379	1.02E-05	1.19E-05
ATF1	984	868	-0.289687649	1.57E-05	1.83E-05
GPATCH2L	986	555	-0.937824251	6.88E-36	1.39E-35
FAM160A2	988	515	-1.048662985	6.48E-43	1.44E-42
NDNL2	988	665	-0.679881077	2.29E-21	3.73E-21
GOLPH3L	988	720	-0.565238511	8.36E-16	1.24E-15
DDIT3	989	680	-0.649160151	7.87E-20	1.25E-19
NRBF2	989	961	-0.150158466	0.021514883	0.022824307
FAM171A2	990	364	-1.552214451	1.34E-77	4.21E-77
UNK	990	506	-1.077015516	7.83E-45	1.78E-44
STRADA	990	634	-0.751670061	3.27E-25	5.69E-25
RPAIN	990	713	-0.582250824	1.28E-16	1.93E-16
MARCH8	991	423	-1.33695177	1.84E-62	5.02E-62

INPP5A	991	679	-0.654197859	4.04E-20	6.43E-20
HOXB2	992	917	-0.222142763	0.000768812	0.000854613
TSSC4	994	741	-0.532496685	2.03E-14	2.91E-14
ETNK2	994	764	-0.488397589	1.56E-12	2.15E-12
GNPTG	994	817	-0.391634149	8.19E-09	1.04E-08
KCTD11	994	856	-0.324359431	1.36E-06	1.63E-06
ZNF496	995	645	-0.734121741	2.24E-24	3.85E-24
SNN	996	320	-1.746798213	4.63E-92	1.67E-91
ST7L	996	406	-1.403390391	1.91E-67	5.48E-67
KIF7	996	501	-1.100059515	1.31E-46	3.06E-46
CCL2	997	387	-1.473984314	1.56E-72	4.70E-72
RAB2B	998	690	-0.64116783	1.35E-19	2.14E-19
TAF12	998	693	-0.634908839	2.77E-19	4.36E-19
KLHL36	998	738	-0.544143375	5.45E-15	7.92E-15
KCTD21	999	707	-0.607498839	5.87E-18	9.04E-18
TRMT5	1000	499	-1.111612655	1.48E-47	3.49E-47
DDX20	1000	558	-0.950387349	3.73E-37	7.69E-37
CLUAP1	1001	400	-1.432094445	8.07E-70	2.37E-69
ING4	1001	499	-1.113054629	1.07E-47	2.52E-47
CRTC2	1001	517	-1.061930164	2.54E-44	5.73E-44
TP53I13	1001	772	-0.483493597	2.08E-12	2.87E-12
GOT1	1001	917	-0.235172711	0.000358153	0.000401035
SMYD2	1003	644	-0.747913388	2.58E-25	4.50E-25
TMEM258	1003	847	-0.352612107	1.53E-07	1.88E-07
PIAS1	1004	509	-1.088746084	3.23E-46	7.48E-46
FAM175B	1004	672	-0.687950507	4.15E-22	6.84E-22
TAF13	1004	1019	-0.087329594	0.173259356	0.17851726
ACP2	1005	553	-0.970568492	1.33E-38	2.80E-38
C11orf54	1005	666	-0.702325795	6.87E-23	1.14E-22
COX11	1005	753	-0.525198107	3.07E-14	4.39E-14
SYT15	1006	507	-1.097297029	7.11E-47	1.66E-46
RHOG	1006	661	-0.714632504	1.44E-23	2.43E-23

KLC2	1006	694	-0.644347113	6.70E-20	1.06E-19
DHTKD1	1006	926	-0.228270582	0.000506702	0.000565343
RNF121	1007	873	-0.3147345	2.30E-06	2.74E-06
VASH2	1008	176	-2.626572681	6.15E-154	3.51E-153
ARNTL2	1009	909	-0.259298351	8.34E-05	9.51E-05
CKB	1010	714	-0.609083689	3.24E-18	5.02E-18
CYB5D2	1011	549	-0.989629319	4.87E-40	1.04E-39
VAC14	1012	653	-0.740778769	3.94E-25	6.84E-25
LOC10050639	1012	911	-0.260410707	7.61E-05	8.68E-05
BCL10	1013	768	-0.508180334	1.36E-13	1.92E-13
DPH1	1014	478	-1.193699505	8.40E-54	2.11E-53
RASL10B	1015	302	-1.857583649	6.90E-102	2.71E-101
HOXB4	1015	461	-1.247365448	1.37E-57	3.57E-57
ACAD10	1015	480	-1.189097792	1.55E-53	3.89E-53
FAM96A	1015	720	-0.604135292	4.68E-18	7.23E-18
USP3	1016	538	-1.0259467	1.38E-42	3.05E-42
SLC39A11	1016	634	-0.789070033	5.91E-28	1.07E-27
MRPL38	1016	655	-0.742057966	2.70E-25	4.70E-25
LMBR1L	1016	700	-0.646197951	3.54E-20	5.65E-20
CIB1	1017	1162	0.083566014	0.176711783	0.182050437
MFAP2	1019	1658	0.593565579	9.33E-26	1.63E-25
SLC30A1	1020	438	-1.328290753	1.29E-63	3.57E-63
TYW3	1020	541	-1.023593029	1.36E-42	3.00E-42
TINF2	1020	723	-0.605225976	3.43E-18	5.31E-18
BCCIP	1021	702	-0.649164306	2.02E-20	3.23E-20
PPP2R5B	1021	781	-0.495312789	3.95E-13	5.51E-13
CKAP2	1023	464	-1.249333811	3.60E-58	9.46E-58
C12orf44	1024	1190	0.108021482	0.078451185	0.08179243
MIS18BP1	1025	277	-1.996390404	4.82E-113	2.06E-112
USF1	1025	486	-1.185320067	8.80E-54	2.22E-53
DEAF1	1025	541	-1.030647786	2.93E-43	6.53E-43
CDAN1	1027	385	-1.524230207	2.32E-78	7.32E-78

CMAS	1027	629	-0.816028635	8.02E-30	1.50E-29
ZNF259	1027	977	-0.18073009	0.005037678	0.00545469
TRIM65	1028	594	-0.900029804	5.79E-35	1.16E-34
AKIP1	1028	832	-0.413909207	6.75E-10	8.81E-10
LRCH1	1029	516	-1.104524387	2.13E-48	5.06E-48
FRMD8	1029	680	-0.706360707	1.29E-23	2.17E-23
RNF214	1030	536	-1.051063808	7.97E-45	1.81E-44
TERF2	1031	608	-0.87062548	3.09E-33	6.04E-33
SORL1	1032	225	-2.30617044	5.26E-136	2.63E-135
C1orf85	1032	970	-0.198110694	0.002122469	0.00232688
PDCD7	1033	608	-0.873421401	1.81E-33	3.54E-33
PIDD	1036	563	-0.988541551	6.49E-41	1.40E-40
BCAR3	1037	688	-0.7006598	1.78E-23	3.00E-23
PEX11B	1037	774	-0.530734799	6.75E-15	9.79E-15
MLH3	1038	422	-1.407215916	1.71E-70	5.06E-70
POMT2	1038	711	-0.654609355	5.08E-21	8.22E-21
HMGCL	1040	727	-0.625280635	1.56E-19	2.46E-19
SLC25A11	1040	818	-0.455135156	1.22E-11	1.66E-11
IGFBP6	1040	1362	0.280418799	2.10E-06	2.51E-06
ENDOD1	1041	485	-1.210637792	2.16E-56	5.58E-56
B3GALT2	1042	2264	1.010794305	4.87E-84	1.62E-83
ARL14EP	1044	585	-0.944337558	2.54E-38	5.31E-38
MON1B	1044	652	-0.787902218	1.31E-28	2.39E-28
EXOC8	1045	720	-0.646158507	1.05E-20	1.69E-20
DCUN1D3	1045	732	-0.622311765	1.82E-19	2.86E-19
CTPS1	1045	1315	0.222835481	0.00018338	0.000207058
EMC8	1046	770	-0.550676877	6.03E-16	8.93E-16
MTMR10	1047	704	-0.681338484	1.26E-22	2.09E-22
COG7	1047	727	-0.634958549	3.72E-20	5.93E-20
ADCY7	1048	460	-1.296657326	5.44E-63	1.49E-62
PLEKHG5	1048	659	-0.778012722	3.95E-28	7.19E-28
MAP2K5	1049	600	-0.914704648	1.40E-36	2.87E-36

RFWD3	1050	544	-1.057435147	4.26E-46	9.85E-46
NAA40	1050	552	-1.036373532	1.16E-44	2.64E-44
TM2D3	1050	662	-0.774210582	5.89E-28	1.07E-27
CNEP1R1	1050	731	-0.631170392	5.19E-20	8.24E-20
RSBN1	1052	672	-0.755325942	6.69E-27	1.20E-26
ZNF263	1052	691	-0.715101465	1.34E-24	2.31E-24
NDUFB1	1055	1045	-0.122464433	0.051688994	0.054216166
METTL15	1056	658	-0.791174722	4.08E-29	7.52E-29
ECD	1056	738	-0.625641489	7.91E-20	1.25E-19
TRAF6	1057	610	-0.901818605	5.13E-36	1.04E-35
YIPF1	1057	626	-0.86446519	1.21E-33	2.38E-33
SALL1	1057	1630	0.516172212	4.76E-20	7.56E-20
NAT9	1057	987	-0.207577763	0.001142793	0.001262781
LACC1	1058	390	-1.548517974	1.61E-82	5.29E-82
IFI6	1058	663	-0.782983228	1.12E-28	2.05E-28
TBC1D24	1058	972	-0.231035784	0.000309285	0.000347162
PACSIN3	1059	586	-0.962454395	4.82E-40	1.03E-39
NOP10	1059	986	-0.211767414	0.000902865	0.001001346
RMDN3	1060	594	-0.944253805	7.08E-39	1.49E-38
IPO13	1060	595	-0.941827067	1.02E-38	2.15E-38
ACADSB	1060	630	-0.859364907	2.06E-33	4.02E-33
ACOT7	1060	806	-0.503936897	5.82E-14	8.25E-14
RANBP10	1062	596	-0.942123906	8.32E-39	1.76E-38
IPMK	1065	330	-1.799039877	2.54E-102	1.00E-101
IRF9	1065	474	-1.276618842	1.70E-62	4.64E-62
SMG8	1065	624	-0.879959872	7.17E-35	1.43E-34
C1orf52	1066	773	-0.572391495	2.77E-17	4.22E-17
ALG10B	1067	508	-1.17938415	1.76E-55	4.50E-55
RHNO1	1067	569	-1.015783994	6.11E-44	1.38E-43
APOPT1	1067	846	-0.443554984	2.03E-11	2.74E-11
ZMYM6	1068	676	-0.768540871	4.55E-28	8.28E-28
MTHFD1	1068	700	-0.718209196	3.98E-25	6.92E-25

CHPT1	1069	580	-0.990861424	2.55E-42	5.63E-42
MEN1	1069	620	-0.894646108	6.10E-36	1.24E-35
PPIE	1069	693	-0.734058971	4.60E-26	8.10E-26
CIRH1A	1071	670	-0.785449855	3.69E-29	6.82E-29
NOB1	1073	765	-0.596842799	1.27E-18	1.97E-18
NDUFS3	1074	743	-0.640284253	6.41E-21	1.03E-20
PRKAB1	1075	631	-0.877349125	5.16E-35	1.03E-34
SLIRP	1076	986	-0.234742902	0.000220793	0.000248619
CENPT	1077	553	-1.07039124	3.78E-48	8.94E-48
GLIS2	1077	755	-0.621194076	5.89E-20	9.36E-20
TUBGCP5	1078	498	-1.222863907	3.10E-59	8.26E-59
LLPH	1078	723	-0.685014002	1.87E-23	3.15E-23
PPT1	1078	1048	-0.149442837	0.016899068	0.017998475
FAN1	1079	581	-1.001809172	1.89E-43	4.22E-43
NFKBIA	1079	642	-0.857774038	7.02E-34	1.38E-33
ARL3	1079	821	-0.502965114	3.85E-14	5.50E-14
TRAF4	1080	897	-0.376575798	6.93E-09	8.82E-09
SRSF8	1081	892	-0.385975284	3.02E-09	3.88E-09
SREBF1	1082	1155	-0.014532023	0.811698541	0.814828886
UNKL	1084	373	-1.647841597	2.86E-92	1.03E-91
MKI67	1085	389	-1.588577358	1.04E-87	3.58E-87
DDX54	1085	596	-0.973035183	1.05E-41	2.29E-41
LPCAT2	1085	702	-0.736876483	1.35E-26	2.40E-26
DPAGT1	1085	920	-0.346713652	7.73E-08	9.56E-08
TMEM48	1087	408	-1.522435259	1.09E-82	3.59E-82
PITHD1	1087	750	-0.644113816	2.31E-21	3.76E-21
WDR3	1088	913	-0.361716167	2.15E-08	2.71E-08
ATG2A	1089	433	-1.4392894	2.37E-76	7.37E-76
TCTN1	1089	502	-1.225969061	4.72E-60	1.27E-59
ACADM	1090	609	-0.948538378	3.23E-40	6.90E-40
MPC2	1090	900	-0.385055604	2.82E-09	3.62E-09
FKBP2	1090	1028	-0.193212246	0.002054479	0.00225424

DHDDS	1092	998	-0.238585511	0.000157006	0.000177665
ASCC1	1093	569	-1.050517219	2.02E-47	4.75E-47
UBE2O	1093	631	-0.901305867	3.76E-37	7.75E-37
BTBD6	1093	718	-0.714962028	1.74E-25	3.03E-25
SOCS7	1094	514	-1.198496849	2.91E-58	7.67E-58
MAPKBP1	1095	768	-0.62047703	3.18E-20	5.06E-20
SENP1	1096	522	-1.178850462	6.70E-57	1.74E-56
ZNF646	1096	652	-0.858028305	2.10E-34	4.18E-34
MRPL23	1097	1241	0.069215214	0.246357022	0.252235739
SNRNP40	1098	651	-0.862872982	8.86E-35	1.77E-34
QPRT	1099	490	-1.274062108	3.08E-64	8.56E-64
TFAM	1099	499	-1.247804042	3.06E-62	8.35E-62
METTL13	1099	552	-1.10217559	2.13E-51	5.23E-51
ZDHHC6	1099	589	-1.008576223	1.07E-44	2.42E-44
ATPAF1	1100	675	-0.813268492	1.20E-31	2.29E-31
EARS2	1100	801	-0.566353752	1.77E-17	2.71E-17
ABCC9	1101	228	-2.380433115	1.89E-150	1.05E-149
NEDD8	1101	1139	-0.059771098	0.326728794	0.333084224
IFI27L2	1101	1190	0.003422729	0.954721609	0.955823211
UEVLD	1102	644	-0.883716006	2.85E-36	5.80E-36
ABLIM1	1102	792	-0.585276264	1.77E-18	2.74E-18
CLK2	1104	533	-1.15925711	7.72E-56	1.98E-55
C10orf137	1104	597	-0.995661711	5.52E-44	1.24E-43
VPS25	1104	870	-0.452377242	3.82E-12	5.22E-12
EBPL	1106	756	-0.657617622	1.84E-22	3.05E-22
CBX4	1106	793	-0.58868299	1.02E-18	1.59E-18
ARHGAP11A	1107	318	-1.908280927	5.67E-115	2.46E-114
PFDN2	1109	1090	-0.133655606	0.029782352	0.03144968
ARHGAP32	1110	478	-1.324201529	9.52E-69	2.77E-68
C17orf80	1110	647	-0.887446435	9.01E-37	1.85E-36
TTC19	1111	624	-0.940965258	1.95E-40	4.18E-40
DYRK2	1113	783	-0.616093756	2.71E-20	4.33E-20

ATHL1	1114	621	-0.951808435	2.72E-41	5.91E-41
SRSF9	1114	848	-0.502337439	1.61E-14	2.31E-14
ZC3H12C	1115	357	-1.751772107	3.14E-103	1.24E-102
SLC2A13	1117	659	-0.870003191	7.97E-36	1.61E-35
TMEM136	1118	548	-1.137396766	7.01E-55	1.78E-54
SDE2	1118	940	-0.358911902	1.76E-08	2.21E-08
WWP2	1119	547	-1.141321674	3.19E-55	8.14E-55
PAGR1	1119	559	-1.110014224	6.97E-53	1.73E-52
FADS3	1119	1586	0.394458359	1.67E-12	2.31E-12
NECAP1	1119	948	-0.347975448	4.36E-08	5.43E-08
MIPEP	1120	247	-2.289640161	3.54E-146	1.92E-145
CCDC82	1120	481	-1.328114309	1.17E-69	3.43E-69
BIVM	1120	618	-0.966544365	1.55E-42	3.42E-42
PLD2	1124	577	-1.070723187	3.38E-50	8.22E-50
DDB2	1125	701	-0.791163028	6.57E-31	1.25E-30
MRPL17	1125	1092	-0.151676521	0.013288639	0.014191874
TLCD2	1126	399	-1.605470552	2.52E-92	9.13E-92
GLOD4	1126	624	-0.960313269	2.57E-42	5.67E-42
MRPL28	1128	983	-0.307228122	1.02E-06	1.22E-06
NDST2	1129	574	-1.08464722	1.91E-51	4.70E-51
RPAP1	1129	646	-0.914163792	3.35E-39	7.09E-39
FBXO42	1129	714	-0.769773883	1.18E-29	2.20E-29
BRF1	1130	671	-0.860662477	1.35E-35	2.73E-35
B3GNT1	1131	668	-0.868403297	3.82E-36	7.75E-36
VPS37B	1131	939	-0.377126242	2.92E-09	3.75E-09
ENTPD1	1132	479	-1.349500773	4.36E-72	1.31E-71
RPAP3	1132	670	-0.865365333	5.70E-36	1.15E-35
RBM34	1133	872	-0.486472197	5.48E-14	7.77E-14
TNRC6C	1134	627	-0.963607668	7.74E-43	1.72E-42
NTPCR	1136	516	-1.24724424	3.14E-64	8.73E-64
SNHG1	1136	582	-1.073596152	6.28E-51	1.54E-50
RAVER2	1137	533	-1.201749192	9.84E-61	2.66E-60

RGS5	1138	133	-3.205726783	5.02E-211	4.10E-210
ZMYM5	1138	577	-1.08858171	3.89E-52	9.63E-52
FAM57A	1138	764	-0.68358039	1.39E-24	2.39E-24
SH3BP5L	1138	939	-0.386027871	1.16E-09	1.51E-09
FLJ42627	1139	773	-0.667951804	1.10E-23	1.86E-23
NTAN1	1139	820	-0.582796308	6.49E-19	1.01E-18
GPRC5A	1140	371	-1.728267108	1.45E-103	5.77E-103
PUS7L	1140	697	-0.818527639	4.40E-33	8.56E-33
CCBL2	1140	720	-0.771689389	4.76E-30	8.92E-30
TXNDC11	1140	1146	-0.101151156	0.093589073	0.097250442
BBS1	1141	450	-1.451026261	7.28E-81	2.36E-80
ZDHHC16	1141	960	-0.357916857	1.37E-08	1.73E-08
FAM60A	1142	772	-0.673614274	4.47E-24	7.63E-24
MINOS1	1142	779	-0.660591793	2.59E-23	4.34E-23
ITPRIP	1143	710	-0.79565885	1.14E-31	2.19E-31
BAIAP2-AS1	1144	607	-1.023043006	1.62E-47	3.80E-47
WBP4	1144	607	-1.023043006	1.62E-47	3.80E-47
ALG9	1144	712	-0.792862282	1.64E-31	3.12E-31
ARL8A	1144	721	-0.774740263	2.41E-30	4.54E-30
COL13A1	1145	1332	0.109522108	0.05913896	0.061930554
ERCC4	1152	701	-0.825378743	7.18E-34	1.41E-33
TTBK2	1153	727	-0.77408962	1.58E-30	2.98E-30
PAN2	1154	422	-1.560052696	8.58E-91	3.04E-90
MUL1	1154	695	-0.840282717	6.28E-35	1.25E-34
TSKU	1154	833	-0.578979199	6.17E-19	9.64E-19
DIABLO	1154	844	-0.560052696	6.32E-18	9.73E-18
NEURL4	1155	565	-1.140294455	7.33E-57	1.90E-56
ZNF25	1156	620	-1.007525653	7.57E-47	1.77E-46
RUSC1	1156	628	-0.989029309	1.74E-45	3.99E-45
RRP15	1158	619	-1.012348315	2.78E-47	6.51E-47
C1orf123	1158	740	-0.754762453	2.08E-29	3.85E-29
B3GNT9	1158	834	-0.58224034	3.57E-19	5.60E-19

FBXO3	1159	534	-1.226693295	7.47E-64	2.07E-63
IFFO1	1159	569	-1.135104385	1.19E-56	3.09E-56
STX4	1160	769	-0.701793678	3.89E-26	6.86E-26
MRE11A	1162	498	-1.331116797	1.91E-72	5.74E-72
C1orf27	1163	618	-1.020896729	4.07E-48	9.64E-48
POLR3C	1165	627	-1.002516983	7.95E-47	1.86E-46
CHTOP	1166	741	-0.762746717	4.08E-30	7.66E-30
C10orf118	1166	798	-0.655831513	1.73E-23	2.92E-23
MRPL45	1167	697	-0.852298376	3.98E-36	8.07E-36
PITPNM2	1169	287	-2.134876664	4.86E-140	2.52E-139
PTCHD3P1	1170	598	-1.077015516	1.16E-52	2.88E-52
C12orf49	1170	719	-0.81116923	2.05E-33	4.02E-33
FHL3	1170	1140	-0.146199081	0.014846464	0.015840421
KCNH1	1172	2259	0.837987325	1.07E-61	2.91E-61
FBXO31	1172	594	-1.08916211	1.10E-53	2.76E-53
KCTD5	1173	941	-0.426660761	1.20E-11	1.62E-11
ZNF268	1178	509	-1.319326354	1.92E-72	5.77E-72
TRPC6	1179	652	-0.963344225	1.90E-44	4.29E-44
PYCR2	1179	737	-0.78655157	5.27E-32	1.01E-31
NBPF14	1180	369	-1.785818514	4.92E-112	2.09E-111
RCC1	1180	663	-0.94043046	8.42E-43	1.87E-42
STARD3	1181	778	-0.71089128	3.77E-27	6.77E-27
CTBS	1182	1040	-0.293370883	1.67E-06	2.00E-06
FBXO22	1183	775	-0.718906234	1.06E-27	1.92E-27
PDE4B	1185	217	-2.557844487	1.81E-175	1.21E-174
RBM19	1185	658	-0.957451946	3.14E-44	7.08E-44
PGM2L1	1185	682	-0.905767791	1.87E-40	4.02E-40
FDXR	1185	869	-0.556183353	3.72E-18	5.74E-18
CAMK2G	1187	937	-0.449923358	7.83E-13	1.09E-12
FRA10AC1	1189	703	-0.866876497	8.39E-38	1.74E-37
NUP107	1190	609	-1.075171816	2.18E-53	5.46E-53
PRPSAP1	1190	770	-0.736755599	5.31E-29	9.77E-29

ZNF248	1191	754	-0.76826136	4.32E-31	8.21E-31
TMEM222	1192	650	-0.983596988	1.94E-46	4.51E-46
FAM149B1	1194	708	-0.862705947	1.17E-37	2.42E-37
BDKRB1	1195	791	-0.703985394	5.16E-27	9.23E-27
OAF	1195	838	-0.620712845	6.11E-22	1.00E-21
PHTF1	1195	888	-0.537103412	2.85E-17	4.35E-17
DNAJC1	1197	883	-0.547662185	7.31E-18	1.12E-17
ATP10A	1198	1753	0.440473712	2.01E-16	3.00E-16
MED21	1199	730	-0.824587666	3.97E-35	7.95E-35
TMEM179B	1199	888	-0.541924453	1.40E-17	2.14E-17
PPME1	1200	6512	2.331341918	0	0
DPH5	1200	722	-0.841688039	2.37E-36	4.83E-36
CNIH4	1200	815	-0.666886817	8.42E-25	1.46E-24
C11orf73	1201	852	-0.604035191	4.53E-21	7.33E-21
MPDU1	1205	842	-0.625865384	2.05E-22	3.39E-22
ALG5	1205	868	-0.581990575	7.12E-20	1.13E-19
NSL1	1206	559	-1.218034095	1.19E-65	3.36E-65
CHFR	1206	834	-0.640834994	2.51E-23	4.21E-23
AAAS	1206	867	-0.584850385	4.74E-20	7.53E-20
UTP6	1206	893	-0.542222203	1.09E-17	1.66E-17
ZNF23	1207	836	-0.638575205	3.29E-23	5.51E-23
VPS72	1207	912	-0.513044322	3.70E-16	5.49E-16
HSPA2	1208	1192	-0.127960595	0.029749769	0.031419523
GIT1	1209	635	-1.037710123	2.93E-51	7.18E-51
EDC3	1209	841	-0.632360915	7.15E-23	1.19E-22
KATNBL1	1209	967	-0.430950826	3.70E-12	5.07E-12
MRPL43	1210	976	-0.41877837	1.30E-11	1.76E-11
ORAI3	1211	666	-0.971329159	3.25E-46	7.53E-46
EHD4	1211	1086	-0.265899138	1.01E-05	1.18E-05
FAM96B	1211	1145	-0.189575643	0.001424254	0.001570682
C12orf4	1213	614	-1.090993366	1.20E-55	3.08E-55
MED17	1213	735	-0.831487771	5.27E-36	1.07E-35

CDR2L	1213	1170	-0.160795397	0.006504103	0.007013331
BRAP	1214	706	-0.890752709	2.69E-40	5.75E-40
NUDT5	1214	881	-0.571278873	2.10E-19	3.31E-19
PTPRU	1216	672	-0.964334467	7.29E-46	1.68E-45
FAM179B	1219	547	-1.264809764	2.87E-70	8.47E-70
USB1	1219	955	-0.460849864	1.15E-13	1.63E-13
PLEKHA6	1220	274	-2.263357725	7.20E-157	4.20E-156
DLG4	1220	750	-0.810643023	9.87E-35	1.97E-34
MRPL37	1220	1057	-0.315630147	1.84E-07	2.26E-07
PPIF	1220	1208	-0.122985069	0.035635862	0.037544583
SLC35B1	1221	913	-0.528100811	3.97E-17	6.02E-17
MAP2K1	1221	1127	-0.224300061	0.000165381	0.000187032
GBP2	1222	432	-1.608865444	2.48E-100	9.61E-100
ASB8	1222	648	-1.023902943	1.05E-50	2.57E-50
LRRC42	1222	775	-0.765700446	1.11E-31	2.12E-31
INPP5B	1224	604	-1.127707479	4.05E-59	1.08E-58
AIP	1224	802	-0.718653792	1.38E-28	2.53E-28
COG1	1225	1098	-0.266628071	8.50E-06	9.95E-06
ARFIP2	1226	1077	-0.295665105	8.91E-07	1.07E-06
PLEKHM1	1227	705	-0.908164462	5.27E-42	1.15E-41
BTBD10	1227	827	-0.67790039	5.11E-26	8.99E-26
TAF1C	1228	471	-1.491235972	2.03E-90	7.16E-90
SCO1	1229	680	-0.96260264	3.34E-46	7.74E-46
NENF	1230	864	-0.618279474	2.17E-22	3.59E-22
MARK2	1231	658	-1.012395649	3.73E-50	9.04E-50
VTI1A	1231	671	-0.984170466	6.05E-48	1.43E-47
IFIT5	1233	515	-1.368252838	5.55E-80	1.78E-79
ATXN3	1234	441	-1.593216209	6.64E-100	2.56E-99
CARS2	1234	904	-0.557672093	6.31E-19	9.86E-19
FUT11	1237	957	-0.478979046	9.43E-15	1.36E-14
MTF1	1239	716	-0.899869071	8.95E-42	1.95E-41
TSR1	1239	758	-0.81763081	9.59E-36	1.94E-35

GGA3	1240	745	-0.843752166	1.18E-37	2.43E-37
PAWR	1241	1481	0.146344149	0.008241075	0.008852071
RABGAP1L	1243	860	-0.640142107	5.97E-24	1.02E-23
NOMO2	1243	1701	0.343832469	1.29E-10	1.70E-10
UBR7	1244	444	-1.59507928	7.26E-101	2.82E-100
PFN1P2	1244	491	-1.449915932	6.79E-88	2.35E-87
NBPF9	1245	527	-1.348995251	4.73E-79	1.50E-78
FLCN	1246	477	-1.493967273	5.84E-92	2.10E-91
TM2D1	1247	722	-0.897115099	7.97E-42	1.74E-41
DCAF11	1248	870	-0.629255004	2.29E-23	3.85E-23
SDHB	1248	1210	-0.153335263	0.008400114	0.009019179
SALL2	1250	682	-0.982808826	1.50E-48	3.58E-48
OIP5-AS1	1250	818	-0.720479722	2.79E-29	5.16E-29
RARA	1250	834	-0.692533182	1.99E-27	3.59E-27
SLC25A39	1250	1035	-0.381021703	2.96E-10	3.90E-10
KIAA1609	1251	980	-0.460952511	5.45E-14	7.74E-14
PYGO2	1252	690	-0.968290671	1.76E-47	4.14E-47
MYBBP1A	1252	882	-0.614108377	1.66E-22	2.75E-22
PITPNM1	1253	780	-0.792564761	2.47E-34	4.91E-34
TRMT1L	1254	569	-1.248761166	7.68E-71	2.28E-70
ZNF710	1255	474	-1.513452776	2.30E-94	8.46E-94
KIAA0391	1258	839	-0.693113582	1.25E-27	2.25E-27
FUT8	1259	685	-0.986826766	3.31E-49	7.94E-49
MAP3K11	1259	788	-0.784735124	6.22E-34	1.23E-33
KIAA0753	1260	630	-1.108724376	3.27E-59	8.70E-59
FAM204A	1260	661	-1.039425933	1.74E-53	4.35E-53
EMP2	1261	322	-2.078160058	6.74E-146	3.66E-145
WDR47	1261	698	-0.96199371	2.60E-47	6.10E-47
MED8	1261	1353	-0.007130812	0.899444041	0.901059463
ZNF22	1263	720	-0.919510203	4.63E-44	1.04E-43
C1QBP	1263	956	-0.510496492	1.07E-16	1.60E-16
VWA9	1264	688	-0.986240369	2.39E-49	5.74E-49

NAA16	1265	407	-1.744761061	3.25E-116	1.42E-115
ARHGEF25	1265	833	-0.71147336	5.29E-29	9.74E-29
POLR2G	1265	1008	-0.436366122	6.49E-13	9.01E-13
TRAP1	1265	1230	-0.149203445	0.009772138	0.01046785
SUGT1	1266	996	-0.454784133	7.88E-14	1.12E-13
BROX	1267	690	-0.985472633	2.12E-49	5.11E-49
FAF1	1267	789	-0.792043695	1.16E-34	2.32E-34
HNRNPA1	1269	690	-0.987748178	1.17E-49	2.83E-49
WDR81	1270	553	-1.308201487	5.76E-77	1.81E-76
PRDM4	1270	933	-0.553603887	3.38E-19	5.30E-19
ATP13A2	1271	744	-0.88131388	2.19E-41	4.76E-41
CCDC85B	1271	930	-0.559385785	1.51E-19	2.39E-19
ABHD13	1273	787	-0.802521254	1.42E-35	2.85E-35
ITPR2	1273	1646	0.262007541	1.02E-06	1.23E-06
GBA	1273	1306	-0.071801898	0.206165644	0.21180733
NOL9	1274	591	-1.216859618	3.80E-69	1.11E-68
LAMTOR2	1276	836	-0.718777858	9.73E-30	1.81E-29
WDR59	1278	721	-0.934541048	9.59E-46	2.20E-45
CDIP1	1279	881	-0.646526716	5.25E-25	9.11E-25
MRPS15	1280	1099	-0.3286768	2.86E-08	3.57E-08
MPHOSPH6	1286	628	-1.142778554	2.56E-63	7.05E-63
CAB39L	1287	428	-1.697053728	9.15E-114	3.92E-113
DUSP16	1288	504	-1.462361331	4.42E-92	1.60E-91
PSMB3	1288	1219	-0.188158843	0.001091716	0.001207194
MXI1	1289	575	-1.273342778	5.84E-75	1.80E-74
NPAT	1289	581	-1.258366571	1.27E-73	3.87E-73
FLYWCH1	1289	858	-0.695927087	1.84E-28	3.36E-28
DLAT	1289	967	-0.523388845	9.93E-18	1.52E-17
UTP11L	1289	1015	-0.453496912	5.47E-14	7.76E-14
DIS3L	1290	371	-1.90660435	1.70E-133	8.32E-133
RBBP5	1290	779	-0.836400208	1.50E-38	3.15E-38
ZNF687	1291	718	-0.955157627	7.64E-48	1.81E-47

ZCCHC17	1291	1114	-0.321464144	4.80E-08	5.98E-08
PSMD9	1292	910	-0.614391995	3.42E-23	5.73E-23
RIN3	1295	595	-1.2307149	1.74E-71	5.17E-71
LIG4	1296	736	-0.925012423	1.33E-45	3.04E-45
PRPF3	1297	785	-0.833138296	1.67E-38	3.50E-38
IFT140	1298	594	-1.236479923	3.66E-72	1.10E-71
DHX33	1298	767	-0.867716276	3.57E-41	7.74E-41
SSH3	1299	598	-1.227908417	1.88E-71	5.60E-71
ABCB10	1300	557	-1.331486766	7.35E-81	2.38E-80
KCTD2	1303	759	-0.888389669	6.14E-43	1.36E-42
SPRY2	1303	859	-0.709831423	1.04E-29	1.93E-29
STK38L	1303	980	-0.519707805	1.07E-17	1.64E-17
TTC7B	1304	694	-1.018660678	1.51E-53	3.79E-53
NUAK1	1305	3268	1.215633801	1.39E-161	8.44E-161
NDRG4	1308	768	-0.876908701	3.40E-42	7.45E-42
S100A16	1308	1837	0.38126471	1.84E-13	2.58E-13
BAHD1	1310	765	-0.884759535	7.12E-43	1.58E-42
BBS2	1315	816	-0.797146118	2.73E-36	5.56E-36
SELENBP1	1316	630	-1.171460131	2.69E-67	7.72E-67
L3HYPDH	1316	809	-0.810672257	2.47E-37	5.11E-37
LETMD1	1318	707	-1.007292626	3.83E-53	9.55E-53
FEZ1	1318	737	-0.947338222	3.64E-48	8.62E-48
ACAT1	1318	846	-0.748345178	8.94E-33	1.74E-32
EBNA1BP2	1318	1176	-0.273186686	2.29E-06	2.72E-06
MMD	1319	411	-1.790958641	1.73E-125	8.10E-125
TOR3A	1319	628	-1.179332476	3.78E-68	1.09E-67
C10orf76	1321	611	-1.221110557	5.16E-72	1.54E-71
ZNF697	1321	696	-1.033195631	1.86E-55	4.76E-55
C1orf21	1323	493	-1.532877886	3.21E-101	1.25E-100
NPRL3	1323	840	-0.764076205	4.84E-34	9.57E-34
BCKDK	1325	1048	-0.447078019	5.27E-14	7.49E-14
SLC35C1	1325	1216	-0.232573507	4.85E-05	5.55E-05

HBE1	1328	38	-5.235836294	0	0
UHRF1BP1L	1329	801	-0.839191333	7.02E-40	1.50E-39
DCUN1D5	1329	1008	-0.507569842	2.52E-17	3.84E-17
FAM102B	1330	388	-1.886022064	1.30E-135	6.46E-135
FAM98B	1330	796	-0.849310286	1.07E-40	2.30E-40
PAQR7	1331	865	-0.730462909	8.73E-32	1.67E-31
SETD1A	1334	685	-1.070307149	3.33E-59	8.86E-59
METTL16	1334	748	-0.943372867	2.12E-48	5.04E-48
PSMD12	1335	1013	-0.506929944	2.33E-17	3.55E-17
SARNP	1335	1055	-0.448321119	3.66E-14	5.21E-14
RABGGTB	1336	1068	-0.431732737	2.55E-13	3.58E-13
TBC1D4	1337	566	-1.348849883	9.70E-85	3.27E-84
CERS5	1337	1054	-0.451848974	2.29E-14	3.27E-14
GDF11	1338	909	-0.666450292	1.96E-27	3.53E-27
STYX	1338	1056	-0.450192657	2.73E-14	3.90E-14
AATF	1339	749	-0.946842713	7.29E-49	1.74E-48
EPC1	1340	608	-1.248814148	1.41E-75	4.36E-75
USP28	1340	742	-0.961466285	4.04E-50	9.81E-50
UBL7	1340	1011	-0.515174379	6.83E-18	1.05E-17
FAM189B	1341	896	-0.690462976	3.70E-29	6.83E-29
DNAJA3	1342	988	-0.550526101	5.20E-20	8.26E-20
CLUH	1343	822	-0.816973382	1.50E-38	3.15E-38
ADRBK1	1343	890	-0.702306439	4.91E-30	9.20E-30
LACTB	1344	801	-0.855383366	1.37E-41	2.99E-41
COX5A	1344	1169	-0.309982584	7.40E-08	9.17E-08
SUCLA2	1345	791	-0.874580949	3.75E-43	8.35E-43
TSNAX	1345	921	-0.655057487	8.63E-27	1.54E-26
ZDHHC18	1346	1245	-0.221257044	9.49E-05	0.000107991
CSNK2A2	1347	773	-0.909933907	4.36E-46	1.01E-45
EIF2B1	1348	831	-0.80662449	6.92E-38	1.44E-37
UROS	1348	874	-0.733839688	2.04E-32	3.95E-32
RIT1	1348	1066	-0.447337434	3.10E-14	4.42E-14

G6PC3	1348	1250	-0.217616778	0.000120875	0.000137177
ODF2L	1350	507	-1.521626131	3.73E-102	1.47E-101
NCDN	1351	835	-0.802903948	1.11E-37	2.31E-37
SETD8	1353	910	-0.680947765	9.82E-29	1.80E-28
AKR1A1	1353	1475	0.015828739	0.770508973	0.774775247
LUC7L	1355	647	-1.17517961	1.41E-69	4.13E-69
PDE8A	1355	739	-0.983370958	1.59E-52	3.93E-52
ADAMTS8	1356	120	-3.606975243	2.42E-276	2.82E-275
HECTD3	1356	926	-0.658997456	2.88E-27	5.18E-27
MNT	1357	807	-0.858504518	3.17E-42	6.97E-42
MRPL49	1357	962	-0.605036298	1.15E-23	1.95E-23
RPS6KA4	1358	656	-1.158440136	3.43E-68	9.94E-68
ERMAP	1360	502	-1.546571758	2.45E-105	9.87E-105
FAM46C	1361	5164	1.815097558	0	0
CAPN5	1362	837	-0.811151551	1.29E-38	2.71E-38
PEX5	1365	669	-1.137547211	1.29E-66	3.67E-66
HEATR6	1366	759	-0.956510069	1.24E-50	3.01E-50
ST3GAL2	1366	962	-0.61457306	1.93E-24	3.32E-24
G2E3	1367	1015	-0.538257891	1.33E-19	2.10E-19
MPG	1367	1379	-0.096115162	0.080767829	0.084162794
TIMM10B	1368	925	-0.673267335	1.72E-28	3.14E-28
NAA25	1369	825	-0.839380798	4.82E-41	1.04E-40
RBM23	1370	560	-1.399401537	1.12E-91	4.00E-91
ECI1	1370	976	-0.597947216	2.07E-23	3.48E-23
NDUFS8	1370	1511	0.032603391	0.544266315	0.55018455
ENTPD7	1371	1281	-0.206682471	0.000224972	0.000253179
PTDSS2	1374	849	-0.803269921	2.55E-38	5.32E-38
TBK1	1374	884	-0.744988105	7.64E-34	1.51E-33
NLRP1	1375	1381	-0.102442675	0.062237335	0.065105338
LYST	1377	433	-1.777814005	1.30E-129	6.22E-129
EMC4	1377	1090	-0.4459248	1.96E-14	2.82E-14
POLG	1377	1116	-0.411915908	1.15E-12	1.59E-12

BBS10	1379	769	-0.951291329	1.19E-50	2.90E-50
ZNRF1	1379	837	-0.829047305	1.68E-40	3.61E-40
SOX9	1379	1097	-0.438783307	4.52E-14	6.42E-14
ANAPC7	1382	924	-0.689517235	6.25E-30	1.17E-29
UBE2D1	1383	618	-1.270846789	4.75E-80	1.53E-79
KIAA0556	1384	792	-0.913995983	1.20E-47	2.83E-47
MMP19	1384	1262	-0.241856409	1.62E-05	1.89E-05
SOLH	1385	687	-1.120228348	5.79E-66	1.64E-65
MCRS1	1385	855	-0.804614027	1.02E-38	2.14E-38
TRAPPC6B	1386	960	-0.638545322	2.08E-26	3.68E-26
BLMH	1386	1003	-0.575330027	3.45E-22	5.69E-22
TMEM194A	1387	401	-1.899018022	1.24E-142	6.56E-142
RARG	1387	739	-1.017045894	1.07E-56	2.77E-56
GSTM2	1388	681	-1.13600524	1.45E-67	4.17E-67
DCAKD	1388	722	-1.051661202	7.74E-60	2.07E-59
VPS18	1390	914	-0.713543188	7.41E-32	1.42E-31
SLC25A30	1393	682	-1.139075989	4.35E-68	1.26E-67
GJC1	1394	638	-1.236326608	2.40E-77	7.53E-77
KIFC3	1394	917	-0.712961298	6.70E-32	1.29E-31
NEDD1	1395	724	-1.054927896	1.99E-60	5.36E-60
CEPT1	1398	728	-1.050078381	4.11E-60	1.10E-59
PARP6	1399	647	-1.221282721	3.58E-76	1.11E-75
SLC35A3	1399	993	-0.603254716	3.15E-24	5.40E-24
NT5C3B	1399	1001	-0.591678364	1.87E-23	3.14E-23
VPS45	1401	811	-0.897407512	8.47E-47	1.98E-46
POU2F1	1402	588	-1.362322665	4.25E-90	1.49E-89
FTSJD1	1402	619	-1.288199411	8.17E-83	2.69E-82
ADO	1403	971	-0.639696184	8.59E-27	1.53E-26
NUP93	1404	953	-0.667719192	8.48E-29	1.56E-28
FH	1404	1243	-0.284441015	4.00E-07	4.86E-07
ANKRD26	1405	417	-1.861175218	1.21E-140	6.33E-140
RLF	1405	979	-0.629913741	3.80E-26	6.70E-26

TCEB2	1405	1261	-0.264726231	2.18E-06	2.60E-06
DOCK9	1407	1285	-0.239578345	1.65E-05	1.92E-05
C11orf30	1408	761	-0.996403351	1.13E-55	2.90E-55
CWC15	1408	891	-0.768874373	1.70E-36	3.48E-36
FAM89B	1408	1015	-0.580891982	7.00E-23	1.17E-22
VTI1B	1408	1158	-0.390736457	7.59E-12	1.03E-11
LDLRAP1	1409	786	-0.95079477	1.14E-51	2.80E-51
SMNDC1	1410	909	-0.742067339	1.84E-34	3.67E-34
DNMBP	1411	985	-0.627246734	4.57E-26	8.06E-26
PDZD8	1412	716	-1.088432972	3.07E-64	8.54E-64
NR2C1	1413	678	-1.168128663	8.41E-72	2.51E-71
SLITRK6	1416	320	-2.254401831	7.06E-181	4.91E-180
WDR37	1416	973	-0.650033931	9.29E-28	1.68E-27
COPZ2	1417	1536	0.007614082	0.885976694	0.887795715
CHORDC1	1419	864	-0.824495748	2.99E-41	6.50E-41
ATP6V0B	1419	1221	-0.325535765	7.00E-09	8.91E-09
CSTF2T	1423	685	-1.163484145	7.52E-72	2.24E-71
TBC1D12	1426	503	-1.612068053	4.37E-117	1.93E-116
DIAPH3	1427	467	-1.720215256	2.58E-128	1.23E-127
GALNS	1428	1317	-0.22546501	4.24E-05	4.87E-05
DVL1	1429	964	-0.676625241	6.18E-30	1.16E-29
AHDC1	1430	977	-0.658309056	1.29E-28	2.37E-28
WNT5B	1433	2553	0.724430552	2.01E-54	5.09E-54
ATP6V0A2	1433	895	-0.787803398	1.24E-38	2.60E-38
FDPS	1433	1430	-0.111747838	0.038164746	0.04017098
APH1B	1436	678	-1.191422947	3.30E-75	1.02E-74
DUSP14	1437	1653	0.093302287	0.072493703	0.075682263
ZSCAN29	1438	572	-1.438701	3.75E-100	1.45E-99
RECQL	1438	1117	-0.473158866	1.47E-16	2.20E-16
APBA2	1438	1162	-0.416177983	2.19E-13	3.08E-13
NBPF10	1440	542	-1.518428431	1.67E-108	6.88E-108
GNG2	1441	756	-1.039336572	6.68E-61	1.80E-60

CMIP	1442	1204	-0.368960148	5.14E-11	6.86E-11
VCAM1	1443	62	-4.64938365	0	0
EPHX1	1443	485	-1.681739023	1.02E-125	4.78E-125
FBXL20	1443	625	-1.315867581	5.75E-88	1.99E-87
C11orf31	1443	731	-1.089852364	9.60E-66	2.72E-65
FAM108C1	1443	773	-1.009255356	3.41E-58	8.97E-58
PROSER1	1443	824	-0.917079433	7.11E-50	1.72E-49
SUFU	1444	726	-1.100753665	7.91E-67	2.26E-66
TCIRG1	1444	915	-0.76695147	3.14E-37	6.48E-37
NDUFA9	1445	1096	-0.50754607	1.06E-18	1.66E-18
KDM6B	1449	714	-1.129785991	7.52E-70	2.21E-69
GLRX5	1451	1029	-0.604528913	3.72E-25	6.46E-25
PLCB3	1455	1016	-0.626843127	8.46E-27	1.51E-26
ZNF281	1455	1366	-0.199786045	0.000234967	0.000264351
CNPY2	1456	1628	0.052365968	0.313704717	0.320057504
ATF7	1457	683	-1.20176777	2.62E-77	8.23E-77
SYF2	1459	707	-1.153922139	1.11E-72	3.35E-72
TP73-AS1	1460	776	-1.020564187	6.48E-60	1.74E-59
LGR4	1461	614	-1.359369993	1.67E-93	6.08E-93
DNAJC11	1461	763	-1.045925592	2.41E-62	6.57E-62
C16orf45	1461	817	-0.947272571	3.42E-53	8.54E-53
SLFN11	1461	1447	-0.122615632	0.021877409	0.023205744
GPR153	1462	415	-1.925484446	5.28E-153	3.00E-152
COASY	1462	1274	-0.30730241	2.60E-08	3.26E-08
NDUFAB1	1462	1351	-0.222640013	4.27E-05	4.90E-05
DRAM2	1463	1011	-0.641871148	5.01E-28	9.11E-28
RBM4	1464	895	-0.818680342	5.19E-42	1.14E-41
NFATC3	1464	942	-0.744840965	6.16E-36	1.25E-35
RUFY2	1465	669	-1.239546925	1.63E-81	5.29E-81
EML3	1466	631	-1.324897569	2.89E-90	1.02E-89
KAT2A	1467	593	-1.415489237	1.04E-99	4.00E-99
ACBD5	1467	736	-1.103815576	3.68E-68	1.07E-67

SPTY2D1	1467	1409	-0.166921635	0.001913488	0.002101311
CHD1L	1468	905	-0.806586647	4.21E-41	9.13E-41
GAB2	1471	541	-1.551821123	2.49E-114	1.07E-113
POLR3A	1471	1104	-0.52278145	5.67E-20	9.00E-20
CACNB3	1472	594	-1.417967211	2.66E-100	1.03E-99
TMEM63A	1472	718	-1.144446298	2.25E-72	6.76E-72
BCL2L2	1472	1086	-0.547477944	1.36E-21	2.22E-21
DAAM1	1474	247	-2.685877953	1.45E-229	1.32E-228
USP36	1474	857	-0.891093791	1.39E-48	3.32E-48
PSKH1	1475	868	-0.873672383	4.50E-47	1.05E-46
MYOCD	1477	634	-1.328839457	2.45E-91	8.75E-91
POLD4	1477	1292	-0.301788132	3.80E-08	4.74E-08
RNF115	1478	943	-0.757040969	2.96E-37	6.12E-37
MRPS21	1478	1088	-0.550692089	6.89E-22	1.13E-21
SAP30BP	1479	912	-0.806240699	2.28E-41	4.97E-41
UCK2	1479	1443	-0.144275128	0.006854196	0.007383697
STK40	1481	666	-1.261701934	1.19E-84	4.02E-84
FLJ31306	1482	575	-1.474635962	5.84E-107	2.38E-106
MEGF6	1483	511	-1.645847777	2.65E-125	1.24E-124
TUFT1	1483	614	-1.380932413	3.84E-97	1.45E-96
VANGL1	1487	435	-1.882041717	5.84E-151	3.27E-150
VWA8	1487	749	-1.098091399	1.68E-68	4.87E-68
RIMKLB	1488	480	-1.740992591	6.02E-136	3.00E-135
NFRKB	1488	693	-1.211171645	7.21E-80	2.31E-79
PEF1	1490	914	-0.813770636	2.64E-42	5.81E-42
APRT	1490	1277	-0.331278182	1.61E-09	2.08E-09
GLRX3	1491	1170	-0.458496104	3.08E-16	4.59E-16
NDN	1493	621	-1.374273368	4.42E-97	1.67E-96
AURKAIP1	1493	1402	-0.199452192	0.000199361	0.00022481
IRAK4	1494	653	-1.302749627	1.22E-89	4.29E-89
ETV6	1494	928	-0.795707814	7.13E-41	1.54E-40
NAE1	1496	703	-1.198237957	5.75E-79	1.83E-78

VEGFB	1496	993	-0.699968928	4.42E-33	8.60E-33
SV2A	1496	1577	-0.032651891	0.530361604	0.536336929
RNF2	1497	760	-1.086727274	7.93E-68	2.28E-67
C1orf63	1497	826	-0.966584911	2.92E-56	7.51E-56
SETDB1	1498	668	-1.273841992	7.35E-87	2.53E-86
CHUK	1498	840	-0.943300767	4.00E-54	1.01E-53
TROVE2	1498	844	-0.936447096	1.73E-53	4.32E-53
KLHL20	1498	973	-0.73125029	1.26E-35	2.55E-35
FOXJ2	1499	894	-0.854378023	4.28E-46	9.90E-46
GSTO1	1499	1376	-0.232244289	1.59E-05	1.85E-05
HSDL1	1500	750	-1.108724376	3.86E-70	1.14E-69
VASH1	1501	962	-0.750539554	2.86E-37	5.90E-37
TAF10	1501	1188	-0.446113517	1.31E-15	1.93E-15
THBS3	1503	910	-0.832630935	2.68E-44	6.05E-44
SCAMP2	1504	1328	-0.288273796	1.07E-07	1.33E-07
MADD	1505	765	-1.08495621	5.27E-68	1.52E-67
SGPP1	1505	1150	-0.496854002	9.90E-19	1.54E-18
NAA60	1507	1086	-0.58137969	2.07E-24	3.55E-24
TGFBR3	1508	119	-3.772327326	0	0
USP32P1	1508	655	-1.311793993	2.05E-91	7.33E-91
VPS11	1509	655	-1.31275037	1.42E-91	5.08E-91
WASF3	1509	788	-1.046049647	2.41E-64	6.71E-64
SORD	1511	689	-1.241652148	3.10E-84	1.04E-83
CCDC92	1514	1077	-0.600071331	7.45E-26	1.31E-25
PMM2	1515	1501	-0.122118193	0.020061021	0.021302218
PPM1D	1516	591	-1.467764094	1.26E-108	5.23E-108
EAPP	1516	761	-1.103025771	2.69E-70	7.96E-70
KDM2B	1517	747	-1.130765313	3.86E-73	1.17E-72
SCAMP3	1517	1225	-0.417163712	4.28E-14	6.09E-14
YY1AP1	1519	892	-0.87673063	1.06E-48	2.52E-48
GGPS1	1519	968	-0.758767293	2.19E-38	4.59E-38
RNF31	1520	781	-1.069401246	4.11E-67	1.18E-66

NOL11	1520	895	-0.872836112	2.22E-48	5.28E-48
SIRT1	1521	565	-1.537421756	1.42E-116	6.22E-116
DHCR7	1521	2664	0.699849553	1.74E-53	4.36E-53
AP5M1	1521	918	-0.83717847	3.28E-45	7.49E-45
NKIRAS2	1521	1151	-0.510856695	8.14E-20	1.29E-19
CFDP1	1523	1214	-0.435871896	3.25E-15	4.76E-15
GTF2H3	1525	866	-0.925094689	2.35E-53	5.87E-53
DDX19A	1526	1087	-0.598127398	6.63E-26	1.16E-25
FKBP4	1526	1191	-0.466305925	4.66E-17	7.06E-17
HECTD2	1527	465	-1.824121817	1.30E-148	7.19E-148
EZH1	1527	664	-1.310169291	2.28E-92	8.27E-92
SCMH1	1527	843	-0.965819902	2.80E-57	7.30E-57
ERI3	1527	1157	-0.509035574	9.07E-20	1.44E-19
SYTL2	1527	2061	0.323920067	2.24E-11	3.01E-11
CAPRIN2	1528	775	-1.088100704	2.48E-69	7.25E-69
KIAA1551	1530	383	-2.106839732	1.16E-179	7.97E-179
KAT7	1530	855	-0.948259704	1.03E-55	2.65E-55
TAF1D	1530	1035	-0.672625261	1.28E-31	2.46E-31
MAP2K3	1530	1330	-0.310829783	8.60E-09	1.09E-08
DERL2	1532	1164	-0.505049615	1.44E-19	2.27E-19
HDAC5	1532	1837	0.153210953	0.00209151	0.002293905
DPP3	1533	1135	-0.542389775	4.51E-22	7.43E-22
NSRP1	1535	1041	-0.668992963	1.98E-31	3.78E-31
RPS6KC1	1535	1066	-0.634755593	8.82E-29	1.62E-28
KIFAP3	1540	766	-1.11623843	9.93E-73	3.00E-72
TMEM119	1541	658	-1.336431749	4.94E-96	1.85E-95
PRCC	1541	924	-0.846626481	1.24E-46	2.88E-46
TRMT112	1541	1400	-0.247164411	3.41E-06	4.03E-06
RNF220	1542	756	-1.137069002	5.77E-75	1.78E-74
FMNL3	1543	990	-0.748962007	3.93E-38	8.20E-38
KRTCAP2	1543	1590	-0.065435672	0.204152566	0.209821978
FGFR1OP2	1549	935	-0.83702325	5.37E-46	1.24E-45

MAP3K12	1550	492	-1.764262371	3.93E-144	2.10E-143
C1orf122	1550	1533	-0.124634894	0.016424855	0.017498185
E2F4	1551	860	-0.959514497	1.54E-57	4.02E-57
ZNF598	1551	1082	-0.628222563	1.46E-28	2.66E-28
S100PBP	1552	720	-1.216784122	8.09E-84	2.69E-83
FGF9	1553	160	-3.387638395	5.03E-301	6.59E-300
STARD9	1553	471	-1.829983241	9.21E-152	5.17E-151
MBTD1	1553	499	-1.746670485	1.96E-142	1.03E-141
CTDSPL2	1553	783	-1.096697993	2.57E-71	7.63E-71
FRMD4A	1553	1348	-0.312961709	5.32E-09	6.79E-09
AAGAB	1554	1078	-0.636353702	3.07E-29	5.66E-29
RSPRY1	1555	1111	-0.59378014	4.81E-26	8.46E-26
PDK2	1557	617	-1.444150926	6.95E-109	2.88E-108
PSPC1	1557	634	-1.404938575	1.53E-104	6.13E-104
TBCEL	1558	811	-1.05064579	7.70E-67	2.20E-66
GMPR2	1558	1141	-0.558120818	1.64E-23	2.77E-23
ELAC2	1559	999	-0.750788721	1.15E-38	2.42E-38
MRPS34	1560	1163	-0.532419308	9.61E-22	1.58E-21
CACYBP	1561	642	-1.390549711	3.27E-103	1.29E-102
THRA	1561	816	-1.044553856	2.39E-66	6.81E-66
NAT10	1561	860	-0.968786348	8.35E-59	2.21E-58
EFHD2	1564	1037	-0.701548994	1.23E-34	2.44E-34
SFSWAP	1566	840	-1.007347356	8.29E-63	2.27E-62
ATG14	1567	637	-1.407364278	1.81E-105	7.27E-105
RNF169	1567	646	-1.387123486	3.20E-103	1.27E-102
GPR180	1568	1986	0.232215688	1.73E-06	2.07E-06
CELF2	1570	141	-3.585721867	0	0
ZFYVE26	1570	783	-1.112404722	1.04E-73	3.16E-73
KIAA0513	1571	1137	-0.575175302	6.34E-25	1.10E-24
GTF3A	1571	1182	-0.519177521	5.61E-21	9.06E-21
TOLLIP	1572	935	-0.858287323	1.29E-48	3.07E-48
B4GALNT4	1576	777	-1.129005407	9.99E-76	3.09E-75

FAM171A1	1577	625	-1.443978941	3.07E-110	1.28E-109
SNF8	1579	1355	-0.329442696	6.50E-10	8.48E-10
ZC3H18	1580	912	-0.901543205	6.03E-53	1.50E-52
PIK3R3	1581	287	-2.570439102	1.12E-234	1.04E-233
FEM1B	1583	800	-1.093313726	2.64E-72	7.93E-72
B3GALT6	1583	1066	-0.679178193	3.45E-33	6.72E-33
UROD	1583	1279	-0.416369367	1.35E-14	1.94E-14
CNST	1584	1274	-0.422931434	5.45E-15	7.92E-15
LGALS8	1585	675	-1.340247809	3.82E-99	1.46E-98
SAT2	1585	995	-0.780438785	6.85E-42	1.50E-41
AP4E1	1585	1014	-0.753149564	1.73E-39	3.68E-39
RHOT1	1585	1035	-0.723576449	6.13E-37	1.26E-36
FAM188A	1586	858	-0.995067594	2.40E-62	6.54E-62
BCL9	1586	982	-0.800322217	1.07E-43	2.39E-43
HN1L	1586	1265	-0.434979762	1.00E-15	1.47E-15
TBC1D10B	1587	1050	-0.704637176	2.21E-35	4.45E-35
NMT2	1589	886	-0.951464897	4.15E-58	1.09E-57
COPS3	1589	1091	-0.651192399	4.86E-31	9.24E-31
RTCA	1590	1193	-0.523157098	1.74E-21	2.84E-21
TMEM57	1592	957	-0.842973882	8.87E-48	2.10E-47
NSMCE1	1592	1161	-0.56419674	1.98E-24	3.40E-24
AKAP10	1593	858	-1.00142109	2.94E-63	8.11E-63
IFITM1	1594	1576	-0.12510847	0.014598054	0.015579637
SOCS3	1595	988	-0.799697853	7.02E-44	1.58E-43
SEMA6D	1598	1160	-0.570866979	5.25E-25	9.11E-25
AMPD2	1600	1693	-0.027214308	0.588268509	0.593512763
KLHL12	1601	921	-0.906424622	4.23E-54	1.07E-53
TRAF5	1601	1349	-0.355807335	2.26E-11	3.05E-11
EPHA2	1601	1868	0.113796771	0.020320912	0.021572322
DPYD	1602	471	-1.874799559	1.20E-161	7.31E-161
CNNM2	1602	1187	-0.541278589	6.49E-23	1.08E-22
VPS29	1603	1171	-0.561757726	2.08E-24	3.57E-24

EIF4A3	1605	1035	-0.741666906	5.91E-39	1.25E-38
PDHX	1607	1321	-0.391463838	2.36E-13	3.31E-13
SQRDL	1608	756	-1.197533643	1.16E-84	3.93E-84
FCF1	1608	1068	-0.699080135	2.35E-35	4.72E-35
MAX	1609	856	-1.019206	1.06E-65	2.99E-65
NAA30	1609	985	-0.816693072	8.16E-46	1.88E-45
TFCP2	1611	800	-1.118608965	2.99E-76	9.31E-76
OPN3	1612	951	-0.870058874	6.21E-51	1.52E-50
SLC6A9	1614	961	-0.856756618	1.01E-49	2.44E-49
GLTP	1616	1317	-0.403896228	3.90E-14	5.57E-14
USP15	1618	1174	-0.571503575	2.40E-25	4.19E-25
FAM214A	1620	716	-1.286686696	3.18E-95	1.18E-94
FAM73A	1620	1089	-0.681714235	3.88E-34	7.68E-34
DNAJC15	1623	845	-1.050364129	1.60E-69	4.69E-69
CDC42EP2	1623	970	-0.851330723	1.80E-49	4.35E-49
RFWD2	1626	1112	-0.656894845	3.43E-32	6.62E-32
SH2B1	1627	889	-0.980683303	1.97E-62	5.39E-62
GSTM3	1627	947	-0.889502296	2.77E-53	6.91E-53
TYRO3	1628	714	-1.297829096	6.00E-97	2.26E-96
UTP20	1629	639	-1.458823143	1.55E-115	6.77E-115
HCFC1R1	1629	871	-1.011966356	9.67E-66	2.73E-65
TRIM37	1629	966	-0.862615886	9.87E-51	2.41E-50
GNL2	1629	1331	-0.400200409	5.08E-14	7.22E-14
NOC2L	1630	1136	-0.629633506	4.78E-30	8.96E-30
C10orf10	1631	700	-1.329054331	1.10E-100	4.27E-100
CSAD	1632	435	-2.016278127	4.23E-181	2.94E-180
PSME2	1632	1060	-0.731301168	1.16E-38	2.43E-38
ANGEL2	1633	774	-1.185843695	1.20E-84	4.03E-84
TRUB1	1633	822	-1.099038868	3.80E-75	1.17E-74
TRAPPC3	1633	1349	-0.384358819	3.86E-13	5.38E-13
PRPF39	1634	876	-1.008129584	1.55E-65	4.37E-65
PWP1	1634	1194	-0.561329523	8.12E-25	1.40E-24

CRY2	1636	674	-1.388076628	8.61E-108	3.53E-107
DENND4B	1636	790	-1.158972566	7.93E-82	2.59E-81
PHF13	1637	1019	-0.792624646	2.48E-44	5.60E-44
RASL11A	1639	142	-3.637577395	0	0
LOC10019098	1640	550	-1.684916667	5.49E-143	2.91E-142
PRMT5	1640	1409	-0.327748579	3.71E-10	4.87E-10
FGD6	1641	915	-0.951455966	5.99E-60	1.60E-59
CUEDC1	1642	817	-1.11577052	2.26E-77	7.11E-77
TMUB2	1643	932	-0.926654996	1.68E-57	4.38E-57
CEP164	1644	933	-0.925985689	1.81E-57	4.73E-57
C11orf68	1647	960	-0.88745862	1.03E-53	2.58E-53
ARRDC4	1648	338	-2.394345467	1.08E-225	9.59E-225
RCOR1	1648	1027	-0.791004437	1.82E-44	4.11E-44
COX15	1649	892	-0.995200159	9.16E-65	2.56E-64
CYBASC3	1651	862	-1.046304722	2.97E-70	8.76E-70
EFS	1651	953	-0.901516377	3.05E-55	7.79E-55
PHACTR4	1655	824	-1.11483935	7.23E-78	2.28E-77
ZFYVE21	1655	876	-1.026552818	2.62E-68	7.58E-68
SPNS1	1655	997	-0.839890183	2.67E-49	6.40E-49
PARN	1656	949	-0.911947056	1.88E-56	4.84E-56
CPSF3L	1656	950	-0.91042763	2.67E-56	6.88E-56
FBXW8	1656	1508	-0.24379062	2.02E-06	2.40E-06
DTWD1	1659	673	-1.410359852	6.70E-112	2.83E-111
WAC-AS1	1660	1053	-0.765402181	2.13E-42	4.71E-42
PIP4K2A	1666	1158	-0.633477523	5.45E-31	1.03E-30
GLIPR1	1666	1551	-0.21191409	3.10E-05	3.58E-05
DIXDC1	1668	1014	-0.826786012	2.11E-48	5.02E-48
CTSF	1670	672	-1.42203934	5.14E-114	2.21E-113
BECN1	1673	1040	-0.794578293	1.87E-45	4.29E-45
C14orf119	1673	1614	-0.160521243	0.00141999	0.001566201
AKR7A2	1675	944	-0.936026707	1.49E-59	3.97E-59
FOXL1	1677	3933	1.121023121	9.03E-171	5.85E-170

M6PR	1677	981	-0.882282023	3.86E-54	9.76E-54
SLC22A17	1678	2100	0.214922236	4.97E-06	5.87E-06
TRIM13	1679	859	-1.07559657	1.28E-74	3.92E-74
DBT	1679	997	-0.860661196	4.86E-52	1.20E-51
SNX7	1679	1104	-0.713586434	3.94E-38	8.22E-38
SDF2	1680	1264	-0.519189145	2.53E-22	4.18E-22
GTF2F2	1682	989	-0.874859655	1.50E-53	3.77E-53
PLEKHO1	1682	1755	-0.047431051	0.335049859	0.341344337
SETD3	1685	1184	-0.617803886	4.99E-30	9.34E-30
RFX5	1686	947	-0.940892581	1.94E-60	5.22E-60
GPR137B	1687	763	-1.253429387	3.40E-95	1.26E-94
MRPL42	1688	1141	-0.673740488	8.35E-35	1.67E-34
CSTF3	1690	931	-0.96889455	1.59E-63	4.40E-63
RNF167	1690	985	-0.887551993	4.43E-55	1.13E-54
CLEC16A	1690	1087	-0.745395682	2.86E-41	6.22E-41
NHLRC2	1693	845	-1.111283103	3.27E-79	1.04E-78
STAM	1694	980	-0.898304596	2.64E-56	6.81E-56
PSMA3	1694	1189	-0.619409535	2.59E-30	4.88E-30
DUT	1697	896	-1.030140303	2.18E-70	6.46E-70
FGD4	1700	1057	-0.794283746	3.94E-46	9.13E-46
ROR1	1701	370	-2.309510341	3.15E-223	2.77E-222
PPP1R12B	1701	1557	-0.236338572	2.94E-06	3.49E-06
SSPN	1702	604	-1.603334958	2.72E-138	1.39E-137
FHOD1	1704	678	-1.438292533	2.87E-118	1.28E-117
ATPIF1	1704	1512	-0.281191572	3.32E-08	4.14E-08
HERC2P2	1706	593	-1.633238013	2.71E-142	1.43E-141
NPIP	1706	777	-1.243355519	4.64E-95	1.72E-94
FXR2	1707	1265	-0.541050049	2.71E-24	4.65E-24
LEMD3	1709	883	-1.06139143	2.47E-74	7.57E-74
MLLT10	1709	885	-1.058127413	5.71E-74	1.74E-73
C14orf101	1709	930	-0.986574152	4.03E-66	1.15E-65
RNF111	1709	1075	-0.777540113	9.40E-45	2.14E-44

SNAPC1	1709	1095	-0.750945903	3.11E-42	6.84E-42
DUSP6	1710	3167	0.780396166	4.38E-76	1.36E-75
OSBPL5	1710	933	-0.982771715	9.52E-66	2.70E-65
PPHLN1	1710	1117	-0.723091515	1.13E-39	2.41E-39
ACVR1B	1711	888	-1.054932554	1.07E-73	3.25E-73
ZNF410	1713	1098	-0.750371473	2.83E-42	6.23E-42
STX5	1714	1459	-0.341111602	2.91E-11	3.90E-11
CCDC144B	1715	694	-1.413925384	4.79E-116	2.09E-115
RBM8A	1716	1080	-0.776742616	7.47E-45	1.70E-44
PCID2	1718	1247	-0.570992947	9.03E-27	1.61E-26
MRPS35	1719	1168	-0.666253646	9.40E-35	1.87E-34
TOP2A	1720	496	-1.902720915	7.63E-177	5.17E-176
HIC1	1722	950	-0.9668101	1.81E-64	5.06E-64
PELP1	1723	1040	-0.837063549	5.73E-51	1.40E-50
NAV2	1725	471	-1.981521773	4.75E-187	3.44E-186
PPP4C	1725	1416	-0.393499472	2.39E-14	3.42E-14
LPCAT3	1727	615	-1.598334143	1.14E-139	5.88E-139
MAPK3	1727	1408	-0.403345125	5.73E-15	8.32E-15
CEP104	1728	1144	-0.703740541	2.73E-38	5.69E-38
NAB2	1732	902	-1.049963967	5.08E-74	1.55E-73
PPP2R1B	1735	724	-1.369598436	6.26E-112	2.65E-111
TSEN15	1735	1412	-0.40591995	3.43E-15	5.01E-15
NFATC2IP	1736	722	-1.374420581	1.39E-112	5.89E-112
TMEM9	1736	1095	-0.773560454	4.79E-45	1.09E-44
COG3	1737	1220	-0.618440982	5.87E-31	1.11E-30
BLOC1S2	1737	1297	-0.53014365	7.44E-24	1.27E-23
TCF7L2	1739	675	-1.474022901	4.31E-125	2.01E-124
ZFP90	1740	852	-1.138886346	1.46E-84	4.92E-84
C1QTNF1	1740	1400	-0.422384855	2.79E-16	4.16E-16
MKL2	1741	603	-1.638410672	8.04E-146	4.35E-145
TIMM17A	1741	1690	-0.151617332	0.002077191	0.00227884
CSK	1743	1003	-0.905975339	1.09E-58	2.87E-58

BAG5	1743	1196	-0.652079556	5.81E-34	1.15E-33
USP1	1744	642	-1.550479214	5.17E-135	2.56E-134
PRKD1	1745	924	-1.025986656	7.36E-72	2.20E-71
CAMKK2	1745	1158	-0.700316159	2.46E-38	5.14E-38
CREG1	1746	598	-1.654560545	2.86E-148	1.57E-147
SLC38A4	1747	249	-2.919386337	7.76E-296	1.00E-294
MSANTD4	1748	1105	-0.770383191	4.90E-45	1.12E-44
EIF2D	1750	1015	-0.894599571	1.03E-57	2.69E-57
TBC1D15	1750	1125	-0.746154297	9.57E-43	2.12E-42
UBE2L6	1754	1058	-0.838033496	5.94E-52	1.47E-51
DHRS7	1754	1516	-0.31910337	2.65E-10	3.49E-10
ZNF605	1755	1105	-0.776149037	8.96E-46	2.06E-45
PDDC1	1757	739	-1.358192298	6.54E-112	2.76E-111
DHX38	1757	1053	-0.847333131	5.39E-53	1.34E-52
CLSTN3	1757	1324	-0.516935445	4.21E-23	7.04E-23
NADSYN1	1760	1347	-0.494549954	1.82E-21	2.96E-21
UNC5B	1760	2139	0.172636678	0.000192509	0.000217146
S100A13	1761	1862	-0.028266212	0.555339605	0.561015132
SLC2A3	1762	2675	0.493600591	1.70E-29	3.16E-29
SKA2	1764	931	-1.030721864	3.69E-73	1.12E-72
CLN5	1767	843	-1.176421879	2.61E-90	9.19E-90
LCOR	1767	963	-0.984418713	4.62E-68	1.34E-67
TPRG1L	1767	1295	-0.557074318	2.31E-26	4.10E-26
KATNAL1	1768	1370	-0.476666757	2.96E-20	4.72E-20
CAMTA2	1769	1203	-0.665021781	1.33E-35	2.68E-35
FAM104A	1773	1179	-0.697353194	1.20E-38	2.52E-38
ZNF740	1774	840	-1.187269153	5.77E-92	2.08E-91
DNAJC7	1774	1175	-0.703069629	3.35E-39	7.09E-39
DDX52	1775	1184	-0.69287432	2.85E-38	5.95E-38
LRP4	1776	1027	-0.898919776	5.23E-59	1.39E-58
DNAJC16	1777	999	-0.939611474	1.84E-63	5.08E-63
SMARCD2	1777	1103	-0.796735266	2.19E-48	5.20E-48

TMEM41B	1780	1528	-0.328957074	5.74E-11	7.66E-11
SCPEP1	1781	1543	-0.31567383	2.94E-10	3.87E-10
KBTBD6	1782	781	-1.298827259	4.64E-106	1.88E-105
C11orf57	1782	1148	-0.743099071	3.38E-43	7.52E-43
DPP8	1782	1174	-0.710789304	4.24E-40	9.07E-40
KIAA0907	1782	1188	-0.693686877	1.72E-38	3.59E-38
ZNF280D	1783	916	-1.069611575	1.99E-78	6.29E-78
MYO1E	1784	1831	-0.0712082	0.137715618	0.142326795
PPP1R8	1785	1059	-0.861945861	2.39E-55	6.11E-55
SIDT2	1785	1303	-0.562811366	4.43E-27	7.93E-27
SMAD9	1787	473	-2.026351922	2.23E-199	1.72E-198
ARFGAP2	1788	865	-1.156299075	6.24E-89	2.17E-88
IDI1	1788	1686	-0.193466576	7.73E-05	8.81E-05
MBD6	1790	732	-1.39876841	4.14E-119	1.85E-118
MYO9A	1790	981	-0.976358922	5.16E-68	1.49E-67
RNF26	1791	1322	-0.546767536	7.26E-26	1.27E-25
ZFAND6	1794	1244	-0.636917781	1.51E-33	2.97E-33
ZNF384	1796	959	-1.013909006	1.63E-72	4.90E-72
NAGLU	1797	1577	-0.297132125	2.26E-09	2.92E-09
RGS2	1798	638	-1.603489068	5.35E-146	2.91E-145
CSNK1G1	1799	1005	-0.948724061	3.07E-65	8.61E-65
PHF23	1801	1184	-0.713853476	8.42E-41	1.82E-40
NUPL1	1803	1387	-0.487155985	2.12E-21	3.46E-21
SNX29	1804	891	-1.126426378	4.24E-86	1.44E-85
S1PR1	1809	126	-3.952421145	0	0
KIAA1279	1809	1351	-0.529889109	9.34E-25	1.61E-24
RRAGC	1810	874	-1.159008888	2.48E-90	8.73E-90
DGKZ	1810	883	-1.14422873	1.56E-88	5.40E-88
ZNF33A	1814	535	-1.870288035	3.53E-182	2.48E-181
ZNF518A	1814	549	-1.833020777	2.54E-177	1.72E-176
ZCRB1	1814	1298	-0.591608448	6.39E-30	1.19E-29
PTPRJ	1815	705	-1.472998761	2.44E-130	1.18E-129

RCBTB1	1815	808	-1.276266726	3.86E-105	1.55E-104
INTS6	1815	1457	-0.425693047	3.86E-17	5.86E-17
LAMTOR5	1815	1534	-0.351395441	1.95E-12	2.68E-12
TSPAN14	1816	1096	-0.83724078	1.22E-53	3.05E-53
FKBP3	1819	1196	-0.713652529	3.58E-41	7.77E-41
SYVN1	1819	1351	-0.537842244	1.60E-25	2.80E-25
MAP2K4	1820	1053	-0.89815739	2.43E-60	6.52E-60
SLC16A7	1821	614	-1.677144737	1.60E-157	9.38E-157
ADAMTSL4	1821	1656	-0.245752625	5.13E-07	6.22E-07
CLPX	1823	999	-0.976482354	3.01E-69	8.79E-69
MCU	1823	1622	-0.277265118	1.72E-08	2.17E-08
MTFR1L	1824	985	-0.997634476	1.01E-71	3.00E-71
TARSL2	1825	1265	-0.637483455	3.78E-34	7.50E-34
SLFN12	1826	877	-1.166762393	4.54E-92	1.64E-91
SNRPE	1826	1163	-0.759560044	7.17E-46	1.65E-45
MICU1	1834	1545	-0.356111177	7.84E-13	1.09E-12
MINPP1	1835	1119	-0.822294403	1.36E-52	3.37E-52
MED24	1836	1141	-0.794991643	9.41E-50	2.27E-49
SIX4	1836	1625	-0.284850717	6.45E-09	8.22E-09
C16orf58	1837	1419	-0.481201413	2.53E-21	4.11E-21
PPP2R5A	1839	877	-1.176997108	5.53E-94	2.03E-93
TMEM159	1842	883	-1.169512094	3.35E-93	1.22E-92
EPS8	1843	839	-1.244037731	1.26E-102	4.98E-102
GGA2	1843	1010	-0.976425154	5.57E-70	1.64E-69
PSMC6	1843	1437	-0.467720385	2.21E-20	3.54E-20
MEIS2	1844	674	-1.560742535	5.30E-144	2.82E-143
AGO4	1846	643	-1.630236286	2.10E-153	1.20E-152
RSRC2	1851	847	-1.236595396	4.10E-102	1.61E-101
ZNF652	1851	877	-1.186380523	9.21E-96	3.44E-95
DEF8	1851	1230	-0.698370956	2.27E-40	4.87E-40
B3GALNT2	1853	993	-1.008721634	3.84E-74	1.17E-73
UBQLN4	1854	1033	-0.952525366	1.29E-67	3.70E-67

EFNB2	1855	1685	-0.247394971	3.39E-07	4.12E-07
TMX2	1856	1356	-0.561563908	5.50E-28	9.99E-28
ETS1	1860	951	-1.076509751	1.42E-82	4.68E-82
DVL2	1861	909	-1.142450232	9.40E-91	3.33E-90
CHST3	1865	1009	-0.994973832	5.59E-73	1.69E-72
HMG20A	1865	1117	-0.848270821	3.04E-56	7.81E-56
C17orf51	1865	1940	-0.051843354	0.267574668	0.273672637
VIMP	1868	1787	-0.172679197	0.000295619	0.00033187
FAM155A	1869	957	-1.074400115	1.05E-82	3.46E-82
PPP1CA	1870	1530	-0.398230993	9.69E-16	1.43E-15
RPTOR	1874	1079	-0.905150464	7.17E-63	1.97E-62
ACOX1	1876	1264	-0.67838774	6.12E-39	1.29E-38
PPP2R3C	1878	931	-1.121068366	6.98E-89	2.43E-88
CDR2	1882	1632	-0.314349947	1.10E-10	1.46E-10
EFHA1	1883	1148	-0.822634734	5.86E-54	1.48E-53
TRAF3	1886	829	-1.294610045	1.39E-111	5.87E-111
ZBED5	1887	1041	-0.96684873	1.79E-70	5.30E-70
POGK	1888	1315	-0.630520341	1.28E-34	2.55E-34
SLC35D1	1890	1191	-0.774937197	4.96E-49	1.19E-48
ATP6V0D1	1891	1302	-0.647144291	3.12E-36	6.33E-36
STRN3	1892	1302	-0.647907016	2.53E-36	5.15E-36
ANAPC16	1893	1050	-0.959009459	9.17E-70	2.69E-69
CHURC1	1898	669	-1.613126252	2.52E-155	1.45E-154
SYNE2	1902	160	-3.680097812	0	0
SPAG7	1903	1222	-0.747755652	1.70E-46	3.95E-46
SPATS2	1903	1624	-0.337448305	3.99E-12	5.46E-12
SEPHS1	1905	614	-1.742204813	1.21E-173	7.94E-173
LRRC47	1905	1209	-0.764701129	2.54E-48	6.04E-48
ZNF592	1908	998	-1.043673827	1.42E-80	4.59E-80
ALDH3B1	1909	701	-1.554055129	4.34E-148	2.39E-147
NFYB	1909	995	-1.048773048	3.02E-81	9.83E-81
GOLGA8B	1915	633	-1.705791363	1.64E-169	1.06E-168

ARID4A	1915	815	-1.341196804	1.65E-119	7.43E-119
GNPAT	1917	1169	-0.822299783	7.29E-55	1.85E-54
SPATA13	1918	718	-1.526271347	6.03E-145	3.24E-144
OTUD7B	1918	920	-1.16862133	7.49E-97	2.82E-96
OTUB1	1920	1368	-0.597762457	3.93E-32	7.58E-32
SLC12A6	1921	1074	-0.947587902	2.10E-69	6.15E-69
RRN3	1925	1105	-0.909536452	4.80E-65	1.35E-64
AK5	1925	1928	-0.10647777	0.021931167	0.023259607
RALGAPA1	1927	748	-1.473970772	2.15E-138	1.10E-137
HPS1	1928	973	-1.095317717	6.58E-88	2.28E-87
DHX8	1931	1000	-1.05807254	2.51E-83	8.29E-83
ANKRD13C	1932	924	-1.172854713	4.25E-98	1.62E-97
EEF2K	1932	1459	-0.513839587	5.52E-25	9.56E-25
TRAPPC1	1932	1839	-0.17989799	0.000128319	0.000145498
EYA3	1933	868	-1.263799066	3.53E-110	1.47E-109
KAZN	1935	1124	-0.892415907	2.28E-63	6.30E-63
TPCN1	1936	1157	-0.851414464	1.13E-58	2.98E-58
SETDB2	1937	768	-1.443370114	7.18E-135	3.56E-134
PHF12	1938	1049	-0.994278269	1.12E-75	3.46E-75
TIMM23	1938	1509	-0.469700141	1.67E-21	2.72E-21
SGSH	1939	835	-1.324189077	1.14E-118	5.11E-118
RAB4A	1939	1000	-1.064037179	2.03E-84	6.81E-84
MDM4	1942	799	-1.390000168	7.52E-128	3.57E-127
COG6	1942	2540	0.27856092	1.22E-10	1.62E-10
ZNF326	1944	431	-2.28199282	3.40E-251	3.44E-250
MEX3B	1946	618	-1.763557343	2.45E-180	1.69E-179
UTP14C	1946	1323	-0.665423024	4.92E-39	1.04E-38
TMEM219	1947	1743	-0.26840469	1.62E-08	2.04E-08
IPW	1948	872	-1.268318013	1.25E-111	5.28E-111
NUP160	1949	919	-1.1933217	1.19E-101	4.67E-101
ERLIN1	1949	934	-1.169964011	1.46E-98	5.56E-98
CTCF	1952	806	-1.384825685	8.77E-128	4.16E-127

TBX5	1954	838	-1.330132694	2.19E-120	9.93E-120
MTMR4	1955	1242	-0.76322781	2.20E-49	5.30E-49
RAB11FIP2	1956	851	-1.309399709	1.14E-117	5.07E-117
SPOP	1956	1081	-0.964264223	1.15E-72	3.47E-72
GAS5	1956	1499	-0.492630363	1.60E-23	2.69E-23
INO80	1957	940	-1.16663547	1.61E-98	6.14E-98
EVL	1958	884	-1.255986866	1.60E-110	6.69E-110
PITRM1	1960	1371	-0.624349459	2.79E-35	5.60E-35
PHB	1960	1617	-0.386258351	1.34E-15	1.97E-15
CDC123	1963	1309	-0.693319451	3.46E-42	7.58E-42
ADAM15	1963	1486	-0.510350433	4.57E-25	7.93E-25
FIBP	1963	1538	-0.460729045	4.65E-21	7.53E-21
ITGBL1	1964	3885	0.875395293	1.08E-112	4.58E-112
IER5	1966	1526	-0.474232735	3.76E-22	6.20E-22
XYLT2	1967	1431	-0.567697661	4.11E-30	7.71E-30
NADK	1968	996	-1.091236949	3.66E-89	1.28E-88
FOPNL	1972	1027	-1.049947746	5.14E-84	1.71E-83
CEP290	1976	409	-2.381134575	1.85E-268	2.07E-267
LBR	1977	829	-1.36259324	2.73E-126	1.29E-125
ARHGAP12	1977	1126	-0.92083042	4.10E-68	1.19E-67
FAM21C	1978	873	-1.288713243	4.06E-116	1.78E-115
ZFYVE1	1979	1236	-0.787817245	1.10E-52	2.72E-52
SEPHS2	1979	1280	-0.737352178	3.68E-47	8.61E-47
FBXO28	1980	1142	-0.902662155	5.03E-66	1.43E-65
C11orf87	1981	107	-4.319270554	0	0
TEX2	1984	1712	-0.3214537	1.32E-11	1.79E-11
DDX47	1985	1260	-0.76443965	3.02E-50	7.34E-50
SFXN3	1987	1993	-0.104374538	0.022429394	0.023781552
TM7SF3	1989	1493	-0.522553487	2.02E-26	3.58E-26
ELOVL1	1989	1561	-0.458297115	3.99E-21	6.47E-21
RHOT2	1991	1386	-0.631290339	1.72E-36	3.52E-36
MMS19	1991	1721	-0.3189705	1.71E-11	2.31E-11

GPT2	1992	2245	0.063773422	0.150436935	0.15537125
TBX5-AS1	1994	869	-1.306961703	1.41E-119	6.36E-119
SYNJ2BP	1994	1158	-0.892754532	2.76E-65	7.78E-65
SF3B4	1995	1098	-0.970235068	8.00E-75	2.46E-74
KLF13	1995	1660	-0.373929881	5.22E-15	7.59E-15
RBM7	1997	1301	-0.726937747	1.89E-46	4.39E-46
PCK2	1998	2026	-0.088646785	0.051215961	0.053727222
LPHN2	2002	204	-3.403525293	0	0
NAV3	2007	4600	1.087868869	4.91E-190	3.60E-189
ATP6V1D	2007	1602	-0.433890845	2.08E-19	3.28E-19
MARCH5	2010	1198	-0.855291969	2.63E-61	7.13E-61
SUPT20H	2013	1331	-0.705560977	1.63E-44	3.70E-44
EMC7	2013	1906	-0.187523429	4.71E-05	5.40E-05
SIK3	2014	720	-1.592719248	9.90E-162	6.02E-161
SCN8A	2015	1451	-0.582456695	3.48E-32	6.72E-32
C17orf103	2017	1151	-0.918047626	4.12E-69	1.20E-68
THUMPD1	2017	1358	-0.67945198	7.58E-42	1.65E-41
DNAJC14	2020	1109	-0.973820303	3.42E-76	1.06E-75
TEP1	2020	1277	-0.770321144	9.23E-52	2.27E-51
NDUFB8	2021	1857	-0.230819882	6.31E-07	7.63E-07
BTRC	2022	1528	-0.51286283	5.47E-26	9.63E-26
TK2	2023	1148	-0.926098054	2.62E-70	7.73E-70
YIF1A	2026	2586	0.243363725	1.14E-08	1.44E-08
MTHFR	2029	981	-1.157168199	8.87E-101	3.44E-100
DPF2	2029	1162	-0.912883172	7.14E-69	2.08E-68
FAM192A	2031	1140	-0.941880791	1.49E-72	4.48E-72
DHX40	2034	1274	-0.783678777	1.26E-53	3.16E-53
SMARCD1	2039	1033	-1.089745897	4.13E-92	1.49E-91
VAMP2	2039	1191	-0.884412738	1.08E-65	3.04E-65
CEP57	2040	1189	-0.887544813	4.14E-66	1.18E-65
RNF6	2040	1384	-0.668449585	3.76E-41	8.16E-41
SMCR8	2041	1157	-0.927611694	4.16E-71	1.23E-70

CYLD	2041	1329	-0.727659454	1.63E-47	3.83E-47
CD55	2043	1387	-0.667445792	4.19E-41	9.08E-41
CD4	2046	2045	-0.109429678	0.01523506	0.016252812
SRGAP2	2049	951	-1.216127114	5.94E-110	2.47E-109
EXOSC10	2049	1039	-1.088448706	2.23E-92	8.09E-92
LARP6	2049	1513	-0.546232373	2.64E-29	4.88E-29
ECHS1	2051	1653	-0.419965143	9.91E-19	1.54E-18
DNM3OS	2053	1763	-0.328425529	2.16E-12	2.96E-12
DENND5B	2055	995	-1.155094339	9.29E-102	3.64E-101
JKAMP	2056	1233	-0.846391841	1.36E-61	3.70E-61
CASP1	2058	403	-2.461115614	2.36E-290	2.96E-289
MEX3A	2058	957	-1.213376528	4.86E-110	2.02E-109
PRKAB2	2058	1531	-0.535493075	1.91E-28	3.49E-28
TDG	2060	1563	-0.507050935	6.18E-26	1.09E-25
SEZ6L2	2061	2180	-0.027740746	0.531156883	0.537071617
NDUFB10	2062	1588	-0.485557796	4.16E-24	7.10E-24
FAM219B	2063	1173	-0.923265184	2.64E-71	7.85E-71
PFKM	2065	1175	-0.922205401	3.08E-71	9.15E-71
COPS7A	2065	1429	-0.639860241	1.17E-38	2.46E-38
ADIPOR2	2065	1519	-0.551744288	4.95E-30	9.28E-30
SART3	2067	1175	-0.923602008	1.76E-71	5.23E-71
MIB2	2067	1238	-0.84825145	3.89E-62	1.06E-61
GTPBP4	2069	1815	-0.297688473	1.31E-10	1.74E-10
PRKCDBP	2070	1620	-0.462361331	2.97E-22	4.91E-22
PCF11	2073	1046	-1.095561641	2.08E-94	7.65E-94
ANP32E	2073	1544	-0.53377174	1.75E-28	3.20E-28
PATL1	2074	1343	-0.735680965	3.76E-49	9.00E-49
FAM212B	2076	817	-1.454122836	5.37E-146	2.91E-145
CYB5B	2079	1397	-0.682282113	2.20E-43	4.91E-43
PPP2R5E	2079	1433	-0.645575524	1.68E-39	3.57E-39
PRPF19	2080	1155	-0.957415053	2.89E-76	9.01E-76
ERGIC2	2081	1571	-0.51431816	7.91E-27	1.41E-26

SUV420H1	2085	1070	-1.071160963	1.33E-91	4.76E-91
LRIG3	2086	452	-2.315068856	4.69E-274	5.40E-273
POLR1D	2089	1409	-0.676865257	5.33E-43	1.19E-42
GAS7	2091	826	-1.448703751	3.15E-146	1.72E-145
DNAJB12	2093	1062	-1.087512921	3.43E-94	1.26E-93
MRPS16	2093	1283	-0.814775517	7.91E-59	2.10E-58
TMEM132A	2093	1854	-0.283655444	6.70E-10	8.75E-10
KIAA2013	2093	2054	-0.135860506	0.00241842	0.002646131
DDIT4	2098	1599	-0.500569115	8.26E-26	1.45E-25
CCNK	2098	1612	-0.48888731	8.68E-25	1.50E-24
PSMB6	2099	1841	-0.297936916	9.35E-11	1.24E-10
PAN3	2100	787	-1.524678163	2.87E-158	1.69E-157
PRKAG1	2103	1599	-0.504003287	3.59E-26	6.33E-26
MTMR2	2103	1687	-0.426713252	1.08E-19	1.71E-19
C2CD5	2105	756	-1.58608647	6.11E-168	3.89E-167
STXBP3	2106	1068	-1.088318165	7.08E-95	2.62E-94
TENM4	2106	1549	-0.551892668	1.31E-30	2.48E-30
HCFC2	2110	1127	-1.013479859	8.32E-85	2.81E-84
TSG101	2110	1432	-0.667935882	1.90E-42	4.19E-42
BMS1	2110	1579	-0.526956204	2.48E-28	4.52E-28
UAP1	2111	3636	0.675701245	4.15E-68	1.20E-67
NDUFV1	2111	1486	-0.615216839	6.28E-37	1.29E-36
EDC4	2112	1416	-0.685512945	2.13E-44	4.82E-44
FAM177A1	2112	1807	-0.333737704	4.73E-13	6.59E-13
RAP1A	2114	1620	-0.49270594	2.68E-25	4.66E-25
GATAD2B	2119	912	-1.325002234	1.27E-129	6.11E-129
CSRNP2	2119	1240	-0.881767843	7.29E-68	2.10E-67
RAB21	2119	1482	-0.624562516	5.02E-38	1.04E-37
AGL	2121	889	-1.363213673	1.94E-135	9.66E-135
UBE2N	2123	1578	-0.536731542	2.09E-29	3.87E-29
MICAL2	2125	4123	0.847507244	3.51E-113	1.50E-112
LAMTOR1	2126	1361	-0.752198906	2.97E-52	7.34E-52

KCNMA1	2129	1387	-0.726932538	2.11E-49	5.07E-49
TCTN3	2129	1452	-0.660858872	4.77E-42	1.05E-41
POLR2C	2129	1784	-0.36378471	3.42E-15	5.00E-15
STAT5B	2130	1135	-1.016885509	4.68E-86	1.59E-85
C14orf2	2130	2039	-0.171716031	0.000121194	0.000137518
FAM111A	2131	899	-1.353861948	1.16E-134	5.72E-134
ZNF362	2131	926	-1.31117087	2.77E-128	1.32E-127
AHSA1	2132	1586	-0.535539043	2.06E-29	3.82E-29
SPPL3	2133	1046	-1.13672549	6.41E-103	2.53E-102
TOB1	2136	1437	-0.680575961	2.45E-44	5.54E-44
TLK2	2137	1115	-1.047267574	1.63E-90	5.76E-90
CBFB	2138	1334	-0.789227562	6.04E-57	1.57E-56
AMZ2	2138	1644	-0.48777593	3.94E-25	6.85E-25
FAT3	2141	371	-2.63751808	0	0
MYL6B	2142	1750	-0.400327934	5.77E-18	8.88E-18
BTG2	2146	1139	-1.022606705	1.78E-87	6.15E-87
HNRNPUL2	2146	1296	-0.836308734	6.06E-63	1.66E-62
LSM12	2147	1588	-0.543835655	2.13E-30	4.02E-30
IDH2	2147	1621	-0.514162476	1.30E-27	2.35E-27
ARID2	2148	941	-1.299451741	1.56E-127	7.38E-127
RPS6KB1	2148	1235	-0.907207327	4.62E-72	1.38E-71
PLEKHA1	2150	1150	-1.011427175	4.27E-86	1.46E-85
ELK3	2150	1656	-0.485358363	4.76E-25	8.25E-25
SPTSSA	2151	1301	-0.834110939	8.23E-63	2.25E-62
LGMN	2151	2068	-0.165495715	0.000194005	0.000218802
LIN7C	2152	1313	-0.821535538	2.85E-61	7.70E-61
CMTM4	2153	1657	-0.486499093	3.50E-25	6.09E-25
FAM63B	2154	1554	-0.579756122	5.12E-34	1.01E-33
UPF3A	2155	1258	-0.885280323	1.94E-69	5.67E-69
MESDC2	2158	1733	-0.425147586	4.85E-20	7.71E-20
CDK17	2159	1564	-0.573847107	1.68E-33	3.30E-33
DENND4A	2161	1153	-1.015030935	5.04E-87	1.73E-86

MEAF6	2163	1171	-0.994016965	3.29E-84	1.10E-83
OGFOD1	2165	2069	-0.174157756	8.54E-05	9.74E-05
ASAP3	2170	1004	-1.220660149	5.28E-117	2.32E-116
SUZ12	2170	1135	-1.043727121	2.19E-91	7.82E-91
SLC11A2	2172	1770	-0.403999119	1.76E-18	2.74E-18
TMEM87A	2173	1273	-0.880180131	2.44E-69	7.12E-69
OSBPL9	2173	1522	-0.622444192	1.00E-38	2.11E-38
CARD16	2174	734	-1.675224348	2.32E-187	1.68E-186
TIPRL	2175	1497	-0.647665555	1.79E-41	3.91E-41
AEN	2175	1659	-0.499425891	1.36E-26	2.42E-26
TECPR2	2176	1280	-0.874259122	1.16E-68	3.36E-68
RRM1	2178	1124	-1.063086294	1.87E-94	6.90E-94
WBP2	2178	1796	-0.38694098	3.24E-17	4.92E-17
TMEM8A	2181	1629	-0.529727542	1.71E-29	3.17E-29
TMPO	2182	1076	-1.1286974	4.54E-104	1.82E-103
FAM134C	2184	1246	-0.918393164	1.00E-74	3.08E-74
NEGR1	2184	1857	-0.342723417	4.65E-14	6.61E-14
EVA1B	2186	1636	-0.526845029	2.78E-29	5.15E-29
NBPF1	2187	916	-1.364258093	9.45E-140	4.88E-139
RPA1	2187	1336	-0.819757588	5.14E-62	1.40E-61
PHRF1	2188	1294	-0.866499497	5.06E-68	1.46E-67
CARKD	2190	1097	-1.10609172	3.74E-101	1.46E-100
BAHCC1	2193	610	-1.95474904	4.71E-233	4.37E-232
FLOT2	2193	1505	-0.651866701	2.90E-42	6.39E-42
BAI2	2195	931	-1.346092243	1.90E-137	9.60E-137
RAB35	2199	1070	-1.147961183	1.06E-107	4.36E-107
ZBED6	2200	1345	-0.818621727	3.14E-62	8.56E-62
TMBIM4	2203	1157	-1.037805006	6.66E-92	2.39E-91
HAX1	2206	1872	-0.345576732	2.20E-14	3.15E-14
ULK2	2207	872	-1.448410965	3.49E-154	2.00E-153
CD82	2208	2760	0.213203719	2.06E-07	2.52E-07
FBRS	2209	1389	-0.778071195	2.16E-57	5.65E-57

WIPI1	2209	2593	0.122504412	0.003296709	0.003593524
BFAR	2212	1668	-0.515956473	1.44E-28	2.63E-28
PGM1	2213	2293	-0.057491472	0.180911501	0.186254174
XPO4	2214	1214	-0.975611176	1.41E-83	4.67E-83
CHST14	2214	1350	-0.822420191	4.25E-63	1.17E-62
C1RL	2220	963	-1.313676349	6.02E-134	2.97E-133
WBP11	2223	1389	-0.787185725	7.00E-59	1.86E-58
GXYLT1	2228	856	-1.488790907	4.65E-162	2.83E-161
UBN1	2230	1649	-0.544176687	1.53E-31	2.92E-31
RBM26	2233	1012	-1.250498337	6.36E-125	2.97E-124
NSF	2233	2078	-0.212511973	1.32E-06	1.59E-06
CDC42EP4	2238	746	-1.693686877	8.45E-196	6.39E-195
TMEM9B	2238	1454	-0.730907143	2.55E-52	6.32E-52
CLCN7	2239	1208	-0.998958449	8.38E-88	2.89E-87
MRPL51	2241	2076	-0.219060581	6.08E-07	7.36E-07
SLC25A29	2244	1254	-0.948259704	7.47E-81	2.42E-80
SLC35E2	2245	821	-1.559985694	9.38E-175	6.26E-174
TMEM109	2245	929	-1.381689319	3.63E-146	1.97E-145
BAZ1A	2246	1017	-1.251762624	7.80E-126	3.67E-125
TRIM25	2246	1268	-0.933527558	7.04E-79	2.23E-78
TNFRSF12A	2247	2450	0.016057249	0.702922641	0.70790855
PRICKLE1	2248	1082	-1.163665912	1.98E-112	8.38E-112
SOCS4	2248	1419	-0.772491822	1.13E-57	2.96E-57
PSMC5	2251	1595	-0.605734009	3.10E-38	6.47E-38
DUSP10	2252	434	-2.484164256	0	0
KIAA0195	2252	969	-1.325362633	1.09E-137	5.52E-137
ZNF37A	2258	811	-1.585996042	5.53E-180	3.82E-179
PCSK7	2262	3135	0.362142138	5.03E-20	7.99E-20
MAP3K3	2265	865	-1.497463388	4.01E-166	2.51E-165
SIAE	2265	1159	-1.07535486	5.76E-100	2.22E-99
GNB5	2267	951	-1.361991521	1.99E-144	1.07E-143
CEP170B	2267	1615	-0.597974602	1.18E-37	2.44E-37

NPC2	2267	2037	-0.263062787	2.24E-09	2.89E-09
JDP2	2271	1176	-1.058164022	1.12E-97	4.25E-97
LARP4	2271	1471	-0.735254835	1.34E-53	3.37E-53
IL6R	2274	168	-3.867423492	0	0
NCAPD2	2280	1244	-0.982771715	4.85E-87	1.67E-86
PDS5B	2289	1031	-1.259397506	2.06E-129	9.89E-129
PDCD11	2291	1476	-0.743009113	5.09E-55	1.30E-54
TM9SF1	2294	1771	-0.482025555	2.65E-26	4.68E-26
ZBTB41	2299	1010	-1.295375549	1.05E-135	5.21E-135
PML	2299	2853	0.202748905	4.88E-07	5.92E-07
PPRC1	2301	1833	-0.436778574	2.86E-22	4.72E-22
MTCH2	2302	1856	-0.419415499	8.78E-21	1.41E-20
ATL3	2303	1216	-1.030095558	7.64E-95	2.83E-94
ZBTB40	2304	893	-1.476133012	1.97E-165	1.23E-164
PIAS3	2305	2047	-0.279980024	1.57E-10	2.08E-10
ANXA7	2307	1854	-0.424101136	3.17E-21	5.15E-21
DGKA	2308	1962	-0.343042558	8.54E-15	1.23E-14
MFAP1	2309	1512	-0.719534409	1.64E-52	4.06E-52
C12orf5	2310	1662	-0.583696845	8.80E-37	1.81E-36
PACS2	2314	1145	-1.123765642	1.74E-109	7.21E-109
LIPA	2314	1493	-0.740899075	2.74E-55	7.01E-55
NCKAP5L	2316	1698	-0.55652317	5.61E-34	1.11E-33
FZD4	2319	712	-1.81227805	1.20E-222	1.05E-221
AQR	2319	1246	-1.004923128	9.43E-92	3.38E-91
CREBL2	2320	1238	-1.014837867	2.94E-93	1.07E-92
TUB	2320	1375	-0.863417563	1.37E-71	4.06E-71
ZNF84	2325	994	-1.334637335	1.34E-143	7.11E-143
LTA4H	2326	1734	-0.532471574	1.27E-31	2.43E-31
DNTTIP2	2333	1273	-0.982678264	5.25E-89	1.83E-88
TULP3	2337	1306	-0.948227213	4.03E-84	1.34E-83
SART1	2337	1601	-0.654408802	3.19E-45	7.29E-45
GOSR1	2340	1565	-0.689070249	1.96E-49	4.72E-49

BANF1	2344	1696	-0.575560776	2.07E-36	4.22E-36
UFC1	2348	1519	-0.737034914	1.40E-55	3.58E-55
SCCPDH	2349	1252	-1.016536527	1.18E-94	4.36E-94
MIR22HG	2349	2559	0.014809058	0.719183587	0.723911398
DDX50	2354	1388	-0.870831128	1.20E-73	3.65E-73
ACTR10	2356	1500	-0.760101414	9.75E-59	2.58E-58
TRNP1	2356	1682	-0.594886209	1.01E-38	2.13E-38
CHAMP1	2357	1668	-0.607556846	3.68E-40	7.88E-40
NUDC	2357	1878	-0.436479071	9.54E-23	1.58E-22
SNX27	2359	1014	-1.326842142	2.21E-144	1.18E-143
B4GALT2	2359	2170	-0.229204752	9.00E-08	1.11E-07
HPS5	2360	1103	-1.206078445	1.01E-124	4.67E-124
KIAA0247	2363	1602	-0.669469857	1.64E-47	3.84E-47
NUTF2	2364	1874	-0.443833458	1.79E-23	3.01E-23
IL4R	2369	2156	-0.244645396	1.18E-08	1.49E-08
CCNT1	2370	1317	-0.956356089	1.77E-86	6.04E-86
UBXN1	2371	1592	-0.683379703	2.30E-49	5.53E-49
GBP1	2372	2587	0.016451668	0.688054655	0.693024481
SNW1	2374	1451	-0.818996791	4.67E-67	1.34E-66
HN1	2375	1520	-0.752580566	3.26E-58	8.57E-58
NDUFS2	2375	1627	-0.654437638	6.21E-46	1.43E-45
GNG5	2376	2776	0.115748356	0.004024331	0.004373834
VPS51	2378	1585	-0.693990251	8.10E-51	1.98E-50
GTF2H1	2380	1167	-1.136881388	1.23E-114	5.32E-114
VAMP4	2385	1217	-1.079384474	8.64E-106	3.49E-105
SPECC1	2386	1833	-0.489111633	5.48E-28	9.95E-28
RAP2A	2387	1208	-1.091302488	9.54E-108	3.91E-107
SPG21	2391	1306	-0.981183609	6.02E-91	2.14E-90
FAM69A	2392	1508	-0.774305337	1.71E-61	4.65E-61
SH2B3	2393	1097	-1.233981247	5.19E-131	2.52E-130
PPP1R14B	2394	2274	-0.182915274	1.48E-05	1.72E-05
GOLGA8A	2395	725	-1.832697132	2.15E-233	2.00E-232

ATXN7L3	2395	1647	-0.648909477	1.22E-45	2.80E-45
ARHGAP29	2397	1212	-1.092564586	2.17E-108	8.96E-108
FOXJ3	2397	1374	-0.911572281	7.79E-81	2.52E-80
FAM107B	2399	981	-1.398832492	5.76E-159	3.42E-158
GALC	2406	1110	-1.224801342	3.39E-130	1.64E-129
ACAP3	2407	925	-1.488435247	7.45E-175	4.98E-174
UBAC2	2408	1373	-0.919228143	2.54E-82	8.35E-82
SOS2	2410	1022	-1.346362326	9.25E-151	5.16E-150
ETFA	2410	1983	-0.390072845	3.80E-19	5.95E-19
NARG2	2412	1094	-1.249341545	1.38E-134	6.84E-134
ZFYVE9	2413	1431	-0.862528619	2.93E-74	8.96E-74
AGO1	2414	1416	-0.878328787	1.48E-76	4.62E-76
ZNF37BP	2416	608	-2.099201602	3.38E-281	4.02E-280
GPR161	2419	1028	-1.343294881	8.55E-151	4.77E-150
CSF1	2420	788	-1.727463889	1.87E-217	1.59E-216
TCEB3	2421	1664	-0.649672022	3.29E-46	7.63E-46
PPP1R15B	2430	1648	-0.668964447	9.54E-49	2.28E-48
NDEL1	2430	2516	-0.058548768	0.153421817	0.158370253
MTMR6	2435	1752	-0.583643373	1.13E-38	2.38E-38
CACNA1C	2436	534	-2.298326862	0	0
IL16	2438	536	-2.294117596	0	0
RC3H1	2441	918	-1.519630612	9.41E-183	6.64E-182
TXNDC12	2441	2315	-0.185184478	9.55E-06	1.12E-05
ABHD4	2444	1540	-0.77503831	6.87E-63	1.88E-62
SPG7	2445	1258	-1.067426919	1.73E-106	7.03E-106
GUK1	2446	2083	-0.34048594	2.16E-15	3.17E-15
HEATR1	2451	1219	-1.116396734	1.19E-114	5.14E-114
UBASH3B	2456	3305	0.319615335	6.00E-17	9.06E-17
FCHSD2	2457	2218	-0.256362868	1.25E-09	1.62E-09
TMX1	2461	1345	-0.980362861	2.00E-93	7.29E-93
CD9	2462	1425	-0.897593218	6.87E-81	2.22E-80
ADSS	2462	1577	-0.751372478	4.08E-60	1.09E-59

LEPREL4	2462	3546	0.417637399	8.81E-29	1.62E-28
FAS	2466	1025	-1.375273266	3.07E-159	1.83E-158
UPF2	2471	1337	-0.994819921	4.70E-96	1.76E-95
DNAJB4	2471	2053	-0.376085759	2.10E-18	3.26E-18
STX2	2472	1534	-0.797104636	1.17E-66	3.34E-66
ERRFI1	2472	1755	-0.602932089	1.73E-41	3.77E-41
HIAT1	2474	1423	-0.906634214	1.25E-82	4.12E-82
AASDHPPT	2477	1310	-1.027751433	1.72E-101	6.73E-101
ABI1	2477	1538	-0.796262741	1.14E-66	3.25E-66
SOAT1	2479	1323	-1.014669586	1.72E-99	6.59E-99
UNC119B	2479	1366	-0.968525164	2.98E-92	1.08E-91
FTSJ3	2479	1643	-0.702150167	5.82E-54	1.47E-53
EPN2	2480	1664	-0.684409063	1.10E-51	2.71E-51
AK2	2481	1959	-0.449528714	4.21E-25	7.32E-25
MAN2A2	2482	1118	-1.259307303	3.85E-140	2.00E-139
TMOD2	2485	764	-1.810325684	4.37E-238	4.16E-237
PSME1	2486	1499	-0.838550289	5.99E-73	1.81E-72
UBR1	2487	1593	-0.751384617	1.04E-60	2.80E-60
NUP133	2488	1360	-0.98010421	2.17E-94	7.99E-94
EFTUD2	2491	1646	-0.706485062	8.77E-55	2.23E-54
APBB1	2493	1295	-1.053655161	2.63E-106	1.07E-105
KANSL1	2495	1081	-1.315397668	1.36E-150	7.61E-150
MCMBP	2496	1169	-1.20306738	2.77E-131	1.35E-130
OXA1L	2497	1510	-0.834371648	1.18E-72	3.54E-72
C11orf24	2497	2755	0.033132122	0.405401734	0.411514642
ZCCHC14	2499	1104	-1.287335105	6.10E-146	3.31E-145
CDKN1B	2502	951	-1.504288919	1.63E-184	1.16E-183
ZNF644	2503	1213	-1.153803116	2.67E-123	1.23E-122
ULK1	2503	1411	-0.935664679	5.11E-88	1.77E-87
UBE2J2	2507	1598	-0.758418964	3.75E-62	1.02E-61
GNPNAT1	2509	2388	-0.18003401	1.26E-05	1.46E-05
DYNC2H1	2515	476	-2.510249297	0	0

CACUL1	2520	1886	-0.526818434	1.70E-33	3.32E-33
CDK5RAP3	2523	1438	-0.919800906	2.99E-86	1.02E-85
ELMSAN1	2526	1148	-1.246456373	2.38E-140	1.24E-139
VPS53	2526	1694	-0.68514514	1.05E-52	2.60E-52
BRD7	2527	1418	-0.942292508	7.06E-90	2.48E-89
MGST3	2527	2008	-0.440390771	1.20E-24	2.07E-24
STRIP1	2535	1102	-1.310585899	4.03E-152	2.27E-151
SRSF4	2535	1471	-0.893912877	1.14E-82	3.75E-82
BMPR1A	2536	1228	-1.154968561	4.25E-125	1.99E-124
CRAMP1L	2537	1057	-1.371872519	3.71E-163	2.29E-162
SGMS1	2538	846	-1.693686877	9.11E-222	7.95E-221
LOC10050721	2547	1290	-1.09015227	1.20E-114	5.22E-114
TUBGCP2	2551	1776	-0.631155693	3.00E-46	6.96E-46
IVD	2558	1445	-0.932671147	1.94E-89	6.78E-89
ENSA	2558	1674	-0.720441112	4.18E-58	1.10E-57
CORO1B	2558	1964	-0.48994571	5.66E-30	1.06E-29
PLXNC1	2560	250	-3.464868186	0	0
MRGPRF	2560	1318	-1.066517816	2.96E-111	1.24E-110
CDK4	2560	1509	-0.87127538	5.36E-80	1.72E-79
BNIP3	2562	4444	0.685863965	5.45E-85	1.84E-84
SF3A3	2562	1783	-0.631688149	1.66E-46	3.85E-46
FBXO21	2564	1186	-1.221016628	5.96E-138	3.04E-137
HS2ST1	2567	1421	-0.961901117	2.14E-94	7.87E-94
PNMA1	2567	1540	-0.845877321	2.31E-76	7.19E-76
DAP3	2570	1853	-0.580629854	2.32E-40	4.98E-40
IGDCC4	2571	1519	-0.867932116	8.02E-80	2.56E-79
FMN1	2572	1303	-1.089777935	1.09E-115	4.76E-115
WDTC1	2572	1527	-0.860914956	8.76E-79	2.78E-78
FBXL3	2576	1261	-1.139288694	2.49E-124	1.15E-123
RAB11FIP3	2581	1171	-1.248913441	8.46E-144	4.50E-143
TNFSF4	2582	5965	1.099308761	5.56E-250	5.60E-249
SMG7	2582	1457	-0.934212499	1.66E-90	5.88E-90

SERPING1	2583	545	-2.353443884	0	0
PLCD3	2586	1936	-0.526367698	2.80E-34	5.55E-34
PSMD11	2586	1997	-0.481612318	2.13E-29	3.94E-29
LLGL1	2589	1441	-0.954049006	6.36E-94	2.33E-93
ATF7IP	2589	1531	-0.866645058	3.60E-80	1.16E-79
TRAFD1	2593	1566	-0.836262369	1.16E-75	3.58E-75
C16orf62	2598	1780	-0.654248565	4.69E-50	1.14E-49
RDH11	2600	2832	0.014585266	0.709487714	0.714243852
PRPF38B	2601	1284	-1.127145573	2.08E-123	9.58E-123
AHCTF1	2601	1461	-0.940834597	3.21E-92	1.16E-91
ZNF629	2604	1453	-0.950419121	7.21E-94	2.64E-93
FAM101B	2605	2080	-0.43342422	1.14E-24	1.97E-24
ZZZ3	2608	1494	-0.912488097	6.27E-88	2.17E-87
CHTF8	2610	1483	-0.924255585	7.18E-90	2.52E-89
ITGB3	2614	2892	0.037084035	0.340348504	0.346606868
SPPL2A	2618	1205	-1.228156327	4.28E-142	2.25E-141
ZNHIT6	2619	1522	-0.891772077	5.22E-85	1.76E-84
CMTM3	2620	1116	-1.33995416	1.53E-162	9.40E-162
WSB2	2621	2160	-0.387810417	1.77E-20	2.83E-20
VPS4A	2623	1878	-0.590745121	2.12E-42	4.69E-42
SIKE1	2626	1523	-0.89467535	1.09E-85	3.69E-85
XPNPEP1	2626	2093	-0.436018981	4.11E-25	7.14E-25
TP53	2627	1503	-0.914295567	7.61E-89	2.65E-88
SLC9A1	2630	1869	-0.601520606	7.51E-44	1.69E-43
CD2BP2	2631	1466	-0.952450521	3.74E-95	1.39E-94
GADD45A	2631	1508	-0.911699196	1.46E-88	5.08E-88
PANX1	2631	1749	-0.697805335	1.47E-56	3.80E-56
KIAA0317	2633	1746	-0.701378338	4.28E-57	1.11E-56
UBE2G1	2634	1795	-0.661995877	9.35E-52	2.30E-51
WBP1L	2635	1047	-1.440265895	3.69E-182	2.59E-181
DIS3	2636	1917	-0.568224409	7.68E-40	1.64E-39
PRDM2	2638	1405	-1.01759881	3.04E-106	1.24E-105

CLNS1A	2640	1494	-0.930082157	7.97E-92	2.86E-91
EVI5	2643	1626	-0.809573543	5.12E-73	1.55E-72
HOXB3	2644	1323	-1.107633491	5.46E-122	2.50E-121
USP44	2647	1571	-0.86139939	4.21E-81	1.37E-80
APAF1	2647	1576	-0.856815036	2.19E-80	7.06E-80
RASAL2	2647	2128	-0.42358442	4.58E-24	7.80E-24
CNOT2	2656	1612	-0.829127779	2.35E-76	7.32E-76
OLR1	2657	1470	-0.962706449	8.99E-98	3.41E-97
DYNLL2	2660	1441	-0.993080286	5.82E-103	2.30E-102
DDX23	2661	1512	-0.924234747	1.38E-91	4.94E-91
RAB34	2663	2112	-0.44316697	3.60E-26	6.36E-26
CTSK	2665	1520	-0.918788585	7.82E-91	2.78E-90
UBE2Q1	2666	1550	-0.891132941	2.13E-86	7.28E-86
RNPS1	2667	1672	-0.782367353	1.49E-69	4.36E-69
ABL2	2667	3421	0.250475903	1.48E-11	2.00E-11
POLDIP2	2668	1766	-0.703997699	3.38E-58	8.89E-58
TUBB3	2676	965	-1.580201644	8.40E-212	6.88E-211
SPATA20	2676	1457	-0.985801614	2.45E-102	9.63E-102
C12orf57	2676	2268	-0.347381852	2.81E-17	4.27E-17
MIER1	2677	1424	-1.019392369	4.30E-108	1.77E-107
USP12	2677	1599	-0.852171576	1.52E-80	4.91E-80
PRR13	2681	1661	-0.799443522	1.71E-72	5.15E-72
PRKRIR	2684	1332	-1.119514965	6.39E-126	3.01E-125
PNPLA2	2684	1527	-0.922408985	4.63E-92	1.67E-91
FUBP1	2686	1935	-0.581850114	2.95E-42	6.50E-42
DDHD1	2687	1240	-1.224380577	4.31E-145	2.32E-144
KDM4A	2689	1394	-1.05656357	5.10E-115	2.21E-114
NECAP2	2690	1779	-0.705264038	7.66E-59	2.03E-58
WIPF2	2694	1505	-0.94871074	9.23E-97	3.47E-96
CSPG4	2695	1605	-0.856436352	9.48E-82	3.09E-81
MOV10	2695	1678	-0.792266933	9.13E-72	2.72E-71
ERO1L	2696	2318	-0.326664306	1.16E-15	1.71E-15

MAML2	2700	1610	-0.854623095	1.31E-81	4.27E-81
SLC16A1	2701	2760	-0.077549749	0.046928516	0.049269263
DMXL2	2702	856	-1.767069349	2.86E-250	2.88E-249
SHANK2	2702	920	-1.663046284	5.52E-230	5.04E-229
SDHD	2702	1873	-0.637401151	1.03E-49	2.48E-49
LZTS2	2703	1730	-0.75251385	5.44E-66	1.54E-65
KCNJ2	2704	136	-4.422140971	0	0
VPS36	2705	1508	-0.951716541	1.18E-97	4.49E-97
CHMP1A	2705	1602	-0.864478822	2.49E-83	8.23E-83
INPP5F	2707	2607	-0.163028681	3.79E-05	4.35E-05
NME4	2711	2625	-0.155232066	8.45E-05	9.63E-05
RHBDF1	2712	1455	-1.007062401	2.41E-107	9.84E-107
KLHDC2	2713	1386	-1.077686166	9.07E-120	4.09E-119
USP8	2713	1592	-0.877773088	1.02E-85	3.48E-85
MPP5	2714	2086	-0.488405939	1.58E-31	3.02E-31
NUCB2	2718	4133	0.495919532	5.18E-45	1.18E-44
C15orf52	2719	1851	-0.663495632	1.42E-53	3.56E-53
GTF2A2	2720	1668	-0.814211739	8.21E-76	2.54E-75
EGLN1	2724	1717	-0.77456104	8.23E-70	2.42E-69
HEXIM1	2731	1516	-0.957883936	1.28E-99	4.91E-99
SLC7A6	2733	1869	-0.656943267	6.19E-53	1.54E-52
ANKRD10	2734	1296	-1.185671901	2.22E-140	1.16E-139
USP5	2739	1863	-0.664745968	3.95E-54	9.97E-54
PI4K2A	2741	1514	-0.965061499	3.31E-101	1.29E-100
AEBP2	2749	1153	-1.36223877	9.40E-175	6.26E-174
DNAJA2	2752	1624	-0.869653213	1.37E-85	4.64E-85
SCFD1	2752	2199	-0.432357242	7.38E-26	1.29E-25
VPS26B	2755	1777	-0.741333013	1.52E-65	4.30E-65
PSMD7	2758	2305	-0.367580082	1.43E-19	2.25E-19
PSMD13	2758	2422	-0.296147968	1.55E-13	2.19E-13
BET1L	2760	2880	-0.047323831	0.217903916	0.223543311
RCOR3	2761	1623	-0.875252264	8.76E-87	3.00E-86

VMP1	2763	3298	0.146631461	7.80E-05	8.90E-05
PHC1	2764	1011	-1.559698994	1.28E-214	1.06E-213
SRP54	2768	2896	-0.043506716	0.256320797	0.262368476
TP53BP2	2771	1310	-1.189564275	5.52E-143	2.92E-142
LMO4	2772	1954	-0.613221166	1.11E-47	2.63E-47
COG4	2774	1642	-0.865238036	1.56E-85	5.29E-85
PHF21A	2779	1761	-0.766895301	5.34E-70	1.57E-69
MAN2C1	2783	1329	-1.17502418	7.48E-141	3.90E-140
ZC3H14	2783	1441	-1.058294949	2.76E-119	1.24E-118
NIPA2	2786	1869	-0.684653065	7.10E-58	1.86E-57
ARL6IP4	2786	1877	-0.678490984	5.47E-57	1.42E-56
GOT2	2799	2554	-0.240877338	1.01E-09	1.32E-09
FTO	2800	1765	-0.77448302	1.07E-71	3.20E-71
PSMC3	2802	2462	-0.29535057	1.15E-13	1.63E-13
MGA	2805	1416	-1.094903881	6.15E-127	2.91E-126
VEZT	2808	1979	-0.613495699	2.59E-48	6.16E-48
ATAD1	2810	1678	-0.852551791	1.63E-84	5.47E-84
GON4L	2811	1541	-0.975940968	1.17E-105	4.73E-105
PCNXL2	2816	795	-1.933344944	2.33E-294	3.00E-293
SETD1B	2816	1150	-1.400737849	1.27E-186	9.17E-186
FAM65A	2816	1545	-0.974764872	1.24E-105	4.99E-105
N4BP1	2819	1537	-0.983790689	2.40E-107	9.83E-107
CUL2	2830	1486	-1.038092313	1.46E-117	6.46E-117
DR1	2830	2132	-0.517318991	3.02E-36	6.14E-36
USP10	2830	2161	-0.497827357	6.56E-34	1.29E-33
SLTM	2831	1378	-1.147460237	5.42E-138	2.77E-137
RPRD2	2831	1427	-1.09705079	1.70E-128	8.12E-128
PYGL	2831	1867	-0.709314198	1.95E-62	5.32E-62
AKT3	2831	2045	-0.577935282	6.29E-44	1.41E-43
ANKRD40	2834	1485	-1.041101203	2.82E-118	1.26E-117
C11orf95	2835	919	-1.733936344	1.04E-255	1.07E-254
PHKB	2838	2163	-0.5005653	2.52E-34	5.00E-34

CCDC90B	2840	1719	-0.833045761	3.68E-82	1.20E-81
SESN2	2841	1223	-1.324698804	4.83E-173	3.17E-172
HEBP1	2841	1476	-1.053430486	7.85E-121	3.56E-120
DIP2B	2845	1329	-1.206811924	4.34E-150	2.41E-149
SUDS3	2854	1746	-0.817656152	5.10E-80	1.64E-79
GGNBP2	2857	1792	-0.781654775	2.91E-74	8.90E-74
SRP68	2857	2380	-0.372263839	1.15E-20	1.85E-20
VKORC1	2859	3872	0.328843957	1.39E-20	2.23E-20
PPP3CB	2862	1709	-0.852595651	4.73E-86	1.61E-85
POMGNT1	2862	1882	-0.71348142	9.84E-64	2.73E-63
PCGF5	2863	1414	-1.126469926	1.64E-135	8.15E-135
UNC45A	2863	1888	-0.709393282	3.91E-63	1.08E-62
VPS26A	2866	1353	-1.191601146	3.25E-148	1.79E-147
RBM17	2868	1711	-0.85392964	1.88E-86	6.42E-86
PEX19	2872	1666	-0.894391724	1.72E-93	6.29E-93
NOTCH2NL	2873	947	-1.709846038	4.11E-254	4.21E-253
TMED3	2873	4805	0.633254062	6.77E-80	2.17E-79
CALCOCO2	2873	1912	-0.696199845	2.36E-61	6.40E-61
BNIP2	2874	1652	-0.907570751	7.63E-96	2.85E-95
MYO18A	2874	1986	-0.641918815	2.07E-53	5.18E-53
COX8A	2879	2460	-0.335633849	2.07E-17	3.16E-17
ORMDL3	2881	2257	-0.460887618	3.65E-30	6.85E-30
CHID1	2888	2590	-0.26584302	9.29E-12	1.26E-11
RFX7	2896	1278	-1.288898142	4.04E-169	2.60E-168
DRAP1	2897	2587	-0.272004006	2.98E-12	4.10E-12
BUB3	2903	1873	-0.740918048	6.71E-69	1.95E-68
SLC25A24	2903	1988	-0.654951191	7.98E-56	2.05E-55
SRM	2906	3067	-0.030930912	0.407276615	0.413364061
RAB3GAP2	2908	1832	-0.775332142	1.58E-74	4.86E-74
SCYL1	2910	2601	-0.27067713	3.37E-12	4.61E-12
SSBP3	2911	1354	-1.213011476	9.35E-155	5.37E-154
KCNQ1OT1	2912	1355	-1.21244188	1.08E-154	6.16E-154

ALKBH5	2912	2476	-0.342723417	3.09E-18	4.78E-18
ANKLE2	2917	2463	-0.352793134	3.37E-19	5.28E-19
VWA5A	2918	1118	-1.492784071	1.91E-212	1.57E-211
ALDH9A1	2919	1790	-0.814238999	3.16E-81	1.03E-80
SNX33	2920	1451	-1.117645226	1.87E-136	9.36E-136
PPA1	2923	1744	-0.853774165	4.77E-88	1.65E-87
ZBTB1	2928	1713	-0.882114778	4.06E-93	1.48E-92
RPL36AL	2928	2092	-0.593757078	1.53E-47	3.60E-47
SOX6	2937	1047	-1.596806199	9.05E-236	8.53E-235
ATP11A	2940	1212	-1.387150832	6.36E-192	4.71E-191
NOMO1	2940	3334	0.072713573	0.046041598	0.048357611
UBE3B	2941	1579	-1.006019989	4.76E-116	2.08E-115
TBC1D16	2942	2086	-0.604782465	2.66E-49	6.38E-49
SERF2	2943	2889	-0.135441714	0.000335515	0.000376172
EPHB2	2947	1962	-0.695646395	8.42E-63	2.31E-62
SZT2	2952	1468	-1.116565129	1.02E-137	5.19E-137
NASP	2953	1205	-1.401872586	7.70E-196	5.83E-195
PLBD2	2959	1994	-0.678168663	2.30E-60	6.18E-60
GBP3	2963	1366	-1.225825519	4.41E-160	2.64E-159
PSMA5	2964	2064	-0.630826853	2.12E-53	5.29E-53
CTBP2	2966	1760	-0.861667545	1.07E-90	3.80E-90
GPBP1L1	2969	1885	-0.764136946	3.33E-74	1.02E-73
TOM1L2	2971	1965	-0.705143669	8.85E-65	2.48E-64
NDFIP2	2971	2149	-0.576007498	9.51E-46	2.19E-45
MTA1	2973	1822	-0.81512088	7.62E-83	2.51E-82
SC5DL	2973	2029	-0.659874974	7.25E-58	1.90E-57
CDC42SE1	2974	2030	-0.659649296	7.51E-58	1.97E-57
NOLC1	2976	2216	-0.534141021	3.34E-40	7.15E-40
ACBD6	2982	1344	-1.258471495	8.56E-168	5.44E-167
AP3M1	2982	2069	-0.636070988	1.74E-54	4.41E-54
ELF1	2985	993	-1.696589685	4.43E-261	4.70E-260
MGRN1	2986	2062	-0.642894209	1.45E-55	3.70E-55

PPP1R3C	2991	2313	-0.47958702	1.71E-33	3.35E-33
PSMB2	2992	2207	-0.547747921	3.26E-42	7.17E-42
EXTL2	2996	1479	-1.127139947	7.56E-142	3.97E-141
IPO8	2997	1667	-0.954989355	1.35E-108	5.58E-108
ATXN1L	3002	1440	-1.168579541	1.80E-150	1.00E-149
FRS2	3005	1437	-1.173029305	1.60E-151	8.95E-151
SEC14L1	3013	2624	-0.308157329	1.13E-15	1.66E-15
DAD1	3019	3250	-0.002355415	0.948460115	0.949676246
GABARAPL2	3023	2079	-0.648815597	3.99E-57	1.04E-56
PSMA6	3023	2515	-0.374146955	5.77E-22	9.50E-22
ZFP91	3027	2298	-0.506234253	3.20E-37	6.60E-37
RELA	3028	2088	-0.644967869	1.23E-56	3.19E-56
ABCD3	3029	1888	-0.790707189	3.66E-80	1.18E-79
CES2	3031	1598	-1.03225082	1.25E-124	5.79E-124
SNAP23	3037	3372	0.042233252	0.241400153	0.247192997
CSGALNACT	3041	2584	-0.343664122	4.58E-19	7.17E-19
NRP1	3044	2097	-0.646365873	3.88E-57	1.01E-56
CNIH	3044	2330	-0.49436278	6.62E-36	1.34E-35
TOX4	3046	1906	-0.785092198	1.22E-79	3.91E-79
PTPN9	3047	1454	-1.176086607	3.04E-154	1.74E-153
UQCRH	3047	2535	-0.374128129	4.00E-22	6.59E-22
RSF1	3051	1549	-1.086669412	3.05E-136	1.53E-135
ARL5B	3053	2231	-0.561261452	6.74E-45	1.54E-44
TLN2	3054	1023	-1.686618293	7.23E-265	7.86E-264
ATG13	3054	2711	-0.280599325	1.58E-13	2.22E-13
SIN3A	3056	1170	-1.493860389	1.25E-222	1.09E-221
CLCC1	3057	1336	-1.30292092	2.16E-181	1.51E-180
EIF3J	3061	2036	-0.696989859	2.25E-65	6.33E-65
GLYR1	3063	1758	-0.909734672	2.01E-102	7.92E-102
NQO1	3065	767	-2.107312967	0	0
PPFIA1	3065	1633	-1.017086659	5.04E-123	2.32E-122
EIF3M	3068	2244	-0.559950183	6.30E-45	1.44E-44

ATXN2	3072	1527	-1.11720253	2.30E-143	1.22E-142
PA2G4	3074	2161	-0.617142469	2.91E-53	7.26E-53
RASSF8	3077	1731	-0.938643095	2.50E-108	1.03E-107
NTM	3084	7888	1.246132411	0	0
MT2A	3086	11654	1.808290873	0	0
UBL3	3088	2577	-0.369704591	6.07E-22	9.98E-22
ADPGK	3091	1772	-0.911419426	1.17E-103	4.66E-103
AP2A2	3098	1845	-0.856440704	1.08E-93	3.97E-93
CAT	3103	857	-1.965020963	0	0
NEMF	3106	1587	-1.077480077	8.64E-137	4.36E-136
LARP4B	3113	1750	-0.939675031	9.03E-110	3.76E-109
MAPK6	3117	2834	-0.246042773	4.79E-11	6.40E-11
MPHOSPH8	3118	1605	-1.066772007	3.97E-135	1.97E-134
DSTYK	3119	1477	-1.187138102	3.56E-160	2.13E-159
DUSP3	3119	1779	-0.918741418	5.70E-106	2.31E-105
IFITM2	3120	2430	-0.469314091	1.42E-33	2.80E-33
ARRB1	3122	909	-1.888842714	0	0
RSL24D1	3122	2673	-0.332735076	1.80E-18	2.80E-18
PCNXL3	3124	1746	-0.94806527	8.99E-112	3.80E-111
KHNYN	3124	1817	-0.89056041	8.35E-101	3.24E-100
PPFIBP1	3125	2241	-0.588437917	7.73E-50	1.87E-49
PRRX1	3127	863	-1.966071131	0	0
DCAF5	3128	1829	-0.882909813	1.70E-99	6.53E-99
PLEKHM2	3130	1840	-0.875181267	4.09E-98	1.56E-97
SYNRG	3130	1983	-0.767202355	1.17E-78	3.70E-78
ZSWIM8	3136	1235	-1.453138894	2.81E-219	2.41E-218
CANT1	3138	1946	-0.798058018	2.77E-84	9.27E-84
MINK1	3139	1570	-1.108264846	1.38E-144	7.40E-144
WAPAL	3143	1989	-0.768823372	2.93E-79	9.33E-79
ARHGEF11	3145	1333	-1.347107613	2.78E-196	2.11E-195
ATMIN	3146	1891	-0.843093683	1.19E-92	4.33E-92
ANP32A	3159	1585	-1.103709473	1.52E-144	8.18E-144

HSD17B12	3162	2629	-0.375047602	5.36E-23	8.95E-23
RGL1	3164	902	-1.919274637	0	0
PITPNA	3164	1696	-1.008337806	3.63E-125	1.70E-124
MYSM1	3171	1578	-1.115565048	1.42E-147	7.78E-147
CUL5	3174	1845	-0.891405688	1.51E-102	5.94E-102
HDGFRP3	3174	1973	-0.794635548	1.30E-84	4.36E-84
C16orf72	3174	2004	-0.772143996	1.31E-80	4.24E-80
ZCCHC11	3179	1333	-1.362620612	7.48E-202	5.84E-201
STARD13	3179	2297	-0.577546536	4.70E-49	1.13E-48
ANPEP	3179	4557	0.410786978	1.86E-35	3.75E-35
BAG3	3180	2350	-0.545090385	2.23E-44	5.04E-44
FMN2	3181	1221	-1.490141547	8.37E-231	7.65E-230
INF2	3183	2420	-0.504104485	9.16E-39	1.93E-38
RABEP1	3185	1475	-1.219302794	1.68E-170	1.09E-169
STIM1	3190	1218	-1.497766667	3.42E-233	3.18E-232
PRKACB	3190	2275	-0.596414255	4.89E-52	1.21E-51
STX6	3193	1840	-0.903931163	1.45E-105	5.86E-105
TUFM	3203	2639	-0.388156823	1.04E-24	1.79E-24
RNPEP	3203	2748	-0.32976617	1.28E-18	1.99E-18
RAI1	3209	2321	-0.576101643	2.76E-49	6.62E-49
VSTM4	3210	429	-3.01224812	0	0
HMCN1	3210	2336	-0.567257399	5.40E-48	1.28E-47
SAMD8	3212	1714	-1.014829159	2.29E-128	1.09E-127
PSMA4	3214	2105	-0.719274072	2.45E-72	7.36E-72
SIK2	3216	717	-2.273946758	0	0
EPS15	3216	1905	-0.864200785	1.16E-98	4.45E-98
GOLGA5	3216	2424	-0.516602084	7.41E-41	1.60E-40
CTDNEP1	3221	2241	-0.632090388	4.48E-58	1.18E-57
ATP5C1	3231	2964	-0.233159679	2.02E-10	2.66E-10
DNM1L	3234	1953	-0.836352105	6.44E-94	2.36E-93
NEDD4	3234	2033	-0.77843384	3.13E-83	1.03E-82
CREBZF	3236	1709	-1.029783587	2.06E-132	1.01E-131

NPTX1	3241	284	-3.621200562	0	0
GSE1	3241	1580	-1.145238838	3.33E-157	1.95E-156
SAP18	3241	1841	-0.92467377	3.24E-111	1.36E-110
IRF2BP2	3243	1512	-1.20959526	2.03E-171	1.32E-170
GOSR2	3244	2725	-0.360241966	5.87E-22	9.66E-22
ARL2BP	3247	2572	-0.444941118	9.11E-32	1.75E-31
PHLDA3	3250	2106	-0.734658658	8.22E-76	2.55E-75
KPNA2	3252	2040	-0.781482481	3.07E-84	1.03E-83
SSU72	3253	2525	-0.47421181	1.43E-35	2.88E-35
A2M	3255	951	-1.883864673	0	0
HK1	3256	2715	-0.370872877	3.55E-23	5.94E-23
GHITM	3256	2834	-0.308985317	6.80E-17	1.03E-16
SSRP1	3257	1985	-0.823129088	4.46E-92	1.61E-91
RAB8B	3261	1280	-1.457895007	4.84E-229	4.40E-228
ERH	3267	2196	-0.681812776	4.51E-67	1.29E-66
SLC41A1	3271	2066	-0.771615881	6.42E-83	2.12E-82
GCLM	3272	2840	-0.313006194	2.37E-17	3.62E-17
PIGK	3274	2681	-0.397007478	3.11E-26	5.49E-26
ETNK1	3275	1698	-1.056382824	1.29E-139	6.65E-139
MAPK8IP3	3275	1787	-0.982679648	3.65E-124	1.69E-123
SPTLC2	3277	3077	-0.199575605	3.50E-08	4.36E-08
ISCU	3280	1979	-0.837648578	1.79E-95	6.66E-95
CARHSP1	3281	2327	-0.604388759	1.03E-54	2.60E-54
BICC1	3284	1766	-1.00369316	7.22E-129	3.46E-128
KLHL21	3285	1557	-1.185848802	2.64E-168	1.68E-167
TRIM5	3285	1694	-1.064183872	1.05E-141	5.52E-141
KSR1	3288	1144	-1.631847623	5.31E-272	6.05E-271
PSMD4	3293	2740	-0.373950995	9.22E-24	1.57E-23
PSMD3	3293	2862	-0.311103216	2.86E-17	4.35E-17
CBL	3296	2160	-0.718409306	5.54E-74	1.69E-73
SMEK1	3298	1866	-0.930366788	2.74E-114	1.18E-113
SLC7A1	3299	3117	-0.190594999	1.21E-07	1.49E-07

ADIPOR1	3302	2963	-0.26500587	3.66E-13	5.12E-13
WEE1	3304	1032	-1.787495092	0	0
ZFHX3	3304	1602	-1.153063915	5.91E-162	3.60E-161
PDPK1	3305	1822	-0.967851688	3.31E-122	1.52E-121
CDK12	3306	1922	-0.891202764	1.08E-106	4.41E-106
SMPD1	3310	3547	-0.008956263	0.79712338	0.800712178
BTBD7	3319	1731	-1.047867281	1.24E-139	6.42E-139
ITGA3	3319	4239	0.244250961	2.21E-13	3.11E-13
EHBP1L1	3322	2269	-0.65871984	2.99E-64	8.32E-64
EIF2S1	3323	2098	-0.772195991	2.58E-84	8.65E-84
SGSM2	3329	1200	-1.580778841	4.11E-263	4.42E-262
CNTNAP1	3332	1684	-1.093220638	4.82E-150	2.67E-149
NAP1L4	3333	2029	-0.824776828	1.69E-94	6.24E-94
SPEN	3337	1641	-1.132700824	5.30E-159	3.15E-158
VAT1L	3338	5171	0.522734974	2.55E-61	6.91E-61
STX12	3341	1718	-1.068274322	5.61E-145	3.02E-144
PPM1A	3341	1877	-0.940575708	7.47E-118	3.32E-117
FADS1	3342	3411	-0.079241354	0.023960364	0.02536693
TAOK2	3344	1999	-0.851020751	6.51E-100	2.51E-99
ESD	3346	1989	-0.859118545	1.45E-101	5.66E-101
CDIPT	3347	1647	-1.131752372	2.92E-159	1.74E-158
CPSF7	3348	1728	-1.062920686	4.15E-144	2.22E-143
HDAC7	3348	2080	-0.795440375	2.66E-89	9.31E-89
DCAF6	3348	2267	-0.671239513	7.18E-67	2.05E-66
PSMC1	3349	2588	-0.480617135	1.94E-37	4.01E-37
COMMD6	3349	2635	-0.45465179	5.68E-34	1.12E-33
PLEKHA5	3350	951	-1.925368225	0	0
PDPR	3350	1769	-1.029951423	4.82E-137	2.43E-136
JUP	3350	2465	-0.551297825	1.38E-47	3.25E-47
RB1	3351	1864	-0.954914202	3.55E-121	1.61E-120
FLT1	3354	1509	-1.261014259	4.63E-189	3.38E-188
DENR	3361	1951	-0.893401141	7.02E-109	2.91E-108

CDC73	3363	2094	-0.792211712	4.44E-89	1.55E-88
CHSY1	3364	2914	-0.315901204	4.42E-18	6.82E-18
TBRG1	3373	1740	-1.063669388	2.47E-145	1.33E-144
ZNF770	3378	2093	-0.799321392	7.93E-91	2.81E-90
QSER1	3381	1450	-1.330121492	7.93E-207	6.37E-206
PSME3	3381	2002	-0.864732418	9.92E-104	3.96E-103
GOLT1B	3383	4341	0.25099987	2.55E-14	3.65E-14
MEF2D	3384	2634	-0.470198599	2.17E-36	4.43E-36
SRSF2	3390	1689	-1.113840321	3.06E-157	1.79E-156
TERF2IP	3394	2489	-0.55614471	6.28E-49	1.50E-48
CSRP2	3394	3183	-0.201323784	1.52E-08	1.91E-08
FAM160B1	3397	2044	-0.841590398	1.37E-99	5.25E-99
TRIM33	3398	1554	-1.237423725	5.45E-186	3.92E-185
RNF40	3402	1957	-0.906463761	7.20E-113	3.07E-112
FAR1	3405	2009	-0.86990161	1.70E-105	6.85E-105
DNAJC8	3407	2273	-0.692628639	8.85E-72	2.64E-71
HEATR5A	3410	3419	-0.104921692	0.002644392	0.002890546
SPCS2	3411	2346	-0.648716117	3.30E-64	9.18E-64
IGF2BP1	3413	2642	-0.478134324	8.89E-38	1.85E-37
RNF187	3419	3081	-0.258900116	4.74E-13	6.61E-13
ATP5H	3421	2667	-0.467924655	1.86E-36	3.79E-36
HBD	3424	235	-3.973674416	0	0
MGAT2	3428	2045	-0.853990642	5.67E-103	2.24E-102
PDXDC1	3428	3575	-0.048148243	0.162443185	0.167461232
BTAF1	3431	2720	-0.44374685	2.58E-33	5.05E-33
TRPM7	3433	1476	-1.326501512	4.18E-209	3.38E-208
TMTC3	3435	1911	-0.954706697	4.03E-124	1.87E-123
NLK	3438	661	-2.487571744	0	0
MTOR	3438	2091	-0.826100859	1.12E-97	4.24E-97
COX6A1	3441	2933	-0.339175197	6.98E-21	1.13E-20
RNH1	3442	3202	-0.212998165	1.77E-09	2.28E-09
ABHD2	3455	1723	-1.112487385	6.60E-160	3.94E-159

ATG2B	3469	1641	-1.188668978	2.78E-178	1.91E-177
RIC8A	3472	2228	-0.748742091	1.61E-83	5.34E-83
NRAS	3472	2657	-0.494693096	9.25E-41	1.99E-40
ACACA	3474	2849	-0.394866508	1.79E-27	3.22E-27
SDHC	3478	2820	-0.411287146	1.54E-29	2.86E-29
MAZ	3479	1702	-1.140166018	2.04E-167	1.29E-166
MARK3	3480	2026	-0.889177508	8.79E-112	3.71E-111
APEX1	3487	2023	-0.89421442	4.64E-113	1.99E-112
R3HDM2	3489	1659	-1.181224087	1.55E-177	1.06E-176
SLC39A13	3492	2560	-0.556634125	2.28E-50	5.56E-50
SSH2	3496	893	-2.07769748	0	0
CCNY	3497	2088	-0.85272046	9.58E-105	3.84E-104
CHML	3505	1081	-1.805772297	0	0
CCSER2	3505	2035	-0.893110026	2.12E-113	9.08E-113
HMGA2	3507	4366	0.207350324	2.01E-10	2.66E-10
PNRC2	3513	1625	-1.220988234	2.96E-188	2.15E-187
AKIRIN1	3513	2800	-0.436001125	4.93E-33	9.59E-33
PKN2	3524	2110	-0.848695301	1.05E-104	4.19E-104
CCDC6	3529	2263	-0.749747221	4.71E-85	1.59E-84
ATF6	3529	2458	-0.63049889	3.53E-63	9.72E-63
BTF3L4	3537	2182	-0.805599493	2.83E-96	1.06E-95
JTB	3542	2856	-0.419292609	4.37E-31	8.30E-31
PCNXL4	3555	2677	-0.517956797	3.47E-45	7.93E-45
KLHL42	3558	1752	-1.130788112	6.05E-169	3.88E-168
PIGS	3558	3266	-0.232266096	2.94E-11	3.94E-11
GRB2	3560	2731	-0.491172303	3.14E-41	6.81E-41
ITFG3	3564	3127	-0.297442493	3.55E-17	5.39E-17
FNBP1L	3566	1039	-1.887835425	0	0
SLC35E2B	3570	1279	-1.589632186	2.78E-284	3.34E-283
SRSF10	3571	2162	-0.832685987	9.61E-103	3.79E-102
TSPAN4	3573	2898	-0.410802695	3.08E-30	5.79E-30
GPATCH8	3588	1744	-1.149504226	7.62E-175	5.09E-174

LEPREL2	3593	4837	0.320199227	3.76E-24	6.42E-24
PINK1	3599	2516	-0.625188556	1.85E-63	5.11E-63
SMC3	3600	1209	-1.682907038	0	0
MEF2A	3601	1473	-1.398364545	1.83E-237	1.74E-236
CCDC47	3602	2510	-0.629835193	2.49E-64	6.94E-64
REXO2	3603	3042	-0.352902875	2.36E-23	3.96E-23
NUMB	3605	2830	-0.457921582	6.71E-37	1.38E-36
NDUFS5	3607	3323	-0.227037506	5.74E-11	7.65E-11
UGGT2	3607	3708	-0.068882555	0.041089547	0.043202945
SPG11	3609	1747	-1.155443911	2.70E-177	1.83E-176
KCTD3	3613	1548	-1.331516159	3.14E-221	2.73E-220
PFKP	3618	4061	0.057918243	0.07869106	0.08203157
LONP2	3626	2319	-0.753600481	4.19E-88	1.45E-87
DENND1B	3628	1178	-1.731559293	0	0
CPSF6	3638	2084	-0.912514641	6.71E-122	3.07E-121
HSPH1	3644	2094	-0.907985893	4.28E-121	1.95E-120
SUCO	3646	2236	-0.814118749	5.87E-101	2.28E-100
MAN1C1	3654	685	-2.524025117	0	0
COPS2	3654	2234	-0.818571824	4.15E-102	1.63E-101
EDEM3	3655	2846	-0.46966012	4.35E-39	9.21E-39
SCYL2	3658	2229	-0.823382834	3.13E-103	1.24E-102
MTA2	3658	2375	-0.731851938	1.54E-84	5.17E-84
ARNT2	3659	1766	-1.159688449	9.09E-181	6.30E-180
ARNT	3662	1672	-1.23978132	7.12E-201	5.54E-200
KIAA0319L	3663	2107	-0.906559763	2.12E-121	9.66E-121
SBF2	3664	1695	-1.220858606	3.16E-196	2.40E-195
GPR176	3664	4070	0.042884915	0.191287482	0.196832777
ZZEF1	3667	1647	-1.263484087	3.88E-207	3.12E-206
PPIB	3668	6190	0.646221395	4.57E-106	1.86E-105
WFDC1	3671	5273	0.413726506	2.81E-41	6.10E-41
SMG5	3689	1858	-1.098203663	4.09E-167	2.58E-166
FNBP4	3695	1908	-1.062237569	1.13E-158	6.72E-158

FAM20B	3697	1817	-1.133521	3.86E-176	2.60E-175
RRAS2	3704	2071	-0.947480921	5.02E-132	2.45E-131
UBE2Q2	3704	2581	-0.629878333	4.15E-66	1.18E-65
SLK	3707	1729	-1.209038622	1.64E-195	1.24E-194
MED1	3707	2068	-0.950740305	7.02E-133	3.44E-132
STIP1	3717	2869	-0.482315096	1.28E-41	2.78E-41
TUBA1C	3727	4389	0.127153075	7.33E-05	8.37E-05
TRAF7	3728	2667	-0.591908411	1.02E-59	2.73E-59
ARAP1	3729	2290	-0.812165575	8.54E-103	3.37E-102
HERC4	3731	2525	-0.672003348	1.87E-74	5.73E-74
SBNO1	3738	2014	-1.000927262	9.43E-146	5.10E-145
ZMPSTE24	3738	2641	-0.609906643	4.51E-63	1.24E-62
ZDHHC20	3747	2545	-0.666794697	9.05E-74	2.76E-73
GTF2A1	3751	1963	-1.042939466	2.38E-156	1.38E-155
LGALS3	3751	2799	-0.531088152	1.03E-49	2.48E-49
OBSCN	3756	1566	-1.370837226	2.75E-240	2.64E-239
ARL6IP1	3757	1835	-1.142525429	3.16E-181	2.20E-180
EMP1	3758	3111	-0.381311048	7.99E-28	1.45E-27
LPPR4	3762	326	-3.637280355	0	0
NXF1	3763	1551	-1.387408979	3.91E-245	3.84E-244
POMP	3763	3124	-0.377213212	2.46E-27	4.43E-27
ATP5F1	3764	2887	-0.491419896	1.57E-43	3.50E-43
RTF1	3768	2007	-1.017482724	7.00E-151	3.91E-150
CTR9	3774	1607	-1.34044887	2.33E-233	2.17E-232
SEC23IP	3777	3235	-0.332199447	5.93E-22	9.75E-22
CPSF2	3779	2468	-0.7233865	1.48E-85	5.03E-85
NGRN	3779	3039	-0.42313222	1.37E-33	2.70E-33
ST5	3779	3261	-0.321414453	9.82E-21	1.58E-20
PI4KB	3785	2052	-0.991986945	2.05E-145	1.11E-144
IDE	3788	1907	-1.098855863	1.04E-171	6.80E-171
FBXO18	3792	3386	-0.272101367	1.38E-15	2.04E-15
TMEM167B	3793	2889	-0.501493544	1.92E-45	4.39E-45

ZNF609	3798	2056	-0.994124017	2.02E-146	1.10E-145
ATP6V0C	3799	2958	-0.469722035	1.44E-40	3.09E-40
PSMB5	3800	3451	-0.247709321	2.68E-13	3.76E-13
GLT8D2	3803	4495	0.132458801	2.96E-05	3.42E-05
KDM1A	3807	2444	-0.748134661	2.87E-91	1.02E-90
CARS	3822	3330	-0.307529977	2.12E-19	3.34E-19
CASP4	3823	1553	-1.408371746	1.10E-254	1.13E-253
IREB2	3823	2190	-0.912498706	5.39E-128	2.56E-127
LMOD1	3824	4588	0.154058492	1.01E-06	1.22E-06
GNAI3	3826	2780	-0.569476367	2.99E-57	7.80E-57
RGS10	3828	3452	-0.257882741	2.46E-14	3.51E-14
EML1	3835	1143	-1.85512555	0	0
DTX4	3836	795	-2.379300331	0	0
PRKG1	3838	1695	-1.287793814	3.63E-223	3.18E-222
NYNRIN	3838	2130	-0.958225657	2.72E-139	1.40E-138
PRDX3	3841	2640	-0.64966841	2.97E-72	8.91E-72
PHC2	3847	2840	-0.546567276	1.44E-53	3.60E-53
GIT2	3850	2156	-0.945225644	1.29E-136	6.50E-136
ZYG11B	3850	2513	-0.72417215	2.74E-87	9.43E-87
PNN	3852	1800	-1.206335173	2.28E-202	1.79E-201
MYO5A	3852	2546	-0.70609966	1.24E-83	4.13E-83
PPP1R9B	3864	2479	-0.749061199	8.39E-93	3.05E-92
MAST2	3865	2422	-0.782993925	4.36E-100	1.68E-99
C17orf85	3868	2078	-1.005116516	8.81E-152	4.96E-151
TANC2	3869	2348	-0.829252696	2.19E-110	9.13E-110
YY1	3871	2260	-0.885107911	3.54E-123	1.63E-122
SRRM1	3871	2483	-0.749346422	5.00E-93	1.82E-92
SNHG16	3871	3295	-0.341152218	1.57E-23	2.65E-23
IRS2	3873	1164	-1.843084819	0	0
LRRC41	3874	2672	-0.644628322	6.98E-72	2.08E-71
PARP1	3877	2303	-0.8601507	1.30E-117	5.75E-117
AKAP13	3880	2678	-0.643625068	8.49E-72	2.53E-71

KAT6B	3882	1249	-1.744751017	0	0
GLCE	3884	1291	-1.697778576	0	0
RHOBTB1	3884	2655	-0.657555716	1.40E-74	4.30E-74
N4BP2L2	3886	1835	-1.191230214	3.84E-200	2.97E-199
CD276	3899	2326	-0.853977434	7.50E-117	3.29E-116
TPP2	3903	2098	-1.004293161	6.22E-153	3.53E-152
SLC39A1	3903	2484	-0.760642665	3.35E-96	1.25E-95
HIF1AN	3904	2136	-0.978765782	1.14E-146	6.24E-146
RBBP4	3905	1631	-1.368290142	4.92E-249	4.93E-248
TGFB1I1	3909	5021	0.252450764	1.73E-16	2.59E-16
ARHGEF7	3914	1959	-1.107250734	1.59E-179	1.10E-178
BSDC1	3914	2276	-0.890867574	7.08E-126	3.33E-125
FAM129A	3928	2875	-0.558957349	7.60E-57	1.97E-56
WDR11	3931	2274	-0.898388485	3.40E-128	1.62E-127
CDC16	3931	2548	-0.734255462	3.23E-91	1.15E-90
APH1A	3932	2253	-0.912140384	1.67E-131	8.16E-131
RCC2	3932	2555	-0.730664406	1.77E-90	6.26E-90
PSMA1	3932	3045	-0.477545469	3.64E-43	8.10E-43
SGPL1	3939	1880	-1.175821131	9.90E-199	7.61E-198
CRTC3	3950	1117	-1.930947843	0	0
PSEN1	3950	2206	-0.949144238	4.54E-141	2.38E-140
MLLT6	3965	1938	-1.141476671	7.21E-191	5.32E-190
GAA	3969	1913	-1.161663065	1.95E-196	1.48E-195
SLC9A3R2	3975	2533	-0.758832159	1.52E-97	5.75E-97
ARGLU1	3980	2032	-1.078592405	5.25E-175	3.52E-174
HIPK1	3993	2594	-0.731018968	6.28E-92	2.25E-91
TALDO1	4006	3184	-0.440046461	4.79E-38	9.98E-38
CASC3	4011	2077	-1.058185126	5.19E-171	3.38E-170
ERP29	4017	4219	-0.03794175	0.232580883	0.238380905
USP32	4018	2291	-0.919224481	4.54E-136	2.27E-135
SERPINF1	4021	3700	-0.228753441	3.25E-12	4.45E-12
RBBP6	4027	2203	-0.978960353	2.82E-151	1.58E-150

KARS	4029	3076	-0.498090678	1.37E-47	3.23E-47
C14orf166	4032	3869	-0.168259286	2.16E-07	2.64E-07
PPP6R3	4033	2302	-0.917689948	3.42E-136	1.71E-135
ANAPC5	4033	3113	-0.482272205	5.28E-45	1.21E-44
MLXIP	4034	1180	-1.8821486	0	0
PRCP	4037	3654	-0.252531329	1.66E-14	2.39E-14
MGST1	4038	1651	-1.399025166	3.25E-266	3.57E-265
PXN	4043	3061	-0.510147535	8.45E-50	2.04E-49
CFL2	4043	4004	-0.122708605	0.000135254	0.000153273
LAYN	4045	1575	-1.46951225	5.93E-287	7.26E-286
ATXN7L3B	4046	2366	-0.882770622	4.31E-128	2.06E-127
ARID4B	4049	1684	-1.37439788	6.95E-260	7.34E-259
LHFP	4054	1238	-1.82005915	0	0
TMCO1	4057	3211	-0.446115013	1.89E-39	4.01E-39
DIP2C	4060	2597	-0.753358089	2.28E-98	8.70E-98
SLC3A2	4062	1967	-1.154917658	3.78E-199	2.91E-198
ZC3H7A	4066	2448	-0.840731033	1.28E-118	5.72E-118
IKBIP	4073	5365	0.288761981	3.62E-22	5.97E-22
BCAR1	4082	3886	-0.179714657	2.68E-08	3.35E-08
TBX2	4095	3305	-0.417937556	1.79E-35	3.61E-35
KRAS	4104	1919	-1.205400395	3.28E-215	2.75E-214
OSBP	4108	3200	-0.469088652	1.27E-43	2.84E-43
SRSF5	4121	2471	-0.846623829	1.29E-121	5.89E-121
SYT11	4129	1727	-1.366248712	1.32E-262	1.42E-261
TAOK3	4129	2390	-0.897506176	2.47E-134	1.22E-133
ZNF503	4133	1024	-2.121698024	0	0
TFDP1	4149	2231	-1.00379752	2.59E-162	1.59E-161
ATP5L	4149	2791	-0.680705909	1.93E-84	6.47E-84
LINC00674	4154	2160	-1.05219428	2.03E-175	1.36E-174
UBE4A	4159	2557	-0.8105089	4.96E-114	2.13E-113
ITFG1	4159	3578	-0.325807673	3.32E-23	5.56E-23
PARD3	4166	1163	-1.949536118	0	0

TGFB2	4167	1494	-1.588553328	0	0
UBE4B	4171	2632	-0.772958199	1.61E-105	6.47E-105
RAB11A	4172	2889	-0.63889333	4.42E-76	1.37E-75
EXOC7	4175	2411	-0.900868923	1.18E-136	5.93E-136
ZRANB2	4178	3008	-0.582732301	6.55E-65	1.83E-64
KIAA0754	4181	2171	-1.054212693	4.29E-177	2.91E-176
RSL1D1	4181	2711	-0.733747306	8.79E-97	3.31E-96
PACS1	4187	2864	-0.656609801	4.71E-80	1.51E-79
PSMB4	4192	3139	-0.526058064	1.97E-54	4.99E-54
GNA13	4197	2567	-0.817999543	7.41E-117	3.25E-116
TCF25	4198	2770	-0.708540566	1.62E-91	5.80E-91
HERPUD1	4198	3566	-0.34411984	9.01E-26	1.58E-25
CBX1	4204	2365	-0.938646862	2.76E-147	1.52E-146
TRHDE	4214	5140	0.177853669	2.69E-09	3.45E-09
PDCD4	4218	2056	-1.145443206	4.90E-204	3.87E-203
ZBTB44	4222	1382	-1.719893339	0	0
STK24	4223	2288	-0.992905571	2.96E-162	1.81E-161
IST1	4225	2741	-0.732973389	1.33E-97	5.01E-97
ATP1A1	4228	2738	-0.735577306	2.88E-98	1.10E-97
UBTF	4234	1984	-1.20233362	4.46E-221	3.87E-220
CRK	4234	2539	-0.84648525	7.31E-125	3.41E-124
TSPAN9	4252	2407	-0.929629831	1.38E-146	7.56E-146
SHOC2	4253	2992	-0.616095056	8.89E-73	2.68E-72
KLF12	4257	1319	-1.799116901	0	0
RAB18	4259	3226	-0.509492667	3.02E-52	7.47E-52
ELK4	4262	2356	-0.96391543	5.70E-156	3.30E-155
FAM178A	4264	1664	-1.466276381	2.17E-301	2.86E-300
UQCRC2	4264	3501	-0.393164752	4.33E-33	8.44E-33
TP53I11	4265	2282	-1.010971326	9.61E-169	6.15E-168
CELF1	4266	2358	-0.964044623	3.78E-156	2.19E-155
RBL2	4271	2161	-1.091599202	2.67E-191	1.97E-190
ARHGEF40	4275	1443	-1.675577496	0	0

BIRC2	4278	2155	-1.097972989	2.09E-193	1.57E-192
SMG6	4283	2165	-1.092979028	3.20E-192	2.38E-191
ACBD3	4284	3942	-0.22875508	6.44E-13	8.95E-13
CCAR1	4286	1552	-1.574227668	0	0
ARHGAP17	4286	2174	-1.088004286	5.96E-191	4.40E-190
PHB2	4290	2690	-0.782095851	1.15E-110	4.82E-110
PIP5K1A	4292	3019	-0.616303695	1.80E-73	5.47E-73
ZRANB1	4295	1887	-1.295288084	9.94E-252	1.01E-250
USP7	4307	2677	-0.79479056	3.58E-114	1.54E-113
METAP2	4315	2822	-0.721366947	5.92E-97	2.23E-96
RNF141	4318	1550	-1.586819404	0	0
MTR	4319	1554	-1.583435189	0	0
SLC39A9	4328	3086	-0.596686813	5.20E-70	1.53E-69
FAM208B	4335	2589	-0.852361404	2.75E-129	1.32E-128
OAT	4345	4304	-0.122402475	7.91E-05	9.02E-05
FRY	4349	3950	-0.247555431	5.49E-15	7.97E-15
EXOC5	4352	2731	-0.780973618	5.81E-112	2.46E-111
ABR	4353	2930	-0.679833731	2.65E-88	9.18E-88
RAB1B	4353	3812	-0.300186277	5.73E-21	9.26E-21
DDX21	4360	3200	-0.554980606	4.08E-62	1.11E-61
RASA3	4360	3616	-0.378657833	1.27E-31	2.44E-31
CCT2	4365	2807	-0.745676966	8.82E-104	3.52E-103
RSU1	4366	3671	-0.358863392	8.55E-29	1.57E-28
PRDX5	4373	4633	-0.025401042	0.403345809	0.409534186
ZC3H13	4379	1653	-1.514239101	0	0
HDAC1	4379	2035	-1.214297031	3.77E-232	3.48E-231
MFN2	4389	2680	-0.820383645	1.03E-122	4.72E-122
GMFB	4390	2504	-0.918710753	2.40E-148	1.32E-147
KCTD10	4399	4368	-0.118927121	0.000112896	0.000128233
USP33	4413	1942	-1.292940922	7.24E-258	7.55E-257
UBE2I	4414	2509	-0.923698535	1.72E-150	9.59E-150
FBLIM1	4416	3117	-0.611306393	1.78E-74	5.45E-74

DZIP1	4418	2388	-0.996314958	1.31E-170	8.50E-170
ACTR1A	4421	3738	-0.350830541	5.41E-28	9.83E-28
UFM1	4424	3616	-0.399681084	2.79E-35	5.60E-35
YTHDF2	4428	2643	-0.853203173	3.08E-132	1.51E-131
PPP2R5C	4428	2844	-0.747458133	1.11E-105	4.48E-105
OAZ2	4431	2769	-0.786991649	1.87E-115	8.13E-115
PARK7	4455	3742	-0.360340249	1.46E-29	2.72E-29
KPNA3	4456	2804	-0.776987259	1.34E-113	5.75E-113
PPP1CC	4458	2923	-0.717671163	3.17E-99	1.22E-98
MTSS1L	4462	3324	-0.533494507	2.88E-59	7.66E-59
PRDX1	4466	3702	-0.379402732	1.84E-32	3.56E-32
RAB5C	4475	3219	-0.583999481	1.17E-69	3.43E-69
EHD1	4477	3170	-0.606773854	1.66E-74	5.10E-74
UBE2Z	4484	3079	-0.651048787	2.40E-84	8.04E-84
FLRT2	4493	848	-2.514267268	0	0
MAPK1IP1L	4502	2628	-0.885325157	5.41E-143	2.87E-142
IFI16	4505	2209	-1.136858063	3.43E-215	2.87E-214
ADCY6	4511	2033	-1.258561447	8.46E-253	8.61E-252
FURIN	4511	4025	-0.273182879	2.24E-18	3.48E-18
ZNF207	4512	2738	-0.829368997	1.94E-128	9.29E-128
HSBP1	4515	2337	-1.058792629	2.02E-192	1.50E-191
REEP3	4516	3198	-0.606599923	4.22E-75	1.30E-74
EI24	4529	3331	-0.551961561	7.74E-64	2.14E-63
XPR1	4536	2100	-1.219755688	4.32E-242	4.19E-241
RER1	4536	3547	-0.463545686	5.49E-47	1.28E-46
SPG20	4538	2229	-1.134384368	5.29E-216	4.47E-215
TRIP11	4544	1628	-1.589586511	0	0
NNMT	4554	9388	0.934959174	7.21E-301	9.44E-300
SCP2	4577	3868	-0.351538873	5.02E-29	9.23E-29
SESN3	4579	525	-3.233367613	0	0
CNP	4588	2513	-0.977179096	1.48E-171	9.64E-171
RNF41	4589	3368	-0.555012044	2.74E-65	7.71E-65

IARS2	4590	3006	-0.71937352	1.60E-102	6.30E-102
CDC27	4592	3435	-0.527536919	9.52E-60	2.54E-59
MSL1	4606	2746	-0.854907162	6.19E-138	3.15E-137
KIF1B	4609	2654	-0.905009773	8.29E-152	4.67E-151
CEP350	4614	1657	-1.586168777	0	0
TMEM50A	4615	2858	-0.800049107	1.43E-123	6.60E-123
ALDH18A1	4620	5727	0.201162376	1.50E-12	2.07E-12
ERC1	4625	2784	-0.84101853	9.81E-135	4.86E-134
ARIH1	4642	2710	-0.885178047	2.45E-147	1.35E-146
PTPN21	4646	2033	-1.301103315	5.20E-274	5.99E-273
EMC1	4649	4610	-0.120878051	5.51E-05	6.30E-05
FUS	4651	2433	-1.043528995	1.49E-193	1.12E-192
LRRC59	4656	5349	0.09145377	0.001534467	0.001691032
ARHGAP1	4660	3530	-0.509386147	5.63E-57	1.46E-56
APPL2	4664	1702	-1.563061127	0	0
VEZF1	4666	1992	-1.336693036	8.68E-287	1.06E-285
POGZ	4675	1985	-1.344551734	6.48E-290	8.11E-289
ZNF664	4677	2394	-1.074884652	3.68E-204	2.91E-203
TOR1AIP1	4682	2338	-1.11057438	2.23E-215	1.87E-214
MAP7D1	4682	3627	-0.477072565	5.65E-51	1.38E-50
DENND5A	4691	2126	-1.25047828	3.46E-260	3.66E-259
GDE1	4703	3180	-0.673278943	1.14E-93	4.18E-93
PIK3C2A	4710	3039	-0.740854761	1.35E-110	5.64E-110
TRIM8	4717	2786	-0.868398719	5.72E-145	3.08E-144
CS	4718	3168	-0.683327459	2.12E-96	7.96E-96
LTBR	4720	2619	-0.958495176	6.41E-171	4.15E-170
ANKFY1	4724	2345	-1.119145418	5.62E-220	4.85E-219
JAM3	4727	3252	-0.648321983	3.60E-88	1.25E-87
DCTN5	4732	2815	-0.858039527	1.58E-142	8.34E-142
SEC24C	4732	3004	-0.764289637	3.54E-117	1.56E-116
ZDHHC7	4737	2843	-0.845283949	3.96E-139	2.03E-138
JMJD1C	4738	2116	-1.271662947	1.05E-269	1.18E-268

DLST	4739	2829	-0.85301486	2.47E-141	1.29E-140
GLUD1	4748	3610	-0.504045474	6.01E-57	1.56E-56
SLC7A5	4762	4267	-0.267069886	1.46E-18	2.27E-18
EIF3I	4763	3942	-0.381667148	7.10E-35	1.42E-34
CMPK1	4782	4359	-0.242341302	9.78E-16	1.44E-15
PFKFB3	4784	4601	-0.164994309	2.99E-08	3.74E-08
OPTN	4787	3743	-0.463651134	1.65E-49	3.97E-49
C1orf43	4794	3740	-0.466916015	3.36E-50	8.18E-50
PLAU	4796	2075	-1.317444698	2.95E-288	3.65E-287
HEXA	4801	2859	-0.856548692	3.73E-144	2.00E-143
ZDHHC17	4817	1725	-1.590262938	0	0
MAPKAPK2	4817	3128	-0.731618787	1.17E-110	4.88E-110
ERBB2	4818	2093	-1.311586458	1.34E-287	1.66E-286
CTTNBP2NL	4824	2591	-1.005445268	6.76E-189	4.93E-188
COPZ1	4824	4141	-0.32897508	4.06E-27	7.29E-27
BTBD1	4825	3555	-0.549403758	2.45E-67	7.02E-67
PTGER2	4826	1114	-2.223803059	0	0
HNRNPH3	4831	2724	-0.935319525	5.81E-168	3.70E-167
CHD8	4831	2830	-0.880244176	7.89E-152	4.44E-151
IPO9	4833	2066	-1.334803116	2.46E-296	3.18E-295
HECTD4	4837	2606	-1.00099983	4.93E-188	3.58E-187
MARVELD1	4838	3723	-0.486669529	1.67E-54	4.24E-54
DNAJC3	4840	2795	-0.90088314	4.15E-158	2.45E-157
NUP98	4842	2681	-0.961556236	3.29E-176	2.22E-175
SNX1	4847	2263	-1.207579874	1.63E-254	1.67E-253
ADAMTS15	4853	302	-4.114980781	0	0
SPRED1	4855	3022	-0.792692011	7.11E-128	3.38E-127
NFATC4	4858	1579	-1.730075695	0	0
CAMSAP2	4872	2909	-0.852715213	3.70E-145	1.99E-144
BTG1	4875	1448	-1.860064992	0	0
API5	4876	3022	-0.798918841	3.89E-130	1.88E-129
NIN	4885	1914	-1.460492108	0	0

ICMT	4916	2848	-0.896260145	3.45E-159	2.06E-158
ITPRIPL2	4925	2539	-1.064587705	1.44E-211	1.18E-210
AMFR	4926	2476	-1.101129689	2.04E-223	1.79E-222
DICER1	4928	2819	-0.914543154	4.99E-165	3.11E-164
JUN	4929	2711	-0.971194244	2.63E-182	1.85E-181
CFH	4943	2048	-1.379895567	0	0
APPBP2	4944	2854	-0.901417784	1.27E-161	7.71E-161
ILF2	4944	3578	-0.575249732	9.30E-75	2.86E-74
ZMYM2	4946	2879	-0.889418827	4.11E-158	2.42E-157
VDAC2	4949	4129	-0.370069	3.07E-34	6.10E-34
INTS3	4950	2195	-1.281931961	3.34E-285	4.03E-284
ZFC3H1	4952	3183	-0.746348533	1.17E-117	5.18E-117
LRP6	4961	2521	-1.085359215	7.73E-220	6.66E-219
NLGN2	4962	3134	-0.771640931	8.45E-125	3.93E-124
ACADVL	4969	3190	-0.748123494	1.55E-118	6.91E-118
LITAF	4970	1901	-1.495211696	0	0
LRP5	4974	2729	-0.974758489	4.60E-185	3.29E-184
KLF6	4979	1800	-1.576583485	0	0
MIA3	4984	3082	-0.802161582	6.83E-134	3.37E-133
KDM2A	4986	2684	-1.002222587	3.77E-194	2.84E-193
TIAL1	4986	2690	-0.999001086	3.94E-193	2.95E-192
HNRNPR	4987	3053	-0.816668989	4.21E-138	2.15E-137
ZMYM4	4988	2531	-1.087478334	1.02E-221	8.90E-221
NPEPPS	4991	2884	-0.899982115	1.05E-162	6.44E-162
GBF1	4992	4734	-0.185282604	2.38E-10	3.13E-10
WAC	4997	3106	-0.794728764	3.93E-132	1.92E-131
HERC1	5007	2472	-1.126992088	1.21E-235	1.14E-234
TARDBP	5008	2912	-0.890948583	1.57E-160	9.46E-160
CAPN1	5011	3952	-0.451239967	2.62E-49	6.29E-49
TENC1	5018	1710	-1.661840522	0	0
USP24	5039	2057	-1.401320038	0	0
SNX6	5039	3461	-0.650672891	1.86E-94	6.86E-94

DDX24	5040	2902	-0.90510059	7.62E-166	4.77E-165
PTEN	5046	3646	-0.577550021	8.47E-77	2.65E-76
SERINC2	5056	4651	-0.2291799	5.04E-15	7.32E-15
TSC22D1	5080	2718	-1.011007417	1.30E-200	1.01E-199
VPS13D	5084	2550	-1.104191159	1.52E-231	1.40E-230
STAT6	5086	1481	-1.888684197	0	0
YARS	5090	4476	-0.294179996	2.14E-23	3.60E-23
KIAA0430	5104	2436	-1.175838572	6.71E-257	6.97E-256
CUL4A	5108	3314	-0.732909298	1.39E-117	6.17E-117
KIAA1033	5110	2433	-1.179311347	2.12E-258	2.22E-257
PUM1	5111	2754	-1.000801408	1.67E-198	1.28E-197
MLF2	5119	5110	-0.111263091	9.54E-05	0.000108589
C11orf58	5124	3113	-0.827689275	4.30E-145	2.32E-144
NMT1	5125	3261	-0.760961939	8.33E-126	3.91E-125
PEBP1	5128	3133	-0.819575868	8.31E-143	4.39E-142
KLC1	5130	3771	-0.552736051	3.04E-72	9.10E-72
PTPRF	5142	6492	0.227609014	2.18E-17	3.32E-17
CYFIP1	5146	3685	-0.590511214	2.16E-81	7.01E-81
PAFAH1B2	5147	4204	-0.400693489	5.97E-41	1.29E-40
S100A10	5155	5097	-0.12504845	1.13E-05	1.32E-05
VPS35	5157	3381	-0.717806405	1.62E-114	6.99E-114
EFCAB14	5161	2750	-1.016943388	9.84E-206	7.85E-205
TP53BP1	5163	3260	-0.77206201	6.76E-130	3.26E-129
POLR2L	5175	3367	-0.728819517	5.85E-118	2.60E-117
ATP5G2	5175	4114	-0.439741445	1.40E-48	3.34E-48
DCTN2	5180	3453	-0.69382614	1.79E-108	7.38E-108
SWAP70	5197	2832	-0.984582169	1.77E-196	1.34E-195
NUDT21	5202	2759	-1.023645318	1.39E-209	1.13E-208
ARF3	5207	3966	-0.501492105	1.01E-61	2.76E-61
KITLG	5208	969	-2.534885254	0	0
NBPF3	5208	1261	-2.154885549	0	0
TAF15	5228	2955	-0.931825387	1.78E-180	1.23E-179

MCAM	5231	5344	-0.077891138	0.005480676	0.005923681
PBXIP1	5241	6414	0.182657869	9.08E-12	1.23E-11
SP1	5246	2707	-1.063247296	9.18E-225	8.13E-224
ANXA11	5248	3829	-0.563524436	2.02E-76	6.30E-76
MAN1A2	5249	4361	-0.376107983	2.85E-37	5.88E-37
UBE3A	5258	2961	-0.937154027	3.19E-183	2.26E-182
SEC11A	5259	4435	-0.354578768	1.29E-33	2.54E-33
DCAF8	5269	2849	-0.995797934	6.61E-203	5.19E-202
SLC38A10	5278	3692	-0.624313174	5.69E-92	2.05E-91
PRSS23	5279	6338	0.155038618	7.37E-09	9.38E-09
SLFN5	5283	3399	-0.744971424	5.38E-125	2.51E-124
SAV1	5285	1381	-2.044914528	0	0
NUPR1	5319	3693	-0.63508615	1.76E-95	6.57E-95
HNRNPF	5322	3198	-0.843522947	1.62E-155	9.33E-155
TNFAIP1	5331	2344	-1.294157988	0	0
TSC2	5333	2800	-1.038244877	8.15E-220	7.01E-219
AHCYL1	5333	5780	0.007397789	0.786924095	0.790670388
CPT1A	5337	1947	-1.563500503	0	0
VPS39	5341	3197	-0.849115516	8.20E-158	4.82E-157
GNPTAB	5354	3219	-0.842728938	3.42E-156	1.98E-155
CCT3	5360	3805	-0.603060923	7.00E-88	2.42E-87
BLOC1S6	5376	3382	-0.777380854	9.12E-137	4.60E-136
BDNF	5379	3200	-0.85799046	1.00E-161	6.09E-161
ARPC3	5389	5641	-0.042791026	0.119206602	0.123361298
KDM5A	5391	2632	-1.143117796	2.32E-259	2.44E-258
CALCOCO1	5404	2605	-1.161468678	1.23E-266	1.35E-265
GTF3C1	5423	3275	-0.83632064	3.72E-156	2.15E-155
ANKRD52	5427	3710	-0.657460098	1.96E-103	7.80E-103
ESYT1	5432	3230	-0.858673691	1.62E-163	9.99E-163
PHGDH	5433	3767	-0.63705739	5.16E-98	1.96E-97
ZMYND11	5439	2956	-0.988419532	7.53E-207	6.06E-206
NPTN	5440	5430	-0.111378829	5.66E-05	6.48E-05

MON2	5447	3441	-0.771358351	1.01E-136	5.10E-136
CDH13	5447	4557	-0.366101873	8.49E-37	1.74E-36
METTL9	5461	3163	-0.896595977	1.01E-176	6.81E-176
DLG5	5464	3290	-0.840594276	1.11E-158	6.59E-158
FGF7	5464	3598	-0.711486673	2.19E-119	9.83E-119
GSPT1	5466	3962	-0.572980956	6.97E-82	2.28E-81
YLPM1	5470	2936	-1.006413241	2.96E-214	2.45E-213
CHP1	5503	4258	-0.478766755	5.64E-60	1.51E-59
PGD	5509	2824	-1.07277475	2.37E-239	2.27E-238
MBNL2	5515	4069	-0.547410981	3.26E-76	1.01E-75
HERC2	5519	2740	-1.118955368	1.41E-256	1.46E-255
SRCAP	5522	2557	-1.219463103	3.79E-294	4.86E-293
SRPR	5524	5161	-0.206787065	1.27E-13	1.79E-13
LPGAT1	5527	2565	-1.216262163	3.34E-293	4.25E-292
SLC30A7	5532	2595	-1.200790994	1.25E-287	1.54E-286
RBM25	5536	2829	-1.077276142	3.82E-242	3.71E-241
ARHGEF2	5545	2839	-1.074528987	1.54E-241	1.49E-240
KCTD12	5547	1097	-2.446868576	0	0
SUPT16H	5549	2755	-1.11889986	6.11E-258	6.38E-257
FZD2	5550	3130	-0.93504949	1.45E-192	1.08E-191
SZRD1	5561	3822	-0.649740935	8.75E-104	3.49E-103
USP48	5578	2440	-1.301591162	0	0
BMP4	5582	1499	-2.005506117	0	0
SH3BGRL3	5599	8653	0.519306847	3.54E-100	1.37E-99
DESI2	5609	3497	-0.790350163	1.10E-146	6.00E-146
ALDH2	5620	2401	-1.335659103	0	0
LUC7L3	5620	2977	-1.025435285	6.65E-227	5.98E-226
SECISBP2L	5627	3357	-0.853917802	9.66E-168	6.13E-167
MRVI1	5640	4659	-0.384399208	1.65E-41	3.60E-41
ZNF469	5648	3383	-0.848161287	1.88E-166	1.18E-165
AIDA	5651	3634	-0.745672146	8.80E-134	4.33E-133
FNDC3A	5657	3453	-0.820911213	9.10E-158	5.34E-157

ATXN2L	5659	3329	-0.874182643	1.83E-175	1.23E-174
ARID1A	5662	2872	-1.087980376	1.39E-251	1.41E-250
GRK5	5714	1892	-1.703313323	0	0
ECM1	5714	11007	0.837123993	1.26E-292	1.59E-291
STMN1	5721	2828	-1.1252096	2.86E-268	3.18E-267
SRGAP1	5732	2784	-1.150603774	1.65E-278	1.94E-277
SRP14	5736	4167	-0.5697603	5.49E-85	1.85E-84
NEK9	5749	2982	-1.055755149	2.58E-243	2.52E-242
PIP4K2B	5755	3268	-0.925132321	4.21E-196	3.19E-195
CCND2	5758	4963	-0.32307971	4.99E-31	9.48E-31
RCN2	5760	4501	-0.464547623	2.06E-59	5.48E-59
CDK2AP1	5765	5144	-0.273154341	4.85E-23	8.10E-23
SVIL	5769	5925	-0.070230485	0.008454977	0.009076837
OLFML3	5774	4551	-0.452111897	9.70E-57	2.51E-56
RNF11	5789	4388	-0.508475007	4.08E-70	1.20E-69
TBX3	5801	2131	-1.553495402	0	0
PPP1R12A	5801	4658	-0.425315356	4.20E-51	1.03E-50
ARF6	5806	3485	-0.845110292	5.12E-170	3.30E-169
TRIM44	5810	3459	-0.856907526	3.75E-174	2.48E-173
HYOU1	5831	5294	-0.248109504	1.20E-19	1.90E-19
ASH1L	5844	3051	-1.046398374	9.32E-244	9.11E-243
EXT2	5850	6586	0.062241511	0.016185286	0.017245313
NES	5854	5444	-0.213480055	3.67E-15	5.36E-15
SEP15	5858	3873	-0.705681068	4.66E-126	2.20E-125
AKAP11	5862	3060	-1.046585691	1.42E-244	1.40E-243
PDE4DIP	5865	2478	-1.351679297	0	0
ANO4	5875	2390	-1.406302609	0	0
RNF10	5877	3948	-0.682682284	2.07E-119	9.29E-119
SKI	5898	3403	-0.902143047	1.12E-192	8.35E-192
FADS2	5913	9507	0.576375507	2.73E-132	1.33E-131
BACE1	5922	3249	-0.974813123	5.76E-220	4.97E-219
BCL9L	5923	3578	-0.835899075	3.18E-170	2.06E-169

AKT1	5924	3808	-0.746262538	2.43E-140	1.26E-139
TMED5	5926	2532	-1.335505597	0	0
BAZ2A	5939	3098	-1.047607264	3.77E-248	3.75E-247
STRAP	5943	5552	-0.206908189	1.47E-14	2.12E-14
ZMIZ1	5945	3217	-0.99468525	2.95E-228	2.67E-227
DDAH1	5949	9091	0.503067444	2.41E-99	9.24E-99
MVP	5955	5309	-0.274385743	6.15E-24	1.05E-23
MYCBP2	5957	2909	-1.142787039	3.07E-286	3.73E-285
DEGS1	5964	4255	-0.595845502	1.57E-95	5.86E-95
PARP4	5971	3279	-0.973441043	2.96E-221	2.58E-220
SPAG9	5976	3307	-0.962381478	2.63E-217	2.24E-216
CERS2	5982	2788	-1.210121725	0	0
TAGLN2	5982	5204	-0.309731324	7.84E-30	1.46E-29
ZDHHC5	5995	3363	-0.942735351	1.11E-210	9.03E-210
NIPAL3	6013	2411	-1.427177683	0	0
BDKRB2	6023	1341	-2.275897398	0	0
SHMT2	6023	4726	-0.458587005	1.25E-60	3.37E-60
RASSF3	6057	2593	-1.332705581	0	0
ADCY9	6085	3088	-1.087308886	5.91E-270	6.66E-269
TMEM97	6103	305	-4.431361819	0	0
ADAMTS7	6107	2315	-1.508176028	0	0
PAPSS2	6120	4527	-0.543700722	2.33E-83	7.71E-83
RAP1B	6125	4379	-0.59283277	3.46E-97	1.31E-96
VAMP3	6128	3804	-0.796623427	3.03E-162	1.85E-161
RAB6A	6132	4288	-0.624777167	1.11E-106	4.51E-106
TNKS2	6141	3258	-1.023211375	7.16E-247	7.11E-246
NEK7	6145	3823	-0.793432186	1.39E-161	8.45E-161
DHCR24	6148	4455	-0.573416109	5.47E-92	1.97E-91
SLC12A4	6152	4520	-0.553457107	1.83E-86	6.26E-86
EP400	6164	3115	-1.093359074	6.94E-276	8.10E-275
CHD9	6174	3182	-1.064996023	7.37E-265	8.00E-264
LOC728392	6179	3071	-1.117389237	2.28E-286	2.78E-285

MARCKSL1	6188	2904	-1.200156119	0	0
DDX42	6197	3395	-0.97688277	6.87E-231	6.29E-230
DAZAP2	6210	4103	-0.70663849	7.91E-134	3.90E-133
TTC17	6219	3261	-1.040092552	9.88E-257	1.02E-255
KPNA6	6221	4137	-0.697285925	5.25E-131	2.54E-130
ENAH	6226	5382	-0.318887562	1.22E-32	2.36E-32
HDGF	6262	4388	-0.621784359	5.43E-108	2.23E-107
SF1	6269	3197	-1.080240962	3.79E-275	4.39E-274
PTPLAD1	6274	3068	-1.140811422	1.57E-300	2.05E-299
RDX	6291	3742	-0.858204179	6.97E-189	5.08E-188
PDGFD	6315	639	-3.413619274	0	0
PLEKHO2	6332	2327	-1.552914421	0	0
CSDA	6337	6439	-0.085687741	0.000783902	0.000870891
MAP1LC3B	6340	3903	-0.808623753	4.12E-172	2.70E-171
ARL1	6341	5183	-0.399647359	8.24E-50	1.99E-49
MAP4K5	6354	2799	-1.291477979	0	0
MED13	6356	2885	-1.248272182	0	0
NT5C2	6382	3196	-1.106465576	4.35E-291	5.47E-290
UBFD1	6392	5049	-0.448994107	7.55E-62	2.05E-61
GLUL	6393	2757	-1.322118202	0	0
SUMO2	6396	4199	-0.715848527	6.77E-141	3.54E-140
SF3B2	6397	4402	-0.647960714	1.58E-118	7.06E-118
PAMR1	6420	6249	-0.147672333	8.25E-09	1.05E-08
ATP2B1	6431	2695	-1.363482192	0	0
SF3B3	6433	3915	-0.825203757	1.00E-180	6.96E-180
DHX9	6440	2902	-1.258737545	0	0
TWF1	6448	4032	-0.786080481	8.80E-167	5.54E-166
GABARAP	6454	5114	-0.444465793	2.62E-61	7.09E-61
CKAP5	6460	3905	-0.834935992	4.11E-185	2.94E-184
MIR100HG	6463	3748	-0.894807415	4.42E-208	3.57E-207
IRF2BPL	6474	3947	-0.822625222	6.68E-181	4.65E-180
TBC1D2B	6477	2283	-1.61311926	0	0

MED13L	6509	3116	-1.171465057	0	0
ZC3H11A	6524	3892	-0.853969448	3.53E-194	2.66E-193
LEPROT	6527	3554	-0.985700734	1.07E-246	1.06E-245
TRIM22	6529	4106	-0.77785279	7.61E-166	4.76E-165
KIRREL	6549	3026	-1.222587019	0	0
TOR1AIP2	6568	3526	-1.006146028	9.78E-257	1.01E-255
DYNLL1	6574	4525	-0.64757804	1.26E-121	5.73E-121
CRISPLD2	6577	2656	-1.416898899	0	0
AJUBA	6577	3664	-0.952734543	5.24E-235	4.91E-234
ARHGEF17	6611	3529	-1.01433346	7.48E-262	7.97E-261
BPTF	6621	2943	-1.278485964	0	0
DRAM1	6623	3824	-0.901126711	1.05E-215	8.85E-215
ILK	6626	6165	-0.212761685	7.35E-17	1.11E-16
PHLDB1	6647	4754	-0.592285827	3.89E-105	1.56E-104
TCF12	6693	2818	-1.356705779	0	0
SMARCC2	6713	2910	-1.314662865	0	0
SH3GLB1	6714	5150	-0.491324481	3.24E-76	1.01E-75
ANO6	6725	4459	-0.701538444	5.85E-143	3.09E-142
TMCO3	6732	3736	-0.958265098	8.24E-243	8.03E-242
FLII	6739	4407	-0.721462005	2.32E-150	1.29E-149
C14orf132	6761	2111	-1.788034444	0	0
LUZP1	6766	5096	-0.517662275	2.79E-84	9.32E-84
THRAP3	6783	4456	-0.714898636	5.85E-149	3.23E-148
RAN	6789	4619	-0.664342915	2.87E-131	1.40E-130
HMGN2	6790	2975	-1.299246282	0	0
SIPA1L1	6842	3755	-0.974329572	1.31E-253	1.34E-252
MSRB3	6858	6092	-0.279596338	2.95E-28	5.38E-28
ANKRD13A	6859	5582	-0.405940507	2.89E-55	7.37E-55
EEA1	6860	2103	-1.814484102	0	0
GOLGA3	6887	6075	-0.289715644	3.09E-30	5.80E-30
SIPA1L2	6897	572	-3.700606291	0	0
PODN	6898	3414	-1.123439446	0	0

OSBPL8	6904	3750	-0.989266245	2.23E-262	2.39E-261
SCD	6907	16177	1.119088582	0	0
RAB5B	6912	4513	-0.723740815	5.19E-155	2.98E-154
TMEM98	6918	2660	-1.487653144	0	0
LDB1	6928	3142	-1.249480125	0	0
INPPL1	6939	4161	-0.846521854	2.26E-203	1.78E-202
COX4I1	6993	4986	-0.596752998	5.55E-112	2.35E-111
EIF4EBP2	6995	3181	-1.245568061	0	0
KHDRBS1	7014	3761	-1.0078455	8.70E-275	1.01E-273
SRSF1	7029	3603	-1.072845179	7.55E-305	9.99E-304
UBAP2L	7035	4634	-0.711016755	5.68E-153	3.23E-152
ARPP19	7062	4435	-0.779867092	5.15E-180	3.56E-179
HELZ	7086	3200	-1.255623936	0	0
TM9SF2	7127	5094	-0.593220341	6.66E-113	2.84E-112
KDM5B	7128	4713	-0.705576031	6.39E-153	3.63E-152
SMARCE1	7146	3228	-1.255219711	0	0
SNTB2	7148	4500	-0.776339009	8.95E-181	6.21E-180
EIF2AK4	7155	5382	-0.519533752	1.36E-89	4.76E-89
RHOC	7156	6062	-0.348083911	1.22E-43	2.73E-43
SUPT6H	7159	4380	-0.817551586	9.68E-198	7.40E-197
TNRC6A	7173	3337	-1.21274932	0	0
TMOD3	7199	3589	-1.112938971	0	0
DCHS1	7213	3211	-1.276301135	0	0
USP47	7231	4509	-0.790112042	1.98E-188	1.44E-187
CDC42BPA	7252	4315	-0.857742742	3.14E-217	2.67E-216
PICALM	7302	6757	-0.22063319	1.21E-19	1.91E-19
CLIP1	7310	3917	-1.008846654	9.22E-287	1.13E-285
MDK	7360	4181	-0.924582099	5.67E-250	5.70E-249
AP1G1	7383	3959	-1.007795467	4.13E-289	5.15E-288
GSTP1	7398	7338	-0.120472772	3.95E-07	4.80E-07
TNFRSF1A	7402	4358	-0.872973315	2.38E-228	2.16E-227
NCSTN	7435	6530	-0.295974126	8.72E-34	1.72E-33

VPS13C	7448	2501	-1.683072391	0	0
GCN1L1	7491	6017	-0.424838341	2.46E-65	6.92E-65
LATS2	7497	3062	-1.400563495	0	0
NRD1	7497	6898	-0.228859649	1.77E-21	2.90E-21
SGIP1	7517	5042	-0.684885262	4.84E-153	2.75E-152
NCOA4	7560	3876	-1.07254204	0	0
DOCK7	7580	3769	-1.116740429	0	0
ARHGAP21	7590	2637	-1.63392669	0	0
ITGA11	7619	10556	0.36166532	2.57E-63	7.10E-63
CEP170	7668	4715	-0.810316942	1.76E-208	1.43E-207
LEPRE1	7681	15169	0.87303555	0	0
CCNL2	7691	3393	-1.289334138	0	0
MARS	7694	6639	-0.321492254	1.58E-40	3.39E-40
PTMS	7696	5784	-0.520765623	1.24E-96	4.67E-96
RALGPS2	7713	2934	-1.503147616	0	0
ARHGAP5	7730	4127	-1.014099352	1.05E-305	1.39E-304
CALM1	7732	4600	-0.857932152	1.47E-231	1.36E-230
TOMM20	7745	5154	-0.696297078	3.24E-162	1.98E-161
NCOR1	7758	5155	-0.698436724	2.36E-163	1.46E-162
TMEM59	7778	7386	-0.183330348	4.99E-15	7.25E-15
AARS	7781	5252	-0.675813039	1.03E-154	5.92E-154
CHD2	7813	5559	-0.599775608	5.27E-126	2.49E-125
PIEZO1	7815	4010	-1.071368012	0	0
ARHGAP23	7818	4364	-0.949872859	1.42E-277	1.67E-276
CREBBP	7824	3244	-1.378856927	0	0
RTN3	7845	5283	-0.679140413	2.61E-157	1.53E-156
MGEA5	7852	5065	-0.74122228	3.25E-183	2.30E-182
TPM3	7855	6587	-0.362714101	2.00E-51	4.92E-51
PCNX	7864	4307	-0.977304374	1.19E-292	1.51E-291
IPO7	7916	5966	-0.516732011	5.69E-98	2.16E-97
KDELC2	7932	4211	-1.022246178	0	0
RPS27L	7943	7353	-0.220075394	3.91E-21	6.34E-21

ABCC4	7945	6163	-0.475138804	1.30E-84	4.38E-84
DCAF7	7952	4675	-0.875075768	3.70E-246	3.66E-245
PBX1	7993	3175	-1.440704873	0	0
HIPK3	8008	2365	-1.868326166	0	0
NAV1	8036	7717	-0.167161834	3.47E-13	4.85E-13
NCOR2	8049	5833	-0.573295857	8.52E-120	3.85E-119
EIF4G3	8067	4584	-0.924149588	1.42E-273	1.63E-272
KIF1C	8114	5961	-0.553583305	1.77E-113	7.57E-113
PTGES3	8161	4585	-0.940548584	5.89E-285	7.09E-284
C12orf75	8168	4772	-0.884113199	3.42E-257	3.56E-256
SAMD4A	8174	3019	-1.545695862	0	0
RAB13	8211	5109	-0.793241376	3.85E-215	3.21E-214
SFPQ	8220	3738	-1.245596201	0	0
KIAA0100	8221	5314	-0.738240042	2.11E-190	1.55E-189
CREB3L1	8236	13542	0.608700713	1.08E-206	8.65E-206
EIF5A	8256	7978	-0.158140223	2.82E-12	3.86E-12
ZCCHC24	8258	3993	-1.157043722	0	0
CAPZA1	8264	4980	-0.839418888	2.16E-238	2.06E-237
NXN	8299	3593	-1.316472948	0	0
CTSD	8352	7913	-0.186621331	1.56E-16	2.34E-16
YME1L1	8368	6212	-0.538549398	2.23E-111	9.37E-111
GPC6	8375	2782	-1.698691146	0	0
XPO6	8380	3481	-1.376172806	0	0
HTRA1	8386	6784	-0.414571036	9.01E-70	2.65E-69
H6PD	8390	3748	-1.271274234	0	0
VAT1	8391	5383	-0.749156705	2.81E-199	2.17E-198
ATM	8433	5160	-0.817399265	1.71E-232	1.58E-231
ATP8B2	8440	4502	-1.015401318	0	0
VASN	8446	4942	-0.881897612	9.31E-265	1.01E-263
CAPZB	8452	8223	-0.148352314	3.08E-11	4.13E-11
SNX19	8456	4579	-0.993667188	0	0
NUFIP2	8475	5334	-0.776719919	3.86E-214	3.19E-213

ACIN1	8490	3656	-1.324222859	0	0
SSR2	8507	8984	-0.030016902	0.16869675	0.173839129
CBX5	8510	3643	-1.332756513	0	0
NEXN	8532	4899	-0.90912105	2.05E-281	2.44E-280
PAPOLA	8545	4611	-0.998725202	0	0
CASC4	8551	7800	-0.241343398	1.06E-26	1.89E-26
PGAM1	8570	9376	0.02095299	0.330604811	0.336903689
MLL2	8588	4186	-1.145474255	0	0
IPO5	8606	5023	-0.885517941	1.35E-271	1.53E-270
SRP9	8637	5911	-0.655852445	9.25E-163	5.70E-162
SMG1	8651	4240	-1.13752702	0	0
SLC2A1	8692	13390	0.514671505	1.66E-151	9.31E-151
TPI1	8697	10733	0.194739268	6.14E-21	9.91E-21
LOXL1	8702	8275	-0.181312081	2.59E-16	3.86E-16
UHMK1	8738	5069	-0.894326318	2.38E-280	2.82E-279
DOCK1	8754	4415	-1.096253322	0	0
SDF4	8778	8425	-0.16793996	2.21E-14	3.17E-14
ZFP36L1	8788	6406	-0.564835448	3.25E-127	1.54E-126
ADM	8859	2526	-1.919013594	0	0
HIF1A	8863	5560	-0.781434606	3.48E-226	3.11E-225
MBTPS1	8874	5698	-0.747853307	5.32E-210	4.33E-209
NR2F2	8885	2523	-1.924955946	0	0
LTBP3	8896	3989	-1.265854041	0	0
STT3A	8908	9790	0.027482926	0.192744097	0.198305488
WASF2	8918	4950	-0.95801605	0	0
CDC42BPB	8920	5439	-0.82242666	2.22E-248	2.21E-247
CNOT1	8924	5441	-0.822543061	1.50E-248	1.50E-247
GDI2	8930	8164	-0.238108369	3.83E-27	6.87E-27
ACLY	8955	9046	-0.094137812	1.19E-05	1.38E-05
WLS	9004	5877	-0.724210534	1.51E-201	1.17E-200
PKD1P1	9010	3875	-1.326055172	0	0
ADD3	9012	2618	-1.892106592	0	0

TPP1	9034	6880	-0.501680725	8.67E-106	3.50E-105
SSH1	9060	5302	-0.881698756	9.52E-284	1.14E-282
SEC22B	9073	5398	-0.857879058	2.46E-271	2.80E-270
TPR	9104	2080	-2.238641405	0	0
SCAF11	9126	4862	-1.017157136	0	0
MLL	9132	3880	-1.343598583	0	0
COTL1	9147	10237	0.053698057	0.009580796	0.010271339
PTP4A2	9163	6551	-0.592829247	1.96E-144	1.05E-143
ARPC5	9168	8206	-0.268652266	1.31E-34	2.60E-34
SARS	9183	7606	-0.380551983	2.88E-65	8.09E-65
WDR26	9289	6501	-0.623586015	4.57E-160	2.74E-159
EPRS	9318	8928	-0.170407679	1.45E-15	2.13E-15
IGF1R	9380	3494	-1.53343269	0	0
PFDN5	9450	5540	-0.879152729	1.95E-294	2.50E-293
CAP1	9465	11605	0.185347736	1.33E-20	2.13E-20
TNKS1BP1	9467	5643	-0.855169348	1.61E-281	1.92E-280
PAFAH1B1	9515	5725	-0.841652339	2.08E-275	2.42E-274
ZBTB4	9538	5159	-0.995319712	0	0
ATN1	9554	4210	-1.291009019	0	0
SORT1	9605	8661	-0.257976381	1.30E-33	2.56E-33
SRSF11	9625	5080	-1.03068242	0	0
PABPC4	9692	6302	-0.729709026	1.19E-219	1.02E-218
S100A11	9704	12412	0.246359771	1.01E-36	2.07E-36
ITGA5	9717	12458	0.249765228	8.70E-38	1.81E-37
TSPAN3	9727	4687	-1.162054499	0	0
FERMT2	9744	8190	-0.359375057	2.65E-62	7.22E-62
ALDH1L2	9779	10449	-0.013118345	0.517726783	0.52376324
TXLNA	9813	5052	-1.066563997	0	0
RBMS2	9816	5617	-0.914059822	0	0
CAND1	9823	4983	-1.087873501	0	0
FAU	9843	7445	-0.511550622	3.49E-119	1.56E-118
PDLIM1	9865	6416	-0.729369285	2.34E-223	2.06E-222

NBR1	9889	7685	-0.472503755	8.28E-104	3.31E-103
CD46	9913	5814	-0.878515035	0	0
SEC23A	9994	11677	0.115811172	3.56E-09	4.56E-09
FOXF1	10012	6222	-0.795004272	7.01E-263	7.52E-262
RERE	10044	3085	-1.811715915	0	0
EIF5	10121	5034	-1.116299096	0	0
SEPN1	10140	6285	-0.798797378	2.16E-268	2.41E-267
WNK1	10156	4896	-1.161381213	0	0
NF1	10198	5269	-1.061409534	0	0
MLEC	10266	7732	-0.517684999	6.60E-127	3.12E-126
IFITM3	10302	7791	-0.511768401	9.53E-125	4.43E-124
HECTD1	10325	4530	-1.297283202	0	0
SAR1A	10364	6983	-0.678386419	8.85E-207	7.10E-206
MDM2	10409	5620	-0.997913815	0	0
JAK1	10426	5818	-0.950314939	0	0
CD151	10457	9735	-0.211940509	1.61E-25	2.82E-25
FLJ23867	10461	2188	-2.366060503	0	0
LDHB	10474	7685	-0.555419721	8.71E-147	4.78E-146
CLSTN1	10483	6855	-0.721547448	7.83E-233	7.26E-232
SERBP1	10493	6554	-0.787704008	4.26E-271	4.82E-270
C12orf23	10604	9611	-0.250574499	5.02E-35	1.00E-34
DDX6	10697	5708	-1.014873382	0	0
POSTN	10705	36480	1.660096558	0	0
ARF1	10719	9726	-0.248976199	5.61E-35	1.12E-34
HMGB1	10750	4219	-1.458088043	0	0
MPRIP	10772	6455	-0.84752151	0	0
DDR2	10784	5298	-1.134097029	0	0
XPOT	10817	8808	-0.405138438	8.50E-86	2.89E-85
TUBA1B	10913	8709	-0.434193153	3.85E-98	1.47E-97
GRN	10948	7000	-0.753964889	2.89E-262	3.08E-261
PLOD1	11047	26726	1.165864919	0	0
MXRA7	11058	8170	-0.545406869	6.49E-150	3.59E-149

EID1	11086	8825	-0.437795105	3.59E-101	1.40E-100
SMURF2	11089	6029	-0.987863011	0	0
AMOTL1	11096	3758	-1.670727097	0	0
KIAA1462	11101	5343	-1.163692097	0	0
PKD1	11159	5582	-1.108078094	0	0
CLMP	11179	3887	-1.632786503	0	0
ADAM10	11262	7651	-0.666467204	1.15E-217	9.84E-217
RAB3B	11287	17128	0.492970245	8.41E-179	5.77E-178
H3F3B	11316	6833	-0.836497426	0	0
CDC42	11375	8201	-0.580719179	2.16E-172	1.42E-171
SMAD3	11381	3072	-1.998101581	0	0
MPZL1	11402	6406	-0.940515579	0	0
LRP10	11496	10046	-0.303235163	1.25E-53	3.15E-53
TAOK1	11586	5742	-1.12148171	0	0
FRMD6	11612	13030	0.057496231	0.001762328	0.001937221
DDOST	11615	13617	0.120695364	3.17E-11	4.24E-11
RPS29	11641	7901	-0.667832205	9.91E-226	8.83E-225
TJP1	11646	6025	-1.059525753	0	0
IQGAP1	11670	11810	-0.091519972	1.15E-06	1.38E-06
P4HA1	11712	25036	0.987292247	0	0
GFRA1	11720	2720	-2.216018389	0	0
LIMA1	11765	7365	-0.784468266	1.41E-301	1.85E-300
WSB1	11782	5895	-1.107745116	0	0
SLC38A1	11783	7117	-0.836090137	0	0
C17orf76-AS1	11805	8639	-0.559186188	3.39E-167	2.14E-166
UACA	11845	6690	-0.932924458	0	0
COPB1	11858	9950	-0.361816647	2.29E-76	7.15E-76
EFEMP2	11924	9531	-0.43189316	4.97E-106	2.01E-105
OS9	11934	9775	-0.39663345	7.00E-91	2.48E-90
DDB1	11990	9379	-0.46305002	5.89E-121	2.67E-120
KTN1	12013	4590	-1.496754797	0	0
CHD4	12095	6730	-0.954456735	0	0

HP1BP3	12171	6607	-0.990104834	0	0
NFE2L1	12205	9710	-0.43865347	1.37E-111	5.79E-111
KPNB1	12214	7926	-0.732595254	3.51E-278	4.12E-277
KIF5B	12224	8179	-0.688444455	7.08E-250	7.11E-249
PMP22	12296	1731	-2.937235816	0	0
STAT3	12303	5215	-1.346995368	0	0
DYNC1LI2	12447	7695	-0.802529207	0	0
MYOF	12526	17697	0.389854655	2.05E-120	9.27E-120
LGALS3BP	12535	7637	-0.823608443	0	0
EIF3F	12586	9862	-0.460592066	1.17E-125	5.50E-125
MYO1C	12590	10404	-0.383864355	3.22E-90	1.13E-89
SDC3	12591	5503	-1.302827011	0	0
GALNT2	12686	9279	-0.559920384	6.00E-180	4.13E-179
FOXN3	12719	6388	-1.102273405	0	0
TMEM123	12731	6634	-1.049119204	0	0
ANKRD11	12753	6279	-1.130954323	0	0
LMNA	12822	11256	-0.296651456	2.97E-57	7.74E-57
YAP1	12861	8543	-0.698912511	8.32E-270	9.36E-269
CHD3	12891	7487	-0.892628899	0	0
ADAR	12913	7481	-0.896245552	0	0
CNN3	12932	14706	0.076735102	9.87E-06	1.15E-05
MYH10	12982	7891	-0.826956992	0	0
PRRC2C	13010	7055	-0.99162735	0	0
ARCN1	13063	11586	-0.281828065	4.70E-53	1.17E-52
MCL1	13125	8591	-0.720143821	5.05E-290	6.33E-289
TMED10	13151	11902	-0.252692859	1.06E-43	2.39E-43
ECE1	13165	9672	-0.553545748	6.88E-183	4.86E-182
RPS13	13254	10672	-0.421321629	1.86E-112	7.91E-112
CD248	13300	7311	-0.972009965	0	0
TNFRSF19	13351	844	-4.092285372	0	0
STAT2	13359	5790	-1.31490114	0	0
GNS	13594	10284	-0.511292889	1.02E-163	6.31E-163

RNF213	13618	6176	-1.249494556	0	0
MORF4L1	13648	9484	-0.63384636	4.23E-241	4.08E-240
THY1	13696	18273	0.307234528	4.02E-80	1.29E-79
LDHA	13812	20549	0.464421573	3.73E-192	2.77E-191
AGRN	13842	15079	0.01476397	0.384067379	0.390366024
SEL1L	13867	8759	-0.771542009	0	0
CPD	13879	8815	-0.763595525	0	0
RPL38	13942	10209	-0.558320351	1.46E-196	1.11E-195
IVNS1ABP	13979	4730	-1.672073447	0	0
HNRNPC	14068	10525	-0.527321383	5.01E-179	3.44E-178
PRKAR1A	14157	7819	-0.96518394	0	0
MAP1A	14195	15969	0.061166747	0.000231965	0.00026101
NUMA1	14224	6340	-1.27449686	0	0
ZEB1	14253	8497	-0.854964518	0	0
NEO1	14461	4843	-1.686918788	0	0
PTPN11	14503	6523	-1.2614682	0	0
COPA	14540	13222	-0.245811225	9.56E-46	2.20E-45
SACS	14646	4182	-1.91696611	0	0
TM9SF3	14656	9903	-0.67427824	1.69E-288	2.10E-287
AP2B1	14690	9466	-0.742731945	0	0
FENDRR	14757	5864	-1.440166828	0	0
EIF3A	14796	9004	-0.82529362	0	0
CAPN2	14804	15482	-0.044119562	0.007750971	0.008340545
PHLDA1	14853	5856	-1.451491272	0	0
GNB1	14964	12450	-0.374074505	6.20E-102	2.43E-101
SLC25A3	15007	14241	-0.184309522	8.23E-28	1.49E-27
CFL1	15046	17075	0.073781207	4.61E-06	5.44E-06
CTTN	15080	8558	-0.92601522	0	0
CSRP1	15167	15402	-0.086542426	1.54E-07	1.89E-07
PRKCA	15305	9426	-0.808009834	0	0
SEPT9	15343	10010	-0.724863001	0	0
ATP5B	15389	12057	-0.460752879	2.59E-153	1.48E-152

C1orf198	15437	10965	-0.602210973	7.90E-249	7.90E-248
CAPRIN1	15527	8510	-0.97627245	0	0
NFAT5	15608	9328	-0.851370365	0	0
CYR61	15663	22992	0.445047026	1.02E-198	7.85E-198
PFN1	15686	17179	0.022444177	0.158375242	0.163375465
ACTA2	15721	47688	1.492208913	0	0
RPS26	15755	12802	-0.408164917	8.27E-126	3.89E-125
NEAT1	15840	6046	-1.498243827	0	0
RPL27	15891	12865	-0.413482833	6.43E-130	3.10E-129
PPAP2B	15892	2327	-2.880481959	0	0
PCBP2	16001	9641	-0.839631746	0	0
S100A6	16042	21950	0.343642546	5.53E-118	2.46E-117
COL16A1	16126	8681	-1.002179856	0	0
CAMK2N1	16139	6198	-1.489400905	0	0
YWHAE	16161	10761	-0.695428696	0	0
POLR2A	16268	5981	-1.552302649	0	0
ARHGEF12	16272	9115	-0.944801399	0	0
TMED2	16282	13176	-0.41408984	2.00E-133	9.81E-133
MXRA8	16307	7145	-1.299209853	0	0
TMBIM6	16415	10915	-0.697426995	0	0
GNG12	16546	7022	-1.345252962	0	0
MFAP4	16561	8307	-1.104114706	0	0
THSD4	16595	9406	-0.927819768	0	0
ZFP106	16785	6309	-1.520413657	0	0
LMO7	16855	17350	-0.06696534	1.74E-05	2.02E-05
PRPF8	16906	8822	-1.047082065	0	0
SH3PXD2A	16920	12172	-0.583887705	5.81E-258	6.08E-257
FKBP10	17067	32427	0.817261696	0	0
PKM	17083	28034	0.605891879	0	0
GLG1	17214	17667	-0.07124967	3.92E-06	4.64E-06
BCAT1	17236	16281	-0.190960065	7.91E-34	1.56E-33
CTDSP2	17277	8439	-1.142433145	0	0

PEAK1	17283	6620	-1.493174917	0	0
RPL22	17288	10458	-0.833888378	0	0
CORO1C	17300	16295	-0.195067062	2.66E-35	5.34E-35
WARS	17326	9107	-1.036615209	0	0
CADM1	17468	16495	-0.191410034	2.07E-34	4.12E-34
NTN4	17485	6756	-1.480600939	0	0
USP22	17496	8890	-1.085494177	0	0
ENO1	17718	21004	0.136723963	1.21E-20	1.95E-20
PEA15	17858	10341	-0.896919187	0	0
PTPN14	17960	7587	-1.351910283	0	0
CD81	18091	16330	-0.256471742	4.07E-61	1.10E-60
PRDX6	18400	29231	0.559069043	0	0
ITM2B	18457	13695	-0.539243186	6.87E-244	6.72E-243
LAMP1	18460	19570	-0.024483173	0.097757929	0.101555364
UBB	18511	14154	-0.495897386	2.74E-210	2.23E-209
TXNIP	18551	1798	-3.475756408	0	0
YBX1	18969	15159	-0.432193415	9.11E-168	5.79E-167
MYL6	18982	20829	0.025237194	0.08084582	0.084232823
ATP2B4	19014	8008	-1.356272562	0	0
NOTCH2	19162	6786	-1.606339188	0	0
TMTC1	19391	4035	-2.373471003	0	0
RPS25	19619	13138	-0.687230213	0	0
GANAB	19624	25843	0.288430041	4.52E-100	1.75E-99
NID2	19677	6732	-1.656127575	0	0
ATP2A2	19970	16207	-0.409941643	3.11E-160	1.87E-159
UBC	20092	14876	-0.542358903	4.36E-268	4.84E-267
ABCC1	20418	4076	-2.433339976	0	0
CDH11	20471	7290	-1.598315235	0	0
CD59	20531	30167	0.446442959	1.20E-261	1.27E-260
PDIA3	20750	32166	0.523700829	0	0
NUCKS1	21196	9916	-1.204686232	0	0
EIF4B	21272	15645	-0.5519784	7.96E-293	1.01E-291

CD63	21379	30192	0.389247579	1.57E-203	1.23E-202
ALDOA	21581	26056	0.163129532	6.68E-35	1.33E-34
SHC1	21654	19857	-0.233710248	3.10E-61	8.39E-61
TXNRD1	21703	6589	-1.828487424	0	0
EIF1	21706	14923	-0.649280673	0	0
NACA	21723	14430	-0.698876433	0	0
NAP1L1	21751	15391	-0.607719135	0	0
HNRNPU	22495	10635	-1.18950871	0	0
APLP2	22495	17219	-0.494327383	1.12E-253	1.15E-252
DKK3	22653	33247	0.444797668	3.07E-286	3.73E-285
UBR4	23172	14011	-0.834547018	0	0
TUBA1A	23228	12893	-0.95800132	0	0
NID1	23423	7779	-1.698993631	0	0
RPLP2	23548	18846	-0.43007056	6.05E-206	4.84E-205
MRC2	23878	27090	0.073354015	9.48E-09	1.20E-08
CLTC	24053	20472	-0.341289168	2.60E-137	1.31E-136
CKAP4	24146	35768	0.458160862	0	0
RPL5	24181	19636	-0.409097212	6.79E-193	5.08E-192
MMP14	24663	18975	-0.48697279	1.74E-270	1.97E-269
GREM1	24999	12654	-1.091001261	0	0
C1R	25322	5751	-2.24723099	0	0
RPS24	25513	21163	-0.378412773	3.43E-176	2.31E-175
RCN1	26174	25965	-0.120290569	1.58E-21	2.58E-21
LASP1	26435	24954	-0.191902612	1.89E-51	4.66E-51
PARVA	26798	17872	-0.693148618	0	0
RPS15A	26902	20027	-0.534491483	0	0
RPL23A	26912	18991	-0.611658112	0	0
TEAD1	27200	12211	-1.264149675	0	0
F3	28613	31006	0.007152275	0.544707264	0.550559025
TPM1	29020	55704	0.832009033	0	0
LTBP2	29186	82158	1.384400186	0	0
FBLN5	29197	19853	-0.665187485	0	0

RPL11	29389	21733	-0.54411331	0	0
GABARAPL1	29450	11426	-1.474671575	0	0
PTRF	29496	26167	-0.281495166	2.80E-117	1.24E-116
HSP90AA1	29935	17991	-0.843282267	0	0
RPS3	30045	24424	-0.407549803	1.44E-237	1.37E-236
ACTN1	30513	39406	0.260266906	4.75E-125	2.22E-124
SRRM2	30758	15414	-1.105444777	0	0
FARP1	31142	14070	-1.254963647	0	0
B2M	31798	25187	-0.444981109	2.31E-295	2.98E-294
ANXA2	31872	26873	-0.354856541	5.53E-195	4.17E-194
DDX5	32219	16815	-1.046887314	0	0
VCL	32398	26383	-0.405020509	4.10E-253	4.19E-252
MACF1	32730	34661	-0.0260247	0.019081062	0.020291981
CCND1	33250	30822	-0.218118237	9.43E-82	3.07E-81
CSDE1	33751	22469	-0.695718706	0	0
RPL19	34115	24839	-0.566523499	0	0
RPL23	34431	24984	-0.571427984	0	0
HSPA8	34557	25465	-0.549186637	0	0
DYNC1H1	35127	21826	-0.79525699	0	0
SERPINH1	35421	65464	0.777372439	0	0
C1S	36111	10426	-1.900976983	0	0
MEG3	36119	12168	-1.678390268	0	0
RPL6	37343	26547	-0.601013374	0	0
TAGLN	38126	87605	1.091513659	0	0
PSAP	38246	24258	-0.765572636	0	0
RPL27A	38631	32149	-0.373709536	8.82E-260	9.31E-259
HSPG2	38783	20831	-1.005416683	0	0
RPL26	38882	27429	-0.61212478	0	0
RPL41	39452	27939	-0.60654243	0	0
ARID5B	39817	18788	-1.192297403	0	0
TIMP2	40951	38697	-0.190401333	1.35E-77	4.26E-77
EEF1G	41005	32882	-0.427226162	0	0

CTSC	43188	15482	-1.58876303	0	0
GAS6	43610	78865	0.746001813	0	0
RPS27	44050	28877	-0.717945525	0	0
RPS8	44727	30645	-0.654218666	0	0
HSP90B1	44933	68682	0.503432287	0	0
RPL13	45261	28629	-0.769515557	0	0
RPLP1	47463	35744	-0.517826577	0	0
CLIC4	48644	41219	-0.347676738	1.26E-285	1.52E-284
LAMC1	49739	25099	-1.095471993	0	0
MFGE8	49937	38518	-0.483300734	0	0
MMP2	54640	76879	0.383907711	0	0
ACTG1	56314	139951	1.204631885	0	0
LUM	57219	7605	-3.020198507	0	0
RPL4	59913	46420	-0.476846928	0	0
EIF4G2	61800	30471	-1.128894367	0	0
RPS2	67664	55950	-0.38297471	0	0
SLC38A2	68169	30602	-1.264214252	0	0
RPLP0	72480	54776	-0.512763414	0	0
GAPDH	78923	87875	0.046282612	5.25E-11	7.01E-11
IGFBP4	79489	31661	-1.436772781	0	0
ITGB1	80008	124078	0.524306794	0	0
FTH1	83708	35041	-1.365045937	0	0
COL4A2	94968	118598	0.211841929	2.00E-252	2.03E-251
FBN1	99392	71331	-0.587324912	0	0
COL4A1	104715	135792	0.266205994	0	0
TPT1	122823	77371	-0.775440297	0	0
KIAA1199	128663	27542	-2.332616377	0	0
CD44	130250	63975	-1.134427601	0	0
VIM	141740	118230	-0.370375175	0	0
LRP1	146030	92905	-0.761161011	0	0
AHNAK	168033	80893	-1.163382197	0	0
MALAT1	179030	91854	-1.071511282	0	0

THBS1	196274	168475	-0.329058925	0	0
QSOX1	214328	49898	-2.211490817	0	0
DCN	457651	45243	-3.447205708	0	0
COL1A1	774687	1025788	0.296322788	0	0
