# Supplementary material for: The exosomal miRNA/FOXN3 axis empowers fibroblasts with tumor-promoting functions via enhanced migration and altered secretome
Source: J Nanobiotechnology. 2026 Jun 5;24:742. doi: 10.1186/s12951-026-04633-6 (PMC13459620; doi:10.1186/s12951-026-04633-6)
Supplement: Supplementary file 2 [file 12951_2026_4633_MOESM3_ESM.pdf]

Table S2 miRNA profiles of A549-derived exosomes

Gene symbol	Raw count	Normalized raw count
hsa-miR-22-3p	168279	14.03461804
hsa-miR-24-3p	166022	13.84638224
hsa-miR-21-5p	107208	8.941242406
hsa-miR-21-3p	75573	6.302855313
hsa-miR-320a	72700	6.063244561
hsa-miR-27b-3p	55321	4.613820528
hsa-miR-1246	45745	3.815173624
hsa-miR-320c	37663	3.141127647
hsa-miR-92b-3p	31968	2.666159589
hsa-miR-92a-3p	31298	2.610280994
hsa-miR-192-5p	31055	2.590014578
hsa-miR-486-5p	29719	2.478590992
hsa-miR-100-5p	28056	2.339895315
hsa-miR-423-5p	26089	2.175845768
hsa-miR-378a-3p	25186	2.100534766
hsa-miR-1307-5p	20114	1.677525462
hsa-miR-320d	16610	1.385288751
hsa-miR-27a-3p	16504	1.376448256
hsa-miR-423-3p	15532	1.295382593
hsa-let-7i-5p	14062	1.172783288
hsa-miR-29a-3p	13674	1.140423743
hsa-miR-10a-5p	11348	0.946433278
hsa-miR-99b-5p	10965	0.914490737
hsa-miR-30a-5p	10855	0.90531664
hsa-miR-191-5p	8155	0.680134242
hsa-miR-345-5p	8085	0.67429618
hsa-miR-140-3p	6159	0.51366607
hsa-miR-130a-3p	5838	0.486894384
hsa-miR-16-5p	4672	0.389648949
hsa-miR-193b-3p	4474	0.373135573
hsa-miR-146a-5p	4109	0.342694249
hsa-let-7a-5p	3626	0.30241162
hsa-let-7f-5p	3037	0.253288497
hsa-miR-138-5p	2902	0.242029377
hsa-miR-182-5p	2842	0.237025324
hsa-miR-4516	2717	0.226600213
hsa-miR-25-3p	2415	0.201413145
hsa-miR-28-3p	2410	0.20099614
hsa-miR-589-5p	2336	0.194824474
hsa-miR-183-5p	2331	0.19440747
hsa-let-7b-5p	2291	0.191071435
hsa-miR-221-3p	2232	0.186150782
hsa-miR-484	2198	0.183315152
hsa-miR-30d-5p	2026	0.168970199
hsa-miR-222-3p	1971	0.16438315
hsa-miR-130b-3p	1946	0.162298128

hsa-miR-138-1-3p	1942	0.161964525
hsa-miR-31-5p	1872	0.156126462
hsa-miR-26a-5p	1853	0.154541846
hsa-miR-3615	1834	0.152957229
hsa-miR-629-5p	1662	0.138612276
hsa-miR-23a-3p	1563	0.130355588
hsa-miR-128	1369	0.114175816
hsa-miR-181a-5p	1323	0.110339375
hsa-miR-744-5p	1319	0.110005771
hsa-miR-339-3p	1206	0.100581471
hsa-miR-501-3p	1104	0.09207458
hsa-miR-19b-3p	1068	0.089072148
hsa-miR-30e-5p	1055	0.087987937
hsa-let-7g-5p	993	0.082817082
hsa-miR-151a-3p	977	0.081482668
hsa-miR-224-5p	893	0.074476993
hsa-miR-151a-5p	864	0.072058367
hsa-miR-186-5p	861	0.071808165
hsa-miR-106b-5p	771	0.064302085
hsa-miR-26b-5p	727	0.060632446
hsa-miR-151b	713	0.059464833
hsa-miR-93-5p	712	0.059381432
hsa-miR-152	695	0.057963617
hsa-miR-323a-3p	679	0.056629203
hsa-miR-425-5p	624	0.052042154
hsa-miR-200a-3p	609	0.050791141
hsa-miR-15a-5p	553	0.046120691
hsa-miR-1910	546	0.045536885
hsa-miR-4306	508	0.042367651
hsa-miR-502-3p	501	0.041783845
hsa-miR-1307-3p	495	0.04128344
hsa-miR-30a-3p	468	0.039031616
hsa-miR-181b-5p	462	0.03853121
hsa-miR-4488	450	0.0375304
hsa-miR-17-5p	449	0.037446999
hsa-miR-103a-3p	435	0.036279386
hsa-miR-4466	418	0.034861571
hsa-miR-877-5p	411	0.034277765
hsa-miR-342-3p	410	0.034194364
hsa-miR-3176	405	0.03377736
hsa-miR-210	399	0.033276954
hsa-miR-320b	394	0.03285995
hsa-let-7a-3p	393	0.032776549
hsa-miR-301a-3p	384	0.032025941
hsa-miR-29c-3p	371	0.030941729
hsa-miR-671-3p	333	0.027772496
hsa-miR-125a-3p	324	0.027021888
hsa-miR-148b-3p	309	0.025770874

hsa-let-7e-5p	306	0.025520672
hsa-miR-134	302	0.025187068
hsa-miR-4508	289	0.024102857
hsa-miR-132-3p	287	0.023936055
hsa-miR-654-3p	285	0.023769253
hsa-miR-10a-3p	281	0.02343565
hsa-miR-1247-5p	279	0.023268848
hsa-miR-126-5p	278	0.023185447
hsa-miR-101-3p	273	0.022768442
hsa-miR-369-3p	273	0.022768442
hsa-miR-27b-5p	270	0.02251824
hsa-miR-33b-5p	260	0.021684231
hsa-miR-486-3p	254	0.021183826
hsa-miR-3187-3p	249	0.020766821
hsa-miR-3677-3p	247	0.020600019
hsa-miR-500a-3p	242	0.020183015
hsa-miR-20a-5p	232	0.019349006
hsa-miR-3180	228	0.019015402
hsa-miR-361-5p	226	0.018848601
hsa-miR-19a-3p	202	0.016846979
hsa-miR-323b-3p	201	0.016763578
hsa-miR-335-5p	197	0.016429975
hsa-miR-424-3p	194	0.016179772
hsa-miR-381-3p	192	0.016012971
hsa-miR-410	191	0.01592957
hsa-miR-122-5p	191	0.01592957
hsa-miR-143-3p	189	0.015762768
hsa-let-7d-5p	189	0.015762768
hsa-miR-7-5p	179	0.014928759
hsa-miR-4497	176	0.014678556
hsa-miR-30c-5p	174	0.014511755
hsa-miR-1268a	172	0.014344953
hsa-miR-98-5p	170	0.014178151
hsa-let-7d-3p	167	0.013927948
hsa-miR-450b-5p	166	0.013844547
hsa-miR-1287	163	0.013594345
hsa-miR-452-5p	162	0.013510944
hsa-miR-941	160	0.013344142
hsa-miR-99b-3p	154	0.012843737
hsa-miR-185-5p	152	0.012676935
hsa-miR-200b-3p	152	0.012676935
hsa-miR-212-3p	152	0.012676935
hsa-miR-1180	151	0.012593534
hsa-miR-23b-3p	151	0.012593534
hsa-miR-92b-5p	141	0.011759525
hsa-miR-3195	135	0.01125912
hsa-miR-30b-5p	135	0.01125912
hsa-miR-769-5p	134	0.011175719

hsa-miR-378d	131	0.010925516
hsa-miR-22-5p	130	0.010842115
hsa-miR-197-3p	129	0.010758715
hsa-miR-760	128	0.010675314
hsa-miR-34a-5p	128	0.010675314
hsa-miR-127-3p	120	0.010008107
hsa-miR-125a-5p	111	0.009257499
hsa-miR-196a-5p	94	0.007839683
hsa-miR-125b-5p	69	0.005754661
hsa-miR-454-3p	85	0.007089075
hsa-miR-181c-5p	83	0.006922274
hsa-miR-29b-3p	69	0.005754661
hsa-miR-196b-5p	36	0.003002432
hsa-let-7c	63	0.005254256
hsa-miR-9-5p	53	0.004420247
hsa-miR-374a-5p	61	0.005087454
hsa-miR-221-5p	55	0.004587049
hsa-miR-181a-3p	59	0.004920652
hsa-miR-374a-3p	52	0.004336846
hsa-miR-4286	15	0.001251013
hsa-miR-27a-5p	84	0.007005675
hsa-miR-582-3p	72	0.006004864
hsa-miR-340-5p	64	0.005337657
hsa-miR-146b-5p	96	0.008006485
hsa-miR-28-5p	31	0.002585428
hsa-miR-374b-5p	53	0.004420247
hsa-miR-92a-1-5p	74	0.006171666
hsa-miR-107	23	0.00191822
hsa-miR-181a-2-3p	46	0.003836441
hsa-miR-532-5p	98	0.008173287
hsa-miR-106b-3p	40	0.003336036
hsa-miR-181d	47	0.003919842
hsa-miR-335-3p	24	0.002001621
hsa-miR-1275	48	0.004003243
hsa-miR-100-3p	30	0.002502027
hsa-miR-582-5p	20	0.001668018
hsa-miR-660-5p	117	0.009757904
hsa-let-7f-2-3p	80	0.006672071
hsa-miR-361-3p	21	0.001751419
hsa-miR-125b-1-3p	34	0.00283563
hsa-miR-30e-3p	50	0.004170044
hsa-miR-30c-2-3p	32	0.002668828
hsa-miR-421	64	0.005337657
hsa-miR-17-3p	23	0.00191822
hsa-miR-18a-5p	16	0.001334414
hsa-miR-15b-3p	32	0.002668828
hsa-miR-194-5p	30	0.002502027
hsa-miR-140-5p	14	0.001167612

hsa-miR-429	62	0.005170855
hsa-miR-324-5p	35	0.002919031
hsa-miR-96-5p	33	0.002752229
hsa-miR-671-5p	39	0.003252635
hsa-let-7i-3p	106	0.008840494
hsa-miR-15b-5p	91	0.007589481
hsa-miR-339-5p	31	0.002585428
hsa-miR-424-5p	86	0.007172476
hsa-miR-561-5p	57	0.004753851
hsa-miR-1299	37	0.003085833
hsa-miR-590-3p	18	0.001501216
hsa-miR-34c-5p	21	0.001751419
hsa-miR-25-5p	74	0.006171666
hsa-miR-548k	18	0.001501216
hsa-miR-455-5p	40	0.003336036
hsa-miR-218-5p	25	0.002085022
hsa-miR-542-3p	9	0.000750608
hsa-miR-126-3p	16	0.001334414
hsa-miR-99a-5p	60	0.005004053
hsa-miR-330-5p	16	0.001334414
hsa-miR-30d-3p	11	0.00091741
hsa-miR-625-5p	24	0.002001621
hsa-miR-331-3p	4	0.000333604
hsa-miR-503-5p	34	0.00283563
hsa-miR-2467-5p	6	0.000500405
hsa-miR-1260b	2	0.000166802
hsa-miR-454-5p	23	0.00191822
hsa-miR-32-3p	8	0.000667207
hsa-miR-16-2-3p	10	0.000834009
hsa-miR-130b-5p	2	0.000166802
hsa-miR-200a-5p	24	0.002001621
hsa-miR-301b	68	0.00567126
hsa-miR-33a-5p	5	0.000417004
hsa-miR-141-3p	45	0.00375304
hsa-miR-4521	6	0.000500405
hsa-miR-550a-3p	118	0.009841305
hsa-miR-652-3p	63	0.005254256
hsa-miR-31-3p	1	8.34009E-05
hsa-miR-147b	7	0.000583806
hsa-miR-195-5p	13	0.001084212
hsa-miR-548ay-5p	9	0.000750608
hsa-miR-374b-3p	4	0.000333604
hsa-miR-769-3p	5	0.000417004
hsa-miR-365a-3p	4	0.000333604
hsa-miR-32-5p	60	0.005004053
hsa-miR-450a-5p	5	0.000417004
hsa-miR-193a-3p	102	0.008506891
hsa-miR-148a-3p	65	0.005421058

hsa-miR-1285-3p	60	0.005004053
hsa-miR-641	8	0.000667207
hsa-miR-33b-3p	8	0.000667207
hsa-miR-4677-3p	7	0.000583806
hsa-miR-1908	57	0.004753851
hsa-miR-379-5p	19	0.001584617
hsa-miR-409-3p	96	0.008006485
hsa-miR-1296	29	0.002418626
hsa-miR-493-5p	64	0.005337657
hsa-miR-550a-5p	13	0.001084212
hsa-miR-132-5p	8	0.000667207
hsa-let-7b-3p	13	0.001084212
hsa-miR-193b-5p	46	0.003836441
hsa-miR-18a-3p	8	0.000667207
hsa-miR-222-5p	18	0.001501216
hsa-miR-7-1-3p	51	0.004253445
hsa-miR-30b-3p	6	0.000500405
hsa-miR-3158-3p	92	0.007672882
hsa-miR-193a-5p	83	0.006922274
hsa-miR-576-3p	30	0.002502027
hsa-let-7f-1-3p	17	0.001417815
hsa-miR-628-5p	7	0.000583806
hsa-let-7e-3p	6	0.000500405
hsa-miR-4326	43	0.003586238
hsa-miR-149-5p	17	0.001417815
hsa-miR-29b-1-5p	1	8.34009E-05
hsa-miR-455-3p	1	8.34009E-05
hsa-miR-148b-5p	41	0.003419436
hsa-miR-190a	2	0.000166802
hsa-miR-200b-5p	34	0.00283563
hsa-let-7g-3p	14	0.001167612
hsa-miR-378a-5p	1	8.34009E-05
hsa-miR-4500	2	0.000166802
hsa-miR-324-3p	19	0.001584617
hsa-miR-9-3p	57	0.004753851
hsa-miR-29a-5p	6	0.000500405
hsa-miR-1294	0	0
hsa-miR-2277-5p	17	0.001417815
hsa-miR-95	3	0.000250203
hsa-miR-375	117	0.009757904
hsa-miR-136-3p	80	0.006672071
hsa-miR-328	102	0.008506891
hsa-miR-550a-3-5p	7	0.000583806
hsa-miR-93-3p	37	0.003085833
hsa-miR-411-5p	21	0.001751419
hsa-miR-340-3p	4	0.000333604
hsa-miR-4775	4	0.000333604
hsa-miR-3182	60	0.005004053

hsa-miR-889	31	0.002585428
hsa-miR-1268b	7	0.000583806
hsa-miR-542-5p	8	0.000667207
hsa-miR-2355-5p	40	0.003336036
hsa-miR-10b-5p	100	0.008340089
hsa-miR-3934-5p	20	0.001668018
hsa-miR-200c-3p	4	0.000333604
hsa-miR-30c-1-3p	6	0.000500405
hsa-miR-574-3p	64	0.005337657
hsa-miR-365a-5p	5	0.000417004
hsa-miR-585	2	0.000166802
hsa-miR-331-5p	23	0.00191822
hsa-miR-497-5p	18	0.001501216
hsa-miR-98-3p	3	0.000250203
hsa-miR-137	0	0
hsa-miR-362-3p	91	0.007589481
hsa-miR-4671-3p	6	0.000500405
hsa-miR-330-3p	16	0.001334414
hsa-miR-1269b	0	0
hsa-miR-181b-3p	0	0
hsa-miR-940	68	0.00567126
hsa-miR-548l	1	8.34009E-05
hsa-miR-1303	7	0.000583806
hsa-miR-185-3p	2	0.000166802
hsa-miR-129-5p	5	0.000417004
hsa-miR-589-3p	53	0.004420247
hsa-miR-873-5p	63	0.005254256
hsa-miR-616-5p	3	0.000250203
hsa-miR-26b-3p	0	0
hsa-miR-194-3p	0	0
hsa-miR-382-5p	9	0.000750608
hsa-miR-548e	4	0.000333604
hsa-miR-625-3p	44	0.003669639
hsa-miR-188-5p	24	0.002001621
hsa-miR-4746-5p	15	0.001251013
hsa-miR-548o-3p	6	0.000500405
hsa-miR-493-3p	15	0.001251013
hsa-miR-370	30	0.002502027
hsa-miR-99a-3p	3	0.000250203
hsa-miR-505-3p	10	0.000834009
hsa-miR-299-3p	8	0.000667207
hsa-miR-1305	42	0.003502837
hsa-miR-142-5p	19	0.001584617
hsa-miR-874	88	0.007339278
hsa-miR-1297	2	0.000166802
hsa-miR-1468	6	0.000500405
hsa-miR-432-5p	7	0.000583806
hsa-miR-24-2-5p	4	0.000333604

hsa-miR-449a	3	0.000250203
hsa-miR-20a-3p	1	8.34009E-05
hsa-miR-378b	34	0.00283563
hsa-miR-2110	24	0.002001621
hsa-miR-378f	24	0.002001621
hsa-miR-219-5p	8	0.000667207
hsa-miR-16-1-3p	0	0
hsa-miR-876-5p	12	0.001000811
hsa-miR-937-3p	11	0.00091741
hsa-miR-548n	1	8.34009E-05
hsa-miR-376c-3p	62	0.005170855
hsa-miR-3909	52	0.004336846
hsa-miR-576-5p	4	0.000333604
hsa-miR-573	3	0.000250203
hsa-miR-548au-5p	3	0.000250203
hsa-miR-190b	2	0.000166802
hsa-miR-342-5p	2	0.000166802
hsa-miR-362-5p	1	8.34009E-05
hsa-miR-642a-5p	0	0
hsa-miR-487b	64	0.005337657
hsa-miR-584-5p	62	0.005170855
hsa-miR-425-3p	43	0.003586238
hsa-miR-1286	15	0.001251013
hsa-miR-1254	5	0.000417004
hsa-miR-1266	46	0.003836441
hsa-miR-548c-5p	0	0
hsa-miR-106a-5p	0	0
hsa-miR-4517	0	0
hsa-miR-2277-3p	41	0.003419436
hsa-miR-3065-3p	2	0.000166802
hsa-miR-4768-5p	0	0
hsa-miR-212-5p	0	0
hsa-miR-2116-5p	51	0.004253445
hsa-miR-543	46	0.003836441
hsa-miR-154-5p	19	0.001584617
hsa-miR-219-1-3p	5	0.000417004
hsa-miR-5000-3p	4	0.000333604
hsa-miR-141-5p	2	0.000166802
hsa-miR-431-5p	27	0.002251824
hsa-miR-196b-3p	8	0.000667207
hsa-miR-3144-3p	5	0.000417004
hsa-miR-26a-2-3p	2	0.000166802
hsa-miR-3940-5p	32	0.002668828
hsa-miR-642a-3p	31	0.002585428
hsa-miR-532-3p	0	0
hsa-miR-2116-3p	7	0.000583806
hsa-miR-203a	3	0.000250203
hsa-miR-3614-5p	1	8.34009E-05

hsa-miR-382-3p	69	0.005754661
hsa-miR-24-1-5p	2	0.000166802
hsa-miR-503-3p	1	8.34009E-05
hsa-miR-19b-1-5p	0	0
hsa-miR-34c-3p	113	0.0094243
hsa-miR-205-5p	5	0.000417004
hsa-miR-1301	2	0.000166802
hsa-miR-3912	2	0.000166802
hsa-miR-301a-5p	2	0.000166802
hsa-miR-34a-3p	1	8.34009E-05
hsa-miR-548j	1	8.34009E-05
hsa-miR-4484	0	0
hsa-miR-873-3p	34	0.00283563
hsa-miR-3664-3p	27	0.002251824
hsa-miR-3928	22	0.00183482
hsa-miR-1276	19	0.001584617
hsa-miR-937-5p	12	0.001000811
hsa-miR-4661-5p	3	0.000250203
hsa-miR-188-3p	2	0.000166802
hsa-miR-3913-5p	0	0
hsa-miR-494	70	0.005838062
hsa-miR-4448	12	0.001000811
hsa-miR-338-3p	4	0.000333604
hsa-miR-3065-5p	2	0.000166802
hsa-miR-942	2	0.000166802
hsa-miR-26a-1-3p	1	8.34009E-05
hsa-miR-411-3p	0	0
hsa-miR-409-5p	0	0
hsa-miR-664a-3p	0	0
hsa-miR-545-5p	0	0
hsa-miR-378g	22	0.00183482
hsa-miR-4664-3p	22	0.00183482
hsa-miR-3174	14	0.001167612
hsa-miR-199b-3p	7	0.000583806
hsa-miR-139-5p	3	0.000250203
hsa-miR-29b-2-5p	2	0.000166802
hsa-miR-125b-2-3p	2	0.000166802
hsa-miR-3141	0	0
hsa-miR-215	57	0.004753851
hsa-miR-3605-5p	43	0.003586238
hsa-miR-628-3p	16	0.001334414
hsa-miR-3944-3p	4	0.000333604
hsa-miR-195-3p	3	0.000250203
hsa-miR-1262	1	8.34009E-05
hsa-miR-3662	0	0
hsa-miR-580	0	0
hsa-miR-33a-3p	0	0
hsa-miR-4435	26	0.002168423

hsa-miR-627	21	0.001751419
hsa-miR-744-3p	5	0.000417004
hsa-miR-377-5p	3	0.000250203
hsa-let-7a-2-3p	2	0.000166802
hsa-miR-939-5p	2	0.000166802
hsa-miR-103a-2-5p	1	8.34009E-05
hsa-miR-548d-5p	1	8.34009E-05
hsa-miR-545-3p	1	8.34009E-05
hsa-miR-4791	0	0
hsa-miR-3177-3p	86	0.007172476
hsa-miR-182-3p	27	0.002251824
hsa-miR-766-3p	6	0.000500405
hsa-miR-4802-3p	5	0.000417004
hsa-miR-3200-3p	2	0.000166802
hsa-miR-3676-3p	1	8.34009E-05
hsa-miR-500a-5p	0	0
hsa-miR-491-5p	0	0
hsa-miR-4523	0	0
hsa-miR-487a	40	0.003336036
hsa-miR-3126-5p	36	0.003002432
hsa-miR-539-3p	18	0.001501216
hsa-miR-4762-5p	14	0.001167612
hsa-miR-3676-5p	9	0.000750608
hsa-miR-1292-5p	6	0.000500405
hsa-miR-1197	4	0.000333604
hsa-miR-3611	2	0.000166802
hsa-miR-561-3p	2	0.000166802
hsa-miR-101-5p	1	8.34009E-05
hsa-miR-191-3p	1	8.34009E-05
hsa-miR-4714-3p	1	8.34009E-05
hsa-miR-365b-5p	0	0
hsa-miR-758-3p	0	0
hsa-miR-4671-5p	0	0
hsa-miR-548al	0	0
hsa-miR-4802-5p	0	0
hsa-miR-654-5p	0	0
hsa-miR-3129-3p	0	0
hsa-miR-3115	0	0
hsa-miR-1915-3p	110	0.009174098
hsa-miR-3661	28	0.002335225
hsa-miR-5587-5p	16	0.001334414
hsa-miR-615-3p	16	0.001334414
hsa-miR-326	12	0.001000811
hsa-miR-3911	6	0.000500405
hsa-miR-489	4	0.000333604
hsa-miR-3173-5p	3	0.000250203
hsa-miR-659-5p	3	0.000250203
hsa-miR-651	3	0.000250203

hsa-miR-3690	1	8.34009E-05
hsa-miR-570-3p	1	8.34009E-05
hsa-miR-3167	1	8.34009E-05
hsa-miR-142-3p	1	8.34009E-05
hsa-miR-3189-3p	0	0
hsa-miR-6724-5p	0	0
hsa-miR-345-3p	0	0
hsa-miR-4796-5p	0	0
hsa-miR-551a	0	0
hsa-miR-4767	0	0
hsa-miR-3164	0	0
hsa-miR-656	32	0.002668828
hsa-miR-376a-3p	24	0.002001621
hsa-miR-766-5p	16	0.001334414
hsa-miR-3120-3p	8	0.000667207
hsa-miR-4717-3p	3	0.000250203
hsa-miR-4504	2	0.000166802
hsa-miR-4436b-3p	2	0.000166802
hsa-miR-499a-5p	2	0.000166802
hsa-miR-369-5p	2	0.000166802
hsa-miR-377-3p	2	0.000166802
hsa-miR-1306-5p	1	8.34009E-05
hsa-miR-3684	1	8.34009E-05
hsa-miR-548ar-3p	0	0
hsa-miR-3134	0	0
hsa-miR-3619-5p	0	0
hsa-miR-4645-3p	0	0
hsa-miR-4324	0	0
hsa-miR-548u	0	0
hsa-miR-551b-3p	0	0
hsa-miR-1277-3p	0	0
hsa-miR-548a-3p	0	0
hsa-miR-4440	0	0
hsa-miR-597	0	0
hsa-miR-664a-5p	0	0
hsa-miR-4766-3p	0	0
hsa-miR-4473	0	0
hsa-miR-136-5p	0	0
hsa-miR-5010-3p	0	0
hsa-miR-15a-3p	0	0
hsa-miR-4461	0	0
hsa-miR-1293	0	0
hsa-miR-1224-5p	7	0.000583806
hsa-miR-4788	6	0.000500405
hsa-miR-3918	4	0.000333604
hsa-miR-660-3p	4	0.000333604
hsa-miR-4690-3p	3	0.000250203
hsa-miR-2276	3	0.000250203

hsa-miR-3116	3	0.000250203
hsa-miR-3688-3p	3	0.000250203
hsa-miR-3159	2	0.000166802
hsa-miR-2278	1	8.34009E-05
hsa-miR-1306-3p	1	8.34009E-05
hsa-miR-590-5p	1	8.34009E-05
hsa-miR-4725-3p	1	8.34009E-05
hsa-miR-767-5p	1	8.34009E-05
hsa-miR-181c-3p	0	0
hsa-miR-3682-3p	0	0
hsa-miR-4796-3p	0	0
hsa-miR-3194-5p	0	0
hsa-miR-3591-5p	0	0
hsa-miR-29c-5p	0	0
hsa-miR-154-3p	0	0
hsa-miR-944	0	0
hsa-miR-548av-5p	24	0.002001621
hsa-miR-4424	8	0.000667207
hsa-miR-23a-5p	7	0.000583806
hsa-miR-4638-3p	6	0.000500405
hsa-miR-363-3p	5	0.000417004
hsa-miR-4676-3p	5	0.000417004
hsa-miR-495-3p	5	0.000417004
hsa-miR-433	4	0.000333604
hsa-miR-2114-5p	3	0.000250203
hsa-miR-2682-5p	1	8.34009E-05
hsa-miR-5699	1	8.34009E-05
hsa-miR-153	1	8.34009E-05
hsa-miR-548ah-3p	1	8.34009E-05
hsa-miR-548s	0	0
hsa-miR-1179	0	0
hsa-miR-6511a-3p	0	0
hsa-miR-3155b	0	0
hsa-miR-548v	0	0
hsa-miR-449b-5p	0	0
hsa-miR-23b-5p	0	0
hsa-miR-6501-5p	0	0
hsa-miR-4454	0	0
hsa-miR-3130-5p	0	0
hsa-miR-676-3p	0	0
hsa-miR-4651	0	0
hsa-miR-2964a-3p	0	0
hsa-miR-6720-3p	0	0
hsa-miR-379-3p	0	0
hsa-miR-4532	33	0.002752229
hsa-miR-485-3p	21	0.001751419
hsa-miR-380-3p	15	0.001251013
hsa-miR-5010-5p	15	0.001251013

hsa-miR-450a-3p	13	0.001084212
hsa-miR-636	12	0.001000811
hsa-miR-891a	12	0.001000811
hsa-miR-3121-3p	8	0.000667207
hsa-miR-3935	7	0.000583806
hsa-miR-3074-5p	4	0.000333604
hsa-miR-204-5p	4	0.000333604
hsa-miR-4470	3	0.000250203
hsa-miR-3150a-5p	3	0.000250203
hsa-miR-3620-5p	3	0.000250203
hsa-miR-4728-3p	2	0.000166802
hsa-miR-4741	2	0.000166802
hsa-miR-4785	2	0.000166802
hsa-miR-127-5p	1	8.34009E-05
hsa-miR-197-5p	1	8.34009E-05
hsa-miR-6500-3p	1	8.34009E-05
hsa-miR-4665-5p	1	8.34009E-05
hsa-miR-4660	1	8.34009E-05
hsa-miR-5094	1	8.34009E-05
hsa-miR-655	1	8.34009E-05
hsa-miR-3150a-3p	1	8.34009E-05
hsa-miR-876-3p	1	8.34009E-05
hsa-miR-3622a-5p	0	0
hsa-miR-4443	0	0
hsa-miR-664b-5p	0	0
hsa-miR-4761-3p	0	0
hsa-miR-5585-3p	0	0
hsa-miR-1304-5p	0	0
hsa-miR-299-5p	0	0
hsa-miR-548p	0	0
hsa-miR-586	0	0
hsa-miR-3657	0	0
hsa-miR-5001-3p	0	0
hsa-miR-5697	0	0
hsa-miR-643	0	0
hsa-miR-501-5p	0	0
hsa-miR-5008-5p	0	0
hsa-miR-3664-5p	0	0
hsa-miR-548z	0	0
hsa-miR-485-5p	0	0
hsa-miR-1278	0	0
hsa-miR-4684-3p	0	0
hsa-miR-1272	0	0
hsa-miR-3144-5p	0	0
hsa-miR-5187-5p	0	0
hsa-miR-4492	73	0.006088265
hsa-miR-2114-3p	11	0.00091741
hsa-miR-144-5p	10	0.000834009

hsa-miR-6509-5p	9	0.000750608
hsa-miR-887	9	0.000750608
hsa-miR-3155a	5	0.000417004
hsa-miR-4425	5	0.000417004
hsa-miR-192-3p	3	0.000250203
hsa-miR-4689	3	0.000250203
hsa-miR-1343	3	0.000250203
hsa-miR-378e	2	0.000166802
hsa-miR-4659a-3p	2	0.000166802
hsa-miR-943	2	0.000166802
hsa-miR-664b-3p	2	0.000166802
hsa-miR-4479	2	0.000166802
hsa-miR-1228-3p	2	0.000166802
hsa-miR-4511	2	0.000166802
hsa-miR-376b-3p	2	0.000166802
hsa-miR-4758-3p	2	0.000166802
hsa-miR-1237-3p	2	0.000166802
hsa-miR-3064-5p	1	8.34009E-05
hsa-miR-4688	1	8.34009E-05
hsa-miR-1538	1	8.34009E-05
hsa-miR-3198	1	8.34009E-05
hsa-miR-3168	1	8.34009E-05
hsa-miR-4677-5p	1	8.34009E-05
hsa-miR-610	1	8.34009E-05
hsa-miR-139-3p	1	8.34009E-05
hsa-miR-4749-5p	1	8.34009E-05
hsa-miR-4467	1	8.34009E-05
hsa-miR-1250	1	8.34009E-05
hsa-miR-4485	1	8.34009E-05
hsa-miR-3163	0	0
hsa-miR-5583-5p	0	0
hsa-miR-146b-3p	0	0
hsa-miR-548ak	0	0
hsa-miR-3074-3p	0	0
hsa-miR-548ay-3p	0	0
hsa-miR-618	0	0
hsa-miR-3177-5p	0	0
hsa-miR-581	0	0
hsa-miR-4640-3p	0	0
hsa-miR-1255b-5p	0	0
hsa-miR-1234-3p	0	0
hsa-miR-19a-5p	0	0
hsa-miR-23c	0	0
hsa-miR-3157-3p	0	0
hsa-miR-3129-5p	0	0
hsa-miR-935	0	0
hsa-miR-6721-5p	0	0
hsa-miR-3939	0	0

hsa-miR-3128	0	0
hsa-miR-5580-5p	0	0
hsa-miR-548t-5p	0	0
hsa-miR-3936	0	0
hsa-miR-548au-3p	0	0
hsa-miR-3942-3p	0	0
hsa-miR-570-5p	0	0
hsa-miR-3130-3p	31	0.002585428
hsa-miR-5096	24	0.002001621
hsa-miR-3656	11	0.00091741
hsa-miR-4421	8	0.000667207
hsa-miR-4667-5p	7	0.000583806
hsa-miR-3940-3p	7	0.000583806
hsa-miR-3124-5p	6	0.000500405
hsa-miR-4746-3p	6	0.000500405
hsa-miR-3183	6	0.000500405
hsa-miR-148a-5p	5	0.000417004
hsa-miR-665	4	0.000333604
hsa-miR-4515	3	0.000250203
hsa-miR-4758-5p	3	0.000250203
hsa-miR-184	3	0.000250203
hsa-miR-3960	3	0.000250203
hsa-miR-1271-5p	3	0.000250203
hsa-miR-135a-5p	3	0.000250203
hsa-miR-615-5p	2	0.000166802
hsa-miR-3140-3p	2	0.000166802
hsa-miR-3687	2	0.000166802
hsa-miR-3944-5p	2	0.000166802
hsa-miR-877-3p	2	0.000166802
hsa-miR-3127-5p	1	8.34009E-05
hsa-miR-675-3p	1	8.34009E-05
hsa-miR-217	1	8.34009E-05
hsa-miR-1288	1	8.34009E-05
hsa-miR-4672	1	8.34009E-05
hsa-miR-1252	1	8.34009E-05
hsa-miR-34b-3p	1	8.34009E-05
hsa-miR-616-3p	1	8.34009E-05
hsa-miR-4683	1	8.34009E-05
hsa-miR-3181	1	8.34009E-05
hsa-miR-3160-3p	1	8.34009E-05
hsa-miR-4661-3p	1	8.34009E-05
hsa-miR-1227-3p	1	8.34009E-05
hsa-miR-34b-5p	0	0
hsa-miR-517a-3p	0	0
hsa-miR-3680-5p	0	0
hsa-miR-4474-3p	0	0
hsa-miR-3691-5p	0	0
hsa-miR-4727-3p	0	0

hsa-miR-3146	0	0
hsa-miR-3605-3p	0	0
hsa-miR-4797-3p	0	0
hsa-miR-548a-5p	0	0
hsa-miR-5587-3p	0	0
hsa-miR-4669	0	0
hsa-miR-3620-3p	0	0
hsa-miR-3917	0	0
hsa-miR-4793-3p	0	0
hsa-miR-4765	0	0
hsa-miR-3190-3p	0	0
hsa-miR-186-3p	0	0
hsa-miR-6505-5p	0	0
hsa-miR-3934-3p	0	0
hsa-miR-3677-5p	0	0
hsa-miR-4751	0	0
hsa-miR-675-5p	0	0
hsa-miR-624-5p	0	0
hsa-miR-3145-5p	0	0
hsa-miR-6513-5p	0	0
hsa-miR-337-3p	0	0
hsa-miR-598	0	0
hsa-miR-3153	0	0
hsa-miR-329	0	0
hsa-miR-4483	0	0
hsa-miR-4706	0	0
hsa-miR-3148	0	0
hsa-miR-4762-3p	0	0
hsa-miR-4457	0	0
hsa-miR-3145-3p	0	0
hsa-miR-1273c	0	0
hsa-miR-4784	0	0
hsa-miR-548f	0	0
hsa-miR-149-3p	0	0
hsa-miR-124-3p	0	0
hsa-miR-4519	0	0
hsa-miR-579	0	0
hsa-miR-1185-5p	0	0
hsa-miR-1273d	0	0
hsa-miR-4999-5p	0	0
hsa-miR-1233-1-5p	0	0
hsa-miR-3157-5p	0	0
hsa-miR-135b-5p	0	0
hsa-miR-512-3p	0	0
hsa-miR-3187-5p	0	0
hsa-miR-224-3p	0	0
hsa-miR-6508-3p	0	0
hsa-miR-4642	0	0

hsa-miR-4781-3p	0	0
hsa-miR-5687	0	0
hsa-miR-1257	0	0
hsa-miR-548q	0	0
hsa-miR-548ap-3p	0	0
hsa-miR-4742-5p	0	0
hsa-miR-3192	0	0
hsa-miR-1256	0	0
hsa-miR-708-5p	0	0
hsa-miR-2467-3p	0	0
hsa-miR-3914	0	0
hsa-miR-548ax	0	0
hsa-miR-4442	0	0
hsa-miR-2964a-5p	0	0
hsa-miR-548c-3p	0	0
hsa-miR-5583-3p	0	0
hsa-miR-3188	0	0
hsa-miR-3136-5p	0	0
hsa-miR-5689	0	0
hsa-miR-4783-3p	0	0
hsa-miR-376a-5p	0	0
hsa-miR-3120-5p	0	0
hsa-miR-1537	0	0
hsa-miR-548aq-3p	0	0
hsa-miR-4738-3p	0	0
hsa-miR-4787-3p	0	0
hsa-miR-544a	0	0
hsa-miR-548ac	0	0
hsa-miR-1226-5p	0	0
hsa-miR-216a-5p	0	0
hsa-miR-346	0	0
hsa-miR-4786-5p	0	0
hsa-miR-1185-1-3p	0	0
hsa-miR-5090	0	0
hsa-miR-4687-5p	0	0
hsa-miR-451a	60	0.005004053
hsa-miR-1225-5p	39	0.003252635
hsa-miR-3663-3p	23	0.00191822
hsa-miR-888-5p	19	0.001584617
hsa-miR-1290	18	0.001501216
hsa-miR-4664-5p	18	0.001501216
hsa-miR-4449	17	0.001417815
hsa-miR-144-3p	17	0.001417815
hsa-miR-496	11	0.00091741
hsa-miR-3679-5p	11	0.00091741
hsa-miR-4687-3p	10	0.000834009
hsa-miR-320e	8	0.000667207
hsa-miR-3131	8	0.000667207

hsa-miR-4634	7	0.000583806
hsa-miR-371a-5p	7	0.000583806
hsa-miR-337-5p	6	0.000500405
hsa-miR-3122	5	0.000417004
hsa-miR-3173-3p	5	0.000417004
hsa-miR-483-5p	5	0.000417004
hsa-miR-431-3p	4	0.000333604
hsa-miR-3648	4	0.000333604
hsa-miR-223-3p	3	0.000250203
hsa-miR-204-3p	3	0.000250203
hsa-miR-1291	3	0.000250203
hsa-miR-1292-3p	3	0.000250203
hsa-miR-1228-5p	3	0.000250203
hsa-miR-3663-5p	3	0.000250203
hsa-miR-323a-5p	3	0.000250203
hsa-miR-6515-5p	2	0.000166802
hsa-miR-3180-3p	2	0.000166802
hsa-miR-4739	2	0.000166802
hsa-miR-1185-2-3p	2	0.000166802
hsa-miR-4721	2	0.000166802
hsa-miR-378i	2	0.000166802
hsa-miR-541-3p	2	0.000166802
hsa-miR-4659a-5p	2	0.000166802
hsa-miR-4700-3p	2	0.000166802
hsa-miR-4755-3p	2	0.000166802
hsa-miR-3165	2	0.000166802
hsa-miR-1236-5p	2	0.000166802
hsa-miR-214-3p	2	0.000166802
hsa-miR-6087	2	0.000166802
hsa-miR-155-5p	2	0.000166802
hsa-miR-4743-3p	1	8.34009E-05
hsa-miR-509-3-5p	1	8.34009E-05
hsa-miR-4674	1	8.34009E-05
hsa-miR-3200-5p	1	8.34009E-05
hsa-miR-541-5p	1	8.34009E-05
hsa-miR-663a	1	8.34009E-05
hsa-miR-483-3p	1	8.34009E-05
hsa-miR-1289	1	8.34009E-05
hsa-miR-4638-5p	1	8.34009E-05
hsa-miR-4525	1	8.34009E-05
hsa-miR-5001-5p	1	8.34009E-05
hsa-miR-4710	1	8.34009E-05
hsa-miR-3925-5p	1	8.34009E-05
hsa-miR-1273f	1	8.34009E-05
hsa-miR-1295a	1	8.34009E-05
hsa-miR-3685	1	8.34009E-05
hsa-miR-549a	1	8.34009E-05
hsa-miR-5006-5p	1	8.34009E-05

hsa-miR-3135a	1	8.34009E-05
hsa-miR-1	1	8.34009E-05
hsa-miR-4757-3p	1	8.34009E-05
hsa-miR-3655	1	8.34009E-05
hsa-miR-4753-5p	1	8.34009E-05
hsa-miR-3162-5p	1	8.34009E-05
hsa-miR-4646-5p	1	8.34009E-05
hsa-miR-4734	1	8.34009E-05
hsa-miR-516b-5p	1	8.34009E-05
hsa-miR-383	1	8.34009E-05
hsa-miR-4670-3p	1	8.34009E-05
hsa-miR-150-5p	1	8.34009E-05
hsa-miR-1227-5p	1	8.34009E-05
hsa-miR-3133	1	8.34009E-05
hsa-miR-199b-5p	1	8.34009E-05
hsa-miR-3929	1	8.34009E-05
hsa-miR-4745-5p	1	8.34009E-05
hsa-miR-4700-5p	1	8.34009E-05
hsa-miR-505-5p	1	8.34009E-05
hsa-miR-4725-5p	1	8.34009E-05
hsa-miR-187-3p	1	8.34009E-05
hsa-miR-4770	1	8.34009E-05
hsa-miR-632	1	8.34009E-05
hsa-miR-4507	1	8.34009E-05
hsa-miR-1244	1	8.34009E-05
hsa-miR-933	1	8.34009E-05
hsa-miR-1298	1	8.34009E-05
hsa-miR-211-5p	1	8.34009E-05
hsa-miR-4695-5p	1	8.34009E-05
hsa-miR-296-3p	1	8.34009E-05
hsa-miR-145-5p	1	8.34009E-05
hsa-miR-1285-5p	1	8.34009E-05
hsa-miR-3919	1	8.34009E-05
