# Supplementary material for: The exosomal miRNA/FOXN3 axis empowers fibroblasts with tumor-promoting functions via enhanced migration and altered secretome
Source: J Nanobiotechnology. 2026 Jun 5;24:742. doi: 10.1186/s12951-026-04633-6 (PMC13459620; doi:10.1186/s12951-026-04633-6)
Supplement: Supplementary file 3 [file 12951_2026_4633_MOESM4_ESM.pdf]

Table S3 miRNA profiles of A549 cells

Gene symbol	Raw count	Normalized raw count
hsa-miR-22-3p	130257	1.742974984
hsa-miR-24-3p	82593	1.10518078
hsa-miR-21-5p	3362473	44.99340783
hsa-miR-21-3p	304775	4.078208471
hsa-miR-320a	28255	0.378081471
hsa-miR-27b-3p	657593	8.799282562
hsa-miR-1246	71	0.000950054
hsa-miR-320c	2360	0.03157927
hsa-miR-92b-3p	8891	0.118970885
hsa-miR-92a-3p	104693	1.400901909
hsa-miR-192-5p	23047	0.30839298
hsa-miR-486-5p	9308	0.124550782
hsa-miR-100-5p	149123	1.995421809
hsa-miR-423-5p	1600	0.021409675
hsa-miR-378a-3p	17092	0.228708848
hsa-miR-1307-5p	11274	0.150857919
hsa-miR-320d	251	0.003358643
hsa-miR-27a-3p	31673	0.423817888
hsa-miR-423-3p	6286	0.084113259
hsa-let-7i-5p	597998	8.001839091
hsa-miR-29a-3p	16503	0.220827412
hsa-miR-10a-5p	113351	1.516755011
hsa-miR-99b-5p	42461	0.568172619
hsa-miR-30a-5p	338827	4.533859869
hsa-miR-191-5p	36933	0.494202193
hsa-miR-345-5p	2844	0.038055696
hsa-miR-140-3p	5958	0.079724276
hsa-miR-130a-3p	4197	0.056160252
hsa-miR-16-5p	84560	1.131501299
hsa-miR-193b-3p	1187	0.015883302
hsa-miR-146a-5p	1703	0.022787922
hsa-let-7a-5p	140057	1.87410924
hsa-let-7f-5p	129324	1.730490468
hsa-miR-138-5p	2413	0.032288465
hsa-miR-182-5p	46262	0.619033977
hsa-miR-4516	4	5.35242E-05
hsa-miR-25-3p	21573	0.288669318
hsa-miR-28-3p	10018	0.134051325
hsa-miR-589-5p	888	0.011882369
hsa-miR-183-5p	18045	0.241460985
hsa-let-7b-5p	73725	0.986517659
hsa-miR-221-3p	34157	0.457056408
hsa-miR-484	1321	0.017676363
hsa-miR-30d-5p	52278	0.699534353
hsa-miR-222-3p	22064	0.295239412
hsa-miR-130b-3p	1682	0.02250692

hsa-miR-138-1-3p	187	0.002502256
hsa-miR-31-5p	105137	1.406843094
hsa-miR-26a-5p	72136	0.965255176
hsa-miR-3615	540	0.007225765
hsa-miR-629-5p	643	0.008604013
hsa-miR-23a-3p	1535	0.020539906
hsa-miR-128	845	0.011306984
hsa-miR-181a-5p	34322	0.459264281
hsa-miR-744-5p	1735	0.023216116
hsa-miR-339-3p	839	0.011226698
hsa-miR-501-3p	604	0.008082152
hsa-miR-19b-3p	25569	0.34213998
hsa-miR-30e-5p	31494	0.421422681
hsa-let-7g-5p	35185	0.470812124
hsa-miR-151a-3p	23427	0.313477778
hsa-miR-224-5p	1140	0.015254393
hsa-miR-151a-5p	13251	0.177312248
hsa-miR-186-5p	7927	0.106071556
hsa-miR-106b-5p	4548	0.060857
hsa-miR-26b-5p	35124	0.46999588
hsa-miR-151b	241	0.003224832
hsa-miR-93-5p	22635	0.302879989
hsa-miR-152	5211	0.069728634
hsa-miR-323a-3p	62	0.000829625
hsa-miR-425-5p	2738	0.036637306
hsa-miR-200a-3p	5075	0.067908811
hsa-miR-15a-5p	2030	0.027163525
hsa-miR-1910	48	0.00064229
hsa-miR-4306	146	0.001953633
hsa-miR-502-3p	182	0.00243535
hsa-miR-1307-3p	4132	0.055290484
hsa-miR-30a-3p	5443	0.072833037
hsa-miR-181b-5p	8615	0.115277716
hsa-miR-4488	0	0
hsa-miR-17-5p	22519	0.301327788
hsa-miR-103a-3p	20203	0.270337284
hsa-miR-4466	26	0.000347907
hsa-miR-877-5p	34	0.000454956
hsa-miR-342-3p	378	0.005058036
hsa-miR-3176	292	0.003907266
hsa-miR-210	973	0.013019758
hsa-miR-320b	153	0.0020473
hsa-let-7a-3p	2402	0.032141274
hsa-miR-301a-3p	2532	0.03388081
hsa-miR-29c-3p	803	0.01074498
hsa-miR-671-3p	302	0.004041076
hsa-miR-125a-3p	569	0.007613816
hsa-miR-148b-3p	3802	0.050874739

hsa-let-7e-5p	10289	0.137677588
hsa-miR-134	56	0.000749339
hsa-miR-4508	0	0
hsa-miR-132-3p	2896	0.038751511
hsa-miR-654-3p	63	0.000843006
hsa-miR-10a-3p	191	0.00255578
hsa-miR-1247-5p	29	0.00038805
hsa-miR-126-5p	3388	0.045334986
hsa-miR-101-3p	4225	0.056534922
hsa-miR-369-3p	74	0.000990197
hsa-miR-27b-5p	10623	0.142146858
hsa-miR-33b-5p	2059	0.027551575
hsa-miR-486-3p	172	0.00230154
hsa-miR-3187-3p	42	0.000562004
hsa-miR-3677-3p	131	0.001752917
hsa-miR-500a-3p	402	0.005379181
hsa-miR-20a-5p	11618	0.155460999
hsa-miR-3180	19	0.00025424
hsa-miR-361-5p	328	0.004388983
hsa-miR-19a-3p	4959	0.06635661
hsa-miR-323b-3p	13	0.000173954
hsa-miR-335-5p	657	0.008791348
hsa-miR-424-3p	1523	0.020379334
hsa-miR-381-3p	460	0.006155281
hsa-miR-410	88	0.001177532
hsa-miR-122-5p	1	1.3381E-05
hsa-miR-143-3p	31	0.000414812
hsa-let-7d-5p	4606	0.061633101
hsa-miR-7-5p	5905	0.07901508
hsa-miR-4497	6	8.02863E-05
hsa-miR-30c-5p	25542	0.341778692
hsa-miR-1268a	1413	0.018907419
hsa-miR-98-5p	9389	0.125634646
hsa-let-7d-3p	665	0.008898396
hsa-miR-450b-5p	297	0.003974171
hsa-miR-1287	142	0.001900109
hsa-miR-452-5p	530	0.007091955
hsa-miR-941	1683	0.022520301
hsa-miR-99b-3p	489	0.006543332
hsa-miR-185-5p	1077	0.014411387
hsa-miR-200b-3p	5472	0.073221087
hsa-miR-212-3p	71	0.000950054
hsa-miR-1180	759	0.010156214
hsa-miR-23b-3p	3817	0.051075455
hsa-miR-92b-5p	202	0.002702971
hsa-miR-3195	13	0.000173954
hsa-miR-30b-5p	3982	0.053283327
hsa-miR-769-5p	3173	0.042458061

hsa-miR-378d	53	0.000709195
hsa-miR-22-5p	61	0.000816244
hsa-miR-197-3p	130	0.001739536
hsa-miR-760	35	0.000468337
hsa-miR-34a-5p	4747	0.063519828
hsa-miR-127-3p	995	0.013314141
hsa-miR-125a-5p	6363	0.085143599
hsa-miR-196a-5p	3837	0.051343076
hsa-miR-125b-5p	3453	0.046204754
hsa-miR-454-3p	3425	0.045830085
hsa-miR-181c-5p	3425	0.045830085
hsa-miR-29b-3p	2626	0.035138628
hsa-miR-196b-5p	2446	0.03273004
hsa-let-7c	2325	0.031110933
hsa-miR-9-5p	2313	0.030950361
hsa-miR-374a-5p	2245	0.03004045
hsa-miR-221-5p	2186	0.029250968
hsa-miR-181a-3p	2111	0.028247389
hsa-miR-374a-3p	2099	0.028086817
hsa-miR-4286	2096	0.028046674
hsa-miR-27a-5p	1948	0.026066279
hsa-miR-582-3p	1920	0.025691609
hsa-miR-340-5p	1807	0.024179551
hsa-miR-146b-5p	1795	0.024018979
hsa-miR-28-5p	1767	0.023644309
hsa-miR-374b-5p	1661	0.022225918
hsa-miR-92a-1-5p	1600	0.021409675
hsa-miR-107	1538	0.02058005
hsa-miR-181a-2-3p	1457	0.019496185
hsa-miR-532-5p	1441	0.019282088
hsa-miR-106b-3p	1384	0.018519368
hsa-miR-181d	1374	0.018385558
hsa-miR-335-3p	1338	0.01790384
hsa-miR-1275	1253	0.016766451
hsa-miR-100-3p	1211	0.016204447
hsa-miR-582-5p	1210	0.016191066
hsa-miR-660-5p	1194	0.01597697
hsa-let-7f-2-3p	1147	0.01534806
hsa-miR-361-3p	1130	0.015120583
hsa-miR-125b-1-3p	1122	0.015013534
hsa-miR-30e-3p	1022	0.01367543
hsa-miR-30c-2-3p	1015	0.013581762
hsa-miR-421	942	0.012604946
hsa-miR-17-3p	929	0.012430992
hsa-miR-18a-5p	917	0.01227042
hsa-miR-15b-3p	817	0.010932315
hsa-miR-194-5p	780	0.010437216
hsa-miR-140-5p	712	0.009527305

hsa-miR-429	710	0.009500543
hsa-miR-324-5p	694	0.009286446
hsa-miR-96-5p	683	0.009139255
hsa-miR-671-5p	612	0.008189201
hsa-let-7i-3p	605	0.008095533
hsa-miR-15b-5p	591	0.007908199
hsa-miR-339-5p	538	0.007199003
hsa-miR-424-5p	537	0.007185622
hsa-miR-561-5p	520	0.006958144
hsa-miR-1299	487	0.00651657
hsa-miR-590-3p	480	0.006422902
hsa-miR-34c-5p	467	0.006248949
hsa-miR-25-5p	462	0.006182044
hsa-miR-548k	453	0.006061614
hsa-miR-455-5p	452	0.006048233
hsa-miR-218-5p	426	0.005700326
hsa-miR-542-3p	378	0.005058036
hsa-miR-126-3p	377	0.005044655
hsa-miR-99a-5p	376	0.005031274
hsa-miR-330-5p	351	0.004696747
hsa-miR-30d-3p	351	0.004696747
hsa-miR-625-5p	343	0.004589699
hsa-miR-331-3p	315	0.00421503
hsa-miR-503-5p	295	0.003947409
hsa-miR-2467-5p	294	0.003934028
hsa-miR-1260b	294	0.003934028
hsa-miR-454-5p	254	0.003398786
hsa-miR-32-3p	254	0.003398786
hsa-miR-16-2-3p	243	0.003251594
hsa-miR-130b-5p	236	0.003157927
hsa-miR-200a-5p	230	0.003077641
hsa-miR-301b	223	0.002983973
hsa-miR-33a-5p	217	0.002903687
hsa-miR-141-3p	211	0.002823401
hsa-miR-4521	211	0.002823401
hsa-miR-550a-3p	207	0.002769877
hsa-miR-652-3p	207	0.002769877
hsa-miR-31-3p	207	0.002769877
hsa-miR-147b	202	0.002702971
hsa-miR-195-5p	195	0.002609304
hsa-miR-548ay-5p	194	0.002595923
hsa-miR-374b-3p	192	0.002569161
hsa-miR-769-3p	190	0.002542399
hsa-miR-365a-3p	188	0.002515637
hsa-miR-32-5p	182	0.00243535
hsa-miR-450a-5p	173	0.002314921
hsa-miR-193a-3p	170	0.002274778
hsa-miR-148a-3p	169	0.002261397

hsa-miR-1285-3p	166	0.002221254
hsa-miR-641	165	0.002207873
hsa-miR-33b-3p	165	0.002207873
hsa-miR-4677-3p	162	0.00216773
hsa-miR-1908	150	0.002007157
hsa-miR-379-5p	149	0.001993776
hsa-miR-409-3p	148	0.001980395
hsa-miR-1296	148	0.001980395
hsa-miR-493-5p	132	0.001766298
hsa-miR-550a-5p	130	0.001739536
hsa-miR-132-5p	130	0.001739536
hsa-let-7b-3p	127	0.001699393
hsa-miR-193b-5p	126	0.001686012
hsa-miR-18a-3p	123	0.001645869
hsa-miR-222-5p	120	0.001605726
hsa-miR-7-1-3p	117	0.001565582
hsa-miR-30b-3p	116	0.001552201
hsa-miR-3158-3p	115	0.00153882
hsa-miR-193a-5p	112	0.001498677
hsa-miR-576-3p	102	0.001364867
hsa-let-7f-1-3p	100	0.001338105
hsa-miR-628-5p	100	0.001338105
hsa-let-7e-3p	98	0.001311343
hsa-miR-4326	96	0.00128458
hsa-miR-149-5p	93	0.001244437
hsa-miR-29b-1-5p	93	0.001244437
hsa-miR-455-3p	92	0.001231056
hsa-miR-148b-5p	91	0.001217675
hsa-miR-190a	86	0.00115077
hsa-miR-200b-5p	81	0.001083865
hsa-let-7g-3p	81	0.001083865
hsa-miR-378a-5p	81	0.001083865
hsa-miR-4500	79	0.001057103
hsa-miR-324-3p	77	0.001030341
hsa-miR-9-3p	76	0.00101696
hsa-miR-29a-5p	75	0.001003578
hsa-miR-1294	75	0.001003578
hsa-miR-2277-5p	73	0.000976816
hsa-miR-95	72	0.000963435
hsa-miR-375	70	0.000936673
hsa-miR-136-3p	68	0.000909911
hsa-miR-328	67	0.00089653
hsa-miR-550a-3-5p	67	0.00089653
hsa-miR-93-3p	65	0.000869768
hsa-miR-411-5p	65	0.000869768
hsa-miR-340-3p	64	0.000856387
hsa-miR-4775	63	0.000843006
hsa-miR-3182	62	0.000829625

hsa-miR-889	62	0.000829625
hsa-miR-1268b	62	0.000829625
hsa-miR-542-5p	60	0.000802863
hsa-miR-2355-5p	59	0.000789482
hsa-miR-10b-5p	57	0.00076272
hsa-miR-3934-5p	57	0.00076272
hsa-miR-200c-3p	56	0.000749339
hsa-miR-30c-1-3p	55	0.000735958
hsa-miR-574-3p	54	0.000722577
hsa-miR-365a-5p	54	0.000722577
hsa-miR-585	54	0.000722577
hsa-miR-331-5p	53	0.000709195
hsa-miR-497-5p	53	0.000709195
hsa-miR-98-3p	50	0.000669052
hsa-miR-137	50	0.000669052
hsa-miR-362-3p	49	0.000655671
hsa-miR-4671-3p	49	0.000655671
hsa-miR-330-3p	47	0.000628909
hsa-miR-1269b	46	0.000615528
hsa-miR-181b-3p	45	0.000602147
hsa-miR-940	44	0.000588766
hsa-miR-548l	44	0.000588766
hsa-miR-1303	43	0.000575385
hsa-miR-185-3p	43	0.000575385
hsa-miR-129-5p	41	0.000548623
hsa-miR-589-3p	40	0.000535242
hsa-miR-873-5p	39	0.000521861
hsa-miR-616-5p	39	0.000521861
hsa-miR-26b-3p	39	0.000521861
hsa-miR-194-3p	39	0.000521861
hsa-miR-382-5p	38	0.00050848
hsa-miR-548e	38	0.00050848
hsa-miR-625-3p	37	0.000495099
hsa-miR-188-5p	37	0.000495099
hsa-miR-4746-5p	37	0.000495099
hsa-miR-548o-3p	37	0.000495099
hsa-miR-493-3p	35	0.000468337
hsa-miR-370	34	0.000454956
hsa-miR-99a-3p	33	0.000441575
hsa-miR-505-3p	32	0.000428193
hsa-miR-299-3p	32	0.000428193
hsa-miR-1305	31	0.000414812
hsa-miR-142-5p	31	0.000414812
hsa-miR-874	30	0.000401431
hsa-miR-1297	30	0.000401431
hsa-miR-1468	28	0.000374669
hsa-miR-432-5p	27	0.000361288
hsa-miR-24-2-5p	27	0.000361288

hsa-miR-449a	27	0.000361288
hsa-miR-20a-3p	27	0.000361288
hsa-miR-378b	26	0.000347907
hsa-miR-2110	26	0.000347907
hsa-miR-378f	26	0.000347907
hsa-miR-219-5p	26	0.000347907
hsa-miR-16-1-3p	26	0.000347907
hsa-miR-876-5p	25	0.000334526
hsa-miR-937-3p	25	0.000334526
hsa-miR-548n	25	0.000334526
hsa-miR-376c-3p	24	0.000321145
hsa-miR-3909	24	0.000321145
hsa-miR-576-5p	24	0.000321145
hsa-miR-573	24	0.000321145
hsa-miR-548au-5p	24	0.000321145
hsa-miR-190b	24	0.000321145
hsa-miR-342-5p	24	0.000321145
hsa-miR-362-5p	24	0.000321145
hsa-miR-642a-5p	24	0.000321145
hsa-miR-487b	23	0.000307764
hsa-miR-584-5p	23	0.000307764
hsa-miR-425-3p	23	0.000307764
hsa-miR-1286	23	0.000307764
hsa-miR-1254	23	0.000307764
hsa-miR-1266	22	0.000294383
hsa-miR-548c-5p	22	0.000294383
hsa-miR-106a-5p	22	0.000294383
hsa-miR-4517	22	0.000294383
hsa-miR-2277-3p	21	0.000281002
hsa-miR-3065-3p	21	0.000281002
hsa-miR-4768-5p	21	0.000281002
hsa-miR-212-5p	21	0.000281002
hsa-miR-2116-5p	20	0.000267621
hsa-miR-543	20	0.000267621
hsa-miR-154-5p	20	0.000267621
hsa-miR-219-1-3p	20	0.000267621
hsa-miR-5000-3p	20	0.000267621
hsa-miR-141-5p	20	0.000267621
hsa-miR-431-5p	19	0.00025424
hsa-miR-196b-3p	19	0.00025424
hsa-miR-3144-3p	19	0.00025424
hsa-miR-26a-2-3p	19	0.00025424
hsa-miR-3940-5p	18	0.000240859
hsa-miR-642a-3p	18	0.000240859
hsa-miR-532-3p	18	0.000240859
hsa-miR-2116-3p	17	0.000227478
hsa-miR-203a	17	0.000227478
hsa-miR-3614-5p	17	0.000227478

hsa-miR-382-3p	16	0.000214097
hsa-miR-24-1-5p	16	0.000214097
hsa-miR-503-3p	16	0.000214097
hsa-miR-19b-1-5p	16	0.000214097
hsa-miR-34c-3p	15	0.000200716
hsa-miR-205-5p	15	0.000200716
hsa-miR-1301	15	0.000200716
hsa-miR-3912	15	0.000200716
hsa-miR-301a-5p	15	0.000200716
hsa-miR-34a-3p	15	0.000200716
hsa-miR-548j	15	0.000200716
hsa-miR-4484	15	0.000200716
hsa-miR-873-3p	14	0.000187335
hsa-miR-3664-3p	14	0.000187335
hsa-miR-3928	14	0.000187335
hsa-miR-1276	14	0.000187335
hsa-miR-937-5p	14	0.000187335
hsa-miR-4661-5p	14	0.000187335
hsa-miR-188-3p	14	0.000187335
hsa-miR-3913-5p	14	0.000187335
hsa-miR-494	13	0.000173954
hsa-miR-4448	13	0.000173954
hsa-miR-338-3p	13	0.000173954
hsa-miR-3065-5p	13	0.000173954
hsa-miR-942	13	0.000173954
hsa-miR-26a-1-3p	13	0.000173954
hsa-miR-411-3p	13	0.000173954
hsa-miR-409-5p	13	0.000173954
hsa-miR-664a-3p	13	0.000173954
hsa-miR-545-5p	13	0.000173954
hsa-miR-378g	12	0.000160573
hsa-miR-4664-3p	12	0.000160573
hsa-miR-3174	12	0.000160573
hsa-miR-199b-3p	12	0.000160573
hsa-miR-139-5p	12	0.000160573
hsa-miR-29b-2-5p	12	0.000160573
hsa-miR-125b-2-3p	12	0.000160573
hsa-miR-3141	12	0.000160573
hsa-miR-215	11	0.000147192
hsa-miR-3605-5p	11	0.000147192
hsa-miR-628-3p	11	0.000147192
hsa-miR-3944-3p	11	0.000147192
hsa-miR-195-3p	11	0.000147192
hsa-miR-1262	11	0.000147192
hsa-miR-3662	11	0.000147192
hsa-miR-580	11	0.000147192
hsa-miR-33a-3p	11	0.000147192
hsa-miR-4435	10	0.00013381

hsa-miR-627	10	0.00013381
hsa-miR-744-3p	10	0.00013381
hsa-miR-377-5p	10	0.00013381
hsa-let-7a-2-3p	10	0.00013381
hsa-miR-939-5p	10	0.00013381
hsa-miR-103a-2-5p	10	0.00013381
hsa-miR-548d-5p	10	0.00013381
hsa-miR-545-3p	10	0.00013381
hsa-miR-4791	10	0.00013381
hsa-miR-3177-3p	9	0.000120429
hsa-miR-182-3p	9	0.000120429
hsa-miR-766-3p	9	0.000120429
hsa-miR-4802-3p	9	0.000120429
hsa-miR-3200-3p	9	0.000120429
hsa-miR-3676-3p	9	0.000120429
hsa-miR-500a-5p	9	0.000120429
hsa-miR-491-5p	9	0.000120429
hsa-miR-4523	9	0.000120429
hsa-miR-487a	8	0.000107048
hsa-miR-3126-5p	8	0.000107048
hsa-miR-539-3p	8	0.000107048
hsa-miR-4762-5p	8	0.000107048
hsa-miR-3676-5p	8	0.000107048
hsa-miR-1292-5p	8	0.000107048
hsa-miR-1197	8	0.000107048
hsa-miR-3611	8	0.000107048
hsa-miR-561-3p	8	0.000107048
hsa-miR-101-5p	8	0.000107048
hsa-miR-191-3p	8	0.000107048
hsa-miR-4714-3p	8	0.000107048
hsa-miR-365b-5p	8	0.000107048
hsa-miR-758-3p	8	0.000107048
hsa-miR-4671-5p	8	0.000107048
hsa-miR-548al	8	0.000107048
hsa-miR-4802-5p	8	0.000107048
hsa-miR-654-5p	8	0.000107048
hsa-miR-3129-3p	8	0.000107048
hsa-miR-3115	8	0.000107048
hsa-miR-1915-3p	7	9.36673E-05
hsa-miR-3661	7	9.36673E-05
hsa-miR-5587-5p	7	9.36673E-05
hsa-miR-615-3p	7	9.36673E-05
hsa-miR-326	7	9.36673E-05
hsa-miR-3911	7	9.36673E-05
hsa-miR-489	7	9.36673E-05
hsa-miR-3173-5p	7	9.36673E-05
hsa-miR-659-5p	7	9.36673E-05
hsa-miR-651	7	9.36673E-05

hsa-miR-3690	7	9.36673E-05
hsa-miR-570-3p	7	9.36673E-05
hsa-miR-3167	7	9.36673E-05
hsa-miR-142-3p	7	9.36673E-05
hsa-miR-3189-3p	7	9.36673E-05
hsa-miR-6724-5p	7	9.36673E-05
hsa-miR-345-3p	7	9.36673E-05
hsa-miR-4796-5p	7	9.36673E-05
hsa-miR-551a	7	9.36673E-05
hsa-miR-4767	7	9.36673E-05
hsa-miR-3164	7	9.36673E-05
hsa-miR-656	6	8.02863E-05
hsa-miR-376a-3p	6	8.02863E-05
hsa-miR-766-5p	6	8.02863E-05
hsa-miR-3120-3p	6	8.02863E-05
hsa-miR-4717-3p	6	8.02863E-05
hsa-miR-4504	6	8.02863E-05
hsa-miR-4436b-3p	6	8.02863E-05
hsa-miR-499a-5p	6	8.02863E-05
hsa-miR-369-5p	6	8.02863E-05
hsa-miR-377-3p	6	8.02863E-05
hsa-miR-1306-5p	6	8.02863E-05
hsa-miR-3684	6	8.02863E-05
hsa-miR-548ar-3p	6	8.02863E-05
hsa-miR-3134	6	8.02863E-05
hsa-miR-3619-5p	6	8.02863E-05
hsa-miR-4645-3p	6	8.02863E-05
hsa-miR-4324	6	8.02863E-05
hsa-miR-548u	6	8.02863E-05
hsa-miR-551b-3p	6	8.02863E-05
hsa-miR-1277-3p	6	8.02863E-05
hsa-miR-548a-3p	6	8.02863E-05
hsa-miR-4440	6	8.02863E-05
hsa-miR-597	6	8.02863E-05
hsa-miR-664a-5p	6	8.02863E-05
hsa-miR-4766-3p	6	8.02863E-05
hsa-miR-4473	6	8.02863E-05
hsa-miR-136-5p	6	8.02863E-05
hsa-miR-5010-3p	6	8.02863E-05
hsa-miR-15a-3p	6	8.02863E-05
hsa-miR-4461	6	8.02863E-05
hsa-miR-1293	6	8.02863E-05
hsa-miR-1224-5p	5	6.69052E-05
hsa-miR-4788	5	6.69052E-05
hsa-miR-3918	5	6.69052E-05
hsa-miR-660-3p	5	6.69052E-05
hsa-miR-4690-3p	5	6.69052E-05
hsa-miR-2276	5	6.69052E-05

hsa-miR-3116	5	6.69052E-05
hsa-miR-3688-3p	5	6.69052E-05
hsa-miR-3159	5	6.69052E-05
hsa-miR-2278	5	6.69052E-05
hsa-miR-1306-3p	5	6.69052E-05
hsa-miR-590-5p	5	6.69052E-05
hsa-miR-4725-3p	5	6.69052E-05
hsa-miR-767-5p	5	6.69052E-05
hsa-miR-181c-3p	5	6.69052E-05
hsa-miR-3682-3p	5	6.69052E-05
hsa-miR-4796-3p	5	6.69052E-05
hsa-miR-3194-5p	5	6.69052E-05
hsa-miR-3591-5p	5	6.69052E-05
hsa-miR-29c-5p	5	6.69052E-05
hsa-miR-154-3p	5	6.69052E-05
hsa-miR-944	5	6.69052E-05
hsa-miR-548av-5p	4	5.35242E-05
hsa-miR-4424	4	5.35242E-05
hsa-miR-23a-5p	4	5.35242E-05
hsa-miR-4638-3p	4	5.35242E-05
hsa-miR-363-3p	4	5.35242E-05
hsa-miR-4676-3p	4	5.35242E-05
hsa-miR-495-3p	4	5.35242E-05
hsa-miR-433	4	5.35242E-05
hsa-miR-2114-5p	4	5.35242E-05
hsa-miR-2682-5p	4	5.35242E-05
hsa-miR-5699	4	5.35242E-05
hsa-miR-153	4	5.35242E-05
hsa-miR-548ah-3p	4	5.35242E-05
hsa-miR-548s	4	5.35242E-05
hsa-miR-1179	4	5.35242E-05
hsa-miR-6511a-3p	4	5.35242E-05
hsa-miR-3155b	4	5.35242E-05
hsa-miR-548v	4	5.35242E-05
hsa-miR-449b-5p	4	5.35242E-05
hsa-miR-23b-5p	4	5.35242E-05
hsa-miR-6501-5p	4	5.35242E-05
hsa-miR-4454	4	5.35242E-05
hsa-miR-3130-5p	4	5.35242E-05
hsa-miR-676-3p	4	5.35242E-05
hsa-miR-4651	4	5.35242E-05
hsa-miR-2964a-3p	4	5.35242E-05
hsa-miR-6720-3p	4	5.35242E-05
hsa-miR-379-3p	4	5.35242E-05
hsa-miR-4532	3	4.01431E-05
hsa-miR-485-3p	3	4.01431E-05
hsa-miR-380-3p	3	4.01431E-05
hsa-miR-5010-5p	3	4.01431E-05

hsa-miR-450a-3p	3	4.01431E-05
hsa-miR-636	3	4.01431E-05
hsa-miR-891a	3	4.01431E-05
hsa-miR-3121-3p	3	4.01431E-05
hsa-miR-3935	3	4.01431E-05
hsa-miR-3074-5p	3	4.01431E-05
hsa-miR-204-5p	3	4.01431E-05
hsa-miR-4470	3	4.01431E-05
hsa-miR-3150a-5p	3	4.01431E-05
hsa-miR-3620-5p	3	4.01431E-05
hsa-miR-4728-3p	3	4.01431E-05
hsa-miR-4741	3	4.01431E-05
hsa-miR-4785	3	4.01431E-05
hsa-miR-127-5p	3	4.01431E-05
hsa-miR-197-5p	3	4.01431E-05
hsa-miR-6500-3p	3	4.01431E-05
hsa-miR-4665-5p	3	4.01431E-05
hsa-miR-4660	3	4.01431E-05
hsa-miR-5094	3	4.01431E-05
hsa-miR-655	3	4.01431E-05
hsa-miR-3150a-3p	3	4.01431E-05
hsa-miR-876-3p	3	4.01431E-05
hsa-miR-3622a-5p	3	4.01431E-05
hsa-miR-4443	3	4.01431E-05
hsa-miR-664b-5p	3	4.01431E-05
hsa-miR-4761-3p	3	4.01431E-05
hsa-miR-5585-3p	3	4.01431E-05
hsa-miR-1304-5p	3	4.01431E-05
hsa-miR-299-5p	3	4.01431E-05
hsa-miR-548p	3	4.01431E-05
hsa-miR-586	3	4.01431E-05
hsa-miR-3657	3	4.01431E-05
hsa-miR-5001-3p	3	4.01431E-05
hsa-miR-5697	3	4.01431E-05
hsa-miR-643	3	4.01431E-05
hsa-miR-501-5p	3	4.01431E-05
hsa-miR-5008-5p	3	4.01431E-05
hsa-miR-3664-5p	3	4.01431E-05
hsa-miR-548z	3	4.01431E-05
hsa-miR-485-5p	3	4.01431E-05
hsa-miR-1278	3	4.01431E-05
hsa-miR-4684-3p	3	4.01431E-05
hsa-miR-1272	3	4.01431E-05
hsa-miR-3144-5p	3	4.01431E-05
hsa-miR-5187-5p	3	4.01431E-05
hsa-miR-4492	2	2.67621E-05
hsa-miR-2114-3p	2	2.67621E-05
hsa-miR-144-5p	2	2.67621E-05

hsa-miR-6509-5p	2	2.67621E-05
hsa-miR-887	2	2.67621E-05
hsa-miR-3155a	2	2.67621E-05
hsa-miR-4425	2	2.67621E-05
hsa-miR-192-3p	2	2.67621E-05
hsa-miR-4689	2	2.67621E-05
hsa-miR-1343	2	2.67621E-05
hsa-miR-378e	2	2.67621E-05
hsa-miR-4659a-3p	2	2.67621E-05
hsa-miR-943	2	2.67621E-05
hsa-miR-664b-3p	2	2.67621E-05
hsa-miR-4479	2	2.67621E-05
hsa-miR-1228-3p	2	2.67621E-05
hsa-miR-4511	2	2.67621E-05
hsa-miR-376b-3p	2	2.67621E-05
hsa-miR-4758-3p	2	2.67621E-05
hsa-miR-1237-3p	2	2.67621E-05
hsa-miR-3064-5p	2	2.67621E-05
hsa-miR-4688	2	2.67621E-05
hsa-miR-1538	2	2.67621E-05
hsa-miR-3198	2	2.67621E-05
hsa-miR-3168	2	2.67621E-05
hsa-miR-4677-5p	2	2.67621E-05
hsa-miR-610	2	2.67621E-05
hsa-miR-139-3p	2	2.67621E-05
hsa-miR-4749-5p	2	2.67621E-05
hsa-miR-4467	2	2.67621E-05
hsa-miR-1250	2	2.67621E-05
hsa-miR-4485	2	2.67621E-05
hsa-miR-3163	2	2.67621E-05
hsa-miR-5583-5p	2	2.67621E-05
hsa-miR-146b-3p	2	2.67621E-05
hsa-miR-548ak	2	2.67621E-05
hsa-miR-3074-3p	2	2.67621E-05
hsa-miR-548ay-3p	2	2.67621E-05
hsa-miR-618	2	2.67621E-05
hsa-miR-3177-5p	2	2.67621E-05
hsa-miR-581	2	2.67621E-05
hsa-miR-4640-3p	2	2.67621E-05
hsa-miR-1255b-5p	2	2.67621E-05
hsa-miR-1234-3p	2	2.67621E-05
hsa-miR-19a-5p	2	2.67621E-05
hsa-miR-23c	2	2.67621E-05
hsa-miR-3157-3p	2	2.67621E-05
hsa-miR-3129-5p	2	2.67621E-05
hsa-miR-935	2	2.67621E-05
hsa-miR-6721-5p	2	2.67621E-05
hsa-miR-3939	2	2.67621E-05

hsa-miR-3128	2	2.67621E-05
hsa-miR-5580-5p	2	2.67621E-05
hsa-miR-548t-5p	2	2.67621E-05
hsa-miR-3936	2	2.67621E-05
hsa-miR-548au-3p	2	2.67621E-05
hsa-miR-3942-3p	2	2.67621E-05
hsa-miR-570-5p	2	2.67621E-05
hsa-miR-3130-3p	1	1.3381E-05
hsa-miR-5096	1	1.3381E-05
hsa-miR-3656	1	1.3381E-05
hsa-miR-4421	1	1.3381E-05
hsa-miR-4667-5p	1	1.3381E-05
hsa-miR-3940-3p	1	1.3381E-05
hsa-miR-3124-5p	1	1.3381E-05
hsa-miR-4746-3p	1	1.3381E-05
hsa-miR-3183	1	1.3381E-05
hsa-miR-148a-5p	1	1.3381E-05
hsa-miR-665	1	1.3381E-05
hsa-miR-4515	1	1.3381E-05
hsa-miR-4758-5p	1	1.3381E-05
hsa-miR-184	1	1.3381E-05
hsa-miR-3960	1	1.3381E-05
hsa-miR-1271-5p	1	1.3381E-05
hsa-miR-135a-5p	1	1.3381E-05
hsa-miR-615-5p	1	1.3381E-05
hsa-miR-3140-3p	1	1.3381E-05
hsa-miR-3687	1	1.3381E-05
hsa-miR-3944-5p	1	1.3381E-05
hsa-miR-877-3p	1	1.3381E-05
hsa-miR-3127-5p	1	1.3381E-05
hsa-miR-675-3p	1	1.3381E-05
hsa-miR-217	1	1.3381E-05
hsa-miR-1288	1	1.3381E-05
hsa-miR-4672	1	1.3381E-05
hsa-miR-1252	1	1.3381E-05
hsa-miR-34b-3p	1	1.3381E-05
hsa-miR-616-3p	1	1.3381E-05
hsa-miR-4683	1	1.3381E-05
hsa-miR-3181	1	1.3381E-05
hsa-miR-3160-3p	1	1.3381E-05
hsa-miR-4661-3p	1	1.3381E-05
hsa-miR-1227-3p	1	1.3381E-05
hsa-miR-34b-5p	1	1.3381E-05
hsa-miR-517a-3p	1	1.3381E-05
hsa-miR-3680-5p	1	1.3381E-05
hsa-miR-4474-3p	1	1.3381E-05
hsa-miR-3691-5p	1	1.3381E-05
hsa-miR-4727-3p	1	1.3381E-05

hsa-miR-3146	1	1.3381E-05
hsa-miR-3605-3p	1	1.3381E-05
hsa-miR-4797-3p	1	1.3381E-05
hsa-miR-548a-5p	1	1.3381E-05
hsa-miR-5587-3p	1	1.3381E-05
hsa-miR-4669	1	1.3381E-05
hsa-miR-3620-3p	1	1.3381E-05
hsa-miR-3917	1	1.3381E-05
hsa-miR-4793-3p	1	1.3381E-05
hsa-miR-4765	1	1.3381E-05
hsa-miR-3190-3p	1	1.3381E-05
hsa-miR-186-3p	1	1.3381E-05
hsa-miR-6505-5p	1	1.3381E-05
hsa-miR-3934-3p	1	1.3381E-05
hsa-miR-3677-5p	1	1.3381E-05
hsa-miR-4751	1	1.3381E-05
hsa-miR-675-5p	1	1.3381E-05
hsa-miR-624-5p	1	1.3381E-05
hsa-miR-3145-5p	1	1.3381E-05
hsa-miR-6513-5p	1	1.3381E-05
hsa-miR-337-3p	1	1.3381E-05
hsa-miR-598	1	1.3381E-05
hsa-miR-3153	1	1.3381E-05
hsa-miR-329	1	1.3381E-05
hsa-miR-4483	1	1.3381E-05
hsa-miR-4706	1	1.3381E-05
hsa-miR-3148	1	1.3381E-05
hsa-miR-4762-3p	1	1.3381E-05
hsa-miR-4457	1	1.3381E-05
hsa-miR-3145-3p	1	1.3381E-05
hsa-miR-1273c	1	1.3381E-05
hsa-miR-4784	1	1.3381E-05
hsa-miR-548f	1	1.3381E-05
hsa-miR-149-3p	1	1.3381E-05
hsa-miR-124-3p	1	1.3381E-05
hsa-miR-4519	1	1.3381E-05
hsa-miR-579	1	1.3381E-05
hsa-miR-1185-5p	1	1.3381E-05
hsa-miR-1273d	1	1.3381E-05
hsa-miR-4999-5p	1	1.3381E-05
hsa-miR-1233-1-5p	1	1.3381E-05
hsa-miR-3157-5p	1	1.3381E-05
hsa-miR-135b-5p	1	1.3381E-05
hsa-miR-512-3p	1	1.3381E-05
hsa-miR-3187-5p	1	1.3381E-05
hsa-miR-224-3p	1	1.3381E-05
hsa-miR-6508-3p	1	1.3381E-05
hsa-miR-4642	1	1.3381E-05

hsa-miR-4781-3p	1	1.3381E-05
hsa-miR-5687	1	1.3381E-05
hsa-miR-1257	1	1.3381E-05
hsa-miR-548q	1	1.3381E-05
hsa-miR-548ap-3p	1	1.3381E-05
hsa-miR-4742-5p	1	1.3381E-05
hsa-miR-3192	1	1.3381E-05
hsa-miR-1256	1	1.3381E-05
hsa-miR-708-5p	1	1.3381E-05
hsa-miR-2467-3p	1	1.3381E-05
hsa-miR-3914	1	1.3381E-05
hsa-miR-548ax	1	1.3381E-05
hsa-miR-4442	1	1.3381E-05
hsa-miR-2964a-5p	1	1.3381E-05
hsa-miR-548c-3p	1	1.3381E-05
hsa-miR-5583-3p	1	1.3381E-05
hsa-miR-3188	1	1.3381E-05
hsa-miR-3136-5p	1	1.3381E-05
hsa-miR-5689	1	1.3381E-05
hsa-miR-4783-3p	1	1.3381E-05
hsa-miR-376a-5p	1	1.3381E-05
hsa-miR-3120-5p	1	1.3381E-05
hsa-miR-1537	1	1.3381E-05
hsa-miR-548aq-3p	1	1.3381E-05
hsa-miR-4738-3p	1	1.3381E-05
hsa-miR-4787-3p	1	1.3381E-05
hsa-miR-544a	1	1.3381E-05
hsa-miR-548ac	1	1.3381E-05
hsa-miR-1226-5p	1	1.3381E-05
hsa-miR-216a-5p	1	1.3381E-05
hsa-miR-346	1	1.3381E-05
hsa-miR-4786-5p	1	1.3381E-05
hsa-miR-1185-1-3p	1	1.3381E-05
hsa-miR-5090	1	1.3381E-05
hsa-miR-4687-5p	1	1.3381E-05
hsa-miR-451a	0	0
hsa-miR-1225-5p	0	0
hsa-miR-3663-3p	0	0
hsa-miR-888-5p	0	0
hsa-miR-1290	0	0
hsa-miR-4664-5p	0	0
hsa-miR-4449	0	0
hsa-miR-144-3p	0	0
hsa-miR-496	0	0
hsa-miR-3679-5p	0	0
hsa-miR-4687-3p	0	0
hsa-miR-320e	0	0
hsa-miR-3131	0	0

hsa-miR-4634	0	0
hsa-miR-371a-5p	0	0
hsa-miR-337-5p	0	0
hsa-miR-3122	0	0
hsa-miR-3173-3p	0	0
hsa-miR-483-5p	0	0
hsa-miR-431-3p	0	0
hsa-miR-3648	0	0
hsa-miR-223-3p	0	0
hsa-miR-204-3p	0	0
hsa-miR-1291	0	0
hsa-miR-1292-3p	0	0
hsa-miR-1228-5p	0	0
hsa-miR-3663-5p	0	0
hsa-miR-323a-5p	0	0
hsa-miR-6515-5p	0	0
hsa-miR-3180-3p	0	0
hsa-miR-4739	0	0
hsa-miR-1185-2-3p	0	0
hsa-miR-4721	0	0
hsa-miR-378i	0	0
hsa-miR-541-3p	0	0
hsa-miR-4659a-5p	0	0
hsa-miR-4700-3p	0	0
hsa-miR-4755-3p	0	0
hsa-miR-3165	0	0
hsa-miR-1236-5p	0	0
hsa-miR-214-3p	0	0
hsa-miR-6087	0	0
hsa-miR-155-5p	0	0
hsa-miR-4743-3p	0	0
hsa-miR-509-3-5p	0	0
hsa-miR-4674	0	0
hsa-miR-3200-5p	0	0
hsa-miR-541-5p	0	0
hsa-miR-663a	0	0
hsa-miR-483-3p	0	0
hsa-miR-1289	0	0
hsa-miR-4638-5p	0	0
hsa-miR-4525	0	0
hsa-miR-5001-5p	0	0
hsa-miR-4710	0	0
hsa-miR-3925-5p	0	0
hsa-miR-1273f	0	0
hsa-miR-1295a	0	0
hsa-miR-3685	0	0
hsa-miR-549a	0	0
hsa-miR-5006-5p	0	0

hsa-miR-3135a	0	0
hsa-miR-1	0	0
hsa-miR-4757-3p	0	0
hsa-miR-3655	0	0
hsa-miR-4753-5p	0	0
hsa-miR-3162-5p	0	0
hsa-miR-4646-5p	0	0
hsa-miR-4734	0	0
hsa-miR-516b-5p	0	0
hsa-miR-383	0	0
hsa-miR-4670-3p	0	0
hsa-miR-150-5p	0	0
hsa-miR-1227-5p	0	0
hsa-miR-3133	0	0
hsa-miR-199b-5p	0	0
hsa-miR-3929	0	0
hsa-miR-4745-5p	0	0
hsa-miR-4700-5p	0	0
hsa-miR-505-5p	0	0
hsa-miR-4725-5p	0	0
hsa-miR-187-3p	0	0
hsa-miR-4770	0	0
hsa-miR-632	0	0
hsa-miR-4507	0	0
hsa-miR-1244	0	0
hsa-miR-933	0	0
hsa-miR-1298	0	0
hsa-miR-211-5p	0	0
hsa-miR-4695-5p	0	0
hsa-miR-296-3p	0	0
hsa-miR-145-5p	0	0
hsa-miR-1285-5p	0	0
hsa-miR-3919	0	0
