# Supplementary material for: The exosomal miRNA/FOXN3 axis empowers fibroblasts with tumor-promoting functions via enhanced migration and altered secretome
Source: J Nanobiotechnology. 2026 Jun 5;24:742. doi: 10.1186/s12951-026-04633-6 (PMC13459620; doi:10.1186/s12951-026-04633-6)
Supplement: Supplementary file 4 [file 12951_2026_4633_MOESM5_ESM.pdf]

Table S4 mRNA profiles of MRC5 cells with FOXN3 shRNAs and MRC5 cells with scrambled shRNAs

Gene symbol	Count (MRC5 with scrambled shRNAs [scr])	Count (MRC5 with FOXN3 shRNAs [sh])	Log2(sh/scr) normalized	*P* value	*Q* value (Benjamini et al. 1995)
RNF128	268	553	1.460198612	3.07E-45	1.03E-43
C10orf10	302	569	1.329032235	1.53E-40	4.42E-39
GCNT4	220	411	1.316787002	2.33E-29	4.72E-28
SEMA7A	3755	6592	1.227055467	0	0
BMF	219	384	1.225327573	8.66E-25	1.50E-23
SLC2A3	2795	4587	1.129854756	2.81E-243	7.45E-241
GALNT3	275	445	1.109525849	1.55E-24	2.66E-23
HDAC9	395	611	1.044471859	3.93E-30	8.22E-29
DTNA	617	951	1.039326983	1.37E-45	4.59E-44
TNFSF4	4531	6983	1.039169614	0	0
C3orf52	338	518	1.031080983	2.73E-25	4.82E-24
DSP	3589	5474	1.024165643	2.05E-247	5.94E-245
TGFB2	784	1185	1.011113631	5.93E-54	2.38E-52
PMAIP1	3619	5461	1.008726181	8.77E-241	2.28E-238
GLT25D2	429	645	1.003473645	8.44E-30	1.74E-28
CPA4	4016	6022	0.999635571	2.14E-261	7.02E-259
INTU	492	736	0.996199582	2.20E-33	5.16E-32
AMIGO2	575	854	0.985826246	7.87E-38	2.09E-36
OXTR	5111	7560	0.979932775	0	0
RGS4	2442	3602	0.975887113	4.04E-151	5.35E-149
C21orf7	596	870	0.960855202	5.70E-37	1.46E-35
AMMECR1	1688	2424	0.937226927	8.54E-96	6.37E-94
ANKRD46	614	879	0.932776642	1.30E-35	3.18E-34
TGM2	11370	16093	0.916353171	0	0
CEACAM19	321	449	0.899294279	6.00E-18	7.37E-17
ITGB8	518	715	0.880143276	1.05E-26	1.96E-25
DNAH5	1787	2463	0.878029125	2.27E-87	1.56E-85
GRPR	424	577	0.859659186	4.54E-21	6.54E-20
SCG5	443	592	0.833442609	1.51E-20	2.12E-19

PALM2	401	531	0.820261756	3.93E-18	4.90E-17
A2M	757	1001	0.818228901	1.28E-32	2.92E-31
HIPK3	3623	4787	0.817089132	8.54E-149	1.11E-146
LCOR	3021	3978	0.812169224	1.00E-122	1.01E-120
RGS5	903	1187	0.809674174	1.04E-37	2.74E-36
SERPINB7	3707	4854	0.804074125	8.49E-147	1.05E-144
UGCG	2289	2983	0.797198647	1.17E-89	8.30E-88
DCP2	2268	2928	0.783647045	1.57E-85	1.06E-83
CLDN12	2167	2782	0.775575398	5.99E-80	3.62E-78
KRT18	4655	5971	0.774343533	6.72E-169	1.03E-166
LEPREL1	8594	11007	0.77217176	0	0
LRRN3	5185	6636	0.771122029	3.78E-186	6.54E-184
KATNAL1	3882	4964	0.769855129	2.30E-139	2.68E-137
KBTBD8	331	423	0.768978578	2.33E-13	2.07E-12
TMEM2	5369	6859	0.768496983	3.25E-191	5.96E-189
GDF5	804	1027	0.768320907	3.71E-30	7.76E-29
ENTPD7	2048	2616	0.768298957	4.22E-74	2.38E-72
KLF9	826	1055	0.768181444	6.33E-31	1.37E-29
RHOBTB1	1189	1516	0.76567317	1.88E-43	5.92E-42
DDAH1	8922	11290	0.754758565	8.45E-304	3.19E-301
SORT1	4775	6041	0.754438785	2.13E-163	3.16E-161
IGFBP3	6424	8107	0.750848382	8.62E-217	2.03E-214
FGF1	786	976	0.727503967	2.75E-26	5.03E-25
NEGR1	2509	3115	0.727271824	4.83E-80	2.95E-78
GXYLT1	3340	4118	0.717247859	1.01E-102	8.10E-101
PRICKLE1	1252	1537	0.711034735	6.59E-39	1.79E-37
PLEKHA8	1401	1717	0.708585215	5.21E-43	1.61E-41
VGLL3	5633	6859	0.699246916	1.83E-162	2.68E-160
INMT	524	637	0.696878692	1.55E-16	1.72E-15
GPR176	6600	8019	0.696108446	4.04E-188	7.19E-186
RNF38	1780	2149	0.686940373	1.20E-50	4.53E-49
DUSP5	1560	1878	0.682803166	4.96E-44	1.59E-42

DYRK2	1616	1942	0.680268134	3.48E-45	1.15E-43
PIGA	341	407	0.670409187	1.83E-10	1.27E-09
KRTAP2-3	677	805	0.664985081	5.51E-19	7.16E-18
ARRDC4	1166	1384	0.662428286	2.83E-31	6.23E-30
NREP	2402	2847	0.660358474	3.74E-62	1.73E-60
CHIC2	708	838	0.658353015	2.22E-19	2.95E-18
NNMT	1955	2304	0.652124241	1.44E-49	5.28E-48
KCTD20	4672	5506	0.652116467	7.09E-116	6.55E-114
MEST	11944	14059	0.650360042	2.38E-291	8.71E-289
KIAA1199	6075	7136	0.647383338	1.07E-147	1.33E-145
OGFRL1	2262	2648	0.642456325	2.89E-55	1.20E-53
ZNF652	1661	1944	0.642128277	5.24E-41	1.54E-39
SOX5	483	565	0.64137981	5.12E-13	4.42E-12
CCDC80	24859	29068	0.640815681	0	0
MRVI1	4652	5439	0.640642461	5.36E-111	4.67E-109
DNAJC27	373	435	0.636991902	3.01E-10	2.03E-09
PTPDC1	502	585	0.635901392	3.03E-13	2.66E-12
FGD6	875	1019	0.634951261	7.07E-22	1.07E-20
KIT	2396	2787	0.633237226	1.21E-56	5.15E-55
ACTA2	4913	5706	0.63102764	2.59E-113	2.32E-111
OLR1	706	819	0.6293474	1.23E-17	1.48E-16
ATP8B1	5369	6223	0.628108973	2.10E-122	2.11E-120
NEDD9	1728	1997	0.623883247	4.65E-40	1.33E-38
PLCB4	5666	6535	0.621008766	6.65E-126	6.84E-124
ZC3HAV1L	340	392	0.62047104	5.20E-09	3.11E-08
DMD	772	888	0.617110961	2.17E-18	2.75E-17
MBTD1	730	836	0.61075861	4.09E-17	4.71E-16
PRPS1	3968	4522	0.603701098	1.73E-83	1.12E-81
PLEKHA1	1465	1668	0.602370756	8.58E-32	1.92E-30
DIAPH2	2139	2435	0.602137422	1.36E-45	4.58E-44
LMO7	21831	24716	0.594219194	0	0
MYEF2	882	998	0.593413292	3.40E-19	4.47E-18

KLHL24	1347	1519	0.588524151	5.56E-28	1.08E-26
ZNF367	376	424	0.588483735	6.90E-09	4.07E-08
EIF4EBP2	7537	8494	0.587605848	7.15E-148	9.00E-146
AKAP12	3953	4454	0.587305735	2.18E-78	1.29E-76
MPP5	7023	7906	0.586012652	4.11E-137	4.65E-135
KLHL28	1091	1228	0.585811591	9.32E-23	1.48E-21
RAB11FIP1	2233	2507	0.582130877	3.12E-44	1.02E-42
TET3	797	893	0.579232583	1.15E-16	1.28E-15
CDK6	13720	15360	0.578049866	4.21E-258	1.34E-255
DNAJB4	3553	3973	0.576343237	7.54E-68	3.89E-66
ABHD4	1157	1293	0.575485542	3.56E-23	5.75E-22
NEDD4L	1314	1466	0.57307196	7.11E-26	1.29E-24
POU2F1	1242	1382	0.569244574	3.28E-24	5.52E-23
GAN	355	395	0.56918576	5.75E-08	3.08E-07
GRAMD3	797	883	0.562985845	9.47E-16	1.00E-14
NAP1L5	745	824	0.560556044	1.07E-14	1.04E-13
DAAM2	947	1046	0.558598653	3.88E-18	4.86E-17
DOCK9	1591	1756	0.557511141	2.92E-29	5.89E-28
PRKAB2	2452	2705	0.556821747	4.66E-44	1.51E-42
CD274	456	503	0.556676707	1.95E-09	1.22E-08
ARHGAP42	770	849	0.55605824	6.71E-15	6.65E-14
FAM214A	1835	2022	0.555155066	3.38E-33	7.89E-32
BTBD3	4210	4635	0.553901237	1.60E-73	8.93E-72
MAP9	3178	3498	0.553563296	7.08E-56	2.96E-54
COL4A1	50732	55773	0.55182297	0	0
KIAA1467	454	499	0.55149965	3.07E-09	1.89E-08
BMPER	394	433	0.551323527	3.39E-08	1.86E-07
KLHL15	793	871	0.550503985	5.33E-15	5.34E-14
ANKRD52	9324	10240	0.550346938	2.85E-158	3.99E-156
CREBRF	2267	2481	0.545289476	4.12E-39	1.13E-37
SULF1	1093	1196	0.54507612	1.08E-19	1.46E-18
SLC35D2	940	1026	0.541450201	6.30E-17	7.16E-16

PRDM1	440	480	0.540683014	1.13E-08	6.49E-08
C1orf198	2549	2779	0.539786593	7.72E-43	2.37E-41
LUM	2785	3035	0.539171321	1.57E-46	5.40E-45
SYNM	1055	1149	0.538287931	1.37E-18	1.76E-17
XRCC4	644	701	0.537505888	6.69E-12	5.31E-11
TSEN15	1979	2152	0.536058597	3.66E-33	8.47E-32
RMND5A	1045	1135	0.534341487	3.70E-18	4.63E-17
CYR61	19529	21199	0.533530267	8.43E-308	3.39E-305
LGR4	1454	1577	0.532307523	1.88E-24	3.20E-23
LZTFL1	1097	1188	0.530123442	1.10E-18	1.41E-17
BMP2K	1461	1581	0.529033321	2.96E-24	5.00E-23
TMEM64	1841	1990	0.527430936	6.00E-30	1.25E-28
SH3TC2	783	844	0.523382823	1.95E-13	1.75E-12
RNF217	431	464	0.521589068	5.51E-08	2.95E-07
LRIF1	1349	1452	0.521303237	7.38E-22	1.12E-20
SGK1	891	959	0.521257515	5.82E-15	5.81E-14
POU2F2	395	425	0.52076232	2.07E-07	1.04E-06
PNRC2	4109	4418	0.51975822	1.01E-62	4.72E-61
PAWR	1410	1515	0.518774763	1.50E-22	2.35E-21
TMEM231	581	623	0.515846131	4.56E-10	3.02E-09
DDX3Y	4326	4633	0.514065149	2.05E-64	9.95E-63
CTGF	28226	30228	0.514012965	0	0
TRIM55	1794	1917	0.510822578	2.28E-27	4.33E-26
KIF3B	4871	5202	0.510000548	4.32E-71	2.33E-69
MAPK6	5569	5943	0.50892522	1.20E-80	7.45E-79
CPEB4	2656	2833	0.508227587	2.73E-39	7.54E-38
TEAD4	437	466	0.507848807	1.07E-07	5.54E-07
ZAK	14549	15501	0.506593426	1.65E-205	3.49E-203
AKT3	3414	3634	0.505247493	2.17E-49	7.95E-48
TET2	2167	2305	0.504219729	7.59E-32	1.71E-30
CLDN1	2131	2264	0.502495497	3.99E-31	8.71E-30
GPR37	1651	1754	0.502460759	1.75E-24	3.00E-23

CTSO	999	1061	0.502020205	2.09E-15	2.14E-14
FGD4	1695	1799	0.501062045	5.98E-25	1.04E-23
PTPN13	6458	6852	0.500589842	5.33E-90	3.82E-88
NRK	648	687	0.499468418	2.07E-10	1.43E-09
CPED1	9686	10263	0.498631734	6.72E-133	7.41E-131
GREM1	30746	32576	0.498562868	0	0
FMN1	7659	8114	0.498409396	2.15E-105	1.79E-103
NAALADL2	1161	1229	0.497269075	2.51E-17	2.95E-16
SLC1A4	758	802	0.49655652	8.43E-12	6.63E-11
NIPSNAP3A	1559	1647	0.494371759	1.85E-22	2.88E-21
IL7R	6199	6545	0.49350982	6.87E-84	4.50E-82
BBS4	967	1020	0.492133489	2.16E-14	2.05E-13
ATP10D	5679	5989	0.491830352	1.86E-76	1.07E-74
FCHSD2	2825	2978	0.491245018	7.77E-39	2.11E-37
JAZF1	1041	1096	0.489429861	3.26E-15	3.30E-14
LPCAT2	1532	1610	0.486796523	2.01E-21	2.96E-20
FAM217B	1281	1344	0.484414794	5.38E-18	6.66E-17
ZBTB20	412	432	0.483539107	9.93E-07	4.57E-06
ADAMTS15	392	411	0.483436871	1.83E-06	8.12E-06
TP53INP2	2749	2879	0.481813014	2.38E-36	5.95E-35
CRIM1	31149	32615	0.481501912	0	0
KLHDC3	1885	1972	0.48024716	2.78E-25	4.91E-24
NRAS	6342	6634	0.480093266	6.50E-81	4.07E-79
TTTY15	1524	1593	0.479035496	1.21E-20	1.70E-19
SMIM13	2833	2959	0.477931227	8.67E-37	2.19E-35
UBE2W	2038	2128	0.477496231	6.93E-27	1.29E-25
LYPLA1	3364	3512	0.477267271	3.10E-43	9.71E-42
MAPK8	751	783	0.475351532	8.86E-11	6.31E-10
TPM1	29035	30268	0.475152448	0	0
SPATA2	553	576	0.473941463	2.90E-08	1.60E-07
SCUBE3	1889	1967	0.473526388	1.28E-24	2.20E-23
STX3	752	783	0.473431777	1.04E-10	7.35E-10

PKP4	1919	1998	0.473354003	5.74E-25	1.00E-23
IGIP	851	886	0.473299699	6.37E-12	5.07E-11
HSPB7	713	742	0.472669242	3.38E-10	2.27E-09
NPR3	866	901	0.472312213	4.65E-12	3.73E-11
YAF2	761	791	0.470933373	1.03E-10	7.29E-10
STARD4-AS1	717	745	0.470419438	3.72E-10	2.48E-09
HDAC5	1780	1848	0.469239647	7.17E-23	1.15E-21
CCPG1	579	601	0.468953774	2.01E-08	1.13E-07
SSPN	664	689	0.468472873	1.94E-09	1.22E-08
PTGER4	517	536	0.467220852	1.28E-07	6.61E-07
DAB2	13487	13981	0.46705021	3.53E-160	5.05E-158
TP53INP1	4535	4699	0.466403349	5.96E-55	2.44E-53
TRIB3	818	847	0.465413258	3.71E-11	2.74E-10
STK39	3626	3754	0.465201857	4.68E-44	1.51E-42
SNX24	907	939	0.465174739	3.35E-12	2.73E-11
PCTP	539	558	0.465131981	8.03E-08	4.23E-07
TRIT1	676	699	0.463421341	2.15E-09	1.34E-08
CAMK2D	9656	9983	0.463199882	3.14E-113	2.79E-111
LRRC32	593	613	0.463007101	2.13E-08	1.19E-07
PGGT1B	1383	1429	0.462356892	1.33E-17	1.60E-16
ZNF267	525	542	0.461127561	1.55E-07	7.87E-07
PALLD	19722	20359	0.46101297	6.82E-227	1.67E-224
MDM1	566	584	0.460318448	5.40E-08	2.90E-07
CALD1	63113	65115	0.460204853	0	0
FAM167A	516	532	0.459207312	2.24E-07	1.12E-06
NEK4	1300	1340	0.458873509	2.15E-16	2.37E-15
LPP	29822	30697	0.456872786	0	0
TRAF5	735	756	0.455794116	8.74E-10	5.64E-09
DNAJB9	1370	1408	0.454623573	6.99E-17	7.94E-16
SH3RF1	4163	4278	0.454465056	6.22E-48	2.22E-46
PPP1R3B	773	794	0.453822725	3.90E-10	2.60E-09
ZBTB10	903	927	0.452995483	1.47E-11	1.13E-10

TTC39B	533	547	0.452557432	2.21E-07	1.10E-06
FAM73A	3364	3449	0.451152555	1.75E-38	4.70E-37
VPS36	3173	3252	0.450631868	2.61E-36	6.52E-35
SERPINE1	91906	94132	0.44967833	0	0
RASA2	709	725	0.447347499	3.61E-09	2.20E-08
LIMS1	5799	5924	0.445919634	1.74E-63	8.30E-62
ENDOD1	3542	3617	0.445381518	2.28E-39	6.31E-38
CCDC71L	3582	3656	0.444652865	1.16E-39	3.27E-38
FZD6	5706	5822	0.444187224	5.44E-62	2.48E-60
MTHFD2	4081	4162	0.443506386	1.05E-44	3.47E-43
TUFT1	843	858	0.440597148	2.45E-10	1.68E-09
GPR180	3044	3098	0.440520917	2.56E-33	5.99E-32
IFI16	6481	6595	0.440308357	6.97E-69	3.70E-67
LEPROT	5845	5945	0.439625917	4.21E-62	1.93E-60
SH3PXD2A	14519	14767	0.439586805	2.02E-151	2.71E-149
NDFIP2	3419	3475	0.438590687	7.03E-37	1.79E-35
SEMA6D	1787	1816	0.4383767	4.87E-20	6.66E-19
MSRB3	5527	5613	0.437427578	3.28E-58	1.41E-56
FZD4	1291	1311	0.437330817	7.76E-15	7.64E-14
MTMR9	2217	2251	0.437109418	2.47E-24	4.18E-23
DNAL1	958	972	0.43608279	2.49E-11	1.87E-10
TSC22D3	428	434	0.435236378	8.53E-06	3.45E-05
B3GALTL	724	734	0.434942498	7.23E-09	4.25E-08
PARP12	388	393	0.433624792	2.43E-05	9.11E-05
MYO1E	2717	2752	0.433618036	5.63E-29	1.13E-27
UBXN2B	562	569	0.433010654	3.92E-07	1.90E-06
SLC4A7	8241	8327	0.430129545	6.66E-83	4.27E-81
PRKAA2	384	388	0.430102473	3.14E-05	0.000115767
GABARAPL1	6695	6762	0.429518092	1.69E-67	8.66E-66
SLC38A1	6359	6422	0.429374888	3.70E-64	1.79E-62
AKAP13	4100	4140	0.42915899	5.89E-42	1.78E-40
NEDD4	8906	8991	0.428856105	6.94E-89	4.86E-87

OTUD4	3610	3643	0.42828029	5.42E-37	1.39E-35
PURA	2901	2922	0.425558014	1.13E-29	2.33E-28
SESN2	2229	2244	0.424828191	4.24E-23	6.84E-22
CEBPD	896	902	0.424780833	3.50E-10	2.34E-09
LMOD1	2313	2328	0.424477924	7.19E-24	1.20E-22
LMLN	1033	1039	0.423507532	1.87E-11	1.43E-10
CTAGE5	1172	1178	0.422519101	9.65E-13	8.12E-12
RCHY1	1034	1039	0.4221116	2.15E-11	1.63E-10
BRWD3	2116	2126	0.421954101	1.03E-21	1.54E-20
ATP6V1G1	3615	3632	0.421920687	6.34E-36	1.56E-34
SYNE1	9196	9238	0.421726212	1.53E-88	1.06E-86
INPP5A	2235	2245	0.421592745	8.32E-23	1.33E-21
CBLB	701	704	0.421313116	3.77E-08	2.06E-07
WEE1	1656	1663	0.421237628	2.81E-17	3.28E-16
DYNC2LI1	548	550	0.420407857	1.22E-06	5.53E-06
ASNS	2219	2227	0.420344022	1.60E-22	2.51E-21
SDC2	3725	3738	0.420178275	1.18E-36	2.98E-35
ICK	2051	2058	0.420067622	6.56E-21	9.33E-20
TRAF6	1221	1224	0.418692489	5.50E-13	4.74E-12
ITGB3	1225	1228	0.418680943	5.05E-13	4.37E-12
PDLIM5	7009	7026	0.418647084	6.75E-67	3.36E-65
TMEM66	9169	9188	0.418138591	1.02E-86	6.97E-85
ITGA1	6155	6162	0.416791957	1.99E-58	8.62E-57
SCML1	1064	1065	0.416507411	2.16E-11	1.63E-10
LATS2	4496	4500	0.416435098	4.41E-43	1.37E-41
TES	1532	1533	0.416093531	1.01E-15	1.07E-14
OSTM1	3218	3220	0.416048494	2.91E-31	6.38E-30
CTDSPL	2189	2190	0.415811047	9.17E-22	1.38E-20
APH1B	1604	1603	0.414252415	3.02E-16	3.30E-15
ABHD13	1389	1388	0.4141131	2.91E-14	2.74E-13
HMBOX1	722	721	0.413152554	4.57E-08	2.47E-07
BRWD1	4248	4242	0.413112986	3.89E-40	1.11E-38

TUBE1	556	555	0.41255502	1.66E-06	7.44E-06
SERPINB1	1101	1099	0.412529049	1.58E-11	1.21E-10
FOXN2	1547	1544	0.412351688	1.39E-15	1.45E-14
TMEM41B	2452	2447	0.412207254	9.08E-24	1.51E-22
ZNF264	1816	1812	0.411970885	5.40E-18	6.67E-17
ZNF281	2404	2398	0.411546894	2.93E-23	4.74E-22
HSPB8	1789	1783	0.410305447	1.31E-17	1.57E-16
SGPP1	2279	2271	0.410078913	5.61E-22	8.54E-21
RAB36	477	475	0.409090379	1.10E-05	4.34E-05
RNFT1	663	660	0.408609286	2.25E-07	1.12E-06
WDR89	586	583	0.407747351	1.19E-06	5.44E-06
ALG10B	851	846	0.406650663	5.35E-09	3.20E-08
ZNF24	5092	5062	0.40662722	3.14E-46	1.07E-44
TTC33	1185	1178	0.406604612	5.75E-12	4.59E-11
TRIM23	1521	1511	0.405635639	7.19E-15	7.09E-14
ATXN1L	2255	2240	0.405523431	2.75E-21	4.03E-20
CALCOCO1	3250	3224	0.403564157	1.14E-29	2.35E-28
EFHC1	862	855	0.403388682	5.78E-09	3.44E-08
LOC92249	482	478	0.403129604	1.35E-05	5.28E-05
DENND6A	834	827	0.402992077	1.06E-08	6.09E-08
TTN	924	916	0.402606878	1.78E-09	1.12E-08
SOWAHC	997	988	0.402069669	4.36E-10	2.90E-09
LACTB2	508	503	0.400882035	8.98E-06	3.62E-05
MT1E	3448	3410	0.399164097	1.10E-30	2.35E-29
SKP2	1656	1637	0.398503781	1.63E-15	1.69E-14
SOS2	2399	2371	0.398214637	9.59E-22	1.44E-20
LINC00152	2226	2199	0.397546143	3.18E-20	4.39E-19
KIAA0247	1669	1648	0.39688442	1.68E-15	1.73E-14
AGPAT9	389	384	0.396488287	0.0001229	0.000403159
ZNF844	453	447	0.395915913	3.51E-05	0.000128255
TTC8	1919	1893	0.395471831	1.80E-17	2.14E-16
BAK1	1141	1125	0.394778342	5.88E-11	4.26E-10

ABHD5	2087	2057	0.394263325	9.43E-19	1.22E-17
STAT3	7896	7780	0.393800297	3.91E-66	1.94E-64
IRF2	667	657	0.393358741	6.16E-07	2.90E-06
RASAL2	3267	3218	0.393350003	2.59E-28	5.06E-27
ZNF667	451	444	0.392584375	4.29E-05	0.000153659
TBC1D19	946	931	0.392093116	3.26E-09	2.00E-08
PDCD1LG2	1759	1731	0.392002374	7.18E-16	7.66E-15
ZBTB5	559	550	0.391735467	5.51E-06	2.29E-05
ZNF708	542	533	0.390994813	7.97E-06	3.24E-05
CFH	2003	1969	0.390452822	1.01E-17	1.23E-16
CITED2	9981	9807	0.389779643	2.40E-81	1.51E-79
CHML	3117	3062	0.38946826	1.48E-26	2.74E-25
AGO4	1404	1379	0.389231653	8.48E-13	7.18E-12
IGF1R	3590	3525	0.388791545	3.14E-30	6.59E-29
FNIP2	1616	1586	0.388117705	2.00E-14	1.90E-13
FAM101B	2888	2834	0.387921148	1.57E-24	2.69E-23
CARD16	1108	1087	0.387546191	2.53E-10	1.73E-09
PHACTR2	6553	6428	0.387366529	2.38E-53	9.46E-52
YOD1	837	821	0.387306731	3.93E-08	2.15E-07
CRYAB	403	395	0.386224946	0.0001443	0.000466583
C5orf28	1005	985	0.38615226	1.96E-09	1.23E-08
NCR3LG1	501	491	0.386064553	2.28E-05	8.60E-05
NAPB	788	772	0.38555735	1.13E-07	5.84E-07
LOC100129550	435	426	0.384990161	8.31E-05	0.000281468
LCLAT1	1441	1411	0.384799784	8.14E-13	6.92E-12
FKBP14	7377	7221	0.384316539	7.34E-59	3.23E-57
JHDM1D	597	584	0.383389569	4.44E-06	1.87E-05
ACSL4	11531	11274	0.382633971	3.91E-90	2.81E-88
SNX4	1651	1612	0.380663755	3.59E-14	3.37E-13
PARP9	838	818	0.380302731	7.00E-08	3.71E-07
UBL3	4299	4195	0.379821832	3.31E-34	7.84E-33
ARID3A	1272	1241	0.379556577	3.40E-11	2.52E-10

PSTPIP2	2163	2110	0.379361465	5.69E-18	7.01E-17
COMMD8	1787	1742	0.378357121	4.88E-15	4.90E-14
SHROOM3	4428	4312	0.376854088	1.26E-34	3.01E-33
MAP3K1	1329	1294	0.376648644	1.82E-11	1.39E-10
KDM4C	1125	1095	0.376158	6.66E-10	4.35E-09
TAB3	1828	1779	0.375952572	3.67E-15	3.71E-14
RAB3B	56353	54783	0.374387989	0	0
LCA5	571	555	0.374149158	1.22E-05	4.80E-05
PVR	6069	5895	0.373185124	6.87E-46	2.32E-44
ATP6V1B2	4495	4366	0.373143146	1.99E-34	4.74E-33
UBN2	2664	2587	0.372838103	4.88E-21	7.01E-20
GFPT1	13542	13144	0.372115693	1.51E-99	1.16E-97
RUNX1T1	576	559	0.371931603	1.27E-05	4.98E-05
ARHGAP24	1796	1741	0.370280985	1.70E-14	1.63E-13
FAM222B	1240	1202	0.370248907	1.85E-10	1.29E-09
CCND2	12048	11673	0.369533843	1.69E-87	1.17E-85
COL11A1	605	586	0.369117654	9.10E-06	3.66E-05
LNPEP	1640	1588	0.368667229	2.95E-13	2.60E-12
SLC30A1	1763	1707	0.368582715	3.93E-14	3.67E-13
PRKCI	7475	7233	0.367672704	2.05E-54	8.22E-53
FAM206A	829	802	0.367382267	2.36E-07	1.17E-06
ALCAM	34204	33068	0.366422872	1.61E-240	4.10E-238
CCBE1	5692	5502	0.36617261	1.65E-41	4.90E-40
SPATA7	479	463	0.366138669	9.06E-05	0.000304773
MICB	566	547	0.365890912	2.12E-05	8.04E-05
LGMN	1352	1305	0.364106787	6.24E-11	4.50E-10
NDUFA5	2564	2472	0.362434613	3.26E-19	4.29E-18
LRRC57	889	857	0.362263917	1.34E-07	6.90E-07
TDG	1937	1867	0.362050105	7.34E-15	7.23E-14
PTPRU	553	533	0.362008184	3.24E-05	0.000119406
IRS2	1125	1084	0.361591887	3.22E-09	1.97E-08
DOCK11	1596	1536	0.359869696	2.29E-12	1.89E-11

SFXN5	765	736	0.35939815	1.23E-06	5.60E-06
NKIRAS2	1892	1819	0.358385586	2.85E-14	2.69E-13
GOLGA7	2379	2287	0.358253226	1.54E-17	1.84E-16
AP4E1	2168	2084	0.358142653	4.12E-16	4.47E-15
SOCS2	2321	2231	0.358096123	3.90E-17	4.49E-16
LIN7C	4290	4123	0.357868945	2.82E-30	5.93E-29
LDLRAP1	1102	1059	0.357730497	6.93E-09	4.08E-08
ARHGAP5	6969	6695	0.357284532	5.94E-48	2.13E-46
C5orf51	2920	2803	0.356155507	6.26E-21	8.93E-20
PLAG1	2103	2018	0.355629456	1.83E-15	1.88E-14
FCHO2	1777	1705	0.35548019	2.74E-13	2.42E-12
IFFO2	1505	1444	0.355459387	1.77E-11	1.35E-10
AGTPBP1	567	544	0.355410048	3.68E-05	0.000133844
BCAP29	4173	4003	0.355148827	4.75E-29	9.57E-28
SETD7	11902	11416	0.355005349	1.50E-79	9.05E-78
TMEM67	511	490	0.35461059	9.30E-05	0.000312125
FAR2	630	604	0.354348853	1.45E-05	5.64E-05
GRPEL2	1134	1087	0.354083432	6.15E-09	3.65E-08
KIAA1919	578	554	0.353968615	3.35E-05	0.000123063
FHL2	5698	5461	0.353861667	9.24E-39	2.50E-37
RNF138	1055	1011	0.35369213	2.14E-08	1.20E-07
CUX1	6162	5904	0.353446171	1.12E-41	3.35E-40
ABAT	1336	1280	0.353375934	3.06E-10	2.07E-09
FGF5	18540	17760	0.353142469	1.63E-121	1.62E-119
ZMYM5	1209	1158	0.35297314	2.21E-09	1.37E-08
ODF2L	1817	1740	0.352681018	2.36E-13	2.09E-12
RASSF3	2414	2311	0.352243715	3.34E-17	3.87E-16
MAN1A2	9025	8638	0.351922516	1.10E-59	4.92E-58
TENM3	6195	5928	0.351593297	1.91E-41	5.67E-40
MB21D2	1020	976	0.351536032	4.48E-08	2.43E-07
C8orf42	635	607	0.350092056	1.73E-05	6.63E-05
TBC1D4	1950	1864	0.350079868	5.04E-14	4.69E-13

MBLAC2	657	628	0.35002332	1.24E-05	4.86E-05
SNX2	2484	2374	0.349806893	2.02E-17	2.39E-16
PLEKHF2	1956	1869	0.349512331	5.09E-14	4.73E-13
RCOR3	2129	2034	0.349295861	4.15E-15	4.18E-14
IQCE	1232	1177	0.349264196	2.36E-09	1.46E-08
ACTG2	6398	6111	0.34893962	4.30E-42	1.30E-40
DUSP16	846	808	0.348849761	7.74E-07	3.60E-06
JARID2	1192	1138	0.348268454	4.75E-09	2.86E-08
ZNF678	639	610	0.348145443	1.82E-05	6.97E-05
STAG2	6819	6506	0.347362769	2.45E-44	8.04E-43
FLT1	3025	2886	0.347288289	1.40E-20	1.96E-19
PPM1D	1282	1223	0.347180274	1.43E-09	9.03E-09
FBXO3	1687	1609	0.346856484	4.03E-12	3.25E-11
TRAPPC6B	2503	2387	0.346692407	3.05E-17	3.54E-16
PLD1	812	774	0.346005971	1.59E-06	7.12E-06
TMOD3	6574	6265	0.345695185	2.17E-42	6.63E-41
DSEL	16447	15670	0.345332857	4.23E-103	3.47E-101
DIXDC1	1374	1309	0.345235225	4.70E-10	3.11E-09
ANKRD42	630	600	0.344762804	2.54E-05	9.50E-05
SGPL1	2488	2369	0.344443845	6.19E-17	7.05E-16
STX17	1724	1641	0.343967596	3.65E-12	2.96E-11
XPA	638	607	0.343292224	2.45E-05	9.20E-05
BAIAP2L1	1254	1193	0.343208827	3.35E-09	2.05E-08
XPO4	3186	3031	0.343199717	4.27E-21	6.15E-20
ARL4C	2971	2826	0.342964992	9.40E-20	1.27E-18
C16orf52	2212	2103	0.342249596	4.85E-15	4.86E-14
C5orf15	5599	5323	0.342222518	1.26E-35	3.09E-34
ZCCHC3	1086	1032	0.341570999	4.37E-08	2.37E-07
SREK1IP1	2510	2385	0.341454034	8.71E-17	9.81E-16
NABP1	17610	16729	0.341108431	1.82E-107	1.56E-105
SLC8A1	2893	2748	0.340967813	4.66E-19	6.08E-18
WASF1	796	756	0.340769935	2.93E-06	1.27E-05

SPCS3	17482	16603	0.340725834	1.88E-106	1.61E-104
INO80D	1889	1794	0.34070932	5.93E-13	5.11E-12
IFT74	913	867	0.340569265	5.59E-07	2.65E-06
LOC646719	1245	1182	0.340236425	5.27E-09	3.16E-08
MMP14	31021	29430	0.33919448	1.23E-185	2.10E-183
CLDND1	2138	2028	0.338947931	2.53E-14	2.40E-13
ATG14	1784	1692	0.338766085	3.48E-12	2.83E-11
STAT5B	1390	1318	0.338417619	8.57E-10	5.53E-09
TBL1XR1	7137	6764	0.337711019	9.70E-44	3.07E-42
DNAJC24	743	704	0.33736535	7.83E-06	3.18E-05
KBTBD2	2250	2131	0.336757723	8.25E-15	8.08E-14
JMY	1605	1520	0.336650158	5.57E-11	4.04E-10
COL8A1	7091	6715	0.336550435	3.63E-43	1.13E-41
MAP1B	36779	34811	0.335813136	3.64E-215	8.41E-213
ATP6V1A	8158	7721	0.335724338	3.77E-49	1.37E-47
THAP6	1139	1077	0.334402635	4.18E-08	2.27E-07
MIER3	1664	1573	0.334015369	3.62E-11	2.68E-10
CLEC14A	2485	2349	0.333952993	6.10E-16	6.55E-15
HMGB2	2737	2587	0.333836751	2.17E-17	2.56E-16
TBCEL	1511	1428	0.333644451	2.98E-10	2.02E-09
PCDH7	1667	1575	0.333249856	3.87E-11	2.85E-10
JUNB	978	924	0.333210518	4.16E-07	2.01E-06
GLIS3	1010	954	0.33285801	2.78E-07	1.38E-06
TRAK2	7410	6997	0.332415081	6.52E-44	2.08E-42
DENND1B	1479	1396	0.331829021	5.75E-10	3.78E-09
ZNF175	797	752	0.331305069	5.58E-06	2.33E-05
TNIP1	4727	4460	0.331270977	1.97E-28	3.89E-27
SESN1	1485	1400	0.330116028	6.59E-10	4.30E-09
MIA3	6781	6391	0.329695775	1.15E-39	3.22E-38
IBTK	7562	7127	0.329679207	4.95E-44	1.59E-42
SNX13	3038	2861	0.32854976	1.51E-18	1.92E-17
ATP9A	6998	6589	0.328268994	1.59E-40	4.60E-39

STK40	1485	1398	0.328053561	8.52E-10	5.51E-09
MCFD2	33545	31578	0.327974189	6.04E-187	1.06E-184
PLEKHA6	1568	1476	0.327919294	2.95E-10	2.00E-09
COL5A1	34462	32432	0.327563695	1.68E-191	3.13E-189
KIAA1147	1103	1038	0.327525785	1.30E-07	6.71E-07
FN1	750913	706304	0.32679563	0	0
DAAM1	723	680	0.326691231	2.03E-05	7.72E-05
SPIRE1	2798	2631	0.326367418	5.50E-17	6.28E-16
ATP7B	451	424	0.326088963	0.0007808	0.002194364
RAP2C	3954	3717	0.325977949	2.68E-23	4.35E-22
NIPA1	1493	1403	0.325452975	1.06E-09	6.80E-09
WDR19	1026	964	0.325226452	4.35E-07	2.09E-06
STRN	2932	2754	0.324795588	1.46E-17	1.75E-16
NAA20	2979	2798	0.324719971	8.24E-18	1.01E-16
SLC2A10	3759	3530	0.3244714	4.99E-22	7.62E-21
C15orf57	689	647	0.324413861	3.63E-05	0.000132161
PRKCE	1066	1001	0.324386668	2.80E-07	1.38E-06
ATAD2B	917	861	0.324243636	1.92E-06	8.51E-06
RUNDC1	957	898	0.323348652	1.23E-06	5.60E-06
PPID	1957	1836	0.323074435	4.21E-12	3.40E-11
ATF7	1857	1742	0.32292294	1.51E-11	1.16E-10
AFF1	4201	3940	0.322614976	3.73E-24	6.28E-23
MINOS1	2800	2626	0.322592221	1.27E-16	1.41E-15
ITGA4	1728	1620	0.322042727	8.59E-11	6.12E-10
VHL	2709	2539	0.321652133	4.84E-16	5.23E-15
BBX	7071	6627	0.321593792	2.90E-39	7.99E-38
PDE1C	844	791	0.321586828	5.93E-06	2.46E-05
ST5	2499	2342	0.321542306	6.61E-15	6.54E-14
HSPA4L	666	624	0.321175984	5.88E-05	0.000205163
ATP10A	1583	1483	0.321009474	5.99E-10	3.93E-09
CPD	18455	17289	0.32099482	3.40E-99	2.58E-97
CCNH	1342	1257	0.32075211	1.23E-08	7.06E-08

SRGN	2556	2393	0.320084692	4.42E-15	4.44E-14
ADAM19	11940	11178	0.320011375	2.00E-64	9.75E-63
BCL6	1033	967	0.319899672	6.28E-07	2.95E-06
GMFB	10116	9469	0.319797169	8.73E-55	3.54E-53
LINC00657	39472	36938	0.319428265	7.78E-208	1.70E-205
RAB18	5899	5520	0.31934999	1.40E-32	3.20E-31
CEP135	1198	1121	0.319310502	8.54E-08	4.49E-07
TMEM209	1088	1018	0.319211137	3.36E-07	1.64E-06
PARP8	495	463	0.3187358	0.0005908	0.001702849
FNDC3B	12802	11974	0.318688093	2.32E-68	1.21E-66
SGMS2	1574	1472	0.318494262	9.25E-10	5.96E-09
ZCCHC10	709	663	0.318375375	4.01E-05	0.000144488
SPICE1	647	605	0.318321562	8.76E-05	0.000295559
SLC35A1	954	892	0.318206576	1.92E-06	8.51E-06
TANK	2231	2086	0.318200776	3.30E-13	2.89E-12
NME7	783	732	0.317983473	1.63E-05	6.28E-05
AGL	2056	1922	0.317920203	2.88E-12	2.36E-11
CCDC68	2464	2303	0.317664287	2.20E-14	2.09E-13
MRPS9	1131	1057	0.317528579	2.31E-07	1.15E-06
AP1S2	3981	3718	0.316548031	3.91E-22	6.00E-21
CCNT2	2760	2577	0.316176402	8.66E-16	9.18E-15
ZNF75D	527	492	0.316007485	0.0004429	0.00130645
PPP6C	3205	2992	0.31593795	4.64E-18	5.77E-17
FYN	1083	1011	0.315901886	4.79E-07	2.29E-06
OSMR	9426	8798	0.315682052	7.72E-50	2.85E-48
SLC35A3	2729	2547	0.315578697	1.43E-15	1.48E-14
GNG12	23421	21844	0.314586518	3.12E-120	3.01E-118
CBX6	3686	3437	0.314245912	2.54E-20	3.53E-19
PSKH1	1893	1765	0.314145904	3.66E-11	2.71E-10
GOLPH3L	1271	1185	0.314075161	5.94E-08	3.18E-07
SERINC5	1902	1772	0.313013489	3.91E-11	2.87E-10
GDAP2	1797	1674	0.312861251	1.37E-10	9.63E-10

PPP3R1	5315	4951	0.312802389	2.50E-28	4.92E-27
MITF	2941	2739	0.312494614	2.36E-16	2.60E-15
ACVR2A	524	488	0.312466468	0.0005367	0.001557095
MTF2	1145	1066	0.312011972	3.23E-07	1.58E-06
PAG1	1780	1657	0.311848493	1.93E-10	1.34E-09
PCGF5	3009	2801	0.311810008	1.26E-16	1.40E-15
LYST	1608	1496	0.3109949	1.60E-09	1.01E-08
PIK3R1	2315	2153	0.310488258	4.91E-13	4.25E-12
HELZ	5487	5103	0.310480198	9.21E-29	1.83E-27
HBP1	2147	1996	0.309941661	3.71E-12	3.01E-11
RRM2B	8470	7873	0.30970364	2.96E-43	9.27E-42
PURB	5918	5499	0.309211722	1.22E-30	2.61E-29
OMA1	494	459	0.309135244	0.0008892	0.002470953
SLC7A6	2865	2662	0.309127564	1.21E-15	1.27E-14
RAB9A	634	589	0.308936925	0.0001682	0.000536863
RRAS2	4705	4371	0.308920787	1.19E-24	2.05E-23
MOB1B	8685	8068	0.308837367	4.65E-44	1.51E-42
NEAT1	22796	21176	0.308801534	1.17E-112	1.03E-110
TWISTNB	2117	1966	0.308394184	6.76E-12	5.36E-11
RAPGEF2	5734	5323	0.307849793	1.77E-29	3.61E-28
HMGCR	2591	2405	0.30768001	3.63E-14	3.40E-13
ATF2	2713	2518	0.307541367	9.46E-15	9.22E-14
CPT2	958	889	0.307309895	4.24E-06	1.79E-05
ERCC6	929	862	0.307161404	5.99E-06	2.47E-05
VTA1	3807	3532	0.306982905	5.25E-20	7.17E-19
PCK2	884	820	0.306729672	1.04E-05	4.12E-05
SLC25A4	1022	948	0.3067159	2.13E-06	9.37E-06
PGRMC2	6088	5645	0.306157354	7.44E-31	1.60E-29
SLC38A9	797	739	0.306146772	2.94E-05	0.00010879
SIKE1	2577	2388	0.305262431	6.85E-14	6.31E-13
USP6NL	1021	946	0.305081354	2.45E-06	1.07E-05
TMEM87B	4219	3908	0.304681511	1.12E-21	1.67E-20

POLR3GL	1260	1167	0.304532959	1.75E-07	8.83E-07
ZNF260	2162	2002	0.304227583	8.11E-12	6.38E-11
ARMC9	2089	1934	0.303927434	1.90E-11	1.45E-10
ITGA3	19627	18170	0.303870879	4.40E-94	3.24E-92
C21orf91	1023	947	0.303782317	2.66E-06	1.16E-05
CLTCL1	753	697	0.303660923	5.66E-05	0.000198005
RNF141	3705	3429	0.303466493	4.41E-19	5.77E-18
SCOC	5402	4998	0.303009393	5.24E-27	9.85E-26
PERP	13075	12097	0.302990495	6.42E-63	2.99E-61
MOAP1	909	841	0.302977638	1.02E-05	4.05E-05
TBCCD1	519	480	0.302451999	0.0008716	0.00242761
ANKRD33B	1796	1661	0.302416855	5.94E-10	3.89E-09
PANK3	2362	2184	0.302116023	1.31E-12	1.09E-11
NFIL3	1232	1139	0.301917623	3.06E-07	1.51E-06
XIAP	5564	5143	0.301639866	1.61E-27	3.08E-26
CELSR1	515	476	0.301541273	0.0009477	0.002616514
LACC1	804	743	0.301318841	3.67E-05	0.000133611
ANO6	9188	8490	0.301165829	3.35E-44	1.09E-42
RREB1	1952	1803	0.300598476	1.41E-10	9.92E-10
SPARC	150650	139121	0.300291667	0	0
ANGPT1	3131	2891	0.300097232	5.07E-16	5.47E-15
SEC24A	6964	6430	0.300054666	1.14E-33	2.69E-32
AGPAT4	638	589	0.299863342	0.0002545	0.000784693
SYNJ2BP	3312	3057	0.299566011	8.40E-17	9.49E-16
CCDC91	1025	946	0.299440311	3.67E-06	1.56E-05
SLC7A1	4121	3802	0.2989162	1.94E-20	2.71E-19
HIVEP2	2322	2142	0.29874264	3.63E-12	2.95E-11
IL1RAP	937	864	0.298134396	1.05E-05	4.18E-05
CEP85	526	485	0.29807408	0.0009657	0.002661522
FAM179B	1305	1203	0.297738967	2.08E-07	1.04E-06
VAPB	3477	3205	0.297633422	2.40E-17	2.82E-16
TMEM87A	2987	2753	0.297459504	4.23E-15	4.26E-14

PSG5	7235	6668	0.297413219	2.67E-34	6.35E-33
ZNF25	1199	1105	0.297366843	6.68E-07	3.13E-06
VPS4B	4436	4088	0.297287963	1.20E-21	1.79E-20
TMEM117	573	528	0.297154922	0.0005959	0.001715288
ZSWIM4	533	491	0.296739623	0.0009444	0.002609202
AP1AR	783	721	0.296139084	6.36E-05	0.000220494
SPTY2D1	2520	2320	0.295853203	7.64E-13	6.53E-12
PAIP2	2142	1972	0.295853203	3.90E-11	2.87E-10
CCNDBP1	1334	1228	0.295704026	1.87E-07	9.41E-07
FERMT2	6676	6144	0.295346393	2.42E-31	5.33E-30
OSBPL9	2531	2329	0.295155264	7.77E-13	6.62E-12
RNF11	9334	8589	0.295146836	4.40E-43	1.37E-41
B4GALT1	5615	5166	0.294913753	1.51E-26	2.79E-25
PLEKHA3	1270	1168	0.294363909	4.17E-07	2.01E-06
NID2	12468	11466	0.294284256	1.29E-56	5.46E-55
UBE2D1	1368	1258	0.294215824	1.52E-07	7.76E-07
NUPR1	1106	1017	0.294120425	2.37E-06	1.04E-05
PPP1R2	3391	3118	0.294062275	1.44E-16	1.59E-15
ERCC8	631	580	0.293565027	0.0003747	0.001122292
PLXNA2	778	715	0.293325218	7.93E-05	0.000269955
CEP112	690	634	0.29303861	0.0002049	0.000642825
EXTL2	2671	2454	0.292907405	2.84E-13	2.50E-12
KIF1B	7742	7113	0.292903988	1.75E-35	4.27E-34
SRFBP1	713	655	0.292744962	0.0001631	0.000522058
SSR3	27421	25184	0.292378393	1.15E-120	1.14E-118
NPAS2	674	619	0.29234295	0.0002518	0.000777558
NCOA3	3798	3487	0.29189859	4.11E-18	5.13E-17
MYOF	43784	40194	0.291728528	1.21E-190	2.18E-188
AP3M2	2154	1977	0.291446753	6.90E-11	4.95E-10
ANKRD13C	2414	2215	0.291033154	5.39E-12	4.30E-11
CDV3	15365	14097	0.290892538	1.00E-67	5.16E-66
KDM1B	1424	1306	0.290357882	1.27E-07	6.57E-07

OXR1	3189	2923	0.289497863	3.25E-15	3.29E-14
GFRA1	2000	1833	0.289358918	4.43E-10	2.94E-09
ZNF254	778	713	0.289284053	0.0001004	0.000335125
UBE2V2	3266	2993	0.28921962	1.62E-15	1.68E-14
ATP1B1	2464	2258	0.289195362	4.52E-12	3.63E-11
SLC31A1	4463	4087	0.288180569	1.71E-20	2.40E-19
ABCA1	7714	7064	0.288158329	3.17E-34	7.53E-33
HSD17B12	5144	4710	0.287988549	2.40E-23	3.92E-22
GULP1	2891	2647	0.287941718	8.56E-14	7.82E-13
LYSMD3	3746	3429	0.287589136	2.20E-17	2.60E-16
OXCT1	3707	3393	0.287461447	3.33E-17	3.87E-16
SECISBP2L	8248	7549	0.287393345	2.78E-36	6.90E-35
ARL5A	3244	2969	0.287355406	3.10E-15	3.15E-14
WNT5A	24111	22065	0.287220391	1.69E-102	1.35E-100
TLE4	2071	1895	0.287022427	3.09E-10	2.09E-09
LIFR	4465	4084	0.286474823	2.88E-20	3.98E-19
CBFB	4474	4092	0.28639302	2.71E-20	3.77E-19
SELK	1159	1060	0.28633583	2.65E-06	1.15E-05
NSD1	6656	6086	0.285991032	2.55E-29	5.17E-28
TRAPPC2	720	658	0.285242809	0.000227	0.0007062
PAN3	2357	2154	0.285218623	2.55E-11	1.92E-10
AKIRIN1	6556	5990	0.28489228	1.10E-28	2.19E-27
SLC30A4	648	592	0.284755494	0.0004805	0.001407799
OTUD3	1537	1404	0.284577902	7.72E-08	4.08E-07
MESDC2	3481	3179	0.284223334	6.66E-16	7.12E-15
NEK7	9207	8408	0.284183655	2.10E-39	5.85E-38
UBE2E1	3812	3481	0.284105827	2.96E-17	3.45E-16
IFRD1	2038	1861	0.284076136	6.56E-10	4.29E-09
GGCT	942	860	0.283761732	2.74E-05	0.000102067
SHOC2	3687	3366	0.283739892	1.07E-16	1.19E-15
XPR1	5080	4637	0.283515361	2.21E-22	3.43E-21
PPME1	2332	2127	0.282404377	5.17E-11	3.76E-10

PRSS23	14517	13237	0.281984977	3.74E-60	1.68E-58
RAD23B	15875	14475	0.281958888	1.34E-65	6.58E-64
C2orf69	1727	1574	0.28131959	1.83E-08	1.03E-07
PTBP3	3891	3546	0.281203688	3.07E-17	3.57E-16
PPT1	2469	2250	0.281150297	1.76E-11	1.34E-10
HECTD2	834	760	0.281104167	9.32E-05	0.000312448
DSTN	19203	17499	0.281092897	1.81E-78	1.08E-76
ZNF697	1798	1638	0.280694468	1.01E-08	5.84E-08
TRUB1	1966	1791	0.280654147	2.10E-09	1.31E-08
TPST2	1347	1227	0.28053753	7.21E-07	3.36E-06
HDHD2	1435	1307	0.280360536	3.19E-07	1.57E-06
POLR3G	784	714	0.280222552	0.0001592	0.000511327
RAI14	32364	29474	0.28020507	4.15E-130	4.50E-128
MOK	1029	937	0.280030103	1.54E-05	5.95E-05
GCFC2	1036	943	0.279457805	1.50E-05	5.82E-05
TAF8	724	659	0.2794409	0.0002966	0.0009054
TAPT1	891	811	0.279428614	5.98E-05	0.000208206
MSI2	946	861	0.279325186	3.56E-05	0.000130001
MTR	3692	3360	0.279210812	3.24E-16	3.53E-15
PPARA	1456	1325	0.279134136	2.97E-07	1.46E-06
BET1	1689	1537	0.279099969	3.40E-08	1.87E-07
ARL13B	731	665	0.278635066	0.0002893	0.000884588
RP2	2868	2609	0.278604052	7.02E-13	6.01E-12
LRRFIP1	7994	7272	0.278586759	4.24E-33	9.79E-32
ANAPC16	1771	1611	0.278544414	1.70E-08	9.63E-08
TMEM245	8023	7298	0.278511485	3.40E-33	7.91E-32
PHF17	1604	1459	0.278457873	8.06E-08	4.24E-07
ZNF542	763	694	0.278404737	0.0002156	0.000674021
HECA	1171	1065	0.278264486	4.62E-06	1.94E-05
DDIT4	1236	1124	0.278115424	2.55E-06	1.11E-05
TMEM165	4785	4351	0.277980224	2.22E-20	3.09E-19
TMOD2	3393	3085	0.277861191	6.88E-15	6.79E-14

ZFYVE9	2566	2333	0.277817269	1.28E-11	9.92E-11
COQ10B	2641	2401	0.277703234	6.62E-12	5.26E-11
CMPK1	9957	9051	0.277518199	1.71E-40	4.91E-39
KDM4B	2758	2507	0.277491671	2.37E-12	1.96E-11
KIAA1737	636	578	0.277194859	0.0007719	0.002173188
DCAF12	1465	1331	0.276762038	3.48E-07	1.70E-06
TCP11L1	1431	1300	0.276640083	4.82E-07	2.30E-06
ATAD1	3000	2725	0.276445861	3.27E-13	2.86E-12
TSPAN5	5523	5016	0.276237352	5.40E-23	8.70E-22
LBH	5188	4711	0.276006984	1.16E-21	1.73E-20
GLIPR1	10267	9323	0.276003614	3.03E-41	8.93E-40
SLC16A1	4088	3712	0.275953646	2.15E-17	2.54E-16
NUAK1	1245	1130	0.275329162	2.99E-06	1.29E-05
ERRFI1	2522	2289	0.275301319	2.96E-11	2.21E-10
GATAD2B	1664	1510	0.275045248	6.85E-08	3.64E-07
ZBTB34	788	715	0.274899744	0.0002068	0.000648205
HEATR5B	2018	1831	0.274857748	2.90E-09	1.78E-08
C6orf120	3451	3131	0.274761166	8.41E-15	8.22E-14
HMGA2	33016	29946	0.274350098	4.95E-127	5.14E-125
KIF13A	6096	5529	0.274307801	7.22E-25	1.25E-23
DDX60L	1127	1022	0.274059813	9.72E-06	3.88E-05
ZNF627	868	787	0.273820725	0.0001052	0.000349795
EFCAB14	6163	5587	0.273593161	5.38E-25	9.43E-24
RNF6	3755	3404	0.273570261	7.69E-16	8.18E-15
YARS	6210	5629	0.273437512	3.81E-25	6.70E-24
SSFA2	20050	18171	0.273187712	3.30E-77	1.90E-75
FAM212B	1277	1157	0.272782471	2.79E-06	1.21E-05
FBXL2	947	858	0.272765354	5.47E-05	0.000191928
CLCN5	808	732	0.272640487	0.0001953	0.000614019
PPM1B	2251	2039	0.27244785	5.20E-10	3.43E-09
LMBR1L	1082	980	0.272305287	1.67E-05	6.43E-05
RRAGB	699	633	0.272065176	0.0005463	0.001581896

ATP2B4	68394	61931	0.271944106	2.08E-256	6.46E-254
P4HA1	14829	13427	0.271867818	5.15E-57	2.20E-55
CSRP2	1501	1359	0.271773611	4.19E-07	2.02E-06
NEXN	8639	7821	0.271640891	6.94E-34	1.63E-32
ABI2	5575	5047	0.271606413	1.91E-22	2.98E-21
IKZF5	1023	926	0.271430085	3.02E-05	0.000111702
ITGAV	17944	16242	0.2713799	2.33E-68	1.21E-66
MCCC2	1792	1622	0.271355314	3.39E-08	1.86E-07
GPC4	642	581	0.271116998	0.0009636	0.002657011
MYL12A	18561	16797	0.271081278	1.73E-70	9.23E-69
CALCOCO2	3902	3531	0.271015175	4.12E-16	4.47E-15
IER3	1859	1682	0.270803067	2.02E-08	1.13E-07
FBXW7	1206	1091	0.270573326	6.34E-06	2.61E-05
MAMDC2	1153	1043	0.270498777	1.02E-05	4.06E-05
GABARAPL2	3574	3233	0.270486005	7.87E-15	7.74E-14
PDCD10	1781	1611	0.270421109	4.18E-08	2.27E-07
SLC38A6	932	843	0.270354808	7.32E-05	0.000251217
CD164	20814	18826	0.270324385	2.26E-78	1.33E-76
KIAA1609	3292	2977	0.270047018	9.76E-14	8.88E-13
RBM7	1891	1710	0.269999094	1.69E-08	9.58E-08
LRIG3	720	651	0.269812769	0.0005049	0.001473538
OGT	6470	5849	0.269576409	2.07E-25	3.68E-24
CCP110	1518	1372	0.269260823	4.67E-07	2.23E-06
R3HCC1L	976	882	0.26904964	5.40E-05	0.000189827
WWC2	4152	3752	0.269005516	8.33E-17	9.41E-16
TMED5	8032	7257	0.268766132	5.80E-31	1.26E-29
DIRC2	1014	916	0.268513983	4.01E-05	0.000144611
RASSF8	4189	3784	0.268458336	7.08E-17	8.04E-16
H3F3B	13310	12022	0.268318486	5.18E-50	1.92E-48
ZBTB7B	1219	1101	0.268268475	6.84E-06	2.80E-05
UTRN	9925	8964	0.268227678	1.04E-37	2.74E-36
TMEM47	10266	9270	0.26791921	7.40E-39	2.01E-37

KIAA1432	5153	4649	0.266659965	3.87E-20	5.31E-19
ABI1	3509	3165	0.266297684	3.62E-14	3.40E-13
BTG3	1968	1775	0.266240936	1.42E-08	8.09E-08
TBC1D12	958	864	0.266157788	7.65E-05	0.000261346
DNM3OS	1007	908	0.265852651	5.12E-05	0.000180569
NAPG	2457	2215	0.265560973	2.63E-10	1.79E-09
SUV420H1	2305	2077	0.264886597	1.03E-09	6.62E-09
UNKL	1208	1088	0.264210234	1.05E-05	4.16E-05
CHRM2	8306	7480	0.26403653	7.39E-31	1.60E-29
ARF3	11477	10335	0.263944939	5.85E-42	1.77E-40
CDC40	2060	1855	0.263926981	8.97E-09	5.21E-08
PRDM2	2355	2120	0.263489337	8.45E-10	5.47E-09
MAN2A1	9601	8642	0.263332684	3.22E-35	7.81E-34
GLS	9865	8879	0.263230283	4.12E-36	1.02E-34
GOLGA8B	1229	1106	0.263018602	9.66E-06	3.86E-05
TOB1	2495	2245	0.262827761	2.99E-10	2.02E-09
LOC100507217	1117	1005	0.262718447	2.52E-05	9.44E-05
FEM1C	2712	2440	0.262676101	5.25E-11	3.82E-10
MTX2	1235	1111	0.262499907	9.59E-06	3.84E-05
YRDC	726	653	0.262265575	0.0006978	0.001981035
SOCS7	1191	1071	0.261937199	1.45E-05	5.62E-05
ATF7IP	3760	3381	0.261869486	1.32E-14	1.28E-13
TM9SF2	11129	10006	0.261693525	4.78E-40	1.36E-38
BTG2	1940	1744	0.261495519	3.29E-08	1.81E-07
PSEN1	3956	3555	0.260959265	3.45E-15	3.49E-14
PTAR1	10123	9095	0.260660772	2.70E-36	6.72E-35
N4BP1	2747	2468	0.260637616	5.66E-11	4.10E-10
GPATCH2	776	697	0.260254135	0.0005067	0.001478108
RBMS3	2865	2573	0.26006845	2.45E-11	1.84E-10
ZNF451	3836	3445	0.260053394	1.12E-14	1.08E-13
APPBP2	5006	4495	0.259814956	1.18E-18	1.52E-17
WNT5B	5807	5214	0.259749681	2.24E-21	3.29E-20

LOC541471	2612	2345	0.259585158	1.99E-10	1.38E-09
DNAJA1	11278	10125	0.259562792	6.67E-40	1.89E-38
STX2	3318	2978	0.259181999	8.11E-13	6.90E-12
HS2ST1	4284	3845	0.25917725	4.12E-16	4.46E-15
COL4A2	58907	52870	0.259162377	4.13E-200	8.31E-198
METTL15	1687	1514	0.259057363	3.36E-07	1.64E-06
PWWP2A	1257	1128	0.25893455	1.07E-05	4.25E-05
SLC20A2	1617	1451	0.258879972	5.98E-07	2.82E-06
SDE2	1753	1573	0.258844806	2.03E-07	1.02E-06
C21orf59	1305	1171	0.258843401	7.34E-06	2.99E-05
MEF2A	3153	2829	0.258719138	3.26E-12	2.66E-11
RAD1	1985	1780	0.257890366	3.62E-08	1.98E-07
LOX	38422	34451	0.257765374	1.01E-129	1.08E-127
IFT88	879	788	0.257484596	0.0002526	0.000779523
ERGIC2	4179	3746	0.257345273	1.52E-15	1.58E-14
EOGT	2294	2056	0.257127005	3.56E-09	2.17E-08
RANBP6	4027	3608	0.256645998	5.87E-15	5.86E-14
PTPN4	1373	1230	0.256478822	5.23E-06	2.19E-05
DLC1	17313	15509	0.256402061	7.81E-59	3.43E-57
FSTL1	58769	52644	0.256365894	3.23E-195	6.28E-193
KDM5A	5359	4799	0.255922129	2.73E-19	3.61E-18
KCTD21	1136	1017	0.255508976	3.67E-05	0.0001336
HDDC2	1412	1264	0.255408507	4.24E-06	1.79E-05
TTC21B	1220	1092	0.25524384	1.94E-05	7.38E-05
AASDHPPT	3523	3153	0.255072827	4.02E-13	3.50E-12
GNAQ	2206	1974	0.254841331	9.72E-09	5.63E-08
XRN1	5568	4982	0.254717942	8.41E-20	1.14E-18
WNK4	969	867	0.25468746	0.0001454	0.00046949
FBXL20	1736	1553	0.254443014	3.78E-07	1.84E-06
ALPK2	5952	5324	0.254288177	5.45E-21	7.80E-20
PLOD2	12480	11161	0.254000493	4.21E-42	1.28E-40
MRPS35	3100	2772	0.253811174	1.28E-11	9.91E-11

MECOM	1814	1622	0.253751495	2.25E-07	1.12E-06
ZNF780A	1245	1113	0.253459982	1.83E-05	7.01E-05
TTC13	962	860	0.253451898	0.0001659	0.000530112
TVP23B	1921	1717	0.253184652	1.06E-07	5.52E-07
TSPYL4	3108	2777	0.252692805	1.50E-11	1.15E-10
EXOC2	2976	2659	0.252661382	4.05E-11	2.97E-10
PPP1R12B	1698	1517	0.252536758	6.22E-07	2.92E-06
CREB1	3504	3130	0.252312014	8.45E-13	7.16E-12
BCKDHB	993	887	0.252292519	0.0001402	0.00045459
TMTC3	6248	5581	0.252281325	1.28E-21	1.90E-20
SNAPC1	1489	1330	0.252234624	3.14E-06	1.35E-05
RNF185	2725	2434	0.25222507	2.86E-10	1.94E-09
ZNF714	1011	903	0.252167027	0.0001229	0.000403261
PPIC	1226	1095	0.252124023	2.36E-05	8.87E-05
ATXN1	1656	1479	0.252071511	8.97E-07	4.15E-06
FNBP1L	1319	1178	0.252047106	1.16E-05	4.59E-05
CD2AP	4128	3686	0.251765232	9.40E-15	9.17E-14
AJUBA	3818	3409	0.251683628	9.48E-14	8.64E-13
ZNF469	6615	5906	0.251592332	1.12E-22	1.77E-21
CDC42SE1	4001	3570	0.250715577	3.11E-14	2.93E-13
RWDD2B	1252	1117	0.250546755	2.19E-05	8.30E-05
TANC2	6083	5427	0.250524036	8.27E-21	1.17E-19
MRPL39	788	703	0.250481191	0.0007608	0.002144914
AHR	4327	3860	0.250385858	3.11E-15	3.16E-14
CYB5R4	1259	1123	0.250231776	2.13E-05	8.07E-05
DUSP11	1267	1130	0.25005838	2.03E-05	7.73E-05
DCAF10	3819	3406	0.250035647	1.38E-13	1.25E-12
C10orf12	878	783	0.2499435	0.0003915	0.001166448
MGLL	7683	6851	0.249796963	1.03E-25	1.86E-24
ZFP36L1	7059	6294	0.249665489	9.87E-24	1.63E-22
SERINC1	23776	21199	0.249642322	7.16E-76	4.07E-74
ZDBF2	1363	1215	0.249322883	1.05E-05	4.17E-05

CRIPT	874	779	0.24914218	0.0004221	0.001249717
RFK	2666	2376	0.249010187	7.52E-10	4.89E-09
APOOL	1406	1253	0.248941952	7.89E-06	3.20E-05
LACTB	1532	1365	0.248636786	3.19E-06	1.37E-05
EIF1AD	1053	938	0.248306523	0.000115	0.000378986
FOXD1	3232	2878	0.247791526	1.56E-11	1.20E-10
ATF4	8490	7558	0.247392097	1.04E-27	2.00E-26
ZNF268	1585	1411	0.247387279	2.44E-06	1.07E-05
FOSL2	2012	1791	0.247287164	1.11E-07	5.75E-07
SOCS4	3657	3255	0.247149048	8.53E-13	7.22E-12
CASS4	936	833	0.246960098	0.0002998	0.0009144
KLF7	2235	1989	0.246920577	2.32E-08	1.30E-07
CSNK1G1	2577	2293	0.246695949	2.07E-09	1.29E-08
IER5	3080	2740	0.246397674	6.03E-11	4.36E-10
PPP1R3C	1466	1304	0.246210898	6.47E-06	2.66E-05
ARRDC3	3498	3111	0.246000237	3.37E-12	2.75E-11
WTIP	1038	923	0.245748241	0.0001519	0.000489383
ARHGAP29	3473	3088	0.245642474	4.34E-12	3.49E-11
TMEM128	911	810	0.245622986	0.0003901	0.001163724
MED13L	7041	6259	0.245303965	7.03E-23	1.13E-21
CTHRC1	9670	8595	0.245133882	9.07E-31	1.95E-29
LRRC58	4704	4181	0.245112115	8.83E-16	9.35E-15
CYB561D1	997	886	0.244865326	0.000217	0.000677076
SRF	2676	2378	0.244822731	1.38E-09	8.75E-09
PROSC	1434	1274	0.244472385	9.51E-06	3.81E-05
RALGAPA1	1826	1622	0.244239186	5.98E-07	2.82E-06
ZKSCAN2	840	746	0.243938434	0.0007213	0.002042313
TOMM20	13370	11871	0.243594137	2.28E-41	6.76E-40
RNF13	1523	1352	0.243331342	5.59E-06	2.33E-05
MARCKS	12184	10815	0.24319795	9.95E-38	2.63E-36
PIGM	1130	1003	0.243150965	9.28E-05	0.000311423
BTBD1	9013	8000	0.243144741	2.47E-28	4.85E-27

ZFAND1	1013	899	0.242910978	0.0002182	0.000680606
GDE1	5518	4895	0.242315535	7.51E-18	9.19E-17
H1F0	3559	3157	0.24223446	4.86E-12	3.89E-11
RNF170	1876	1664	0.242147737	5.31E-07	2.53E-06
MGEA5	9866	8751	0.242134731	1.32E-30	2.80E-29
GOLT1B	6780	6013	0.241951817	1.64E-21	2.43E-20
GFM2	1307	1159	0.241773557	2.93E-05	0.000108646
TFAM	1633	1448	0.241688943	3.02E-06	1.31E-05
MEIS2	2325	2061	0.24126592	2.68E-08	1.48E-07
ZBTB41	2623	2325	0.24116504	3.54E-09	2.16E-08
ETS1	4153	3681	0.24109601	1.11E-13	1.00E-12
ZNF791	1169	1036	0.240901205	8.24E-05	0.000279458
BACH1	4515	4001	0.240786773	1.04E-14	1.01E-13
USP38	2450	2171	0.240730108	1.23E-08	7.03E-08
CAV2	11358	10064	0.240647142	1.48E-34	3.54E-33
ZDHHC17	2571	2278	0.240590269	5.50E-09	3.28E-08
RBMS1	5773	5115	0.240565146	2.39E-18	3.02E-17
GDF6	894	792	0.240377731	0.0005919	0.001705723
SDC4	8815	7808	0.240144615	4.41E-27	8.31E-26
SRD5A3	1208	1070	0.240142474	6.63E-05	0.000229033
FAM122A	1092	967	0.23976707	0.0001527	0.000491379
MRPS36	952	843	0.239723189	0.0004079	0.001210426
MRPL42	3036	2688	0.239513479	2.84E-10	1.93E-09
SLC35F5	5382	4765	0.239485955	4.59E-17	5.27E-16
RNF44	1089	964	0.239253229	0.0001613	0.000516922
HEATR2	1315	1164	0.23918039	3.40E-05	0.000124673
ADAM17	1757	1555	0.238952521	1.70E-06	7.58E-06
ZNF644	2875	2544	0.238688846	9.62E-10	6.19E-09
PARD3B	1656	1465	0.238350124	3.58E-06	1.53E-05
KHNYN	2682	2372	0.237946904	3.92E-09	2.38E-08
PIP4K2C	1297	1147	0.237839044	4.27E-05	0.000153231
CUL3	6360	5624	0.237721961	1.33E-19	1.78E-18

C7orf73	2762	2442	0.237502012	2.47E-09	1.53E-08
ALG9	1786	1579	0.237431222	1.64E-06	7.33E-06
RYK	4605	4071	0.237334197	1.42E-14	1.37E-13
TMEM123	12743	11262	0.236910226	2.22E-37	5.73E-36
LINC00294	1170	1034	0.236879788	0.0001086	0.000360057
EIF2S2	10004	8840	0.236693444	1.18E-29	2.42E-28
PPM1K	1047	925	0.23641596	0.0002583	0.000795949
BTF3L4	3251	2872	0.236324325	1.23E-10	8.64E-10
DCP1A	1969	1739	0.235946953	5.72E-07	2.70E-06
DPY19L3	4211	3719	0.235904004	2.64E-13	2.34E-12
ZBTB6	1222	1079	0.235602711	8.38E-05	0.000283477
TRAK1	4190	3699	0.235337188	3.46E-13	3.03E-12
SFXN1	4255	3756	0.235190063	2.36E-13	2.10E-12
PCNXL4	6493	5731	0.235053819	1.49E-19	1.99E-18
MTF1	1883	1662	0.235039514	1.11E-06	5.09E-06
ZNF320	952	840	0.234579886	0.0005482	0.001587247
DYNC2H1	4089	3607	0.234203492	8.63E-13	7.30E-12
PARP14	2348	2071	0.234047277	6.13E-08	3.28E-07
BLZF1	1644	1450	0.233994733	5.90E-06	2.45E-05
MTMR10	1449	1278	0.233982373	2.11E-05	8.03E-05
MET	3911	3449	0.233792724	2.94E-12	2.41E-11
LIPA	2547	2246	0.2337111	1.79E-08	1.01E-07
BVES	1167	1029	0.233590553	0.0001392	0.000451768
RAB11FIP2	2193	1933	0.233087957	1.88E-07	9.47E-07
TXNIP	7040	6205	0.233007913	1.03E-20	1.45E-19
UBE2G1	4549	4009	0.232845115	6.54E-14	6.03E-13
PIK3C3	3024	2665	0.232829525	9.85E-10	6.33E-09
COG2	1010	890	0.23267408	0.0004161	0.00123399
RAPH1	3221	2838	0.23251806	3.00E-10	2.03E-09
C11orf95	2948	2597	0.232261621	1.75E-09	1.10E-08
SLC39A14	4284	3773	0.231905752	4.34E-13	3.77E-12
ERBB2IP	9798	8628	0.231691017	6.92E-28	1.35E-26

CASC4	11589	10205	0.231672231	1.13E-32	2.58E-31
ADAM9	51722	45545	0.23166675	1.14E-139	1.36E-137
PLS3	19922	17536	0.231109346	1.17E-54	4.74E-53
SATB2	1411	1242	0.231099317	3.43E-05	0.000125783
LMBRD1	1767	1555	0.230764672	3.68E-06	1.57E-05
GARS	16063	14134	0.230580583	3.30E-44	1.07E-42
PPCS	1090	959	0.230426717	0.0002835	0.000868377
DSCR3	2368	2083	0.23014589	9.11E-08	4.77E-07
FAM49B	1693	1489	0.229913912	6.38E-06	2.62E-05
ZNF302	1294	1138	0.229815072	8.01E-05	0.000272444
CYB5D1	1219	1072	0.229758912	0.0001296	0.000422967
PTPN2	1144	1006	0.229695385	0.0002101	0.000657622
EXOC5	7982	7019	0.229667357	1.24E-22	1.95E-21
E2F3	2295	2018	0.229584153	1.54E-07	7.87E-07
TAGLN	39986	35159	0.229551201	2.18E-106	1.85E-104
PLAGL2	1325	1165	0.229489727	6.74E-05	0.000232509
MYOCD	1101	968	0.229416615	0.0002817	0.000863079
RCAN1	5736	5043	0.229389333	1.15E-16	1.28E-15
CHMP2B	2209	1942	0.229301914	2.73E-07	1.35E-06
TBC1D8B	2769	2434	0.229116246	8.85E-09	5.15E-08
DCTN6	1594	1401	0.228957458	1.30E-05	5.06E-05
TFDP2	2079	1827	0.228739008	6.52E-07	3.06E-06
RB1	4436	3898	0.228626857	3.77E-13	3.29E-12
UBE2J1	5661	4974	0.228501716	2.38E-16	2.62E-15
TMTC1	11477	10083	0.228331498	1.84E-31	4.07E-30
EDIL3	9204	8086	0.228317352	1.49E-25	2.66E-24
DUSP3	7508	6596	0.22831488	3.85E-21	5.57E-20
CDC42BPA	8363	7346	0.228090465	2.54E-23	4.12E-22
KIAA0226	1809	1589	0.228078848	3.71E-06	1.58E-05
FBXO11	4546	3993	0.22802752	2.25E-13	2.00E-12
PPP3CC	1008	885	0.227405853	0.000575	0.001660719
WIPF1	1530	1343	0.227079784	2.28E-05	8.60E-05

DICER1	5321	4670	0.226877278	2.97E-15	3.02E-14
MIER1	3356	2945	0.22667705	3.81E-10	2.54E-09
COL5A2	47520	41700	0.226664679	7.37E-123	7.52E-121
PPP1R15B	3011	2642	0.22653989	3.08E-09	1.89E-08
RHOB	4290	3763	0.226057773	1.67E-12	1.39E-11
CD46	11165	9789	0.225402274	6.96E-30	1.44E-28
ZFYVE16	3772	3307	0.225345501	4.16E-11	3.05E-10
FAP	6156	5397	0.225316587	3.51E-17	4.06E-16
SLMO2	1896	1662	0.22511355	2.97E-06	1.29E-05
CCL2	948	831	0.22511355	0.0009527	0.002629195
ZSCAN29	1223	1072	0.225032634	0.0001758	0.000558752
FAM200B	1198	1050	0.224913552	0.0002065	0.000647349
RFWD2	2145	1880	0.224907146	6.85E-07	3.21E-06
APBB2	3641	3191	0.224826	1.00E-10	7.08E-10
HOXB2	1884	1651	0.224693287	3.34E-06	1.43E-05
SFXN3	3363	2947	0.224650413	5.29E-10	3.49E-09
MTRR	1613	1413	0.224167159	1.78E-05	6.81E-05
PLAGL1	2121	1858	0.224158013	8.63E-07	4.00E-06
RNF121	1016	890	0.224128971	0.0006615	0.001884515
N4BP2L2	3514	3078	0.224031172	2.45E-10	1.68E-09
MACF1	34029	29803	0.223844937	2.78E-86	1.89E-84
KIAA1033	6538	5726	0.223830425	6.34E-18	7.78E-17
MYO5A	10350	9064	0.22374113	2.01E-27	3.83E-26
CAP2	4672	4091	0.223565395	3.27E-13	2.86E-12
HLA-B	6682	5850	0.223308774	3.33E-18	4.19E-17
UBE2B	3180	2784	0.223284577	1.97E-09	1.23E-08
NOTCH2NL	2087	1827	0.223198165	1.18E-06	5.37E-06
CPS1	995	871	0.223128325	0.0007964	0.002233959
GNB4	7724	6761	0.223040615	9.47E-21	1.34E-19
NPAT	1339	1172	0.222968741	0.0001011	0.000337031
SCFD1	5082	4448	0.222912544	3.65E-14	3.42E-13
GID4	1114	975	0.222877023	0.0003929	0.001170407

TTC19	1534	1342	0.22223832	3.36E-05	0.000123464
FAM134C	2366	2069	0.221635703	2.80E-07	1.38E-06
ARMC10	1585	1386	0.221596549	2.63E-05	9.85E-05
SPTLC2	5765	5041	0.221541478	1.11E-15	1.16E-14
RIT1	2077	1816	0.221415118	1.53E-06	6.88E-06
FAM129A	3183	2783	0.221405883	2.66E-09	1.64E-08
SH3BP5L	2211	1933	0.221294744	7.15E-07	3.34E-06
ZNF609	4394	3841	0.221099226	2.91E-12	2.38E-11
DBT	2137	1868	0.221059679	1.13E-06	5.14E-06
LEPROTL1	2836	2479	0.221052871	2.05E-08	1.15E-07
FGF2	9073	7930	0.220893339	1.20E-23	1.97E-22
SPG20	4053	3542	0.220726168	2.17E-11	1.64E-10
EXOC8	1427	1247	0.220628262	7.19E-05	0.000246806
RUNX1	5906	5161	0.220621406	6.66E-16	7.12E-15
ADIPOR1	4379	3826	0.220387556	3.74E-12	3.03E-11
CARD8	1013	885	0.220267318	0.0008402	0.002347076
ZBTB18	1013	885	0.220267318	0.0008402	0.002347076
MTHFR	1377	1203	0.220260215	9.90E-05	0.000330514
RGMB	22192	19387	0.220202013	4.70E-55	1.94E-53
VDR	1068	933	0.220189471	0.0006094	0.00174842
CLASP2	3051	2665	0.220005485	7.12E-09	4.19E-08
CCNG1	17676	15435	0.219565778	5.91E-44	1.89E-42
NBR1	13211	11536	0.219555527	2.79E-33	6.52E-32
MLL3	5982	5222	0.219126679	6.95E-16	7.43E-15
PIP4K2A	2289	1998	0.218991252	6.06E-07	2.85E-06
KDELC1	1109	968	0.218971719	0.0005156	0.001500037
MDM2	16152	14096	0.218725145	5.23E-40	1.48E-38
WDR44	2575	2247	0.218579824	1.28E-07	6.60E-07
LGALS8	1411	1231	0.218264906	9.47E-05	0.000316882
RAP1A	4231	3691	0.218165184	1.41E-11	1.09E-10
MKNK2	2302	2008	0.218023568	6.34E-07	2.98E-06
GLRX5	1840	1605	0.218019663	8.48E-06	3.43E-05

F3	24587	21440	0.217561323	2.41E-59	1.07E-57
ISG20L2	1070	933	0.217490321	0.0007067	0.002004959
CNN1	2179	1900	0.217485354	1.34E-06	6.07E-06
DHCR24	4234	3691	0.2171426	1.74E-11	1.33E-10
PEX2	1606	1400	0.217107066	3.44E-05	0.000126063
MAP2K4	3157	2752	0.217078341	6.38E-09	3.78E-08
APPL2	4003	3489	0.216884114	6.48E-11	4.66E-10
MOXD1	1860	1621	0.216733601	8.61E-06	3.48E-05
IPO7	17907	15605	0.216636829	2.48E-43	7.81E-42
C18orf25	2170	1891	0.216606452	1.56E-06	7.01E-06
ZBTB38	12009	10464	0.216470556	1.42E-29	2.91E-28
KCTD9	5472	4768	0.216468137	2.49E-14	2.36E-13
SCRN1	16245	14155	0.216468137	2.08E-39	5.79E-38
AMPD3	1321	1151	0.216409499	0.0001812	0.000574447
WSB1	6577	5729	0.216005799	7.54E-17	8.55E-16
CYCS	9421	8206	0.215951082	1.92E-23	3.14E-22
KIF3A	2493	2171	0.215628975	2.99E-07	1.47E-06
ELF2	1277	1112	0.215550395	0.0002465	0.00076253
CDKN1B	1563	1361	0.21550142	5.02E-05	0.000177547
BCAT1	33513	29181	0.215460617	1.14E-78	6.84E-77
DNAJC1	1399	1218	0.215270302	0.0001272	0.000415676
ZDHHC2	4629	4030	0.215231408	3.19E-12	2.61E-11
CHD1	2884	2510	0.214768331	4.05E-08	2.21E-07
RPRD1B	1286	1119	0.214471525	0.0002525	0.00077948
ARHGAP18	4243	3692	0.214470006	2.98E-11	2.22E-10
KDSR	5185	4511	0.214255429	2.12E-13	1.89E-12
NMD3	4954	4310	0.214246132	7.21E-13	6.17E-12
IMPACT	2498	2173	0.214066829	3.57E-07	1.74E-06
INA	1420	1235	0.213772244	0.0001268	0.000414447
G2E3	2583	2246	0.213462416	2.45E-07	1.22E-06
SGMS1	1515	1317	0.213089684	7.96E-05	0.000270964
GSTA4	1100	956	0.212731131	0.0007902	0.00221761

DTX3L	1983	1723	0.212390156	6.83E-06	2.80E-05
CTDSPL2	1830	1590	0.212335249	1.56E-05	6.02E-05
TAP2	3026	2629	0.212254286	2.80E-08	1.55E-07
SF3B1	20830	18097	0.212239849	4.18E-48	1.50E-46
KDM3A	2065	1794	0.21219024	4.51E-06	1.90E-05
ZNF780B	1264	1098	0.212033723	0.0003364	0.001016602
UBXN2A	1377	1196	0.211840962	0.0001849	0.000585082
FKBP7	2320	2015	0.211807165	1.22E-06	5.55E-06
MORF4L1	15532	13490	0.211798868	3.68E-36	9.11E-35
STARD3NL	2738	2378	0.2117784	1.36E-07	6.98E-07
RPGRIP1L	1483	1288	0.211766127	0.000105	0.000349458
OPTN	6540	5680	0.211752426	3.79E-16	4.12E-15
ZFAND6	2897	2516	0.211724369	5.96E-08	3.19E-07
ATF6	5943	5159	0.211052197	1.01E-14	9.84E-14
PAQR3	1787	1551	0.210811184	2.26E-05	8.53E-05
FAM160B1	4899	4252	0.210806437	2.27E-12	1.88E-11
SSH1	11597	10063	0.210460954	4.37E-27	8.24E-26
NAMPT	3409	2958	0.210425585	5.15E-09	3.09E-08
RHEB	4320	3748	0.210241772	5.01E-11	3.65E-10
ISCU	3262	2830	0.210197403	1.14E-08	6.55E-08
DIEXF	1404	1218	0.210123329	0.0001814	0.000574995
TIPRL	3313	2874	0.21007399	8.96E-09	5.21E-08
ZCCHC14	2221	1926	0.209550442	2.66E-06	1.16E-05
CBLL1	1813	1572	0.209374424	2.25E-05	8.51E-05
SEPT10	6934	6012	0.209309304	1.17E-16	1.30E-15
VPS54	1998	1732	0.209034479	8.92E-06	3.60E-05
ZFAND3	5182	4492	0.209001044	8.52E-13	7.22E-12
PSAT1	3202	2775	0.208666595	1.99E-08	1.12E-07
PSMD10	2602	2255	0.208658603	4.20E-07	2.02E-06
ZNF322	1961	1699	0.208248449	1.17E-05	4.60E-05
COL1A2	346299	300016	0.208173353	0	0
LEMD3	2005	1737	0.208147649	9.42E-06	3.78E-05

PKN2	6456	5593	0.208133883	1.88E-15	1.93E-14
PAM	17766	15390	0.208026467	1.12E-39	3.17E-38
PDHB	3503	3034	0.207782229	5.06E-09	3.04E-08
GSK3B	6354	5503	0.20770537	3.56E-15	3.60E-14
VPS25	2105	1823	0.20764646	5.96E-06	2.46E-05
WDR60	1306	1131	0.207596164	0.0003631	0.001090486
SMARCA5	9441	8175	0.207431183	9.75E-22	1.46E-20
MON2	5918	5124	0.207312911	3.46E-14	3.26E-13
TRIB2	1646	1425	0.207149715	6.50E-05	0.000224936
MMGT1	3001	2598	0.207110244	6.97E-08	3.70E-07
ZNF326	2613	2262	0.207043936	4.92E-07	2.34E-06
CHURC1	2345	2030	0.207043936	1.89E-06	8.39E-06
QKI	9744	8435	0.207026067	2.67E-22	4.13E-21
IL1R1	3497	3027	0.206923011	6.06E-09	3.60E-08
LOC100129034	4653	4026	0.206338117	2.26E-11	1.70E-10
IQGAP1	31303	27084	0.206292028	2.04E-67	1.04E-65
MRPL19	4537	3925	0.206106126	4.21E-11	3.08E-10
RBM12B	1718	1486	0.205856211	5.04E-05	0.000177908
RAB8B	2798	2420	0.205763217	2.33E-07	1.16E-06
NUDT9	1465	1267	0.205667992	0.000184	0.000582639
GINM1	2409	2083	0.205380578	1.68E-06	7.48E-06
ZFAND5	11911	10299	0.205361854	1.76E-26	3.25E-25
ZNF330	1514	1309	0.205252024	0.0001482	0.000477718
CHM	2586	2235	0.204704688	7.61E-07	3.54E-06
SC5DL	3023	2612	0.20432605	9.49E-08	4.95E-07
NBEAL1	3283	2836	0.203994914	2.83E-08	1.56E-07
RUFY2	2060	1779	0.203574305	1.14E-05	4.51E-05
TYW3	1788	1544	0.203478148	4.38E-05	0.000156692
RANBP10	1252	1081	0.203284093	0.0006355	0.001814562
FRA10AC1	1230	1062	0.203277582	0.0007099	0.002012645
KDM5B	6508	5619	0.203251281	6.84E-15	6.76E-14
NACC2	6096	5262	0.202900477	5.34E-14	4.94E-13

LDLR	3178	2743	0.202787629	5.67E-08	3.04E-07
PREPL	6118	5280	0.202629954	5.21E-14	4.83E-13
ITPRIP	1496	1291	0.202530957	0.0001994	0.000626439
MAP1LC3B	9141	7888	0.202459683	3.85E-20	5.28E-19
SIK2	2815	2429	0.202379699	3.42E-07	1.67E-06
DOPEY1	1625	1402	0.202198763	0.0001087	0.000360178
BAZ2B	2491	2149	0.202092593	1.67E-06	7.48E-06
CAMK2N1	8256	7121	0.201789014	3.15E-18	3.97E-17
ZDHHC20	6655	5739	0.20151284	5.94E-15	5.92E-14
PPA2	1961	1691	0.201439256	2.28E-05	8.62E-05
UBR3	6679	5759	0.20133834	5.62E-15	5.61E-14
FSTL3	4433	3822	0.201196548	2.02E-10	1.40E-09
EZH1	1392	1200	0.201027326	0.0003701	0.001109616
HSDL1	1710	1474	0.200892331	8.03E-05	0.000272979
MAP7D3	3190	2749	0.200502615	7.63E-08	4.03E-07
USP47	9763	8413	0.200447945	5.34E-21	7.65E-20
ARPC5	15722	13548	0.200447269	8.05E-33	1.85E-31
CSNK1G3	2536	2185	0.200230666	1.70E-06	7.58E-06
MPZL1	11364	9791	0.200209517	4.00E-24	6.71E-23
ZZZ3	3654	3148	0.200111039	9.37E-09	5.43E-08
CCNA2	2397	2065	0.200064004	3.33E-06	1.43E-05
BLCAP	3400	2929	0.200025579	3.09E-08	1.70E-07
MTMR1	1268	1092	0.199570243	0.0007441	0.002102139
PIK3R3	2655	2286	0.199265674	1.10E-06	5.03E-06
TIMM10B	1568	1350	0.19918598	0.0001817	0.000575805
NECAP1	2275	1958	0.198666351	6.91E-06	2.83E-05
PDHX	2775	2388	0.198467197	7.01E-07	3.28E-06
ELTD1	2237	1925	0.198445321	8.44E-06	3.41E-05
UAP1	5224	4495	0.198318351	1.03E-11	8.04E-11
SRSF10	6118	5263	0.19797742	2.02E-13	1.80E-12
TRHDE	18804	16176	0.197967448	5.63E-38	1.50E-36
ZDHHC18	1845	1587	0.197833444	5.53E-05	0.000193956

MRAS	3518	3026	0.197808636	2.60E-08	1.44E-07
DNAJC10	11067	9518	0.197618296	5.94E-23	9.54E-22
CCNYL1	1871	1609	0.197506899	5.05E-05	0.000178404
MSMO1	2392	2057	0.197476536	4.60E-06	1.94E-05
PDE8A	2349	2020	0.197460711	5.61E-06	2.34E-05
SLC35B4	3107	2671	0.197009866	1.89E-07	9.52E-07
ZFP36	1260	1083	0.196761641	0.0009224	0.00255295
DDX20	1544	1327	0.19664775	0.0002469	0.000763676
DNAJC15	2589	2225	0.196562503	2.09E-06	9.23E-06
TRIM22	5575	4791	0.19650714	3.40E-12	2.77E-11
TBC1D15	2445	2101	0.196393829	4.10E-06	1.74E-05
MBTPS2	4076	3502	0.196177164	2.83E-09	1.75E-08
NUDCD1	1986	1706	0.195904156	3.46E-05	0.000126516
BDH2	1368	1175	0.195744658	0.0005951	0.001713465
UGP2	6059	5204	0.195693388	5.01E-13	4.33E-12
FMN2	3228	2772	0.195438811	1.39E-07	7.14E-07
DSG2	3341	2869	0.195420117	8.45E-08	4.44E-07
CHD9	7674	6589	0.195232894	5.00E-16	5.40E-15
SLC35A5	2394	2055	0.194867373	6.14E-06	2.54E-05
TXNDC12	2589	2222	0.194615983	2.66E-06	1.15E-05
ORMDL3	2631	2258	0.194586369	2.21E-06	9.73E-06
NFU1	1502	1289	0.194519583	0.0003505	0.001054975
RNF169	1918	1646	0.194513747	5.36E-05	0.000188345
ARPP19	10024	8601	0.194270122	2.87E-20	3.98E-19
SCAMP1	4670	4007	0.194252092	3.08E-10	2.08E-09
FMNL3	2535	2175	0.194181785	3.55E-06	1.52E-05
KLF12	3815	3273	0.194082677	1.31E-08	7.50E-08
PNPT1	1752	1503	0.193994366	0.0001181	0.000388437
FAM126B	1836	1575	0.193937902	8.14E-05	0.000276536
C14orf101	1630	1398	0.193644528	0.00021	0.000657283
HN1L	5029	4312	0.193237756	8.20E-11	5.85E-10
ARF4	28974	24843	0.193232664	7.52E-55	3.06E-53

RPS27A	11480	9843	0.193199489	9.73E-23	1.54E-21
CNBP	10307	8837	0.193156244	1.44E-20	2.02E-19
MED21	1519	1302	0.19275971	0.0003689	0.001106464
ZCCHC7	1503	1288	0.192439716	0.0004055	0.001204519
DIRAS1	1433	1228	0.192424083	0.0005547	0.001605204
AGO2	1957	1677	0.192391065	5.48E-05	0.000192088
LYRM7	1893	1622	0.19225154	7.34E-05	0.000251688
CCNY	3764	3225	0.192184664	2.28E-08	1.28E-07
PIGK	3945	3380	0.192150078	1.06E-08	6.12E-08
EFEMP1	15535	13309	0.192032066	7.73E-30	1.60E-28
DNAJB11	4843	4149	0.192014786	2.39E-10	1.64E-09
XPNPEP3	1597	1368	0.191856049	0.0002791	0.000855773
BTAF1	4126	3534	0.19171035	5.33E-09	3.18E-08
HERPUD1	3122	2674	0.19168106	3.85E-07	1.87E-06
QSER1	4845	4149	0.191419123	2.70E-10	1.84E-09
HAS2	3269	2799	0.191214242	2.20E-07	1.10E-06
UGGT2	4916	4208	0.190801921	2.30E-10	1.58E-09
CNST	4068	3482	0.190748656	8.14E-09	4.76E-08
RGL2	1881	1610	0.190712971	8.87E-05	0.000298961
RIOK3	5164	4420	0.190709501	8.33E-11	5.94E-10
TMX4	2109	1805	0.190591874	3.36E-05	0.000123336
BAZ2A	5928	5071	0.189876959	4.30E-12	3.46E-11
SRP19	2109	1804	0.189792375	3.63E-05	0.000132215
IDI1	1852	1584	0.189640369	0.0001103	0.00036487
IDS	8720	7457	0.189419338	5.26E-17	6.01E-16
RECK	4915	4202	0.189036877	3.42E-10	2.29E-09
GNPTAB	6637	5674	0.188987008	3.00E-13	2.64E-12
SNAP23	3825	3270	0.18898302	3.07E-08	1.69E-07
SLC35D1	2770	2368	0.188935236	2.47E-06	1.08E-05
ZNF608	1583	1353	0.188652716	0.0003771	0.001128999
ALDH9A1	4714	4029	0.188622179	8.52E-10	5.51E-09
NUFIP2	10976	9381	0.18861337	7.86E-21	1.12E-19

EMC7	4345	3713	0.18838127	4.05E-09	2.46E-08
CHD6	4310	3683	0.18834566	4.71E-09	2.83E-08
NEK6	5292	4522	0.188300062	8.65E-11	6.16E-10
RGS3	2480	2119	0.188195599	9.02E-06	3.63E-05
SLC35C2	1477	1262	0.188194216	0.0006128	0.001756568
ALS2	3587	3064	0.187790684	9.98E-08	5.19E-07
USP9Y	5093	4350	0.187651815	2.25E-10	1.55E-09
GOT1	1741	1487	0.187640576	0.0002087	0.000653894
NAE1	2330	1990	0.187590608	1.80E-05	6.89E-05
MEIS1	2978	2543	0.18733984	1.29E-06	5.83E-06
SLC7A11	13499	11526	0.18719152	6.95E-25	1.21E-23
FAS	3672	3135	0.187051516	7.96E-08	4.20E-07
DCBLD1	1771	1512	0.187046059	0.0001932	0.000607891
HLA-A	5310	4533	0.186906432	1.12E-10	7.89E-10
SRSF8	1578	1347	0.186804777	0.0004412	0.001301992
IL13RA1	8256	7047	0.186718375	9.38E-16	9.92E-15
GOPC	6025	5142	0.186520501	7.08E-12	5.60E-11
EPS8	4205	3588	0.186226222	1.08E-08	6.20E-08
STAM	2711	2313	0.186092285	4.48E-06	1.89E-05
PDE12	2343	1999	0.18607365	2.00E-05	7.63E-05
MYO6	5508	4699	0.18597734	6.34E-11	4.56E-10
PPIL4	2994	2554	0.185836435	1.47E-06	6.64E-06
FBXO33	1557	1328	0.185638334	0.0005241	0.001522581
PPP2R1B	3256	2777	0.185578609	5.34E-07	2.54E-06
PAFAH1B1	14387	12266	0.185051197	7.82E-26	1.41E-24
C10orf137	1458	1243	0.184987708	0.000826	0.002311375
SYAP1	6129	5224	0.184655324	7.72E-12	6.09E-11
ARID4A	2024	1725	0.184539204	8.49E-05	0.000287049
THUMPD1	2944	2509	0.184486931	2.15E-06	9.48E-06
C1GALT1	2646	2255	0.184466504	7.06E-06	2.89E-05
EDEM3	7499	6390	0.184269839	4.23E-14	3.94E-13
PLEKHA2	2386	2033	0.184168304	2.06E-05	7.84E-05

NUDT16	2795	2381	0.183871469	4.20E-06	1.78E-05
ZCRB1	2362	2012	0.183773472	2.36E-05	8.89E-05
TADA2B	1525	1299	0.18374432	0.0006833	0.001943116
OSTC	6373	5428	0.183600192	3.99E-12	3.23E-11
TARS	9670	8236	0.183580072	1.29E-17	1.55E-16
ME1	4486	3820	0.18329515	6.23E-09	3.70E-08
PPP6R3	5735	4883	0.183126423	5.26E-11	3.82E-10
ABHD10	2477	2109	0.183117358	1.61E-05	6.21E-05
MYL12B	15861	13503	0.18294837	1.09E-27	2.09E-26
ZFR	8784	7478	0.182926549	4.90E-16	5.29E-15
ZFY	1901	1618	0.182605208	0.0001648	0.000526967
MDN1	4923	4190	0.182564636	1.35E-09	8.56E-09
MIR100HG	9200	7828	0.182162027	1.36E-16	1.51E-15
RSF1	5485	4667	0.182155978	1.73E-10	1.21E-09
ADO	1533	1304	0.181738304	0.0007606	0.002144845
LRRC40	1926	1638	0.181479786	0.0001645	0.000526
ELMOD2	2327	1979	0.181452533	3.45E-05	0.000126333
FAM114A2	1671	1421	0.181346953	0.0004528	0.001333373
PSMD12	4371	3717	0.181327442	1.41E-08	8.04E-08
CHD2	9723	8268	0.181288999	2.72E-17	3.18E-16
KIFAP3	2368	2013	0.180830223	3.14E-05	0.000115862
TEP1	2272	1931	0.180537461	4.69E-05	0.000166373
STRADB	1865	1585	0.180459342	0.0002274	0.000706856
TBC1D2	2297	1952	0.180354328	4.34E-05	0.00015509
BLOC1S2	3415	2902	0.180314073	6.21E-07	2.92E-06
TSPAN3	8846	7517	0.180283893	1.05E-15	1.10E-14
TMED10	21142	17963	0.180068587	3.07E-35	7.46E-34
VWA8	2406	2044	0.179910686	2.99E-05	0.000110799
KIAA0556	1701	1445	0.179838483	0.0004512	0.00132935
CAPN7	2683	2279	0.179706002	1.07E-05	4.24E-05
TTPAL	3088	2623	0.179697187	2.32E-06	1.02E-05
LMAN2L	1534	1303	0.179690733	0.0008725	0.002429471

UHMK1	23874	20277	0.179556047	2.38E-39	6.57E-38
HIF1AN	4732	4019	0.179538635	5.17E-09	3.10E-08
REEP3	8433	7162	0.179468696	6.43E-15	6.38E-14
ABL2	9594	8147	0.1792886	9.89E-17	1.11E-15
CBS	2101	1784	0.179191585	0.0001025	0.000341512
SBDS	6977	5924	0.179116939	1.48E-12	1.24E-11
MYADM	5568	4727	0.178917785	2.72E-10	1.85E-09
PIK3CB	2284	1939	0.178902284	5.27E-05	0.000185522
WBP11	3760	3192	0.178880121	2.13E-07	1.07E-06
MORF4L2	14947	12689	0.178874558	4.48E-25	7.87E-24
ACADSB	1866	1584	0.178775481	0.0002602	0.000801526
SPOCK1	1983	1683	0.178502631	0.0001707	0.000543308
MAPK14	3798	3223	0.178316415	2.03E-07	1.02E-06
SGTB	2721	2309	0.178283348	1.09E-05	4.33E-05
CSNK2A2	1669	1416	0.177989442	0.0005856	0.001689063
ACTR2	45426	38535	0.177803249	7.37E-72	4.03E-70
CCDC14	6049	5131	0.177695497	6.37E-11	4.59E-10
CFL2	11019	9344	0.177271009	1.39E-18	1.77E-17
TMCO1	5188	4399	0.177149253	1.61E-09	1.02E-08
NR3C1	6424	5445	0.176612288	2.20E-11	1.66E-10
JMJD1C	4908	4160	0.176600411	4.95E-09	2.98E-08
ACP1	4015	3403	0.176559295	1.23E-07	6.37E-07
MOCS2	2217	1879	0.176508428	8.54E-05	0.000288508
SETD8	2451	2077	0.176282864	3.70E-05	0.000134345
PDP1	2500	2118	0.175926626	3.21E-05	0.000118355
PCMTD1	2643	2239	0.175830235	1.93E-05	7.37E-05
NCOA2	2302	1950	0.175738422	6.74E-05	0.000232508
BBS7	1883	1595	0.175675556	0.0003145	0.000954855
NUCB2	5797	4910	0.175568671	2.65E-10	1.80E-09
TAF13	2181	1847	0.175346228	0.0001087	0.000360265
PRKAR2A	3748	3174	0.175333307	3.91E-07	1.90E-06
SNRNP27	2278	1929	0.175237528	7.72E-05	0.000263602

FBXL17	2152	1822	0.174997013	0.0001248	0.000408678
MPDZ	3469	2937	0.174975557	1.11E-06	5.09E-06
TDP2	2157	1826	0.174812721	0.0001246	0.000408309
OSBPL8	10887	9216	0.174758291	6.82E-18	8.35E-17
C5orf42	3302	2795	0.174660295	2.11E-06	9.29E-06
MEX3C	3168	2681	0.174351016	3.53E-06	1.51E-05
UBE4B	4864	4116	0.174251885	9.32E-09	5.40E-08
ZMAT3	21994	18611	0.1741977	2.75E-34	6.54E-33
ENTPD4	7533	6374	0.174126622	9.23E-13	7.79E-12
SLC16A4	1727	1461	0.173840227	0.0006414	0.001829875
SH3BGRL	6757	5716	0.173775193	1.48E-11	1.14E-10
B2M	39886	33735	0.173515845	2.69E-60	1.22E-58
TNPO1	22721	19216	0.173433856	4.63E-35	1.12E-33
PCYOX1	13228	11187	0.173380384	4.41E-21	6.35E-20
ITSN1	4655	3936	0.173101185	2.41E-08	1.34E-07
CCSER2	6733	5693	0.173091767	1.94E-11	1.48E-10
SIL1	2522	2132	0.172791294	4.14E-05	0.000148914
FAM204A	1808	1528	0.172401997	0.0005345	0.0015515
RFX7	2521	2130	0.172009444	4.51E-05	0.000160684
MAGI2-AS3	7840	6624	0.17200115	6.29E-13	5.40E-12
PRDX4	6269	5296	0.171819924	1.31E-10	9.23E-10
ITCH	6991	5905	0.171590356	1.23E-11	9.50E-11
FNDC3A	7621	6437	0.171560285	1.50E-12	1.26E-11
DMPK	4168	3520	0.171372283	1.74E-07	8.79E-07
ZNF12	2012	1699	0.171207679	0.0002868	0.000877728
ITSN2	2056	1736	0.171078815	0.0002488	0.000769026
ENTPD6	3333	2814	0.170953143	3.15E-06	1.35E-05
ZBTB44	1944	1641	0.170699152	0.0003791	0.001133482
MTRF1L	2515	2123	0.170698103	5.29E-05	0.000186079
BNC2	4970	4195	0.170577091	1.36E-08	7.73E-08
ADAMTS2	10466	8833	0.170417384	1.82E-16	2.01E-15
ZKSCAN8	2006	1693	0.170412499	0.0003131	0.000951214

FAM35A	1717	1449	0.170319687	0.0008604	0.00239737
XBP1	4771	4026	0.170207618	2.83E-08	1.56E-07
PCMTD2	2067	1744	0.170013783	0.0002625	0.000807787
DDX18	4461	3763	0.169668273	8.76E-08	4.60E-07
C11orf57	1763	1487	0.169524304	0.0007774	0.002186222
SETD5	6390	5388	0.169086051	1.75E-10	1.23E-09
FRY	2548	2148	0.168780847	5.77E-05	0.000201822
PPP1CB	11779	9929	0.168655397	5.53E-18	6.83E-17
CCDC47	7016	5914	0.168637627	2.58E-11	1.94E-10
GLO1	7596	6402	0.168434903	4.20E-12	3.39E-11
CHST3	3179	2679	0.168273696	7.51E-06	3.06E-05
LIMA1	12424	10469	0.168146039	8.93E-19	1.15E-17
RAPGEF6	1976	1665	0.168091362	0.0004203	0.00124521
RNMT	2946	2482	0.167897817	1.70E-05	6.53E-05
PPP2R3A	4920	4145	0.167865918	2.73E-08	1.51E-07
CARS	4422	3725	0.167693532	1.41E-07	7.25E-07
SEC23A	20935	17634	0.167594978	2.42E-30	5.12E-29
ANXA1	28667	24146	0.167545411	6.67E-41	1.95E-39
RYBP	1851	1559	0.167468165	0.0006724	0.001912937
ARHGAP12	2750	2316	0.167355766	3.44E-05	0.000125989
TM7SF3	3956	3331	0.16706506	7.08E-07	3.30E-06
ZMPSTE24	7570	6374	0.167057851	6.88E-12	5.45E-11
ZNF622	2333	1964	0.166760754	0.000144	0.000465991
SETD2	5660	4764	0.166523492	3.38E-09	2.06E-08
ZBTB21	1857	1563	0.166496095	0.0007098	0.002012952
KIAA0232	4142	3486	0.166397148	4.34E-07	2.08E-06
TRAF3	2437	2051	0.166373373	0.0001063	0.000353209
CDH2	3219	2709	0.166299948	8.49E-06	3.43E-05
COL4A3BP	2920	2457	0.16608162	2.29E-05	8.63E-05
CYTH3	2816	2369	0.165782996	3.31E-05	0.000121801
LTN1	4441	3736	0.165762015	1.87E-07	9.40E-07
TMEM131	8782	7387	0.165591175	2.44E-13	2.16E-12

C20orf112	2101	1767	0.16537801	0.0003483	0.001049556
HSPA13	5532	4652	0.165202072	6.74E-09	3.98E-08
COPRS	3061	2574	0.16516114	1.62E-05	6.27E-05
TP53BP1	6705	5638	0.165098278	1.79E-10	1.25E-09
TRIM59	1996	1678	0.164783127	0.0005146	0.001497483
GABPA	3219	2706	0.164701394	1.04E-05	4.14E-05
LARP4	5187	4360	0.164579897	2.25E-08	1.26E-07
NTN4	2527	2124	0.164510234	9.57E-05	0.000320084
PRKG1	3564	2995	0.164210798	3.76E-06	1.60E-05
TRIM25	4249	3570	0.163952862	4.63E-07	2.21E-06
SLC35F6	3354	2818	0.163941054	7.52E-06	3.06E-05
JAM3	5046	4239	0.163735892	4.11E-08	2.23E-07
RAB2A	10758	9036	0.163498414	1.25E-15	1.31E-14
OTUD7B	2585	2171	0.163347577	8.97E-05	0.000301874
ACOT9	2791	2344	0.163342577	4.70E-05	0.000166888
UBE3A	7831	6575	0.162946478	1.04E-11	8.08E-11
SERTAD2	2087	1752	0.162724306	0.0004554	0.00134054
WDR26	14203	11921	0.162461702	6.76E-20	9.16E-19
MAGI1	2377	1995	0.162398975	0.0001887	0.000595454
CSRP1	18730	15719	0.162310672	1.11E-25	1.98E-24
FAM91A1	5566	4671	0.162242668	1.14E-08	6.56E-08
PBXIP1	3841	3223	0.162074356	2.17E-06	9.57E-06
CNN2	16012	13432	0.161672746	5.05E-22	7.69E-21
POLK	2594	2176	0.161652209	0.0001036	0.000344886
BTBD10	2618	2196	0.161565089	9.71E-05	0.000324505
ZNF516	3671	3079	0.161440885	3.99E-06	1.69E-05
NCKAP1	21309	17870	0.161228875	1.28E-28	2.53E-27
CTDSP2	10409	8729	0.16120895	8.84E-15	8.63E-14
ATL3	4340	3639	0.16099914	5.72E-07	2.70E-06
FAM198B	2587	2169	0.160902131	0.0001142	0.000376824
GBE1	8171	6850	0.160743468	7.37E-12	5.82E-11
STAM2	4254	3566	0.160638801	7.84E-07	3.64E-06

HSP90B1	84579	70899	0.160617908	1.20E-107	1.04E-105
CPSF3	2850	2387	0.159388779	6.06E-05	0.000210764
APOL6	2609	2185	0.159288467	0.0001257	0.000411566
USP9X	16345	13686	0.159003607	9.54E-22	1.44E-20
SOS1	5040	4220	0.158971397	1.04E-07	5.43E-07
TNFRSF10B	12184	10201	0.158874871	1.39E-16	1.54E-15
MICAL2	11706	9800	0.158757602	5.70E-16	6.13E-15
IRGQ	2702	2262	0.158723386	0.000101	0.000336867
PPP2R2A	4363	3651	0.158123327	8.58E-07	3.98E-06
CGGBP1	6261	5239	0.158050493	3.79E-09	2.31E-08
DLG1	3484	2915	0.157903391	1.12E-05	4.44E-05
LARS	7891	6602	0.157847131	3.91E-11	2.88E-10
WDR48	2257	1888	0.157604478	0.0004189	0.001241997
PTPRM	3752	3138	0.157337656	5.61E-06	2.33E-05
NDFIP1	7040	5887	0.157109331	5.27E-10	3.47E-09
DNAJC21	4353	3640	0.157080562	1.05E-06	4.80E-06
UTP14C	2496	2087	0.156964798	0.0002203	0.000686536
EPG5	14425	12061	0.156930342	6.64E-19	8.62E-18
C1S	5340	4464	0.156649417	6.93E-08	3.68E-07
AASS	2315	1935	0.156473505	0.0003903	0.001163702
GPX8	14851	12413	0.156443884	2.65E-19	3.52E-18
TUSC3	6942	5802	0.156351062	8.42E-10	5.45E-09
PLA2G12A	2482	2074	0.15606491	0.0002498	0.000771743
RABGGTB	2657	2220	0.15591358	0.0001534	0.000493407
RAB3GAP1	6703	5600	0.155772025	1.89E-09	1.18E-08
EVI5	4533	3787	0.155741392	7.85E-07	3.65E-06
ZNF160	2174	1816	0.155564394	0.0006346	0.001812505
BAG4	3165	2643	0.155123057	3.95E-05	0.000142822
G3BP2	13368	11163	0.15509329	3.01E-17	3.50E-16
XPOT	9470	7907	0.154918131	1.24E-12	1.04E-11
MEF2D	3217	2686	0.154895501	3.51E-05	0.000128292
BLOC1S6	6970	5819	0.154764722	1.16E-09	7.44E-09

CRYBG3	2664	2222	0.153416866	0.0001928	0.000607051
TNS1	11132	9285	0.153413133	2.50E-14	2.37E-13
ATP6V0A1	2591	2161	0.153342188	0.0002379	0.000737361
SEPT11	67281	56115	0.15333945	2.37E-78	1.39E-76
HYOU1	7156	5968	0.15324628	1.04E-09	6.64E-09
YWHAZ	50750	42319	0.153049846	2.60E-59	1.15E-57
RICTOR	4343	3621	0.152848356	2.11E-06	9.31E-06
SPCS2	3509	2925	0.152528809	2.10E-05	8.00E-05
GNPNAT1	4961	4135	0.152408504	4.34E-07	2.08E-06
COMMD7	2215	1846	0.152247986	0.0007436	0.002101085
HNRNPA0	5726	4772	0.152218504	5.88E-08	3.15E-07
NFE2L3	3864	3220	0.152117726	8.54E-06	3.45E-05
ANKRD40	3755	3129	0.152040883	1.16E-05	4.56E-05
C11orf58	9609	8005	0.151667236	2.59E-12	2.14E-11
DERL1	4574	3810	0.151486764	1.42E-06	6.40E-06
SH3GLB1	10248	8535	0.151272809	5.68E-13	4.89E-12
SPTSSA	3319	2764	0.151161118	4.16E-05	0.000149284
AK1	3114	2593	0.151005394	7.31E-05	0.000250927
MIB1	6628	5519	0.150985415	7.24E-09	4.26E-08
RNF19A	2277	1896	0.150976805	0.0006975	0.001980714
AGPAT3	6571	5471	0.150843745	8.67E-09	5.05E-08
RCC2	3530	2939	0.150809307	2.48E-05	9.29E-05
SENP6	5703	4747	0.150447149	8.95E-08	4.69E-07
AP3M1	5014	4173	0.150275056	5.52E-07	2.61E-06
ZC3H11A	9045	7527	0.1501066	1.84E-11	1.40E-10
DYRK1A	2413	2008	0.150083485	0.0005221	0.00151717
PDS5A	6886	5730	0.15002109	4.68E-09	2.82E-08
CMAS	2316	1927	0.14987345	0.0006892	0.001958961
TMEM181	6980	5807	0.149718129	3.96E-09	2.41E-08
IKBIP	9263	7706	0.149654804	1.22E-11	9.45E-11
MBNL2	9314	7748	0.149575204	1.10E-11	8.54E-11
ITFG1	6013	5002	0.149572231	4.83E-08	2.61E-07

RALA	4257	3541	0.149471885	4.46E-06	1.88E-05
INHBA	5142	4277	0.149421729	4.62E-07	2.21E-06
MOSPD1	2459	2045	0.14918124	0.0005005	0.00146167
PRRC2B	14925	12412	0.149156802	9.97E-18	1.21E-16
CAP1	26569	22093	0.148997538	3.00E-30	6.29E-29
SAR1A	12343	10261	0.148630381	7.91E-15	7.77E-14
COPS8	3645	3030	0.148551111	2.45E-05	9.20E-05
BTN2A1	2492	2071	0.148175617	0.0005023	0.001466505
CTR9	5516	4583	0.147821961	2.41E-07	1.20E-06
NUP50	5298	4401	0.14753566	4.39E-07	2.10E-06
PICALM	16585	13777	0.147534889	3.97E-19	5.20E-18
ABCD3	5019	4169	0.147453558	8.95E-07	4.14E-06
PAPSS2	13048	10835	0.147022598	2.81E-15	2.85E-14
KLHDC10	3791	3148	0.147009216	2.07E-05	7.88E-05
LAYN	4392	3647	0.146984277	4.62E-06	1.94E-05
LYPLA2	2275	1889	0.146908288	0.0009828	0.002702048
DHX36	4090	3396	0.146887747	9.96E-06	3.97E-05
AXL	35192	29220	0.146860804	2.03E-38	5.44E-37
TTC1	2957	2455	0.146740913	0.000175	0.000556466
RNF14	3932	3264	0.146529867	1.55E-05	6.01E-05
NDST1	13678	11354	0.146495484	7.75E-16	8.25E-15
SUZ12	3918	3252	0.146361992	1.65E-05	6.35E-05
CAB39	7629	6331	0.146091565	1.97E-09	1.23E-08
AFF4	20336	16876	0.146089189	1.15E-22	1.80E-21
SMARCA2	3538	2936	0.146070052	4.40E-05	0.000157118
ZMYM2	4155	3448	0.146063429	9.54E-06	3.82E-05
SELT	7910	6564	0.146049676	1.01E-09	6.47E-09
CORO1C	31445	26088	0.145707842	5.55E-34	1.31E-32
ATG12	4901	4066	0.1456862	1.62E-06	7.26E-06
KIAA0754	4310	3575	0.145407504	7.17E-06	2.93E-05
ALDH1L2	6743	5593	0.145383864	1.98E-08	1.12E-07
ACSL1	2327	1930	0.145281768	0.0009841	0.002705092

GFM1	3528	2925	0.144738196	5.31E-05	0.00018671
LITAF	3779	3133	0.144692383	2.89E-05	0.000107406
AFAP1	7053	5847	0.144611683	1.13E-08	6.51E-08
RPS27L	9261	7677	0.144526796	6.20E-11	4.48E-10
MMADHC	5994	4968	0.144298221	1.51E-07	7.69E-07
TRIM2	4621	3829	0.143914702	4.25E-06	1.80E-05
KIAA1430	4017	3328	0.143689104	1.86E-05	7.12E-05
HNRNPUL2	3425	2837	0.143414294	7.98E-05	0.000271725
FILIP1L	10366	8584	0.143014814	7.71E-12	6.08E-11
NCEH1	6948	5752	0.142618053	2.31E-08	1.29E-07
ERP44	3613	2991	0.142582787	5.63E-05	0.000197243
ARFIP1	2825	2338	0.142176194	0.0003837	0.00114607
FDFT1	3143	2601	0.142076259	0.0001819	0.000576234
STX7	3305	2735	0.142042693	0.0001244	0.00040775
FYTTD1	6212	5140	0.141862661	1.49E-07	7.61E-07
COG5	3678	3043	0.141724986	5.37E-05	0.000188945
RNF41	3721	3078	0.141454973	5.03E-05	0.000177813
PCMT1	3646	3015	0.140995572	6.34E-05	0.00021992
ELOVL6	2569	2124	0.140729008	0.0008052	0.002257674
TUG1	13577	11224	0.140574312	1.41E-14	1.35E-13
RNF115	3156	2609	0.140551871	0.0002077	0.000650947
PPP1R15A	3610	2984	0.14040083	7.40E-05	0.000253502
SLC39A10	4402	3638	0.14013853	1.26E-05	4.92E-05
GAPVD1	4822	3985	0.140100205	4.88E-06	2.04E-05
DNAJC13	12117	10013	0.139993865	4.51E-13	3.91E-12
ARMC1	3622	2993	0.139957864	7.60E-05	0.000259934
NSA2	3437	2840	0.13989321	0.0001169	0.000385016
SMURF2	23746	19620	0.139792661	4.47E-24	7.47E-23
ITM2B	14016	11579	0.139588115	8.29E-15	8.11E-14
LMBR1	3243	2679	0.139517689	0.0001901	0.00059935
LAMC1	48206	39816	0.1392877	7.94E-47	2.76E-45
DHRS7	3170	2618	0.139134389	0.0002339	0.000726264

MAST4	3226	2664	0.138999776	0.000209	0.000654478
DOCK5	3442	2842	0.138811589	0.0001309	0.000426742
PRCP	4411	3642	0.138777294	1.50E-05	5.82E-05
EPRS	17078	14100	0.138708263	1.68E-17	2.00E-16
MTMR6	4988	4117	0.138284115	4.51E-06	1.90E-05
MSL1	4620	3813	0.138185811	1.03E-05	4.10E-05
NR2F2	9464	7809	0.137839871	3.03E-10	2.05E-09
C20orf194	3036	2505	0.137790944	0.0003638	0.001092184
YTHDF3	8815	7273	0.137742139	1.26E-09	8.01E-09
CAPZA1	18286	15086	0.137622938	2.34E-18	2.96E-17
BPTF	8421	6947	0.137550665	3.08E-09	1.89E-08
ATP11B	3733	3079	0.13727849	8.21E-05	0.000278651
ARIH1	5609	4626	0.137173815	1.41E-06	6.36E-06
ATP6AP2	10771	8883	0.137118829	2.36E-11	1.78E-10
KIAA1109	10627	8764	0.137079139	3.24E-11	2.41E-10
ACTR10	3591	2961	0.136850969	0.000118	0.000388441
RB1CC1	6423	5294	0.13626303	2.95E-07	1.45E-06
API5	7923	6530	0.136188321	1.26E-08	7.23E-08
TPP2	6296	5189	0.136173127	3.93E-07	1.90E-06
SDCBP	8330	6865	0.136095357	5.51E-09	3.28E-08
ZFP91	5236	4315	0.136047594	3.81E-06	1.62E-05
PEX19	4017	3310	0.135864887	5.30E-05	0.000186429
TSG101	3037	2502	0.135587013	0.0004528	0.001333517
HEXB	10951	9021	0.135448789	2.86E-11	2.14E-10
ANKRD28	6219	5122	0.135176768	5.63E-07	2.66E-06
HDGF	10042	8270	0.135064736	2.11E-10	1.46E-09
STT3B	7893	6499	0.134796131	1.90E-08	1.07E-07
PHKB	4886	4023	0.134770006	9.81E-06	3.92E-05
STK17B	4372	3599	0.134454833	3.02E-05	0.000111641
SCAF8	4083	3361	0.134413107	5.56E-05	0.000194897
SLC9A7	7227	5949	0.134402416	8.22E-08	4.33E-07
VPS37A	2773	2282	0.134003307	0.0009287	0.002569384

MTMR3	3490	2872	0.133980844	0.0002039	0.000639938
ATG13	3828	3150	0.133913131	0.0001011	0.000337143
MALAT1	459685	378257	0.133873323	0	0
VCPIP1	3465	2851	0.133764819	0.0002201	0.000686081
U2SURP	6640	5463	0.133662313	3.21E-07	1.57E-06
EMP1	12561	10334	0.133599597	2.12E-12	1.76E-11
ACTR3	26653	21925	0.133431046	1.56E-24	2.67E-23
IREB2	4801	3949	0.133304564	1.47E-05	5.69E-05
SEC24C	8500	6988	0.132568899	9.80E-09	5.67E-08
NRP2	2931	2409	0.132193557	0.0007871	0.00221008
C17orf51	3256	2676	0.132129548	0.000405	0.001203438
SCN9A	11497	9449	0.132128235	3.00E-11	2.24E-10
PPP3CA	7274	5977	0.131824749	1.33E-07	6.86E-07
RLIM	6881	5654	0.131805771	2.92E-07	1.44E-06
ARL2BP	4730	3886	0.131597849	2.18E-05	8.28E-05
NUS1	6488	5330	0.131513845	6.73E-07	3.15E-06
PHF3	4716	3874	0.131412367	2.31E-05	8.70E-05
LTA4H	3095	2542	0.131176753	0.0006198	0.001775908
ATP5C1	6769	5559	0.131034797	4.27E-07	2.05E-06
PPHLN1	2881	2366	0.131032545	0.0009716	0.002674968
WDFY3	6836	5614	0.131028799	3.75E-07	1.82E-06
PSMA4	6069	4984	0.130997379	1.70E-06	7.58E-06
CUL5	4692	3853	0.130931304	2.60E-05	9.71E-05
HSP90AA1	99058	81337	0.130790419	3.33E-83	2.15E-81
GNL3L	3480	2857	0.130565862	0.0003036	0.000925582
SEC14L1	5738	4710	0.130331223	3.66E-06	1.56E-05
YY1	4394	3606	0.130016659	5.31E-05	0.000186824
AOX1	3449	2830	0.129776056	0.0003518	0.001058486
MAP4K4	25314	20768	0.129578927	4.08E-22	6.25E-21
YIPF6	3500	2871	0.12935054	0.0003331	0.001007617
RPRD2	3082	2528	0.129281733	0.000765	0.002155771
UBA3	3581	2937	0.129132879	0.0002911	0.000889928

TMEM173	4142	3397	0.129085796	9.81E-05	0.00032779
ADCY9	3863	3168	0.12900279	0.0001705	0.000542863
SEC63	8666	7106	0.128823584	1.88E-08	1.06E-07
SLU7	3032	2486	0.128708675	0.0008918	0.002476547
MGA	3596	2948	0.128495635	0.0003034	0.000925106
ZNF638	6640	5443	0.128370926	9.39E-07	4.33E-06
AQR	3317	2719	0.128349271	0.0005295	0.001537749
SNAI2	5630	4615	0.128347857	6.34E-06	2.61E-05
PTPN14	17056	13980	0.128237149	4.09E-15	4.13E-14
PEA15	24629	20183	0.127934712	4.79E-21	6.89E-20
YIPF5	7964	6526	0.127857898	8.85E-08	4.64E-07
C5orf24	9784	8017	0.127790202	3.11E-09	1.91E-08
AP3S1	4685	3838	0.127457795	4.33E-05	0.000154948
ZNF148	5258	4307	0.127321313	1.51E-05	5.84E-05
SZRD1	11106	9097	0.127275589	3.23E-10	2.17E-09
ECT2	3979	3259	0.127175585	0.0001699	0.00054141
HMGB1	16872	13816	0.126861119	1.13E-14	1.09E-13
ITPRIPL2	7626	6244	0.126696138	2.15E-07	1.08E-06
NPEPPS	8031	6575	0.126563386	1.06E-07	5.52E-07
CYP51A1	3697	3026	0.126209076	0.000323	0.000978968
TCEA1	6566	5374	0.126133703	1.67E-06	7.46E-06
TIMM23	3561	2914	0.125870573	0.000432	0.001276621
YIPF4	3169	2593	0.125746678	0.00091	0.002521975
UBE2Q1	3714	3038	0.125300186	0.0003464	0.001044112
CAND1	11324	9261	0.125008366	4.57E-10	3.03E-09
NRIP1	4262	3485	0.124780195	0.0001353	0.000440233
BNIP3	5614	4589	0.124302875	1.28E-05	5.02E-05
CNOT7	5656	4623	0.124199374	1.21E-05	4.75E-05
C6orf62	7855	6420	0.124114154	2.56E-07	1.27E-06
TM2D2	3599	2941	0.123862814	0.0005	0.001460353
ACAP2	7859	6422	0.123829045	2.71E-07	1.34E-06
ZCCHC6	3254	2659	0.123821658	0.0009376	0.002592779

SLC30A7	5004	4088	0.123465539	4.30E-05	0.000153838
CLTC	64853	52979	0.123399431	4.22E-49	1.53E-47
HTT	8099	6616	0.12336758	1.99E-07	9.99E-07
SGIP1	6086	4971	0.123193901	6.75E-06	2.76E-05
ZMYND11	6588	5380	0.122917749	2.97E-06	1.29E-05
CSDA	8435	6888	0.122849206	1.26E-07	6.51E-07
PARVA	23122	18881	0.122821115	2.16E-18	2.74E-17
UFL1	4050	3307	0.122753269	0.0002535	0.000782171
DCAF7	9462	7725	0.122541904	2.37E-08	1.32E-07
FKTN	4078	3329	0.122379227	0.0002523	0.000779089
CYLD	4033	3292	0.122263062	0.0002768	0.000849353
SAR1B	4777	3899	0.12215141	7.70E-05	0.000262865
DMXL2	3797	3099	0.122094888	0.0004265	0.001261055
GNAI3	9109	7433	0.121804058	5.21E-08	2.81E-07
HIATL1	3998	3262	0.121630442	0.0003168	0.000961682
RANBP2	12802	10444	0.121457281	1.23E-10	8.69E-10
NAA25	4851	3957	0.121277018	7.65E-05	0.000261414
MARS	6136	5005	0.121223718	8.74E-06	3.53E-05
MAPK1IP1L	6900	5628	0.121178098	2.44E-06	1.07E-05
OAZ2	5173	4219	0.121052028	4.57E-05	0.000162672
SETX	8030	6549	0.121026775	3.81E-07	1.85E-06
TMED7	10523	8581	0.12082376	6.51E-09	3.85E-08
STAT1	10174	8296	0.120752932	1.18E-08	6.75E-08
ASCC3	18469	15059	0.120672348	1.60E-14	1.54E-13
PTRF	65584	53469	0.120510932	2.25E-47	7.91E-46
ZKSCAN1	6734	5490	0.12049456	3.67E-06	1.56E-05
CDC42	18290	14911	0.120474071	2.37E-14	2.25E-13
IER3IP1	4100	3342	0.120239955	0.0003131	0.00095123
DCN	42297	34458	0.119435758	1.29E-30	2.75E-29
NDUFV2	5390	4391	0.119416393	4.07E-05	0.000146442
MYL9	32350	26353	0.119353626	9.10E-24	1.51E-22
RTN4	67947	55343	0.119143128	6.23E-48	2.22E-46

SEPT7	14372	11706	0.119139475	2.30E-11	1.73E-10
CACNA2D1	3531	2876	0.119138986	0.0009212	0.002550363
CSNK1A1	17966	14631	0.118911271	8.60E-14	7.85E-13
FYCO1	5422	4415	0.118740456	4.27E-05	0.000153309
YES1	5000	4070	0.118252831	9.11E-05	0.000306014
PHF20	4026	3277	0.118180632	0.0004495	0.001324892
SMC5	5464	4447	0.118027053	4.45E-05	0.000158799
IGF2R	23207	18882	0.117603681	5.08E-17	5.82E-16
DDX1	8900	7241	0.117535746	2.12E-07	1.06E-06
RAPGEF1	4334	3526	0.117485453	0.0002957	0.000902804
SMARCA1	9149	7443	0.117422305	1.48E-07	7.55E-07
BMPR2	17656	14362	0.117250265	3.08E-13	2.71E-12
HOOK3	9725	7910	0.117131576	6.49E-08	3.46E-07
CALM1	16255	13221	0.117099878	2.82E-12	2.32E-11
FUBP1	4642	3775	0.116882254	0.0001947	0.000612244
G3BP1	8592	6987	0.116831279	4.04E-07	1.95E-06
DDX21	10253	8335	0.116360136	3.54E-08	1.94E-07
IKBKAP	4434	3604	0.116142373	0.000297	0.000906456
VPS13C	7378	5996	0.115922726	3.19E-06	1.37E-05
TMBIM6	25274	20539	0.115864062	6.79E-18	8.33E-17
EIF3E	12785	10388	0.11561789	9.62E-10	6.19E-09
C4orf3	8586	6976	0.115565999	5.47E-07	2.59E-06
KPNA4	10779	8757	0.115437407	2.07E-08	1.16E-07
FBN1	54156	43997	0.115436102	3.01E-36	7.48E-35
ERLEC1	7772	6313	0.115191982	2.03E-06	8.98E-06
DPH3	4051	3290	0.114961631	0.0006211	0.001778661
ATP1B3	9230	7496	0.114942437	2.40E-07	1.20E-06
CBX1	5898	4789	0.114650745	3.82E-05	0.000138386
PON2	4768	3871	0.114474204	0.0002184	0.000681016
VMP1	5611	4555	0.114345273	6.18E-05	0.000214847
CDC123	3936	3195	0.114237842	0.0008033	0.002252891
ASPH	71795	58278	0.114220125	1.59E-46	5.45E-45

SAMD4A	6368	5169	0.114196998	2.03E-05	7.73E-05
LARP4B	4916	3990	0.114055962	0.0001842	0.000583308
SSB	7625	6188	0.113887991	3.31E-06	1.42E-05
CHMP4B	4520	3668	0.113822998	0.0003457	0.001042436
RSU1	7367	5978	0.113737786	4.99E-06	2.09E-05
MATR3	19678	15967	0.113661808	8.86E-14	8.09E-13
C16orf72	5281	4285	0.113636195	0.0001127	0.00037219
PSMA5	4172	3385	0.113568808	0.0006034	0.001732879
BCL2L13	4542	3685	0.113489045	0.0003484	0.001049555
MED1	5028	4079	0.113382989	0.0001704	0.000542745
PIK3C2A	7253	5883	0.113126319	6.64E-06	2.72E-05
ASAP2	5500	4461	0.113087661	8.80E-05	0.000297017
GNAS	44115	35768	0.112552298	2.04E-28	4.01E-27
SCAF11	9445	7653	0.111626735	3.95E-07	1.91E-06
SNX1	5437	4405	0.111483323	0.0001218	0.000399745
TOP2B	12226	9905	0.11142854	8.44E-09	4.92E-08
TMEM50A	6774	5487	0.111161721	1.90E-05	7.25E-05
SARS	8576	6946	0.111029637	1.54E-06	6.92E-06
DIP2C	4624	3745	0.110976453	0.0004201	0.001245263
P4HA2	8052	6521	0.110898179	3.30E-06	1.42E-05
CTTNBP2NL	5511	4463	0.110851811	0.00012	0.000394025
SEC22B	8621	6981	0.110730618	1.54E-06	6.94E-06
PRDX6	27124	21962	0.110591623	1.68E-17	2.00E-16
MYBL1	4917	3981	0.110504648	0.000293	0.000895376
UBE2K	4759	3853	0.110475863	0.0003683	0.00110494
TMEM200A	4538	3674	0.110447149	0.0005067	0.001478425
MAVS	9839	7964	0.11013366	3.30E-07	1.62E-06
COPS2	7880	6378	0.1100606	4.98E-06	2.08E-05
CAMSAP2	7941	6427	0.109976909	4.65E-06	1.96E-05
SP100	6063	4905	0.109373446	6.91E-05	0.00023814
SREK1	5429	4392	0.109343702	0.0001668	0.000532698
EID1	21600	17472	0.109165581	6.49E-14	5.99E-13

SRP9	14300	11565	0.108902251	1.16E-09	7.44E-09
NRD1	15075	12190	0.108692256	4.50E-10	2.99E-09
SLAIN2	4952	4004	0.108582791	0.0003572	0.00107347
COL1A1	432191	349431	0.108490706	1.42E-245	3.84E-243
FAM46C	4949	4001	0.108375718	0.0003682	0.001104922
ZMYM4	6706	5420	0.1079925	3.61E-05	0.000131467
PTPN1	5882	4753	0.107683749	0.0001144	0.000377245
ATP2C1	8853	7152	0.107332447	2.39E-06	1.05E-05
MTSS1L	4423	3573	0.107262803	0.0008622	0.002401866
TPM2	72813	58812	0.107066633	1.49E-41	4.44E-40
RAB3GAP2	4919	3973	0.10701587	0.0004555	0.001340514
SSR1	22738	18352	0.105984066	8.36E-14	7.65E-13
MAP4K5	6742	5440	0.105582156	5.16E-05	0.000181911
SERBP1	22258	17947	0.104570872	3.22E-13	2.82E-12
RAB11A	8656	6979	0.104471964	5.68E-06	2.36E-05
USP33	5452	4395	0.104229735	0.000327	0.000990767
PPP1R12A	11546	9307	0.104147114	1.74E-07	8.83E-07
FAM208B	6402	5159	0.103720901	0.000107	0.000355519
NFE2L2	7568	6098	0.103576193	2.60E-05	9.72E-05
SMS	12809	10320	0.103437254	4.63E-08	2.51E-07
FRYL	5238	4220	0.103379071	0.0004787	0.001403132
SRSF11	9737	7844	0.103264325	1.97E-06	8.72E-06
SKIL	5054	4071	0.103109689	0.000623	0.001782417
LGALS3	7693	6196	0.102942968	2.50E-05	9.39E-05
IPO5	17389	14005	0.102919146	2.37E-10	1.63E-09
FAM3C	6851	5515	0.102198432	7.89E-05	0.000268899
RAB5B	8823	7098	0.101295421	9.00E-06	3.62E-05
DNAJB6	8911	7168	0.101135428	8.40E-06	3.40E-05
MAPKAPK2	5872	4721	0.100392647	0.0003318	0.001003919
ABHD12	6275	5044	0.100104948	0.0002161	0.000675284
JAK1	15507	12464	0.099999662	6.25E-09	3.71E-08
SCD	6494	5219	0.099818149	0.0001751	0.000556844

DEGS1	8372	6728	0.099757526	2.06E-05	7.84E-05
VPS13D	5504	4423	0.099696904	0.00056	0.001620135
PPFIBP1	6828	5486	0.099443696	0.0001266	0.00041384
TBC1D23	5713	4589	0.099083409	0.0004773	0.001399391
GJA1	16014	12862	0.098933415	5.22E-09	3.12E-08
PHC2	5680	4561	0.098611372	0.0005279	0.001533217
AMOTL1	8045	6459	0.098370531	3.87E-05	0.000140079
TNKS	6576	5278	0.097933183	0.0002126	0.000665177
RAB10	8789	7054	0.097894681	1.87E-05	7.14E-05
WBP5	6911	5546	0.097705269	0.000152	0.00048941
THRAP3	11782	9453	0.097411834	8.19E-07	3.80E-06
TMEM59	9100	7299	0.096984407	1.60E-05	6.20E-05
ACSL3	7106	5699	0.096823236	0.0001415	0.000458324
VOPP1	7126	5715	0.096813147	0.0001387	0.000450417
PPP2CB	6107	4897	0.096586483	0.000433	0.001278851
TWF1	14406	11545	0.095750495	8.42E-08	4.43E-07
DDX5	31035	24853	0.094675996	7.51E-15	7.39E-14
MTCH2	6719	5380	0.094511774	0.0003054	0.000930038
ANKRD13A	10807	8653	0.09445835	4.72E-06	1.98E-05
AP1G1	9364	7495	0.093955675	2.26E-05	8.55E-05
YME1L1	12981	10390	0.093946259	6.09E-07	2.86E-06
C12orf75	19829	15866	0.093478663	8.53E-10	5.51E-09
TMF1	7475	5980	0.093224037	0.0001729	0.000550091
ROCK1	10034	8026	0.09300835	1.42E-05	5.51E-05
RBFOX2	19604	15679	0.092837628	1.38E-09	8.79E-09
LAMP2	15170	12132	0.092748449	1.02E-07	5.31E-07
RAB1A	11874	9496	0.092737954	2.49E-06	1.09E-05
RALGAPB	7424	5933	0.091717204	0.000232	0.000720478
SYPL1	6103	4877	0.091627514	0.0008546	0.002383381
MKLN1	7260	5799	0.090986722	0.000305	0.000929147
LRPPRC	9744	7783	0.090964356	2.88E-05	0.000107069
KIDINS220	8514	6800	0.090849788	9.44E-05	0.000316133

PTEN	7411	5918	0.090593603	0.0002808	0.000860567
ITGB1	263717	210358	0.089008242	4.96E-101	3.86E-99
KIAA0368	8189	6532	0.088989635	0.0001771	0.000562124
TRIO	23117	18439	0.088958363	3.00E-10	2.03E-09
PPAP2B	10374	8271	0.08831353	2.82E-05	0.00010482
RHOBTB3	13920	11096	0.088032613	1.33E-06	6.00E-06
PFN2	16840	13418	0.087429642	1.28E-07	6.59E-07
PAFAH1B2	8926	7111	0.087190781	0.0001258	0.000411657
AKAP11	9024	7188	0.086975473	0.0001202	0.000394537
PTPRF	8478	6750	0.086315668	0.0002165	0.000676006
TRIM44	6798	5412	0.086203609	0.0009403	0.00259904
CMTM6	10995	8752	0.085989171	2.71E-05	0.000101287
HSPH1	10946	8712	0.085824229	2.93E-05	0.000108629
ARPC3	12294	9784	0.085694051	9.78E-06	3.90E-05
COPZ1	7632	6073	0.08550033	0.0005092	0.001482892
ECE1	13994	11130	0.084797328	3.05E-06	1.32E-05
RDX	10370	8243	0.083977638	6.93E-05	0.000238883
ASH1L	7666	6093	0.083830874	0.0006388	0.001823126
HIGD1A	8291	6589	0.083665539	0.0003941	0.001173591
PRKAR1A	22094	17553	0.083215121	8.69E-09	5.06E-08
CDH6	11363	9025	0.082807191	4.03E-05	0.000145173
UGGT1	15353	12187	0.081974574	2.31E-06	1.01E-05
NUCKS1	25494	20225	0.081134085	1.69E-09	1.06E-08
CREB3L2	12695	10070	0.08095542	2.22E-05	8.40E-05
S100A11	18284	14496	0.080225268	4.56E-07	2.18E-06
SPTAN1	33622	26652	0.079990385	9.06E-12	7.09E-11
PRRC1	8912	7063	0.079683996	0.0004704	0.001381252
PLXNA1	8261	6546	0.079449303	0.0007884	0.00221317
GALNT10	9103	7209	0.0786092	0.0004903	0.001434117
DYNC1LI2	15423	12211	0.078250066	6.28E-06	2.59E-05
RCN1	44774	35448	0.078195153	1.44E-14	1.39E-13
NOTCH3	12208	9663	0.077868331	6.37E-05	0.000220973

C12orf23	16480	13044	0.077822235	3.43E-06	1.47E-05
ANKRD17	10491	8297	0.076661621	0.0002638	0.000811737
AIDA	9777	7732	0.076601917	0.0004327	0.001278215
OS9	11387	8994	0.074799214	0.0002093	0.000655211
NF2	9804	7743	0.07467429	0.000594	0.001710612
SEC31A	28912	22827	0.074224989	4.55E-09	2.74E-08
CALR	80626	63646	0.07397683	1.63E-22	2.55E-21
USO1	13236	10447	0.073773647	8.09E-05	0.000274811
ADAR	16359	12909	0.073444817	1.29E-05	5.03E-05
GNS	14791	11665	0.072618845	4.11E-05	0.000148109
ITGA5	29482	23248	0.072424343	7.70E-09	4.52E-08
WNK1	14288	11266	0.072323523	5.97E-05	0.000208097
RELN	15014	11838	0.072269106	3.93E-05	0.00014209
MYH9	121195	95487	0.071198188	1.06E-30	2.27E-29
ZEB1	14590	11493	0.07092768	6.98E-05	0.000240218
HSPA9	12884	10149	0.070909149	0.0001868	0.000589879
UBR5	13185	10384	0.070616849	0.0001673	0.000534155
NAP1L1	24830	19547	0.070015075	3.04E-07	1.49E-06
PTPN11	24037	18921	0.069883623	4.93E-07	2.35E-06
NCOR1	11828	9306	0.069179075	0.0004806	0.001407649
LRP10	16642	13085	0.068237232	4.42E-05	0.000157726
VCL	48521	38145	0.06803681	3.51E-12	2.85E-11
RPS24	32229	25327	0.067468906	1.91E-08	1.07E-07
TTC37	14149	11118	0.067349327	0.000202	0.000634226
TPD52L2	11813	9279	0.066817977	0.0007553	0.002131149
CCT8	12241	9601	0.064687295	0.0009047	0.002508935
CCND1	160644	125938	0.063998696	1.04E-32	2.39E-31
SEPT2	41062	32173	0.063198701	2.88E-09	1.77E-08
NFE2L1	16904	13241	0.062799542	0.0001537	0.000494309
MLEC	19057	14922	0.062272021	6.76E-05	0.000232969
PRNP	13180	10319	0.062104929	0.000951	0.002625249
LTBP2	29430	23030	0.061379001	1.06E-06	4.85E-06

TMED2	20647	16156	0.061290009	4.47E-05	0.000159499
GLG1	24169	18868	0.057935855	3.02E-05	0.000111667
ACTN1	49549	38681	0.057921283	2.32E-09	1.44E-08
SCARB2	15525	12119	0.057829523	0.000846	0.00236264
YWHAE	30592	23880	0.05780054	2.83E-06	1.23E-05
COL12A1	116111	90512	0.055828458	1.18E-18	1.51E-17
MYLK	45908	35762	0.054833972	5.32E-08	2.86E-07
DDX3X	20699	16122	0.054621788	0.0002749	0.000844122
KPNB1	28662	22317	0.054155953	2.19E-05	8.31E-05
CDKN1A	43051	33464	0.051715286	6.84E-07	3.20E-06
CCDC50	25308	19647	0.049867668	0.0002429	0.000752305
HNRNPC	22322	17326	0.049624459	0.0006052	0.001737265
HNRNPK	29301	22727	0.048609488	0.000119	0.000391276
EIF3A	29882	23141	0.04632663	0.0002137	0.00066856
ITGA6	25956	20084	0.045130691	0.0007779	0.002187209
LOXL2	36729	28329	0.040512191	0.0003358	0.001015022
HNRNPA2B1	41258	31717	0.035734562	0.0008087	0.002266598
PDE5A	57674	44299	0.034505179	0.0001313	0.000427628
TPM4	83500	64110	0.033925343	6.05E-06	2.50E-05
MSN	65534	50269	0.032577529	0.0001193	0.000391938
SFRP1	245912	181983	-0.019186319	1.31E-05	5.12E-05
CALU	104609	76940	-0.028049112	3.42E-05	0.000125465
HSPA5	56281	41174	-0.035762219	0.0001098	0.000363373
TIMP2	53243	38942	-0.036112751	0.0001456	0.000470071
ANXA2	119666	87486	-0.036737102	6.79E-09	4.01E-08
RPL6	52190	38087	-0.039322615	4.26E-05	0.000153036
GALNT5	35049	25553	-0.040728473	0.0005159	0.001500568
TLN1	61382	44743	-0.04100153	3.71E-06	1.58E-05
LMAN1	25501	18525	-0.045928148	0.0008478	0.002367134
ACTB	304427	220964	-0.047132846	1.90E-32	4.30E-31
PLOD1	23672	17164	-0.048643664	0.000666	0.001895941
GANAB	29806	21587	-0.050287883	7.91E-05	0.000269714

MEG3	23969	17359	-0.050333748	0.0003964	0.001179805
RPL11	36071	26081	-0.052688101	5.43E-06	2.26E-05
UACA	69769	50430	-0.053151799	1.75E-10	1.22E-09
CTSC	25389	18350	-0.053271378	0.0001149	0.000378812
RPL14	19330	13962	-0.054183889	0.0006213	0.001778584
FLNA	173926	125542	-0.055151386	1.29E-25	2.30E-24
ALDOA	48682	35139	-0.055163173	3.21E-08	1.76E-07
MAP4	48913	35291	-0.05576551	2.11E-08	1.18E-07
PABPC1	59311	42772	-0.056480896	4.14E-10	2.76E-09
THBS1	90310	65123	-0.056566453	1.13E-14	1.09E-13
HDLBP	77601	55918	-0.057610351	3.10E-13	2.72E-12
SLC38A2	81587	58768	-0.05815636	4.51E-14	4.20E-13
GNB1	28783	20725	-0.058692736	6.18E-06	2.55E-05
COPG1	16595	11948	-0.058827355	0.0005824	0.001680718
CD109	17154	12336	-0.060518224	0.0003224	0.000977457
DDOST	14058	10098	-0.062169646	0.0008267	0.002312767
DDX17	20925	15020	-0.063190678	3.38E-05	0.000124109
PAPPA	15705	11241	-0.067301428	0.0001321	0.00043011
PDIA3	29700	21243	-0.068323277	9.49E-08	4.95E-07
SYNCRIP	17670	12637	-0.068495897	3.69E-05	0.00013419
MYL6	56001	40047	-0.068606286	1.85E-13	1.65E-12
RPL26	32023	22895	-0.068923773	2.28E-08	1.27E-07
CDK2AP1	11960	8550	-0.069068933	0.0006211	0.001778433
RPS27	23414	16732	-0.069609388	1.39E-06	6.29E-06
ENAH	11186	7992	-0.069913615	0.0008093	0.002267731
NACA	23732	16952	-0.070226063	9.53E-07	4.39E-06
CCT4	13719	9798	-0.070463996	0.0001852	0.000585351
GOLGA3	11384	8129	-0.070705642	0.0006333	0.001809222
TGFBR2	19384	13829	-0.072017342	5.61E-06	2.33E-05
RPL5	30310	21614	-0.072675631	1.00E-08	5.81E-08
GSTP1	16310	11630	-0.072753553	2.59E-05	9.68E-05
ITGA2	30607	21819	-0.07312453	6.91E-09	4.08E-08

PLXNB2	22990	16389	-0.073126505	5.15E-07	2.45E-06
CDC42BPB	11849	8446	-0.073273024	0.000305	0.000929093
RPL19	33056	23558	-0.073542963	1.41E-09	8.93E-09
PXN	12995	9260	-0.073720403	0.0001421	0.000460291
VDAC2	11108	7909	-0.074879751	0.0003544	0.00106539
RPL27	19156	13638	-0.075012119	2.61E-06	1.14E-05
AAK1	11858	8442	-0.075051836	0.0002165	0.000675868
FSCN1	20891	14872	-0.075130745	8.87E-07	4.11E-06
SEL1L	12240	8712	-0.075375567	0.0001606	0.000515079
DBN1	16470	11722	-0.075469681	1.17E-05	4.60E-05
IGFBP5	147752	105154	-0.075521797	1.60E-39	4.48E-38
MFGE8	36781	26174	-0.075674154	5.05E-11	3.68E-10
ADD3	15727	11187	-0.076268161	1.50E-05	5.83E-05
ACO1	11289	8030	-0.076293671	0.0002445	0.00075665
FAM115A	11638	8278	-0.076336866	0.0001943	0.000611172
ECM1	11113	7904	-0.076441347	0.0002661	0.000817972
KLF6	9275	6595	-0.076822491	0.0008157	0.002284043
EEF1B2	17360	12336	-0.077740146	3.59E-06	1.53E-05
CD44	181388	128888	-0.077808934	5.81E-51	2.21E-49
HECTD1	15873	11276	-0.078167309	8.39E-06	3.40E-05
PSMC1	8634	6132	-0.078519264	0.0009668	0.002663438
HSP90AB1	61612	43754	-0.078644322	1.01E-18	1.30E-17
AMOTL2	11134	7906	-0.078799996	0.0001694	0.000539894
CD81	22567	16022	-0.079008263	7.93E-08	4.19E-07
C15orf52	8786	6237	-0.079202109	0.0007865	0.002208821
RAC1	17326	12299	-0.079245472	2.38E-06	1.04E-05
RPL7	35600	25266	-0.079527828	1.14E-11	8.83E-11
RPS3A	43599	30937	-0.07980961	4.76E-14	4.43E-13
MBTPS1	9072	6437	-0.07988004	0.0005806	0.001676303
SUMO2	8365	5935	-0.079965379	0.0009424	0.002604241
SMCHD1	9922	7036	-0.080723343	0.0002774	0.000850691
CHCHD2	10613	7525	-0.080916905	0.0001639	0.000524628

PSMD2	33577	23801	-0.081299014	1.62E-11	1.24E-10
APLP2	26708	18926	-0.081750283	1.54E-09	9.71E-09
TJP1	9990	7079	-0.081786971	0.0002195	0.000684476
KDELR2	28600	20259	-0.082300052	3.13E-10	2.11E-09
HSPB1	21109	14948	-0.082753635	5.48E-08	2.94E-07
MMP2	17700	12532	-0.082980554	6.04E-07	2.84E-06
LAPTM4A	13889	9833	-0.08308705	9.61E-06	3.84E-05
TXLNA	8293	5871	-0.083135704	0.0006226	0.001781691
STARD7	8323	5888	-0.084173834	0.0005205	0.001513039
PGAM1	22353	15801	-0.085300472	8.30E-09	4.85E-08
RPL12	27466	19410	-0.08569457	1.39E-10	9.78E-10
KHDRBS1	11239	7941	-0.085968944	3.84E-05	0.000138885
ARPC2	22902	16179	-0.086199021	3.79E-09	2.31E-08
TSPYL1	9020	6372	-0.086229035	0.0002163	0.000675376
SRRM2	30351	21440	-0.086287013	1.11E-11	8.60E-11
EEF1G	52848	37327	-0.08647718	2.63E-19	3.49E-18
IGFBP4	53747	37957	-0.086666127	1.10E-19	1.48E-18
FUCA2	7166	5060	-0.086878527	0.000895	0.002484279
BRD2	8778	6198	-0.086937384	0.0002343	0.000727319
GOLGA2	8724	6159	-0.08714151	0.000237	0.000734949
RPS18	57330	40456	-0.087784464	2.18E-21	3.21E-20
ARHGAP21	10816	7630	-0.088259963	3.40E-05	0.000124684
SEC16A	11427	8059	-0.088621824	1.89E-05	7.22E-05
USP14	7939	5598	-0.088893678	0.0003488	0.001050685
SURF4	25306	17843	-0.088969153	1.65E-10	1.15E-09
PRKCSH	12998	9161	-0.089560527	4.03E-06	1.71E-05
SMARCE1	7975	5619	-0.090018987	0.0002847	0.000871739
HNRNPM	11521	8116	-0.090273043	1.22E-05	4.79E-05
RPL27A	45405	31973	-0.090844927	2.33E-18	2.95E-17
CRTAP	11819	8320	-0.09130041	7.48E-06	3.04E-05
TOMM70A	7244	5099	-0.091420077	0.0004457	0.001314059
CDC37	11136	7838	-0.091521506	1.31E-05	5.11E-05

DDX42	7830	5508	-0.092331616	0.0002273	0.00070687
LGALS3BP	8577	6033	-0.092445407	0.0001119	0.000369841
APC	6381	4487	-0.092879218	0.0008158	0.00228372
TOR1AIP2	9121	6412	-0.093265449	5.86E-05	0.000204593
STOM	18815	13226	-0.093354351	7.58E-09	4.45E-08
GBP3	6810	4787	-0.09338086	0.0005081	0.001480531
RPS8	38803	27272	-0.09359556	8.81E-17	9.92E-16
SEC62	14731	10353	-0.093654358	2.93E-07	1.44E-06
PDIA4	14459	10161	-0.093773253	3.64E-07	1.77E-06
NUCB1	8679	6099	-0.093803976	8.07E-05	0.000274515
EEF1A1	551935	387686	-0.094457605	3.65E-222	8.75E-220
HSPA4	15622	10970	-0.094863508	8.89E-08	4.66E-07
SUN1	5947	4176	-0.094888232	0.0009659	0.00266144
TMEM214	6480	4550	-0.094975136	0.0005645	0.001632747
RRBP1	26828	18833	-0.095324543	1.88E-12	1.57E-11
PSMD8	7288	5114	-0.095918667	0.000222	0.000691661
DIAPH1	6044	4241	-0.095947046	0.0007693	0.002166353
ILF3	15061	10568	-0.09596306	1.09E-07	5.66E-07
HNRNPH1	16071	11276	-0.096052187	3.99E-08	2.18E-07
FST	6152	4316	-0.096208507	0.0006674	0.001899629
GPX4	8908	6249	-0.096324086	4.14E-05	0.000148939
RPL23	28238	19807	-0.096475396	2.64E-13	2.34E-12
BACE1	10120	7098	-0.096572679	1.18E-05	4.66E-05
ZC3H13	6193	4343	-0.096794362	0.0005939	0.001710701
CDC42EP3	26922	18878	-0.096927529	7.41E-13	6.33E-12
PSAP	30319	21260	-0.096928442	2.72E-14	2.57E-13
NONO	17575	12322	-0.097136117	6.39E-09	3.78E-08
SEPN1	12136	8507	-0.0974185	1.31E-06	5.93E-06
PEAK1	9857	6909	-0.097519582	1.27E-05	4.98E-05
ATP5B	30963	21692	-0.098230054	6.54E-15	6.49E-14
PRDX1	15696	10994	-0.09852843	2.63E-08	1.46E-07
BGN	5702	3992	-0.099204187	0.0007354	0.002078724

MGAT4B	7208	5045	-0.09959261	0.0001387	0.000450387
SERINC3	10940	7657	-0.099609444	2.66E-06	1.16E-05
RPL23A	20895	14623	-0.099766307	8.05E-11	5.75E-10
NCSTN	8434	5902	-0.099860969	3.58E-05	0.000130731
PPP2R1A	13716	9596	-0.100202615	1.23E-07	6.38E-07
TAGLN2	20817	14558	-0.100797871	5.62E-11	4.08E-10
TAOK2	5683	3974	-0.100908711	0.0006096	0.001748588
LARP1	15638	10933	-0.101214552	1.18E-08	6.76E-08
HINT1	7007	4898	-0.101451991	0.0001303	0.000425026
RTF1	6459	4514	-0.10175227	0.0002297	0.000714074
CTSB	54402	38008	-0.102204447	6.41E-27	1.20E-25
CHPF	8388	5860	-0.102274065	2.45E-05	9.20E-05
ZFC3H1	5345	3734	-0.102315889	0.0007535	0.00212652
BRK1	7506	5243	-0.102499613	6.34E-05	0.00021996
ARL1	8215	5738	-0.102560475	2.83E-05	0.000105203
ZNFX1	6737	4705	-0.102759445	0.0001453	0.000469475
LANCL1	5109	3567	-0.103177588	0.0008958	0.002485959
LTBR	5844	4080	-0.103234894	0.0003791	0.00113369
PSMD3	6521	4552	-0.10344053	0.0001687	0.000538026
ODC1	9372	6542	-0.103473063	6.44E-06	2.65E-05
PXDN	81051	56546	-0.104252881	8.58E-41	2.51E-39
MAN2B2	6653	4641	-0.104417204	0.0001256	0.000411242
H2AFV	5653	3943	-0.104570855	0.0004	0.00118969
PABPC4	12417	8660	-0.104725593	1.48E-07	7.59E-07
EIF4G1	37188	25935	-0.104784659	8.98E-20	1.21E-18
SH3BP4	13129	9152	-0.105445945	5.34E-08	2.87E-07
RPL37A	36947	25754	-0.105508571	6.68E-20	9.07E-19
SUGP2	6228	4341	-0.10558939	0.0001759	0.000559109
SPECC1L	5855	4081	-0.105594328	0.0002754	0.000845562
UBE2I	4857	3385	-0.105757521	0.000907	0.002514756
SEC13	10134	7062	-0.105902876	1.59E-06	7.13E-06
DNAJC8	5292	3686	-0.106604859	0.000482	0.001411665

SDF4	13427	9351	-0.106792306	2.55E-08	1.42E-07
RAF1	5213	3630	-0.10699218	0.0005075	0.001479747
MCM3AP	6562	4569	-0.10710502	9.43E-05	0.000315876
TPT1	89591	62367	-0.107418816	1.59E-47	5.62E-46
RRM2	7413	5160	-0.107534314	3.09E-05	0.000114097
TMEM127	5326	3707	-0.107648182	0.0004072	0.001208703
CRK	6921	4817	-0.107693449	5.53E-05	0.000193991
CHD3	13503	9398	-0.107702166	1.78E-08	1.00E-07
RBMX	10054	6997	-0.107809066	1.15E-06	5.23E-06
RPL36	15630	10876	-0.10801759	1.22E-09	7.79E-09
TARDBP	9062	6305	-0.108180989	3.58E-06	1.53E-05
ARPC1A	10980	7639	-0.108270226	3.30E-07	1.62E-06
NUDT21	6428	4472	-0.108297609	9.35E-05	0.000313254
PAMR1	8554	5951	-0.108314958	6.55E-06	2.69E-05
SHMT2	4981	3465	-0.108427927	0.0005748	0.001660649
MRPL51	4560	3172	-0.108488922	0.0009796	0.002693986
GNL3	4674	3251	-0.108622046	0.0008339	0.002331898
PDK1	5911	4111	-0.108760717	0.0001684	0.0005372
KIAA1191	6252	4348	-0.108813706	0.0001083	0.000359244
PRPF8	22952	15953	-0.10963999	7.80E-14	7.15E-13
DAP	19239	13370	-0.109882216	7.03E-12	5.56E-11
MRPL3	5247	3646	-0.110026097	0.000337	0.001017929
COL16A1	7947	5522	-0.110067325	1.02E-05	4.05E-05
CBX3	8997	6251	-0.110204889	2.57E-06	1.12E-05
DSTYK	4827	3353	-0.110522212	0.0005527	0.001599906
BCAP31	6604	4587	-0.110637081	5.25E-05	0.00018508
UBE4A	6574	4564	-0.111320533	4.92E-05	0.000174487
TPM3	28683	19911	-0.111478114	2.02E-17	2.39E-16
SLC39A7	13200	9163	-0.111493873	8.35E-09	4.88E-08
CBL	5859	4067	-0.111537327	0.0001233	0.00040427
COX4I1	8604	5971	-0.111882824	3.06E-06	1.32E-05
RPL34	17401	12075	-0.111994896	3.05E-11	2.27E-10

BUB3	6538	4536	-0.112276605	4.46E-05	0.000159105
PDIA6	15702	10893	-0.112394862	2.40E-10	1.65E-09
PSMA1	7738	5368	-0.112423925	8.71E-06	3.52E-05
UGDH	4305	2986	-0.11264694	0.00089	0.002472085
MAGT1	7827	5428	-0.112886592	7.11E-06	2.90E-05
NXPE3	5817	4034	-0.112912088	0.000108	0.000358203
CLOCK	5356	3713	-0.113418514	0.0001903	0.000599587
SOX9	5919	4103	-0.113522171	8.62E-05	0.000291142
EXOC7	6279	4352	-0.113704124	5.11E-05	0.00018046
TMCO3	6207	4302	-0.113736489	5.62E-05	0.00019684
SMC4	9350	6480	-0.11382042	7.51E-07	3.50E-06
GSN	17810	12343	-0.113842297	8.47E-12	6.65E-11
HUWE1	27072	18761	-0.113912613	3.54E-17	4.09E-16
FAM129B	34342	23792	-0.114345377	1.61E-21	2.39E-20
LPAR1	21543	14923	-0.114529444	4.13E-14	3.85E-13
ATP5A1	24298	16828	-0.114821714	8.56E-16	9.08E-15
RPLP1	33451	23165	-0.114950526	3.33E-21	4.83E-20
PRKCA	12165	8423	-0.115178117	1.13E-08	6.51E-08
FBXW2	5425	3756	-0.115273943	0.0001357	0.000441205
GDI1	9572	6625	-0.115747811	3.60E-07	1.75E-06
NOMO1	5107	3533	-0.116430178	0.000185	0.000585158
COX7A2	4746	3283	-0.116545219	0.0003091	0.000940548
PSMA7	7611	5264	-0.116774399	4.71E-06	1.98E-05
AP2A1	10075	6968	-0.116811178	1.38E-07	7.07E-07
RPL4	57755	39941	-0.116923264	1.42E-36	3.58E-35
ANXA11	10955	7576	-0.116929117	3.82E-08	2.09E-07
TALDO1	6765	4678	-0.117045941	1.52E-05	5.90E-05
IRS1	8376	5791	-0.117296814	1.41E-06	6.38E-06
SASH1	9485	6557	-0.117459747	2.76E-07	1.36E-06
XPO6	8558	5915	-0.117743379	9.91E-07	4.56E-06
NFIC	13030	9005	-0.117886771	1.49E-09	9.42E-09
HGSNAT	6800	4698	-0.118335901	1.17E-05	4.60E-05

RBBP7	4875	3368	-0.118357949	0.0002057	0.000645026
RPL9	35473	24507	-0.118375325	1.28E-23	2.10E-22
TCP1	12977	8965	-0.118429282	1.36E-09	8.64E-09
CSE1L	8406	5806	-0.118722753	1.01E-06	4.65E-06
GPC6	3873	2675	-0.118760478	0.0009016	0.002500935
PI4KB	5785	3995	-0.118969324	4.82E-05	0.000171003
TMX1	4759	3286	-0.119173842	0.0002222	0.000692165
BMP1	5114	3531	-0.119223208	0.0001286	0.000419887
FRMD6	22090	15249	-0.11952665	1.47E-15	1.53E-14
PHB	5543	3826	-0.119678002	6.30E-05	0.000218662
OSBPL3	6998	4829	-0.120066069	6.47E-06	2.66E-05
QARS	6697	4621	-0.120157747	1.01E-05	4.02E-05
AEBP1	5584	3853	-0.120164624	5.53E-05	0.000193804
BTF3	17913	12360	-0.1201761	5.07E-13	4.38E-12
PPM1G	5391	3719	-0.12048603	7.12E-05	0.000244656
LMAN2	6779	4675	-0.120953974	7.77E-06	3.16E-05
FKBP10	24076	16597	-0.1215211	2.53E-17	2.96E-16
STRAP	11758	8102	-0.122140561	2.76E-09	1.70E-08
SEC61B	4348	2995	-0.122643806	0.0002836	0.000868561
AHNAK2	9524	6560	-0.122719674	7.63E-08	4.03E-07
PLBD2	4061	2797	-0.122802642	0.0004441	0.001309649
YBX1	42497	29263	-0.123131189	4.37E-30	9.11E-29
HNRNPF	6745	4644	-0.123298339	5.50E-06	2.29E-05
PLEKHM2	4061	2796	-0.123318535	0.0004205	0.001245568
APEX1	5252	3616	-0.123320107	6.05E-05	0.00021048
CCT7	13036	8975	-0.123365281	2.59E-10	1.77E-09
CLASP1	6785	4669	-0.124083095	4.51E-06	1.90E-05
DDX6	16045	11040	-0.124231486	1.64E-12	1.37E-11
ATIC	4276	2942	-0.124312475	0.0002647	0.000813957
SF3B3	10220	7031	-0.124441265	1.65E-08	9.36E-08
TSC2	5110	3514	-0.125056968	6.05E-05	0.000210497
ILF2	8151	5604	-0.125367979	3.81E-07	1.85E-06

CEP170	14757	10144	-0.125620679	7.56E-12	5.97E-11
SND1	20149	13848	-0.125878478	1.09E-15	1.14E-14
SEPW1	3543	2435	-0.125897561	0.0007748	0.002180941
SPTBN1	24408	16774	-0.125975192	1.04E-18	1.34E-17
CTNNB1	18536	12738	-0.12603904	1.38E-14	1.33E-13
IQSEC1	3740	2570	-0.126117779	0.0005407	0.001567607
EPAS1	14651	10066	-0.126356504	6.84E-12	5.42E-11
UBC	28018	19239	-0.127168031	1.31E-21	1.95E-20
HSPB6	10382	7127	-0.127565432	5.56E-09	3.31E-08
ACVR1	3339	2292	-0.127656918	0.0009396	0.002597804
POGZ	3849	2642	-0.127701073	0.000381	0.001138624
CLIC1	20788	14266	-0.128017954	1.25E-16	1.39E-15
SF3B4	3493	2397	-0.128084602	0.0006875	0.001954512
PAICS	10019	6875	-0.128154769	8.80E-09	5.12E-08
VCP	34022	23343	-0.128325836	2.50E-26	4.58E-25
RAB6A	9068	6221	-0.128485753	4.10E-08	2.23E-07
LEPREL2	7930	5440	-0.128562083	2.84E-07	1.40E-06
PHAX	4283	2938	-0.12863515	0.0001602	0.000514075
SMC1A	5513	3781	-0.12891762	1.77E-05	6.80E-05
FLOT1	7634	5234	-0.12937313	4.02E-07	1.94E-06
KDELC2	6699	4592	-0.129670978	1.96E-06	8.66E-06
TPGS2	5738	3933	-0.12976565	1.05E-05	4.17E-05
PCBP1	10034	6877	-0.129893461	5.44E-09	3.25E-08
UHRF1	3740	2563	-0.13005266	0.0003635	0.001091493
SFPQ	13561	9290	-0.130560935	9.40E-12	7.33E-11
MEGF8	5883	4030	-0.130620066	7.10E-06	2.90E-05
SERPINB6	6022	4125	-0.130696457	5.47E-06	2.28E-05
GPR124	9377	6423	-0.130727004	1.39E-08	7.94E-08
RPL7A	51709	35419	-0.130739793	1.52E-40	4.42E-39
RNF10	7418	5081	-0.130765672	4.46E-07	2.14E-06
GNAI2	23138	15848	-0.130811249	4.66E-19	6.08E-18
TMEM8A	4138	2834	-0.130941755	0.0001599	0.000513166

LDHB	21896	14995	-0.131033686	3.57E-18	4.48E-17
CPNE1	4837	3312	-0.131257733	4.29E-05	0.000153701
FLNB	15557	10652	-0.131287414	2.14E-13	1.91E-12
GMPS	3926	2688	-0.131374903	0.000225	0.000700318
SIPA1L3	3491	2390	-0.131477607	0.0004988	0.001457685
MTCH1	12928	8850	-0.131597611	2.00E-11	1.52E-10
VKORC1	5331	3649	-0.131752899	1.63E-05	6.27E-05
TBCD	3695	2529	-0.131855196	0.0003285	0.000994739
CTPS1	3814	2610	-0.132102905	0.0002564	0.000790372
F2R	26593	18197	-0.132193783	4.38E-22	6.70E-21
POLR1A	4111	2813	-0.132227687	0.0001453	0.000469391
MXRA8	18882	12919	-0.132365051	3.60E-16	3.92E-15
TMSB4X	30735	21020	-0.132967684	1.51E-25	2.70E-24
ANLN	12482	8536	-0.133064904	2.72E-11	2.03E-10
ERLIN1	3298	2255	-0.133311834	0.0006038	0.001733584
ILK	9460	6468	-0.133368373	6.18E-09	3.67E-08
AP1M1	4280	2926	-0.133528895	9.09E-05	0.000305804
RPS13	15349	10492	-0.133702821	1.16E-13	1.05E-12
CHMP1A	4263	2914	-0.133716046	9.19E-05	0.000308485
ITM2C	9177	6272	-0.133944916	9.01E-09	5.23E-08
DPP9	4381	2994	-0.134033862	7.06E-05	0.000242861
TMEM259	6014	4110	-0.134034342	3.22E-06	1.38E-05
PFDN5	10022	6848	-0.134263704	1.73E-09	1.09E-08
FXYD5	6647	4541	-0.134541185	8.95E-07	4.14E-06
SIGMAR1	4103	2803	-0.134555278	0.0001131	0.000373461
DYNLT3	5765	3938	-0.134705365	4.62E-06	1.94E-05
NHP2	3231	2207	-0.134741989	0.0006026	0.001731278
PDPR	3565	2435	-0.134828173	0.0003114	0.000946763
EIF3B	11129	7601	-0.134910693	1.83E-10	1.27E-09
TUBA1A	36339	24819	-0.134921599	1.01E-30	2.17E-29
EDC4	3329	2273	-0.135339055	0.0004702	0.001381333
TRA2A	5245	3581	-0.135428122	1.12E-05	4.43E-05

MAP7D1	9367	6395	-0.135490574	4.28E-09	2.59E-08
QSOX1	37429	25552	-0.135568154	7.10E-32	1.60E-30
SPOCD1	5102	3483	-0.135580294	1.44E-05	5.62E-05
EFTUD2	5240	3577	-0.135664562	1.09E-05	4.33E-05
POLDIP2	4551	3106	-0.135973625	4.00E-05	0.000144131
PLOD3	9539	6510	-0.136028357	2.69E-09	1.66E-08
GPSM1	5992	4089	-0.136137429	2.37E-06	1.04E-05
PEG10	6565	4480	-0.136144147	7.82E-07	3.64E-06
ACIN1	9310	6353	-0.136191017	3.99E-09	2.42E-08
RNPEP	5157	3519	-0.1362144	1.19E-05	4.66E-05
PSMB4	7628	5205	-0.136254548	9.88E-08	5.15E-07
GTF3C1	8754	5973	-0.13633453	1.12E-08	6.43E-08
FAM50A	3472	2369	-0.136336617	0.0003217	0.000975757
KIF1C	15691	10706	-0.13636561	2.02E-14	1.92E-13
HNRPLL	3179	2169	-0.136390832	0.0005745	0.001660107
RPS20	26111	17815	-0.136413096	5.49E-23	8.84E-22
SMAD3	19432	13258	-0.136419105	1.65E-17	1.97E-16
RPS12	21540	14696	-0.136442587	3.02E-19	3.98E-18
EEF2	143631	97990	-0.136508596	5.06E-119	4.81E-117
FOXK2	3316	2262	-0.136692946	0.0004244	0.001255922
NIPSNAP1	4442	3030	-0.136739467	4.49E-05	0.000160074
NEO1	11609	7918	-0.136883599	4.00E-11	2.94E-10
PNN	5774	3938	-0.136955865	3.17E-06	1.36E-05
MTDH	15056	10268	-0.1370312	5.12E-14	4.75E-13
RPL32	18536	12640	-0.137181354	6.11E-17	6.97E-16
MED15	4662	3179	-0.137223856	2.73E-05	0.00010186
EML4	4202	2865	-0.137388891	6.69E-05	0.000230829
ETF1	11919	8126	-0.137493798	1.81E-11	1.38E-10
BIRC2	9576	6528	-0.137629963	1.64E-09	1.03E-08
GTF2I	4580	3122	-0.137724929	3.01E-05	0.000111466
TOR4A	4519	3080	-0.137921074	3.32E-05	0.000122044
HMGN2	12997	8858	-0.137973621	1.90E-12	1.57E-11

UBB	23297	15877	-0.138053719	3.94E-21	5.69E-20
B4GALT2	3073	2094	-0.138234193	0.0006032	0.001732802
ATRX	13800	9403	-0.138323112	3.48E-13	3.04E-12
ERGIC1	18199	12398	-0.138599638	5.75E-17	6.56E-16
CBX5	16787	11436	-0.138609773	9.03E-16	9.55E-15
SRSF4	3350	2282	-0.138710172	0.0003263	0.000988883
CTSD	11405	7768	-0.138899209	3.14E-11	2.34E-10
BAG2	3640	2479	-0.139028047	0.0001739	0.000553326
LONP2	6121	4168	-0.139259959	1.08E-06	4.95E-06
DHX30	3234	2202	-0.139353078	0.0003901	0.001163626
WDR45B	4102	2793	-0.139359786	6.48E-05	0.00022447
ACADVL	10740	7312	-0.139503886	9.77E-11	6.93E-10
NXN	5182	3528	-0.139506323	6.98E-06	2.85E-05
ALDH18A1	6111	4160	-0.139672821	1.03E-06	4.71E-06
TOMM22	2803	1908	-0.139758441	0.0009288	0.002569127
SOCS5	6849	4662	-0.139792224	2.25E-07	1.12E-06
CREB3L1	18290	12448	-0.139988978	2.39E-17	2.81E-16
MYO10	23106	15724	-0.140147128	1.51E-21	2.23E-20
P4HB	57589	39190	-0.140155566	3.05E-51	1.16E-49
RPLP0	76176	51838	-0.140174345	3.11E-67	1.57E-65
DRP2	3668	2496	-0.140223573	0.0001446	0.000467386
STK10	3250	2211	-0.140588561	0.0003356	0.001014424
NDUFV1	3831	2606	-0.14073181	9.72E-05	0.000324783
WIPI1	3318	2257	-0.140755336	0.000286	0.000875318
TUFM	6163	4192	-0.140841942	7.53E-07	3.51E-06
SDC3	18498	12573	-0.141888236	5.92E-18	7.30E-17
SPIN1	10609	7210	-0.142065378	5.90E-11	4.27E-10
RRN3	2924	1987	-0.142199307	0.0005826	0.001681003
PTPN12	5289	3594	-0.142252435	3.70E-06	1.57E-05
YWHAH	5719	3886	-0.142324873	1.47E-06	6.64E-06
DPP3	3561	2419	-0.142719535	0.0001393	0.000452169
AP2M1	30875	20971	-0.142891347	2.81E-29	5.69E-28

UQCRH	4668	3169	-0.14362477	1.14E-05	4.51E-05
ITGB1BP1	3614	2453	-0.143897141	0.0001095	0.000362697
ITFG3	4118	2795	-0.143943415	3.62E-05	0.000131736
DNAJB14	2863	1943	-0.144089638	0.0005656	0.001635129
TNRC6A	8588	5827	-0.144416749	2.17E-09	1.35E-08
FAM127A	10329	7008	-0.144473775	5.17E-11	3.76E-10
CEP164	2811	1907	-0.144626478	0.0006068	0.001741344
GNB2L1	48038	32589	-0.144638872	1.21E-45	4.07E-44
PTMS	8414	5708	-0.144654352	2.97E-09	1.83E-08
RIPK1	2646	1795	-0.144677086	0.0008761	0.002438495
CLPTM1	8225	5579	-0.144856996	4.25E-09	2.57E-08
SNRPD2	4507	3057	-0.144898766	1.37E-05	5.32E-05
PRSS12	3966	2690	-0.144926373	4.49E-05	0.00016002
TRAF7	4171	2829	-0.144949004	2.84E-05	0.000105868
EBNA1BP2	3974	2695	-0.145154468	4.29E-05	0.000153769
DIS3	4887	3314	-0.14522337	5.65E-06	2.35E-05
NECAP2	3404	2308	-0.145435681	0.0001485	0.000478767
TSN	4754	3223	-0.145585583	7.20E-06	2.94E-05
BSG	19839	13448	-0.145795546	4.25E-20	5.81E-19
CUL7	4067	2756	-0.146236971	3.06E-05	0.000113096
THUMPD3	2683	1818	-0.146342723	0.0007032	0.001995492
SEPHS2	2751	1864	-0.146402148	0.0005983	0.001720715
WHSC1	6870	4653	-0.14699678	5.16E-08	2.78E-07
PCGF3	3931	2662	-0.147233661	3.70E-05	0.000134192
DBNL	5108	3459	-0.14725138	2.56E-06	1.11E-05
TUBGCP2	4795	3247	-0.147271299	5.17E-06	2.16E-05
PPIA	52305	35417	-0.147354719	2.62E-51	1.00E-49
NFX1	3208	2172	-0.147497907	0.0001888	0.000595601
ABLIM3	8156	5522	-0.147518756	2.61E-09	1.61E-08
CCM2	2545	1723	-0.147590823	0.0008764	0.002438152
GSK3A	2612	1768	-0.14788449	0.0007314	0.00206905
CYB5R3	16841	11398	-0.148044977	8.97E-18	1.09E-16

TULP4	2803	1897	-0.148099934	0.0004587	0.001349254
CHAMP1	2905	1966	-0.14812271	0.0003604	0.001082611
MAP3K7	5409	3660	-0.148366117	1.08E-06	4.96E-06
ACOT7	4342	2938	-0.148373198	1.25E-05	4.90E-05
R3HDM1	3312	2241	-0.148407892	0.0001354	0.000440242
ELF1	2650	1793	-0.14846474	0.0006378	0.001820761
ABCF1	4366	2954	-0.148490172	1.16E-05	4.59E-05
NOTCH1	3488	2359	-0.14907249	8.37E-05	0.000283248
FGFR1	10774	7286	-0.149202938	4.53E-12	3.63E-11
IMPAD1	15955	10789	-0.149295325	3.57E-17	4.12E-16
TNC	36780	24870	-0.14936284	1.61E-37	4.18E-36
PRKDC	30353	20524	-0.149375053	2.91E-31	6.37E-30
ATXN7L3	3087	2087	-0.149622751	0.0002041	0.000640553
DPYSL2	7945	5371	-0.149704366	2.50E-09	1.54E-08
TSPYL2	3775	2551	-0.150261614	3.72E-05	0.000134999
USP39	2541	1717	-0.150354204	0.0007105	0.002013611
UROD	3643	2461	-0.150730206	4.83E-05	0.000171339
TRNP1	11770	7951	-0.150753963	2.76E-13	2.44E-12
COL6A3	352355	238016	-0.150818996	0	0
PLCG1	5183	3501	-0.1508682	1.23E-06	5.57E-06
SLC44A2	9912	6695	-0.15094	1.92E-11	1.46E-10
PIGT	3608	2437	-0.150940956	5.13E-05	0.000181039
ELAC2	3457	2335	-0.150945922	7.37E-05	0.000252414
DZIP1	4335	2928	-0.150964308	9.02E-06	3.63E-05
KIAA1462	14378	9711	-0.151019106	6.03E-16	6.47E-15
DRAP1	12391	8368	-0.151185735	5.61E-14	5.19E-13
ARFIP2	2387	1612	-0.151194691	0.0009698	0.002670566
KITLG	9918	6697	-0.151382126	1.66E-11	1.27E-10
EIF3J	4788	3233	-0.151397513	2.89E-06	1.25E-05
CNP	6312	4262	-0.15141448	7.76E-08	4.10E-07
GPI	11311	7637	-0.151496422	6.19E-13	5.32E-12
MICA	3586	2421	-0.151620277	5.02E-05	0.000177516

USP22	19446	13127	-0.151784049	3.27E-21	4.75E-20
NPDC1	3025	2042	-0.151800144	0.0001926	0.000606687
BICD2	4606	3109	-0.151911663	4.14E-06	1.76E-05
LDHA	61008	41179	-0.151937509	4.19E-63	1.96E-61
SET	22441	15144	-0.152238518	2.30E-24	3.90E-23
S100A13	5225	3526	-0.152246431	8.91E-07	4.12E-06
MRC2	19957	13467	-0.152314233	7.27E-22	1.10E-20
HECTD3	2798	1888	-0.152385066	0.0003191	0.000968388
SF1	7391	4987	-0.152445239	4.85E-09	2.91E-08
LDOC1	2793	1884	-0.152864477	0.0003097	0.000942058
CCDC88A	7711	5201	-0.152976798	2.00E-09	1.25E-08
RNF20	3754	2532	-0.152999114	2.85E-05	0.000105867
SSU72	4042	2726	-0.153131628	1.38E-05	5.38E-05
PRKACA	5328	3593	-0.153253015	5.90E-07	2.78E-06
INTS1	7056	4758	-0.153343157	8.87E-09	5.16E-08
TCF25	5522	3723	-0.15357314	3.49E-07	1.70E-06
QRICH1	3744	2524	-0.153716393	2.68E-05	0.000100171
SF3B5	2501	1686	-0.153748389	0.000598	0.00172007
NR1D2	6490	4375	-0.15378333	3.19E-08	1.75E-07
FAR1	5437	3665	-0.153845498	4.10E-07	1.98E-06
INTS10	2568	1731	-0.153887346	0.0004991	0.00145807
PTOV1	4562	3075	-0.153927904	3.46E-06	1.48E-05
PARP1	5648	3807	-0.153933387	2.41E-07	1.20E-06
COTL1	35610	24000	-0.154095898	1.49E-38	4.02E-37
UBAP2L	10346	6972	-0.15427649	2.47E-12	2.04E-11
AK2	5177	3488	-0.154564146	6.90E-07	3.23E-06
MBD1	2694	1815	-0.154628171	0.0003405	0.00102814
KANK1	3120	2102	-0.154631228	0.0001156	0.000380874
XRCC6	18185	12251	-0.154697279	1.25E-20	1.75E-19
NISCH	6328	4263	-0.154728413	3.93E-08	2.15E-07
CD151	22050	14854	-0.154775041	1.07E-24	1.84E-23
LAMB1	38420	25880	-0.15486777	7.57E-42	2.27E-40

EIF3I	10672	7187	-0.155216849	8.25E-13	7.01E-12
RFWD3	2385	1606	-0.155365242	0.0007085	0.002009592
RPL10A	21854	14714	-0.155555736	1.02E-24	1.76E-23
INPPL1	8319	5600	-0.155831158	2.25E-10	1.55E-09
SGSH	2282	1536	-0.155968444	0.0008846	0.002459956
NCKAP5	3539	2382	-0.156016217	3.45E-05	0.000126079
TLK2	2881	1939	-0.156104725	0.0001847	0.000584721
CTTN	24243	16316	-0.156128695	1.99E-27	3.81E-26
MDH2	8461	5694	-0.15623356	1.43E-10	1.00E-09
DYNLRB1	4520	3041	-0.156624825	2.62E-06	1.14E-05
ICMT	7790	5241	-0.156629088	6.89E-10	4.49E-09
MED12	2738	1842	-0.156697253	0.0002537	0.000782496
RRP36	2915	1961	-0.156754216	0.0001592	0.0005112
RNF26	2663	1791	-0.157134959	0.0002971	0.000906387
EIF3G	5668	3812	-0.157139507	1.30E-07	6.71E-07
MLL4	2410	1620	-0.157887202	0.0005448	0.001578279
BCAR1	5967	4011	-0.157891679	5.30E-08	2.85E-07
SLFN5	7882	5298	-0.157961774	3.94E-10	2.63E-09
TSR1	2730	1835	-0.157968756	0.0002314	0.000718975
LRP1	83406	56061	-0.15800156	3.02E-92	2.20E-90
LONP1	3720	2500	-0.158222395	1.67E-05	6.44E-05
TKT	11040	7419	-0.158291395	1.17E-13	1.06E-12
DLST	5938	3990	-0.158436215	5.15E-08	2.78E-07
SMARCD2	2778	1866	-0.158945481	0.0001866	0.000589157
SH3PXD2B	3908	2625	-0.158960913	9.32E-06	3.74E-05
CRIP2	2796	1878	-0.159015166	0.0001767	0.000561187
SLC16A3	12867	8642	-0.159086454	8.33E-16	8.84E-15
FBXO30	3496	2348	-0.159120645	2.71E-05	0.000101299
POLR2A	15247	10240	-0.159157559	1.85E-18	2.35E-17
PSMB1	7010	4706	-0.159760992	2.44E-09	1.51E-08
TOP2A	13326	8946	-0.159777004	1.93E-16	2.13E-15
RPL29	23521	15790	-0.159786095	8.23E-28	1.59E-26

CCDC6	6069	4074	-0.159860709	2.79E-08	1.55E-07
CAPNS1	18560	12457	-0.160087911	2.31E-22	3.58E-21
TPR	9172	6156	-0.160090992	8.06E-12	6.35E-11
MRPL28	3035	2037	-0.160098404	8.39E-05	0.000283639
RRAGA	5796	3890	-0.160135308	5.43E-08	2.92E-07
CRELD2	2475	1661	-0.16022432	0.0003787	0.001133125
FKBP15	2796	1876	-0.160552401	0.0001534	0.000493478
SLC25A39	3063	2055	-0.160654841	7.35E-05	0.000251936
HOXB3	3954	2652	-0.161079964	6.30E-06	2.60E-05
ZNF292	3324	2229	-0.161371634	3.35E-05	0.000123214
DOCK4	2996	2009	-0.161407928	8.19E-05	0.000277948
AP2S1	5846	3920	-0.161444043	3.73E-08	2.04E-07
CS	11140	7469	-0.161610098	2.85E-14	2.69E-13
CLEC11A	3832	2569	-0.161738539	8.06E-06	3.27E-05
DLGAP5	2676	1794	-0.161746094	0.0001914	0.000602883
MTOR	4763	3193	-0.161805864	6.41E-07	3.01E-06
HARS	5136	3443	-0.16182689	2.34E-07	1.16E-06
TM9SF1	3324	2228	-0.162019018	3.13E-05	0.000115342
TOMM7	6542	4384	-0.162331829	4.83E-09	2.91E-08
MTMR12	2173	1456	-0.162525687	0.0007327	0.002071682
DAB2IP	2394	1604	-0.162596879	0.0003909	0.001165417
SYNPO	4539	3041	-0.16267654	1.03E-06	4.74E-06
ETS2	5520	3698	-0.162770911	7.03E-08	3.73E-07
BCL9L	7458	4996	-0.162863188	3.63E-10	2.43E-09
RASGRF2	5028	3368	-0.16294038	2.61E-07	1.29E-06
DKK1	3050	2043	-0.162967907	6.03E-05	0.000210081
ASXL1	4291	2874	-0.163101708	1.92E-06	8.50E-06
HMG20B	2801	1876	-0.163130024	0.0001191	0.000391495
SPON2	18833	12613	-0.163199239	1.80E-23	2.95E-22
RPL31	17660	11827	-0.163249041	4.03E-22	6.19E-21
NDUFA11	3717	2489	-0.163420326	8.98E-06	3.62E-05
ALDH2	2763	1850	-0.16355816	0.0001274	0.000415984

STEAP3	5426	3632	-0.163972713	7.34E-08	3.88E-07
DCTN2	6675	4468	-0.163986519	2.36E-09	1.46E-08
TRRAP	7223	4834	-0.164228566	4.97E-10	3.29E-09
ELK3	4332	2899	-0.164325778	1.44E-06	6.48E-06
ALMS1	2036	1362	-0.164858726	0.0009183	0.002542755
PVRL2	6642	4443	-0.16493145	2.11E-09	1.31E-08
NFIX	10632	7112	-0.164933649	3.51E-14	3.30E-13
MCAM	9420	6301	-0.164994118	9.65E-13	8.13E-12
PI4KA	4853	3246	-0.165061728	3.00E-07	1.48E-06
FGFRL1	2745	1836	-0.165087959	0.0001163	0.000383117
GNA12	6106	4084	-0.165092592	9.03E-09	5.24E-08
DDX23	4834	3233	-0.165191834	3.10E-07	1.52E-06
PTBP1	11709	7831	-0.165197284	1.65E-15	1.71E-14
PELP1	2001	1338	-0.16549092	0.0009736	0.0026797
SNX7	2372	1586	-0.165559107	0.0003278	0.000992962
SMARCC2	6387	4270	-0.165750249	3.60E-09	2.20E-08
PDXK	5499	3676	-0.165880388	4.25E-08	2.31E-07
NADK	3125	2089	-0.165891566	3.61E-05	0.000131456
UPF1	5435	3633	-0.165966538	5.01E-08	2.71E-07
MFSD10	2470	1651	-0.16601879	0.0002373	0.000735729
ANAPC5	6855	4582	-0.166027076	9.14E-10	5.89E-09
SHCBP1	2876	1922	-0.166303208	7.10E-05	0.000244112
CERS2	7630	5099	-0.166316587	9.69E-11	6.88E-10
PLD3	11721	7832	-0.16649086	9.77E-16	1.03E-14
SMPD1	6494	4339	-0.166592667	2.23E-09	1.39E-08
ARHGAP35	7558	5049	-0.166854709	1.04E-10	7.34E-10
MAGED1	35967	24025	-0.166985267	3.31E-45	1.10E-43
ELFN2	4155	2775	-0.167208574	1.58E-06	7.10E-06
MTHFD1	4070	2718	-0.167331207	1.99E-06	8.80E-06
PGK1	21426	14305	-0.16769091	7.98E-28	1.54E-26
TP53I11	3066	2047	-0.167694463	3.55E-05	0.000129456
JTB	4119	2750	-0.167710376	1.64E-06	7.33E-06

GIPC1	5597	3736	-0.168007161	2.18E-08	1.22E-07
TSPAN17	3702	2471	-0.168057753	5.27E-06	2.20E-05
HARS2	1966	1312	-0.16834347	0.000888	0.002468268
HSPG2	32556	21726	-0.168348739	1.04E-41	3.12E-40
TMEM109	2737	1826	-0.168756538	8.46E-05	0.000285857
C1orf63	2066	1378	-0.169112234	0.0006207	0.001778153
WBSCR16	2234	1490	-0.169164723	0.000371	0.001112138
COPZ2	1939	1293	-0.169438397	0.0008946	0.002483829
ARF5	2068	1379	-0.169461597	0.0006016	0.001728787
OSBPL5	2185	1457	-0.169480271	0.0004203	0.001245466
PHLDA2	2221	1481	-0.169485621	0.0003765	0.001127417
AHCYL1	6957	4639	-0.169499343	3.08E-10	2.08E-09
BIRC5	2500	1667	-0.169521859	0.0001609	0.000515874
RPL13	38811	25876	-0.169698856	3.92E-50	1.46E-48
HSBP1	6898	4599	-0.169705799	3.49E-10	2.34E-09
CNTNAP1	4832	3221	-0.169959662	1.44E-07	7.40E-07
ST3GAL1	3814	2542	-0.170188682	2.89E-06	1.25E-05
RIF1	5392	3593	-0.170479429	2.51E-08	1.40E-07
GSTM3	2029	1352	-0.170521582	0.0006285	0.001796508
POLE	1939	1292	-0.170554602	0.0008289	0.002318386
RPL13A	3632	2420	-0.170605023	4.72E-06	1.98E-05
STIP1	9325	6213	-0.170661539	2.20E-13	1.96E-12
NUP93	2535	1689	-0.170664287	0.0001309	0.000426779
TJP2	4195	2795	-0.170670396	8.63E-07	4.00E-06
TOB2	3559	2371	-0.170824138	5.73E-06	2.38E-05
RPS6KB1	2953	1967	-0.171032267	3.52E-05	0.000128711
APEH	3357	2236	-0.171100217	1.02E-05	4.07E-05
SLC35C1	2162	1440	-0.17114558	0.0003973	0.001182248
RPS14	21968	14631	-0.171223021	1.36E-29	2.78E-28
RPS16	15370	10236	-0.171312981	3.27E-21	4.75E-20
TANC1	4535	3020	-0.171401869	2.79E-07	1.38E-06
YPEL5	5082	3384	-0.171514675	5.28E-08	2.84E-07

ARHGAP11A	3783	2519	-0.171527523	2.66E-06	1.16E-05
WBSCR22	1864	1241	-0.171746613	0.0009672	0.002663971
TBCB	3506	2334	-0.171869303	5.93E-06	2.46E-05
VAV2	2719	1810	-0.171934322	6.61E-05	0.000228581
IDH1	5426	3612	-0.171939023	1.74E-08	9.82E-08
IKBKB	2321	1545	-0.171987553	0.0002267	0.000705209
EPN1	8580	5710	-0.17233477	1.21E-12	1.02E-11
CIRH1A	2529	1683	-0.172379729	0.0001146	0.000377761
HNRNPL	10449	6953	-0.172505252	4.26E-15	4.29E-14
KTN1	16040	10672	-0.172691438	2.18E-22	3.38E-21
SMARCA4	9872	6566	-0.173175513	1.92E-14	1.83E-13
NPR2	1833	1219	-0.173356528	0.0009597	0.002646706
CSGALNACT2	2839	1888	-0.173371953	3.96E-05	0.000142893
CTSA	6418	4268	-0.173411482	6.37E-10	4.17E-09
KEAP1	2746	1826	-0.173492728	5.24E-05	0.000184591
SCARA3	8473	5634	-0.173561174	1.18E-12	9.89E-12
PAK2	10595	7041	-0.174379208	1.41E-15	1.47E-14
TSPAN4	5135	3412	-0.174594498	2.62E-08	1.45E-07
GCN1L1	12393	8234	-0.174707981	5.07E-18	6.28E-17
PHF12	2065	1372	-0.174709168	0.0004134	0.001226269
SAV1	2890	1920	-0.17481105	2.91E-05	0.000108134
RPS23	24840	16499	-0.175134456	1.17E-34	2.81E-33
ECI2	3063	2034	-0.175473556	1.57E-05	6.05E-05
TOR1A	3411	2265	-0.175531573	5.13E-06	2.15E-05
RC3H2	3112	2066	-0.175849674	1.29E-05	5.03E-05
FBN2	16994	11282	-0.175850496	2.06E-24	3.51E-23
SOCS6	3341	2218	-0.175868485	6.16E-06	2.54E-05
PLXND1	4345	2884	-0.176132881	2.43E-07	1.21E-06
RAB13	9594	6368	-0.176139972	1.68E-14	1.61E-13
NARS	10411	6910	-0.176198902	1.29E-15	1.35E-14
VEPH1	2367	1571	-0.176224394	0.0001375	0.000446862
DYNLL1	10578	7020	-0.176371813	7.21E-16	7.69E-15

SHC3	3115	2067	-0.176541643	1.18E-05	4.64E-05
PCYT2	1801	1195	-0.176635431	0.0008587	0.002393608
SRSF2	7281	4831	-0.176662625	2.04E-11	1.55E-10
TRPV2	3678	2440	-0.1768882	1.84E-06	8.18E-06
ARFGAP1	5403	3584	-0.177037913	7.20E-09	4.24E-08
U2AF1	2982	1978	-0.1770857	1.71E-05	6.58E-05
PEPD	1932	1281	-0.177672487	0.0005168	0.001502817
RPS4X	28542	18923	-0.177793472	1.08E-40	3.16E-39
RAVER1	2715	1800	-0.17780316	3.81E-05	0.000138025
FDPS	2336	1548	-0.178482671	0.0001258	0.000411622
TNS3	11404	7557	-0.178502285	2.37E-17	2.79E-16
EIF2B3	2306	1528	-0.178595838	0.0001377	0.000447427
TOMM5	3466	2296	-0.178996881	2.81E-06	1.22E-05
VAT1	14358	9511	-0.17903373	1.48E-21	2.20E-20
ATPAF1	1804	1195	-0.179036589	0.0007252	0.002052315
CNPY2	3699	2450	-0.179201419	1.27E-06	5.77E-06
GRAMD1A	2200	1457	-0.179350515	0.0001849	0.000584944
GOLGB1	12612	8352	-0.179451326	3.32E-19	4.36E-18
C6orf48	3665	2427	-0.179486958	1.37E-06	6.19E-06
RNF167	2497	1653	-0.179956965	6.44E-05	0.000222962
PRRC2A	18534	12268	-0.180122073	1.17E-27	2.24E-26
PDDC1	2576	1705	-0.180208722	4.81E-05	0.000170778
PRDX5	4678	3096	-0.180334258	4.23E-08	2.30E-07
MAST2	3566	2360	-0.180367712	1.70E-06	7.58E-06
NEK9	5860	3878	-0.180435729	8.36E-10	5.42E-09
SLC20A1	13803	9133	-0.180668992	4.00E-21	5.77E-20
PPIL2	1977	1308	-0.180798198	0.0003543	0.001065458
FIS1	2960	1958	-0.181064279	1.20E-05	4.73E-05
KRTCAP2	2041	1350	-0.181164643	0.0002764	0.000848443
PRCC	1976	1307	-0.181171674	0.000346	0.00104311
FOXK1	6929	4583	-0.181202739	2.06E-11	1.56E-10
SMARCAL1	1946	1287	-0.181347525	0.000379	0.00113373

GAS5	2876	1902	-0.181394298	1.55E-05	5.98E-05
KIF3C	2474	1636	-0.18152062	6.04E-05	0.000210226
CSF1	1665	1101	-0.181555577	0.0009973	0.002740116
TPX2	6051	4001	-0.181660824	3.43E-10	2.30E-09
DST	31608	20899	-0.181703707	9.83E-47	3.41E-45
SRP68	5236	3462	-0.181707241	5.17E-09	3.10E-08
ADCY7	3025	2000	-0.181783011	8.92E-06	3.60E-05
CD2BP2	4105	2714	-0.181809369	2.28E-07	1.14E-06
TBC1D5	4450	2942	-0.181855958	7.07E-08	3.75E-07
NCAPD2	4975	3289	-0.181895386	1.20E-08	6.87E-08
SNX17	6101	4033	-0.182040193	2.67E-10	1.82E-09
PKM	124671	82409	-0.182099974	7.67E-180	1.27E-177
RBCK1	3761	2486	-0.182137878	6.97E-07	3.26E-06
RAB8A	3634	2402	-0.182170137	1.07E-06	4.90E-06
ERAL1	1699	1123	-0.182175793	0.0008508	0.002374743
SLC6A8	2781	1838	-0.182314846	1.95E-05	7.43E-05
EMC3	2595	1715	-0.18237383	3.68E-05	0.000133632
HOMER3	2406	1590	-0.182457745	7.02E-05	0.000241537
RND3	24358	16095	-0.182630972	9.41E-37	2.38E-35
STIM2	1668	1102	-0.182842933	0.0009103	0.00252228
LSM14B	1656	1094	-0.182937803	0.0009444	0.002608675
BAZ1A	5846	3862	-0.182949533	5.19E-10	3.42E-09
SNPH	1927	1273	-0.182972021	0.0003603	0.001082733
MRPL17	2509	1657	-0.183386736	4.51E-05	0.000160523
LSM4	3042	2009	-0.183390457	7.04E-06	2.88E-05
GYS1	6066	4006	-0.183430945	2.22E-10	1.53E-09
PUF60	4181	2761	-0.183505024	1.37E-07	7.01E-07
TPMT	2093	1382	-0.183662564	0.0001902	0.000599718
CDK1	2302	1520	-0.183664378	9.09E-05	0.000305731
CCT3	14441	9534	-0.183864986	9.85E-23	1.56E-21
HGS	5370	3545	-0.183984329	2.13E-09	1.33E-08
EBPL	1633	1078	-0.184015481	0.0009591	0.00264564

TYK2	3057	2018	-0.184038246	6.22E-06	2.56E-05
SNHG8	1627	1074	-0.184068126	0.0009769	0.002688176
CORO1B	4107	2711	-0.184107702	1.61E-07	8.18E-07
UBXN1	2574	1699	-0.184174069	3.33E-05	0.000122523
HSPA8	93449	61665	-0.184575221	9.17E-139	1.06E-136
GEMIN5	2181	1439	-0.184771046	0.0001273	0.000416089
IPO13	1975	1303	-0.184863438	0.0002644	0.000813441
COPE	5172	3412	-0.184952497	3.50E-09	2.14E-08
STAT2	5859	3865	-0.185033904	3.20E-10	2.15E-09
HERC2P2	1651	1089	-0.185184034	0.0008353	0.002334712
RPS3	32799	21634	-0.185199262	3.50E-50	1.30E-48
VAC14	1680	1108	-0.18535122	0.0007436	0.002101469
TNK2	1797	1185	-0.185551218	0.0004792	0.001404395
ZMYM3	2698	1779	-0.185671706	1.86E-05	7.09E-05
MVP	22623	14916	-0.185777422	2.38E-35	5.79E-34
NAGK	2310	1523	-0.185824778	7.34E-05	0.000251642
SNRPA	1928	1271	-0.185988889	0.0002885	0.000882411
MGRN1	4796	3161	-0.186298492	1.02E-08	5.89E-08
DNPEP	1672	1102	-0.186298492	0.0007207	0.002040935
CLIP3	8063	5314	-0.186366363	1.09E-13	9.93E-13
RAB31	3505	2310	-0.186369461	9.68E-07	4.46E-06
TNPO2	5590	3684	-0.18642309	6.12E-10	4.01E-09
EWSR1	9272	6110	-0.186556055	1.53E-15	1.59E-14
NIPBL	6086	4010	-0.186739965	1.00E-10	7.09E-10
IVNS1ABP	4472	2946	-0.187010626	2.84E-08	1.57E-07
SGOL2	2022	1332	-0.187036783	0.0001892	0.000596792
CERCAM	7909	5209	-0.187336726	1.42E-13	1.27E-12
MCM5	1775	1169	-0.187391963	0.0004584	0.001348656
RPL3	54882	36144	-0.187424749	1.17E-84	7.82E-83
SHC1	32496	21399	-0.187566634	6.28E-51	2.38E-49
DDX10	1625	1070	-0.18767679	0.0007863	0.002208617
OPA3	1584	1043	-0.187681046	0.0009163	0.002537928

SLC7A2	2040	1343	-0.187957716	0.0001649	0.000527144
DIDO1	5113	3366	-0.187982718	2.44E-09	1.51E-08
OGFR	2154	1418	-0.188008586	0.0001078	0.000357821
TOX2	2124	1398	-0.188267274	0.000118	0.000388243
PIEZO1	14660	9648	-0.188431159	4.27E-24	7.15E-23
VCAN	37903	24944	-0.188467076	1.22E-59	5.41E-58
TSEN34	2026	1333	-0.188805262	0.0001627	0.000521029
FAU	9468	6229	-0.188907002	3.45E-16	3.75E-15
PPIP5K2	5735	3773	-0.188929254	2.17E-10	1.50E-09
UCHL1	17494	11508	-0.189070941	1.29E-28	2.56E-27
APAF1	3691	2428	-0.189091184	3.45E-07	1.68E-06
NDUFS8	2297	1511	-0.189095064	5.79E-05	0.000202539
NQO1	9138	6011	-0.189121291	1.04E-15	1.10E-14
SNRNP70	6300	4144	-0.189175694	2.69E-11	2.02E-10
CAT	3486	2293	-0.189184083	7.20E-07	3.36E-06
TOMM40	3096	2036	-0.189515778	2.89E-06	1.25E-05
SQLE	2374	1561	-0.189697266	4.12E-05	0.000148328
OGDH	5174	3402	-0.189744781	1.40E-09	8.90E-09
SEH1L	2003	1317	-0.189754944	0.0001648	0.000526995
BAX	3495	2298	-0.189761526	6.46E-07	3.03E-06
TCF12	8121	5339	-0.189935708	3.11E-14	2.93E-13
DNAJC14	2227	1464	-0.190033873	6.94E-05	0.000239001
INTS3	4678	3075	-0.190153319	7.94E-09	4.65E-08
NUP188	5097	3350	-0.190335126	1.66E-09	1.04E-08
SOD2	4466	2935	-0.190470616	1.64E-08	9.29E-08
C1R	2424	1593	-0.1904913	3.18E-05	0.000117129
HRH1	1651	1085	-0.190492946	0.000596	0.001715046
CDK14	2587	1700	-0.190593176	1.71E-05	6.57E-05
UQCRC1	7171	4712	-0.190682647	7.90E-13	6.73E-12
AP1S1	4811	3161	-0.190803632	4.35E-09	2.62E-08
CHID1	3288	2160	-0.191026855	1.18E-06	5.39E-06
B3GALT6	2020	1327	-0.191034791	0.0001399	0.00045404

FTH1	85554	56196	-0.191215733	4.73E-136	5.30E-134
FLII	11735	7708	-0.191237233	3.96E-20	5.42E-19
ASAP1	10703	7030	-0.191266508	1.68E-18	2.14E-17
RABAC1	5008	3289	-0.191433422	1.87E-09	1.17E-08
NOP10	3012	1978	-0.191527212	3.13E-06	1.35E-05
PLEKHG5	1564	1027	-0.191652199	0.0007754	0.002181635
LTBP1	73132	48019	-0.191745326	3.56E-117	3.34E-115
MTRNR2L2	2274	1493	-0.191865957	4.96E-05	0.000175624
SLC38A10	6398	4199	-0.192423073	8.77E-12	6.87E-11
FLYWCH1	2054	1348	-0.192463553	0.0001098	0.000363383
UBE2J2	2906	1907	-0.192577728	4.16E-06	1.76E-05
GLUD1	10900	7152	-0.192757362	4.49E-19	5.87E-18
WTAP	3929	2578	-0.192757772	8.43E-08	4.43E-07
EIF2B1	2367	1553	-0.192849745	3.18E-05	0.000117099
EFR3A	8674	5691	-0.192863132	1.65E-15	1.71E-14
EMILIN1	15260	10012	-0.192872634	4.29E-26	7.83E-25
TXN	9156	6007	-0.192920667	2.71E-16	2.96E-15
FBL	2741	1798	-0.193157176	7.33E-06	2.99E-05
STK25	5490	3601	-0.193256418	2.17E-10	1.50E-09
SRM	6755	4430	-0.193496939	1.77E-12	1.48E-11
GSS	2670	1751	-0.193508526	9.29E-06	3.73E-05
TUBGCP6	2187	1434	-0.193756065	5.89E-05	0.000205487
WDR36	5983	3923	-0.193759596	3.04E-11	2.26E-10
TXNL4A	3514	2304	-0.193821343	3.51E-07	1.71E-06
CLSTN3	1693	1110	-0.193870165	0.0004056	0.001204512
UBQLN1	10735	7038	-0.19393264	5.17E-19	6.74E-18
FARSA	2978	1952	-0.194238569	2.62E-06	1.14E-05
RPLP2	18312	12003	-0.194242202	2.19E-31	4.84E-30
ETNK1	4339	2844	-0.194288989	1.40E-08	7.97E-08
CAPN2	61607	40377	-0.19440843	1.28E-101	1.01E-99
MMP16	2910	1907	-0.194562178	3.28E-06	1.41E-05
USMG5	1732	1135	-0.194594501	0.0003309	0.001001285

FADD	1639	1074	-0.194669729	0.0004769	0.001398498
CSTF3	2849	1866	-0.195354504	3.81E-06	1.62E-05
PIK3R2	6495	4254	-0.195357361	2.99E-12	2.44E-11
ELOVL5	5378	3522	-0.195522714	2.08E-10	1.44E-09
PRICKLE2	2393	1567	-0.195663085	2.22E-05	8.39E-05
HK1	9556	6257	-0.195783618	2.20E-17	2.60E-16
C6orf89	9352	6123	-0.195884115	4.63E-17	5.31E-16
PPDPF	9753	6385	-0.196007305	9.57E-18	1.16E-16
CASP8AP2	1867	1222	-0.196325511	0.0001705	0.000542832
TK2	2333	1527	-0.196334113	2.64E-05	9.88E-05
BAG6	8889	5818	-0.196345699	2.33E-16	2.57E-15
VIM	321181	210210	-0.196403117	0	0
ARNT	2651	1735	-0.196448876	7.39E-06	3.01E-05
SRCAP	6442	4216	-0.196481662	2.78E-12	2.29E-11
RAD50	3345	2189	-0.196582125	4.70E-07	2.25E-06
SOLH	2384	1560	-0.196686075	2.09E-05	7.93E-05
GADD45GIP1	2485	1626	-0.196766463	1.38E-05	5.39E-05
PANK2	1588	1039	-0.196863126	0.0005088	0.001482113
COX6A1	4202	2749	-0.197017123	1.52E-08	8.66E-08
DGKZ	2851	1865	-0.197140277	3.10E-06	1.34E-05
OBSCN	1596	1044	-0.197186808	0.0004824	0.001412395
S100A10	8491	5554	-0.197255164	8.02E-16	8.53E-15
ELMSAN1	2205	1442	-0.197555359	3.96E-05	0.000142882
BTRC	3069	2007	-0.197575897	1.24E-06	5.62E-06
GNL1	4161	2721	-0.1976412	1.62E-08	9.19E-08
C12orf44	1953	1277	-0.197781292	0.0001078	0.000357732
GLRX	4015	2625	-0.197930433	2.76E-08	1.53E-07
CUEDC2	1629	1065	-0.197981042	0.0004002	0.00118987
LOC728392	11059	7230	-0.198001253	2.83E-20	3.93E-19
TIMM50	1978	1293	-0.198168019	9.45E-05	0.000316335
PAF1	3274	2140	-0.198291393	5.00E-07	2.38E-06
MXD4	5673	3708	-0.198318488	3.66E-11	2.70E-10

MUM1	2093	1368	-0.19835195	5.82E-05	0.000203519
RNPS1	4359	2849	-0.19838945	6.54E-09	3.87E-08
CASC5	2497	1632	-0.198402632	1.12E-05	4.44E-05
TMED1	1498	979	-0.198504727	0.0006658	0.001895755
PHC1	1567	1024	-0.198637333	0.0004957	0.001449385
C22orf28	5101	3333	-0.198806652	3.18E-10	2.14E-09
RASA1	8182	5346	-0.198841568	1.62E-15	1.68E-14
PTDSS1	6194	4047	-0.198866402	4.12E-12	3.33E-11
COMMD2	1575	1029	-0.198956715	0.0004699	0.001380664
MCC	2486	1624	-0.199122532	1.10E-05	4.33E-05
SCFD2	1854	1211	-0.199290247	0.0001443	0.000466642
FBLN1	4087	2669	-0.199590806	1.59E-08	9.05E-08
GAPDH	212108	138500	-0.199760927	0	0
PPP1CA	4240	2768	-0.20006819	7.96E-09	4.66E-08
FCGRT	1777	1160	-0.200166744	0.0001865	0.000589005
PLK1	1946	1270	-0.200531081	8.96E-05	0.000301793
STX4	2483	1620	-0.200938317	9.27E-06	3.72E-05
THY1	26894	17543	-0.201236733	2.25E-48	8.11E-47
SGTA	3461	2257	-0.201630391	1.51E-07	7.69E-07
PNPLA6	5325	3472	-0.201862446	6.96E-11	4.99E-10
MRPS21	1957	1276	-0.201863295	7.70E-05	0.000263007
COG1	2842	1853	-0.201891541	1.89E-06	8.38E-06
PRR12	2813	1834	-0.201963782	2.11E-06	9.31E-06
FXR2	3105	2024	-0.202231847	6.08E-07	2.86E-06
NDUFB11	2002	1305	-0.202240036	6.18E-05	0.000214752
SKIV2L	3214	2095	-0.202267553	3.85E-07	1.87E-06
PRMT2	5316	3465	-0.202333621	6.55E-11	4.70E-10
WDR41	2547	1660	-0.202463586	6.09E-06	2.52E-05
FOXF2	2044	1332	-0.202648982	5.00E-05	0.00017713
ABL1	7876	5132	-0.202789809	1.63E-15	1.69E-14
SH3BGRL3	25380	16535	-0.203016892	1.67E-46	5.71E-45
GRN	9037	5887	-0.203159163	1.24E-17	1.50E-16

MAP1S	1529	996	-0.203218628	0.0004363	0.001287596
DLGAP4	7068	4604	-0.203262075	3.91E-14	3.66E-13
NIN	11068	7209	-0.203371363	2.79E-21	4.07E-20
CHTF8	3172	2066	-0.203400385	3.97E-07	1.92E-06
TELO2	1477	962	-0.203408895	0.0005404	0.001566955
PMPCA	3126	2036	-0.203428085	4.80E-07	2.29E-06
RPS2	85143	55452	-0.203496297	1.90E-152	2.57E-150
TFE3	6340	4129	-0.20353829	7.33E-13	6.27E-12
ATG7	1686	1098	-0.20357435	0.000216	0.000675131
PPP2R5D	3509	2285	-0.203713651	9.26E-08	4.84E-07
SRSF9	2216	1443	-0.20373445	2.19E-05	8.30E-05
LRRC41	5237	3410	-0.203816734	6.67E-11	4.79E-10
KIF22	1350	879	-0.203872205	0.0009162	0.002537977
PRR13	4474	2913	-0.203907423	1.58E-09	9.96E-09
AHDC1	2065	1344	-0.204456512	3.93E-05	0.000142183
NOC2L	5255	3420	-0.204542307	5.33E-11	3.87E-10
HIC1	2182	1420	-0.20460804	2.34E-05	8.83E-05
PRKD1	1488	968	-0.205143442	0.0004629	0.001361157
KLHL36	1339	871	-0.205259205	0.0008899	0.002472309
FAM178A	5108	3321	-0.20598867	7.36E-11	5.28E-10
MINK1	5670	3686	-0.206140532	6.55E-12	5.21E-11
LAP3	3883	2524	-0.206307665	1.29E-08	7.38E-08
MRPL4	1742	1132	-0.206718534	0.0001354	0.000440255
BAHCC1	1365	887	-0.20674281	0.0007283	0.002060593
SUMF1	1676	1089	-0.206866063	0.0001797	0.00057008
SNX29	2423	1574	-0.207206732	6.44E-06	2.65E-05
YY1AP1	2184	1418	-0.207963192	1.72E-05	6.62E-05
TRPM7	4955	3217	-0.20801699	9.43E-11	6.70E-10
MCM2	1548	1005	-0.208057838	0.0002944	0.000899302
SREBF1	1841	1195	-0.208326877	7.72E-05	0.000263539
DDAH2	2057	1335	-0.20854992	2.88E-05	0.000107079
NOMO2	2564	1664	-0.208588697	3.00E-06	1.30E-05

LY6E	5162	3350	-0.208616914	3.40E-11	2.52E-10
MKI67	12434	8068	-0.208855363	7.13E-25	1.24E-23
RPTOR	3058	1984	-0.209024249	3.20E-07	1.57E-06
RNH1	6534	4239	-0.209090357	7.81E-14	7.15E-13
WIZ	2995	1943	-0.20911797	4.19E-07	2.02E-06
FGD1	1691	1097	-0.209161002	0.000143	0.000462842
SF3B2	12548	8140	-0.209204603	3.67E-25	6.47E-24
ANKRD10	1961	1272	-0.209338733	4.15E-05	0.000149284
ERCC1	2703	1753	-0.209583384	1.45E-06	6.55E-06
SPG7	4253	2758	-0.209706266	1.48E-09	9.37E-09
LRP3	1630	1057	-0.209744456	0.0001813	0.000574583
NUMA1	10526	6825	-0.209904217	1.71E-21	2.52E-20
NSMAF	2335	1514	-0.209905213	7.32E-06	2.98E-05
EP400	6877	4459	-0.209907014	1.40E-14	1.35E-13
LENG8	5422	3515	-0.210148292	7.86E-12	6.20E-11
NAV1	14451	9367	-0.210358228	5.11E-29	1.03E-27
CPSF1	4647	3012	-0.210425743	2.28E-10	1.57E-09
RPL37	19172	12425	-0.210602862	4.97E-38	1.33E-36
COL3A1	55298	35837	-0.2106253	2.33E-106	1.96E-104
COL6A1	49136	31841	-0.21074264	8.22E-95	6.09E-93
ESYT1	14901	9656	-0.21075944	5.66E-30	1.18E-28
TBC1D2B	3889	2520	-0.210823369	6.19E-09	3.67E-08
BOD1L1	7712	4997	-0.210890702	2.69E-16	2.94E-15
SERPINF1	1957	1268	-0.210936879	3.71E-05	0.000134663
ADAM23	1812	1174	-0.210998415	7.19E-05	0.000246849
RPL35	11921	7723	-0.211119854	2.21E-24	3.75E-23
TSTA3	1962	1271	-0.211208879	3.55E-05	0.000129539
MED24	2402	1556	-0.211241959	4.74E-06	1.99E-05
SPAG5	2612	1692	-0.211273197	1.82E-06	8.09E-06
MCM7	3781	2449	-0.211422944	9.14E-09	5.30E-08
SAP30L	2021	1309	-0.211452093	2.65E-05	9.91E-05
EP300	6102	3952	-0.211547101	2.80E-13	2.47E-12

SEMA3F	3848	2492	-0.211652599	6.52E-09	3.86E-08
DCUN1D1	2233	1446	-0.211763567	9.75E-06	3.89E-05
ROBO1	7717	4995	-0.212403296	1.64E-16	1.81E-15
TUBB	98878	63991	-0.212628419	3.51E-192	6.62E-190
ARIH2	4035	2611	-0.212814084	2.32E-09	1.44E-08
GLYR1	5246	3394	-0.213079111	9.14E-12	7.14E-11
DDX54	2144	1387	-0.213184986	1.29E-05	5.05E-05
GNB2	11076	7165	-0.213246217	3.52E-23	5.69E-22
ABR	6657	4306	-0.213371717	1.45E-14	1.39E-13
SYMPK	3528	2282	-0.213399638	2.14E-08	1.20E-07
RCOR1	3015	1950	-0.213531746	2.22E-07	1.11E-06
BRD9	2344	1516	-0.213550685	4.92E-06	2.06E-05
VPS52	1731	1119	-0.214243557	8.23E-05	0.000278994
CLUH	3131	2024	-0.214262087	1.18E-07	6.11E-07
TRIP6	4765	3080	-0.214393731	6.24E-11	4.50E-10
VPS28	1671	1080	-0.214528289	0.0001071	0.000355732
KIF4A	1962	1268	-0.214618164	2.68E-05	0.000100042
CKAP2	5105	3299	-0.214730051	1.22E-11	9.46E-11
SECISBP2	1758	1136	-0.214820104	6.93E-05	0.000238895
VSTM4	1441	931	-0.215065131	0.0003109	0.000945653
PKI55	1305	843	-0.215293139	0.0005922	0.001706283
C16orf45	1336	863	-0.215335412	0.0005083	0.001480786
MED13	6115	3950	-0.215347714	1.02E-13	9.28E-13
BTG1	4365	2819	-0.215646044	3.12E-10	2.10E-09
MRPL30	2843	1836	-0.215695909	3.79E-07	1.84E-06
UQCRQ	3269	2111	-0.215770667	5.08E-08	2.74E-07
CEP170B	5092	3288	-0.215869988	1.02E-11	7.96E-11
ETHE1	2260	1459	-0.216190758	5.66E-06	2.35E-05
AFF3	5284	3411	-0.21628358	3.83E-12	3.11E-11
UBL5	3910	2524	-0.216304565	2.33E-09	1.44E-08
SLC25A22	1670	1078	-0.216338793	9.46E-05	0.000316518
WDR37	2498	1612	-0.216769601	1.72E-06	7.65E-06

TRIP12	20616	13303	-0.216860682	4.99E-43	1.54E-41
RPL41	35958	23201	-0.216973659	8.45E-74	4.74E-72
RBM41	1522	982	-0.217021297	0.0001852	0.00058549
STAT6	5876	3791	-0.217103808	2.00E-13	1.79E-12
TRABD	1302	840	-0.217116084	0.0005419	0.001570632
FOSL1	7936	5120	-0.217116084	1.33E-17	1.60E-16
LTBP3	5297	3417	-0.217293129	2.89E-12	2.37E-11
PHRF1	3268	2108	-0.217380985	4.09E-08	2.23E-07
ARPC1B	6597	4255	-0.217498841	6.19E-15	6.15E-14
SNRPD1	2932	1891	-0.217583608	1.97E-07	9.93E-07
PHF19	1811	1168	-0.21759414	4.35E-05	0.000155376
USP5	6858	4423	-0.21761053	1.77E-15	1.82E-14
RBM10	2824	1821	-0.217857035	3.20E-07	1.57E-06
SUCO	7058	4551	-0.217923712	6.31E-16	6.76E-15
ZDHHC8	3032	1955	-0.217949014	1.17E-07	6.06E-07
RIC8A	4780	3082	-0.217991625	2.85E-11	2.13E-10
SBF1	6570	4236	-0.218038649	6.06E-15	6.03E-14
NME4	5312	3424	-0.218420313	2.10E-12	1.74E-11
SCAP	3062	1973	-0.218931195	8.92E-08	4.67E-07
COL6A2	77416	49878	-0.219076018	1.16E-159	1.64E-157
EDF1	6566	4230	-0.219204955	4.49E-15	4.51E-14
ATP6V0A2	1515	976	-0.219212609	0.0001658	0.000529904
ALDH3B1	1779	1146	-0.219307335	4.45E-05	0.000158844
NOL6	4693	3023	-0.21937735	3.28E-11	2.43E-10
MBOAT7	7937	5112	-0.219553837	5.95E-18	7.33E-17
PRPF19	3447	2220	-0.21962949	1.26E-08	7.21E-08
ACSS2	1691	1089	-0.219720574	6.67E-05	0.00023039
FTSJ3	4895	3152	-0.219889193	1.12E-11	8.70E-11
IER5L	1317	848	-0.219967044	0.0004267	0.001261593
METTL3	1519	978	-0.220063368	0.0001536	0.000494089
GTF3C5	1815	1168	-0.220777142	3.32E-05	0.000122039
VWA5A	3245	2088	-0.220944635	2.80E-08	1.55E-07

KLHL21	3197	2057	-0.221024819	3.50E-08	1.92E-07
HNRNPUL1	14248	9167	-0.221085711	2.42E-31	5.32E-30
DAXX	2741	1763	-0.221517722	3.11E-07	1.52E-06
WAPAL	5070	3261	-0.221519174	3.42E-12	2.78E-11
RNF2	1404	903	-0.221592911	0.000249	0.000769433
LAMTOR4	1485	955	-0.221738161	0.0001633	0.000522773
ZNF142	1356	872	-0.221805007	0.000314	0.000953622
AGPAT5	1199	771	-0.221876762	0.0007002	0.001987486
CAMK2G	2451	1576	-0.221950818	1.25E-06	5.67E-06
BRF1	1243	799	-0.222406756	0.0005421	0.001570841
MTX3	2592	1666	-0.222525186	5.80E-07	2.74E-06
SORBS3	5046	3243	-0.222659051	3.01E-12	2.46E-11
HTRA1	5043	3241	-0.222691073	3.03E-12	2.48E-11
HOXB6	1273	818	-0.222907539	0.0004513	0.001329366
FAM105B	2020	1298	-0.222912778	9.90E-06	3.95E-05
HIAT1	2489	1599	-0.22324416	8.99E-07	4.15E-06
ARID4B	5403	3471	-0.223257185	4.54E-13	3.94E-12
TFIP11	1935	1243	-0.223355138	1.47E-05	5.70E-05
GTF3A	2950	1895	-0.223364974	8.75E-08	4.59E-07
FEN1	1468	943	-0.22337016	0.0001602	0.000513969
FAM171A1	3901	2505	-0.223881263	6.96E-10	4.53E-09
RGL1	1576	1012	-0.223906113	8.85E-05	0.000298328
PCOLCE	6725	4318	-0.224018893	5.40E-16	5.81E-15
TMEM158	4863	3122	-0.224223923	5.36E-12	4.28E-11
DAPK3	3935	2526	-0.224356865	5.42E-10	3.56E-09
ARHGEF18	2209	1418	-0.224383754	3.31E-06	1.42E-05
EPB41L1	2921	1875	-0.224419631	8.85E-08	4.64E-07
DLL4	3503	2248	-0.224796821	4.44E-09	2.68E-08
CLN8	1764	1132	-0.224824471	3.13E-05	0.000115485
TRAPPC1	3871	2484	-0.224889002	6.83E-10	4.45E-09
TMA7	5163	3313	-0.224919263	1.03E-12	8.66E-12
KIAA0146	1523	977	-0.225333343	0.0001056	0.000351073

C12orf10	1277	819	-0.225671036	0.0003773	0.001129269
MID1	3212	2060	-0.225675424	1.71E-08	9.67E-08
CAPG	1469	942	-0.225883299	0.0001351	0.000439731
UBA52	12960	8310	-0.225993204	7.99E-30	1.65E-28
RPS19	27851	17858	-0.226006482	4.07E-62	1.87E-60
TOP3A	1190	763	-0.22605448	0.0005864	0.001690946
PITPNM3	1424	913	-0.226110249	0.0001688	0.000538366
POLR2J	1123	720	-0.226136984	0.0008353	0.002335223
SCARF2	2976	1908	-0.226161223	5.35E-08	2.88E-07
DCUN1D3	1560	1000	-0.226393897	8.08E-05	0.000274827
TP53I3	2727	1748	-0.226457384	1.86E-07	9.36E-07
MICAL1	3423	2194	-0.226545635	5.13E-09	3.08E-08
KREMEN1	1406	901	-0.226845452	0.0001772	0.000562593
ANAPC2	1358	870	-0.227244042	0.0002233	0.000695149
NDUFS7	1163	745	-0.227386634	0.0006312	0.001803423
TGFBI	75134	48125	-0.227527238	1.48E-166	2.24E-164
AKT1	9650	6181	-0.227536546	6.70E-23	1.08E-21
SH2B3	4690	3004	-0.227550978	6.49E-12	5.16E-11
AKR7A2	1972	1263	-0.227652781	8.36E-06	3.39E-05
RPS6KB2	2072	1327	-0.227703501	4.92E-06	2.06E-05
AKIRIN2	1735	1111	-0.227924714	2.86E-05	0.000106443
COPS6	8507	5447	-0.228036455	1.86E-20	2.61E-19
YIF1A	3311	2120	-0.228050614	7.33E-09	4.31E-08
FKBP2	1882	1205	-0.22808135	1.30E-05	5.07E-05
CDK4	4356	2787	-0.22913882	2.67E-11	2.00E-10
MRPS2	1418	907	-0.229530945	0.00014	0.000454056
RAB22A	5845	3738	-0.229784324	9.93E-15	9.66E-14
LIX1L	1298	830	-0.22995501	0.0002623	0.000807632
RNASEH1	2019	1291	-0.229999778	5.29E-06	2.21E-05
SPATA20	3097	1980	-0.230218821	1.66E-08	9.41E-08
PTPN23	3404	2176	-0.230400349	3.18E-09	1.95E-08
WRB	2078	1328	-0.230788376	3.58E-06	1.53E-05

MDK	2573	1644	-0.231089026	2.43E-07	1.21E-06
H2AFZ	10441	6671	-0.231132816	2.39E-25	4.23E-24
RNF114	4915	3140	-0.231274726	9.21E-13	7.78E-12
USP19	2467	1576	-0.231338049	4.17E-07	2.01E-06
LOXL3	1204	769	-0.231627757	0.0004004	0.001190108
PSMB5	7428	4744	-0.231717674	1.42E-18	1.81E-17
TPI1	28759	18367	-0.231745379	3.74E-67	1.89E-65
PHPT1	2731	1744	-0.231877142	9.46E-08	4.94E-07
PHB2	6007	3836	-0.231889812	2.46E-15	2.51E-14
ADCY6	4587	2929	-0.231990582	4.52E-12	3.63E-11
SDHA	5250	3352	-0.232143142	1.28E-13	1.16E-12
YIF1B	2113	1349	-0.232250287	2.58E-06	1.13E-05
ALDH1B1	1540	983	-0.232514897	5.87E-05	0.000204759
TNFRSF1A	8145	5199	-0.232528412	2.42E-20	3.37E-19
PCNXL3	3713	2370	-0.232546124	4.37E-10	2.91E-09
MUS81	1092	697	-0.232590163	0.0007128	0.002019457
DENND4B	1603	1023	-0.232816149	4.05E-05	0.000145913
KPNA2	10752	6861	-0.23296214	1.99E-26	3.66E-25
SIRT2	2017	1287	-0.233046899	4.05E-06	1.72E-05
NDUFAF3	1627	1038	-0.233255676	3.43E-05	0.000125728
CDK16	4751	3031	-0.233285223	1.44E-12	1.20E-11
SPECC1	3157	2014	-0.233338446	7.80E-09	4.57E-08
KIAA1279	3791	2418	-0.23361208	2.40E-10	1.65E-09
OAZ1	22098	14094	-0.233682552	7.99E-53	3.14E-51
PDGFRA	19900	12690	-0.23392423	7.76E-48	2.75E-46
LRRC8C	6160	3927	-0.234350621	5.61E-16	6.03E-15
MAN2A2	2281	1454	-0.234487046	8.22E-07	3.82E-06
F2RL2	2551	1626	-0.23458351	1.83E-07	9.24E-07
UBE2M	2870	1829	-0.23484353	3.06E-08	1.68E-07
RPS6KA4	2660	1695	-0.234988841	9.56E-08	4.99E-07
FASN	8880	5658	-0.235115368	1.79E-22	2.80E-21
POLRMT	1422	906	-0.235186378	9.47E-05	0.000316938

EXOG	1364	869	-0.23526343	0.0001311	0.000427112
BCKDK	2237	1425	-0.235451205	9.51E-07	4.38E-06
TENC1	3507	2234	-0.235456113	8.39E-10	5.44E-09
LAMA5	4865	3099	-0.235484918	4.85E-13	4.20E-12
C17orf62	1719	1095	-0.235486543	1.73E-05	6.64E-05
TUBB6	15234	9704	-0.235491213	1.76E-37	4.56E-36
LOXL1	11709	7457	-0.23579849	2.88E-29	5.81E-28
PIAS3	4034	2569	-0.235852062	4.31E-11	3.16E-10
STXBP1	4050	2579	-0.235958003	3.87E-11	2.85E-10
XPO7	6236	3971	-0.235966435	2.38E-16	2.62E-15
EMG1	1024	652	-0.236119714	0.0008834	0.002457097
ARHGEF40	3961	2522	-0.236144294	6.12E-11	4.42E-10
ZFP36L2	6570	4183	-0.236203241	3.57E-17	4.12E-16
AGRN	22004	14009	-0.2362597	1.09E-53	4.37E-52
ZNF598	1643	1046	-0.236297497	2.50E-05	9.36E-05
DNMT1	6163	3923	-0.236523324	3.05E-16	3.33E-15
FBLN2	1073	683	-0.236540461	0.0006504	0.001854456
FBXO21	2591	1649	-0.236765485	1.14E-07	5.89E-07
RNF40	5624	3579	-0.236887919	5.39E-15	5.39E-14
G6PC3	3217	2047	-0.237052702	3.29E-09	2.02E-08
FTL	84826	53970	-0.237196714	2.96E-203	6.15E-201
SCYL1	5149	3276	-0.237204781	6.91E-14	6.35E-13
OSBP	6138	3905	-0.237293966	2.83E-16	3.09E-15
MICALL2	1654	1052	-0.237672398	2.11E-05	8.02E-05
PGD	8650	5501	-0.237854098	2.17E-22	3.38E-21
PITPNC1	1321	840	-0.238017102	0.0001413	0.000457763
TATDN2	3647	2319	-0.238055248	2.53E-10	1.73E-09
UBA1	22638	14393	-0.238227043	4.87E-56	2.05E-54
EMC4	2158	1372	-0.238262252	1.12E-06	5.12E-06
BANF1	2674	1700	-0.238312587	5.91E-08	3.17E-07
DNASE1L1	1551	986	-0.238387003	3.63E-05	0.00013205
RAE1	1614	1026	-0.238457716	2.50E-05	9.39E-05

C1orf122	1581	1005	-0.238489735	3.02E-05	0.00011183
SRPRB	4331	2753	-0.238543431	4.98E-12	3.98E-11
MRPL23	2121	1348	-0.238771993	1.32E-06	5.96E-06
AGAP3	2293	1457	-0.239083346	4.76E-07	2.27E-06
NPLOC4	7729	4911	-0.239112812	2.31E-20	3.21E-19
IFNAR1	4558	2896	-0.23918719	1.22E-12	1.02E-11
SLC12A6	2257	1434	-0.239209263	5.77E-07	2.73E-06
RBM15B	3910	2484	-0.239351302	4.61E-11	3.37E-10
CST3	6921	4396	-0.239636987	1.81E-18	2.30E-17
MRPL43	1806	1147	-0.23978037	7.41E-06	3.02E-05
PHLDA1	24098	15304	-0.239852505	2.70E-60	1.22E-58
EIF2B5	2025	1286	-0.239879134	2.06E-06	9.09E-06
SOCS3	2148	1364	-0.239998217	9.99E-07	4.59E-06
HYAL2	2320	1473	-0.240215243	3.61E-07	1.76E-06
ATG2A	1706	1083	-0.240432272	1.26E-05	4.93E-05
ATXN2	4592	2915	-0.240474627	7.70E-13	6.57E-12
PHLDB1	10631	6748	-0.240593299	1.04E-27	2.00E-26
ENO1	70190	44549	-0.240720324	2.86E-173	4.62E-171
CD248	31970	20289	-0.240868853	5.01E-80	3.04E-78
IPO9	5982	3796	-0.240995786	2.48E-16	2.71E-15
CD68	3461	2196	-0.241158755	4.42E-10	2.94E-09
MASP1	1417	899	-0.241294606	6.50E-05	0.000225026
PLEKHA4	1477	937	-0.241396741	4.52E-05	0.000160863
CKS2	1791	1136	-0.241650371	6.91E-06	2.83E-05
GNG11	6015	3815	-0.241729549	1.68E-16	1.86E-15
CCSAP	1066	676	-0.241960155	0.0005144	0.001497203
SYT1	1137	721	-0.242008958	0.0003337	0.001008948
SLC25A11	1946	1234	-0.242017184	2.68E-06	1.16E-05
ARHGAP22	3911	2480	-0.242045284	2.83E-11	2.12E-10
ABHD16A	1279	811	-0.242090313	0.000141	0.00045713
UNC45A	4870	3088	-0.242096888	1.10E-13	1.00E-12
IRF2BPL	3096	1963	-0.242193167	3.13E-09	1.92E-08

EFS	1617	1025	-0.242543637	1.81E-05	6.92E-05
ADRBK1	1983	1257	-0.242547896	2.05E-06	9.07E-06
LPHN2	1581	1002	-0.242802727	2.20E-05	8.33E-05
STX5	2026	1284	-0.24283684	1.55E-06	6.96E-06
TRAM2	17431	11047	-0.242848572	4.02E-45	1.33E-43
RILPL1	972	616	-0.242873831	0.0008732	0.002430855
MT2A	45431	28788	-0.243057366	7.03E-115	6.39E-113
IL11	1321	837	-0.243178807	0.0001023	0.000340905
IDH3G	1946	1233	-0.243186779	2.41E-06	1.05E-05
RPS7	24741	15676	-0.243194204	1.81E-63	8.62E-62
SNHG1	1449	918	-0.243339404	4.67E-05	0.000165948
TXNDC17	3012	1908	-0.243508467	4.25E-09	2.57E-08
TP53	2034	1288	-0.244034954	1.32E-06	5.97E-06
CHORDC1	3282	2078	-0.244227453	7.80E-10	5.06E-09
HDAC6	1220	772	-0.245056263	0.0001692	0.000539277
TFDP1	6933	4386	-0.245421817	2.73E-19	3.61E-18
ZGPAT	1067	675	-0.245448637	0.0004271	0.001262312
UBP1	6235	3944	-0.245577877	1.59E-17	1.90E-16
RAB4A	2052	1298	-0.245588216	1.02E-06	4.68E-06
PGM3	8036	5083	-0.245645202	3.83E-22	5.88E-21
ETV5	4688	2965	-0.245788333	1.42E-13	1.28E-12
DHX34	1072	678	-0.245795596	0.0004066	0.001207135
HDGFRP2	4222	2670	-0.245934705	2.20E-12	1.83E-11
PDP2	1455	920	-0.246161255	3.71E-05	0.000134462
H6PD	5068	3202	-0.247291085	1.05E-14	1.02E-13
PDE7B	1567	990	-0.247352618	1.70E-05	6.55E-05
TMEM167B	4736	2992	-0.247406774	7.51E-14	6.89E-13
PPP2R5B	1488	940	-0.247489733	2.75E-05	0.000102472
TPST1	1659	1048	-0.247523038	9.50E-06	3.81E-05
TIMP1	33763	21327	-0.247609927	6.85E-89	4.82E-87
RPL30	14294	9029	-0.247619445	1.16E-38	3.11E-37
PFKL	6684	4222	-0.247633023	5.97E-19	7.76E-18

CTNNBIP1	1390	878	-0.247639906	5.01E-05	0.000177351
SH3BP2	3113	1966	-0.247890123	1.24E-09	7.93E-09
PLEKHA5	1688	1066	-0.247955334	7.67E-06	3.12E-05
YAP1	21926	13846	-0.248021258	1.51E-58	6.59E-57
EEF2K	3274	2067	-0.248363801	4.35E-10	2.89E-09
MDFIC	6282	3966	-0.248387134	5.33E-18	6.60E-17
NABP2	1394	880	-0.248503001	4.61E-05	0.00016396
SMIM3	2533	1599	-0.248525006	3.95E-08	2.16E-07
CHN1	5112	3227	-0.248542127	6.00E-15	5.97E-14
IFRD2	1467	926	-0.248632641	2.89E-05	0.000107314
ST6GALNAC4	911	575	-0.248736966	0.000978	0.002690085
FUS	10459	6601	-0.248836285	5.35E-29	1.07E-27
CNOT8	2466	1556	-0.249178608	5.53E-08	2.97E-07
TECR	1924	1214	-0.249188246	1.59E-06	7.13E-06
TLE3	905	571	-0.249274915	0.0009923	0.002726968
LRSAM1	970	612	-0.249300963	0.0006514	0.001857034
ATP5G3	6012	3792	-0.249733913	1.86E-17	2.21E-16
CEP57L1	945	596	-0.249849867	0.0007459	0.002106689
NCDN	2288	1443	-0.249863621	1.54E-07	7.86E-07
RAB5C	9125	5751	-0.250859589	7.00E-26	1.27E-24
RHOT2	3148	1984	-0.250871383	6.46E-10	4.22E-09
NUP85	1179	743	-0.250977471	0.0001551	0.000498274
TUBG1	2063	1300	-0.251080066	5.56E-07	2.64E-06
FDXR	1695	1068	-0.251221495	5.63E-06	2.34E-05
DCBLD2	78724	49597	-0.251398537	9.37E-211	2.12E-208
GRINA	7351	4631	-0.251464633	2.97E-21	4.32E-20
KALRN	1062	669	-0.251553518	0.0003206	0.000972602
RIN1	2494	1571	-0.251626153	3.48E-08	1.91E-07
VASP	5196	3273	-0.251635697	1.70E-15	1.75E-14
EPHB2	9046	5696	-0.252178718	6.49E-26	1.18E-24
RAI1	4774	3006	-0.252201425	2.05E-14	1.95E-13
TSC1	4584	2886	-0.252383613	6.42E-14	5.93E-13

SHB	2988	1881	-0.252528168	1.38E-09	8.77E-09
EIF5B	11155	7022	-0.252584481	1.16E-31	2.58E-30
LSM7	1506	948	-0.252610674	1.69E-05	6.52E-05
CLK3	1325	834	-0.252720939	5.42E-05	0.000190282
RPS11	22944	14441	-0.252794149	2.19E-63	1.03E-61
MDC1	2218	1396	-0.252808292	1.74E-07	8.83E-07
MSANTD3	2393	1506	-0.252946495	5.64E-08	3.03E-07
C11orf31	2374	1494	-0.252987655	6.34E-08	3.38E-07
IDH2	2506	1577	-0.253051623	2.72E-08	1.51E-07
PKN1	4638	2918	-0.253370805	3.69E-14	3.45E-13
CLCN6	879	553	-0.253431553	0.0009779	0.002690521
LMF2	5961	3750	-0.253511646	8.82E-18	1.08E-16
HIP1	4944	3110	-0.253612031	5.10E-15	5.11E-14
WASF2	8734	5494	-0.253633489	2.46E-25	4.36E-24
PRIM2	911	573	-0.253763783	0.0007781	0.002187031
CLU	11563	7272	-0.253939515	4.45E-33	1.03E-31
FZR1	2158	1357	-0.254122012	2.23E-07	1.11E-06
PLIN2	4224	2656	-0.254202556	4.22E-13	3.67E-12
PXK	3035	1908	-0.254483213	7.73E-10	5.02E-09
VPS18	2456	1544	-0.254485676	3.15E-08	1.73E-07
NLGN2	6813	4283	-0.254516398	3.07E-20	4.24E-19
MMS22L	1306	821	-0.254548638	5.45E-05	0.00019132
ACSF2	1007	633	-0.254634147	0.0003931	0.001170635
SYVN1	1793	1127	-0.254735841	2.23E-06	9.78E-06
ZC3H3	1023	643	-0.254763371	0.0003509	0.00105599
NRP1	19498	12254	-0.254921259	5.57E-55	2.29E-53
KIAA0895L	1055	663	-0.255010092	0.0002798	0.000857718
CMIP	2301	1446	-0.2550413	8.03E-08	4.23E-07
BBS9	1122	705	-0.255225382	0.0001769	0.000561707
NEURL1B	974	612	-0.255237988	0.0004758	0.001395959
UCKL1	885	556	-0.25544044	0.0008588	0.002393589
C19orf43	4126	2592	-0.255525971	6.05E-13	5.21E-12

GSR	2929	1840	-0.255550295	1.31E-09	8.33E-09
ATN1	9506	5971	-0.255713701	7.77E-28	1.50E-26
ALG1	1159	728	-0.255718079	0.0001344	0.000437322
SSNA1	1086	682	-0.256028327	0.0002153	0.000673182
DCAKD	1223	768	-0.256094056	8.57E-05	0.000289488
LRRC15	3566	2239	-0.256300043	1.92E-11	1.46E-10
METTL13	1518	953	-0.25647154	1.18E-05	4.63E-05
ABCA2	3457	2170	-0.256673429	3.64E-11	2.69E-10
PI4K2A	3966	2489	-0.256966315	1.28E-12	1.07E-11
KHSRP	6948	4360	-0.257117487	5.55E-21	7.93E-20
ADAMTS5	1490	935	-0.257121929	1.35E-05	5.26E-05
RPSA	37060	23253	-0.257293891	1.18E-104	9.77E-103
MRPS12	1181	741	-0.257311385	0.0001056	0.00035116
NOP2	1852	1162	-0.257321898	1.20E-06	5.46E-06
JRK	1546	970	-0.257331535	9.14E-06	3.67E-05
SCAF1	5918	3713	-0.25737225	3.90E-18	4.87E-17
RIN3	1846	1158	-0.257615168	1.22E-06	5.53E-06
FOXM1	1937	1215	-0.257715508	6.59E-07	3.09E-06
USP20	1403	880	-0.257787448	2.30E-05	8.67E-05
FKBP8	8726	5473	-0.257836485	4.54E-26	8.26E-25
LETM1	3848	2413	-0.258128752	2.22E-12	1.84E-11
ZNF410	1952	1224	-0.258197363	5.70E-07	2.69E-06
CAPN1	11599	7273	-0.258225817	3.37E-34	7.95E-33
PHKA1	882	553	-0.258347044	0.0007693	0.002166806
COX8A	4902	3073	-0.258570586	2.08E-15	2.13E-14
PLAU	24419	15306	-0.258754684	2.51E-70	1.34E-68
SSC5D	1575	987	-0.259077707	6.58E-06	2.70E-05
GLE1	1907	1195	-0.259142094	7.03E-07	3.28E-06
PIN1	2681	1680	-0.259157855	4.05E-09	2.46E-08
MAPK9	2989	1873	-0.259159866	5.27E-10	3.47E-09
ARHGEF1	2638	1653	-0.259205708	5.36E-09	3.20E-08
GNG5	4258	2668	-0.259265152	1.21E-13	1.09E-12

TP73-AS1	1253	785	-0.259469724	5.68E-05	0.000198696
QSOX2	1783	1117	-0.259525385	1.56E-06	7.01E-06
UBTD2	2034	1274	-0.25980227	2.81E-07	1.39E-06
RNF25	1041	652	-0.259874067	0.000238	0.000737477
FAM96A	1584	992	-0.260008178	5.75E-06	2.39E-05
YIPF2	1974	1236	-0.260291115	4.01E-07	1.94E-06
GPRC5A	2375	1487	-0.260370734	2.68E-08	1.49E-07
TSSC4	1273	797	-0.260428658	4.66E-05	0.000165532
ISOC2	1492	934	-0.260600949	1.03E-05	4.09E-05
SAMM50	2989	1871	-0.260701207	4.21E-10	2.80E-09
ANAPC11	1695	1061	-0.260708485	2.56E-06	1.11E-05
LRFN4	1195	748	-0.260748311	7.82E-05	0.000266636
SRRT	4600	2879	-0.260913941	8.85E-15	8.64E-14
ZNF496	874	547	-0.260940315	0.0007238	0.002048766
BRAT1	1272	796	-0.261106203	4.49E-05	0.000160087
C9orf16	1117	699	-0.261112693	0.0001313	0.000427704
FRMD8	2143	1341	-0.261170481	1.17E-07	6.07E-07
UFM1	6979	4367	-0.261225669	1.15E-21	1.71E-20
FBXW11	5455	3413	-0.26138665	2.69E-17	3.14E-16
LOC100506548	3454	2161	-0.261416879	1.67E-11	1.28E-10
KANK2	9273	5801	-0.261582404	2.52E-28	4.93E-27
MFN1	3480	2177	-0.261593767	1.36E-11	1.05E-10
SPTLC1	5512	3448	-0.261663982	1.71E-17	2.03E-16
SLC10A3	1647	1030	-0.262044086	3.17E-06	1.37E-05
SETD1A	1324	828	-0.262048318	2.95E-05	0.000109187
NMRAL1	886	554	-0.262268591	0.000626	0.001790408
PRAF2	2914	1822	-0.262325787	5.51E-10	3.62E-09
ATP5D	3050	1907	-0.262352267	2.19E-10	1.51E-09
PTPRS	8725	5455	-0.262423803	6.65E-27	1.24E-25
ARMC6	1320	825	-0.262919773	2.86E-05	0.000106323
NCLN	6775	4234	-0.263047545	2.43E-21	3.56E-20
SRI	3870	2418	-0.26336719	7.15E-13	6.12E-12

TRIP10	2814	1758	-0.263535126	9.17E-10	5.91E-09
CNRIP1	1399	874	-0.263538646	1.58E-05	6.10E-05
CYBASC3	1748	1092	-0.263580197	1.39E-06	6.27E-06
RRAS	3051	1906	-0.263581929	1.81E-10	1.26E-09
EHMT2	2712	1694	-0.263771172	1.78E-09	1.12E-08
MCRS1	2050	1280	-0.264327968	1.59E-07	8.11E-07
GNL2	4402	2748	-0.264625009	1.48E-14	1.42E-13
FAM89B	1655	1033	-0.264838831	2.38E-06	1.04E-05
SART1	3642	2272	-0.265615956	2.24E-12	1.85E-11
TIMM17A	4965	3097	-0.265770203	2.41E-16	2.65E-15
C11orf73	1475	920	-0.265857056	7.81E-06	3.17E-05
SUGP1	1076	671	-0.266141275	0.0001323	0.000430772
RBPJ	17809	11104	-0.266374905	1.26E-54	5.09E-53
GNPTG	1251	780	-0.266383629	3.71E-05	0.000134632
TRIOBP	8502	5301	-0.266385587	5.68E-27	1.07E-25
TCF19	1349	841	-0.266560511	1.82E-05	6.96E-05
INF2	6746	4205	-0.266774386	8.61E-22	1.30E-20
RPS21	8520	5310	-0.266989438	3.90E-27	7.37E-26
CNOT3	1879	1171	-0.267071859	4.03E-07	1.95E-06
ABRACL	1433	893	-0.267154397	9.56E-06	3.83E-05
FBXW5	3556	2215	-0.267796494	2.76E-12	2.27E-11
MPST	1132	705	-0.268026664	7.92E-05	0.000269857
MRFAP1L1	2584	1609	-0.268289612	2.39E-09	1.48E-08
TRMT1	1333	830	-0.268341407	1.81E-05	6.92E-05
MRPL36	1725	1074	-0.268450237	1.06E-06	4.88E-06
RPUSD4	869	541	-0.268575451	0.0005307	0.001540721
TYRO3	2016	1255	-0.268656143	1.30E-07	6.69E-07
PLTP	1841	1146	-0.268730451	4.52E-07	2.16E-06
TRPM4	2331	1451	-0.268749353	1.36E-08	7.76E-08
MRPS34	2585	1609	-0.268847823	2.21E-09	1.37E-08
GMPPB	972	605	-0.26886904	0.0002443	0.000756294
DDB2	1488	926	-0.269138296	5.55E-06	2.31E-05

KIF13B	1848	1150	-0.269178764	4.12E-07	1.99E-06
SLC39A3	1088	677	-0.269298686	0.0001016	0.000338523
NFRKB	1612	1003	-0.269378006	2.22E-06	9.75E-06
TAF10	1678	1044	-0.269468872	1.37E-06	6.18E-06
GNG2	3112	1936	-0.269610976	4.68E-11	3.42E-10
TLE1	1841	1145	-0.269989897	4.01E-07	1.94E-06
S1PR2	1460	908	-0.270052035	6.35E-06	2.61E-05
PIGU	1132	704	-0.270074492	7.02E-05	0.000241493
TGFB1I1	4956	3081	-0.270625373	7.76E-17	8.78E-16
USP24	7564	4702	-0.270722693	7.00E-25	1.22E-23
SLC4A2	6503	4042	-0.27088397	1.22E-21	1.82E-20
IFNGR1	1506	936	-0.270989203	4.22E-06	1.79E-05
CCDC130	811	504	-0.271126049	0.0007316	0.002069156
ATXN10	8816	5478	-0.271322826	7.65E-29	1.53E-27
FUT8	2630	1634	-0.271502684	1.13E-09	7.22E-09
TCIRG1	3224	2003	-0.271537191	1.54E-11	1.18E-10
PHF5A	961	597	-0.271653368	0.0002302	0.000715304
DDX56	2323	1443	-0.271765723	1.01E-08	5.84E-08
PRMT1	5831	3622	-0.271804645	1.10E-19	1.48E-18
TBX2	10186	6327	-0.27184199	3.52E-33	8.19E-32
PSIP1	4500	2795	-0.271924587	1.48E-15	1.54E-14
ZNF146	7102	4411	-0.271967468	1.19E-23	1.96E-22
LYAR	826	513	-0.272030824	0.0006277	0.001794662
CLIP4	4163	2585	-0.272307146	1.54E-14	1.48E-13
MT1L	1356	842	-0.272312908	1.16E-05	4.56E-05
HRAS	1894	1176	-0.272396139	2.17E-07	1.08E-06
TRIM28	12512	7768	-0.27254518	1.48E-40	4.30E-39
GTSE1	1263	784	-0.272776948	2.24E-05	8.48E-05
LMNA	45214	28064	-0.272896771	3.68E-142	4.45E-140
ARID5B	22965	14252	-0.273120263	3.07E-73	1.71E-71
SYNJ2	5721	3550	-0.273296188	1.58E-19	2.10E-18
SLCO3A1	967	600	-0.273401257	0.0002012	0.00063191

TBL3	1760	1092	-0.273450441	5.27E-07	2.51E-06
NELFE	2026	1257	-0.273497393	7.34E-08	3.88E-07
TMEM9B	3251	2017	-0.27352034	9.15E-12	7.15E-11
MAT2A	9326	5785	-0.273789339	6.48E-31	1.40E-29
APRT	3001	1861	-0.274213132	5.11E-11	3.73E-10
FAM114A1	16110	9988	-0.274536636	2.06E-52	7.98E-51
CSNK1E	4823	2990	-0.274633194	7.55E-17	8.54E-16
KIAA2013	3310	2052	-0.274648354	4.92E-12	3.94E-11
CLPB	1055	654	-0.274728326	9.58E-05	0.000320345
FAM3A	1318	817	-0.274790255	1.29E-05	5.06E-05
RPS9	13578	8415	-0.275083682	1.29E-44	4.25E-43
CLIP2	6982	4327	-0.275121104	9.24E-24	1.53E-22
LPPR2	4271	2646	-0.275608705	3.46E-15	3.50E-14
IMPDH2	8304	5144	-0.275763669	4.55E-28	8.91E-27
SSH3	1193	739	-0.275795642	3.13E-05	0.000115565
C17orf76-AS1	12804	7931	-0.275867762	2.17E-42	6.63E-41
EEF1D	11268	6979	-0.2759871	1.53E-37	3.99E-36
S1PR3	2895	1793	-0.276035728	8.51E-11	6.07E-10
EVA1B	2795	1731	-0.276090427	1.78E-10	1.24E-09
GCC1	1668	1033	-0.276126903	8.27E-07	3.84E-06
WDR6	5826	3608	-0.276154231	3.18E-20	4.38E-19
MEX3D	1447	896	-0.276342153	4.35E-06	1.84E-05
VARS	7035	4356	-0.276394338	3.98E-24	6.69E-23
FGF7	6059	3751	-0.276652311	4.90E-21	7.03E-20
HPS1	1916	1186	-0.276841419	1.19E-07	6.14E-07
FHOD1	2811	1740	-0.276844016	1.41E-10	9.93E-10
NUP37	1068	661	-0.277037338	7.61E-05	0.000260276
PALM	1640	1015	-0.277063956	9.44E-07	4.35E-06
NLK	1789	1107	-0.277346034	2.96E-07	1.46E-06
CRAT	3153	1951	-0.27735926	1.01E-11	7.85E-11
C12orf57	1728	1069	-0.277689233	4.57E-07	2.18E-06
TAF6	2501	1547	-0.277879729	1.26E-09	8.06E-09

GEMIN4	1072	663	-0.278071999	6.96E-05	0.000239484
POMT1	1732	1071	-0.278328318	4.18E-07	2.01E-06
KIAA1217	1957	1210	-0.278484577	7.36E-08	3.89E-07
CCNB1	4251	2628	-0.278684852	2.06E-15	2.11E-14
PROSER1	1833	1133	-0.278906793	1.83E-07	9.21E-07
ZWINT	1846	1141	-0.27895163	1.64E-07	8.36E-07
CKAP2L	1330	822	-0.279063815	8.76E-06	3.53E-05
PLEKHG2	4411	2726	-0.279168067	5.53E-16	5.95E-15
ZBTB17	1068	660	-0.279221586	6.72E-05	0.000231946
PITPNM1	1717	1061	-0.279313251	4.29E-07	2.06E-06
MFSD5	1447	894	-0.279566054	3.40E-06	1.46E-05
NELFB	2788	1722	-0.279993287	1.07E-10	7.54E-10
DKC1	2835	1751	-0.28001752	7.42E-11	5.32E-10
FZD7	9284	5734	-0.280052486	4.60E-32	1.04E-30
NUP62	2575	1590	-0.280393535	5.16E-10	3.41E-09
LPCAT1	4691	2896	-0.280681767	4.64E-17	5.31E-16
AKT1S1	4313	2662	-0.281029013	7.65E-16	8.15E-15
ATP5I	1841	1136	-0.28137466	1.35E-07	6.94E-07
ARL16	1248	770	-0.281535452	1.41E-05	5.48E-05
BZW1	26866	16566	-0.282404113	7.52E-91	5.45E-89
MFAP1	2577	1589	-0.282421281	3.87E-10	2.58E-09
TGFB1	8338	5141	-0.282500228	2.01E-29	4.10E-28
SNHG5	7390	4556	-0.282654486	2.73E-26	4.99E-25
HMBS	1264	779	-0.283149098	1.11E-05	4.40E-05
KCNMA1	10248	6315	-0.28333561	5.72E-36	1.41E-34
RPS15	12593	7760	-0.283341324	8.31E-44	2.64E-42
B3GNT9	930	573	-0.283543445	0.0001605	0.00051474
SNED1	732	451	-0.283564083	0.0008119	0.002274491
TNFRSF21	2792	1720	-0.283738245	5.99E-11	4.33E-10
FAH	1921	1183	-0.284255314	5.41E-08	2.90E-07
PRKCDBP	5643	3475	-0.28429724	1.15E-20	1.63E-19
SEPT9	34789	21418	-0.28465531	7.75E-119	7.31E-117

STOML2	3717	2288	-0.284899504	3.46E-14	3.25E-13
MRTO4	2171	1336	-0.285287586	6.62E-09	3.91E-08
ZDHHC12	832	512	-0.285287586	0.0003297	0.000998008
CCDC86	1539	947	-0.285404769	1.03E-06	4.73E-06
POLD1	811	499	-0.285509967	0.0003885	0.001159574
NSDHL	1068	657	-0.28579424	4.60E-05	0.000163556
MCM4	3913	2407	-0.285886837	6.05E-15	6.02E-14
GSTK1	2315	1424	-0.285910915	1.95E-09	1.22E-08
PLEKHO1	2107	1296	-0.285972464	1.02E-08	5.89E-08
HELZ2	1465	901	-0.286149522	1.77E-06	7.86E-06
CDC27	10312	6342	-0.286162255	7.70E-37	1.95E-35
INTS5	1005	618	-0.286364626	7.48E-05	0.00025587
EPHA4	3033	1865	-0.286417736	5.91E-12	4.71E-11
MBD3	3506	2155	-0.286985995	1.24E-13	1.12E-12
CNTROB	1505	925	-0.287086084	1.18E-06	5.39E-06
CYB5A	986	606	-0.287117721	8.40E-05	0.000283942
SAP130	2119	1302	-0.287502007	7.80E-09	4.57E-08
PLEC	50374	30951	-0.28754114	1.70E-174	2.79E-172
ERH	2941	1807	-0.287558146	1.03E-11	8.01E-11
DHX37	1382	849	-0.287769025	3.07E-06	1.32E-05
MIEN1	1216	747	-0.287810949	1.20E-05	4.72E-05
RASSF2	972	597	-0.288073251	8.96E-05	0.000301816
SLC7A14	933	573	-0.28818981	0.0001234	0.000404563
ECH1	1218	748	-0.288251826	1.15E-05	4.53E-05
ANGEL2	1814	1114	-0.288273091	8.57E-08	4.50E-07
MAP2K2	6779	4163	-0.288296781	4.06E-25	7.14E-24
POLR2F	1293	794	-0.288359231	6.12E-06	2.53E-05
INCENP	1497	919	-0.288785323	1.10E-06	5.03E-06
RPL8	34031	20891	-0.288815861	1.74E-119	1.67E-117
ARHGDIA	22625	13887	-0.289030692	3.52E-80	2.17E-78
CNKSR3	1292	793	-0.289061167	5.87E-06	2.43E-05
AIP	1178	723	-0.289119855	1.51E-05	5.85E-05

PLXNA3	7082	4346	-0.289316537	2.48E-26	4.55E-25
PRC1	3207	1968	-0.289342001	9.00E-13	7.60E-12
GCLC	2932	1799	-0.289537785	8.13E-12	6.39E-11
SOX6	740	454	-0.289680841	0.0005905	0.001702508
CUEDC1	2292	1406	-0.289858318	1.44E-09	9.14E-09
ADAM15	3231	1982	-0.289871656	6.76E-13	5.79E-12
SETD1B	2240	1374	-0.289964596	2.18E-09	1.36E-08
TRAPPC5	1303	799	-0.290417544	4.87E-06	2.04E-05
FAM214B	3366	2064	-0.290440074	2.04E-13	1.82E-12
VAMP5	1047	642	-0.290464108	4.18E-05	0.00015
C14orf159	977	599	-0.290650427	7.48E-05	0.000255866
MAPK8IP3	3902	2392	-0.290844257	2.37E-15	2.42E-14
UBQLN4	2584	1584	-0.290881603	1.15E-10	8.11E-10
THOP1	2328	1427	-0.290953592	9.37E-10	6.04E-09
SLC25A43	1188	728	-0.291372349	1.20E-05	4.72E-05
HIBADH	2226	1364	-0.291457817	2.05E-09	1.28E-08
RPS19BP1	1804	1105	-0.292000837	6.46E-08	3.44E-07
CAD	4441	2720	-0.292125788	2.16E-17	2.55E-16
TRAPPC9	1099	673	-0.292360845	2.40E-05	9.01E-05
ANKRD26	1942	1189	-0.292642354	1.91E-08	1.07E-07
ORC6	678	415	-0.293021805	0.0008847	0.002459453
MCU	2312	1415	-0.293187213	8.09E-10	5.25E-09
AKR1B1	4229	2588	-0.293326811	9.39E-17	1.05E-15
E4F1	938	574	-0.293385054	9.03E-05	0.000303796
PYGB	15903	9731	-0.293486839	1.59E-58	6.92E-57
IFT80	2334	1428	-0.29365645	6.34E-10	4.15E-09
EPB41L5	4777	2922	-0.293996567	8.47E-19	1.10E-17
PKD1P1	5825	3561	-0.294823482	1.09E-22	1.73E-21
MXRA5	777	475	-0.294834953	0.0003433	0.001036279
CENPB	4186	2559	-0.294840032	9.54E-17	1.07E-15
IKBKG	1914	1170	-0.294930168	1.90E-08	1.07E-07
DPP7	2112	1291	-0.294968702	3.52E-09	2.15E-08

COL18A1	17076	10436	-0.295249087	2.11E-63	9.98E-62
EHD4	2155	1317	-0.295280392	2.36E-09	1.46E-08
PCBP4	4526	2766	-0.295283296	4.99E-18	6.19E-17
MAD2L1BP	838	512	-0.295654302	0.0001931	0.000607699
SMARCD3	1622	991	-0.295664725	2.14E-07	1.07E-06
CD9	5835	3565	-0.295678448	7.70E-23	1.23E-21
LSS	3477	2124	-0.295907169	2.96E-14	2.79E-13
WDR81	1508	921	-0.296211235	5.45E-07	2.59E-06
NBPF3	1644	1004	-0.296298898	1.67E-07	8.50E-07
BCAS3	863	527	-0.296405466	0.0001493	0.000481112
MKKS	1839	1123	-0.29641142	3.10E-08	1.70E-07
COL27A1	1497	914	-0.296656019	5.77E-07	2.72E-06
SMPD4	2986	1823	-0.296747472	1.64E-12	1.37E-11
DUSP10	2616	1597	-0.296846096	3.79E-11	2.80E-10
WDR1	35705	21787	-0.297506431	2.09E-132	2.28E-130
MAD1L1	1441	879	-0.297983133	8.43E-07	3.91E-06
NLN	4261	2599	-0.298083286	2.41E-17	2.83E-16
ZNF335	1248	761	-0.298497444	4.41E-06	1.87E-05
UCK2	1681	1025	-0.298543683	9.88E-08	5.14E-07
POLR3F	943	575	-0.298543683	6.57E-05	0.000227412
FAM189B	2488	1517	-0.298613268	8.90E-11	6.33E-10
SLC25A24	5506	3357	-0.298679941	4.97E-22	7.60E-21
CHMP7	3059	1865	-0.298732345	6.34E-13	5.44E-12
RPL38	10413	6347	-0.299086892	2.76E-40	7.91E-39
EHD2	26490	16144	-0.299297632	7.82E-100	6.05E-98
C1orf86	1147	699	-0.299348899	1.02E-05	4.06E-05
GLI3	1618	986	-0.299399924	1.59E-07	8.07E-07
FABP5	1029	627	-0.299553502	2.88E-05	0.000107102
ACOX3	1226	747	-0.299626699	4.96E-06	2.08E-05
CAPN10	668	407	-0.299667176	0.0007484	0.002113221
SLC25A1	4256	2593	-0.299723813	1.72E-17	2.05E-16
CXorf38	1369	834	-0.299851026	1.37E-06	6.20E-06

KDM2B	1827	1113	-0.29987091	2.42E-08	1.35E-07
OTUD6B	1246	759	-0.299980146	4.05E-06	1.72E-05
PPARG	2172	1323	-0.30005891	1.15E-09	7.38E-09
ZNF512	2256	1374	-0.300232932	5.42E-10	3.56E-09
AURKA	3061	1864	-0.300449053	4.67E-13	4.05E-12
RAB11FIP3	2641	1608	-0.300664763	1.76E-11	1.35E-10
PSMB7	1973	1201	-0.301002673	5.95E-09	3.53E-08
KCTD17	1091	664	-0.301243823	1.49E-05	5.79E-05
TACC3	2440	1485	-0.301266085	9.44E-11	6.70E-10
SHANK2	1308	796	-0.301370073	2.11E-06	9.29E-06
C19orf48	889	541	-0.301402693	9.21E-05	0.000309286
C17orf89	1397	850	-0.301645143	9.31E-07	4.30E-06
IRF3	1519	924	-0.302004981	3.03E-07	1.49E-06
SAT2	1710	1040	-0.302260665	5.39E-08	2.90E-07
IQGAP3	2130	1295	-0.302749201	1.21E-09	7.74E-09
SNX8	1816	1104	-0.302871899	1.97E-08	1.11E-07
CLTB	3989	2425	-0.302890244	8.63E-17	9.72E-16
POLR2E	5251	3192	-0.302979412	1.28E-21	1.90E-20
USP11	3600	2188	-0.303232037	2.48E-15	2.53E-14
ADRM1	3400	2066	-0.30354236	1.37E-14	1.32E-13
VSIG10	856	520	-0.303947042	0.0001098	0.000363403
TDP1	1159	704	-0.304081101	6.71E-06	2.75E-05
LMNB2	8118	4931	-0.304091955	9.60E-33	2.21E-31
RPL18	18299	11112	-0.304494174	8.76E-72	4.76E-70
PFKP	10973	6663	-0.30456208	8.84E-44	2.80E-42
SERTAD1	952	578	-0.304739949	4.33E-05	0.000155009
HAUS1	850	516	-0.304939644	0.0001105	0.000365492
HDHD1	1023	621	-0.30498884	2.21E-05	8.39E-05
FIBCD1	1084	658	-0.305053136	1.26E-05	4.92E-05
ELOF1	1562	948	-0.305283357	1.55E-07	7.88E-07
CSRNP2	3088	1874	-0.305399668	1.59E-13	1.42E-12
ZNF3	2480	1505	-0.305424502	3.74E-11	2.76E-10

RPL18A	21063	12782	-0.30544521	7.99E-83	5.11E-81
CUTA	6252	3792	-0.306206682	6.48E-26	1.18E-24
CKB	3009	1825	-0.306235511	2.80E-13	2.47E-12
MARVELD1	6744	4090	-0.306351561	7.55E-28	1.46E-26
MIB2	1748	1060	-0.306488788	2.53E-08	1.41E-07
NUDT22	889	539	-0.306746014	7.01E-05	0.000241048
CUL9	1343	814	-0.307206473	9.88E-07	4.55E-06
C1orf21	1579	957	-0.30726821	1.11E-07	5.76E-07
ELMOD3	670	406	-0.307529236	0.00054	0.001566185
SLC9A3R2	3723	2256	-0.307546417	3.43E-16	3.74E-15
METRNL	1015	615	-0.30766928	2.04E-05	7.76E-05
NFATC4	2135	1293	-0.308361663	5.92E-10	3.88E-09
LINC00641	1767	1070	-0.308539112	1.73E-08	9.81E-08
KIF20A	2923	1770	-0.308548337	4.17E-13	3.63E-12
PSMB10	758	459	-0.308551563	0.0002226	0.000693037
GPR125	3303	2000	-0.308624838	1.27E-14	1.23E-13
USP46	4134	2503	-0.308727966	6.31E-18	7.75E-17
CEP290	1665	1008	-0.308874407	4.32E-08	2.35E-07
BAD	846	512	-0.309361721	9.23E-05	0.000309727
TUBB4B	15303	9261	-0.309422486	3.90E-62	1.80E-60
AKAP8	1702	1030	-0.309434568	2.90E-08	1.60E-07
OSBPL6	737	446	-0.309468777	0.0002616	0.000805613
ASB1	4429	2680	-0.309595865	3.46E-19	4.54E-18
JUND	1294	783	-0.309601273	1.30E-06	5.90E-06
TSNAX	2438	1475	-0.30983104	2.99E-11	2.22E-10
CPOX	1848	1118	-0.309892437	7.11E-09	4.19E-08
EPHX1	1248	755	-0.309917253	1.96E-06	8.70E-06
SLC25A42	620	375	-0.310225488	0.0007905	0.002217925
PDCD5	2424	1466	-0.310352464	3.17E-11	2.35E-10
AGAP1	2052	1241	-0.310375484	1.01E-09	6.46E-09
GGA2	1791	1083	-0.310579963	1.13E-08	6.49E-08
CYFIP2	2011	1216	-0.310617721	1.43E-09	9.08E-09

CORO2B	1644	994	-0.31074041	4.41E-08	2.39E-07
RALGDS	1388	839	-0.31111272	4.78E-07	2.28E-06
NPRL3	1754	1060	-0.311432351	1.46E-08	8.33E-08
TMEM35	882	533	-0.311490991	5.86E-05	0.000204694
ECHDC1	4162	2515	-0.311566433	2.52E-18	3.18E-17
PNPLA2	2911	1759	-0.311607225	2.82E-13	2.49E-12
SMYD4	639	386	-0.312062952	0.000612	0.001754742
MEA1	1409	851	-0.312288443	3.56E-07	1.73E-06
TRIP11	4865	2938	-0.312453277	2.93E-21	4.27E-20
ACAA2	2277	1375	-0.312550541	9.34E-11	6.64E-10
PLD2	1368	826	-0.312702412	5.09E-07	2.42E-06
RNASEH2A	795	480	-0.312768323	0.0001282	0.000418772
FOXF1	9617	5806	-0.312890215	1.63E-40	4.70E-39
SLC25A29	1302	786	-0.312976099	9.38E-07	4.33E-06
BRCA2	767	463	-0.313062252	0.0001665	0.000531647
NOL12	661	399	-0.313109394	0.0004727	0.001387572
MRPL53	999	603	-0.313174544	1.72E-05	6.61E-05
TULP3	2305	1391	-0.313492199	6.34E-11	4.56E-10
IFI27L2	1576	951	-0.313598157	6.45E-08	3.44E-07
JAK2	1987	1199	-0.313608082	1.28E-09	8.14E-09
BAG1	943	569	-0.313676987	2.88E-05	0.000107069
CENPO	948	572	-0.31371978	2.74E-05	0.000102093
GAMT	1530	923	-0.313976968	9.69E-08	5.05E-07
LOC642852	4171	2516	-0.314109258	1.27E-18	1.63E-17
ORC2	1779	1073	-0.314264303	8.66E-09	5.05E-08
FAM20C	5762	3475	-0.314404551	3.69E-25	6.50E-24
NDUFA13	4414	2662	-0.314423927	1.19E-19	1.60E-18
NINL	748	451	-0.314758705	0.0001861	0.00058785
PHKG2	957	577	-0.314795474	2.36E-05	8.89E-05
ELP5	1221	736	-0.315133397	1.75E-06	7.79E-06
FOXRED2	984	593	-0.315474079	1.74E-05	6.68E-05
CAV1	64696	38985	-0.31560525	2.55E-266	8.58E-264

EXOSC1	951	573	-0.31575807	2.37E-05	8.92E-05
DCTPP1	810	488	-0.315888628	9.53E-05	0.000318758
RPS29	5327	3209	-0.31604736	1.35E-23	2.21E-22
CSTF2	865	521	-0.316264628	5.41E-05	0.000190144
TWF2	3201	1928	-0.316265494	8.08E-15	7.93E-14
SDC1	4749	2860	-0.316456477	2.93E-21	4.27E-20
ADPRHL2	900	542	-0.316480018	3.78E-05	0.000136938
SEC24D	15896	9572	-0.316619344	2.95E-67	1.50E-65
ALKBH7	608	366	-0.317075543	0.0006918	0.001965732
ZNHIT6	3052	1837	-0.317251204	2.83E-14	2.68E-13
DNM1	1703	1025	-0.317302394	1.33E-08	7.59E-08
HSF1	2454	1477	-0.317313291	9.03E-12	7.07E-11
ARL14EP	1178	709	-0.317329875	2.29E-06	1.00E-05
TIMM13	2092	1259	-0.317452437	2.96E-10	2.01E-09
CAAP1	1047	630	-0.317685577	8.18E-06	3.32E-05
MYO9B	12901	7760	-0.318202209	1.98E-55	8.26E-54
UBE2N	5204	3130	-0.318306173	2.22E-23	3.62E-22
PPRC1	3307	1989	-0.318327638	1.98E-15	2.03E-14
NCAPH2	1297	780	-0.318480319	6.48E-07	3.04E-06
RNF24	2468	1484	-0.318699171	6.47E-12	5.15E-11
EEA1	8926	5367	-0.318745787	5.18E-39	1.42E-37
SIPA1L2	632	380	-0.318773008	0.0005077	0.001479973
TRAPPC12	2094	1259	-0.318831027	2.46E-10	1.68E-09
FBXO9	3058	1838	-0.319299508	1.87E-14	1.78E-13
HMGA1	75921	45614	-0.319870174	0	0
CDCA8	1400	841	-0.32009699	2.05E-07	1.03E-06
TAF1C	1162	698	-0.320158995	2.21E-06	9.70E-06
GPR153	746	448	-0.320524766	0.0001466	0.000473054
EYA4	1544	927	-0.320879377	4.55E-08	2.47E-07
C12orf43	603	362	-0.321016173	0.0006297	0.001799556
PATZ1	798	479	-0.321210959	8.32E-05	0.000281907
CROT	2916	1750	-0.321483666	5.15E-14	4.78E-13

VDAC3	4296	2578	-0.321589598	6.23E-20	8.49E-19
SLC22A17	1780	1068	-0.321813462	3.93E-09	2.39E-08
SPAST	1495	897	-0.321813462	6.84E-08	3.64E-07
CCDC115	1000	600	-0.321813462	1.02E-05	4.06E-05
TBCE	1020	612	-0.321813462	8.33E-06	3.38E-05
SERP1	10189	6113	-0.321907861	4.36E-45	1.44E-43
HDAC10	1117	670	-0.322244053	3.02E-06	1.30E-05
SLC39A1	4278	2566	-0.32226318	6.30E-20	8.57E-19
DCXR	917	550	-0.322337983	2.32E-05	8.73E-05
CYC1	2994	1795	-0.322938246	1.85E-14	1.77E-13
RAB34	4714	2826	-0.323038161	6.89E-22	1.05E-20
SIRT7	654	392	-0.32328485	0.0003385	0.001022372
TOP3B	861	516	-0.32349004	3.88E-05	0.000140304
ZNF579	1083	649	-0.323590728	3.91E-06	1.66E-05
RBX1	2278	1365	-0.323714664	2.12E-11	1.61E-10
MAPK12	1654	991	-0.32385014	1.14E-08	6.53E-08
MAPK10	656	393	-0.324014371	0.0003219	0.000976202
TBX5	2189	1311	-0.324452137	4.73E-11	3.45E-10
FAM64A	830	497	-0.324713353	5.03E-05	0.000177798
SLC25A20	887	531	-0.325070112	2.72E-05	0.000101584
TUBA1B	56745	33970	-0.325079927	3.92E-247	1.11E-244
LSMD1	1460	874	-0.325111053	7.31E-08	3.87E-07
LRRC14	680	407	-0.32535382	0.0002365	0.00073351
UQCR11	3661	2191	-0.325496158	1.41E-17	1.69E-16
MED16	2261	1353	-0.325647021	1.95E-11	1.48E-10
PYGL	3580	2141	-0.32652266	2.57E-17	3.01E-16
PFN1	33691	20148	-0.326574483	8.89E-149	1.14E-146
UBTF	4321	2584	-0.326607029	1.38E-20	1.94E-19
DTD2	995	595	-0.326654725	8.04E-06	3.26E-05
U2AF2	8041	4808	-0.326785902	6.23E-37	1.59E-35
LDB1	4494	2687	-0.326851671	2.25E-21	3.30E-20
SELENBP1	1032	617	-0.326948445	5.36E-06	2.24E-05

CARM1	4341	2595	-0.327140752	9.86E-21	1.40E-19
UBL4A	1382	826	-0.327391797	1.35E-07	6.92E-07
C1orf35	594	355	-0.327491775	0.0005453	0.001579467
ARL5B	1977	1181	-0.328151775	2.62E-10	1.79E-09
FIBP	2694	1609	-0.328433393	1.54E-13	1.39E-12
SIRPA	4057	2423	-0.328466766	1.29E-19	1.73E-18
ARF1	21783	13009	-0.32854046	5.96E-98	4.50E-96
IQSEC2	1745	1042	-0.328719627	2.73E-09	1.68E-08
CTSK	1335	797	-0.329035981	1.93E-07	9.69E-07
KLHL22	995	594	-0.329081463	6.95E-06	2.84E-05
GFER	980	585	-0.329192993	8.09E-06	3.28E-05
POP7	774	462	-0.329288583	7.27E-05	0.000249667
ID2	3960	2363	-0.329728669	2.59E-19	3.44E-18
CIZ1	4951	2954	-0.329897991	9.06E-24	1.50E-22
FLRT3	828	494	-0.329967594	3.94E-05	0.000142289
SSBP3	2107	1257	-0.330053533	5.39E-11	3.91E-10
PMVK	917	547	-0.330228769	1.49E-05	5.79E-05
RPL26L1	1365	814	-0.33064812	1.23E-07	6.37E-07
PARP10	535	319	-0.330830336	0.0009237	0.002556038
SNAPC4	1191	710	-0.331130352	7.53E-07	3.51E-06
NPM3	718	428	-0.331220916	0.000122	0.000400329
PDLIM4	2365	1409	-0.33201644	2.77E-12	2.28E-11
COMMD4	984	586	-0.332605519	6.32E-06	2.60E-05
COA6	729	434	-0.33307164	9.95E-05	0.000332143
ANKS3	593	353	-0.33321179	0.0004458	0.001314046
MPP1	1813	1079	-0.333531929	7.98E-10	5.18E-09
CRTC1	815	485	-0.33366318	3.76E-05	0.000136268
TONSL	763	454	-0.333838628	6.60E-05	0.000228298
NINJ1	1785	1062	-0.333988176	1.02E-09	6.57E-09
FUBP3	6070	3611	-0.334145964	1.96E-29	4.00E-28
HPS6	1256	747	-0.334504184	2.91E-07	1.44E-06
C19orf25	929	552	-0.335858198	9.52E-06	3.81E-05

GRK5	2390	1420	-0.335967557	1.21E-12	1.01E-11
LARP6	3814	2266	-0.336004851	2.81E-19	3.72E-18
RNF166	606	360	-0.336168755	0.0003445	0.001039285
BCKDHA	655	389	-0.33657262	0.000195	0.000613042
ERBB2	4329	2570	-0.337113309	8.61E-22	1.30E-20
DGCR14	881	523	-0.337178941	1.50E-05	5.83E-05
GUK1	6150	3647	-0.338724079	1.57E-30	3.34E-29
AHRR	4265	2529	-0.338826573	1.10E-21	1.65E-20
RSL24D1	3865	2290	-0.339968684	6.49E-20	8.81E-19
UBE2E2	849	503	-0.340054022	1.85E-05	7.06E-05
NME1	866	513	-0.340256067	1.51E-05	5.83E-05
KSR1	677	401	-0.340401465	0.0001292	0.000421571
SLC52A2	2587	1532	-0.340711625	6.91E-14	6.35E-13
HOXB-AS3	689	408	-0.340782699	0.0001107	0.000365853
RCN3	4541	2689	-0.34078815	3.24E-23	5.24E-22
HAUS6	2778	1645	-0.340806884	8.28E-15	8.11E-14
RABEP2	1152	682	-0.341144941	5.62E-07	2.66E-06
CIT	2049	1213	-0.341188302	2.48E-11	1.87E-10
ZNF558	745	441	-0.341309638	5.68E-05	0.000198726
METTL21B	620	367	-0.341336021	0.0002399	0.000743245
SLC4A8	1261	746	-0.342168608	1.52E-07	7.76E-07
LSM2	756	447	-0.342959271	4.62E-05	0.000164381
STK11	1839	1087	-0.343417408	1.99E-10	1.38E-09
TMSB10	65549	38744	-0.343448731	0	0
GPS1	4538	2682	-0.34359524	1.54E-23	2.53E-22
SLC25A15	731	432	-0.343687962	5.97E-05	0.00020812
HCFC1R1	562	332	-0.344234757	0.0004239	0.001254719
TCF3	3821	2257	-0.344391707	3.76E-20	5.16E-19
PRMT7	1021	603	-0.344600827	1.98E-06	8.75E-06
MZT2A	569	336	-0.344815288	0.000382	0.001141147
FANCA	564	333	-0.345020854	0.0004023	0.00119554
ARHGEF12	24491	14460	-0.345031998	2.55E-120	2.48E-118

USF2	2168	1280	-0.345068815	3.96E-12	3.20E-11
NSMF	2084	1230	-0.34554483	9.64E-12	7.52E-11
APBB1	2323	1371	-0.345608451	6.33E-13	5.44E-12
DOK4	1629	961	-0.346226136	1.60E-09	1.01E-08
ATP5G1	1526	900	-0.346605924	5.04E-09	3.03E-08
MFHAS1	994	586	-0.347193055	2.30E-06	1.01E-05
DPYSL3	7355	4336	-0.347208453	8.02E-38	2.12E-36
TRNAU1AP	704	415	-0.347311961	6.95E-05	0.00023919
ADCY3	1291	761	-0.34736851	7.13E-08	3.78E-07
NDUFB10	3264	1924	-0.347380126	1.06E-17	1.28E-16
TESK1	967	570	-0.347401839	3.11E-06	1.34E-05
RRS1	3921	2311	-0.34755225	5.74E-21	8.18E-20
SOX12	2330	1373	-0.347846198	4.24E-13	3.69E-12
DBNDD1	1300	766	-0.347943194	6.13E-08	3.27E-07
IFFO1	1823	1074	-0.348168436	1.39E-10	9.80E-10
SHARPIN	1401	825	-0.3488388	1.75E-08	9.89E-08
ZNF580	1126	663	-0.34897392	4.33E-07	2.08E-06
SACS	22457	13221	-0.349181779	5.94E-113	5.25E-111
FAM168A	1257	740	-0.349235342	9.12E-08	4.77E-07
PLCB3	3177	1870	-0.349474688	1.89E-17	2.24E-16
NLE1	576	339	-0.349631407	0.0002936	0.000896811
PTP4A2	14877	8755	-0.349752416	1.09E-75	6.15E-74
RAB1B	10366	6098	-0.350299112	1.92E-53	7.64E-52
CLMP	7905	4650	-0.350382615	3.43E-41	1.01E-39
PYGO2	1109	652	-0.351163364	4.54E-07	2.17E-06
PAFAH2	1201	706	-0.351343931	1.50E-07	7.66E-07
PHLDB3	667	392	-0.351680975	8.93E-05	0.000300992
S100A6	54861	32241	-0.351732296	7.21E-277	2.50E-274
CDR2L	2549	1498	-0.351741618	1.85E-14	1.77E-13
DVL1	3303	1941	-0.35182472	2.73E-18	3.44E-17
AKAP9	4495	2641	-0.352084682	2.38E-24	4.04E-23
MCM6	2114	1242	-0.352158071	2.87E-12	2.36E-11

TTL	5091	2991	-0.352169023	2.26E-27	4.29E-26
C20orf27	1835	1078	-0.352270753	7.58E-11	5.43E-10
PAGR1	1527	897	-0.35236804	2.87E-09	1.77E-08
PDCL3	952	559	-0.352961159	2.64E-06	1.15E-05
GPX1	9405	5522	-0.353084924	2.35E-49	8.57E-48
TBC1D8	1189	698	-0.353297642	1.49E-07	7.63E-07
SOX11	2132	1251	-0.353973517	1.82E-12	1.51E-11
RPS5	15900	9329	-0.354080286	1.31E-82	8.31E-81
LRR1	692	406	-0.354140179	5.90E-05	0.000205653
ISCA2	716	420	-0.354418128	4.33E-05	0.000155059
LGALS1	77898	45693	-0.35446099	0	0
DOCK6	1485	871	-0.354566175	3.84E-09	2.34E-08
H2AFX	3209	1882	-0.35470503	4.59E-18	5.71E-17
HCFC1	4853	2846	-0.354789066	1.63E-26	3.02E-25
CDH4	1366	801	-0.354931204	1.55E-08	8.83E-08
POLR2L	8882	5207	-0.355280033	3.10E-47	1.08E-45
CDK5	812	476	-0.355366022	1.27E-05	4.97E-05
STC1	6852	4013	-0.356691864	4.24E-37	1.09E-35
SDCCAG3	1658	971	-0.356748674	3.83E-10	2.56E-09
DDX39A	1638	959	-0.357180505	4.67E-10	3.09E-09
NCOR2	12621	7389	-0.357223059	5.00E-67	2.50E-65
NDOR1	1162	680	-0.357851286	1.47E-07	7.52E-07
WDR46	2683	1570	-0.357930364	1.36E-15	1.42E-14
TMEM80	670	392	-0.358155309	6.48E-05	0.000224466
TEF	1089	637	-0.358486545	3.45E-07	1.68E-06
ANPEP	6845	4003	-0.358816794	1.85E-37	4.80E-36
CTBP1	4727	2763	-0.359537171	1.76E-26	3.25E-25
NPC1	2115	1236	-0.359826788	1.02E-12	8.55E-12
NYNRIN	2521	1473	-0.360086556	6.82E-15	6.74E-14
HDGFRP3	3838	2242	-0.360416302	6.76E-22	1.03E-20
RPL10	38885	22691	-0.361941417	1.52E-207	3.27E-205
BTBD6	1796	1048	-0.361996501	3.95E-11	2.90E-10

NT5DC2	6624	3865	-0.362082127	6.75E-37	1.72E-35
DGCR6L	1150	671	-0.362097058	1.24E-07	6.41E-07
CDT1	641	374	-0.362133955	7.89E-05	0.000268925
SWI5	1049	612	-0.362258988	4.37E-07	2.10E-06
TRO	2009	1172	-0.362352863	2.69E-12	2.22E-11
ATP1A1	11012	6424	-0.362380585	2.90E-60	1.30E-58
CKS1B	1358	792	-0.362759012	8.64E-09	5.04E-08
GPAA1	4244	2475	-0.362843999	2.51E-24	4.24E-23
NDUFB7	1952	1138	-0.363300364	4.85E-12	3.88E-11
RHBDF1	3122	1820	-0.363379955	2.32E-18	2.93E-17
SH3BP1	1328	774	-0.363697543	1.16E-08	6.66E-08
RAPGEF4	604	352	-0.363820989	0.0001187	0.000390242
TNIP2	1239	722	-0.363953314	3.50E-08	1.92E-07
MEN1	1493	870	-0.363974728	1.42E-09	8.97E-09
TMEM51	908	529	-0.364272443	2.31E-06	1.01E-05
ALKBH6	563	328	-0.364286976	0.0001989	0.000624929
NUMBL	2118	1233	-0.365377658	4.61E-13	4.00E-12
ZNF358	1273	741	-0.36553484	2.00E-08	1.12E-07
WDR61	1706	993	-0.365599892	8.13E-11	5.80E-10
NUBP2	1201	699	-0.365719659	4.93E-08	2.66E-07
FAM53B	744	433	-0.365783465	1.76E-05	6.75E-05
ALYREF	2052	1194	-0.366075763	9.64E-13	8.13E-12
ILVBL	1808	1052	-0.366107841	2.11E-11	1.59E-10
PIDD	1344	782	-0.366140494	7.66E-09	4.50E-08
PISD	1650	960	-0.366207582	1.55E-10	1.08E-09
SLC35F2	686	399	-0.366667698	3.60E-05	0.000131177
ZMIZ1	10008	5820	-0.366910505	3.47E-56	1.47E-54
SLC35A2	1632	949	-0.367008933	1.79E-10	1.25E-09
SHANK3	951	553	-0.367013729	1.12E-06	5.11E-06
LLGL1	3139	1825	-0.367256433	8.59E-19	1.11E-17
SPRN	762	443	-0.367332167	1.29E-05	5.03E-05
MAPK7	1082	629	-0.367416445	2.00E-07	1.01E-06

RXRA	2257	1312	-0.367486567	5.89E-14	5.45E-13
SOX4	3953	2297	-0.368044967	2.43E-23	3.95E-22
TARS2	1024	595	-0.36810201	4.05E-07	1.95E-06
G6PD	7394	4295	-0.36854478	2.62E-42	7.97E-41
MYBBP1A	2331	1354	-0.368569134	1.95E-14	1.86E-13
ZBTB2	1512	878	-0.369013163	6.78E-10	4.42E-09
B4GALNT4	1557	904	-0.369222135	3.72E-10	2.49E-09
OLFML2A	751	436	-0.369332641	1.35E-05	5.25E-05
NDRG4	2397	1391	-0.369955357	6.78E-15	6.70E-14
GLCCI1	986	572	-0.370420368	5.70E-07	2.70E-06
PIKFYVE	5558	3224	-0.370561959	1.53E-32	3.48E-31
ATAT1	676	392	-0.37101746	3.37E-05	0.000123507
GPR173	821	476	-0.371268517	4.80E-06	2.01E-05
SLC26A2	3518	2039	-0.371741576	2.58E-21	3.78E-20
CHERP	1672	969	-0.371854145	6.31E-11	4.55E-10
PPP1R9B	5386	3121	-0.372053741	8.21E-32	1.84E-30
CCDC137	1353	784	-0.372084148	4.03E-09	2.44E-08
VPS9D1	732	424	-0.372627252	1.47E-05	5.71E-05
ACAP3	3689	2136	-0.37316601	2.02E-22	3.14E-21
PACSIN3	1059	613	-0.373591479	1.75E-07	8.83E-07
SLC2A13	1681	973	-0.373655883	4.60E-11	3.36E-10
ENO2	2120	1227	-0.373776884	1.41E-13	1.27E-12
SAMD4B	4809	2782	-0.374462375	6.56E-29	1.31E-27
EYA3	2106	1218	-0.374839171	1.46E-13	1.31E-12
BTBD2	4209	2433	-0.375589058	1.21E-25	2.17E-24
SIPA1	1967	1137	-0.375612572	8.29E-13	7.03E-12
ITPR3	1545	893	-0.375722626	2.24E-10	1.54E-09
ARSB	3338	1929	-0.37597868	1.05E-20	1.48E-19
STK24	5038	2911	-0.376184151	1.88E-30	3.98E-29
FAM102A	1279	739	-0.376217863	7.50E-09	4.41E-08
TNFRSF19	4227	2442	-0.37641878	7.61E-26	1.37E-24
C19orf53	2832	1636	-0.376496385	7.58E-18	9.26E-17

BOP1	2647	1529	-0.376617656	8.62E-17	9.72E-16
NPRL2	561	324	-0.376854826	0.0001263	0.000413082
CCNB2	1690	976	-0.376918062	2.84E-11	2.12E-10
MARCKSL1	6281	3627	-0.377065392	1.05E-37	2.76E-36
KIFC2	646	373	-0.377206403	3.83E-05	0.000138766
NAA50	12401	7159	-0.377474346	7.40E-73	4.10E-71
PFDN2	1797	1037	-0.378022383	5.94E-12	4.73E-11
FZD8	513	296	-0.378209518	0.0002349	0.000728783
C6orf1	747	431	-0.378268242	9.03E-06	3.63E-05
ENTPD1	981	566	-0.378298952	3.63E-07	1.76E-06
ITGA7	1136	655	-0.379243891	4.08E-08	2.22E-07
FAM155A	4288	2471	-0.380057763	1.22E-26	2.27E-25
OST4	1743	1004	-0.380661168	9.04E-12	7.07E-11
UPRT	667	384	-0.381428319	2.36E-05	8.89E-05
PDZRN3	761	438	-0.381813452	6.19E-06	2.55E-05
PODXL	1175	676	-0.382413473	1.86E-08	1.05E-07
TENM4	1256	722	-0.38361359	5.47E-09	3.26E-08
MAPK3	2893	1663	-0.383626023	8.62E-19	1.11E-17
E2F1	588	338	-0.383640777	6.59E-05	0.000227842
BCL10	2088	1200	-0.383935174	5.25E-14	4.86E-13
TRPV4	517	297	-0.384549218	0.0001766	0.000561184
ASB6	1760	1011	-0.3846403	4.48E-12	3.60E-11
CHAF1B	646	371	-0.384962846	2.71E-05	0.000101296
CAMTA1	1010	580	-0.385078356	1.53E-07	7.81E-07
GPR56	1060	608	-0.386768904	6.70E-08	3.57E-07
VPS16	1184	679	-0.38703347	1.13E-08	6.51E-08
ABCC10	1748	1002	-0.387670545	3.70E-12	3.00E-11
GBP1	2567	1471	-0.388133918	3.47E-17	4.02E-16
SIVA1	975	558	-0.389984965	1.81E-07	9.13E-07
TUBG2	554	317	-0.390251004	8.29E-05	0.000280888
GALNT15	1851	1059	-0.390450174	6.12E-13	5.26E-12
GMIP	1112	636	-0.390905986	2.34E-08	1.30E-07

CDCA3	883	505	-0.390977918	6.44E-07	3.02E-06
PRKAA1	10026	5734	-0.390980198	3.78E-63	1.77E-61
STUB1	1380	789	-0.39141893	4.69E-10	3.10E-09
LSM10	934	534	-0.391430676	2.98E-07	1.47E-06
TGFBR3	1011	578	-0.391489468	9.68E-08	5.05E-07
HIPK2	24032	13736	-0.391842642	2.49E-149	3.26E-147
BLOC1S3	644	368	-0.39220279	2.01E-05	7.68E-05
CENPW	631	360	-0.394490967	2.21E-05	8.35E-05
ANGPTL2	4297	2451	-0.394807161	1.55E-28	3.07E-27
UNC93B1	954	544	-0.395230483	1.73E-07	8.76E-07
SLC27A4	1358	774	-0.395925876	4.22E-10	2.81E-09
FMNL2	2837	1616	-0.396786821	1.49E-19	1.99E-18
STX1B	783	446	-0.396816466	2.01E-06	8.88E-06
IRAK1	7270	4141	-0.39682403	1.57E-47	5.55E-46
VAMP1	546	311	-0.396834239	7.23E-05	0.000248075
WLS	24127	13740	-0.397114403	1.22E-153	1.66E-151
ATP6V0E2	1095	623	-0.39847467	1.68E-08	9.53E-08
SMAD9	1930	1098	-0.398570661	6.78E-14	6.24E-13
POLR3K	531	302	-0.39901118	8.36E-05	0.000283062
RASSF1	973	553	-0.400008193	9.43E-08	4.93E-07
SLC16A2	5591	3176	-0.400743301	1.32E-37	3.45E-36
LAMTOR2	1368	777	-0.400929595	2.26E-10	1.56E-09
KIAA1107	567	322	-0.401135915	4.41E-05	0.000157406
FAM196B	1289	732	-0.401184578	7.27E-10	4.73E-09
HRSP12	1046	594	-0.401195884	2.87E-08	1.58E-07
SMG9	1135	644	-0.402407572	6.76E-09	3.99E-08
RFNG	1306	741	-0.402457317	5.01E-10	3.31E-09
DTYMK	1329	754	-0.402552544	3.50E-10	2.35E-09
NDRG1	4204	2385	-0.402621271	6.24E-29	1.25E-27
APCDD1L	3425	1943	-0.402665955	7.05E-24	1.17E-22
MRPS26	841	477	-0.402964403	5.84E-07	2.76E-06
FBXO17	566	321	-0.403076624	4.13E-05	0.000148674

PCED1A	917	520	-0.403257979	1.78E-07	9.01E-07
C16orf13	1196	678	-0.403708079	2.39E-09	1.48E-08
FAM63B	3237	1835	-0.403725171	9.27E-23	1.47E-21
WDR62	690	391	-0.404275623	5.64E-06	2.35E-05
AP5S1	535	303	-0.405068966	6.22E-05	0.000216007
ASPM	4792	2713	-0.405586729	3.60E-33	8.34E-32
CANT1	5499	3113	-0.405711578	7.81E-38	2.08E-36
C19orf60	1009	571	-0.406211392	3.51E-08	1.92E-07
CDC42EP5	753	426	-0.406644303	1.86E-06	8.27E-06
METTL14	1100	622	-0.407364907	7.83E-09	4.59E-08
CACTIN	766	433	-0.407825235	1.42E-06	6.41E-06
METRN	1081	611	-0.407970106	1.00E-08	5.81E-08
KIF5B	25857	14614	-0.408051655	6.11E-173	9.76E-171
EMP3	10098	5707	-0.408112983	1.01E-68	5.32E-67
SLC29A1	630	356	-0.408322456	1.20E-05	4.71E-05
EBAG9	588	332	-0.409480782	2.23E-05	8.44E-05
DCHS1	1674	945	-0.409761162	8.10E-13	6.89E-12
DTX4	2404	1357	-0.409864044	9.34E-18	1.14E-16
EXOSC9	1245	702	-0.411450675	5.72E-10	3.76E-09
RGS12	1302	734	-0.411725349	2.27E-10	1.56E-09
FASTK	2046	1153	-0.4122615	1.72E-15	1.77E-14
MPP6	669	377	-0.412289556	5.32E-06	2.22E-05
MMP1	11264	6346	-0.412647681	5.46E-78	3.18E-76
RUSC1	1988	1119	-0.413955589	3.37E-15	3.41E-14
PTPRN	1354	762	-0.414212704	7.83E-11	5.60E-10
DTD1	2417	1360	-0.41445869	3.48E-18	4.37E-17
SCRIB	3745	2107	-0.414623273	2.57E-27	4.88E-26
SCAMP3	3245	1825	-0.415169882	6.09E-24	1.02E-22
DECR2	683	384	-0.415627136	3.59E-06	1.53E-05
MPG	2243	1261	-0.415709213	4.50E-17	5.17E-16
BUB1	2213	1244	-0.415864834	7.08E-17	8.03E-16
CDC25B	3672	2064	-0.415970956	5.74E-27	1.08E-25

MAT2B	2827	1589	-0.416000627	3.89E-21	5.63E-20
ABHD11	520	292	-0.417391122	4.92E-05	0.000174469
TUBA4A	661	371	-0.418078953	4.57E-06	1.93E-05
SRXN1	3277	1839	-0.418302061	1.77E-24	3.02E-23
RASA3	5086	2854	-0.418393995	4.37E-37	1.12E-35
PEX10	540	303	-0.418489482	3.37E-05	0.000123777
SMAP2	3693	2072	-0.418617128	2.05E-27	3.91E-26
AGPAT1	5328	2987	-0.419744713	5.44E-39	1.48E-37
SMARCB1	2924	1639	-0.419975325	3.63E-22	5.59E-21
ATAD3A	1904	1067	-0.420321171	5.37E-15	5.37E-14
GAL3ST4	639	358	-0.420704212	5.82E-06	2.41E-05
FAM213B	1448	811	-0.421135651	8.49E-12	6.66E-11
RAN	13870	7767	-0.421386285	2.61E-99	1.99E-97
C9orf89	937	524	-0.423330104	3.37E-08	1.85E-07
CECR5	626	350	-0.423655603	6.31E-06	2.60E-05
DOK1	1118	625	-0.423839962	1.57E-09	9.90E-09
TBX5-AS1	2444	1366	-0.42413467	4.18E-19	5.48E-18
STX1A	562	314	-0.42465344	1.80E-05	6.91E-05
PPM1M	804	449	-0.425327925	2.80E-07	1.38E-06
MCL1	17644	9844	-0.426708971	1.01E-128	1.08E-126
TEAD2	2139	1193	-0.427190308	4.04E-17	4.65E-16
STK4	2388	1331	-0.428140134	5.28E-19	6.87E-18
HHEX	646	360	-0.428385127	3.57E-06	1.53E-05
GNB5	1389	774	-0.428488996	1.06E-11	8.25E-11
UBE2S	4937	2750	-0.429050895	1.09E-37	2.87E-36
GPR137	896	499	-0.429306785	4.52E-08	2.45E-07
COMMD6	2844	1583	-0.430108078	1.66E-22	2.59E-21
QPCTL	577	321	-0.43084589	1.07E-05	4.22E-05
RANBP1	4485	2495	-0.430916038	1.17E-34	2.81E-33
MRPL41	1383	769	-0.431593521	8.59E-12	6.74E-11
SMTN	7219	4014	-0.431606256	7.06E-55	2.88E-53
ERLIN2	6501	3613	-0.432312268	9.65E-50	3.56E-48

REPIN1	2465	1369	-0.433313068	5.70E-20	7.77E-19
TSR3	1565	869	-0.433582443	2.99E-13	2.63E-12
C11orf48	1629	904	-0.434439794	8.92E-14	8.13E-13
NFKBIA	1238	687	-0.434477179	8.01E-11	5.73E-10
TK1	2127	1179	-0.436104183	1.23E-17	1.48E-16
MYD88	2178	1207	-0.436426146	4.81E-18	5.97E-17
SSX2IP	5060	2801	-0.43804327	5.02E-40	1.43E-38
ZFP1	804	445	-0.438238034	1.29E-07	6.64E-07
CACNB3	1820	1007	-0.438722635	1.82E-15	1.87E-14
E2F4	1806	999	-0.439089178	2.22E-15	2.27E-14
KLHL17	718	397	-0.439692705	5.58E-07	2.64E-06
CDC42EP4	2306	1275	-0.439743134	2.93E-19	3.87E-18
VWA1	834	461	-0.440128501	6.68E-08	3.56E-07
C2CD2	3826	2114	-0.440709365	5.25E-31	1.14E-29
ZFYVE27	1204	665	-0.441257015	7.88E-11	5.64E-10
CHST12	891	492	-0.441614984	2.16E-08	1.21E-07
CENPK	585	323	-0.441750328	5.69E-06	2.36E-05
FGL2	681	376	-0.441770005	9.79E-07	4.51E-06
PDGFRL	777	429	-0.441784819	1.70E-07	8.61E-07
ALG12	980	541	-0.442001023	4.20E-09	2.54E-08
ABHD14B	1125	621	-0.442107696	3.03E-10	2.05E-09
GPS2	1117	616	-0.443474798	3.12E-10	2.10E-09
CAMK1	1041	574	-0.443695295	1.22E-09	7.79E-09
RAB11B	2107	1161	-0.44467021	4.55E-18	5.67E-17
IGFBP6	5707	3144	-0.444979214	3.43E-46	1.16E-44
ANO8	701	386	-0.445661465	5.51E-07	2.61E-06
SOBP	632	348	-0.445685121	1.98E-06	8.78E-06
RRP9	881	485	-0.44600514	1.93E-08	1.09E-07
SEC14L2	654	360	-0.446141597	1.29E-06	5.84E-06
SAMD1	640	352	-0.447344345	1.57E-06	7.06E-06
RTKN	797	438	-0.448496723	7.84E-08	4.14E-07
TRIM3	954	524	-0.449270323	3.97E-09	2.41E-08

CACNA1A	1648	905	-0.449574413	9.92E-15	9.66E-14
NAB2	1957	1074	-0.450497631	2.89E-17	3.37E-16
SLC38A5	1059	581	-0.450940389	4.90E-10	3.24E-09
AFMID	713	391	-0.451581337	3.18E-07	1.56E-06
RPL28	20049	10990	-0.452186762	2.78E-162	4.03E-160
NRM	564	309	-0.452936193	5.13E-06	2.15E-05
KCTD10	6358	3482	-0.453504682	5.09E-53	2.01E-51
CDC20	2541	1391	-0.454121824	2.99E-22	4.61E-21
SHKBP1	2985	1634	-0.454170816	7.33E-26	1.32E-24
RFXANK	897	491	-0.454232829	8.16E-09	4.77E-08
PHLPP2	1760	963	-0.454815594	6.22E-16	6.67E-15
TLN2	3715	2032	-0.455309677	6.41E-32	1.45E-30
SERPINE2	10209	5584	-0.455318579	1.25E-84	8.34E-83
ZNF503	2575	1408	-0.455772967	1.14E-22	1.80E-21
ESRRA	995	544	-0.455937742	1.11E-09	7.11E-09
MFSD6	728	398	-0.456017888	1.86E-07	9.40E-07
GAS7	518	283	-0.456997913	1.06E-05	4.19E-05
SELO	828	452	-0.458155863	2.37E-08	1.32E-07
HSD17B1	565	308	-0.460168385	3.65E-06	1.56E-05
JAG1	2186	1191	-0.460967856	7.33E-20	9.93E-19
GINS2	659	359	-0.461142489	5.43E-07	2.58E-06
CHTF18	562	306	-0.461886346	3.59E-06	1.53E-05
SALL1	2274	1238	-0.462068808	1.11E-20	1.57E-19
EVL	1218	663	-0.462281226	8.65E-12	6.78E-11
PPM1F	4458	2426	-0.462664934	4.76E-39	1.30E-37
RAC2	1570	854	-0.463304452	8.03E-15	7.88E-14
PITPNM2	1899	1032	-0.464644803	1.08E-17	1.31E-16
SOAT1	5947	3231	-0.465029193	5.66E-52	2.19E-50
DNAJB1	7323	3978	-0.465239388	1.24E-63	5.97E-62
C8orf82	753	409	-0.46539689	6.61E-08	3.52E-07
MTA2	5881	3194	-0.465545046	1.62E-51	6.21E-50
HPCAL1	1427	775	-0.465564988	1.02E-13	9.28E-13

TINF2	1258	683	-0.466022307	2.75E-12	2.26E-11
SCAND1	1278	693	-0.467808447	1.55E-12	1.29E-11
BIN3	1223	663	-0.468191497	4.46E-12	3.59E-11
CMTM7	583	316	-0.468419193	1.74E-06	7.76E-06
TMCO4	969	525	-0.469027111	6.78E-10	4.42E-09
FBRSL1	1110	601	-0.469970649	3.66E-11	2.71E-10
PEX16	549	297	-0.471191086	3.09E-06	1.33E-05
LHFPL2	2500	1352	-0.471680812	2.19E-23	3.58E-22
PCDH18	15905	8601	-0.47175193	1.88E-139	2.21E-137
ARL6IP1	8536	4614	-0.47239004	6.50E-76	3.72E-74
TUBB2A	2645	1428	-0.474119611	7.22E-25	1.25E-23
APMAP	7330	3955	-0.474983372	4.31E-66	2.13E-64
SPATA13	4260	2298	-0.475322501	3.33E-39	9.15E-38
ATP13A2	1752	944	-0.476991878	3.53E-17	4.09E-16
TIMELESS	1722	927	-0.478291767	5.48E-17	6.27E-16
PITPNB	4734	2548	-0.478542297	6.83E-44	2.18E-42
ATP6V1C1	5110	2750	-0.478739541	2.84E-47	9.98E-46
SLC14A1	3813	2052	-0.478743668	1.03E-35	2.52E-34
ADAT1	1563	841	-0.478987941	1.34E-15	1.41E-14
STK16	714	384	-0.479665632	6.39E-08	3.41E-07
CLIC4	71124	38248	-0.479800021	0	0
C12orf49	1995	1072	-0.480931709	1.29E-19	1.73E-18
PCDHGA12	548	294	-0.483207606	1.85E-06	8.20E-06
SRRM1	6825	3661	-0.483439141	1.24E-63	6.00E-62
FSD1	552	296	-0.483918959	1.64E-06	7.34E-06
ATP8B2	7787	4174	-0.484484894	1.27E-72	7.02E-71
RHOQ	7161	3837	-0.48503017	4.57E-67	2.30E-65
LBR	2424	1298	-0.485947184	6.55E-24	1.09E-22
RCC1	1299	694	-0.489241731	1.11E-13	1.01E-12
ANO4	6278	3349	-0.491422521	2.08E-60	9.43E-59
PGBD1	572	305	-0.492053773	7.26E-07	3.38E-06
CABLES1	1753	934	-0.493179409	3.58E-18	4.48E-17

MRGPRF	2679	1424	-0.496593303	3.00E-27	5.68E-26
MMD	1221	649	-0.496620685	2.88E-13	2.54E-12
LTBP4	4267	2266	-0.497922118	1.34E-42	4.13E-41
KIF7	1563	830	-0.497982405	1.23E-16	1.37E-15
FENDRR	10513	5581	-0.498426734	1.81E-102	1.43E-100
DNAJC16	2965	1573	-0.499361302	2.86E-30	6.01E-29
EEF1A2	895	474	-0.501848492	2.79E-10	1.90E-09
IL4R	3050	1614	-0.503016532	1.74E-31	3.87E-30
POSTN	8991	4755	-0.503884112	1.22E-89	8.66E-88
HMMR	2147	1135	-0.504477762	9.24E-23	1.47E-21
D2HGDH	895	473	-0.504895367	2.23E-10	1.54E-09
SVIL	12838	6783	-0.505272936	8.49E-128	8.96E-126
SPIN4	648	342	-0.506845356	6.03E-08	3.22E-07
PLEKHG4	870	459	-0.507369116	3.31E-10	2.22E-09
DDX50	3455	1822	-0.50801062	4.70E-36	1.16E-34
FAM65C	1610	849	-0.508072098	1.14E-17	1.38E-16
GOLGA4	11974	6313	-0.508355387	1.23E-120	1.20E-118
GPD1L	865	456	-0.508514177	3.42E-10	2.29E-09
FZD1	3327	1753	-0.509243738	6.28E-35	1.52E-33
SEMA3B	1038	546	-0.511681456	4.62E-12	3.71E-11
SMAD6	700	368	-0.512497024	1.28E-08	7.33E-08
MYO19	1666	875	-0.513881347	1.40E-18	1.79E-17
STRA13	991	520	-0.515221302	1.04E-11	8.08E-11
MGAT2	5049	2649	-0.515397702	3.22E-53	1.27E-51
C11orf68	2417	1266	-0.517787937	1.42E-26	2.63E-25
NR1D1	611	320	-0.517948343	8.06E-08	4.24E-07
ARSI	575	301	-0.518646337	1.87E-07	9.42E-07
TRAM1	24304	12711	-0.519964109	6.53E-253	1.98E-250
ID1	5486	2867	-0.521060586	8.62E-59	3.77E-57
ADAMTS1	22573	11789	-0.52200469	1.04E-236	2.59E-234
COASY	2028	1059	-0.522202931	6.93E-23	1.11E-21
GTPBP1	2932	1529	-0.524144565	1.46E-32	3.33E-31

SLC6A15	971	506	-0.525181779	7.37E-12	5.82E-11
LRCH2	2649	1379	-0.526673255	7.95E-30	1.65E-28
SNHG3	842	438	-0.527737232	1.48E-10	1.04E-09
SVIP	921	479	-0.528023369	2.02E-11	1.53E-10
PTGS1	16045	8344	-0.528160595	1.97E-172	3.10E-170
LIMK1	3771	1961	-0.528205483	5.98E-42	1.80E-40
RPS6KA2	4762	2476	-0.528404174	1.52E-52	5.92E-51
GLTPD1	1403	729	-0.529372158	1.10E-16	1.23E-15
NME3	970	504	-0.529408882	5.32E-12	4.25E-11
PAQR5	903	469	-0.529985933	2.72E-11	2.04E-10
IMP4	1498	778	-0.530043432	9.48E-18	1.15E-16
BRSK1	676	351	-0.530400084	8.06E-09	4.71E-08
VAT1L	2733	1418	-0.531475796	3.35E-31	7.32E-30
TTC39C	937	486	-0.531940602	9.91E-12	7.72E-11
KCTD12	7574	3927	-0.532475691	1.27E-83	8.31E-82
EPHB4	3057	1585	-0.53248158	8.18E-35	1.97E-33
TMEM194A	2104	1089	-0.534978619	1.13E-24	1.95E-23
SEPT8	7383	3820	-0.535482389	2.23E-82	1.41E-80
CPT1C	1120	579	-0.536711347	6.21E-14	5.74E-13
ANKRD12	4111	2125	-0.536874399	6.89E-47	2.40E-45
ID3	5050	2610	-0.537081449	3.15E-57	1.35E-55
CCDC142	542	280	-0.537713893	1.71E-07	8.65E-07
CHL1	513	265	-0.537814334	3.62E-07	1.76E-06
RACGAP1	3362	1727	-0.54589951	8.52E-40	2.41E-38
BOK	1509	775	-0.546172459	8.85E-19	1.14E-17
DPP4	4493	2307	-0.546508926	1.08E-52	4.20E-51
SAMD14	653	335	-0.547769764	5.34E-09	3.19E-08
CPT1A	4705	2413	-0.548214676	2.13E-55	8.85E-54
GPR161	2078	1063	-0.551901926	1.04E-25	1.87E-24
TMEM132B	2266	1158	-0.553360476	5.18E-28	1.01E-26
KIF20B	2582	1319	-0.553892304	9.73E-32	2.17E-30
GCSH	1513	771	-0.557457091	1.78E-19	2.37E-18

HS6ST1	6662	3394	-0.557816657	4.29E-80	2.63E-78
TOP1MT	710	361	-0.560668056	5.13E-10	3.39E-09
RARG	1562	794	-0.561031409	2.86E-20	3.97E-19
ADAM10	15978	8092	-0.566366478	1.38E-194	2.65E-192
RNF215	624	316	-0.566469339	4.08E-09	2.47E-08
ABCC1	16452	8331	-0.566549262	2.05E-200	4.19E-198
DENND2A	1719	868	-0.570650465	8.70E-23	1.39E-21
PSD3	6415	3238	-0.571194148	1.75E-80	1.08E-78
CCDC18	795	401	-0.572200492	2.11E-11	1.59E-10
ABCC4	11880	5986	-0.57371852	1.46E-148	1.85E-146
RAB14	9142	4603	-0.574783245	3.73E-115	3.42E-113
NR4A2	976	491	-0.576005991	8.24E-14	7.54E-13
CYP2S1	766	385	-0.577333815	3.40E-11	2.52E-10
CCDC85B	3580	1799	-0.577612269	1.30E-46	4.50E-45
BDKRB2	2813	1413	-0.578195955	4.62E-37	1.19E-35
SDPR	3522	1769	-0.578308729	5.57E-46	1.89E-44
NT5DC3	1804	905	-0.58005751	1.69E-24	2.90E-23
RPS28	8812	4407	-0.584520467	2.04E-114	1.84E-112
TSPAN9	2704	1352	-0.584847868	2.13E-36	5.33E-35
GEM	1427	712	-0.587884057	3.73E-20	5.14E-19
CTDSP1	2200	1096	-0.590103594	2.19E-30	4.63E-29
EXT1	11865	5910	-0.590329933	4.38E-156	6.06E-154
TUBA1C	21699	10798	-0.591712304	6.01E-285	2.14E-282
AMD1	5800	2883	-0.593329932	5.62E-78	3.25E-76
FAM43A	672	334	-0.593460999	1.97E-10	1.37E-09
ALKBH5	4102	2037	-0.59472938	6.48E-56	2.72E-54
WDR24	712	353	-0.597056926	4.51E-11	3.30E-10
FAM69A	2187	1084	-0.597436332	7.41E-31	1.60E-29
HSPA2	918	455	-0.597475477	7.25E-14	6.66E-13
C4orf48	892	442	-0.597845209	1.58E-13	1.41E-12
SKI	7170	3552	-0.598189405	2.47E-97	1.85E-95
RANBP3	2686	1330	-0.598880927	1.15E-37	3.01E-36

NBPF14	1340	663	-0.600000093	1.13E-19	1.52E-18
ATP6V1F	2249	1110	-0.603571853	3.08E-32	6.97E-31
LXN	1728	851	-0.606720049	2.25E-25	4.00E-24
SMARCC1	9192	4525	-0.607308874	1.36E-127	1.42E-125
CENPF	8322	4095	-0.607914705	6.62E-116	6.16E-114
ARNTL2	3498	1716	-0.612328605	2.03E-50	7.58E-49
GALM	738	362	-0.612478987	6.88E-12	5.45E-11
UXS1	3368	1648	-0.616023764	4.06E-49	1.47E-47
ZEB2	4698	2297	-0.617143725	5.15E-68	2.67E-66
TRIM16	844	412	-0.61944653	1.26E-13	1.13E-12
EI24	5898	2879	-0.619505902	1.77E-85	1.19E-83
SLC44A1	2106	1028	-0.61951304	1.16E-31	2.59E-30
PKIB	693	338	-0.620679974	1.73E-11	1.32E-10
TUBB3	11927	5817	-0.620731866	1.87E-171	2.91E-169
INSC	857	416	-0.627559544	4.19E-14	3.91E-13
PITX1	4728	2279	-0.637676979	1.95E-72	1.07E-70
SALL2	1092	525	-0.641431397	3.45E-18	4.34E-17
APH1A	3714	1784	-0.642706068	4.48E-58	1.92E-56
SEP15	6650	3191	-0.6441936	6.36E-103	5.14E-101
DAZAP2	6479	3099	-0.648816271	1.39E-101	1.09E-99
DCPS	542	259	-0.650188622	5.63E-10	3.70E-09
SNRPA1	1585	757	-0.650965503	2.55E-26	4.68E-25
CNOT6	3162	1510	-0.651136686	7.83E-51	2.96E-49
TWIST2	571	272	-0.654731962	1.52E-10	1.06E-09
SYDE1	4380	2084	-0.65642346	1.08E-70	5.79E-69
CPNE7	749	354	-0.666064227	9.60E-14	8.74E-13
BDKRB1	4103	1933	-0.670685385	7.69E-69	4.06E-67
SYT11	3851	1806	-0.677283099	8.01E-66	3.94E-64
PTX3	4880	2277	-0.684594725	1.84E-84	1.22E-82
PPIF	2987	1391	-0.687422686	8.83E-53	3.46E-51
ACVRL1	1057	490	-0.693969591	4.64E-20	6.35E-19
PFKFB3	3947	1820	-0.701665937	1.25E-71	6.75E-70

NUPL1	4308	1985	-0.702727111	3.25E-78	1.90E-76
MAP3K11	2755	1263	-0.710045548	1.42E-51	5.47E-50
RCBTB1	2472	1133	-0.71037875	1.70E-46	5.80E-45
SLC4A4	7865	3592	-0.715507284	1.14E-145	1.39E-143
NUP98	7343	3341	-0.720937486	6.38E-138	7.29E-136
AMOT	597	271	-0.724285948	8.28E-13	7.03E-12
AIM1	1378	625	-0.725495661	1.31E-27	2.50E-26
GREM2	730	329	-0.734656748	1.12E-15	1.18E-14
GTDC2	612	275	-0.738947903	1.66E-13	1.49E-12
NDNL2	1079	484	-0.74146378	9.24E-23	1.47E-21
TMOD1	539	241	-0.746099995	2.99E-12	2.45E-11
TNRC6B	5115	2284	-0.748019458	5.78E-103	4.70E-101
TFPI2	8927	3970	-0.753884287	1.24E-180	2.09E-178
TSPO	5084	2259	-0.755127628	5.11E-104	4.22E-102
ANKRD18B	714	316	-0.760847384	3.10E-16	3.38E-15
KCNF1	739	327	-0.761131597	9.25E-17	1.04E-15
NACAD	1277	564	-0.763839326	5.76E-28	1.12E-26
NSF	6417	2817	-0.772587288	5.48E-136	6.09E-134
HGF	7566	3280	-0.79068283	1.98E-166	2.98E-164
FAM105A	526	228	-0.790876843	4.14E-13	3.61E-12
CENPE	2058	892	-0.790975235	1.16E-46	4.01E-45
PLAT	13138	5592	-0.817157273	4.11E-304	1.60E-301
NCAPD3	2078	880	-0.824468094	1.89E-50	7.12E-49
PRRX2	1413	589	-0.847269795	1.91E-36	4.80E-35
TFG	8446	3511	-0.851234119	2.62E-210	5.83E-208
MAPRE2	2485	1031	-0.854049387	1.37E-63	6.59E-62
TIMP3	32362	13331	-0.864363633	0	0
BRCC3	1767	720	-0.880081096	4.90E-48	1.76E-46
PIEZO2	761	307	-0.894505666	3.37E-22	5.19E-21
TMEM9	1802	726	-0.896405426	1.92E-50	7.21E-49
FAM117B	658	265	-0.896943093	1.72E-19	2.29E-18
DEK	9115	3666	-0.898883739	1.48E-248	4.40E-246

ATOH8	838	332	-0.920614871	2.08E-25	3.71E-24
PER3	1401	546	-0.944331968	4.50E-43	1.39E-41
TBXAS1	920	356	-0.954604488	2.29E-29	4.66E-28
GABRB1	796	303	-0.978298505	1.25E-26	2.32E-25
FKBP5	871	325	-1.007080869	2.51E-30	5.30E-29
LIF	2447	904	-1.021467292	6.59E-84	4.34E-82
ADAMTS9	566	206	-1.043005584	2.20E-21	3.23E-20
WNT16	2614	949	-1.046627017	4.50E-93	3.29E-91
VASN	3815	1357	-1.076110205	4.19E-141	5.02E-139
C19orf24	769	265	-1.121839107	7.68E-32	1.72E-30
TRPA1	13632	4672	-1.12973293	0	0
FOXN3	5602	1827	-1.201313218	1.05E-245	2.91E-243
LINC00475	818	259	-1.243996614	1.73E-39	4.83E-38
NPTX1	44135	13655	-1.277341752	0	0
FAM32A	3866	1157	-1.325300641	4.25E-198	8.41E-196
PEX12	381	379	0.407558982	9.07E-05	0.000305188
PLSCR4	298	366	0.71168345	1.87E-10	1.30E-09
FLJ33630	323	365	0.591514431	6.67E-08	3.55E-07
C14orf28	356	362	0.439264588	4.13E-05	0.000148429
LMCD1	252	362	0.937718095	9.84E-16	1.04E-14
METTL21D	371	361	0.375731782	0.0003983	0.001184852
PGM5P2	375	360	0.356258443	0.0007672	0.002161522
FIGN	368	359	0.37943021	0.0003655	0.001096796
INADL	347	355	0.448035494	3.55E-05	0.000129494
TET1	365	353	0.366923851	0.0006151	0.001762721
ZCCHC2	348	351	0.427535856	8.23E-05	0.000278924
PDE4D	275	351	0.767191544	2.70E-11	2.02E-10
CCDC81	302	347	0.615539245	4.86E-08	2.63E-07
DEPDC1B	332	346	0.474740928	1.66E-05	6.41E-05
BBS12	317	345	0.537265653	1.48E-06	6.67E-06
ADM2	282	345	0.706053331	8.08E-10	5.24E-09
VLDLR	311	343	0.556446128	7.26E-07	3.38E-06

GPR63	334	342	0.449300354	4.71E-05	0.000167242
MYLIP	305	342	0.580339214	2.77E-07	1.37E-06
C1QTNF6	345	335	0.372716865	0.00071	0.00201252
KIF27	283	329	0.632437663	5.25E-08	2.83E-07
STXBP6	265	329	0.727247356	7.43E-10	4.83E-09
PDIK1L	292	328	0.582879577	4.38E-07	2.10E-06
CPE	316	327	0.464518209	4.08E-05	0.000146777
SATB1	306	327	0.510911115	7.58E-06	3.08E-05
FAT3	283	324	0.610343892	1.67E-07	8.49E-07
SLC4A5	295	318	0.523463943	6.38E-06	2.62E-05
RAB30	321	317	0.397061675	0.0004762	0.001396728
JPH2	306	317	0.466103319	5.07E-05	0.000178848
TTC30A	323	315	0.378969795	0.0008544	0.002383415
C12orf73	275	315	0.611072342	2.41E-07	1.20E-06
ST6GAL1	254	309	0.697930473	8.65E-09	5.05E-08
ZHX2	308	307	0.410460436	0.000391	0.00116546
CCDC176	248	306	0.718343664	4.26E-09	2.57E-08
VANGL2	297	302	0.43923775	0.0001805	0.000572474
ZNF215	287	299	0.474246879	6.36E-05	0.000220685
GPR85	503	293	-0.364505604	0.0004339	0.001281199
C14orf79	281	289	0.455651494	0.0001509	0.000486251
GPSM3	491	286	-0.364555746	0.0005075	0.001479847
DBF4B	495	285	-0.381314474	0.0002718	0.000834781
ANO1	138	285	1.461445784	3.69E-24	6.21E-23
ZNF641	286	284	0.405027914	0.0007553	0.002130837
C9orf142	482	280	-0.368454188	0.0005028	0.001467626
PEMT	505	277	-0.45124528	1.71E-05	6.58E-05
C7	274	277	0.430862215	0.0004262	0.001260682
ABHD8	505	276	-0.456462989	1.39E-05	5.42E-05
SGK196	276	276	0.415152132	0.000679	0.001931234
RNF150	222	274	0.718768348	2.68E-08	1.49E-07
SAPCD2	467	273	-0.359369467	0.0008213	0.002298699

PDE4B	474	272	-0.386128276	0.0003125	0.000950006
CDADC1	268	272	0.436525783	0.0004097	0.001215494
DRD1	258	272	0.491387718	8.14E-05	0.000276471
ABCA7	470	270	-0.384549218	0.0003492	0.001051566
ZNF689	480	267	-0.431042532	5.87E-05	0.000204825
PORCN	468	267	-0.394516656	0.0002579	0.000794774
ACER2	193	267	0.883391026	5.48E-11	3.98E-10
AK4	236	266	0.587791518	4.57E-06	1.92E-05
OGG1	495	265	-0.486284034	5.13E-06	2.15E-05
THNSL1	464	265	-0.392980314	0.0002884	0.000882467
RAMP1	230	264	0.6140562	2.05E-06	9.03E-06
PLXDC2	219	264	0.684759192	1.68E-07	8.55E-07
FAM227A	252	263	0.476791198	0.0001639	0.00052451
CHCHD6	449	262	-0.361996501	0.000957	0.002640433
OSBPL7	486	259	-0.492852084	4.80E-06	2.01E-05
LAGE3	464	259	-0.426020576	9.31E-05	0.000312293
ZNF513	473	258	-0.459316986	2.34E-05	8.83E-05
ELOVL2	255	258	0.43202595	0.0006525	0.001859257
PCDH10	168	258	1.034061964	1.97E-13	1.76E-12
LOC100287314	255	257	0.426423244	0.0007757	0.002181839
RNF152	499	256	-0.547743874	3.37E-07	1.65E-06
ZNF709	233	255	0.545319424	2.74E-05	0.000101995
ZNF300	486	254	-0.520975685	1.50E-06	6.75E-06
PPP1R35	454	253	-0.428402781	0.0001021	0.000340242
KAZALD1	455	252	-0.43729068	7.36E-05	0.00025207
RGS19	444	252	-0.401983811	0.0002923	0.00089327
ARL6	214	252	0.650965069	1.02E-06	4.70E-06
GFOD1	230	250	0.535446365	4.41E-05	0.000157483
BEX4	229	250	0.541732628	3.65E-05	0.000132886
FBXW9	227	249	0.548605577	3.08E-05	0.000113751
WISP1	470	248	-0.507168504	3.90E-06	1.66E-05
SLC38A4	434	247	-0.398031869	0.0003878	0.001157646

SIX5	489	246	-0.576024018	1.26E-07	6.49E-07
TRIM46	425	245	-0.37952896	0.0007828	0.002199316
HAUS4	433	244	-0.412333745	0.0002498	0.000771913
DOM3Z	423	243	-0.384549218	0.0006932	0.001968821
CHCHD5	423	242	-0.390498484	0.0005791	0.001672413
LPHN1	456	241	-0.504848546	5.94E-06	2.46E-05
OSGIN1	455	241	-0.501681267	6.87E-06	2.81E-05
WDR78	212	241	0.600121013	8.79E-06	3.54E-05
PABPN1	428	240	-0.419424259	0.0002162	0.000675378
ACTR3B	231	240	0.470293686	0.0003775	0.001129823
ICAM1	209	240	0.614683595	5.84E-06	2.42E-05
HTRA2	456	238	-0.522920119	2.93E-06	1.27E-05
ZNF785	414	238	-0.383517062	0.0008152	0.002283196
ZNF429	233	238	0.44578375	0.0007511	0.002120245
FAM173A	450	236	-0.51598601	4.45E-06	1.88E-05
RFX8	413	236	-0.39220279	0.0006399	0.001825865
MIR143HG	223	234	0.484616951	0.0003079	0.000937281
KLHL13	430	233	-0.468854573	3.96E-05	0.000143023
SLC25A10	409	233	-0.396618757	0.0005966	0.001716406
CLCN4	423	232	-0.451380726	8.29E-05	0.000280824
ADAM12	207	232	0.579646169	2.36E-05	8.89E-05
SLC2A6	433	229	-0.503867295	1.06E-05	4.19E-05
GATA6	210	229	0.540110402	8.13E-05	0.00027629
RNF144B	180	227	0.749847523	1.50E-07	7.67E-07
SYT16	154	225	0.962146782	9.98E-11	7.08E-10
ALKBH4	414	224	-0.470979904	5.14E-05	0.000181249
PRRT3	489	223	-0.717638623	1.32E-10	9.30E-10
IFIT2	436	222	-0.558616327	1.21E-06	5.50E-06
CTH	165	222	0.843245784	9.07E-09	5.26E-08
F11R	154	222	0.942781457	2.69E-10	1.83E-09
COL4A6	466	220	-0.667674299	3.96E-09	2.41E-08
SNAI1	408	219	-0.482486151	3.96E-05	0.000142967

CCDC102B	406	218	-0.481999461	4.21E-05	0.000150975
ATP8B3	422	213	-0.571237437	1.10E-06	5.03E-06
GSG2	199	211	0.4996267	0.0004258	0.001259701
OTUD1	178	211	0.660517889	5.89E-06	2.44E-05
NRXN3	126	211	1.158971397	3.08E-13	2.70E-12
RGS2	198	210	0.500041029	0.000435	0.001284311
AKAP6	192	210	0.544435149	0.0001442	0.000466548
HOXA5	408	209	-0.549914078	3.66E-06	1.56E-05
SAC3D1	425	208	-0.615727181	1.69E-07	8.57E-07
MFSD3	367	207	-0.410997164	0.0007748	0.002180451
WDR17	181	207	0.608793202	3.01E-05	0.000111371
RASL11A	381	206	-0.471994529	9.94E-05	0.000331721
LIMS2	195	206	0.494322345	0.0005653	0.001634606
TRABD2A	375	204	-0.463169312	0.0001477	0.000476481
ZNF699	182	204	0.579782834	7.34E-05	0.000251703
SYNPO2L	158	204	0.783796726	2.29E-07	1.14E-06
ARHGDIB	135	204	1.010761877	1.41E-10	9.90E-10
PTGS2	54	202	2.318476112	1.75E-31	3.87E-30
HAGHL	378	200	-0.503234103	3.94E-05	0.000142317
SMIM4	375	200	-0.491738464	6.09E-05	0.000211896
GBP2	395	198	-0.581200091	1.68E-06	7.50E-06
LPIN3	386	198	-0.547948285	7.17E-06	2.93E-05
LINC00346	427	197	-0.700888308	4.09E-09	2.47E-08
NLRP10	156	197	0.751801732	9.39E-07	4.33E-06
FBXL19-AS1	375	196	-0.52088481	2.39E-05	8.96E-05
TTI2	361	196	-0.465993051	0.0001809	0.000573714
PRRT2	354	196	-0.437743574	0.000465	0.001366908
CEND1	397	195	-0.610512752	5.25E-07	2.49E-06
TMEM133	184	195	0.498920489	0.0007171	0.002031303
TUBD1	172	194	0.588800219	8.83E-05	0.000297905
TNFRSF1B	434	193	-0.753942063	2.63E-10	1.80E-09
RIMS1	161	193	0.676692291	9.59E-06	3.84E-05

C9orf37	343	190	-0.437057026	0.000581	0.00167696
SPSB2	357	189	-0.502385708	6.63E-05	0.000229113
ZNF501	164	188	0.612188979	6.43E-05	0.000222719
GEMIN2	139	188	0.850799911	9.86E-08	5.14E-07
CDK20	170	187	0.552655655	0.0002773	0.000850721
SFMBT2	162	187	0.622196589	5.25E-05	0.00018509
METTL7A	408	186	-0.718114399	4.30E-09	2.60E-08
RPP25L	335	184	-0.449303198	0.0004869	0.001424997
ZNF793	173	184	0.50408586	0.0009092	0.0025205
CYGB	348	183	-0.512091526	6.13E-05	0.000213035
B4GALNT1	333	183	-0.448526397	0.0005168	0.001502643
FAM84B	165	183	0.564529756	0.0002461	0.000761643
PKMYT1	424	181	-0.812922436	2.63E-11	1.97E-10
PFKFB2	166	181	0.539958587	0.0004612	0.001356344
ANKRD39	329	178	-0.471058211	0.000306	0.000931528
PANK1	343	177	-0.539307084	3.04E-05	0.000112251
NOX4	155	177	0.606633277	0.0001199	0.00039382
C3orf62	150	176	0.64576506	5.01E-05	0.000177239
ZFPM1	334	174	-0.525608665	5.81E-05	0.000202945
KCNQ3	153	172	0.584029044	0.0002476	0.000765639
SPEF2	151	172	0.603012147	0.0001623	0.000520011
NPHP1	141	171	0.693453294	2.07E-05	7.87E-05
RRNAD1	313	170	-0.465475779	0.0004959	0.001449601
RFX3	115	170	0.979053017	1.17E-08	6.73E-08
SCML2	122	169	0.88529423	1.73E-07	8.76E-07
RPL39L	344	168	-0.6187952	2.28E-06	1.00E-05
WASH3P	332	168	-0.567569877	1.73E-05	6.64E-05
MURC	143	167	0.638985087	9.10E-05	0.000305757
FAM117A	137	165	0.683442263	3.65E-05	0.000132795
GABRE	138	164	0.66417968	5.98E-05	0.000208414
HHIP	76	164	1.524776623	2.07E-15	2.12E-14
SIK1	120	162	0.848111539	8.07E-07	3.75E-06

WASH2P	295	161	-0.458502134	0.000854	0.002382635
SDHAP2	304	160	-0.510847287	0.0001858	0.000587186
IL1RAPL1	141	159	0.588483735	0.0003887	0.001159854
CA13	132	159	0.683640968	5.04E-05	0.000177934
GPR137C	335	158	-0.669084405	5.74E-07	2.71E-06
PRR5	315	157	-0.589435138	1.49E-05	5.78E-05
ANKRD20A12P	137	157	0.611740798	0.0002624	0.000807775
LOC441081	291	155	-0.493598806	0.0003943	0.001173614
GHR	134	155	0.625187347	0.0002176	0.000679047
MAF	69	155	1.58275208	2.20E-15	2.24E-14
C8orf37	126	154	0.704658749	4.18E-05	0.000150046
SH3RF2	125	152	0.697295361	5.50E-05	0.000192939
FES	315	145	-0.704146797	3.95E-07	1.91E-06
ZDHHC14	280	145	-0.534221795	0.000187	0.000590194
PAQR4	283	144	-0.55958111	8.97E-05	0.000301829
PPM1L	122	144	0.654339796	0.0002061	0.000646379
ALS2CL	88	143	1.11559185	5.84E-09	3.48E-08
CCDC146	266	142	-0.490383184	0.000757	0.002135061
RPSAP52	269	141	-0.516758878	0.000381	0.001138851
DGKI	124	141	0.600507174	0.000669	0.001903789
ABCG2	393	140	-1.073950354	5.35E-16	5.76E-15
SAMD11	320	136	-0.819313122	5.54E-09	3.30E-08
TNFRSF25	278	136	-0.6163261	2.30E-05	8.66E-05
C1orf51	278	133	-0.648506505	9.41E-06	3.77E-05
KIAA1755	269	133	-0.601027795	4.65E-05	0.000165372
CCDC103	111	133	0.676018701	0.0002415	0.000747894
LOC613037	253	132	-0.523447324	0.00049	0.001433625
C8orf4	106	131	0.720654679	0.0001162	0.000382637
CXADR	90	130	0.945666848	1.26E-06	5.73E-06
AKAP5	105	129	0.71213387	0.0001538	0.000494196
NAT6	254	128	-0.573532555	0.0001481	0.000477689
SNAPC5	262	127	-0.629586183	2.80E-05	0.000104258

SLC35G1	110	127	0.622477105	0.0008559	0.002386387
PMS2P5	275	126	-0.710855753	1.77E-06	7.87E-06
LPAR2	254	126	-0.596252632	8.53E-05	0.000288221
B3GALT2	104	126	0.691992337	0.0002647	0.000814144
ZNF214	104	125	0.680496698	0.0003438	0.001037604
JAK3	99	125	0.751579796	9.41E-05	0.000315248
KIF26B	90	125	0.88908332	6.45E-06	2.65E-05
ZNF853	259	124	-0.647459846	1.95E-05	7.45E-05
CORIN	105	124	0.655102924	0.0005657	0.001634993
ANKRD1	39	124	2.083946223	1.11E-17	1.35E-16
CENPQ	278	123	-0.761274436	3.39E-07	1.66E-06
MED11	246	123	-0.584847868	0.0001446	0.000467275
TOX	241	119	-0.602919441	0.0001109	0.000366408
ACBD4	253	118	-0.685198394	9.11E-06	3.66E-05
MRM1	226	115	-0.55953678	0.0004651	0.001366788
C20orf24	286	114	-0.911829191	1.58E-09	9.94E-09
SYNGR3	258	114	-0.76318511	8.48E-07	3.93E-06
PSMC3IP	229	112	-0.616696734	0.0001206	0.000395896
FZD5	76	112	0.97457954	3.99E-06	1.69E-05
AKR1C1	315	109	-1.115871562	7.24E-14	6.65E-13
ANKRD16	261	109	-0.84456954	6.52E-08	3.47E-07
PPP1R3F	231	109	-0.668412585	3.35E-05	0.000123139
HSD17B2	211	108	-0.551059555	0.0008511	0.00237519
TRPC4	82	108	0.812487629	0.0001031	0.000343383
RNF112	239	107	-0.74424769	3.54E-06	1.52E-05
LCTL	207	106	-0.550414371	0.0009656	0.002662041
GPC2	235	105	-0.747119297	3.96E-06	1.68E-05
KCNC4	213	104	-0.61911777	0.0001983	0.000623267
HUNK	205	103	-0.577827441	0.0006	0.001725141
AVIL	76	103	0.853725145	7.61E-05	0.000260248
CALHM2	229	100	-0.780195467	2.26E-06	9.94E-06
RAPGEF3	204	100	-0.61341702	0.0003042	0.000926945

TPD52L1	76	100	0.811080808	0.0001908	0.00060106
ZNF487P	67	100	0.992919131	9.61E-06	3.84E-05
CORO1A	224	98	-0.777492946	3.12E-06	1.35E-05
ZNF688	202	96	-0.65809685	0.0001302	0.00042485
KIF21B	77	96	0.733328092	0.0008082	0.002265705
C16orf74	203	95	-0.680328177	7.82E-05	0.000266761
RFX2	190	95	-0.584847868	0.0008386	0.002343323
GCSAM	69	94	0.861216527	0.0001403	0.000454798
FAM213A	187	92	-0.608180372	0.0006001	0.001725057
TMEM86A	71	92	0.788966968	0.0004751	0.001394436
SLC37A2	207	91	-0.770540186	8.69E-06	3.51E-05
PGBD3	196	91	-0.691763072	8.20E-05	0.00027824
ZNRF2	63	91	0.945666848	5.03E-05	0.000177946
NS3BP	175	86	-0.609794225	0.0008763	0.002438355
WDR31	65	86	0.819049073	0.0004846	0.00141863
FBXO36	196	85	-0.790166776	9.75E-06	3.89E-05
VGF	181	84	-0.692376333	0.000152	0.000489475
TPT1-AS1	177	84	-0.660135996	0.0003291	0.000996266
OIP5	173	84	-0.627158673	0.0006928	0.001968192
C7orf69	56	84	1.000114632	4.49E-05	0.00016003
PLS1	52	84	1.107029836	9.26E-06	3.72E-05
TMED8	50	82	1.128847947	8.58E-06	3.47E-05
PDGFD	176	77	-0.777492946	3.58E-05	0.00013072
PTGES	175	77	-0.769272439	4.44E-05	0.000158488
KIF21A	49	76	1.048369801	5.47E-05	0.000191874
FAM86A	220	75	-1.137388891	2.14E-10	1.48E-09
CRLF1	165	72	-0.781245081	5.86E-05	0.000204684
CAMK4	157	72	-0.709543616	0.0003128	0.000950762
SLC51A	52	72	0.884637415	0.0006516	0.001857089
SETBP1	48	72	1.000114632	0.0001582	0.000508199
TEX9	51	70	0.872009807	0.0008977	0.002490686
SLC44A3	43	69	1.097411834	6.60E-05	0.000228231

ZNF517	162	68	-0.83723503	2.38E-05	8.95E-05
TMEM119	152	67	-0.766686191	0.000148	0.000477344
ADAMTS3	144	65	-0.732405057	0.0003865	0.00115417
ZNF483	45	65	0.945666848	0.000612	0.001754983
PTGER1	141	63	-0.747119297	0.0003524	0.00105988
SCN3A	147	62	-0.830323903	6.42E-05	0.000222448
ENAM	27	59	1.542907679	1.57E-06	7.04E-06
TRIM16L	131	58	-0.760289875	0.0004704	0.001381447
RASL11B	136	57	-0.839420695	0.0001043	0.000346917
IL13RA2	127	57	-0.740642541	0.0007634	0.0021517
DNAH7	24	55	1.611549345	1.70E-06	7.60E-06
CPEB1	220	54	-1.61132008	1.15E-16	1.29E-15
S100A2	137	54	-0.927992449	2.23E-05	8.45E-05
LPAR3	136	52	-0.971870991	1.13E-05	4.46E-05
LINC00622	29	52	1.257610855	0.0001129	0.000372906
C1orf226	136	51	-0.999885368	6.94E-06	2.84E-05
METTL12	118	48	-0.882528417	0.000162	0.000519119
CARD14	28	48	1.19275971	0.0003737	0.001119989
FGG	27	48	1.24522713	0.0002324	0.000721775
SLCO2A1	19	48	1.752187119	2.10E-06	9.25E-06
ANO3	123	47	-0.972773522	2.93E-05	0.000108641
LIPH	18	47	1.799815982	1.73E-06	7.72E-06
THEMIS2	115	45	-0.938484823	8.80E-05	0.00029691
IL21R	114	45	-0.925884786	0.0001132	0.000373525
HP	21	43	1.449099464	9.09E-05	0.000305661
PHOSPHO2	23	42	1.283907598	0.0004213	0.001247539
MPP7	23	41	1.24914218	0.000649	0.00185087
RLTPR	98	40	-0.877629617	0.0006234	0.001783187
ITGB1BP2	16	40	1.737080227	1.67E-05	6.45E-05
FAIM2	102	39	-0.971870991	0.0001432	0.000463539
GABRA4	134	38	-1.403009545	5.25E-09	3.14E-08
VWCE	95	38	-0.906775963	0.0005346	0.001551315

SNCA	93	36	-0.954081678	0.0003496	0.001052534
GALNT12	88	33	-0.999885368	0.000299	0.000912032
RAB26	98	32	-1.199557712	9.76E-06	3.90E-05
MAP2K6	86	30	-1.104222027	0.0001057	0.000351218
APCDD1	85	29	-1.136257809	8.00E-05	0.000272232
TMEM171	84	29	-1.119184296	0.0001072	0.000355899
COL14A1	75	26	-1.113226841	0.0002679	0.000823067
PPL	75	26	-1.113226841	0.0002679	0.000823067
LINC00161	143	25	-2.100863015	1.21E-15	1.27E-14
DACH1	109	25	-1.709176003	1.17E-09	7.50E-09
RNF208	77	24	-1.266671908	4.28E-05	0.000153611
PRSS3	74	24	-1.209338733	0.0001098	0.00036346
CMAHP	68	24	-1.087348209	0.000662	0.001885422
LOC100507032	76	23	-1.309213426	3.04E-05	0.000112256
PHOSPHO1	91	22	-1.63321089	7.36E-08	3.89E-07
SH2B2	66	22	-1.169810369	0.0003743	0.001121315
STAG3L3	63	21	-1.169810369	0.0005093	0.001482818
PINLYP	57	19	-1.169810369	0.0009459	0.002612123
TNFAIP6	63	17	-1.474664951	3.24E-05	0.000119158
TPTE2P5	55	17	-1.278744741	0.0004946	0.00144636
CACNA1G	54	16	-1.33973537	0.0003456	0.001042506
HRCT1	54	14	-1.532380448	7.54E-05	0.000257834
CTSL2	53	14	-1.505413401	0.0001085	0.000359537
RAB20	59	13	-1.767051199	4.62E-06	1.94E-05
ATG2B	4152	2878	-0.11358772	0.0010008	0.00274921
LRRFIP2	3829	3107	0.113706713	0.0010015	0.002750505
NDUFAF2	1515	1286	0.178724981	0.001003	0.002753917
ZCCHC9	1450	950	-0.19490135	0.0010033	0.002754199
IFIT1	441	257	-0.363858165	0.0010051	0.002758549
MBNL1-AS1	696	625	0.259921015	0.0010067	0.002762379
LDB3	82	100	0.701456317	0.001007	0.002762693
GEMIN6	400	230	-0.383214007	0.0010088	0.002766897

ZNF771	314	174	-0.436525121	0.0010091	0.00276701
TMEM147	1437	941	-0.195641302	0.0010132	0.002777746
MPHOSPH6	1754	1164	-0.176405558	0.001019	0.002793064
LIMK2	1573	1332	0.175227543	0.0010217	0.002799746
LIG1	1281	832	-0.20746291	0.0010231	0.002803195
MN1	227	231	0.440352686	0.0010273	0.002814061
LAMA4	7878	6243	0.079562181	0.0010278	0.002814773
MPV17	1894	1263	-0.16942956	0.0010279	0.002814226
PKNOX2	179	89	-0.592930215	0.0010314	0.002823254
ZNF654	1582	1339	0.174558493	0.0010347	0.002831725
ZNF337	1508	1280	0.178659513	0.001035	0.002831793
PRR24	421	244	-0.371786954	0.0010411	0.002848056
C19orf26	424	246	-0.370253817	0.001045	0.00285791
FLJ35946	1378	1176	0.186464304	0.0010466	0.00286189
MAPRE1	10710	7652	-0.06989757	0.00105	0.002870322
MGC16121	226	230	0.44046322	0.0010507	0.002871839
FAM83D	1183	764	-0.215653399	0.0010533	0.002878068
APEX2	698	430	-0.283738245	0.0010682	0.002918329
CCNB1IP1	931	816	0.224940116	0.0010688	0.002919273
EIF3D	8109	5752	-0.080308205	0.0010757	0.002937427
PSEN2	608	552	0.275749075	0.001076	0.002937766
COL5A3	840	742	0.236181991	0.0010776	0.002941511
ATOX1	2102	1411	-0.15989255	0.0010789	0.002944376
AACS	2116	1421	-0.159280941	0.001084	0.002957608
ZNF48	490	290	-0.341576717	0.0010858	0.002961771
ARL6IP5	10647	7607	-0.069895338	0.0010868	0.002963947
LINC00265	381	218	-0.390310731	0.0010968	0.002990607
RBM39	8227	6510	0.077443232	0.0010978	0.0029925
SRD5A1	1676	1413	0.168891448	0.0010979	0.002992313
NFYA	3402	2340	-0.124722479	0.0010986	0.002993492
USP45	1409	1200	0.183514926	0.0011131	0.003032378
FARP1	11544	8265	-0.066904349	0.001122	0.003055752

DYNLL2	3229	2636	0.122415061	0.0011234	0.003059101
SLC12A7	1064	682	-0.226502375	0.0011262	0.00306605
ASXL2	2729	2244	0.132852413	0.0011298	0.003075129
TMED9	11317	8099	-0.067523744	0.0011326	0.003082083
TRAP1	2874	1962	-0.135582888	0.0011336	0.003083963
TRIM39	324	314	0.369922878	0.0011345	0.003085862
SMARCAD1	3817	3095	0.112652355	0.0011383	0.003095664
MEG8	162	79	-0.620917123	0.0011393	0.0030977
CDC37L1	810	717	0.239203343	0.0011408	0.00310092
ABCD4	829	520	-0.257708347	0.0011408	0.003100313
SH3BGRL2	482	447	0.306393817	0.0011453	0.003111936
CCDC3	81	32	-0.924697871	0.0011517	0.003128682
CDIP1	1661	1100	-0.179396418	0.0011562	0.003140193
LIMD2	523	481	0.294378079	0.0011621	0.003155348
ZNF107	644	394	-0.293712927	0.0011636	0.003158689
PCNXL2	2054	1378	-0.160708162	0.0011639	0.003159032
IGF2BP3	14328	10309	-0.059780716	0.0011651	0.003161421
PPAP2A	420	244	-0.368356048	0.0011664	0.003164257
DCAF17	1832	1536	0.160910844	0.0011666	0.0031641
AMER1	423	246	-0.366847216	0.0011702	0.003173152
GORAB	776	689	0.243599462	0.0011734	0.003181261
TMEM45A	1087	941	0.207066819	0.0011774	0.003191424
LINC00472	66	84	0.763075435	0.0011788	0.003194636
WDR34	1154	745	-0.216178762	0.0011824	0.003203629
TRIM27	2866	1957	-0.135242722	0.0011845	0.003208459
SRP54	5920	4727	0.090479821	0.0011856	0.00321093
C21orf33	2114	1421	-0.15791669	0.0011995	0.003247697
AES	13132	9432	-0.0622989	0.0012024	0.003254845
PMM1	2187	1473	-0.155043658	0.0012116	0.003279156
RPL23AP82	571	345	-0.311742252	0.0012136	0.003283932
NXF1	3665	2531	-0.118953559	0.0012193	0.003298472
ZMYND8	3594	2480	-0.120098156	0.0012249	0.003312877

KAT2B	719	642	0.251733658	0.0012264	0.003316293
TMEM184B	5968	4197	-0.092736941	0.0012277	0.003319136
ABCF3	2640	1796	-0.140598448	0.0012277	0.003318464
LOC100506746	87	104	0.672648354	0.0012291	0.003321386
RHBDL1	123	56	-0.720007452	0.0012312	0.003326379
ARHGAP10	2483	1684	-0.145039991	0.0012323	0.003328528
NSMCE1	1916	1281	-0.165674954	0.0012347	0.003334466
ZDHHC24	630	385	-0.295341251	0.0012419	0.003353214
DGAT2	199	102	-0.549047147	0.0012437	0.003357128
HBS1L	4748	3313	-0.1040296	0.0012474	0.00336637
EIF3K	4445	3094	-0.107558091	0.0012506	0.003374406
ROCK2	9175	6535	-0.07436879	0.0012626	0.003406007
MCM9	391	370	0.335508795	0.0012656	0.003413349
DIP2B	3616	2936	0.114609422	0.0012678	0.00341862
MRPS6	1392	912	-0.19490135	0.0012679	0.003418198
SLC30A9	7803	6179	0.078496605	0.0012695	0.003421786
WWTR1	9772	7694	0.070232098	0.0012718	0.003427055
LIN7A	366	209	-0.393188574	0.0012752	0.003435483
KPNA1	6194	4937	0.087915394	0.0012771	0.003439928
SNRK	1077	932	0.206535742	0.0012785	0.003442988
GSDMB	284	279	0.389526324	0.0012831	0.003454663
AUTS2	267	145	-0.46563471	0.0012915	0.003476548
TMEM14A	961	612	-0.235852646	0.0012916	0.003476057
SSBP4	1376	901	-0.195729327	0.0012922	0.003476746
PRELID1	1443	948	-0.190960204	0.0012932	0.00347868
VPS8	3051	2091	-0.129936986	0.0012939	0.003479995
SLC16A5	138	65	-0.671004512	0.0012946	0.003481067
SLC25A37	807	506	-0.258279157	0.0012971	0.003487106
PAK4	1908	1276	-0.16528071	0.0012975	0.003487379
ACBD6	1632	1081	-0.179122402	0.001299	0.003490607
HEXIM2	143	68	-0.657256364	0.0013022	0.003498544
SH3GL1	5663	3977	-0.094733836	0.0013028	0.003499365

CASK	2493	1692	-0.144001183	0.0013036	0.003500616
ADIPOR2	3536	2873	0.11559185	0.0013036	0.003500037
BCL2L1	5299	3713	-0.09798268	0.0013038	0.003499644
ZC3H18	2724	1857	-0.137600756	0.0013042	0.003500175
CDK17	3604	2926	0.11448289	0.0013081	0.003509838
DLG3	1260	820	-0.204575787	0.001312	0.003519591
LCORL	632	570	0.266189493	0.0013142	0.003524593
CCDC124	1967	1318	-0.162494456	0.0013164	0.003529776
B9D1	389	368	0.335087743	0.0013221	0.003544189
TMEM70	1079	694	-0.221535165	0.0013244	0.003549593
HEATR6	1277	832	-0.20295096	0.0013267	0.003554989
SCN1B	512	306	-0.327460026	0.0013289	0.003560321
MAGI3	1535	1013	-0.18445235	0.001332	0.003567643
GNAT2	30	47	1.062850388	0.0013334	0.003570792
ATXN3	1076	692	-0.221682003	0.0013356	0.00357579
CHD4	16255	11731	-0.055405424	0.001336	0.003576251
CXXC1	1221	793	-0.207518298	0.0013409	0.003588584
NOC4L	666	410	-0.284746136	0.0013421	0.003590902
PDK4	114	51	-0.74531254	0.0013444	0.003596343
ATR	3204	2613	0.120985109	0.0013445	0.003595884
PRR11	4877	3408	-0.101916505	0.0013454	0.003597553
MRPL49	2203	1486	-0.152883247	0.0013558	0.003624606
PRDX2	6997	4948	-0.08473886	0.0013621	0.003640585
PDLIM2	2980	2041	-0.130884017	0.0013633	0.003642921
FAM63A	381	361	0.337359971	0.0013706	0.003661779
CD163L1	713	636	0.250276821	0.0013756	0.003674386
RQCD1	2801	1913	-0.134952978	0.0013795	0.003683911
NTNG1	110	125	0.599576703	0.0013806	0.003685937
TSLP	438	257	-0.354010379	0.0014001	0.003737373
CCDC22	571	346	-0.307566576	0.0014001	0.00373658
HECW1	313	175	-0.423655603	0.0014076	0.003755786
PUSL1	565	342	-0.309102411	0.0014089	0.003758474

SKP1	17145	13336	0.052690237	0.0014099	0.003760319
CTBS	1780	1492	0.160522426	0.0014101	0.003759949
LIN37	477	283	-0.338035081	0.0014119	0.003764043
PACRGL	454	422	0.309702833	0.0014119	0.003763273
RAD17	1991	1659	0.151972797	0.0014132	0.003765838
IFITM10	91	38	-0.844714995	0.0014164	0.003773422
LPIN1	4296	2989	-0.108178965	0.0014203	0.003783058
METTL4	554	505	0.281549543	0.0014221	0.00378709
ERV3-1	596	363	-0.300190651	0.0014246	0.003793027
CSK	2498	1697	-0.14263478	0.0014295	0.0038051
XPO5	2488	1690	-0.142811107	0.0014421	0.003838012
IFNE	288	159	-0.441889914	0.0014492	0.003856091
RNF130	1138	736	-0.213570755	0.001457	0.003875946
MYC	1337	1139	0.183920413	0.0014571	0.003875267
LOC344595	882	773	0.22484189	0.0014573	0.003875076
RPS15A	24791	18028	-0.04442498	0.0014574	0.003874386
TCP11L2	547	499	0.282651114	0.001458	0.003875194
OGFOD1	4435	3090	-0.106175135	0.0014588	0.003876441
LOC150776	668	412	-0.282051634	0.0014589	0.003876054
PSMF1	4659	3252	-0.103540941	0.0014592	0.003875963
SYNPO2	264	261	0.398664009	0.0014619	0.003882271
SGCB	9389	7395	0.070730771	0.0014667	0.003894282
LINC00476	219	222	0.434780938	0.0014693	0.003900153
PSMG1	1351	1150	0.182758318	0.0014785	0.003923955
ADH5	10427	7456	-0.068698236	0.001481	0.003929682
ORMDL1	1733	1454	0.161907747	0.0014825	0.003932734
ICAM3	463	274	-0.341684169	0.0014883	0.003947473
KIAA1407	263	260	0.398600955	0.0014954	0.003965344
RAP2A	2224	1842	0.143268405	0.0014956	0.003965039
MEGF9	2407	1986	0.137781613	0.0015032	0.003984281
FGFR1OP2	1838	1537	0.15713253	0.0015046	0.00398711
PSMB3	4250	2957	-0.108176466	0.0015072	0.003993182

XRCC5	24419	17755	-0.044626629	0.0015072	0.003992475
ISCA1	1527	1290	0.171823135	0.0015172	0.004018139
TSHZ1	1123	726	-0.214164343	0.0015216	0.004028917
RABL3	1512	1278	0.172581828	0.0015239	0.004033948
LOC550643	1500	990	-0.184309939	0.0015253	0.00403676
LOC253039	426	398	0.317067132	0.0015256	0.004036912
NAGS	96	41	-0.812258364	0.0015263	0.004037894
EFHD2	4143	2880	-0.109454877	0.0015274	0.004040005
ZDHHC7	7425	5263	-0.081353499	0.001529	0.004043154
ROBO4	103	45	-0.779495299	0.0015314	0.00404872
PCBP3	162	80	-0.602769776	0.0015323	0.004050197
ELK4	5871	4132	-0.091613871	0.0015361	0.004059477
ZADH2	1885	1262	-0.163700481	0.0015382	0.004064054
SLC43A2	968	842	0.213965318	0.0015384	0.004063757
CAPRIN1	26808	19521	-0.0424845	0.0015426	0.004074089
FAM188A	1970	1641	0.151531741	0.0015476	0.004086394
WDR3	2934	2399	0.124716418	0.0015514	0.004095455
POLD2	3478	2401	-0.119470397	0.0015554	0.004105296
SLC45A3	224	226	0.427976172	0.001563	0.004124522
SRGAP2C	1034	895	0.206875534	0.0015713	0.004145361
KCTD4	66	83	0.745797444	0.0015722	0.004146866
ERCC5	147	71	-0.634773094	0.0015727	0.004147421
ZBED6	2822	2311	0.126951404	0.0015737	0.004148988
RERE	6323	5031	0.085388102	0.0015803	0.004165738
FAM20B	5450	3827	-0.094890197	0.0015808	0.004166097
PARL	1763	1176	-0.168992283	0.0015815	0.004167164
CYSTM1	754	668	0.240435711	0.0016014	0.004218496
SAFB	3480	2403	-0.119098526	0.0016028	0.004221338
FANCM	781	490	-0.257388667	0.0016108	0.004241474
CELF1	6373	5069	0.084880635	0.0016168	0.004256542
DAG1	6947	5512	0.081337922	0.001619	0.004261253
ZNF518A	1649	1386	0.16448799	0.0016275	0.004282921

OCRL	4433	3091	-0.105057577	0.0016374	0.004307907
RBM14	1693	1127	-0.171942326	0.0016419	0.004318765
TMEM129	1355	889	-0.192885396	0.0016427	0.004319975
ATPIF1	3854	2673	-0.112744602	0.0016498	0.004337924
POLG	2335	1583	-0.145609163	0.001658	0.004358453
CACFD1	307	297	0.367376407	0.0016602	0.004363328
CCDC34	552	502	0.278171229	0.0016648	0.004374404
NUDT3	135	64	-0.661663465	0.0016734	0.004396195
ADIRF	59	21	-1.075173495	0.0016742	0.004397388
ZCCHC24	5973	4759	0.087354864	0.0016745	0.004397124
PLP2	4955	3971	0.095769435	0.0016747	0.004396823
AURKAIP1	3744	2594	-0.114249824	0.0016763	0.004399953
EIF4G3	14531	11331	0.056293331	0.0016778	0.004402962
CCDC104	1608	1353	0.166046565	0.0016789	0.004405086
SARM1	307	172	-0.420677959	0.0016796	0.004405848
UBR1	4879	3912	0.096473019	0.0016799	0.004405766
EIF1	19001	13763	-0.050128239	0.001683	0.004413031
KGFLP1	55	72	0.80371742	0.0016833	0.004412877
UBA7	722	450	-0.266921704	0.0016918	0.004434152
MTFP1	123	136	0.560100468	0.0017	0.004454762
AARS	6494	5161	0.083695378	0.0017036	0.004463278
POR	1664	1107	-0.17284808	0.0017055	0.004467299
SMARCD1	2641	1802	-0.136333159	0.0017056	0.004466738
CXorf40B	754	472	-0.260625532	0.0017117	0.004481757
SUSD5	878	768	0.222037503	0.0017204	0.004503361
YTHDC1	4300	3462	0.102421197	0.0017219	0.004506467
DDX47	3460	2390	-0.118609288	0.0017264	0.004517322
NLGN4Y	1980	1331	-0.157837727	0.0017295	0.004524346
SMG1	12639	9882	0.060144771	0.001731	0.004527539
ZNF596	426	397	0.313437709	0.0017391	0.004547664
CASP1	914	797	0.217537691	0.0017418	0.004553798
ANKRA2	1048	905	0.203503112	0.0017552	0.004587772

SLC35E2B	2780	1902	-0.132415505	0.0017579	0.004594047
DAB1	153	75	-0.61341702	0.0017609	0.004600911
PRRC2C	21831	16900	0.045797162	0.0017619	0.004602329
UFSP2	1241	810	-0.200357171	0.0017748	0.004635214
THADA	1826	1525	0.155274609	0.001778	0.004642493
RPUSD1	880	559	-0.239503109	0.0017785	0.004643007
AGPAT2	1503	994	-0.181375121	0.0017837	0.004655466
LSM5	1319	1122	0.181780243	0.0017862	0.004661093
GNA13	4948	3964	0.095263594	0.0017872	0.004662675
CNIH4	919	586	-0.234012065	0.0017891	0.004666769
FLCN	1466	968	-0.183654019	0.0017907	0.004669956
ACYP2	218	220	0.42832752	0.0017913	0.004670323
PIAS2	1545	1302	0.168274742	0.001795	0.004679171
CEP104	2609	1780	-0.136467571	0.0018015	0.004695032
ZBTB7A	3363	2321	-0.119850124	0.001802	0.004695292
PUS7	938	816	0.214133361	0.001806	0.00470473
ZFX	2481	2041	0.133506579	0.0018073	0.004707268
DDX52	2870	1967	-0.129901648	0.001811	0.004715851
CACUL1	4187	3373	0.103267532	0.0018148	0.004724735
KIAA0930	3703	2566	-0.114021247	0.0018152	0.004724736
DNLZ	416	244	-0.354550249	0.001822	0.00474144
CHMP1B	2114	1752	0.14417953	0.0018321	0.004766753
SRPR	8667	6175	-0.073941436	0.0018331	0.004768363
AMDHD2	540	327	-0.30851664	0.0018346	0.004771354
GLI2	2052	1383	-0.154077443	0.0018354	0.004772539
TRIM33	4024	3246	0.105184826	0.0018365	0.004774412
PTPRK	11987	8607	-0.062736133	0.0018372	0.004775055
CDC45	452	268	-0.33893764	0.001838	0.004776219
TRIM11	1090	705	-0.213480841	0.0018457	0.004795313
NDUFA10	3454	2387	-0.117917385	0.0018516	0.004809647
TMEM132A	3020	2075	-0.126285081	0.0018523	0.0048104
IGFBP7	10307	8092	0.06609588	0.001857	0.004821579

NPM1	54447	41659	0.028927781	0.0018591	0.00482592
SEMA5A	13769	10744	0.057259557	0.0018611	0.004830273
ZNF841	1479	1249	0.171303556	0.001862	0.004831533
ZNF223	246	244	0.403374964	0.0018672	0.004843945
GPCPD1	2483	2042	0.133050736	0.0018674	0.00484351
C1orf131	320	181	-0.406930076	0.0018701	0.004849362
RIC8B	1140	740	-0.208284517	0.0018715	0.004852169
MASTL	836	733	0.225462388	0.0018847	0.004885379
MECP2	3420	2775	0.113643578	0.0018858	0.004887102
RBL2	3046	2094	-0.125502368	0.0018877	0.004890979
SPSB1	857	750	0.222747523	0.0018883	0.004891579
ALB	29	45	1.049024233	0.0018947	0.004907042
CHMP5	4420	3553	0.100143451	0.0018966	0.004910992
TNRC6C	1325	1126	0.180366599	0.0018987	0.004915407
TMEM91	141	68	-0.636936379	0.001902	0.004922967
UBXN6	4684	3276	-0.100653587	0.0019107	0.004944566
WDR90	659	408	-0.276557181	0.0019184	0.004963259
B4GALT7	1442	952	-0.183885554	0.0019286	0.004988629
IDUA	468	279	-0.331091276	0.0019305	0.004992636
TMBIM1	7128	5053	-0.081205025	0.0019385	0.005012127
RNF145	6469	4573	-0.085249662	0.0019508	0.005042881
C2orf44	980	849	0.208134936	0.0019553	0.005053512
COPS7B	1463	967	-0.182189843	0.001964	0.005075014
CDC16	5703	4546	0.088028783	0.0019694	0.005087968
TRADD	644	398	-0.279140126	0.0019725	0.005094985
MSTO2P	66	25	-0.985385798	0.0019744	0.005098765
RPL39	15627	11285	-0.054482293	0.0019774	0.005105341
PGAM5	1066	689	-0.214479418	0.0019789	0.005108109
CCDC43	1856	1547	0.152428618	0.0019928	0.005142952
NES	28461	20757	-0.040236275	0.0019928	0.00514196
LRBA	3759	3038	0.107925086	0.0019946	0.005145488
MTUS1	347	199	-0.3870151	0.001996	0.00514803

PPP2R3C	1856	1245	-0.160898836	0.0019973	0.005149838
RBM18	1856	1245	-0.160898836	0.0019973	0.005149838
TBC1D17	1447	956	-0.182830267	0.0020074	0.005174416
SZT2	2545	1736	-0.136746577	0.0020098	0.005179454
ADAMTSL1	2407	1981	0.13414487	0.0020128	0.005185977
LZTS2	3253	2244	-0.120546015	0.0020216	0.005207536
ZNF432	709	629	0.242426521	0.0020257	0.005217084
GNA11	2175	1472	-0.148085598	0.0020278	0.005221523
COMTD1	134	64	-0.650937059	0.002028	0.005220789
GINS4	1370	902	-0.187824423	0.0020338	0.005234863
GLTSCR2	3686	2556	-0.113016103	0.0020365	0.005240638
ADK	2350	1936	0.135570328	0.0020373	0.005241563
SH2D4A	858	750	0.22106508	0.0020376	0.005241385
LOXL1-AS1	910	581	-0.23217625	0.0020394	0.005244744
POLE3	2256	1862	0.138238137	0.0020471	0.005263483
NAA10	3197	2204	-0.121442388	0.0020518	0.005274427
KAT2A	1570	1043	-0.17487327	0.0020565	0.005285451
GATA2	365	211	-0.375501333	0.0020574	0.005286761
C1orf43	6511	4605	-0.08452585	0.0020584	0.005288291
DYNC1LI1	4068	2832	-0.107346282	0.0020615	0.005295219
CASP9	442	409	0.303206605	0.0020687	0.005312464
IPMK	992	858	0.205789659	0.0020736	0.005323943
RELA	3394	2346	-0.11763142	0.00208	0.005339417
C1orf123	914	584	-0.231073664	0.0020901	0.005364201
KLF5	614	551	0.258965795	0.002091	0.005365461
VRK3	976	845	0.207222325	0.0020939	0.005371706
ZNF575	117	54	-0.700325086	0.0020975	0.005379881
GOT2	7151	5072	-0.080438126	0.0021009	0.005387318
KDM5C	5267	3701	-0.093914192	0.0021086	0.005406008
HDAC7	4308	3006	-0.104021109	0.0021095	0.005407231
HOPX	62	23	-1.015482223	0.0021096	0.005406504
EHBP1L1	5289	3717	-0.093704155	0.0021112	0.005409259

TMX3	10506	8240	0.064654885	0.0021204	0.005431783
NBPF16	395	231	-0.35880767	0.002124	0.00544006
GUCD1	3725	2585	-0.111924013	0.0021281	0.005449287
NDUFA8	1780	1192	-0.163340874	0.0021311	0.005455808
SLC50A1	709	443	-0.263326797	0.0021319	0.00545673
RRP1	1016	655	-0.218181459	0.0021397	0.005475629
DYRK3	1026	885	0.201870761	0.0021399	0.005475131
MAPRE3	1401	1185	0.173582235	0.0021425	0.005480492
AAED1	596	536	0.262072802	0.0021437	0.005482382
TBC1D10A	687	428	-0.267547171	0.0021449	0.005484414
ST3GAL3	834	529	-0.24162753	0.002155	0.00550903
DNAJC22	243	132	-0.465266252	0.0021579	0.005515408
ADAMTS6	440	407	0.302677402	0.0021581	0.00551475
TROAP	559	341	-0.297924412	0.0021629	0.005525887
WIPF2	3493	2829	0.110977666	0.0021727	0.005549705
CKLF	60	22	-1.032306845	0.002173	0.005549419
ZNF28	678	603	0.246024861	0.0021773	0.005559235
COLEC10	510	308	-0.312414764	0.0021827	0.005571902
A4GALT	507	306	-0.313301963	0.0021887	0.005586061
NHLRC3	451	416	0.298608227	0.0022004	0.00561474
DYNC1H1	71499	54551	0.024834711	0.0022148	0.005650494
ZNF74	625	386	-0.280103211	0.0022256	0.005676718
PPP1R10	3345	2312	-0.117712681	0.0022258	0.005676141
TMBIM4	2806	2292	0.123244167	0.0022299	0.005685457
HSD3BP4	38	54	0.92211212	0.0022313	0.005687788
KPNA5	396	370	0.317176972	0.0022339	0.005693377
HIST1H2AC	577	520	0.265092436	0.0022383	0.005703309
LPCAT4	1044	675	-0.214010173	0.0022517	0.005736391
LRPAP1	6494	4595	-0.083890391	0.0022518	0.005735355
HBE1	297	167	-0.415462697	0.0022537	0.005739193
FAM195A	402	236	-0.35325651	0.0022551	0.005741405
GHDC	320	182	-0.398981323	0.0022613	0.005756011

PSMD9	2195	1488	-0.145694282	0.0022771	0.005795181
UCHL3	1503	997	-0.177027468	0.0022772	0.005794072
SDHC	5033	3533	-0.095372717	0.0022784	0.005795959
PDCD6	2718	1862	-0.130540251	0.002285	0.005811715
AATF	2405	1638	-0.138949405	0.0022898	0.005822648
ARID5A	237	235	0.402925829	0.0022921	0.005827369
ORC1	361	209	-0.373343763	0.0023005	0.005847554
DNMT3B	207	209	0.429024306	0.0023011	0.005847937
PATL1	2424	1992	0.13198008	0.0023032	0.00585185
NDUFB8	3555	2464	-0.113695172	0.0023084	0.005864063
DERL2	1959	1626	0.146361992	0.0023166	0.005883543
C16orf58	1998	1348	-0.152583955	0.002324	0.005901246
AHCTF1P1	49	65	0.822810101	0.0023246	0.005901564
CALCRL	46	62	0.845786486	0.0023262	0.005904334
CPEB3	94	108	0.615450782	0.002338	0.00593301
SCYL2	5661	4509	0.086902718	0.0023396	0.005935962
ZNF555	375	352	0.323836965	0.0023576	0.005980497
RASSF7	312	177	-0.402644537	0.0023585	0.00598161
GPR126	1306	1108	0.177955116	0.0023595	0.005982867
MOB3C	524	318	-0.305387915	0.0023674	0.006001662
SNRPG	2365	1610	-0.139627363	0.0023756	0.006021253
PSMC5	4749	3328	-0.097816191	0.0023766	0.006022502
CLTA	6942	4923	-0.080661494	0.0023768	0.006021699
MARCH2	731	459	-0.256225121	0.0023804	0.006029795
ANKRD36	824	523	-0.240681259	0.0023807	0.006029177
TMEM50B	1890	1571	0.148449078	0.0023977	0.006071071
NACC1	7954	5662	-0.075216681	0.0023982	0.006071156
RPS6KA3	4936	3464	-0.095751333	0.0024168	0.00611688
RSPH3	386	361	0.318550121	0.002428	0.006144041
ZWILCH	2202	1494	-0.144482189	0.002433	0.006155308
IPO4	2835	1947	-0.126943719	0.0024337	0.006155862
LINS	361	340	0.328688041	0.0024376	0.006164637

ZNF480	1357	895	-0.185309002	0.0024393	0.006167587
THSD7A	78	32	-0.870250087	0.002442	0.006173066
TBC1D9B	7463	5304	-0.077522779	0.0024485	0.006188355
PIANP	732	460	-0.255057656	0.0024701	0.006241758
C14orf93	209	111	-0.497791134	0.0024726	0.006246714
FMR1	1987	1647	0.144400814	0.0024791	0.006261849
COPS7A	3643	2529	-0.111407826	0.0024945	0.006299448
ZC3H6	981	847	0.203260965	0.0024994	0.00631066
TBRG4	1582	1054	-0.170722601	0.0025073	0.006329355
TRPT1	434	258	-0.335171845	0.0025225	0.00636638
VSIG10L	111	51	-0.706838393	0.0025227	0.006365492
C7orf50	2481	1694	-0.135335729	0.0025364	0.006398801
CCNG2	2374	1951	0.132045975	0.0025444	0.006417691
FHL3	1121	730	-0.203665777	0.0025453	0.006418693
USP25	3028	2463	0.117203554	0.002548	0.006424065
BDNF	7815	6167	0.073475105	0.0025587	0.006449924
BCL9	1491	990	-0.175627696	0.0025616	0.00645571
MAGED2	12278	8833	-0.059948027	0.0025758	0.006490256
POLI	1201	1023	0.183722126	0.0025825	0.006505831
PDE9A	299	169	-0.407970106	0.0025826	0.006504912
MIR155HG	141	69	-0.615874764	0.0025832	0.006504932
TPRN	676	422	-0.264628116	0.0025865	0.00651203
ATP13A3	26262	20252	0.040239731	0.0026005	0.006545865
CDK9	1642	1097	-0.16673847	0.0026172	0.00658656
REV3L	5096	3582	-0.093447809	0.0026177	0.006586497
ARG2	326	310	0.342548383	0.0026213	0.006594278
LOC100190986	1726	1440	0.153788479	0.0026264	0.006605874
CDK15	1109	722	-0.204036492	0.0026386	0.006635154
FBXO10	635	566	0.249197593	0.0026467	0.006654254
ARHGAP28	308	175	-0.400423297	0.0026483	0.006656951
CANX	60623	46306	0.026486024	0.0026526	0.006666338
GPRC5B	573	352	-0.287807578	0.0026567	0.006675331

SNRPE	2914	2373	0.118863355	0.0026732	0.006715228
GGA3	1691	1132	-0.16385057	0.0026765	0.006722342
GNPDA2	1258	1068	0.178931857	0.0026885	0.006750954
ZNF256	230	228	0.402552095	0.0026895	0.006752269
EGLN1	2849	2322	0.120064482	0.0027001	0.006777455
MRPL34	922	592	-0.224017443	0.0027012	0.006778743
NF1	13267	10345	0.056243599	0.0027037	0.006783827
TMEM199	763	670	0.22763017	0.0027113	0.006801474
APOPT1	1565	1043	-0.170271368	0.0027135	0.006805592
ZNF527	285	275	0.363621831	0.0027228	0.006827667
C6orf226	170	87	-0.551295309	0.0027426	0.00687571
CDKN2C	700	439	-0.257981851	0.0027671	0.006935856
SEC61G	5302	3733	-0.09104903	0.0027709	0.006943989
ATXN7L3B	3996	3215	0.101414286	0.0027774	0.006958787
GLIPR2	1315	867	-0.185806769	0.0027783	0.006959808
LIG4	1910	1288	-0.153287913	0.0027815	0.006966269
GABARAP	8324	5937	-0.07239072	0.0027853	0.006974393
SPRYD7	1597	1337	0.158787284	0.0027859	0.006974617
LMNB1	2142	1767	0.137495692	0.0028092	0.007031473
SPAG9	8645	6802	0.069245174	0.0028114	0.007035593
CHST14	1383	915	-0.180805376	0.0028114	0.007034184
IFI27	30	45	1.000114632	0.0028206	0.007055642
NEURL2	72	29	-0.896791875	0.0028211	0.007055636
CLCN3	6605	4682	-0.081281495	0.002823	0.007058877
CNOT11	2384	1957	0.130411652	0.0028258	0.007064422
UBE2C	1075	699	-0.205820167	0.0028313	0.007076944
NOSIP	1526	1016	-0.171702428	0.0028357	0.0070865
CSNK1G2	2625	1799	-0.129970104	0.0028358	0.007085132
KRBA1	316	301	0.34499106	0.0028371	0.007087141
TACC2	1155	985	0.18545491	0.0028451	0.00710558
LSM11	189	99	-0.517733672	0.0028526	0.00712281
CC2D1A	1404	930	-0.179088183	0.0028587	0.007136782

ULBP2	322	306	0.341623096	0.0028646	0.007150089
HSPA1B	712	628	0.23403945	0.0028673	0.007155203
ZNF44	508	461	0.275090385	0.0028694	0.007159116
GPER	208	111	-0.49087172	0.0028728	0.00716629
C16orf87	1590	1331	0.158635937	0.0028737	0.00716691
ZNF385A	372	348	0.318936816	0.0028779	0.007176012
SREBF2	2733	2230	0.121710382	0.0028883	0.007200504
INPP5E	898	576	-0.225494502	0.0028891	0.007200952
GEMIN7	622	386	-0.273161601	0.0028921	0.007207207
CTDNEP1	4094	2858	-0.103353054	0.0029002	0.007225756
ALG13	1714	1429	0.152790939	0.0029019	0.007228615
YWHAQ	32941	24095	-0.035998419	0.0029037	0.007231656
FTX	142	70	-0.605311971	0.0029045	0.007232149
PAPOLA	12323	9620	0.057907412	0.0029087	0.007241322
TMEM167A	10256	8037	0.063412952	0.00291	0.007243112
TRAPPC3	2359	1609	-0.136858961	0.002919	0.007263859
FLAD1	1096	714	-0.203099687	0.0029198	0.007264524
CD320	1166	763	-0.196660695	0.0029239	0.007273274
IL17D	233	127	-0.460349326	0.0029337	0.007296176
RRAD	60	75	0.737080227	0.0029376	0.007304505
STX10	1260	829	-0.188827595	0.0029387	0.007305728
HSD11B1	100	45	-0.736850962	0.0029465	0.007323634
S100A4	422	251	-0.334403503	0.0029573	0.007349023
PXDC1	1612	1348	0.157120884	0.0029582	0.007349859
TOP1	15168	10964	-0.053104504	0.0029639	0.007362427
PLA2R1	59	74	0.741962448	0.002965	0.007363603
MAML1	2133	1759	0.137023649	0.0029672	0.007367702
GABBR1	1775	1193	-0.15807285	0.0029702	0.007373636
EFHA2	2245	1847	0.133620553	0.0029715	0.007375458
GRSF1	8443	6645	0.069665617	0.0029716	0.007374111
RNF149	1325	1120	0.172658504	0.0029882	0.007413934
CEP95	623	555	0.24840774	0.0030008	0.007443707

GFPT2	1167	764	-0.196007886	0.0030037	0.007449511
AKR1C2	93	41	-0.766454675	0.0030048	0.007450639
VIMP	2286	1879	0.132291795	0.0030054	0.007450745
C17orf85	3019	2083	-0.120255786	0.0030079	0.007455462
PAQR8	623	387	-0.271746465	0.0030106	0.007460667
BZW2	3219	2227	-0.116358887	0.0030135	0.007466314
DUSP1	4050	2827	-0.103497893	0.0030155	0.007469747
SCRN3	1166	993	0.183449966	0.0030295	0.00750291
PRKAR2B	1420	942	-0.176939833	0.0030307	0.007504542
CCDC136	741	468	-0.247812881	0.0030492	0.007548663
OAF	3177	2197	-0.116978089	0.0030535	0.007557778
ZNF184	508	309	-0.302069527	0.0030545	0.007558779
DPH2	887	769	0.209201625	0.0030567	0.007562771
FAT4	7561	5966	0.073339055	0.0030633	0.007577623
CDH24	1687	1131	-0.161708913	0.0030666	0.007584319
PLK2	4618	3239	-0.096567573	0.0030883	0.007636442
AP5M1	1904	1578	0.144215858	0.0030952	0.007652083
NFAT5	17332	13443	0.048569122	0.0031085	0.007683298
BRMS1L	787	688	0.221197061	0.0031152	0.007698408
SENP1	1263	1070	0.175908289	0.0031279	0.00772835
IFIT5	1149	979	0.184154099	0.0031298	0.007731338
PCSK7	6205	4921	0.080672438	0.0031305	0.007730927
PHF20L1	6205	4921	0.080672438	0.0031305	0.007730927
PRUNE	475	433	0.281591643	0.0031329	0.007734397
FAM103A1	434	399	0.293845836	0.0031453	0.007763621
NFATC2	161	167	0.467939546	0.0031565	0.00778967
SNRNP48	1540	1290	0.159592846	0.0031585	0.007792957
IQCH	40	55	0.87458375	0.0031683	0.007815569
ZER1	3076	2125	-0.11844053	0.0031723	0.007824003
SNCAIP	298	285	0.35080172	0.0031738	0.007826039
GET4	1385	918	-0.178167786	0.0032032	0.007897127
EAPP	1192	1013	0.18040207	0.0032166	0.007928463

ZC3H12C	1442	1212	0.164470666	0.003217	0.007928011
VEGFB	2001	1355	-0.147276184	0.0032365	0.007974316
SNX16	502	455	0.273331313	0.0032412	0.007984399
LYNX1	714	450	-0.250846941	0.0032576	0.008023331
BPNT1	1363	1149	0.168745368	0.0032717	0.008056431
PRDM11	73	87	0.668271069	0.0032917	0.008103986
ALKBH8	867	752	0.2098528	0.0033051	0.008135274
EFCAB7	221	219	0.402036632	0.0033052	0.008134053
LIG3	1258	829	-0.186535784	0.003316	0.008159066
IL11RA	516	315	-0.296867105	0.0033176	0.008161342
RRP15	1941	1606	0.141823907	0.0033251	0.008178108
ST3GAL5	834	533	-0.230759719	0.0033297	0.008187961
SLC17A5	3713	2991	0.103194727	0.0033354	0.008200145
SH3D19	4160	3338	0.097552558	0.0033468	0.008226658
ZNF568	334	315	0.330655858	0.0033475	0.008226803
HMGCS1	1547	1033	-0.167480811	0.0033492	0.00822851
TMEM203	1547	1033	-0.167480811	0.0033492	0.00822851
ACLY	24150	17598	-0.04145958	0.0033527	0.008234537
SNAPC2	929	599	-0.217970462	0.003357	0.008243621
CNTLN	1178	773	-0.192647088	0.003369	0.00827137
CEPT1	1262	832	-0.185904345	0.0033696	0.008271194
CKAP5	12501	9750	0.05658275	0.0033963	0.008335278
SEC11C	927	800	0.202582793	0.003402	0.008347501
MESDC1	803	700	0.217107066	0.0034033	0.008349149
COL4A5	7187	5112	-0.076349718	0.003404	0.008349153
SERGEF	309	177	-0.388705346	0.0034053	0.008350651
ABCB1	205	205	0.415152132	0.0034067	0.00835252
PDGFC	4299	3010	-0.099085492	0.0034122	0.008364274
MLPH	1239	816	-0.187382998	0.0034185	0.008378194
GYPC	1462	1227	0.162344069	0.0034211	0.008382811
LONRF1	486	295	-0.305089228	0.003447	0.008444509
ABCD1	1009	655	-0.208207231	0.0034477	0.008444745

TREX1	183	96	-0.515585206	0.0034516	0.008452452
PIM1	345	324	0.324549583	0.0034596	0.008470546
PKN3	224	122	-0.461465453	0.0034657	0.008483865
CEP44	941	811	0.200659323	0.0034789	0.008514463
ZNF625	204	204	0.415152132	0.0034859	0.008529888
ERCC4	1638	1098	-0.161905171	0.003488	0.00853318
SMG6	4621	3244	-0.095279138	0.0034899	0.008536161
ZNF621	2643	2156	0.121332885	0.0034932	0.008542658
XRN2	7820	6161	0.07114806	0.0034943	0.008543745
RBM24	1047	896	0.190461327	0.0034971	0.008548804
NXNL2	62	24	-0.954081678	0.0035076	0.008572753
NOC3L	2236	1524	-0.137905154	0.00351	0.008577115
CAPN5	3065	2119	-0.117351354	0.0035148	0.008587043
GNB3	298	170	-0.394625453	0.0035367	0.008638894
FLJ43663	275	155	-0.412011271	0.0035387	0.008642089
TTLL4	1021	875	0.192524188	0.0035446	0.008654878
MICAL3	2966	2408	0.114468926	0.0035516	0.008670088
FBXO18	4375	3066	-0.097773188	0.003554	0.008674415
PAFAH1B3	453	273	-0.315457967	0.003563	0.008694554
ELMO2	2678	1841	-0.125514202	0.0035698	0.00870941
NTM	4263	2985	-0.098985992	0.0035823	0.008738375
FRMD3	444	267	-0.318567803	0.0035884	0.008751518
PGAP2	552	340	-0.283981389	0.0035908	0.008755559
PVRL1	618	385	-0.267596261	0.0035948	0.008763577
BNIP2	4246	3403	0.095854912	0.0035949	0.008762078
MAFK	1684	1402	0.150746343	0.0036	0.008772914
MAP1A	51247	37689	-0.028171903	0.0036018	0.008775545
TECPR2	2679	1842	-0.125269388	0.0036276	0.008836556
PLCE1	485	440	0.274670908	0.0036321	0.008845801
KIAA1841	853	547	-0.225852777	0.003636	0.008853671
ALAS1	1561	1044	-0.165196694	0.0036383	0.008857514
MIR17HG	157	80	-0.557540522	0.003639	0.008857508

NUTF2	3336	2315	-0.111954964	0.0036408	0.008860155
ARHGAP17	4468	3134	-0.096471874	0.0036431	0.008863945
KHDC1	99	45	-0.722351392	0.003644	0.008864384
RORA	513	463	0.267205499	0.0036587	0.008898447
ACAA1	1931	1307	-0.147936891	0.0036723	0.00892979
PLEKHJ1	1418	943	-0.173375725	0.0036768	0.008939084
MORN4	563	504	0.255440943	0.0036779	0.008939842
MAN2C1	2165	1474	-0.139478369	0.0036797	0.008942558
PIGW	606	377	-0.269601138	0.0036857	0.008955519
FAM192A	2588	1777	-0.127241804	0.0036955	0.008977557
TJAP1	1028	669	-0.204610017	0.0036969	0.008979165
TM4SF18	26	40	1.036640508	0.0037285	0.009054149
CWC25	1048	896	0.189084052	0.0037333	0.009063898
ZNF619	178	181	0.439264588	0.0037393	0.009076673
NHLRC2	1993	1645	0.138298006	0.0037399	0.009076562
RNF103	1305	1101	0.169916794	0.003752	0.009104102
POLD3	813	707	0.213606994	0.0037596	0.00912074
APOA1BP	1226	808	-0.186379649	0.003769	0.009141845
FAM65A	4442	3116	-0.096362028	0.0037819	0.009171312
PCDHB13	277	266	0.356692401	0.0037886	0.009185656
IFIT3	744	472	-0.24136363	0.0037952	0.009199886
ERAP1	2327	1907	0.127985765	0.0038028	0.009216597
WBP1	69	28	-0.886017403	0.003804	0.009217655
LRFN3	544	335	-0.284293424	0.0038052	0.009218679
AMBRA1	1498	1000	-0.167885492	0.0038078	0.009223282
SPG21	2714	1868	-0.123774134	0.0038086	0.009223343
FANK1	62	76	0.708883335	0.0038095	0.009223752
ZNF711	222	219	0.395523325	0.0038098	0.009222735
SLC29A4	770	490	-0.236924565	0.0038169	0.0092381
NAT9	1372	911	-0.175605391	0.0038188	0.009240878
ARMCX6	1505	1005	-0.167415854	0.0038215	0.009245588
TBCK	1333	1123	0.167833279	0.0038248	0.009251968

C17orf70	1257	830	-0.183649276	0.0038321	0.009267634
GRIK2	1864	1543	0.142488334	0.0038408	0.009286858
HCG11	544	488	0.258426628	0.0038527	0.009313833
SLC22A18	403	240	-0.332593301	0.0038568	0.009321961
COPS5	2981	2418	0.113170001	0.0038645	0.009338901
PPP1R7	3625	2525	-0.106545476	0.0038856	0.009387933
USP51	104	115	0.560202465	0.0038991	0.009418673
COBLL1	521	469	0.263456682	0.0039043	0.009429591
C1orf233	554	342	-0.280737519	0.0039066	0.009433282
PELI1	269	259	0.360498057	0.0039082	0.009435303
ADPGK	3486	2811	0.104663016	0.0039133	0.009445726
SFT2D3	388	359	0.303079323	0.0039187	0.009457065
GRIK4	220	120	-0.459316986	0.0039208	0.009460302
ATP6V1E1	6251	4950	0.078493654	0.003922	0.009461189
IGF2BP2	7314	5209	-0.074502058	0.0039281	0.009474255
EML1	4076	3269	0.096847457	0.0039412	0.009503928
ACN9	268	151	-0.412532319	0.0039438	0.009508416
DEPDC7	443	267	-0.315314825	0.0039657	0.009559182
PBX3	1004	860	0.191801427	0.0039707	0.009569483
ELN	58	72	0.727096138	0.0039818	0.009594269
FAM172A	2337	1914	0.127085227	0.003984	0.009597783
LOC644961	91	103	0.593858019	0.0039926	0.009616708
STX16	4532	3622	0.091790817	0.0039946	0.00961969
PSMA6	6523	5159	0.07670795	0.0039952	0.009619162
NOLC1	4483	3584	0.09225827	0.0040047	0.009640152
PABPC1L	723	458	-0.243495917	0.0040058	0.009641082
ZKSCAN5	1449	966	-0.169810369	0.004007	0.009642064
ATP5O	5002	3524	-0.090139002	0.0040073	0.009640823
CCDC107	83	36	-0.789962298	0.0040095	0.00964434
SLC4A3	294	280	0.344762804	0.004014	0.00965334
GCDH	659	414	-0.255495566	0.0040175	0.009659742
SMOX	778	496	-0.234277903	0.0040181	0.009659433

NDEL1	3153	2185	-0.113939759	0.004028	0.009681435
SPRYD3	2090	1720	0.134057754	0.0040291	0.009682028
ATCAY	107	50	-0.682458665	0.0040337	0.009691297
ARL6IP4	3910	2732	-0.102058992	0.004045	0.009716568
KIAA1524	1752	1181	-0.153841678	0.0040454	0.009715642
GRWD1	1614	1083	-0.160455204	0.0040483	0.009720649
LMTK2	1295	1092	0.16917289	0.0040511	0.009725619
FAM96B	1310	868	-0.178647732	0.0040519	0.009725622
TMEM205	1018	663	-0.203504654	0.0040567	0.009735326
ANKRD32	645	570	0.23681489	0.0040672	0.009758574
SAP30BP	2315	1583	-0.133198806	0.0040888	0.009808466
SLC38A7	1309	1103	0.168119825	0.00409	0.009809471
ZNF766	860	553	-0.221905048	0.0040923	0.009813157
YAE1D1	527	324	-0.286657017	0.0040943	0.009815979
CXorf57	55	69	0.742316875	0.0040995	0.009826601
KIAA0100	13147	9490	-0.055101506	0.0041124	0.009855722
PTPRJ	1679	1396	0.148848843	0.0041128	0.009854801
MUL1	1440	960	-0.169810369	0.0041222	0.009875281
SNX9	5364	3788	-0.086720775	0.0041229	0.009875108
GTDC1	655	578	0.234726718	0.0041239	0.009875729
TTC3	35600	26088	-0.033338763	0.004127	0.009881115
KDELR1	15393	11144	-0.050855137	0.0041341	0.00989614
INTS8	2587	2109	0.120431173	0.0041359	0.009898747
RSRC2	3201	2588	0.108467072	0.0041441	0.009916292
STK38L	766	488	-0.235311113	0.0041499	0.00992825
SUOX	818	524	-0.2273819	0.0041522	0.009932003
PTGFR	1736	1170	-0.154106286	0.0041539	0.009934028
ZFAS1	2235	1526	-0.135367737	0.0041564	0.009938124
HADHB	6195	4905	0.078300986	0.0041796	0.00999181
HCN2	802	513	-0.229491279	0.0041903	0.010015341
PRDM8	1778	1200	-0.152068787	0.0041917	0.01001691
ZNF100	330	310	0.324954323	0.0041946	0.010021812

DMAP1	943	611	-0.210933259	0.0041951	0.010021094
TRIM62	953	618	-0.209717244	0.0042064	0.010046044
CDKN3	747	475	-0.238028598	0.0042083	0.010048848
DEAF1	1026	669	-0.201800483	0.0042141	0.010060712
ZNF551	259	250	0.364128129	0.0042145	0.010059668
MRPL33	2249	1844	0.128707126	0.0042158	0.010060918
SETD3	2873	2332	0.114161927	0.0042158	0.010059018
CIAPIN1	1734	1439	0.146114825	0.004222	0.01007185
NCKIPSD	1063	695	-0.197904582	0.0042563	0.010151781
KAT6B	3719	2595	-0.104028078	0.0042814	0.01020953
ZNF717	195	195	0.415152132	0.0042886	0.01022477
MRGBP	1090	714	-0.195180024	0.004289	0.010223797
SNAI3-AS1	156	80	-0.548321992	0.0043006	0.010249614
IMMP1L	35	49	0.900578959	0.0043008	0.010248042
ITGA10	419	384	0.289308199	0.0043087	0.010264973
ZCCHC5	48	62	0.784385941	0.0043142	0.010275915
DPAGT1	1432	955	-0.169306723	0.0043385	0.010331838
LOC100505876	2070	1703	0.133599799	0.0043434	0.010341663
PDK2	1171	771	-0.187786179	0.004347	0.010348273
GABBR2	1898	1286	-0.146437218	0.0043495	0.010352209
TYMS	448	271	-0.310053749	0.0043534	0.010359413
NOL3	445	269	-0.311047032	0.0043664	0.010388418
MAN2B1	3432	2388	-0.108094584	0.0043792	0.01041699
CNPY3	1429	953	-0.169305665	0.0043797	0.010416059
RPL21P44	41	55	0.838959841	0.004396	0.010452894
MGC72080	319	185	-0.370879021	0.0044009	0.01046253
THBS2	2919	2367	0.112737627	0.004404	0.010467891
SPATA5	651	574	0.233545325	0.0044443	0.010561654
C9orf3	376	348	0.303506776	0.0044567	0.010589087
SWT1	276	264	0.351021794	0.0044933	0.010674006
EXOSC6	827	531	-0.224023337	0.0044949	0.010675721
CAMKK1	461	280	-0.304187792	0.004503	0.010693023

CHAF1A	1213	801	-0.183553271	0.0045232	0.010738859
PHF8	1452	1215	0.158066992	0.0045377	0.010771361
SYNRG	4998	3979	0.086207129	0.0045634	0.010830246
KIAA0195	1904	1291	-0.145392346	0.0045643	0.01083043
RHOC	16007	11601	-0.049301644	0.0045707	0.010843349
CSDE1	50778	38808	0.027302644	0.004576	0.010853926
EPB41L3	743	473	-0.236369895	0.0045774	0.010855269
MTHFD1L	3880	2713	-0.101015473	0.0045852	0.010871691
SNHG11	222	122	-0.448526397	0.0045859	0.010871234
LIPE	154	79	-0.547853661	0.0045873	0.010872487
C21orf58	276	157	-0.398751576	0.0045917	0.010880736
POLR3H	1589	1067	-0.159406817	0.0045983	0.01089428
ZNF736	161	165	0.450557468	0.0045989	0.010893752
PRKCD	1278	847	-0.17830183	0.0046049	0.01090591
CHP1	8702	6829	0.065479449	0.0046169	0.010932226
MRPL24	2104	1434	-0.137937549	0.0046214	0.010940697
MBD2	2860	2320	0.11326179	0.0046242	0.01094535
ZNF439	723	632	0.221081043	0.0046281	0.010952412
GLCE	3986	2790	-0.099524456	0.0046316	0.010958606
F2RL1	1452	970	-0.166832669	0.0046325	0.01095878
HIST2H2BE	954	818	0.193263709	0.0046554	0.01101074
GCLM	3325	2682	0.105107028	0.0046574	0.011013358
BCS1L	763	487	-0.232609153	0.0046636	0.011026055
GSE1	3192	2216	-0.111350638	0.0046664	0.011030516
TRMT10A	444	404	0.278947748	0.0046805	0.011061679
HELLS	917	594	-0.211306671	0.0046814	0.011061884
NCAM2	616	545	0.238478011	0.0046824	0.011062101
NHP2L1	3813	2665	-0.101638866	0.0046947	0.011089048
HSPBP1	1060	694	-0.195904565	0.0047044	0.011109825
ETFB	2774	1915	-0.119471264	0.004716	0.01113517
DNAJA3	2433	1670	-0.127736086	0.0047213	0.011145602
FAM50B	192	103	-0.483309842	0.004748	0.011205307

SEMA6C	192	103	-0.483309842	0.004748	0.011205307
PITPNA	4504	3596	0.090338325	0.0047494	0.011205429
ENTHD2	638	401	-0.254802056	0.0047542	0.011214607
TRANK1	1114	732	-0.190681547	0.0047575	0.011220329
DCLRE1A	483	436	0.267457078	0.0047621	0.011228996
SLC1A7	59	23	-0.943928962	0.004765	0.011233769
MFAP4	1310	870	-0.175327374	0.0047681	0.01123889
YKT6	8818	6316	-0.066288174	0.0047803	0.011265648
EIF3H	8923	6393	-0.065883614	0.0047859	0.011276607
SPSB3	712	452	-0.240402337	0.0047881	0.011279606
ZNF75A	794	689	0.210517107	0.0047924	0.011287779
TMEM201	898	581	-0.21302515	0.0047928	0.011286425
RNF34	1757	1187	-0.150642125	0.0047965	0.011293062
IRF9	937	804	0.194298585	0.0048	0.011299236
KIAA0947	4430	3113	-0.09384899	0.004837	0.011384058
USP40	3184	2211	-0.110989179	0.0048526	0.011418624
SLC28A3	124	132	0.505349941	0.0048616	0.011437787
MAN1C1	951	815	0.19250685	0.0048918	0.011506588
FAM46A	5424	4306	0.082143273	0.0049008	0.011525486
SFR1	174	176	0.431640255	0.0049063	0.011536314
ENG	17769	12904	-0.046392017	0.0049086	0.0115396
IL16	168	88	-0.517733672	0.0049145	0.01155132
LOC645166	985	642	-0.202398295	0.0049246	0.011572746
SPC24	209	114	-0.459316986	0.0049323	0.011588632
HCFC2	2685	2182	0.115881145	0.0049722	0.011680372
ZMAT2	3025	2447	0.109231091	0.0049998	0.011742812
GAK	3695	2581	-0.102492091	0.0050142	0.011774401
SERPINH1	28367	21812	0.036060608	0.005067	0.011896209
ZNF226	1029	877	0.184557897	0.0050712	0.011903945
PRPF4B	7555	5393	-0.07119159	0.0050727	0.011905194
ALDH1A3	163	85	-0.524185086	0.0050818	0.011924282
TIGD5	298	172	-0.377751634	0.0051066	0.011980132

SYNE2	887	574	-0.212731236	0.0051168	0.012001818
NAGA	2226	1523	-0.132385519	0.0051309	0.012032618
GTF2A2	2504	2040	0.119486722	0.0051318	0.012032493
EDC3	1832	1513	0.139144616	0.0051555	0.012085831
HEG1	10750	8394	0.05824584	0.0051565	0.012085868
BROX	2005	1649	0.133141294	0.0051566	0.012083744
FBXO32	351	326	0.308553066	0.0051764	0.012127972
LOC100131564	490	441	0.263149038	0.0051867	0.012149854
ADAT2	415	250	-0.31603111	0.0052006	0.012180084
CHST11	398	365	0.290280165	0.0052385	0.01226655
EPHA2	4404	3096	-0.093256866	0.0052387	0.012264765
PDXDC1	5302	4210	0.082435695	0.0052431	0.012272703
NR2C2	3684	2574	-0.102108876	0.005244	0.012272429
SLC9A5	452	275	-0.301739022	0.0052502	0.012284687
MSL2	1224	1032	0.168991544	0.0052518	0.012286088
ZNF286A	974	635	-0.202013049	0.0052538	0.012288495
PDSS1	222	217	0.382287498	0.0052666	0.012316007
NFKB1	1561	1299	0.150083025	0.0052669	0.012314365
TOR3A	1748	1182	-0.149323018	0.0052844	0.012353166
USE1	331	194	-0.355622433	0.0052871	0.012357154
DSE	4813	3832	0.08631327	0.0053227	0.012438042
L1CAM	349	206	-0.345430567	0.0053253	0.01244158
TCAIM	2100	1723	0.129685505	0.0053261	0.012441287
UBASH3B	5846	4629	0.078402865	0.0053349	0.012459377
NDUFA6	2693	1859	-0.119535328	0.005341	0.012471307
KIAA1804	53	20	-0.990840228	0.0053469	0.012482789
RPS6	43705	32120	-0.029174314	0.0053806	0.012559177
LRP11	10150	7933	0.059610858	0.0053984	0.012598218
ALG2	1992	1638	0.132869841	0.0053994	0.012598189
LRRC45	600	376	-0.259077707	0.0054067	0.012612988
SNX27	2555	1760	-0.122595731	0.0054126	0.012624435
INTS9	471	425	0.266887913	0.0054156	0.012629077

VBP1	3579	2876	0.099659264	0.005417	0.012629809
NIPA2	4073	3259	0.093489669	0.0054319	0.012662321
PARD6G	1136	749	-0.185773079	0.0054404	0.012679661
TSPAN14	3952	2769	-0.098065761	0.0054547	0.012710722
PLSCR1	624	550	0.233037721	0.005455	0.012709035
ETV3	476	429	0.265168206	0.0054848	0.012775933
NRIP3	2466	2009	0.119456896	0.0054928	0.012792223
PIM2	267	255	0.348809637	0.005505	0.012818239
FOSB	27	40	0.982192724	0.0055375	0.012891553
TSTD3	247	238	0.361602663	0.0055377	0.012889528
MTG1	893	579	-0.209944695	0.0055442	0.012902393
DEXI	1279	850	-0.174329386	0.0055489	0.012910836
HMGB3	2355	1922	0.122033408	0.0055514	0.012914203
EEPD1	273	260	0.344762804	0.0055644	0.012942119
RGL3	148	76	-0.54637372	0.0055695	0.012951525
SHISA4	788	506	-0.223906113	0.0055718	0.012954416
NFKBIB	811	522	-0.220499976	0.0055886	0.012991066
DHRS1	736	470	-0.231892878	0.0055941	0.013001466
EVI5L	2401	1650	-0.126017247	0.0056079	0.013031005
CDR1	279	265	0.340879369	0.0056182	0.013052636
POLM	359	213	-0.337978282	0.0056244	0.013064489
RBM43	1185	1000	0.170265073	0.0056462	0.013112776
TIPARP	2811	1945	-0.116161167	0.005651	0.013121335
ANKRD27	1355	904	-0.168746042	0.0056788	0.013183544
ZNF594	595	526	0.237325263	0.0056837	0.013192307
ORAOV1	1047	687	-0.192727306	0.0056891	0.013202503
ANP32E	5502	4362	0.080185762	0.0056891	0.013200122
NTMT1	828	534	-0.217638894	0.0056936	0.013208047
CEP85L	1388	1161	0.157512536	0.0056943	0.013207104
FAM66C	71	83	0.640444444	0.005701	0.013220335
VTI1B	2211	1514	-0.131181688	0.0057016	0.013219178
TMEM110	1017	666	-0.195573465	0.005707	0.013229128

GCH1	50	63	0.748575865	0.0057291	0.0132781
MARCH4	2885	2335	0.110003363	0.0057612	0.013350001
CEP57	2186	1789	0.126012118	0.0057657	0.013357849
ZNF566	529	472	0.250671269	0.0057692	0.013363359
KIAA0586	1842	1519	0.13700094	0.0057706	0.013364201
WDFY2	1493	1002	-0.160179525	0.0057817	0.013387408
TMEM71	30	43	0.934526291	0.0057936	0.013412485
FEZ2	5022	3991	0.083640382	0.0058288	0.013491386
KIAA1522	439	398	0.273699623	0.0058361	0.013505791
KIAA2018	2277	1860	0.123320462	0.0058473	0.013529219
PSPC1	1953	1606	0.132932075	0.0058488	0.013530334
EBF1	248	140	-0.409761162	0.0058572	0.01354721
ZNF473	539	480	0.247901265	0.0058786	0.013594129
RPP30	1268	843	-0.173798077	0.0059016	0.013644894
ZBED5	2299	1877	0.122574316	0.0059161	0.013675745
TMEM175	803	517	-0.220083575	0.0059182	0.013678024
FAN1	1039	883	0.18044182	0.0059361	0.013716916
VPS13A	4884	3448	-0.087151294	0.0059483	0.013742558
SLC35B1	2026	1663	0.130306126	0.0059509	0.013746119
PRMT6	766	664	0.208990981	0.0059655	0.013777218
COLEC12	115	56	-0.622982997	0.0059692	0.01378324
FAM86EP	181	97	-0.484780913	0.0059738	0.013791154
SENP5	3676	2949	0.097241188	0.0059772	0.013796505
SKA2	3381	2720	0.101308767	0.0059845	0.013810887
FBXL13	34	47	0.882278142	0.0059921	0.013825823
KIFC1	1177	779	-0.180266955	0.0059922	0.013823419
DACT1	403	368	0.284078059	0.0060236	0.013893437
HYDIN	36	49	0.859936974	0.0060395	0.013927412
POLA1	729	634	0.213716158	0.0060449	0.013937333
PKD1L1	40	53	0.821144491	0.0060497	0.013945798
UBE3B	3182	2565	0.104177122	0.0060813	0.014015996
DNAJC5	7032	5016	-0.072245591	0.0060852	0.014022463

PLCD3	2653	2153	0.113875776	0.0060924	0.014036403
LDLRAD3	446	272	-0.298284927	0.0060959	0.014041767
CDC25A	602	531	0.234100506	0.0060995	0.014047561
SFMBT1	123	130	0.495005439	0.0061001	0.014046364
ST13	15674	12147	0.047378778	0.0061011	0.014046083
SHANK1	768	493	-0.224366533	0.0061071	0.014057259
REEP6	184	99	-0.479053204	0.0061089	0.014058843
MRPL10	1117	737	-0.18474053	0.0061149	0.014069987
CMSS1	716	457	-0.23261329	0.0061269	0.014095028
IRF2BP2	5193	3674	-0.084064467	0.0061944	0.014247722
FAM195B	2216	1519	-0.12968388	0.0062059	0.014271437
EIF5	15788	12233	0.047101959	0.0062183	0.014297354
CLPP	1462	981	-0.160466138	0.0062218	0.01430269
HNRNPAB	8833	6336	-0.064179044	0.0062375	0.014336333
N4BP2L2-IT2	92	102	0.564015518	0.0062402	0.014339849
NCAPH	841	544	-0.213347017	0.0062641	0.014392111
RSBN1	1410	1177	0.15457129	0.0062707	0.014404469
DOPEY2	1743	1181	-0.146411473	0.0062905	0.014447299
C19orf12	1719	1164	-0.147326355	0.0063356	0.014548191
WDR43	4127	3297	0.09121258	0.0063456	0.014568638
LRP5	2352	1617	-0.12541625	0.0063591	0.014596802
PLEKHN1	475	292	-0.286807013	0.0063942	0.014674788
NUDC	6176	4392	-0.076642567	0.0064275	0.014748338
AP1B1	6499	4628	-0.074676752	0.0064355	0.014764041
TRIM37	2287	1866	0.121644752	0.0064507	0.014796251
A1BG	494	305	-0.280549667	0.0064666	0.014829984
PGLS	2261	1552	-0.127680303	0.0064773	0.014851798
SRPK1	5647	4470	0.077942332	0.0064898	0.014877742
TTYH2	193	105	-0.463059388	0.0064905	0.014876543
TDRKH	90	100	0.567155225	0.006498	0.014891024
MED22	1296	864	-0.169810369	0.0064997	0.014892215
GOLGA1	1540	1037	-0.155362325	0.0065086	0.014909895

FAM86C2P	86	39	-0.725710404	0.0065114	0.014912116
SYT14	86	39	-0.725710404	0.0065114	0.014912116
TRAF3IP2-AS1	207	114	-0.445444812	0.0065288	0.014947864
SYNC	878	753	0.193581057	0.006538	0.014966138
HERC2P9	358	330	0.297658569	0.0065529	0.014997392
CHRAC1	1113	735	-0.183485306	0.0065551	0.01499973
DYRK1B	908	777	0.190374433	0.0065628	0.015014548
PIP5K1C	5956	4232	-0.077851995	0.0065714	0.015031649
EXOSC4	504	312	-0.276725573	0.0066303	0.015163532
LOC100130691	89	99	0.568775321	0.0066303	0.015160755
RAD23A	4293	3021	-0.091807857	0.0066393	0.015178581
FCHSD1	1410	945	-0.162156796	0.0066421	0.015182078
CCDC51	427	387	0.273249628	0.006643	0.015179986
RPGR	427	387	0.273249628	0.006643	0.015179986
TTC28	2661	1840	-0.117110612	0.0066459	0.015182527
NLRC5	927	792	0.188083223	0.0066795	0.015256446
RPL24	23048	16821	-0.039225958	0.0066812	0.015257549
PPP4C	3725	2609	-0.09859135	0.006686	0.015265583
JOSD1	2699	2187	0.111680375	0.0066863	0.015263508
PPP2R5A	1638	1356	0.142573953	0.006694	0.015278206
CRYZL1	1061	899	0.176120496	0.0066949	0.015277551
LOC100289137	72	31	-0.800576559	0.0067084	0.015305512
FNBP1	2048	1400	-0.133636756	0.0067435	0.015382924
C9orf78	2292	1575	-0.126103084	0.0067677	0.015435105
FNIP1	3449	2770	0.098859979	0.0067715	0.015440942
HSPA1A	991	843	0.181799706	0.0067722	0.015439859
UHRF2	3868	2713	-0.096546615	0.0067832	0.015461958
ZNF788	537	477	0.24421931	0.0067935	0.015482784
C1QBP	3447	2408	-0.102353775	0.0067952	0.015483772
SLC41A3	1172	777	-0.177833934	0.0067998	0.015491409
MRFAP1	18032	13938	0.043616271	0.0068086	0.015508552
DHRS7B	454	409	0.264560677	0.006817	0.015524814

FZD9	101	48	-0.65809685	0.0068223	0.015534034
GRIPAP1	1849	1258	-0.140461171	0.0068816	0.015666403
LOC100131094	63	26	-0.861688074	0.006919	0.015748582
RC3H1	3048	2458	0.104774145	0.0069206	0.015749214
LOC284889	84	38	-0.729237778	0.0069246	0.015755664
TEKT4P2	312	183	-0.354550249	0.0069317	0.015768918
RABGEF1	2018	1654	0.128185192	0.0069472	0.015801237
WDFY3-AS2	137	70	-0.553596934	0.0069553	0.015816722
ARL4D	655	416	-0.239759247	0.0069601	0.015824827
BTBD9	1474	1226	0.149374586	0.0069707	0.015845959
EGR1	869	745	0.19303638	0.0069828	0.015870642
NTAN1	2163	1483	-0.129362936	0.0069918	0.015888194
FLYWCH2	720	461	-0.228078024	0.0069949	0.015892252
CCNK	3787	2655	-0.097191428	0.0070094	0.015922297
TMEM200C	145	75	-0.535938268	0.0070108	0.015922646
ERGIC3	7994	5725	-0.066489748	0.0070501	0.016008855
ATP6AP1	5165	4096	0.080599498	0.0070777	0.016068718
HEBP2	2671	2164	0.111472655	0.0070843	0.016080618
PPP6R2	3817	2677	-0.096669916	0.007086	0.016081649
EHD3	2927	2033	-0.110660399	0.0070867	0.01608035
PNKP	1252	834	-0.170963142	0.0070925	0.016090628
CIRBP-AS1	119	59	-0.597022582	0.0070938	0.016090452
RIOK2	2530	2054	0.114450928	0.0070977	0.01609653
PDAP1	7031	5020	-0.070890399	0.0070996	0.016097753
POLH	4026	3216	0.091072366	0.0071121	0.016123294
ME2	2423	1670	-0.12179417	0.0071184	0.016134719
STK38	4376	3083	-0.090125717	0.0071217	0.016139078
ARMC8	1951	1601	0.129911662	0.0071297	0.016154298
APOBEC3B	391	236	-0.313229616	0.0071539	0.016206168
GLIS1	197	108	-0.452012186	0.0071556	0.016207175
PRPF6	4476	3156	-0.088960699	0.0072089	0.016324798
TPP1	7126	5090	-0.070274695	0.0072094	0.016323015

HDDC3	382	230	-0.316786645	0.0072098	0.016321091
MAP3K13	992	843	0.180344642	0.007213	0.016325247
ZNF883	94	44	-0.680005101	0.0072177	0.016332848
ATP6V0C	7276	5200	-0.069481788	0.0072555	0.016415419
VPS13B	7208	5667	0.068138323	0.007277	0.016461177
ANXA5	53563	40876	0.025169391	0.0072808	0.016466727
HABP4	1833	1508	0.133581774	0.0072862	0.016476075
GSPT1	12975	10080	0.050913232	0.0073021	0.016508824
MTX1	1226	816	-0.17216579	0.0073064	0.016515615
STX12	5084	3599	-0.083215796	0.0073298	0.016565509
RUVBL1	2722	2203	0.10995456	0.0073464	0.016600051
GALT	516	321	-0.269645637	0.0073623	0.016632851
LTK	82	37	-0.732946507	0.0073633	0.01663067
ZNF837	82	37	-0.732946507	0.0073633	0.01663067
UBAC2	1874	1540	0.13196153	0.0073698	0.01664091
PPFIA1	3796	3037	0.093319048	0.0073832	0.01666812
FBXL19	1619	1095	-0.149019984	0.0073851	0.016669236
DBP	203	112	-0.442828863	0.0073852	0.016666566
PARK7	7118	5085	-0.070072027	0.0074095	0.01671844
ANKIB1	4765	3786	0.083350329	0.0074433	0.016791684
RALBP1	7931	6221	0.064795855	0.0074584	0.016822704
RTCA	3372	2708	0.098775334	0.0074696	0.016844848
ZNF449	799	688	0.199365194	0.0074848	0.016876073
ANKZF1	920	785	0.186210925	0.0074866	0.016877072
CUL1	4659	3704	0.0842159	0.0074891	0.016879664
LRP4	804	692	0.198728668	0.0074961	0.016892348
SMAD2	4531	3197	-0.087958615	0.0075062	0.016912067
WSB2	6866	4901	-0.071241579	0.0075093	0.016915854
XYLT1	2914	2352	0.106039315	0.0075158	0.016927615
FEM1A	1306	873	-0.165949206	0.0075492	0.016999752
SCARB1	1819	1238	-0.139982097	0.0075494	0.016997168
PXMP2	426	260	-0.297189675	0.0075615	0.017021255

FOXO4	297	278	0.319774084	0.0075734	0.017045045
SETD5-AS1	471	422	0.256668071	0.0075856	0.017069328
KCNN2	569	502	0.234410843	0.0075888	0.01707345
SMC2	3713	2972	0.094000932	0.0076016	0.017099246
ACBD5	2181	1780	0.122039603	0.0076104	0.017115741
LRP12	3766	3013	0.093319805	0.00762	0.017134303
HSPA1L	82	92	0.581162083	0.0076217	0.017135026
ABHD6	451	277	-0.288089325	0.0076224	0.017133542
NLGN1	736	473	-0.222713451	0.0076936	0.01729046
NUDT12	898	767	0.187663265	0.0077334	0.017376744
KLF4	508	316	-0.269751807	0.0078108	0.017547587
FARSB	3756	2635	-0.096241969	0.0078247	0.017575544
MRE11A	1418	1180	0.150081459	0.0078295	0.017583107
ALG3	1504	1014	-0.153594783	0.0078322	0.017586002
NIT2	1029	678	-0.186733672	0.0078416	0.017603927
IFNGR2	1629	1103	-0.147401681	0.0078422	0.017602299
FAM98B	1715	1414	0.136725675	0.0078561	0.017630184
ZNF680	367	336	0.287833302	0.0078764	0.017672525
APLP1	1090	721	-0.181104839	0.0078934	0.017707618
ATP11A	2387	1646	-0.121082099	0.0079099	0.017741265
YWHAG	16734	12942	0.044430374	0.0079109	0.017740462
CDK5RAP3	3307	2310	-0.102478062	0.0079464	0.017816773
DENND2D	27	39	0.945666848	0.0079551	0.017833149
C11orf45	73	32	-0.774672427	0.0079708	0.017865121
PCGF2	646	411	-0.237243639	0.0079738	0.017868647
RBM42	2806	1948	-0.1113692	0.0079767	0.017871875
CTNNBL1	1609	1089	-0.14800824	0.0079778	0.017871047
DTX3	496	308	-0.272257638	0.0080064	0.017932049
TENM1	57	23	-0.894175926	0.0080305	0.017981178
TGFBR3L	57	23	-0.894175926	0.0080305	0.017981178
IFT46	1176	988	0.163847018	0.0080434	0.018005226
CCT2	10808	7796	-0.056141452	0.0080437	0.018002549

TRPS1	1103	930	0.169021962	0.0080467	0.018005967
EML5	210	204	0.373331956	0.0081422	0.018216422
LAMTOR1	3186	2223	-0.104086187	0.0081577	0.018247957
NOL9	1977	1619	0.126942246	0.0081613	0.018252614
NDUFB5	2458	1995	0.114055962	0.008186	0.018304685
CISD3	576	363	-0.250947132	0.0081998	0.018332151
SLC13A3	171	92	-0.479138427	0.0082011	0.018331881
CALM3	14410	10450	-0.048415261	0.0082262	0.0183846
DAW1	41	53	0.785520582	0.0082358	0.018402828
BCOR	696	604	0.210613375	0.0082391	0.018406813
WDR85	647	412	-0.235969243	0.0082528	0.018434067
PPP1R12C	4261	3003	-0.089635445	0.0082593	0.018445388
AVPI1	927	607	-0.195720691	0.0082677	0.018460825
TRIP4	1324	887	-0.162744981	0.0083036	0.018537719
GPBP1	3699	2959	0.093126528	0.0083139	0.018557271
TSPAN12	185	101	-0.458017846	0.0083349	0.018600786
TMEM33	4295	3420	0.086490325	0.0083383	0.018604983
ORAI3	1086	916	0.169547532	0.0083392	0.018603662
PFKM	4306	3036	-0.089024397	0.0083551	0.018635972
NAP1L3	188	185	0.391944741	0.0083645	0.018653549
DEF6	35	47	0.840457966	0.0083646	0.018648701
DNAH6	35	47	0.840457966	0.0083646	0.018648701
ZNF395	537	475	0.238157557	0.0083682	0.018651653
WDR75	2442	1982	0.114045894	0.0084028	0.018725537
RNFT2	55	22	-0.906775963	0.0084188	0.018757804
AIMP1	2578	2088	0.11102158	0.0084225	0.018762601
CREG1	627	548	0.220862582	0.0084277	0.018771028
ZNHIT1	2688	1864	-0.112979146	0.0084517	0.018821029
ADAMTS19	71	31	-0.780398677	0.0084527	0.018819799
RSBN1L	967	821	0.179018464	0.0084719	0.018859189
PLCD1	795	515	-0.211230296	0.0084782	0.018869846
EIF2S3	11236	8113	-0.054669004	0.0084882	0.018888837

UBR7	2488	1720	-0.117425789	0.0085061	0.018925301
GPM6B	147	77	-0.517733672	0.0085151	0.018941983
ANP32A	3909	2748	-0.093265449	0.008523	0.018955959
GOLPH3	5725	4522	0.074837431	0.0085362	0.018982002
SLIT2	687	440	-0.227654444	0.0085422	0.018991896
RBBP5	2070	1691	0.123398024	0.0085475	0.019000441
CCDC144B	1035	875	0.172876286	0.0086038	0.019122101
SPRTN	911	776	0.18375773	0.0086625	0.019249038
MARK2	1669	1133	-0.143683962	0.0086637	0.019248389
CLK2	1426	960	-0.155715539	0.008687	0.019296674
FAM193B	1237	826	-0.167479682	0.0087072	0.019338065
SLC25A5	5164	3662	-0.080705078	0.008739	0.019405373
FAM108A1	2447	1691	-0.11798531	0.0087493	0.01942464
ARSA	1680	1141	-0.14301031	0.0087507	0.019424431
UBE2A	5230	4140	0.077971953	0.0087796	0.019485039
C11orf84	1159	771	-0.172925669	0.0088089	0.019546564
YDJC	562	354	-0.251668638	0.0088295	0.019588873
HERPUD2	1715	1412	0.134683644	0.0088559	0.019643932
PDLIM3	86	95	0.558742985	0.0088683	0.019667918
GRB2	6423	4582	-0.072117706	0.0088685	0.01966481
PPP1R14B	6252	4457	-0.073092686	0.0088844	0.019696585
SUV39H2	671	583	0.212335249	0.0088911	0.019707821
RGS17	411	251	-0.296298898	0.008893	0.01970864
PTS	1404	1167	0.148413757	0.0088962	0.019712277
SUMO3	5118	3629	-0.080855959	0.0089174	0.019755704
SSBP1	3416	2738	0.095966603	0.0089303	0.019780787
TMEM44	777	503	-0.212204067	0.0089491	0.019818783
MANF	2210	1521	-0.123874085	0.0089703	0.019862135
CHIC1	1832	1250	-0.136339277	0.0089754	0.019869989
C2orf74	163	163	0.415152132	0.0090206	0.019966479
H2AFY	6548	4674	-0.071244456	0.0090324	0.019988981
PLXNB3	627	399	-0.236924565	0.0090411	0.020004771

SLC39A11	1038	686	-0.18237383	0.0090531	0.020027811
AP3D1	11932	8628	-0.052585669	0.0090569	0.020032612
SMIM15	4339	3062	-0.087736172	0.0090613	0.020038884
MYH15	116	58	-0.584847868	0.0090662	0.02004608
POLR3D	1801	1228	-0.137335489	0.0090736	0.020058958
PEX13	1685	1388	0.135411108	0.009083	0.020076168
ZNF500	566	357	-0.249725847	0.0090946	0.020098095
MLLT6	2539	1758	-0.115173193	0.0091033	0.02011381
SPRYD4	323	192	-0.335275722	0.0091438	0.020199642
TMEM98	3007	2096	-0.105534019	0.0091828	0.020282298
BBS1	664	577	0.212540209	0.0091838	0.020280799
PSMD6-AS2	147	149	0.434648307	0.0091863	0.020282709
VAPA	6550	5154	0.069349762	0.0092015	0.020312708
RAP1B	11419	8882	0.052672294	0.009202	0.020310335
RFTN1	4700	3730	0.081667005	0.0092258	0.020359261
CARKD	1781	1214	-0.137766963	0.0092494	0.020407669
REST	7285	5718	0.065733779	0.0092711	0.020451931
LOC100505687	231	221	0.35130565	0.0092795	0.020466729
SMCR8	2492	2019	0.111488974	0.0092832	0.020471413
RAD51	306	284	0.307511409	0.0092899	0.020482606
RBMS2	10769	7773	-0.055188736	0.0092958	0.020491874
HOXB5	1519	1257	0.142014912	0.0092995	0.020496316
NAGLU	2120	1457	-0.125911256	0.0093063	0.0205077
DDX31	752	486	-0.214624216	0.0093269	0.020549502
BIVM	1388	934	-0.156360981	0.0093394	0.020573529
AGTRAP	936	795	0.179598462	0.0093558	0.020605956
STYX	2969	2390	0.102185657	0.009366	0.02062477
ZNF606	579	508	0.226417281	0.0093835	0.020659705
TAX1BP1	9128	6567	-0.059912205	0.0093931	0.02067717
HS1BP3	831	711	0.190153215	0.0093954	0.020678567
MOB1A	3009	2421	0.101471104	0.0093979	0.020680377
BRIP1	1400	1163	0.147576401	0.0093995	0.020680251

C20orf111	1433	1189	0.145862237	0.0094231	0.020728427
TUBGCP3	1414	1174	0.14680242	0.0094292	0.020738247
SRSF3	9668	6964	-0.058149138	0.0094516	0.020783814
AUNIP	121	126	0.473568818	0.0095274	0.020946831
TBC1D9	2864	2308	0.103763863	0.0095376	0.020965558
RNF146	2079	1696	0.121398543	0.0095489	0.020986672
KLF10	3220	2251	-0.1013425	0.0095788	0.021048591
EXOSC2	1207	806	-0.1674218	0.0095846	0.02105775
EIF4ENIF1	1410	950	-0.154543612	0.0095904	0.021066811
SECTM1	336	201	-0.3261136	0.0095968	0.021077122
STXBP5-AS1	286	267	0.315976727	0.0096599	0.021211839
HAX1	2633	2128	0.107942761	0.0096707	0.021231942
SPRY4	4783	3387	-0.082755673	0.0096898	0.021270189
NTHL1	382	232	-0.304295701	0.0096918	0.02127063
IL32	540	476	0.233154298	0.0096983	0.021281227
TRIM26	2144	1475	-0.12443782	0.0097286	0.021344013
LOC643837	760	492	-0.212188971	0.0097362	0.021356791
SPEN	4177	2946	-0.088557581	0.0097619	0.021409543
ZMIZ2	2925	2038	-0.106130441	0.0097759	0.021436379
POGLUT1	1534	1268	0.140408394	0.0097841	0.02145051
SMPDL3A	768	660	0.196511845	0.009787	0.021453233
TP53I13	1075	713	-0.177210546	0.009787	0.021449515
MMAA	650	565	0.212963281	0.0097989	0.021471716
CASP3	2476	1714	-0.115492073	0.0098066	0.021484769
CYP20A1	2024	1389	-0.128010559	0.00981	0.021488562
TMEM143	204	114	-0.424383196	0.0098252	0.021517963
PTGIR	254	240	0.333358041	0.0098624	0.021595568
ANKFY1	6945	5455	0.066756634	0.0098678	0.021603611
TMEM43	7151	5613	0.065779188	0.0098901	0.02164867
C16orf55	190	105	-0.440457959	0.0099104	0.02168932
KANSL3	3270	2288	-0.100051452	0.0099365	0.021742621
FN3KRP	1575	1068	-0.14528805	0.0099431	0.021753215

FAM111B	774	502	-0.20949407	0.009971	0.021810451
FURIN	8379	6554	0.06074973	0.0099762	0.021818009
SYNGAP1	1680	1143	-0.140483698	0.009982	0.02182688
RIN2	5895	4648	0.072270387	0.0099902	0.021841015
FAM110B	91	99	0.536714112	0.0099921	0.021841322
SCP2	8395	6031	-0.061980958	0.009995	0.021843763
PIH1D1	1005	664	-0.182788223	0.0100569	0.021975162
PCSK5	343	206	-0.320412107	0.0100629	0.021984562
ATP11C	4304	3422	0.084313814	0.0100707	0.02199759
CSNK2B	4658	3297	-0.083404621	0.0100994	0.022056572
DNAJB2	2534	2050	0.109359517	0.0101048	0.022064445
CXCL12	505	316	-0.261206698	0.0101095	0.022070787
MAP3K6	1378	928	-0.155227046	0.0101196	0.022089059
NEU1	2060	1680	0.120969027	0.0101534	0.022158852
RAB32	2289	1580	-0.119640773	0.0101557	0.022159961
ZNF850	286	168	-0.352401782	0.0101568	0.022158465
PVRL3	9565	6891	-0.057899479	0.010157	0.022155152
PTGER2	2798	1947	-0.107990947	0.0101578	0.022152875
ANKRD13B	301	178	-0.342734114	0.0102007	0.022242691
PAPSS1	4350	3073	-0.086215502	0.0102162	0.022272507
USF1	1313	882	-0.158864224	0.0102427	0.022326272
EDEM2	1794	1225	-0.135246009	0.0103028	0.022453345
PRKD3	3462	2428	-0.096685171	0.0103209	0.022488929
EFHA1	3289	2636	0.095853494	0.0103902	0.0226359
AKR1E2	89	97	0.539331543	0.0104305	0.022719781
CAPRIN2	1975	1613	0.123045917	0.0104341	0.022723652
ORAI2	4427	3516	0.082757829	0.0104355	0.022722855
HEBP1	2042	1403	-0.126315726	0.0104575	0.022766749
ZNF804A	956	630	-0.186506658	0.0104746	0.022799999
KDM6B	1373	1140	0.146854331	0.0104944	0.022838958
LRRC49	570	361	-0.24381095	0.0105185	0.022887584
CCNT1	3292	2638	0.095632361	0.010536	0.022921562

GAS2L1	1697	1156	-0.138693035	0.0105506	0.022949238
CLK1	3841	3063	0.088615535	0.0105589	0.022963443
PRPF18	1263	1053	0.152802929	0.0105731	0.022990269
RUNX2	1184	791	-0.166767349	0.0105906	0.02302418
WDHD1	1320	1098	0.149492257	0.0106048	0.02305099
MEIS3P1	476	423	0.244848222	0.0106106	0.023059715
ARMC5	500	313	-0.260613306	0.0106571	0.023156756
LDOC1L	2956	2377	0.100647766	0.0106799	0.023202218
IRAK1BP1	64	74	0.624605497	0.0106874	0.023214443
GPBP1L1	3769	3007	0.089295205	0.0106918	0.023219967
SLC26A1	177	97	-0.452540576	0.0107081	0.023251276
FAM135A	632	404	-0.230417134	0.0107176	0.023267834
RBAK	1527	1261	0.139020345	0.0107299	0.02329052
FBXL7	1374	926	-0.154145774	0.0107547	0.023340339
EIF4E	5343	4221	0.075086944	0.0107908	0.02341447
KIAA1704	1127	945	0.161050851	0.0108055	0.023442306
B3GALNT1	287	267	0.310941137	0.0108108	0.023449746
PRMT5	3604	2879	0.091120909	0.0108358	0.023499875
OBSL1	1885	1291	-0.130923391	0.010846	0.023518
SHROOM4	76	85	0.576615554	0.0108715	0.023569089
DNMT3A	613	534	0.2161048	0.0108765	0.023575765
AQP7P1	87	95	0.542064244	0.0108867	0.023593747
MTPAP	1044	879	0.16696549	0.0108973	0.023612688
PPP4R1	7025	5513	0.065491508	0.0109118	0.023640126
WDR27	363	220	-0.307313893	0.0109225	0.023659176
REXO4	1573	1068	-0.143454892	0.0109444	0.023702409
NUDT1	523	329	-0.253571231	0.0109481	0.023706371
RTTN	1005	665	-0.180617124	0.0109512	0.023708827
CCDC66	458	408	0.248373686	0.010955	0.023712961
WDFY1	6645	4751	-0.068885861	0.0109571	0.023713341
USP10	4456	3152	-0.084329566	0.0109748	0.023747659
RPS25	15148	11008	-0.045422819	0.0109799	0.023754477

ZNF227	700	604	0.202345759	0.0110209	0.023839114
PHTF2	5821	4588	0.071750506	0.0110441	0.02388505
PWP2	1410	952	-0.151509552	0.0110618	0.023919283
CTBP2	3615	2887	0.090727592	0.0110647	0.023921396
COX14	715	616	0.200139241	0.0110808	0.023952027
ZNF22	811	693	0.18830557	0.0110812	0.023948602
DENND3	4907	3481	-0.080187326	0.0110903	0.023964176
NUBPL	720	620	0.199423441	0.0110987	0.023978287
NRBP2	3377	2368	-0.096920967	0.0111081	0.023994444
PSME4	12459	9025	-0.050037309	0.0111232	0.024022791
CNOT6L	1571	1295	0.136421049	0.0111379	0.024050318
MEG9	514	323	-0.255082063	0.0111894	0.02415742
ENGASE	888	583	-0.191911661	0.0112077	0.024192766
EXOC3	2582	1793	-0.110961381	0.0112187	0.024212261
ZBTB45	588	374	-0.237625753	0.011239	0.024251905
BTN3A1	780	668	0.19152611	0.0112407	0.024251234
NUP88	2197	1785	0.115541336	0.0113036	0.024382716
POP1	2207	1523	-0.120018556	0.0113471	0.024472433
P4HA3	312	186	-0.331091276	0.0113504	0.024475188
ZNF524	282	166	-0.349359789	0.0113508	0.024471979
HMOX2	1923	1319	-0.128762066	0.011353	0.02447241
WASL	4395	3489	0.082102564	0.0113645	0.024492922
NAA35	1560	1286	0.136496745	0.0113778	0.024517315
CASP4	3581	2860	0.09080476	0.0113785	0.024514575
THAP7-AS1	126	65	-0.539759979	0.0114153	0.024589789
UEVLD	1741	1428	0.129231908	0.0114652	0.024692977
MAP7	36	47	0.799815982	0.0114974	0.024757928
RHPN2	320	294	0.292896382	0.0114975	0.024753863
ANKRD35	238	137	-0.381633549	0.01152	0.024798024
NCOA1	1773	1453	0.127994298	0.0115256	0.024805933
IGFBP2	212	120	-0.405877727	0.0115575	0.024870244
MRPL21	970	641	-0.182508259	0.0115731	0.024899473

MYSM1	4362	3463	0.082184749	0.0115921	0.024935933
CENPI	713	461	-0.213983194	0.0115949	0.024937742
MFSD12	2027	1394	-0.124963396	0.0115968	0.024937506
TROVE2	2764	2226	0.102848109	0.0116152	0.024972758
FNDC4	778	666	0.190904154	0.0116232	0.024985767
SLC2A1	9441	6805	-0.057192386	0.0116411	0.025019914
DGKH	394	355	0.264775527	0.0116466	0.025027355
TNRC18	8258	5936	-0.061149195	0.0117031	0.025144502
FTSJ1	2145	1479	-0.121203464	0.0117311	0.025200263
SEC23IP	5856	4613	0.070941871	0.0117405	0.02521598
SKIV2L2	5319	4200	0.074386423	0.0117477	0.025227259
ZDHHC4	1069	711	-0.173188256	0.0117579	0.025244632
TRPV1	181	100	-0.440837566	0.0117648	0.025255115
TMEM161B	747	641	0.194368246	0.0117697	0.02526138
WAC	5548	4376	0.072797082	0.0118147	0.025353552
PCGF1	295	273	0.303338128	0.011818	0.025356338
WBP2	2460	1706	-0.112888537	0.011825	0.025367029
TMEM57	1901	1304	-0.128662531	0.011896	0.02551477
RHOU	265	155	-0.358572012	0.0119063	0.025532596
LOC401397	2586	2087	0.105860457	0.0119433	0.025607462
TIMMDC1	2709	1886	-0.107278586	0.0119769	0.025675026
WBP4	1876	1533	0.123850001	0.0119796	0.02567639
FBXO25	751	644	0.193399912	0.0119809	0.025674907
SGOL1	264	247	0.319125244	0.0119915	0.025693025
CCDC92	2756	1920	-0.106317445	0.0119967	0.025699833
NCK2	1104	736	-0.169810369	0.0120126	0.025729387
SCAPER	781	668	0.189677686	0.0120231	0.025747606
QPRT	816	696	0.185670286	0.0120341	0.025766684
SNAP47	1152	770	-0.166058234	0.0120479	0.025791854
PDS5B	2988	2088	-0.101906304	0.0120993	0.025897269
LRRC37A4P	1190	797	-0.163157812	0.0121244	0.025944484
TRIM14	1190	797	-0.163157812	0.0121244	0.025944484

NOA1	1101	734	-0.169810369	0.0121293	0.025948197
NTF3	469	416	0.242147737	0.0121671	0.026024562
KIAA0319L	4337	3068	-0.084246829	0.0121803	0.026048323
SH2B1	2244	1551	-0.117721858	0.0122416	0.026174992
RTL1	93	45	-0.632153583	0.0122472	0.026182529
CCDC23	190	106	-0.426783022	0.0122521	0.02618832
THSD4	10944	7913	-0.052691341	0.0122637	0.026208699
SEMA4D	88	42	-0.651962064	0.0122655	0.026207982
SLC27A3	239	138	-0.377190219	0.0122705	0.026214189
LYRM1	904	766	0.176173751	0.0122877	0.026246434
IFIH1	101	107	0.498407635	0.0122943	0.026256097
MRPL48	715	463	-0.211778917	0.0123252	0.026317591
CBR3	242	140	-0.374428089	0.0123256	0.026313996
ARRB1	559	489	0.222138314	0.0123355	0.026330465
KLHDC8B	729	626	0.195395974	0.0123479	0.026352448
RFX5	2127	1467	-0.120799031	0.0123726	0.026400645
MED23	3439	2748	0.09154502	0.0123946	0.026443144
MYL6B	2546	1769	-0.110146239	0.0124219	0.026496866
FOXO3	3421	2734	0.091747271	0.0124281	0.026505489
SIX4	3412	2727	0.091849185	0.0124447	0.026536375
APBA3	329	198	-0.317435022	0.0124735	0.026593297
TMEM60	553	484	0.222879699	0.0125051	0.026655996
PINK1	3610	2880	0.089222106	0.0125564	0.026760709
ZFYVE26	2060	1676	0.117529943	0.0125605	0.026764931
PA2G4	9165	6605	-0.057424188	0.0125724	0.026785701
SETMAR	397	244	-0.287105728	0.0125818	0.026801115
LINC00856	57	24	-0.832775382	0.0125897	0.026813413
GGCX	3614	2883	0.089126462	0.0126037	0.026838707
ACD	530	335	-0.246679132	0.012607	0.026841125
EXD3	127	66	-0.529138436	0.0126189	0.026861734
MED10	1427	966	-0.147738109	0.0126307	0.026882393
CPNE3	11355	8217	-0.051491902	0.0126485	0.026915616

RAG1	141	142	0.425347899	0.0126771	0.026971927
ERO1L	5592	3985	-0.073632505	0.0126833	0.026980455
ZNF597	133	135	0.436685293	0.0127606	0.027140208
MTERFD1	621	398	-0.226672706	0.012775	0.027166338
RALY	5485	3907	-0.074278233	0.0127763	0.027164305
PBLD	488	431	0.235958853	0.0127921	0.027193241
C1D	1038	872	0.163745728	0.0128354	0.02728065
NAA30	2615	2108	0.104216052	0.0128501	0.027307369
TCEAL3	1701	1162	-0.134620941	0.0128884	0.027383985
VPS39	5858	4180	-0.071753119	0.0128885	0.027379562
TANGO6	1338	903	-0.152128091	0.0129604	0.027527564
SNHG12	429	266	-0.27439927	0.0129669	0.027536786
IP6K2	3492	2455	-0.093178403	0.0129895	0.027580001
CARHSP1	2273	1573	-0.115926882	0.0130074	0.02761324
KLHL42	2954	2371	0.097977968	0.0130175	0.027630052
EFNB1	2388	1656	-0.112948032	0.0130834	0.027765238
LOC81691	330	199	-0.314545462	0.0131119	0.027820951
MIDN	3189	2553	0.094241572	0.0131316	0.027857916
PRR14L	4338	3441	0.080949971	0.0131745	0.027944123
EPS15	3948	3140	0.084794701	0.0131779	0.027946756
DNAJC11	2367	1641	-0.113332335	0.0131827	0.027952064
NOP56	4529	3210	-0.08146711	0.0131958	0.027975182
VPS53	5017	3964	0.075284156	0.0132504	0.028086009
CDKL5	382	344	0.263988058	0.0132776	0.028138934
C9orf69	1506	1241	0.135933477	0.0132795	0.028138265
ELK1	2214	1795	0.112480754	0.0133505	0.028283749
FLOT2	3626	2553	-0.091033256	0.0133641	0.028307739
FBXO45	1122	938	0.156739284	0.013386	0.028349386
KCTD3	4073	3236	0.083271925	0.0134361	0.028450691
IARS	23129	16922	-0.03565067	0.0134746	0.028527386
GMPR	353	320	0.273555853	0.0134829	0.028537621
MEF2C	353	320	0.273555853	0.0134829	0.028537621

POLA2	1188	797	-0.160731075	0.0135056	0.02857826
CCBL2	1861	1519	0.122195946	0.0135663	0.028701835
SEMA3C	4840	3827	0.076358985	0.0135843	0.028735171
LZTR1	2520	1752	-0.109268827	0.0135988	0.028760876
SLC12A2	3216	2573	0.093336182	0.01365	0.028864372
MORN2	414	370	0.253046635	0.0136501	0.028859547
SLC26A6	980	650	-0.177189899	0.0136555	0.028866206
ARAP3	1031	686	-0.172611719	0.0136572	0.028864801
GALE	835	548	-0.192448173	0.013665	0.028876487
GTF3C3	2016	1640	0.117352308	0.0136866	0.02891724
MED19	702	455	-0.210452353	0.0137063	0.028953814
CSAD	926	612	-0.182328409	0.0137161	0.028969564
ESF1	1761	1206	-0.13101287	0.013738	0.029010935
BNIP3L	9908	7711	0.053476231	0.013764	0.029061034
RMI2	288	266	0.300509566	0.0137749	0.029079045
CLINT1	7491	5862	0.061386778	0.0138129	0.029154349
XRCC1	1439	976	-0.144961407	0.0139483	0.029435203
EIF1B	1660	1361	0.128635957	0.0140088	0.029557812
ACSL5	75	83	0.561372873	0.0140253	0.029587586
PPP1R18	8245	5933	-0.059605579	0.0140472	0.029628707
TICRR	467	413	0.237871363	0.0140531	0.029636091
SLC16A14	483	426	0.233982373	0.0140833	0.029694769
SVEP1	3047	2134	-0.098677192	0.0140896	0.029703045
GBA	3426	2409	-0.092938626	0.0141183	0.029758596
SCO1	2068	1680	0.115377179	0.0141233	0.029764033
FECH	1636	1342	0.129364055	0.0141542	0.029824139
AIG1	811	690	0.182046579	0.0141656	0.029843072
DENND4A	3016	2417	0.095733176	0.014255	0.030026302
AMZ1	60	26	-0.791298746	0.0142842	0.030082903
AZI1	830	545	-0.191702975	0.0143113	0.03013488
P2RX5	74	82	0.563250771	0.0143197	0.030147364
ZDHHC3	4150	2936	-0.084107237	0.014356	0.030218776

FAM193A	2076	1433	-0.119615702	0.0143867	0.030278265
ZNF626	231	218	0.331587415	0.0143977	0.030296269
RAB28	745	637	0.189205079	0.0144216	0.030341456
NUDT19	775	661	0.185606093	0.0144303	0.030354618
SIRT6	615	395	-0.223581625	0.0144676	0.030427794
DOT1L	1828	1492	0.122133597	0.0144737	0.030435507
MAP2K3	5088	4016	0.07381273	0.0144829	0.030449749
POLB	891	588	-0.184457145	0.0144888	0.030456948
MIS12	839	712	0.178358562	0.0145691	0.030620672
PQLC1	1271	857	-0.153444789	0.0145962	0.030672509
KIAA1644	233	135	-0.372218416	0.0146003	0.030675806
STXBP2	73	81	0.565177576	0.0146193	0.030710727
BBS2	1781	1455	0.123483768	0.0146507	0.030771453
UCHL5	1804	1473	0.122710224	0.0146509	0.030766713
CFD	43	53	0.716807832	0.0146873	0.030837976
CEP120	1903	1309	-0.124658333	0.0146909	0.030840141
PGM2L1	1953	1345	-0.122933645	0.0147569	0.03097367
TMEM198B	448	280	-0.262919773	0.0147801	0.031017003
TUBB2B	245	143	-0.36161447	0.0147943	0.031041522
TTC7B	1519	1034	-0.139733552	0.0148048	0.031058361
MSX2	980	651	-0.174972074	0.0148397	0.031126454
COPB2	24406	18737	0.033806247	0.014845	0.031132239
BDNF-AS	337	306	0.275935193	0.0148462	0.031129457
SNHG7	480	302	-0.253333725	0.0148783	0.031191641
TAF4	728	474	-0.20389926	0.0149039	0.031240055
ZNF772	877	742	0.173994476	0.0149052	0.031237539
GALNT2	20140	15498	0.037170498	0.0149101	0.031242452
DTX2	525	333	-0.241643114	0.0149198	0.03125749
USP28	1023	858	0.16139554	0.0149407	0.031296054
GOSR2	4128	2921	-0.083828481	0.0149431	0.031295804
LAMA1	559	487	0.216225621	0.014962	0.031330095
TMEM68	989	831	0.164030088	0.0149661	0.031333362

GAB2	979	823	0.164735703	0.0150332	0.031468684
AHSA2	1933	1331	-0.123178935	0.0150776	0.031556359
TMEM100	58	25	-0.798972674	0.0151412	0.031681485
TTLL3	58	25	-0.798972674	0.0151412	0.031681485
TTC30B	141	141	0.415152132	0.0151545	0.031701163
RTEL1	40	50	0.737080227	0.015165	0.031717864
MLST8	964	640	-0.17580911	0.0152031	0.031792308
TMEM39B	471	296	-0.254977752	0.0152095	0.031800304
NDUFS5	6816	4887	-0.064824099	0.0152504	0.031880462
GTF3C2	2255	1563	-0.113657523	0.0152561	0.031886944
TBCA	4660	3685	0.076486796	0.0152728	0.031916489
COPB1	18060	13916	0.039098823	0.0152758	0.031917442
SEMA4B	819	538	-0.191105147	0.0152776	0.031915878
UBALD2	895	756	0.171650684	0.0152909	0.031938231
FEZ1	1686	1154	-0.131809181	0.015295	0.031941434
STPG1	253	236	0.314801606	0.0153355	0.032020721
MTCP1	58	67	0.623260327	0.0153357	0.032015741
UBE2Z	7867	6146	0.058986315	0.0153733	0.032088938
IKZF2	1462	1204	0.135044213	0.0153757	0.032088491
JAM2	121	63	-0.526431182	0.0153763	0.032084332
PUS10	419	373	0.247377518	0.0153835	0.032094053
TMEM39A	2648	1847	-0.104567124	0.0154049	0.032133358
HM13	8028	5777	-0.05956799	0.0154353	0.032191326
USP2	38	48	0.752187119	0.0154452	0.032206517
TSFM	843	555	-0.187892728	0.0154781	0.032269772
EIF2B2	1312	887	-0.149609579	0.0154815	0.032271497
CYP1B1	81	88	0.534733748	0.0154932	0.032290422
PGAP1	1721	1407	0.124527363	0.0155143	0.032328957
HNRNPH3	7648	5978	0.059732505	0.0155193	0.032334043
ELAVL1	4938	3513	-0.076071128	0.0155722	0.03243891
PCDH9	884	747	0.172214005	0.0155799	0.032449386
MRPL47	2049	1415	-0.1189658	0.0155824	0.032449209

GON4L	3303	2322	-0.093256866	0.0156105	0.032502402
EPB41	549	350	-0.234299095	0.0156594	0.032598662
SLITRK6	182	102	-0.420217166	0.0156691	0.032613407
TCF21	1900	1308	-0.123484746	0.0157125	0.032698222
FDX1L	847	558	-0.186944716	0.0157402	0.032750435
COX10	598	384	-0.223887042	0.0157538	0.032773248
KNSTRN	2139	1480	-0.116187175	0.0157548	0.032769941
ABCF2	5114	3642	-0.074569107	0.0157981	0.032854418
FPGS	1911	1316	-0.123016157	0.0157996	0.032852073
CLEC2B	572	366	-0.229019367	0.0158009	0.032849283
HNRPDL	11197	8689	0.049301942	0.0158402	0.032925614
CELF2	1196	994	0.148252499	0.015875	0.032992296
CCDC90B	3478	2772	0.087821457	0.0159089	0.033057253
GRIA4	211	121	-0.38708382	0.0159268	0.033088926
XKR8	757	495	-0.197712643	0.0159535	0.033138834
SPPL2B	1258	849	-0.152143332	0.0159675	0.033162359
LPAR6	331	201	-0.304483584	0.0159878	0.033199168
CLASRP	905	599	-0.180209657	0.0159929	0.033204042
NKIRAS1	663	570	0.197105181	0.0160543	0.033326036
NOL10	2377	1652	-0.109776085	0.0160594	0.033331033
SRRD	553	353	-0.232459165	0.0160724	0.033352558
EML3	1696	1162	-0.130373969	0.0161012	0.033406773
TTC14	2843	2280	0.096771857	0.0161419	0.033485533
HNRNPA3	21882	16816	0.035242101	0.0161616	0.03352095
EVC	3915	2768	-0.085016233	0.0162515	0.033701801
USP15	4229	3351	0.079427258	0.0163505	0.033901325
PSMB6	5062	3605	-0.074556116	0.0163579	0.03391103
NAT14	1128	757	-0.160249731	0.016397	0.033986426
LOC100506668	280	258	0.29709637	0.0164118	0.034011563
CNIH2	304	183	-0.317075543	0.0164361	0.034056244
ALS2CR11	54	63	0.637544553	0.0164487	0.034076617
ZNF584	531	338	-0.236536483	0.016459	0.034092217

RNASE4	377	338	0.257610855	0.0164894	0.034149612
KIRREL3	895	755	0.169741094	0.0165011	0.03416807
SYTL5	238	139	-0.360724559	0.0165407	0.03424432
RELB	303	277	0.285720314	0.0165784	0.034316762
FLVCR1	451	283	-0.257173249	0.0166296	0.034416964
RAB15	548	477	0.214965505	0.0166351	0.03442277
EIF4B	24744	18132	-0.03338854	0.0166389	0.034424924
ERCC2	2724	2187	0.098378649	0.0166399	0.034421256
PTPLA	801	680	0.178884635	0.0166562	0.034449095
TRAPPC4	1288	871	-0.149235838	0.0167189	0.03457308
TRIP13	1323	896	-0.147090292	0.0167957	0.034726184
TBP	495	434	0.225418649	0.0168059	0.034741504
PDE6D	563	489	0.211851675	0.0168127	0.034749834
MRPL20	1858	1279	-0.123582106	0.0168245	0.034768321
HOXB8	105	109	0.469090939	0.0168322	0.034778572
GPATCH8	4378	3465	0.07773553	0.0168393	0.0347874
SLC25A21	205	195	0.343002346	0.0168742	0.034853786
SHISA5	9458	6832	-0.054075035	0.0169051	0.034911699
LOC100506023	158	155	0.387495789	0.0169325	0.034962532
PRPF38A	1375	1134	0.137141153	0.0169609	0.035015388
SDR39U1	61	27	-0.760697704	0.0169652	0.035018586
ZNF721	1431	1178	0.134467999	0.0169815	0.035046376
ALDH16A1	776	509	-0.193238864	0.0170016	0.035082041
TRMT6	996	664	-0.169810369	0.0170442	0.035164062
NSMCE4A	746	488	-0.197142351	0.0170448	0.035159552
BCL7C	1390	944	-0.143073986	0.0170473	0.035158741
EIF3F	9882	7682	0.05183104	0.0171366	0.035337209
ZNF200	483	424	0.227193207	0.0172614	0.035588574
PGM5	95	100	0.489152713	0.0173515	0.035768382
ZNF569	587	508	0.206620125	0.0173708	0.035802386
TIMM17B	807	531	-0.188704681	0.0173767	0.035808618
CCAR1	6750	5286	0.062441055	0.0174208	0.035893463

CCDC85A	293	176	-0.320173104	0.0174535	0.035954907
TXNRD1	26675	20447	0.031553046	0.0174901	0.036024251
MBNL1	14960	11551	0.042059711	0.0174955	0.036029555
MGC2752	1423	968	-0.140704578	0.0175565	0.036149201
TXNL1	4220	3342	0.078620866	0.0176081	0.036249357
ACADS	269	160	-0.334382136	0.0176534	0.036336751
MIR31HG	892	591	-0.178733448	0.0176573	0.036338625
APC2	263	156	-0.338364639	0.0176681	0.036354896
VPS51	2025	1400	-0.117342949	0.0176818	0.036377112
DDX41	2240	1555	-0.11143202	0.0177053	0.036419393
KLHL9	5428	3875	-0.071072279	0.0177218	0.03644733
FAM60A	1273	1053	0.141425149	0.0177491	0.036497446
COX5B	2724	1905	-0.100783574	0.0177937	0.036583144
ITGB5	11528	8936	0.047710982	0.0178684	0.036730547
NCKAP5L	2614	2100	0.099282319	0.0179021	0.036793833
BACH2	63	71	0.587619328	0.017907	0.036797822
RBM48	433	383	0.238129499	0.0179094	0.036796767
TNFAIP8L1	658	427	-0.208699382	0.0179225	0.036817617
PI4K2B	3125	2496	0.090913876	0.0179277	0.036822186
ACO2	3041	2135	-0.095157615	0.0179562	0.036874658
ZFPL1	869	575	-0.180642089	0.0179735	0.036904059
CERS1	59	26	-0.767051199	0.01803	0.037014018
LOC728323	74	81	0.545548769	0.0180347	0.037017572
AP3B1	8177	6377	0.056458373	0.0180869	0.037118555
TIMM9	910	604	-0.176165864	0.0181192	0.037178768
KRAS	5140	3665	-0.072803029	0.0181232	0.03718076
ABCB7	766	651	0.180465283	0.0181446	0.037218616
RXRB	2364	1645	-0.10799032	0.0181451	0.037213413
ATXN7L1	879	741	0.168762509	0.018176	0.037270769
ZSCAN25	569	493	0.208311126	0.0181832	0.037279314
LOC100128361	76	36	-0.66285038	0.0181859	0.037278692
C11orf63	118	120	0.439399678	0.0182042	0.037310108

PTCH1	721	615	0.185739283	0.0182086	0.037312992
HAGH	1000	837	0.15845166	0.0182158	0.037321612
CCT5	15089	11647	0.041613333	0.0182335	0.037351679
DIABLO	1959	1590	0.1140615	0.0182364	0.037351474
MARCH6	10833	8405	0.04903903	0.0182618	0.037397425
ANGPTL4	836	552	-0.183682544	0.018343	0.037557522
CFLAR	4180	3310	0.078480406	0.0183637	0.037593631
EIF5A	27150	19923	-0.031365162	0.018414	0.037690516
APLF	175	169	0.364820456	0.0184227	0.037702052
CLDN20	47	56	0.667918202	0.0184271	0.037704793
ARHGAP39	399	355	0.24658241	0.0184353	0.037715539
ZCCHC17	2008	1628	0.112493562	0.0184416	0.037722102
SRPK2	3120	2193	-0.093488085	0.0184884	0.037811682
PHKA2	680	582	0.190636539	0.0184885	0.037805613
ZC3HAV1	4501	3557	0.075567541	0.0185193	0.037862346
IFI6	458	403	0.230584372	0.0185405	0.037899587
GNE	1832	1262	-0.122555461	0.0185509	0.037914515
POLR2G	1630	1117	-0.130090647	0.0185572	0.037921264
DHX16	2344	1631	-0.108063656	0.0185628	0.037926508
C4orf46	1349	916	-0.143318713	0.0185647	0.037924133
ZNF646	1356	921	-0.142931985	0.0185703	0.037929235
TBPL1	986	658	-0.168347931	0.0185776	0.037938121
ECHS1	4812	3426	-0.074959359	0.0186084	0.037994614
EFCAB11	173	97	-0.419563254	0.0186414	0.038055746
ZFP37	207	196	0.336375018	0.0186743	0.038116733
TBX3	5740	4105	-0.068516383	0.0186761	0.038114205
DOHH	483	306	-0.243339404	0.0187423	0.038243036
GP1BA	64	29	-0.726866873	0.018751	0.038254512
LOC729683	145	79	-0.46097621	0.0187979	0.038343872
ANKRD23	94	47	-0.584847868	0.0188192	0.038381149
LOC100507373	72	79	0.549007878	0.0188277	0.03839205
HSPD1	22887	17569	0.033655841	0.0188483	0.038427885

PCBP2	18001	13145	-0.03841078	0.0189031	0.03853331
CCND3	4432	3149	-0.077911992	0.0189656	0.038654308
MEPCE	1926	1330	-0.119029326	0.018973	0.038663105
TSHZ3	2306	1604	-0.108566239	0.0189919	0.038695258
SMURF1	3661	2587	-0.085805588	0.0190168	0.038739635
POC1A	545	349	-0.227877062	0.0190503	0.038801531
ITPK1	824	544	-0.183885554	0.0190712	0.038837776
CDC23	1561	1068	-0.132406759	0.0190774	0.038844007
TRAPPC10	3130	2201	-0.09285138	0.0190961	0.038875697
IL8	69	32	-0.693372325	0.0192001	0.039081133
LINC00085	211	122	-0.375209719	0.0192087	0.039092281
UAP1L1	940	626	-0.171345968	0.0193847	0.039443957
SP140	166	161	0.371029579	0.0194295	0.039528707
XRCC2	286	172	-0.31845445	0.0194633	0.03959102
C17orf96	79	38	-0.640701103	0.0194671	0.039592314
BLVRB	1334	906	-0.143023579	0.019474	0.039599716
MRPL11	1067	716	-0.160376552	0.0194935	0.03963287
PPFIBP2	278	255	0.290564496	0.0195003	0.039640331
CTF1	379	235	-0.27438496	0.0195288	0.039691779
E2F5	152	149	0.386393139	0.0195291	0.039685833
FAM83G	608	393	-0.214389879	0.019531	0.039683289
NAB1	1582	1294	0.125240149	0.0195998	0.039816665
CYBRD1	7968	6214	0.056456726	0.0196295	0.039870393
CHUK	2381	1917	0.102434848	0.0196653	0.039936547
TAF5L	1637	1123	-0.128544262	0.019704	0.040008655
RARRES1	97	49	-0.570050866	0.019707	0.040008207
FAT1	95935	72766	0.016359388	0.019707	0.0400017
WDR13	1510	1032	-0.133953447	0.0197159	0.040013345
DDX11	648	421	-0.207021448	0.0197561	0.040088334
TPRG1L	3765	2988	0.081682415	0.0197704	0.04011092
ADAMTS7	1384	942	-0.139892846	0.0198651	0.040296364
ALDOC	829	548	-0.182044077	0.019889	0.04033837

CIDEB	839	708	0.170230681	0.0198956	0.040345187
GTF2H3	2191	1769	0.106496695	0.0199104	0.040368648
NUAK2	615	529	0.197833444	0.0199256	0.040392765
ABCE1	6879	5380	0.060559449	0.0199531	0.040441865
PBK	1769	1218	-0.123267783	0.0199782	0.040486263
IFNAR2	868	731	0.167328495	0.0199832	0.040489791
TRAF3IP1	1423	1169	0.1314914	0.0199902	0.040497398
BOC	367	227	-0.277935634	0.0200016	0.040513901
SLC11A2	2643	2120	0.097039972	0.0200153	0.040535104
PUS1	576	371	-0.219497493	0.0200696	0.040638326
SIX1	599	387	-0.215070305	0.0200735	0.040639643
RBM8A	3211	2261	-0.090909541	0.0200761	0.040638428
ZNF502	253	234	0.302523277	0.0200801	0.040639869
BAG3	5021	3581	-0.072460075	0.0200875	0.040648197
ZNF134	1046	872	0.15266932	0.0201018	0.04067046
TAF9B	2140	1729	0.107479204	0.0202116	0.04088596
RECQL4	672	438	-0.202378231	0.0202141	0.040884443
CDR2	1898	1311	-0.118660175	0.0202399	0.040930091
RPAP2	935	784	0.161039421	0.0202599	0.04096372
PCNT	3146	2214	-0.091711317	0.0202738	0.040985223
NBEA	197	187	0.339994772	0.0203084	0.041048475
C15orf41	930	780	0.16139554	0.0203113	0.041047665
LNX2	830	549	-0.181153055	0.0204063	0.04123295
FAM108C1	1512	1034	-0.133069822	0.0204375	0.041289362
CAST	15356	11844	0.040506062	0.0204587	0.041325531
BAIAP3	87	43	-0.601526609	0.020467	0.041335589
TUBGCP4	1028	689	-0.162112245	0.0205045	0.041404555
MROH1	1239	839	-0.14728134	0.020508	0.041404874
PKNOX1	1853	1505	0.115052737	0.0205379	0.041458572
GLTSCR1	716	469	-0.195219533	0.0205992	0.041575648
LIAS	366	327	0.252599119	0.0206304	0.041631815
PYCR1	3199	2253	-0.090621547	0.0207041	0.041773777

GATC	1729	1408	0.118861597	0.0207158	0.041790572
HOXA2	108	56	-0.532380448	0.0207226	0.041794144
SCARNA9	108	56	-0.532380448	0.0207226	0.041794144
KCNJ14	72	34	-0.667310028	0.0207503	0.041839922
PODN	733	481	-0.192624173	0.0207517	0.041835969
NOMO3	186	106	-0.396086225	0.0207608	0.041847425
ANKRD49	515	448	0.214078432	0.0208043	0.041928492
SCD5	1692	1379	0.12004502	0.0208054	0.041923931
KBTBD6	1825	1259	-0.120466049	0.0208491	0.042005097
CTNNAL1	6559	4709	-0.062903021	0.0208649	0.042030185
TANGO2	1219	825	-0.14807997	0.0208722	0.042038095
PUM1	5819	4566	0.065311745	0.0208731	0.042033017
UNC119	875	581	-0.175592722	0.0208898	0.042059834
DGKA	3421	2721	0.084870984	0.0209079	0.042089438
YLPM1	6641	5196	0.061153064	0.020929	0.042125261
HTATSF1	4403	3478	0.07492322	0.0209342	0.042128869
DDX46	6755	4853	-0.061926778	0.0209607	0.0421753
MED7	607	522	0.197505422	0.0209663	0.042179792
ASB16	112	114	0.440687224	0.0210061	0.042253128
MNF1	1195	808	-0.149431288	0.0210122	0.042258515
SP1	5401	4245	0.067690137	0.0210135	0.042254206
MED6	995	831	0.155304083	0.0210411	0.042303056
TMSB15B	192	110	-0.388450655	0.021051	0.042316064
ZSCAN16	95	48	-0.569740976	0.021078	0.042363448
SOGA3	384	239	-0.268943561	0.0210999	0.042400744
UCK1	947	632	-0.168287736	0.0211064	0.042406943
PHF6	3661	2906	0.081949061	0.021118	0.042423435
SPATS2L	16892	13010	0.038432942	0.0211233	0.042427065
PLCB1	2566	2059	0.097574791	0.0211794	0.04253306
DDB1	20077	15429	0.03525298	0.02119	0.042547355
SYNE3	657	428	-0.203130442	0.0211935	0.042547595
NKAP	1066	887	0.149950703	0.0212401	0.042634214

TBC1D1	4964	3909	0.070448601	0.0212416	0.042630364
IRF2BP1	985	659	-0.164693128	0.021274	0.042688467
PCDHGC4	119	63	-0.502385708	0.0212741	0.042681758
ZNF436	1236	1021	0.139456255	0.0212905	0.042707799
ZNF777	852	565	-0.177450431	0.0212911	0.042702179
PAX6	54	62	0.61446094	0.0212945	0.042702225
ATAD3B	555	357	-0.221411565	0.0213687	0.042844045
TRMT5	1511	1034	-0.132115343	0.0213798	0.042859425
SLC3A2	3477	2456	-0.086380375	0.0214151	0.04292332
SMC6	2960	2364	0.090784991	0.0214229	0.042931948
PI4KAP2	328	201	-0.291348182	0.0214471	0.042973623
ENSA	4491	3545	0.073901037	0.0214534	0.042979345
REXO2	4634	3655	0.072765494	0.0214545	0.04297448
TEX10	2203	1532	-0.108901066	0.0214686	0.042995898
CD99L2	5648	4042	-0.067518635	0.0214805	0.043012761
COX17	711	605	0.182237714	0.0214872	0.043019216
NFATC3	1767	1437	0.116910154	0.0214915	0.043020914
DISP1	461	292	-0.24364625	0.0214917	0.043014562
POLR3A	3523	2799	0.083259144	0.0215237	0.043071523
NRGN	785	518	-0.184588424	0.0216499	0.043317191
PNISR	5444	3893	-0.06863259	0.0217721	0.043554655
TFCP2	1752	1425	0.117111276	0.0218206	0.043644763
FAM229B	670	572	0.187006183	0.0218866	0.043769704
LINC00707	133	72	-0.470205302	0.0219301	0.043849572
RCOR2	161	90	-0.42391165	0.0219426	0.043863997
SLITRK5	161	90	-0.42391165	0.0219426	0.043863997
RBM26-AS1	32	41	0.772704136	0.0219566	0.043881578
SGSM2	2122	1474	-0.110536	0.0220097	0.043980499
ERP29	4149	3281	0.076524068	0.0220156	0.043985361
CIC	3811	2700	-0.082058069	0.0220358	0.044018534
DGCR11	310	189	-0.298729849	0.0220364	0.04401276
CCDC57	346	310	0.256648309	0.0221241	0.04418074

HN1	7931	5719	-0.056587746	0.0221338	0.044193162
CCDC77	478	304	-0.237787163	0.0221805	0.044279212
FBF1	449	284	-0.245672383	0.0221855	0.044282164
RASIP1	164	92	-0.418837917	0.0221993	0.044302519
ZBTB33	1954	1353	-0.115116496	0.0222003	0.044297473
ZNF57	125	125	0.415152132	0.0222121	0.044313856
ADNP	6004	4304	-0.065093767	0.0222183	0.04431914
XYLB	398	249	-0.261470557	0.0222792	0.04443348
AGPAT6	5847	4189	-0.065938575	0.0222854	0.044438731
IFT172	1212	821	-0.14678344	0.0223722	0.044604794
SPTBN4	125	67	-0.484542962	0.0223922	0.044637396
FANCG	629	409	-0.205807042	0.0223992	0.044644263
HMGN5	242	224	0.303643817	0.0224533	0.04474496
CISH	101	104	0.457380367	0.0225355	0.044901462
ARRDC1	472	300	-0.238672227	0.0225501	0.044923467
C11orf74	474	414	0.21989584	0.0225985	0.045012738
ZNF19	74	80	0.527626861	0.0226004	0.045009363
KXD1	3821	2708	-0.081570387	0.0226481	0.045097008
TMEM8B	824	546	-0.178591255	0.0226828	0.04515895
TMEM55A	1464	1199	0.127068237	0.0226859	0.045157856
IL12A	199	115	-0.375982438	0.0227101	0.045198844
SMIM14	1625	1325	0.120704773	0.0227279	0.045227088
TTLL1	469	298	-0.23912346	0.0227366	0.04523708
PION	124	124	0.415152132	0.022753	0.045262562
CHMP2A	2984	2099	-0.092393237	0.0227729	0.045294778
PTPRE	63	70	0.567155225	0.0227864	0.04531458
COA3	1140	770	-0.150951342	0.0228245	0.045382979
IRAK3	280	169	-0.313251449	0.0228355	0.045394018
PINX1	280	169	-0.313251449	0.0228355	0.045394018
PCDHGA2	453	287	-0.243308182	0.0228361	0.045384293
MYO1C	18262	13349	-0.036960971	0.0228624	0.045429328
PCDHGA8	229	213	0.310657964	0.0229354	0.04556708

PSMA2	4868	3473	-0.071994626	0.0229364	0.045561786
SERPING1	556	480	0.203101655	0.0229487	0.045579
RFXAP	241	223	0.303162695	0.0229605	0.045595254
DENR	6255	4897	0.062040443	0.0229655	0.045597884
MOSPD3	518	332	-0.226616725	0.0229909	0.045640994
MKL1	1893	1534	0.111776204	0.0230096	0.045670759
MRI1	1196	810	-0.147071445	0.0230141	0.045672548
ABLIM1	1843	1495	0.113241545	0.0230241	0.045685079
ZNF441	815	687	0.168662171	0.0230259	0.045681332
CPSF3L	2100	1459	-0.110257313	0.0230465	0.04571491
PCDHB10	100	103	0.457796469	0.0230788	0.045771778
SHPRH	878	737	0.162595811	0.0230878	0.045782299
TMEM168	2222	1790	0.103252903	0.0231012	0.045801608
PALB2	818	542	-0.17865586	0.0231337	0.045858694
SLX4	999	670	-0.161171451	0.0231357	0.045855457
ATP6V0B	2581	1807	-0.099181503	0.0231593	0.0458948
EBP	764	504	-0.184996773	0.0231845	0.04593752
HIPK1	4922	3874	0.069731428	0.0231917	0.04594452
NSUN5	515	330	-0.226954276	0.0231932	0.045940168
ATP5F1	8978	6979	0.051778367	0.0232218	0.045989454
MC1R	336	207	-0.283678334	0.02323	0.045998301
PITRM1	5575	3991	-0.067069394	0.0232381	0.04600706
MFSD2A	253	151	-0.329436704	0.0232641	0.046051282
TATDN1	785	663	0.171468348	0.0232686	0.046052765
MMACHC	264	242	0.28962125	0.0232993	0.046106352
USP36	2234	1556	-0.106634994	0.0233007	0.046101772
CA5BP1	229	135	-0.347236059	0.0233052	0.046103342
PDGFB	49	57	0.633332302	0.0233111	0.046107575
PIBF1	964	805	0.155107769	0.0233249	0.046127634
PRTG	165	159	0.361712873	0.0233316	0.046133585
TMPO	4602	3279	-0.07385295	0.0233399	0.046142637
EED	775	655	0.172450728	0.0233414	0.046138283

TRMT112	2274	1585	-0.105597282	0.0234011	0.04624891
LIME1	131	71	-0.46852375	0.0234019	0.046243172
SRBD1	1002	835	0.152117726	0.0234152	0.046262068
PTPRG	3465	2752	0.082777249	0.0234339	0.046291774
CCDC71	704	462	-0.192530445	0.0235005	0.046415894
HIVEP3	374	233	-0.267556183	0.0235269	0.046460715
MALSU1	938	627	-0.165970348	0.0235498	0.046498533
GRB10	1267	861	-0.14217925	0.0235868	0.046564303
MORC3	2777	2220	0.092184631	0.0236296	0.04664139
KIAA0101	1341	914	-0.137891035	0.0237963	0.04696296
NFKBID	134	73	-0.4611125	0.0238677	0.047096366
COX15	2545	2040	0.096055628	0.0238841	0.0471213
DUSP6	4918	3870	0.069413963	0.0238907	0.047126802
PDIA5	1213	1001	0.138014555	0.0240006	0.047336183
ME3	280	255	0.280222552	0.024025	0.04737678
VEZT	4216	3330	0.074799442	0.0240822	0.047482057
IFI30	168	95	-0.407309683	0.0241048	0.047519123
AP2A2	5441	3894	-0.067466813	0.0241218	0.047544999
PRKCZ	200	116	-0.370723063	0.02419	0.047671936
RWDD1	3321	2640	0.084072338	0.0242019	0.047687826
SAMHD1	1077	726	-0.153824665	0.024224	0.04772373
PHLDA3	2960	2083	-0.091782205	0.0242411	0.047749946
NDUFA9	2982	2099	-0.091425959	0.0242588	0.047777234
KCNG1	759	501	-0.18413715	0.0242743	0.047800313
GPR135	137	75	-0.454061261	0.0243084	0.047859748
CCDC12	592	384	-0.209338733	0.0243232	0.047881356
TBL1X	3360	2374	-0.085989166	0.0243568	0.04793994
MICALL1	2955	2357	0.08894576	0.0243716	0.047961505
TP53TG1	602	391	-0.207442748	0.0243793	0.047969149
FAHD1	2032	1411	-0.111030282	0.0243897	0.047981906
POLR2J3	99	51	-0.541779146	0.0244516	0.048096168
HSD11B1L	212	124	-0.358572012	0.024487	0.048158081

PREX1	303	185	-0.296640391	0.0245303	0.048235683
SHISA3	254	152	-0.325605042	0.0245381	0.048243382
COA5	638	545	0.187851938	0.0246168	0.048390539
GAS1	86	43	-0.584847868	0.0246459	0.048440043
CPZ	297	181	-0.299321102	0.0247412	0.048619628
RPS6KA6	1280	871	-0.140247055	0.0247548	0.048638838
KAL1	59	66	0.576903202	0.0247561	0.048633606
STXBP3	3664	2904	0.079774082	0.0247847	0.048682208
ASUN	2058	1661	0.105961223	0.0247868	0.048678502
ZC3H8	290	263	0.274162031	0.024791	0.048679123
ATAD2	3134	2210	-0.088806678	0.0247962	0.048681676
LY96	469	409	0.217665052	0.0248183	0.048717383
KLHL8	1415	1159	0.127230645	0.0248871	0.048844775
VRK2	1449	992	-0.131493437	0.0248881	0.048839044
POLR1D	2425	1946	0.097679095	0.0248978	0.048850313
GSTO1	8051	6268	0.053989323	0.0249606	0.048965798
SH3D21	255	234	0.291163414	0.0249641	0.048965024
DOK5	79	84	0.503688806	0.0251105	0.049244327
RPAP3	2263	1820	0.100853998	0.0251934	0.049399141
ARAF	2330	1627	-0.102963572	0.0251977	0.049399949
ANAPC13	4535	3574	0.071589215	0.0252242	0.049443972
TRMT44	334	299	0.255449513	0.025271	0.049528046
ZNF776	1134	938	0.141391319	0.0252809	0.049539541
FGD5-AS1	5660	4056	-0.065592269	0.0252899	0.049549509
LOC143666	66	31	-0.675045677	0.0253051	0.049567561
YPEL4	66	31	-0.675045677	0.0253051	0.049567561
CCDC144A	148	144	0.375623768	0.0253557	0.049654852
PRR7	310	190	-0.291116665	0.0254613	0.049853889
LRRC27	339	303	0.253184652	0.0255451	0.050010172
ATF1	1297	1066	0.13218109	0.0255452	0.050002389
CDK5RAP2	3368	2381	-0.085172386	0.0255465	0.049997036
GLYCTK	195	113	-0.37199922	0.0255548	0.05000552

STAMBP	1906	1542	0.109406778	0.0256283	0.050141469
RPL15	51226	38991	0.021417045	0.025632	0.050140734
GTF2H4	715	606	0.176526684	0.025661	0.0501897
TMX2	3498	2775	0.081109614	0.025712	0.050281568
ITPR2	1578	1286	0.119945569	0.0257223	0.050293768
NRF1	492	315	-0.228154355	0.0257321	0.050304928
LOC644656	43	51	0.661312719	0.0257773	0.050385573
TMEM126A	1123	929	0.141544706	0.0258351	0.050490586
ERAP2	288	261	0.273133127	0.0258739	0.050558523
UCP2	199	187	0.325421971	0.0259638	0.050726209
TMEM25	884	590	-0.168179283	0.0259705	0.050731224
STX18	1632	1328	0.117766221	0.0260029	0.050786636
LOC401320	240	143	-0.331867127	0.0260564	0.050883029
ELP6	799	530	-0.177051012	0.0260717	0.050905045
ABHD16B	234	139	-0.336271515	0.0260919	0.050936446
CDK5R1	124	67	-0.472954988	0.0261106	0.050964903
LANCL2	1202	991	0.136672198	0.0261155	0.050966516
CNNM2	937	782	0.154271691	0.0261682	0.051061405
ZNF236	1098	742	-0.150234831	0.0261683	0.051053678
SOD3	486	311	-0.228889602	0.0261854	0.051078975
ABCC9	1255	854	-0.140227257	0.0262248	0.051147831
CERS6	545	352	-0.215528669	0.0262666	0.051221292
VPS41	4603	3625	0.070558684	0.0262957	0.05126999
TMPRSS9	56	63	0.585077133	0.0263172	0.051303986
ZFAND2B	668	438	-0.193765101	0.0264055	0.051467957
ANAPC7	2759	1939	-0.093680521	0.0264394	0.051526036
PRKY	1375	940	-0.133546825	0.026525	0.051684761
PQBP1	1354	925	-0.134550336	0.026583	0.051789566
PDZD8	2128	1482	-0.106800571	0.026598	0.051810739
ZNF17	348	310	0.248333041	0.0266035	0.051813453
GNAI1	3095	2463	0.08562935	0.0266398	0.051875882
PIF1	467	298	-0.232958087	0.0266414	0.051870906

INSIG1	1319	900	-0.136295526	0.0266603	0.051899576
SMAD1	522	451	0.204229758	0.0266692	0.051908881
PTDSS2	1891	1311	-0.113329546	0.0267247	0.052008719
TMEM219	2042	1420	-0.108939805	0.0267749	0.052098286
DGAT1	899	601	-0.165803993	0.0267832	0.052106406
ZNF205	308	189	-0.289391985	0.026789	0.052109398
PHF1	2125	1480	-0.106713534	0.0268266	0.052174479
TRIM45	271	164	-0.309444905	0.0268602	0.052231653
DDX55	1089	736	-0.150074151	0.0269484	0.052394921
CSRNP3	40	48	0.678186538	0.0269878	0.0524634
ATG3	2701	2158	0.091353357	0.0270034	0.052485514
SUCLA2	2935	2339	0.08768349	0.0270175	0.052504774
IDH3B	2122	1478	-0.106626255	0.0270572	0.052573642
APBB1IP	314	282	0.260082735	0.0270819	0.052613538
THAP9-AS1	1919	1551	0.107996107	0.0271064	0.052652914
ASPSCR1	787	522	-0.177161697	0.0271255	0.052681752
KIF18A	1546	1063	-0.125246591	0.0271525	0.052725963
ZNF77	144	80	-0.432844775	0.0271568	0.052726048
MXD3	259	156	-0.316253937	0.0271754	0.052754088
FLJ39051	69	33	-0.648978206	0.0272009	0.052795268
FAM8A1	3198	2542	0.083946223	0.0272159	0.052816164
DNAH10	145	141	0.374794394	0.0272471	0.052868538
SAE1	7020	5057	-0.05803712	0.0272996	0.052962009
NUDT17	52	23	-0.76172563	0.0273213	0.052995918
LOC283070	54	61	0.591001967	0.0273964	0.053133409
PARN	2235	1560	-0.103576671	0.0274493	0.053227596
CLCN2	208	122	-0.354550249	0.0274804	0.05327964
NTNG2	167	95	-0.398696552	0.0274915	0.053292948
KIAA1551	1184	804	-0.143249543	0.0274927	0.053286816
FJX1	1764	1220	-0.116817281	0.0275002	0.053293082
RPL13P5	147	82	-0.426968208	0.027518	0.053319393
PSMD7	5556	4355	0.063778251	0.0275339	0.053341809

WWP1	2446	1960	0.095581382	0.0275597	0.053383475
BBIP1	468	299	-0.231210914	0.0275857	0.053425556
HEXA	3482	2466	-0.082591271	0.0276162	0.053476363
NHSL2	87	44	-0.568359745	0.0276296	0.053494002
RAB40C	710	468	-0.186158363	0.0276673	0.053558562
PKD2	6154	4813	0.060564111	0.0277124	0.053637578
FAM177A1	4172	3292	0.07337731	0.0277499	0.053701747
STARD3	1512	1039	-0.126110354	0.0278333	0.053854912
ISYNA1	356	222	-0.266165433	0.0279035	0.053982321
BET1L	3424	2424	-0.083140871	0.0279065	0.053979799
PLK1S1	580	377	-0.206336245	0.0279336	0.054023838
PPP1R3E	176	101	-0.386068004	0.0280771	0.054292835
ZNF790	220	204	0.30621776	0.0281054	0.054339228
GRAMD1C	640	545	0.183336457	0.0281593	0.054434979
ANK3	153	86	-0.415970956	0.028172	0.054450933
SEC23B	3378	2680	0.081215804	0.0281745	0.054447423
ANKS6	1119	758	-0.146788151	0.0281867	0.054462546
DBR1	797	670	0.164733503	0.0282417	0.054560374
PCDHB12	83	87	0.483056196	0.0282495	0.054566933
DNAJC25	965	803	0.150023177	0.0283128	0.054680652
GDF11	2444	1712	-0.098409452	0.028322	0.054689934
CCDC134	189	178	0.328643138	0.0283228	0.054683013
PGS1	874	584	-0.166512779	0.0283278	0.054684208
BATF3	57	26	-0.717298164	0.0283351	0.05468569
C3orf70	57	26	-0.717298164	0.0283351	0.05468569
TMEM65	1478	1206	0.12173577	0.0284196	0.054835931
MAPKBP1	1784	1235	-0.115452442	0.0284212	0.054830485
RNF139	1797	1455	0.110580876	0.0284705	0.054917137
WARS	8333	6478	0.05186462	0.0284971	0.054959897
DOCK10	3656	2894	0.077950983	0.0284986	0.054954347
AFAP1-AS1	36	44	0.704658749	0.0285154	0.054978246
DPY30	1111	918	0.139859374	0.0285454	0.055027621

FLJ38109	569	488	0.193604627	0.028558	0.055043363
BUB1B	1581	1089	-0.122681282	0.0285678	0.055053639
BCAR3	2108	1469	-0.105888339	0.0286392	0.055182648
ATRN	6804	4900	-0.05844926	0.028651	0.055196954
FBXL16	599	512	0.188739939	0.0286535	0.05519326
NEK10	257	155	-0.314348012	0.0286766	0.055229127
DTWD2	266	242	0.278732934	0.0287185	0.055301287
EIF4EBP1	1316	899	-0.134614336	0.0287233	0.055302086
NUFIP1	861	575	-0.16729915	0.0287916	0.055424945
MPV17L2	641	420	-0.194782897	0.0288228	0.055476433
MTMR2	3475	2462	-0.082030084	0.0288346	0.055490485
RAB12	2214	1779	0.09956342	0.0288715	0.055553053
ZBTB22	810	539	-0.172484503	0.0289366	0.055669563
LRRC37A2	162	92	-0.401135915	0.0289934	0.055770385
FAM69B	316	195	-0.281298303	0.0290005	0.055775359
TMEM154	450	287	-0.233722133	0.0290462	0.055854662
SCN8A	2733	2181	0.089656442	0.0290676	0.055887233
ZHX3	6192	4451	-0.061123839	0.0291079	0.055956062
RMDN3	1868	1296	-0.112276605	0.029114	0.055958999
GCC2	4410	3145	-0.072566507	0.0291225	0.055966771
MITD1	608	519	0.186815347	0.0291545	0.056019695
DHPS	1187	807	-0.141527225	0.0291572	0.056016171
KLHL3	316	283	0.256029626	0.0291631	0.056018801
CPXM1	221	131	-0.339327426	0.0292093	0.056098902
UXT	702	594	0.174144032	0.0292439	0.056156672
DESI2	5290	4149	0.064648067	0.0292492	0.056158191
TMEM62	381	336	0.233822367	0.0293328	0.056310048
KIF2C	1686	1165	-0.11812245	0.0293584	0.056350499
SLC35E4	418	265	-0.242358451	0.0293844	0.056391849
TFB1M	780	518	-0.175369894	0.0293848	0.056383937
MIOS	1151	949	0.136744291	0.0294089	0.056421461
ZNF14	282	255	0.269954216	0.029413	0.056420638

CEP41	1170	795	-0.142329633	0.0294319	0.056448074
SLC19A2	552	474	0.195370924	0.0294866	0.05654442
CDPF1	276	168	-0.301054902	0.0295205	0.056600612
ZNF629	2834	1996	-0.090575906	0.0295343	0.056618347
C4orf52	845	564	-0.168104047	0.0295526	0.0566448
LOC202181	348	217	-0.266240132	0.0296768	0.056874
MINPP1	2474	1980	0.093807062	0.029685	0.056881053
LEO1	2014	1623	0.103751448	0.0297471	0.056991238
ARHGEF10	3568	2825	0.078287384	0.0297581	0.057003608
ACER3	774	651	0.165476109	0.0298519	0.057174519
GRHPR	3652	2592	-0.079468915	0.0298766	0.057212972
PLEKHB2	10806	7850	-0.045915896	0.0299188	0.057284972
CBR4	923	769	0.151805082	0.0299451	0.057326566
DAZAP1	4354	3105	-0.072596007	0.0300981	0.057610634
USP32P2	61	67	0.550503985	0.0301196	0.057642758
MGC27345	500	322	-0.219715275	0.030136	0.057665309
NCOA4	14613	11258	0.038850296	0.0301591	0.057700728
ZFP106	14392	11090	0.039144406	0.0301639	0.057700995
IMPG2	80	40	-0.584847868	0.0302264	0.057811734
DANCR	683	450	-0.186808445	0.0302352	0.057819693
PPP1R21	1344	1100	0.126122517	0.0302584	0.057855152
PRPF39	1787	1446	0.10968005	0.0302728	0.057873812
PRX	252	152	-0.314200278	0.0303453	0.058003492
ZNF554	186	175	0.327204432	0.0303685	0.058038882
THUMPD2	489	423	0.20597533	0.0304556	0.058196437
FAM168B	10654	8245	0.045348345	0.0305307	0.058330993
SPHK2	680	448	-0.186883882	0.0305354	0.058330971
SWAP70	4432	3489	0.070007848	0.0305469	0.058343976
EFCAB2	147	142	0.365226906	0.030557	0.05835435
ZNF526	991	668	-0.153884823	0.0305771	0.058383874
RAB3A	387	244	-0.250300287	0.030644	0.058502567
WHAMMP2	140	136	0.373331956	0.0307306	0.058659003

DTX2P1-UPK3E	75	37	-0.604213193	0.0307331	0.058654693
TRMT61A	560	364	-0.206336245	0.0307739	0.058723633
PPP4R4	201	118	-0.35325651	0.0307892	0.058743771
FRRS1	133	130	0.382237509	0.0308191	0.05879192
SLC27A1	981	661	-0.154450733	0.0308273	0.058798457
WASH7P	204	120	-0.350382615	0.0308332	0.058800767
EREG	104	55	-0.503927873	0.0308442	0.058812655
DNAJC18	1232	840	-0.137388891	0.0308591	0.058832054
CDO1	60	28	-0.684383542	0.0308792	0.058856831
NLRC4	60	28	-0.684383542	0.0308792	0.058856831
OLA1	5029	3947	0.065637098	0.0308863	0.058856966
MRPL14	1047	708	-0.149288045	0.0309189	0.058909957
MCOLN1	926	622	-0.158945481	0.0309853	0.059027593
ABHD2	8563	6650	0.050390147	0.031027	0.05909783
GNG12-AS1	70	34	-0.626668044	0.0310512	0.059130523
LOC100506472	70	34	-0.626668044	0.0310512	0.059130523
SH3YL1	930	774	0.150254982	0.0310679	0.059148673
MLLT1	3856	2742	-0.076724349	0.0311217	0.059241987
DGKE	947	637	-0.156918921	0.0311989	0.0593799
RBMXL1	861	576	-0.164792294	0.0312007	0.059374315
PCDHGB4	737	621	0.168080781	0.0312534	0.059465581
SETDB2	1268	866	-0.134963684	0.0313624	0.059663829
C3orf17	2707	2159	0.088820488	0.0314292	0.05978171
NUP214	3332	2360	-0.082449409	0.0314701	0.059850359
SLC25A33	482	417	0.206166369	0.0315052	0.059908008
SNW1	3417	2422	-0.081379251	0.0315182	0.059923521
PWP1	4004	3159	0.073178095	0.0315468	0.05996876
EFNB3	146	82	-0.417120423	0.0315883	0.060038441
FTSJD1	1730	1198	-0.114991998	0.0315895	0.060031679
HK2	2909	2315	0.085641029	0.0316021	0.060046423
GCNT1	430	375	0.217706068	0.0316103	0.060052835
SLC7A5	3534	2797	0.077730346	0.031768	0.060343238

ANKRD13D	879	589	-0.1624434	0.031813	0.06041939
ZNF816	295	265	0.260429537	0.0319206	0.060614577
PLRG1	3289	2608	0.080446993	0.0319567	0.060673893
GJA3	132	73	-0.439417429	0.0319627	0.060676015
SSTR1	1210	994	0.131462841	0.0319629	0.060667218
KIAA1967	4719	3374	-0.068869066	0.032085	0.060889684
UST	1294	885	-0.132936125	0.0321544	0.061011961
SERPINB8	1828	1269	-0.111421869	0.0321664	0.061025448
FKBP1A	18232	13347	-0.034805194	0.0321909	0.061062754
COPG2	607	517	0.183619896	0.0322637	0.06119144
MPP2	333	296	0.24522713	0.0322988	0.061248669
DCTN3	2858	2275	0.086012761	0.0323655	0.061365892
TERF2	1196	815	-0.138193293	0.0324143	0.061448972
EVI2A	77	81	0.488215594	0.0325572	0.061710618
LOC646762	45	52	0.623738754	0.0325769	0.061738401
TSPAN11	223	133	-0.330465333	0.0326333	0.061835991
APBA2	2237	1565	-0.100250467	0.0326434	0.061845683
KLHL18	1550	1069	-0.120854231	0.0326615	0.061870551
C22orf39	546	468	0.19275971	0.0327486	0.062026127
CCDC126	421	268	-0.236435101	0.0327729	0.062062742
GLOD4	1995	1390	-0.106151732	0.0328304	0.062162206
IVD	3336	2364	-0.081737122	0.0328397	0.062170386
FAM21A	73	36	-0.604747426	0.0329085	0.062291172
DDX11L2	86	44	-0.551681004	0.0330313	0.062514108
ZNF740	1292	884	-0.132335664	0.0330577	0.062554478
ANKLE2	5900	4241	-0.06115834	0.0330781	0.062583534
CCDC25	3031	2142	-0.08568324	0.0330985	0.062612745
TSSC1	967	652	-0.153491793	0.0330994	0.062604922
HSD17B4	4861	3479	-0.067428323	0.0331399	0.062671864
NFATC2IP	2666	2126	0.088616948	0.0331493	0.062680158
FAM22G	94	49	-0.524726876	0.0331978	0.062762321
ZNF275	2102	1689	0.099558791	0.0332424	0.062837172

LOC439994	164	94	-0.387811021	0.033259	0.062858917
STARD8	141	79	-0.420618472	0.0332643	0.062859553
DLX1	63	30	-0.655237196	0.0332993	0.062916114
DNASE2	1587	1096	-0.118902198	0.0333891	0.063076169
SDSL	257	156	-0.305070199	0.0334412	0.06316502
LAMP1	20363	15617	0.032319349	0.0334432	0.06315913
BRD3	1881	1308	-0.108985176	0.0334579	0.063177314
ARSK	2550	2036	0.090392446	0.0335196	0.063284242
NUP133	3292	2609	0.07968474	0.0335603	0.063351476
DAP3	3918	3091	0.073108388	0.0336002	0.063417276
ZNF486	331	294	0.24413707	0.0336395	0.063481785
APOO	411	359	0.219997582	0.0336498	0.063491625
ZNF407	1392	956	-0.126924556	0.0336818	0.06354237
OARD1	649	550	0.176365272	0.033685	0.063538733
RNF216	4523	3232	-0.069700666	0.0337208	0.063596718
GTPBP8	698	589	0.170192729	0.0337386	0.063620616
ZNF83	2185	1753	0.097344848	0.033864	0.063847446
PDIA3P	364	229	-0.25343872	0.0338732	0.063855085
KIF17	363	320	0.233254489	0.0338784	0.063855302
SYPL2	100	101	0.429507425	0.0338966	0.063879949
B3GNT2	820	548	-0.166295885	0.0339041	0.063884299
USP54	544	466	0.191875435	0.0339104	0.063886514
MIAT	448	287	-0.227295864	0.0339658	0.063981234
GGA1	1983	1382	-0.10577493	0.0339801	0.063998429
SHISA9	236	142	-0.317743798	0.034174	0.064349156
ZBTB3	236	142	-0.317743798	0.034174	0.064349156
TFRC	11796	9109	0.042218991	0.0341814	0.064348355
SDHAF2	1241	848	-0.134214814	0.0341867	0.064348675
KDM3B	5183	3716	-0.06488476	0.0341945	0.064353604
MIPEP	999	827	0.142554783	0.0342253	0.064401861
ANTXR1	14604	10664	-0.038462757	0.0342476	0.064434205
SYTL2	3093	2188	-0.084241964	0.0342691	0.064464806

GCNT2	223	205	0.293732331	0.0343439	0.064595751
DHX38	3622	2574	-0.077622361	0.0345833	0.065036152
SUDS3	3083	2447	0.081831344	0.0346066	0.065070218
NR2C2AP	119	65	-0.457297819	0.0346151	0.065076416
ARL8B	6023	4333	-0.059963891	0.034719	0.065261954
ADAMTS13	108	58	-0.481754375	0.0347301	0.06527281
DPH3P1	99	100	0.429651701	0.0347356	0.065273294
PRPF38B	3505	2772	0.076664945	0.0349629	0.065690563
ENPP1	1994	1604	0.101160864	0.0350589	0.065861035
POLR2M	818	547	-0.165407878	0.0350627	0.065858268
THAP1	671	567	0.1721881	0.0351309	0.065976292
FHOD3	726	482	-0.17578427	0.0351745	0.06604831
BFSP1	122	67	-0.449496015	0.0352351	0.066152149
KLHL26	286	176	-0.285287586	0.0352694	0.066206517
TMEM14C	3998	3151	0.071683414	0.0353727	0.0663904
GHITM	9255	7172	0.04729463	0.0354373	0.066501631
RNF19B	636	539	0.176410639	0.0354657	0.066544857
KCNQ1OT1	3810	2712	-0.075281687	0.0354785	0.066558873
POLG2	327	204	-0.265569352	0.0354934	0.066576773
RGP1	860	577	-0.160613209	0.0357142	0.066980942
ARHGAP44	324	202	-0.266486388	0.0357367	0.067013059
CSGALNACT1	788	526	-0.167980698	0.0357409	0.06701082
CCDC85C	1947	1357	-0.105680032	0.0358061	0.067122953
ADCK4	602	395	-0.192758702	0.0359864	0.067450821
ZNRF1	1184	808	-0.136089751	0.0360051	0.067475747
PPAP2C	592	388	-0.194388392	0.0360308	0.067513641
PEX3	971	656	-0.150623349	0.0360453	0.067530771
PPP2R4	6851	4943	-0.055775547	0.0360532	0.067535299
THOC3	271	166	-0.291957478	0.0361897	0.067780866
KLHL7	2387	1908	0.092014737	0.0362388	0.067862602
BCAM	1757	1220	-0.111080912	0.0362586	0.06788943
RABL2B	407	355	0.217942362	0.0363146	0.067984078

DGKQ	1322	907	-0.128395589	0.0363292	0.068001249
C1orf56	128	71	-0.435100749	0.0363541	0.068032459
SLC39A4	128	71	-0.435100749	0.0363541	0.068032459
AADAT	579	379	-0.196213368	0.0364174	0.068135556
TMEM97	806	539	-0.165342434	0.0365001	0.06828
YPEL3	443	384	0.208951744	0.0365591	0.068380148
CCZ1	727	611	0.164369148	0.0365653	0.068381411
ATP8B4	95	50	-0.510847287	0.0366745	0.068575385
SYNGR2	1738	1404	0.107266985	0.0367337	0.068675853
AIMP2	408	260	-0.234905397	0.0367446	0.068685807
DHX58	87	45	-0.535938268	0.0367467	0.068679518
ANKMY2	1310	1070	0.123196117	0.0367603	0.068694636
PSMB8	1078	888	0.135426535	0.0368905	0.068927628
MTIF3	1196	817	-0.134657274	0.0369964	0.069115103
LOC400027	432	375	0.211011415	0.0370122	0.069134264
EFEMP2	13317	9716	-0.039682588	0.0370371	0.069170354
ZNF274	894	743	0.148239511	0.0370719	0.069225056
NEDD1	1890	1522	0.102734256	0.0371185	0.069301628
RNF220	1341	921	-0.126884044	0.0371657	0.069379347
CCDC122	306	190	-0.272380103	0.0371829	0.06940095
MKRN2	2904	2307	0.083128682	0.0371919	0.069407349
PSMC3	7636	5522	-0.052474083	0.0372169	0.069443614
TMEM150A	441	283	-0.224824471	0.0373551	0.069691016
TOPORS	2304	1843	0.093067486	0.0373951	0.069755345
ETFA	5664	4072	-0.060931572	0.0374878	0.069917746
SPOPL	2001	1608	0.099698371	0.037496	0.069922531
MRPS11	1067	725	-0.142355144	0.0375298	0.069975185
USP30	644	425	-0.184445715	0.0376676	0.070221581
SLC2A4RG	2947	2084	-0.084739651	0.0376771	0.070228717
ADAM33	1123	923	0.132196757	0.0376979	0.070256935
EXOSC3	1008	683	-0.146386023	0.0377628	0.070367326
MRPS22	1726	1394	0.106950228	0.0379655	0.070734483

SRA1	2096	1466	-0.100601482	0.0379747	0.070741158
SNF8	3121	2211	-0.0821572	0.0380257	0.070825549
SARS2	504	327	-0.208980966	0.0380372	0.070836339
VARS2	777	519	-0.167027928	0.0381099	0.070961162
SAMD5	140	79	-0.410350137	0.0381523	0.071029429
NAAA	544	355	-0.200635495	0.0382532	0.071206729
BCCIP	2772	1957	-0.08713137	0.0382909	0.071266243
ALKBH1	547	467	0.187033849	0.0383058	0.07128317
FAM160B2	2428	1707	-0.093153231	0.0383816	0.071413641
NUDT16L1	877	590	-0.156709756	0.0383888	0.071416271
DCLRE1B	501	325	-0.209218754	0.038402	0.071430167
ZNF273	157	149	0.339699903	0.0384757	0.071556615
ZNF30	212	195	0.294561991	0.0385192	0.071626776
ZNF565	214	128	-0.326314855	0.0386206	0.071804614
TMEM38B	1361	936	-0.1249345	0.0386529	0.071853952
LRCH1	1338	1091	0.120725117	0.0386966	0.071924531
RFC3	762	638	0.158917558	0.0386987	0.071917611
MCTS1	2592	2065	0.087228195	0.0387265	0.071958675
C17orf53	205	122	-0.33359063	0.0387552	0.072001282
CDK5RAP1	990	818	0.13982445	0.0387814	0.072039194
PLK4	919	620	-0.152644514	0.0388142	0.072089286
MIIP	452	291	-0.220151488	0.0388728	0.072187383
DFFB	358	226	-0.248484683	0.0389548	0.072328944
PIK3CA	2754	2190	0.084554442	0.0389766	0.072358566
COIL	1139	935	0.130422655	0.039003	0.072396768
ZNF703	1728	1395	0.106314036	0.0390319	0.072439677
ARFGEF1	5756	4496	0.058727575	0.0390427	0.072448808
SPC25	403	351	0.215843324	0.0391828	0.072697944
RGS10	1986	1387	-0.102745703	0.0391863	0.072693599
ZNF518B	2705	2152	0.085201616	0.0392196	0.072744534
SNRPB	4163	2973	-0.070551963	0.0392355	0.072763328
LDB2	1208	989	0.126574103	0.0392404	0.072761492

C2orf76	175	164	0.321493025	0.0392509	0.07277011
SNORA18	93	49	-0.509296835	0.0392517	0.072760785
AGA	1378	1122	0.11864892	0.0392791	0.072800802
MED4	930	628	-0.151314025	0.0393184	0.072862821
ROGDI	649	429	-0.182088699	0.0393332	0.072879264
FSD1L	408	355	0.214402004	0.0393369	0.072875343
BTN2A3P	248	151	-0.300639439	0.039359	0.072905445
MED28	1129	927	0.13074789	0.0393685	0.072912157
TUB	2032	1631	0.098008512	0.0393757	0.072914715
SLC36A4	823	552	-0.161072032	0.0393798	0.072911429
VEGFC	3717	2647	-0.074628362	0.0394715	0.073070394
KIAA1468	3451	2727	0.075452358	0.039499	0.073110412
DPM3	540	461	0.186959475	0.0396454	0.073370424
TMEM115	2731	1928	-0.087172131	0.0396579	0.073382654
AKT2	4905	3517	-0.06475567	0.0397695	0.073578388
TMCC1	870	723	0.148132378	0.039853	0.073721866
ZNF583	344	303	0.232061361	0.0399507	0.073891545
B4GALT3	1086	893	0.132860109	0.0399667	0.073910119
DRAM2	1477	1020	-0.118948542	0.0400626	0.074076526
C2orf16	161	93	-0.376605935	0.0401293	0.074188794
WDR18	1116	761	-0.137216537	0.0401484	0.074213211
AP4S1	333	294	0.23544611	0.0401862	0.074272006
CDAN1	1088	741	-0.138980977	0.0401951	0.074277443
TGIF2	579	492	0.180247099	0.0402134	0.074300153
GPRASP1	504	432	0.19275971	0.0402293	0.074318589
TRMT11	569	484	0.181730527	0.0402648	0.074373175
ZKSCAN3	448	387	0.203986966	0.0402962	0.074420056
MAPKAPK5	1034	852	0.135841282	0.0403308	0.074473005
TOR1B	2123	1701	0.095430902	0.0404066	0.074601837
FPGT	869	722	0.147794792	0.0404341	0.074641585
IST1	4047	2889	-0.071130513	0.0404384	0.074638429
TNFAIP1	3669	2894	0.072830149	0.0405326	0.074801259

SLC45A1	221	133	-0.317467992	0.0405682	0.074855867
PLEKHG4B	80	41	-0.549223959	0.0405883	0.074881917
DPY19L2	278	249	0.256212991	0.0406137	0.074917512
DCTN4	6120	4773	0.056516815	0.0406476	0.074969067
ANAPC10	289	258	0.251453705	0.0406482	0.074959057
GSTM2	1173	802	-0.13337674	0.0407111	0.075063874
NPTN	9783	7109	-0.045478175	0.040729	0.075085791
IGF2BP1	5852	4213	-0.058929722	0.0407668	0.075144283
MEIS3	1819	1267	-0.106576887	0.0407714	0.075141689
SLC46A3	135	130	0.360704348	0.0408185	0.075217348
SLC26A10	62	30	-0.632153583	0.0408279	0.075223539
WIBG	732	488	-0.169810369	0.0408284	0.075213417
MYPN	2113	1693	0.095441338	0.0408585	0.075257795
ACYP1	209	125	-0.326422716	0.0409002	0.075323397
ZNF787	811	544	-0.160943131	0.041	0.075495967
TLK1	3316	2622	0.07637581	0.0410225	0.075526349
INTS4	1244	853	-0.129216707	0.04103	0.07552896
FIP1L1	2634	2096	0.085535503	0.0410795	0.075608945
C7orf63	76	79	0.471005366	0.0410904	0.075617798
C14orf132	4157	2970	-0.069927685	0.0411304	0.075680219
EIF6	5037	3615	-0.063416952	0.041249	0.075887224
HSDL2	3883	3058	0.070568831	0.0412562	0.075889195
ADCY10P1	124	69	-0.430519722	0.041359	0.076067109
GOLGA8S	197	182	0.300894952	0.0413641	0.07606531
PAX8	46	52	0.592029894	0.0413717	0.076062293
UNC13A	46	52	0.592029894	0.0413717	0.076062293
EPS15L1	1748	1216	-0.108409824	0.0414136	0.076122549
C11orf54	477	309	-0.211230296	0.0414451	0.076169224
C1orf159	431	277	-0.222649761	0.0414517	0.076170143
BMPR1A	3027	2399	0.079696614	0.0415218	0.076287573
TPCN2	702	467	-0.172896349	0.0415272	0.076286391
PPM1H	57	62	0.536458428	0.0415738	0.076360601

ZNF217	4978	3572	-0.063682018	0.0416084	0.076412989
MARCH8	709	595	0.162256173	0.0416244	0.076431026
WDR25	577	379	-0.191221339	0.0416257	0.076422188
ZNF276	363	230	-0.243183555	0.0416954	0.07653879
CYBA	517	337	-0.202263557	0.0417633	0.076652264
RNASET2	1006	683	-0.14352069	0.0417758	0.076663903
ARHGEF19	271	167	-0.283292617	0.0417831	0.076666026
ASCC1	1020	693	-0.142489763	0.0418004	0.076686349
APOL1	134	129	0.360290197	0.0418213	0.076713466
PPP1R8	2654	2111	0.08491034	0.0418247	0.076708377
POMT2	1249	1020	0.122947807	0.0418495	0.076742493
KIAA1549	1391	1131	0.116628641	0.0418533	0.076738259
PCCA	699	465	-0.172909608	0.0419497	0.076903742
MYLK4	91	48	-0.507680008	0.0420175	0.077016626
CD59	67489	51213	0.017009828	0.0420259	0.077020639
CHST2	320	283	0.23788228	0.0420475	0.077048861
ADAMTS12	4066	3198	0.068711855	0.0420648	0.077069382
DR1	4122	3241	0.068246648	0.0420719	0.077070885
ZNF283	254	229	0.265671233	0.0420875	0.07708822
LOC731275	172	161	0.319804255	0.0421061	0.077111017
ZNF417	591	501	0.176804605	0.0421635	0.077204668
TGFB3	45	51	0.595724377	0.0422162	0.077289871
RPS4Y1	10899	7935	-0.042741512	0.0422694	0.077375877
PRPS2	1402	967	-0.120746423	0.042367	0.07754311
RAB3IL1	130	73	-0.417391122	0.0423971	0.07758686
TMEM48	3124	2216	-0.08028444	0.0424664	0.077702258
BCL2L12	435	280	-0.220436442	0.0424748	0.077706187
GSTT1	1186	812	-0.131400246	0.0425633	0.077856597
FAM109A	686	456	-0.17402262	0.0426016	0.077915351
KCNQ5	1183	968	0.125781011	0.0426471	0.077987063
URB1	3595	2560	-0.074695829	0.0426659	0.07800996
SAMD8	4666	3344	-0.065459328	0.042667	0.078000474

BTN3A3	1067	877	0.132240703	0.0427304	0.078104999
TTC17	4094	3219	0.068253606	0.0427544	0.078137395
ZNF846	455	294	-0.214898259	0.0428914	0.078376272
LOC100216545	98	98	0.415152132	0.0428931	0.078367914
ZNF778	508	331	-0.202845148	0.043004	0.078558899
LRIG1	779	650	0.153968522	0.0430239	0.07858368
NAT10	2650	1871	-0.087030669	0.0430406	0.078602777
FBXO15	44	50	0.599576703	0.0430707	0.078628816
PCDHB6	44	50	0.599576703	0.0430707	0.078628816
PSG4	44	50	0.599576703	0.0430707	0.078628816
TAF4B	44	50	0.599576703	0.0430707	0.078628816
PIGL	213	128	-0.319557488	0.0430723	0.078603033
BOD1	3240	2301	-0.078580698	0.0430735	0.078593668
FAM13A	1428	986	-0.119184296	0.0431064	0.078642263
SIN3A	3059	2169	-0.080877922	0.0431419	0.078695377
TMTC4	535	456	0.184647065	0.0432034	0.078796087
PHACTR4	2577	1818	-0.088188206	0.0432401	0.078851469
FN3K	207	124	-0.324138515	0.0432492	0.078856448
DOLK	1389	958	-0.120796906	0.0432496	0.07884578
TAF11	1066	876	0.131947469	0.0433013	0.078928438
USP3	1106	755	-0.135650704	0.0433587	0.079021509
ARHGEF11	3263	2318	-0.078166289	0.0434245	0.079129873
LOC338799	195	116	-0.334197187	0.0434675	0.079196606
EP400NL	189	112	-0.33973537	0.043499	0.079242448
ANO7	55	26	-0.665767864	0.0436948	0.07958736
PRR14	1005	683	-0.142085886	0.0439158	0.079978248
QDPR	1601	1111	-0.111962359	0.0439629	0.080052244
APCDD1L-AS1	291	181	-0.269877324	0.0439845	0.080080011
EIF1AY	1208	987	0.123653667	0.0441073	0.080291784
ST20	73	76	0.473255086	0.0441944	0.080438676
POGK	2331	1860	0.089505749	0.0441968	0.080431159
PHF21A	2406	1918	0.08811821	0.044203	0.08043068

VAMP3	10269	7934	0.042976736	0.0442417	0.080489372
GMPR2	1966	1375	-0.100679571	0.0442964	0.080577197
ACCS	97	52	-0.484320992	0.0443422	0.080642803
LRRC4	97	52	-0.484320992	0.0443422	0.080642803
APOL2	1598	1109	-0.111855911	0.0443593	0.080656129
AQP1	89	90	0.431271797	0.0444243	0.080762689
NMNAT2	118	115	0.377999133	0.0444357	0.080771638
SNRNP25	1199	822	-0.129469228	0.0444564	0.080797372
LOC152217	339	214	-0.248522345	0.0444927	0.080851552
FAM84A	81	83	0.45034156	0.0445353	0.080917216
NUP35	473	406	0.194791676	0.0445421	0.080917768
RAB21	3657	2882	0.071561841	0.0445505	0.080921208
PRKD2	1663	1156	-0.109494639	0.0445702	0.080945087
LARP7	2162	1729	0.092723478	0.0445703	0.080933551
MIRLET7BHG	148	85	-0.384910298	0.0446104	0.080994555
STRIP2	488	418	0.191773926	0.0446543	0.081062502
ACBD3	7527	5844	0.050033338	0.0446866	0.081109205
MOB2	498	426	0.18985982	0.0447014	0.08112429
CIB1	2166	1732	0.092557819	0.0447608	0.081220322
GPR155	1143	782	-0.132432759	0.0448401	0.081352248
CAPZA2	8091	6274	0.048219639	0.0449227	0.081490251
GPATCH2L	1188	971	0.124160496	0.0449958	0.081610966
METTL16	1661	1340	0.105333059	0.0450239	0.081650127
ERCC6L2	699	586	0.160760341	0.0451054	0.081786015
EDNRA	1371	1114	0.115672793	0.045134	0.081825936
TCEB1	3746	2672	-0.07227876	0.0451789	0.081895328
SPR	607	401	-0.182942148	0.0452918	0.082088036
MRPL15	2107	1686	0.093566354	0.0453612	0.082201898
SKAP2	2337	1864	0.088896258	0.0454057	0.082270541
CRYL1	560	368	-0.190568929	0.0454587	0.082354547
C16orf59	211	127	-0.31726237	0.0455195	0.082452784
ZNF746	1137	778	-0.132238062	0.0457161	0.082796926

MSL3	1338	1088	0.116752572	0.0457174	0.082787119
CDKN2AIP	1099	901	0.128559757	0.0457189	0.082777875
PSMC6	4596	3602	0.063571515	0.0457324	0.082790235
PMM2	2323	1853	0.089025859	0.0457354	0.082783619
ZNF845	441	380	0.200372895	0.0457908	0.082871873
EXD2	1388	1127	0.114632079	0.0457937	0.082865121
DCTN5	7775	5633	-0.049787072	0.0459844	0.0831981
NAPEPLD	1080	886	0.129499423	0.0459955	0.083206152
KIF2A	3817	3004	0.069597758	0.0460053	0.08321178
TBC1D10B	3091	2194	-0.079357997	0.0460204	0.083226892
LRRC47	2220	1560	-0.093861516	0.0460561	0.083279409
C10orf114	134	76	-0.403009545	0.0460915	0.083331393
TMEM54	507	331	-0.200002398	0.0461162	0.083363909
LOC100288778	369	235	-0.235807928	0.0461979	0.083499466
MLF1	174	162	0.312058639	0.0463639	0.083787388
FAM86B3P	52	57	0.547602428	0.0465241	0.084064616
PGRMC1	5289	3804	-0.060325597	0.0465281	0.084059631
ANKS1A	1612	1120	-0.11020088	0.0465734	0.08412929
THRA	1309	1065	0.117540465	0.0466097	0.08418265
LATS1	2786	1972	-0.083383574	0.0467115	0.084354259
PTK2	7012	5449	0.051317627	0.0468265	0.08454973
THAP2	455	391	0.196454194	0.0468398	0.084561521
IPO11	1272	1036	0.119077464	0.0468466	0.084561579
LOC100506714	209	191	0.285221828	0.047008	0.084840608
TOX4	3870	3044	0.068786924	0.0470928	0.084981313
SCRN2	714	477	-0.166782676	0.0471456	0.085064168
AVEN	1013	833	0.132906358	0.0471934	0.085138168
MELK	1797	1254	-0.103900929	0.0473338	0.085379091
PYCARD	255	157	-0.284580556	0.0473451	0.085387019
STXBP4	994	818	0.134007123	0.047421	0.085511643
OFD1	839	696	0.145568627	0.0474225	0.085495743
PRMT3	839	696	0.145568627	0.0474225	0.085495743

TCTN3	4173	2986	-0.067718623	0.0474502	0.085527128
CLDN18	61	65	0.506782607	0.0475663	0.085724038
METAP2	6414	4632	-0.054436969	0.047688	0.085930765
KCNK1	149	86	-0.377751634	0.0476967	0.085934117
TRMT2A	1083	740	-0.134283935	0.0477657	0.086045922
ZFHX2	108	106	0.388185084	0.0479051	0.086284654
IGF2	71	36	-0.564669986	0.0479239	0.086306047
TTF2	1182	811	-0.128304084	0.0479395	0.08632158
SLK	5855	4221	-0.05693221	0.0479584	0.086343126
B3GNT5	126	71	-0.412380672	0.0481705	0.086712501
NDUFC2	462	300	-0.207778219	0.0482547	0.086851571
WDR20	1521	1055	-0.112625022	0.0482837	0.086891131
ARMCX2	3731	2663	-0.0713578	0.0484028	0.087092786
POP5	698	466	-0.16774495	0.0484252	0.087120668
MED18	512	335	-0.196830583	0.0484456	0.087144637
SSR4	4779	3431	-0.06292751	0.0485094	0.087246863
B9D2	93	93	0.415152132	0.0485616	0.08732809
C19orf10	9597	6980	-0.044204325	0.0487931	0.087731846
MED8	2272	1812	0.088772252	0.0488316	0.087788305
NID1	7420	5374	-0.050270734	0.0490435	0.088156557
ANKRD9	348	221	-0.239888805	0.0490526	0.088160259
MYCBP2	9166	6662	-0.0451848	0.0490668	0.088173025
IFT52	1454	1007	-0.114811454	0.0491124	0.088242176
NCL	34732	25633	-0.023111021	0.0491338	0.088267907
TGM1	37	43	0.631963521	0.0492585	0.088479253
C18orf21	557	472	0.176261664	0.0492586	0.088466551
SELRC1	784	652	0.149170442	0.0494063	0.088719154
ARCN1	23173	17719	0.028004488	0.0494819	0.088842054
ZNF668	861	582	-0.149841953	0.0495157	0.088889957
ADAP1	90	48	-0.491738464	0.0495226	0.088882985
JMJD7	90	48	-0.491738464	0.0495226	0.088882985
USP18	607	402	-0.179348883	0.0495491	0.088911437

TMEM184C	2622	1854	-0.08487431	0.0495922	0.088975887
POLR3E	1230	846	-0.124776615	0.0496004	0.088977791
SPRY2	2696	2139	0.081268118	0.049754	0.089240521
ZNF530	177	164	0.305098586	0.0497642	0.089245968
PCED1B	59	63	0.509789006	0.0498729	0.089427923
PRKRIR	3244	2561	0.074085565	0.0500408	0.089716106
GAREML	146	138	0.33385203	0.0500448	0.08971049
FUOM	381	244	-0.227757718	0.0500762	0.089747327
GLRB	381	244	-0.227757718	0.0500762	0.089747327
ALG8	1736	1211	-0.104415951	0.0501321	0.089828135
OCEL1	272	169	-0.271431273	0.0502234	0.089978717
AFF2	56	27	-0.637315288	0.0502405	0.089983507
FGFBP3	56	27	-0.637315288	0.0502405	0.089983507
TICAM2	56	27	-0.637315288	0.0502405	0.089983507
ZNF577	311	274	0.232413445	0.050427	0.090291539
PNPLA8	2074	1456	-0.095253407	0.0504316	0.090286921
NELFCD	2734	1936	-0.082782159	0.0504357	0.090281123
DUSP18	466	399	0.191210923	0.050446	0.09028669
KLF3	3243	2560	0.073966918	0.0504492	0.090279475
DHX15	8019	6214	0.047252035	0.05046	0.090285743
GAS6	31462	23203	-0.024146904	0.0504604	0.09027358
GALK1	710	475	-0.164739379	0.0504812	0.090297694
ABCB9	437	283	-0.211679095	0.0505032	0.090324222
MYRF	374	325	0.21255358	0.0505499	0.090394606
LOH12CR1	269	167	-0.272605938	0.0505527	0.090386699
OPHN1	1601	1291	0.104667825	0.0505547	0.090377395
CERK	4286	3361	0.064410824	0.050577	0.090404243
EIF2D	1867	1306	-0.100414899	0.050593	0.090419774
BCL7B	1178	809	-0.1269758	0.0506347	0.090481367
SPIRE2	147	85	-0.375129277	0.0507058	0.090595395
ARHGEF9	1406	973	-0.115932753	0.0507745	0.090705195
GLRX2	672	563	0.159825821	0.0508369	0.090803584

SUSD2	48	53	0.558110086	0.0508475	0.0908096
TBXA2R	266	165	-0.27380809	0.0508799	0.090854362
MGST2	289	256	0.240226449	0.0509504	0.090967149
DSN1	967	658	-0.140276174	0.0509534	0.09095952
MANEA	2785	2207	0.079561434	0.050969	0.09097433
PARP16	204	123	-0.314758705	0.050974	0.090970213
UBL7	1453	1007	-0.113818888	0.0511441	0.091260685
SLC2A8	571	377	-0.18377409	0.0511511	0.091260154
CCBL1	333	211	-0.243127047	0.051159	0.091261095
S1PR1	772	642	0.149124582	0.0512529	0.091415569
SURF2	317	200	-0.249330709	0.0512751	0.091442096
CDCA5	964	656	-0.1401852	0.0514618	0.091761876
MAP3K5	3366	2654	0.072285326	0.0515331	0.091875809
NBPF7	83	84	0.432430123	0.0515669	0.091922977
TSPAN31	899	742	0.138250203	0.0515911	0.091952946
SEPT6	1673	1166	-0.105717525	0.0515955	0.091947655
ZFP64	663	442	-0.169810369	0.0515964	0.091936104
LINC00174	183	109	-0.332363382	0.0516597	0.092035651
HDHD3	180	107	-0.335233978	0.0516976	0.09209008
FRG1	1045	714	-0.134354831	0.0517254	0.092126423
ARNT2	1965	1573	0.09414149	0.0518055	0.092255889
TM9SF3	22483	16532	-0.028421153	0.0518222	0.092272332
LOC150381	34	40	0.649617385	0.0519512	0.092488937
KCTD16	47	52	0.561002998	0.0519792	0.092525553
CD27-AS1	229	140	-0.294768639	0.0519888	0.092529341
TAPBP	7466	5791	0.04862912	0.0520341	0.09259674
LASP1	32809	25005	0.023277115	0.0522039	0.092885713
LOC401074	226	138	-0.296502374	0.0522307	0.092920142
ATP6V0E1	7077	5494	0.049871123	0.0523455	0.093110952
PFN1P2	1458	1179	0.10872513	0.0524324	0.093252279
TEAD1	29222	22292	0.024623268	0.0525509	0.093449589
KLHL5	13629	9966	-0.0364411	0.0525733	0.093476065

CTSS	64	32	-0.584847868	0.0525839	0.093481643
LAT	133	76	-0.39220279	0.0526918	0.093660078
FOXL1	5049	3632	-0.060081343	0.0527348	0.093723165
B3GNT1	1111	908	0.124057518	0.052746	0.093729688
DERA	1606	1118	-0.107399573	0.052758	0.093737647
NADSYN1	2389	1686	-0.087650186	0.0530447	0.094233519
RPSAP58	286	179	-0.260903428	0.0531791	0.094458836
SESN3	1989	1591	0.093042691	0.0531891	0.094463263
EXOC6B	2155	1516	-0.092265984	0.0532879	0.094625178
IFITM1	736	613	0.151333439	0.0533232	0.094674397
HIP1R	934	769	0.13471318	0.0533453	0.094700091
RBM12	3301	2603	0.072424305	0.0533749	0.094739084
TACC1	15124	11613	0.034053091	0.0534046	0.094778263
SYNJ1	2037	1628	0.091806851	0.0535856	0.095086048
UMPS	1338	925	-0.117400713	0.0537067	0.095287278
EMC8	1582	1101	-0.107782999	0.053718	0.095293717
C1QL4	54	26	-0.639295652	0.0538623	0.095529283
LOC100505865	54	26	-0.639295652	0.0538623	0.095529283
FAM208A	6176	4805	0.05301581	0.0538685	0.095519923
VPS11	1658	1334	0.101466791	0.0538774	0.095522079
TBC1D16	4083	2924	-0.066534125	0.0539966	0.095719797
ZNF773	386	334	0.206399387	0.0540005	0.095713094
DZANK1	149	140	0.325266628	0.0541249	0.095919998
ACOT2	624	415	-0.173282561	0.0541267	0.095909548
LOC100294145	1446	1169	0.108359509	0.0542356	0.096088749
RBKS	45	50	0.567155225	0.0543029	0.096194331
PNRC1	2533	2011	0.082218136	0.0543069	0.096187756
RAB5A	3571	2810	0.069394128	0.0543225	0.096201784
ZNF133	720	483	-0.160821586	0.0543476	0.096232575
SMU1	3838	3015	0.066955422	0.0543568	0.096235169
EMC10	3278	2335	-0.074241173	0.0543669	0.096239359
ARFGEF2	5517	4301	0.055940283	0.0544034	0.09629022

RBM28	1287	1045	0.11464302	0.0544255	0.096315615
ISPD	167	155	0.307572245	0.0546019	0.09661403
CCHCR1	918	624	-0.141795993	0.0546216	0.096635253
GTF2A1	5979	4317	-0.054722986	0.0546367	0.096648144
LOC100506233	160	94	-0.352187111	0.0546709	0.096694997
EPHB3	181	108	-0.329806253	0.0546993	0.096731403
METTL17	1048	717	-0.132441561	0.0547196	0.096753633
RFC1	3778	2701	-0.06897693	0.0547656	0.09682117
CTDP1	1393	965	-0.114442279	0.0548797	0.097009144
LENG9	105	58	-0.441112391	0.0548841	0.097003134
ATF6B	3305	2355	-0.07377108	0.0548916	0.097002668
PRICKLE3	290	182	-0.256962318	0.0550048	0.097188985
SULF2	88	88	0.415152132	0.0550266	0.097213622
GIGYF1	2127	1697	0.089318663	0.0551022	0.097333428
C10orf55	67	34	-0.563474217	0.0551309	0.09737027
ABCB6	762	513	-0.15568004	0.0551357	0.097364983
OXLD1	335	213	-0.238155533	0.0551559	0.097386795
FAM107B	4468	3207	-0.0632527	0.0551936	0.097439598
RBMX2	1038	710	-0.132763382	0.0553082	0.097628047
E2F6	908	748	0.135498104	0.0553151	0.097626418
DNAJC9	2169	1527	-0.091177859	0.0553214	0.097623612
C14orf37	743	618	0.149396759	0.0553686	0.097693163
ZFHX3	3469	2731	0.070061605	0.0554878	0.09788957
TST	625	416	-0.17212053	0.0555149	0.097923559
ANP32A-IT1	80	81	0.43307404	0.0555837	0.098030988
CROCCP2	369	320	0.209603221	0.0555908	0.098029584
WDR74	721	484	-0.15984008	0.0556245	0.098075244
ZNF571	72	74	0.454680496	0.0556448	0.098097126
TGFBRAP1	898	610	-0.142754071	0.0557034	0.098186579
COX11	1987	1395	-0.095174619	0.0557155	0.098193863
HSD17B11	1212	986	0.117421985	0.0557846	0.098301883
KRR1	1430	992	-0.11245099	0.0558798	0.098455652

EXOSC10	3266	2327	-0.073901448	0.0559145	0.098502816
CENPC1	818	677	0.142207122	0.0559762	0.098597558
TIAM2	122	117	0.354779514	0.0560695	0.098747936
TAOK3	5304	4137	0.056656314	0.0560705	0.098735839
CCNL1	2437	1936	0.083124833	0.056304	0.099132838
FANCL	209	127	-0.303522314	0.0563055	0.099121614
AMIGO3	313	198	-0.245510095	0.0563373	0.099163499
KIF16B	916	754	0.134369057	0.0563389	0.099152376
ATP5S	218	197	0.269019626	0.0563982	0.099242663
CYB561	195	178	0.283555249	0.0565479	0.099492029
KIAA0513	2054	1444	-0.093213308	0.0566754	0.099702203
C19orf71	97	53	-0.456840256	0.0566763	0.099689826
LRRC3	147	138	0.324004244	0.0567767	0.099852253
NUBP1	454	296	-0.20194299	0.0567955	0.09987127
BTBD11	234	144	-0.285287586	0.0568749	0.099996714
FRAT2	153	143	0.317635626	0.0570129	0.100225154
DNPH1	548	362	-0.183034064	0.0570302	0.100241557
AFG3L2	3335	2378	-0.072785915	0.0570407	0.100245748
C11orf70	71	73	0.455229571	0.0570476	0.100243693
C7orf49	729	490	-0.157984934	0.0570628	0.100256279
MVB12A	767	517	-0.153910165	0.0570671	0.100249808
CYHR1	1160	798	-0.124512022	0.0570732	0.100246356
DHX35	674	451	-0.164469026	0.0570934	0.100267684
EVC2	375	241	-0.222705317	0.057123	0.100305594
BHLHE41	177	163	0.296274736	0.0571279	0.100299967
TOMM6	2539	1797	-0.083517854	0.0572098	0.100429673
PLEK2	194	117	-0.314395991	0.0572774	0.10053411
DKFZP686I1521	57	28	-0.61038296	0.0573427	0.10062739
LAT2	57	28	-0.61038296	0.0573427	0.10062739
ULK2	1086	887	0.123134038	0.0573653	0.100645878
KIAA1143	2880	2277	0.076217612	0.0574023	0.100696611
SFT2D1	1132	778	-0.125879766	0.0574539	0.100772837

LOC440288	70	36	-0.544205884	0.0574846	0.10081248
FAM173B	726	488	-0.157936269	0.0576511	0.101090342
SLC25A32	3369	2653	0.070456379	0.0576968	0.101156126
TMEFF2	100	55	-0.447344345	0.0577035	0.101153635
PLAC9	152	89	-0.357041951	0.0578214	0.101346169
KIAA0753	2210	1760	0.086681191	0.0578891	0.10145054
SLC25A51	740	498	-0.156227397	0.0579095	0.101472029
BMP3	42	47	0.577423561	0.0579349	0.101502188
MLF2	10829	7899	-0.040005965	0.0580048	0.101610312
MSS51	158	93	-0.349469805	0.0580136	0.101611417
SCAI	1336	1082	0.110932623	0.0580838	0.101720201
SMEK1	4429	3466	0.061442789	0.0581126	0.10175635
PLCD4	247	153	-0.275827257	0.0581349	0.101781093
AHCY	5224	3764	-0.057736137	0.0581942	0.101870535
PRDX3	7988	6184	0.045858118	0.0581971	0.101861295
CASP2	2016	1417	-0.093503749	0.0582407	0.101923283
ZNF415	425	276	-0.207642442	0.0583335	0.102071264
NPHP4	559	370	-0.180170881	0.0583637	0.102109752
KRT15	152	142	0.316971738	0.0583845	0.102131898
RNF182	517	438	0.175918721	0.0586741	0.102624029
MGMT	658	440	-0.165431928	0.0586822	0.102623798
FAM120C	1183	815	-0.122425977	0.0587564	0.102739218
LOC100130557	52	56	0.522067336	0.0588376	0.102866683
MMAB	366	317	0.207791324	0.0588437	0.102862891
FAM58A	817	553	-0.147904466	0.0588686	0.102892015
SLC26A11	824	558	-0.147227084	0.0589211	0.102969242
RHBDL2	65	33	-0.562821562	0.0591567	0.103366457
TARSL2	1298	1052	0.111996453	0.0592708	0.103551305
SERAC1	781	647	0.143595296	0.0592708	0.103536877
RNASEH2B	499	328	-0.190191869	0.0594729	0.103875267
TAB1	1198	826	-0.12126209	0.0595305	0.103961441
NLRX1	429	279	-0.205560394	0.0596084	0.104082784

PCSK4	73	38	-0.526744914	0.059655	0.104149484
SAAL1	570	378	-0.177423553	0.0596679	0.104157443
C4orf29	396	256	-0.214204488	0.0596921	0.104185195
CPSF2	7003	5432	0.048662524	0.0597085	0.104199211
DNAJC4	933	636	-0.137698184	0.0597199	0.104204423
RAB24	926	631	-0.138220057	0.0597386	0.104222412
C4orf32	175	161	0.294857898	0.0598299	0.104367177
INTS2	1227	847	-0.119549243	0.0600335	0.104707689
FAM161B	436	373	0.189999627	0.0601399	0.104878472
HSD17B7P2	51	55	0.524086503	0.0602385	0.105035839
GIGYF2	4880	3810	0.058061982	0.0603196	0.105162426
FBXO44	895	609	-0.140293322	0.0603779	0.105249346
HOTAIRM1	344	220	-0.229752909	0.0604626	0.105382183
HSPA14	817	675	0.139703556	0.0607086	0.105796203
IFT140	845	697	0.137359446	0.060832	0.105996505
BRMS1	1971	1574	0.090659896	0.0608546	0.106020964
SLC1A2	267	167	-0.261839507	0.0608567	0.106009855
LRRC20	443	289	-0.201085074	0.0609377	0.106136027
GCAT	663	444	-0.163297062	0.0609388	0.106123073
NOL8	2541	1800	-0.082247337	0.0609408	0.106111848
SCAMP2	2210	1559	-0.08827331	0.0609727	0.106152449
INPP4B	871	592	-0.141923411	0.060976	0.106143428
SENP3	112	63	-0.414922867	0.0609969	0.10616497
KIAA1549L	3145	2241	-0.073765237	0.0611321	0.106385357
ADCK1	245	152	-0.273558294	0.0611781	0.106450563
COX18	987	675	-0.133010451	0.061183	0.106444234
APOBEC3C	3402	2429	-0.070868519	0.0612051	0.106467777
DPM2	980	670	-0.133468522	0.0612349	0.106504698
SEMA3D	527	348	-0.183563524	0.0612779	0.106564638
MGAT5B	312	198	-0.240893467	0.0613404	0.106658506
SLC7A11-AS1	162	150	0.304120819	0.0613898	0.106729495
MLLT4	2950	2329	0.074147817	0.0614432	0.106807376

USP12	4376	3143	-0.062318334	0.0615147	0.106916783
DHFR	3410	2435	-0.070697835	0.0615182	0.106907984
SLC39A9	5285	4119	0.055542786	0.0615923	0.107021775
RAPGEFL1	159	94	-0.343141972	0.0615948	0.107011192
FSIP1	50	54	0.526183444	0.061671	0.107121224
LOC440028	50	54	0.526183444	0.061671	0.107121224
MAP3K12	1617	1129	-0.103122061	0.0618324	0.107379009
TYW1	1076	739	-0.126879677	0.0619516	0.10757104
ASF1A	1805	1445	0.094222787	0.0621081	0.107827846
LARP1B	1235	1002	0.113523598	0.0621231	0.107838789
ATP6V1D	5525	4302	0.054185192	0.0623158	0.10815817
NANS	1890	1327	-0.095065732	0.0623401	0.108185302
SLC35E3	572	481	0.165173879	0.0623617	0.108207637
DALRD3	521	344	-0.183722676	0.062552	0.108522733
ZSCAN2	484	318	-0.19082815	0.062583	0.10856148
PRELID2	124	118	0.343598871	0.0626339	0.108634674
ZNF446	233	144	-0.279109011	0.0627391	0.108801925
LRRC37BP1	1294	896	-0.115114848	0.0627434	0.108794268
IFI44	161	149	0.303403774	0.0628478	0.108960131
NT5C3B	1481	1031	-0.107375176	0.0629456	0.109114477
RFT1	1366	948	-0.111846388	0.0630494	0.109279154
ZNF761	1077	740	-0.126268942	0.0630673	0.10929501
LINC00094	1509	1215	0.10251564	0.0630974	0.109331996
C3orf38	1383	1117	0.106980161	0.063149	0.109406184
TCERG1	4032	3159	0.06312443	0.0631767	0.109438963
CRCP	2183	1737	0.085437755	0.0632435	0.109539312
FADS2	3989	2860	-0.064859845	0.0632877	0.109600685
CD47	9174	7083	0.041961767	0.063417	0.109809442
DPF1	249	155	-0.268725395	0.0635054	0.109947186
NCAPG2	2567	1820	-0.080992714	0.0635734	0.110049587
CERS5	2537	2010	0.079224114	0.0636242	0.110122202
PAR-SN	313	199	-0.238242095	0.0637027	0.110242703

ERVK13-1	724	601	0.146527425	0.0637293	0.110273508
SLC45A4	326	208	-0.233136304	0.0638173	0.110410402
PNMAL1	71	37	-0.525141622	0.0639945	0.110701621
PPIE	1187	964	0.114937248	0.0640213	0.110732528
DNAJB12	2177	1732	0.085249654	0.0642016	0.111028981
BID	2579	1829	-0.080604566	0.0642121	0.11103177
FLRT2	2797	2210	0.075318248	0.0642753	0.111125584
PPAPDC1B	1414	983	-0.109366667	0.0643011	0.111154865
C10orf76	1747	1224	-0.098123918	0.0645106	0.111501444
PTPLB	864	711	0.133970379	0.0646289	0.111690364
GALNT1	5735	4461	0.052725794	0.0646714	0.1117483
GK	2421	1714	-0.083083838	0.0646784	0.111745019
BORA	359	231	-0.220938861	0.0647264	0.111812391
HYAL3	139	81	-0.363938938	0.0647548	0.111845999
ZW10	1722	1206	-0.098703104	0.0648911	0.112065869
C3orf14	346	222	-0.22506023	0.064907	0.112077656
LOC100134868	215	132	-0.288646598	0.0649298	0.112101576
BRD8	2800	2212	0.07507669	0.0650022	0.112210981
SNX22	58	29	-0.584847868	0.0650044	0.112199235
SLC18B1	261	231	0.238995177	0.0650329	0.112232892
LTV1	2026	1615	0.088052123	0.065034	0.112219184
RGL4	291	184	-0.246161255	0.0650668	0.112260369
CRABP2	369	238	-0.217507111	0.0650809	0.112269123
PCDHGA11	145	85	-0.355366022	0.0650825	0.112256318
C17orf67	148	87	-0.351357738	0.0651925	0.112430498
NDUFB6	1766	1238	-0.097321897	0.0652233	0.112467902
ASIC1	611	408	-0.167451096	0.0652727	0.112537616
GALNT18	160	95	-0.336920355	0.0653204	0.11260431
LOC100506178	57	60	0.489152713	0.0653437	0.112628747
REEP2	1390	966	-0.109837657	0.0654654	0.112822941
C16orf91	317	202	-0.234975416	0.0655452	0.112944874
AK3	5279	4112	0.05472773	0.0656972	0.113191178

AEBP2	2603	2060	0.077621157	0.0657313	0.113234134
NDN	559	470	0.164964605	0.0657662	0.113278637
KLF2	533	353	-0.179315218	0.0657823	0.113290775
PPOX	399	259	-0.208284517	0.0657935	0.113294426
DDRGK1	1814	1273	-0.095789905	0.0658141	0.113314202
KLHL35	53	26	-0.612328605	0.0660612	0.113723874
SQSTM1	14240	10432	-0.03378124	0.0661186	0.113806876
RGS9	88	87	0.398664009	0.0661792	0.113895497
RCCD1	557	370	-0.174999925	0.0662127	0.11393736
CDS2	4645	3343	-0.05938311	0.0662698	0.114019883
OXSM	250	156	-0.265229934	0.0663155	0.114082859
ARHGEF17	7127	5522	0.047048095	0.0663567	0.114137949
ATP2B1	4946	3857	0.056369038	0.0664416	0.114268134
COPA	26341	19418	-0.02476337	0.0664776	0.114314349
GPATCH1	581	387	-0.171052466	0.0664946	0.114327673
SLC12A4	6201	4488	-0.051276082	0.0665158	0.114348439
EMD	2586	1835	-0.07979008	0.0665432	0.114379695
ZNF2	373	241	-0.214990352	0.0666521	0.114551142
LOC100506990	297	260	0.223200824	0.0667916	0.114774944
CECR6	35	40	0.60779721	0.0670043	0.115124697
SNX14	5059	3943	0.055593603	0.067175	0.115401955
TTI1	1216	841	-0.116813391	0.0672315	0.115483083
XPNPEP1	3903	3058	0.063157076	0.0674092	0.11577246
WDR11	4599	3591	0.058217587	0.0674749	0.115869334
CCZ1B	219	135	-0.282819331	0.0676895	0.116221736
NVL	1106	762	-0.122336351	0.0678267	0.116441288
C12orf76	204	184	0.266288746	0.0678405	0.116448946
DUSP4	305	194	-0.237600459	0.0678987	0.116532807
OIP5-AS1	1028	706	-0.126948044	0.0679267	0.11656481
RGCC	541	359	-0.176492618	0.0679445	0.116579224
HACE1	809	667	0.136699191	0.067953	0.116577839
TRIM5	3048	2173	-0.073022597	0.0679844	0.116615633

ACTRT3	94	92	0.384125236	0.0680075	0.11663918
KIAA1324	77	41	-0.494082404	0.0680607	0.116714393
ZNF671	414	354	0.189270724	0.0680811	0.116733224
ATF5	824	560	-0.142065378	0.0681229	0.116788774
TMEM156	181	165	0.281628459	0.0681561	0.116829718
ELL	1086	884	0.118246303	0.0682229	0.116928044
RPL23AP7	531	352	-0.1779843	0.0682672	0.116987968
ZNF707	477	314	-0.188072575	0.0683332	0.117084875
LUC7L3	7222	5242	-0.047128935	0.068545	0.117431654
GKAP1	55	58	0.491773413	0.0686365	0.117572265
DPF3	538	357	-0.176529967	0.0686555	0.117588608
PKP3	105	59	-0.416450337	0.0687252	0.117691815
TOMM40L	483	409	0.175229786	0.0687547	0.117726114
TCTN2	657	547	0.150799594	0.0689057	0.117968406
MRPL1	1108	901	0.116793262	0.0689392	0.118009571
C15orf37	140	82	-0.356578881	0.0690014	0.118099749
ANXA4	3100	2441	0.070356211	0.069061	0.118185583
STRADA	1426	993	-0.106956227	0.0691053	0.118245163
RLF	1567	1095	-0.101922178	0.0691586	0.118320046
EME1	207	127	-0.289650139	0.0692084	0.118389115
FAM115C	398	341	0.19215544	0.0692111	0.118377451
ZNF443	146	86	-0.348407672	0.0692193	0.118375192
CYB5R1	2575	2037	0.07702568	0.0693553	0.11859151
TMUB1	853	581	-0.138855446	0.0695101	0.118839898
RAB3IP	100	97	0.371208784	0.0695333	0.118863129
MT1X	371	240	-0.213232649	0.0695719	0.118912918
DZIP1L	482	408	0.174688137	0.0699317	0.119511363
ZNF470	613	512	0.155408868	0.0699518	0.11952936
HOXA3	391	254	-0.207187979	0.0699791	0.119559704
FAM211B	111	63	-0.401983811	0.0701365	0.119812032
RNF126	1319	916	-0.110872929	0.0701422	0.119805356
ILF3-AS1	421	275	-0.199236483	0.0702014	0.119890117

SPOP	2233	1773	0.082361417	0.0702927	0.12002951
SDF2L1	721	487	-0.150925355	0.0702938	0.120015043
ZNF561	2721	2149	0.074680658	0.0704037	0.120186111
PCDHGC3	3141	2242	-0.071285533	0.0704172	0.120192745
ACP2	1463	1177	0.101336683	0.0705221	0.12035524
SLC18A2	319	204	-0.22983514	0.0705256	0.120344774
TEX2	3268	2335	-0.069833302	0.0705609	0.120388471
ZNF251	1280	888	-0.112360097	0.0706875	0.120587923
PLEKHA8P1	150	139	0.305274514	0.0707233	0.120632596
CD63	45131	33412	-0.018600271	0.0708288	0.120795965
ACSF3	546	363	-0.173779271	0.0708484	0.120812882
SND1-IT1	51	25	-0.61341702	0.0709403	0.120952957
GORASP1	2131	1694	0.084055413	0.0710601	0.121140589
MRPL9	1198	829	-0.11603177	0.0711346	0.121251081
BAI2	1758	1234	-0.095440544	0.0711418	0.121246676
MED31	99	96	0.370758012	0.0713085	0.121514198
RNF157	455	299	-0.190568929	0.0713692	0.12160093
ARHGAP33	395	257	-0.204932162	0.0715194	0.121840227
TAF6L	482	318	-0.184854249	0.0715483	0.121872865
INCA1	70	71	0.435616234	0.0715525	0.121863323
CIRBP	7405	5379	-0.046009615	0.0717205	0.122132709
NKX3-1	214	132	-0.281920735	0.0717608	0.122184589
RPF2	1286	1039	0.107457143	0.0718717	0.122356841
ZNF493	340	294	0.20543354	0.0719213	0.122424414
RCBTB2	615	412	-0.162789941	0.0719582	0.122470478
SEC22C	2857	2253	0.072498409	0.0720003	0.122525405
ASL	1657	1161	-0.098053499	0.0720004	0.122508923
STON1	692	574	0.145430831	0.0720624	0.122597575
CDK7	2705	2136	0.074435185	0.0721816	0.122783673
FKRP	1434	1154	0.101750332	0.0721825	0.122768397
DHX29	8995	6554	-0.041595477	0.0725241	0.123332644
ZNF37BP	1231	996	0.109539017	0.0725289	0.123323891

PDCL	1429	1150	0.101780076	0.072596	0.123421212
FAM76B	911	746	0.126876708	0.0726957	0.12357377
CA12	2023	1427	-0.088358853	0.0727529	0.123654171
NAPRT1	265	167	-0.250992125	0.0728925	0.123874516
FAM86FP	178	162	0.279268704	0.0729881	0.124020065
RBBP4	4386	3156	-0.059656475	0.0730076	0.124036262
ZNF514	710	588	0.143149262	0.0730249	0.124048648
TTLL5	1262	1020	0.108009374	0.0730706	0.124109473
PPIEL	195	176	0.267253437	0.0731175	0.124172102
SCN5A	810	551	-0.140717457	0.0731509	0.124211967
TSSK4	135	79	-0.357882717	0.0731701	0.124227561
RARS2	3364	2642	0.066604893	0.073179	0.124225686
FIGNL1	926	634	-0.131377221	0.0731916	0.12423022
PTCH2	103	58	-0.4133674	0.0733875	0.124537239
REC8	103	58	-0.4133674	0.0733875	0.124537239
ZNF713	69	70	0.435910692	0.0733996	0.124532313
UQCC	1848	1300	-0.092301002	0.0734057	0.124525754
STX6	3544	2780	0.064858411	0.0734117	0.124518883
C9orf116	156	93	-0.331091276	0.0734224	0.124520117
FGFR3	153	91	-0.334441071	0.0734865	0.124611955
FCRLB	150	89	-0.337933128	0.0735208	0.124653137
ZC3H4	2440	1731	-0.080123291	0.0735281	0.12464855
SSH2	2824	2227	0.072513601	0.0736028	0.124758123
FAM219B	2337	1852	0.079578496	0.0737336	0.124962909
ULK3	600	501	0.155000234	0.0737659	0.125000609
RNF168	1568	1097	-0.100209902	0.0737839	0.12501402
USP8	3950	3091	0.061373133	0.0738741	0.125149807
RRN3P2	52	55	0.496072127	0.073896	0.125169891
BLM	463	305	-0.187050819	0.0740323	0.125383729
MTMR4	2622	1864	-0.077113694	0.0740453	0.125388791
ZNF562	1699	1359	0.093011735	0.0740544	0.125387082
PIN4	928	759	0.125127212	0.0741177	0.125477236

DAGLB	1525	1066	-0.101449673	0.0743107	0.125786927
PGP	741	502	-0.146634047	0.0744077	0.125934007
FAM86HP	104	100	0.358568603	0.0744326	0.125958928
RPF1	1915	1349	-0.090301912	0.0744867	0.126033371
TRMT10B	470	310	-0.18524041	0.0745853	0.126183017
TMEM185A	811	552	-0.139881516	0.0746514	0.126277701
REV1	1791	1259	-0.093334923	0.0747662	0.126454817
ARFGAP3	5169	4023	0.053538667	0.0748685	0.126610545
ZSWIM6	3150	2477	0.068394172	0.0749269	0.1266921
SPDYE1	171	156	0.282701836	0.0750827	0.126938307
EFR3B	187	114	-0.298852314	0.0752274	0.127165744
PMS2P4	92	51	-0.435984482	0.0754513	0.12752685
SPON1	296	258	0.216926022	0.075633	0.127816669
TGOLN2	20650	15777	0.026833253	0.0756615	0.127847584
PES1	3830	2749	-0.063285354	0.0757194	0.12792801
AGAP9	51	54	0.497614292	0.0757386	0.127943112
LOC100132891	418	356	0.183526431	0.0757568	0.1279565
C18orf54	882	603	-0.133468522	0.0757758	0.127971223
DYM	1741	1391	0.091358349	0.0758038	0.128001162
KBTBD7	437	287	-0.191430411	0.0759279	0.128193269
KDM6A	1236	999	0.108029972	0.0760344	0.128355751
FASTKD5	972	793	0.121516684	0.0760821	0.128418903
NDUFA7	1272	1027	0.106489643	0.0761144	0.128455916
NOP14-AS1	331	213	-0.220825655	0.0762663	0.128694894
RSBN1L-AS1	176	160	0.277648608	0.0764005	0.128903904
ACTL6A	2276	1804	0.079850913	0.0765784	0.129186485
TGDS	604	405	-0.16147451	0.0768585	0.12964155
FAF2	6029	4367	-0.050124067	0.076893	0.129682091
FBXO46	372	319	0.193405934	0.0769772	0.129806508
AIFM2	525	441	0.163613365	0.0770345	0.129885606
ARSD	1160	803	-0.115500781	0.0770941	0.129968487
MON1B	1264	878	-0.110551487	0.0770961	0.129954205

YTHDF2	5308	3836	-0.053413519	0.077099	0.129941623
ASAP3	1419	990	-0.104222027	0.0771032	0.129930995
POLE2	289	184	-0.236211595	0.0771975	0.130072333
PVT1	242	214	0.237755881	0.0772258	0.130102476
UBA2	5851	4236	-0.050828497	0.0772335	0.130097899
PHF13	1779	1251	-0.092832589	0.07731	0.130209108
ABTB2	258	227	0.230473364	0.0773324	0.130229177
TERF2IP	4269	3072	-0.059567815	0.0773781	0.13028846
GATAD1	2301	1823	0.07920571	0.0773944	0.130298364
BRD7	3166	2263	-0.069272539	0.0774416	0.130360279
PIGS	3862	3022	0.061307628	0.0774861	0.130417482
HAS3	98	55	-0.418197999	0.0775056	0.13043265
ABHD15	841	574	-0.135902932	0.0775877	0.130553249
CCNJL	181	164	0.272858249	0.0776497	0.130639859
NEK3	539	452	0.161189631	0.077682	0.130676495
ZNF706	1198	969	0.109092794	0.0776989	0.130687374
IP6K1	2934	2310	0.070176112	0.0777169	0.13070004
TMEM141	813	554	-0.138217244	0.0777201	0.130687625
CES2	3200	2288	-0.068832721	0.077724	0.130676691
MTFMT	764	519	-0.142685968	0.077731	0.130670817
UTP11L	2038	1620	0.083991893	0.0777471	0.130680103
LOC100507634	40	44	0.552655655	0.0778108	0.130769547
DESI1	3733	2923	0.062266452	0.077873	0.130856417
FOXC1	1050	724	-0.121175594	0.0779351	0.130943093
C12orf29	1236	858	-0.111477059	0.0779929	0.131022554
ZNF510	827	679	0.130676377	0.0780082	0.131030685
MYNN	1409	1133	0.100628381	0.0780306	0.1310506
AKD1	115	109	0.337846406	0.0782096	0.13133348
VGLL4	1143	791	-0.115923672	0.0782222	0.131337023
USP13	2678	2113	0.073288938	0.0782255	0.131324871
USB1	2357	1672	-0.080224779	0.0782327	0.131319145
BNIP1	550	367	-0.168499424	0.0784796	0.131715839

MGC21881	169	154	0.281059236	0.0786222	0.131937376
DKK2	57	29	-0.559756887	0.0786791	0.132015057
SNX33	2070	1463	-0.085548866	0.0787075	0.132045037
NCBP1	5199	4044	0.052700974	0.0787093	0.132030159
PNO1	1551	1086	-0.099022451	0.0788288	0.132212853
CMTM4	1737	1387	0.090522165	0.0789107	0.132332412
ARNTL	826	678	0.130295623	0.0790802	0.132598848
RFESD	58	60	0.464061732	0.0790906	0.132598471
AKIP1	1201	971	0.108459181	0.0790966	0.132590638
MIF4GD	65	34	-0.51975284	0.0791429	0.132650384
SMO	1457	1018	-0.102111184	0.0791526	0.132648763
ATP13A1	3779	2713	-0.062963339	0.0792016	0.132713031
MFN2	7288	5297	-0.045195319	0.0792199	0.132725897
STK32B	1508	1055	-0.100241298	0.0794394	0.133075664
RPUSD2	350	301	0.197560697	0.0795125	0.133180173
TXLNG	941	768	0.12206372	0.0795348	0.133199702
LYRM5	1119	907	0.112116551	0.0795656	0.133233359
ZNF454	39	43	0.556014668	0.0796102	0.133290062
RASSF4	264	167	-0.245537695	0.0796243	0.133295818
RAB33B	816	670	0.130744075	0.0798548	0.133663769
CDKN2A	370	317	0.192109701	0.0798553	0.133646588
MFAP3	2749	2167	0.071944378	0.079924	0.133743547
EDEM1	6861	5311	0.045716795	0.0801122	0.13404045
FAM86JP	76	41	-0.475223377	0.0802506	0.134254073
SHFM1	2484	1963	0.075547131	0.0802858	0.134294947
PPIP5K1	1091	885	0.11325039	0.080293	0.134288882
NSUN3	272	238	0.222507054	0.0803662	0.134393181
ESCO1	1018	828	0.117117243	0.0804091	0.134446893
EPS8L1	110	63	-0.388927658	0.0804212	0.134449055
CDC25C	326	210	-0.219330505	0.080486	0.134539344
SRGAP1	9316	7179	0.03922439	0.0805136	0.134567505
LSR	52	26	-0.584847868	0.0806061	0.134704016

RMI1	988	680	-0.123824164	0.0807187	0.134874103
TCF4	3725	2674	-0.063088828	0.0807787	0.1349563
HIC2	522	438	0.162033195	0.0808818	0.135110281
PDPK1	5243	3790	-0.053042566	0.0810155	0.135315503
AARS2	1604	1125	-0.096597009	0.0811147	0.135463017
TRAFD1	3827	2749	-0.062154862	0.0811568	0.135515137
NRIP2	80	79	0.397004785	0.0811736	0.135525034
ZNF180	541	453	0.159034588	0.0812839	0.13569107
MXI1	572	383	-0.163518623	0.0813538	0.135789497
LOC338817	48	51	0.502614973	0.08155	0.136098817
C7orf41	1153	933	0.109708605	0.0817362	0.136391198
UQCRB	6685	5176	0.046062189	0.0817545	0.136403475
ZBTB37	173	157	0.275144653	0.0818242	0.13650154
PDGFA	271	237	0.221746339	0.082008	0.136789896
PSME2	4548	3545	0.055705505	0.0820151	0.136783355
TNFSF13B	79	43	-0.462363862	0.0820285	0.136787397
AAGAB	2580	1836	-0.075652875	0.0820629	0.136826386
PYCR2	1554	1089	-0.097830418	0.0821398	0.136936372
ITGA8	158	95	-0.318773008	0.0821559	0.136944752
GALNT7	4473	3224	-0.057238883	0.0822397	0.137066221
TOM1	1542	1235	0.094860408	0.0825424	0.137552278
ZNF8	373	243	-0.203067185	0.0825845	0.137604087
C21orf2	540	452	0.158515497	0.0825936	0.137600752
WDR35	1075	872	0.113215512	0.0826397	0.137659216
LCAT	393	257	-0.197608821	0.0826416	0.137643909
AKAP1	774	527	-0.139378473	0.0826788	0.137687419
ZNF865	876	600	-0.130816237	0.0826842	0.137678054
OLFML2B	134	79	-0.347156311	0.0827695	0.137801624
CREBBP	5402	4197	0.051016955	0.0828383	0.137897677
GNAZ	79	78	0.396773602	0.0832905	0.138632046
PILRB	887	608	-0.129710649	0.0833172	0.138657877
SERPINB2	974	793	0.118551225	0.0833652	0.138719304

CHSY3	106	101	0.34544316	0.0834253	0.138800679
EEFSEC	400	262	-0.195281056	0.0834479	0.138819843
SIDT2	1771	1412	0.088328008	0.0835422	0.138958001
S100A16	5255	3800	-0.052539214	0.0835598	0.138968859
HERC2P7	222	197	0.242788085	0.0836748	0.139141407
CDC42EP1	2467	1754	-0.076956836	0.0837645	0.139272081
KIN	969	789	0.118680766	0.083816	0.139339122
PJA1	1187	959	0.107434917	0.083828	0.139340428
GLT8D1	3598	2582	-0.063554054	0.0838675	0.139387463
SASS6	427	362	0.176905759	0.0839635	0.139528372
LOC158572	71	38	-0.486667474	0.0839884	0.139551189
MRPL32	1713	1367	0.089640223	0.0840606	0.139652439
RAD18	1558	1247	0.093918364	0.0840792	0.139664731
PPP3CB	5509	4278	0.050298151	0.0842302	0.139896974
ZFP41	705	478	-0.145460508	0.0842317	0.139880858
EMC9	259	164	-0.244104151	0.0842604	0.139909821
PPP1R3D	407	346	0.180895375	0.0842712	0.139909138
LUC7L2	180	110	-0.295341251	0.0843469	0.140016079
RALB	5825	4519	0.048897639	0.0844137	0.140108214
TTBK2	918	749	0.121623697	0.0844454	0.140142193
EIF2S1	6848	4975	-0.045834044	0.0844792	0.140179607
C2CD5	1680	1181	-0.093300137	0.0844986	0.140193175
ATPBD4	272	173	-0.237682482	0.0845895	0.140325355
POC1B	1132	916	0.109697677	0.0845996	0.140323377
MOGS	3277	2347	-0.066405699	0.0849018	0.140805794
EDN1	321	277	0.202464811	0.0849873	0.140928899
GPR39	63	33	-0.517733672	0.0850067	0.140942235
GABPB1-AS1	586	488	0.151132615	0.0851001	0.141078333
PPP4R1L	308	198	-0.222277789	0.0852718	0.141344233
CHAC1	1262	1017	0.103759901	0.0853229	0.141410127
MLX	1996	1411	-0.085241601	0.0853578	0.141449078
NUB1	2507	1979	0.073961748	0.0854755	0.141625361

SNHG9	171	104	-0.302260665	0.0854961	0.14164056
AGPS	3997	3122	0.058705096	0.0856075	0.1418063
LAMTOR3	1618	1293	0.091662799	0.0858892	0.142253948
NOM1	1813	1278	-0.089338957	0.0859354	0.142311514
TSNARE1	74	40	-0.472373139	0.0860597	0.142498537
MARCH9	391	256	-0.195872666	0.0860806	0.142514077
TNFRSF12A	6523	4736	-0.046714416	0.0861792	0.14265848
RPS26	18386	13526	-0.027720255	0.0862906	0.142823864
AKTIP	642	532	0.14400508	0.0863245	0.142860954
RANGAP1	10548	7712	-0.036640388	0.0864357	0.143025944
FBLN5	6268	4548	-0.047620794	0.0864563	0.143041114
MRPS30	1003	815	0.11570249	0.0865277	0.143140223
GPATCH4	1453	1165	0.096447384	0.0865401	0.143141653
DENND5A	5614	4357	0.049458181	0.0865888	0.143203253
DUSP7	1507	1056	-0.09791745	0.0866373	0.143264482
CSNK1D	7288	5301	-0.044106287	0.0866648	0.14329094
LMF1	675	457	-0.147541205	0.0867023	0.143333851
MAD2L2	914	628	-0.126277474	0.0868221	0.143512769
SV2A	2645	1885	-0.073551067	0.0868379	0.143519932
PRPF31	2917	2084	-0.069977977	0.0869121	0.143623527
UTP3	2937	2309	0.068077039	0.0869751	0.14370846
PLCL2	117	110	0.326147126	0.0870018	0.143733492
NOB1	1675	1178	-0.092669425	0.0870565	0.143804903
CCDC88B	35	39	0.571271334	0.0871348	0.143915145
TRIM41	1573	1104	-0.095626367	0.0872201	0.144036876
RRP7B	153	92	-0.318673755	0.0872506	0.144068153
TMEM106A	570	475	0.152117726	0.0873541	0.144220019
STAMBPL1	2079	1472	-0.082959955	0.0873591	0.144209078
IQCG	335	217	-0.211313921	0.0873679	0.144204561
LRRTM2	312	201	-0.219198396	0.0874622	0.144340966
FAM175A	819	560	-0.133284493	0.0874806	0.144352306
SF3A2	1359	949	-0.102913332	0.0875188	0.144396087

RBM12B-AS1	54	56	0.467619552	0.0875627	0.144449484
PEX5	1282	1032	0.102198841	0.0877049	0.144664834
GLI1	77	76	0.396293104	0.0877053	0.1446464
TMEM9B-AS1	66	35	-0.499958971	0.0877092	0.14463372
LOC100505783	62	63	0.438235745	0.0878527	0.144851124
ELP3	1735	1383	0.088017625	0.0878772	0.144872266
ST6GALNAC6	2927	2301	0.06799037	0.087923	0.144928592
ZNF548	579	482	0.15062193	0.0880859	0.145178
SLTM	5126	3707	-0.052429232	0.0881056	0.14519126
GDAP1	184	113	-0.288230862	0.0881642	0.145268571
ZIK1	319	275	0.201027326	0.0882945	0.145463946
TMEM134	500	333	-0.171253786	0.088325	0.145494975
PTN	4392	3423	0.05553537	0.0884273	0.145644338
NCAPG	1808	1275	-0.088745299	0.0884465	0.145656598
SNAPIN	809	553	-0.13370809	0.0884469	0.145638065
CLDN11	1092	884	0.11029755	0.0885129	0.145727445
ZRANB2	6372	4934	0.046163581	0.0885449	0.145760806
ZNF549	419	355	0.176020912	0.0885589	0.145764615
ANKRD36BP1	734	604	0.133920618	0.0886637	0.145917841
MAK16	1060	859	0.111817903	0.0888056	0.146132056
C14orf166	5715	4433	0.048681996	0.0888196	0.14613587
HEXIM1	2498	1971	0.073306431	0.0888923	0.146236173
ZNF280B	175	158	0.267721768	0.0889765	0.146355366
RSG1	178	109	-0.292396974	0.0891039	0.146545544
PI4KAP1	222	139	-0.260322662	0.0891631	0.146623545
BBC3	409	269	-0.189342539	0.0893371	0.146890168
CSPG4	8539	6227	-0.040377715	0.0893515	0.14689448
IFT43	535	447	0.155888072	0.0894389	0.147018799
ICAM5	545	365	-0.163207634	0.0894873	0.147078954
EHD1	7209	5571	0.043289299	0.0895251	0.14712159
DFNB59	80	44	-0.447344345	0.0895885	0.147206414
LINC00839	316	204	-0.216203274	0.0896513	0.147290209

NR2F1	3922	2822	-0.059719416	0.0896843	0.147325048
SNX25	1414	1134	0.096790652	0.089716	0.147357592
PRKAG1	3272	2345	-0.065432694	0.0897587	0.147408278
GK5	2355	1674	-0.0772754	0.0898029	0.147461482
FAM122B	2566	2023	0.072127281	0.08985	0.147519381
KRT19	61	62	0.438611105	0.0901561	0.148002457
ZNF426	639	529	0.142603923	0.0902575	0.14814937
C17orf59	336	218	-0.208980966	0.0903068	0.148210704
MED14	3712	2902	0.060002941	0.0903379	0.148242211
CERS4	213	189	0.242684936	0.090346	0.14823595
ZNF189	1230	991	0.103450779	0.090361	0.148241074
URI1	3357	2630	0.063042394	0.0904424	0.148355122
KISS1	191	171	0.255575819	0.0905137	0.148452559
WDR4	677	459	-0.145509548	0.0905386	0.148473721
VPS4A	5746	4165	-0.049089366	0.0907153	0.148743882
LAMB3	525	351	-0.16569426	0.0907751	0.148822414
CEP350	4816	3746	0.052667639	0.0908107	0.148861113
C17orf100	103	59	-0.388705346	0.090874	0.148945319
ACE	53	27	-0.557880821	0.0909422	0.1490375
CLSPN	1664	1171	-0.091762226	0.0912052	0.149448808
MAP2K5	660	447	-0.147039061	0.0913544	0.149673585
PCM1	8901	6857	0.038762226	0.0913975	0.14972451
SDHD	3111	2441	0.065246032	0.0915138	0.149895317
SPPL2A	2594	2044	0.071368848	0.0915525	0.149939047
ZNF808	422	357	0.173833207	0.0915547	0.149922925
TMEM108	229	144	-0.254126655	0.0915765	0.149938937
RAP2B	3158	2477	0.064734829	0.0916619	0.150058977
C5orf30	971	789	0.115706136	0.0917004	0.150102329
OCIAD1	5924	4591	0.047388923	0.0917764	0.150206928
BST1	281	244	0.211463149	0.0918311	0.150276672
RSAD1	1337	934	-0.102352879	0.0918571	0.150299617
KDM5D	1434	1149	0.095485906	0.0919037	0.150356058

GPN3	801	656	0.127045704	0.0919525	0.150406303
HMGN3	801	656	0.127045704	0.0919525	0.150406303
TMEM178B	52	54	0.469599916	0.0921548	0.150707451
ZFYVE20	2625	1872	-0.072584856	0.0922386	0.15082468
PRR5L	43	46	0.512449333	0.0922446	0.150814707
MAPK8IP2	75	74	0.395786807	0.0923742	0.151006745
MLKL	372	317	0.184332351	0.0923975	0.151025091
ST3GAL4	678	460	-0.14449928	0.0925015	0.151175134
SDAD1	3717	2905	0.059551607	0.0925923	0.151303717
ZNF573	228	201	0.233313809	0.0926074	0.151308588
GTF2IRD2B	148	89	-0.318567803	0.0926892	0.151422346
COL24A1	523	437	0.155974465	0.0929251	0.151787867
NFS1	1082	750	-0.113585867	0.0930386	0.151953325
ZXDA	304	196	-0.218065538	0.0932497	0.152278167
CABLES2	654	443	-0.146831805	0.0932893	0.152312841
PLGRKT	654	443	-0.146831805	0.0932893	0.152312841
TMEM11	950	655	-0.121280475	0.0933136	0.152322479
PPAT	1330	1068	0.098637533	0.0934133	0.152465288
PHLDB2	1110	897	0.107772345	0.0934983	0.152584111
TRHDE-AS1	4273	3330	0.055424992	0.0935154	0.152591945
FDPSL2A	89	50	-0.416725109	0.0935747	0.152668693
NCOA7	1732	1221	-0.089223598	0.0936783	0.152817788
ARHGEF25	961	663	-0.120375429	0.0938309	0.153046581
WDR33	3000	2355	0.065916691	0.0940689	0.153414664
AFAP1L1	527	440	0.154852694	0.0941064	0.153455815
FADS1	2877	2057	-0.068871295	0.0942149	0.153612594
CDK2	2523	1798	-0.073595054	0.0942588	0.153664062
ICAM4	75	41	-0.456114554	0.0942591	0.153634369
TMEM184A	75	41	-0.456114554	0.0942591	0.153634369
NANP	697	574	0.135044213	0.0942744	0.153629197
LOC285033	132	122	0.30149535	0.0942749	0.153609815
CPNE2	1022	707	-0.116460944	0.0943603	0.15372884

TRIM6	469	394	0.163759839	0.0943794	0.153739981
SIGIRR	344	224	-0.203757701	0.0944248	0.153793735
SBDSP1	2352	1857	0.074237887	0.0945149	0.153920462
AGPAT4-IT1	42	45	0.514687805	0.0945443	0.153938079
MAP3K8	42	45	0.514687805	0.0945443	0.153938079
SRSF5	5140	3720	-0.051313606	0.0945663	0.153943654
TMED10P1	227	200	0.232459834	0.0945797	0.153945388
CCDC159	173	106	-0.291555641	0.0946198	0.153990571
VILL	275	176	-0.228704058	0.0946759	0.154061699
ZYG11A	56	29	-0.534221795	0.0947656	0.154187468
U2AF1L4	81	79	0.379082877	0.0948459	0.154298094
PRTFDC1	620	419	-0.15016584	0.0948745	0.154324334
OAT	8505	6554	0.039216569	0.0950065	0.154518875
TMEM104	1955	1551	0.081182211	0.095115	0.15467514
LOC100132832	246	156	-0.241960155	0.0952287	0.154839957
CNOT4	1832	1456	0.083742984	0.0954434	0.155168774
PIGB	669	454	-0.144161782	0.0954471	0.155154531
DISP2	95	54	-0.399815974	0.0955109	0.155237867
ZNF419	812	664	0.124855646	0.0955467	0.155275917
FKBP4	4100	2955	-0.057313648	0.0957241	0.155543926
KCNE4	284	246	0.207919518	0.0958393	0.155710687
LRIG2	733	602	0.13110242	0.0959699	0.15590262
STARD5	164	100	-0.298543683	0.0960232	0.155968824
SENP8	166	150	0.268931391	0.0960441	0.155982504
SLC12A8	143	131	0.288703797	0.0961108	0.156070456
EVA1A	1522	1069	-0.094554374	0.0961637	0.156135902
RHOD	98	56	-0.39220279	0.096295	0.156328783
BRCA1	1139	919	0.105521151	0.0963412	0.156383305
SLC2A11	263	229	0.215436931	0.0963681	0.156406733
WDR55	1034	716	-0.115052561	0.096376	0.156399103
KCTD2	1496	1196	0.092259346	0.0964345	0.156473624
ZNF710	1009	698	-0.116475101	0.0964412	0.156464167

ZNF581	673	457	-0.143260208	0.0964894	0.15652188
TNFAIP8	687	467	-0.141735417	0.0965366	0.156578091
ZNF334	226	199	0.23159779	0.0965932	0.156649501
CASP7	985	799	0.11322391	0.096622	0.156675861
FBXO4	199	177	0.246133061	0.0966687	0.156720973
STEAP1	199	177	0.246133061	0.0966687	0.156720973
FUT2	67	36	-0.481012057	0.0967662	0.156848423
KCNMB3	158	96	-0.303666116	0.0968596	0.156979358
BMP2	506	423	0.15667241	0.0968684	0.156973253
CETN3	709	583	0.132862388	0.0969712	0.157119296
DNAJA2	4711	3405	-0.053226402	0.0969939	0.157135626
SYNGR1	1117	776	-0.110348497	0.0971514	0.157370346
ACAD9	2244	1595	-0.07736412	0.0973478	0.15766793
GTF2E1	769	630	0.127520362	0.0974211	0.157766161
ZNF271	2543	2003	0.070783091	0.097577	0.157998193
GLT8D2	2978	2337	0.065466112	0.0975941	0.158005227
KLHDC2	1711	1362	0.086039075	0.0976831	0.158128842
C16orf88	1042	722	-0.114132404	0.0977183	0.158165244
ZNF266	1357	1088	0.096409968	0.0977835	0.158250126
IMPDH1	2711	2132	0.068534457	0.0978067	0.158267198
EYS	51	26	-0.556833492	0.0978365	0.158284608
MAP3K14-AS1	51	26	-0.556833492	0.0978365	0.158284608
TMEM189	671	553	0.136118846	0.097897	0.158351566
NRG1	1527	1073	-0.093897854	0.0979059	0.158345385
ZNF681	106	100	0.331087867	0.097907	0.158326614
UPF3B	1002	812	0.111821256	0.0980117	0.158475434
YIPF1	1142	921	0.104862542	0.0980333	0.158489719
TMEM254	457	384	0.164064277	0.0980557	0.158505312
PCF11	2200	1739	0.075906541	0.0981397	0.158620474
ZC3H12A	202	126	-0.265779428	0.0981526	0.158620884
CILP2	59	31	-0.513294607	0.0981776	0.158640685
ABCB8	1028	712	-0.114738986	0.0982449	0.158728713

NR2F6	1813	1281	-0.085956318	0.0982924	0.158785003
SNTB1	874	712	0.119396093	0.0985096	0.159115109
LRP8	946	653	-0.11960506	0.0985385	0.159141255
TRMT1L	1868	1483	0.082176274	0.0985931	0.159208723
CENPM	234	148	-0.245759222	0.0985962	0.159193128
GPD2	5188	3757	-0.050445232	0.0986567	0.159270121
MPC1	828	676	0.122544611	0.0986645	0.159262032
MROH6	140	84	-0.321813462	0.0987309	0.159348604
CCDC19	70	38	-0.466203372	0.0990498	0.159842583
BAIAP2	1230	858	-0.104456631	0.0990551	0.159830523
SAMD12	615	416	-0.14885075	0.099089	0.159864448
CEP63	1601	1127	-0.091333661	0.0991118	0.159880542
RECQL	3569	2566	-0.060846599	0.0991229	0.159877734
KCNJ2	642	530	0.138571194	0.0991801	0.159949207
EPOR	329	214	-0.205324656	0.0994009	0.160284649
LOC100129917	174	107	-0.286324378	0.0994029	0.160267133
NXT1	339	221	-0.202086772	0.0995237	0.160441103
PDCD2	2098	1489	-0.079518792	0.09955	0.160462668
EXOSC5	270	173	-0.227035238	0.1001016	0.16133097
DCAF4	646	533	0.1377535	0.1001808	0.16143776
MGAT5	17794	13097	-0.02700234	0.1001866	0.161426219
HTATSF1P2	790	646	0.124833643	0.1002163	0.161453189
TBKBP1	688	566	0.13354562	0.1002681	0.161515627
MPPE1	633	429	-0.14607572	0.1004153	0.161731812
STARD4	877	714	0.118499363	0.1005339	0.161901941
KIAA1024	164	148	0.267053493	0.1005487	0.161904912
WDR59	1478	1038	-0.094687694	0.1006332	0.162020008
TRIM61	141	129	0.286828035	0.1007846	0.162242812
DROSHA	4073	2937	-0.056596417	0.101031	0.162618338
ZNF34	165	101	-0.2929586	0.1010328	0.162600316
LOC100288842	73	40	-0.452744332	0.1010793	0.162643637
NAT8L	73	40	-0.452744332	0.1010793	0.162643637

PLEKHM1	1725	1372	0.084836251	0.1011376	0.162705825
STK33	123	114	0.305527641	0.1014295	0.163154373
LOC100128822	54	28	-0.532380448	0.1019363	0.163948446
PPP1R13L	169	152	0.262200209	0.1019709	0.163982891
RHOG	1479	1039	-0.094274266	0.1019876	0.163988604
FCF1	2356	1858	0.072563094	0.1019985	0.163984997
GPR108	1439	1010	-0.095559167	0.1021229	0.164163678
UBE2D3	15182	11615	0.028779429	0.1022327	0.164319078
ATXN7	2030	1607	0.078042333	0.1022466	0.164320208
KYNU	78	76	0.377677426	0.1025381	0.164767332
C10orf54	306	263	0.196683278	0.1026649	0.164949916
UBE2Q2	4267	3322	0.053982095	0.102696	0.164978575
SLC10A7	483	404	0.157484236	0.1029554	0.165374002
ATG5	1802	1431	0.082576793	0.1030402	0.165488741
ZNF70	420	354	0.168512164	0.1030546	0.165490631
PYROXD1	907	737	0.1157142	0.1030839	0.165516303
CDKN1C	196	174	0.243385783	0.1032124	0.16570124
ZNF124	284	183	-0.218895149	0.1034648	0.166085046
CYB561D2	472	315	-0.168282899	0.1035241	0.166158794
NDUFA4	7339	5663	0.041135164	0.1036745	0.166378726
TTC31	949	656	-0.117560141	0.1037615	0.166496942
LOC100506710	3726	2908	0.057551727	0.1037795	0.166504344
IMPA2	245	156	-0.236083588	0.1038945	0.16666739
SLC9A1	4319	3119	-0.054461632	0.1040387	0.166877221
EIF2AK3	1462	1168	0.091249095	0.104139	0.167016575
BBS5	662	545	0.134577145	0.1041746	0.167052178
ZNF827	1195	961	0.10074985	0.1041868	0.167050334
DPYD	1415	993	-0.095784298	0.1042235	0.167087542
FLT3LG	105	61	-0.368356048	0.1044857	0.167486376
COX5A	2937	2104	-0.066056429	0.104672	0.167763411
TRMT61B	365	310	0.179523883	0.1047737	0.167904828
FAM186B	47	49	0.475273124	0.1047901	0.167909562

ARVCF	435	289	-0.174793776	0.1049041	0.168070557
PEAR1	2227	1758	0.073985644	0.1049301	0.168090596
RNF135	843	580	-0.124327599	0.1052962	0.168655317
CCDC93	2348	1673	-0.073842831	0.1053108	0.168657077
SBF2	3949	3078	0.055657996	0.1054174	0.168806062
SPDYE6	195	173	0.242450046	0.1054898	0.168900381
RHOV	57	30	-0.510847287	0.1055725	0.169011023
RABL2A	103	97	0.328564447	0.1055862	0.169011171
PFDN4	1372	1098	0.093749705	0.1056329	0.169064257
PSMG3	723	494	-0.134332474	0.105685	0.169125796
PIAS1	1427	1002	-0.094950695	0.1057438	0.169198265
KRT80	361	237	-0.191959646	0.1057853	0.169242968
FUT4	462	387	0.159592846	0.1059232	0.169441733
MOB3A	2126	1680	0.075471768	0.105997	0.169537977
PRSS53	166	102	-0.287461958	0.1062012	0.169842875
PHF16	562	466	0.144911956	0.1062985	0.169976563
TCEAL8	3471	2712	0.059157945	0.1064378	0.170177569
TIPIN	418	352	0.167224618	0.1066252	0.170455275
CPM	364	309	0.178820519	0.1066638	0.170495113
LIN54	1949	1382	-0.080824343	0.1067072	0.1705425
NDUFAF6	339	289	0.184936351	0.1069752	0.170948951
LOC100289341	85	48	-0.409276304	0.1070691	0.1710771
PPARD	3394	2440	-0.060953285	0.1071865	0.171242702
RPL22L1	5084	3944	0.048847653	0.107296	0.171395567
TCTN1	748	612	0.125645515	0.107367	0.171487069
LAMC2	83	80	0.362040795	0.1073781	0.17148272
THBS3	2005	1423	-0.079514443	0.1073908	0.171481102
SLBP	2607	1863	-0.069610777	0.1073976	0.17146991
NBPF1	1850	1310	-0.082806327	0.1074736	0.171569223
MRPS15	2094	1488	-0.077734784	0.1076055	0.171757872
GLI4	272	175	-0.221099598	0.1076284	0.171772372
ZNF655	5802	4491	0.045638558	0.1081584	0.172596204

AAAS	1586	1118	-0.089320451	0.1083157	0.172824992
DNAJB5	802	551	-0.126397786	0.1083722	0.172892951
HIF1A	15631	11496	-0.02812598	0.1085894	0.173217289
PPP2R2D	1327	930	-0.097713618	0.108596	0.173205639
GALNS	1524	1073	-0.091060695	0.1086494	0.173268595
TRIM24	1079	870	0.104544573	0.1088871	0.173625548
LRCH4	2413	1900	0.070323635	0.1088886	0.17360572
TBC1D14	1371	962	-0.09596764	0.1090973	0.173916132
ANKRD50	5039	3909	0.04881426	0.1091075	0.173910131
LINC00565	352	231	-0.192530445	0.1092149	0.174058992
DEF8	2564	1832	-0.069824627	0.1092632	0.17411383
TIMM22	1186	828	-0.103249205	0.1093121	0.174169352
RRM1	6777	4934	-0.042736924	0.1093361	0.174185339
CREB5	742	607	0.125429461	0.1094095	0.174280012
SLFN12	2209	1743	0.073331283	0.1095147	0.174425264
FERMT3	36	39	0.530629349	0.1095466	0.174453712
NOXO1	52	27	-0.530400084	0.1097	0.174664518
TMC4	52	27	-0.530400084	0.1097	0.174664518
VAMP4	2460	1756	-0.071213339	0.1097074	0.174642802
ZNF528	1141	918	0.101419399	0.1099351	0.174982929
DAGLA	413	274	-0.176813757	0.1101114	0.175241131
HLA-DMA	173	107	-0.278009109	0.1101324	0.175252237
CYS1	74	41	-0.436749229	0.1102806	0.175465657
FNBP4	3666	2860	0.056960493	0.1102863	0.17545235
FLJ14186	454	303	-0.168222372	0.110466	0.175715792
ZCCHC8	1145	921	0.101077595	0.1105349	0.175802921
SLC16A7	3295	2576	0.06000626	0.1105472	0.175800046
WASH5P	641	436	-0.14084409	0.1106161	0.17588708
NDUFB1	2021	1598	0.076350218	0.1107988	0.176155075
PCIF1	821	565	-0.123979223	0.110926	0.176334755
RPL23AP53	1960	1391	-0.079579103	0.1111292	0.176635366
LAMTOR5	3025	2369	0.062495188	0.1111462	0.176639753

ZNF414	296	192	-0.209338733	0.1112971	0.176857073
TRIM4	1669	1179	-0.086268105	0.1114201	0.177029865
RANBP17	1115	777	-0.105905075	0.1114237	0.177013013
AFTPH	1380	1103	0.091916656	0.1116968	0.177424325
CBR1	1475	1038	-0.091756379	0.1117993	0.177564475
LRRC8B	176	157	0.250341262	0.1118021	0.177546258
NOTCH2	18024	13760	0.025713383	0.1118285	0.1775656
ULBP1	88	84	0.348037936	0.1118589	0.177591185
THAP8	260	167	-0.223511389	0.1119368	0.177692195
FAM72D	109	64	-0.353032193	0.1119424	0.17767846
MOSPD2	1585	1118	-0.08841052	0.1120151	0.177771224
ZNF485	121	72	-0.333786104	0.1121226	0.177919113
LOC728431	115	68	-0.342875078	0.1121723	0.177963957
SUZ12P1	115	68	-0.342875078	0.1121723	0.177963957
AP4M1	520	350	-0.156004569	0.1124045	0.178298207
RUFY1	1503	1198	0.087935031	0.112516	0.178452384
CWC22	1397	1116	0.091157138	0.1125247	0.178443439
PTTG1	1725	1220	-0.084563082	0.1125551	0.178469018
WHSC1L1	4806	3730	0.049491114	0.1125998	0.178517088
ZNRF3	487	406	0.152710087	0.1126309	0.178543707
BCAT2	936	648	-0.115362585	0.1127155	0.178655053
COMMD5	600	407	-0.144781574	0.1128227	0.17880221
CDC73	4706	3407	-0.050847241	0.112913	0.178922611
EIF4A3	2575	1841	-0.068930674	0.1130475	0.179112882
ZNF16	316	270	0.188186981	0.1130701	0.179125964
TAF12	1163	812	-0.103147333	0.1131589	0.179243806
BRAF	790	644	0.120360167	0.1132686	0.1793948
E2F7	2199	1566	-0.07461126	0.1133075	0.179433484
RBM27	3812	2749	-0.056489081	0.1133119	0.179417763
MSC	80	45	-0.414922867	0.1133278	0.179420053
SPATC1L	455	304	-0.16664309	0.1133551	0.179440505
SLC13A4	55	29	-0.508226587	0.1135733	0.179763125

FBXW4	762	523	-0.127827919	0.1135773	0.179746533
SIN3B	3499	2519	-0.058939411	0.1136019	0.179762559
LOC100506054	215	136	-0.245577877	0.113626	0.179777917
POLR2C	2800	2006	-0.065953089	0.1136803	0.179841058
MBD6	1429	1005	-0.092658283	0.1140377	0.180383455
GTF2B	1066	859	0.10367473	0.1140997	0.180458712
ZSCAN12P1	52	53	0.442632868	0.114101	0.18043778
HOXB7	819	564	-0.123016157	0.1141072	0.180424702
ISG15	1451	1021	-0.091912521	0.1141508	0.180470682
C19orf47	469	314	-0.163671232	0.1141959	0.180519055
ZMYND19	844	582	-0.121071714	0.1143104	0.180677143
LYSMD2	365	309	0.174862506	0.1143949	0.180787755
BCL2L2	3051	2388	0.061672788	0.1144242	0.180811167
LINC00665	124	114	0.293845836	0.1144445	0.180820219
PAAF1	962	667	-0.113198001	0.1144727	0.180841872
CRADD	280	241	0.198758451	0.1145121	0.180881259
UBE3C	9433	7249	0.035217453	0.1145872	0.180976891
SNX5	4086	2951	-0.054333152	0.1146973	0.181127733
WDR91	706	483	-0.132492863	0.1147103	0.181125334
NT5C	692	473	-0.133779723	0.1149178	0.181430066
PHC3	6805	4957	-0.041975771	0.114998	0.181533551
ZNF394	678	463	-0.135120948	0.1150908	0.181657041
LOC645212	320	273	0.185981178	0.1151991	0.18180504
ZNF232	251	161	-0.225474544	0.1152621	0.181881328
POLR2H	941	652	-0.114170627	0.1154345	0.182130274
FBXL12	841	580	-0.120900768	0.1154588	0.182145597
GSTM4	274	177	-0.215274401	0.1155125	0.182207207
HMHA1	209	132	-0.247812881	0.1156128	0.182342343
SSSCA1	834	575	-0.121333296	0.1157074	0.182468494
METAP1	3463	2703	0.057691256	0.1159106	0.182765751
ST3GAL2	1927	1368	-0.07913621	0.1161831	0.183172265
ZNF790-AS1	43	45	0.480740473	0.1162202	0.183207444

LOC100862671	171	106	-0.274779929	0.1162369	0.183210727
GNPDA1	1599	1129	-0.086972321	0.1162688	0.183237716
HAUS3	911	738	0.111321894	0.1163066	0.183274103
ASGR1	69	38	-0.445444812	0.1163284	0.183285339
NEFH	248	159	-0.226161223	0.1163881	0.18335621
AGAP7	89	51	-0.388155957	0.1165014	0.183511478
HS3ST3A1	633	431	-0.139365499	0.1166012	0.183645377
C6orf47	947	766	0.109132098	0.1166236	0.183657549
ZYX	20766	15318	-0.023843272	0.1166311	0.183646046
RUVBL2	3899	2814	-0.055329694	0.116672	0.183687263
ZNF547	174	155	0.248333041	0.1169149	0.184046429
ZNF207	7088	5167	-0.040899588	0.1169707	0.184111025
MAP3K4	1541	1087	-0.08836279	0.1173127	0.184626012
WDR67	429	286	-0.169810369	0.1174074	0.184751579
UVSSA	842	581	-0.120129938	0.1175298	0.184920929
FUZ	232	148	-0.233375498	0.1175578	0.184929966
HEY1	232	148	-0.233375498	0.1175578	0.184929966
PPP4R2	2950	2117	-0.063541553	0.1175897	0.18494507
EME2	165	102	-0.278744741	0.117782	0.185224161
POLQ	937	758	0.109300932	0.1179919	0.185530834
C9orf114	828	571	-0.12098789	0.1180498	0.185598322
GEN1	1357	1084	0.091096168	0.1180528	0.185579602
C7orf26	711	487	-0.130775656	0.1180772	0.185594666
ZBTB47	1571	1109	-0.087271683	0.1182658	0.185867599
ABHD3	263	227	0.20278163	0.1183773	0.186019362
ARMC4	200	126	-0.251424135	0.118604	0.18635207
TIMM10	557	377	-0.147960672	0.1186551	0.18640879
MSMP	229	146	-0.234227097	0.1186657	0.186402034
ELOVL4	1201	963	0.096523684	0.118881	0.186716659
GATA3	104	61	-0.354550249	0.1190191	0.186909944
TNFRSF14	92	87	0.334533672	0.1190207	0.186888959
ARL2	481	323	-0.159350597	0.1190841	0.186964812

ZNF550	151	136	0.264210234	0.1191803	0.187092354
LPIN2	4712	3656	0.049078663	0.1192766	0.187219937
GAA	1730	1225	-0.082838157	0.1194322	0.187440576
CD4	4095	3185	0.052582052	0.1195063	0.187533133
C7orf13	173	154	0.247310445	0.1195584	0.187591276
INPP1	1145	800	-0.102123561	0.1195843	0.187608297
ZFP30	1035	834	0.103640653	0.1196211	0.187642404
MNAT1	2060	1626	0.073835052	0.1196575	0.187675914
LYPLAL1	540	447	0.142467556	0.1197639	0.187819072
PCDHGC5	239	153	-0.228326834	0.119818	0.187880268
PIK3R4	1793	1271	-0.081259326	0.1198334	0.187880817
TIA1	6026	4657	0.043352341	0.1200377	0.188177455
MRPL37	3487	2512	-0.057997768	0.1200623	0.188192214
TMEM240	65	64	0.392784319	0.1201612	0.188323637
RPP14	1466	1033	-0.089892718	0.1202796	0.188485402
WDR63	367	310	0.171640284	0.1204173	0.188677572
MED9	523	353	-0.151990631	0.1206223	0.188974894
IRAK2	537	363	-0.149800408	0.1209667	0.189490705
LPP-AS2	194	171	0.233091804	0.1211877	0.189813006
ALKBH3	656	448	-0.135044951	0.1213599	0.190058754
EPHX4	277	238	0.196227729	0.1213604	0.190035749
CACNB1	292	190	-0.204816819	0.1214676	0.190179594
ARV1	637	523	0.130669706	0.1219508	0.190912104
ZNF343	905	627	-0.114300217	0.1222157	0.191302879
ROMO1	1828	1297	-0.079935459	0.1222619	0.191351122
C9orf117	41	43	0.483864882	0.1224243	0.19156917
PPARGC1B	41	43	0.483864882	0.1224243	0.19156917
SS18L1	1256	1005	0.093511169	0.1225357	0.191707414
RAB9B	57	57	0.415152132	0.1225469	0.191700809
IL18BP	144	88	-0.295341251	0.1226408	0.191823607
LRRC28	329	216	-0.19190414	0.1226735	0.191850564
GZF1	1291	1032	0.092106102	0.1231499	0.192571448

AMMECR1L	2471	1941	0.066857239	0.1232032	0.192630635
CYB5D2	859	594	-0.117043069	0.1232806	0.192727503
GALC	1656	1314	0.081414735	0.1234274	0.192932711
ARAP1	3027	2175	-0.061721142	0.1234789	0.192989091
ATG4C	650	533	0.128847947	0.1235429	0.193064794
SCLT1	733	598	0.121484418	0.1235562	0.193061391
GSG1	49	50	0.444298477	0.1235636	0.193048707
ZNF18	486	327	-0.156513546	0.1236645	0.193182081
C11orf83	336	221	-0.189262732	0.1237514	0.193293702
UBAP1	2004	1582	0.074019223	0.1238552	0.193431574
BHLHE40	659	540	0.127833074	0.1239966	0.193628143
ZBTB9	643	439	-0.135445666	0.1240221	0.193643559
PTER	311	265	0.184229911	0.1240857	0.193718622
METTL22	874	708	0.111268212	0.124174	0.193832198
TMEM140	296	193	-0.201844197	0.1242013	0.193850449
TGS1	1922	1366	-0.077498721	0.1243106	0.193996761
SPRED2	2620	2055	0.064723714	0.1245277	0.194311223
CCDC149	1036	834	0.102247418	0.1245839	0.194374543
C21orf119	67	37	-0.441483693	0.124848	0.194762286
SLC29A3	160	143	0.253095374	0.1251392	0.195192014
AMT	160	99	-0.277419343	0.1252025	0.195266288
LOC648987	313	205	-0.195386616	0.1255118	0.19572419
ZSCAN5A	56	30	-0.485312195	0.1258357	0.19620471
DDX58	1488	1050	-0.087833067	0.1260418	0.196501526
DPP8	2627	2060	0.064380269	0.1260559	0.19649894
TNKS1BP1	7882	6066	0.03733588	0.1261003	0.196543512
PTMA	21885	16157	-0.022630906	0.1262082	0.196687099
TTLL11	280	240	0.19275971	0.126262	0.196746328
ZNF35	602	495	0.13281717	0.1263079	0.196793147
VPS29	3233	2327	-0.059250165	0.1263111	0.196773597
LOC286437	315	268	0.182033304	0.126346	0.196791063
OXNAD1	315	268	0.182033304	0.126346	0.196791063

C14orf1	859	696	0.111581306	0.1263615	0.196778308
KPTN	330	217	-0.18961885	0.126466	0.196916341
LHPP	176	110	-0.262919773	0.1265323	0.196995046
KAT7	2127	1676	0.071354247	0.1266291	0.19712107
PCDHGB6	63	62	0.392068519	0.1267551	0.197292594
PHYHD1	111	66	-0.334869615	0.1269322	0.197543486
METTL6	954	663	-0.109828264	0.1270279	0.197667728
RHOBTB2	1131	791	-0.100697198	0.1270731	0.19771343
FZD2	4347	3375	0.050019538	0.1272109	0.19790311
ACOX1	3581	2790	0.055054735	0.1272653	0.19796289
GPATCH3	415	277	-0.168073228	0.1273355	0.198047456
DZIP3	1119	898	0.097729446	0.1274014	0.198125187
MYO9A	2933	2107	-0.062034625	0.1275473	0.198327253
ERCC3	2084	1485	-0.073740215	0.1275661	0.198331743
ZNF32	349	295	0.17264005	0.1276223	0.198394392
DHDDS	2289	1635	-0.070274695	0.1278027	0.198649963
SON	23137	17090	-0.021897284	0.1281089	0.199101076
IL17RA	1861	1471	0.075871323	0.1283945	0.199520147
FBXO34	921	744	0.107253597	0.1284398	0.199565606
DCAF13	3681	2657	-0.05514739	0.1286548	0.19987469
C2orf47	610	501	0.131153492	0.1287034	0.199925305
CCNF	1045	729	-0.104360091	0.1287692	0.200002604
DCTD	4699	3643	0.047925359	0.1288113	0.20004299
WHAMMP1	397	333	0.161535302	0.1291408	0.20052972
CSPP1	1856	1467	0.075824292	0.1291491	0.20051759
CACNG7	215	137	-0.235008635	0.1293203	0.200758309
SS18	6643	5123	0.040306109	0.1293226	0.200736882
RBPMS2	95	89	0.321029954	0.1293644	0.200776743
SLC4A11	167	104	-0.268112443	0.1294256	0.200846668
CTIF	4909	3562	-0.047589519	0.1294709	0.200892028
LOC283143	113	104	0.295412887	0.1296879	0.201203606
FAM76A	416	348	0.157655909	0.1297565	0.201284932

EPN2	3421	2667	0.055951853	0.1297625	0.201269299
SRRM3	142	87	-0.291651492	0.1298127	0.201321978
DRG2	1406	991	-0.0894875	0.1298425	0.201343099
POLR2B	8705	6369	-0.035625294	0.129867	0.20135606
PCNP	10079	7732	0.032713167	0.1299215	0.201415578
RTFDC1	5567	4304	0.043930126	0.1299511	0.201436396
TRPC6	495	334	-0.152428291	0.1299551	0.201417445
RFTN2	164	102	-0.269974531	0.1303718	0.202038138
RETSAT	2759	1980	-0.063492895	0.1304546	0.202141353
CD160	139	85	-0.294398005	0.1304963	0.202168178
ZNF286B	139	85	-0.294398005	0.1304963	0.202168178
NR1H2	1997	1422	-0.074760736	0.1305339	0.202188814
HINT3	1820	1439	0.076280273	0.1305765	0.202229556
ARMC7	321	211	-0.190178167	0.1306602	0.202333997
PKIA	646	529	0.126885689	0.130679	0.202337982
TTF1	1104	772	-0.100915288	0.1308824	0.202627728
RABGAP1	3513	2737	0.05504399	0.1310107	0.202801167
DHX9	13206	9708	-0.028795406	0.1311075	0.202925818
LOC283922	248	160	-0.217116084	0.1311239	0.202925896
CRLF3	938	652	-0.109563827	0.1311702	0.202972424
TMPO-AS1	79	45	-0.39677552	0.1311956	0.202986475
TSPAN13	1223	978	0.092634098	0.1312289	0.203012712
IWS1	4150	3003	-0.05155473	0.1312562	0.203029818
SMIM8	283	242	0.189357126	0.1312592	0.203009258
ZNF365	161	100	-0.271908557	0.1313067	0.203057409
ACVR1B	1680	1331	0.07920147	0.1313222	0.203056253
NARF	1407	992	-0.089058171	0.1315278	0.20334886
ARHGAP1	7202	5257	-0.039005733	0.1316829	0.20356336
ACTG1	214463	160046	-0.007090018	0.1319002	0.203874041
CTSL1	5736	4432	0.043064989	0.1319098	0.20386353
XRCC3	692	475	-0.127692393	0.1319842	0.203953264
PRRX1	4455	3228	-0.049632721	0.132105	0.204114566

NMNAT1	1007	702	-0.105368616	0.1322608	0.204330052
DDR1	2165	1704	0.069710442	0.1322768	0.204329319
CDK11A	308	202	-0.193422926	0.1323253	0.204378947
RNASEH2C	604	412	-0.13675208	0.132383	0.204442718
LCE2A	38	40	0.489152713	0.1323958	0.204437165
SDHAF1	298	195	-0.196686075	0.1324565	0.20450544
ITGBL1	2431	1740	-0.067310456	0.1326003	0.204702087
ATP5H	6209	4523	-0.041928804	0.1326231	0.204711892
GGT7	1276	897	-0.093296307	0.1327302	0.204851944
NAA15	6731	4909	-0.040239558	0.1328023	0.204937814
HERC4	12270	9014	-0.029743762	0.1328571	0.204996988
FAM162A	873	706	0.108838661	0.1329879	0.205173354
PPAPDC2	677	553	0.123275778	0.133017	0.205192817
LINC00630	100	93	0.310454753	0.1330971	0.205290959
SUFU	1858	1321	-0.076967903	0.1333786	0.205699662
SSR2	11414	8379	-0.030802357	0.1333918	0.205694623
CBX8	315	207	-0.190568929	0.1335404	0.205898202
TMED3	5743	4179	-0.043494671	0.1336393	0.206025207
GDPGP1	88	51	-0.371854145	0.1337528	0.206174682
ADAMTS14	292	249	0.185329505	0.1337929	0.206210872
UTP23	1677	1189	-0.080981842	0.1338091	0.206210329
LOC100506314	226	196	0.209683013	0.1339594	0.206416525
MGC57346	157	140	0.2498144	0.1339866	0.206432796
CPQ	1669	1322	0.078890354	0.1341406	0.206644587
STK35	2258	1775	0.06792567	0.1343009	0.20686591
IFT81	1362	1085	0.087120471	0.134522	0.207167986
PANK4	1362	1085	0.087120471	0.134522	0.207167986
OGFOD3	1215	853	-0.095186535	0.1345812	0.207220837
NAA16	1288	906	-0.092397506	0.1346129	0.207243963
DIS3L	857	594	-0.113680142	0.1346548	0.207282809
DLEU2	94	55	-0.358077006	0.1346822	0.20728655
FBXO2	94	55	-0.358077006	0.1346822	0.20728655

PRRT1	149	92	-0.280454433	0.1349004	0.207583958
FBXO5	1117	782	-0.099236541	0.1350039	0.207717571
SPAG1	189	166	0.227949139	0.1350128	0.207705612
ZNF576	431	289	-0.161466245	0.1350614	0.207754667
NOL11	2872	2064	-0.061460647	0.1351464	0.207859804
NTPCR	994	693	-0.105238368	0.1352584	0.208006392
HNRNPA1L2	560	461	0.134492055	0.1352915	0.208031505
SUCLG2	4870	3535	-0.047059426	0.1353137	0.208039978
DCUN1D5	2214	1741	0.068413113	0.1353169	0.208019208
GTPBP3	476	321	-0.153236144	0.1355436	0.208342077
RPL7L1	3635	2829	0.053488944	0.1356129	0.208422851
FBXL5	2959	2128	-0.060459414	0.1356658	0.208478397
LSM12	3393	2447	-0.056395197	0.1358411	0.208722112
GLB1L	256	220	0.196511845	0.1358458	0.208703591
SLMO1	184	116	-0.250428829	0.1358923	0.208749232
SNX18	8829	6781	0.034400133	0.1359864	0.208868008
ARHGEF39	93	87	0.318936816	0.1361613	0.209110831
ZNF484	437	364	0.151457302	0.1361773	0.209109646
CCDC28B	456	379	0.148316156	0.1362642	0.20921734
SDCCAG8	1860	1323	-0.076337428	0.1363347	0.209299692
ANAPC4	1628	1290	0.079422498	0.1363362	0.209276308
AAMP	4112	3193	0.05022442	0.1363506	0.209272553
FLJ27352	53	53	0.415152132	0.1364863	0.20945502
ZNF649	432	360	0.152117726	0.1366824	0.209730115
TBRG1	3530	2548	-0.055150774	0.1367442	0.209799086
SH3GLB2	1158	812	-0.096931489	0.136992	0.210153352
BTN2A2	893	721	0.106491216	0.1372375	0.21050403
NENF	1180	828	-0.095932055	0.1372988	0.210572098
MAGI2	1242	873	-0.093459483	0.1373297	0.210593698
DHRS4L2	418	280	-0.162923983	0.1376396	0.211042923
GATAD2A	4541	3293	-0.048455393	0.1376607	0.211049291
PLIN4	71	40	-0.412666893	0.1377672	0.211186582

KIAA1429	6185	4507	-0.041454013	0.1377945	0.211202326
DPF2	2615	1876	-0.063998987	0.1378301	0.211230915
NDUFB4	3656	2641	-0.054029637	0.1378474	0.211231505
DNAH14	209	182	0.21558764	0.1379941	0.211430304
DGCR2	3327	2399	-0.056636577	0.1380373	0.211470372
PRR3	425	285	-0.16134879	0.1380879	0.211521927
RBM15	493	408	0.142133637	0.1380879	0.21149595
SGCE	1279	900	-0.091867226	0.1382074	0.211652961
RAB40A	57	31	-0.463541572	0.1387035	0.212386496
SEC24B	3864	3003	0.051461512	0.1389313	0.212709232
BARD1	349	294	0.16774125	0.139125	0.21297951
SNAP25	340	225	-0.180457613	0.1392549	0.213152191
ADSSL1	175	110	-0.254699267	0.1393968	0.213343247
MBNL3	596	407	-0.135131404	0.1395491	0.213550115
CRIPAK	512	347	-0.146056016	0.1395924	0.213590117
CLIP1	9635	7391	0.032637039	0.13962	0.213606114
ZNF7	852	591	-0.112543168	0.1396763	0.213665908
PPTC7	1345	1071	0.086504439	0.1398196	0.213858848
RRP1B	3087	2223	-0.058545403	0.1398629	0.213898862
AMIGO1	155	138	0.247552183	0.1402321	0.214437202
RNF32	52	52	0.415152132	0.1402421	0.214426205
FAM120B	2662	1911	-0.063030661	0.1402687	0.214440415
TMEM116	128	78	-0.299445649	0.1403011	0.214463694
ZNF880	234	202	0.202998895	0.1403085	0.214448671
C16orf80	909	733	0.104685036	0.1406109	0.214884518
DDHD2	3286	2561	0.055526904	0.1406279	0.214884057
COQ5	1070	858	0.096590888	0.1407453	0.21503708
VPS37B	2183	1716	0.067889554	0.1411602	0.215644526
ABCA8	59	58	0.390490078	0.141183	0.215652997
GMPPA	2318	1659	-0.067414548	0.1413452	0.215874209
ATP9B	859	694	0.107429663	0.1413791	0.215899611
LOC374443	279	238	0.185848583	0.1416178	0.216237523

C10orf118	2148	1689	0.068327467	0.1417876	0.216470218
DPYSL4	710	489	-0.122832428	0.1418453	0.216531845
TSC22D2	4430	3434	0.047735472	0.1418878	0.216570124
LOC148413	433	291	-0.15819574	0.1419976	0.216711251
TRIM66	871	605	-0.110585445	0.1421324	0.216890394
FOXO3B	409	274	-0.162772818	0.1423096	0.217134198
KANSL1L	649	530	0.122926013	0.1424706	0.217353177
PAXBP1	2018	1589	0.070345082	0.1424917	0.217358798
QTRT1	689	474	-0.124464792	0.1426803	0.217619819
MKRN1	4210	3266	0.048856689	0.1427096	0.217637828
ZNF45	1042	836	0.097371702	0.1427657	0.217696785
UNG	1212	852	-0.093312231	0.1427903	0.217707616
ZNF674	391	327	0.15727416	0.1428721	0.217805631
ASB16-AS1	166	104	-0.259447581	0.1428764	0.217785475
ZNF85	277	181	-0.198744147	0.1428769	0.217759657
PTGES2	1779	1265	-0.076776994	0.1429044	0.217774969
LOC100132247	454	306	-0.154008513	0.1429636	0.217838411
PBX1	3052	2198	-0.058411444	0.1430345	0.217919916
ATRAID	2973	2321	0.057979191	0.1431829	0.218119319
HELQ	675	464	-0.125610565	0.1431998	0.21811825
SIRT3	500	413	0.139365819	0.143288	0.218226042
ATHL1	832	577	-0.112860078	0.1433064	0.21822739
KCNAB3	110	66	-0.321813462	0.1433339	0.218229128
LUZP2	110	66	-0.321813462	0.1433339	0.218229128
CARD11	89	52	-0.360141581	0.1433494	0.218212746
HLCS	2043	1608	0.069730334	0.1435823	0.218540547
NUDT5	1425	1007	-0.085746104	0.1437095	0.218707391
XXYLT1	1019	818	0.098170829	0.1439056	0.218979103
FOCAD	5105	3711	-0.044950828	0.1439868	0.219075927
USP16	3824	2971	0.051018214	0.1444799	0.219799397
INIP	812	657	0.109565775	0.1446077	0.219966833
PRPF40A	10222	7834	0.031295669	0.1446545	0.220011194

CRHR1-IT1	65	63	0.370064242	0.1447168	0.220079019
PARP3	1100	771	-0.097548627	0.1447199	0.220057001
SLC12A9	1573	1115	-0.081322829	0.1448287	0.220195505
FBXO22	1765	1394	0.074714509	0.1448799	0.220246494
PMS1	1196	955	0.09050738	0.1449185	0.220278211
MAU2	2007	1432	-0.071856992	0.1449397	0.22028356
POFUT2	6290	4587	-0.040356978	0.1450415	0.220411458
SKIDA1	43	44	0.448318996	0.1452011	0.220627071
ZSCAN18	772	534	-0.116608974	0.1454725	0.221012452
POP4	1688	1199	-0.078331114	0.1455055	0.221035568
RNF214	1006	703	-0.101881579	0.1455065	0.221010136
ARHGEF26	176	111	-0.249863621	0.1455765	0.221089515
DHX32	1305	920	-0.089191909	0.1457918	0.221389587
TRAPPC8	3374	2627	0.054108359	0.145792	0.221362763
COPS3	3234	2520	0.055256187	0.1457921	0.221336065
BEST1	66	37	-0.419788622	0.1459896	0.221608887
C1orf85	1577	1249	0.078742949	0.1461291	0.221793545
TMEM120A	1068	748	-0.09864934	0.1462223	0.22190793
RAVER2	670	546	0.119891987	0.1463215	0.222031458
CDKN2D	308	203	-0.186298492	0.146682	0.222551309
KIAA0922	1101	772	-0.096989585	0.1468135	0.222723837
INO80E	794	550	-0.114555257	0.1468291	0.22272031
PLA2G6	173	109	-0.251291771	0.1468449	0.2227036
RSC1A1	173	109	-0.251291771	0.1468449	0.2227036
SPATS2	4345	3151	-0.048394291	0.1469614	0.222839566
ACRC	90	84	0.315616458	0.1470837	0.222997912
C11orf1	267	228	0.187346214	0.1471143	0.223017146
UBE2G2	3690	2868	0.05157634	0.1472445	0.22318726
USP53	3491	2716	0.052995254	0.1473804	0.223366073
FARS2	438	295	-0.155063784	0.147507	0.223530824
TMEM53	129	79	-0.292294375	0.147937	0.224155184
NXT2	326	275	0.169711786	0.1479595	0.224161998

CEBPB	2796	2011	-0.060299147	0.1479977	0.224192534
RHBDD1	2104	1654	0.067976662	0.1480405	0.224230072
PMS2P3	199	127	-0.232787802	0.1481704	0.224399556
DARS	3380	2631	0.053740134	0.1482193	0.224446331
TMEM241	459	310	-0.151073806	0.1483017	0.2245438
ANO10	5199	4018	0.043395541	0.1483339	0.224565366
TMEM63B	2237	1756	0.06587972	0.1484656	0.224737426
ENOPH1	2099	1500	-0.069587534	0.1486928	0.225053988
PGM2	1827	1441	0.072745833	0.1488325	0.225238071
COX6C	3967	3079	0.049565602	0.1488336	0.225212358
NPIP	1369	967	-0.08638252	0.1488585	0.225222603
GUF1	2404	1884	0.063514201	0.1488621	0.225200699
ZNF354C	564	385	-0.135684585	0.1490187	0.22541031
FLJ46906	72	41	-0.397220865	0.1491651	0.225604324
CNN3	19631	14952	0.022356941	0.1493094	0.225795183
ORMDL2	886	714	0.103769507	0.1493569	0.225839508
NUF2	1015	710	-0.100436666	0.1493841	0.225853295
ZNF763	163	144	0.236348979	0.1495112	0.226017911
ZNF586	340	286	0.165632532	0.1495895	0.226108912
SF3A1	5207	3788	-0.043863944	0.1498047	0.226406632
WDR5	2014	1438	-0.070847876	0.1498883	0.226505542
METTL9	7890	5773	-0.035551943	0.150145	0.226865947
CXorf56	890	717	0.103319915	0.1502398	0.226981693
LMTK3	75	43	-0.387401804	0.1503534	0.22712571
BCDIN3D	107	98	0.288394989	0.1504978	0.227316221
ZNF670	210	182	0.208701254	0.1505372	0.227348241
CEP250	3683	2664	-0.052135183	0.1505545	0.22734673
AMPD2	4162	3017	-0.049010137	0.1506086	0.227400904
ZFP69	219	141	-0.220083575	0.1506259	0.227399494
PFDN1	3236	2335	-0.055636926	0.1506765	0.227448236
ZNF605	2248	1764	0.065360657	0.150724	0.227492446
SEC31B	401	269	-0.160843932	0.1509775	0.227847381

ERICH1	717	495	-0.119392462	0.1510899	0.227989493
ZNF783	439	296	-0.153471632	0.1511295	0.228021495
FBXO48	114	69	-0.309213426	0.1513852	0.228379758
ABTB1	501	413	0.13648331	0.151544	0.22859157
PARP11	678	467	-0.122710592	0.1515509	0.228574214
TWSG1	18663	13775	-0.022976462	0.1516837	0.22874688
METTL18	230	198	0.199018701	0.1520489	0.229269885
RAB40B	658	536	0.119297548	0.1520594	0.229257888
TCOF1	3260	2353	-0.055218512	0.1522234	0.229477489
ZMYM6	1642	1298	0.075988388	0.1525935	0.230007455
MRPL45P2	105	63	-0.321813462	0.1527629	0.230235004
DYRK4	604	414	-0.12976565	0.1528249	0.230300501
ARID1A	6388	4662	-0.039263177	0.1529902	0.230521799
PC	800	555	-0.112360097	0.1530238	0.230544506
GOSR1	3000	2162	-0.057443846	0.1532232	0.23081707
EHHADH	570	467	0.127612762	0.153276	0.230868572
LOC338758	90	53	-0.34878051	0.1532877	0.230858393
STIL	1115	783	-0.094807366	0.15336	0.230939228
CAMSAP1	3314	2393	-0.054601074	0.1534234	0.231006769
MPDU1	2278	1632	-0.065974558	0.1535033	0.231099163
FAM118B	977	683	-0.101320852	0.1536669	0.231317578
ICT1	1086	762	-0.096009069	0.1536847	0.231316339
SDCBP2-AS1	140	125	0.251653399	0.1538742	0.231573566
IQCK	388	260	-0.162392897	0.153915	0.231607063
ZNF623	1719	1223	-0.075993009	0.1539752	0.23166964
ZNF775	200	128	-0.228704058	0.1540906	0.231815292
C2CD2L	534	364	-0.13774916	0.1541692	0.231905502
ARL10	347	231	-0.171890679	0.1541813	0.231895622
LOC650368	155	97	-0.261059431	0.1543931	0.232186147
CA11	61	34	-0.428122365	0.1546261	0.232494555
YJEFN3	61	34	-0.428122365	0.1546261	0.232494555
MGARP	224	193	0.200254247	0.1548505	0.232789789

NUDT15	1273	898	-0.088292937	0.1549234	0.2328712
SPNS1	1831	1305	-0.073429853	0.1549257	0.232846569
EDA2R	2604	2036	0.060160245	0.1549536	0.232860399
SGSM3	2212	1584	-0.066626918	0.1555543	0.233734908
DENND4C	3867	3001	0.049380686	0.1556115	0.233792617
MTRF1	361	241	-0.167813559	0.1556347	0.23379931
SLC41A2	273	179	-0.193789232	0.1557536	0.233949751
RELT	263	172	-0.197502103	0.1558877	0.234122978
BEND3	177	155	0.223670987	0.1561327	0.234448505
C4orf33	177	155	0.223670987	0.1561327	0.234448505
DNAJC6	48	48	0.415152132	0.1564746	0.234919442
MZT1	2108	1508	-0.068086307	0.1566574	0.235165512
ZNF720	466	385	0.139680623	0.1566924	0.235189663
CYB5R2	64	36	-0.414922867	0.1567036	0.235178065
MYBL2	982	687	-0.100260794	0.1568811	0.235416156
ZNF805	949	762	0.098535042	0.1568831	0.235390731
NRSN2	2168	1552	-0.067084067	0.1569155	0.235411107
FRMD6-AS1	181	115	-0.239203704	0.1569188	0.235387718
ECHDC3	62	60	0.367846417	0.1569763	0.235445531
KIF15	733	594	0.111801864	0.1570982	0.23560006
MAOA	194	124	-0.2305644	0.1571709	0.23568063
DIP2A	2773	1996	-0.059183764	0.1572732	0.235805693
MAPK11	1044	732	-0.097054027	0.1574367	0.236022404
ARHGAP5-AS1	55	54	0.38867992	0.1574743	0.236050295
C1orf112	479	325	-0.144433806	0.1575278	0.236102104
ZNF174	420	283	-0.154435143	0.157737	0.236387292
TAF5	389	261	-0.160568217	0.1579266	0.236642918
ZNF563	146	91	-0.266877787	0.1580675	0.236825496
HEATR5A	4676	3617	0.0446708	0.1581266	0.236885663
TMEM222	1216	857	-0.089623988	0.1583104	0.23713248
NDC80	1238	873	-0.088805624	0.1583378	0.237144976
ECE2	81	76	0.323229642	0.1583791	0.237178307

ARHGAP26	204	131	-0.223850209	0.1585253	0.237368721
GDPD5	287	189	-0.187512371	0.1585893	0.237435892
RHBDF2	273	232	0.180375986	0.1586126	0.237442349
HNRNPR	8647	6337	-0.033247542	0.1587174	0.237570661
ANK1	268	228	0.181952955	0.1587228	0.237550151
KIAA0825	243	208	0.190779346	0.1587495	0.23756162
LOC729013	162	102	-0.252272529	0.1587979	0.237605524
KCND1	277	182	-0.190795394	0.1589827	0.23785346
PREB	1644	1169	-0.076783237	0.158987	0.237831275
C9orf9	118	72	-0.297565916	0.1592895	0.238255136
RABGAP1L	2168	1701	0.065170516	0.1594199	0.238421512
ARF6	8319	6385	0.033428635	0.1596975	0.238808096
L2HGDH	424	286	-0.152896986	0.1598215	0.238964829
DHTKD1	2202	1727	0.064605746	0.159846	0.238972718
LPGAT1	5371	3912	-0.042132219	0.1599015	0.239027027
ZNF600	341	286	0.16139554	0.1599758	0.239109429
PJA2	11972	8805	-0.028117143	0.1603871	0.239695334
RPS6KA5	53	29	-0.454787328	0.160646	0.240053472
LOC100128288	112	68	-0.304739949	0.1607572	0.240190894
CDK11B	536	366	-0.13523722	0.1608552	0.240308463
GDPD3	47	47	0.415152132	0.1608585	0.240284618
SLC25A36	3621	2812	0.050360549	0.161041	0.24052835
PGBD4	165	145	0.228739008	0.1610537	0.240518481
C6orf70	692	478	-0.118609288	0.1611009	0.240560133
TRAPPC2L	1002	702	-0.098187441	0.1612396	0.240738311
MYO18A	4848	3526	-0.044205092	0.1613914	0.240936132
SPEG	3726	2892	0.04959201	0.1615514	0.241146055
MAD2L1	1614	1275	0.0750088	0.1616146	0.241211485
CCDC9	1148	808	-0.091543312	0.1616175	0.241186962
SLC22A5	272	231	0.179438332	0.1616253	0.241169598
CDCA4	794	641	0.106337481	0.1617239	0.241287898
FKBP3	3283	2372	-0.053758608	0.1617735	0.241332985

STX11	39	40	0.451678008	0.1619647	0.241574778
VWA7	39	40	0.451678008	0.1619647	0.241574778
ANKRD34A	393	327	0.149913455	0.1619964	0.241578707
RNF7	4127	2994	-0.047867085	0.1620463	0.241624104
TAPBPL	281	185	-0.187892728	0.1621867	0.241804552
HAUS5	366	245	-0.163909768	0.1622955	0.241937778
TMEM106B	7042	5149	-0.036540825	0.1623973	0.242060662
GRASP	298	197	-0.181964569	0.1628852	0.242758844
METTL8	1154	920	0.088214674	0.162996	0.242894795
PACS2	2157	1545	-0.066267207	0.1630366	0.242926354
MARCH3	332	221	-0.17198474	0.1631611	0.243082745
ELAC1	468	386	0.137244449	0.1633246	0.243297165
FAM131B	380	255	-0.16035004	0.1633766	0.243345493
DUSP12	992	695	-0.098175011	0.1633983	0.243334156
ZNF521	992	695	-0.098175011	0.1633983	0.243334156
FKBP9	10892	8334	0.028964249	0.1636209	0.243622056
MDH1	5260	4060	0.04156906	0.1638427	0.243923141
BLMH	1469	1042	-0.080326987	0.1639213	0.244010978
HDAC8	668	461	-0.11992922	0.1639629	0.24404374
FAM92A1	956	669	-0.099852276	0.1641709	0.244309459
RABGGTA	956	669	-0.099852276	0.1641709	0.244309459
GNB1L	150	94	-0.259077707	0.1642656	0.244406582
NAIF1	430	356	0.142692713	0.1643647	0.2445249
ANTXR2	11046	8119	-0.028997951	0.1644059	0.244557012
VPRBP	1366	967	-0.083217557	0.1645527	0.244746046
BICD1	1208	852	-0.088542987	0.1647025	0.244939628
GUCY1B3	241	206	0.188763323	0.1650877	0.245483186
KBTBD4	377	253	-0.160275007	0.1652405	0.245681043
LYPD1	1201	847	-0.088650145	0.1654272	0.245929286
HECW2	583	476	0.122617822	0.1655888	0.246140203
TRUB2	971	778	0.095450991	0.1656284	0.246169669
ZNF525	169	148	0.223726061	0.1657063	0.246256095

GMEB1	548	375	-0.132133166	0.1657428	0.246280911
EXOC4	4168	3228	0.046437433	0.1658553	0.24641868
ANP32B	5550	4280	0.040275157	0.1659588	0.246542992
NAF1	656	533	0.11559185	0.1661124	0.24674182
SSRP1	6769	4948	-0.036945085	0.1661262	0.246732867
CRH	59	33	-0.423096798	0.1661675	0.24676474
POLR2K	1797	1415	0.070363776	0.1662634	0.246877726
INPP5J	53	52	0.387671395	0.1664091	0.247064598
IGFLR1	38	39	0.452626837	0.1664884	0.247138182
SLC26A4	38	39	0.452626837	0.1664884	0.247138182
IMPA1	1626	1157	-0.075786261	0.1667245	0.247444404
WDR7	2107	1509	-0.066445377	0.1668697	0.247630475
PFAS	1656	1179	-0.074986823	0.1672867	0.248219632
KANSL1	2106	1652	0.064860382	0.1675601	0.248595729
PCID2	2130	1526	-0.065946337	0.1677291	0.248816805
TMEM41A	992	794	0.093951019	0.1679192	0.249069115
FAM21B	62	35	-0.409761162	0.1682551	0.249537616
RAP1GAP2	1331	942	-0.083559475	0.1683408	0.249635036
AXIN2	125	112	0.256722769	0.1688913	0.250406702
LOC100653515	125	112	0.256722769	0.1688913	0.250406702
CCT6P1	136	121	0.246552528	0.168897	0.250370461
ZNF136	503	413	0.130735513	0.1691088	0.250654583
TMEM246	871	608	-0.103449263	0.1691716	0.250717726
RAB2B	1203	957	0.085106319	0.1696134	0.251342694
RRP12	1948	1393	-0.068646288	0.1698604	0.251678721
DNM1P41	65	37	-0.397762316	0.1699661	0.251805436
GJD3	259	170	-0.19226522	0.1700288	0.251868292
KRT34	45	45	0.415152132	0.1700536	0.251860047
SLC22A23	45	45	0.415152132	0.1700536	0.251860047
NAGPA	567	389	-0.128426448	0.1703229	0.252213966
WNK3	72	68	0.332689972	0.1703618	0.2522416
CENPL	638	440	-0.120900768	0.1703622	0.252212192

ACOT13	1299	1031	0.081795034	0.1704118	0.252255661
C19orf66	599	412	-0.124759534	0.1706997	0.252651727
CENPJ	946	758	0.095509797	0.1709114	0.252935135
CCDC101	157	138	0.22905584	0.170966	0.252985855
GPAM	1211	963	0.08456097	0.1709846	0.252983341
GANC	1145	912	0.086910263	0.171075	0.253086979
ARID2	1833	1309	-0.070589557	0.1710775	0.253060656
ZNF829	264	224	0.178112934	0.1711899	0.253196759
CTBP1-AS1	742	515	-0.111694623	0.1712604	0.253271
RMND5B	413	279	-0.150724528	0.1712741	0.253261236
C19orf40	68	39	-0.386908491	0.1713454	0.253336533
HUS1	1167	823	-0.088688094	0.1713593	0.253326973
SYCP2	78	73	0.319574472	0.1714194	0.253385836
BAP1	3231	2512	0.052007845	0.1714241	0.253362703
PPP2R5E	4032	3123	0.046589062	0.1715203	0.253474701
CCDC150	151	95	-0.253396999	0.171863	0.253951152
CRAMP1L	1953	1397	-0.068207797	0.1720214	0.25415494
KNDC1	298	251	0.167527165	0.1722759	0.254500854
CAMK2N2	71	41	-0.377042983	0.1724315	0.254685296
RGS11	71	41	-0.377042983	0.1724315	0.254685296
ARPC5L	2268	1628	-0.063167809	0.1725026	0.254745122
KIAA1731	1370	971	-0.081480561	0.1727844	0.255130973
ARL3	1883	1480	0.067716308	0.1728488	0.255195836
FADS3	1834	1310	-0.070274695	0.172854	0.255173237
STAT5A	51	28	-0.449918288	0.1730107	0.255374261
GIT2	5992	4614	0.038132512	0.173092	0.255464021
FAM24B	74	43	-0.368036479	0.1732578	0.255663247
NAALAD2	74	43	-0.368036479	0.1732578	0.255663247
FAM110A	148	93	-0.255142423	0.1733536	0.255759153
NPB	98	59	-0.316914663	0.1733607	0.255739361
NNT	4875	3549	-0.042837513	0.173457	0.255851201
SH3BP5	151	133	0.232029828	0.1735692	0.255986319

DMTF1	3642	2639	-0.04958744	0.1737375	0.25620414
FAM72B	95	57	-0.321813462	0.1738144	0.256287217
TXNDC15	6141	4727	0.037603393	0.1738584	0.256321752
EPT1	3513	2544	-0.050452774	0.1741173	0.256673065
SSBP2	1086	866	0.088566959	0.1742017	0.256767166
LINC00674	3600	2793	0.048970799	0.1742684	0.256835091
PLA2G4A	83	49	-0.345177455	0.1744519	0.257075002
LOC388796	314	209	-0.172109485	0.1747359	0.257463149
AGFG2	412	341	0.142279534	0.1747537	0.257458834
SH3BP5-AS1	253	215	0.180351407	0.1748001	0.257496756
MAP2	156	137	0.227781996	0.1748289	0.257508749
LOC96610	1068	852	0.08916582	0.1748543	0.257515708
POLDIP3	3650	2831	0.048567417	0.1748908	0.257539047
ZNF781	71	67	0.331494203	0.1749996	0.257668689
UBFD1	8144	6247	0.0325781	0.1752194	0.257961924
GBX2	182	158	0.21113824	0.1755558	0.258426581
GOLIM4	8774	6437	-0.031694106	0.1758184	0.258782505
ASB8	1564	1113	-0.075634788	0.1758927	0.258861406
FBXO6	161	141	0.223786606	0.1759574	0.258925995
HENMT1	54	30	-0.432844775	0.1760917	0.259093014
L3MBTL1	145	128	0.235243042	0.1761407	0.259134429
LSG1	2919	2107	-0.055131765	0.1761637	0.259137748
ZBTB8OS	751	606	0.105657018	0.1763718	0.259413175
FER	872	700	0.098178919	0.1763759	0.259388528
ZNF628	267	176	-0.186112181	0.1770175	0.260301398
MMP3	171	109	-0.234516058	0.1770204	0.260274978
TMEM42	335	224	-0.165510232	0.1771496	0.260434123
CRY1	1232	871	-0.0851055	0.1771789	0.260446563
LOC100131067	207	134	-0.212245635	0.1771905	0.260432802
SNX21	874	611	-0.101308768	0.1772599	0.260504068
PCYOX1L	737	595	0.106377181	0.1774504	0.260753279
GCSHP3	335	280	0.156417863	0.1774597	0.260736135

FOXJ2	2334	1825	0.060244035	0.1775329	0.260813014
DUS3L	460	313	-0.140319072	0.1781032	0.261619904
NRBF2	1312	1040	0.07996794	0.1781338	0.261634039
PTPN18	628	510	0.11488482	0.1781494	0.261626039
FTO	3836	2783	-0.04780968	0.1781674	0.261621594
NDE1	564	460	0.12109083	0.1781742	0.261600874
LRRC37B	222	190	0.190591874	0.1784151	0.261923617
LOC100132077	237	202	0.184620366	0.1784915	0.262004904
DEDD2	768	619	0.10398523	0.1785591	0.262073224
ROR2	57	32	-0.417737882	0.1786368	0.262156447
BTBD19	387	321	0.145391863	0.1787013	0.262220141
VPS37D	181	116	-0.22671276	0.1787863	0.262314032
SQRDL	940	659	-0.09723016	0.1789891	0.262565104
TRMT2B	940	659	-0.09723016	0.1789891	0.262565104
CREB3	3772	2736	-0.048109314	0.1791305	0.26272608
MRPL12	2566	1848	-0.058404282	0.1793526	0.26302093
C5orf22	1732	1363	0.069498764	0.1795524	0.263282967
HIST1H2BC	152	96	-0.247812881	0.179645	0.26338773
ILKAP	864	604	-0.101330631	0.1796663	0.263387867
KIAA1715	5259	4055	0.040065549	0.1797811	0.263525209
C10orf2	485	331	-0.136001399	0.1798609	0.263611137
DOK6	298	198	-0.174659769	0.1798843	0.263614409
ITGB3BP	429	291	-0.144806363	0.1799219	0.263638457
KCNJ8	234	153	-0.197824745	0.1800408	0.263781647
RPS6KL1	57	55	0.363621831	0.1800897	0.263822249
ZC3H15	4694	3625	0.042315285	0.1805705	0.264495474
PRICKLE4	178	114	-0.227691285	0.1806227	0.264540911
TMEM180	60	34	-0.404275623	0.1807161	0.264646536
CCDC82	1701	1339	0.069924951	0.1807909	0.264724923
PSENEN	1252	886	-0.083703827	0.1807917	0.264695009
DENND6B	165	144	0.218754919	0.1808819	0.264795942
TSPAN15	50	49	0.386005786	0.1809167	0.264815711

POMGNT1	4266	3101	-0.044998308	0.1812088	0.265212167
MTMR14	1249	991	0.081335617	0.181359	0.265400877
PSMD5	4312	3135	-0.044739603	0.181365	0.265378436
BCL7A	489	401	0.128919903	0.1813689	0.265352959
NSUN4	767	534	-0.107234704	0.1813818	0.265340604
C11orf30	1445	1142	0.07564529	0.1816341	0.265678506
NAT1	312	208	-0.169810369	0.1817867	0.26587056
TMEM86B	130	81	-0.267365678	0.1820953	0.266290614
INPP4A	2027	1589	0.063925168	0.1821337	0.26631545
FAM21C	2568	1850	-0.0579678	0.1824215	0.266705
LOC100506394	820	659	0.099806687	0.182473	0.266749033
ABCA5	105	95	0.270762222	0.1824945	0.266749076
NDST2	1297	919	-0.081889581	0.1825135	0.266745571
PSPH	1086	865	0.086900066	0.1825755	0.266804851
TMEM183A	154	135	0.225181188	0.1828172	0.267126778
EBF4	63	60	0.344762804	0.1829208	0.267246788
GLTSCR1L	1318	1044	0.078923473	0.1829488	0.267256239
HR	302	201	-0.172200916	0.1829775	0.267266901
METTL5	887	621	-0.099188704	0.1830962	0.267408923
USPL1	1296	1027	0.079522595	0.1832773	0.267642031
UBE2H	12239	9346	0.026087388	0.1837453	0.26829399
DLAT	3545	2749	0.048273411	0.1839019	0.268491215
ZNF285	159	101	-0.239519341	0.1840537	0.268681296
YY2	444	302	-0.140858995	0.184192	0.268851744
IMMT	5776	4218	-0.038359515	0.1842456	0.268898349
AHSA1	4045	3130	0.045175086	0.1842947	0.268938557
HSD17B7	143	126	0.232560718	0.184364	0.26900817
LAPTM4B	6112	4702	0.036782127	0.1845499	0.26924781
MRPS18B	1991	1561	0.064129448	0.1846459	0.269356427
RMND1	1059	746	-0.090302922	0.1847166	0.269427897
RAB3D	779	543	-0.105518999	0.1849155	0.269686451
TEAD3	2115	1656	0.062197141	0.1849619	0.269722522

NDUFS1	6078	4676	0.036830387	0.1851955	0.270031661
NDUFS2	4300	3127	-0.044405309	0.1852353	0.270058048
CCDC78	72	42	-0.362455447	0.1854395	0.270324076
KIF23	3697	2682	-0.047893675	0.1854695	0.270336127
CLDN23	90	54	-0.321813462	0.1855779	0.270462573
SBNO1	7555	5797	0.033026862	0.1855822	0.270437167
ZNF610	230	196	0.184371925	0.1856506	0.270505244
SYBU	75	44	-0.35423494	0.1859258	0.270874505
KIF5A	87	52	-0.327351646	0.1859508	0.270879214
CSTF1	1454	1034	-0.076638952	0.1861217	0.271096508
KCTD7	1177	832	-0.085306755	0.1861355	0.271084868
ST7	570	464	0.118315018	0.1862091	0.271160383
ZNF234	836	671	0.097961956	0.1865613	0.27164142
C7orf43	921	646	-0.09651486	0.1866346	0.271716464
PCDHGA7	661	458	-0.114150542	0.1871153	0.272384365
GPC1	22731	17273	0.019009652	0.187297	0.272617001
CPEB2	7542	5527	-0.033298406	0.187418	0.272761341
CDC42SE2	1943	1392	-0.065974558	0.1874553	0.27278369
BABAM1	3185	2305	-0.05137449	0.1876652	0.27305729
PECR	320	214	-0.165308977	0.1876828	0.273050989
NECAB1	137	86	-0.256615197	0.1877855	0.273168425
HMGN1	11383	8378	-0.02705091	0.1877939	0.273148854
SMCR7L	3373	2617	0.049033738	0.1879105	0.273286456
DHX33	2285	1643	-0.060709553	0.1879787	0.273353801
CLEC3B	62	59	0.343598871	0.1880139	0.273373015
C5orf54	259	219	0.173130904	0.188066	0.273416811
C1RL	1070	852	0.086466671	0.1880685	0.273388523
IPW	2194	1716	0.060638159	0.1881857	0.273526958
C9orf85	379	314	0.143718842	0.1884771	0.27391852
PARD6B	98	89	0.276175719	0.1885459	0.273986603
DDIT3	601	488	0.114668289	0.1886175	0.274058743
LINC00467	142	125	0.231189297	0.1886192	0.274029148

WWP2	1738	1242	-0.069610777	0.1886613	0.274058404
ZNF614	610	495	0.113771414	0.188872	0.274332449
NUP155	2155	1686	0.061068799	0.1889887	0.274469999
TTYH3	14733	10869	-0.023679885	0.1893379	0.274945094
TTC26	312	261	0.157655909	0.1894926	0.275137608
C16orf71	52	29	-0.427306591	0.1895643	0.275209597
MORN1	92	84	0.283907598	0.189713	0.275393431
L3MBTL2	1389	987	-0.077772478	0.1898228	0.275520696
MBD5	1530	1090	-0.074051386	0.1898407	0.275514517
DLD	5948	4576	0.036834536	0.189916	0.275591731
ZBTB49	229	150	-0.195232966	0.1903848	0.276239804
PABPC5	55	53	0.361712873	0.1903969	0.276209134
TMC7	55	53	0.361712873	0.1903969	0.276209134
INSR	1043	735	-0.089770871	0.1906432	0.276518068
RASGRP3	186	120	-0.217116084	0.1910685	0.277102737
HAPLN3	536	437	0.120552411	0.1913511	0.277480194
LOC100129794	48	47	0.384778483	0.1913961	0.2775132
LOC388692	321	268	0.154811835	0.1914781	0.277599815
RPAP1	1464	1042	-0.075408144	0.1916714	0.277847723
ZFP112	476	390	0.127664682	0.191708	0.277868406
ACTR8	1892	1484	0.064731135	0.1917392	0.277881347
MED29	2651	2065	0.054757243	0.191918	0.278107997
HDAC2	8428	6457	0.030825845	0.1920667	0.278291184
NGFRAP1	9116	6696	-0.029949265	0.1921287	0.278348608
ZFYVE28	297	198	-0.169810369	0.1926497	0.27907101
ABCC3	321	215	-0.163084506	0.1928249	0.279292231
PCDHGB1	330	275	0.152117726	0.1933445	0.280012315
TMEM185B	2916	2108	-0.052963721	0.1938449	0.280704333
NXPH4	270	179	-0.177847688	0.193898	0.280748591
HSF2BP	58	33	-0.398434744	0.1941655	0.281103292
MYO1B	12695	9687	0.025013584	0.1943265	0.281303707
MLXIP	2097	1506	-0.06245296	0.1946023	0.281670215

OSR1	1963	1538	0.063147462	0.1946887	0.281762422
PCDHGA5	108	97	0.260177472	0.1947036	0.281751342
P4HTM	997	702	-0.090970342	0.194865	0.281952157
CACNA1C	703	489	-0.108538092	0.1953904	0.282679496
NOP14	2478	1786	-0.057291975	0.1954432	0.282722954
HES4	356	240	-0.153690704	0.1954797	0.282742964
TMEM144	85	78	0.291163414	0.1955299	0.282782696
ZNF823	252	213	0.172581828	0.1956064	0.28286049
CMYA5	54	52	0.360704348	0.1958023	0.283094465
DPY19L2P2	54	52	0.360704348	0.1958023	0.283094465
KANSL2	1066	848	0.085080864	0.1961895	0.283604958
PGBD2	310	259	0.155836014	0.196231	0.283631968
TIGD7	428	352	0.133116764	0.1963374	0.283752878
C22orf32	610	422	-0.116414112	0.1964019	0.283796647
COQ6	610	422	-0.116414112	0.1964019	0.283796647
RDH14	138	87	-0.250428829	0.1965402	0.283947132
TTC3P1	102	92	0.266288746	0.1966269	0.284039491
UBE2R2	6235	4561	-0.035887258	0.1966368	0.284020812
HBEGF	778	625	0.099238166	0.1966818	0.284052835
SEC61A1	44916	33995	0.013245247	0.1968778	0.284303022
STRIP1	2294	1651	-0.059373139	0.1969007	0.284303052
TMEM120B	1552	1107	-0.072321204	0.1970582	0.284497564
KCTD6	426	290	-0.139648398	0.1971019	0.284527645
REL	113	101	0.253184652	0.19731	0.284795041
RNPC3	571	394	-0.120142984	0.1973658	0.284842479
NUDT18	343	285	0.147905475	0.1975859	0.285127118
CWF19L2	831	666	0.095825832	0.1978599	0.285489495
HNRNPU	28355	21511	0.01662357	0.1979491	0.285585152
PDLIM7	10588	7791	-0.027397548	0.1982698	0.286014576
UBE2D2	4767	3475	-0.040916516	0.198414	0.28618947
FANCI	1829	1310	-0.066336132	0.1985067	0.286289998
RAB43	70	41	-0.356578881	0.1985094	0.286260831

EFNA4	237	156	-0.188188898	0.1985747	0.286321835
GOLM1	5027	3874	0.039278397	0.1985959	0.286319307
TMEM200B	329	221	-0.158889083	0.1986478	0.286360988
GUSBP4	82	49	-0.327690029	0.1986751	0.286367197
FIG4	1183	938	0.080361886	0.1987928	0.286503642
BAG5	2779	2008	-0.053654434	0.1988671	0.286577603
COG8	838	587	-0.098437609	0.1990174	0.28676094
SYTL3	285	239	0.161200831	0.199489	0.287407183
CYP2U1	1870	1466	0.063998965	0.1994934	0.287380266
PTPMT1	1095	870	0.083308568	0.1997492	0.287715601
THOC5	2526	1822	-0.056179548	0.1998461	0.287821876
CENPT	2613	1886	-0.055225317	0.1998615	0.287810687
LRRC59	14312	10560	-0.023463326	0.1998906	0.287819415
DCAF5	3072	2224	-0.050869296	0.2000809	0.28806
NEIL2	1076	760	-0.086454622	0.200245	0.288263048
PHLPP1	629	436	-0.11357975	0.2003604	0.288395839
UBAC1	1390	989	-0.075890325	0.2004566	0.28850094
CCNJ	892	713	0.092010498	0.2004981	0.288510684
MRPL54	892	713	0.092010498	0.2004981	0.288510684
CCDC74A	84	77	0.28962125	0.2006864	0.288714842
FABP3	84	77	0.28962125	0.2006864	0.288714842
ATF3	206	176	0.188083223	0.2010258	0.289153115
PIGX	794	637	0.097306497	0.2010887	0.289210194
PRADC1	597	413	-0.116437018	0.2011517	0.28926737
LOC100288198	53	51	0.359657019	0.2013845	0.289568641
NDUFB2-AS1	39	39	0.415152132	0.2014992	0.289700159
LOC729970	110	68	-0.278744741	0.2015586	0.289752164
TAZ	399	271	-0.142943763	0.2015899	0.289763679
VPS33A	1670	1194	-0.068893134	0.2021499	0.290535127
KCNH1	46	45	0.383443272	0.2025855	0.2911276
CLCN7	2808	2030	-0.052911076	0.2027542	0.291336429
DFFA	1775	1271	-0.066702863	0.2027998	0.291368257

NOL7	2846	2212	0.051567855	0.2031549	0.29184476
DBI	4353	3170	-0.042375048	0.2032131	0.291894704
NGLY1	1281	1013	0.07651583	0.2032588	0.291926719
ZNF33B	926	739	0.089714303	0.2036407	0.292441476
C2orf68	612	424	-0.114315256	0.2036861	0.29247293
PIGN	2023	1453	-0.062309485	0.2036944	0.292451224
LOC729737	59	56	0.339864004	0.2042803	0.29325859
SUB1	8326	6376	0.030180203	0.2043441	0.293316327
PHF15	1802	1291	-0.065957879	0.2044064	0.293371971
SACM1L	3809	2946	0.044497274	0.2044935	0.293463202
JUP	3635	2640	-0.046265303	0.2045593	0.293523741
C1QTNF1	818	573	-0.098413572	0.2045722	0.293508498
PRSS35	416	342	0.132564928	0.2046504	0.293586837
DPM1	2268	1770	0.057480852	0.2049353	0.293961628
DUSP22	238	157	-0.185044883	0.204977	0.293987562
NOD1	205	175	0.186883144	0.2051106	0.294145379
ENOSF1	685	552	0.103716411	0.2051222	0.294128147
PLXNB1	2988	2163	-0.050994351	0.2052412	0.294264853
FDX1	1345	1062	0.074329725	0.2055164	0.294625547
PPWD1	1423	1122	0.072289146	0.2055178	0.29459367
HOXA4	101	62	-0.288863041	0.2055263	0.294571929
ZC4H2	449	307	-0.133324658	0.2056434	0.294705784
CARS2	2488	1795	-0.05585051	0.2058962	0.295034233
KIAA1377	832	666	0.094090781	0.2059653	0.295099245
SLC25A26	407	277	-0.139990686	0.2063108	0.295560283
CNTD1	53	30	-0.405877727	0.2067023	0.296070082
PTGDS	53	30	-0.405877727	0.2067023	0.296070082
RABEPK	1352	962	-0.075834221	0.2070535	0.29652197
GGNBP2	4012	3100	0.043098741	0.207311	0.29685656
G0S2	235	155	-0.18524041	0.2073906	0.296936378
KLHL29	1409	1111	0.072339337	0.2074425	0.296976552
CEBPG	1357	1071	0.073689891	0.2074747	0.296988501

MFSD11	1293	919	-0.077433377	0.2078348	0.297469829
ZNF607	387	319	0.136374989	0.2078874	0.297510871
ARHGEF28	5837	4269	-0.036176774	0.2079172	0.297519324
VPS33B	830	582	-0.096940052	0.2079972	0.297599704
THAP4	1684	1205	-0.06770686	0.2080338	0.29761789
CTU1	185	120	-0.209338733	0.2080688	0.297633757
RPL35A	20097	14870	-0.019423379	0.2082447	0.29785119
LOC283663	45	44	0.382730654	0.2084656	0.298132832
MPND	376	255	-0.145083283	0.2084749	0.298111872
ERCC6L	852	598	-0.095555814	0.2084802	0.298085252
RGS20	925	651	-0.09164369	0.2084894	0.298064151
STMN3	1127	798	-0.082874732	0.2086241	0.2982225
LRRC8D	1417	1117	0.071941559	0.208694	0.298288196
CEP152	707	493	-0.104970437	0.2089092	0.298561506
MAGOHB	1060	842	0.082980005	0.2089132	0.298533082
ING1	617	428	-0.112507561	0.2089301	0.298522993
ECD	2139	1539	-0.059791119	0.2095683	0.299400438
TNFAIP2	776	543	-0.099952323	0.2096171	0.299435793
ZNF692	628	436	-0.111284292	0.2097886	0.2996465
MRPL16	1279	909	-0.077511933	0.2098574	0.2997103
ZNF41	729	509	-0.103101027	0.2099678	0.29983357
MRPS16	3410	2475	-0.047191082	0.2101469	0.300055006
ZNF90	59	34	-0.380028076	0.2102078	0.300107588
ODF2	2919	2113	-0.051029312	0.2102428	0.300123074
NBPF9	1054	745	-0.085410405	0.2103488	0.300240046
NPTXR	683	550	0.102698172	0.2103518	0.300209818
GPATCH11	620	501	0.10769452	0.21036	0.300187228
TLCD1	182	118	-0.209999459	0.2104088	0.300222457
AGER	114	71	-0.267990763	0.2104573	0.300257263
NHSL1	769	538	-0.100225294	0.2104609	0.300227975
ARHGEF2	3437	2495	-0.046957904	0.210589	0.300376371
ZC3H14	4164	3215	0.042000801	0.2106806	0.300472523

IL18R1	86	52	-0.310672905	0.2107009	0.300449959
NMB	86	52	-0.310672905	0.2107009	0.300449959
SMPD2	158	137	0.209403467	0.2108062	0.300548396
PDCD2L	202	132	-0.198665232	0.2110426	0.30085109
TP53BP2	2874	2232	0.050429097	0.2111636	0.30098908
ARID1B	3655	2656	-0.045464128	0.2111907	0.300993338
SDF2	1927	1508	0.061431989	0.2114424	0.301317525
DNALI1	633	511	0.106269923	0.2118152	0.301814245
SEC11A	6350	4650	-0.034373744	0.2118773	0.301868188
CACHD1	460	376	0.124250932	0.2119006	0.301866905
GYG1	5914	4327	-0.035614991	0.2120198	0.302002182
ATP6V0E2-AS1	65	38	-0.359288168	0.2120803	0.302053744
RABL5	736	591	0.098604496	0.2121867	0.302170747
MPP3	454	311	-0.130625585	0.2122205	0.302184357
TMLHE	909	725	0.088852832	0.2123083	0.302274823
TTC38	1110	786	-0.082806327	0.2124623	0.302459458
LINC00842	342	231	-0.150951342	0.2124693	0.30243482
DCAF16	1622	1274	0.06674359	0.2124968	0.302439471
HDAC11	493	339	-0.125150241	0.2125246	0.302444411
KRTAP2-1	68	40	-0.350382615	0.2125354	0.302408061
LOC100508120	68	40	-0.350382615	0.2125354	0.302408061
UIMC1	1272	1005	0.075248963	0.2125519	0.302379689
CCDC121	71	42	-0.342277565	0.2127244	0.302573214
MROH8	71	42	-0.342277565	0.2127244	0.302573214
KRT10	1284	913	-0.076806305	0.2130873	0.303037434
FOXC2	426	291	-0.134682146	0.2131123	0.303021133
N6AMT1	426	291	-0.134682146	0.2131123	0.303021133
TIFA	262	220	0.163088844	0.2131449	0.30301566
USP49	189	123	-0.204575787	0.2133133	0.303220347
WRN	2007	1569	0.059956867	0.2133728	0.30327031
EID3	108	67	-0.27364618	0.2137163	0.303723873
REEP4	1214	862	-0.078856515	0.2140225	0.304124346

PPP1R16A	658	458	-0.107587854	0.2142707	0.304442404
CTSF	640	445	-0.109114437	0.2142907	0.304436085
SLC35G2	596	482	0.108872948	0.214316	0.304437185
AAMDC	579	401	-0.11480898	0.2143814	0.304495368
FAM127C	2387	1722	-0.055961292	0.2144498	0.30455784
FRG1B	332	224	-0.152532378	0.2146179	0.304761754
NBR2	152	132	0.211618738	0.2146714	0.304803023
TMEM135	1327	1047	0.073245203	0.2153788	0.305772559
ZNF560	57	54	0.33714962	0.2160122	0.306636891
TCTA	1868	1341	-0.063033086	0.2166599	0.307521228
HIST1H2BD	403	331	0.13120351	0.2167099	0.30755718
ZNF615	389	320	0.133453882	0.2168397	0.307706356
SLC4A1AP	2171	1694	0.05722628	0.2169557	0.307835839
GYG2	1148	909	0.078381689	0.2171168	0.308029331
DOCK1	7770	5951	0.030369651	0.2172439	0.30817451
RPP40	487	335	-0.124608545	0.2172786	0.308188705
MRPS33	1043	828	0.082115647	0.2174736	0.30843011
FAM53C	2937	2279	0.049209791	0.2175083	0.308444282
GPR75	173	112	-0.212121174	0.2175884	0.308522716
THAP3	512	353	-0.121323495	0.2177924	0.308776815
EIF2B4	1595	1141	-0.068105501	0.2178006	0.308753312
WDR53	343	232	-0.148931639	0.2178181	0.308743011
WWOX	75	69	0.294857898	0.2179169	0.308847807
CHAC2	240	159	-0.178855509	0.2181133	0.309091
EPM2AIP1	4671	3599	0.039016789	0.2183631	0.309409862
BRE	965	681	-0.087722012	0.2185099	0.30958269
NMT1	7242	5313	-0.031709332	0.2185247	0.309568384
CDK10	818	654	0.092341924	0.2185586	0.309581215
SRSF6	9537	7289	0.027337515	0.2187338	0.309794147
LRRC8E	498	343	-0.122785034	0.2187781	0.309821629
ARHGEF6	393	323	0.132156984	0.2190833	0.310218564
ASTE1	230	152	-0.182410406	0.2191334	0.310254292

KDELR3	8898	6805	0.028266195	0.2192475	0.310380559
ARHGAP6	319	215	-0.154067632	0.2193175	0.310444241
WDR76	892	628	-0.091127019	0.2193578	0.310466053
MRPS7	1577	1128	-0.068263461	0.2193598	0.310433636
GGACT	92	83	0.266629607	0.219471	0.310555759
PRDM15	751	602	0.096102711	0.219553	0.31063646
ADI1	5606	4309	0.035533486	0.2196896	0.310794342
SLC5A6	1039	735	-0.084227367	0.2199404	0.31111382
RCN2	6234	4566	-0.034075164	0.2200926	0.311293789
FBXW8	2186	1705	0.05663047	0.2202377	0.311463682
SUGT1	3276	2537	0.046340294	0.2202528	0.311449695
OTUB1	2758	1996	-0.051358604	0.2203332	0.311527917
CSRNP1	888	708	0.088341815	0.2203668	0.31154017
MMS19	3089	2240	-0.048489007	0.2204827	0.311668599
TM9SF4	5586	4086	-0.035974238	0.2205441	0.311719984
LPCAT3	1261	897	-0.076236254	0.2206199	0.311791721
CELSR3	403	275	-0.136196088	0.2208515	0.312083735
PIGV	595	413	-0.111595755	0.2209141	0.312136711
ENDOG	115	72	-0.260412918	0.2210165	0.312245981
LEPREL4	2599	1879	-0.052839435	0.2210359	0.312237942
LOC654342	368	250	-0.14262554	0.2210429	0.312212411
EHMT1	2998	2173	-0.049160077	0.2210937	0.312248751
SAFB2	2213	1595	-0.057294895	0.2211333	0.312269298
MACROD1	157	101	-0.221257134	0.2212477	0.312395408
NBAS	6309	4842	0.03334386	0.2219538	0.31335692
BEND7	261	174	-0.169810369	0.2219823	0.313361623
ZNF416	279	233	0.155216965	0.2220698	0.313449641
SEMA4C	1084	768	-0.082034409	0.2221574	0.313537763
SMC3	4751	3659	0.038370341	0.2222054	0.313569995
GALNT11	2157	1554	-0.057887541	0.2223477	0.313735264
GDF15	1121	795	-0.080607381	0.2223975	0.313769986
EID2B	191	163	0.186451458	0.2223983	0.313735581

FLRT1	51	29	-0.399292215	0.2224929	0.313833464
ZSCAN21	167	108	-0.213664659	0.2225086	0.313819952
ZNF333	617	429	-0.10914071	0.2226081	0.313924827
PSMD1	12190	9295	0.023980776	0.2228168	0.314183551
RILP	154	99	-0.222277789	0.2236623	0.315340103
FAM57A	1177	836	-0.078387341	0.223864	0.315588749
COQ2	704	492	-0.101764982	0.2238756	0.315569283
SNUPN	1000	707	-0.085065748	0.2242724	0.31609281
DDX51	715	500	-0.100863015	0.2243362	0.316146989
TRMT13	709	569	0.097795157	0.2246497	0.316553022
NCOA6	3260	2524	0.045992078	0.2249018	0.316872446
ITPA	1572	1125	-0.067524084	0.2250699	0.317073334
MLLT3	1111	788	-0.08043915	0.225427	0.317540497
POT1	982	694	-0.08563523	0.2254354	0.31751644
KIAA0141	1891	1359	-0.061451775	0.2254399	0.317486845
PDLIM1	10610	7816	-0.025770152	0.2256356	0.317726554
TMEM107	334	226	-0.148373198	0.2256531	0.317715214
ANKRD20A9P	57	33	-0.373343763	0.2257751	0.317833163
PTP4A3	57	33	-0.373343763	0.2257751	0.317833163
CRKL	4355	3176	-0.040309675	0.2258408	0.317871811
TEX264	1030	729	-0.083501486	0.2258998	0.317918808
SORCS2	134	117	0.219427661	0.2264075	0.318597411
M6PR	2754	1994	-0.050711018	0.2264915	0.318679519
THG1L	443	304	-0.128083243	0.2267296	0.318978597
DGUOK	1498	1071	-0.068927012	0.2268285	0.319081652
ORAI1	803	564	-0.094552693	0.2268768	0.319113526
RPRD1A	3578	2602	-0.044380294	0.2271269	0.319429218
CSNK2A1	6221	4774	0.033204192	0.2271661	0.319448345
NPHP3	819	654	0.090579316	0.2273345	0.319649101
CCDC28A	446	364	0.122046872	0.2274527	0.319779073
ASH1L-AS1	69	41	-0.33582032	0.2275835	0.319926904
RALGPS2	5316	4087	0.03585327	0.2278151	0.320216298

RAB7A	15745	11637	-0.021022444	0.2281398	0.320636615
PAQR7	1536	1099	-0.067834698	0.2283437	0.320886987
LOC153684	103	64	-0.271348395	0.228369	0.320886269
FUCA1	234	197	0.166839232	0.2284356	0.32094369
ZNF37A	2427	1888	0.052836788	0.2284408	0.320914797
SORL1	983	695	-0.085026307	0.2284654	0.320913117
MSX1	201	132	-0.19150544	0.2285378	0.32097859
SLC5A3	3958	2883	-0.042048644	0.2285653	0.320981004
GUSB	906	639	-0.088542987	0.2288692	0.321371535
LOC440300	473	385	0.118170394	0.2291092	0.321672327
ZDHHC5	7640	5611	-0.030162594	0.2291702	0.321721669
GLRX3	4624	3561	0.03829317	0.2294335	0.322055076
NR4A1	119	75	-0.250846941	0.2296775	0.322361163
PDCD7	1350	963	-0.072199572	0.2297108	0.322371611
BRI3	4750	3469	-0.038255541	0.2298089	0.322472898
VN1R1	61	57	0.317304808	0.2299813	0.322678516
MAZ	3709	2865	0.042657003	0.2301705	0.322907625
RNF213	12098	9223	0.023691569	0.2302343	0.322960697
TMEM79	214	181	0.173531032	0.2303858	0.323136825
MBP	112	99	0.23715383	0.2305546	0.323337158
TTLL12	1302	928	-0.073380606	0.2306807	0.323477661
HHAT	248	208	0.16139554	0.2307935	0.323599403
MVK	371	305	0.132542188	0.231059	0.323935112
PRPSAP2	1315	1036	0.071113335	0.2313233	0.324269148
PGPEP1	2639	1910	-0.051266581	0.2313926	0.324329884
TYSND1	508	351	-0.118205335	0.2314267	0.324341191
GTF2F1	4509	3291	-0.039129352	0.2314893	0.324392307
CNDP2	3293	2548	0.045114897	0.2315877	0.324493773
PM20D2	1111	879	0.077228386	0.2316678	0.324569412
ATP5G2	5661	4348	0.034447148	0.2319194	0.324885435
MPHOSPH9	1457	1145	0.067498853	0.2320316	0.325006086
ZNF263	1215	959	0.073798538	0.2322652	0.325296611

RMDN2	366	301	0.13307197	0.2323208	0.325337863
MAF1	2378	1718	-0.053866547	0.2328179	0.325997449
WDR82	6613	4850	-0.032168023	0.2329798	0.326187492
ARHGEF37	84	76	0.270762222	0.2331151	0.326340135
RNF123	1857	1335	-0.060981942	0.2332005	0.326423026
ZNF512B	2593	2014	0.050593609	0.2334264	0.326702493
SRP72	10333	7887	0.025441504	0.2336423	0.326967905
ATMIN	3497	2703	0.043595848	0.2336704	0.32697059
MSH2	2125	1532	-0.056894412	0.2337918	0.327103733
KDM4D	41	40	0.379528222	0.2340909	0.327485346
RHOJ	517	419	0.111938095	0.2341435	0.32752222
MRPL22	1075	851	0.078046509	0.2343737	0.327807313
FKBPL	218	184	0.170529763	0.2344523	0.327880412
LOC653653	78	71	0.279497032	0.2348875	0.328452267
LOC730101	430	351	0.122286502	0.23504	0.328628597
RPA3	494	401	0.114243327	0.2350653	0.328627073
SUPT7L	1734	1357	0.061468954	0.2355831	0.329313987
ABHD14A	126	80	-0.240199697	0.2357261	0.329476822
ZNF350	416	340	0.12410335	0.2357365	0.329454417
MEF2BNB	308	208	-0.151194691	0.2359922	0.329774873
E2F8	163	140	0.195706994	0.235998	0.329745867
LRRN4CL	110	69	-0.257683125	0.2360402	0.329767886
ESPNL	139	89	-0.22805551	0.2360451	0.329737731
MTRNR2L8	111	98	0.23544611	0.236167	0.329871077
BOLA3	846	596	-0.090193201	0.2363805	0.330132237
CAMK1G	188	160	0.182491375	0.2364008	0.330123556
SPATA18	1304	1027	0.070644444	0.2364191	0.330112059
MAGOH	602	485	0.103373392	0.2364728	0.330149995
TTC32	60	56	0.315616458	0.2364741	0.330114863
C3orf58	842	671	0.087644665	0.2364815	0.330088266
FAM86C1	277	186	-0.159431223	0.2366598	0.330300087
MORC2	2688	2086	0.049358151	0.2366773	0.330287511

FKBP11	1019	722	-0.081931178	0.2371364	0.330891047
MUTYH	219	145	-0.179725838	0.2372086	0.330954697
CHCHD10	284	191	-0.15716616	0.2373342	0.331092861
ZFPM2	2161	1559	-0.055926012	0.2378203	0.331733845
LOC284454	488	337	-0.118980425	0.2378291	0.331709027
C3orf72	85	52	-0.293799086	0.2379195	0.331797875
SEL1L3	1525	1092	-0.066684255	0.2379233	0.331766077
KDM2A	5784	4440	0.033644256	0.2379925	0.33182544
SCYL3	597	481	0.103458094	0.2380376	0.331851221
DNAJC17	327	270	0.138820903	0.2384297	0.332360635
MAP3K14	389	266	-0.133191778	0.239238	0.333449953
DFNA5	2105	1518	-0.056496311	0.2392671	0.333453254
URM1	1766	1381	0.060380108	0.2397672	0.334112812
ZNHIT2	250	167	-0.16692786	0.2397712	0.334081058
PMS2P1	333	226	-0.144047273	0.2398247	0.334118162
CWC27	1781	1280	-0.061391574	0.2398895	0.334171059
JMJD4	94	84	0.252880703	0.2402324	0.334611306
LOC728554	241	202	0.160474278	0.2402533	0.33458427
MREG	241	202	0.160474278	0.2402533	0.33458427
SAMD13	79	48	-0.303666116	0.2403993	0.334731536
GBAS	2880	2089	-0.048104188	0.2404925	0.334823779
COL8A2	146	94	-0.220083575	0.2405675	0.33489086
CETN2	2622	2035	0.049513241	0.2410293	0.335496238
CGREF1	40	39	0.378626256	0.2410726	0.335500235
FOXD2	40	39	0.378626256	0.2410726	0.335500235
KCTD1	578	466	0.104412594	0.241244	0.335682489
KIF18B	767	539	-0.093789173	0.2413328	0.335768569
MBOAT2	2246	1622	-0.054431976	0.241358	0.335766102
SNTB2	8320	6119	-0.028135497	0.2416159	0.336087275
EGFR	8897	6798	0.026943541	0.241715	0.336187701
MED25	1228	875	-0.073803507	0.2417318	0.336173531
HINT2	393	269	-0.131771008	0.2417601	0.336175243

ALX3	53	50	0.331087867	0.2418714	0.336292486
GUSBP11	255	213	0.155508315	0.2421644	0.336662318
RTN4IP1	326	269	0.13788634	0.2424025	0.336955667
BCORL1	354	291	0.132421925	0.2424888	0.337038104
SLC9A3R1	528	427	0.108850272	0.2425845	0.33713349
PROSAPIP1	651	455	-0.101638866	0.2427328	0.337301951
PAOX	278	187	-0.156894481	0.2431453	0.337837461
KLHDC1	230	153	-0.172950077	0.2432038	0.337881023
SLC8A1-AS1	58	34	-0.355366022	0.243338	0.338029747
LMO4	2096	1633	0.055038206	0.2434846	0.3381957
LOC90784	640	514	0.098848586	0.243494	0.338171094
ENC1	4733	3641	0.03673195	0.2436084	0.338292119
TIAL1	4953	3622	-0.036363943	0.2436167	0.338266024
NUDT11	1389	993	-0.069028845	0.2438303	0.338524902
EEF1E1	561	390	-0.109374515	0.2438563	0.338523276
BMP4	404	277	-0.129317185	0.243913	0.338564125
MAP4K2	977	692	-0.082434393	0.2441958	0.33891901
FAM98A	7444	5697	0.029276483	0.2444114	0.339180427
CTXN1	662	531	0.097032776	0.2444165	0.339149652
MRPS14	1285	917	-0.071622589	0.2449988	0.339919824
CAB39L	1192	849	-0.074395645	0.245026	0.339919682
NAIP	619	432	-0.103755965	0.2450686	0.33994097
MAP4K3	4278	3295	0.038494114	0.2452016	0.340087493
NFKB2	1356	1066	0.068002391	0.2453674	0.340279579
LRRK2	235	197	0.160687005	0.2455511	0.340496429
RNF4	3706	2860	0.041304397	0.2455893	0.340511475
FAM151B	104	92	0.23827437	0.2456405	0.340525615
TRAPPC2P1	104	92	0.23827437	0.2456405	0.340525615
C1orf52	1092	863	0.07561174	0.2457826	0.340665765
CCDC59	970	769	0.080150983	0.245853	0.340725427
MFAP2	590	475	0.102364691	0.2459057	0.340760498
TTN-AS1	46	44	0.351021794	0.2460713	0.340952008

TMEM170A	186	158	0.179774069	0.246198	0.34108966
CHPF2	8655	6369	-0.027314816	0.246199	0.341053119
AGAP6	472	326	-0.118762763	0.2465224	0.341463181
CHEK1	1626	1167	-0.063370565	0.246555	0.341470274
ZNF140	772	616	0.089481635	0.2469961	0.342043149
SPA17	225	189	0.163613365	0.247057	0.34208942
BDP1	5960	4369	-0.032857093	0.2475135	0.342683554
BLVRA	1954	1408	-0.057631002	0.2475301	0.342668418
SPRY1	109	96	0.231930308	0.2478172	0.343027758
SRP14	9544	7286	0.025685084	0.2478232	0.34299782
RARA	943	748	0.080932631	0.2478731	0.343028861
SCMH1	1234	972	0.070837956	0.2481065	0.343313742
UBE2L3	7763	5707	-0.028729593	0.2481557	0.343343591
ZSCAN26	864	687	0.084430918	0.2482632	0.343454218
COX7C	5323	3897	-0.034723503	0.2482689	0.34342394
TNFAIP3	265	178	-0.158962987	0.2484368	0.343618056
LRTOMT	244	204	0.156840136	0.2484371	0.343580271
SUMF2	4887	3757	0.035784143	0.2484705	0.343588328
CACNB2	52	49	0.329422258	0.2488841	0.344102921
HSD17B6	52	49	0.329422258	0.2488841	0.344102921
PAK1	3435	2500	-0.043229872	0.2489254	0.344102802
SNTA1	835	589	-0.088356432	0.249005	0.344174605
LRP2BP	135	117	0.208701254	0.2490448	0.344191364
CUL2	4345	3345	0.037802166	0.2501608	0.345695346
CXorf23	942	747	0.080533315	0.2504982	0.34612326
KRT8	920	651	-0.083824186	0.2506152	0.346246475
TRAIP	214	142	-0.176567735	0.2507544	0.346400433
SUPT16H	8562	6542	0.026936056	0.2509131	0.346581133
ALAD	2001	1443	-0.056497736	0.2512127	0.346956548
TRAF2	473	327	-0.117397416	0.251382	0.347151825
GABPB2	305	252	0.139766623	0.2515815	0.347369571
ZNF69	305	252	0.139766623	0.2515815	0.347369571

DDX59	624	501	0.098416706	0.2515845	0.347315994
LOC493754	726	510	-0.094320169	0.2516859	0.347417484
PITHD1	1858	1338	-0.058520254	0.2522809	0.348200137
CCNE1	370	303	0.126944655	0.2524581	0.348406163
NEK11	124	108	0.215843324	0.252651	0.348633778
ZFP62	917	649	-0.083551124	0.2529143	0.348958361
NT5E	73512	54779	-0.009204711	0.2529614	0.348984801
ZNF197	1597	1250	0.061715914	0.2530801	0.349109789
CPPED1	989	783	0.078193918	0.2531249	0.349132978
VEZF1	4201	3235	0.038185058	0.2533418	0.349393425
ZNF287	434	353	0.117125273	0.2536147	0.349731145
HSPB11	1091	776	-0.076370412	0.253743	0.349869328
PAXBP1-AS1	108	95	0.230120238	0.2538624	0.349995098
ZNF653	131	84	-0.225953447	0.2542287	0.350461343
CTNND1	75	68	0.273796283	0.2543235	0.350533836
SNORA40	75	68	0.273796283	0.2543235	0.350533836
COL7A1	7971	5863	-0.02796954	0.2545348	0.350766889
LCMT1	1311	937	-0.069394601	0.2550386	0.351422217
MORC2-AS1	201	133	-0.180617124	0.2551973	0.351602028
MLYCD	429	349	0.11740152	0.255225	0.351601342
BAZ1B	10308	7600	-0.024540987	0.2552947	0.35165837
IMMP2L	139	120	0.203101655	0.2554802	0.351875063
CAMTA2	2938	2134	-0.046122088	0.2555133	0.351881748
ATE1	892	708	0.081857782	0.2555893	0.351947497
SEC16B	456	315	-0.118529864	0.2556057	0.351931067
NUDT6	77	47	-0.297045557	0.2563479	0.352914002
MLH3	1222	872	-0.071692113	0.2564542	0.353021373
MTA1	2805	2036	-0.047111078	0.2565209	0.353074171
RAB27B	1525	1094	-0.064044373	0.2567984	0.353417014
ZNF329	689	551	0.092700468	0.2569424	0.353576128
ATG9A	3237	2355	-0.043778174	0.2570771	0.353722469
ARPC4	4169	3210	0.038024057	0.25718	0.353824976

SARNP	2813	2042	-0.046974555	0.2572128	0.353830907
UBE3D	171	112	-0.195345461	0.257567	0.354279098
KCND2	74	45	-0.302448138	0.2576714	0.35438358
PCDHGA4	364	298	0.126526012	0.257947	0.354723345
NFKBIZ	1085	772	-0.075870158	0.2581372	0.354945816
RBBP8	1290	1014	0.067838718	0.2583533	0.355203656
LSM1	1556	1218	0.061824205	0.2584198	0.355255849
SS18L2	446	308	-0.118961227	0.2591883	0.356273115
PSMC2	6318	4637	-0.031124079	0.2592446	0.356311108
ZNF93	203	171	0.167668729	0.2595203	0.356650699
HBD	68	41	-0.314758705	0.2598218	0.357025675
CBFA2T2	1283	917	-0.0693754	0.2603061	0.357651746
OLFM2	298	202	-0.145804906	0.2605197	0.357905715
TBX19	65	39	-0.321813462	0.2605925	0.357966223
C2orf42	482	390	0.109593109	0.2609061	0.358357458
DIO2	1677	1206	-0.06050065	0.2609897	0.358432767
USP4	3753	2737	-0.040296724	0.2610317	0.358450936
ZNF593	583	407	-0.103314957	0.2612706	0.3587394
GTF2H2	59	35	-0.338207901	0.2613445	0.358801405
TASP1	265	220	0.146663296	0.2615359	0.359024503
SEC22A	964	684	-0.07988469	0.2620729	0.359722099
ZNF91	1016	803	0.075723622	0.2621967	0.359852377
PAPOLG	1029	813	0.075236407	0.2622837	0.359932007
ADCK3	241	201	0.153314487	0.2624721	0.360150896
PMP22	1870	1348	-0.057065642	0.2626063	0.36029537
TCHP	749	597	0.087917345	0.2626871	0.360366512
JMJD8	461	319	-0.116058195	0.2628424	0.36053982
XRRA1	293	242	0.139258515	0.2629048	0.360585718
CTCF	2340	1694	-0.050922523	0.2629507	0.360608971
GAR1	1033	816	0.074952935	0.2631829	0.360887687
GJC1	2949	2281	0.044592756	0.2631979	0.360868564
CCDC69	145	94	-0.210168107	0.2632372	0.360882746

CXorf40A	397	273	-0.125085924	0.2635044	0.361209218
TMEM138	795	561	-0.087801958	0.2636235	0.361332768
ACTR5	207	174	0.16460867	0.2639055	0.361679467
UTP15	1115	879	0.072043492	0.2640902	0.361892796
OPA1	6706	4926	-0.029883724	0.2643245	0.362174012
SMIM12	1750	1260	-0.058779057	0.264457	0.362315702
RSL1D1	7445	5693	0.028069383	0.2644742	0.362299423
WHAMM	744	593	0.087881615	0.2644833	0.362271975
TRIM8	5061	3706	-0.034407461	0.2645919	0.362380968
EIF2AK4	11919	8801	-0.022371705	0.2646105	0.362366524
ECI1	987	701	-0.078483509	0.2647769	0.362554536
ORC5	1065	758	-0.075431545	0.2653041	0.363236523
LSM14A	5220	3824	-0.033815152	0.265449	0.363394961
TATDN3	584	469	0.098771685	0.265613	0.363579495
LOC100132273	87	54	-0.272903862	0.2656605	0.363604611
ZNF331	615	493	0.096153368	0.2663106	0.364454321
CLEC16A	2219	1724	0.051002239	0.2664485	0.364602909
TAF9	3236	2499	0.042291425	0.2665173	0.364656994
ZNF280D	1846	1439	0.055816171	0.2669234	0.365172616
CCDC170	182	154	0.174144032	0.2669606	0.365183356
PDE10A	1170	921	0.069916663	0.2670821	0.365309357
PDE4DIP	5175	3791	-0.033828274	0.2673348	0.365614911
FAM89A	259	215	0.146536694	0.2674291	0.365703737
POM121C	2765	2008	-0.046368079	0.2674695	0.365718822
TUSC1	142	122	0.19614235	0.2675922	0.36584642
SCPEP1	1967	1420	-0.054953896	0.2683681	0.366866948
COX7B	2800	2034	-0.045955016	0.2687075	0.367290653
LOC100289495	43	41	0.346439382	0.2687422	0.36729765
PDE4A	1307	1026	0.065923722	0.2690589	0.36769027
C17orf103	804	639	0.083772562	0.2691787	0.367813543
CD82	2785	2023	-0.046028876	0.2692307	0.367844311
CLHC1	448	363	0.111622948	0.2694465	0.36807857

PSMG4	448	363	0.111622948	0.2694465	0.36807857
JDP2	896	635	-0.081590009	0.2696014	0.368229631
SLC9A3	213	142	-0.169810369	0.2699092	0.368609571
BYSL	1152	822	-0.071778286	0.2699678	0.368649118
LIMD1	2610	1894	-0.047461344	0.2702136	0.368944289
LIN9	384	264	-0.12541625	0.2702323	0.368929376
SIRT1	1452	1137	0.062342933	0.2709094	0.369813346
LYPD6	126	81	-0.222277789	0.2710362	0.369945831
ZNF408	370	254	-0.127544642	0.2712435	0.370188221
AMY2B	49	46	0.324004244	0.2713905	0.370348226
CD58	126	109	0.206056533	0.2717803	0.370839537
MLF1IP	941	668	-0.079194489	0.2719413	0.371018544
PIGH	258	174	-0.153131628	0.2721424	0.371252299
ACTR1B	2200	1592	-0.051511056	0.2722606	0.371372864
KIAA0196	5713	4379	0.031505052	0.2723008	0.371387104
COX6B1	3733	2724	-0.039456674	0.2723143	0.371364789
DTNB	136	88	-0.212879091	0.2726605	0.371775815
HSF4	136	88	-0.212879091	0.2726605	0.371775815
GAB1	899	712	0.078708257	0.272721	0.371797236
SLC25A30	2000	1556	0.052994192	0.2728174	0.37188794
KIF14	1411	1012	-0.064356566	0.2730617	0.372180243
UBN1	3566	2749	0.039752336	0.2731263	0.372227609
ZNF460	136	117	0.19805401	0.2731281	0.372189377
MUC1	146	95	-0.204816819	0.2734928	0.372645503
RARS	9317	6868	-0.024823322	0.2740002	0.373296098
CCNC	2906	2113	-0.044589804	0.2741432	0.373450015
MSRA	853	604	-0.08284506	0.2741762	0.373454187
PVRIG	110	70	-0.236924565	0.2742132	0.373463678
RDH10	2239	1621	-0.050818306	0.2744832	0.373790536
LOC730102	229	191	0.153377172	0.2744941	0.373764543
LOC729852	666	468	-0.093861516	0.2745589	0.373811899
FKBP1B	217	145	-0.166490011	0.2746411	0.37388289

ASH2L	2205	1596	-0.051165872	0.2749129	0.374212076
SARDH	94	59	-0.256793671	0.2749354	0.374201806
ZNF76	765	540	-0.087348209	0.2752766	0.374625238
FAM228B	94	83	0.235602711	0.2753195	0.374642619
MYPOP	392	270	-0.122742115	0.2754019	0.374713805
CCDC41	505	407	0.103897538	0.2757653	0.375167284
MNT	1600	1151	-0.06003194	0.2759212	0.37533844
MKI67IP	1188	934	0.068111751	0.276109	0.375552865
SPAG16	771	613	0.084308346	0.276126	0.375535011
MAP3K2	4716	3622	0.03437496	0.2762471	0.37565871
UBE2Q2P1	276	228	0.139517689	0.2763043	0.375695356
PSMB2	6010	4412	-0.030780068	0.2764912	0.375908477
SLC46A1	163	107	-0.192109036	0.2767468	0.376215018
GTF2E2	2054	1485	-0.052821119	0.2767948	0.376239143
UNC50	1136	894	0.069536033	0.2767993	0.37620423
DDR2	7454	5697	0.027339719	0.2768643	0.376251505
SYT15	828	586	-0.083577971	0.2771495	0.376597995
COL15A1	1071	844	0.071508556	0.2773504	0.376829821
IDH3A	2564	1861	-0.047166075	0.2774489	0.376922581
ORC3	2348	1821	0.048450646	0.2777198	0.377249422
EXT2	8621	6580	0.02538449	0.2777312	0.377223764
EXTL3	5283	3873	-0.032753777	0.2777707	0.377236357
ZNF770	3467	2673	0.039924133	0.2778006	0.377235818
MAMLD1	696	490	-0.091153425	0.2778134	0.377211975
LNP1	125	108	0.204255349	0.2780565	0.377500972
AP1G2	357	245	-0.127990193	0.2781901	0.37764118
PFDN6	1136	811	-0.071036884	0.2782436	0.377672573
TAOK1	15463	11444	-0.019076711	0.2783005	0.377708633
TIGD1	252	209	0.14523134	0.2783988	0.377800896
MAGEH1	1010	797	0.073448468	0.2784166	0.377783904
NLRP1	2124	1650	0.05083439	0.2785335	0.377901356
CHTOP	1650	1188	-0.058779057	0.2785577	0.377893004

LOC619207	135	116	0.19631753	0.2792738	0.378823263
WRNIP1	2056	1598	0.051579275	0.2792998	0.378817281
PRKX	1155	825	-0.070274695	0.2793788	0.378883199
ANAPC15	48	45	0.322042727	0.2794182	0.378874777
KCNE1	48	45	0.322042727	0.2794182	0.378874777
MID2	204	171	0.160579305	0.2796176	0.379083242
FAM171A2	638	448	-0.09490556	0.2796488	0.379084155
ZNF674-AS1	247	205	0.146265	0.2797204	0.379140056
MALT1	2367	1716	-0.048858021	0.2798639	0.379293173
HERC3	2964	2290	0.042954282	0.2800598	0.379517449
PTPN22	54	50	0.304120819	0.2801267	0.379566771
TARBP1	1376	987	-0.064206348	0.2802039	0.379630106
C5	315	215	-0.135863037	0.2802346	0.379630461
TERF1	1954	1520	0.052792988	0.2802491	0.379608788
FIBIN	1066	760	-0.072983983	0.2806944	0.380170635
CSTB	3788	2766	-0.038483043	0.2807208	0.380164992
TBC1D7	508	409	0.102424478	0.281328	0.380945848
METAP1D	211	141	-0.166395705	0.2816431	0.381331057
NELFA	774	547	-0.085640602	0.281813	0.381519652
CAMLG	1949	1516	0.052687795	0.2818337	0.381506283
GNPAT	2724	2107	0.044615743	0.281976	0.381657403
SH3BGR	93	82	0.233545325	0.2822775	0.38202391
ITGAE	769	611	0.083340914	0.2823798	0.382120796
EIF4G2	87498	65979	0.007909003	0.2823862	0.382087971
ENPP2	333	228	-0.131336221	0.282793	0.382596887
PSMD11	5171	3966	0.032393504	0.2828136	0.382583168
PDXDC2P	843	668	0.079467603	0.2834978	0.383467116
NETO2	4607	3538	0.034258582	0.2835106	0.383442753
TMCO6	137	89	-0.20714652	0.283541	0.383442305
ALDH3A2	232	193	0.149628174	0.2835744	0.38344578
PDCD6IP	12883	9524	-0.020676928	0.2836032	0.383443113
CHMP4A	1190	851	-0.068578405	0.283812	0.383683692

ATG10	277	188	-0.144001183	0.2838506	0.38369433
MME	982	775	0.073625418	0.2840072	0.383864321
MSANTD2	841	596	-0.081641338	0.2845186	0.38451382
GAS6-AS1	82	51	-0.269974531	0.2848015	0.384854318
ZNF428	444	359	0.108576299	0.2851484	0.385281269
THNSL2	41	39	0.343002346	0.2852559	0.385384766
TMEM192	1068	841	0.07041819	0.2855089	0.385684698
DHX40	2789	2156	0.043761375	0.2856797	0.385873576
ZNF468	658	525	0.089381971	0.285711	0.385874062
FRAT1	124	80	-0.217116084	0.2857429	0.385875244
THEM6	139	119	0.191028822	0.2858321	0.385953896
HOOK2	563	394	-0.099787161	0.2860116	0.386154457
C6orf132	871	618	-0.079913749	0.286235	0.3864142
NDUFS6	3453	2519	-0.03984708	0.2863566	0.386536385
LZTS1	1427	1025	-0.062209293	0.2864887	0.386672778
WFDC1	1232	882	-0.066999563	0.2865455	0.386707633
BEND6	545	381	-0.1013131	0.2866551	0.386813544
RAC3	498	347	-0.106057948	0.2867253	0.386866338
MEGF6	750	596	0.083573867	0.2870408	0.387250067
NHS	295	201	-0.138367321	0.2874419	0.387749216
ZC3H7A	5043	3868	0.032457701	0.2878924	0.388314841
GID8	2729	2110	0.044022736	0.2880289	0.388456989
RNF8	1166	916	0.067003847	0.2885918	0.389173977
KIRREL	4641	3399	-0.034173204	0.2886555	0.38921773
CCDC106	502	350	-0.10518031	0.2887293	0.38927516
GSDMD	341	234	-0.128111078	0.2887835	0.389306096
PPT2	375	305	0.117070779	0.2888365	0.389335328
NOP16	868	616	-0.07961256	0.2888616	0.389327088
CREBL2	3222	2485	0.040441489	0.2892017	0.38974319
NDUFB9	3543	2586	-0.039097059	0.2892518	0.389768579
TRDMT1	329	269	0.124670721	0.289425	0.389959741
CCDC97	902	713	0.075926775	0.2894583	0.389962526

MTIF2	1976	1429	-0.052424899	0.2897051	0.390252815
NBEAL2	542	379	-0.100942871	0.2897198	0.390230305
ZNF696	366	252	-0.123267783	0.2899041	0.390436431
PEX14	850	603	-0.080152707	0.2906991	0.391464707
N6AMT2	299	204	-0.1364242	0.2908522	0.391628517
PNPLA3	273	225	0.136176182	0.2908847	0.391630015
SPCS1	3617	2641	-0.038557165	0.2909282	0.391646167
STOX2	123	106	0.200558081	0.291046	0.391762476
CYP27A1	73	45	-0.282819331	0.2914907	0.392318628
ZDHHC16	2088	1621	0.049914511	0.2915229	0.392319521
TMEM170B	158	134	0.177460574	0.291602	0.392383579
SFXN2	128	110	0.196511845	0.2916054	0.392345807
ZNF582	97	85	0.224630226	0.2916317	0.392338818
HIRIP3	499	348	-0.104800378	0.2918889	0.392642383
KPNA3	10701	7902	-0.022303738	0.2919683	0.39270682
DLEU1	233	157	-0.154413264	0.2927616	0.393731378
ZNF225	415	336	0.110502028	0.2927955	0.393734457
ISOC1	505	406	0.100348471	0.2927973	0.393694324
HCCS	1580	1138	-0.058271869	0.2931329	0.394103005
TMEM59L	770	611	0.081466066	0.293191	0.394138591
ZNF346	707	499	-0.087518268	0.2933371	0.394292484
CD83	64	58	0.273133127	0.293565	0.394556129
NKRF	783	621	0.080733093	0.2935742	0.394525956
TRIM58	305	250	0.128270984	0.2936616	0.394600819
APP	35498	26846	0.012121534	0.2938939	0.394870432
AK5	7391	5441	-0.026745614	0.2940067	0.394979276
NHLRC1	86	76	0.23681489	0.2941644	0.395148585
ADAM1A	254	172	-0.1472678	0.2942067	0.395162779
PHF2	1905	1481	0.051932775	0.2943591	0.395324792
C17orf75	774	614	0.081057221	0.2944034	0.395341702
OPRL1	89	56	-0.253226377	0.2945353	0.39547619
SKA3	730	580	0.083308568	0.295117	0.396214557

LOC645638	473	381	0.103102946	0.2952959	0.396412014
CASP10	67	41	-0.293385054	0.2953702	0.396468976
GGH	1917	1490	0.051614125	0.2958416	0.397058916
ABI3BP	115	74	-0.220884554	0.2959314	0.397136735
LINC00493	756	600	0.081728398	0.2960467	0.39724858
PRKAB1	1052	751	-0.07109776	0.2963473	0.397609186
C19orf70	1438	1034	-0.060675358	0.2965542	0.397843883
EZR	7086	5414	0.026875554	0.2973555	0.398875897
DACH2	155	102	-0.188546932	0.2975944	0.399153372
RAB23	3490	2548	-0.038709627	0.2976646	0.399204605
TMEM237	1975	1534	0.050597961	0.2983324	0.400057004
WDR52	304	249	0.12722655	0.2984908	0.400226329
ZSCAN23	248	205	0.140435921	0.2988431	0.400655568
PARVB	1176	842	-0.06684379	0.2988819	0.400664466
PHF10	573	402	-0.096187506	0.299193	0.401038396
SEC14L1P1	112	72	-0.222277789	0.2993956	0.401266754
URB2	829	656	0.077475845	0.2999515	0.401968606
RPAIN	1083	774	-0.06947564	0.3003467	0.402454933
ATRIP	497	347	-0.103158057	0.3003498	0.402415748
OSTF1	2281	1655	-0.047683098	0.3004297	0.402479519
LOC100128252	526	368	-0.100204901	0.3004357	0.402444193
CLCC1	3634	2655	-0.037694427	0.3006523	0.402691151
ZNF546	311	213	-0.130909018	0.3008182	0.40286993
SOGA1	3819	2792	-0.036743847	0.3009171	0.402959026
FTSJD2	2537	1844	-0.045132732	0.3010777	0.40313084
LOC284023	122	79	-0.211804458	0.3011783	0.403222104
ADCK5	304	208	-0.132335664	0.3012005	0.403208479
VMA21	3394	2614	0.038424708	0.3015152	0.403586409
UBLCP1	2851	2076	-0.042509464	0.3015996	0.403656046
HYLS1	290	198	-0.135400338	0.3017855	0.403861501
AGGF1	2579	1994	0.044005768	0.3018655	0.403925077
NDUFC1	1439	1124	0.058727575	0.3021547	0.404268624

COG6	3260	2512	0.039116632	0.3025669	0.404776606
DTWD1	1208	947	0.063968008	0.3026921	0.404878986
TRNT1	1208	947	0.063968008	0.3026921	0.404878986
TBC1D25	746	528	-0.083485569	0.3028198	0.404984452
NUSAP1	2335	1808	0.04612426	0.3029832	0.405159559
MTFR1	1610	1161	-0.056540584	0.3030159	0.405159693
MID1IP1	1152	904	0.065406093	0.3032976	0.405492928
ZNF239	303	248	0.126174459	0.303389	0.40557158
SMAD4	4054	3115	0.035038206	0.3034464	0.405604758
TAPT1-AS1	57	52	0.282701836	0.3041105	0.406448733
HSD17B14	119	77	-0.212879091	0.3048404	0.407380626
PLEKHO2	2493	1812	-0.045147796	0.3051475	0.407747236
NSUN5P1	200	134	-0.162614868	0.3053395	0.407959999
SP4	321	262	0.122145646	0.3054662	0.408085477
RBM5	4844	3714	0.031927082	0.3056213	0.408249006
NUDT7	139	91	-0.195994301	0.3058258	0.408478339
MOCOS	259	176	-0.142224537	0.3059142	0.408552567
SUGT1P1	93	59	-0.24136363	0.3061066	0.408765687
POLD4	1846	1335	-0.052410679	0.3062738	0.408945168
FAM65B	207	139	-0.159393753	0.3064268	0.409105565
MANBA	2224	1723	0.046918045	0.3064422	0.409082295
FDXACB1	166	110	-0.178527586	0.3065796	0.409221884
TRIAP1	1369	1070	0.059640482	0.3066768	0.409307765
FOXRED1	644	454	-0.089216259	0.3067457	0.409355784
C11orf49	918	724	0.072647675	0.3068137	0.409402661
SPESP1	68	61	0.258426628	0.3068959	0.409468458
ZNF639	1517	1093	-0.057775553	0.3070578	0.409640605
DEPDC5	528	423	0.095271866	0.3075493	0.410252444
SMAD5	7595	5797	0.025408653	0.3078428	0.410599901
NR1H3	190	127	-0.16601879	0.3080096	0.410778454
GALNT16	170	143	0.165632532	0.3082376	0.411038522
YTHDC2	3044	2347	0.040001615	0.3082516	0.411013102

EVI2B	74	46	-0.270739278	0.3082863	0.411015437
MCAT	394	273	-0.114142547	0.3084925	0.411246236
TSC22D1	6776	4987	-0.02710954	0.3085035	0.411216903
EPC2	1564	1219	0.055609745	0.3086552	0.411375082
ZFP82	173	115	-0.173986045	0.3086738	0.411355901
RILPL2	412	286	-0.111477059	0.3088945	0.411605933
LOC729603	235	159	-0.14848186	0.3091311	0.411877167
ZCWPW2	126	82	-0.204575787	0.3095653	0.41241157
LOC90834	110	95	0.203648027	0.3103909	0.413467193
SLC43A3	528	370	-0.097860527	0.3107709	0.413929201
VDAC1	15934	11805	-0.017558307	0.3109611	0.414138168
NHEJ1	590	415	-0.092451486	0.3109684	0.414103701
KCNIP4	73	65	0.247695386	0.3111512	0.414280657
SOCS2-AS1	73	65	0.247695386	0.3111512	0.414280657
DMXL1	3767	2755	-0.036211583	0.3112455	0.414339729
OSBPL10	550	386	-0.095678639	0.311622	0.414796623
ZNF277	1090	780	-0.067629974	0.3116562	0.414797848
XDH	120	103	0.194762063	0.3116724	0.414775044
ZNF252P	1550	1208	0.055504371	0.311757	0.41484332
TIMM8B	1367	1068	0.059050536	0.3118894	0.414975187
CCDC24	89	78	0.22482092	0.312008	0.415088665
C16orf70	715	506	-0.083653725	0.312126	0.415201277
FAM220A	967	690	-0.071767396	0.31224	0.415308518
ZNF737	433	349	0.104012143	0.3123356	0.415391335
KLHL25	295	202	-0.13120753	0.312515	0.41558551
SYTL4	800	568	-0.078956939	0.3125151	0.415541297
ADAT3	87	55	-0.246431651	0.3126108	0.415624178
PPIH	532	373	-0.097098483	0.3126638	0.415650252
DAK	671	474	-0.086273576	0.3127907	0.415774683
PTPRQ	56	51	0.280222552	0.3128608	0.415823459
IRF7	143	94	-0.190130353	0.3131671	0.416186149
FAM120AOS	998	785	0.06880497	0.3132159	0.416206559

DDTL	68	42	-0.279993287	0.3132569	0.416216629
LOC401052	123	80	-0.205434279	0.313441	0.416416864
ZDHHC6	1489	1073	-0.057541546	0.3135088	0.41646246
HIVEP1	1997	1447	-0.049617279	0.3135181	0.41643046
ROM1	100	64	-0.228704058	0.3135557	0.416435954
ZNF669	333	271	0.117922806	0.3139642	0.416933996
DFNB31	352	243	-0.119466983	0.3141493	0.417135291
ABCA3	44	41	0.313272518	0.314489	0.41749744
PRKCH	44	41	0.313272518	0.314489	0.41749744
TNNT1	44	41	0.313272518	0.314489	0.41749744
ZNF222	212	176	0.146663296	0.3152658	0.418439457
LHFP	2509	1825	-0.044063875	0.3152663	0.418395467
ETV4	760	539	-0.080562014	0.3153221	0.418424915
ESPL1	797	566	-0.078625539	0.3154317	0.418525855
BAIAP2-AS1	1472	1148	0.056497102	0.3155884	0.41868917
SYN1	99	86	0.212060266	0.3155905	0.418647297
LRRCC1	360	292	0.113123594	0.3156095	0.418627893
PSMD6	4473	3431	0.032538499	0.3156701	0.418663696
CALM2	36967	27505	-0.011391934	0.3160718	0.419151862
SETD9	188	157	0.155184029	0.3162935	0.419401242
CTPS2	1233	965	0.06158018	0.3163418	0.419420595
ASNSD1	2471	1910	0.043629759	0.3163963	0.419448143
C6orf106	5588	4276	0.029081964	0.3164505	0.419475429
MATN2	716	507	-0.082821709	0.3165552	0.419569566
EZH2	998	713	-0.069985607	0.3166215	0.419612774
CLCF1	570	455	0.090056758	0.3167079	0.419682613
MRPL40	1220	955	0.061843622	0.3168186	0.419784638
PCDHGA6	511	358	-0.098211572	0.3169861	0.419961858
MCTP2	1658	1290	0.053079191	0.3172821	0.420309391
GPANK1	584	411	-0.091677843	0.3174432	0.420478107
ZDHHC21	2395	1852	0.044210574	0.3175071	0.420517965
CDH11	9231	7032	0.022600258	0.3175496	0.420529529

AIFM1	1789	1390	0.051083627	0.3175933	0.420542725
ZNF799	109	94	0.201556659	0.3177711	0.420733419
KIAA0391	1915	1387	-0.050224473	0.3178285	0.420764677
INTS7	1178	845	-0.064164161	0.3180367	0.420995489
NEK2	883	629	-0.074201289	0.3182929	0.421289942
KIF9	139	118	0.178854108	0.3186765	0.421752792
LOC100130744	174	116	-0.169810369	0.3186843	0.42171836
LSM6	436	351	0.102295027	0.3188647	0.421912212
TFPI	10948	8329	0.02069999	0.3189268	0.421949536
ZNF137P	124	106	0.188876276	0.319082	0.422110019
NPIPL3	457	319	-0.10348561	0.3191391	0.422140801
STT3A	14153	10480	-0.018317044	0.3192402	0.422229598
IL6ST	61880	46126	-0.008740876	0.3192683	0.422222012
SLC7A7	107	69	-0.217790398	0.3199316	0.423054266
IER2	1412	1017	-0.058268278	0.3203443	0.42355501
ARHGAP32	1029	736	-0.068313179	0.3207843	0.42409181
ZNF345	94	60	-0.232546124	0.3207875	0.424050972
HMGN4	2749	2003	-0.041592354	0.3208881	0.4241389
TFAP2A	603	425	-0.089543029	0.3209194	0.424135357
DHFRL1	414	288	-0.108409824	0.3209666	0.424152624
TSSK3	117	76	-0.207285074	0.3213568	0.424623213
CRBN	1167	914	0.062613641	0.3215128	0.424784334
MON1A	541	380	-0.094477044	0.3216293	0.424893201
MLH1	2229	1619	-0.046141499	0.321778	0.425044548
TUSC2	977	698	-0.069979394	0.3218098	0.425041493
ZBTB4	8969	6833	0.022724118	0.3218625	0.42506594
NLRC3	55	50	0.277648608	0.3219021	0.42505068
RYR3	55	50	0.277648608	0.3219021	0.42505068
ZDHHC1	314	216	-0.124581115	0.3219351	0.425026617
OSGEP	607	428	-0.088933588	0.3225734	0.42582422
ZNF101	195	131	-0.15875518	0.3227642	0.426030975
KIAA1586	1128	884	0.063503339	0.3228348	0.42607898

C19orf44	158	133	0.166653819	0.322889	0.426105378
PPIL1	1643	1278	0.052707488	0.3229131	0.426091991
CD36	77	68	0.235828432	0.3229968	0.426134735
SLC7A5P2	77	68	0.235828432	0.3229968	0.426134735
FAM149B1	1222	956	0.06099037	0.3232169	0.426357387
FAM160A2	943	742	0.069313548	0.3233258	0.426455838
ZNF132	426	343	0.102507278	0.323458	0.426585003
ATL1	904	712	0.0707066	0.3237994	0.426990138
RPL23AP64	104	67	-0.219198396	0.3238119	0.426961388
KCTD5	1706	1326	0.051615261	0.3240332	0.427207965
DGKG	91	58	-0.234661513	0.3244305	0.427686486
PRPSAP1	1304	938	-0.06013191	0.3245185	0.427757252
ITFG2	778	615	0.075968387	0.3249425	0.428270777
LSM3	1971	1429	-0.048769728	0.3249555	0.428242595
PARG	1200	939	0.061314789	0.325018	0.428279704
FLJ31306	1561	1127	-0.05483089	0.3252059	0.428481931
SLC43A1	528	422	0.091857201	0.3254971	0.428820265
MBIP	818	582	-0.075929558	0.3257966	0.429169335
POLE4	707	501	-0.08174748	0.3258431	0.429185215
TMEM234	480	336	-0.099421041	0.3263067	0.429750404
VKORC1L1	1612	1254	0.052837736	0.3263162	0.429717522
MAP1LC3A	967	691	-0.069678047	0.3263616	0.429731823
MAPKAPK5-AS	379	263	-0.111982917	0.3269453	0.430454965
SF3B14	3724	2861	0.034818557	0.3270493	0.430546325
SNHG4	210	174	0.14385011	0.3271668	0.430655456
THYN1	863	615	-0.073622017	0.3275794	0.431153095
MXRA7	11919	9060	0.019471888	0.32806	0.431739983
C8orf44	87	76	0.220136149	0.3280893	0.431732971
IRF1	1275	917	-0.060351476	0.3282139	0.431851297
LETMD1	1136	815	-0.063938739	0.3285038	0.432187114
SPG11	3626	2653	-0.035602118	0.3286379	0.432317869
NAV3	7052	5196	-0.025478912	0.3287229	0.432383978

TFAP4	141	93	-0.18524041	0.3287722	0.432403184
KLHDC4	645	456	-0.085113204	0.3288068	0.432403014
OPN3	298	243	0.120796115	0.3289414	0.432534306
MARCH5	3324	2557	0.03668391	0.3291329	0.432740485
AXIN1	923	659	-0.070900051	0.3293175	0.432937497
TXN2	2822	2058	-0.040322874	0.329438	0.433050217
ATP6V1G2	111	72	-0.209338733	0.3294938	0.433077786
MBD4	1449	1045	-0.056402521	0.3296755	0.433270865
CCNL2	6208	4743	0.026823443	0.3302971	0.43404208
ARSJ	10859	8029	-0.020446902	0.3303039	0.434005212
SLC35B2	3932	2880	-0.034042378	0.3305805	0.434322828
INTS12	1028	736	-0.066910461	0.330939	0.434747936
HADH	1103	791	-0.064531059	0.3312225	0.435074516
PPFIA4	54	49	0.274974474	0.3312462	0.435059795
LOC100506190	326	225	-0.119794831	0.331414	0.435234198
ARHGAP31	2430	1877	0.042624468	0.3318062	0.435703374
C17orf97	195	162	0.147671821	0.3320859	0.436024681
GPX7	482	386	0.094719833	0.3324581	0.436467287
SLC25A17	1397	1007	-0.057116206	0.3324914	0.43646508
SART3	2534	1956	0.041641978	0.3330319	0.437128443
C1GALT1C1	1291	1008	0.05815877	0.3331639	0.437255734
MAX	1163	910	0.061239485	0.3331925	0.437247132
SNORA63	48	44	0.28962125	0.3334015	0.4374753
GPR107	8386	6189	-0.023124376	0.3335181	0.437582167
ZNF467	122	104	0.184854512	0.3337635	0.437858045
BUD31	1337	963	-0.058239631	0.3337805	0.437834231
PMPCB	2901	2235	0.038876668	0.3337869	0.437796485
BLOC1S4	635	449	-0.084889015	0.3338411	0.43782156
DDX49	1465	1141	0.054550259	0.3338691	0.437812153
ADD1	20080	14904	-0.014907558	0.3339532	0.437876291
CORO7	42	39	0.308236928	0.3340034	0.437873018
SPDYE3	42	39	0.308236928	0.3340034	0.437873018

PCCB	1864	1351	-0.049222054	0.3340262	0.437833762
MAGEF1	1346	1050	0.05686305	0.3340799	0.437858101
PDF	66	41	-0.271689983	0.3343457	0.438160292
CRELD1	823	649	0.072478179	0.3343833	0.438163515
FAM108B1	961	755	0.067092345	0.3346919	0.438521713
PRPF4	2135	1551	-0.045885252	0.3348702	0.438709225
SBNO2	2721	2098	0.040029853	0.335043	0.438889374
HTATIP2	865	617	-0.072277512	0.3358188	0.439859381
IFT122	1145	896	0.061375171	0.3358811	0.439894781
ZNF141	145	96	-0.179794458	0.3359806	0.439978787
CCDC109B	489	343	-0.096473757	0.3361194	0.440114263
MTM1	939	738	0.06764779	0.3362571	0.440248271
RNF216P1	1097	787	-0.063975853	0.3366712	0.440744125
TMEM101	802	571	-0.074959359	0.3367092	0.440747591
GTF3C6	3777	2766	-0.034287496	0.3368542	0.440891054
LINC00115	70	62	0.240065425	0.3370757	0.441134642
C20orf96	413	288	-0.104920838	0.3370794	0.441093079
DSCC1	377	262	-0.10984558	0.3373623	0.441416889
GTF2H2B	1011	724	-0.066569263	0.3374117	0.441435139
LOC100506100	105	68	-0.211630545	0.3378534	0.441966606
KCTD18	1093	856	0.062541432	0.3379171	0.442003479
ZEB1-AS1	227	187	0.135498104	0.3379671	0.442022495
CBX4	1387	1081	0.055550867	0.3382215	0.442308731
MSRB2	888	634	-0.070924704	0.3384947	0.442619541
SLC7A6OS	221	150	-0.143931737	0.3386598	0.442788943
SCAND2P	445	357	0.09727087	0.3387004	0.442795575
ADC	228	155	-0.141613477	0.3387779	0.442850396
MRPS31P5	287	234	0.120609925	0.3387928	0.442823383
WDYHV1	526	370	-0.092385397	0.3390779	0.443149582
FANCC	582	463	0.085145172	0.3392045	0.443268458
RPIA	427	343	0.099124638	0.3393986	0.443475637
TRIM21	507	405	0.091088293	0.3394358	0.443477732

CD3EAP	170	142	0.155508315	0.3395936	0.443637278
SLC36A1	1611	1252	0.0514302	0.3396364	0.443646696
LOC441454	92	59	-0.225766775	0.3397252	0.44371612
ZSCAN32	799	569	-0.074614719	0.3398116	0.443782517
SNAP29	2800	2043	-0.039585491	0.3401876	0.444226998
PDE8B	101	87	0.199884145	0.3403716	0.444420652
APOBEC3G	142	94	-0.180006136	0.3404795	0.444514924
CNOT1	14789	10960	-0.017124574	0.3410599	0.445225967
CDKL3	126	107	0.179339195	0.341066	0.445187341
CFB	111	95	0.190591874	0.3413416	0.44550028
ZNF789	306	211	-0.121136522	0.3414407	0.445583015
LOC646471	267	183	-0.129843962	0.3415673	0.445701531
FAM175B	1688	1222	-0.050918487	0.3417562	0.445901323
LOC100272217	57	35	-0.288454865	0.3422338	0.446454293
MARVELD2	57	35	-0.288454865	0.3422338	0.446454293
CASKIN2	848	605	-0.071976991	0.3425993	0.446860792
MANBAL	2094	1620	0.044884503	0.3426066	0.446823569
SRSF7	4043	2964	-0.032728624	0.3427601	0.446976953
ALDH4A1	822	586	-0.073085598	0.3428298	0.44702105
ZBTB42	149	99	-0.174659769	0.3429729	0.447160784
LY6G5B	232	158	-0.139048115	0.3431913	0.447398687
C8orf59	733	521	-0.077377694	0.3433002	0.447493907
RABEP1	5051	3712	-0.0292202	0.3434707	0.447669232
ZNF618	3424	2631	0.035080679	0.3436978	0.447918419
TOM1L1	240	197	0.130313356	0.3437946	0.447997623
PKD1	7508	5538	-0.023911465	0.3441302	0.448388069
PNPO	1963	1425	-0.046946122	0.344203	0.448436011
EXOC6	1061	831	0.062647858	0.3442605	0.448464083
ATXN2L	6288	4631	-0.026125329	0.3443769	0.448568765
PEBP1	10159	7727	0.020374037	0.344709	0.448954395
ABT1	833	656	0.070531451	0.3447785	0.448997994
SLC35E1	5211	3831	-0.028687097	0.344786	0.448960783

ZNF304	726	516	-0.077446351	0.345193	0.449443749
CHMP6	830	592	-0.072362029	0.3452335	0.449449431
TOE1	271	186	-0.127838098	0.3453247	0.44949778
TRIML2	271	186	-0.127838098	0.3453247	0.44949778
BFAR	3448	2649	0.034840201	0.3453515	0.449462174
ARID3B	207	171	0.139517689	0.3457803	0.449973176
TRIM65	1047	751	-0.064224498	0.3460802	0.450316437
HSBP1L1	611	485	0.081964499	0.3461108	0.450309178
POMP	8014	6106	0.022857212	0.3462384	0.450428179
AGAP8	73	46	-0.251110471	0.3463206	0.450488028
RSRC1	772	609	0.072993512	0.3463639	0.450497299
CROCC	586	414	-0.086117765	0.3463934	0.450488706
TTC12	296	204	-0.121875892	0.3465824	0.450687408
PTPN3	140	118	0.168512164	0.3465992	0.450662141
ZNF324B	188	156	0.145965499	0.3466688	0.450705655
CTSZ	2657	1938	-0.040077525	0.3469167	0.450980858
LOC100134229	58	52	0.257610855	0.3469457	0.450971541
SHQ1	1043	817	0.062820957	0.3470419	0.451049405
DHX57	2201	1701	0.043376128	0.3472021	0.451210524
SCNN1D	208	141	-0.145736234	0.3474268	0.45145545
GINS1	650	515	0.079284846	0.3474443	0.451431164
SLC25A16	1320	1029	0.055857184	0.3474899	0.451443313
PRUNE2	340	275	0.109049004	0.3474966	0.451404882
LOC401321	236	161	-0.136574039	0.3475216	0.451390291
TFEB	222	151	-0.140858995	0.3477277	0.451610837
PRKRA	1930	1401	-0.04699176	0.3478268	0.451692465
CKAP4	45099	33596	-0.009653839	0.3478523	0.451678466
PRKACB	6345	4674	-0.025810298	0.3480252	0.451855864
TOR1AIP1	3745	2874	0.033246475	0.3481401	0.451957958
UBALD1	535	377	-0.089822236	0.3483472	0.452179661
CLN6	954	683	-0.066951556	0.3486834	0.452568933
MAN1B1	5711	4203	-0.027171796	0.348735	0.452588785

DECR1	4175	3200	0.031447839	0.3489014	0.452757578
TSPYL5	1098	859	0.061004114	0.3489543	0.452779043
C2orf88	120	102	0.180686878	0.34911	0.452933839
RBBP6	5293	4047	0.027919327	0.3493718	0.453226353
IL17RC	653	463	-0.080918667	0.349884	0.453843459
HOXC8	70	44	-0.254699267	0.3498943	0.453809617
DDX60	784	618	0.071905316	0.3499776	0.453870397
RBM6	3466	2662	0.034391049	0.3503892	0.454356876
FAM111A	2413	1758	-0.041740713	0.3506726	0.454677028
ZNF26	477	335	-0.094676039	0.35104	0.455106008
RP9	437	306	-0.098949495	0.351225	0.455298382
ZNF544	1165	838	-0.060155674	0.351549	0.455670962
ARHGAP27	63	56	0.24522713	0.3517963	0.455944057
NUP54	1608	1164	-0.051024217	0.3524705	0.456770415
NMI	504	402	0.088923899	0.3526457	0.45694985
DENND5B	1650	1195	-0.050303275	0.3530544	0.457431922
RBM3	18644	14126	0.014793715	0.3530758	0.45741201
ICA1L	383	267	-0.105352518	0.3533228	0.457684454
MOV10	3326	2434	-0.035306869	0.3534781	0.457837946
RRAGC	2841	2187	0.037706521	0.3536723	0.458041804
DARS2	1483	1153	0.052026047	0.3539924	0.458408752
LUZP1	6891	5081	-0.024448762	0.354132	0.458541873
KLHL2	1162	836	-0.05988309	0.3543334	0.458754963
CACNA1H	133	88	-0.180698685	0.3544698	0.458883804
CACYBP	3758	2883	0.032757902	0.3544769	0.458845349
GPR162	325	263	0.109775213	0.3551053	0.459610983
E2F2	93	60	-0.217116084	0.3552322	0.459727521
ARMCX4	1590	1151	-0.0509868	0.3555295	0.460064462
CCDC174	989	775	0.063377921	0.355547	0.460039222
ROBO2	967	693	-0.065508406	0.3557971	0.460315036
MCM10	674	533	0.076539073	0.3559168	0.460422058
RING1	1788	1297	-0.048016125	0.3559295	0.46039063

F10	419	336	0.096663121	0.3562844	0.460801917
B3GNTL1	237	162	-0.133741114	0.3563611	0.46085324
BCL2L11	94	81	0.200413283	0.3563629	0.460807675
KRI1	971	696	-0.065231858	0.356816	0.461345756
LINC00526	64	40	-0.262919773	0.3568575	0.461351448
MTAP	2727	2100	0.038226759	0.3568886	0.461343782
SPDYE5	177	147	0.147218927	0.3569642	0.461393653
PPP1R13B	216	147	-0.140063026	0.3569863	0.461374308
RNF181	2090	1520	-0.044279487	0.3570509	0.461409876
CAMKK2	2673	1951	-0.039093928	0.3573544	0.461754289
SEMA3A	16588	12306	-0.015625923	0.3573996	0.461764668
PSG9	109	93	0.186126618	0.3575186	0.46187057
KPNA6	11445	8695	0.018692602	0.3576249	0.461960018
LARGE	1129	812	-0.060341722	0.3576309	0.461919804
C7orf10	374	301	0.101877349	0.3579033	0.462223728
SUSD1	494	394	0.08883672	0.3579457	0.462230594
KIAA1614	269	185	-0.12492877	0.3579523	0.462191127
KCMF1	4405	3373	0.030042431	0.3581182	0.462357458
BAHD1	1587	1232	0.04985226	0.3584015	0.462675289
RBBP9	1629	1180	-0.050047613	0.358565	0.462838297
PTGR1	3398	2488	-0.034547235	0.3587253	0.462997294
TAP1	1831	1418	0.046377873	0.359042	0.463357959
RBM45	559	395	-0.085843498	0.359261	0.463592604
TNKS2	7471	5693	0.02303987	0.3592829	0.46357283
ARHGAP23	9784	7235	-0.020279255	0.3594045	0.463681701
SFXN4	445	356	0.093224037	0.359515	0.463776212
P2RX6	90	58	-0.218719969	0.3597096	0.463979255
RALGAPA2	520	414	0.086271276	0.3599165	0.464197983
BSN	61	38	-0.267657692	0.3602053	0.464522371
KAT8	467	373	0.090905212	0.3602175	0.464490043
FRS3	120	79	-0.187957716	0.360864	0.465275471
MYEOV2	2429	1873	0.040140541	0.3610604	0.465480568

TRIM17	78	68	0.217212754	0.3611141	0.46550153
RHD	51	46	0.266288746	0.3612263	0.46559802
DKFZP434I0714	192	130	-0.147442556	0.3612266	0.465550238
MARC2	154	103	-0.165133882	0.361236	0.465514148
SNN	983	770	0.062819161	0.3613121	0.465564026
FAM109B	541	382	-0.086903824	0.3613134	0.46551757
SETD4	616	488	0.079102929	0.3614913	0.465698571
KLRAP1	213	145	-0.139648398	0.3617363	0.465966079
HSD17B10	1480	1150	0.051188817	0.3623468	0.466704212
TAF2	3481	2550	-0.033852435	0.3626295	0.467019977
ZNF599	161	108	-0.160877244	0.3627488	0.467125359
TXNDC11	1800	1394	0.046385786	0.3630595	0.467477049
AKAP7	209	172	0.134057754	0.3631313	0.467521189
SPATA2L	269	219	0.118476829	0.363162	0.467512468
PCBD1	689	490	-0.076570102	0.3631817	0.467489468
KIAA1009	328	265	0.107448677	0.3633844	0.467702047
GTPBP5	1029	739	-0.062444581	0.3636257	0.467964298
ING4	678	482	-0.077099995	0.3637778	0.468111631
SHF	127	84	-0.181215132	0.3642314	0.468646868
C8orf31	45	41	0.28085104	0.3644968	0.468915793
CBWD6	45	41	0.28085104	0.3644968	0.468915793
NT5C2	5682	4339	0.026115886	0.3645779	0.468947364
PRKAR1B	693	493	-0.076115574	0.3646116	0.468942302
KCNK6	255	208	0.121238413	0.364699	0.469006344
SHC4	560	396	-0.084774265	0.3647599	0.46903616
TRMU	786	561	-0.07137641	0.3648513	0.469105344
GARNL3	175	118	-0.153415931	0.36491	0.469132366
NEMF	5044	3856	0.027688908	0.3649604	0.469148648
FBRS	2784	2034	-0.0376874	0.3650626	0.469231706
MIRLET7DHG	93	80	0.197921416	0.3651471	0.46929186
GRIA1	67	59	0.231705991	0.3653869	0.46955156
TOPBP1	3068	2358	0.035417367	0.3654862	0.469630714

EPB41L2	16297	12353	0.01540717	0.3658355	0.470031081
UBQLN2	3854	2827	-0.031932557	0.3660602	0.470271203
STK36	1466	1139	0.051034775	0.3660848	0.470254384
HIGD2A	1377	995	-0.053607997	0.3662089	0.470365219
ZFP90	2130	1645	0.042386285	0.3662681	0.470392752
PPFIA3	107	70	-0.197031838	0.3665039	0.470647065
TMEM52B	210	143	-0.139222049	0.3665696	0.470682966
C2orf43	1203	938	0.056175317	0.3670439	0.47124335
SRPX	4894	3742	0.027947589	0.3677174	0.472059354
L3MBTL3	1632	1183	-0.049038852	0.36781	0.472129657
C12orf65	805	633	0.068368848	0.3681538	0.472522216
XYLT2	3538	2593	-0.03315971	0.3685904	0.473033846
SF3A3	4959	3791	0.027681363	0.3691636	0.473720674
PAR5	955	748	0.062689669	0.3692402	0.47377009
SLC15A4	1147	895	0.057246328	0.3693252	0.473830384
MYH3	52	32	-0.285287586	0.3693559	0.473820944
LOC100505715	137	115	0.1626101	0.3694316	0.473869236
SESTD1	1325	957	-0.054249398	0.3697846	0.474273226
GPR89A	299	207	-0.115362585	0.3700115	0.47449088
TAF1A	299	207	-0.115362585	0.3700115	0.47449088
PHIP	7524	5731	0.022439179	0.3703062	0.474795463
GRK4	114	75	-0.188919192	0.3707921	0.475369616
FBXL3	3431	2514	-0.033492344	0.3711904	0.475831202
SCAMP5	104	68	-0.197824745	0.3714546	0.476120961
WARS2	748	589	0.070381496	0.3716755	0.476355109
ADAP2	407	326	0.094995302	0.3717406	0.476389447
FOXL2	61	54	0.239302296	0.3717724	0.476381183
USP7	7617	5801	0.022230855	0.3718466	0.47642725
ZNF664	5747	4387	0.025577789	0.3718694	0.476407434
BLOC1S1	50	45	0.263149038	0.371916	0.476369135
C12orf60	50	45	0.263149038	0.371916	0.476369135
IFLTD1	50	45	0.263149038	0.371916	0.476369135

SRGAP2	1866	1356	-0.045439676	0.3719289	0.476287743
UBR4	26826	20284	0.01186236	0.3719535	0.476270321
C1RL-AS1	398	319	0.095940125	0.3722762	0.47663447
MUT	2303	1776	0.040269302	0.3722786	0.47658854
OR7E12P	87	75	0.201027326	0.3733987	0.477973407
CSTF2T	1421	1028	-0.051914158	0.3739071	0.47857499
ZNF433	299	242	0.110013695	0.3740379	0.478693164
DHODH	212	174	0.130175173	0.3741142	0.478716985
ZNF702P	212	174	0.130175173	0.3741142	0.478716985
DVL2	1558	1129	-0.049497616	0.3744928	0.47912765
LEPRE1	8298	6315	0.021171209	0.3748097	0.479483809
CDCA7L	777	555	-0.070274695	0.3749607	0.479627753
MANEAL	66	58	0.228739008	0.3753974	0.480137084
PTGES3	14797	10975	-0.015931637	0.3755247	0.480250497
GNAL	44	40	0.277648608	0.3756279	0.480333221
PDZK1	91	59	-0.209999459	0.375828	0.480539702
C1orf170	111	73	-0.189439176	0.3758941	0.480574961
ZNF701	643	508	0.075161891	0.3760178	0.480683726
UQCR10	2404	1754	-0.039636017	0.3762135	0.480884531
STX8	1100	792	-0.058779057	0.3763063	0.48095382
TMEM256	101	66	-0.198665232	0.376491	0.481140528
CLUAP1	1183	922	0.055540714	0.3765383	0.481151649
ERMP1	1534	1190	0.048815222	0.3766927	0.481299561
RAB11FIP5	5579	4259	0.02566031	0.3774102	0.482166816
C12orf52	673	531	0.073257488	0.3775416	0.482285145
TICAM1	744	531	-0.071438629	0.3775737	0.482276783
PPCDC	250	172	-0.124367398	0.3776049	0.48226715
SRSF1	16946	12839	0.014740196	0.3777137	0.482356652
PUM2	6498	4954	0.023750257	0.378093	0.482791551
DHRS11	318	221	-0.109828264	0.3783236	0.483036384
ATPAF2	379	304	0.097025607	0.3784927	0.483202777
SELM	4496	3439	0.028499212	0.3786018	0.483292522

SLC35E2	1792	1302	-0.045689057	0.3789968	0.48374724
H2AFJ	1689	1226	-0.047058217	0.3791443	0.483885907
COX10-AS1	293	203	-0.114268805	0.379312	0.484050323
SAMD9L	2717	1986	-0.036996811	0.3796707	0.48445838
ZCCHC11	2579	1985	0.037479366	0.3797657	0.48453
ZNF616	616	487	0.076143553	0.3799776	0.484750649
HEPH	685	488	-0.074070709	0.3802368	0.48503169
ZNF219	358	250	-0.102879361	0.3806662	0.485529671
SLC15A3	88	57	-0.211389473	0.3807895	0.485637172
PRELP	62	39	-0.25364196	0.3808856	0.485710053
ZNF503-AS2	108	71	-0.189988251	0.3810914	0.485922793
MDM4	1873	1362	-0.044472064	0.3812272	0.486046137
MAPKAPK3	1602	1162	-0.048111947	0.3812788	0.486062174
RNASEL	398	279	-0.097351177	0.3812921	0.486029416
PLAUR	4317	3303	0.028900009	0.3815941	0.486364529
TRPC3	98	64	-0.199557712	0.3816147	0.48634107
GDPD1	81	70	0.204585146	0.3817793	0.486501101
ULK1	2474	1905	0.038097629	0.3818209	0.486504277
PMS2	745	532	-0.070662048	0.3823974	0.487188997
MORC4	3715	2726	-0.031424517	0.3824531	0.487210124
FBXO27	75	48	-0.228704058	0.382788	0.487561988
ZCWPW1	75	48	-0.228704058	0.382788	0.487561988
PTPRA	4709	3464	-0.027829661	0.3835497	0.488457167
ZNF764	378	303	0.096083691	0.3838448	0.488783067
PAK1IP1	1100	858	0.056698161	0.3839447	0.488860247
ZNF691	229	187	0.122842804	0.3839707	0.488843376
PRLR	290	201	-0.113705267	0.3840689	0.488918472
C1orf53	115	76	-0.182410406	0.3848925	0.489891769
MOV10L1	115	76	-0.182410406	0.3848925	0.489891769
KIAA1456	400	320	0.093224037	0.3849973	0.489950063
CC2D2A	1752	1273	-0.045618224	0.3851021	0.490033407
ZFYVE21	1691	1228	-0.046413967	0.3853703	0.490324545

ALKBH2	258	178	-0.120341693	0.3854405	0.490363814
OR51E2	173	143	0.140395241	0.3856904	0.490631602
THAP9	224	183	0.123497048	0.3866379	0.4917867
ZNF318	2301	1773	0.039083684	0.3868244	0.491973651
ADA	377	264	-0.098874462	0.3869226	0.492048255
NR2C1	1184	922	0.054321707	0.3870022	0.492099215
PTK2B	139	93	-0.16463013	0.3871278	0.492208674
HIST1H2BG	43	39	0.274289596	0.3871731	0.492216128
SPAG7	2220	1619	-0.040304559	0.3872598	0.49227611
CLEC2D	283	229	0.109697677	0.3885266	0.493836007
CLDN7	56	35	-0.262919773	0.3887633	0.494061197
NHLRC4	56	35	-0.262919773	0.3887633	0.494061197
CBWD1	627	495	0.074115214	0.3887733	0.49399834
ZSCAN9	408	326	0.091454944	0.3895375	0.494918916
C22orf23	605	478	0.075227608	0.3900863	0.495565546
VIPAS39	1183	921	0.05397512	0.3902673	0.495744944
LOC440434	635	452	-0.075281687	0.3906381	0.496165368
PTGR2	363	254	-0.099988919	0.3906725	0.496158519
RRN3P1	82	53	-0.214479418	0.3909236	0.496426682
USP48	4534	3466	0.027639395	0.391232	0.496767727
LINC00260	480	339	-0.086597001	0.3915161	0.497077835
BCLAF1	16278	12084	-0.014673233	0.391862	0.497466206
PPP2CA	7687	5680	-0.021377607	0.3918946	0.497456935
FAM102B	1301	1011	0.051314167	0.3919142	0.497431052
SAMD3	69	44	-0.233940706	0.3922614	0.497821082
LOC100132707	53	33	-0.268374203	0.3925264	0.498081213
LOC100506874	53	33	-0.268374203	0.3925264	0.498081213
PTCHD4	910	712	0.061162828	0.3925989	0.498097138
UBIAD1	777	556	-0.067677584	0.3926532	0.4981153
PACSIN2	4117	3150	0.028910512	0.3926812	0.498100023
ASB7	1042	813	0.057124112	0.3932073	0.498716585
ZNF611	961	691	-0.060698589	0.3932951	0.498777155

RTKN2	234	161	-0.12429571	0.3935843	0.499093042
ARHGEF10L	931	669	-0.061622825	0.3937238	0.499219121
ZNF574	632	450	-0.074847425	0.3944616	0.500103704
ZNF195	1313	950	-0.051715366	0.3945048	0.500107593
GTF2H5	1834	1417	0.042998251	0.3950176	0.500706767
ENDOV	400	281	-0.094277738	0.3950706	0.500722925
SNRNP35	547	388	-0.080332049	0.3956217	0.501370439
ISL2	109	72	-0.183107192	0.3958396	0.501595538
B4GALT5	1909	1474	0.042071553	0.3959189	0.501645005
TXLNG2P	1397	1012	-0.049970599	0.3959277	0.501605038
KHDRBS3	621	490	0.073340613	0.3960859	0.501754468
CTNS	560	443	0.077032003	0.3970075	0.502870824
CEP68	733	576	0.067407745	0.3972542	0.503132152
CDK8	763	546	-0.067629974	0.3972792	0.503112541
ZNF235	171	116	-0.144719388	0.3974756	0.503310157
SLC6A17	89	58	-0.202600304	0.3975256	0.503322271
SNRNP200	11473	8707	0.017157079	0.3980318	0.503912034
ENOX2	629	448	-0.074409153	0.3983228	0.504229178
LPPR4	3110	2386	0.032831594	0.3985379	0.504450178
RCAN3	668	526	0.070366829	0.3986483	0.504538652
C7orf25	874	684	0.061515177	0.3993904	0.50542648
RTN3	7261	5527	0.021480491	0.3995869	0.505623787
QRSL1	1140	823	-0.054917357	0.3997566	0.505787125
TBC1D13	2025	1476	-0.041077055	0.4000875	0.506154327
CYB5RL	138	115	0.152117726	0.4002621	0.506323876
SEMA6B	109	92	0.170529763	0.4005012	0.506574895
SOX13	217	149	-0.12723058	0.4006101	0.506661146
ELFN1	368	258	-0.097182569	0.4006612	0.506674335
TRMT12	485	385	0.08202583	0.4008818	0.506901826
ZXDB	959	690	-0.059782322	0.4008923	0.506863628
GTF2IRD1	1254	907	-0.052210761	0.4008947	0.506815235
PSD	106	70	-0.183485306	0.4014789	0.507502276

ZNF317	1490	1081	-0.047793676	0.4015581	0.507550808
NAPA	2919	2138	-0.034060226	0.4016224	0.507580668
GBF1	10337	7849	0.017915348	0.4021626	0.508211775
LRRC42	1546	1197	0.046034965	0.4028684	0.509052005
TMEM44-AS1	96	63	-0.192530445	0.4028875	0.509024573
BOLA1	182	124	-0.138446198	0.4031558	0.509311846
BRI3BP	260	180	-0.115362585	0.4031856	0.509297856
C18orf8	753	539	-0.06721246	0.4032708	0.509353766
WDR47	2222	1622	-0.038932865	0.4032972	0.509335503
POLR3B	1331	1033	0.049481815	0.4038423	0.509972172
DUSP19	128	107	0.156619118	0.4038717	0.509957657
FBXL4	971	699	-0.059026708	0.4038902	0.509929277
MYH10	9614	7303	0.018504559	0.4039501	0.509953172
EFCAB13	296	206	-0.107800707	0.4039596	0.509913592
ZNF675	294	237	0.104223036	0.4041476	0.510099087
FAM131C	58	51	0.229596479	0.4041734	0.510080052
WDR83OS	1039	749	-0.057005899	0.4041913	0.510050967
LUC7L	1801	1391	0.04247637	0.4050926	0.511136523
PTOV1-AS1	744	584	0.06581788	0.4051162	0.511114538
USP42	979	764	0.05741591	0.4054818	0.511523952
PSRC1	731	574	0.066331462	0.4055634	0.511575049
CAPS	137	92	-0.159317995	0.405604	0.511574519
TUBGCP5	1728	1257	-0.043966436	0.4056518	0.511582988
ANK2	161	133	0.139517689	0.4057047	0.511597873
DNER	336	235	-0.100648344	0.4058556	0.51173633
SMAP1	1560	1133	-0.046246036	0.4061151	0.512011748
ACADM	1583	1150	-0.045875263	0.4064283	0.512354738
ACPP	60	38	-0.24381095	0.4065286	0.51242922
RASL10B	510	404	0.079010178	0.4069218	0.512873019
EPC1	1254	974	0.050608461	0.4071707	0.513134843
RP9P	289	233	0.104412594	0.4073059	0.513253184
GBAP1	200	137	-0.130671975	0.4075183	0.513468898

COMMD9	1446	1049	-0.047900743	0.4075578	0.513466814
KRCC1	2205	1698	0.038209935	0.4076246	0.51349902
VRK1	782	613	0.063870598	0.4076799	0.513516729
GRK6	1290	934	-0.050724479	0.4076951	0.513483909
CEP97	1008	786	0.056257711	0.4082887	0.514179613
TAF1	3184	2441	0.031784091	0.408341	0.514193486
FLJ20021	113	95	0.164828778	0.4084756	0.514310938
TCEANC	83	54	-0.204999797	0.4085968	0.514411565
DUSP28	93	61	-0.193269342	0.4086024	0.514366623
SURF1	553	393	-0.077598036	0.4091247	0.514972015
ABCC5	2594	1898	-0.035546355	0.409405	0.515272759
PCDHB16	553	437	0.075505931	0.4094993	0.515339358
STAG3L2	225	155	-0.122504654	0.4095026	0.515291458
NUDT8	68	59	0.21033234	0.4095408	0.515287525
HMOX1	1188	859	-0.052652668	0.409669	0.515396684
REEP5	7544	5739	0.02062183	0.40984	0.515559787
EIF4E2	2441	1877	0.036108487	0.4104774	0.51630938
DPCD	777	609	0.063679761	0.4105996	0.51641102
CCDC132	1535	1188	0.045448312	0.4106091	0.516370765
PSMA3	3567	2731	0.02987023	0.4109813	0.516786669
TBC1D22B	609	480	0.071744309	0.4111487	0.516944982
C16orf46	57	36	-0.247812881	0.4112417	0.517009693
MTO1	1278	992	0.049676321	0.411514	0.517299804
C8orf40	373	298	0.091288832	0.4115302	0.51726796
OCIAD2	344	241	-0.098223287	0.4115741	0.517271003
ANKRD61	78	67	0.195839103	0.4120238	0.517783889
ZC3H10	261	181	-0.112907978	0.4121709	0.517916486
VPS37C	845	661	0.060851062	0.4124605	0.518228114
CAMK1D	88	75	0.184539204	0.4125265	0.518258804
LOC100128881	141	95	-0.154543612	0.4127285	0.518460274
RPA1	4464	3286	-0.026852415	0.4129298	0.518660877
PPP1R37	1459	1059	-0.047125162	0.4130411	0.518748249

LOC100127983	100	66	-0.184309939	0.4131074	0.518779243
MED20	1379	1000	-0.048470325	0.4131745	0.518811154
HMGCL	1214	943	0.050713386	0.4137792	0.519518098
EIF4H	23705	17635	-0.011597686	0.4138162	0.519512224
C11orf75	80	52	-0.206336245	0.4142751	0.520009672
FOXD2-AS1	80	52	-0.206336245	0.4142751	0.520009672
CDC7	421	335	0.085492994	0.4143665	0.520045821
SIRT5	673	529	0.067813349	0.4144506	0.520099
SEPHS1	2176	1589	-0.0384073	0.4145228	0.520137183
KDM8	169	115	-0.140237254	0.4146393	0.520230966
UNK	883	635	-0.060504714	0.4146755	0.520224004
KATNB1	868	624	-0.060996882	0.4149325	0.520493946
KNTC1	2227	1714	0.037417683	0.4151667	0.520735329
FAM199X	4399	3238	-0.026920484	0.4151776	0.520696512
TPBG	5178	3817	-0.024803647	0.4151783	0.520644973
STRN4	3366	2471	-0.030788034	0.4153025	0.520748269
TMEM255B	54	34	-0.252272529	0.415898	0.521442432
SLC25A40	595	424	-0.073673272	0.4159572	0.521464237
FOXP1	5050	3853	0.02485093	0.4161141	0.521608405
LRRC34	117	98	0.159497256	0.4161466	0.521596631
FBLIM1	4672	3567	0.025823073	0.4162831	0.521715223
PSME1	3258	2391	-0.031220342	0.4164392	0.521858355
KIAA0907	2096	1530	-0.038954932	0.416699	0.522131393
KATNBL1	1881	1371	-0.041119146	0.4168958	0.522325379
LOC100505738	265	214	0.106770569	0.4169397	0.522327842
SNRPC	2443	1787	-0.035962097	0.4180123	0.523618953
SLC19A1	451	358	0.081984286	0.4181407	0.523727168
PIK3CD	1035	806	0.054373108	0.4181836	0.523728172
SENP2	2895	2122	-0.03298656	0.418462	0.524024117
C1orf220	62	54	0.215843324	0.4185825	0.524122334
PPP6R1	6192	4716	0.022310379	0.4190651	0.524673846
FOXQ1	114	76	-0.169810369	0.4195903	0.525278656

HES6	77	50	-0.207778219	0.4200493	0.525773963
NRG4	77	50	-0.207778219	0.4200493	0.525773963
FAM71F2	51	32	-0.25727321	0.4204674	0.526165087
RUNDC3B	51	32	-0.25727321	0.4204674	0.526165087
UFSP1	51	32	-0.25727321	0.4204674	0.526165087
PCDHGA1	159	108	-0.142843321	0.420477	0.526071422
WRAP73	433	306	-0.08568324	0.4206009	0.526173567
IMP3	1267	918	-0.049698334	0.4213474	0.52705457
FBXL8	97	82	0.172791294	0.4214365	0.527113008
S100PBP	1460	1130	0.045506535	0.4216832	0.527368602
HNRNPU-AS1	710	557	0.065010435	0.4218685	0.527547385
PELI2	507	401	0.076768621	0.4219118	0.527548559
DIS3L2	1017	792	0.054404788	0.4219876	0.527590424
LARS2	1636	1264	0.042975847	0.4220292	0.527589508
RNF31	1714	1248	-0.042597044	0.4222121	0.527765111
MMP19	1464	1133	0.045384439	0.422305	0.527828264
TSKU	574	409	-0.073797762	0.4235349	0.52931238
DEPDC1	2959	2170	-0.032262522	0.4236335	0.52938251
ATP6V1H	3375	2584	0.0298707	0.4238581	0.529610047
RBM17	3176	2331	-0.031109776	0.4240027	0.52973761
DNMBP	3859	2838	-0.028200323	0.4241348	0.529849499
ANKRD44	630	450	-0.070274695	0.4243507	0.53006598
CORO2A	154	127	0.137050278	0.424594	0.530316761
UNC13B	2597	1994	0.033971527	0.4246326	0.530311732
POU6F1	223	154	-0.118961227	0.4249662	0.530675125
ARHGAP19	889	640	-0.058959382	0.4250042	0.53066941
NSRP1	2774	2033	-0.033205441	0.4253189	0.531009061
PROCR	1154	835	-0.05164299	0.4253643	0.531012566
ZNF202	649	510	0.067430901	0.4255495	0.531190531
GLMN	722	566	0.063955348	0.425653	0.531266442
FAM200A	634	453	-0.069819658	0.4258567	0.531440839
NIT1	634	453	-0.069819658	0.4258567	0.531440839

HLA-DPB1	74	48	-0.209338733	0.4259197	0.53143955
CASP6	844	607	-0.060394351	0.4260108	0.531499953
IL15	232	188	0.111759988	0.4262065	0.531690963
ATM	9755	7405	0.017509994	0.4264013	0.531880612
MGC16275	84	55	-0.195805578	0.4264334	0.53186736
TCEA3	177	145	0.127455672	0.4267428	0.53220004
ROBO3	445	353	0.081014979	0.4268432	0.532271973
LYRM9	200	163	0.120024096	0.4268722	0.532254825
B3GAT3	1658	1207	-0.042866199	0.4270094	0.532372538
TM6SF1	493	350	-0.079080593	0.4271629	0.532510592
TRMT10C	1463	1063	-0.045636041	0.4272078	0.532513282
SUV420H2	149	123	0.138498117	0.4272515	0.53251455
ATP5J2	1024	797	0.053588046	0.4274702	0.532733727
STC2	3104	2278	-0.031208679	0.4278474	0.533150542
DRAM1	5185	3824	-0.024109334	0.4279895	0.533274173
FAM127B	1996	1457	-0.038958712	0.4279956	0.533228537
EARS2	1923	1403	-0.039691622	0.4280508	0.533243882
STIM1	2358	1725	-0.035815225	0.4281096	0.53326386
NT5DC1	1776	1370	0.040696443	0.4284348	0.53361559
GTPBP2	2416	1768	-0.035350048	0.4284987	0.533641721
EXOSC7	1011	787	0.053804675	0.4285402	0.533640153
FASTKD1	1041	752	-0.05401337	0.4286286	0.533696818
NIP7	1670	1216	-0.042552742	0.4287091	0.533743645
UBE2F	642	459	-0.068927012	0.4288345	0.53384649
SMYD5	1049	816	0.052778511	0.428995	0.533992843
PARD6G-AS1	61	53	0.212335249	0.4302212	0.535438866
PLXNC1	61	53	0.212335249	0.4302212	0.535438866
PCDHB15	101	85	0.166331585	0.4302355	0.535376516
KLC4	501	396	0.075841959	0.4303579	0.535475333
JAGN1	1049	758	-0.053592793	0.4304354	0.535518266
TMEM206	661	473	-0.067657956	0.4305784	0.535642617
MAEA	3334	2449	-0.029909199	0.4308754	0.535958517

NRBP1	5921	4372	-0.022395321	0.4312534	0.536375148
TNFRSF10A	181	124	-0.130497445	0.4313312	0.536418391
FAM184B	96	81	0.170039634	0.4314777	0.536547057
SBSN	91	60	-0.185751913	0.4314861	0.536503832
PNKD	1683	1299	0.041518386	0.4315505	0.536530384
PAPD7	2204	1695	0.036313181	0.4315525	0.536479361
UCN2	174	119	-0.132973601	0.4319544	0.536925321
ALPK1	444	352	0.080167884	0.4322792	0.537275456
HEIH	365	257	-0.090975973	0.432808	0.537879089
SDK1	222	180	0.112589362	0.4328928	0.537930748
TMEM248	8319	6320	0.018666573	0.433536	0.538676271
CARD10	574	452	0.070424167	0.4336002	0.538702239
NAA40	1274	924	-0.048248389	0.4336407	0.538698879
OSBPL1A	3914	2880	-0.027422813	0.4337113	0.538732787
ADARB1	1410	1091	0.045108071	0.4338283	0.538824409
HSD3B7	587	419	-0.071258128	0.4343272	0.539390331
C6orf136	347	277	0.090102445	0.4344431	0.539480378
MTBP	338	270	0.091088293	0.4354015	0.540616629
MKL2	1991	1454	-0.03831382	0.4362061	0.541561718
LOC100271722	50	44	0.230727561	0.4362773	0.541596111
IGSF8	1039	751	-0.05315871	0.4363436	0.54162437
GM2A	2759	2023	-0.032497005	0.43645	0.541702507
ZNF221	217	150	-0.11758041	0.4365221	0.541738022
FAM207A	513	365	-0.07591023	0.4365946	0.541773962
GLUL	5071	3866	0.023723495	0.4367167	0.541871497
FLJ42709	1106	859	0.050530783	0.4370244	0.542199312
PPP1R26	820	590	-0.059756824	0.4374893	0.542722078
NADKD1	1434	1109	0.044366473	0.4376224	0.542833075
NQO2	685	537	0.063970232	0.4376584	0.542823735
CYTH1	928	723	0.055022974	0.4378457	0.543001878
CCDC167	384	271	-0.087661328	0.4379352	0.543058875
FAM210A	894	697	0.056035956	0.4379579	0.543032942

PSMG3-AS1	342	273	0.090056758	0.4380088	0.54304199
DND1	171	117	-0.132335664	0.4381058	0.543108117
AGXT2L2	491	349	-0.077343856	0.438178	0.543143611
CDKN2AIPNL	813	635	0.058653371	0.4385913	0.543601863
LIPT1	143	97	-0.144806363	0.4387994	0.543805686
SH2D5	1135	881	0.049673758	0.439	0.544000085
TSEN54	322	226	-0.095585784	0.439252	0.544258253
PANX2	264	184	-0.105680032	0.4395536	0.544577735
EPB41L4A-AS1	366	258	-0.089320451	0.4404716	0.545660768
AFG3L1P	551	434	0.070794856	0.4405381	0.545688862
FAM86DP	881	635	-0.057233296	0.4406166	0.545731827
ARMCX5	538	424	0.071610224	0.4406959	0.545775868
FLI1	607	477	0.067444881	0.440839	0.545898813
BTBD7	4293	3277	0.025541632	0.4408937	0.545912216
C5orf34	248	200	0.104812011	0.4409399	0.545915131
LOC100507577	275	192	-0.103173176	0.440999	0.545934009
ARSG	138	114	0.139517689	0.4414202	0.546401229
CYP2R1	152	125	0.133008903	0.4416895	0.546680206
TOLLIP	2251	1647	-0.03557337	0.4418661	0.546844476
NUMB	6988	5168	-0.020121406	0.4421762	0.54717377
TCEB3	4206	3098	-0.025959574	0.4422327	0.547189347
PAN2	697	500	-0.064078429	0.44306	0.548158544
DHX8	2788	2137	0.031508479	0.4431142	0.54817122
OTUB2	279	224	0.098385742	0.4434474	0.548528885
PRDM4	2676	2052	0.032104747	0.443867	0.548993354
LOC730183	65	42	-0.214898259	0.4441008	0.549228006
WDR92	984	711	-0.053656624	0.444552	0.549731414
NFYC	931	672	-0.055167803	0.4446299	0.549773194
CCNE2	492	350	-0.076151262	0.4447158	0.54982478
PCDHB7	75	64	0.186333441	0.4448059	0.549881648
DYNLT1	2770	2032	-0.031833442	0.4449163	0.549963506
TAF1B	1032	802	0.051383303	0.4451324	0.550176011

CC2D1B	1991	1532	0.037075208	0.4452532	0.550270763
PLIN3	12905	9580	-0.014680449	0.4456196	0.550668917
DPH1	992	717	-0.05321487	0.4464366	0.551623803
UGDH-AS1	1255	972	0.046492987	0.4465146	0.551665412
SIAH1	367	292	0.085340438	0.4468303	0.552000745
VWA9	1350	981	-0.045482234	0.4471657	0.552360287
ALG6	679	487	-0.06433767	0.4471974	0.552344668
FBXL6	400	283	-0.084045815	0.4475514	0.552727132
CHRNB1	142	117	0.135769732	0.4476113	0.552746211
DCP1B	1034	748	-0.051973879	0.4476397	0.552726465
EMC1	11080	8219	-0.015770972	0.4477963	0.55286505
GINS3	229	185	0.107329804	0.4480807	0.553161332
ANKRD11	13317	9888	-0.014366334	0.4482812	0.553354044
KAT5	526	375	-0.073020072	0.4483675	0.55340572
SERINC2	6909	5253	0.019816804	0.4485553	0.553582633
NECAB3	777	559	-0.059914184	0.4485844	0.553563725
TCFL5	768	600	0.059008322	0.4487895	0.55376201
ZNF792	229	159	-0.111168701	0.4493149	0.554355355
C1orf213	116	78	-0.157426645	0.449393	0.554396741
OSBPL11	1560	1204	0.041441495	0.4497446	0.554775618
NOP9	1240	900	-0.047191082	0.4498477	0.554847776
CTC1	404	286	-0.083188014	0.449881	0.554833904
HNRNPD	11755	8723	-0.015226112	0.450286	0.55527837
LOC642846	62	40	-0.217116084	0.4503443	0.555295341
CHCHD3	2281	1670	-0.034666212	0.4505307	0.555470158
HAUS7	338	238	-0.090909541	0.4506789	0.555597944
RPL22	21412	16183	0.011211653	0.4509852	0.555920449
EPM2A	258	180	-0.104222027	0.4509924	0.555874297
LOC100126784	130	88	-0.147784063	0.4514603	0.556395963
RHOA	39442	29410	-0.008273793	0.4517191	0.556659904
FRMPD4	233	188	0.105554839	0.451853	0.556769795
PAPLN	72	47	-0.200184018	0.4519263	0.556804981

GHRLOS	54	47	0.214853481	0.4524695	0.557419101
CNNM3	620	444	-0.066556407	0.4526042	0.557529908
ADM	2502	1834	-0.032936019	0.4526383	0.557516814
CDC42EP2	910	657	-0.054821043	0.4526388	0.557462361
IQCC	183	149	0.118620814	0.4526719	0.557447926
C15orf38	322	257	0.089859803	0.4527311	0.557465758
LRWD1	920	716	0.053477858	0.4528986	0.557616796
C6orf57	190	131	-0.121280475	0.4529561	0.557632546
LOC100652772	583	458	0.067003847	0.4530207	0.55765696
THOC2	5414	4123	0.022151705	0.4531481	0.557758609
USP1	2457	1885	0.032818798	0.4532011	0.55776873
ADPRM	353	249	-0.088370309	0.4534726	0.55804773
RPN2	20992	15866	0.011250961	0.4538795	0.558493322
ZBTB48	379	301	0.08271777	0.4538879	0.558448426
PARD3	2696	1978	-0.031625939	0.4540028	0.558534633
LOC646903	113	94	0.149562021	0.4544371	0.559013709
GMEB2	850	613	-0.056423636	0.4551526	0.559838543
NUP205	4827	3679	0.023338981	0.4553913	0.56007693
GTF3C4	1771	1364	0.038431564	0.4553918	0.560022259
TSPAN6	1395	1078	0.043244188	0.4556222	0.560250172
LETM2	379	268	-0.084812716	0.455701	0.560291815
UTP18	1751	1278	-0.039139116	0.4557876	0.560342903
CDC14B	2460	1887	0.032588239	0.456124	0.560701225
CBWD2	383	304	0.081879063	0.4561817	0.560716813
NARG2	2453	1798	-0.033002081	0.4562016	0.560685882
TRIM68	745	582	0.058930859	0.4562614	0.560704046
PITRM1-AS1	127	105	0.140712963	0.4562955	0.560690657
PLEKHH2	907	655	-0.054455512	0.4563734	0.560731074
TMEM38A	59	38	-0.219563404	0.4566727	0.561043493
LRP6	6058	4609	0.020764321	0.4570783	0.561486337
ZNF362	1002	725	-0.051677477	0.4573071	0.561712035
TRIM56	1340	1036	0.043943134	0.4574044	0.561776185

VASH2	120	81	-0.151888461	0.4574781	0.561811292
HAUS8	295	207	-0.095932055	0.4575079	0.561792504
GATSL3	308	246	0.090880096	0.457582	0.561828001
TTC28-AS1	194	134	-0.11867152	0.4578591	0.562112905
LIN7B	127	86	-0.1472678	0.4582629	0.562553196
TP53RK	1528	1179	0.041071307	0.4585003	0.562789185
ZDHHC9	3758	2870	0.026237802	0.4588119	0.563116073
FMOD	96	64	-0.169810369	0.4588904	0.563156958
PBX2	3984	3041	0.025480301	0.4589129	0.563129069
C1orf74	248	173	-0.104415951	0.4590818	0.56328086
SH3RF3-AS1	230	160	-0.108409824	0.4594548	0.56368301
ATG4B	1787	1305	-0.038337696	0.4604511	0.564849641
LOC257396	259	181	-0.101810269	0.4604821	0.564832099
ZFAND2A	387	274	-0.083005541	0.4605474	0.564856473
IDNK	98	82	0.157994292	0.4611381	0.565497464
LOC100505648	98	82	0.157994292	0.4611381	0.565497464
PP7080	599	429	-0.066426224	0.4612297	0.565526228
TIGD2	738	531	-0.059756824	0.4613687	0.565641056
PTRHD1	354	250	-0.086669134	0.4614477	0.565682225
FAM171B	1599	1166	-0.040450019	0.4614885	0.565676538
AMZ2P1	347	276	0.084884736	0.4619232	0.56615362
MARS2	430	340	0.076350218	0.4620178	0.566213858
IFITM2	4114	3032	-0.025119909	0.4620334	0.566177314
ZNF836	254	204	0.098892787	0.4622933	0.566440095
KLF13	2412	1850	0.032447495	0.4625024	0.566640524
COMMD10	871	678	0.053764686	0.4627765	0.566892656
SMYD3	871	678	0.053764686	0.4627765	0.566892656
AMPH	252	176	-0.102696173	0.4628066	0.566845965
TAF1D	2416	1853	0.032394558	0.4628664	0.566863421
C20orf196	56	36	-0.222277789	0.4630793	0.567012615
ELMO3	56	36	-0.222277789	0.4630793	0.567012615
LHX4	56	36	-0.222277789	0.4630793	0.567012615

CUL4B	10895	8084	-0.015372838	0.4630846	0.566907713
MED30	159	109	-0.129546499	0.4639921	0.567962759
TFPT	473	373	0.072487579	0.4642226	0.568189112
CCDC40	117	79	-0.15143184	0.4644666	0.568431929
AP1S3	386	306	0.080082937	0.4645201	0.568441571
BCAS2	1718	1323	0.038235157	0.464523	0.568389261
DBF4	1095	794	-0.048567826	0.4649899	0.568904698
PIK3IP1	1930	1484	0.036042376	0.4650849	0.56896494
DDX28	245	171	-0.103633292	0.4651144	0.568945155
LEF1	124	84	-0.146726756	0.4652231	0.569022262
PODNL1	93	62	-0.169810369	0.4659954	0.569910886
PAN3-AS1	83	70	0.169395717	0.4662381	0.570151733
C5orf4	690	496	-0.06110411	0.4665366	0.570460764
NIF3L1	764	596	0.056891824	0.466555	0.57042723
NAV2	2480	1819	-0.032042446	0.4668944	0.570786246
HERC6	107	89	0.149418576	0.4671085	0.570991908
UROS	2364	1733	-0.032806249	0.4671295	0.57096151
LACE1	177	122	-0.121716081	0.4672793	0.571088607
PROSER2	238	166	-0.1046262	0.4674016	0.571182013
CINP	1429	1103	0.041579006	0.4679453	0.571790349
SEMA3E	63	54	0.19275971	0.4682856	0.57214996
USP44	1551	1131	-0.040447625	0.4683183	0.572133844
SSR4P1	68	58	0.185670286	0.4685142	0.572316962
BRPF1	1092	792	-0.048248389	0.4685168	0.572264067
NDUFA3	1278	988	0.043847242	0.4689822	0.572776358
PET112	878	683	0.052816771	0.4689979	0.572739335
C3orf33	195	135	-0.115362585	0.4690967	0.5728039
TACO1	978	708	-0.050932973	0.4691599	0.572824862
ZNF564	533	381	-0.069192404	0.4692852	0.572921717
APOBEC3F	300	211	-0.09256737	0.4694526	0.573069883
ERI3	1891	1454	0.036030038	0.4697832	0.573417255
FNTA	2057	1580	0.034534896	0.4700357	0.5736693

ACTR6	1055	818	0.048081881	0.4702938	0.573927988
FXN	528	415	0.067725539	0.4703573	0.573949323
LRCH3	1516	1169	0.040157308	0.4703836	0.573925139
NDRG3	3061	2341	0.028274021	0.471081	0.574719726
SLX4IP	149	102	-0.131591047	0.4716358	0.575340209
PMS2CL	206	143	-0.111477059	0.471826	0.57551589
CCDC112	842	608	-0.054596778	0.471839	0.575475343
ZNF491	266	213	0.094579316	0.4722081	0.575869135
TCN2	142	97	-0.134682146	0.4722458	0.575858811
YPEL2	376	298	0.079731801	0.472339	0.575916066
SAMD10	121	82	-0.146159101	0.4723473	0.575869751
TMTC2	63	41	-0.204575787	0.4724396	0.575925989
GRAMD4	676	486	-0.060914801	0.4724541	0.575887258
MADD	2451	1798	-0.031825332	0.4725101	0.57589908
DTL	1235	955	0.044213728	0.4728531	0.576260756
CFDP1	1914	1400	-0.036011871	0.4728621	0.576215338
GAS8	389	308	0.078312327	0.4728767	0.576176766
CLPTM1L	5205	3845	-0.021762434	0.4732824	0.576614681
GEMIN8	480	378	0.07050396	0.4734453	0.576756741
STAU1	8018	6086	0.017404048	0.473562	0.576842573
GOLGA8A	1444	1114	0.040830622	0.4737442	0.577008036
AP4B1	441	348	0.073460782	0.4737884	0.57700545
CCT6A	15457	11693	0.012536914	0.4738314	0.57700135
DHRS4	174	120	-0.120900768	0.4739916	0.577139996
MMP11	153	125	0.123548574	0.4741174	0.577236783
SIMC1	489	349	-0.071455297	0.4742891	0.577389433
THOC6	275	193	-0.095678639	0.4743211	0.577371912
PSMC4	4678	3453	-0.022889396	0.474647	0.577712115
PLD6	111	92	0.144298221	0.4748292	0.57787746
C9orf40	471	371	0.070844259	0.4754735	0.578605087
RNF111	2560	1961	0.030597857	0.4754979	0.57857821
C5orf45	125	103	0.135868374	0.4758289	0.578924348

ZNF782	210	146	-0.109268827	0.476251	0.579381287
FBXO28	3832	2824	-0.025205341	0.4763107	0.579397378
ZNF462	1657	1210	-0.038414423	0.4763646	0.57940634
STYXL1	986	765	0.049024233	0.4765967	0.579631998
TTK	1213	938	0.044232409	0.4766351	0.579622203
LINC00116	268	188	-0.096348207	0.4769841	0.579989926
METTL20	185	128	-0.116229329	0.4774973	0.580557323
CIDECP	356	252	-0.083301376	0.4775436	0.580556902
UNC119B	3297	2519	0.026849367	0.477626	0.580600379
AGAP5	153	105	-0.127990193	0.4777654	0.580713199
ALOX12	82	69	0.166124584	0.4778135	0.580715041
PLK3	1473	1074	-0.040611305	0.4779606	0.580837113
STXBP5	4065	3100	0.024164995	0.4781077	0.580959165
NDUFA12	1381	1006	-0.041930883	0.4781807	0.580991189
RUSC2	3416	2609	0.026341101	0.478273	0.581046657
ZNF595	221	154	-0.105963887	0.4782827	0.58100179
CCDC39	274	219	0.091907108	0.4785239	0.581238045
ELP2	3765	2873	0.02506026	0.4789006	0.581638933
ENOX1	52	45	0.20656551	0.4790676	0.581784979
SLC16A12	77	65	0.170733404	0.4792621	0.581964405
SLC48A1	587	421	-0.064388138	0.4793951	0.582069241
HSPA12A	1459	1125	0.04009725	0.479512	0.582154393
PROX2	60	39	-0.206336245	0.4795556	0.582150626
PPP1CC	9015	6837	0.016188065	0.479746	0.582325008
TBC1D24	1534	1182	0.039083684	0.4798256	0.582364809
RFC5	715	515	-0.058218678	0.479845	0.582331629
RAB27A	1370	998	-0.041912041	0.4801345	0.582626187
ANKH	1870	1368	-0.035817908	0.4803542	0.58283598
DPY19L1	3713	2736	-0.025364953	0.4804856	0.582938594
UCKL1-AS1	87	58	-0.169810369	0.4807469	0.583198899
RAP1GDS1	5158	3811	-0.021490033	0.4808099	0.583218499
TRIM32	1908	1466	0.034976064	0.4809935	0.583384384

QRICH2	62	53	0.188876276	0.4811073	0.583465595
BPHL	610	438	-0.062726241	0.48231	0.584867258
OXSR1	5421	4007	-0.020884365	0.4823946	0.584912838
PAPD4	2695	1980	-0.029632711	0.4828787	0.585442799
ZSWIM7	335	266	0.082417282	0.4829667	0.585492587
ZFP69B	396	313	0.075814359	0.4834083	0.585970843
FAM227B	94	63	-0.162156796	0.4834188	0.585898064
LOC100506469	94	63	-0.162156796	0.4834188	0.585898064
C12orf5	3845	2933	0.024545605	0.4834291	0.585825052
SNRPF	2149	1575	-0.033161522	0.4834489	0.585792006
LCMT2	390	277	-0.078436016	0.4835693	0.585880873
ACPL2	554	397	-0.065594837	0.4840286	0.58638028
CRISPLD2	4542	3353	-0.022723091	0.4843206	0.586677009
STAG3	77	51	-0.179209067	0.4846036	0.586962721
ZFYVE19	558	400	-0.06511299	0.4856409	0.588161974
MMP15	258	181	-0.096229237	0.4858527	0.588361249
PDGFRB	10404	7722	-0.014939713	0.4858664	0.588320676
SNHG10	170	138	0.114285652	0.4859112	0.588317636
TTLL7	1810	1324	-0.035934443	0.4860761	0.58846015
COMMD3	179	145	0.111245445	0.4866501	0.589097831
GSTCD	1076	833	0.045862455	0.486913	0.589358734
ZBTB25	215	173	0.10158751	0.4870272	0.589439697
HDAC1	5443	4024	-0.020619599	0.4870623	0.589424941
PCDHB9	115	78	-0.1449357	0.4871159	0.589432507
ARMC2	129	88	-0.136643505	0.4871323	0.589395069
FAM120A	12713	9623	0.013406249	0.4872387	0.589466569
CHSY1	4892	3724	0.021580801	0.4872777	0.589456435
RAB11B-AS1	122	83	-0.140545774	0.4873126	0.589441471
KIF11	3790	2794	-0.024713696	0.4873203	0.589393473
GPN1	1930	1413	-0.03468725	0.4873877	0.589417799
FAIM	308	245	0.08500353	0.4876236	0.589645854
MARK3	5283	3905	-0.02088273	0.4880569	0.590112495

ZNF71	486	382	0.067768456	0.4882877	0.590334265
LRRK1	506	362	-0.067995556	0.4882949	0.590285701
HES1	246	197	0.094689446	0.48884	0.590887193
TMEM204	480	343	-0.069673698	0.4892885	0.591371942
PACS1	4899	3729	0.021453637	0.4895462	0.591626055
FAXC	81	68	0.16276497	0.4896834	0.59173439
LRRC17	8186	6210	0.016586733	0.490156	0.592248107
GBA2	1715	1254	-0.036519097	0.4908244	0.592998159
FLVCR1-AS1	46	40	0.213518271	0.4908523	0.592974365
MYL5	142	116	0.123386007	0.4910386	0.593141835
IRX2	91	61	-0.161905171	0.4911376	0.593175176
MAP7D2	91	61	-0.161905171	0.4911376	0.593175176
MFSD1	8828	6694	0.015934053	0.4912401	0.593212683
LOC100288069	179	124	-0.114467335	0.4912403	0.59315541
SMEK2	4733	3496	-0.021897605	0.4913973	0.593287423
HEATR3	1079	835	0.04530537	0.491659	0.593545824
LOC100507053	114	94	0.136850969	0.4927302	0.594781398
TRA2B	6763	5136	0.01813398	0.4927733	0.594775759
NSFL1C	3430	2618	0.025408653	0.4931725	0.595199977
RFFL	140	96	-0.129168384	0.493228	0.595209266
LOC400657	294	234	0.085844506	0.4933793	0.595334182
MRPL46	792	616	0.052582052	0.4935656	0.595501214
ZNF589	358	254	-0.079978959	0.4938253	0.595756831
HAUS2	1613	1241	0.036908809	0.4939922	0.595900548
PSIMCT-1	190	132	-0.110309357	0.4940355	0.595895048
LOC100507117	56	48	0.19275971	0.4941063	0.595922714
LOC100272216	54	35	-0.210452353	0.4942101	0.59599018
GMNC	61	52	0.184854512	0.4943153	0.596059342
FXR1	9151	6937	0.01553461	0.4944782	0.596198094
RAB38	64	42	-0.192530445	0.4946201	0.596311389
RABL6	5502	4069	-0.020129727	0.4951105	0.596844899
ADRB2	219	153	-0.102247085	0.495535	0.597298847

AHNAK	239528	179255	-0.003028819	0.495963	0.597756784
PDCD4	3591	2647	-0.024875326	0.4961893	0.597971725
TMEM161A	771	557	-0.053901401	0.4966312	0.598446341
HMGXB4	2523	1931	0.02936009	0.4967675	0.598552697
CENPA	526	377	-0.065346144	0.4969055	0.598661004
PPP2R5C	6162	4561	-0.01889638	0.49785	0.599740912
RIOK1	1007	731	-0.04696824	0.4979044	0.599748452
WDR83	194	135	-0.107945113	0.4987032	0.600652595
CDK13	4395	3347	0.022157517	0.4989938	0.60094451
MSH6	4309	3282	0.022374272	0.4990407	0.600942856
LOC654433	609	438	-0.060359227	0.4991664	0.601036069
ASTN2	285	201	-0.088614286	0.4994172	0.601279985
ENTPD5	706	550	0.054915567	0.4994417	0.601251312
SYF2	1895	1388	-0.034038149	0.4994583	0.601213169
LOC100128398	144	99	-0.12541625	0.4995766	0.601297459
DCLK2	402	317	0.072439471	0.5001249	0.601899329
LOC652276	710	553	0.054612588	0.5006248	0.602442682
BMP8A	95	64	-0.154703477	0.5009358	0.602758661
PAPD5	1880	1377	-0.034051971	0.5009882	0.602763561
RTN2	715	516	-0.055420044	0.5010024	0.602722336
BOLA3-AS1	187	130	-0.109374515	0.5010474	0.602718259
BRD1	1835	1409	0.03404368	0.5012267	0.602875758
ACTN4	31425	23432	-0.008280515	0.5014423	0.603076904
LMBRD2	1244	960	0.041271957	0.5014709	0.603053022
DDX12P	51	33	-0.212879091	0.5017458	0.603325406
PIGC	553	397	-0.062988341	0.5017585	0.603282375
NOP58	4007	2957	-0.023236135	0.5018007	0.603274896
FAM126A	10991	8163	-0.01399916	0.5020253	0.603486654
MKNK1	807	627	0.051048901	0.5023651	0.603836888
HHLA3	123	84	-0.135044951	0.5023654	0.603778884
LOC653160	99	82	0.143347516	0.5026331	0.604042331
NDUFAF7	790	614	0.051538134	0.5028021	0.604187219

AZI2	2846	2094	-0.027522088	0.5028854	0.604228999
PDK3	253	202	0.09037004	0.5032322	0.604587389
C9orf172	198	138	-0.105680032	0.5032457	0.604545303
MTMR7	213	171	0.098295026	0.5034948	0.60478615
RIMS2	410	323	0.071062387	0.5042199	0.605598692
KRBA2	186	150	0.104812011	0.5045391	0.605923688
HPS3	1557	1138	-0.037116255	0.5046711	0.606023743
LOC643529	45	39	0.208701254	0.5057338	0.607241331
LAMB2	18075	13454	-0.010808352	0.5060039	0.607507065
PSMD13	6486	4925	0.017946833	0.5062069	0.607692286
KRBOX4	697	503	-0.055448124	0.506228	0.607658999
RRP8	614	442	-0.059040154	0.5067588	0.608237492
FASTKD3	520	373	-0.064183861	0.5069463	0.608403918
FICD	314	249	0.080533315	0.507041	0.608458984
LOC100131089	108	89	0.135998061	0.5070631	0.608426848
LOC100506599	85	57	-0.16134879	0.507207	0.608511575
PITPNA-AS1	85	57	-0.16134879	0.507207	0.608511575
USP6	50	43	0.197560697	0.5074799	0.608751011
KIAA0020	3054	2332	0.026019858	0.507815	0.609094361
MLLT10	2015	1545	0.031979131	0.5080995	0.609376934
FAM222A	55	47	0.18838127	0.5081221	0.609345361
TIAM1	184	128	-0.108409824	0.5081971	0.609376594
GLT25D1	11171	8457	0.013611677	0.5084799	0.609657045
ING5	777	562	-0.052192336	0.508614	0.609759101
COX7A1	275	194	-0.088222834	0.5088907	0.610032157
KLF16	1708	1312	0.034611877	0.5094055	0.610590504
TMEM19	1546	1189	0.036360528	0.5095062	0.610652408
PSMD4	5933	4392	-0.018731597	0.509653	0.610769533
MAP3K10	543	390	-0.062325942	0.5100885	0.611232609
FARP2	747	540	-0.052996704	0.5105332	0.611706651
LOC100271836	177	123	-0.109938913	0.5105904	0.611716417
LOC283335	58	38	-0.19490135	0.5105941	0.611661995

MAFG-AS1	103	85	0.138042541	0.5106336	0.611650433
MLLT11	3984	3035	0.022631001	0.5108643	0.611867923
LOC646329	577	415	-0.060307851	0.5110182	0.611993411
NBPF10	1301	949	-0.039988838	0.5113607	0.612344699
TEX261	5398	4103	0.019406309	0.5118763	0.612903211
MRPL18	2418	1777	-0.029218431	0.5122131	0.613247594
CDYL	1570	1207	0.035813249	0.5126995	0.613770836
MSANTD4	2457	1806	-0.028947833	0.5127432	0.61376421
TADA3	3282	2419	-0.025012338	0.5130299	0.614048339
FBXO42	1220	941	0.040537612	0.5132831	0.614292347
DDA1	3286	2422	-0.024981484	0.5132911	0.614242899
PHYH	1083	788	-0.043613576	0.5137057	0.614680066
STAU2	2261	1731	0.029796864	0.5137883	0.614719829
BEND3P3	550	430	0.060057173	0.5140836	0.615013994
ZNF92	653	471	-0.0562038	0.5143481	0.615271434
RWDD3	326	258	0.077651233	0.5147285	0.615667314
LOC100507156	294	208	-0.084080495	0.5147866	0.61567762
DCUN1D4	2936	2242	0.026086442	0.5150515	0.615935316
ANKDD1A	261	184	-0.089191909	0.5153749	0.616262884
ELP4	495	355	-0.064457369	0.5155761	0.616444333
OXA1L	3026	2229	-0.025863239	0.5161005	0.617012124
CCDC89	144	117	0.11559185	0.5164633	0.617386549
SH3KBP1	6997	5186	-0.016962154	0.5165774	0.617463667
HTR7P1	1265	975	0.039488871	0.5167636	0.617626915
CHRNA1	74	62	0.159895077	0.5167758	0.617582304
MMP25	65	43	-0.180950927	0.5168576	0.617620811
FUNDC1	529	380	-0.062116172	0.5169926	0.617722789
C8orf58	558	436	0.059215145	0.5170421	0.617722695
IL15RA	192	134	-0.103721179	0.5175202	0.618234636
ADAMTS10	298	211	-0.0829172	0.5177123	0.618404766
CYB5B	4585	3389	-0.020909965	0.5177557	0.618397355
MX1	107	88	0.133116764	0.5182623	0.618943008

SNRPN	96	65	-0.147442556	0.5185324	0.619206232
MAP3K3	1672	1284	0.034222487	0.5186771	0.619319642
PML	2951	2253	0.025795525	0.5187054	0.619294108
TMEM81	110	75	-0.137388891	0.5188731	0.619434855
SWSAP1	69	58	0.16460867	0.5189147	0.619425195
GART	3201	2442	0.024692655	0.5200523	0.620723614
ASNA1	2168	1660	0.029970617	0.5201412	0.620770254
FAM13B	3199	2358	-0.024905142	0.5201938	0.620773597
LINC00857	185	129	-0.105002073	0.5202448	0.620774982
SKA1	586	422	-0.058505534	0.5203765	0.620872621
PIGO	1434	1048	-0.037254175	0.5204661	0.620920062
GIT1	1762	1291	-0.033572792	0.5206396	0.621067554
BCHE	64	54	0.170039634	0.5206723	0.621047068
NUDT13	225	158	-0.094848311	0.5211075	0.621506672
ZBTB39	765	594	0.050155315	0.5211633	0.621513731
AHCTF1	4801	3651	0.020108865	0.5214877	0.621841031
MED27	1012	736	-0.044279487	0.5216053	0.621921746
YTHDF1	2596	1910	-0.027565613	0.5216133	0.62187166
ZNF738	436	312	-0.067629974	0.5222476	0.622568289
FAM134A	4231	3126	-0.021528776	0.522397	0.62268687
MYO1D	49	42	0.19275971	0.5224137	0.622617395
TMEM178A	49	42	0.19275971	0.5224137	0.622617395
APOL3	54	46	0.183826586	0.5225874	0.622705215
ST6GALNAC3	54	46	0.183826586	0.5225874	0.622705215
DENND2C	135	93	-0.122504654	0.5229121	0.623002661
ZNF687	1021	789	0.043266471	0.5230802	0.62314341
RHOT1	2303	1762	0.028851645	0.5234878	0.623569334
GPRIN1	1001	728	-0.044279487	0.5238772	0.623973501
PELO	2838	2167	0.025976696	0.5239099	0.623952751
ZNF138	316	250	0.077155668	0.5239462	0.623936304
OGFOD2	440	315	-0.066999563	0.5241959	0.624174027
ANAPC1	3419	2522	-0.023854015	0.5242223	0.624145745

CLDN15	128	88	-0.12541625	0.5244224	0.624324336
SMCR7	632	456	-0.055738602	0.5246118	0.624490055
ZNF155	424	333	0.066610044	0.524693	0.62452708
MPI	798	619	0.048702795	0.5247058	0.624482585
ZNF658	153	106	-0.114315256	0.5250652	0.624850617
SCAMP4	2819	2076	-0.026224902	0.5251107	0.624845105
RASA4	1339	978	-0.038097459	0.5254683	0.625210881
CMC4	171	119	-0.10788262	0.5255575	0.625257283
MZT2B	519	373	-0.061406776	0.5257009	0.625368174
MSRB1	823	638	0.047816125	0.525991	0.625653561
GALNT6	5208	3854	-0.019220745	0.5261769	0.62581488
FANCB	310	220	-0.07961256	0.5261855	0.625765338
GLB1	4041	2985	-0.021829288	0.5261988	0.625721418
CCDC94	388	277	-0.071018544	0.5264024	0.625903734
FAF1	1992	1462	-0.031122204	0.5264627	0.625915666
IQCB1	603	470	0.055654886	0.5269655	0.626453631
MIR22HG	2388	1756	-0.02835786	0.527238	0.626717769
ADTRP	52	34	-0.197824745	0.5274014	0.626852235
EIF3L	11621	8638	-0.012812866	0.5274409	0.626839327
ZNF280C	606	437	-0.056532382	0.5275089	0.62686035
GUSBP1	377	269	-0.071806219	0.5278428	0.627197315
RAB7L1	678	490	-0.053351392	0.5280157	0.627342937
SORD	2198	1615	-0.02950509	0.5280621	0.627338131
IBA57	591	426	-0.057152568	0.5282224	0.627468747
ZNF821	255	180	-0.087348209	0.5287723	0.628062108
SUPT3H	340	242	-0.075375567	0.5293914	0.628737476
TM4SF1	16542	12314	-0.010682068	0.5294702	0.628771047
BIN1	1340	979	-0.037700104	0.5296147	0.628882693
TESK2	69	46	-0.169810369	0.5300447	0.629333333
SLFN11	1107	854	0.040804885	0.5307527	0.630113944
TMEM258	633	457	-0.054859203	0.530787	0.63009455
ERF	2755	2029	-0.026131322	0.5313149	0.630661135

SEMA4G	318	226	-0.077551861	0.5315952	0.630933694
CACNG8	68	57	0.160579305	0.5324845	0.631928941
C2orf49	475	341	-0.063003642	0.5334806	0.633050733
PGM1	2223	1634	-0.028947833	0.5335336	0.633053379
ZNF397	754	585	0.049024233	0.5336537	0.633135555
ACTR1A	11223	8342	-0.012841023	0.5337044	0.633135341
SYDE2	374	267	-0.071046396	0.5338012	0.633189902
HERC1	6763	5014	-0.016549226	0.5338031	0.633131837
RFC4	679	491	-0.052536418	0.5339785	0.633279536
C3orf67	118	81	-0.127640915	0.5340905	0.633321945
STL	118	81	-0.127640915	0.5340905	0.633321945
COA4	1851	1358	-0.031669284	0.5344289	0.633632768
PCBD2	431	338	0.064487509	0.5348021	0.634014832
APBB3	404	289	-0.068133669	0.5349363	0.634113589
C1orf27	1484	1086	-0.035314857	0.5353157	0.634502975
OAS3	179	125	-0.102879361	0.535325	0.634453612
SURF6	1638	1257	0.033201425	0.535507	0.634608907
CLN3	820	595	-0.04758211	0.5355504	0.634600015
DNM2	6481	4804	-0.016828151	0.5356957	0.634711784
RND2	104	71	-0.135540467	0.5359144	0.634910484
LOC100505933	90	61	-0.145963627	0.5359184	0.634854796
PSMB9	187	150	0.097076362	0.5359565	0.634839531
CCDC125	262	208	0.082168848	0.5362362	0.635110504
ZNF23	1559	1197	0.033954356	0.5363884	0.635230378
MCM3AP-AS1	58	49	0.171880981	0.5363914	0.635173535
NFXL1	745	578	0.048981199	0.5364669	0.635202501
DGKD	866	629	-0.046154876	0.5367392	0.635464512
ZDHHC13	581	419	-0.056435788	0.5368412	0.635524888
AQP11	110	90	0.125645515	0.5369334	0.635543367
ARSE	110	90	0.125645515	0.5369334	0.635543367
SUPT20H	2911	2221	0.024846685	0.537053	0.635594335
TMEM17	160	129	0.104451292	0.5373685	0.635907373

LZIC	1222	941	0.038174475	0.5379757	0.636565414
MTERFD2	1197	922	0.038567635	0.5379842	0.636515027
SLC41A1	3038	2240	-0.024471006	0.5380412	0.636521921
ADNP2	1604	1231	0.033308752	0.5384554	0.636951482
ZNF319	604	436	-0.055068283	0.5388257	0.637328939
CTNNA1	27492	20733	0.008069152	0.5389398	0.637403425
EXOC1	3558	2710	0.022378473	0.5389615	0.637368584
CENPP	66	44	-0.169810369	0.539126	0.63750249
LRRC29	119	97	0.120247211	0.5391805	0.637506498
TIMM44	1309	1007	0.036750718	0.5395462	0.637878244
COQ10A	244	194	0.084327636	0.5400081	0.638363775
CLNS1A	3197	2437	0.023539639	0.5400601	0.638364723
ADCK2	646	467	-0.052959483	0.54088	0.639273193
MARCH7	8624	6532	0.014319745	0.541041	0.639402787
ZBTB40	2237	1645	-0.028325541	0.5410554	0.639359051
PIM3	803	622	0.046666724	0.5410631	0.639307593
ZFYVE1	2029	1553	0.029441096	0.5411201	0.63931428
ZNF354B	360	257	-0.071076415	0.5413904	0.639572905
VTI1A	1661	1274	0.032465336	0.5418316	0.640033496
EML2	958	740	0.042651747	0.5420378	0.640216262
NDUFAB1	2864	2111	-0.024952782	0.5421251	0.640258665
ZNF507	2086	1533	-0.029229329	0.5425478	0.640697119
PREP	2835	2163	0.024837062	0.5425728	0.640665958
ATP6V0D1	4663	3450	-0.019509936	0.5426826	0.64073478
TNPO3	5231	3973	0.018294155	0.542809	0.64082333
RNPEPL1	2059	1513	-0.029379711	0.5431161	0.641125109
CIAO1	4092	3113	0.020651563	0.543284	0.641262511
ZNF282	1405	1028	-0.035577734	0.543421	0.64136351
ANKRD36B	601	434	-0.054517817	0.5438567	0.64181689
AKR1A1	1784	1309	-0.031498386	0.5440909	0.642032459
CDC5L	3943	2914	-0.021140701	0.5442432	0.642151334
HADHA	14322	10823	0.011019611	0.5446136	0.642527487

HLA-L	94	64	-0.13943672	0.5451794	0.643103645
LPPR3	94	64	-0.13943672	0.5451794	0.643103645
WDR66	536	418	0.056422073	0.5453348	0.643195521
FAM78A	67	56	0.156417863	0.5464298	0.644425962
CCDC138	132	107	0.112224999	0.5466817	0.644661995
NR4A3	168	135	0.099650306	0.5468566	0.644807245
THEM4	915	707	0.043090603	0.5471275	0.645065557
SLC9A8	961	742	0.042034888	0.5473127	0.645222895
ADORA2B	278	220	0.077570773	0.5478472	0.645761346
ZSWIM3	278	220	0.077570773	0.5478472	0.645761346
ZNF567	330	260	0.071197731	0.5482865	0.646187433
TMEM30A	40698	30384	-0.006493956	0.5483805	0.646237071
H2AFY2	462	332	-0.061557478	0.5483894	0.64618633
IFT57	2315	1769	0.027073986	0.5484776	0.646229222
MAP6	109	89	0.122701238	0.5485498	0.646253067
ALDH7A1	2286	1682	-0.027495566	0.548601	0.646252312
GLA	1079	787	-0.040107192	0.5486213	0.646215098
METTL25	221	176	0.086681191	0.5486892	0.646233969
KLC1	8757	6631	0.013941875	0.5487442	0.64623759
ALDH5A1	62	52	0.16139554	0.5490679	0.646557674
ACAD8	792	575	-0.046786342	0.5493161	0.646788816
CRY2	1337	978	-0.035940963	0.5493232	0.64673608
SHC2	144	100	-0.11091668	0.549438	0.64681008
BSDC1	3644	2692	-0.021692417	0.5497609	0.647129047
ADRBK2	945	688	-0.042753633	0.5497652	0.647072971
HELB	81	67	0.141391319	0.5499205	0.647194558
F8A1	213	150	-0.090738798	0.5500596	0.647297111
WBP1L	2555	1882	-0.025904531	0.5500802	0.647260203
MRPS28	880	640	-0.044279487	0.5500961	0.647217691
SPTLC3	118	96	0.117471583	0.5504636	0.64758891
USP21	640	463	-0.05190758	0.550737	0.647849368
SLC37A3	3037	2315	0.023517417	0.5507572	0.647812001

AEN	3664	2707	-0.021572484	0.5508719	0.647885707
APPL1	2992	2207	-0.023871414	0.5510506	0.648034655
MEX3B	758	550	-0.047614098	0.5511286	0.648065201
COQ4	953	694	-0.04238842	0.5514791	0.64841613
FAM72A	70	47	-0.159542034	0.5516531	0.648559526
MRP63	911	663	-0.043290052	0.5521334	0.649062886
VMAC	155	108	-0.106084771	0.5521859	0.649063355
LOC643401	261	185	-0.081372404	0.5523335	0.649175557
ZUFSP	666	517	0.049794235	0.5523409	0.649123056
HOMER1	725	562	0.047741267	0.5523478	0.649069857
AP2B1	21127	15942	0.008896844	0.552571	0.649270934
ARMCX3	8515	6448	0.013997346	0.552742	0.649410627
LOC100506123	410	294	-0.064655623	0.5528278	0.649450182
RBM25	9429	7006	-0.013361653	0.5528339	0.649396012
LFNG	53	35	-0.183485306	0.5530137	0.649546017
MFF	2669	1967	-0.025150216	0.5531017	0.64958806
GMCL1	629	455	-0.05204134	0.5531699	0.64960691
COQ3	411	322	0.063074426	0.5533865	0.64980003
NSG1	299	236	0.073793507	0.5534909	0.649861321
EIF2AK1	6951	5157	-0.015536368	0.5536508	0.649987832
ZNHIT3	896	652	-0.043474636	0.5537526	0.65004607
FLJ37453	47	40	0.182491375	0.5538408	0.650057693
HPX	47	40	0.182491375	0.5538408	0.650057693
GSTZ1	217	153	-0.089011258	0.553885	0.650017653
H1FX	1326	1019	0.035225408	0.5540606	0.650162497
CD55	1748	1283	-0.031031883	0.5540913	0.650137283
ZBTB1	3048	2323	0.023278383	0.5541146	0.650103397
ARFGAP2	2535	1935	0.025499951	0.5543582	0.650327868
PSG1	276	196	-0.078662481	0.5544994	0.650432345
MTHFD2L	90	74	0.132752401	0.5546351	0.650530139
BECN1	2787	2055	-0.024422477	0.5561125	0.652201603
RAD51AP1	699	542	0.048152528	0.5561557	0.652190814

AKAP8L	1993	1465	-0.028888914	0.556306	0.65230572
ZNF121	531	383	-0.056215337	0.5564383	0.65239939
CHKA	623	484	0.050927016	0.556571	0.652493619
GNG7	123	85	-0.117971437	0.5567309	0.652619562
C6orf170	359	282	0.06686345	0.5568055	0.652645609
PYROXD2	466	364	0.058760627	0.5568654	0.652654416
ANXA2P2	660	478	-0.050303275	0.5569728	0.65271891
PPIB	6160	4672	0.016262055	0.557308	0.653050278
FAM216A	912	664	-0.042698451	0.5573226	0.653005946
PDCD11	4964	3676	-0.018214217	0.5575252	0.653181909
EFTUD1	1339	980	-0.035150175	0.5577961	0.653437762
HPS5	2468	1884	0.025608702	0.5579594	0.653567575
C2orf27A	85	70	0.135044213	0.5590003	0.654725272
C12orf66	258	183	-0.080375285	0.5594203	0.655155659
CRTC2	1016	741	-0.040202823	0.5596035	0.655308485
COG4	3605	2664	-0.021253045	0.5599582	0.655662257
TCF7L2	908	701	0.041874278	0.5600884	0.655753038
PCYT1A	3197	2435	0.02235516	0.5607207	0.656431636
GTF2IRD2	66	55	0.152117726	0.5607614	0.656417649
ELL3	171	137	0.0953317	0.561203	0.656872794
ZKSCAN7	298	235	0.072500558	0.5612062	0.656814843
DNAJC3	6658	5047	0.015489348	0.5612132	0.656761242
EGFL7	444	347	0.059528118	0.5612166	0.656703547
C7orf31	214	151	-0.087910115	0.5614553	0.656921141
HLA-C	9624	7153	-0.012936069	0.5615303	0.656947166
SNRPD3	4500	3419	0.018801553	0.5618125	0.65721561
DIAPH3	2450	1870	0.025408653	0.5624849	0.657940348
MIS18A	479	345	-0.058277162	0.5626693	0.658094321
TNIK	1506	1155	0.032323213	0.5630436	0.658470273
PCDHGA10	288	205	-0.07529277	0.5631171	0.658494333
TTC7A	995	767	0.039682184	0.5633746	0.658733611
TMEM55B	722	524	-0.047279894	0.5634715	0.658785034

PRPF40B	276	218	0.074812	0.5637703	0.659072547
CDKL1	88	60	-0.137388891	0.5639529	0.659224122
GPKOW	974	751	0.040043267	0.5639771	0.659190593
FAM174A	668	518	0.048256127	0.5642028	0.659392504
B3GALNT2	1565	1148	-0.031887883	0.5651758	0.660467738
SUCLG1	3191	2430	0.022099837	0.5655879	0.6608873
IL17RB	120	83	-0.116699033	0.5657253	0.660985783
VPS35	9832	7309	-0.01265875	0.5657924	0.661002156
MRPL50	961	741	0.040089243	0.5661413	0.66134782
CCDC113	219	174	0.083308568	0.5663456	0.661524339
ADAM8	56	47	0.162386061	0.5666302	0.661794709
SLC24A1	348	249	-0.067789432	0.5666412	0.66174557
METTL10	1302	1000	0.034422683	0.566735	0.661793053
LRRC1	696	505	-0.047651786	0.5675792	0.662716708
PQLC2	494	385	0.055499536	0.5679972	0.663142611
MICU1	3002	2216	-0.022813964	0.5681129	0.663215533
RBL1	849	618	-0.043005584	0.5685192	0.663627628
RCE1	310	244	0.069765064	0.5686752	0.663747527
GAREM	131	91	-0.11047623	0.5695467	0.664702358
ZNF511	259	184	-0.0780942	0.5697229	0.664845732
FAM218A	46	39	0.176992395	0.570379	0.665549035
RBM38	434	339	0.058742362	0.5708534	0.666040221
TOM1L2	5457	4140	0.016674856	0.5711208	0.66628974
LYN	356	255	-0.066227862	0.5711512	0.666262834
MTERF	557	433	0.051841829	0.5713916	0.666480811
TCF20	2297	1692	-0.025869157	0.5714679	0.666507446
PPP1R14A	64	43	-0.158583114	0.5714795	0.666458492
TXNDC9	2816	2146	0.023154874	0.5716559	0.666601795
TM2D1	1197	920	0.035434746	0.5717135	0.66660665
ZNF169	124	86	-0.112779424	0.5723282	0.667260882
DUSP23	107	87	0.116628641	0.5724865	0.667382933
AMZ2	3336	2465	-0.02137951	0.5727153	0.667587177

RPUSD3	800	582	-0.043828715	0.5727217	0.667532217
VEGFA	2426	1788	-0.025080682	0.5728111	0.667573943
C5orf56	71	48	-0.149632487	0.5732644	0.66803969
UBAP2	2799	2133	0.023124611	0.5733228	0.668045265
KIAA0317	4355	3224	-0.018668843	0.573414	0.668088955
PTCD3	2957	2183	-0.022669981	0.5734508	0.668069377
UBXN8	582	452	0.050455751	0.5734609	0.668018613
C9orf41	1292	992	0.033958087	0.5734928	0.667993369
NGDN	697	506	-0.046869139	0.573631	0.66809189
PASK	334	239	-0.067685353	0.5751323	0.669777733
DNTTIP2	3596	2735	0.020299944	0.5754206	0.670050776
LLPH	2121	1620	0.026401324	0.5754696	0.670045272
LOC728743	135	94	-0.107074614	0.5755543	0.670081233
STEAP2	497	387	0.054239846	0.5757434	0.670238695
WDSUB1	322	253	0.067228828	0.5757754	0.670213306
CROCCP3	644	467	-0.048486007	0.5758907	0.670284938
MR1	590	458	0.049784776	0.5760149	0.670366835
ZBTB26	309	243	0.068501607	0.5763972	0.670749031
RPL32P3	219	155	-0.083510523	0.5765352	0.670846999
CIR1	2030	1494	-0.027147448	0.5767431	0.671026159
NALCN	413	297	-0.060526719	0.5768078	0.671038782
PRKG2	594	461	0.049455951	0.5772748	0.671519384
NEURL4	1407	1079	0.032224668	0.5773358	0.671527609
CRNKL1	2011	1480	-0.027163774	0.5783048	0.672591834
LOC100379224	54	36	-0.169810369	0.5785485	0.672812453
NARS2	619	480	0.048247128	0.5789256	0.67318821
LOC220729	576	417	-0.050869296	0.5790212	0.673236482
PDXP	782	569	-0.043587823	0.5791519	0.673325569
SELPLG	373	292	0.06194487	0.5792989	0.673433667
SERPINI1	120	97	0.108174378	0.579881	0.674047368
RCL1	470	339	-0.056223352	0.5799858	0.674074795
ZNF212	470	339	-0.056223352	0.5799858	0.674074795

GAB3	129	104	0.104364594	0.580015	0.674014483
CENPN	1689	1241	-0.029514081	0.5800785	0.67402535
SLC9A6	2366	1744	-0.024897902	0.5803608	0.674290408
ING3	508	367	-0.053896302	0.5811182	0.675107526
BMS1P4	316	226	-0.068449654	0.5814421	0.675420725
SGK223	3398	2512	-0.020697257	0.5815147	0.675442125
SLC24A6	565	409	-0.050997893	0.5817238	0.675621982
LINC00663	61	41	-0.158033201	0.5819156	0.675781651
AZIN1	13721	10217	-0.010261856	0.5821896	0.676036926
C17orf80	1356	1040	0.032378482	0.5825162	0.676353113
SMIM10	652	505	0.046563555	0.5826481	0.676443165
POC5	321	252	0.066002568	0.5834246	0.677281478
MRPS25	1686	1239	-0.029276217	0.5834543	0.677252894
CNIH3	351	275	0.06311272	0.5836501	0.677417033
CYFIP1	9741	7244	-0.012131207	0.5838125	0.677542363
HLA-F-AS1	82	56	-0.135044951	0.5839093	0.677591602
SDHB	2623	1999	0.023212796	0.5842017	0.677867678
PYGO1	217	172	0.079865654	0.584447	0.678089154
PLCH2	295	232	0.068561983	0.5848326	0.678473387
THOC1	1090	838	0.035846146	0.5850896	0.678708341
XPO1	16228	12249	0.009330897	0.5851159	0.678675649
CTU2	676	491	-0.04614809	0.5852635	0.678783547
NASP	4542	3448	0.0175842	0.5857538	0.679289011
RAB6B	242	172	-0.077446351	0.5857606	0.679233588
ABCB10	1570	1202	0.029824469	0.5860258	0.679477895
HP1BP3	13166	9803	-0.010369743	0.5860286	0.679417928
MRPS5	1986	1517	0.026507594	0.5863798	0.679761719
FBXO7	4130	3137	0.018395879	0.5866087	0.679963843
ZNF506	418	301	-0.058587324	0.5867064	0.680013756
ITGA11	1512	1158	0.030329246	0.5867562	0.680008273
PSMD14	5764	4276	-0.015656351	0.5867802	0.679972734
GJC2	143	100	-0.100863015	0.5868567	0.679998199

PRRG1	2610	1926	-0.023289972	0.5869104	0.679997137
RAD51D	389	304	0.0594533	0.5870144	0.680054358
KIAA0355	2372	1749	-0.024421589	0.5871206	0.680114154
TEFM	529	411	0.051022803	0.5872662	0.680219492
PIAS4	684	497	-0.045598342	0.5875122	0.680441191
MRPS17	609	472	0.047496763	0.5879409	0.680874395
DCTN1	17991	13410	-0.00881401	0.588315	0.681244225
C9orf72	313	224	-0.067511793	0.5883511	0.681222778
BRF2	585	424	-0.049220228	0.5883963	0.681211685
PAXIP1	478	372	0.053444135	0.5887239	0.681527697
NAA38	1342	1029	0.032010442	0.5887715	0.681519388
ETAA1	1168	897	0.034291748	0.5888452	0.681541427
SNRPB2	2896	2139	-0.021972988	0.5891453	0.681825327
C14orf80	187	132	-0.087348209	0.5896125	0.682239271
LOC100499489	187	132	-0.087348209	0.5896125	0.682239271
NANOS1	187	132	-0.087348209	0.5896125	0.682239271
MAST3	1130	827	-0.035211406	0.5896335	0.682136851
MB21D1	177	141	0.087097934	0.589657	0.682068978
ZNF684	177	141	0.087097934	0.589657	0.682068978
AURKB	532	385	-0.051415668	0.5899704	0.682336446
CNOT2	2740	2023	-0.022527442	0.5902107	0.682550985
QTRTD1	1354	1038	0.031730837	0.5903978	0.682704033
HMG20A	1785	1313	-0.027905026	0.5906336	0.682913266
APOLD1	411	296	-0.058389086	0.5911028	0.683392344
CTTNBP2	635	461	-0.046837709	0.5914443	0.683723662
ZFP3	110	89	0.109525849	0.5914479	0.683664342
SPATA5L1	469	365	0.053460673	0.5921389	0.684399612
CYTH2	2944	2175	-0.021610139	0.5922528	0.684467736
APIP	1092	799	-0.035553316	0.5924167	0.684593566
TSC22D4	2823	2085	-0.022029613	0.5929311	0.685124538
PRR16	1239	908	-0.033259853	0.5935763	0.685806358
IDI2	181	144	0.085231246	0.5936655	0.685845829

SLIRP	1992	1521	0.025954637	0.5936925	0.685813363
RBM34	2185	1667	0.024772956	0.5939	0.685989481
TAF15	7009	5307	0.013840056	0.5943585	0.686455413
LIMCH1	79	54	-0.133741114	0.5943787	0.686415048
DNAJC28	65	44	-0.147784063	0.594422	0.686401502
TMEM216	150	120	0.093224037	0.5947383	0.686703054
JOSD2	850	620	-0.040042494	0.5950516	0.687001056
ZNF813	298	234	0.066348331	0.5952957	0.687219227
NMT2	3445	2619	0.019664209	0.5954371	0.687318754
NCOA5	1693	1245	-0.028284099	0.595451	0.687271074
TPM3P9	375	293	0.059162201	0.5956504	0.687437524
NSUN2	4345	3219	-0.017591468	0.5960927	0.687884202
R3HCC1	981	717	-0.037127886	0.5963015	0.688061422
DNAJC30	1035	757	-0.036113431	0.5965714	0.688309127
BAMBI	246	194	0.072550469	0.5972951	0.689080253
PUS7L	2504	1848	-0.023117674	0.5973261	0.689052166
ATG4D	430	335	0.054976568	0.5977319	0.689456493
FBXL15	314	225	-0.065687426	0.5977709	0.689437601
GRAMD1B	396	309	0.05725854	0.5977761	0.689379783
HDX	461	333	-0.054092442	0.5978441	0.689394338
ANKMY1	162	114	-0.091807857	0.5980037	0.689514562
HIST2H2BC	54	45	0.152117726	0.5987651	0.690328526
RNF187	3883	2875	-0.018457619	0.5991338	0.690689644
VANGL1	1978	1457	-0.025889417	0.5992106	0.690714305
TMEM186	288	206	-0.068272343	0.5996235	0.691126286
ZSCAN30	1331	1020	0.031210713	0.5996713	0.691117478
LSAMP	1182	907	0.033096552	0.5998057	0.691208373
YARS2	897	655	-0.038460947	0.5998577	0.6912043
MZF1	333	239	-0.063359427	0.6000752	0.691390959
TDRD3	548	425	0.04843908	0.6001025	0.69135847
OSGIN2	1401	1073	0.030345252	0.6006348	0.691907681
DYNC1I2	11891	8853	-0.010479588	0.6009569	0.692214707

PCDHGB3	438	341	0.053993001	0.6010202	0.692223612
SOD1	7241	5380	-0.013430646	0.6011237	0.692278868
SHROOM1	421	328	0.055027713	0.6012499	0.692360149
HTRA3	189	150	0.081728398	0.6013422	0.692402411
TMEM223	199	141	-0.081921136	0.6014236	0.692432152
METTL21A	895	689	0.037768432	0.6020691	0.693111261
CEP192	2371	1807	0.023252975	0.6022215	0.693222586
ZNF839	717	522	-0.04277118	0.6022294	0.693167658
SLC33A1	2244	1655	-0.024089327	0.602365	0.693259711
WDPCP	225	160	-0.076700965	0.6027092	0.693591748
CEP128	530	384	-0.049733917	0.6027683	0.693595628
KCTD13	425	331	0.054520508	0.6029275	0.693714736
LOC100288911	228	180	0.074115214	0.6032661	0.694040209
CMTM1	55	37	-0.156754216	0.6039744	0.694790866
ZNF33A	1668	1227	-0.027831908	0.6040163	0.694774959
BRPF3	3213	2443	0.019885015	0.6040679	0.694770107
LOC441666	108	75	-0.11091668	0.6041759	0.694830186
ACSS3	137	96	-0.09791745	0.6054316	0.696177846
GGT1	137	96	-0.09791745	0.6054316	0.696177846
EXPH5	331	259	0.061273013	0.6054781	0.696134917
CCT6B	62	42	-0.146726756	0.6055884	0.696165311
LOC399744	62	42	-0.146726756	0.6055884	0.696165311
STK32C	206	163	0.077379759	0.605819	0.696334022
SLC25A13	1033	756	-0.035229983	0.6058768	0.696336206
WFS1	4545	3448	0.016631612	0.6061615	0.696599109
HEXDC	148	104	-0.093861516	0.6065073	0.696932281
ZC3HC1	829	605	-0.039284828	0.6065124	0.696873814
PRKAG2	1799	1374	0.026338949	0.6068812	0.697233195
ZXDC	2521	1920	0.022262324	0.6069315	0.697226719
PNP	1879	1384	-0.025968992	0.6074042	0.697705344
NSL1	1490	1140	0.028873625	0.6075681	0.697829317
TOMM34	2987	2272	0.020417729	0.6076503	0.697859322

ZNF347	649	472	-0.044279487	0.6077984	0.69796508
ATP8A1	86	70	0.118170394	0.6080825	0.698162558
LOC100287015	86	70	0.118170394	0.6080825	0.698162558
MYO5B	86	70	0.118170394	0.6080825	0.698162558
KIAA1161	233	166	-0.073994582	0.6091892	0.699304305
FAM104A	2135	1628	0.024016762	0.6094201	0.699504849
KIF9-AS1	130	91	-0.099421041	0.6094709	0.699466477
LOC100132356	130	91	-0.099421041	0.6094709	0.699466477
LOC284385	304	218	-0.064591057	0.6096511	0.699576629
TADA1	424	330	0.053553891	0.6098515	0.699742042
IL6R	390	304	0.055749331	0.6102932	0.700184353
HPS4	1297	952	-0.030992869	0.6107571	0.700652034
IL17RD	222	158	-0.075482986	0.6108055	0.700643082
MECR	638	464	-0.044279487	0.6108569	0.700637522
ZNRD1	353	254	-0.059687555	0.610859	0.700575417
TRIM7	112	78	-0.106800571	0.61097	0.700638216
TRIM38	2833	2094	-0.020917028	0.6110393	0.700653173
HNRNPA1	2450	1866	0.022319369	0.6111263	0.700688367
C17orf58	263	188	-0.069178006	0.6113022	0.700825554
KDM1A	5120	3881	0.015436755	0.6116026	0.701105364
DKK3	61730	46151	-0.004457751	0.6116705	0.701118711
DUSP8	377	294	0.056403763	0.6121349	0.701586448
CRYBB2P1	623	453	-0.044568981	0.6127898	0.702272398
SAT1	1978	1509	0.024702512	0.6129561	0.702398397
MPHOSPH8	3308	2448	-0.019203545	0.613764	0.70325953
PTBP2	1013	778	0.034360018	0.6139964	0.703461004
CDON	105	73	-0.109268827	0.6144696	0.703938446
PTPLAD1	7647	5685	-0.012581393	0.6147207	0.704161325
CABYR	157	125	0.086315668	0.6147402	0.704118874
ZSWIM8	2361	1743	-0.02267334	0.6148777	0.704211682
SLC37A4	334	261	0.059353836	0.6153473	0.704684702
PPP1R11	1850	1412	0.02536695	0.6153629	0.704637773

HSF2	809	623	0.038244592	0.6156436	0.704894425
CITED4	145	102	-0.092331616	0.6159197	0.705113271
OPLAH	145	102	-0.092331616	0.6159197	0.705113271
DTNBP1	433	313	-0.053052236	0.6160249	0.705136529
FAM210B	5317	4029	0.014961471	0.6160253	0.705072167
SLC25A35	73	50	-0.130816237	0.6164135	0.705451591
LPXN	1430	1094	0.028749723	0.6164191	0.705393275
HPRT1	1364	1044	0.029430199	0.61646	0.705375253
MTA3	1397	1069	0.029081964	0.6164637	0.705314652
GMDS	704	513	-0.041464471	0.6165219	0.705316432
POLN	67	55	0.130422655	0.6166346	0.705380589
WDR77	2313	1762	0.02260079	0.6168648	0.705579096
STK19	286	205	-0.065239106	0.6169781	0.705643959
WWC1	461	358	0.050344969	0.6171282	0.705750777
PEF1	2143	1581	-0.023642351	0.6172626	0.705839661
S100A3	116	81	-0.10297886	0.6174278	0.705963777
KCTD11	962	704	-0.035309333	0.6177548	0.706272814
SPDYE7P	735	536	-0.040359118	0.6183799	0.70692258
ZNF354A	524	406	0.047065047	0.6184952	0.706989535
SNX3	7879	5959	0.012209844	0.6185034	0.706933954
FERMT1	219	156	-0.074232709	0.6190418	0.707484477
LOC100133091	127	89	-0.097799124	0.6193279	0.707746472
YBEY	388	280	-0.055477693	0.6197444	0.708157429
LAMA2	1458	1115	0.028205122	0.6197646	0.708115572
JKAMP	3052	2320	0.019521975	0.6197947	0.708085029
LCA5L	48	40	0.152117726	0.6198347	0.708065798
THOC7	2189	1668	0.022999466	0.6203725	0.70861515
DCAF15	812	625	0.037528594	0.6216689	0.710030844
C9orf64	121	97	0.096201737	0.621791	0.710105149
LOC100505806	1191	874	-0.031316097	0.6221474	0.710447002
CCDC65	85	69	0.114285652	0.622203	0.710445372
DNTTIP1	766	590	0.038522694	0.6226589	0.710900781

HIATL2	112	90	0.099650306	0.6227757	0.710969011
FBXO38	2837	2098	-0.020199341	0.623039	0.711204339
ZC2HC1A	1048	768	-0.033308369	0.6230606	0.711163843
DNAL4	333	260	0.058141578	0.6231772	0.711231787
LOC100506060	103	83	0.103691036	0.623275	0.711278144
C10orf25	84	58	-0.119184296	0.6237341	0.711704304
SERPINB9	84	58	-0.119184296	0.6237341	0.711704304
RPL36AL	5427	4111	0.014486595	0.6237579	0.711633648
GADD45A	3075	2337	0.019223455	0.6238744	0.711701357
PDSS2	1331	978	-0.029452069	0.6240501	0.711836576
SLC39A6	8313	6285	0.011695665	0.6241893	0.711930149
PELI3	405	315	0.052582052	0.6244252	0.712134048
FOXP2	388	302	0.053644029	0.6248622	0.712567166
TEX30	419	303	-0.052480318	0.6255156	0.713247032
UBOX5	332	239	-0.059020492	0.6255349	0.713203678
DNAJC19	671	489	-0.041326169	0.6260538	0.713730034
CEP70	757	583	0.038354715	0.6262308	0.713866479
ATP2A2	26421	19904	0.006525451	0.6262842	0.713861941
ALS2CR8	665	513	0.040756617	0.6274827	0.715162583
CHST7	264	207	0.06424497	0.6284342	0.716181526
SP110	1394	1066	0.028129009	0.6284883	0.716177655
CPSF4	883	646	-0.035727141	0.6285793	0.716215863
LOC155060	465	337	-0.049329993	0.628711	0.716300364
TRIQK	1764	1346	0.024979981	0.6290255	0.716593169
FGGY	306	220	-0.060875997	0.6290273	0.716529622
FANCE	328	256	0.057600127	0.629051	0.716491148
PGAM2	56	38	-0.144275277	0.6292629	0.71666687
PKDCC	887	649	-0.035563495	0.6293693	0.716722505
IFI35	251	197	0.065660397	0.6295355	0.716846252
TEK	1375	1011	-0.02849649	0.6297555	0.717031215
RANBP9	3908	2965	0.016753769	0.6300995	0.717357378
CDC6	891	652	-0.035401336	0.6301542	0.717354054

FASTKD2	2652	2017	0.02028244	0.6308259	0.718053065
THAP5	1903	1451	0.02392409	0.6308899	0.718060231
NEDD8	1823	1344	-0.024629292	0.6311761	0.718320362
DMWD	1948	1485	0.023621385	0.6312653	0.718356256
CBY1	522	404	0.045457618	0.6312816	0.718309136
PCNX	9522	7194	0.010681662	0.6320805	0.719152474
TMSB15A	183	130	-0.078179894	0.6324018	0.719452404
LRRC37A3	363	262	-0.055250605	0.63264	0.719657596
ISY1	329	237	-0.058048393	0.6327284	0.719692461
GATS	639	493	0.040923847	0.632894	0.719815088
LOC100507557	52	43	0.140977168	0.6329325	0.719793169
CHCHD7	1886	1438	0.023886132	0.6329622	0.719761174
ELL2	16547	12479	0.008084783	0.632974	0.719708881
ANKRD54	1081	793	-0.03182162	0.6333358	0.720054576
B4GALT4	2193	1670	0.022094422	0.6339382	0.720673675
ACAT2	884	647	-0.035128526	0.6341164	0.720810364
LOC202781	120	96	0.093224037	0.6342032	0.720843288
ERC1	5921	4399	-0.013513121	0.6343386	0.720931386
MFSD8	1240	949	0.029292003	0.6345661	0.721124209
C1orf216	1248	917	-0.029472164	0.6348493	0.721380145
DNAAF2	647	499	0.040426235	0.634963	0.721443593
NBN	5062	3758	-0.014590309	0.6349721	0.721388037
CCDC102A	462	335	-0.048579624	0.6349938	0.721346973
HFE	772	594	0.037014215	0.6351146	0.721418325
MAP6D1	190	150	0.074115214	0.6351951	0.721444021
BPGM	1680	1238	-0.025297787	0.6359906	0.722281649
DCAF11	1647	1257	0.025296226	0.6364002	0.722680881
SLC9A9	303	218	-0.059837527	0.6364936	0.722721142
MINA	1260	926	-0.029187503	0.6364952	0.722657047
TTC5	584	451	0.042311196	0.6367611	0.722892997
FRMD5	233	183	0.066665825	0.6369544	0.723046548
ZBTB43	1299	955	-0.028676661	0.6372791	0.723349281

GTF2F2	2979	2205	-0.018897336	0.6373986	0.723419003
GLUD1P3	243	174	-0.066716876	0.6374044	0.723359598
COL13A1	2866	2178	0.019117476	0.637791	0.72373245
NDUFS3	1860	1418	0.023707043	0.6378636	0.723748826
RAD54L	280	219	0.060656174	0.6381777	0.724039251
MEX3A	1899	1401	-0.023630818	0.6382157	0.724016475
ENY2	2239	1654	-0.021743162	0.6384075	0.724168052
LOC100009676	110	77	-0.099421041	0.6384307	0.72412839
C1orf50	394	285	-0.052081579	0.6385687	0.72421897
LINC00595	61	50	0.128270984	0.6386208	0.724212098
DOCK7	10290	7663	-0.01010964	0.6387912	0.724339447
POLL	723	528	-0.038305586	0.6390034	0.72451412
LOC100129361	1560	1191	0.025779516	0.6391328	0.724594879
ZNF135	217	155	-0.070274695	0.6391659	0.724566404
FRS2	2694	2048	0.019617996	0.6394742	0.724849908
RALGPS1	154	109	-0.083450084	0.6398087	0.725163049
ZSCAN31	92	64	-0.108409824	0.6398882	0.725187228
PBDC1	879	675	0.034176469	0.6402785	0.725563532
TCEAL7	585	426	-0.042431062	0.6404527	0.725694955
GXYLT2	284	222	0.059820879	0.6404957	0.725677652
ZNF814	1005	737	-0.032306845	0.6405455	0.725668008
NEK8	115	92	0.093224037	0.6413581	0.726522509
GNG4	60	41	-0.134186459	0.6415171	0.726603484
SH3GL1P1	60	41	-0.134186459	0.6415171	0.726603484
PLEKHF1	172	136	0.076350218	0.641797	0.72682139
BCL11A	70	57	0.118759129	0.6419253	0.726900624
YWHAB	20610	15532	0.007051239	0.642102	0.727034614
NPC2	1861	1373	-0.023594298	0.6421104	0.726977958
DET1	411	319	0.049570162	0.6421402	0.726945653
RPL36A	281	202	-0.061062706	0.6423996	0.727173241
KIAA1598	570	415	-0.042698451	0.6427031	0.727450662
NUDCD2	593	432	-0.041848661	0.6427617	0.727450896

SDHAP1	266	191	-0.062701476	0.6428084	0.727437662
CISD1	1011	775	0.03163735	0.642839	0.727406159
TOR2A	228	179	0.066077895	0.6435755	0.728173457
TMEM130	377	293	0.051488273	0.6437603	0.728316345
XRCC6BP1	97	78	0.100641508	0.6437741	0.728265787
MTHFSD	574	418	-0.042395663	0.6438873	0.72832766
C3orf37	1081	828	0.030488282	0.6440737	0.728472335
NPHP3-ACAD11	215	169	0.067838718	0.6444867	0.728873367
SNAPC3	3213	2380	-0.017807276	0.6445453	0.728873401
GSTO2	114	80	-0.095809788	0.6446017	0.72887097
RPP38	532	387	-0.043940548	0.644705	0.728921694
ERN1	285	205	-0.060185878	0.6448024	0.728965644
TVP23C	372	269	-0.052544317	0.6452168	0.729367881
ADHFE1	270	194	-0.061750623	0.645344	0.72944551
RWDD4	2570	1901	-0.019857696	0.6455458	0.729607307
LOC285074	255	183	-0.063501467	0.6456491	0.729657864
EIF4A2	15307	11415	-0.008108567	0.6459771	0.729962385
LOXL4	402	312	0.049502659	0.6462621	0.730218159
COG3	2271	1728	0.020927643	0.646304	0.730199187
CHCHD1	1035	793	0.030914135	0.6466494	0.730523154
GPR3	119	95	0.090189977	0.6468302	0.730661133
SNHG15	279	218	0.059215145	0.6468508	0.730618203
PGAP3	465	360	0.045918322	0.6472464	0.730998773
SMIM11	456	331	-0.047050476	0.6477479	0.731498809
SAYSD1	368	286	0.051461512	0.6479192	0.731625888
KIFC3	2861	2118	-0.018664777	0.6482152	0.731893767
NPAS1	266	208	0.060309414	0.6486103	0.732273538
IL17RE	193	152	0.070622608	0.6488575	0.732486245
ZCCHC4	640	467	-0.039497223	0.6491224	0.732718867
KIAA1328	300	234	0.056698161	0.6493401	0.732898183
MAFG	2221	1690	0.020965985	0.6493559	0.732849548
ALG5	1323	1011	0.027122067	0.6495272	0.732976414

TFB2M	931	714	0.032295038	0.6496058	0.732998742
UBE2D4	372	289	0.050919003	0.649651	0.732983379
CAPZB	14154	10676	0.008313518	0.6497069	0.732980077
TRIM52	494	382	0.044213728	0.6498278	0.733049979
MTSS1	771	564	-0.035883566	0.6498463	0.733004505
AGO3	790	607	0.034995995	0.6500393	0.733155745
IAH1	1590	1213	0.024704917	0.6501302	0.733191954
ALG14	393	305	0.049432062	0.6504468	0.733482538
ZDHHC23	118	83	-0.092451486	0.6504687	0.733440803
CNTRL	775	567	-0.035695443	0.6507069	0.733642971
NDUFAF5	529	385	-0.043257145	0.6507429	0.733617248
TMEM177	218	156	-0.067629974	0.6509334	0.733765561
CCT6P3	248	178	-0.063310748	0.6511877	0.733985797
NMRK1	725	530	-0.036836504	0.6513838	0.734140321
AVL9	1524	1123	-0.025352843	0.6514292	0.734125102
UBE2L6	1199	917	0.028314112	0.651481	0.734117004
SRC	1602	1181	-0.024713051	0.6515888	0.734172155
C14orf2	2619	1938	-0.019295357	0.6518323	0.734379988
DIMT1	1491	1138	0.025372432	0.6518792	0.734366496
HCG18	1688	1245	-0.02401703	0.6523369	0.734815556
PTCHD3P1	1244	915	-0.027990705	0.6523985	0.734818527
LEMD2	2247	1661	-0.020795919	0.6524722	0.734835138
POLR1E	1614	1190	-0.024526873	0.6528228	0.735163514
METTL1	411	298	-0.048673931	0.6537174	0.736104343
OLFML3	1548	1141	-0.024954548	0.6540096	0.736366837
PTPLAD2	155	110	-0.07961256	0.6540149	0.736306217
TMEM106C	1155	849	-0.028904261	0.6540701	0.736301833
SGCD	1363	1041	0.026336638	0.6542833	0.736475206
HLX	214	168	0.066002568	0.6543282	0.736459183
ANXA7	5092	3855	0.013650573	0.6543963	0.736469359
NIPAL3	12035	8970	-0.00890412	0.6547303	0.736778632
WIPI2	4389	3258	-0.014753535	0.6548849	0.736886012

PAIP1	5343	3970	-0.013358878	0.6551132	0.737076287
LINC00339	144	102	-0.082347528	0.6551464	0.737013847
PSTK	144	102	-0.082347528	0.6551464	0.737013847
PIWIL4	60	49	0.12297138	0.6555181	0.737332052
UBXN7	3149	2390	0.017268992	0.6558652	0.737655823
APOE	122	86	-0.089320451	0.6560558	0.737803582
MTMR11	859	659	0.032772466	0.6578549	0.739760078
KIAA1656	69	56	0.113982597	0.657954	0.739804702
ZNF284	235	184	0.062197141	0.6585842	0.740446482
SLC35A4	2429	1797	-0.019619949	0.6586142	0.740413409
CEBPZ	2557	1943	0.018985872	0.6587686	0.740520065
RELL1	1448	1105	0.025136899	0.659608	0.741396726
FAM22D	179	141	0.070887707	0.6597995	0.741545115
MRPS18A	850	623	-0.033078546	0.6602126	0.741942389
ZNF425	279	201	-0.057917489	0.6605906	0.742300244
TMEM14B	1862	1375	-0.022269323	0.6607557	0.742418825
C17orf104	126	89	-0.086394361	0.6613846	0.743058339
DCUN1D2	800	586	-0.033947204	0.6616563	0.743296551
UQCRFS1	3757	2848	0.015520162	0.6618729	0.743472858
NUP107	2101	1553	-0.020866201	0.6621693	0.743738674
PIGP	454	330	-0.045074141	0.662191	0.743696065
KCNS3	290	226	0.055422004	0.6621996	0.743638613
DDHD1	1890	1439	0.021832489	0.6622492	0.743627322
MRPL42P5	55	45	0.125645515	0.6623958	0.743724889
CCDC110	68	47	-0.117721858	0.6627836	0.744093131
CFL1	43454	32691	0.004556656	0.662829	0.744077121
SNX12	3306	2451	-0.016564109	0.663268	0.744502803
C10orf32	727	532	-0.035376987	0.6638193	0.745054466
ERI1	2049	1559	0.020853075	0.6640441	0.745239598
GDI2	18415	13877	0.006966447	0.6642756	0.745432258
VPS26A	4625	3502	0.01387785	0.6644628	0.745575147
SERF2	2298	1747	0.019652942	0.6647172	0.745793394

ID4	391	303	0.047301318	0.6654909	0.746594193
ENO3	61	42	-0.123267783	0.6656971	0.74675831
YIPF3	6877	5198	0.01132942	0.6660214	0.74705475
SLC39A13	4190	3174	0.014504016	0.6660824	0.747055937
BMS1	4397	3330	0.014154776	0.6661831	0.747101602
MGME1	1469	1083	-0.024649021	0.6662579	0.747118223
NOG	247	193	0.059241937	0.6662756	0.74707085
ZNF420	298	232	0.053964606	0.6663893	0.747131086
MRPL52	2477	1882	0.018824891	0.6665025	0.747190725
HECTD4	4654	3457	-0.013798475	0.666789	0.747444621
FILIP1	119	84	-0.087348209	0.6669845	0.747596521
LOC646214	79	55	-0.107268903	0.6678767	0.748495454
NCF2	79	55	-0.107268903	0.6678767	0.748495454
CHST10	386	280	-0.048021889	0.6679446	0.748470502
SLC25A3	22722	16967	-0.006206196	0.6682621	0.748758959
IGHMBP2	720	527	-0.035041813	0.6683051	0.748739786
MATN3	329	238	-0.051973879	0.6684097	0.748789611
FUK	348	252	-0.050511441	0.6684769	0.748797504
NEIL3	227	163	-0.062668201	0.6685017	0.748757998
LYRM4	770	591	0.033451816	0.6686158	0.748818437
C1orf174	716	550	0.034624163	0.6691473	0.749346361
LOC643201	50	41	0.128847947	0.66928	0.749360127
RAB11FIP4	50	41	0.128847947	0.66928	0.749360127
SLC27A5	50	41	0.128847947	0.66928	0.749360127
JRKL	1683	1282	0.022513217	0.6707658	0.750888754
RNGTT	2468	1827	-0.018713629	0.670943	0.751019639
PXMP4	449	347	0.04337235	0.6711787	0.751215927
ZMAT5	231	166	-0.061557478	0.6712835	0.751265703
SMAD7	976	717	-0.029755897	0.671563	0.751510964
JMJD6	1252	922	-0.026243775	0.6717082	0.751605948
MTFR1L	2147	1588	-0.019959147	0.6726279	0.752567374
HAT1	2320	1763	0.019059801	0.6728112	0.752704901

MVB12B	524	404	0.039940613	0.6729661	0.752810521
C9orf156	293	228	0.053285291	0.6729915	0.752771413
GNRH1	276	215	0.054820525	0.6733658	0.753122453
RBM33	2948	2185	-0.016951113	0.6739506	0.75370874
PHF14	1627	1201	-0.022825968	0.6741534	0.753867849
FOXP4	1592	1213	0.022891346	0.6741814	0.753831469
MPRIP	17786	13275	-0.006878098	0.6743982	0.754006264
COL11A2	77	62	0.102561901	0.6744797	0.754029696
ZNF585B	715	549	0.034015039	0.6748775	0.754406685
CRTC3	1686	1284	0.022192798	0.674926	0.75439317
ZSCAN20	540	394	-0.039611646	0.6751216	0.754544117
MRPL44	1477	1126	0.023689133	0.6751304	0.754486194
ZBTB8A	841	617	-0.031683179	0.6752592	0.754562422
CHD1L	3085	2340	0.016390172	0.6755327	0.75480032
PDRG1	617	451	-0.036990924	0.6755336	0.754733646
SBF2-AS1	108	86	0.086529384	0.6755819	0.754719913
CSDC2	1035	761	-0.028510277	0.6756224	0.754697523
LOC100506730	307	222	-0.052526847	0.6758559	0.754890633
ANKAR	164	117	-0.072035153	0.6761745	0.755178794
SLC1A5	8488	6322	-0.009891489	0.6765891	0.755574051
DUT	2759	2094	0.017268117	0.6766059	0.75552501
YEATS4	452	329	-0.043083057	0.6768144	0.755690112
MEAF6	2953	2189	-0.01675727	0.6771995	0.756052366
MRPS18C	179	128	-0.068663646	0.6772695	0.756062696
MOCS3	598	437	-0.037360073	0.6772958	0.756024257
CALML4	423	327	0.043785104	0.6774049	0.756078289
APBA1	1300	992	0.025052534	0.6774604	0.756072515
RAD9A	173	136	0.067986745	0.6782653	0.756902994
CEP19	134	106	0.076983396	0.6788389	0.757475263
ETV1	3466	2627	0.015296678	0.6788851	0.757458945
PCDHB14	305	237	0.051229948	0.6789876	0.757505466
CDH13	8723	6498	-0.009676514	0.6791061	0.757569814

CDK12	5729	4261	-0.011939155	0.679261	0.757674651
MGAT3	190	149	0.064465044	0.680365	0.758838148
TMEM63A	1660	1264	0.021965354	0.6804351	0.758848467
PEX11A	254	198	0.055824065	0.6804434	0.758789706
HAS2-AS1	54	44	0.119696248	0.680551	0.758841823
CADM4	309	240	0.050579699	0.6809139	0.759178426
ITPKC	1069	817	0.027298262	0.6809697	0.759172747
PTCD2	537	392	-0.038916302	0.6813101	0.75948426
LOC400680	63	51	0.11029755	0.6824561	0.760693614
RHNO1	1159	885	0.026020926	0.6832912	0.76155627
EPSTI1	120	85	-0.082347528	0.6834865	0.761705822
RGAG4	821	629	0.030829927	0.6838837	0.762080339
CCS	955	702	-0.028877571	0.6839351	0.762069447
ZFAND4	202	145	-0.063150261	0.6839998	0.762073356
ANKHD1	187	134	-0.065653138	0.6840432	0.762053474
BASP1	9015	6717	-0.009358331	0.6841951	0.762154574
C6orf203	705	541	0.033157468	0.6847733	0.762730355
ANKEF1	453	330	-0.041892894	0.6848664	0.762765941
BICC1	2123	1571	-0.019269059	0.6849655	0.762808031
MAP2K7	1413	1077	0.023408916	0.685428	0.763254862
CASD1	835	613	-0.030736992	0.6854453	0.763205883
MTERFD3	359	278	0.046253171	0.6854616	0.763155788
UBXN4	10676	7959	-0.008559997	0.6855246	0.763157788
WDR73	1035	791	0.027270964	0.6861079	0.763738848
TCF7	580	424	-0.036836504	0.6861112	0.763674287
ZFAT	457	333	-0.041519856	0.6862011	0.76370613
SGK494	255	184	-0.055639349	0.6866286	0.764113609
PPP5C	3389	2515	-0.015149105	0.686744	0.764173693
CCDC111	363	281	0.045752714	0.6870731	0.764471638
TCEB2	4501	3345	-0.013087222	0.6879447	0.765373013
ZNF181	455	351	0.040756617	0.6881238	0.765503888
LRRC66	49	40	0.122370383	0.6883205	0.765620122

PKDREJ	49	40	0.122370383	0.6883205	0.765620122
MAPK1	10789	8044	-0.008424033	0.6887019	0.765941765
SNX6	11241	8382	-0.008251825	0.6887313	0.765906083
DNM3	85	68	0.093224037	0.6888167	0.765898516
UTS2D	85	68	0.093224037	0.6888167	0.765898516
SLC25A38	1449	1104	0.022834709	0.6890877	0.766097184
CDC14A	270	210	0.052582052	0.6893431	0.76631277
DVL3	4987	3772	0.012309605	0.6895913	0.766520279
NFYB	1412	1076	0.023090121	0.6896077	0.766470124
MRPL13	1533	1132	-0.022331607	0.6896554	0.766454744
CD37	62	43	-0.112779424	0.6897086	0.766342854
LOC100289230	62	43	-0.112779424	0.6897086	0.766342854
POMZP3	62	43	-0.112779424	0.6897086	0.766342854
ZNF774	62	43	-0.112779424	0.6897086	0.766342854
RBM26	3149	2387	0.015456941	0.6900821	0.766586954
DHRS12	91	64	-0.092642508	0.6901006	0.76653911
MTL5	102	72	-0.087348209	0.6903565	0.766720826
RBP1	102	72	-0.087348209	0.6903565	0.766720826
GSKIP	1420	1082	0.022961701	0.6904231	0.766692241
TPCN1	2127	1616	0.018759297	0.6906672	0.766894864
RNF219	538	414	0.037176727	0.6907069	0.766870575
GIPR	58	47	0.111759988	0.6907414	0.766840504
RAD51B	146	115	0.070817624	0.6912565	0.767344015
COX19	1112	849	0.025831803	0.6915881	0.76764373
MEMO1	2451	1816	-0.01745415	0.6928582	0.768984918
SP140L	1440	1063	-0.022775083	0.6930822	0.769165022
DUS4L	425	328	0.041385105	0.693604	0.769675452
ACAD11	169	121	-0.066864067	0.6936375	0.769644102
PCNA	3888	2888	-0.013805345	0.6937827	0.76973664
ZNF438	233	168	-0.05671659	0.6942959	0.77023744
LINC00087	111	88	0.080167884	0.6945799	0.770483876
ZNF786	227	177	0.056209195	0.6950099	0.770892157

UBE2E3	7266	5487	0.010008342	0.6951814	0.771013788
NFATC1	252	182	-0.054333152	0.6952594	0.771031573
TXNDC16	218	157	-0.058411444	0.69549	0.771218602
FAM122C	137	108	0.072007551	0.6956053	0.771243489
POLR2J4	137	108	0.072007551	0.6956053	0.771243489
ETNK2	1111	848	0.025429485	0.6963003	0.77191103
SPATA6	489	357	-0.038758259	0.6963065	0.771849134
ZNF224	95	67	-0.088614286	0.6965767	0.772079978
EXO5	366	283	0.044110536	0.6966384	0.772079656
LINC00341	483	372	0.038431564	0.6966703	0.772046371
ZNF382	158	113	-0.068449654	0.6966749	0.771982735
JUN	6493	4905	0.010520059	0.6969991	0.7722733
NCS1	6974	5267	0.010147227	0.6971175	0.772335735
TXNRD3	543	397	-0.036661059	0.6971398	0.772291794
NME6	493	360	-0.038438608	0.6975027	0.772625109
LOC100134259	80	64	0.093224037	0.6976427	0.772711365
KAT6A	4481	3390	0.012616679	0.6979654	0.773000076
SNHG16	7174	5343	-0.009975596	0.698957	0.774029511
DPH5	1133	835	-0.025147627	0.6992043	0.774234469
KIAA1211	62	50	0.104812011	0.7000131	0.775026717
LOC152225	62	50	0.104812011	0.7000131	0.775026717
ZNF341	218	170	0.056358743	0.7000546	0.774969332
METTL2A	661	507	0.032487607	0.7001634	0.775020947
CLK4	851	651	0.028650543	0.7001692	0.774958449
USP32P1	962	735	0.026859488	0.7011837	0.776012384
MRPL55	532	389	-0.036503959	0.7012885	0.77605935
NDUFV3	2261	1675	-0.017647765	0.7013077	0.776011681
SEPSECS	559	409	-0.035595308	0.7013727	0.776014633
PDZD11	2375	1760	-0.017199953	0.7016001	0.776197253
FAM185A	188	147	0.060235625	0.7019978	0.776568272
BTN3A2	1157	853	-0.024619086	0.7022431	0.776770651
NDUFS4	2509	1860	-0.016657718	0.7028744	0.777399887

CAND2	196	141	-0.06000636	0.703078	0.7775561
FAM98C	298	231	0.047732653	0.7032688	0.777697983
IPO8	4657	3522	0.012146159	0.703328	0.777694496
NUDCD3	4610	3428	-0.012247509	0.7036077	0.777934698
ZNF747	249	180	-0.052996704	0.7040203	0.77832176
RPH3AL	59	41	-0.109938913	0.7040575	0.778293752
ARHGAP20	1097	837	0.024888134	0.704272	0.778461846
LOC284751	84	67	0.088923899	0.7043516	0.778446204
SNORD17	84	67	0.088923899	0.7043516	0.778446204
PRDM5	243	189	0.052582052	0.7045149	0.778523049
DEDD	819	602	-0.028947833	0.7054964	0.779538462
WASF3	1678	1276	0.020037745	0.7057051	0.779699863
HEATR1	4433	3353	0.012321252	0.706228	0.780208371
CUTC	348	269	0.043670998	0.7066657	0.780622767
ZNF672	761	559	-0.029896039	0.706802	0.780704006
ASS1	185	133	-0.060946893	0.7068714	0.780711424
FBXL18	2566	1946	0.016142671	0.7069498	0.780728842
ZNF768	862	659	0.027742728	0.7074869	0.781252729
ZMYM6NB	529	387	-0.035782024	0.7076746	0.781390744
EPB41L4A	196	153	0.05783013	0.7077176	0.781368902
MRPS10	2899	2197	0.015131668	0.7080212	0.781634812
TMEM208	1088	802	-0.024852283	0.7082438	0.781811234
LOC441155	52	36	-0.115362585	0.7085193	0.782046104
LGALSL	57	46	0.105824074	0.7090944	0.782611543
ATAD5	614	471	0.032640536	0.7092131	0.782673159
XKR6	81	57	-0.091807857	0.7092688	0.782665236
C15orf39	560	430	0.034061964	0.7100881	0.783499974
ZNF784	217	169	0.054480336	0.7103168	0.783682857
TRIM35	1272	969	0.022622032	0.7103725	0.783674933
YPEL1	88	70	0.08500353	0.7106318	0.783891502
FUT10	816	600	-0.02845452	0.7107701	0.783974668
ZNF43	541	396	-0.034976032	0.7108459	0.7839888

RWDD2A	437	319	-0.038924724	0.7109734	0.784059965
COQ9	1194	910	0.023287746	0.7110696	0.784096642
RBM22	1958	1487	0.018176014	0.7114544	0.784451565
MLL5	5475	4137	0.010878125	0.7115769	0.784517192
CENPV	418	305	-0.039541568	0.7127928	0.785788149
LOC100505702	127	100	0.070323635	0.7130278	0.785977577
SPRED3	1703	1260	-0.01950257	0.713064	0.785948
ZNF383	204	159	0.055609745	0.7131188	0.785938814
TMEM126B	1468	1085	-0.021004794	0.7131472	0.785900596
C1orf54	828	633	0.027726864	0.7132108	0.785901153
AP5B1	1011	745	-0.025318535	0.7132696	0.785896382
PIP5K1A	4758	3597	0.011591019	0.7133872	0.785956466
USP35	832	612	-0.027899744	0.7134887	0.785998697
STARD10	445	325	-0.038213486	0.7135158	0.785959079
RGAG1	79	63	0.088651307	0.7137377	0.786133938
ZRANB1	2910	2160	-0.014835709	0.7143797	0.786771571
ZNF430	613	470	0.031925815	0.7155554	0.787996703
TMEM179B	1283	977	0.022061429	0.7160967	0.788523066
NEIL1	85	60	-0.087348209	0.7161108	0.788468893
CYP2E1	70	56	0.093224037	0.7162966	0.788603736
ZBTB11	2441	1851	0.015984732	0.7165405	0.788802496
UBA5	4064	3074	0.012368895	0.7171311	0.789382939
RBM23	1755	1299	-0.018917468	0.7172375	0.789430267
ASPHD1	407	297	-0.039413732	0.7173029	0.789432534
SLC25A19	61	49	0.099124638	0.7180332	0.790166438
FAHD2A	757	579	0.02842218	0.7186288	0.790751932
TLCD2	334	258	0.042675095	0.7186826	0.790741298
LRRC8A	4889	3695	0.011187091	0.719346	0.791401285
SETD6	220	159	-0.053324626	0.719385	0.791374307
TCEAL4	3443	2606	0.013323035	0.7194084	0.791330216
AAR2	1274	970	0.021843507	0.7197379	0.791622653
CCDC8	633	485	0.030931379	0.7198149	0.791637543

SLMAP	3953	2939	-0.012470464	0.7198195	0.791572639
C12orf4	1813	1377	0.018301766	0.720034	0.791738662
MLL	9089	6855	0.008187224	0.7200508	0.791687247
ZHX1	2765	2095	0.014822895	0.7201384	0.791713623
IDE	4674	3533	0.011388147	0.7205909	0.792141138
NAP1L4	6203	4620	-0.009921142	0.7206107	0.792093046
ARHGEF3	469	343	-0.036227215	0.7207619	0.792189296
PLA2G15	2216	1681	0.016513975	0.7207669	0.792124917
KATNAL2	100	71	-0.078956939	0.721038	0.7923529
SOCS1	199	155	0.054651916	0.7212243	0.792487754
TMEM18	880	672	0.026109841	0.7213052	0.792506673
RBPMS	4281	3237	0.011861662	0.721419	0.79256182
C5orf55	156	112	-0.062895165	0.7216539	0.792749987
RIMKLB	999	762	0.024458451	0.7218935	0.792943239
DDX24	7272	5488	0.009080414	0.7221448	0.793149315
ADSL	2679	2030	0.014937278	0.7223003	0.793250161
PER2	525	403	0.033614548	0.7224563	0.7933515
ARL15	787	579	-0.027648156	0.7234122	0.794331213
MDGA1	1011	771	0.0241719	0.7234527	0.794305643
ZNF442	74	59	0.088341815	0.7235009	0.794288548
SMNDC1	2055	1523	-0.01707032	0.7235371	0.79425825
MRS2	1551	1179	0.019517164	0.723643	0.794304527
ZFHX4	1101	839	0.023080379	0.7244473	0.795117331
SMA4	304	235	0.043741565	0.7246019	0.795216881
ZNF767	632	484	0.030234621	0.7261086	0.796800277
UTP20	3608	2682	-0.01273797	0.726321	0.796963107
DDI2	287	222	0.044661071	0.7266582	0.797262845
SPDL1	2259	1713	0.015993012	0.7267372	0.797279256
DDX19B	578	424	-0.031853096	0.7269671	0.797461287
IGBP1	2225	1650	-0.01618718	0.7270235	0.797452875
LRP5L	179	129	-0.05743639	0.7272259	0.79760466
CAPN3	100	79	0.07507669	0.727308	0.797624518

DDIT4L	228	177	0.049867668	0.7276787	0.797960772
TTC23	967	713	-0.024461681	0.728387	0.798667102
ITPKA	143	112	0.062635717	0.728482	0.79870097
CADM1	9493	7081	-0.007758835	0.7292315	0.799452324
SETDB1	1333	1014	0.020533004	0.7301502	0.80038905
HSCB	532	390	-0.03279999	0.7301831	0.800354755
SMG8	1323	978	-0.02075456	0.7301988	0.800301477
OBFC1	944	720	0.024362179	0.7303763	0.800425637
NAA60	1468	1086	-0.019675733	0.7305077	0.800499143
OSGEPL1	391	301	0.037747011	0.7305113	0.800432673
RAB35	2734	2030	-0.014381384	0.730919	0.800808965
TM7SF2	202	146	-0.053234792	0.7310759	0.800910373
WHAMMP3	215	167	0.050663575	0.7311574	0.800929227
TPGS1	351	256	-0.040175089	0.7318352	0.801601225
AKAP10	2050	1555	0.016442802	0.7321256	0.801848785
ULK4	181	141	0.054857597	0.7326552	0.802358233
CCDC15	378	276	-0.038565836	0.7327484	0.802389731
PIGG	2123	1610	0.016108449	0.7329245	0.80251208
SFSWAP	1781	1352	0.017559767	0.7332616	0.802810598
MSH3	1979	1467	-0.01675061	0.7333671	0.802855494
SP2	775	592	0.026552997	0.733506	0.802936979
PARS2	206	149	-0.052179875	0.7336567	0.803031432
INO80C	309	225	-0.042529705	0.7336794	0.80298563
EID2	1050	775	-0.022968981	0.7339506	0.803211909
GGT5	97	69	-0.076236254	0.7340695	0.803271439
C5AR1	53	37	-0.103314957	0.7348251	0.803992377
CHAD	53	37	-0.103314957	0.7348251	0.803992377
REPS1	1589	1207	0.018458683	0.735041	0.804122583
NIPAL2	440	322	-0.035290704	0.7358771	0.804966534
MRPL38	1289	953	-0.020552013	0.7360486	0.805083431
MOCS1	869	663	0.024804825	0.7363544	0.805347261
WAC-AS1	3153	2386	0.013021005	0.7368897	0.805861879

ZYG11B	6179	4604	-0.009333408	0.7369927	0.805903818
CCDC73	95	75	0.074115214	0.7372702	0.806136402
TMCC2	290	224	0.042597964	0.7375918	0.8064173
SIM2	51	41	0.100278794	0.7383205	0.807143129
MTFR2	172	134	0.054976568	0.738588	0.807364759
CBX7	321	234	-0.040912636	0.7386157	0.807324106
SPPL3	1922	1458	0.016534515	0.7389122	0.807577302
SRPX2	2315	1718	-0.015130025	0.7392216	0.807844557
ARHGEF4	101	72	-0.07313435	0.7393417	0.807904998
SLC31A2	425	311	-0.035396129	0.7395096	0.808017584
TAF3	1106	842	0.021692885	0.7398886	0.808360715
PQLC3	897	684	0.024040472	0.7402808	0.808718256
GPHN	572	438	0.030067855	0.7403306	0.808701816
MMP24	1149	849	-0.021390207	0.740501	0.808816954
LTB4R	218	158	-0.049251445	0.7409778	0.80926678
STRBP	352	257	-0.038654938	0.7411921	0.809429882
TBK1	2401	1819	0.014662271	0.7412252	0.809395079
ZKSCAN4	323	249	0.039763709	0.7414716	0.809593119
PCDHGB7	427	328	0.034611877	0.7414924	0.809544919
CNEP1R1	1412	1073	0.019062119	0.7417888	0.80979746
WDTC1	2363	1754	-0.01481875	0.7418409	0.809783388
TMEM56	90	64	-0.076700965	0.742006	0.809892637
TNFSF9	86	68	0.076350218	0.7422032	0.810036882
FBXO8	794	606	0.025330918	0.7423948	0.81017505
SLC30A6	3143	2378	0.012758575	0.742408	0.810118385
ZNF532	5077	3781	-0.010056279	0.7429465	0.81063504
TNFRSF10D	16279	12161	-0.005598081	0.7435195	0.811189162
CLN5	2193	1662	0.015166702	0.7440545	0.811701751
DNM1L	6510	4912	0.008805149	0.7442498	0.811843698
SNRNP40	1377	1019	-0.019222376	0.7444682	0.812010835
IPPK	662	487	-0.027757313	0.7445324	0.812009695
ZNF587	2482	1843	-0.014294913	0.7446477	0.812064327

SPHK1	534	392	-0.030833956	0.7452347	0.812633309
UQCRC2	7253	5408	-0.008330566	0.7453164	0.812651293
RABIF	775	571	-0.025553433	0.7454052	0.812677025
FAM169A	116	91	0.064965777	0.7456185	0.812838386
WRAP53	414	318	0.03454813	0.745775	0.812937813
ALG10	264	204	0.043183354	0.7458638	0.81296346
ZRSR2	472	362	0.032354969	0.7458753	0.812904862
HJURP	1350	999	-0.019250692	0.7465404	0.813558581
GTPBP10	1366	1038	0.019001092	0.7466703	0.813628894
IRAK4	2245	1701	0.014819828	0.7468749	0.813780727
ZNF233	77	61	0.079102929	0.746979	0.813822922
DPY19L4	3510	2654	0.011849472	0.7470533	0.813832631
ZNF557	608	447	-0.028644361	0.7472903	0.814019662
GGN	94	67	-0.073347529	0.7475195	0.814198089
HIST1H4C	90	71	0.073046155	0.7476394	0.814257486
SLC6A16	150	117	0.056698161	0.7478268	0.814390422
SLC16A6	226	175	0.046184281	0.7483072	0.814842277
SPIN3	612	468	0.028129009	0.7485173	0.814999824
ORC4	1842	1397	0.016211091	0.7490565	0.81551564
FAM49A	686	524	0.026510367	0.7491174	0.81551068
CMTM3	1194	908	0.020113498	0.749155	0.815480281
BRD4	4134	3077	-0.010861813	0.7492782	0.815543169
C9orf123	1186	877	-0.02030313	0.7493928	0.815596547
EXTL1	272	210	0.041934808	0.7494297	0.815565467
HIRA	1649	1222	-0.017194982	0.749592	0.81567085
PHF23	2646	2003	0.013501491	0.7496037	0.815612264
ZNF384	1712	1269	-0.016838501	0.7501096	0.816091446
TMEM164	1414	1074	0.018364005	0.7507846	0.816754487
ZBTB12	177	128	-0.052453418	0.7512095	0.817145402
ZNF79	405	311	0.034144804	0.7513148	0.817188564
C1orf109	776	592	0.024692655	0.7514605	0.817275685
ZRANB2-AS1	68	54	0.082576793	0.7514788	0.817224193

SRGAP2D	107	84	0.066002568	0.751538	0.817217168
ZBTB24	858	654	0.02346512	0.7516952	0.817316818
IFI27L1	384	281	-0.035384049	0.7520401	0.817620457
ZNF81	669	511	0.026469212	0.7525162	0.818066619
FGFR1OP	825	629	0.023818029	0.7528027	0.8183067
CCDC171	113	81	-0.065176828	0.7534632	0.818953152
GOLGA8T	158	123	0.053885889	0.7539397	0.819399526
NUDT14	181	131	-0.051270754	0.7539443	0.819333011
TMEM218	679	500	-0.026331348	0.7541755	0.819512838
ZNF143	919	700	0.022442192	0.7541828	0.819449253
HMGXB3	3475	2627	0.011555354	0.7543311	0.819538842
DDX26B	508	389	0.03009379	0.7545879	0.819746436
ERMAP	1058	805	0.020873193	0.7547348	0.819834462
NBPF15	648	477	-0.026852415	0.7550922	0.820151212
PCGF6	648	495	0.026586844	0.7552531	0.82025442
AGFG1	5245	3959	0.009345426	0.7559398	0.82092856
USP27X	204	148	-0.047819845	0.7561423	0.821076899
KAZN	1462	1083	-0.017757937	0.7562995	0.821176064
MRPL2	656	501	0.02626692	0.756677	0.821514347
LOC254128	132	95	-0.059386379	0.7568591	0.821640341
CISD2	1225	931	0.019223455	0.7569423	0.82165913
ZNF250	425	326	0.032561255	0.7569443	0.821589621
CCDC53	1070	791	-0.020709065	0.7569711	0.821547151
ATXN7L2	128	100	0.059008322	0.7571546	0.82167475
DPY19L1P1	98	77	0.067228828	0.7575503	0.82203251
DNAJC7	3548	2640	-0.011315948	0.7577569	0.822185079
LOC93622	738	544	-0.024862033	0.7578376	0.822200999
TCEA2	493	362	-0.030445818	0.7578565	0.822149884
DOLPP1	906	690	0.022237443	0.7580377	0.822274787
CDCA7	559	411	-0.028557757	0.758061	0.822228471
SLC40A1	350	256	-0.03605898	0.7585721	0.822711182
CSRP2BP	705	538	0.025135047	0.7586078	0.82267829

MICALCL	50	40	0.093224037	0.758737	0.822746765
PPAPDC1A	76	54	-0.07788788	0.7587443	0.822683072
WDR5B	598	440	-0.027489829	0.759045	0.822937488
ACOX2	470	360	0.030488282	0.7606411	0.824596159
GPX3	54	38	-0.091807857	0.7608071	0.824704257
CARD6	975	742	0.0211691	0.7610023	0.824844056
GADD45B	1069	813	0.020217536	0.7610336	0.824806295
PPM1A	3652	2718	-0.010989177	0.7612065	0.824921845
ARL17A	235	171	-0.0435123	0.7614978	0.825165755
DNA2	412	316	0.032432353	0.7614993	0.825095536
WASH1	193	140	-0.048021889	0.7616403	0.825176566
ATL2	1501	1139	0.016995902	0.7620575	0.825556803
NUP153	5319	4014	0.009037711	0.7621339	0.82556768
TMEM194B	170	132	0.050155315	0.7623248	0.825702752
UFD1L	3518	2618	-0.011138254	0.7624177	0.825731517
CHEK2	420	308	-0.032306845	0.7627635	0.826034173
ELOVL1	4923	3668	-0.009391969	0.7629245	0.826136689
PEX26	1790	1328	-0.015552309	0.7635206	0.826710356
SLC25A45	89	70	0.068701718	0.7636396	0.826767302
RRP7A	1301	988	0.018114117	0.7638025	0.826871839
TMEM198	76	60	0.074115214	0.764136	0.827160972
LOC90246	80	57	-0.073885949	0.7648441	0.827855499
POM121	3196	2416	0.011505178	0.7650912	0.828050984
UHRF1BP1	630	481	0.025837197	0.7652032	0.828100283
TECPR1	1728	1310	0.015615726	0.7653848	0.828224829
EMC2	1988	1506	0.014556145	0.7654677	0.828242577
CABIN1	3895	2900	-0.010418296	0.7660309	0.828779957
SPIN2B	157	122	0.05126872	0.7663558	0.829059458
BCYRN1	106	83	0.062271109	0.7663741	0.829007219
PTP4A1	16461	12388	0.005043438	0.7664898	0.829060362
ERO1LB	405	297	-0.032306845	0.7669135	0.829446658
IFITM3	9541	7188	0.006602056	0.7672149	0.829700527

NCRNA00185	69	49	-0.078662481	0.7676004	0.830045455
EBF3	163	118	-0.050932973	0.7676067	0.829980198
AHCYL2	1642	1245	0.015843747	0.767875	0.830198234
TMEM160	409	300	-0.031986211	0.7680437	0.830308463
INPP5B	1088	827	0.01943281	0.7680965	0.830293476
SCAF4	2000	1485	-0.014384937	0.7685982	0.830763725
C2CD3	1237	916	-0.018273865	0.7689621	0.831084982
ANXA2R	58	41	-0.085276859	0.7692295	0.831301806
FEM1B	1483	1125	0.016558535	0.7693754	0.83138735
MCPH1	1153	876	0.018762394	0.7694915	0.831440726
ZNF230	182	141	0.046908844	0.769903	0.831813117
CRMP1	1410	1045	-0.017040089	0.7699115	0.831750123
TMEM159	1359	1007	-0.017329641	0.7702737	0.832069301
ZNF665	110	86	0.060057173	0.7704379	0.832174454
GPR68	84	60	-0.070274695	0.7705053	0.832175077
MED26	481	368	0.028821004	0.7708633	0.832489549
TRIM47	127	99	0.055824065	0.7708775	0.832432692
IARS2	9595	7228	0.006465832	0.771262	0.832775772
FAM73B	1120	851	0.018884436	0.7712802	0.832723206
GPN2	834	635	0.02186134	0.7713981	0.832778358
FANCF	666	491	-0.024647021	0.7715307	0.832849283
TSPAN10	309	226	-0.036131934	0.7722951	0.833602194
SAMD9	2493	1886	0.012598925	0.7725494	0.83380443
ZNF445	2364	1757	-0.012963712	0.7730917	0.834317395
LINC00847	282	217	0.037152012	0.7734388	0.834619629
CNTN3	1307	992	0.017305016	0.773584	0.834704042
GFOD2	1698	1260	-0.015260593	0.7736949	0.834751395
KIAA2026	2525	1910	0.012441382	0.7739242	0.83492644
TMEM217	73	52	-0.074232709	0.7740639	0.835004844
IK	4575	3409	-0.009271012	0.7740771	0.83494673
MGAT1	9400	7081	0.006444491	0.7742626	0.83507448
LINC00338	114	89	0.057995549	0.7742957	0.835037959

FLJ45340	3736	2782	-0.010219903	0.7749598	0.835681743
PANX1	3309	2463	-0.010826532	0.7756445	0.836347698
TTC21A	103	74	-0.06189503	0.775705	0.836340499
TMEM99	344	264	0.033281496	0.7759203	0.836500207
PARPBP	348	255	-0.033437927	0.7761583	0.836684409
HS3ST3B1	156	113	-0.050071125	0.7762245	0.836683321
VPS26B	3891	2898	-0.009931254	0.7767646	0.83719309
EPB41L4B	101	79	0.060721397	0.7773799	0.83778375
HNMT	352	258	-0.033052231	0.7774231	0.837757755
HSPE1	585	431	-0.025596624	0.7774976	0.837765605
NDUFB2	1121	830	-0.018450905	0.7776971	0.837908076
TTC27	1363	1034	0.016602755	0.7780722	0.83823973
SUPT5H	7787	5868	0.006953376	0.7781946	0.838299048
KCNAB1	323	248	0.033958087	0.7783616	0.838406482
RNF122	156	121	0.04861315	0.7789207	0.838936136
INPP5K	849	646	0.020921743	0.7790919	0.839047947
PDHA1	3090	2335	0.010967844	0.7794339	0.839343665
MILR1	49	39	0.085844506	0.7797641	0.83962667
SLC6A9	360	264	-0.032306845	0.7798901	0.839689805
AMN1	223	172	0.040516987	0.7799771	0.839710896
IFT20	488	373	0.027446614	0.7800134	0.839641021
ZNF830	488	373	0.027446614	0.7800134	0.839641021
ZFP14	391	287	-0.030965739	0.7800752	0.839598722
COQ7	640	488	0.023961374	0.7801903	0.839650036
SIGLEC15	92	66	-0.064015705	0.7807048	0.8400949
SMG1P1	92	66	-0.064015705	0.7807048	0.8400949
ETFDH	1228	932	0.017243431	0.7811164	0.840428926
SLC30A5	5294	3993	0.008267009	0.7824098	0.841747762
POLR2D	1813	1373	0.014104832	0.7825314	0.841805905
ACAD10	1134	861	0.017816634	0.7826086	0.841816258
BTD	742	565	0.021983812	0.7828599	0.842013873
SP3	4753	3586	0.008689218	0.7832611	0.842372604

USP31	791	602	0.021237924	0.783425	0.842476093
THAP7	339	260	0.032378482	0.7834286	0.842407283
SAP18	5612	4232	0.00797675	0.7838102	0.8427448
TMEM230	6688	5041	0.007287239	0.7844021	0.843308443
USP34	13672	10217	-0.005100528	0.784684	0.843538694
RINT1	1999	1513	0.013285647	0.7848812	0.84367791
ETV6	1105	839	0.017848478	0.784962	0.84369193
TRAM1L1	96	69	-0.061285912	0.785322	0.844005972
HMCN1	1170	888	0.017275184	0.7858343	0.844483749
SIAE	1571	1166	-0.01496326	0.7862313	0.844837446
ARHGEF7	3601	2719	0.009830684	0.7864446	0.844993757
EPHA5	55	39	-0.080805363	0.7864494	0.844926021
LINC00673	214	165	0.04000736	0.7870158	0.845461619
RDH13	239	184	0.03784728	0.7871477	0.84553035
WDR45	1229	911	-0.016809825	0.7876272	0.845972513
MPLKIP	688	524	0.022310379	0.7876731	0.845948841
PCDHGB2	218	168	0.03928523	0.7888953	0.847188355
MIR210HG	113	88	0.054404788	0.7889635	0.847188581
WWC3	2326	1759	0.012056518	0.7893556	0.847536525
REP15	100	72	-0.058779057	0.7896606	0.847790995
RPE	2191	1629	-0.012450749	0.7897602	0.847824775
MCEE	499	381	0.025903314	0.7898697	0.847869297
POFUT1	7049	5261	-0.006929424	0.7899112	0.847840735
C16orf95	70	50	-0.070274695	0.7900103	0.847874035
PBRM1	7621	5689	-0.006653103	0.7902451	0.848053004
C19orf52	661	488	-0.022616992	0.7905872	0.848346971
SMG7	5141	3877	0.008043855	0.7911614	0.848890028
ROR1	276	212	0.03454813	0.7912249	0.848884991
GMNN	957	727	0.018588571	0.791401	0.849000844
AUH	507	387	0.025499951	0.7914669	0.848998406
GDNF	367	281	0.029942199	0.7915105	0.848972021
CDK2AP2	755	558	-0.021059391	0.7915265	0.848916059

TRPC1	1336	991	-0.015810914	0.7916023	0.848924233
HARBI1	180	131	-0.043277963	0.7916159	0.848865796
NUP160	2638	1963	-0.01123226	0.7917923	0.848981815
YEATS2	4372	3259	-0.008711917	0.7920047	0.849136439
C1orf192	117	91	0.052582052	0.7924388	0.849455607
LOC100272228	117	91	0.052582052	0.7924388	0.849455607
PNPLA4	117	91	0.052582052	0.7924388	0.849455607
C8orf33	1821	1353	-0.013416951	0.7934086	0.850348829
ZZEF1	5019	3785	0.008045486	0.7935568	0.850434535
HTR7	104	75	-0.056468896	0.7937478	0.850529386
ZNF391	104	75	-0.056468896	0.7937478	0.850529386
PYCRL	642	489	0.0224133	0.7938006	0.850476256
C7orf60	1427	1059	-0.015130614	0.7938131	0.850416497
SFT2D2	445	340	0.026881542	0.7939415	0.850480892
STK17A	7694	5796	0.006475897	0.794295	0.85078638
ZBED4	2037	1541	0.012573013	0.794298	0.850716431
PTRH2	646	492	0.022276282	0.7944145	0.850768021
ACAT1	2903	2193	0.010513372	0.7947238	0.851026164
CMC2	486	358	-0.025844595	0.7948274	0.851063946
METTL23	740	563	0.020761783	0.7949654	0.851138468
R3HDM2	2730	2032	-0.010848417	0.7953358	0.851461908
FAM212A	74	53	-0.066380779	0.7957869	0.851871626
ANXA6	30205	22601	-0.003248648	0.7958037	0.851816355
TRAF1	142	110	0.046764726	0.7967414	0.852746716
UBE2T	711	541	0.020931166	0.7973247	0.853297729
PEX6	901	667	-0.018688213	0.79768	0.853567953
TMEM136	901	667	-0.018688213	0.79768	0.853567953
SHPK	580	428	-0.023289972	0.7978599	0.853650438
ARL8A	1487	1104	-0.014512344	0.798008	0.853735618
ZNF519	169	123	-0.043212799	0.7982367	0.853906916
NUP43	2056	1555	0.012226448	0.7989462	0.854592523
GPR137B	968	717	-0.017881797	0.7992783	0.854874302

ANGEL1	631	466	-0.022157919	0.7993364	0.854863016
AR	3127	2329	-0.009916448	0.7996489	0.855123823
L3HYPDH	1346	1020	0.015042874	0.7997373	0.85514496
FBXO41	473	361	0.025310785	0.7998944	0.85523949
ARL4A	725	536	-0.020595863	0.8000031	0.855282235
ZNF876P	63	45	-0.070274695	0.8005528	0.855796512
ZNF440	1149	852	-0.016301331	0.8005616	0.855732512
LRRC56	91	71	0.057104611	0.8008632	0.855981404
ZNF211	686	507	-0.021070697	0.8009672	0.856019069
AASDH	845	642	0.018774088	0.8017975	0.856832971
BIRC3	150	116	0.044314436	0.8020107	0.856987217
BANP	485	370	0.024692655	0.8022906	0.857212772
KRBOX1	177	129	-0.041226163	0.8028432	0.857729685
CHPT1	1264	958	0.015253229	0.8031405	0.857973659
EIF1AX	8184	6112	-0.00600947	0.8033566	0.858130951
SEMA4F	914	694	0.017893629	0.8035249	0.858237064
AGO1	3354	2499	-0.009379656	0.8036335	0.858279523
ARRB2	283	217	0.032045121	0.803726	0.85830468
STAG1	5462	4117	0.007316266	0.8039599	0.858480862
SCN2A	204	149	-0.03810469	0.8042306	0.858696332
PEX11B	1483	1123	0.013991462	0.8044221	0.858827123
CLUHP3	78	61	0.06048725	0.8045233	0.858861526
SLC29A2	52	37	-0.075834221	0.804569	0.858836671
VPS45	2099	1587	0.011752093	0.8046329	0.858831361
ZNF213	581	429	-0.022408384	0.8051612	0.859321612
C3orf18	542	413	0.023001062	0.8053079	0.859404528
IPP	1398	1038	-0.014405785	0.8054259	0.859456745
PARP6	820	607	-0.018775262	0.8059209	0.859911301
TYW5	779	592	0.019125979	0.8060296	0.859953625
TOB2P1	82	59	-0.059756824	0.806046	0.859897483
HIBCH	1217	903	-0.015379143	0.806209	0.859997643
SLC23A2	2491	1882	0.010693738	0.8063372	0.860060787

DNAJC2	1765	1312	-0.012748332	0.80645	0.8601074
EFNA5	324	248	0.029498439	0.8067035	0.860304188
INPP5F	2504	1864	-0.010680571	0.8067796	0.860311671
TAB2	4477	3339	-0.007964093	0.8072452	0.860734483
PFKFB4	644	490	0.020873193	0.8074384	0.860866732
GLIS2	848	644	0.018148555	0.8079576	0.861346561
FLNC	101723	76374	0.001659707	0.8080047	0.861323004
CNIH	5291	3988	0.007277124	0.8080055	0.861250188
SNX19	8588	6466	0.005703448	0.8083301	0.861522442
LOC728190	332	244	-0.029149962	0.8085614	0.861695224
PPP2R2C	52	41	0.072264418	0.8088421	0.86188374
PTGFRN	52	41	0.072264418	0.8088421	0.86188374
ARFRP1	1490	1128	0.013606869	0.809266	0.86222478
DDX19A	2497	1859	-0.010516919	0.8099388	0.862867838
CPSF7	3419	2580	0.008948775	0.8101172	0.862984053
ARGLU1	3536	2668	0.008792524	0.8102693	0.863072227
TXNRD2	872	662	0.017655214	0.8105189	0.86326429
STAG3L4	488	372	0.023573605	0.8105409	0.863213969
ZC3H7B	15821	11899	0.004151674	0.810623	0.863227547
SNCG	86	62	-0.056916313	0.8106304	0.863161658
RFX1	758	561	-0.019044946	0.8106384	0.863096377
TMEM5	819	622	0.01820326	0.8106468	0.863031574
HIST1H1C	307	235	0.029574233	0.8112682	0.863619214
CLP1	676	514	0.019897245	0.8118825	0.864199319
SNX30	2663	2011	0.010012785	0.8124178	0.86469524
ZBTB14	457	337	-0.024293442	0.8125808	0.864794775
FAM22A	124	96	0.045918322	0.8132014	0.865381339
MANSC1	315	241	0.02883345	0.8135936	0.865724725
ASB13	255	187	-0.032306845	0.8140518	0.866138273
C6orf211	2609	1943	-0.010058911	0.8140905	0.866105543
SUPT6H	12359	9239	-0.004601271	0.8147057	0.866685984
RPL13AP20	90	65	-0.054333152	0.814906	0.866825019

COX7A2L	3506	2645	0.008593858	0.8152579	0.867125262
C8orf76	56	44	0.067228828	0.8154861	0.867219852
CDNF	56	44	0.067228828	0.8154861	0.867219852
SCG2	56	44	0.067228828	0.8154861	0.867219852
DCLRE1C	755	559	-0.01847623	0.8165303	0.868182047
FAM161A	205	150	-0.035509277	0.8168007	0.868395474
ADAMTSL4	1294	961	-0.014077149	0.8168502	0.868373913
LOC100506451	178	137	0.037450784	0.8176512	0.869151265
DCAF6	6382	4807	0.006280327	0.8178695	0.869309127
SHMT1	298	219	-0.029229329	0.8179651	0.869336544
CEP76	487	371	0.022649546	0.8180465	0.869348948
SIAH2	891	676	0.016749947	0.8181757	0.869412061
TMEM92	60	43	-0.065473709	0.8181762	0.869338429
DLG5	6896	5150	-0.00603521	0.8184431	0.869547788
LOC728407	94	68	-0.051973879	0.8189059	0.869965332
LOC339803	536	408	0.021488283	0.8189201	0.869906128
LRP1B	111	86	0.04700102	0.8189599	0.869874213
NSUN6	335	256	0.027134846	0.8190471	0.869892734
CD97	3432	2589	0.008497544	0.8191994	0.869980268
SRR	271	199	-0.030372289	0.8196259	0.870358995
CDKN2B	499	380	0.022111735	0.8201714	0.870863961
DUS1L	1488	1106	-0.012871009	0.820329	0.870957121
ZNF418	186	136	-0.036543838	0.8203821	0.870939227
CLPX	2239	1667	-0.010448292	0.8210013	0.871522284
CCDC58	421	321	0.023905196	0.82135	0.871818107
ZNF131	1366	1015	-0.013325624	0.8217613	0.872180425
PEX1	1161	862	-0.014456066	0.8217745	0.872120083
LOC100506124	79	57	-0.055738602	0.8220197	0.872305946
PIK3C2B	277	212	0.02933042	0.8220231	0.872235267
SLC6A6	1641	1241	0.012080008	0.8221153	0.872258853
ASF1B	509	376	-0.021780863	0.8224134	0.872500786
MAMSTR	77	60	0.055256187	0.8224182	0.87243155

C14orf119	2886	2178	0.009084786	0.8226698	0.872624104
FUT11	1110	824	-0.014691302	0.8228857	0.872778803
ALDH6A1	474	361	0.02226391	0.8234674	0.873321391
DHRS3	517	382	-0.021439511	0.8237947	0.873594075
TAMM41	474	350	-0.022380005	0.8239071	0.873638886
KDM5B-AS1	64	46	-0.061285912	0.8239981	0.873661078
RECQL5	713	528	-0.018212015	0.8241191	0.873714922
SLC25A14	478	364	0.022079964	0.8241825	0.873707757
AUP1	3059	2308	0.008735249	0.8244373	0.873903585
SLC1A1	2149	1600	-0.010441446	0.824689	0.874095965
CDK19	2062	1535	-0.010653545	0.8247909	0.874129589
RAD54L2	1782	1347	0.011404646	0.824967	0.874241837
TBL2	2248	1674	-0.010190376	0.824995	0.874197098
C22orf29	1355	1007	-0.013077036	0.8257192	0.874890054
DENND1A	1020	757	-0.015051815	0.8260112	0.875124974
GTF2H1	3353	2529	0.008266686	0.8260411	0.87508221
RUFY3	3599	2714	0.00797675	0.826101	0.875071257
MTMR9LP	144	111	0.039642997	0.8261801	0.875080649
OSBPL2	2174	1642	0.010254318	0.8261857	0.875012149
CCDC117	1513	1125	-0.012334854	0.8262589	0.875015297
PIP4K2B	7524	5621	-0.005520902	0.8263694	0.875057841
RBM4B	637	484	0.018865807	0.826638	0.875267923
BIRC6	13319	10017	0.004116863	0.8274214	0.876022949
TMUB2	1458	1084	-0.012473831	0.8275086	0.876040781
EAF1	1599	1209	0.011796438	0.8284352	0.876947151
MIPOL1	1115	828	-0.014188906	0.8284533	0.876891758
PTTG1IP	17574	13212	0.003558407	0.8286434	0.877018515
STK11IP	657	499	0.018298577	0.8292073	0.877540761
TMEM26	68	49	-0.057600865	0.8292968	0.877560859
ITPR1	379	289	0.024023776	0.8295205	0.877722997
CLGN	237	174	-0.030647621	0.8296383	0.877773119
UBR2	4577	3449	0.006927969	0.8296492	0.877710059

PEX7	305	233	0.026672844	0.8301157	0.878129005
ZNF497	87	63	-0.050511441	0.8304542	0.878412442
NDUFA1	2701	2013	-0.008994336	0.8305627	0.878452639
PET100	840	637	0.016056176	0.8305905	0.878407413
DNAH17	85	66	0.050155315	0.8306643	0.878410966
URGCP	1759	1309	-0.011138254	0.8307342	0.87841031
PRMT10	887	658	-0.015694389	0.8307689	0.878372431
BUD13	561	415	-0.019737303	0.830862	0.87839629
MARK4	3387	2526	-0.008001216	0.8311726	0.878650028
GOLGA5	3968	2991	0.007368016	0.8312752	0.878683875
CEMP1	106	82	0.044783682	0.8312998	0.878635359
TMEM243	432	329	0.022208403	0.8317626	0.87904986
LOC550112	350	258	-0.024831725	0.8320519	0.879281007
DUS2L	522	386	-0.020296828	0.8322177	0.879381623
TDRD7	785	582	-0.016521369	0.8324068	0.879506889
MST1	110	80	-0.044279487	0.8326628	0.879702722
LOC154761	131	101	0.039940613	0.8328564	0.879832614
CCDC127	679	503	-0.017701043	0.8330278	0.879939063
COA1	1902	1416	-0.010543849	0.8333251	0.880178434
UBXN11	185	142	0.033517791	0.8334893	0.88027725
TMPPE	247	189	0.029027324	0.8335057	0.880219943
RPS6KA1	187	137	-0.033710245	0.8335792	0.880222891
ANXA9	72	52	-0.054333152	0.8341462	0.880709607
CYTL1	72	52	-0.054333152	0.8341462	0.880709607
BDH1	91	66	-0.048248389	0.8342476	0.880704725
TLR6	284	217	0.026956245	0.8342568	0.880639769
ZBTB46	691	512	-0.017389769	0.8344904	0.880811649
MPC2	4873	3671	0.006515024	0.8346412	0.880896168
CDYL2	218	167	0.030672099	0.8346578	0.880839099
COMT	3198	2385	-0.008028541	0.8353303	0.881474077
IGDCC4	644	477	-0.01791929	0.8353554	0.881425959
NKTR	8935	6723	0.004789552	0.8354216	0.881421087

MED17	1836	1387	0.010553861	0.8354556	0.881382297
PTK7	7827	5849	-0.005117324	0.8357023	0.881567857
CEP89	1253	948	0.012724681	0.8360388	0.881848191
SLC9B2	839	636	0.015508087	0.8364015	0.882156042
UBTD1	1350	1021	0.012175591	0.837154	0.882874979
INVS	1168	868	-0.013121195	0.8374742	0.883137819
C19orf54	296	226	0.025877729	0.8375448	0.883137558
PARP4	10582	7912	-0.00434586	0.8377082	0.883235038
SPRED1	8383	6308	0.004868162	0.8379331	0.883397345
LURAP1L	468	356	0.020520843	0.8381546	0.883556164
UPP1	423	322	0.021555157	0.8383453	0.883682368
PURG	118	86	-0.041226163	0.8384677	0.883736588
ZNF879	197	151	0.031505052	0.8384775	0.883672126
MIER2	1413	1051	-0.011846665	0.8385521	0.883676007
ZNF660	76	55	-0.051415668	0.8386065	0.883658587
TIMM8A	515	381	-0.019629303	0.8387164	0.883699554
COPS4	3405	2567	0.007580629	0.8390766	0.884004298
ZNF587B	431	328	0.021160077	0.8398054	0.884697358
CAMKMT	234	172	-0.028947833	0.8399159	0.884738921
FBXL14	271	207	0.026490048	0.8408168	0.885612968
ST7L	960	727	0.01407309	0.8411686	0.885908578
GOLGA6L5	238	175	-0.02845452	0.8412728	0.885943454
ZRANB3	273	201	-0.026553318	0.8413076	0.88590515
TPRA1	1183	895	0.012661646	0.8414022	0.885929864
KIF24	238	182	0.028129009	0.8415389	0.885998938
TMEM220	176	129	-0.033052231	0.8415715	0.885958317
SLC22A4	394	291	-0.022024345	0.8417424	0.886063387
DRG1	2978	2221	-0.007982229	0.841921	0.886176426
FAM180A	149	109	-0.035832064	0.8420393	0.886226079
CCNI	7874	5885	-0.004902165	0.842046	0.886158236
GCHFR	61	44	-0.056153587	0.8421657	0.886171888
TRAPPC6A	61	44	-0.056153587	0.8421657	0.886171888

DHRS4-AS1	539	399	-0.018744395	0.8423661	0.886270504
FOPNL	3295	2484	0.00753884	0.842525	0.886362779
ZNF185	447	340	0.020412047	0.8426092	0.886376474
PUS3	496	377	0.019376535	0.8426453	0.886339644
CCDC84	492	364	-0.019567733	0.8427972	0.886424476
UTY	964	716	-0.013921427	0.8432872	0.886865022
PLAA	3911	2947	0.006861656	0.8438464	0.887378158
CRYZ	1513	1126	-0.011053028	0.8440346	0.887501162
LIN52	406	300	-0.021365095	0.8441104	0.887505871
PLEKHM3	1308	989	0.011832017	0.8441468	0.887469311
R3HDM4	2071	1543	-0.00943736	0.8441538	0.887401692
CUBN	103	75	-0.042529705	0.8442701	0.887449046
PCDHB8	126	97	0.03778505	0.8448218	0.887954061
C16orf62	3697	2786	0.006992346	0.8452656	0.888345531
WDR54	463	352	0.019715367	0.8452686	0.888273762
TRIM13	1797	1357	0.009982444	0.8459045	0.888866996
BRIX1	1830	1363	-0.009905955	0.8462145	0.889117748
NEU3	465	344	-0.01967002	0.8463303	0.889164398
NDUFB3	1619	1223	0.01047355	0.846519	0.889287668
H1FX-AS1	105	81	0.040756617	0.8470946	0.88981721
KRTAP1-5	581	441	0.017392624	0.8471279	0.889777186
STS	1490	1109	-0.010900833	0.8473106	0.889894027
NSMCE2	689	511	-0.01602856	0.8474626	0.889978657
KCNK2	906	686	0.013849658	0.8480691	0.890540429
TBC1D20	4024	3004	-0.006593361	0.848101	0.890498938
MRPS23	1882	1402	-0.009628147	0.8483844	0.890721349
NET1	2310	1722	-0.008655577	0.8489611	0.891251761
DLGAP1-AS1	109	84	0.03928523	0.8498386	0.892060114
VAX2	109	84	0.03928523	0.8498386	0.892060114
SH3TC1	88	64	-0.044279487	0.8501074	0.892229526
ASAH1	3306	2467	-0.007176877	0.8501248	0.892172603
RBM19	1926	1435	-0.009404835	0.8501525	0.892126489

LOC728730	348	257	-0.022166815	0.8502048	0.892106252
LOC100132352	270	206	0.024837062	0.8509439	0.892806561
FAM162B	138	106	0.03454813	0.8515153	0.893330822
PODXL2	88	68	0.043183354	0.8515683	0.89327351
RNF207	88	68	0.043183354	0.8515683	0.89327351
FAM211A	69	50	-0.049516135	0.8516908	0.893289182
IL6	115	84	-0.038020496	0.8526797	0.894251041
TRAF4	1565	1182	0.01021951	0.852694	0.894190802
FAM53A	50	39	0.056698161	0.8527416	0.894165402
ZNF765	572	434	0.016832027	0.8531492	0.894517449
FAM26E	1302	984	0.011152904	0.853338	0.894640138
CENPBD1	572	424	-0.01679875	0.8542783	0.895550559
ESCO2	421	320	0.019403804	0.8546836	0.895900009
FMNL1	146	107	-0.033205441	0.8548829	0.896033487
CLSTN1	13891	10393	-0.003386173	0.8548852	0.895960522
SCCPDH	2900	2186	0.007392633	0.8549887	0.89599363
MRPL27	1377	1025	-0.010752518	0.8551184	0.896054198
MCMBP	2803	2113	0.007473155	0.85587	0.896766291
ADRA1D	54	39	-0.054333152	0.8561051	0.896937199
DLG4	2086	1555	-0.008672446	0.8561111	0.896867959
KLHL20	1987	1481	-0.0088681	0.8564006	0.897095808
C19orf55	427	316	-0.01915938	0.8564383	0.897022136
ZNF578	427	316	-0.01915938	0.8564383	0.897022136
C15orf62	96	70	-0.040527352	0.8565432	0.897018893
PKIG	1995	1487	-0.008831967	0.8566939	0.897101309
MCM8	998	742	-0.012468497	0.8569963	0.897342539
RASSF5	216	159	-0.026852415	0.8570794	0.897354037
MCM3	2222	1676	0.008315464	0.8571759	0.897379615
GALK2	608	461	0.015847559	0.8574308	0.8975711
C10orf88	1085	807	-0.011902332	0.8576429	0.897717689
EIF2AK2	4433	3338	0.005852724	0.8580605	0.898079288
AGAP11	54	42	0.052582052	0.8581169	0.898062865

SMYD2	1259	937	-0.011005198	0.8581828	0.898056388
POLR2I	734	545	-0.014371701	0.8586699	0.898490603
CA5B	259	191	-0.024227328	0.8587164	0.898463817
RDH11	4143	3120	0.006022341	0.8587935	0.898468973
SMAGP	758	574	0.01400502	0.8593452	0.898970663
C11orf80	351	267	0.020520843	0.8595953	0.899118993
NFKBIE	351	267	0.020520843	0.8595953	0.899118993
CYP4V2	451	334	-0.018127199	0.8603413	0.899785991
PHF11	770	583	0.013789569	0.8604112	0.899783496
SLC35F3	353	261	-0.020466245	0.8605544	0.899857688
POLR1B	1175	888	0.011122957	0.8609687	0.900215302
FBXO31	1179	891	0.01108575	0.8611966	0.900378061
TTC40	58	42	-0.050511441	0.8612152	0.900321902
ZNF398	1106	836	0.011375594	0.8620321	0.901100323
ZNF749	232	177	0.024776687	0.8620509	0.901044299
FLJ10038	361	267	-0.020006963	0.8621208	0.901041703
AMFR	5934	4435	-0.004918691	0.8622	0.901048925
EHBP1	4391	3280	-0.005701588	0.8626113	0.901403109
PTRH1	236	180	0.024362179	0.8631928	0.901935123
SEMA6A	1763	1314	-0.008915067	0.8639454	0.902645703
LOC145783	135	99	-0.032306845	0.8641179	0.902750297
MRPS31	794	590	-0.013271921	0.8641743	0.902733497
EMP2	1375	1024	-0.010063772	0.8643873	0.902880193
ASPHD2	137	105	0.031365566	0.8656144	0.904048279
NOV	137	105	0.031365566	0.8656144	0.904048279
MASP2	139	102	-0.031363599	0.8661042	0.904446071
PCDHGA3	207	158	0.025445922	0.8661208	0.904387552
NARFL	613	455	-0.014868397	0.8663165	0.904516031
TWIST1	389	288	-0.018549212	0.8672197	0.905383205
TSTD2	635	481	0.014432434	0.8672472	0.905335975
CDC34	2454	1831	-0.007351326	0.8675663	0.905593248
ACACA	4898	3660	-0.005196994	0.8676777	0.905633618

NICN1	574	435	0.015116796	0.867779	0.905663403
TGFBR1	3854	2902	0.00584308	0.8677796	0.905588127
MBOAT1	213	157	-0.02493674	0.8680508	0.905757374
TMEM251	213	157	-0.02493674	0.8680508	0.905757374
TSEN2	456	346	0.016890345	0.8682793	0.905881956
MBLAC1	87	67	0.038297826	0.8690517	0.906611905
TRAF3IP2	779	579	-0.012907848	0.8691001	0.90658641
UFC1	2698	2033	0.006871999	0.8698512	0.907293907
DONSON	1206	898	-0.010290425	0.8700871	0.907463999
EFNB2	1123	836	-0.010630949	0.870493	0.907811283
ZNF585A	362	275	0.018594053	0.8707403	0.907993123
FHL1	4629	3484	0.005196193	0.8711201	0.908313205
MVD	914	691	0.011643677	0.8715302	0.908664735
P2RX4	321	244	0.019459982	0.8725766	0.909679533
RMDN1	3377	2522	-0.006021772	0.8726667	0.909697308
POPDC3	768	581	0.012583984	0.8726963	0.909652057
ZNF431	425	315	-0.016958881	0.8730249	0.909918375
DHCR7	1789	1334	-0.008242589	0.8731874	0.910011634
PPIG	3580	2674	-0.00580799	0.8735374	0.910300239
USP37	946	715	0.01125519	0.8736517	0.910343192
TMEM242	1088	810	-0.010532612	0.8736768	0.910293186
CDCP1	954	721	0.011162125	0.8741654	0.910726095
SLC16A13	95	73	0.035121082	0.8745361	0.911036075
OTUD5	2918	2198	0.006363635	0.8746318	0.911059503
DNASE1	333	253	0.018767339	0.8748322	0.911192072
MAN1A1	3505	2618	-0.005797215	0.8750882	0.911382503
ZNF117	1409	1050	-0.009130152	0.8753417	0.911570335
PPA1	5403	4065	0.004646802	0.8754578	0.911614992
PRDM10	1029	766	-0.010674553	0.8754823	0.911564263
FUNDC2	1483	1119	0.00884357	0.8757346	0.911750809
FAM136A	1971	1486	0.007658471	0.8759615	0.911910725
SCN4B	241	178	-0.022003774	0.8760236	0.911899206

LEPR	1382	1043	0.009133674	0.8760966	0.911898998
CCDC152	128	98	0.029861976	0.8762724	0.912005694
FTSJ2	1180	891	0.009862609	0.8763546	0.912015134
AGK	1503	1134	0.008727763	0.8765337	0.912125248
RPN1	17479	13085	-0.002556592	0.8768839	0.912413496
MAGEE1	292	216	-0.019784925	0.8772389	0.912706549
CDCA2	1418	1070	0.008905396	0.8776226	0.913029525
RHBDD2	1930	1455	0.007570437	0.8786499	0.914021947
SOGA2	4978	3721	-0.004723708	0.8786503	0.913946015
PLEKHH3	540	409	0.014293568	0.8786671	0.913887195
DACT3	745	554	-0.012202318	0.8788915	0.914044263
C1QTNF2	171	126	-0.02542046	0.8793986	0.914495235
HLA-E	12317	9218	-0.002973103	0.8798052	0.914841655
TNFRSF11B	3021	2275	0.005992493	0.8798448	0.914806525
LRRC2	105	77	-0.032306845	0.8800337	0.914926545
TADA2A	477	354	-0.015087774	0.8802275	0.915051616
NR3C2	365	277	0.017141644	0.8802987	0.915049316
TCF7L1	706	534	0.01232369	0.8804127	0.915091434
GNRHR2	78	57	-0.037360073	0.8805302	0.915098934
LAMA3	78	57	-0.037360073	0.8805302	0.915098934
MGC45800	175	129	-0.024831725	0.880799	0.915263794
ZMYM1	1323	986	-0.009001378	0.8808646	0.915255562
MGC12916	78	60	0.036640508	0.8813131	0.915645249
RAD52	426	316	-0.01577674	0.8816463	0.915915027
ITPKB	434	329	0.015544673	0.8816567	0.915849426
EPS8L2	1296	978	0.008992784	0.8818241	0.915946892
ZNF592	2081	1553	-0.007067004	0.8826399	0.916717825
BRAP	1601	1194	-0.00801834	0.8832193	0.917243076
SAP30	379	281	-0.016475586	0.8834224	0.917377477
DCK	1978	1476	-0.007197573	0.883464	0.917344216
HOMER2	82	60	-0.035509277	0.8835144	0.917320071
SLC2A12	647	481	-0.012576686	0.883648	0.917382354

ESD	5540	4167	0.004275256	0.883917	0.917585089
CHFR	1469	1108	0.008275617	0.8842144	0.917817322
VASH1	1037	783	0.00982045	0.8845253	0.918063561
MPP4	230	170	-0.020946983	0.8846188	0.918084113
ZNF677	148	113	0.025877729	0.8847256	0.918118396
SEPT7P2	527	399	0.013737916	0.8847632	0.918080961
ZSCAN22	283	215	0.018686739	0.8850338	0.918285215
CD276	5186	3901	0.004373924	0.8850851	0.91826201
TMA16	1324	987	-0.008629001	0.8857083	0.918832041
MXD1	924	688	-0.010332155	0.8857103	0.918757543
ZNF10	539	408	0.013436011	0.886017	0.918999146
RNMTL1	529	393	-0.013586278	0.8863476	0.919265489
LRRC23	195	144	-0.022253181	0.8871435	0.920014299
LENG1	295	224	0.01793591	0.8873369	0.920138283
MFSD9	242	179	-0.019895328	0.8875488	0.920281359
SERTAD3	895	676	0.010287696	0.8875886	0.920246039
BHLHB9	559	423	0.012961512	0.8880167	0.920613293
UPF3A	2777	2091	0.005818017	0.8881004	0.920623414
ACBD7	61	47	0.039003646	0.8881789	0.920589893
ZNF540	61	47	0.039003646	0.8881789	0.920589893
ATP5L	5759	4331	0.004033982	0.8882991	0.92059951
TMEM187	297	220	-0.017807276	0.888452	0.920681384
UPF2	4174	3120	-0.00473244	0.8886085	0.920766988
LOC100505549	90	66	-0.032306845	0.8888727	0.92092584
PHF7	90	66	-0.032306845	0.8888727	0.92092584
CELSR2	826	624	0.010556379	0.8891728	0.921121776
PCDHGA9	90	69	0.031823492	0.8893093	0.921186524
IL27RA	203	150	-0.021365095	0.8894152	0.921219664
PSME3	7854	5904	0.003417255	0.8894992	0.921230075
ECHDC2	254	193	0.018924482	0.8896628	0.921322856
EBLN2	125	92	-0.027070197	0.8901556	0.921718224
TXNDC5	125	92	-0.027070197	0.8901556	0.921718224

MGST1	7681	5774	0.00342909	0.8903392	0.921793367
CORO6	164	125	0.023384412	0.8903566	0.921734853
KATNA1	846	639	0.0103104	0.8904491	0.921753951
PMF1	207	153	-0.020946983	0.8905015	0.921731593
HOMEZ	502	373	-0.013359602	0.8910984	0.922234498
RAD54B	502	373	-0.013359602	0.8910984	0.922234498
KBTBD3	258	191	-0.018646296	0.8911338	0.922156139
KLHL12	2258	1686	-0.006288818	0.891142	0.922088008
TCTEX1D4	94	69	-0.030912263	0.891291	0.922165577
COL9A2	65	50	0.036640508	0.8915873	0.922357235
MIR137HG	65	50	0.036640508	0.8915873	0.922357235
F8	213	162	0.020292514	0.8916176	0.922273682
ZFP28	581	432	-0.012354719	0.8916325	0.922212511
LINC00654	168	128	0.022834709	0.8916386	0.922142169
CUL4A	8521	6376	-0.00321899	0.8916907	0.92211953
TMEM182	129	95	-0.026219515	0.8918918	0.922250928
WDR12	1431	1079	0.007823325	0.8919266	0.922210376
FITM2	262	194	-0.018358028	0.8919785	0.922187472
TUT1	759	565	-0.010696886	0.8927225	0.922880003
VPS72	1385	1033	-0.00789359	0.8930082	0.923098732
PARP2	1108	836	0.008769098	0.8933802	0.923406651
CCDC163P	98	72	-0.029632711	0.8935593	0.923515182
FLVCR2	135	103	0.024837062	0.8942921	0.924195801
C22orf46	878	654	-0.009778172	0.8945009	0.924334964
ULBP3	223	165	-0.019425554	0.8945512	0.924310154
MRPL45	1382	1042	0.007749794	0.8947758	0.924465563
CHD8	7148	5348	-0.003388037	0.8955719	0.925211315
NUDT4	71	52	-0.03415527	0.8956434	0.925170082
RELL2	71	52	-0.03415527	0.8956434	0.925170082
ING2	566	428	0.011960875	0.8959876	0.925410496
CDIPT	3261	2454	0.00497294	0.8963197	0.925676782
POLR3C	2494	1863	-0.005681659	0.8965972	0.925886551

CCRN4L	663	501	0.010953865	0.8969175	0.926140565
TARBP2	760	574	0.01020345	0.8972066	0.92636224
DNHD1	349	259	-0.015122807	0.8972104	0.92628946
RPA2	1321	996	0.007739313	0.8972496	0.926253157
HOXB4	1029	767	-0.008792368	0.8972698	0.926197198
FAM83H	235	174	-0.018421319	0.8973138	0.926165814
MAPK13	188	143	0.020434617	0.8974278	0.926206754
UTP6	2550	1905	-0.005554118	0.8977809	0.926494437
PDE7A	1212	914	0.008028503	0.8978939	0.926534217
BAALC	1917	1444	0.006372537	0.8981004	0.926670541
BTBD8	75	55	-0.032306845	0.8985003	0.927006364
MNS1	147	112	0.022834709	0.8985981	0.927030466
VAMP2	2275	1713	0.005810738	0.8987847	0.927146171
ELF4	1839	1373	-0.006437723	0.8993908	0.927694576
MAPK8IP1	1077	803	-0.008394225	0.8996405	0.927875333
ZNF248	1030	777	0.008494298	0.9003897	0.928571183
NDUFA2	1276	962	0.007632602	0.9003925	0.928497117
MAPKAP1	5870	4413	0.003551376	0.900659	0.928695087
ATG16L2	200	148	-0.019250692	0.9009985	0.928968277
ADAMTSL5	606	451	-0.011038228	0.9010452	0.928939508
MYO1F	114	87	0.025205613	0.9013757	0.929203335
C6orf164	52	38	-0.037360073	0.9023311	0.930111243
CNPY4	557	421	0.011295037	0.9025391	0.930248601
STRN3	3367	2517	-0.004606373	0.9025434	0.93017608
C8orf46	52	40	0.036640508	0.9029728	0.930464675
LINC00854	52	40	0.036640508	0.9029728	0.930464675
MIPEPP3	52	40	0.036640508	0.9029728	0.930464675
HILPDA	326	242	-0.014712785	0.9033108	0.930658971
SLC25A44	1546	1154	-0.006744964	0.9033362	0.930608123
STARD9	931	694	-0.008693373	0.9033518	0.930547226
ZNF471	759	573	0.009587385	0.9034552	0.930576748
ECSIT	573	433	0.010984018	0.9038698	0.930926811

MAP2K1	2752	2071	0.004999215	0.9042114	0.931201641
PCDHGB5	2961	2228	0.004816874	0.9042672	0.931182137
AP5Z1	1537	1158	0.00667022	0.9044587	0.931302379
NDUFAF4	650	484	-0.010280539	0.9045209	0.931289396
REXO1	1729	1291	-0.006296737	0.9045548	0.931247353
PROS1	783	591	0.009297955	0.9048987	0.931524395
APTX	1753	1309	-0.006208767	0.9052327	0.93179113
C8orf48	171	130	0.01966743	0.9058194	0.932318051
IL31RA	56	41	-0.034650786	0.9059331	0.932357995
HDAC4	803	606	0.009069938	0.9060519	0.932403241
DGCR6	126	93	-0.022968981	0.9062775	0.932558388
MARCH1	283	210	-0.015260593	0.9065524	0.93276419
ZSWIM1	421	313	-0.012505444	0.906556	0.932690782
LUST	435	329	0.012224315	0.9067506	0.932814008
C11orf82	508	378	-0.011290131	0.9073063	0.933308593
RRN3P3	130	99	0.022140939	0.9075077	0.933438644
MND1	130	96	-0.022253181	0.9077501	0.9336109
C11orf71	179	136	0.018799196	0.9078998	0.933687715
NEK1	2007	1511	0.005615176	0.9080924	0.933808666
PTPN9	3004	2260	0.004590092	0.9081005	0.933739936
GORASP2	6584	4927	-0.003102732	0.9081523	0.933716079
FLJ42627	1865	1393	-0.005828241	0.9082233	0.933712052
TGIF1	1226	924	0.007157909	0.9084064	0.933823148
RBFA	738	557	0.009208643	0.9085439	0.933887424
RPS15AP10	93	71	0.02574044	0.9089883	0.934267173
SUPT4H1	1047	781	-0.007714857	0.9090029	0.934205074
NCK1	1742	1301	-0.00597153	0.9091191	0.934247408
C9orf91	459	347	0.011593641	0.9091539	0.934206091
TMEM121	134	99	-0.021580439	0.9091563	0.934131489
CRLS1	1560	1175	0.006266859	0.9095509	0.934459805
ATP7A	3883	2904	-0.003978122	0.909574	0.934406495
HACL1	564	426	0.0103104	0.9104582	0.935237668

TNFSF12	544	405	-0.010532612	0.9104886	0.935191793
PCDHB2	244	181	-0.015739319	0.9104984	0.935124752
DCAF8	5477	4098	-0.003313764	0.9105252	0.935075129
SMG5	4481	3352	-0.003646446	0.9109479	0.935432063
CNPPD1	1801	1356	0.005711129	0.9114337	0.935853769
FAM118A	592	447	0.009829787	0.9125375	0.936909948
STOML1	697	526	0.009056275	0.912577	0.936873226
FOXJ3	3195	2403	0.004172849	0.9138221	0.938074194
C12orf45	272	206	0.014189818	0.9143474	0.93853604
ANXA3	68	52	0.028129009	0.9148965	0.939022354
KARS	6954	5205	-0.002792772	0.9150046	0.939055862
GSPT2	1211	904	-0.006652056	0.9155758	0.93956471
PTPN21	3355	2523	0.003979572	0.9157762	0.939692949
TXNL4B	517	385	-0.010153703	0.9158513	0.939692549
PHGDH	2427	1826	0.004664789	0.9160209	0.939789152
KLF11	284	215	0.013597862	0.9161314	0.939825103
INSIG2	842	628	-0.007903542	0.916375	0.939997541
COG7	1168	880	0.006687286	0.916453	0.940000183
TRIB1	1704	1273	-0.005540785	0.9165647	0.940037361
EXOSC8	660	498	0.00883185	0.9170239	0.940430815
HEMK1	1116	833	-0.006796495	0.9171914	0.940525154
PLEKHM1P	292	221	0.013230132	0.9172593	0.940517401
RAD51C	783	584	-0.007891807	0.9194669	0.942703333
MSL3P1	119	88	-0.020234013	0.9196761	0.942840209
NUDT2	791	590	-0.007810608	0.9198868	0.942978599
PNMA2	121	92	0.019850851	0.9199828	0.942999383
ERI2	680	507	-0.008396867	0.9201522	0.943095414
FLJ23867	2242	1676	-0.004611997	0.9203121	0.943181668
CWF19L1	1131	852	0.006478538	0.9203429	0.943135659
DNAH1	401	303	0.010867689	0.9203743	0.94309023
MLL2	8987	6729	-0.002295349	0.9205581	0.943201013
ARRDC2	243	184	0.013901584	0.9206687	0.943236743

SEZ6L2	1324	997	0.005914419	0.9213229	0.943829297
AGPHD1	80	61	0.023961374	0.9214035	0.943834227
CHCHD4	494	368	-0.009653144	0.9217729	0.944134944
ABCA11P	417	315	0.010456577	0.9218736	0.944160513
CGRRF1	403	300	-0.010665206	0.9219499	0.944160966
MRPS27	2867	2156	0.003967405	0.922359	0.94450226
ENKD1	186	141	0.015544673	0.922363	0.944428724
GLTP	3035	2270	-0.003852087	0.9225428	0.944535188
ZNF800	1606	1200	-0.005285355	0.9227102	0.944628909
ZNF700	905	682	0.007006079	0.9229273	0.944773538
CCDC96	131	97	-0.018358028	0.9235001	0.945282211
SUN2	3814	2867	0.003389193	0.9235041	0.945208606
METTL2B	1247	939	0.00588773	0.9239817	0.945619747
ADSS	4183	3144	0.003215351	0.9239987	0.945559423
C4orf27	1102	830	0.006211149	0.9246064	0.946103614
PLA2G16	356	269	0.010881063	0.9248688	0.946294286
RER1	8806	6614	0.002188333	0.924957	0.946306794
GAS2L3	649	484	-0.008059299	0.92511	0.94638563
NRDE2	657	490	-0.00795949	0.9255803	0.946789021
GPT2	1372	1025	-0.00550444	0.9255957	0.946726977
TSR2	1150	866	0.005957201	0.9261297	0.947195415
N4BP2	715	539	0.007494163	0.9267044	0.947705293
STMN1	12714	9523	-0.001777824	0.9267873	0.947712255
LINC00672	143	106	-0.01679875	0.9268325	0.94768067
LOC100289019	92	68	-0.020946983	0.9268731	0.947644433
KLC2	2452	1844	0.004031808	0.9270183	0.947715042
PHTF1	1608	1210	0.004891773	0.9282792	0.948926211
LYRM2	2327	1750	0.004035843	0.9288269	0.94940817
AGTR1	55	42	0.026109841	0.9289404	0.949446333
BCR	3467	2606	0.003301379	0.9289429	0.949370957
CWC15	2094	1575	0.004242518	0.9290237	0.949375614
USP32	4518	3395	0.002879435	0.9292554	0.949534481

ALG11	513	387	0.008526872	0.9293405	0.94954354
LOC389906	864	645	-0.00658002	0.9294215	0.94954846
PWWP2B	311	235	0.010898308	0.9296532	0.949707271
AGBL5	1190	889	-0.005554118	0.9300697	0.950054754
FH	3924	2949	0.003052913	0.9300938	0.950001484
FAM104B	305	227	-0.010964813	0.9301737	0.950005216
KCNJ2-AS1	57	42	-0.02542046	0.9301928	0.949946856
ZNF552	226	168	-0.012709408	0.9303504	0.950029884
NUPL2	799	602	0.006720116	0.9305205	0.950125697
XAB2	1953	1469	0.004294578	0.9306092	0.950138354
PIGF	537	405	0.008151952	0.9308858	0.950342807
LOC100130872	165	125	0.014614202	0.9312415	0.950628089
AQP3	104	77	-0.018501046	0.9312884	0.950597974
LYSMD1	331	250	0.01024901	0.9317527	0.950994055
LINC00662	169	128	0.014272695	0.9320413	0.95121059
TRAPPC11	3265	2454	0.003204389	0.9330611	0.952173356
PER1	452	341	0.008601098	0.9330872	0.952121949
C14orf169	436	325	-0.008736285	0.9334486	0.952412759
ZGLP1	63	48	0.022834709	0.9335143	0.952401769
SMIM7	2166	1620	-0.003887298	0.9339392	0.952757207
KCNAB2	262	198	0.01108575	0.9343089	0.953056258
GABPB1	857	640	-0.006071167	0.935125	0.953810685
SNX11	774	583	0.006314449	0.935737	0.954356688
ZNF324	379	286	0.00896943	0.9360938	0.954642433
ACVR2B	270	201	-0.010611774	0.9363997	0.954876272
ZNF461	387	292	0.008786934	0.9367374	0.955142373
LOC642361	191	142	-0.012529577	0.9368601	0.955189277
MIS18BP1	1685	1260	-0.004172726	0.9374518	0.955714381
PRPH2	195	145	-0.012269092	0.9375245	0.955710217
RNF113A	381	284	-0.008747936	0.9376959	0.955806755
UBA6	6483	4869	0.002116059	0.9377115	0.955744375
SYS1	1023	770	0.005276338	0.9382716	0.956237024

TRPC4AP	6170	4634	0.002139687	0.9385535	0.956446042
ZNF490	411	310	0.008281953	0.9385537	0.956367995
LOC146880	850	640	0.005761196	0.9385564	0.956292555
ZNF543	207	154	-0.011548285	0.9394013	0.957075088
THAP10	310	234	0.009392446	0.9394662	0.957062986
CEP78	981	733	-0.005287806	0.939529	0.957048654
HERC2P3	298	222	-0.009600522	0.9395338	0.956975275
CPSF6	4554	3421	0.002435944	0.9398961	0.957266026
NNAT	77	57	-0.018744395	0.9400837	0.957378803
NOS1AP	79	60	0.018261979	0.9404914	0.957715635
CREM	1017	760	-0.005096224	0.9406561	0.957805101
RSPRY1	2115	1590	0.003521234	0.9407795	0.957852441
COMMD1	922	694	0.005321044	0.9408961	0.95789284
GPSM2	552	412	-0.006871798	0.9410645	0.957985998
TBCC	934	703	0.005254271	0.9412592	0.958105896
CNOT10	862	644	-0.005475049	0.9413067	0.958075957
KIAA0430	4409	3301	-0.002396207	0.9418707	0.958571609
ZNF682	233	176	0.010397606	0.9418891	0.958512048
RBP5	83	63	0.017392624	0.9419133	0.958458384
CENPH	572	427	-0.006626945	0.9421396	0.958610348
KLLN	152	115	0.012714669	0.9425809	0.958981005
ZNF613	231	172	-0.010332155	0.942708	0.959031979
TPRKB	739	552	-0.005753966	0.9428869	0.959135652
LNX1	156	118	0.012392962	0.9433041	0.959481591
THAP11	771	576	-0.005509917	0.9441333	0.960246676
BCL2	89	66	-0.01618718	0.94434	0.960378485
PSMG2	1591	1190	-0.00382013	0.9443421	0.960302129
GCA	91	69	0.015881948	0.9444715	0.960355301
ZNF154	265	200	0.009159772	0.9454109	0.961232054
ZNF862	994	743	-0.004731509	0.9455183	0.961262834
FAHD2B	95	72	0.015221525	0.9456278	0.961295695
ZNF444	823	615	-0.005153888	0.9460035	0.961599059

DAD1	4728	3551	0.002147457	0.9460042	0.961521296
PCDHB5	394	297	0.007419433	0.9460992	0.961539402
RAD21	9995	7488	-0.001474	0.9461436	0.961506036
GTPBP4	4832	3629	0.002103734	0.9465245	0.961814687
CASC3	4872	3659	0.002087414	0.94672	0.961934869
CEP72	97	72	-0.014835709	0.9467276	0.96186413
ATG16L1	1399	1052	0.003890874	0.9467672	0.961825885
ZNF570	271	202	-0.008785427	0.9472142	0.962201476
TMED4	1708	1284	0.003489359	0.9472539	0.962163422
ZC3H12B	101	75	-0.014240661	0.9478137	0.962653458
UTP14A	1325	991	-0.003883265	0.9483629	0.963132712
PRPF3	2490	1871	0.002815948	0.9486082	0.963303363
CBX2	196	148	0.009895653	0.9492691	0.963895894
FAM131A	450	339	0.006512404	0.9494413	0.963992134
MAML2	2213	1663	0.002936849	0.9494688	0.963941495
NCBP2	6152	4608	-0.001762655	0.9494728	0.963866915
TIRAP	458	345	0.006400895	0.9498676	0.964189187
HERC2	6819	5108	-0.001649528	0.950216	0.964464171
ACOT8	438	327	-0.006488102	0.9504167	0.96458928
TCEAL1	788	589	-0.004775864	0.9510296	0.965132754
DDX27	2338	1750	-0.002767876	0.9510959	0.965121319
SFI1	450	336	-0.006311637	0.9511066	0.965053579
TMEM69	613	458	-0.005387344	0.9512832	0.965154097
HLTF	4660	3490	-0.001950787	0.9513309	0.96512391
PRIM1	337	254	0.007232037	0.9514041	0.96511956
ABCC6	54	41	0.017816634	0.9519882	0.965633393
FRMD4A	3054	2294	0.002317461	0.9531584	0.966741668
ACACB	240	181	0.008107423	0.9540161	0.967532778
SLC25A28	554	417	0.005313539	0.9542333	0.967674277
ZNF259	2299	1721	-0.002607237	0.954315	0.967678405
HDAC3	1955	1469	0.00281792	0.9544243	0.967710417
MAMDC4	139	105	0.010456577	0.9548456	0.968058817

EIF5A2	2052	1536	-0.002700383	0.9552967	0.968437302
IL10RB	1373	1032	0.003263477	0.9557624	0.968830506
PICK1	264	199	0.007382633	0.9560865	0.969080225
SLC35B3	2503	1874	-0.002385206	0.9563868	0.969305656
RPS6KC1	2596	1950	0.002335872	0.9564669	0.969307967
GABRA5	66	50	0.014614202	0.956481	0.969243425
OCLN	145	108	-0.009869456	0.9566221	0.969307577
C14orf142	272	205	0.00716939	0.9567146	0.969322417
ATP5J	2543	1904	-0.002345852	0.9567643	0.969293914
ARL6IP6	879	661	0.003939238	0.9572687	0.969726023
TBC1D22A	1489	1119	0.003018414	0.9573911	0.969771162
AHI1	1925	1441	-0.002635979	0.9577321	0.970037649
PID1	272	203	-0.006974792	0.9579914	0.970221395
ZNF529	813	608	-0.004031897	0.957995	0.970146155
HIST1H2BK	1954	1468	0.002573633	0.9583839	0.970461135
BCL3	630	471	-0.004472637	0.9589838	0.970989581
ARMCX1	2026	1522	0.002486316	0.9590618	0.970989689
DUSP14	3234	2422	-0.001968682	0.959077	0.970926082
CASP8	1108	829	-0.003361743	0.9591076	0.970878145
TSC22D1-AS1	74	55	-0.01294152	0.9593849	0.971080012
LOC730091	78	59	0.012392962	0.9598931	0.97151536
SLC25A23	2130	1600	0.002370606	0.9599777	0.971522115
INTS6	2927	2192	-0.002022816	0.959997	0.971462634
ZNF84	2170	1630	0.002329054	0.9603118	0.971702248
SLC25A12	1376	1034	0.002907847	0.9605383	0.971852544
EIF4E3	308	230	-0.006144358	0.9606122	0.97184837
IKZF4	762	573	0.003896273	0.9606455	0.971803104
UHRF1BP1L	3969	2973	-0.001703967	0.960757	0.971836931
KCTD15	933	698	-0.003497913	0.9609551	0.971958408
NFIA	320	239	-0.005909155	0.9613869	0.972316196
MAP10	82	61	-0.011662535	0.961461	0.972312149
ESYT2	6456	4846	0.001305958	0.961619	0.972392997

ACP6	328	245	-0.005761934	0.9618798	0.972577798
EIF2A	4185	3135	-0.001610048	0.9619228	0.97254225
PLEKHG3	193	144	-0.007379904	0.9625577	0.973105212
IFT27	870	654	0.003427367	0.9630109	0.973484394
KRIT1	2137	1605	0.002138521	0.9638354	0.974238704
HCN3	98	74	0.009895653	0.9641158	0.974403557
HIST3H2A	98	74	0.009895653	0.9641158	0.974403557
PIGQ	1101	824	-0.002946095	0.9642706	0.974441443
SIK3	2202	1649	-0.002070938	0.9644751	0.974568981
NGRN	5324	3989	-0.001331316	0.9644838	0.974498728
CMBL	2234	1673	-0.002039609	0.9647588	0.974697533
HKR1	854	639	-0.003268007	0.9650958	0.974958854
ZNF253	225	168	-0.006311637	0.9654164	0.975203662
RBM4	1524	1141	-0.002411979	0.9655806	0.975290423
TCEANC2	717	539	0.003464286	0.9660568	0.97569227
BCAS4	635	475	-0.003676947	0.9661377	0.975694852
GIN1	424	317	-0.004429293	0.9666722	0.97615553
ATP5SL	1539	1156	0.002300298	0.9669854	0.976392618
NDUFAF1	950	711	-0.002925822	0.9670377	0.97636632
IKBKE	1146	861	0.00263023	0.9674227	0.976675809
SLC25A25	978	732	-0.002838685	0.9675508	0.976725944
MCCC1	1341	1004	-0.002397836	0.9679012	0.977000498
GRIN3B	118	88	-0.008059299	0.9680285	0.977049826
XPC	4403	3305	0.001315559	0.9680674	0.977009964
TRERF1	3213	2407	-0.001532707	0.9682353	0.977100157
MRRF	1018	762	-0.002722527	0.9682475	0.977033343
MKS1	295	222	0.004996854	0.9685862	0.977295987
SENP7	1266	951	0.002391973	0.9688617	0.977494692
MAFF	1286	966	0.002356583	0.9690811	0.977636858
FANCD2	755	565	-0.003073645	0.9691299	0.977606953
RFC2	925	695	0.002711744	0.9698233	0.97822722
HINFP	516	386	-0.003618086	0.9699615	0.978287351

HSPBAP1	142	106	-0.006674533	0.9709454	0.979200428
SLC25A46	3869	2899	-0.001253264	0.9714952	0.9796755
CXXC5	1081	812	0.002337241	0.971884	0.979988239
BBS10	1622	1218	0.001892445	0.9721159	0.980142746
ZNF182	383	288	0.003876551	0.972235	0.980183491
ST3GAL6	158	118	-0.005985567	0.9725119	0.980383268
MAML3	411	309	0.003620576	0.973138	0.980935103
CEP55	2499	1876	0.001461058	0.97328	0.98099877
ZNF404	419	315	0.003553716	0.9733789	0.981019139
CNNM4	423	318	0.003521234	0.9734967	0.981058501
THTPA	381	285	-0.003676947	0.9737673	0.981251719
INO80	2615	1963	0.001401358	0.9737837	0.981188948
ATG4A	1035	775	-0.00221042	0.9740012	0.981328657
SH3RF3	1502	1125	-0.00180768	0.9743847	0.981635648
BACE2	1381	1037	0.001854706	0.9747831	0.981957544
CMC1	479	360	0.003123382	0.9749847	0.982081216
LOC100288637	190	142	-0.004956357	0.9750343	0.982051788
SUPV3L1	1123	841	-0.00202809	0.9751512	0.982090045
ZNF229	736	551	-0.002501316	0.9751916	0.982051331
CBLN3	218	164	0.004519812	0.9755731	0.982356095
C15orf40	449	336	-0.00310208	0.9759715	0.982677881
ZNF818P	206	154	-0.004561855	0.9760715	0.982699142
CCDC120	210	157	-0.004472637	0.9763124	0.982862191
PMEPA1	1000	751	0.002036945	0.9764321	0.982903247
CREBZF	3407	2557	0.001102345	0.9764612	0.98285305
GRPEL1	1661	1247	0.001561524	0.9767168	0.983030884
SUMO1	6333	4752	0.000797888	0.9767716	0.983006619
UBE2O	1709	1283	0.001520905	0.9769971	0.983154184
CSNK2A3	61	46	0.00797675	0.9771819	0.983260672
ATP6V1E2	59	44	-0.008059299	0.9773897	0.983350613
OVGP1	59	44	-0.008059299	0.9773897	0.983350613
LINC00667	1124	844	0.001825	0.9776139	0.983457003

MPHOSPH10	2529	1895	-0.001217057	0.9776182	0.983381917
NT5C3	904	677	-0.002014807	0.9778506	0.983536272
SEC61A2	908	680	-0.002005419	0.9779051	0.983511594
DSTNP2	298	224	0.003338533	0.9789082	0.984441022
OSR2	306	230	0.00325434	0.9791662	0.984620985
ZNF559	593	444	-0.002320296	0.9793416	0.984717787
FAM13A-AS1	71	53	-0.006674533	0.9794531	0.98475039
SNHG6	318	239	0.003135984	0.9795344	0.984752686
LOC100131691	75	56	-0.006311637	0.980029	0.985130643
LOC100133331	75	56	-0.006311637	0.980029	0.985130643
DHRS13	79	59	-0.005985567	0.980561	0.985546144
MGST3	4140	3103	-0.000814939	0.9808227	0.985729603
FIZ1	382	287	0.00263023	0.9811881	0.986017279
UNC5B	947	711	0.001637266	0.9815656	0.986316985
NFKBIL1	430	323	0.002349637	0.9821711	0.986845837
AKNA	1039	780	0.001502507	0.9822804	0.986876081
CCDC90A	1384	1037	-0.001275917	0.9826415	0.987159166
SUV39H1	462	347	0.002194943	0.9827367	0.987175191
CREB3L4	113	85	0.004364105	0.9830184	0.987378596
TIGD6	410	307	-0.002233122	0.9834669	0.987749397
WDR70	1235	927	0.001282334	0.9835126	0.987715628
GOLGA6L4	111	83	-0.004224303	0.9837327	0.987857005
EXO1	538	404	0.001901252	0.9838641	0.987909312
EIF3M	4473	3356	0.000652089	0.9840451	0.98801147
FAM219A	1648	1235	-0.001053069	0.9843656	0.988253502
ZNF718	141	106	0.003521234	0.9846965	0.988506085
THSD1	618	464	0.001670099	0.984809	0.988539361
KLHL11	486	364	-0.001865732	0.9849599	0.988611144
C4orf21	490	367	-0.001849554	0.985029	0.988600859
INE1	131	98	-0.003561026	0.9851007	0.988593142
C11orf87	143	107	-0.003252219	0.9857824	0.989197485
RHBDD3	546	409	-0.001647976	0.9859185	0.989254363

PLEKHG1	147	110	-0.0031605	0.9859912	0.98924762
LOC100506844	151	113	-0.003073645	0.9861918	0.989369222
STARD13	4496	3373	0.000542415	0.9866945	0.989793804
LOC100527964	179	134	-0.002574455	0.9874065	0.990428236
TTC9C	1042	782	0.001037367	0.9877488	0.990691838
CCDC61	265	199	0.001928203	0.9885144	0.991339968
MMP17	265	199	0.001928203	0.9885144	0.991339968
LYSMD4	273	205	0.00187509	0.9886635	0.991369785
FAM46B	277	208	0.001849684	0.9887355	0.991362189
SIPA1L1	3679	2760	0.000506722	0.988756	0.991302883
LRRC16A	267	200	-0.00168761	0.9899159	0.992345964
OSCP1	267	200	-0.00168761	0.9899159	0.992345964
ZNF624	287	215	-0.001561945	0.9903233	0.99263454
LOC100216546	1034	775	-0.000815838	0.990405	0.992636558
MRPL35	2250	1688	0.000542034	0.990594	0.992746066
SNIP1	1078	808	-0.000777848	0.9906591	0.992731467
PNMA1	3968	2975	-0.000370226	0.9914694	0.993463494
CDKAL1	549	412	0.00099032	0.9915106	0.993424857
C11orf24	4194	3146	0.000343941	0.9918514	0.993686414
ZSCAN12	749	562	0.000756544	0.9924251	0.994181204
TM2D3	965	724	0.000612887	0.9930348	0.99471192
SMUG1	511	383	-0.000826767	0.9931644	0.994761787
TRAPPC13	1261	946	0.000495945	0.9935572	0.995075159
TAF7	6024	4517	-0.000204724	0.9941875	0.995626343
PPIL3	667	500	-0.000606535	0.9942705	0.995629415
RIPK2	2437	1828	0.000311951	0.9943663	0.995645357
POLR1C	695	521	-0.000577474	0.9944316	0.995630709
KDM4A	2981	2236	0.000275945	0.9944884	0.995607479
CDC26	907	680	-0.000415673	0.995421	0.996461045
UVRAG	1115	836	-0.000316731	0.9961314	0.997092062
TIMM21	1227	920	-0.000277351	0.9964463	0.997327108
LAS1L	1683	1262	-0.000171135	0.9974318	0.998233321

GGPS1	1827	1370	-0.000148609	0.9976764	0.998397864
DGCR8	1843	1382	-0.000146323	0.9977022	0.998343406
ASCC2	3000	2250	0.000114632	0.9977031	0.998264178
STK3	1268	951	0.000114632	0.9985068	0.998988012
ADAL	436	327	0.000114632	0.9991244	0.999525649
FABP4	428	321	0.000114632	0.9991325	0.999453446
JPX	116	87	0.000114632	0.9995484	0.999789176
OSBP2	92	69	0.000114632	0.9995978	0.999758325
FGF14	88	66	0.000114632	0.9996066	0.99968689
ATP6AP1L	60	45	0.000114632	0.9996752	0.999675183
