# Supplementary material for: The exosomal miRNA/FOXN3 axis empowers fibroblasts with tumor-promoting functions via enhanced migration and altered secretome
Source: J Nanobiotechnology. 2026 Jun 5;24:742. doi: 10.1186/s12951-026-04633-6 (PMC13459620; doi:10.1186/s12951-026-04633-6)
Supplement: Supplementary file 5 [file 12951_2026_4633_MOESM6_ESM.pdf]

Table S5 CUT&RUN data (MRC5 with scrambled shRNAs vs MRC5 with FOXN3 shRNAs; *P* < 0.01)

Peaks			Gene				
Chromosome	Start	End	Gene symbol	Strand	Chromosome	Start (TSS)	End (TSS)
chr1	856760	861760	SAMD11	+	chr1	858232	858834
chr1	892467	897467	KLHL17	+	chr1	892823	893212
chr1	893189	898189	NOC2L	-	chr1	892823	893212
chr1	898377	903377	PLEKHN1	+	chr1	901043	901378
chr1	945303	950303	ISG15	+	chr1	948863	949210
chr1	1164129	1169129	B3GALT6	+	chr1	1167037	1167380
chr1	1165911	1170911	SDF4	-	chr1	1167037	1167380
chr1	1212316	1217316	SCNN1D	+	chr1	1213977	1214663
chr1	1240447	1245447	PUSL1	+	chr1	1242896	1243367
chr1	1243489	1248489	ACAP3	-	chr1	1247502	1247836
chr1	1256636	1261636	GLTPD1	+	chr1	1261014	1261329
chr1	1258571	1263571	CPSF3L	-	chr1	1261014	1261329
chr1	1381569	1386569	ATAD3C	+	chr1	1381644	1382003
chr1	1403643	1408643	ATAD3B	+	chr1	1404660	1405010
chr1	1675931	1680931	SLC35E2	-	chr1	1679266	1679641
chr1	2142659	2147659	C1orf86	-	chr1	2145847	2146178
chr1	2144120	2149120	AL590822.1	-	chr1	2145847	2146178
chr1	2255081	2260081	AL589739.1	+	chr1	2255820	2256553
chr1	2319767	2324767	RER1	+	chr1	2322961	2323605
chr1	2321646	2326646	MORN1	-	chr1	2322961	2323605
chr1	2353919	2358919	PLCH2	+	chr1	2357644	2358051
chr1	6300752	6305752	HES3	+	chr1	6301483	6301820
chr1	6481348	6486348	ESPN	+	chr1	6481881	6482872
chr1	6611741	6616741	TAS1R1	+	chr1	6615134	6615488
chr1	6613095	6618095	NOL9	-	chr1	6615134	6615488
chr1	7840880	7845880	PER3	+	chr1	7842164	7842621
chr1	8010851	8015851	PARK7	+	chr1	8013328	8013906
chr1	9708290	9713290	PIK3CD	+	chr1	9707948	9708412
chr1	10486659	10491659	APITD1	+	chr1	10491623	10491982

chr1	10486659	10491659	APITD1-CORT	+	chr1	10491623	10491982
chr1	11068914	11073914	TARDBP	+	chr1	11069823	11070285
chr1	11118581	11123581	SRM	-	chr1	11119880	11120209
chr1	11118581	11123581	SRM	-	chr1	11123278	11123630
chr1	11818344	11823344	C1orf167	+	chr1	11820211	11820737
chr1	12223560	12228560	TNFRSF1B	+	chr1	12227357	12227725
chr1	13356319	13361319	PRAMEF5	+	chr1	13358023	13358514
chr1	14023193	14028193	PRDM2	+	chr1	14024622	14024962
chr1	16170859	16175859	SPEN	+	chr1	16173802	16174212
chr1	16301127	16306127	ZBTB17	-	chr1	16304935	16305252
chr1	16327231	16332231	C1orf64	+	chr1	16331688	16332135
chr1	16327231	16332231	C1orf64	+	chr1	16329125	16329574
chr1	16398627	16403627	FAM131C	-	chr1	16402658	16403573
chr1	16675570	16680570	SZRD1	+	chr1	16675371	16676341
chr1	16938557	16943557	NBPF1	-	chr1	16940151	16940469
chr1	17063268	17068268	CROCC	+	chr1	17066606	17067005
chr1	17336923	17341923	ATP13A2	-	chr1	17341629	17341964
chr1	19227775	19232775	ALDH4A1	-	chr1	19230935	19231345
chr1	19635320	19640320	PQLC2	+	chr1	19640207	19640686
chr1	19637140	19642140	AKR7A2	-	chr1	19640207	19640686
chr1	20205506	20210506	OTUD3	+	chr1	20205869	20206230
chr1	20502417	20507417	PLA2G2C	-	chr1	20504497	20504956
chr1	20613912	20618912	VWA5B1	+	chr1	20613735	20614106
chr1	21501877	21506877	EIF4G3	-	chr1	21501801	21502236
chr1	23033832	23038832	EPHB2	+	chr1	23037675	23038080
chr1	24113960	24118960	LYPLA2	+	chr1	24114462	24114796
chr1	24879102	24884102	NCMAP	+	chr1	24879414	24879728
chr1	24954707	24959707	SRRM1	+	chr1	24959405	24959889
chr1	25068348	25073348	CLIC4	+	chr1	25069408	25069730
chr1	26184403	26189403	AUNIP	-	chr1	26185614	26186092
chr1	26643354	26648354	UBXN11	-	chr1	26646675	26647226
chr1	27186133	27191133	SFN	+	chr1	27190953	27191610

chr1	28239757	28244757	RPA2	-	chr1	28242430	28242826
chr1	28258004	28263004	SMPDL3B	+	chr1	28261414	28261797
chr1	28518947	28523947	PTAFR	-	chr1	28523134	28523605
chr1	28523570	28528570	AL353354.2	+	chr1	28523134	28523605
chr1	28652013	28657013	MED18	+	chr1	28653390	28653698
chr1	28692614	28697614	PHACTR4	+	chr1	28696633	28697376
chr1	28692614	28697614	PHACTR4	+	chr1	28695040	28695421
chr1	28915212	28920212	RAB42	+	chr1	28919285	28919833
chr1	29555954	29560954	MECR	-	chr1	29556906	29557326
chr1	31380108	31385108	SDC3	-	chr1	31379400	31380231
chr1	31537338	31542338	PUM1	-	chr1	31538974	31539343
chr1	31848197	31853197	FABP3	-	chr1	31850155	31850575
chr1	31901114	31906114	AC114494.1	-	chr1	31901087	31901918
chr1	31901114	31906114	AC114494.1	-	chr1	31903043	31903403
chr1	32641787	32646787	TXLNA	+	chr1	32642497	32643008
chr1	32641787	32646787	TXLNA	+	chr1	32645619	32646092
chr1	32927158	32932158	RP1-27O5.3	+	chr1	32931065	32931401
chr1	32927170	32932170	ZBTB8B	+	chr1	32931065	32931401
chr1	33348095	33353095	HPCA	+	chr1	33351487	33351841
chr1	35243290	35248290	GJB3	+	chr1	35243273	35243851
chr1	35323917	35328917	SMIM12	-	chr1	35325874	35326497
chr1	35449454	35454454	ZMYM6NB	-	chr1	35449649	35450097
chr1	35657249	35662249	SFPQ	-	chr1	35660292	35660657
chr1	36035471	36040471	TFAP2E	+	chr1	36035748	36036102
chr1	36234068	36239068	CLSPN	-	chr1	36235912	36236433
chr1	36550976	36555976	ADPRHL2	+	chr1	36554115	36554738
chr1	36613598	36618598	TRAPPC3	-	chr1	36615874	36616204
chr1	36617680	36622680	MAP7D1	+	chr1	36622362	36622801
chr1	36928538	36933538	MRPS15	-	chr1	36930103	36930443
chr1	38269151	38274151	C1orf122	+	chr1	38273790	38274325
chr1	38272357	38277357	YRDC	-	chr1	38273790	38274325
chr1	38510950	38515950	POU3F1	-	chr1	38513633	38513955

chr1	39543488	39548488	MACF1	+	chr1	39545472	39546007
chr1	40366428	40371428	MYCL	-	chr1	40366623	40366967
chr1	40366428	40371428	MYCL	-	chr1	40369310	40369626
chr1	40708119	40713119	TMCO2	+	chr1	40713004	40713363
chr1	40912274	40917274	ZFP69B	+	chr1	40913237	40913655
chr1	41441507	41446507	CTPS1	+	chr1	41445750	41446071
chr1	42628889	42633889	GUCA2A	-	chr1	42630926	42631281
chr1	42925501	42930501	CCDC30	+	chr1	42926091	42926454
chr1	43634320	43639320	WDR65	+	chr1	43638110	43638491
chr1	43749788	43754788	C1orf210	-	chr1	43753184	43753489
chr1	43852053	43857053	SZT2	+	chr1	43854156	43854501
chr1	43853979	43858979	MED8	-	chr1	43854156	43854501
chr1	44167995	44172995	ST3GAL3	+	chr1	44171712	44172633
chr1	44495639	44500639	SLC6A9	-	chr1	44498919	44499577
chr1	45136864	45141864	C1orf228	+	chr1	45138658	45138992
chr1	45138727	45143727	TMEM53	-	chr1	45138658	45138992
chr1	45138727	45143727	TMEM53	-	chr1	45142023	45142440
chr1	45237423	45242423	RPS8	+	chr1	45241317	45241643
chr1	45262397	45267397	PLK3	+	chr1	45265630	45265935
chr1	45474319	45479319	UROD	+	chr1	45477609	45478016
chr1	45475501	45480501	HECTD3	-	chr1	45477609	45478016
chr1	45789045	45794045	HPDL	+	chr1	45791056	45791422
chr1	45987219	45992219	PRDX1	-	chr1	45990253	45990631
chr1	46149386	46154386	TMEM69	+	chr1	46151740	46152192
chr1	46765803	46770803	UQCRH	+	chr1	46769996	46770445
chr1	46767780	46772780	LRRC41	-	chr1	46769996	46770445
chr1	46969168	46974168	DMBX1	+	chr1	46971295	46971674
chr1	46969168	46974168	DMBX1	+	chr1	46973440	46973790
chr1	47655216	47660216	PDZK1IP1	-	chr1	47656799	47657115
chr1	50887672	50892672	DMRTA2	-	chr1	50888301	50888934
chr1	51698443	51703443	RNF11	+	chr1	51702007	51702386
chr1	51698443	51703443	RNF11	+	chr1	51700179	51701003

chr1	53064544	53069544	GPX7	+	chr1	53068443	53068765
chr1	53188626	53193626	ZYG11B	+	chr1	53189166	53189592
chr1	53304683	53309683	ZYG11A	+	chr1	53306647	53307034
chr1	53921572	53926572	DMRTB1	+	chr1	53924698	53925053
chr1	55265440	55270440	TTC22	-	chr1	55269586	55269987
chr1	55268236	55273236	C1orf177	+	chr1	55269586	55269987
chr1	55679286	55684286	USP24	-	chr1	55679283	55679613
chr1	57107495	57112495	PRKAA2	+	chr1	57108158	57108533
chr1	57107495	57112495	PRKAA2	+	chr1	57111315	57111642
chr1	57109474	57114474	PPAP2B	-	chr1	57111315	57111642
chr1	63152469	63157469	DOCK7	-	chr1	63157207	63157565
chr1	65609732	65614732	AK4	+	chr1	65612013	65612386
chr1	66995566	67000566	SGIP1	+	chr1	66998701	66999092
chr1	68961404	68966404	DEPDC1	-	chr1	68962286	68962606
chr1	84540245	84545245	PRKACB	+	chr1	84544114	84544484
chr1	85154986	85159986	SSX2IP	-	chr1	85156108	85156445
chr1	85357396	85362396	LPAR3	-	chr1	85360118	85360434
chr1	89146405	89151405	PKN2	+	chr1	89150034	89150449
chr1	89146405	89151405	PKN2	+	chr1	89146801	89147199
chr1	89986895	89991895	LRRC8B	+	chr1	89989953	89990339
chr1	94739124	94744124	ARHGAP29	-	chr1	94743150	94743862
chr1	95282398	95287398	SLC44A3	+	chr1	95286889	95287253
chr1	100500153	100505153	HIAT1	+	chr1	100503939	100504482
chr1	109285796	109290796	STXBP3	+	chr1	109286666	109287069
chr1	109789141	109794141	CELSR2	+	chr1	109789990	109790330
chr1	109848163	109853163	MYBPHL	-	chr1	109848351	109848708
chr1	109939073	109944073	SORT1	-	chr1	109941856	109942176
chr1	110033174	110038174	CYB561D1	+	chr1	110037347	110037829
chr1	110078994	110083994	GPR61	+	chr1	110079506	110079868
chr1	110195203	110200203	GSTM4	+	chr1	110198778	110199121
chr1	110689608	110694608	SLC6A17	+	chr1	110693608	110694221
chr1	110932204	110937204	SLC16A4	-	chr1	110934934	110935270

chr1	111739893	111744893	CHI3L2	+	chr1	111742198	111742849
chr1	111739893	111744893	CHI3L2	+	chr1	111740172	111740627
chr1	112012914	112017914	C1orf162	+	chr1	112014380	112014712
chr1	112081340	112086340	RAP1A	+	chr1	112086263	112086581
chr1	112935303	112940303	CTTNBP2NL	+	chr1	112935308	112935637
chr1	114468314	114473314	HIPK1	+	chr1	114472229	114472538
chr1	115211543	115216543	DENND2C	-	chr1	115216489	115216816
chr1	116181074	116186074	VANGL1	+	chr1	116185227	116185611
chr1	116650876	116655876	MAB21L3	+	chr1	116653889	116654336
chr1	116911790	116916790	ATP1A1	+	chr1	116913183	116913559
chr1	117112161	117117161	CD58	-	chr1	117112827	117113688
chr1	117293507	117298507	CD2	+	chr1	117294491	117294856
chr1	117293507	117298507	CD2	+	chr1	117297390	117297746
chr1	119530679	119535679	TBX15	-	chr1	119532584	119533062
chr1	145825603	145830603	GPR89A	-	chr1	145825657	145826303
chr1	147141118	147146118	ACP6	-	chr1	147141248	147141779
chr1	148346006	148351006	NBPF20	-	chr1	148349779	148350092
chr1	150035869	150040869	VPS45	+	chr1	150037724	150038130
chr1	150600588	150605588	ENSA	-	chr1	150604711	150605088
chr1	150950993	150955993	ANXA9	+	chr1	150954097	150954927
chr1	151430441	151435441	POGZ	-	chr1	151433734	151434097
chr1	151479486	151484486	CGN	+	chr1	151480009	151480412
chr1	151679409	151684409	AL589765.1	+	chr1	151679787	151680129
chr1	151679409	151684409	RIIAD1	+	chr1	151679787	151680129
chr1	151687790	151692790	CELF3	-	chr1	151689752	151690093
chr1	152195169	152200169	HRNR	-	chr1	152198581	152198886
chr1	152296179	152301179	FLG	-	chr1	152297151	152297473
chr1	152745348	152750348	LCE1F	+	chr1	152747804	152748171
chr1	153112427	153117427	SPRR2B	-	chr1	153113256	153113781
chr1	153326830	153331830	S100A9	+	chr1	153326667	153327125
chr1	153957334	153962334	RAB13	-	chr1	153959723	153960404
chr1	153959735	153964735	RPS27	+	chr1	153959723	153960404

chr1	154126092	154131092	NUP210L	-	chr1	154127198	154127848
chr1	154241487	154246487	HAX1	+	chr1	154242592	154242961
chr1	154952314	154957314	FLAD1	+	chr1	154956112	154956442
chr1	155142373	155147373	TRIM46	+	chr1	155143193	155143588
chr1	155223774	155228774	FAM189B	-	chr1	155224582	155225048
chr1	156027451	156032451	RAB25	+	chr1	156031554	156031890
chr1	156335503	156340503	RHBG	+	chr1	156335798	156336179
chr1	156336164	156341164	CCT3	-	chr1	156335798	156336179
chr1	156336164	156341164	CCT3	-	chr1	156340564	156341134
chr1	156785154	156790154	SH2D2A	-	chr1	156789588	156789904
chr1	156827310	156832310	INSRR	-	chr1	156830363	156830886
chr1	157013662	157018662	ARHGEF11	-	chr1	157013405	157013805
chr1	158572729	158577729	OR10Z1	+	chr1	158573087	158573415
chr1	159830947	159835947	VSIG8	-	chr1	159831271	159831689
chr1	159894022	159899022	TAGLN2	-	chr1	159897922	159898504
chr1	160309562	160314562	NCSTN	+	chr1	160309310	160309866
chr1	161057889	161062889	PVRL4	-	chr1	161059358	161060060
chr1	161280547	161285547	SDHC	+	chr1	161283804	161284201
chr1	161547601	161552601	FCGR2B	+	chr1	161548590	161548966
chr1	161716048	161721048	DUSP12	+	chr1	161719701	161720301
chr1	162036064	162041064	NOS1AP	+	chr1	162038901	162039276
chr1	162527823	162532823	UAP1	+	chr1	162528377	162528715
chr1	162597663	162602663	DDR2	+	chr1	162599447	162599775
chr1	167686929	167691929	MPZL1	+	chr1	167690703	167691082
chr1	167901521	167906521	DCAF6	+	chr1	167902612	167902997
chr1	170497770	170502770	GORAB	+	chr1	170498380	170498881
chr1	173442905	173447905	PRDX6	+	chr1	173445592	173446009
chr1	179557248	179562248	TDRD5	+	chr1	179559113	179559602
chr1	180878790	180883790	KIAA1614	+	chr1	180878463	180878839
chr1	190445259	190450259	BRINP3	-	chr1	190446424	190446845
chr1	200705186	200710186	CAMSAP2	+	chr1	200707467	200708132
chr1	200838583	200843583	GPR25	+	chr1	200840808	200841161

chr1	200991328	200996328	KIF21B	-	chr1	200992525	200992885
chr1	201588513	201593513	AC096677.1	+	chr1	201591033	201591394
chr1	201588911	201593911	NAV1	+	chr1	201591033	201591394
chr1	203143441	203148441	MYBPH	-	chr1	203143826	203144276
chr1	203154377	203159377	CHI3L1	-	chr1	203154431	203154787
chr1	203441456	203446456	PRELP	+	chr1	203443672	203444085
chr1	203827231	203832231	SNRPE	+	chr1	203827738	203828280
chr1	204345293	204350293	PLEKHA6	-	chr1	204349401	204349833
chr1	205008825	205013825	CNTN2	+	chr1	205011139	205011859
chr1	205179227	205184227	DSTYK	-	chr1	205179740	205180070
chr1	205717904	205722904	NUCKS1	-	chr1	205717820	205718175
chr1	205817760	205822760	PM20D1	-	chr1	205819615	205819987
chr1	206944339	206949339	IL10	-	chr1	206944346	206944744
chr1	207035199	207040199	IL20	+	chr1	207036391	207036713
chr1	207118311	207123311	PIGR	-	chr1	207118408	207119207
chr1	209824311	209829311	LAMB3	-	chr1	209826137	209826915
chr1	210402644	210407644	SERTAD4	+	chr1	210406857	210407304
chr1	213187668	213192668	ANGEL2	-	chr1	213188659	213189042
chr1	214153024	214158024	PROX1	+	chr1	214156746	214157156
chr1	214153024	214158024	PROX1	+	chr1	214153580	214153989
chr1	220218500	220223500	EPRS	-	chr1	220218456	220218921
chr1	220262304	220267304	BPNT1	-	chr1	220264732	220265058
chr1	220263944	220268944	IARS2	+	chr1	220264732	220265058
chr1	220444296	220449296	RAB3GAP2	-	chr1	220447421	220447786
chr1	221048199	221053199	HLX	+	chr1	221050432	221050937
chr1	224359958	224364958	DEGS1	+	chr1	224363424	224363868
chr1	226594280	226599280	PARP1	-	chr1	226594193	226594548
chr1	227504675	227509675	CDC42BPA	-	chr1	227505938	227506492
chr1	227504675	227509675	CDC42BPA	-	chr1	227508663	227508972
chr1	227747744	227752744	ZNF678	+	chr1	227748266	227748617
chr1	228191252	228196252	WNT3A	+	chr1	228193762	228194309
chr1	228350016	228355016	IBA57	+	chr1	228350230	228350667

chr1	230190036	230195036	GALNT2	+	chr1	230193334	230193639
chr1	231111227	231116227	ARV1	+	chr1	231113275	231113835
chr1	231113121	231118121	TTC13	-	chr1	231113275	231113835
chr1	233082851	233087851	NTPCR	+	chr1	233083178	233083485
chr1	235290751	235295751	TOMM20	-	chr1	235294063	235294408
chr1	235290751	235295751	TOMM20	-	chr1	235291658	235291983
chr1	236302332	236307332	GPR137B	+	chr1	236304215	236304558
chr1	236302332	236307332	GPR137B	+	chr1	236306237	236306773
chr1	236443819	236448819	ERO1LB	-	chr1	236444955	236445345
chr1	240174148	240179148	FMN2	+	chr1	240177212	240177933
chr1	247493648	247498648	ZNF496	-	chr1	247496410	247496849
chr1	247898417	247903417	OR14K1	+	chr1	247898749	247899086
chr1	247920482	247925482	OR1C1	-	chr1	247922771	247923260
chr1	248486399	248491399	OR2M7	-	chr1	248486378	248486774
chr1	248721297	248726297	OR2T29	-	chr1	248722444	248722764
chr10	976064	981064	LARP4B	-	chr10	977360	977670
chr10	5563424	5568424	CALML3	+	chr10	5565914	5566274
chr10	6183381	6188381	PFKFB3	+	chr10	6186887	6187391
chr10	11652253	11657253	USP6NL	-	chr10	11653062	11653733
chr10	11780865	11785865	ECHDC3	+	chr10	11784387	11784772
chr10	12234464	12239464	CDC123	+	chr10	12239130	12239531
chr10	12236643	12241643	NUDT5	-	chr10	12239130	12239531
chr10	13342912	13347912	PHYH	-	chr10	13346152	13346663
chr10	15135679	15140679	RPP38	+	chr10	15139961	15140431
chr10	15137818	15142818	C10orf111	-	chr10	15139961	15140431
chr10	15411561	15416561	FAM171A1	-	chr10	15410805	15411739
chr10	16475442	16480442	PTER	+	chr10	16480382	16480714
chr10	21813111	21818111	SKIDA1	-	chr10	21815043	21815703
chr10	22601403	22606403	COMMD3	+	chr10	22601284	22601726
chr10	22601403	22606403	COMMD3	+	chr10	22604950	22605261
chr10	22601817	22606817	COMMD3-BMI1	+	chr10	22604950	22605261
chr10	23477756	23482756	PTF1A	+	chr10	23481102	23481500

chr10	25460491	25465491	GPR158	+	chr10	25464378	25464797
chr10	27387921	27392921	ANKRD26	-	chr10	27389405	27390035
chr10	27440253	27445253	MASTL	+	chr10	27443365	27443844
chr10	27442695	27447695	YME1L1	-	chr10	27443365	27443844
chr10	30024210	30029210	SVIL	-	chr10	30026367	30026679
chr10	30661877	30666877	MTPAP	-	chr10	30662179	30662540
chr10	31319366	31324366	ZNF438	-	chr10	31324325	31324745
chr10	32216242	32221242	ARHGAP12	-	chr10	32218163	32218526
chr10	38379764	38384764	ZNF37A	+	chr10	38383503	38383828
chr10	43760867	43765867	RASGEF1A	-	chr10	43761686	43762588
chr10	43903114	43908114	HNRNPF	-	chr10	43904553	43904988
chr10	45451355	45456355	RASSF4	+	chr10	45455999	45456326
chr10	45809556	45814556	OR13A1	-	chr10	45812082	45812430
chr10	45866161	45871161	ALOX5	+	chr10	45867144	45867561
chr10	46990587	46995587	GPRIN2	+	chr10	46993739	46994143
chr10	47654081	47659081	ANTXRL	+	chr10	47653989	47654393
chr10	49511198	49516198	MAPK8	+	chr10	49514719	49515735
chr10	49889421	49894421	WDFY4	+	chr10	49891279	49891597
chr10	50322054	50327054	VSTM4	-	chr10	50323139	50323501
chr10	50503687	50508687	C10orf71	+	chr10	50504975	50505305
chr10	51545998	51550998	MSMB	+	chr10	51545632	51545998
chr10	51824148	51829148	FAM21A	+	chr10	51827089	51827551
chr10	52149266	52154266	AC069547.2	+	chr10	52152432	52152769
chr10	61664914	61669914	CCDC6	-	chr10	61665270	61665676
chr10	62534589	62539589	CDK1	+	chr10	62538953	62539269
chr10	70316913	70321913	TET1	+	chr10	70317472	70317811
chr10	70583798	70588798	STOX1	+	chr10	70586907	70587222
chr10	71331494	71336494	NEUROG3	-	chr10	71332477	71333077
chr10	71441155	71446155	RP11-242G20.1	+	chr10	71442349	71442713
chr10	72041932	72046932	NPFFR1	-	chr10	72045152	72045682
chr10	72140882	72145882	LRRC20	-	chr10	72142667	72143118
chr10	72206207	72211207	NODAL	-	chr10	72207677	72208047

chr10	72572217	72577217	SGPL1	+	chr10	72575880	72576217
chr10	73531755	73536755	C10orf54	-	chr10	73532307	73532771
chr10	73975392	73980392	ASCC1	-	chr10	73979197	73979523
chr10	75528549	75533549	FUT11	+	chr10	75530755	75531569
chr10	75530878	75535878	AC022400.2	-	chr10	75530755	75531569
chr10	77357498	77362498	C10orf11	+	chr10	77360141	77360537
chr10	79684878	79689878	DLG5	-	chr10	79687335	79687729
chr10	81103725	81108725	PPIF	+	chr10	81107792	81108203
chr10	81203883	81208883	ZCCHC24	-	chr10	81203486	81204285
chr10	81268857	81273857	EIF5AL1	+	chr10	81268637	81268986
chr10	81740870	81745870	SFTPD	-	chr10	81741733	81742066
chr10	82294158	82299158	SH2D4B	+	chr10	82296328	82296656
chr10	85929994	85934994	C10orf99	+	chr10	85934172	85934485
chr10	85999717	86004717	LRIT1	-	chr10	86004311	86005196
chr10	86001309	86006309	RGR	+	chr10	86004311	86005196
chr10	88124750	88129750	GRID1	-	chr10	88126171	88126649
chr10	88280072	88285072	WAPAL	-	chr10	88282343	88282710
chr10	88850418	88855418	FAM35A	+	chr10	88853928	88854582
chr10	88853123	88858123	GLUD1	-	chr10	88853928	88854582
chr10	90746914	90751914	FAS	+	chr10	90751137	90751525
chr10	90749647	90754647	ACTA2	-	chr10	90751137	90751525
chr10	90749647	90754647	ACTA2	-	chr10	90752114	90752512
chr10	91314898	91319898	SLC16A12	-	chr10	91315691	91316043
chr10	92976408	92981408	PCGF5	+	chr10	92980575	92980923
chr10	94332333	94337333	IDE	-	chr10	94333887	94334282
chr10	95750246	95755246	PLCE1	+	chr10	95752693	95753071
chr10	96302047	96307047	HELLS	+	chr10	96305255	96305585
chr10	97319671	97324671	SORBS1	-	chr10	97319933	97320522
chr10	97730286	97735286	CC2D2B	+	chr10	97730634	97730978
chr10	97730286	97735286	RP11-690P14.4	+	chr10	97730634	97730978
chr10	98478771	98483771	PIK3AP1	-	chr10	98480148	98480738
chr10	99182417	99187417	PGAM1	+	chr10	99185471	99185888

chr10	99184331	99189331	AL355490.1	-	chr10	99185471	99185888
chr10	99340580	99345580	HOGA1	+	chr10	99343501	99343850
chr10	99340631	99345631	PI4K2A	+	chr10	99343501	99343850
chr10	99340631	99345631	PI4K2A	+	chr10	99343501	99343850
chr10	99391844	99396844	MORN4	-	chr10	99392300	99392629
chr10	99493378	99498378	ZFYVE27	+	chr10	99494668	99495290
chr10	100026507	100031507	LOXL4	-	chr10	100027362	100027769
chr10	100173441	100178441	PYROXD2	-	chr10	100178047	100178620
chr10	100173441	100178441	PYROXD2	-	chr10	100174853	100175380
chr10	101987876	101992876	CHUK	-	chr10	101989352	101989950
chr10	101987876	101992876	CHUK	-	chr10	101992103	101992538
chr10	102044969	102049969	BLOC1S2	-	chr10	102045986	102046368
chr10	102044969	102049969	BLOC1S2	-	chr10	102049269	102049579
chr10	102491860	102496860	PAX2	+	chr10	102495250	102495613
chr10	102743624	102748624	C10orf2	+	chr10	102746354	102746711
chr10	102752875	102757875	LZTS2	+	chr10	102755895	102756462
chr10	102988051	102993051	LBX1	-	chr10	102987549	102988200
chr10	103878710	103883710	LDB1	-	chr10	103882022	103882593
chr10	103889287	103894287	PPRC1	+	chr10	103889895	103890267
chr10	103982585	103987585	ELOVL3	+	chr10	103986812	103987227
chr10	104175446	104180446	FBXL15	+	chr10	104179510	104179907
chr10	104179796	104184796	PSD	-	chr10	104179510	104179907
chr10	104260244	104265244	SUFU	+	chr10	104261813	104262229
chr10	104260244	104265244	SUFU	+	chr10	104264981	104265314
chr10	104260982	104265982	ACTR1A	-	chr10	104261813	104262229
chr10	104260982	104265982	ACTR1A	-	chr10	104264981	104265314
chr10	104951556	104956556	NT5C2	-	chr10	104955107	104955535
chr10	105109391	105114391	PCGF6	-	chr10	105113579	105114138
chr10	105124224	105129224	TAF5	+	chr10	105127733	105128074
chr10	105613801	105618801	SH3PXD2A	-	chr10	105617633	105618284
chr10	111752626	111757626	ADD3	+	chr10	111752382	111752706
chr10	112063209	112068209	SMNDC1	-	chr10	112067190	112067529

chr10	112323949	112328949	SMC3	+	chr10	112327841	112328169
chr10	114039993	114044993	TECTB	+	chr10	114042531	114042848
chr10	114130276	114135276	ACSL5	+	chr10	114131500	114131809
chr10	115995518	116000518	VWA2	+	chr10	115995471	115995841
chr10	118346397	118351397	PNLIPRP1	+	chr10	118347168	118347653
chr10	118997104	119002104	SLC18A2	+	chr10	119000186	119001081
chr10	119133478	119138478	PDZD8	-	chr10	119133776	119134120
chr10	120513261	120518261	CACUL1	-	chr10	120513178	120513587
chr10	120963601	120968601	GRK5	+	chr10	120963713	120964071
chr10	126478796	126483796	RP11-12J10.3	-	chr10	126479728	126480103
chr10	126478939	126483939	METTL10	-	chr10	126479728	126480103
chr10	126486854	126491854	FAM175B	+	chr10	126488802	126489147
chr10	127462890	127467890	MMP21	-	chr10	127465265	127465673
chr10	127462890	127467890	MMP21	-	chr10	127466164	127466597
chr10	131760605	131765605	EBF3	-	chr10	131761320	131761932
chr10	134347824	134352824	INPP5A	+	chr10	134348568	134348982
chr10	134347824	134352824	INPP5A	+	chr10	134350468	134351147
chr10	134754827	134759827	TTC40	-	chr10	134756282	134757162
chr10	134880933	134885933	GPR123	+	chr10	134882354	134882796
chr10	135148975	135153975	CALY	-	chr10	135152841	135153264
chr10	135200838	135205838	RP11-108K14.8	+	chr10	135200709	135201016
chr10	135341161	135346161	AL161645.2	-	chr10	135341967	135342337
chr11	189580	194580	SCGB1C1	+	chr11	189490	189823
chr11	304131	309131	IFITM2	+	chr11	309029	309367
chr11	366296	371296	B4GALNT4	+	chr11	366312	366628
chr11	556904	561904	RASSF7	+	chr11	559328	559759
chr11	559279	564279	C11orf35	-	chr11	559328	559759
chr11	633793	638793	DRD4	+	chr11	638746	639087
chr11	690938	695938	EPS8L2	+	chr11	693419	693737
chr11	691928	696928	TMEM80	+	chr11	693419	693737
chr11	705215	710215	DEAF1	-	chr11	706183	706530
chr11	743829	748829	TALDO1	+	chr11	746178	746576

chr11	796816	801816	SLC25A22	-	chr11	798176	798544
chr11	829343	834343	CD151	+	chr11	831947	832455
chr11	1148080	1153080	MUC5AC	+	chr11	1151589	1151916
chr11	2180939	2185939	INS-IGF2	-	chr11	2180437	2180972
chr11	2191607	2196607	TH	-	chr11	2195952	2196323
chr11	2319727	2324727	TSPAN32	+	chr11	2320833	2321193
chr11	2322779	2327779	C11orf21	-	chr11	2325365	2325771
chr11	2905611	2910611	CDKN1C	-	chr11	2910061	2910366
chr11	2917451	2922451	SLC22A18	+	chr11	2921632	2921965
chr11	2949185	2954185	PHLDA2	-	chr11	2949399	2949704
chr11	2949185	2954185	PHLDA2	-	chr11	2951173	2951706
chr11	3012107	3017107	NAP1L4	-	chr11	3016749	3017195
chr11	3238543	3243543	MRGPRG	-	chr11	3242062	3242415
chr11	3238543	3243543	MRGPRG	-	chr11	3240474	3240818
chr11	3252116	3257116	MRGPRE	-	chr11	3253667	3254328
chr11	3815454	3820454	PGAP2	+	chr11	3815142	3815686
chr11	3860713	3865713	RHOG	-	chr11	3864467	3864838
chr11	4824347	4829347	OR52R1	-	chr11	4827299	4827770
chr11	5461244	5466244	OR51I1	-	chr11	5461059	5461486
chr11	5637494	5642494	TRIM34	+	chr11	5638213	5638631
chr11	5637494	5642494	TRIM34	+	chr11	5640679	5641023
chr11	6254441	6259441	FAM160A2	-	chr11	6258946	6259634
chr11	8985058	8990058	TMEM9B	-	chr11	8986959	8987350
chr11	9402669	9407669	IPO7	+	chr11	9406067	9406442
chr11	10326360	10331360	AMPD3	+	chr11	10328926	10329232
chr11	11373404	11378404	CSNK2A3	-	chr11	11373326	11374069
chr11	12029816	12034816	DKK3	-	chr11	12029929	12030421
chr11	12395232	12400232	PARVA	+	chr11	12398912	12399335
chr11	18812768	18817768	PTPN5	-	chr11	18814896	18815308
chr11	19135146	19140146	ZDHHC13	+	chr11	19137500	19138036
chr11	22211222	22216222	ANO5	+	chr11	22214993	22215358
chr11	27012128	27017128	FIBIN	+	chr11	27015854	27016299

chr11	27492822	27497822	LGR4	-	chr11	27494278	27494695
chr11	27526820	27531820	LIN7C	-	chr11	27527237	27527603
chr11	32455676	32460676	WT1	-	chr11	32458327	32458712
chr11	33182417	33187417	CSTF3	-	chr11	33186948	33187342
chr11	33274718	33279718	HIPK3	+	chr11	33279255	33279716
chr11	33756491	33761491	CD59	-	chr11	33757594	33758035
chr11	34069730	34074730	CAPRIN1	+	chr11	34073773	34074189
chr11	34533852	34538852	ELF5	-	chr11	34538393	34538706
chr11	34933876	34938876	PDHX	+	chr11	34933890	34934511
chr11	35680853	35685853	TRIM44	+	chr11	35681318	35681783
chr11	44582477	44587477	CD82	+	chr11	44586677	44587150
chr11	45685672	45690672	CHST1	-	chr11	45686162	45686636
chr11	46718868	46723868	ZNF408	+	chr11	46718944	46719452
chr11	46938693	46943693	LRP4	-	chr11	46938918	46939708
chr11	47232993	47237993	DDB2	+	chr11	47236535	47237058
chr11	47266351	47271351	NR1H3	+	chr11	47267939	47268262
chr11	47425183	47430183	SLC39A13	+	chr11	47424714	47425196
chr11	47614711	47619711	C1QTNF4	-	chr11	47618702	47619009
chr11	51511782	51516782	OR4C46	+	chr11	51515215	51515591
chr11	55107127	55112127	OR4A16	+	chr11	55109256	55109683
chr11	55999237	56004237	OR5T2	-	chr11	56001113	56001646
chr11	56057066	56062066	OR8H1	-	chr11	56056544	56057128
chr11	56082283	56087283	OR8K3	+	chr11	56082078	56082715
chr11	56124191	56129191	OR8J1	+	chr11	56128584	56128918
chr11	57361360	57366360	SERPING1	+	chr11	57364448	57364802
chr11	57978717	57983717	OR1S1	+	chr11	57982545	57983344
chr11	59820560	59825560	MS4A3	+	chr11	59823262	59823676
chr11	59950639	59955639	MS4A6A	-	chr11	59953883	59954193
chr11	61272772	61277772	LRRC10B	+	chr11	61277062	61277543
chr11	61272772	61277772	LRRC10B	+	chr11	61273415	61273720
chr11	61444405	61449405	DAGLA	+	chr11	61448238	61448609
chr11	61972640	61977640	SCGB2A1	+	chr11	61977253	61977694

chr11	62429281	62434281	METTL12	+	chr11	62429395	62429899
chr11	63382855	63387855	PLA2G16	-	chr11	63385077	63385475
chr11	63970650	63975650	FERMT3	+	chr11	63973162	63973560
chr11	64123120	64128120	RPS6KA4	+	chr11	64126794	64127142
chr11	64123120	64128120	RPS6KA4	+	chr11	64125713	64126080
chr11	64511428	64516428	RASGRP2	-	chr11	64511540	64511890
chr11	64610541	64615541	CDC42BPG	-	chr11	64611026	64611381
chr11	64681525	64686525	PPP2R5B	+	chr11	64682077	64682507
chr11	64875817	64880817	TM7SF2	+	chr11	64876001	64876319
chr11	65358447	65363447	AP001362.1	-	chr11	65360250	65360928
chr11	65402068	65407068	SIPA1	+	chr11	65402662	65403091
chr11	65402068	65407068	SIPA1	+	chr11	65405148	65405477
chr11	65402068	65407068	SIPA1	+	chr11	65403551	65404137
chr11	65486918	65491918	RNASEH2C	-	chr11	65489797	65490146
chr11	65546773	65551773	AP5B1	-	chr11	65548730	65549048
chr11	65666544	65671544	FOSL1	-	chr11	65666530	65666853
chr11	65766050	65771050	BANF1	+	chr11	65767633	65768039
chr11	65834334	65839334	PACS1	+	chr11	65835309	65835716
chr11	65835590	65840590	RP11-1167A19.2	-	chr11	65835309	65835716
chr11	66083015	66088015	CD248	-	chr11	66084525	66084855
chr11	66102811	66107811	RIN1	-	chr11	66102469	66102960
chr11	66111096	66116096	BRMS1	-	chr11	66114155	66114754
chr11	66111921	66116921	RP11-867G23.8	+	chr11	66114155	66114754
chr11	66113663	66118663	B3GNT1	-	chr11	66114155	66114754
chr11	66243984	66248984	DPP3	+	chr11	66245756	66246184
chr11	66380553	66385553	RBM14	+	chr11	66381140	66381520
chr11	66380597	66385597	RBM14-RBM4	+	chr11	66381140	66381520
chr11	66380597	66385597	RBM4	+	chr11	66381140	66381520
chr11	66508422	66513422	C11orf80	+	chr11	66512926	66513258
chr11	66620618	66625618	LRFN4	+	chr11	66620896	66621363
chr11	66739248	66744248	C11orf86	+	chr11	66742538	66742906
chr11	67192431	67197431	RPS6KB2	+	chr11	67192231	67192541

chr11	67772548	67777548	ALDH3B1	+	chr11	67775986	67776329
chr11	67802983	67807983	TCIRG1	+	chr11	67802630	67802988
chr11	68224686	68229686	PPP6R3	+	chr11	68228652	68228972
chr11	69632292	69637292	FGF3	-	chr11	69635432	69636350
chr11	69632292	69637292	FGF3	-	chr11	69633370	69633693
chr11	70113306	70118306	PPFIA1	+	chr11	70116468	70116942
chr11	71312899	71317899	KRTAP5-11	-	chr11	71314458	71314825
chr11	71316987	71321987	AP000867.1	-	chr11	71321135	71321522
chr11	71495056	71500056	FAM86C1	+	chr11	71499077	71499516
chr11	71706087	71711087	IL18BP	+	chr11	71706541	71707488
chr11	71897102	71902102	FOLR1	+	chr11	71897452	71897802
chr11	72503144	72508144	ARAP1	-	chr11	72507166	72507486
chr11	72503226	72508226	STARD10	-	chr11	72507166	72507486
chr11	74108018	74113018	PGM2L1	-	chr11	74109707	74110304
chr11	75269601	75274601	SERPINH1	+	chr11	75273124	75273459
chr11	75467057	75472057	DGAT2	+	chr11	75468928	75469287
chr11	75920280	75925280	WNT11	-	chr11	75920091	75920495
chr11	76090515	76095515	PRKRIR	-	chr11	76092145	76092488
chr11	76810386	76815386	OMP	+	chr11	76812146	76812792
chr11	76835810	76840810	MYO7A	+	chr11	76836923	76837697
chr11	77296936	77301936	AQP11	+	chr11	77301267	77301673
chr11	77347350	77352350	CLNS1A	-	chr11	77352290	77352602
chr11	85520684	85525684	SYTL2	-	chr11	85523069	85523533
chr11	85779424	85784424	PICALM	-	chr11	85783998	85784361
chr11	92699386	92704386	MTNR1B	+	chr11	92703406	92703849
chr11	93059637	93064637	CCDC67	+	chr11	93061962	93062353
chr11	93391305	93396305	KIAA1731	+	chr11	93392695	93393345
chr11	93858594	93863594	PANX1	+	chr11	93862023	93862353
chr11	94964205	94969205	SESN3	-	chr11	94965414	94966029
chr11	95519629	95524629	CEP57	+	chr11	95523383	95523857
chr11	95522073	95527073	FAM76B	-	chr11	95523383	95523857
chr11	102334486	102339486	RP11-315O6.2	+	chr11	102336999	102337320

chr11	104033607	104038607	PDGFD	-	chr11	104034668	104034979
chr11	107458317	107463317	ELMOD1	+	chr11	107462547	107462855
chr11	107458971	107463971	AP000889.3	+	chr11	107462547	107462855
chr11	111174270	111179270	COLCA1	-	chr11	111174400	111174781
chr11	111324855	111329855	POU2AF1	-	chr11	111329335	111329694
chr11	111381579	111386579	BTG4	-	chr11	111384500	111384962
chr11	111382010	111387010	C11orf88	+	chr11	111384500	111384962
chr11	111407505	111412505	LAYN	+	chr11	111411140	111411480
chr11	111740805	111745805	ALG9	-	chr11	111743545	111743973
chr11	111750467	111755467	FDXACB1	-	chr11	111755341	111755745
chr11	112034595	112039595	TEX12	+	chr11	112038838	112039520
chr11	112037753	112042753	AP002884.3	+	chr11	112038838	112039520
chr11	114429117	114434117	NXPE1	-	chr11	114428990	114429425
chr11	115374175	115379175	CADM1	-	chr11	115374219	115374714
chr11	115374175	115379175	CADM1	-	chr11	115378158	115378487
chr11	116657266	116662266	ZNF259	-	chr11	116658936	116659358
chr11	116692522	116697522	APOA4	-	chr11	116693205	116694082
chr11	117686740	117691740	DSCAML1	-	chr11	117687023	117687676
chr11	117745882	117750882	FXYD6-FXYD2	-	chr11	117748221	117748539
chr11	117746701	117751701	FXYD6	-	chr11	117748221	117748539
chr11	118838917	118843917	FOXR1	+	chr11	118840984	118841338
chr11	118887901	118892901	RPS25	-	chr11	118892554	118893227
chr11	119052666	119057666	PDZD3	+	chr11	119053333	119054214
chr11	119052666	119057666	PDZD3	+	chr11	119056456	119056885
chr11	119065979	119070979	CCDC153	-	chr11	119067445	119067754
chr11	119598294	119603294	PVRL1	-	chr11	119599155	119599549
chr11	120192338	120197338	TMEM136	+	chr11	120197076	120197476
chr11	122932438	122937438	HSPA8	-	chr11	122932803	122933413
chr11	123813080	123818080	OR6T1	-	chr11	123815600	123816181
chr11	123982569	123987569	VWA5A	+	chr11	123984499	123984888
chr11	124265764	124270764	OR8B3	-	chr11	124267240	124267556
chr11	124757700	124762700	RP11-664I21.6	+	chr11	124757863	124758410

chr11	125699711	125704711	PATE4	+	chr11	125699362	125699713	
chr11	125699711	125704711	PATE4	+	chr11	125703765	125704093	
chr11	126170147	126175147	DCPS	+	chr11	126174988	126175363	
chr11	126170147	126175147	DCPS	+	chr11	126174210	126174561	
chr11	126172713	126177713	RP11-712L6.5	-	chr11	126174988	126175363	
chr11	126172713	126177713	RP11-712L6.5	-	chr11	126174210	126174561	
chr11	126222035	126227035	ST3GAL4	+	chr11	126224764	126225079	
chr11	128735768	128740768	KCNJ1	-	chr11	128737171	128737650	
chr11	128757751	128762751	KCNJ5	+	chr11	128758452	128758762	
chr11	130025957	130030957	ST14	+	chr11	130026794	130027150	
chr12	566030	571030	B4GALNT3	+	chr12	570134	570521	
chr12	1701831	1706831	FBXL14	-	chr12	1702831	1703140	
chr12	2900619	2905619	FKBP4	+	chr12	2904352	2904998	
chr12	2918288	2923288	ITFG2	+	chr12	2920829	2921210	
chr12	2981924	2986924	RHNO1	+	chr12	2984139	2984556	
chr12	4763300	4768300	RP11-234B24.6	+	chr12	4765253	4765570	
chr12	4826007	4831007	GALNT8	+	chr12	4826881	4827700	
chr12	5149585	5154585	KCNA5	+	chr12	5152114	5152857	
chr12	6557356	6562356	TAPBPL	+	chr12	6558349	6558806	
chr12	6599022	6604022	NCAPD2	+	chr12	6601816	6602251	
chr12	6601507	6606507	MRPL51	-	chr12	6601816	6602251	
chr12	6601507	6606507	MRPL51	-	chr12	6605837	6606191	
chr12	6715142	6720142	CHD4	-	chr12	6715924	6716351	
chr12	6744113	6749113	LPAR5	-	chr12	6749087	6749439	
chr12	6997022	7002022	SPSB2	-	chr12	6998519	6998915	
chr12	7078488	7083488	PHB2	-	chr12	7082023	7082329	
chr12	7902701	7907701	CLEC4C	-	chr12	7904647	7905198	
chr12	10560856	10565856	KLRC4	-	chr12	10560517	10561116	
chr12	11060661	11065661	TAS2R13	-	chr12	11061470	11061897	
chr12	12508501	12513501	LOH12CR2	-	chr12	12512235	12512598	
chr12	12875351	12880351	APOLD1	+	chr12	12878680	12879073	
chr12	13040216	13045216	GPRC5A	+	chr12	13040213	13040660	

chr12	13151707	13156707	HEBP1	-	chr12	13151571	13152204
chr12	14719783	14724783	PLBD1	-	chr12	14723775	14724214
chr12	14848019	14853019	GUCY2C	-	chr12	14851867	14852224
chr12	15113162	15118162	ARHGDIB	-	chr12	15116882	15117250
chr12	18397048	18402048	PIK3C2G	+	chr12	18401994	18402482
chr12	19553479	19558479	AEBP2	+	chr12	19557420	19557806
chr12	20518679	20523679	PDE3A	+	chr12	20522988	20523364
chr12	21909291	21914291	LDHB	-	chr12	21913320	21913630
chr12	24102466	24107466	SOX5	-	chr12	24105888	24106300
chr12	27233947	27238947	C12orf71	-	chr12	27235789	27236320
chr12	27233947	27238947	C12orf71	-	chr12	27238548	27238991
chr12	27482287	27487287	ARNTL2	+	chr12	27484662	27484985
chr12	27482287	27487287	ARNTL2	+	chr12	27482188	27482570
chr12	31742531	31747531	DENND5B	-	chr12	31745352	31745840
chr12	31796594	31801594	METTL20	+	chr12	31797924	31798249
chr12	32108804	32113804	KIAA1551	+	chr12	32110814	32111615
chr12	32548963	32553963	FGD4	+	chr12	32552386	32552710
chr12	46662300	46667300	SLC38A1	-	chr12	46663251	46663608
chr12	48353852	48358852	TMEM106C	+	chr12	48355808	48356119
chr12	48872786	48877786	C12orf54	+	chr12	48877144	48877477
chr12	49391592	49396592	DDN	-	chr12	49394318	49394747
chr12	49737200	49742200	DNAJC22	+	chr12	49739055	49739417
chr12	50221033	50226033	NCKAP5L	-	chr12	50221648	50222101
chr12	50494102	50499102	GPD1	+	chr12	50496124	50496492
chr12	50494102	50499102	GPD1	+	chr12	50498994	50499383
chr12	50675829	50680829	LIMA1	-	chr12	50679764	50680125
chr12	52278244	52283244	ANKRD33	+	chr12	52280661	52281114
chr12	52297192	52302192	ACVRL1	+	chr12	52301205	52301585
chr12	52297192	52302192	ACVRL1	+	chr12	52298757	52299253
chr12	52844410	52849410	KRT6B	-	chr12	52845409	52845822
chr12	54363410	54368410	HOXC11	+	chr12	54367810	54368276
chr12	55816538	55821538	OR6C76	+	chr12	55818627	55819144

chr12	56071830	56076830	METTL7B	+	chr12	56076444	56076814
chr12	56121991	56126991	CD63	-	chr12	56123741	56124046
chr12	56508443	56513443	ZC3H10	+	chr12	56512028	56512384
chr12	56508534	56513534	ESYT1	+	chr12	56512028	56512384
chr12	56861450	56866450	MIP	-	chr12	56864968	56865399
chr12	57123912	57128912	NACA	-	chr12	57126152	57127072
chr12	57850418	57855418	GLI1	+	chr12	57852635	57852964
chr12	57939614	57944614	DCTN2	-	chr12	57942645	57943279
chr12	57940281	57945281	KIF5A	+	chr12	57942645	57943279
chr12	58025638	58030638	B4GALNT1	-	chr12	58027657	58027990
chr12	58128296	58133296	TSPAN31	+	chr12	58131530	58132170
chr12	58134440	58139440	AGAP2	-	chr12	58137429	58137788
chr12	58161269	58166269	CYP27B1	-	chr12	58161185	58161614
chr12	65668923	65673923	MSRB3	+	chr12	65670764	65671092
chr12	66316467	66321467	AC090673.2	-	chr12	66319132	66319459
chr12	68724661	68729661	MDM1	-	chr12	68726208	68726646
chr12	75602148	75607148	KCNC2	-	chr12	75605707	75606132
chr12	75781350	75786350	GLIPR1L2	+	chr12	75782228	75782610
chr12	75903916	75908916	KRR1	-	chr12	75904546	75904946
chr12	77153868	77158868	ZDHHC17	+	chr12	77157946	77158350
chr12	80796274	80801274	PTPRQ	+	chr12	80800088	80800465
chr12	86264573	86269573	NTS	+	chr12	86268135	86268538
chr12	91575400	91580400	DCN	-	chr12	91580219	91580605
chr12	93111781	93116781	PLEKHG7	+	chr12	93112486	93113233
chr12	95608022	95613022	VEZT	+	chr12	95610271	95610634
chr12	95609758	95614758	FGD6	-	chr12	95610271	95610634
chr12	98983869	98988869	SLC25A3	+	chr12	98986395	98986766
chr12	100038031	100043031	FAM71C	+	chr12	100039600	100040288
chr12	100657418	100662418	SCYL2	+	chr12	100660910	100661266
chr12	103888249	103893249	C12orf42	-	chr12	103889260	103889568
chr12	105625568	105630568	C12orf75	+	chr12	105625375	105625982
chr12	106973185	106978185	RFX4	+	chr12	106975213	106975803

chr12	107164873	107169873	RIC8B	+	chr12	107167283	107167631
chr12	107167196	107172196	RP11-144F15.1	-	chr12	107167283	107167631
chr12	107708690	107713690	BTBD11	+	chr12	107712741	107713195
chr12	109745525	109750525	FOXN4	-	chr12	109746058	109746422
chr12	110269712	110274712	TRPV4	-	chr12	110272692	110273036
chr12	110509991	110514991	C12orf76	-	chr12	110512182	110512548
chr12	111048332	111053332	TCTN1	+	chr12	111052736	111053158
chr12	111141255	111146255	HVCN1	-	chr12	111140993	111141607
chr12	111468328	111473328	CUX2	+	chr12	111472166	111472486
chr12	112276282	112281282	MAPKAPK5	+	chr12	112275904	112276407
chr12	112447620	112452620	ERP29	+	chr12	112450784	112451138
chr12	112449470	112454470	TMEM116	-	chr12	112450784	112451138
chr12	112818396	112823396	HECTD4	-	chr12	112820203	112820646
chr12	113491014	113496014	DTX1	+	chr12	113495909	113496288
chr12	114844747	114849747	TBX5	-	chr12	114847433	114847850
chr12	119769017	119774017	CCDC60	+	chr12	119772918	119773242
chr12	120134073	120139073	RP1-127H14.3	+	chr12	120136071	120136691
chr12	120736619	120741619	SIRT4	+	chr12	120740701	120741082
chr12	120872393	120877393	COX6A1	+	chr12	120876523	120876971
chr12	120872501	120877501	AL021546.6	+	chr12	120876523	120876971
chr12	120880741	120885741	GATC	+	chr12	120881157	120881508
chr12	120967783	120972783	RNF10	+	chr12	120967932	120968535
chr12	121452805	121457805	C12orf43	-	chr12	121457746	121458975
chr12	121734611	121739611	CAMKK2	-	chr12	121735235	121735584
chr12	122060955	122065955	ORAI1	+	chr12	122062284	122062687
chr12	122060955	122065955	ORAI1	+	chr12	122061108	122061473
chr12	122109037	122114037	MORN3	-	chr12	122109867	122110195
chr12	122513128	122518128	MLXIP	+	chr12	122517994	122518317
chr12	123379491	123384491	VPS37B	-	chr12	123383919	123384309
chr12	123461107	123466107	ARL6IP4	+	chr12	123461918	123462392
chr12	124065578	124070578	TMED2	+	chr12	124069083	124069792
chr12	124152160	124157160	TCTN2	+	chr12	124152512	124153007

chr12	124452892	124457892	ZNF664	+	chr12	124457559	124458444
chr12	124452892	124457892	ZNF664	+	chr12	124455792	124456166
chr12	124454288	124459288	FAM101A	+	chr12	124457559	124458444
chr12	124454288	124459288	FAM101A	+	chr12	124455792	124456166
chr12	124455878	124460878	CCDC92	-	chr12	124457559	124458444
chr12	124455878	124460878	CCDC92	-	chr12	124455792	124456166
chr12	125050635	125055635	NCOR2	-	chr12	125054226	125054640
chr12	125050635	125055635	NCOR2	-	chr12	125051479	125051812
chr12	125474746	125479746	BRI3BP	+	chr12	125478101	125478528
chr12	132565328	132570328	EP400NL	+	chr12	132565754	132566719
chr12	132904435	132909435	GALNT9	-	chr12	132905715	132906028
chr12	133260692	133265692	PXMP2	+	chr12	133263781	133264159
chr12	133261135	133266135	RP13-672B3.2	+	chr12	133263781	133264159
chr12	133262451	133267451	POLE	-	chr12	133263781	133264159
chr12	133262451	133267451	POLE	-	chr12	133267331	133267746
chr12	133403944	133408944	GOLGA3	-	chr12	133408676	133409117
chr13	20109403	20114403	TPTE2	-	chr13	20110022	20110383
chr13	20204288	20209288	MPHOSPH8	+	chr13	20209130	20209503
chr13	20529310	20534310	ZMYM2	+	chr13	20533833	20534280
chr13	21711153	21716153	SAP18	+	chr13	21713237	21713732
chr13	24877804	24882804	C1QTNF9	+	chr13	24881900	24882472
chr13	26795291	26800291	RNF6	-	chr13	26795657	26796117
chr13	26824776	26829776	CDK8	+	chr13	26827501	26827955
chr13	28191403	28196403	POLR1D	+	chr13	28195038	28195413
chr13	28193041	28198041	LNX2	-	chr13	28195038	28195413
chr13	28543776	28548776	CDX2	-	chr13	28544575	28545286
chr13	32886111	32891111	BRCA2	+	chr13	32889902	32890256
chr13	32887981	32892981	ZAR1L	-	chr13	32889902	32890256
chr13	33000815	33005815	N4BP2L1	-	chr13	33001607	33002097
chr13	33157064	33162064	PDS5B	+	chr13	33159562	33160019
chr13	36917068	36922068	SPG20OS	+	chr13	36919620	36920126
chr13	41109823	41114823	AL133318.1	-	chr13	41109928	41110517

chr13	42028195	42033195	RGCC	+	chr13	42032794	42033116
chr13	46035560	46040560	COG3	+	chr13	46035867	46036181
chr13	46035560	46040560	COG3	+	chr13	46040183	46040798
chr13	50066246	50071246	PHF11	+	chr13	50068519	50068837
chr13	50652807	50657807	DLEU1	+	chr13	50652661	50653030
chr13	50652807	50657807	DLEU1	+	chr13	50656719	50657046
chr13	51793003	51798003	FAM124A	+	chr13	51794290	51794658
chr13	51793003	51798003	FAM124A	+	chr13	51796469	51796897
chr13	52155144	52160144	WDFY2	+	chr13	52155534	52155876
chr13	53023263	53028263	VPS36	-	chr13	53024223	53024717
chr13	60967091	60972091	TDRD3	+	chr13	60971173	60971480
chr13	73298561	73303561	BORA	+	chr13	73303521	73303939
chr13	73300325	73305325	MZT1	-	chr13	73303521	73303939
chr13	74991752	74996752	AL355390.1	-	chr13	74993381	74993736
chr13	77459040	77464040	KCTD12	-	chr13	77460843	77461568
chr13	78268523	78273523	SLAIN1	+	chr13	78268508	78268945
chr13	100622663	100627663	ZIC5	-	chr13	100622955	100623331
chr13	111364450	111369450	CARS2	-	chr13	111366704	111367089
chr13	113297858	113302858	C13orf35	+	chr13	113301986	113302466
chr13	113341143	113346143	ATP11A	+	chr13	113343687	113344139
chr13	113773628	113778628	F10	+	chr13	113773361	113773669
chr13	113773628	113778628	F10	+	chr13	113777525	113778212
chr13	114317970	114322970	GRK1	+	chr13	114322267	114322602
chr14	20707398	20712398	OR11H4	+	chr14	20707387	20707835
chr14	20970196	20975196	RNASE10	+	chr14	20974537	20974846
chr14	21027590	21032590	RNASE9	-	chr14	21029680	21030003
chr14	21057482	21062482	RNASE12	-	chr14	21059365	21059919
chr14	23424870	23429870	HAUS4	-	chr14	23426055	23426839
chr14	23502939	23507939	PSMB5	-	chr14	23503439	23503793
chr14	23838518	23843518	IL25	+	chr14	23842446	23842839
chr14	23842517	23847517	CMTM5	+	chr14	23842446	23842839
chr14	24419295	24424295	DHRS4	+	chr14	24419484	24420253

chr14	24419295	24424295	DHRS4	+	chr14	24422969	24423309
chr14	24456548	24461548	DHRS4-AS1	-	chr14	24459448	24459771
chr14	24681179	24686179	TM9SF1	-	chr14	24684997	24685307
chr14	24681179	24686179	TM9SF1	-	chr14	24684997	24685307
chr14	24681575	24686575	CHMP4A	-	chr14	24684997	24685307
chr14	24683776	24688776	MDP1	-	chr14	24684997	24685307
chr14	24710380	24715380	TINF2	-	chr14	24712897	24713500
chr14	24802799	24807799	ADCY4	-	chr14	24805259	24805606
chr14	24805088	24810088	RP11-934B9.3	-	chr14	24805259	24805606
chr14	24805088	24810088	RP11-934B9.3	-	chr14	24808801	24809226
chr14	24807751	24812751	RIPK3	-	chr14	24808801	24809226
chr14	31925147	31930147	RP11-176H8.1	-	chr14	31929244	31929838
chr14	31925216	31930216	DTD2	-	chr14	31929244	31929838
chr14	32541820	32546820	ARHGAP5	+	chr14	32544955	32545336
chr14	34930062	34935062	SPTSSA	-	chr14	34931384	34931781
chr14	34930480	34935480	EGLN3	-	chr14	34931384	34931781
chr14	35510613	35515613	FAM177A1	+	chr14	35515560	35515880
chr14	35587552	35592552	KIAA0391	+	chr14	35588232	35588568
chr14	35588255	35593255	KIAA0391	+	chr14	35588232	35588568
chr14	45427911	45432911	FAM179B	+	chr14	45432720	45433042
chr14	48142499	48147499	MDGA2	-	chr14	48142180	48142926
chr14	48142657	48147657	MDGA2	-	chr14	48142180	48142926
chr14	50100448	50105448	DNAAF2	-	chr14	50104126	50104442
chr14	50153640	50158640	POLE2	-	chr14	50157895	50158214
chr14	50156323	50161323	KLHDC1	+	chr14	50157895	50158214
chr14	50696776	50701776	SOS2	-	chr14	50701571	50701912
chr14	51561279	51566279	TRIM9	-	chr14	51564833	51565217
chr14	52730931	52735931	PTGDR	+	chr14	52734852	52735463
chr14	53161118	53166118	ERO1L	-	chr14	53164151	53164491
chr14	53256886	53261886	GNPNAT1	-	chr14	53257114	53257491
chr14	55030315	55035315	SAMD4A	+	chr14	55031440	55031808
chr14	55030315	55035315	SAMD4A	+	chr14	55034407	55035077

chr14	55921944	55926944	TBPL2	-	chr14	55923137	55923487
chr14	57734226	57739226	EXOC5	-	chr14	57737249	57737668
chr14	59651864	59656864	DAAM1	+	chr14	59655908	59656882
chr14	59947661	59952661	JKAMP	+	chr14	59952604	59952949
chr14	59949648	59954648	L3HYPDH	-	chr14	59952604	59952949
chr14	60555129	60560129	PCNXL4	+	chr14	60557828	60558237
chr14	60972169	60977169	SIX6	+	chr14	60975817	60976181
chr14	60980761	60985761	C14orf39	-	chr14	60984647	60985037
chr14	61992346	61997346	RP11-47I22.4	+	chr14	61993347	61993714
chr14	64803330	64808330	ESR2	-	chr14	64803064	64803489
chr14	64851249	64856249	MTHFD1	+	chr14	64851480	64851810
chr14	65167320	65172320	PLEKHG3	+	chr14	65171879	65172777
chr14	65377579	65382579	CHURC1	+	chr14	65379370	65379811
chr14	65377703	65382703	CHURC1-FNTB	+	chr14	65379370	65379811
chr14	65377703	65382703	FNTB	+	chr14	65379370	65379811
chr14	68065504	68070504	PIGH	-	chr14	68065117	68065505
chr14	68281807	68286807	ZFYVE26	-	chr14	68283009	68283340
chr14	68282996	68287996	RAD51B	+	chr14	68283009	68283340
chr14	69444657	69449657	ACTN1	-	chr14	69445323	69445655
chr14	70262506	70267506	SLC10A1	-	chr14	70265512	70265864
chr14	73389540	73394540	DCAF4	+	chr14	73393098	73393509
chr14	74766272	74771272	VRTN	+	chr14	74769314	74769625
chr14	74768259	74773259	ABCD4	-	chr14	74769314	74769625
chr14	74959380	74964380	NPC2	-	chr14	74964154	74964606
chr14	77607577	77612577	ZDHHC22	-	chr14	77608919	77609340
chr14	81424361	81429361	CEP128	-	chr14	81424880	81425506
chr14	85991443	85996443	RP11-497E19.2	+	chr14	85996022	85996412
chr14	85992988	85997988	FLRT2	+	chr14	85996022	85996412
chr14	87368622	87373622	RP11-322L20.1	+	chr14	87369660	87370025
chr14	90107515	90112515	RP11-944C7.1	-	chr14	90111726	90112173
chr14	90524609	90529609	KCNK13	+	chr14	90528907	90529414
chr14	91882688	91887688	CCDC88C	-	chr14	91883935	91884299

chr14	92245551	92250551	CATSPERB	-	chr14	92250483	92250810
chr14	92412831	92417831	FBLN5	-	chr14	92416334	92416649
chr14	94402401	94407401	RP11-131H24.4	+	chr14	94405679	94406058
chr14	94546091	94551091	DDX24	-	chr14	94551038	94551912
chr14	94567682	94572682	IFI27	+	chr14	94568900	94569317
chr14	94758108	94763108	SERPINA10	-	chr14	94759330	94759636
chr14	94982681	94987681	SERPINA12	-	chr14	94982586	94983230
chr14	94982681	94987681	SERPINA12	-	chr14	94985252	94985642
chr14	95054895	95059895	RP11-986E7.7	+	chr14	95056123	95056524
chr14	95235062	95240062	GSC	-	chr14	95236880	95237243
chr14	95235062	95240062	GSC	-	chr14	95235803	95236181
chr14	95940673	95945673	SYNE3	-	chr14	95944577	95945113
chr14	95940673	95945673	SYNE3	-	chr14	95942307	95942786
chr14	96149254	96154254	TCL1B	+	chr14	96153894	96154287
chr14	98228826	98233826	RP11-204N11.1	-	chr14	98228773	98229116
chr14	98228826	98233826	RP11-204N11.1	-	chr14	98230048	98230418
chr14	101355765	101360765	AL117190.3	+	chr14	101359605	101359947
chr14	102024188	102029188	DIO3	+	chr14	102026335	102026672
chr14	102602340	102607340	WDR20	+	chr14	102603807	102604343
chr14	103240313	103245313	TRAF3	+	chr14	103244092	103244517
chr14	103562981	103567981	EXOC3L4	+	chr14	103566887	103567367
chr14	103586279	103591279	TNFAIP2	+	chr14	103590849	103591361
chr14	103992021	103997021	TRMT61A	+	chr14	103993487	103993854
chr14	105187023	105192023	ADSSL1	+	chr14	105188237	105189336
chr14	105328117	105333117	CEP170B	+	chr14	105331067	105331516
chr14	105633661	105638661	JAG2	-	chr14	105633769	105634305
chr14	105780426	105785426	BRF1	-	chr14	105780378	105780688
chr15	23098505	23103505	NIPA1	-	chr15	23100093	23100454
chr15	25065294	25070294	SNRPN	+	chr15	25069885	25070244
chr15	33599663	33604663	RYR3	+	chr15	33603276	33603609
chr15	35086840	35091840	ACTC1	-	chr15	35088758	35089089
chr15	35278988	35283988	ZNF770	-	chr15	35280994	35281480

chr15	40073531	40078531	FSIP1	-	chr15	40074109	40074462
chr15	40631668	40636668	C15orf52	-	chr15	40632224	40632754
chr15	40639734	40644734	PHGR1	+	chr15	40642538	40642889
chr15	40728420	40733420	BAHD1	+	chr15	40730761	40731348
chr15	40728420	40733420	BAHD1	+	chr15	40729219	40729660
chr15	40855756	40860756	C15orf57	-	chr15	40855699	40856016
chr15	41119407	41124407	PPP1R14D	-	chr15	41123185	41123533
chr15	41132716	41137716	SPINT1	+	chr15	41136848	41137329
chr15	41241660	41246660	CHAC1	+	chr15	41244806	41245238
chr15	41519537	41524537	CHP1	+	chr15	41521380	41521711
chr15	42447339	42452339	PLA2G4F	-	chr15	42447346	42447668
chr15	42636801	42641801	CAPN3	+	chr15	42640588	42641020
chr15	42864357	42869357	STARD9	+	chr15	42867788	42868107
chr15	43211507	43216507	TTBK2	-	chr15	43212360	43212677
chr15	43473816	43478816	CCNDBP1	+	chr15	43477341	43477800
chr15	43619372	43624372	ADAL	+	chr15	43620922	43621238
chr15	45000175	45005175	B2M	+	chr15	45004316	45004705
chr15	45002014	45007014	PATL2	-	chr15	45005301	45005658
chr15	45002014	45007014	PATL2	-	chr15	45004316	45004705
chr15	47424820	47429820	FKSG62	-	chr15	47425585	47425905
chr15	48936546	48941546	FBN1	-	chr15	48936063	48936972
chr15	52040258	52045258	TMOD2	+	chr15	52043367	52043685
chr15	52042282	52047282	LYSMD2	-	chr15	52043367	52043685
chr15	52482066	52487066	GNB5	-	chr15	52482492	52483246
chr15	53081773	53086773	ONECUT1	-	chr15	53082774	53083441
chr15	53081773	53086773	ONECUT1	-	chr15	53081624	53082172
chr15	55607658	55612658	PIGB	+	chr15	55611311	55611806
chr15	55609811	55614811	RAB27A	-	chr15	55611311	55611806
chr15	55879645	55884645	PYGO1	-	chr15	55882829	55883292
chr15	56033788	56038788	PRTG	-	chr15	56034185	56034560
chr15	57207321	57212321	TCF12	+	chr15	57210110	57210458
chr15	57209269	57214269	ZNF280D	-	chr15	57210110	57210458

chr15	57880606	57885606	GCOM1	+	chr15	57882200	57882535
chr15	57880639	57885639	MYZAP	+	chr15	57882200	57882535
chr15	57880731	57885731	POLR2M	+	chr15	57882200	57882535
chr15	59153874	59158874	RNF111	+	chr15	59157299	59157908
chr15	59224352	59229352	SLTM	-	chr15	59226320	59226638
chr15	59661392	59666392	FAM81A	+	chr15	59662876	59663307
chr15	63886052	63891052	FBXL22	+	chr15	63888817	63889143
chr15	65424674	65429674	PDCD7	-	chr15	65425742	65426142
chr15	66991066	66996066	SMAD6	+	chr15	66994986	66995338
chr15	68520556	68525556	RP11-315D16.2	-	chr15	68523613	68524021
chr15	68867808	68872808	CORO2B	+	chr15	68867884	68868212
chr15	69107060	69112060	SPESP1	+	chr15	69109609	69110036
chr15	69741623	69746623	RPLP1	+	chr15	69741431	69741830
chr15	70389015	70394015	TLE3	-	chr15	70392647	70392987
chr15	72403099	72408099	SENP8	+	chr15	72403335	72403739
chr15	72610787	72615787	CELF6	-	chr15	72614362	72614731
chr15	72610970	72615970	CELF6	-	chr15	72614362	72614731
chr15	72667317	72672317	HEXA	-	chr15	72668339	72668722
chr15	73731999	73736999	C15orf60	+	chr15	73731935	73732318
chr15	74162449	74167449	TBC1D21	+	chr15	74162883	74163226
chr15	74420119	74425119	RP11-247C2.2	-	chr15	74420932	74421284
chr15	74420119	74425119	RP11-247C2.2	-	chr15	74424690	74425076
chr15	74462512	74467512	ISLR	+	chr15	74464721	74465276
chr15	74658581	74663581	CYP11A1	-	chr15	74658354	74658668
chr15	74725308	74730308	SEMA7A	-	chr15	74725100	74725408
chr15	74887341	74892341	CLK3	+	chr15	74890173	74890822
chr15	74887341	74892341	CLK3	+	chr15	74891488	74891824
chr15	75016451	75021451	CYP1A1	-	chr15	75019348	75019679
chr15	75070898	75075898	CSK	+	chr15	75073759	75074114
chr15	75101557	75106557	LMAN1L	+	chr15	75105454	75106115
chr15	75248305	75253305	RPP25	-	chr15	75251105	75251434
chr15	75484484	75489484	C15orf39	+	chr15	75488635	75489635

chr15	77282200	77287200	PSTPIP1	+	chr15	77285953	77286477
chr15	79381615	79386615	RASGRF1	-	chr15	79382704	79383356
chr15	81068184	81073184	KIAA1199	+	chr15	81069064	81069572
chr15	81068184	81073184	KIAA1199	+	chr15	81072179	81072499
chr15	83316112	83321112	CPEB1	-	chr15	83316957	83317363
chr15	83316112	83321112	CPEB1	-	chr15	83320164	83320895
chr15	83651459	83656459	FAM103A1	+	chr15	83654686	83655187
chr15	83653161	83658161	HOMER2	-	chr15	83654686	83655187
chr15	84319338	84324338	ADAMTSL3	+	chr15	84322031	84322530
chr15	84901025	84906025	GOLGA6L4	+	chr15	84902931	84903262
chr15	85196074	85201074	WDR73	-	chr15	85198799	85199122
chr15	85200294	85205294	NMB	-	chr15	85201351	85201689
chr15	85520171	85525171	PDE8A	+	chr15	85525109	85526031
chr15	85520171	85525171	PDE8A	+	chr15	85523666	85524050
chr15	86336761	86341761	KLHL25	-	chr15	86338892	86339517
chr15	89161027	89166027	AEN	+	chr15	89164966	89165483
chr15	89627190	89632190	ABHD2	+	chr15	89631017	89631324
chr15	90300322	90305322	MESP2	+	chr15	90303385	90303701
chr15	90541124	90546124	ZNF710	+	chr15	90541606	90541919
chr15	91537359	91542359	PRC1	-	chr15	91537085	91537998
chr15	91564333	91569333	VPS33B	-	chr15	91565623	91566103
chr15	93351614	93356614	FAM174B	-	chr15	93353832	93354212
chr15	94771267	94776267	MCTP2	+	chr15	94775790	94776153
chr15	100256529	100261529	DKFZP779J2370	-	chr15	100257849	100258481
chr15	102191094	102196094	TM2D3	-	chr15	102191722	102192317
chr15	102354866	102359866	OR4F15	+	chr15	102355140	102355456
chr15	102461798	102466798	OR4F4	-	chr15	102464778	102465094
chr16	102128	107128	POLR3K	-	chr16	104150	104586
chr16	219346	224346	HBA2	+	chr16	221822	222210
chr16	324480	329480	RGS11	-	chr16	325955	326998
chr16	443225	448225	NME4	+	chr16	444125	444920
chr16	606922	611922	C16orf11	+	chr16	606644	607347

chr16	606922	611922	C16orf11	+	chr16	611163	611818
chr16	754329	759329	FBXL16	-	chr16	754041	754360
chr16	773436	778436	HAGHL	+	chr16	773229	773590
chr16	851943	856943	PRR25	+	chr16	856010	856333
chr16	1027752	1032752	AC009041.2	+	chr16	1027290	1028213
chr16	1199741	1204741	CACNA1H	+	chr16	1204081	1204520
chr16	1380102	1385102	BAIAP3	+	chr16	1380964	1381350
chr16	1678760	1683760	LA16c-431H6.6	+	chr16	1682704	1683108
chr16	1724757	1729757	HN1L	+	chr16	1724552	1725030
chr16	1842201	1847201	SPSB3	-	chr16	1844769	1845258
chr16	1842201	1847201	SPSB3	-	chr16	1842224	1843182
chr16	1843472	1848472	IGFALS	-	chr16	1844769	1845258
chr16	2028133	2033133	AC005606.1	+	chr16	2032230	2032552
chr16	2030708	2035708	GFER	+	chr16	2032230	2032552
chr16	2202199	2207199	TRAF7	+	chr16	2204912	2205562
chr16	2260451	2265451	BRICD5	-	chr16	2265068	2265390
chr16	2263308	2268308	PGP	-	chr16	2265068	2265390
chr16	2506581	2511581	C16orf59	+	chr16	2508246	2508601
chr16	2506581	2511581	C16orf59	+	chr16	2511079	2511473
chr16	2518000	2523000	NTN3	+	chr16	2519021	2519418
chr16	2521647	2526647	TBC1D24	+	chr16	2523055	2523395
chr16	2521647	2526647	TBC1D24	+	chr16	2524038	2524798
chr16	2906671	2911671	PRSS22	-	chr16	2910762	2911080
chr16	3029040	3034040	PKMYT1	-	chr16	3029561	3030432
chr16	3543980	3548980	C16orf90	-	chr16	3543508	3544456
chr16	3766098	3771098	TRAP1	-	chr16	3767837	3768167
chr16	4782879	4787879	ANKS3	-	chr16	4787473	4787873
chr16	4816125	4821125	ZNF500	-	chr16	4818545	4818964
chr16	4816125	4821125	ZNF500	-	chr16	4820850	4821165
chr16	11889623	11894623	ZC3H7A	-	chr16	11890146	11890711
chr16	11943942	11948942	RSL1D1	-	chr16	11943679	11944022
chr16	12991977	12996977	SHISA9	+	chr16	12996109	12996514

chr16	14838423	14843423	NPIPA2	+	chr16	14841296	14841636
chr16	15013159	15018159	NPIPA1	+	chr16	15017609	15018282
chr16	15186674	15191674	RRN3	-	chr16	15190758	15191081
chr16	15486111	15491111	MPV17L	+	chr16	15489580	15490118
chr16	15486111	15491111	RP11-1021N1.1	+	chr16	15489580	15490118
chr16	16039934	16044934	ABCC1	+	chr16	16042643	16043029
chr16	16039934	16044934	ABCC1	+	chr16	16041626	16041950
chr16	16322852	16327852	NOMO3	+	chr16	16324108	16324768
chr16	19724278	19729278	IQCK	+	chr16	19725875	19726205
chr16	19728057	19733057	KNOP1	-	chr16	19732066	19732984
chr16	19728057	19733057	KNOP1	-	chr16	19731020	19731414
chr16	20708712	20713712	ACSM1	-	chr16	20713510	20713820
chr16	21312904	21317904	CRYM	-	chr16	21317653	21318277
chr16	22446986	22451986	CDR2	-	chr16	22450542	22451149
chr16	23463001	23468001	COG7	-	chr16	23462800	23463162
chr16	23606177	23611177	NDUFAB1	-	chr16	23610138	23610458
chr16	23649213	23654213	DCTN5	+	chr16	23650154	23650477
chr16	23649213	23654213	DCTN5	+	chr16	23651938	23652279
chr16	23649213	23654213	DCTN5	+	chr16	23652905	23653256
chr16	23651131	23656131	PALB2	-	chr16	23651938	23652279
chr16	23651131	23656131	PALB2	-	chr16	23652905	23653256
chr16	28505396	28510396	CLN3	-	chr16	28510360	28510855
chr16	28759608	28764608	NPIPB9	+	chr16	28761107	28761415
chr16	28992647	28997647	LAT	+	chr16	28996563	28996961
chr16	29981462	29986462	TAOK2	+	chr16	29984499	29984848
chr16	30060911	30065911	ALDOA	+	chr16	30062568	30062881
chr16	30062799	30067799	FAM57B	-	chr16	30062568	30062881
chr16	30106736	30111736	YPEL3	-	chr16	30107196	30107525
chr16	30536642	30541642	ZNF768	-	chr16	30537245	30537731
chr16	30544673	30549673	ZNF747	-	chr16	30545208	30545581
chr16	30545168	30550168	ZNF747	-	chr16	30545208	30545581
chr16	30633833	30638833	ZNF689	-	chr16	30636852	30637222

chr16	31069313	31074313	ZNF668	+	chr16	31072052	31072438
chr16	31099449	31104449	PRSS53	-	chr16	31101379	31101847
chr16	31113928	31118928	BCKDK	+	chr16	31118116	31118543
chr16	31209706	31214706	C16orf98	+	chr16	31213646	31214023
chr16	31213271	31218271	PYCARD	-	chr16	31213646	31214023
chr16	31267811	31272811	ITGAM	+	chr16	31271918	31272301
chr16	31362955	31367955	ITGAX	+	chr16	31365942	31366369
chr16	33200656	33205656	TP53TG3C	+	chr16	33202562	33203084
chr16	46720055	46725055	ORC6	+	chr16	46723309	46723655
chr16	46721930	46726930	VPS35	-	chr16	46723309	46723655
chr16	46863823	46868823	C16orf87	-	chr16	46865168	46865672
chr16	46914790	46919790	GPT2	+	chr16	46916821	46917343
chr16	48480813	48485813	SIAH1	-	chr16	48481549	48482062
chr16	50096352	50101352	HEATR3	+	chr16	50096526	50096986
chr16	50276548	50281548	ADCY7	+	chr16	50279436	50280012
chr16	50276548	50281548	ADCY7	+	chr16	50280483	50280914
chr16	50578741	50583741	NKD1	+	chr16	50579702	50580194
chr16	52580214	52585214	TOX3	-	chr16	52580364	52580812
chr16	55539410	55544410	LPCAT2	+	chr16	55541720	55542165
chr16	55865749	55870749	CES1	-	chr16	55870629	55871054
chr16	56595461	56600461	MT4	+	chr16	56600426	56600924
chr16	56638611	56643611	MT2A	+	chr16	56641924	56642248
chr16	56992262	56997262	CETP	+	chr16	56992642	56992959
chr16	57019897	57024897	NLRC5	+	chr16	57020659	57021047
chr16	57572833	57577833	GPR114	+	chr16	57574397	57574881
chr16	58006839	58011839	TEPP	+	chr16	58011090	58011551
chr16	58029950	58034950	USB1	+	chr16	58030875	58031297
chr16	66549715	66554715	RP11-403P17.5	-	chr16	66552209	66552539
chr16	66609851	66614851	CMTM2	+	chr16	66611725	66612566
chr16	66729110	66734110	CMTM4	-	chr16	66730281	66730673
chr16	66874782	66879782	CA7	+	chr16	66878715	66879317
chr16	66958047	66963047	RRAD	-	chr16	66959965	66960375

chr16	67183617	67188617	B3GNT9	-	chr16	67185412	67185825
chr16	67280061	67285061	FHOD1	-	chr16	67284737	67285240
chr16	67357201	67362201	LRRC36	+	chr16	67359876	67360193
chr16	67359166	67364166	KCTD19	-	chr16	67359876	67360193
chr16	67675322	67680322	RLTPR	+	chr16	67677325	67677726
chr16	67976534	67981534	LCAT	-	chr16	67976594	67976997
chr16	68032989	68037989	DPEP2	-	chr16	68037668	68038118
chr16	68056270	68061270	DDX28	-	chr16	68056231	68056722
chr16	69135967	69140967	HAS3	+	chr16	69139952	69140483
chr16	69372070	69377070	COG8	-	chr16	69375564	69375985
chr16	70284333	70289333	EXOSC6	-	chr16	70286179	70286807
chr16	70471640	70476640	ST3GAL2	-	chr16	70474949	70475389
chr16	70554191	70559191	SF3B3	+	chr16	70558175	70558584
chr16	70555968	70560968	COG4	-	chr16	70558175	70558584
chr16	71322118	71327118	CMTR2	-	chr16	71323176	71323563
chr16	71927739	71932739	ZNF821	-	chr16	71932017	71932587
chr16	73092097	73097097	ZFHX3	-	chr16	73095027	73095453
chr16	73176846	73181846	C16orf47	-	chr16	73176615	73177006
chr16	73176846	73181846	C16orf47	-	chr16	73179924	73180349
chr16	75249398	75254398	CTRB1	+	chr16	75249243	75249638
chr16	75497104	75502104	RP11-77K12.1	-	chr16	75501898	75502588
chr16	75497895	75502895	TMEM170A	-	chr16	75501898	75502588
chr16	75678184	75683184	TERF2IP	+	chr16	75681771	75682256
chr16	75681041	75686041	KARS	-	chr16	75681771	75682256
chr16	76307676	76312676	CNTNAP4	+	chr16	76310341	76310699
chr16	77818927	77823927	VAT1L	+	chr16	77818152	77819009
chr16	81065952	81070952	ATMIN	+	chr16	81070107	81070418
chr16	85144614	85149614	FAM92B	-	chr16	85145560	85145987
chr16	85641515	85646515	GSE1	+	chr16	85645452	85645792
chr16	85828739	85833739	COX4I1	+	chr16	85832867	85833336
chr16	85831714	85836714	EMC8	-	chr16	85832867	85833336
chr16	86606474	86611474	FOXL1	+	chr16	86606996	86607438

chr16	87737790	87742790	FLJ00104	-	chr16	87742182	87742699
chr16	87737790	87742790	FLJ00104	-	chr16	87737442	87738265
chr16	88490379	88495379	ZNF469	+	chr16	88495306	88495635
chr16	88701499	88706499	IL17C	+	chr16	88704596	88705023
chr16	88769371	88774371	CTU2	+	chr16	88773335	88773675
chr16	88771329	88776329	RNF166	-	chr16	88773335	88773675
chr16	88850119	88855119	PIEZO1	-	chr16	88849818	88850406
chr16	88866121	88871121	CDT1	+	chr16	88869775	88870149
chr16	88876852	88881852	APRT	-	chr16	88877444	88877883
chr16	88876852	88881852	APRT	-	chr16	88881008	88881401
chr16	88876852	88881852	APRT	-	chr16	88878343	88878654
chr16	89002697	89007697	RP11-830F9.6	+	chr16	89006670	89007003
chr16	89151283	89156283	ACSF3	+	chr16	89155886	89156291
chr16	89766613	89771613	SPATA2L	-	chr16	89770751	89771082
chr17	1130815	1135815	ABR	-	chr17	1133584	1133931
chr17	1364956	1369956	CRK	-	chr17	1367240	1367649
chr17	1586676	1591676	PRPF8	-	chr17	1591280	1591667
chr17	1729496	1734496	RPA1	+	chr17	1730646	1731130
chr17	2676850	2681850	RAP1GAP2	+	chr17	2681298	2681685
chr17	3180768	3185768	OR3A2	-	chr17	3181339	3181675
chr17	3335635	3340635	OR1E2	-	chr17	3337615	3337971
chr17	3498892	3503892	TRPV1	-	chr17	3501109	3501559
chr17	3597988	3602988	P2RX5-TAX1BP3	-	chr17	3600163	3600553
chr17	3598198	3603198	P2RX5	-	chr17	3600163	3600553
chr17	3748045	3753045	C17orf85	-	chr17	3749326	3749692
chr17	4165774	4170774	ANKFY1	-	chr17	4166182	4166626
chr17	4462613	4467613	GGT6	-	chr17	4463962	4464314
chr17	4706891	4711891	PLD2	+	chr17	4709955	4710406
chr17	4839803	4844803	RNF167	+	chr17	4842820	4843183
chr17	4842046	4847046	SLC25A11	-	chr17	4842820	4843183
chr17	4897743	4902743	KIF1C	+	chr17	4901538	4901978
chr17	4899405	4904405	INCA1	-	chr17	4901538	4901978

chr17	5370880	5375880	DHX33	-	chr17	5371386	5371717
chr17	5388631	5393631	DERL2	-	chr17	5393374	5393711
chr17	6337019	6342019	AIPL1	-	chr17	6338372	6338839
chr17	6540578	6545578	TXNDC17	+	chr17	6543882	6544307
chr17	6542747	6547747	KIAA0753	-	chr17	6543882	6544307
chr17	6912236	6917236	RNASEK	+	chr17	6914367	6914773
chr17	6912454	6917454	RNASEK-C17orf4	+	chr17	6914367	6914773
chr17	6914168	6919168	AC027763.2	-	chr17	6914367	6914773
chr17	6914314	6919314	C17orf49	+	chr17	6914367	6914773
chr17	7344880	7349880	CHRNB1	+	chr17	7345225	7346080
chr17	7344880	7349880	CHRNB1	+	chr17	7344557	7344883
chr17	7479285	7484285	CD68	+	chr17	7482551	7483017
chr17	7491988	7496988	SOX15	-	chr17	7493495	7493873
chr17	7491988	7496988	SOX15	-	chr17	7492589	7493014
chr17	7617172	7622172	DNAH2	+	chr17	7616885	7617346
chr17	7754883	7759883	TMEM88	+	chr17	7758858	7759259
chr17	7754883	7759883	TMEM88	+	chr17	7757275	7757649
chr17	7757564	7762564	CYB5D1	+	chr17	7758858	7759259
chr17	7757564	7762564	CYB5D1	+	chr17	7757275	7757649
chr17	7902412	7907412	GUCY2D	+	chr17	7907033	7907901
chr17	8867529	8872529	PIK3R5	-	chr17	8869104	8870032
chr17	9544515	9549515	USP43	+	chr17	9544319	9544629
chr17	10045331	10050331	AC000003.2	+	chr17	10046099	10046532
chr17	10420360	10425360	MYH1	-	chr17	10423131	10423479
chr17	12920004	12925004	ELAC2	-	chr17	12922123	12922452
chr17	13503744	13508744	HS3ST3A1	-	chr17	13504453	13504891
chr17	15521326	15526326	CDRT1	-	chr17	15522241	15523075
chr17	15521326	15526326	CDRT1	-	chr17	15523509	15524076
chr17	16873932	16878932	TNFRSF13B	-	chr17	16878023	16878444
chr17	17738825	17743825	SREBF1	-	chr17	17743096	17743495
chr17	18160348	18165348	MIEF2	+	chr17	18165238	18165572
chr17	18160730	18165730	FLII	-	chr17	18165238	18165572

chr17	18376551	18381551	LGALS9C	+	chr17	18379734	18380080
chr17	19027282	19032282	GRAPL	+	chr17	19031263	19031620
chr17	19395198	19400198	SLC47A1	+	chr17	19395733	19396134
chr17	19769749	19774749	ULK2	-	chr17	19772169	19772509
chr17	20945573	20950573	USP22	-	chr17	20950017	20950439
chr17	20945573	20950573	USP22	-	chr17	20945389	20946031
chr17	21023177	21028177	DHRS7B	+	chr17	21025774	21026296
chr17	21155222	21160222	C17orf103	-	chr17	21160003	21160331
chr17	21184484	21189484	MAP2K3	+	chr17	21188519	21188911
chr17	21276009	21281009	KCNJ12	+	chr17	21275276	21276037
chr17	21476222	21481222	C17orf51	-	chr17	21476848	21477341
chr17	22018937	22023937	MTRNR2L1	+	chr17	22020540	22020919
chr17	25953324	25958324	LGALS9	+	chr17	25958018	25958875
chr17	25953324	25958324	LGALS9	+	chr17	25955181	25955516
chr17	26126025	26131025	NOS2	-	chr17	26127173	26127594
chr17	26218891	26223891	RP1-66C13.4	-	chr17	26220392	26220833
chr17	26220278	26225278	LYRM9	-	chr17	26220392	26220833
chr17	26987707	26992707	SDF2	-	chr17	26992362	26992760
chr17	27049415	27054415	NEK8	+	chr17	27051538	27051874
chr17	27331958	27336958	SEZ6	-	chr17	27332885	27333454
chr17	27417037	27422037	TIAF1	-	chr17	27419144	27420000
chr17	27417037	27422037	TIAF1	-	chr17	27421587	27422081
chr17	27619636	27624636	NUFIP2	-	chr17	27620734	27621245
chr17	29150294	29155294	CRLF3	-	chr17	29150318	29150625
chr17	29229862	29234862	ADAP2	+	chr17	29233334	29233999
chr17	29715142	29720142	RAB11FIP4	+	chr17	29715815	29716277
chr17	32688750	32693750	CCL1	-	chr17	32688620	32688970
chr17	32904268	32909268	TMEM132E	+	chr17	32907765	32908198
chr17	33901565	33906565	AP2B1	+	chr17	33903927	33904359
chr17	34307032	34312032	CCL16	-	chr17	34310199	34310562
chr17	34307192	34312192	CTB-186H2.3	+	chr17	34310199	34310562
chr17	36760683	36765683	SRCIN1	-	chr17	36764273	36764578

chr17	36902113	36907113	CTB-58E17.5	+	chr17	36904998	36905366
chr17	36904570	36909570	PCGF2	-	chr17	36904998	36905366
chr17	37779493	37784493	PPP1R1B	+	chr17	37779600	37779929
chr17	37789818	37794818	STARD3	+	chr17	37794137	37794534
chr17	37885540	37890540	MIEN1	-	chr17	37888968	37889384
chr17	37890680	37895680	GRB7	+	chr17	37893771	37894571
chr17	37890680	37895680	GRB7	+	chr17	37892575	37892890
chr17	38082354	38087354	ORMDL3	-	chr17	38083396	38083884
chr17	38211043	38216043	THRA	+	chr17	38211694	38212161
chr17	39141887	39146887	KRT40	-	chr17	39145684	39146086
chr17	39379400	39384400	KRTAP9-2	+	chr17	39380485	39380801
chr17	39524552	39529552	KRT33B	-	chr17	39526329	39526763
chr17	39552344	39557344	KRT31	-	chr17	39555926	39556392
chr17	39595673	39600673	KRT38	-	chr17	39595993	39596401
chr17	39635892	39640892	KRT35	-	chr17	39635561	39635964
chr17	39677281	39682281	KRT15	-	chr17	39678277	39678696
chr17	39779594	39784594	KRT17	-	chr17	39783130	39783506
chr17	39941683	39946683	JUP	-	chr17	39941347	39942031
chr17	40159900	40164900	NKIRAS2	+	chr17	40161301	40161607
chr17	40263251	40268251	DHX58	-	chr17	40265691	40266190
chr17	40436065	40441065	STAT5A	+	chr17	40437779	40438310
chr17	40539086	40544086	STAT3	-	chr17	40543938	40544330
chr17	40715586	40720586	MLX	+	chr17	40719417	40719791
chr17	41115015	41120015	AARSD1	-	chr17	41116062	41116397
chr17	41155242	41160242	IFI35	+	chr17	41160161	41160957
chr17	41472827	41477827	ARL4D	+	chr17	41477001	41477604
chr17	41737822	41742822	MEOX1	-	chr17	41742519	41742827
chr17	41834656	41839656	SOST	-	chr17	41838888	41839224
chr17	41854303	41859303	C17orf105	+	chr17	41856074	41856397
chr17	41854303	41859303	C17orf105	+	chr17	41858048	41858750
chr17	41854856	41859856	DUSP3	-	chr17	41856074	41856397
chr17	41854856	41859856	DUSP3	-	chr17	41858048	41858750

chr17	42439743	42444743	FAM171A2	-	chr17	42443723	42444154
chr17	42439743	42444743	FAM171A2	-	chr17	42440348	42440698
chr17	42631425	42636425	FZD2	+	chr17	42632358	42633017
chr17	42730262	42735262	C17orf104	+	chr17	42733241	42733757
chr17	42832899	42837899	ADAM11	+	chr17	42833474	42833986
chr17	43043939	43048939	C1QL1	-	chr17	43048543	43049054
chr17	43295311	43300311	FMNL1	+	chr17	43295101	43295479
chr17	43968248	43973248	MAPT	+	chr17	43969449	43969815
chr17	44907067	44912067	WNT9B	+	chr17	44906872	44907222
chr17	45327712	45332712	ITGB3	+	chr17	45329446	45329752
chr17	45327763	45332763	ITGB3	+	chr17	45329446	45329752
chr17	46015372	46020372	PNPO	+	chr17	46016124	46016466
chr17	46669823	46674823	HOXB5	-	chr17	46671692	46672187
chr17	46669823	46674823	HOXB5	-	chr17	46673177	46673688
chr17	46702339	46707339	HOXB9	-	chr17	46704440	46705035
chr17	46982231	46987231	UBE2Z	+	chr17	46984665	46985048
chr17	47020979	47025979	SNF8	-	chr17	47023961	47024424
chr17	47490746	47495746	PHB	-	chr17	47494267	47494627
chr17	47649720	47654720	NXPH3	+	chr17	47653798	47654107
chr17	48277493	48282493	COL1A1	-	chr17	48277467	48277796
chr17	48616919	48621919	SPATA20	+	chr17	48618471	48619003
chr17	48943839	48948839	TOB1	-	chr17	48947631	48947992
chr17	50935981	50940981	AC102948.2	+	chr17	50935653	50935991
chr17	54907960	54912960	DGKE	+	chr17	54907297	54908016
chr17	54914634	54919634	C17orf67	-	chr17	54915298	54915647
chr17	54989899	54994899	TRIM25	-	chr17	54990387	54990700
chr17	55036915	55041915	COIL	-	chr17	55037725	55038401
chr17	56829730	56834730	PPM1E	+	chr17	56832507	56832848
chr17	58498331	58503331	USP32	-	chr17	58500912	58501542
chr17	58498331	58503331	USP32	-	chr17	58498384	58498812
chr17	58602080	58607080	APPBP2	-	chr17	58603063	58604106
chr17	60884205	60889205	MARCH10	-	chr17	60885129	60885690

chr17	61522239	61527239	CYB561	-	chr17	61523389	61523708
chr17	61550922	61555922	ACE	+	chr17	61554882	61555274
chr17	61597195	61602195	KCNH6	+	chr17	61600687	61601019
chr17	61901012	61906012	PSMC5	+	chr17	61901384	61902082
chr17	62072211	62077211	C17orf72	+	chr17	62076801	62077133
chr17	62491654	62496654	POLG2	-	chr17	62493174	62493785
chr17	62499206	62504206	CEP95	+	chr17	62501524	62502108
chr17	64957526	64962526	CACNG4	+	chr17	64961643	64961988
chr17	65024009	65029009	AC005544.1	+	chr17	65025184	65025534
chr17	65037206	65042206	CACNG1	+	chr17	65039856	65040223
chr17	66504421	66509421	PRKAR1A	+	chr17	66505060	66505418
chr17	71306814	71311814	CDC42EP4	-	chr17	71311598	71312158
chr17	71638728	71643728	SDK2	-	chr17	71640379	71640899
chr17	72346771	72351771	AC103809.2	-	chr17	72349317	72349669
chr17	72417490	72422490	GPRC5C	+	chr17	72418129	72418479
chr17	72459055	72464055	CD300A	+	chr17	72463056	72463425
chr17	72577318	72582318	C17orf77	+	chr17	72579252	72579634
chr17	72707617	72712617	CD300LF	-	chr17	72710724	72711297
chr17	72769122	72774122	TMEM104	+	chr17	72768942	72769285
chr17	73162876	73167876	HN1	-	chr17	73166506	73166976
chr17	73198254	73203254	NUP85	+	chr17	73198009	73198405
chr17	73254255	73259255	MRPS7	+	chr17	73257410	73257952
chr17	73256944	73261944	GGA3	-	chr17	73257410	73257952
chr17	73508641	73513641	TSEN54	+	chr17	73509840	73510163
chr17	73508641	73513641	TSEN54	+	chr17	73508688	73509329
chr17	73517661	73522661	LLGL2	+	chr17	73518285	73518682
chr17	73517661	73522661	LLGL2	+	chr17	73520525	73521010
chr17	73780474	73785474	H3F3B	-	chr17	73785270	73785694
chr17	73851088	73856088	WBP2	-	chr17	73854667	73855107
chr17	73993487	73998487	CDK3	+	chr17	73994556	73995003
chr17	74520168	74525168	PRCD	+	chr17	74521121	74521482
chr17	74581538	74586538	ST6GALNAC2	-	chr17	74583543	74583916

chr17	74721366	74726366	JMJD6	-	chr17	74724895	74725242
chr17	75079298	75084298	SEC14L1	+	chr17	75084246	75085164
chr17	76138934	76143934	C17orf99	+	chr17	76143510	76143992
chr17	76219240	76224240	AC087645.1	-	chr17	76221375	76221720
chr17	76223891	76228891	TMEM235	+	chr17	76225977	76226404
chr17	76354658	76359658	SOCS3	-	chr17	76357817	76358172
chr17	76371221	76376221	PGS1	+	chr17	76375884	76376269
chr17	77004449	77009449	CANT1	-	chr17	77009240	77009748
chr17	77701181	77706181	ENPP7	+	chr17	77702586	77702891
chr17	78973635	78978635	AC127496.1	+	chr17	78975986	78976948
chr17	79489373	79494373	ACTG1	-	chr17	79489805	79490118
chr17	79649454	79654454	ARL16	-	chr17	79651911	79652281
chr17	79789678	79794678	FAM195B	-	chr17	79791202	79791652
chr17	79791426	79796426	PPP1R27	-	chr17	79791202	79791652
chr17	79827782	79832782	ARHGDIA	-	chr17	79829252	79829606
chr17	79877699	79882699	SIRT7	-	chr17	79878558	79878864
chr17	79903609	79908609	MYADML2	-	chr17	79907425	79907772
chr17	80005067	80010067	GPS1	+	chr17	80007373	80007923
chr17	80008207	80013207	RFNG	-	chr17	80012240	80012775
chr17	80169206	80174206	CCDC57	-	chr17	80170160	80170583
chr18	266550	271550	THOC1	-	chr18	269277	269743
chr18	901444	906444	ADCYAP1	+	chr18	903608	904006
chr18	2570008	2575008	METTL4	-	chr18	2573148	2573557
chr18	8335538	8340538	AP001094.1	-	chr18	8339917	8340378
chr18	8605943	8610943	RAB12	+	chr18	8609314	8610321
chr18	11147087	11152087	PIEZO2	-	chr18	11148215	11148638
chr18	11907722	11912722	MPPE1	-	chr18	11907365	11907735
chr18	12090352	12095352	ANKRD62	+	chr18	12092472	12092847
chr18	12250818	12255818	CIDEA	+	chr18	12254516	12255167
chr18	12304168	12309168	TUBB6	+	chr18	12307251	12307919
chr18	12375813	12380813	AFG3L2	-	chr18	12378106	12378505
chr18	19745904	19750904	GATA6	+	chr18	19746028	19746338

chr18	21029287	21034287	RIOK3	+	chr18	21030328	21031081
chr18	25755910	25760910	CDH2	-	chr18	25757493	25757827
chr18	29168189	29173189	TTR	+	chr18	29170713	29171310
chr18	29339343	29344343	SLC25A52	-	chr18	29343576	29343885
chr18	29531599	29536599	TRAPPC8	-	chr18	29531784	29532163
chr18	30048947	30053947	GAREM	-	chr18	30052061	30052438
chr18	43650738	43655738	PSTPIP2	-	chr18	43650508	43650901
chr18	44235496	44240496	LOXHD1	-	chr18	44235612	44236126
chr18	44701152	44706152	IER3IP1	-	chr18	44702405	44702820
chr18	44701152	44706152	IER3IP1	-	chr18	44703626	44703994
chr18	44701245	44706245	IER3IP1	-	chr18	44702405	44702820
chr18	44701245	44706245	IER3IP1	-	chr18	44703626	44703994
chr18	47362493	47367493	RP11-886H22.1	-	chr18	47364430	47364830
chr18	47791392	47796392	CCDC11	-	chr18	47794641	47794973
chr18	48490861	48495861	ELAC1	+	chr18	48495624	48495933
chr18	48490889	48495889	RP11-729L2.2	+	chr18	48495624	48495933
chr18	48490910	48495910	SMAD4	+	chr18	48495624	48495933
chr18	54315074	54320074	WDR7	+	chr18	54319951	54320323
chr18	54317331	54322331	TXNL1	-	chr18	54319951	54320323
chr18	56939818	56944818	RAX	-	chr18	56941142	56941631
chr18	61033243	61038243	KDSR	-	chr18	61033130	61033544
chr18	61088216	61093216	VPS4B	-	chr18	61091192	61091499
chr18	61608101	61613101	AC009802.1	-	chr18	61611070	61611415
chr18	63413988	63418988	CDH7	+	chr18	63418068	63418661
chr18	67952637	67957637	SOCS6	+	chr18	67956132	67956463
chr18	71957751	71962751	CYB5A	-	chr18	71962100	71962485
chr18	74532751	74537751	RP11-162A12.2	-	chr18	74537386	74537762
chr18	77436301	77441301	CTDP1	+	chr18	77439170	77439698
chr18	77710164	77715164	PQLC1	-	chr18	77711143	77711507
chr18	78003929	78008929	PARD6G	-	chr18	78004209	78004621
chr19	847514	852514	ELANE	+	chr19	851236	851671
chr19	913787	918787	KISS1R	+	chr19	916928	917425

chr19	1245052	1250052	MIDN	+	chr19	1249371	1249693
chr19	1512103	1517103	ADAMTSL5	-	chr19	1516847	1517339
chr19	1748872	1753872	ONECUT3	+	chr19	1749346	1750220
chr19	1810775	1815775	ATP8B3	-	chr19	1815476	1815811
chr19	1846952	1851952	REXO1	-	chr19	1848301	1848756
chr19	1862067	1867067	KLF16	-	chr19	1865135	1865501
chr19	2033380	2038380	BTBD2	-	chr19	2036222	2036544
chr19	2095172	2100172	MOB3A	-	chr19	2098593	2098979
chr19	2426392	2431392	TIMM13	-	chr19	2426476	2426785
chr19	2943469	2948469	ZNF77	-	chr19	2944723	2945307
chr19	2973944	2978944	TLE6	+	chr19	2975963	2976413
chr19	3356061	3361061	NFIC	+	chr19	3357936	3358317
chr19	3800627	3805627	MATK	-	chr19	3802428	3802750
chr19	3929601	3934601	NMRK2	+	chr19	3930881	3931206
chr19	4041654	4046654	AC016586.1	-	chr19	4041829	4042333
chr19	4275098	4280098	SHD	+	chr19	4278119	4278451
chr19	4533736	4538736	PLIN5	-	chr19	4538622	4538959
chr19	4538579	4543579	CTB-50L17.14	-	chr19	4538622	4538959
chr19	5451926	5456926	ZNRF4	+	chr19	5454391	5455114
chr19	5717188	5722188	CATSPERD	+	chr19	5717970	5718382
chr19	5849985	5854985	FUT3	-	chr19	5853778	5854822
chr19	6131758	6136758	ACSBG2	+	chr19	6136718	6137370
chr19	6766099	6771099	SH2D3A	-	chr19	6766795	6767150
chr19	7057151	7062151	MBD3L3	-	chr19	7058173	7058483
chr19	7738014	7743014	C19orf59	+	chr19	7742308	7742670
chr19	7739887	7744887	CTD-3214H19.16	+	chr19	7742308	7742670
chr19	7765532	7770532	FCER2	-	chr19	7766163	7766536
chr19	7965228	7970228	MAP2K7	+	chr19	7968243	7968660
chr19	7966927	7971927	AC010336.1	-	chr19	7968243	7968660
chr19	8424673	8429673	ANGPTL4	+	chr19	8427948	8428304
chr19	8451365	8456365	RAB11B	+	chr19	8453028	8453393
chr19	8837816	8842816	OR2Z1	+	chr19	8839711	8840084

chr19	8939574	8944574	MBD3L1	+	chr19	8943562	8943933
chr19	8941504	8946504	ZNF558	-	chr19	8943562	8943933
chr19	9358106	9363106	OR7E24	+	chr19	9361071	9361404
chr19	9935068	9940068	UBL5	+	chr19	9935771	9936120
chr19	10211972	10216972	ANGPTL6	-	chr19	10211987	10212470
chr19	10394143	10399143	ICAM4	+	chr19	10395220	10395795
chr19	10442816	10447816	RAVER1	-	chr19	10445074	10445854
chr19	10523949	10528949	PDE4A	+	chr19	10525453	10525899
chr19	10523949	10528949	PDE4A	+	chr19	10524629	10524955
chr19	10825255	10830255	DNM2	+	chr19	10826792	10827165
chr19	11196538	11201538	LDLR	+	chr19	11196628	11197133
chr19	11371657	11376657	DOCK6	-	chr19	11371880	11372257
chr19	11481861	11486861	SWSAP1	+	chr19	11482774	11483259
chr19	11848324	11853324	ZNF823	-	chr19	11852127	11852492
chr19	12172014	12177014	ZNF844	+	chr19	12173447	12173783
chr19	12833325	12838325	TNPO2	-	chr19	12836540	12836951
chr19	12898810	12903810	JUNB	+	chr19	12903293	12903627
chr19	12911194	12916194	PRDX2	-	chr19	12913211	12913703
chr19	12990782	12995782	DNASE2	-	chr19	12993794	12994102
chr19	13043351	13048351	FARSA	-	chr19	13045923	13046251
chr19	13045892	13050892	CALR	+	chr19	13045923	13046251
chr19	13225417	13230417	NACC1	+	chr19	13229592	13229945
chr19	13225417	13230417	NACC1	+	chr19	13228352	13229041
chr19	13226881	13231881	TRMT1	-	chr19	13229592	13229945
chr19	13226881	13231881	TRMT1	-	chr19	13228352	13229041
chr19	13257729	13262729	IER2	+	chr19	13259059	13259406
chr19	13257729	13262729	IER2	+	chr19	13259901	13260375
chr19	13259697	13264697	STX10	-	chr19	13259901	13260375
chr19	13902774	13907774	ZSWIM4	+	chr19	13905783	13906142
chr19	14135460	14140460	RLN3	+	chr19	14137161	14137484
chr19	14990764	14995764	OR7A17	-	chr19	14992215	14992546
chr19	15156695	15161695	CASP14	+	chr19	15159716	15160051

chr19	15194291	15199291	OR1I1	+	chr19	15198395	15198790
chr19	15559262	15564262	WIZ	-	chr19	15561944	15562270
chr19	15917436	15922436	OR10H1	-	chr19	15920827	15921158
chr19	15917436	15922436	OR10H1	-	chr19	15922227	15922532
chr19	16292691	16297691	FAM32A	+	chr19	16297007	16297415
chr19	16292691	16297691	FAM32A	+	chr19	16293894	16294285
chr19	16432128	16437128	KLF2	+	chr19	16434101	16434598
chr19	16681693	16686693	SLC35E1	-	chr19	16681356	16681725
chr19	18300492	18305492	MPV17L2	+	chr19	18302639	18303275
chr19	18390932	18395932	JUND	-	chr19	18393029	18393399
chr19	18547611	18552611	ISYNA1	-	chr19	18551316	18551700
chr19	18547611	18552611	ISYNA1	-	chr19	18549287	18549910
chr19	18714740	18719740	TMEM59L	+	chr19	18719498	18719811
chr19	18717051	18722051	CRLF1	-	chr19	18719498	18719811
chr19	19026984	19031984	DDX49	+	chr19	19027015	19027395
chr19	19645557	19650557	CILP2	+	chr19	19648375	19648900
chr19	19738239	19743239	LPAR2	-	chr19	19741772	19742215
chr19	19752976	19757976	GMIP	-	chr19	19754085	19754512
chr19	19752976	19757976	GMIP	-	chr19	19754820	19755286
chr19	19776105	19781105	ZNF101	+	chr19	19778524	19778861
chr19	20047139	20052139	AC007204.1	-	chr19	20051487	20051843
chr19	20955610	20960610	ZNF66	+	chr19	20955655	20956166
chr19	21538232	21543232	ZNF738	+	chr19	21541170	21541522
chr19	21538232	21543232	ZNF738	+	chr19	21540254	21540584
chr19	22713787	22718787	ZNF98	-	chr19	22715622	22716045
chr19	30013906	30018906	VSTM2B	+	chr19	30014379	30014975
chr19	33179367	33184367	NUDT19	+	chr19	33179943	33180382
chr19	33568286	33573286	GPATCH1	+	chr19	33571833	33572152
chr19	33619496	33624496	WDR88	+	chr19	33619582	33619927
chr19	34109361	34114361	CHST8	+	chr19	34110099	34110495
chr19	34891789	34896789	PDCD2L	+	chr19	34893798	34894173
chr19	35630654	35635654	FXYD7	+	chr19	35633499	35633836

chr19	35631855	35636855	LGI4	-	chr19	35633499	35633836
chr19	35642133	35647133	FXYD5	+	chr19	35644616	35645025
chr19	35642133	35647133	FXYD5	+	chr19	35646758	35647132
chr19	35779528	35784528	MAG	+	chr19	35782574	35783309
chr19	35931309	35936309	FFAR2	+	chr19	35935855	35936162
chr19	36032997	36037997	TMEM147	+	chr19	36036260	36036614
chr19	36129147	36134147	ETV2	+	chr19	36132380	36132836
chr19	36191929	36196929	ZBTB32	+	chr19	36192289	36193083
chr19	36245544	36250544	C19orf55	+	chr19	36245485	36245796
chr19	36343263	36348263	KIRREL2	+	chr19	36343999	36344479
chr19	36389882	36394882	HCST	+	chr19	36393252	36393602
chr19	36391705	36396705	NFKBID	-	chr19	36393252	36393602
chr19	36422760	36427760	LRFN3	+	chr19	36422615	36422948
chr19	36542283	36547283	WDR62	+	chr19	36541956	36542421
chr19	36626977	36631977	CAPNS1	+	chr19	36628636	36629061
chr19	36642271	36647271	COX7A1	-	chr19	36645474	36646008
chr19	36868601	36873601	ZFP14	-	chr19	36871412	36871730
chr19	37337763	37342763	ZNF345	+	chr19	37341576	37341999
chr19	37340189	37345189	ZNF790	-	chr19	37341576	37341999
chr19	39134789	39139789	ACTN4	+	chr19	39135037	39135492
chr19	39134789	39139789	ACTN4	+	chr19	39138549	39139226
chr19	39329318	39334318	AC104534.3	-	chr19	39330530	39330999
chr19	39734146	39739146	IFNL3	-	chr19	39735307	39735759
chr19	39986035	39991035	DLL3	+	chr19	39989706	39990159
chr19	39986035	39991035	DLL3	+	chr19	39990754	39991160
chr19	40335554	40340554	FBL	-	chr19	40336831	40337163
chr19	40789943	40794943	AKT2	-	chr19	40790336	40790658
chr19	40930432	40935432	SERTAD1	-	chr19	40932287	40932640
chr19	41095289	41100289	LTBP4	+	chr19	41097012	41097358
chr19	41195377	41200377	NUMBL	-	chr19	41198304	41198923
chr19	41721608	41726608	AXL	+	chr19	41724367	41724855
chr19	41858316	41863316	TGFB1	-	chr19	41859181	41859512

chr19	41880715	41885715	BCKDHA	+	chr19	41882167	41882530
chr19	42377690	42382690	CD79A	+	chr19	42379364	42379670
chr19	42462042	42467042	RABAC1	-	chr19	42466453	42466828
chr19	42757784	42762784	AC006486.9	-	chr19	42760760	42761314
chr19	42757809	42762809	ERF	-	chr19	42760760	42761314
chr19	42826261	42831261	MEGF8	+	chr19	42828445	42828789
chr19	42945700	42950700	CXCL17	-	chr19	42945977	42946614
chr19	44026149	44031149	ZNF575	+	chr19	44025608	44026276
chr19	44077452	44082452	PINLYP	+	chr19	44079385	44080137
chr19	44083125	44088125	XRCC1	-	chr19	44087543	44087937
chr19	44122526	44127526	ZNF428	-	chr19	44124120	44124450
chr19	44142491	44147491	CADM4	-	chr19	44144147	44144476
chr19	44173199	44178199	PLAUR	-	chr19	44176248	44176825
chr19	44451875	44456875	ZNF221	+	chr19	44452848	44453162
chr19	44594992	44599992	ZNF224	+	chr19	44597973	44598405
chr19	44750818	44755818	ZNF233	+	chr19	44751452	44751774
chr19	44904274	44909274	CTC-512J12.6	-	chr19	44905194	44905790
chr19	44904274	44909274	ZNF285	-	chr19	44905194	44905790
chr19	45143598	45148598	PVR	+	chr19	45145494	45145842
chr19	45277626	45282626	CBLC	+	chr19	45277585	45277892
chr19	45454342	45459342	CLPTM1	+	chr19	45458658	45459402
chr19	45455764	45460764	CTB-129P6.11	-	chr19	45458658	45459402
chr19	45538798	45543798	CLASRP	+	chr19	45540956	45541364
chr19	45578953	45583953	GEMIN7	+	chr19	45583423	45583769
chr19	45579046	45584046	MARK4	+	chr19	45583423	45583769
chr19	45591154	45596154	PPP1R37	+	chr19	45590910	45591343
chr19	45678503	45683503	BLOC1S3	+	chr19	45681330	45681642
chr19	45679580	45684580	AC005779.2	+	chr19	45681330	45681642
chr19	45679995	45684995	TRAPPC6A	-	chr19	45681330	45681642
chr19	45687146	45692146	AC006126.3	+	chr19	45688810	45689195
chr19	46270984	46275984	SIX5	-	chr19	46273878	46274187
chr19	46284310	46289310	DMPK	-	chr19	46285339	46285946

chr19	46404362	46409362	MYPOP	-	chr19	46404195	46404519
chr19	46416536	46421536	NANOS2	-	chr19	46418749	46419173
chr19	46796803	46801803	HIF3A	+	chr19	46801725	46802322
chr19	46973320	46978320	PNMAL1	-	chr19	46974447	46974753
chr19	47100831	47105831	CALM3	+	chr19	47104314	47104777
chr19	47102618	47107618	AC011551.3	-	chr19	47104314	47104777
chr19	47102957	47107957	PPP5D1	-	chr19	47104314	47104777
chr19	47831904	47836904	C5AR2	+	chr19	47832258	47832619
chr19	48213100	48218100	EHD2	+	chr19	48216832	48217165
chr19	48213100	48218100	EHD2	+	chr19	48213623	48214011
chr19	48821266	48826266	EMP3	+	chr19	48823319	48823832
chr19	48823651	48828651	CCDC114	-	chr19	48823319	48823832
chr19	49139195	49144195	DBP	-	chr19	49143764	49144172
chr19	49195728	49200728	FUT2	+	chr19	49196176	49196580
chr19	49242478	49247478	RASIP1	-	chr19	49245747	49246189
chr19	49433439	49438439	DHDH	+	chr19	49435248	49435618
chr19	49454572	49459572	BAX	+	chr19	49459373	49459849
chr19	49923506	49928506	CTD-3148I10.1	+	chr19	49924578	49924944
chr19	50829410	50834410	NR1H2	+	chr19	50832242	50832677
chr19	51069802	51074802	LRRC4B	-	chr19	51071889	51072224
chr19	51270221	51275221	GPR32	+	chr19	51273092	51273732
chr19	51511337	51516337	KLK9	-	chr19	51513355	51513691
chr19	51511390	51516390	KLK9	-	chr19	51513355	51513691
chr19	51811602	51816602	IGLON5	+	chr19	51812556	51813155
chr19	51811602	51816602	IGLON5	+	chr19	51815753	51816073
chr19	51960210	51965210	SIGLEC8	-	chr19	51960358	51960751
chr19	52294916	52299916	FPR3	+	chr19	52295187	52295616
chr19	52531993	52536993	ZNF614	-	chr19	52534965	52535369
chr19	52835998	52840998	ZNF610	+	chr19	52835859	52836200
chr19	53027405	53032405	ZNF808	+	chr19	53027331	53027736
chr19	53236826	53241826	ZNF611	-	chr19	53239903	53240337
chr19	53695139	53700139	ZNF665	-	chr19	53697812	53698249

chr19	54105251	54110251	CTB-167G5.5	-	chr19	54106715	54107023
chr19	54492042	54497042	CACNG6	+	chr19	54494682	54495041
chr19	54615337	54620337	PRPF31	+	chr19	54619378	54619805
chr19	54617555	54622555	TFPT	-	chr19	54619378	54619805
chr19	54783539	54788539	LILRB2	-	chr19	54786110	54786495
chr19	54922893	54927893	TTYH1	+	chr19	54924800	54925149
chr19	54973394	54978394	LENG9	-	chr19	54974958	54975303
chr19	55080887	55085887	LILRA2	+	chr19	55083702	55084090
chr19	55081846	55086846	LILRB1	+	chr19	55083702	55084090
chr19	55579888	55584888	EPS8L1	+	chr19	55581534	55581890
chr19	55581159	55586159	RDH13	-	chr19	55581534	55581890
chr19	55659222	55664222	TNNT1	-	chr19	55658936	55659259
chr19	55719374	55724374	PTPRH	-	chr19	55722126	55722712
chr19	55864682	55869682	COX6B2	-	chr19	55868522	55868894
chr19	55873128	55878128	FAM71E2	-	chr19	55875122	55875549
chr19	55880331	55885331	IL11	-	chr19	55884711	55885077
chr19	55884704	55889704	TMEM190	+	chr19	55884711	55885077
chr19	56107888	56112888	ZNF524	+	chr19	56110806	56111557
chr19	56277007	56282007	RFPL4AL1	+	chr19	56276834	56277193
chr19	56631149	56636149	ZNF787	-	chr19	56631144	56631496
chr19	56640468	56645468	ZNF444	+	chr19	56644145	56644467
chr19	56683889	56688889	GALP	+	chr19	56685613	56686094
chr19	57015712	57020712	ZNF471	+	chr19	57017940	57018329
chr19	57103132	57108132	ZNF71	+	chr19	57107058	57107378
chr19	57350596	57355596	PEG3	-	chr19	57350789	57351178
chr19	57350597	57355597	ZIM2	-	chr19	57350789	57351178
chr19	57787919	57792919	ZNF460	+	chr19	57791830	57792165
chr19	58007783	58012783	ZNF773	+	chr19	58012759	58013114
chr19	58107753	58112753	ZNF530	+	chr19	58110440	58110802
chr19	58122101	58127101	ZNF134	+	chr19	58122926	58123489
chr19	58277523	58282523	ZNF586	+	chr19	58281526	58281847
chr19	58277523	58282523	ZNF586	+	chr19	58279889	58280560

chr19	58513217	58518217	ZNF606	-	chr19	58515177	58515483
chr19	58541900	58546900	ZSCAN1	+	chr19	58545813	58546207
chr19	58974923	58979923	ZNF324	+	chr19	58975688	58976082
chr19	59083442	59088442	MZF1	-	chr19	59083724	59084161
chr2	675939	680939	TMEM18	-	chr2	678794	679173
chr2	1374495	1379495	TPO	+	chr2	1376911	1377353
chr2	2333532	2338532	MYT1L	-	chr2	2335771	2336224
chr2	3594047	3599047	RP13-512J5.1	-	chr2	3598022	3598367
chr2	3638926	3643926	COLEC11	+	chr2	3638899	3639300
chr2	3702285	3707285	ALLC	+	chr2	3707071	3707568
chr2	10179476	10184476	KLF11	+	chr2	10183198	10184001
chr2	11269679	11274679	C2orf50	+	chr2	11273145	11273516
chr2	11269679	11274679	C2orf50	+	chr2	11271309	11271791
chr2	11600427	11605427	AC099344.1	+	chr2	11604465	11605068
chr2	11604797	11609797	E2F6	-	chr2	11609073	11609384
chr2	11604797	11609797	E2F6	-	chr2	11604465	11605068
chr2	11814221	11819221	LPIN1	+	chr2	11815994	11816553
chr2	15827406	15832406	AC008271.1	+	chr2	15830650	15831011
chr2	17931625	17936625	GEN1	+	chr2	17933423	17933812
chr2	18769330	18774330	NT5C1B-RDH14	-	chr2	18772683	18773129
chr2	18769338	18774338	NT5C1B	-	chr2	18772683	18773129
chr2	20423694	20428694	SDC1	-	chr2	20425215	20425858
chr2	23745714	23750714	AC011239.1	-	chr2	23750273	23750766
chr2	24297813	24302813	SF3B14	-	chr2	24302497	24302993
chr2	24582083	24587083	ITSN2	-	chr2	24582761	24583103
chr2	25390272	25395272	POMC	-	chr2	25392692	25393149
chr2	25563959	25568959	DNMT3A	-	chr2	25564141	25564700
chr2	26253476	26258476	RAB10	+	chr2	26256397	26256721
chr2	26466094	26471094	HADHA	-	chr2	26468821	26469374
chr2	26983652	26988652	SLC35F6	+	chr2	26987189	26987515
chr2	26983657	26988657	CENPA	+	chr2	26987189	26987515
chr2	27306115	27311115	KHK	+	chr2	27306219	27306566

chr2	27360778	27365778	C2orf53	-	chr2	27363740	27364090
chr2	27497185	27502185	SLC30A3	-	chr2	27500692	27501133
chr2	27529813	27534813	UCN	-	chr2	27532065	27532536
chr2	27647157	27652157	NRBP1	+	chr2	27651523	27652228
chr2	27802397	27807397	ZNF512	+	chr2	27802836	27803241
chr2	27885176	27890176	SUPT7L	-	chr2	27889996	27890389
chr2	28007686	28012686	AC110084.1	-	chr2	28012391	28012749
chr2	28676512	28681512	PLB1	+	chr2	28679323	28679639
chr2	36579569	36584569	CRIM1	+	chr2	36583200	36583718
chr2	36777911	36782911	AC007401.2	-	chr2	36777838	36778448
chr2	37568217	37573217	QPCT	+	chr2	37571823	37572294
chr2	38975176	38980176	GEMIN6	+	chr2	38978710	38979106
chr2	38977136	38982136	SRSF7	-	chr2	38978710	38979106
chr2	39349986	39354986	SOS1	-	chr2	39352184	39353011
chr2	39662953	39667953	MAP4K3	-	chr2	39664006	39664342
chr2	39888622	39893622	TMEM178A	+	chr2	39892756	39893174
chr2	44062603	44067603	ABCG8	+	chr2	44062701	44063473
chr2	47167494	47172494	MCFD2	-	chr2	47171404	47171804
chr2	47167494	47172494	MCFD2	-	chr2	47169236	47169603
chr2	48538276	48543276	FOXN2	+	chr2	48541989	48542349
chr2	48981380	48986380	LHCGR	-	chr2	48983228	48983533
chr2	54481909	54486909	TSPYL6	-	chr2	54482375	54482815
chr2	55458199	55463199	CLHC1	-	chr2	55460848	55461204
chr2	56407758	56412758	CCDC85A	+	chr2	56412722	56413029
chr2	61243894	61248894	PUS10	-	chr2	61248662	61249115
chr2	65212111	65217111	SLC1A4	+	chr2	65215231	65215641
chr2	68381476	68386476	PNO1	+	chr2	68385027	68385340
chr2	68383192	68388192	WDR92	-	chr2	68385027	68385340
chr2	69868057	69873057	ANXA4	+	chr2	69872786	69873384
chr2	71061452	71066452	CD207	-	chr2	71064457	71064804
chr2	71452713	71457713	PAIP2B	-	chr2	71454420	71454761
chr2	71500191	71505191	ZNF638	+	chr2	71500812	71501275

chr2	72373667	72378667	CYP26B1	-	chr2	72376594	72376902
chr2	73437850	73442850	SMYD5	+	chr2	73441563	73442225
chr2	74668049	74673049	RTKN	-	chr2	74670909	74671245
chr2	74681956	74686956	WBP1	+	chr2	74684217	74684561
chr2	74706700	74711700	TTC31	+	chr2	74706607	74707009
chr2	75870409	75875409	MRPL19	+	chr2	75871115	75871473
chr2	79313645	79318645	REG1B	-	chr2	79315845	79316190
chr2	84685669	84690669	SUCLG1	-	chr2	84685861	84686277
chr2	85819348	85824348	RNF181	+	chr2	85822682	85823061
chr2	85826479	85831479	USP39	+	chr2	85828659	85828980
chr2	85828819	85833819	TMEM150A	-	chr2	85828659	85828980
chr2	86114637	86119637	ST3GAL5	-	chr2	86114696	86115016
chr2	86421393	86426393	IMMT	-	chr2	86421702	86422150
chr2	87034019	87039019	CD8A	-	chr2	87035278	87035891
chr2	90454701	90459701	CH17-132F21.1	+	chr2	90456817	90457164
chr2	95813679	95818679	MRPS5	-	chr2	95813483	95813824
chr2	96998025	97003025	NCAPH	+	chr2	97002316	97002712
chr2	97478482	97483482	CNNM3	+	chr2	97481650	97482061
chr2	99919705	99924705	LYG1	-	chr2	99924696	99925048
chr2	99950316	99955316	EIF5B	+	chr2	99950467	99951013
chr2	100104997	100109997	REV1	-	chr2	100106199	100106512
chr2	102002557	102007557	CREG2	-	chr2	102004197	102004673
chr2	102309812	102314812	MAP4K4	+	chr2	102313345	102313706
chr2	102604806	102609806	IL1R2	+	chr2	102606915	102607847
chr2	105468469	105473469	POU3F3	+	chr2	105473193	105473578
chr2	105468469	105473469	POU3F3	+	chr2	105472148	105472659
chr2	105881085	105886085	AC012360.2	-	chr2	105883003	105883312
chr2	109604328	109609328	EDAR	-	chr2	109605132	109605797
chr2	109742304	109747304	SH3RF3	+	chr2	109742668	109742993
chr2	113029671	113034671	ZC3H6	+	chr2	113031414	113031761
chr2	113029671	113034671	ZC3H6	+	chr2	113032954	113033474
chr2	113295992	113300992	POLR1B	+	chr2	113299471	113300159

chr2	113667048	113672048	IL37	+	chr2	113668073	113668520
chr2	120120997	120125997	DBI	+	chr2	120125651	120126050
chr2	120122904	120127904	C2orf76	-	chr2	120125651	120126050
chr2	120767081	120772081	EPB41L5	+	chr2	120771310	120771623
chr2	121489699	121494699	GLI2	+	chr2	121494262	121494794
chr2	121489699	121494699	GLI2	+	chr2	121492941	121493293
chr2	122492999	122497999	MKI67IP	-	chr2	122497451	122497837
chr2	127976154	127981154	CYP27C1	-	chr2	127977785	127978090
chr2	128172503	128177503	PROC	+	chr2	128173561	128173976
chr2	128399939	128404939	GPR17	+	chr2	128404351	128404684
chr2	130938823	130943823	SMPD4	-	chr2	130942185	130942510
chr2	131365554	131370554	POTEJ	+	chr2	131367839	131368348
chr2	131858920	131863920	PLEKHB2	+	chr2	131859287	131859687
chr2	132040830	132045830	CYP4F31P	+	chr2	132041137	132041865
chr2	132557734	132562734	C2orf27B	-	chr2	132560579	132560929
chr2	134874054	134879054	MGAT5	+	chr2	134878910	134879225
chr2	135803538	135808538	MAP3K19	-	chr2	135806261	135806682
chr2	135806335	135811335	RAB3GAP1	+	chr2	135809677	135809983
chr2	135806335	135811335	RAB3GAP1	+	chr2	135810855	135811455
chr2	135806335	135811335	RAB3GAP1	+	chr2	135806261	135806682
chr2	135806335	135811335	RAB3GAP1	+	chr2	135809034	135809349
chr2	136285525	136290525	R3HDM1	+	chr2	136288732	136289444
chr2	136287306	136292306	ZRANB3	-	chr2	136288732	136289444
chr2	148598586	148603586	ACVR2A	+	chr2	148601369	148601807
chr2	150182999	150187999	LYPD6	+	chr2	150187301	150187832
chr2	153570907	153575907	ARL6IP6	+	chr2	153574394	153574717
chr2	153573011	153578011	PRPF40A	-	chr2	153574394	153574717
chr2	158110610	158115610	GALNT5	+	chr2	158110887	158111210
chr2	163007414	163012414	GCG	-	chr2	163012391	163013037
chr2	163693740	163698740	KCNH7	-	chr2	163695177	163695492
chr2	166092412	166097412	SCN2A	+	chr2	166092525	166092933
chr2	171568361	171573361	SP5	+	chr2	171570240	171570555

chr2	171569577	171574577	AC007405.2	-	chr2	171570240	171570555
chr2	172086324	172091324	TLK1	-	chr2	172087088	172087458
chr2	172860990	172865990	METAP1D	+	chr2	172865246	172865767
chr2	172863266	172868266	SLC25A12	-	chr2	172865246	172865767
chr2	172966128	172971128	DLX2	-	chr2	172969633	172969972
chr2	175111926	175116926	OLA1	-	chr2	175112598	175112906
chr2	175350322	175355322	GPR155	-	chr2	175350782	175351136
chr2	175627700	175632700	CHRNA1	-	chr2	175628695	175629000
chr2	176960958	176965958	HOXD12	+	chr2	176963156	176963626
chr2	176990922	176995922	HOXD8	+	chr2	176994338	176994726
chr2	177012450	177017450	HOXD4	+	chr2	177016174	177016525
chr2	178255925	178260925	NFE2L2	-	chr2	178257463	178257831
chr2	180724732	180729732	ZNF385B	-	chr2	180726838	180727160
chr2	186599855	186604855	FSIP2	+	chr2	186602950	186603569
chr2	187347383	187352383	ZC3H15	+	chr2	187351087	187351400
chr2	189152896	189157896	GULP1	+	chr2	189153430	189153856
chr2	196932036	196937036	DNAH7	-	chr2	196936972	196937504
chr2	198554330	198559330	AC011997.1	+	chr2	198557765	198558239
chr2	200334489	200339489	SATB2	-	chr2	200339474	200339837
chr2	200334489	200339489	SATB2	-	chr2	200334242	200334575
chr2	201371231	201376231	SGOL2	+	chr2	201374270	201374987
chr2	201671817	201676817	BZW1	+	chr2	201676764	201677331
chr2	201727922	201732922	CLK1	-	chr2	201728800	201729114
chr2	202044104	202049104	CASP10	+	chr2	202045305	202045842
chr2	202249081	202254081	STRADB	+	chr2	202249230	202249644
chr2	202506793	202511793	TMEM237	-	chr2	202508210	202508720
chr2	202644412	202649412	ALS2	-	chr2	202644635	202644943
chr2	204398633	204403633	RAPH1	-	chr2	204400302	204400666
chr2	207304763	207309763	ADAM23	+	chr2	207308351	207309033
chr2	208888784	208893784	PLEKHM3	-	chr2	208889747	208890443
chr2	209009392	209014392	CRYGB	-	chr2	209012322	209012732
chr2	209053297	209058297	C2orf80	-	chr2	209053151	209053608

chr2	217494051	217499051	IGFBP2	+	chr2	217498923	217499271
chr2	218866218	218871218	TNS1	-	chr2	218870681	218871005
chr2	218866218	218871218	TNS1	-	chr2	218866457	218866901
chr2	218986512	218991512	CXCR2	+	chr2	218988202	218988682
chr2	219642972	219647972	CYP27A1	+	chr2	219646345	219647748
chr2	219721044	219726044	WNT6	+	chr2	219721864	219722364
chr2	219721044	219726044	WNT6	+	chr2	219725313	219726093
chr2	219820877	219825877	CDK5R2	+	chr2	219822120	219822945
chr2	220279599	220284599	DES	+	chr2	220282196	220282636
chr2	220360089	220365089	GMPPA	+	chr2	220363510	220363987
chr2	223162215	223167215	PAX3	-	chr2	223167211	223167585
chr2	227662975	227667975	IRS1	-	chr2	227663155	227663742
chr2	228245211	228250211	TM4SF20	-	chr2	228250091	228250653
chr2	230786455	230791455	TRIP12	-	chr2	230791305	230791996
chr2	232256754	232261754	B3GNT7	+	chr2	232259944	232260474
chr2	232256754	232261754	B3GNT7	+	chr2	232256457	232257239
chr2	232313406	232318406	AC017104.2	+	chr2	232313354	232313664
chr2	232393706	232398706	NMUR1	-	chr2	232394700	232395742
chr2	232568105	232573105	PTMA	+	chr2	232569617	232569969
chr2	233381673	233386673	PRSS56	+	chr2	233384316	233384653
chr2	233893882	233898882	NEU2	+	chr2	233895627	233896280
chr2	234212962	234217962	SAG	+	chr2	234216353	234216853
chr2	234576999	234581999	UGT1A9	+	chr2	234576600	234577054
chr2	234618138	234623138	UGT1A5	+	chr2	234620692	234621284
chr2	234634254	234639254	UGT1A3	+	chr2	234637107	234638727
chr2	235404197	235409197	ARL4C	-	chr2	235405698	235406149
chr2	235857117	235862117	SH3BP4	+	chr2	235859738	235860097
chr2	238703532	238708532	RBM44	+	chr2	238704139	238704448
chr2	239138818	239143818	AC016757.3	-	chr2	239141216	239141539
chr2	239225582	239230582	TRAF3IP1	+	chr2	239229623	239229980
chr2	240968406	240973406	OR6B2	-	chr2	240972121	240972524
chr2	240983989	240988989	OR6B3	-	chr2	240986879	240987213

chr2	241507126	241512126	ANKMY1	-	chr2	241509096	241509485
chr2	241507126	241512126	ANKMY1	-	chr2	241511989	241512929
chr2	242163819	242168819	AC104841.2	+	chr2	242164885	242165273
chr2	242163819	242168819	AC104841.2	+	chr2	242166324	242166629
chr2	242573128	242578128	ATG4B	+	chr2	242572447	242573239
chr20	302707	307707	SOX12	+	chr20	306569	307868
chr20	357761	362761	TRIB3	+	chr20	362089	362396
chr20	655323	660323	SCRT2	-	chr20	656438	656998
chr20	655323	660323	SCRT2	-	chr20	654958	656065
chr20	810858	815858	FAM110A	+	chr20	813984	814387
chr20	1203200	1208200	RAD21L1	+	chr20	1205485	1205903
chr20	1973232	1978232	PDYN	-	chr20	1977839	1978527
chr20	2503848	2508848	ZNF343	-	chr20	2505394	2505912
chr20	2503848	2508848	ZNF343	-	chr20	2508817	2509411
chr20	2670024	2675024	EBF4	+	chr20	2671511	2671933
chr20	2779783	2784783	CPXM1	-	chr20	2779847	2780185
chr20	2792114	2797114	TMEM239	+	chr20	2793071	2793736
chr20	2792114	2797114	TMEM239	+	chr20	2793071	2793736
chr20	2792133	2797133	C20orf141	+	chr20	2793071	2793736
chr20	2817849	2822849	VPS16	+	chr20	2822347	2822692
chr20	3020768	3025768	GNRH2	+	chr20	3023149	3023545
chr20	3020768	3025768	GNRH2	+	chr20	3022174	3022613
chr20	3023091	3028091	MRPS26	+	chr20	3023149	3023545
chr20	3048766	3053766	OXT	+	chr20	3050550	3051112
chr20	4228221	4233221	ADRA1D	-	chr20	4230015	4230377
chr20	5295878	5300878	PROKR2	-	chr20	5296994	5297381
chr20	5983236	5988236	CRLS1	+	chr20	5986740	5987301
chr20	12986127	12991127	SPTLC3	+	chr20	12990194	12990587
chr20	13198918	13203918	ISM1	+	chr20	13200540	13200851
chr20	17203252	17208252	PCSK2	+	chr20	17207463	17208520
chr20	18038332	18043332	OVOL2	-	chr20	18041789	18042302
chr20	18265621	18270621	ZNF133	+	chr20	18270234	18270698

chr20	18484637	18489637	SEC23B	+	chr20	18488433	18488966
chr20	22564593	22569593	FOXA2	-	chr20	22564542	22564994
chr20	23431994	23436994	CST11	-	chr20	23432364	23432849
chr20	23668177	23673177	CST4	-	chr20	23669375	23669860
chr20	30059596	30064596	REM1	+	chr20	30059509	30059931
chr20	30098731	30103731	HM13	+	chr20	30101518	30102003
chr20	30431920	30436920	FOXS1	-	chr20	30433083	30433618
chr20	30861967	30866967	KIF3B	+	chr20	30862204	30862700
chr20	31171376	31176376	C20orf112	-	chr20	31174275	31174599
chr20	31171376	31176376	C20orf112	-	chr20	31176062	31176449
chr20	31171781	31176781	RP11-410N8.4	+	chr20	31174275	31174599
chr20	31171781	31176781	RP11-410N8.4	+	chr20	31176062	31176449
chr20	31330303	31335303	COMMD7	-	chr20	31330176	31330528
chr20	31615954	31620954	BPIFB6	+	chr20	31615744	31616078
chr20	31663950	31668950	BPIFB4	+	chr20	31667620	31668351
chr20	32246590	32251590	C20orf144	+	chr20	32250411	32250728
chr20	32250804	32255804	ACTL10	+	chr20	32253781	32254267
chr20	32260769	32265769	NECAB3	-	chr20	32261683	32262519
chr20	33756376	33761376	PROCR	+	chr20	33756831	33757351
chr20	34545894	34550894	SCAND1	-	chr20	34545689	34546049
chr20	34545894	34550894	SCAND1	-	chr20	34548969	34549417
chr20	35578746	35583746	SAMHD1	-	chr20	35581417	35581758
chr20	35969588	35974588	SRC	+	chr20	35969766	35970121
chr20	35969588	35974588	SRC	+	chr20	35974400	35974744
chr20	37430848	37435848	PPP1R16B	+	chr20	37434940	37435253
chr20	37551455	37556455	FAM83D	+	chr20	37554730	37555431
chr20	39653958	39658958	TOP1	+	chr20	39656910	39657337
chr20	39762100	39767100	PLCG1	+	chr20	39763927	39764262
chr20	39944812	39949812	ZHX3	-	chr20	39946486	39946846
chr20	41817110	41822110	PTPRT	-	chr20	41819794	41820225
chr20	42132820	42137820	L3MBTL1	+	chr20	42136946	42137257
chr20	42292254	42297254	MYBL2	+	chr20	42294171	42294496

chr20	42814718	42819718	JPH2	-	chr20	42816781	42817106
chr20	43092484	43097484	C20orf62	-	chr20	43096739	43097306
chr20	43092484	43097484	C20orf62	-	chr20	43093123	43093490
chr20	43535203	43540203	PABPC1L	+	chr20	43535589	43536076
chr20	43987077	43992077	SYS1	+	chr20	43987989	43988420
chr20	43988340	43993340	SYS1-DBNDD2	+	chr20	43987989	43988420
chr20	44035029	44040029	TP53TG5	-	chr20	44035145	44035657
chr20	44041217	44046217	PIGT	+	chr20	44046052	44046363
chr20	44458949	44463949	SNX21	+	chr20	44458774	44459326
chr20	44646856	44651856	SLC12A5	+	chr20	44650038	44651417
chr20	44646856	44651856	SLC12A5	+	chr20	44647730	44648076
chr20	44743411	44748411	CD40	+	chr20	44746717	44747318
chr20	45316918	45321918	TP53RK	-	chr20	45318793	45319143
chr20	45943746	45948746	AL031666.2	+	chr20	45944308	45944774
chr20	45984067	45989067	ZMYND8	-	chr20	45983599	45984496
chr20	46127101	46132101	NCOA3	+	chr20	46127190	46127661
chr20	47803404	47808404	STAU1	-	chr20	47806181	47806540
chr20	48530580	48535580	SPATA2	-	chr20	48531491	48531824
chr20	49571863	49576863	MOCS3	+	chr20	49576450	49576847
chr20	49573592	49578592	DPM1	-	chr20	49576450	49576847
chr20	50177870	50182870	NFATC2	-	chr20	50180626	50181052
chr20	51585446	51590446	TSHZ2	+	chr20	51588797	51589530
chr20	53088636	53093636	DOK5	+	chr20	53091053	53091447
chr20	54579028	54584028	CBLN4	-	chr20	54581155	54581546
chr20	55840185	55845185	BMP7	-	chr20	55841222	55841609
chr20	55922566	55927566	RAE1	+	chr20	55923702	55924031
chr20	55962963	55967963	RBM38	+	chr20	55964627	55964947
chr20	55962963	55967963	RBM38	+	chr20	55967945	55968379
chr20	56099208	56104208	CTCFL	-	chr20	56102337	56102699
chr20	57580802	57585802	CTSZ	-	chr20	57583441	57583940
chr20	58149064	58154064	PHACTR3	+	chr20	58151186	58151501
chr20	58149064	58154064	PHACTR3	+	chr20	58149753	58150269

chr20	60639366	60644366	TAF4	-	chr20	60642226	60642905
chr20	60715322	60720322	SS18L1	+	chr20	60719634	60719986
chr20	60716996	60721996	PSMA7	-	chr20	60719634	60719986
chr20	61144160	61149160	C20orf166	+	chr20	61147232	61147690
chr20	61432687	61437687	OGFR	+	chr20	61436599	61437693
chr20	61444096	61449096	COL9A3	+	chr20	61445444	61445934
chr20	61902546	61907546	NKAIN4	-	chr20	61905713	61906133
chr20	62008253	62013253	CHRNA4	-	chr20	62010907	62011242
chr20	62102493	62107493	KCNQ2	-	chr20	62102481	62102929
chr20	62129005	62134005	EEF1A2	-	chr20	62132154	62132762
chr20	62180873	62185873	C20orf195	+	chr20	62184904	62185421
chr20	62256894	62261894	GMEB2	-	chr20	62258166	62258671
chr20	62335317	62340317	ZGPAT	+	chr20	62337537	62337863
chr20	62336716	62341716	RP4-583P15.15	+	chr20	62337537	62337863
chr20	62581507	62586507	AL118506.1	+	chr20	62583038	62583651
chr20	62608988	62613988	PRPF6	+	chr20	62610076	62610436
chr20	62609861	62614861	SAMD10	-	chr20	62610076	62610436
chr20	62677689	62682689	TCEA2	+	chr20	62682146	62682752
chr20	62677689	62682689	TCEA2	+	chr20	62678475	62678871
chr20	62678613	62683613	ZNF512B	-	chr20	62682146	62682752
chr20	62678613	62683613	ZNF512B	-	chr20	62678475	62678871
chr20	62679494	62684494	SOX18	-	chr20	62682146	62682752
chr20	62737024	62742024	NPBWR2	-	chr20	62741818	62742124
chr21	28216228	28221228	ADAMTS1	-	chr21	28218392	28218760
chr21	30444618	30449618	CCT8	-	chr21	30445024	30445379
chr21	31809097	31814097	KRTAP15-1	+	chr21	31814076	31814846
chr21	33242128	33247128	HUNK	+	chr21	33243051	33243387
chr21	34541041	34546041	C21orf54	-	chr21	34542105	34542427
chr21	34635163	34640163	IL10RB	+	chr21	34635060	34635421
chr21	37525580	37530580	DOPEY2	+	chr21	37526009	37526357
chr21	38374950	38379950	RIPPLY3	+	chr21	38375265	38375592
chr21	38442026	38447026	TTC3	+	chr21	38445311	38445640

chr21	38443970	38448970	PIGP	-	chr21	38445311	38445640
chr21	39525628	39530628	KCNJ15	+	chr21	39528132	39528456
chr21	40814281	40819281	SH3BGR	+	chr21	40814089	40814502
chr21	43734261	43739261	TFF3	-	chr21	43738093	43739110
chr21	44495553	44500553	CBS	-	chr21	44496405	44496903
chr21	44585618	44590618	CRYAA	+	chr21	44589957	44590364
chr21	44845508	44850508	SIK1	-	chr21	44846847	44847364
chr21	45135475	45140475	PDXK	+	chr21	45137723	45138135
chr21	45281567	45286567	AGPAT3	+	chr21	45284288	45284620
chr21	45428700	45433700	TRAPPC10	+	chr21	45430543	45430877
chr21	45702221	45707221	AIRE	+	chr21	45707191	45707503
chr21	45757785	45762785	C21orf2	-	chr21	45757901	45758228
chr21	45766546	45771546	TRPM2	+	chr21	45769247	45769799
chr21	46010886	46015886	KRTAP10-6	-	chr21	46013528	46014261
chr21	46073076	46078076	KRTAP12-4	-	chr21	46073682	46074123
chr21	47514511	47519511	COL6A2	+	chr21	47515987	47516532
chr21	47647238	47652238	LSS	-	chr21	47647074	47647420
chr21	48051579	48056579	PRMT2	+	chr21	48055405	48055883
chr22	17487612	17492612	GAB4	-	chr22	17491965	17492362
chr22	17701379	17706379	CECR1	-	chr22	17700994	17701412
chr22	18589597	18594597	TUBA8	+	chr22	18592825	18593170
chr22	19114821	19119821	TSSK2	+	chr22	19117475	19117866
chr22	19277739	19282739	CLTCL1	-	chr22	19277486	19277847
chr22	19513568	19518568	CLDN5	-	chr22	19514246	19514642
chr22	19513568	19518568	CLDN5	-	chr22	19516852	19517558
chr22	19740726	19745726	TBX1	+	chr22	19740440	19740773
chr22	19840919	19845919	C22orf29	-	chr22	19843604	19844315
chr22	19840962	19845962	GNB1L	-	chr22	19843604	19844315
chr22	20001037	20006037	TANGO2	+	chr22	20002331	20002716
chr22	20099961	20104961	RANBP1	+	chr22	20100681	20101004
chr22	20099961	20104961	RANBP1	+	chr22	20104747	20105217
chr22	20103415	20108415	TRMT2A	-	chr22	20104747	20105217

chr22	20113479	20118479	ZDHHC8	+	chr22	20118295	20119227
chr22	20846700	20851700	MED15	+	chr22	20849543	20849856
chr22	20848670	20853670	KLHL22	-	chr22	20849543	20849856
chr22	21124667	21129667	SERPIND1	+	chr22	21125134	21125475
chr22	21354985	21359985	THAP7	-	chr22	21354733	21355104
chr22	21904250	21909250	RIMBP3C	-	chr22	21907460	21907810
chr22	22003059	22008059	PPIL2	+	chr22	22006740	22007175
chr22	22003059	22008059	PPIL2	+	chr22	22002766	22003073
chr22	22088623	22093623	YPEL1	-	chr22	22090694	22091025
chr22	23518391	23523391	BCR	+	chr22	23518094	23518535
chr22	24101708	24106708	C22orf15	+	chr22	24106539	24106847
chr22	24101708	24106708	C22orf15	+	chr22	24104220	24104683
chr22	24106913	24111913	MMP11	+	chr22	24107365	24107680
chr22	24179815	24184815	DERL3	-	chr22	24181849	24182411
chr22	24305589	24310589	DDTL	+	chr22	24309939	24310263
chr22	25611989	25616989	CRYBB2	+	chr22	25613979	25614291
chr22	25799844	25804844	LRP5L	-	chr22	25800965	25801321
chr22	26876343	26881343	SRRD	+	chr22	26876542	26876936
chr22	28195986	28200986	MN1	-	chr22	28197272	28197580
chr22	29165162	29170162	CCDC117	+	chr22	29167660	29168043
chr22	29276080	29281080	ZNRF3	+	chr22	29280741	29281078
chr22	29465566	29470566	KREMEN1	+	chr22	29469510	29470063
chr22	29831072	29836072	RFPL1	+	chr22	29832166	29832519
chr22	30812969	30817969	KIAA1658	-	chr22	30814235	30814931
chr22	30818018	30823018	MTFP1	+	chr22	30821779	30822259
chr22	30818018	30823018	MTFP1	+	chr22	30822814	30823337
chr22	30941169	30946169	SEC14L6	-	chr22	30943740	30944302
chr22	31552668	31557668	RNF185	+	chr22	31553876	31554304
chr22	31552668	31557668	RNF185	+	chr22	31556123	31556486
chr22	32435519	32440519	SLC5A1	+	chr22	32438596	32438927
chr22	32599218	32604218	RFPL2	-	chr22	32599063	32599447
chr22	33194187	33199187	TIMP3	+	chr22	33196605	33197045

chr22	33194187	33199187	TIMP3	+	chr22	33199166	33199572
chr22	34317329	34322329	LARGE	-	chr22	34319667	34320188
chr22	36876577	36881577	TXN2	-	chr22	36877407	36877828
chr22	37098103	37103103	CACNG2	-	chr22	37099568	37100032
chr22	37170800	37175800	IFT27	-	chr22	37174037	37174428
chr22	37253530	37258530	NCF4	+	chr22	37254755	37255074
chr22	37569594	37574594	IL2RB	-	chr22	37572938	37573413
chr22	37822005	37827005	ELFN2	-	chr22	37824621	37825027
chr22	37914049	37919049	CARD10	-	chr22	37915402	37915747
chr22	38027161	38032161	SH3BP1	+	chr22	38030224	38030598
chr22	38243834	38248834	ANKRD54	-	chr22	38247998	38248330
chr22	38667540	38672540	TMEM184B	-	chr22	38667620	38667929
chr22	39374852	39379852	APOBEC3B	+	chr22	39376398	39376747
chr22	39639256	39644256	PDGFB	-	chr22	39640895	39641224
chr22	39963258	39968258	CACNA1I	+	chr22	39965278	39965583
chr22	41256630	41261630	DNAJB7	-	chr22	41260490	41260869
chr22	41597709	41602709	L3MBTL2	+	chr22	41600931	41601320
chr22	41635438	41640438	CHADL	-	chr22	41635264	41635976
chr22	41680755	41685755	RANGAP1	-	chr22	41683458	41683799
chr22	41984394	41989394	PMM1	-	chr22	41989232	41989633
chr22	42193183	42198183	CCDC134	+	chr22	42192996	42193516
chr22	42466755	42471755	FAM109B	+	chr22	42471243	42471732
chr22	43252612	43257612	ARFGAP3	-	chr22	43254673	43255377
chr22	44707231	44712231	KIAA1644	-	chr22	44711278	44711606
chr22	45556222	45561222	NUP50	+	chr22	45559707	45560183
chr22	45635150	45640150	KIAA0930	-	chr22	45637090	45637467
chr22	46371509	46376509	WNT7B	-	chr22	46372300	46372729
chr22	46446249	46451249	FLJ27365	+	chr22	46450271	46450614
chr22	46448524	46453524	C22orf26	-	chr22	46450271	46450614
chr22	46723272	46728272	TRMU	+	chr22	46725620	46726216
chr22	47132658	47137658	CERK	-	chr22	47137096	47137477
chr22	50620844	50625844	TRABD	+	chr22	50621829	50622226

chr22	50744556	50749556	PLXNB2	-	chr22	50746762	50747423
chr22	50744556	50749556	PLXNB2	-	chr22	50744820	50745192
chr22	50916485	50921485	ADM2	+	chr22	50920094	50920554
chr22	51220591	51225591	RABL2B	-	chr22	51223629	51224019
chr3	8785226	8790226	SSUH2	-	chr3	8787130	8787467
chr3	9003957	9008957	RAD18	-	chr3	9006852	9007278
chr3	9687617	9692617	MTMR14	+	chr3	9692336	9692820
chr3	9830679	9835679	ARPC4	+	chr3	9835249	9835638
chr3	9831266	9836266	ARPC4-TTLL3	+	chr3	9835249	9835638
chr3	9833195	9838195	TADA3	-	chr3	9835249	9835638
chr3	9920438	9925438	CIDEC	-	chr3	9923677	9924123
chr3	9928738	9933738	JAGN1	+	chr3	9929507	9929917
chr3	10203049	10208049	IRAK2	+	chr3	10206769	10207285
chr3	11886893	11891893	TAMM41	-	chr3	11887812	11888183
chr3	11886893	11891893	TAMM41	-	chr3	11889694	11890293
chr3	14216358	14221358	LSM3	+	chr3	14218085	14218390
chr3	14440576	14445576	SLC6A6	+	chr3	14444995	14445603
chr3	15561758	15566758	COLQ	-	chr3	15562040	15562474
chr3	16710313	16715313	DAZL	-	chr3	16710354	16710778
chr3	16710313	16715313	DAZL	-	chr3	16711242	16711614
chr3	23983251	23988251	NR1D2	+	chr3	23987480	23987987
chr3	23986582	23991582	NKIRAS1	-	chr3	23987480	23987987
chr3	25830030	25835030	NGLY1	-	chr3	25834082	25834396
chr3	27762706	27767706	EOMES	-	chr3	27763546	27763893
chr3	31570630	31575630	STT3B	+	chr3	31573706	31574032
chr3	32019763	32024763	ZNF860	+	chr3	32020449	32020782
chr3	32143681	32148681	GPD1L	+	chr3	32147045	32147517
chr3	32276671	32281671	CMTM8	+	chr3	32277667	32277989
chr3	32429663	32434663	CMTM7	+	chr3	32432732	32433232
chr3	37490106	37495106	ITGA9	+	chr3	37491236	37491663
chr3	38026050	38031050	VILL	+	chr3	38028877	38029208
chr3	38069753	38074753	PLCD1	-	chr3	38069990	38070409

chr3	39194553	39199553	CSRNP1	-	chr3	39197457	39197870
chr3	39232587	39237587	XIRP1	-	chr3	39232428	39232914
chr3	39321726	39326726	CX3CR1	-	chr3	39323839	39324144
chr3	40495283	40500283	RPL14	+	chr3	40498202	40498769
chr3	42638606	42643606	NKTR	+	chr3	42641057	42641422
chr3	42641072	42646072	SEC22C	-	chr3	42641057	42641422
chr3	42641072	42646072	SEC22C	-	chr3	42645926	42646279
chr3	42742819	42747819	HHATL	-	chr3	42747442	42747869
chr3	42742819	42747819	HHATL	-	chr3	42743068	42744017
chr3	44550628	44555628	ZNF852	-	chr3	44552649	44553147
chr3	44899861	44904861	TMEM42	+	chr3	44904773	44905090
chr3	45593386	45598386	LIMD1	+	chr3	45593383	45593703
chr3	45727048	45732048	SACM1L	+	chr3	45730565	45730929
chr3	45956034	45961034	LZTFL1	-	chr3	45956640	45956959
chr3	46035816	46040816	FYCO1	-	chr3	46036300	46036764
chr3	46408133	46413133	CCR5	+	chr3	46410145	46410481
chr3	46733694	46738694	ALS2CL	-	chr3	46734867	46735227
chr3	46739323	46744323	TMIE	+	chr3	46740055	46740474
chr3	47840899	47845899	DHX30	+	chr3	47844054	47844404
chr3	47840899	47845899	DHX30	+	chr3	47844858	47845468
chr3	48129269	48134269	MAP4	-	chr3	48132444	48132864
chr3	48470094	48475094	PLXNB1	-	chr3	48472118	48472424
chr3	48502945	48507945	TREX1	+	chr3	48505441	48505861
chr3	48597948	48602948	PFKFB4	-	chr3	48600814	48601260
chr3	48599706	48604706	UCN2	-	chr3	48600814	48601260
chr3	48776286	48781286	IP6K2	-	chr3	48777466	48777801
chr3	49040995	49045995	WDR6	+	chr3	49044738	49045048
chr3	49054392	49059392	NDUFAF3	+	chr3	49055119	49055579
chr3	49054392	49059392	NDUFAF3	+	chr3	49058740	49059136
chr3	49058226	49063226	DALRD3	-	chr3	49058740	49059136
chr3	49205544	49210544	KLHDC8B	+	chr3	49208972	49209592
chr3	49937778	49942778	CTD-2330K9.3	+	chr3	49939355	49939781

chr3	49937778	49942778	CTD-2330K9.3	+	chr3	49941166	49941586
chr3	49939799	49944799	MST1R	-	chr3	49941166	49941586
chr3	50225545	50230545	GNAT1	+	chr3	50229926	50230275
chr3	50312958	50317958	LSMEM2	+	chr3	50314862	50315308
chr3	50358837	50363837	HYAL2	-	chr3	50358682	50358987
chr3	50358837	50363837	HYAL2	-	chr3	50360451	50361431
chr3	50540175	50545175	CACNA2D2	-	chr3	50540546	50540899
chr3	50540175	50545175	CACNA2D2	-	chr3	50542663	50542992
chr3	50540175	50545175	CACNA2D2	-	chr3	50543503	50543809
chr3	50603083	50608083	HEMK1	+	chr3	50607013	50607420
chr3	50606958	50611958	C3orf18	-	chr3	50607013	50607420
chr3	51425231	51430231	RBM15B	+	chr3	51428487	51428825
chr3	51972861	51977861	PARP3	+	chr3	51975421	51975730
chr3	51972861	51977861	PARP3	+	chr3	51977566	51977959
chr3	51974457	51979457	RRP9	-	chr3	51975421	51975730
chr3	51974457	51979457	RRP9	-	chr3	51977566	51977959
chr3	52089066	52094066	DUSP7	-	chr3	52090616	52090948
chr3	52187206	52192206	POC1A	-	chr3	52190918	52191251
chr3	52276341	52281341	PPM1M	+	chr3	52278968	52279631
chr3	52276341	52281341	PPM1M	+	chr3	52277665	52278145
chr3	52564529	52569529	SMIM4	+	chr3	52567756	52568132
chr3	52567570	52572570	NT5DC2	-	chr3	52567756	52568132
chr3	52718433	52723433	PBRM1	-	chr3	52719873	52720294
chr3	52738548	52743548	GLT8D1	-	chr3	52742551	52742937
chr3	52803465	52808465	NEK4	-	chr3	52807345	52807700
chr3	53186525	53191525	PRKCD	+	chr3	53187819	53188164
chr3	53186525	53191525	PRKCD	+	chr3	53190188	53190530
chr3	55522473	55527473	WNT5A	-	chr3	55523726	55524039
chr3	58219733	58224733	ABHD6	+	chr3	58223725	58224122
chr3	58299841	58304841	RPP14	+	chr3	58300787	58301139
chr3	61235633	61240633	FHIT	-	chr3	61239542	61239906
chr3	63846733	63851733	ATXN7	+	chr3	63847376	63847701

chr3	66115785	66120785	SLC25A26	+	chr3	66115651	66116064
chr3	69099984	69104984	TMF1	-	chr3	69103692	69104037
chr3	69590234	69595234	FRMD4B	-	chr3	69590154	69590459
chr3	72909565	72914565	SHQ1	-	chr3	72911492	72911821
chr3	85004632	85009632	CADM2	+	chr3	85006167	85006516
chr3	97537384	97542384	CRYBG3	+	chr3	97541480	97541831
chr3	97998232	98003232	OR5H2	+	chr3	98001726	98002119
chr3	98106010	98111010	OR5K3	+	chr3	98109433	98109801
chr3	99533178	99538178	CMSS1	+	chr3	99537602	99538233
chr3	100710859	100715859	ABI3BP	-	chr3	100712384	100712732
chr3	100710859	100715859	ABI3BP	-	chr3	100713510	100713848
chr3	101289439	101294439	PCNP	+	chr3	101288974	101289486
chr3	108671242	108676242	GUCA1C	-	chr3	108675067	108675804
chr3	112279393	112284393	ATG3	-	chr3	112282893	112283236
chr3	112692469	112697469	CD200R1	-	chr3	112693671	112694006
chr3	112692469	112697469	CD200R1	-	chr3	112694479	112694833
chr3	113158957	113163957	WDR52	-	chr3	113161545	113162059
chr3	113158957	113163957	WDR52	-	chr3	113159090	113159499
chr3	113413993	113418993	KIAA2018	-	chr3	113413556	113414039
chr3	113543529	113548529	GRAMD1C	+	chr3	113545783	113546141
chr3	121899030	121904030	CASR	+	chr3	121903208	121903520
chr3	122281924	122286924	PARP9	-	chr3	122285486	122285865
chr3	122510142	122515142	DIRC2	+	chr3	122512549	122512931
chr3	122511171	122516171	HSPBAP1	-	chr3	122512549	122512931
chr3	124773302	124778302	HEG1	-	chr3	124774658	124775154
chr3	125237541	125242541	SNX4	-	chr3	125238696	125239195
chr3	125654382	125659382	ALG1L	-	chr3	125657414	125657733
chr3	126193262	126198262	ZXDC	-	chr3	126194352	126194741
chr3	127540551	127545551	MGLL	-	chr3	127541778	127542313
chr3	127868797	127873797	EEFSEC	+	chr3	127868489	127868865
chr3	128210528	128215528	GATA2	-	chr3	128213041	128213465
chr3	128716972	128721972	EFCC1	+	chr3	128716908	128717299

chr3	129243983	129248983	RHO	+	chr3	129247278	129247658
chr3	129610919	129615919	TMCC1	-	chr3	129613772	129614106
chr3	129610919	129615919	TMCC1	-	chr3	129612823	129613226
chr3	130742194	130747194	NEK11	+	chr3	130743487	130743872
chr3	132312581	132317581	ACKR4	+	chr3	132313949	132314267
chr3	134092821	134097821	AMOTL2	-	chr3	134097540	134098019
chr3	136661572	136666572	IL20RB	+	chr3	136663574	136663967
chr3	137892291	137897291	DBR1	-	chr3	137894051	137894359
chr3	138063039	138068039	MRAS	+	chr3	138067729	138068091
chr3	138323948	138328948	FAIM	+	chr3	138326316	138326765
chr3	138723610	138728610	PRR23A	-	chr3	138726397	138726814
chr3	141493490	141498490	GRK7	+	chr3	141498152	141498460
chr3	142439416	142444416	TRPC1	+	chr3	142443177	142443613
chr3	142834673	142839673	CHST2	+	chr3	142839286	142839658
chr3	148802841	148807841	HLTF	-	chr3	148807778	148808217
chr3	149468786	149473786	COMMD2	-	chr3	149470819	149471214
chr3	149684672	149689672	ANKUB1	-	chr3	149686407	149686752
chr3	149685566	149690566	AC117395.1	+	chr3	149686407	149686752
chr3	153835292	153840292	ARHGEF26	+	chr3	153839860	153840176
chr3	153835292	153840292	ARHGEF26	+	chr3	153835000	153835345
chr3	154738413	154743413	MME	+	chr3	154738583	154738998
chr3	157257535	157262535	C3orf55	+	chr3	157258245	157258551
chr3	157822792	157827792	SHOX2	-	chr3	157827780	157828359
chr3	158388982	158393982	LXN	-	chr3	158391313	158391652
chr3	158446487	158451487	MFSD1	+	chr3	158446982	158447825
chr3	158448985	158453985	RARRES1	-	chr3	158453883	158454211
chr3	159703037	159708037	IL12A	+	chr3	159704464	159704803
chr3	167449531	167454531	SERPINI1	+	chr3	167449016	167449613
chr3	169379906	169384906	MECOM	-	chr3	169382540	169382923
chr3	169529274	169534274	LRRC34	-	chr3	169530109	169530449
chr3	169680923	169685923	SEC62	+	chr3	169684467	169684910
chr3	176913761	176918761	TBL1XR1	-	chr3	176915387	176916492

chr3	180582429	180587429	FXR1	+	chr3	180581871	180582502
chr3	182880127	182885127	LAMP3	-	chr3	182884668	182884998
chr3	186497494	186502494	EIF4A2	+	chr3	186501889	186502215
chr3	195444253	195449253	MUC20	+	chr3	195448465	195449036
chr3	195444253	195449253	MUC20	+	chr3	195444043	195444869
chr3	196229139	196234139	RNF168	-	chr3	196231641	196232080
chr3	196291982	196296982	FBXO45	+	chr3	196295875	196296300
chr3	196755187	196760187	MFI2	-	chr3	196754756	196755194
chr3	197024671	197029671	DLG1	-	chr3	197029298	197029641
chr4	489489	494489	PIGG	+	chr4	493047	493538
chr4	491445	496445	ZNF721	-	chr4	493047	493538
chr4	696037	701037	PCGF3	+	chr4	698662	699068
chr4	818486	823486	CPLX1	-	chr4	819088	819409
chr4	924661	929661	GAK	-	chr4	929571	929880
chr4	1280139	1285139	MAEA	+	chr4	1280341	1280722
chr4	1791534	1796534	FGFR3	+	chr4	1794709	1795145
chr4	1856474	1861474	LETM1	-	chr4	1857596	1858122
chr4	2040189	2045189	C4orf48	+	chr4	2041152	2041508
chr4	2791250	2796250	SH3BP2	+	chr4	2791229	2791573
chr4	2791250	2796250	SH3BP2	+	chr4	2795145	2795778
chr4	2961835	2966835	GRK4	+	chr4	2964108	2965087
chr4	2963612	2968612	NOP14	-	chr4	2964108	2965087
chr4	3242596	3247596	MSANTD1	+	chr4	3245430	3245754
chr4	3242596	3247596	MSANTD1	+	chr4	3243322	3243639
chr4	3440114	3445114	HGFAC	+	chr4	3443483	3443849
chr4	3764575	3769575	ADRA2C	+	chr4	3766851	3767318
chr4	4248450	4253450	TMEM128	-	chr4	4250286	4250686
chr4	4288424	4293424	ZBTB49	+	chr4	4288602	4288949
chr4	4857893	4862893	MSX1	+	chr4	4860378	4860744
chr4	5709424	5714424	EVC	+	chr4	5709825	5710192
chr4	5709775	5714775	EVC2	-	chr4	5709825	5710192
chr4	6268076	6273076	WFS1	+	chr4	6272106	6272430

chr4	9213631	9218631	USP17L11	+	chr4	9218346	9218743
chr4	9218378	9223378	USP17L12	+	chr4	9218346	9218743
chr4	9246856	9251856	USP17L18	+	chr4	9247375	9247688
chr4	9256350	9261350	USP17L20	+	chr4	9257423	9257748
chr4	9265845	9270845	USP17L22	+	chr4	9267612	9267935
chr4	9332884	9337884	USP17L26	+	chr4	9333283	9333697
chr4	10457534	10462534	ZNF518B	-	chr4	10461679	10462834
chr4	13545174	13550174	NKX3-2	-	chr4	13546962	13547276
chr4	13627847	13632847	BOD1L1	-	chr4	13629314	13629690
chr4	17612754	17617754	MED28	+	chr4	17614484	17614804
chr4	37889208	37894208	TBC1D1	+	chr4	37892574	37892990
chr4	39639210	39644210	SMIM14	-	chr4	39639039	39639351
chr4	39696164	39701164	UBE2K	+	chr4	39698994	39699348
chr4	41358124	41363124	LIMCH1	+	chr4	41360424	41360755
chr4	44727112	44732112	GNPDA2	-	chr4	44728554	44728895
chr4	44727112	44732112	GNPDA2	-	chr4	44727302	44727688
chr4	46994924	46999924	GABRA4	-	chr4	46997417	46997820
chr4	54929357	54934357	CHIC2	-	chr4	54930982	54931714
chr4	55990256	55995256	KDR	-	chr4	55994578	55994939
chr4	57032861	57037861	KIAA1211	+	chr4	57033197	57033516
chr4	57300281	57305281	PPAT	-	chr4	57304188	57304504
chr4	57840388	57845388	POLR2B	+	chr4	57841176	57841523
chr4	57840388	57845388	POLR2B	+	chr4	57843209	57843606
chr4	57843489	57848489	NOA1	-	chr4	57843209	57843606
chr4	57843489	57848489	NOA1	-	chr4	57847682	57847994
chr4	70652179	70657179	SULT1B1	-	chr4	70656025	70656337
chr4	71704162	71709162	GRSF1	-	chr4	71706334	71706717
chr4	71854755	71859755	DCK	+	chr4	71854523	71855020
chr4	72668258	72673258	GC	-	chr4	72671421	72671740
chr4	73433016	73438016	ADAMTS3	-	chr4	73433810	73434232
chr4	74846341	74851341	PF4	-	chr4	74848471	74848878
chr4	76477758	76482758	C4orf26	+	chr4	76480855	76481299

chr4	77995658	78000658	CCNI	-	chr4	77996329	77996726
chr4	78975224	78980224	FRAS1	+	chr4	78977449	78977780
chr4	83348215	83353215	ENOPH1	+	chr4	83350393	83350716
chr4	83349794	83354794	HNRNPDL	-	chr4	83350393	83350716
chr4	83718510	83723510	SCD5	-	chr4	83723407	83723790
chr4	83718510	83723510	SCD5	-	chr4	83719733	83720045
chr4	88893319	88898319	SPP1	+	chr4	88893352	88893736
chr4	89617886	89622886	NAP1L5	-	chr4	89622121	89622475
chr4	90030468	90035468	TIGD2	+	chr4	90035129	90035849
chr4	90031049	90036049	FAM13A	-	chr4	90035129	90035849
chr4	108640108	108645108	PAPSS1	-	chr4	108641559	108641906
chr4	108849025	108854025	CYP2U1	+	chr4	108852615	108852925
chr4	108907370	108912370	HADH	+	chr4	108908097	108908482
chr4	113203165	113208165	ALPK1	+	chr4	113206613	113207061
chr4	113205559	113210559	TIFA	-	chr4	113206613	113207061
chr4	115516111	115521111	UGT8	+	chr4	115519782	115520408
chr4	119272658	119277658	PRSS12	-	chr4	119275161	119275525
chr4	119758338	119763338	SEC24D	-	chr4	119762513	119762860
chr4	123069988	123074988	KIAA1109	+	chr4	123071521	123071837
chr4	123296621	123301621	ADAD1	+	chr4	123300230	123300582
chr4	128648033	128653033	SLC25A31	+	chr4	128651479	128651831
chr4	140222205	140227205	NDUFC1	-	chr4	140224571	140224910
chr4	144102570	144107570	USP38	+	chr4	144101887	144102583
chr4	146858287	146863287	ZNF827	-	chr4	146858587	146858904
chr4	147142209	147147209	RP11-6L6.2	+	chr4	147146781	147147095
chr4	147441623	147446623	SLC10A7	-	chr4	147443943	147444328
chr4	149364350	149369350	NR3C2	-	chr4	149368922	149369267
chr4	153697589	153702589	ARFIP1	+	chr4	153700423	153700866
chr4	153699416	153704416	TIGD4	-	chr4	153700423	153700866
chr4	154619152	154624152	TLR2	+	chr4	154623663	154624168
chr4	154708772	154713772	SFRP2	-	chr4	154711883	154712302
chr4	166125270	166130270	KLHL2	+	chr4	166125260	166125649

chr4	170530284	170535284	CLCN3	+	chr4	170531899	170532252
chr4	171011350	171016350	AADAT	-	chr4	171012404	171012732
chr4	175201328	175206328	CEP44	+	chr4	175204281	175204739
chr4	175203915	175208915	FBXO8	-	chr4	175204281	175204739
chr4	178362157	178367157	AGA	-	chr4	178364046	178364468
chr4	184240427	184245427	CLDN22	-	chr4	184240724	184241118
chr5	136873	141873	PLEKHG4B	+	chr5	137386	137805
chr5	692010	697010	TPPP	-	chr5	695419	695748
chr5	692010	697010	TPPP	-	chr5	692847	693429
chr5	889258	894258	TRIP13	+	chr5	892394	892794
chr5	891439	896439	BRD9	-	chr5	892394	892794
chr5	1198210	1203210	SLC6A19	+	chr5	1202348	1202729
chr5	1444045	1449045	SLC6A3	-	chr5	1446996	1447304
chr5	1522592	1527592	LPCAT1	-	chr5	1525642	1526027
chr5	2748745	2753745	C5orf38	+	chr5	2750375	2750743
chr5	5136943	5141943	ADAMTS16	+	chr5	5139628	5139998
chr5	9544687	9549687	SEMA5A	-	chr5	9545603	9546116
chr5	10438136	10443136	ROPN1L	+	chr5	10442060	10442466
chr5	15496805	15501805	FBXL7	+	chr5	15500771	15501172
chr5	16179371	16184371	MARCH11	-	chr5	16181922	16182286
chr5	16464401	16469401	ZNF622	-	chr5	16466786	16467181
chr5	23503764	23508764	PRDM9	+	chr5	23507054	23507497
chr5	31636017	31641017	PDZD2	+	chr5	31638932	31639841
chr5	34835438	34840438	TTC23L	+	chr5	34839860	34840208
chr5	35046698	35051698	AGXT2	-	chr5	35047749	35048103
chr5	35047512	35052512	AC010368.2	+	chr5	35047749	35048103
chr5	35849297	35854297	IL7R	+	chr5	35851069	35851530
chr5	40676100	40681100	PTGER4	+	chr5	40679515	40679830
chr5	41509230	41514230	PLCXD3	-	chr5	41510123	41510599
chr5	43513747	43518747	C5orf34	-	chr5	43514939	43515414
chr5	51300365	51305365	CTD-2203A3.1	+	chr5	51301865	51302204
chr5	52080230	52085230	ITGA1	+	chr5	52084005	52084421

chr5	52080274	52085274	PELO	+	chr5	52084005	52084421
chr5	52772739	52777739	FST	+	chr5	52776479	52776849
chr5	55067522	55072522	SLC38A9	-	chr5	55068433	55068770
chr5	61806809	61811809	CKS1B	-	chr5	61808906	61809283
chr5	65888676	65893676	MAST4	+	chr5	65888878	65889721
chr5	67482204	67487204	RP11-404L6.2	+	chr5	67485917	67486264
chr5	70747942	70752942	BDP1	+	chr5	70751380	70751935
chr5	72742852	72747852	FOXD1	-	chr5	72747218	72747553
chr5	74628654	74633654	HMGCR	+	chr5	74630282	74630932
chr5	74804081	74809081	POLK	+	chr5	74808451	74808902
chr5	74806463	74811463	COL4A3BP	-	chr5	74808451	74808902
chr5	75695574	75700574	IQGAP2	+	chr5	75698195	75698760
chr5	76502774	76507774	PDE8B	+	chr5	76506021	76506364
chr5	78280410	78285410	ARSB	-	chr5	78285399	78285794
chr5	79285582	79290582	MTX3	-	chr5	79289504	79289826
chr5	79945355	79950355	MTRNR2L2	-	chr5	79950114	79950505
chr5	79946967	79951967	MSH3	+	chr5	79950114	79950505
chr5	79949302	79954302	DHFR	-	chr5	79950114	79950505
chr5	80593909	80598909	ZCCHC9	+	chr5	80597014	80597334
chr5	80688498	80693498	ACOT12	-	chr5	80688631	80689047
chr5	81046116	81051116	SSBP2	-	chr5	81045911	81046401
chr5	83679111	83684111	EDIL3	-	chr5	83681102	83681410
chr5	83679111	83684111	EDIL3	-	chr5	83683402	83684011
chr5	92915543	92920543	NR2F1	+	chr5	92918354	92918809
chr5	93075843	93080843	POU5F2	-	chr5	93076189	93076529
chr5	100237470	100242470	ST8SIA4	-	chr5	100239471	100239859
chr5	110070337	110075337	SLC25A46	+	chr5	110071568	110071876
chr5	112223813	112228813	ZRSR1	+	chr5	112227996	112228305
chr5	112845880	112850880	YTHDC2	+	chr5	112846256	112846577
chr5	114879091	114884091	FEM1C	-	chr5	114880419	114880751
chr5	115909130	115914130	SEMA6A	-	chr5	115908554	115909247
chr5	118784638	118789638	HSD17B4	+	chr5	118786319	118786665

chr5	122844293	122849293	CSNK1G3	+	chr5	122848065	122848812
chr5	124083000	124088000	ZNF608	-	chr5	124087766	124088102
chr5	125692324	125697324	GRAMD3	+	chr5	125694648	125695138
chr5	126365000	126370000	MARCH3	-	chr5	126365659	126366110
chr5	130505928	130510928	HINT1	-	chr5	130510763	130511203
chr5	131405983	131410983	CSF2	+	chr5	131406434	131406958
chr5	131824990	131829990	IRF1	-	chr5	131826821	131827137
chr5	132204514	132209514	LEAP2	+	chr5	132208629	132209011
chr5	133560262	133565262	CTD-2410N18.5	-	chr5	133560745	133561370
chr5	133560333	133565333	PPP2CA	-	chr5	133560745	133561370
chr5	134070691	134075691	CAMLG	+	chr5	134074190	134074587
chr5	134237096	134242096	PCBD2	+	chr5	134241687	134242141
chr5	137088539	137093539	HNRNPA0	-	chr5	137092628	137093161
chr5	137199980	137204980	MYOT	+	chr5	137199698	137200065
chr5	137386150	137391150	FAM13B	-	chr5	137388890	137389205
chr5	137386150	137391150	FAM13B	-	chr5	137386047	137386391
chr5	137608860	137613860	GFRA3	-	chr5	137610861	137611216
chr5	138718742	138723742	SLC23A1	-	chr5	138719765	138720099
chr5	139023384	139028384	CXXC5	+	chr5	139023418	139023801
chr5	139023384	139028384	CXXC5	+	chr5	139025929	139026292
chr5	140170944	140175944	PCDHA2	+	chr5	140175655	140176079
chr5	140251558	140256558	PCDHA12	+	chr5	140255915	140256272
chr5	140342320	140347320	PCDHAC2	+	chr5	140344885	140345302
chr5	140532077	140537077	PCDHB17	+	chr5	140537028	140537359
chr5	140726328	140731328	PCDHGB1	+	chr5	140729272	140729631
chr5	145312642	145317642	SH3RF2	+	chr5	145314829	145315150
chr5	146462847	146467847	PPP2R2B	-	chr5	146464117	146464511
chr5	147246745	147251745	SCGB3A2	+	chr5	147251441	147252067
chr5	148928010	148933010	ARHGEF37	+	chr5	148930761	148931181
chr5	148929507	148934507	CSNK1A1	-	chr5	148930761	148931181
chr5	149533935	149538935	PDGFRB	-	chr5	149535773	149536138
chr5	149566020	149571020	SLC6A7	+	chr5	149566812	149567315

chr5	150154008	150159008	SMIM3	+	chr5	150153781	150154344
chr5	150222585	150227585	IRGM	+	chr5	150223050	150223465
chr5	150471638	150476638	TNIP1	-	chr5	150475055	150475561
chr5	150588211	150593211	GM2A	+	chr5	150590802	150591204
chr5	151065226	151070226	SPARC	-	chr5	151068000	151068631
chr5	158756395	158761395	IL12B	-	chr5	158759955	158760470
chr5	169655951	169660951	C5orf58	+	chr5	169656101	169656434
chr5	170843160	170848160	FGF18	+	chr5	170845713	170846266
chr5	174900565	174905565	SFXN1	+	chr5	174901876	174902365
chr5	176070888	176075888	TSPAN17	+	chr5	176072362	176072687
chr5	176802736	176807736	SLC34A1	+	chr5	176805004	176805334
chr5	176936775	176941775	DOK3	-	chr5	176940500	176941026
chr5	176936775	176941775	DOK3	-	chr5	176936487	176937119
chr5	176942970	176947970	DDX41	-	chr5	176946293	176946651
chr5	177658292	177663292	PHYKPL	-	chr5	177657939	177658598
chr5	178421707	178426707	GRM6	-	chr5	178421749	178422408
chr5	179229888	179234888	SQSTM1	+	chr5	179233797	179234251
chr5	179232452	179237452	MGAT4B	-	chr5	179233797	179234251
chr5	179497618	179502618	RNF130	-	chr5	179501036	179501446
chr5	179634653	179639653	RASGEF1C	-	chr5	179638411	179639043
chr5	179778887	179783887	GFPT2	-	chr5	179782248	179782578
chr5	180463725	180468725	BTNL9	+	chr5	180464272	180464597
chr5	180578443	180583443	OR2V2	+	chr5	180582816	180583406
chr5	180630793	180635793	TRIM7	-	chr5	180630457	180630906
chr6	2984721	2989721	NQO2	+	chr6	2988241	2988602
chr6	3750760	3755760	PXDC1	-	chr6	3752901	3753487
chr6	7723530	7728530	BMP6	+	chr6	7726801	7727214
chr6	10418392	10423392	TFAP2A	-	chr6	10422244	10422616
chr6	10488956	10493956	GCNT2	+	chr6	10492418	10493076
chr6	10719648	10724648	TMEM14C	+	chr6	10719247	10719724
chr6	10880674	10885674	GCM2	-	chr6	10881692	10882182
chr6	13327315	13332315	TBC1D7	-	chr6	13328346	13328890

chr6	18121351	18126351	NHLRC1	-	chr6	18125339	18125962
chr6	18152060	18157060	KDM1B	+	chr6	18152804	18153228
chr6	20398898	20403898	E2F3	+	chr6	20398758	20399154
chr6	24122850	24127850	NRSN1	+	chr6	24126186	24126610
chr6	24663763	24668763	ACOT13	+	chr6	24666500	24666992
chr6	24665761	24670761	TDP2	-	chr6	24666500	24666992
chr6	25881014	25886014	SLC17A3	-	chr6	25884183	25884606
chr6	26030788	26035788	HIST1H3B	-	chr6	26033235	26033573
chr6	26032296	26037296	HIST1H2AB	-	chr6	26033235	26033573
chr6	26284262	26289262	HIST1H4H	-	chr6	26284006	26284355
chr6	27439397	27444397	ZNF184	-	chr6	27442379	27442724
chr6	28223598	28228598	NKAPL	+	chr6	28226900	28227524
chr6	28225511	28230511	ZKSCAN4	-	chr6	28226900	28227524
chr6	28322548	28327548	ZSCAN31	-	chr6	28326036	28326493
chr6	28366011	28371011	ZSCAN12	-	chr6	28366235	28366680
chr6	29421458	29426458	OR2H1	+	chr6	29425993	29426317
chr6	29423348	29428348	OR11A1	-	chr6	29425993	29426317
chr6	29454238	29459238	MAS1L	-	chr6	29459211	29460005
chr6	29647387	29652387	ZFP57	-	chr6	29649544	29649855
chr6	30290756	30295756	TRIM39	+	chr6	30291284	30291601
chr6	30309408	30314408	RPP21	+	chr6	30309481	30309802
chr6	30453744	30458744	HLA-E	+	chr6	30454235	30454570
chr6	30611316	30616316	C6orf136	+	chr6	30614561	30614929
chr6	30840698	30845698	DDR1	+	chr6	30843787	30844221
chr6	30898452	30903452	SFTA2	-	chr6	30901845	30902300
chr6	31078836	31083836	C6orf15	-	chr6	31080498	31081049
chr6	31079027	31084027	PSORS1C1	+	chr6	31080498	31081049
chr6	31147008	31152008	POU5F1	-	chr6	31151086	31151517
chr6	31459158	31464158	MICB	+	chr6	31461673	31461988
chr6	31706040	31711040	CLIC1	-	chr6	31710381	31710870
chr6	31862062	31867062	C2	+	chr6	31865390	31865832
chr6	31863964	31868964	EHMT2	-	chr6	31865390	31865832

chr6	31891975	31896975	CFB	+	chr6	31896926	31897526
chr6	31891975	31896975	CFB	+	chr6	31896926	31897526
chr6	32002542	32007542	CYP21A2	+	chr6	32005098	32005470
chr6	32144373	32149373	AGPAT1	-	chr6	32148690	32149046
chr6	32907347	32912347	HLA-DMB	-	chr6	32907979	32908381
chr6	32935371	32940371	HLA-DMA	-	chr6	32938749	32939121
chr6	33280664	33285664	TAPBP	-	chr6	33282947	33283252
chr6	33755413	33760413	LEMD2	-	chr6	33757731	33758049
chr6	34756357	34761357	UHRF1BP1	+	chr6	34756681	34757329
chr6	35223186	35228186	ZNF76	+	chr6	35226645	35226974
chr6	36409166	36414166	PXT1	-	chr6	36412433	36413152
chr6	36513747	36518747	STK38	-	chr6	36514779	36515385
chr6	36640805	36645805	CDKN1A	+	chr6	36642349	36642660
chr6	37397495	37402495	CMTR1	+	chr6	37399957	37400481
chr6	37466198	37471198	CCDC167	-	chr6	37469736	37470099
chr6	39280829	39285829	KCNK17	-	chr6	39282033	39282436
chr6	41017543	41022543	APOBEC2	+	chr6	41018927	41019316
chr6	41120575	41125575	TREML1	-	chr6	41121443	41121811
chr6	43391604	43396604	ABCC10	+	chr6	43391963	43392278
chr6	43540387	43545387	POLH	+	chr6	43540649	43541146
chr6	43542312	43547312	XPO5	-	chr6	43545675	43545982
chr6	43964817	43969817	C6orf223	+	chr6	43969600	43969928
chr6	44279563	44284563	AARS2	-	chr6	44284006	44284629
chr6	44279563	44284563	AARS2	-	chr6	44279523	44279883
chr6	45344190	45349190	SUPT3H	-	chr6	45345491	45346512
chr6	45344190	45349190	SUPT3H	-	chr6	45346969	45347306
chr6	46757627	46762627	MEP1A	+	chr6	46760604	46761356
chr6	47442025	47447025	CD2AP	+	chr6	47446161	47446502
chr6	52440213	52445213	TRAM2	-	chr6	52440012	52440455
chr6	52532407	52537407	TMEM14A	+	chr6	52532948	52533274
chr6	52532407	52537407	TMEM14A	+	chr6	52536027	52536353
chr6	52709393	52714393	GSTA5	-	chr6	52711528	52712204

chr6	64342225	64347225	PHF3	+	chr6	64346276	64346592
chr6	71011286	71016286	COL9A1	-	chr6	71014945	71015402
chr6	74232020	74237020	EEF1A1	-	chr6	74234485	74235241
chr6	74362378	74367378	SLC17A5	-	chr6	74363060	74363416
chr6	78171990	78176990	HTR1B	-	chr6	78171986	78172298
chr6	85472737	85477737	TBX18	-	chr6	85473375	85473742
chr6	86352010	86357010	SYNCRIP	-	chr6	86353502	86353955
chr6	90061067	90066067	UBE2J1	-	chr6	90063103	90063625
chr6	90527942	90532942	MDN1	-	chr6	90529357	90529669
chr6	96021919	96026919	MANEA	+	chr6	96023139	96023534
chr6	97369105	97374105	KLHL32	+	chr6	97370240	97370578
chr6	100051106	100056106	PRDM13	+	chr6	100051770	100052099
chr6	105626370	105631370	POPDC3	-	chr6	105627252	105627692
chr6	107779268	107784268	PDSS2	-	chr6	107782932	107783279
chr6	108580964	108585964	SNX3	-	chr6	108581606	108582078
chr6	109166119	109171119	ARMC2	+	chr6	109169961	109170724
chr6	110677975	110682975	METTL24	-	chr6	110679330	110679874
chr6	110735265	110740265	DDO	-	chr6	110738673	110739048
chr6	111299718	111304718	RPF2	+	chr6	111300708	111301144
chr6	112371775	112376775	WISP3	+	chr6	112373890	112374271
chr6	112665032	112670032	RFPL4B	+	chr6	112668179	112668500
chr6	116380421	116385421	FRK	-	chr6	116381152	116381500
chr6	116998850	117003850	KPNA5	+	chr6	116998806	116999303
chr6	118225189	118230189	SLC35F1	+	chr6	118226759	118227066
chr6	119469052	119474052	FAM184A	-	chr6	119472058	119472419
chr6	123097120	123102120	FABP7	+	chr6	123097732	123098067
chr6	123106815	123111815	SMPDL3A	+	chr6	123110365	123110828
chr6	123956738	123961738	TRDN	-	chr6	123960491	123960874
chr6	130685070	130690070	SAMD3	-	chr6	130689854	130690181
chr6	132721184	132726184	MOXD1	-	chr6	132725118	132725550
chr6	133118201	133123201	SLC18B1	-	chr6	133120803	133121177
chr6	133118201	133123201	SLC18B1	-	chr6	133119875	133120206

chr6	134637750	134642750	SGK1	-	chr6	134642675	134643040
chr6	135817414	135822414	AHI1	-	chr6	135817116	135817608
chr6	139346319	139351319	ABRACL	+	chr6	139350121	139350500
chr6	142408436	142413436	NMBR	-	chr6	142411821	142412443
chr6	147826563	147831563	SAMD5	+	chr6	147828141	147828451
chr6	148589940	148594940	SASH1	+	chr6	148593269	148593609
chr6	150281643	150286643	ULBP1	+	chr6	150281609	150281986
chr6	150917499	150922499	PLEKHG1	+	chr6	150921019	150921387
chr6	153068433	153073433	VIP	+	chr6	153070309	153070689
chr6	157095563	157100563	ARID1B	+	chr6	157100143	157100635
chr6	157743133	157748133	TMEM242	-	chr6	157747943	157748333
chr6	157798665	157803665	ZDHHC14	+	chr6	157799958	157800308
chr6	157798665	157803665	ZDHHC14	+	chr6	157801999	157802378
chr6	160177860	160182860	ACAT2	+	chr6	160181214	160182063
chr6	160182061	160187061	SOD2	-	chr6	160181214	160182063
chr6	163147303	163152303	PARK2	-	chr6	163149940	163150421
chr7	189469	194469	FAM20C	+	chr7	191910	192217
chr7	762838	767838	HEATR2	+	chr7	765878	766688
chr7	765787	770787	PRKAR1B	-	chr7	765878	766688
chr7	993543	998543	ADAP1	-	chr7	993783	994100
chr7	1080712	1085712	GPR146	+	chr7	1082132	1082658
chr7	1543989	1548989	INTS1	-	chr7	1548270	1549231
chr7	1598957	1603957	TMEM184A	-	chr7	1603586	1603897
chr7	1598957	1603957	TMEM184A	-	chr7	1598726	1599143
chr7	1732461	1737461	AC074389.6	-	chr7	1734712	1735041
chr7	2668085	2673085	TTYH3	+	chr7	2670157	2670515
chr7	5226319	5231319	WIPI2	+	chr7	5229786	5230293
chr7	5310500	5315500	SLC29A4	+	chr7	5313969	5314476
chr7	5463545	5468545	TNRC18	-	chr7	5465793	5466158
chr7	5934856	5939856	CCZ1	+	chr7	5937365	5938323
chr7	6519422	6524422	FLJ20306	+	chr7	6523735	6524397
chr7	6522321	6527321	DAGLB	-	chr7	6523735	6524397

chr7	6522373	6527373	KDELR2	-	chr7	6523735	6524397
chr7	6626148	6631148	C7orf26	+	chr7	6629773	6630351
chr7	8300817	8305817	ICA1	-	chr7	8301954	8302307
chr7	17978624	17983624	SNX13	-	chr7	17983290	17983614
chr7	22671044	22676044	STEAP1B	-	chr7	22671276	22671623
chr7	22762003	22767003	IL6	+	chr7	22764039	22764359
chr7	23508586	23513586	IGF2BP3	-	chr7	23509184	23509654
chr7	24609387	24614387	MPP6	+	chr7	24612279	24612903
chr7	27188722	27193722	HOXA6	-	chr7	27190500	27190859
chr7	27190700	27195700	HOXA3	-	chr7	27190500	27190859
chr7	27190700	27195700	HOXA3	-	chr7	27194416	27195221
chr7	27701114	27706114	HIBADH	-	chr7	27704705	27705094
chr7	30063520	30068520	PLEKHA8	+	chr7	30064190	30064542
chr7	30630797	30635797	GARS	+	chr7	30635144	30635567
chr7	30889510	30894510	AQP1	+	chr7	30890888	30891313
chr7	30889510	30894510	AQP1	+	chr7	30890888	30891313
chr7	30974784	30979784	GHRHR	+	chr7	30975142	30975621
chr7	32932243	32937243	KBTBD2	-	chr7	32931901	32932470
chr7	32993517	32998517	FKBP9	+	chr7	32997550	32998198
chr7	32993517	32998517	FKBP9	+	chr7	32996494	32996819
chr7	34698272	34703272	AC005493.1	-	chr7	34700403	34700767
chr7	34698272	34703272	AC005493.1	-	chr7	34701910	34702250
chr7	36425915	36430915	ANLN	+	chr7	36428519	36428836
chr7	36428234	36433234	KIAA0895	-	chr7	36428519	36428836
chr7	38063797	38068797	SFRP4	-	chr7	38066709	38067067
chr7	39602475	39607475	YAE1D1	+	chr7	39606328	39606957
chr7	39659582	39664582	RALA	+	chr7	39660144	39660470
chr7	39986136	39991136	CDK13	+	chr7	39991095	39991434
chr7	43148698	43153698	HECW1	+	chr7	43151431	43151736
chr7	43618857	43623857	STK17A	+	chr7	43620789	43621237
chr7	43962537	43967537	UBE2D4	+	chr7	43965034	43965400
chr7	43964510	43969510	URGCP	-	chr7	43965034	43965400

chr7	44372676	44377676	CAMK2B	-	chr7	44373518	44373901
chr7	45017197	45022197	MYO1G	-	chr7	45017348	45017851
chr7	45610239	45615239	ADCY1	+	chr7	45614333	45614653
chr7	50859659	50864659	GRB10	-	chr7	50861133	50861438
chr7	55951470	55956470	MRPS17	+	chr7	55951601	55951975
chr7	55951669	55956669	ZNF713	+	chr7	55951601	55951975
chr7	56182593	56187593	NUPR1L	-	chr7	56183714	56184173
chr7	62805739	62810739	AC006455.1	+	chr7	62810622	62810939
chr7	65576091	65581091	CRCP	+	chr7	65576478	65576786
chr7	66090368	66095368	KCTD7	+	chr7	66093999	66094329
chr7	72721364	72726364	NSUN5	-	chr7	72725509	72725914
chr7	73255365	73260365	WBSCR27	-	chr7	73259638	73260036
chr7	73585075	73590075	EIF4H	+	chr7	73587370	73587704
chr7	75003925	75008925	AC006014.1	-	chr7	75008085	75008601
chr7	75675822	75680822	STYXL1	-	chr7	75679302	75679828
chr7	75986848	75991848	YWHAG	-	chr7	75989731	75990069
chr7	76023335	76028335	ZP3	+	chr7	76027436	76027808
chr7	76136245	76141245	UPK3B	+	chr7	76137925	76138253
chr7	77426397	77431397	TMEM60	-	chr7	77429187	77429522
chr7	79081390	79086390	MAGI2	-	chr7	79081114	79081498
chr7	86687515	86692515	KIAA1324L	-	chr7	86688572	86689038
chr7	87502031	87507031	DBF4	+	chr7	87506315	87506766
chr7	87504172	87509172	SLC25A40	-	chr7	87506315	87506766
chr7	90890283	90895283	FZD1	+	chr7	90894055	90894603
chr7	92464408	92469408	CDK6	-	chr7	92466642	92466961
chr7	93202542	93207542	CALCR	-	chr7	93207443	93207804
chr7	94020373	94025373	COL1A2	+	chr7	94025084	94025526
chr7	95398366	95403366	DYNC1I1	+	chr7	95399926	95400762
chr7	96131335	96136335	C7orf76	-	chr7	96136196	96136581
chr7	98740223	98745223	SMURF1	-	chr7	98742083	98742659
chr7	99033045	99038045	CPSF4	+	chr7	99034145	99035028
chr7	99062287	99067287	PTCD1	-	chr7	99064132	99064839

chr7	99062320	99067320	ATP5J2-PTCD1	-	chr7	99064132	99064839
chr7	99062454	99067454	ATP5J2	-	chr7	99064132	99064839
chr7	99066964	99071964	ZNF789	+	chr7	99067946	99068491
chr7	99609704	99614704	ZKSCAN1	+	chr7	99610825	99611190
chr7	99643890	99648890	ZSCAN21	+	chr7	99647209	99647584
chr7	99754838	99759838	C7orf43	-	chr7	99759379	99759750
chr7	99771686	99776686	STAG3	+	chr7	99774919	99775693
chr7	99773495	99778495	GPC2	-	chr7	99774919	99775693
chr7	99868337	99873337	GATS	-	chr7	99870131	99870596
chr7	99868355	99873355	GATS	-	chr7	99870131	99870596
chr7	100078050	100083050	NYAP1	+	chr7	100081553	100082032
chr7	100182276	100187276	LRCH4	-	chr7	100184266	100184794
chr7	100182276	100187276	LRCH4	-	chr7	100185832	100186247
chr7	100196300	100201300	PCOLCE	+	chr7	100198190	100198503
chr7	100196300	100201300	PCOLCE	+	chr7	100199819	100200289
chr7	100252584	100257584	ACTL6B	-	chr7	100253950	100254478
chr7	100461260	100466260	TRIP6	+	chr7	100463394	100463832
chr7	100609404	100614404	MUC12	+	chr7	100612366	100613369
chr7	101271076	101276076	MYL10	-	chr7	101275151	101275478
chr7	102255704	102260704	RASA4	-	chr7	102256992	102257369
chr7	102287272	102292272	SPDYE2B	+	chr7	102287809	102288226
chr7	103846995	103851995	ORC5	-	chr7	103851010	103851385
chr7	105925272	105930272	NAMPT	-	chr7	105924922	105925329
chr7	107106963	107111963	GPR22	+	chr7	107109824	107110182
chr7	108206512	108211512	DNAJB9	+	chr7	108208363	108209023
chr7	108208610	108213610	PNPLA8	-	chr7	108208363	108209023
chr7	108208694	108213694	THAP5	-	chr7	108208363	108209023
chr7	111201073	111206073	IMMP2L	-	chr7	111201679	111202025
chr7	111844966	111849966	DOCK4	-	chr7	111848504	111848994
chr7	112578471	112583471	C7orf60	-	chr7	112578290	112578726
chr7	116961843	116966843	WNT2	-	chr7	116962733	116963180
chr7	120625231	120630231	CPED1	+	chr7	120625659	120626102

chr7	120625231	120630231	CPED1	+	chr7	120627404	120627725
chr7	121949245	121954245	FEZF1	-	chr7	121950885	121951489
chr7	123561786	123566786	SPAM1	+	chr7	123566680	123566992
chr7	126891848	126896848	GRM8	-	chr7	126894251	126894836
chr7	127224899	127229899	ARF5	+	chr7	127227593	127228029
chr7	127227963	127232963	FSCN3	+	chr7	127227593	127228029
chr7	127232165	127237165	GCC1	-	chr7	127235149	127235616
chr7	128466931	128471931	FLNC	+	chr7	128467234	128467882
chr7	129248055	129253055	NRF1	+	chr7	129250897	129251259
chr7	129591289	129596289	UBE2H	-	chr7	129592716	129593026
chr7	130370768	130375768	TSGA13	-	chr7	130373204	130373955
chr7	135193375	135198375	CNOT4	-	chr7	135198042	135198543
chr7	135608042	135613042	AC015987.2	+	chr7	135608987	135609293
chr7	137530338	137535338	DGKI	-	chr7	137534374	137534741
chr7	139166958	139171958	KLRG2	-	chr7	139168421	139169184
chr7	140369453	140374453	ADCK2	+	chr7	140369104	140369668
chr7	141400453	141405453	KIAA1147	-	chr7	141401564	141401904
chr7	141434621	141439621	SSBP1	+	chr7	141435798	141436540
chr7	142370604	142375604	MTRNR2L6	+	chr7	142374677	142375027
chr7	142582007	142587007	TRPV6	-	chr7	142586630	142587046
chr7	142915630	142920630	TAS2R40	+	chr7	142919155	142919494
chr7	142917860	142922860	AC073342.1	-	chr7	142919155	142919494
chr7	142973550	142978550	TMEM139	+	chr7	142978375	142979122
chr7	143989730	143994730	ARHGEF35	-	chr7	143990924	143991500
chr7	148889077	148894077	ZNF282	+	chr7	148890842	148891184
chr7	149156714	149161714	ZNF777	-	chr7	149158011	149158324
chr7	149408372	149413372	KRBA1	+	chr7	149408907	149409481
chr7	149566557	149571557	ATP6V0E2	+	chr7	149568232	149568796
chr7	150327973	150332973	GIMAP6	-	chr7	150330309	150330887
chr7	150379285	150384285	GIMAP2	+	chr7	150383945	150384345
chr7	150776453	150781453	FASTK	-	chr7	150777657	150778010
chr7	150779133	150784133	TMUB1	-	chr7	150781977	150782327

chr7	150779418	150784418	AGAP3	+	chr7	150781977	150782327
chr7	150870332	150875332	GBX1	-	chr7	150874133	150874506
chr7	151572710	151577710	PRKAG2	-	chr7	151574707	151575072
chr7	152453334	152458334	ACTR3B	+	chr7	152455767	152456096
chr7	155171271	155176271	AC008060.7	+	chr7	155176034	155176364
chr7	155247324	155252324	EN2	+	chr7	155251673	155252045
chr7	155247324	155252324	EN2	+	chr7	155250022	155250503
chr7	156684424	156689424	LMBR1	-	chr7	156685665	156686050
chr7	156801845	156806845	MNX1	-	chr7	156801780	156802350
chr7	158621444	158626444	ESYT2	-	chr7	158625078	158625481
chr8	6419430	6424430	ANGPT2	-	chr8	6419460	6419768
chr8	6782098	6787098	DEFA6	-	chr8	6782575	6782905
chr8	6794360	6799360	DEFA4	-	chr8	6797298	6797631
chr8	6836102	6841102	DEFA1	-	chr8	6837539	6837907
chr8	7241580	7246580	ZNF705G	-	chr8	7245547	7245894
chr8	7349868	7354868	DEFB107B	+	chr8	7353734	7354235
chr8	7679194	7684194	DEFB106A	+	chr8	7679080	7679448
chr8	7690493	7695493	DEFB104A	+	chr8	7694610	7695019
chr8	8242508	8247508	SGK223	-	chr8	8243955	8244272
chr8	11623648	11628648	NEIL2	+	chr8	11628511	11629386
chr8	11649582	11654582	FDFT1	+	chr8	11652228	11652613
chr8	11658577	11663577	RP11-297N6.4	-	chr8	11660364	11660784
chr8	11830608	11835608	DEFB136	-	chr8	11832648	11833093
chr8	11830608	11835608	DEFB136	-	chr8	11830679	11831157
chr8	11871543	11876543	RP11-481A20.11	-	chr8	11873722	11874431
chr8	12050142	12055142	FAM86B1	-	chr8	12055128	12055873
chr8	12292415	12297415	FAM86B2	-	chr8	12294113	12294424
chr8	13420852	13425852	C8orf48	+	chr8	13424244	13424638
chr8	21878136	21883136	NPM2	+	chr8	21878040	21878410
chr8	21903006	21908006	DMTN	+	chr8	21904999	21905430
chr8	21965432	21970432	NUDT18	-	chr8	21970122	21970487
chr8	21965432	21970432	NUDT18	-	chr8	21967239	21967709

chr8	22549315	22554315	EGR3	-	chr8	22552832	22553199
chr8	22856013	22861013	PEBP4	-	chr8	22860588	22861072
chr8	23020043	23025043	TNFRSF10D	-	chr8	23023164	23023470
chr8	23081139	23086139	TNFRSF10A	-	chr8	23081486	23081865
chr8	23313708	23318708	ENTPD4	-	chr8	23313216	23313787
chr8	23426657	23431657	FP15737	+	chr8	23428569	23429052
chr8	23562611	23567611	NKX2-6	-	chr8	23564614	23564920
chr8	24767025	24772025	NEFM	+	chr8	24770595	24771001
chr8	26370108	26375108	PNMA2	-	chr8	26370508	26370830
chr8	27165499	27170499	PTK2B	+	chr8	27166446	27167055
chr8	27471048	27476048	CLU	-	chr8	27474512	27474818
chr8	27487885	27492885	SCARA3	+	chr8	27490788	27491105
chr8	27487885	27492885	SCARA3	+	chr8	27489762	27490271
chr8	27939888	27944888	NUGGC	-	chr8	27941206	27941764
chr8	30514268	30519268	GTF2E2	-	chr8	30514402	30514723
chr8	33423143	33428143	RNF122	-	chr8	33425777	33426255
chr8	37755485	37760485	RAB11FIP1	-	chr8	37757820	37758181
chr8	37796147	37801147	GOT1L1	-	chr8	37797127	37797590
chr8	39756294	39761294	IDO1	+	chr8	39756173	39756650
chr8	41383225	41388225	GINS4	+	chr8	41384267	41384854
chr8	42192472	42197472	POLB	+	chr8	42196345	42196827
chr8	42748575	42753575	HOOK3	+	chr8	42749735	42750073
chr8	42992056	42997056	HGSNAT	+	chr8	42996204	42996597
chr8	48650148	48655148	CEBPD	-	chr8	48651027	48651410
chr8	49832799	49837799	SNAI2	-	chr8	49833952	49834347
chr8	53372019	53377019	ST18	-	chr8	53373619	53373990
chr8	54162757	54167757	OPRK1	-	chr8	54162554	54163412
chr8	55366995	55371995	SOX17	+	chr8	55370803	55371138
chr8	61425916	61430916	RAB2A	+	chr8	61430211	61430670
chr8	65489314	65494314	BHLHE22	+	chr8	65489326	65489710
chr8	69239457	69244457	C8orf34	+	chr8	69241328	69241789
chr8	70745799	70750799	SLCO5A1	-	chr8	70747677	70747998

chr8	70846903	70851903	AC090574.1	+	chr8	70849162	70849507
chr8	71482181	71487181	AC120194.1	+	chr8	71483680	71484025
chr8	71517312	71522312	RP11-382J12.1	+	chr8	71517353	71517737
chr8	72986352	72991352	TRPA1	-	chr8	72990473	72990864
chr8	77911780	77916780	PEX2	-	chr8	77912125	77912587
chr8	82442113	82447113	FABP12	-	chr8	82445955	82446338
chr8	87331875	87336875	SLC7A13	-	chr8	87333792	87334334
chr8	95485837	95490837	RAD54B	-	chr8	95485909	95486367
chr8	95960139	95965139	TP53INP1	-	chr8	95961469	95961877
chr8	97246362	97251362	UQCRB	-	chr8	97250320	97250649
chr8	101964116	101969116	YWHAZ	-	chr8	101964385	101965022
chr8	103537468	103542468	KB-1980E6.3	+	chr8	103537043	103537564
chr8	103537468	103542468	KB-1980E6.3	+	chr8	103538096	103538538
chr8	103560300	103565300	ODF1	+	chr8	103559910	103560320
chr8	103904592	103909592	AZIN1	-	chr8	103906799	103907437
chr8	104029791	104034791	ATP6V1C1	+	chr8	104033556	104034005
chr8	104029791	104034791	ATP6V1C1	+	chr8	104034733	104035053
chr8	105347815	105352815	DCSTAMP	+	chr8	105352146	105352693
chr8	118529452	118534452	MED30	+	chr8	118529415	118529923
chr8	119122592	119127592	EXT1	-	chr8	119123632	119124070
chr8	124252138	124257138	C8orf76	-	chr8	124254440	124254839
chr8	124551946	124556946	FBXO32	-	chr8	124556195	124556558
chr8	125739230	125744230	MTSS1	-	chr8	125741894	125742222
chr8	131027875	131032875	FAM49B	-	chr8	131030752	131031105
chr8	133121906	133126906	HHLA1	-	chr8	133124893	133125519
chr8	133491700	133496700	KCNQ3	-	chr8	133494104	133494420
chr8	133771458	133776458	TMEM71	-	chr8	133773367	133773750
chr8	136466200	136471200	KHDRBS3	+	chr8	136468467	136468941
chr8	141644218	141649218	AGO2	-	chr8	141648712	141649182
chr8	142123877	142128877	DENND3	+	chr8	142123665	142124078
chr8	143778029	143783029	LY6K	+	chr8	143782800	143783223
chr8	143805121	143810121	THEM6	+	chr8	143807435	143807814

chr8	143822329	143827329	SLURP1	-	chr8	143824219	143824628
chr8	143866508	143871508	LY6D	-	chr8	143866707	143867129
chr8	144095899	144100899	LY6E	+	chr8	144095679	144095994
chr8	144346103	144351103	GLI4	+	chr8	144348874	144349374
chr8	144440649	144445649	TOP1MT	-	chr8	144444991	144445752
chr8	144622123	144627123	ZC3H3	-	chr8	144623821	144624145
chr8	144653641	144658641	MROH6	-	chr8	144654317	144654693
chr8	144714683	144719683	ZNF623	+	chr8	144717366	144717708
chr8	144951132	144956132	EPPK1	-	chr8	144952954	144953625
chr8	145083082	145088082	SPATC1	+	chr8	145083904	145084278
chr8	145146430	145151430	CYC1	+	chr8	145147373	145147809
chr8	145146430	145151430	CYC1	+	chr8	145149183	145149491
chr8	145574295	145579295	SLC52A2	+	chr8	145575894	145576320
chr8	145574295	145579295	SLC52A2	+	chr8	145574759	145575112
chr8	145640779	145645779	SLC39A4	-	chr8	145643040	145643458
chr8	145689560	145694560	CYHR1	-	chr8	145693019	145693515
chr8	145699852	145704852	PPP1R16A	+	chr8	145701500	145702026
chr8	145700218	145705218	FOXH1	-	chr8	145701500	145702026
chr8	145724856	145729856	GPT	+	chr8	145727614	145727997
chr8	146049349	146054349	ZNF7	+	chr8	146050742	146051119
chr8	146274264	146279264	C8orf33	+	chr8	146276722	146277130
chr9	116917	121917	FOXD4	-	chr9	118215	118577
chr9	4658798	4663798	PPAPDC2	+	chr9	4662274	4662649
chr9	5625525	5630525	KIAA1432	+	chr9	5628869	5629324
chr9	5831617	5836617	ERMP1	-	chr9	5832911	5833306
chr9	18470392	18475392	ADAMTSL1	+	chr9	18474749	18475094
chr9	19045872	19050872	RRAGA	+	chr9	19049516	19049961
chr9	19226933	19231933	DENND4C	+	chr9	19228466	19228827
chr9	21140644	21145644	IFNW1	-	chr9	21140651	21140971
chr9	27105639	27110639	TEK	+	chr9	27110391	27110782
chr9	27520812	27525812	IFNK	+	chr9	27523959	27524526
chr9	32563287	32568287	GVQW1	+	chr9	32567329	32567666

chr9	33075165	33080165	SMU1	-	chr9	33077741	33078129
chr9	33401143	33406143	AQP7	-	chr9	33405026	33405449
chr9	34662545	34667545	CCL27	-	chr9	34665796	34666196
chr9	34664609	34669609	RP11-195F19.5	-	chr9	34665796	34666196
chr9	35359762	35364762	AL160274.1	-	chr9	35363861	35364444
chr9	35645307	35650307	CD72	-	chr9	35646062	35646375
chr9	35745703	35750703	RGP1	+	chr9	35747119	35747851
chr9	35810769	35815769	SPAG8	-	chr9	35814678	35815125
chr9	35810948	35815948	TMEM8B	+	chr9	35814678	35815125
chr9	35813851	35818851	HINT2	-	chr9	35814678	35815125
chr9	36032930	36037930	RECK	+	chr9	36034119	36034590
chr9	36032930	36037930	RECK	+	chr9	36036531	36036885
chr9	37587371	37592371	FBXO10	-	chr9	37587441	37587758
chr9	39881475	39886475	SPATA31A2	+	chr9	39886458	39886811
chr9	42364803	42369803	ANKRD20A2	+	chr9	42368756	42369109
chr9	45723607	45728607	FAM27A	+	chr9	45726912	45727304
chr9	46385610	46390610	FAM27D1	+	chr9	46390538	46391099
chr9	70643089	70648089	AL591479.1	+	chr9	70646762	70647098
chr9	71317075	71322075	PIP5K1B	+	chr9	71316727	71317276
chr9	71732709	71737709	TJP2	+	chr9	71735449	71735788
chr9	72870437	72875437	SMC5	+	chr9	72871670	72871984
chr9	74060320	74065320	TRPM3	-	chr9	74062097	74062517
chr9	74726011	74731011	GDA	+	chr9	74729297	74729656
chr9	74978663	74983663	ZFAND5	-	chr9	74982558	74983142
chr9	77566302	77571302	C9orf40	-	chr9	77567794	77568247
chr9	78502060	78507060	PCSK5	+	chr9	78505662	78505988
chr9	79519503	79524503	PRUNE2	-	chr9	79523937	79524464
chr9	86151961	86156961	FRMD3	-	chr9	86151919	86152278
chr9	86321618	86326618	UBQLN1	-	chr9	86325846	86326339
chr9	87279966	87284966	NTRK2	+	chr9	87282873	87283221
chr9	90494241	90499241	SPATA31E1	+	chr9	90496033	90496353
chr9	93403886	93408886	DIRAS2	-	chr9	93404106	93404459

chr9	95086418	95091418	NOL8	-	chr9	95091026	95091373
chr9	95817489	95822489	SUSD3	+	chr9	95822148	95822471
chr9	95895070	95900070	NINJ1	-	chr9	95896144	95896565
chr9	95943698	95948698	WNK2	+	chr9	95944683	95945063
chr9	96107196	96112196	C9orf129	-	chr9	96107989	96108398
chr9	96335189	96340189	PHF2	+	chr9	96336814	96337207
chr9	97089426	97094426	NUTM2F	-	chr9	97092674	97093096
chr9	97401031	97406031	FBP1	-	chr9	97401940	97402296
chr9	99062934	99067934	HSD17B3	-	chr9	99066067	99066537
chr9	99380612	99385612	CDC14B	-	chr9	99380703	99381014
chr9	99538911	99543911	ZNF510	-	chr9	99541058	99541424
chr9	100259962	100264962	TMOD1	+	chr9	100264383	100264688
chr9	101557747	101562747	ANKS6	-	chr9	101557615	101558301
chr9	108003403	108008403	SLC44A1	+	chr9	108006752	108007123
chr9	110251263	110256263	KLF4	-	chr9	110253086	110253559
chr9	111692961	111697961	FAM206A	+	chr9	111694767	111695263
chr9	111694896	111699896	IKBKAP	-	chr9	111694767	111695263
chr9	111774309	111779309	CTNNAL1	-	chr9	111774394	111774768
chr9	112259090	112264090	PTPN3	-	chr9	112262021	112262474
chr9	112399568	112404568	PALM2	+	chr9	112401050	112401448
chr9	114420115	114425115	GNG10	+	chr9	114420382	114420805
chr9	115245627	115250627	KIAA1958	+	chr9	115249278	115249621
chr9	115247984	115252984	C9orf147	-	chr9	115249278	115249621
chr9	115982141	115987141	FKBP15	-	chr9	115986350	115986784
chr9	116914340	116919340	COL27A1	+	chr9	116917968	116918477
chr9	123966572	123971572	GSN	+	chr9	123969181	123969674
chr9	124131031	124136031	STOM	-	chr9	124133052	124133422
chr9	125147428	125152428	AL162424.1	-	chr9	125147383	125147697
chr9	125311891	125316891	OR1N2	+	chr9	125311881	125312212
chr9	125699612	125704612	RABGAP1	+	chr9	125704385	125704700
chr9	127176223	127181223	PSMB7	-	chr9	127177029	127177352
chr9	127176223	127181223	PSMB7	-	chr9	127179051	127179368

chr9	127209923	127214923	GPR144	+	chr9	127212608	127212963
chr9	127535937	127540937	OLFML2A	+	chr9	127538289	127539209
chr9	127620909	127625909	ARPC5L	+	chr9	127623880	127624299
chr9	127622760	127627760	RPL35	-	chr9	127623880	127624299
chr9	129619444	129624444	ZBTB34	+	chr9	129623204	129623653
chr9	129673553	129678553	RALGPS1	+	chr9	129677549	129677910
chr9	129983044	129988044	GARNL3	+	chr9	129986980	129987418
chr9	130210265	130215265	LRSAM1	+	chr9	130214090	130214435
chr9	130212184	130217184	RPL12	-	chr9	130214090	130214435
chr9	130485652	130490652	PTRH1	-	chr9	130488850	130489162
chr9	130539520	130544520	SH2D3C	-	chr9	130542390	130543076
chr9	130553376	130558376	FPGS	+	chr9	130553898	130554292
chr9	130889241	130894241	PTGES2	-	chr9	130893523	130893870
chr9	130919039	130924039	C9orf16	+	chr9	130918886	130919218
chr9	131083521	131088521	TRUB2	-	chr9	131088398	131089289
chr9	131577253	131582253	ENDOG	+	chr9	131581448	131581827
chr9	131679674	131684674	PHYHD1	+	chr9	131682495	131683184
chr9	131839879	131844879	DOLPP1	+	chr9	131840687	131841047
chr9	132381555	132386555	C9orf50	-	chr9	132385892	132386229
chr9	132561932	132566932	TOR1B	+	chr9	132562514	132562921
chr9	132561932	132566932	TOR1B	+	chr9	132563744	132564106
chr9	132596642	132601642	C9orf78	-	chr9	132599436	132600092
chr9	132931357	132936357	NCS1	+	chr9	132933729	132934140
chr9	133316816	133321816	ASS1	+	chr9	133320543	133320880
chr9	133767725	133772725	QRFP	-	chr9	133769315	133769665
chr9	133997448	134002448	NUP214	+	chr9	133999583	134000016
chr9	134161581	134166581	PPAPDC3	+	chr9	134161109	134161711
chr9	134265980	134270980	PRRC2B	+	chr9	134265619	134266189
chr9	135033834	135038834	NTNG2	+	chr9	135038204	135038611
chr9	136037801	136042801	RALGDS	-	chr9	136039955	136040310
chr9	136037832	136042832	GBGT1	-	chr9	136039955	136040310
chr9	136393786	136398786	ADAMTSL2	+	chr9	136398431	136398822

chr9	136497982	136502982	DBH	+	chr9	136501303	136501723
chr9	136932157	136937157	BRD3	-	chr9	136933777	136934107
chr9	138231595	138236595	C9orf62	+	chr9	138234608	138235032
chr9	138367425	138372425	PPP1R26	+	chr9	138371796	138372117
chr9	138392080	138397080	C9orf116	-	chr9	138393651	138393963
chr9	139301610	139306610	PMPCA	+	chr9	139303397	139303710
chr9	139301610	139306610	PMPCA	+	chr9	139305392	139305711
chr9	139303561	139308561	SDCCAG3	-	chr9	139303397	139303710
chr9	139303561	139308561	SDCCAG3	-	chr9	139305392	139305711
chr9	139438814	139443814	NOTCH1	-	chr9	139438914	139439413
chr9	139549808	139554808	EGFL7	+	chr9	139550671	139551450
chr9	139580375	139585375	AGPAT2	-	chr9	139585337	139585705
chr9	139836198	139841198	C8G	+	chr9	139838741	139839235
chr9	139837648	139842648	FBXW5	-	chr9	139841686	139842026
chr9	139837648	139842648	FBXW5	-	chr9	139838741	139839235
chr9	139840503	139845503	LCN12	+	chr9	139841686	139842026
chr9	139872856	139877856	LCNL1	+	chr9	139877823	139878219
chr9	140079599	140084599	SSNA1	+	chr9	140081189	140082199
chr9	140081489	140086489	ANAPC2	-	chr9	140081189	140082199
chr9	140081489	140086489	ANAPC2	-	chr9	140086230	140086623
chr9	140096647	140101647	NDOR1	+	chr9	140101610	140101943
chr9	140097145	140102145	TPRN	-	chr9	140101610	140101943
chr9	140098590	140103590	TMEM203	-	chr9	140101610	140101943
chr9	140132165	140137165	TUBB4B	+	chr9	140135887	140136271
chr9	140496606	140501606	ARRDC1	+	chr9	140499641	140499989
chrM	-193	4807	MT-ND1	+	chrM	103	16511
chrM	970	5970	MT-ND2	+	chrM	103	16511
chrM	2404	7404	MT-CO1	+	chrM	103	16511
chrM	4086	9086	MT-CO2	+	chrM	103	16511
chrM	4866	9866	MT-ATP8	+	chrM	103	16511
chrM	5027	10027	MT-ATP6	+	chrM	103	16511
chrM	5707	10707	MT-CO3	+	chrM	103	16511

chrM	6559	11559	MT-ND3	+	chrM	103	16511
chrM	6970	11970	MT-ND4L	+	chrM	103	16511
chrM	7260	12260	MT-ND4	+	chrM	103	16511
chrM	8837	13837	MT-ND5	+	chrM	103	16511
chrM	11247	16247	MT-CYB	+	chrM	103	16511
chrM	13173	18173	MT-ND6	-	chrM	103	16511
chrX	1571155	1576155	ASMTL	-	chrX	1572889	1573243
chrX	13061301	13066301	FAM9C	-	chrX	13062306	13063032
chrX	16887037	16892037	RBBP7	-	chrX	16887150	16887503
chrX	23348633	23353633	PTCHD1	+	chrX	23352484	23352877
chrX	24069333	24074333	EIF2S3	+	chrX	24073407	24073751
chrX	34148947	34153947	FAM47A	-	chrX	34149154	34149513
chrX	37635764	37640764	CYBB	+	chrX	37636176	37636505
chrX	37705390	37710390	DYNLT3	-	chrX	37705710	37706156
chrX	43831250	43836250	NDP	-	chrX	43833020	43833429
chrX	47517060	47522060	UXT	-	chrX	47517931	47518280
chrX	47986539	47991539	SPACA5B	+	chrX	47989830	47990158
chrX	48826476	48831476	KCND1	-	chrX	48831013	48831319
chrX	49302870	49307870	GAGE12D	+	chrX	49305926	49306289
chrX	49640970	49645970	USP27X	+	chrX	49642676	49643081
chrX	69478154	69483154	P2RY4	-	chrX	69479577	69479882
chrX	69498443	69503443	RAB41	+	chrX	69503396	69503708
chrX	70149475	70154475	SLC7A3	-	chrX	70150652	70150969
chrX	70334906	70339906	MED12	+	chrX	70337117	70337475
chrX	70518084	70523084	ITGB1BP2	+	chrX	70522426	70523027
chrX	76706148	76711148	FGF16	+	chrX	76706787	76707189
chrX	79266755	79271755	TBX22	+	chrX	79268060	79268722
chrX	80455941	80460941	HMGN5	-	chrX	80460142	80461055
chrX	100642312	100647312	RPL36A	+	chrX	100645052	100645407
chrX	100642499	100647499	RPL36A-HNRNP	+	chrX	100645052	100645407
chrX	100874287	100879287	ARMCX3	+	chrX	100877736	100878155
chrX	107019072	107024072	TSC22D3	-	chrX	107023946	107024305

chrX	110654103	110659103	DCX	-	chrX	110655563	110655961
chrX	118283042	118288042	KIAA1210	-	chrX	118286226	118286708
chrX	118529523	118534523	SLC25A43	+	chrX	118532797	118533126
chrX	118697897	118702897	CXorf56	-	chrX	118701440	118701817
chrX	119724365	119729365	MCTS1	+	chrX	119725375	119726101
chrX	120093837	120098837	CT47A6	-	chrX	120094843	120095226
chrX	125298580	125303580	DCAF12L2	-	chrX	125299496	125299846
chrX	125685334	125690334	DCAF12L1	-	chrX	125689659	125690014
chrX	128910455	128915455	SASH3	+	chrX	128914538	128914870
chrX	131622496	131627496	MBNL3	-	chrX	131623444	131623877
chrX	134879988	134884988	CT45A3	+	chrX	134879925	134880376
chrX	150145482	150150482	HMGB3	+	chrX	150146176	150146565
chrX	151141652	151146652	GABRE	-	chrX	151142672	151143040
chrX	151936740	151941740	MAGEA3	-	chrX	151936610	151937023
chrX	152484615	152489615	MAGEA1	-	chrX	152486717	152487058
chrX	152779634	152784634	ATP2B3	+	chrX	152779485	152779817
chrX	153522296	153527296	TEX28	-	chrX	153526352	153526716
chrX	153765914	153770914	IKBKG	+	chrX	153765848	153766190
chrX	154686776	154691776	F8A3	-	chrX	154687336	154687669
chrY	139489	144489	PLCXD1	+	chrY	141991	142301
chrY	9171573	9176573	TSPY4	+	chrY	9174333	9175042
chrY	9321422	9326422	TSPY6P	+	chrY	9324548	9324906
chrY	9361989	9366989	TSPY10	+	chrY	9366897	9367228
chrY	23546746	23551746	CYorf17	-	chrY	23548947	23549344
chrY	24062714	24067714	RBMY1E	-	chrY	24062964	24063614
chrY	24633044	24638044	PRY	+	chrY	24636287	24636861
