# Supplementary material for: The exosomal miRNA/FOXN3 axis empowers fibroblasts with tumor-promoting functions via enhanced migration and altered secretome
Source: J Nanobiotechnology. 2026 Jun 5;24:742. doi: 10.1186/s12951-026-04633-6 (PMC13459620; doi:10.1186/s12951-026-04633-6)
Supplement: Supplementary file 6 [file 12951_2026_4633_MOESM7_ESM.pdf]

Table S6 List of differentially secreted proteins (*P* value < 0.05)

Protein names	Matrisome	Matrisome subclass	LFQ (MRC5 with FOXN3 shRNAs) rep 1	LFQ (MRC5 with FOXN3 shRNAs) rep 2	LFQ (MRC5 with FOXN3 shRNAs) rep 3	LFQ (MRC5 with scrambled shRNAs) rep 1	LFQ (MRC5 with scrambled shRNAs) rep 2	LFQ (MRC5 with scrambled shRNAs) rep 3
GDF15	Matrisome-associated	Secreted Factors	350130000	346400000	328460000	182060000	186780000	204690000
S100A6	Matrisome-associated	Secreted Factors	779200000	974320000	759360000	602320000	548510000	549300000
INHBA	Matrisome-associated	Secreted Factors	112400000	111690000	108120000	78295000	97162000	83613000
FSTL3	Matrisome-associated	Secreted Factors	195680000	208670000	191300000	148560000	177300000	161890000
FST	Matrisome-associated	Secreted Factors	388190000	403220000	436900000	567740000	549210000	512500000
ANXA1	Matrisome-associated	ECM-affiliated Proteins	847180000	848230000	853660000	1061100000	1056200000	1054800000
ANXA2	Matrisome-associated	ECM-affiliated Proteins	8075900000	7706700000	8083400000	9891300000	9761700000	10172000000
ANXA5	Matrisome-associated	ECM-affiliated Proteins	479940000	472250000	508980000	568310000	631940000	661830000
CLEC3B	Matrisome-associated	ECM-affiliated Proteins	129620000	125140000	137940000	172330000	173670000	185860000
SEMA3B	Matrisome-associated	ECM-affiliated Proteins	106200000	106520000	100520000	139550000	142380000	144080000
ANXA6	Matrisome-associated	ECM-affiliated Proteins	219220000	196670000	236770000	321290000	287670000	324390000
LOXL3	Matrisome-associated	ECM Regulators	505390000	311010000	443200000	264780000	198200000	229680000
PLAU	Matrisome-associated	ECM Regulators	2072800000	2108000000	2029000000	1570100000	1570000000	1792000000
MMP1	Matrisome-associated	ECM Regulators	1976000000	1827000000	1836800000	1480400000	1595700000	1615700000
MASP1	Matrisome-associated	ECM Regulators	1712900000	1709700000	1711700000	2015000000	2129600000	2177300000
ITIH3	Matrisome-associated	ECM Regulators	133410000	143210000	159220000	198140000	164070000	186960000
SERPINB2	Matrisome-associated	ECM Regulators	39820000	43566000	40716000	55221000	51962000	55048000
SERPINB7	Matrisome-associated	ECM Regulators	357170000	371300000	295400000	391420000	467690000	483440000
F2	Matrisome-associated	ECM Regulators	142590000	138130000	143900000	172210000	198150000	210310000
PLOD2	Matrisome-associated	ECM Regulators	103420000	138550000	141900000	196110000	220200000	196810000
SERPING1	Matrisome-associated	ECM Regulators	194970000	182630000	201990000	318160000	301140000	327590000
SERPINI1	Matrisome-associated	ECM Regulators	2890500	2853700	4397100	10735000	5686500	9373200
VWA5A	Core matrisome	ECM Glycoproteins	8867800	9290400	10101000	0	0	0
SVEP1	Core matrisome	ECM Glycoproteins	694120000	706000000	711760000	527150000	493560000	540950000
MFAP4	Core matrisome	ECM Glycoproteins	1413600000	1541800000	1530500000	1083300000	1108800000	1130800000
LTBP4	Core matrisome	ECM Glycoproteins	1021000000	1062700000	1029900000	801270000	904770000	711210000
IGFBP2	Core matrisome	ECM Glycoproteins	220880000	245630000	238410000	202510000	181760000	174860000
IGFBP4	Core matrisome	ECM Glycoproteins	17303000000	15215000000	15367000000	12913000000	13025000000	13839000000
FBLN5	Core matrisome	ECM Glycoproteins	487960000	484130000	435810000	580780000	619960000	598860000
MFAP2	Core matrisome	ECM Glycoproteins	43696000	43412000	42084000	61125000	54650000	64200000
SRPX2	Core matrisome	ECM Glycoproteins	82980000	81631000	83246000	111430000	109410000	135330000

RELN	Core matrisome	ECM Glycoproteins	341670000	377270000	515850000	685150000	621450000	717440000
COL12A1	Core matrisome	Collagens	9316800000	8966000000	8437400000	11288000000	10885000000	10472000000
COL7A1	Core matrisome	Collagens	170010000	166860000	189400000	216700000	219670000	214950000
KRT5	-	-	25796000	42076000	29026000	0	0	0
RTCB	-	-	22300000	28206000	25125000	0	0	0
CRKL	-	-	19577000	21072000	24824000	0	0	0
SNRPA	-	-	16720000	15595000	22465000	0	0	0
LSAMP	-	-	14878000	17496000	20994000	0	0	0
DAP	-	-	18091000	14003000	14146000	0	0	0
RPS3A	-	-	14545000	13065000	15074000	0	0	0
SLC1A5	-	-	14484000	9376200	9204400	0	0	0
KRT16	-	-	5902900	4738900	4937800	0	0	0
KRT6A	-	-	4104000	5353800	4799000	0	0	0
DSG2	-	-	3852100	3569700	4635800	0	0	2113500
KRT2	-	-	607820000	588490000	568730000	135470000	110100000	124160000
MAGOHB	-	-	33179000	35218000	35873000	0	22876000	0
CHST14	-	-	112860000	107140000	80294000	0	0	70923000
MARCKSL1	-	-	19840000	21719000	27649000	17215000	0	0
LRP11	-	-	14898000	18525000	15263000	0	0	12168000
KRT10	-	-	1183900000	1192800000	1114600000	369430000	307740000	308100000
KRT14	-	-	25280000	27741000	27871000	12939000	3911400	6621700
KRT1	-	-	2515300000	2555300000	2630500000	776260000	755040000	775560000
KRT9	-	-	1059600000	1024600000	1058700000	353010000	341530000	374260000
DCD	-	-	118730000	143100000	133030000	44318000	51770000	48909000
MPRIP	-	-	26322000	32748000	28400000	14116000	11166000	14848000
COX6B1	-	-	35589000	35928000	34219000	15860000	23307000	21008000
CAMK2D	-	-	257210000	218070000	326460000	159210000	126110000	173010000
SBSN	-	-	11616000	11130000	11695000	5453000	6747100	7810800
NDUFAB1	-	-	23234000	21697000	20580000	15682000	10807000	12276000
PTGDS	-	-	275700000	318590000	258730000	194800000	160510000	157420000
NME1	-	-	282910000	209150000	237100000	137330000	148220000	178220000
HNRNPL	-	-	85167000	100330000	98543000	76226000	57391000	48261000
CRYZ	-	-	49316000	60680000	55322000	25926000	42151000	39514000
CDC42	-	-	65063000	65821000	59630000	43908000	40240000	44872000

PPA1	-	-	183660000	181140000	227110000	130040000	143450000	132130000
C1QBP	-	-	84900000	80329000	87813000	56540000	56901000	60013000
SBDS	-	-	23757000	21779000	26654000	15622000	20848000	13383000
TUBA1A	-	-	310490000	301190000	346660000	228760000	212410000	221710000
PICALM	-	-	45433000	62492000	55491000	37399000	37463000	39018000
PSG1	-	-	332750000	342150000	341310000	248100000	226650000	233930000
NUDC	-	-	188470000	173960000	164350000	117430000	137290000	114700000
ETFA	-	-	28800000	30334000	28043000	19096000	19582000	22612000
STC1	-	-	95614000	110440000	96409000	62102000	73075000	79073000
HINT1	-	-	74970000	69914000	76393000	52822000	49564000	54413000
PVR	-	-	169010000	160950000	144800000	125020000	90048000	124110000
RARRES1	-	-	53597000	58632000	65193000	43138000	44348000	40335000
HBD	-	-	449680000	450690000	374610000	299810000	340340000	288020000
CSRP2	-	-	267780000	276610000	254130000	185950000	198720000	196910000
HSPA9	-	-	138790000	128600000	153900000	98466000	108660000	101140000
NBL1	-	-	13368000	13214000	12895000	9451700	10691000	9057000
IST1	-	-	20324000	20832000	19211000	17058000	14474000	13232000
HNRNPC	-	-	133360000	133560000	151940000	110480000	100850000	100270000
TWSG1	-	-	919580000	836010000	819490000	602840000	675140000	664310000
CS	-	-	106950000	89552000	102740000	71268000	78336000	76280000
SERBP1	-	-	153970000	139830000	127560000	107600000	106210000	104570000
SEC22B	-	-	141500000	135910000	143500000	114110000	104500000	100020000
DCBLD2	-	-	36996000	32043000	36118000	22969000	27663000	29176000
HIST1H2AJ	-	-	10864000	11209000	10755000	9014300	7661700	8336400
PRKAR2A	-	-	35215000	37435000	31116000	28581000	27919000	23129000
BOLA2	-	-	30621000	29669000	23655000	21974000	21845000	20610000
CTNNA1	-	-	36172000	33779000	34929000	23475000	32216000	25015000
ARPC3	-	-	111460000	115480000	109770000	87617000	88966000	84849000
RNASE4	-	-	309260000	321960000	327910000	270990000	250980000	225390000
MYDGF	-	-	541210000	549820000	479050000	386960000	410310000	434320000
GOT2	-	-	261190000	227160000	242870000	189670000	201630000	182620000
CAP1	-	-	602370000	568880000	557100000	463100000	419610000	475430000
GLRX3	-	-	56262000	56304000	68228000	46596000	50097000	46632000
STXBP1	-	-	38736000	42254000	41661000	34458000	29202000	33990000

MVP	-	-	65638000	57805000	57942000	52405000	48758000	44306000
TIGAR	-	-	42884000	42376000	50803000	34488000	38743000	35921000
VAPA	-	-	50293000	47556000	43409000	39794000	38567000	35179000
ARPC5	-	-	161960000	168360000	158810000	122740000	139560000	131600000
PRDX6	-	-	2608700000	2514600000	2579400000	2043000000	2119700000	2048900000
HEBP1	-	-	72015000	77035000	75099000	56606000	63021000	62565000
AXL	-	-	136480000	146680000	139960000	122980000	114630000	109090000
MIF	-	-	466610000	459610000	477930000	378850000	384080000	390050000
EXT2	-	-	570840000	589440000	588520000	496050000	470970000	483400000
CNN1	-	-	91775000	92144000	102110000	77755000	83786000	76771000
GSTT1	-	-	40814000	36630000	39522000	51318000	45191000	44097000
MAP4	-	-	784810000	706650000	701040000	865970000	855380000	914310000
AP1M1	-	-	31934000	28935000	28614000	34971000	37240000	35404000
EIF3A	-	-	78538000	66226000	68272000	84817000	86996000	84416000
IAH1	-	-	30806000	31559000	31401000	37756000	38290000	36840000
GC	-	-	987630000	824970000	967560000	1098200000	1092000000	1157000000
FUCA1	-	-	31533000	31199000	34398000	41377000	39178000	36424000
ATP6V1A	-	-	49512000	55433000	61025000	66678000	63493000	70088000
CYTL1	-	-	80403000	81765000	87285000	96682000	100210000	104500000
PNP	-	-	226730000	240120000	237830000	294880000	286190000	273120000
HSP90AB2P	-	-	20604000	23755000	23181000	27225000	26737000	28067000
PREP	-	-	73280000	75420000	65532000	88255000	92430000	79793000
CAD	-	-	28293000	23538000	27651000	33235000	31063000	32605000
RAN	-	-	269360000	253770000	254810000	319680000	324810000	304990000
XPO1	-	-	180410000	171740000	178760000	198970000	218290000	230910000
PCBP2	-	-	60203000	48308000	56097000	65445000	64396000	71216000
DPP3	-	-	497710000	460550000	486250000	561360000	629510000	574190000
BCAT1	-	-	89891000	79097000	95724000	109070000	104750000	109790000
CEMIP	-	-	152380000	147130000	139160000	173040000	178470000	184850000
ANTXR1	-	-	171630000	167710000	165740000	207930000	206690000	203060000
EEF1B2	-	-	239340000	242050000	274820000	316230000	307730000	304220000
NAP1L1	-	-	123380000	132170000	136830000	152370000	167540000	163060000
COPB1	-	-	149540000	152460000	179170000	180890000	203070000	208850000
PPP1CA	-	-	102630000	101470000	112880000	128480000	129760000	132700000

HIST1H4A	-	-	116020000	93881000	92723000	116840000	127190000	129940000
IMPA1	-	-	60800000	67674000	68203000	83183000	79933000	80129000
KTN1	-	-	37999000	34260000	29360000	42304000	43447000	39940000
ARF1	-	-	218950000	220780000	222060000	266020000	284900000	272530000
EIF3E	-	-	19562000	16706000	19069000	23081000	22863000	23297000
COPB2	-	-	193830000	170100000	146720000	227230000	206810000	208350000
EPB41L2	-	-	47985000	55459000	55253000	72989000	65986000	61363000
FAP	-	-	22954000	20554000	23110000	30819000	24783000	28539000
PSMC1	-	-	36327000	34656000	37827000	50022000	41893000	45641000
BSG	-	-	86983000	72632000	81305000	94620000	113610000	96575000
HNRNPA2B1	-	-	561020000	560290000	547060000	678350000	729960000	716850000
PSMC2	-	-	53990000	49211000	45874000	56730000	69527000	63695000
DYNC1H1	-	-	510890000	513790000	512570000	680010000	598320000	681130000
RPS2	-	-	35611000	27344000	37155000	41544000	42986000	44247000
DDB1	-	-	287480000	263650000	309750000	342010000	400780000	365950000
RPS27A	-	-	2295400000	2022600000	1918100000	2657600000	2727400000	2667500000
PPM1F	-	-	34585000	31060000	35837000	42190000	44643000	44377000
ADPRHL2	-	-	20575000	21465000	23500000	29228000	26540000	29512000
PFAS	-	-	61734000	56176000	51783000	64763000	82718000	73412000
PTGES3	-	-	111550000	102050000	129260000	144120000	147960000	154720000
STOM	-	-	54634000	56910000	65292000	79073000	77229000	74435000
COPS4	-	-	43609000	49659000	40565000	56206000	61994000	56574000
FHL2	-	-	101150000	110640000	98624000	135150000	136230000	134160000
GART	-	-	20077000	17606000	18552000	23174000	22137000	28165000
TFG	-	-	64879000	66747000	73183000	96416000	76488000	94891000
PEPD	-	-	110640000	120230000	123230000	155190000	158340000	150330000
PSMA3	-	-	208850000	186850000	198580000	272010000	247460000	260570000
SDCBP	-	-	7298600	8666400	7915900	10420000	10325000	10689000
IPO7	-	-	140790000	107660000	127230000	150530000	165670000	182850000
PSMD12	-	-	45271000	40881000	36577000	46735000	56270000	60193000
EIF3H	-	-	19077000	20060000	19386000	25460000	26811000	25669000
PSMD3	-	-	58167000	58531000	52750000	81246000	74317000	70144000
IMPDH2	-	-	23966000	30005000	24583000	32153000	37263000	35239000
ADSS	-	-	107370000	99223000	110560000	139160000	134960000	153040000

DDX6	-	-	20994000	24254000	19263000	30247000	25293000	32281000
AKAP2	-	-	48816000	42970000	42106000	58569000	58668000	66009000
PSMC3	-	-	42235000	49533000	59148000	67699000	68096000	71225000
FASN	-	-	237660000	175870000	215840000	304730000	277320000	283930000
CD59	-	-	237630000	217130000	215000000	280610000	335310000	306050000
ADI1	-	-	22474000	28728000	23013000	30441000	33232000	39588000
POFUT2	-	-	39377000	40591000	51508000	66694000	52246000	66051000
TUBA1C	-	-	57001000	68190000	66240000	79090000	94940000	95772000
TUBB8	-	-	150870000	188190000	174010000	251930000	248930000	224000000
PSMD7	-	-	11027000	10858000	9919700	12811000	17588000	15271000
APEX1	-	-	87354000	101650000	98513000	135250000	142570000	135960000
PSMD13	-	-	49891000	51164000	50360000	60531000	77732000	79797000
PCLO	-	-	4873900000	6372600000	5569700000	6791700000	8560300000	9090300000
VCP	-	-	129390000	145770000	150850000	200730000	214220000	209020000
ACTR1A	-	-	50542000	55322000	55515000	78468000	78942000	80094000
TMOD3	-	-	32069000	29501000	28455000	42487000	45021000	46595000
RPS3	-	-	86434000	89692000	100440000	131990000	156210000	125700000
PSMC6	-	-	40600000	48090000	44187000	67947000	65043000	66203000
SEPT9	-	-	107750000	85674000	80469000	130510000	130270000	150300000
SMPDL3A	-	-	62906000	34598000	34997000	71068000	74958000	69691000
UBR4	-	-	11672000	10423000	12826000	21608000	20001000	16102000
FLNC	-	-	8424200000	8170900000	13837000000	19291000000	19369000000	19008000000
TMSB4X	-	-	809020000	779790000	661410000	1796500000	2107700000	2067900000
TMSB10	-	-	302440000	296690000	452170000	1194900000	1071100000	1209700000
SPAG9	-	-	0	0	11056000	14958000	16218000	14319000
ITGAV	-	-	2078400	0	0	3514000	3460400	2278300
PSME3	-	-	8642900	0	0	13265000	13313000	12760000
GYS1	-	-	9665800	0	0	14881000	12789000	18736000
CD99	-	-	0	0	0	12900000	14009000	10663000
RPL7	-	-	0	0	0	21033000	17526000	15060000
CD55	-	-	0	0	0	23912000	24611000	22698000
GNAS	-	-	0	0	0	23700000	33685000	25329000
ULBP2	-	-	0	0	0	29612000	29337000	31616000
