# Supplementary material for: The exosomal miRNA/FOXN3 axis empowers fibroblasts with tumor-promoting functions via enhanced migration and altered secretome
Source: J Nanobiotechnology. 2026 Jun 5;24:742. doi: 10.1186/s12951-026-04633-6 (PMC13459620; doi:10.1186/s12951-026-04633-6)
Supplement: Supplementary file 7 [file 12951_2026_4633_MOESM8_ESM.pdf]

Table S7 ITGB1 vs drug response

	Samples with high proliferative score and low proliferative score		Samples with low proliferative score		Samples with high proliferative score	
Drug	*P* value (Rho)	Rho (drug response vs ITGB1 expression)	*P* value (Rho)	Rho (drug response vs ITGB1 expression)	*P* value (Rho)	Rho (drug response vs ITGB1 expression)
NVP.TAE684	2.64E-07	-0.44590723	0.000111022	-0.413881301	0.005074951	-0.437898687
PHA.665752	1.41E-06	-0.420588264	0.00822721	-0.289979212	0.000997218	-0.506378987
Pazopanib	4.12E-05	-0.362290537	0.05534614	-0.212442452	0.000237702	-0.557410882
WH.4.023	0.000107526	-0.343448362	0.004908827	-0.307785239	0.007473888	-0.419512195
Bortezomib	0.000165292	-0.334598951	0.006613106	-0.297641463	0.00279448	-0.464540338
AG.014699	0.000235975	-0.327064725	0.007881131	-0.291502955	0.110129973	-0.256472795
BMS.509744	0.000294358	-0.322286439	0.056506622	-0.211462902	0.00024453	-0.556472795
BX.795	0.000391539	-0.316001309	0.020496828	-0.255542616	0.024925769	-0.355534709
GSK269962A	0.000558382	-0.307984628	0.009789485	-0.283731865	0.022238708	-0.362101313
Midostaurin	0.000568617	-0.307568262	0.063121231	-0.206173337	0.001636786	-0.486866792
PF.02341066	0.000590795	-0.306689269	8.68E-05	-0.419671525	0.352148785	-0.15065666
Docetaxel	0.000778621	-0.30026535	0.17799002	-0.150208426	0.015432603	-0.382363977
AZD.0530	0.000844916	-0.298335531	0.09038625	-0.188214935	0.003087245	-0.460225141
Paclitaxel	0.000986718	-0.294634507	0.109032477	-0.178288836	0.135930112	-0.239774859
Dasatinib	0.001274753	-0.288415466	0.038196483	-0.229356001	0.020540644	-0.366604128
AMG.706	0.002146275	-0.275316487	0.0917601	-0.187431296	0.158860156	-0.226829268
A.770041	0.002380128	-0.272639854	0.098430887	-0.183752544	0.016883138	-0.377485929
Vinblastine	0.002421576	-0.272190444	0.187518457	-0.147008566	0.037176843	-0.3315197
BI.2536	0.003660283	-0.261206335	0.297945256	-0.116337792	0.049741248	-0.312945591
Parthenolide	0.0045514	-0.255225217	0.196521331	-0.144091686	0.024685262	-0.356097561
BMS.536924	0.005973506	-0.247565421	0.087539243	-0.189869285	0.002582714	-0.467917448
CMK	0.006010893	-0.247386978	0.681351438	-0.046027928	0.003948511	-0.44934334
Imatinib	0.006149767	-0.24673269	0.036154351	-0.231772222	0.048333277	-0.314821764
JNK.Inhibitor.VIII	0.00721907	-0.242093193	0.622923615	-0.055105084	0.897565581	0.021013133
X17.AAG	0.008442481	-0.237480132	0.013589708	-0.271563687	0.334866761	-0.156097561
AP.24534	0.008699181	-0.236587921	0.039980717	-0.227331599	0.018825872	-0.371482176
Cisplatin	0.010754938	-0.23017722	0.225383145	-0.135341046	0.450694938	-0.122326454

Temsirolimus	0.011972548	-0.226872734	0.523656239	-0.071430904	0.002710374	-0.465853659
CGP.60474	0.015516375	-0.218697438	0.545571656	-0.067708617	0.018323273	-0.372983114
BI.D1870	0.018935702	-0.212227256	0.284497208	-0.119602956	0.694020207	-0.063977486
GW843682X	0.019443181	-0.211354872	0.460439014	-0.0826413	0.079194022	-0.281050657
S.Trityl.L.cysteine	0.020784078	-0.209140867	0.190040601	-0.146181391	0.537184806	-0.100187617
VX.680	0.021541139	-0.207944643	0.123566367	-0.171431992	0.674468367	0.068292683
AZD7762	0.021891846	-0.207402708	0.10483504	-0.180400309	0.139409271	-0.237711069
Bryostatin.1	0.022901825	-0.205882645	0.501076032	-0.0753491	0.02877559	-0.347091932
Cytarabine	0.023108547	-0.205578632	0.109384395	-0.178114694	0.166008753	-0.223076923
A.443654	0.040336023	-0.185916945	0.401106659	-0.093960535	0.508476662	-0.107317073
Axitinib	0.043587561	-0.183042043	0.096378964	-0.1848627	0.069006339	-0.290806754
AZD8055	0.043633895	-0.183002389	0.036244334	-0.231663383	0.044044192	-0.320825516
GDC0941	0.048310752	-0.179169186	0.037264998	-0.230444389	0.153305986	-0.229831144
KIN001.135	0.049620126	-0.178151405	0.248289009	-0.128941325	0.198599507	-0.207317073
WZ.1.84	0.050993918	-0.177107188	0.246361377	-0.129463751	0.030164156	-0.344277674
NVP.BEZ235	0.058677467	-0.171661396	0.223057186	-0.136015847	0.151260529	-0.230956848
Vorinostat	0.068229138	-0.165653842	0.029385499	-0.240697004	0.765269277	-0.048592871
Bexarotene	0.069817979	-0.164721977	0.379226441	-0.09837939	0.00024315	-0.556660413
NU.7441	0.072649965	-0.163102779	0.371223847	-0.10003374	0.30247458	-0.166791745
OSI.906	0.075030003	-0.161780985	0.558591238	-0.065531841	0.171150507	-0.220450281
JNJ.26854165	0.075610467	-0.161463755	0.869486672	0.018426409	0.049315402	-0.313508443
Elesclomol	0.080080164	-0.159084525	0.523656239	-0.071430904	0.023975385	-0.357786116
CHIR.99021	0.08074296	-0.158740859	0.083580466	-0.19224197	0.008099979	-0.415572233
RDEA119	0.081758309	-0.15821875	0.084547571	-0.191654241	0.197362672	-0.207879925
CEP.701	0.082471218	-0.157855257	0.20874088	-0.140282328	0.163121677	-0.224577861
XMD8.85	0.085841785	-0.15616997	0.064905802	-0.204823736	0.115295653	-0.252908068
AZ628	0.098429548	-0.150307813	0.057159872	-0.210918708	0.176027644	-0.218011257
Sunitinib	0.124229874	-0.139931729	0.660657867	-0.049206021	0.164199997	-0.224015009
Thapsigargin	0.13136463	-0.13736084	0.433621633	-0.087647885	0.053407336	-0.308255159
Rapamycin	0.132398212	-0.136997347	0.224555867	-0.135580492	0.747686648	0.052345216
MG.132	0.143078758	-0.133362413	0.925078269	-0.01054648	0.001931049	-0.48011257
BMS.754807	0.160567731	-0.127830705	0.19447927	-0.144744719	0.099588611	-0.264165103

SB.216763	0.164639971	-0.126608045	0.223580924	-0.135863473	0.109065408	-0.257223265
TW.37	0.217583527	-0.112431804	0.427456373	-0.088823344	0.492953653	-0.111257036
GNF.2	0.251365717	-0.104639828	0.913060256	-0.012244365	0.039478193	-0.327767355
PF.562271	0.265602876	-0.101573265	0.288399508	-0.118645175	0.067172454	-0.292682927
JW.7.52.1	0.295091611	-0.095559102	0.966327576	-0.004734488	0.08044204	-0.279924953
CGP.082996	0.312321929	-0.092228181	0.46997571	-0.080899879	0.422543505	-0.130018762
WO2009093972	0.334652698	-0.088084357	0.916602124	-0.011743706	0.078575762	-0.281613508
LFM.A13	0.347586422	-0.085764608	0.465790287	0.081661751	0.015812284	-0.381050657
Embelin	0.349603727	-0.085407724	0.076720904	-0.196573755	0.066449873	-0.293433396
AICAR	0.373756626	-0.081230855	0.552453615	0.066554926	0.188459352	-0.212007505
ABT.263	0.383664839	-0.079565394	0.093929861	-0.186212301	0.066449873	0.293433396
FTI.277	0.400893377	-0.076730146	0.819197127	0.025631537	0.043786808	-0.32120075
KU.55933	0.408051047	-0.075573576	0.818895256	-0.025675073	0.743310435	0.053283302
Z.LLNle.CHO	0.410151058	-0.075236519	0.23594344	-0.132337096	0.487105641	-0.112757974
ZM.447439	0.50619058	-0.060756264	0.397827108	-0.094613568	0.529554099	0.10206379
PLX4720	0.534008011	-0.056843754	0.77111227	-0.032618988	0.719820928	-0.058348968
Nilotinib	0.559396443	-0.053360827	0.714863254	-0.04095604	0.450694938	-0.122326454
MS.275	0.57321534	-0.051497097	0.053083624	-0.21440155	0.094976306	0.267729831
X681640	0.650833502	-0.041385372	0.293777623	-0.117339109	0.162763391	0.224765478
CI.1040	0.692339759	-0.036184113	0.94854381	0.00723778	0.12529216	-0.246341463
Epothilone.B	0.765536194	-0.027275221	0.878664487	0.017120343	0.081493837	-0.278986867
Pyrimethamine	0.824171893	-0.020322584	0.628297483	-0.054256141	0.390892968	0.13902439
AZD.2281	0.828900582	-0.01976743	0.325203916	-0.110003374	0.812412274	0.038649156
AZD6482	0.886646108	-0.013039499	0.824030612	-0.024934969	0.504016291	-0.108442777
AKT.inhibitor.VIII	0.918056648	-0.009411174	0.99078598	0.001295182	0.675314139	-0.068105066
QS11	0.933151864	-0.007673015	0.676795021	-0.046724496	0.981117972	-0.003939962
Camptothecin	0.936369306	-0.007302912	0.488019661	-0.077656483	0.120357914	0.249530957
BMS.708163	0.965942175	-0.003905901	0.439028507	0.0866248	0.677853841	-0.067542214
PD.0325901	0.967094067	-0.003773722	0.816179606	-0.026066892	0.578500418	-0.090243902
AUY922	0.954830371	0.005181433	0.044821866	0.222194408	0.023358845	-0.359287054
RO.3306	0.882766752	0.013488908	0.023306416	0.250340121	0.291605403	-0.17054409
GDC.0449	0.849007278	0.017414637	0.159885226	-0.156651683	0.342547226	0.153658537

Erlotinib	0.828055719	0.019866565	0.687492101	-0.045091914	0.645955424	-0.07467167
CCT018159	0.775485278	0.026085606	0.488998724	0.077482341	0.561957587	0.094183865
Etoposide	0.721961589	0.03254257	0.604610588	-0.058021964	0.353967303	0.150093809
NSC.87877	0.660762611	0.040129668	0.538860358	0.06884054	0.501793642	0.109005629
SL.0101.1	0.645476201	0.042066096	0.624988177	0.054778567	0.0320026	0.340712946
Cyclopamine	0.624281538	0.044782383	0.992334503	-0.001077504	0.461953973	-0.119324578
Bicalutamide	0.617792887	0.045621722	0.788698656	0.030050392	0.214307389	-0.200375235
Vinorelbine	0.480767369	0.064430852	0.799182031	0.028526649	0.670245507	-0.069230769
Lenalidomide	0.463839339	0.066935652	0.610052344	0.057151253	0.664350479	0.07054409
GSK.650394	0.462160342	0.067186793	0.781978986	0.031029942	0.791862198	-0.042964353
ATRA	0.431412672	0.071879162	0.992799066	0.001012201	0.189656222	0.211444653
Methotrexate	0.417071807	0.07413282	0.974374852	-0.003602564	0.065377647	0.294559099
AZD6244	0.400364653	0.076816062	0.685347687	0.045418431	0.933291664	-0.01369606
Obatoclax.Mesylate	0.390758252	0.078388997	0.104197344	0.180726826	0.600896469	-0.084990619
Bleomycin	0.362167134	0.083213546	0.661644391	0.049053647	0.306894503	0.165290807
CCT007093	0.358658052	0.083821571	0.936186791	0.008979201	0.574541562	-0.091181989
IPA.3	0.305133259	0.093602847	0.305007824	0.114661675	0.716362134	0.059099437
DMOG	0.283731997	0.097825979	0.46483648	0.081835893	0.727623504	-0.056660413
VX.702	0.282850386	0.098004421	0.955036296	-0.006323534	0.394123124	0.138086304
PD.173074	0.271461063	0.100343997	0.350660688	-0.104387292	0.025990785	0.353095685
Lapatinib	0.266009503	0.101487349	0.550371645	0.06690321	0.848444158	-0.031144465
Gemcitabine	0.173822127	0.123931412	0.333555111	0.108131347	0.306339755	0.165478424
PD.0332991	0.16960472	0.125147463	0.048578736	0.218515657	0.286267546	-0.172420263
MK.2206	0.156528333	0.129066582	0.432705092	-0.087822027	0.101080545	0.2630394
BAY.61.3606	0.154272675	0.129767133	0.657561135	0.049684912	0.724152254	0.057410882
Sorafenib	0.145574518	0.1325429	0.625125917	0.0547568	0.642632921	-0.075422139
Bosutinib	0.142559589	0.133534246	0.218750764	0.137278377	0.971910316	0.005816135
ABT.888	0.138223633	0.13498822	0.420327288	0.090194713	0.061563889	0.298686679
Doxorubicin	0.109274778	0.145714578	0.395975756	0.09498362	0.112012087	0.255159475
FH535	0.089113631	0.154583817	0.238910686	0.131509921	0.527275363	-0.102626642
GW.441756	0.082849115	0.157663597	0.231463967	0.133599626	0.112012087	0.255159475
BIRB.0796	0.072920525	0.162950773	0.068293569	0.202342211	0.087809108	0.273545966

Shikonin	0.058894954	0.171515999	0.074906685	0.197770981	0.933291664	0.01369606
EHT.1864	0.04318006	0.183392318	0.509796648	0.073825357	0.019145884	0.37054409
SB590885	0.042081414	0.184350619	0.295494363	0.116925522	0.251675072	0.185178236
PF.4708671	0.035394723	0.190682013	0.041996462	0.225133055	0.177167267	0.217448405
Tipifarnib	0.032959774	0.193239684	0.034653005	0.233622482	0.329032577	-0.157973734
Gefitinib	0.025949914	0.201619859	0.025086569	0.247314403	0.338398114	0.154971857
AS601245	0.021792985	0.207554714	0.490469263	0.077221128	0.938801358	-0.012570356
PAC.1	0.020497494	0.209603495	0.263914828	0.124805451	0.229947292	0.19380863
BIBW2992	0.017652274	0.214527178	0.29685433	0.116599005	0.16062575	0.225891182
Mitomycin.C	0.013789501	0.222451333	0.390128946	0.096159079	0.000609351	0.524765478
JNK.9L	0.003847141	0.259851496	0.183065816	0.148488773	0.845730111	-0.031707317
Metformin	0.001421577	0.285725615	0.076554536	0.196682593	0.0030214	0.461163227
Nutlin.3a	0.000935042	0.295923257	0.192385235	0.145419519	0.076542346	0.283489681
Roscovitine	0.000431279	0.313840175	0.119081997	0.173478162	0.025412679	0.354409006
Salubrinal	0.000105891	0.343758984	0.01117668	0.278877654	0.206136012	0.203939962
