# Supplementary material for: The exosomal miRNA/FOXN3 axis empowers fibroblasts with tumor-promoting functions via enhanced migration and altered secretome
Source: J Nanobiotechnology. 2026 Jun 5;24:742. doi: 10.1186/s12951-026-04633-6 (PMC13459620; doi:10.1186/s12951-026-04633-6)
Supplement: Supplementary file 8 [file 12951_2026_4633_MOESM9_ESM.pdf]

Table S8 Sequences of primers and shRNAs

Primer (qPCR mRNA）	Sequence
α-SMA Forward	CCGATAGAACATGGCATCA
α-SMA Reverse	CGTCCAGAGGCATAGAGA
TGF-β1 Forward	GACACCAACTATTGCTTCAG
TGF-β1 Reverse	CAGGCTCCAAATGTAGGG
FN1 Forward	GACACTTATGAGCGTCCTAA
FN1 Reverse	CTACCATCATCCAGCCTTG
FOXN3 Forward	TCTGCCTGACATCCGATT
FOXN3 Reverse	GTTCCAAGATCCAGTTGTAGA
GAPDH Forward	CATCACCATCTTCCAGGAG
GAPDH Reverse	AGGCTGTTGTCATACTTCTC
**Primer (qPCR microRNA）**	**Sequence**
miR-21-5p Forward	ACACTCCAGCTGGGTAGCTTATCAGACTGA
miR-320a Forward	ACACTCCAGCTGGGAAAAGCTGGGTTGAGAGGGCG
miR-24-3p Forward	ACACTCCAGCTGGGTGGCTCAGTTCAGCAG
miR-21-3p Forward	ACACTCCAGCTGGGCAACACCAGTCGATG
miR-22-3p Forward	ACACTCCAGCTGGGAAGCTGCCAGTTGAAG
miR-138-5p Forward	ACACTCCAGCTGGGAGCTGGTGTTGTGAATC
miR-378a-3p Forward	ACACTCCAGCTGGGACTGGACTTGGAGTC
miR-140-3p Forward	ACACTCCAGCTGGGTACCACAGGGTAGAAC
miR-29c-3p Forward	ACACTCCAGCTGGGTAGCACCATTTGAA
miR-345-5p Forward	ACACTCCAGCTGGGGCTGACTCCTAGTC
miR-486-5p Forward	ACACTCCAGCTGGGTCCTGTACTGAGCTG
miR-423-3p Forward	ACACTCCAGCTGGGTCCTGTACTGAGCTG
miR-27a-3p Forward	ACACTCCAGCTGGGTTCACAGTGGCTAAG
miR-361-5p Forward	ACACTCCAGCTGGGTTATCAGAATCTCCA
miR-146a-5p Forward	ACACTCCAGCTGGGTGAGAACTGAATTC
miR-128 Forward	ACACTCCAGCTGGGCGGGGCCGTAGCACT
miR-3187-3p Forward	ACACTCCAGCTGGGTTGGCCATGGGGCT
miR-339-3p Forward	ACACTCCAGCTGGGTGAGCGCCTCGACGACA
miR-502-3p Forward	ACACTCCAGCTGGGAATGCACCTGGGCA

miR-224-5p Forward	ACACTCCAGCTGGGCAAGTCACTAGTGGTT
miR-500a-3p Forward	ACACTCCAGCTGGGATGCACCTGGGCAA
miR-125a-3p Forward	ACACTCCAGCTGGGACAGGTGAGGTTCTT
miR-138-1-3p Forward	ACACTCCAGCTGGGGCTACTTCACAACA
miR-501-3p Forward	ACACTCCAGCTGGGAATGCACCCGGGCAA
miR-151b Forward	ACACTCCAGCTGGGTCGAGGAGCTCA
miR-323a-3p Forward	ACACTCCAGCTGGGCACATTACACGGTC
miR-3176 Forward	ACACTCCAGCTGGGACTGGCCTGGGA
miR-192-5p Forward	ACACTCCAGCTGGGCTGACCTATGAATTG
miR-342-3p Forward	ACACTCCAGCTGGGTCTCACACAGAAATC
miR-381-3p Forward	ACACTCCAGCTGGGTATACAAGGGCAAG
miR-486-3p Forward	ACACTCCAGCTGGGCGGGGCAGCTCAGTA
miR-369-3p Forward	ACACTCCAGCTGGGAATAATACATGGTT
U6 Forward	CTCGCTTCGGCAGCACA
shRNA	Sequence
FOXN3 shRNA Forward	CCACTATGAGTTTGCCACCAACTCGAGTTGGTGGCAAACTCATAGTGG
FOXN3 shRNA Reverse	CCACTATGAGTTTGCCACCAACTCGAGTTGGTGGCAAACTCATAGTGG
Scramble shRNA Forward	CCTAAGGTTAAGTCGCCCTCGCTCGAGCGAGGGCGACTTAACCTTAGG
Scramble shRNA Reverse	CCTAAGGTTAAGTCGCCCTCGCTCGAGCGAGGGCGACTTAACCTTAGG
Primers for FOXN3 3'UTR (luciferase vector)	Sequence
miR-21-5p Forward (KpnI)	GCGGTACCGTCTACGATTACTTGCTATTGG
miR-21-5p Reverse (wildtype) (XhoI)	ATAAGAATCTCGAGCAGGGAAGTAGGAGCTTATG
miR-21-5p Reverse (mutant) (XhoI)	ATAAGAATCTCGAGCAGGGAAGTAGCCCGGGCGGGTATATTATAAGGC
miR-21-3p Forward 1 (KpnI)	GCGGTACCAACCTAAGTGATAACCCTCT
miR-21-3p Reverse 1 (wildtype) (XhoI)	ATAAGAATCTCGAGATGAAACTCCAGAAACAGG
miR-21-3p Reverse 1 (mutant) (XhoI)	ATAAGAATCTCGAGCTCCAGAAACAGCCCGCGGCCACAATGTATATACT
miR-21-3p Forward 2 (KpnI)	GCGGTACCGGCCAATAGACCTACACG
miR-21-3p Reverse 2 (wildtype) (XhoI)	ATAAGAATCTCGAGCAGGTATGGATGCCTTCC
miR-21-3p Reverse 2 (mutant) (XhoI)	ATAAGAATCTCGAGCAGGTATGGATGCCTTCCAAGGCCGCGGCAGTCCCTAGAGTTCC
miR-22-3p Forward (KpnI)	GCGGTACCAGGTTCAACATTCAGGACTG
miR-22-3p Reverse (wildtype) (XhoI)	ATAAGAATCTCGAGTCACACTTAGAAAGCTGCC
miR-22-3p Reverse (mutant) (XhoI)	ATAAGAATCTCGAGTCACACTTAGACCCGGCGGCGATGAGGTATTTAT

miR-320a Forward (KpnI)	GCGGTACCAGGTTCAACATTCAGGACTG
miR-320a Reverse (wildtype) (XhoI)	ATAAGAATCTCGAGTCACACTTAGAAAGCTGCC
miR-320a Reverse (mutant) (XhoI)	ATAAGAATCTCGAGTCACACTTACCCCCGGCCCCGATGAGGTATTT
