# Supplementary material for: The exosomal miRNA/FOXN3 axis empowers fibroblasts with tumor-promoting functions via enhanced migration and altered secretome
Source: J Nanobiotechnology. 2026 Jun 5;24:742. doi: 10.1186/s12951-026-04633-6 (PMC13459620; doi:10.1186/s12951-026-04633-6)
Supplement: Supplementary file 9 [file 12951_2026_4633_MOESM1_ESM.docx]

**Supplementary figures**

**Figure S1. Highly proliferative tumor tissues contain more CAFs than low-proliferative tumor tissues (TCGA-LUAD, related to Figure 1)**
**A**. Heatmap showing unsupervised hierarchical clustering of samples (including tumor and paracancerous tissues, x-axis) in the TCGA-LUAD cohort based on the expression levels of the 11-gene proliferation signature (y-axis). The four subclusters were assigned to the normal group (blue bar), proliferation score–high group (orange bar), proliferation score–intermediate group (green bar), and proliferation score–low group (purple bar). **B**. Proliferation scores were significantly higher in tumor samples in the proliferation score–high group than in those in the proliferation score–low group, as determined by a two-sided Student’s *t* test (*P* < 0.05). The boxes represent the median ± 1 quartile, and the whiskers extend from the hinge to the smallest or largest value. **C**. Kaplan–Meier curves showing overall survival in patients with high-, intermediate-, and low-proliferative tumors. A two-sided log-rank test with *P* < 0.05 was considered statistically significant. **D–F**. mRNA expression levels of the CAF marker COL10A1 (D) and the NF marker FIGF (E), as well as the ratio of COL10A1 to FIGF expression (F), in the normal group and in the proliferation score–high, –intermediate, and –low groups.

**Figure S2. Highly proliferative tumor tissues contain more CAFs than low-proliferative tumor tissues (GSE40419, related to Figure 1)**
**A**. Heatmap showing unsupervised hierarchical clustering of samples (including tumor and paracancerous tissues, x-axis) in the GSE40419 dataset based on the expression levels of the 11-gene proliferation signature (y-axis). The four subclusters were assigned to the normal group (blue bar), proliferation score–high group (orange bar), proliferation score–intermediate group (green bar), and proliferation score–low group (purple bar). **B**. Proliferation scores were significantly higher in tumor samples in the proliferation score–high group than in those in the proliferation score–low group, as determined by a two-sided Student’s *t* test (*P* < 0.05). The boxes represent the median ± 1 quartile, and the whiskers extend from the hinge to the smallest or largest value. **C–E**. mRNA expression levels of the CAF marker COL10A1 (C) and the NF marker FIGF (D), as well as the ratio of COL10A1 to FIGF expression (E), in the normal group and in the proliferation score–high, –intermediate, and –low groups.

**Figure S3. Fibroblasts paired with highly proliferative tumor cells contribute to cell migration and ECM remodeling (related to Figure 2)**
**A–E**. Heatmaps showing the expression of genes associated with migration and ECM remodeling in fibroblasts paired with low-proliferative and highly proliferative tumor cells.

**Figure S4. Tumor-derived exosomes remodel the ECM through exosome-induced CAFs (related to Figure 3)**
The abundance of 51 proteins secreted by A549-derived exosome–induced MRC5 cells was significantly higher than that of the same 51 proteins secreted by untreated MRC5 cells. The mRNA expression levels corresponding to these 51 secreted proteins are shown in four subclusters of LUAD tissues in the GSE87340 dataset (**A**) and the GSE40419 dataset (**B**), namely the normal group (blue bar), proliferation score–low group (purple bar), proliferation score–intermediate group (green bar), and proliferation score–high group (orange bar). The gene symbols to the right of the heatmaps indicate representative genes from the 51 mRNAs. A two-sided Student’s *t* test with *P* < 0.05 was considered statistically significant; otherwise, the difference was considered nonsignificant (n.s.).

**Figure S5. LUAD cell–derived exosomal microRNAs exhibit specific expression profiles (related to Figure 4)**
**A**. Expression levels of A549-derived exosomal microRNAs measured by qPCR. The y-axis denotes the Ct value. The data are representative of three independent experiments (mean ± s.e.m.). The targets of the top five most highly expressed exosomal microRNAs were predicted using miRanda and TargetScan. The overlapping targets were used for enrichment analyses with downregulated genes from the tumor stroma of breast cancer (**B**), ovarian cancer (**C**), colorectal cancer (**D**), glioblastoma (**E**), and oral squamous carcinoma (**F**) samples.

**Figure S6. FOXN3 expression is lower in tumor tissues than in normal tissues (related to Figure 4)**
**A** and **B**. FOXN3 expression levels in normal tissues and in tumor tissues with low, intermediate, and high proliferative statuses from the GSE87340 (A) and GSE40419 (B) datasets. **C** and **D**. Expression levels of FOXN3 (C) and COL10A1 (D) in normal stroma and tumor stroma from the GSE22863 dataset. **E** and **F.** Reanalysis of the publicly available single-cell RNA sequencing dataset GSE131907, which includes annotated fibroblast populations from human lung cancer tissues and non-malignant lung tissues. Violin plots display the distribution of *α-SMA* (left) and *FOXN3* (right) expression levels in fibroblasts isolated from tumor tissues (red) versus non-malignant lung tissues (blue). Statistical significance was assessed using two-sided Wilcoxon rank-sum tests.

**Figure S7. Binding peaks of FOXN3 on the gene bodies of migration-associated genes (related to Figure 6)**
Tracks of FOXN3 binding peaks in MRC5 cells expressing FOXN3 shRNA and in MRC5 cells expressing scrambled shRNA across the gene bodies of ITGA1 (**A**) and SEMA7A (**B**) are shown in red and blue, respectively. The red dashed box denotes the transcription start region.

**Figure S8. The *ACTA2*/*FIGF* ratio is significantly elevated in highly proliferative LUAD tissues across three independent cohorts**

Violin plots display the distribution of the *ACTA2*/*FIGF* ratio across these groups in three independent LUAD cohorts: TCGA-LUAD (left), GSE87340 (middle), and GSE40419 (right). Across all three cohorts, the *ACTA2*/*FIGF* ratio was significantly higher in highly proliferative tumor tissues compared to both low-proliferative tumors and adjacent normal tissues (*P* < 0.001).

**Figure S9. Validation experiments with additional LUAD cell lines and primary fibroblasts** **A**. Quantitative PCR (qPCR) analysis of the relative expression levels of the exosomal microRNAs in exosomes derived from H1975 cells. The y-axis denotes the Ct value. **B**. Exosomes from distinct LUAD cell lines consistently suppress *FOXN3* and induce *α-SMA* in both embryonic and primary adult lung fibroblasts. Quantitative PCR analysis of *FOXN3* and *α-SMA* expression in MRC5 cells and HPF-a cells. **C**. *FOXN3* knockdown upregulates the expression of *α-SMA* and *TGF-β1.* **D.** Transwell migration assay of primary adult human lung fibroblasts (HPF-a) transfected with control shRNA (shScramble) or *FOXN3*-targeting shRNA (sh*FOXN3*). Left panel: representative images of migrated cells stained with crystal violet (scale bar = 200 μm). Right panel: quantification of migrated cell numbers per field. **E**. *FOXN3* knockdown upregulates ECM-associated gene expression in primary adult human lung fibroblasts. Quantitative PCR analysis of *FN1*, *ITGB3*, and *ITGA1*. Expression levels were normalized to *GAPDH*. Data are presented as mean ± SEM from three independent experiments. Statistical significance was assessed using two-sided Student’s *t*-tests.
